# Global Gene Expression Profiling and Alternative Splicing Events during the Chondrogenic Differentiation of Human Cartilage Endplate-Derived Stem Cells

**DOI:** 10.1155/2015/604972

**Published:** 2015-11-15

**Authors:** Jin Shang, Xin Fan, Lei Shangguan, Huan Liu, Yue Zhou

**Affiliations:** Department of Orthopedics, Xinqiao Hospital, Third Military Medical University, Chongqing 400037, China

## Abstract

Low back pain (LBP) is a very prevalent disease and degenerative disc diseases (DDDs) usually account for the LBP. However, the pathogenesis of DDDs is complicated and difficult to elucidate. Alternative splicing is a sophisticated regulatory process which greatly increases cellular complexity and phenotypic diversity of eukaryotic organisms. In addition, the cartilage endplate-derived stem cells have been discovered and identified by our research group. In this paper, we continue to investigate gene expression profiling and alternative splicing events during chondrogenic differentiation of cartilage endplate-derived stem cells. We adopted Affymetrix Human Transcriptome Array 2.0 (HTA 2.0) to compare the transcriptional and splicing changes between the control and differentiated samples. RT-PCR and quantitative PCR are used to validate the microarray results. The GO and KEGG pathway analysis was also performed. After bioinformatics analysis of the data, we detected 1953 differentially expressed genes. In terms of alternative splicing, the Splicing Index algorithm was used to select alternatively spliced genes. We detected 4411 alternatively spliced genes. GO and KEGG pathway analysis also revealed several functionally involved biological processes and signaling pathways. To our knowledge, this is the first study to investigate the alternative splicing mechanisms in chondrogenic differentiation of stem cells on a genome-wide scale.

## 1. Introduction

Low back pain (LBP) is one of the most prevalent diseases which needs medical advice and results in chronic disabilities [1]. It is estimated that approximately 84% of the general population suffer from LBP during their lifetime [2]. Degenerative disc disease (DDD) is a common reason for LBP [3]. The pathogenesis of DDD is difficult to elucidate because of the various DDD definitions and multiple interdependent factors, such as decreased nutrition supply [4], altered mechanical loading [5], hereditary factors [6], and changed extracellular matrix (ECM) composition [7]. Since the necessary nutrients have to diffuse across the intervertebral cartilage endplate (CEP) to supply the intervertebral discs (IVDs), many researchers speculate that CEP degeneration play critical roles in the initiation and development of DDD [8, 9]. CEP refers to a thin layer of hyaline cartilage between the vertebral body and the disc, which protects the adjacent vertebrae from the invading nucleus pulposus (NP). The CEP degeneration has several manifestations, such as proteoglycan loss [10], CEP calcification [11], and ECM synthesis defects [12]. The proteoglycan loss from CEP is closely related to proteoglycan loss in NP, in turn resulting in DDD ultimately [13]. In addition, calcification or sclerosis of CEP reduced the diffusion ability of nutrient molecules into adjacent disc, finally leading to DDD [14]. Therefore, it is crucial to illuminate the mechanisms of CEP degeneration and DDD for developing effective therapies.

Current treatment of DDD primarily concentrates on relieving painful symptoms through removing dislocated disc tissues, leaving the underlying biological changes of discs untreated. The flaws of current methods demand new therapies which directly target the underlying biochemical causes of DDD to both relieve symptoms and repair disc damage. In recent years, researchers have put interests on cell-based therapies for regenerating disc structure and function [15]. Mesenchymal stem cells (MSCs) are considered as an appropriate cell source for disc regeneration. Their capacity of expansion, self-renewal, and multilineage differentiation has been broadly validated [16–19]. In terms of intervertebral disc research, MSCs have been applied for IVD repair and regeneration in plenty of studies [20–23]. Except for exogenous MSCs, the stem cells* in situ* in IVDs are also an optional source. The evidence for stem cells existing in IVDs has been demonstrated [18, 24, 25]. Our research team has isolated cartilage endplate-derived stem cells (CESCs) and validated their chondrogenic and osteogenic differentiation ability [24]. It may be beneficial to modulate the differentiation capacity of CESCs to alleviate CEP calcification and restore CEP structure, partially regaining the nutrition supply of the discs. But the detailed mechanism of CESC differentiation has not been fully understood.

Alternative splicing (AS) is a sophisticated regulatory process by which diverse RNA isoforms are created from one single pre-mRNA, potentially resulting in structurally and functionally different proteins [26, 27]. AS substantially increases the cellular complexity and phenotypic diversity of eukaryotic organisms without enlarging the genome [27]. AS is prevalent in eukaryotic organisms, for it is estimated that approximately 95% of multiexonic genes undergo AS [28, 29]. Usually, a single gene can be alternatively spliced in such common ways: exon skip/inclusion, mutually exclusive exons, alternative 5′/3′ splice sites, intron retention, alternative promoters, and polyadenylation sites [27]. More importantly, AS is elaborately regulated through cell type-, development-, and extracellular signal-related pathways [30]. Abnormal AS of genes is found to be related to a variety of human diseases including neurodegenerative diseases, autoimmune diseases, and cancers [31–33]. Recently, roles of AS in stem cell differentiation have piqued the interest of researchers. Kazantseva and his colleagues found that the depletion of hTAF4-TAFH domain from TAF4 isoforms led to promoted chondrogenic differentiation of human MSCs [34]. Additionally, McAlinden et al. developed a novel AT-qPCR method to quantify all the isoforms of alternatively spliced* Col2a1* gene, identifying the majority of ATDC5 cells as chondroprogenitors induced by the standard chondrogenic differentiation method [35]. Furthermore, the* PTHrP* isoforms became increasingly selective during the osteogenic differentiation of MSCs, displaying the potential to be novel molecular markers of stem cell state [36]. Therefore, investigating the AS regulation in stem cell differentiation is quite meaningful.

CESCs exist in the degenerated discs. Enhanced chondrogenic differentiation and inhibited osteogenic differentiation of CESCs may relieve CEP calcification and restore the nutrition supply, possibly regenerating the degenerated discs. In our study, we tend to investigate the transcription and splicing mechanisms during CESC chondrogenic differentiation. The isolated CESCs were induced to go through chondrogenic differentiation. Then, the samples were analyzed on a genome-wide scale using the Affymetrix Human Transcriptome Array 2.0 (HTA 2.0) system. After data extraction and pretreatment, a comparative analysis of gene expression profiling and AS events was done between the controlled CESCs and differentiated CESCs. Gene ontology (GO) and KEGG pathways analysis was utilized to make functional annotation of genes of interest to exemplify transcription and splicing mechanisms. To our knowledge, genome-wide studies focusing on the differential gene expressions and AS events of stem cell chondrogenic differentiation have not been done before, so our study may facilitate illuminating DDD mechanisms and developing new therapies.

## 2. Materials and Methods

### 2.1. Ethics Statement

The CEP tissues used in our study were obtained from six patients who underwent discectomy and fusion surgeries because of lumbar degenerative diseases in our department (Table 1). Our study was approved by the Ethics Committee of Xinqiao Hospital, Third Military Medical University. All the procedures described below were in accordance with the Helsinki Declaration. Written informed consent was obtained from each patient, and we took extensive precautions to protect the privacy of each donor.

### 2.2. Isolation and Culture of Human CEP-Derived Cells

The CEP-derived cells were obtained according to a previously described protocol [24]. Briefly, the NP, annulus fibrosus (AF), and subchondral bone tissues surrounding the CEP blocks were removed by ophthalmic instruments under dissecting microscope (4x magnification). Then mince CEP samples into pieces no larger than 1 mm^3^ and digest them with 0.2% collagenase II (Sigma, USA) in DMEM/F12 (Hyclone, USA) containing 2% fetal bovine serum (FBS) at 37°C for 12 hours. After digestion, the suspended cells were filtered through a 70 *μ*m cell filter to avoid large cell aggregates. Then the cell suspension was transferred to a 15 mL sterile conical tube and centrifuged for 10 min at 200 g. After the centrifuging, the pellet was resuspended in DMEM/F12 containing 10% FBS and 5 units/mL penicillin and streptomycin. Next, the cell suspension was transferred to a 25 cm^2^ culture flask and cultured in humidified atmosphere containing 5% CO_2_ at 37°C. The cells were subcultured once and transferred to agarose cultures to select CESCs.

### 2.3. Agarose Culture to Select CESCs

The agarose culture method was used according to the protocol published by Thornemo et al. in 2005 [37]. Briefly, a 60 mm-diameter culture dish was coated with 1% low-melting-point agarose containing equal volume of DMEM/F12 (37°C) and 2% low-melting-point agarose. Then, 0.75 mL DMEM/F12, 0.75 mL 2% low-melting-point agarose, and 1.5 mL DMEM/F12 (20% FBS) containing 5 × 10^4^ P1 CEP-derived cells were mixed and transferred to the coated culture dishes. The final concentration of FBS was 10%. Put the culture dishes under 4°C for 15 min until the gel was solidified. Then, the culture dishes were incubated in humidified atmosphere containing 5% CO_2_ at 37°C. Culture medium was changed twice a week. Six weeks later, choose cell clusters with diameter larger than 50 *μ*m and isolate them by sterile Pasteur pipette. Then, subculture these cell clusters in 6-well plates (Costar Corning, USA). Passage 3 CESCs were used in our study.

### 2.4. Chondrogenic Differentiation Assay

Chondrogenic differentiation of CESCs was induced using the pellet method. The complete chondrogenic differentiation medium contains 97 mL basal medium (Cat. number HUXMA-03042-97, Cyagen, USA), 10 *μ*L dexamethasone, 300 *μ*L ascorbate, 1 mL ITS + supplement, 100 *μ*L sodium pyruvate, 100 *μ*L proline, and 1 mL TGF-*β*3. About 2.5 × 10^5^ CESCs were put into a 15 mL polypropylene tube and centrifuged at 150 g for 5 min. The final concentration of TGF-*β*3 in the complete chondrogenic medium is 10 ng/mL. Wash the cells by resuspending them in incomplete chondrogenic medium (without TGF-*β*3) and then centrifuge again. Resuspend the cells in complete chondrogenic medium at a concentration of 5.0 × 10^5^ per milliliter and centrifuge at 150 g for 5 min to form a cell pellet. The pellet was cultured for 21 days by replacing the complete chondrogenic medium every 3 days.

### 2.5. Affymetrix Human Transcriptome Array 2.0

CESCs were induced to undergo chondrogenic differentiation or left untreated in the undifferentiated state. Differentiated and undifferentiated samples were treated with Trizol and sent to Bioassay Laboratory of CapitalBio Corporation (Beijing, China). The gene expression profiling and alternative splicing events were analyzed using HTA 2.0 purchased from Affymetrix Corporation. The Affymetrix HTA 2.0 contained about 339 thousand probe sets (10 probes per exon and 4 probes per junction), covering about 67 thousand transcript clusters and 573 thousand Probe Selection Regions (PSRs). Transcript clusters were referred as genes in this paper for simplicity. The HTA 2.0 allowed probes to target exons and junctions within genes and provided both gene expression and AS information. The labeling, hybridization, scanning, and data extraction of microarray were performed by Bioassay Laboratory of CapitalBio Corporation (Beijing, China) according to the recommended Affymetrix protocols. Briefly, the fluorescence signals of the microarray were scanned and saved as DAT image files. The AGCC software (Affymetrix GeneChip Command Console) transformed DAT files into CEL files to change image signals into digital signals, which recorded the fluorescence density of probes. Next, we used Affymetrix Expression Console software to pretreat CEL files through Robust Multichip Analysis (RMA) algorithm [38], including background correction, probeset signal integration, and quantile normalization. After pretreatment, the obtained chp files were analyzed by Affymetrix Transcriptome Analysis Console software to detect differentially expressed genes (DEGs) and alternatively spliced genes (ASGs). The Expression Console and Transcriptome Analysis Console software were provided by Affymetrix Corporation. To identify significantly enriched gene ontology (GO) terms and functional pathways, the publicly available web tool Kyoto Encyclopedia of Genes and Genomes (KEGG, http://www.genome.jp/kegg/), DAVID (http://david.abcc.ncifcrf.gov/tools.jsp), and commercial database Molecule Annotation System (MAS, CapitalBio System) were used. The results of GO and pathways analysis were provided in the Supplementary Material available online at http://dx.doi.org/10.1155/2015/604972 and presented as tables and histograms in the paper. The microarray data have been submitted to NCBI's Gene Expression Omnibus (GEO) (Accession number GSE63897).

### 2.6. Criteria for Detecting DEGs and ASGs

The fold change of gene expression was calculated using undifferentiated samples as base values. We set the default filter criteria as fold change (linear) of gene expression ≤−2 or ≥2 for significantly DEGs. The Splicing Index (SI) model [39, 40] was used to identify ASGs. SI represented the ratio of the exon signal intensities normalized to the gene signal intensities between two experimental conditions and was used to detect the exon exclusion/inclusion level. The SI value was calculated in the following ways:

(1) Normalized  Intensityi,jANIi,jA=exoni  signal  intensity  in  condition  Agenej  signal  intensity  in  condition  A,SIi,j=log2⁡NIi,jDNIi,jU.

NI(*i*, *j*)_*A*_ stood for the signal intensity of *i*th exon normalized to the *j*th gene in condition *A*. The subscript *U* stood for the undifferentiated condition; the subscript *D* stood for the differentiated condition. We set the default filter criteria as SI (linear) ≤ −2 or ≥2.

### 2.7. DEG Validation by RT-qPCR (Reverse Transcriptase-Quantitative Polymerase Chain Reaction)

RT-qPCR analysis was performed to validate DEGs during chondrogenic differentiation of CESCs. GAPDH was chosen to be an internal control. Total RNA was extracted and used to generate cDNA by using the Takara kits (Japan) according to the manufacturer's instructions. The quality of total RNA was examined by a spectrophotometer (Nanodrop 2000, Thermo Scientific) at 260 nm and 280 nm. Primers were designed using the Primer Premier 6.0 software and listed in Table S1. The cycle parameters of the Reverse Transcriptase (RT) reaction were 37°C for 15 mins and 85°C for 5 s. Next, the cDNA was subjected to real-time quantitative PCR (qPCR) with SYBR Green staining. Higher absolute value of fold changes was privileged when selecting candidate genes for validation.

### 2.8. ASG Validation by Semiquantitative RT-PCR

RT-PCR was performed to identify the ASGs. Total RNA was extracted and used to generate cDNA by using the Takara kits (Japan) according to the manufacturer's instructions. The quality of total RNA was examined by a spectrophotometer (Nanodrop 2000, Thermo Scientific) at 260 nm and 280 nm. An oligo (dT) primer was used to reverse-transcribe 1 *μ*g total RNA into cDNA using the Takara RT-PCR kit (Code number RR047A). Then, 1 *μ*L cDNA template was added for each action. The primers of genes of interest were designed in expressed constitutive exons flanking the target exon, using the Primer Premier 6.0 software. GAPDH was selected as the internal control. The candidate genes for ASG validation were chosen according to the following criteria. (1) Higher absolute value of SI was firstly considered. (2) Whole exon gain/skip was privileged. (3) First and last alternative exons were excluded because of primer design difficulties.

### 2.9. Statistical Analysis

Student's *t*-test was used to determine the significance between groups. Data were expressed as mean values ± standard deviation (SD). A *p* value of less than 0.05 was considered to be statistically significant.

## 3. Results

**Figure 1:**
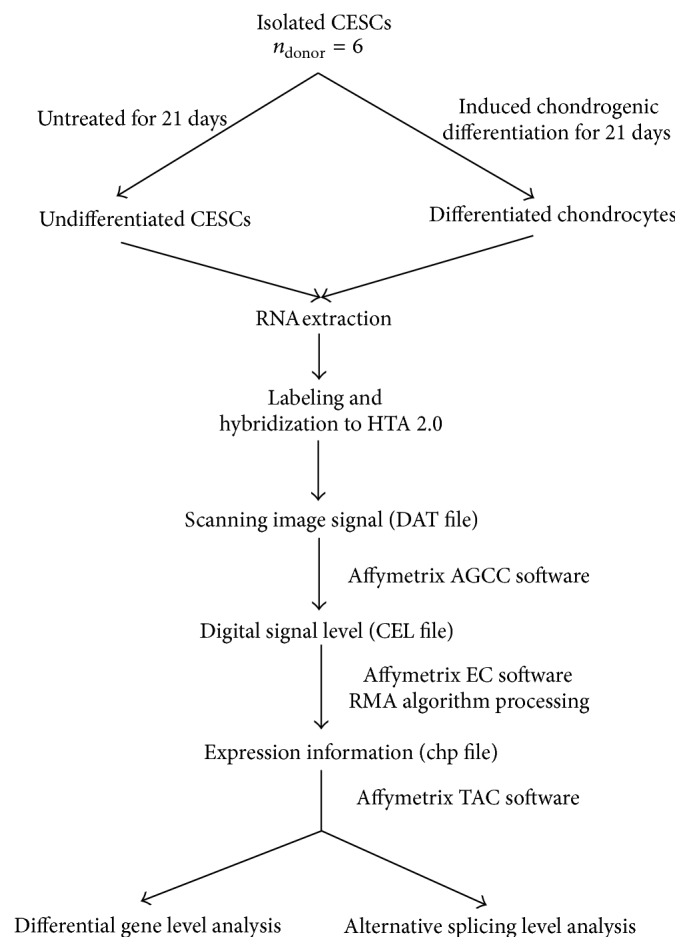
The overall workflow of the HTA data analysis. Briefly, CESCs were isolated and induced into chondrogenic differentiation. Total RNA was extracted, labeled, and hybridized to HTA 2.0. The Affymetrix AGCC, EC, and TAC software were used to scan and analyze the microarray data.

The overall workflow of the HTA data analysis was presented in Figure 1.

### 3.1. DEG Detection, Validation, and Functional Analysis during Chondrogenic Differentiation of CESCs

**Figure 2:**
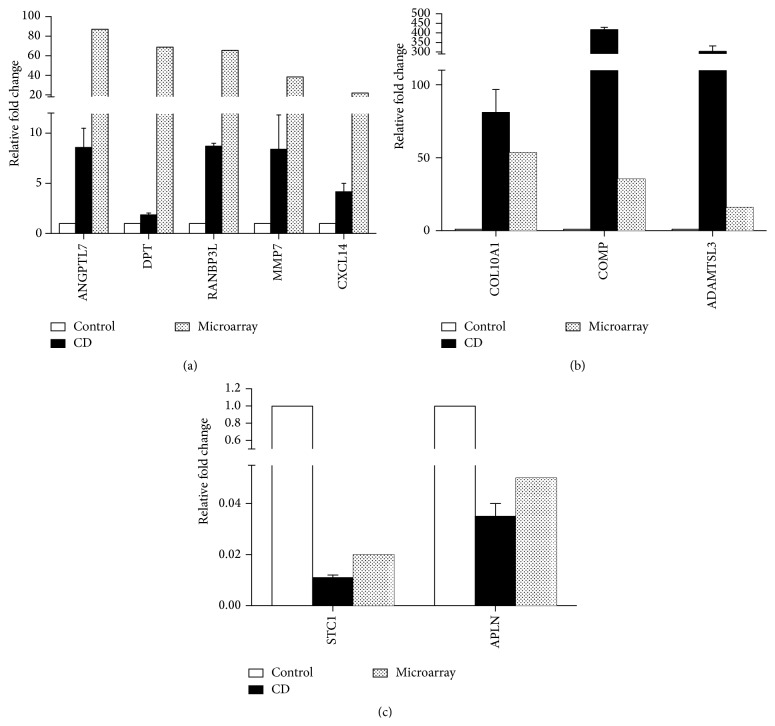
DEGs validated in chondrogenic differentiation of CESCs by RT-qPCR. 10 of 12 DEGs were validated successfully by RT-qPCR in chondrogenic differentiation of CESCs. They are (a) ANGPTL7; DPT; RANBP3L; MMP7; and CXCL14; (b) COL10A1; COMP; and ADAMTSL3; (c) STC1; APLN. The results of microarray were also listed in Figure 2.

Analysis of HTA data was performed using strict statistical methods to detect the differentially expressed genes during chondrogenic differentiation of CESCs. According to the criteria we mentioned, the analysis identified 1953 DEGs, of which 997 (51%) genes were upregulated and 956 (49%) genes were downregulated. The number of upregulated genes was almost the same (997/956) as that of downregulated genes. According to the gene expression results obtained from the microarray, 12 DEGs were selected for validation by RT-qPCR. The qPCR results showed that 10 of the 12 selected DEGs were successfully validated (consistent tendency) (Figure 2) while 2 DEGs were not (data not shown).

**Figure 3:**
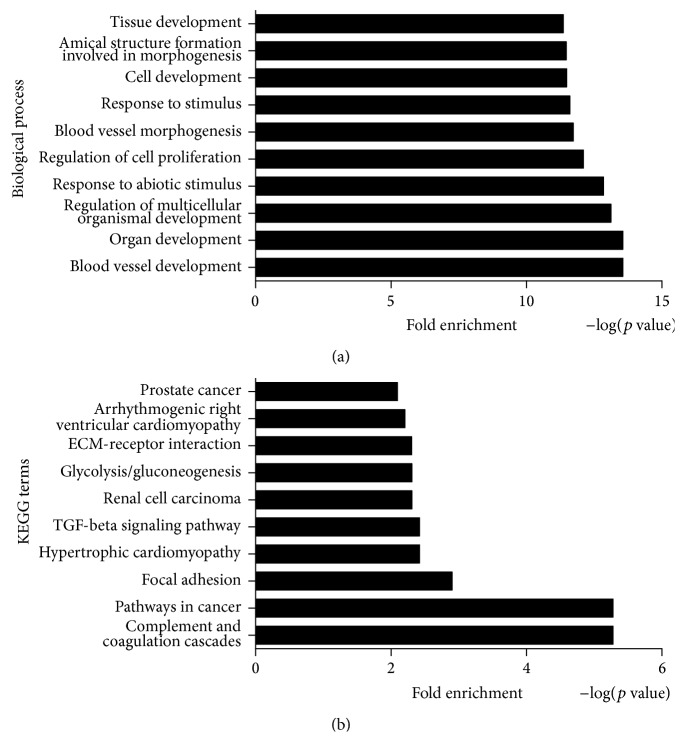
Molecular function analysis at the gene level during chondrogenic differentiation of CESCs. (a) Figure 3(a) showed the top ten GO functions regulated in biological process category during chondrogenic differentiation of CESCs at the level of gene expression. (b) Figure 3(b) showed the top ten KEGG pathways regulated in chondrogenic differentiation of CESCs at the level of gene expression.

GO enrichment analysis of DEGs during chondrogenic differentiation of CESCs was carried out to detect the chondrogenic differentiation-related biological process, molecular function, and cellular component (Table S2). The results showed that many important GO terms were involved in chondrogenic differentiation of CESCs, such as regulation of cell proliferation, cell differentiation, protein binding, and extracellular matrix structural constituent.  Figure 3(a) showed the top ten GO functions of DEGs regulated in biological process category during chondrogenic differentiation of CESCs.

The KEGG tool was used to detect enriched functional pathways in DEGs during chondrogenic differentiation of CESCs (Table S3). According to the results, several cellular pathways were significantly affected, such as TGF-beta signaling pathway, ECM-receptor interaction, complement and coagulation cascades, and cytokine-cytokine receptor interaction.  Figure 3(b) showed the top ten KEGG pathways of DEGs regulated in chondrogenic differentiation of CESCs.

### 3.2. ASGs Detection, Validation, and Functional Analysis during Chondrogenic Differentiation of CESCs

**Figure 4:**
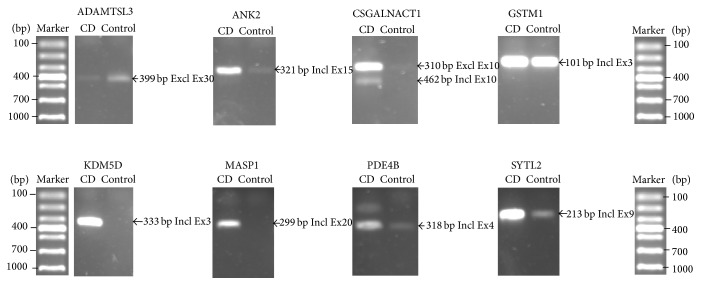
ASGs validated in chondrogenic differentiation of CESCs by RT-PCR. 8 of 10 ASGs were validated successfully by RT-PCR. The control sample refers to undifferentiated CESCs; the CD sample refers to chondrogenically differentiated CESCs. GAPDH was used as internal control, and its RT-PCR result image is found in Supplementary Material (Figure S6).

Based on the SI algorithm mentioned in the methods, this analysis of genome-wide AS identified 14061 alternatively spliced exons, which belonged to 4411 ASGs during chondrogenic differentiation of CESCs (Table S4). In addition, 7946 (56%) alternatively spliced exons with SI value ≥2 were considered as “general exon inclusion” events, while the remaining 6115 (44%) exons were referred to as “general exon exclusion” events. We found that 55% (2438/4411) of the ASGs contained 86% (12088/14061) of the alternatively spliced exons; thus this confirmed that multiple alternative splicing events could happen to the same gene. During chondrogenic differentiation of CESCs, each ASG had 5.0 (12088/2438) alternatively spliced exons on average. We picked out* EGLN3* as a typical example, which had 14 alternatively spliced exons detected and indicated complicated splicing regulation. Moreover, it was noticed that 610 of these 4411 ASGs were also significantly differentially expressed. This result indicated a possible intrinsic link between regulated gene expression and alternative splicing. Based on these results, 10 ASGs were chosen for RT-PCR validation. Primers for RT-PCR were listed in Table S5.  Figure 4 showed that 8 of the 10 selected ASGs were validated successfully.

**Figure 5:**
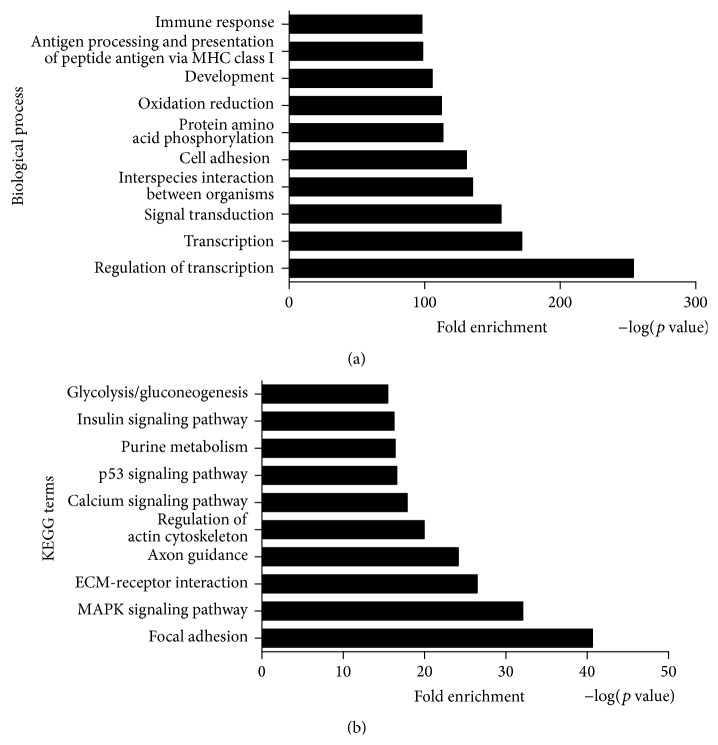
Molecular function analysis at the AS level during chondrogenic differentiation of CESCs. (a) Figure 5(a) showed the top ten GO functions regulated in biological process during chondrogenic differentiation of CESCs at the level of alternative splicing. (b) Figure 5(b) showed the top ten KEGG pathways regulated in chondrogenic differentiation of CESCs at the level of alternative splicing.

GO enrichment analysis was performed on the ASGs during chondrogenic differentiation of CESCs (Table S7). The results suggested that many important GO terms were regulated by AS in chondrogenic differentiation of CESCs, such as regulation of transcription, signal transduction, protein amino acid phosphorylation, and cell cycle.  Figure 5(a) showed the top ten GO functions of ASGs regulated in biological process during chondrogenic differentiation of CESCs.

The 4411 ASGs were analyzed by KEGG pathway analysis in order to determine functional cellular pathways regulated during chondrogenic differentiation of CESCs (Table S8). According to the results, several cellular pathways were regulated, such as MAPK signaling pathway, p53 signaling pathway, Wnt signaling pathway, and apoptosis.  Figure 5(b) showed the top ten KEGG pathways of ASGs regulated in chondrogenic differentiation of CESCs.

## 4. Discussion

Genome-wide analysis of transcription and translation is a powerful approach to fully investigate the mechanisms of chondrogenic differentiation of stem cells. Researchers have adopted this approach to determine the global gene expression and posttranscriptional and epigenetic changes during the differentiation process [41–44]. However, little effort has been made to obtain a coherent view of AS mechanisms of stem cell chondrogenic differentiation on a genome-wide scale. AS is a ubiquitous and essential phenomenon that accounts for distinctive gene isoforms and protein diversity [45], especially in chondrogenic differentiation [34, 36]. In our study, we discovered the gene expression patterns and ASEs during chondrogenic differentiation of CESCs and analyzed the molecular functions and pathways using bioinformatics tools. To our knowledge, our study is the first one to analyze the AS events induced during stem cell chondrogenic differentiation at the whole genome level.

The upregulated and downregulated genes during chondrogenic differentiation of CESCs were detected in our study. Similarly, Herlofsen et al. performed genome-wide analysis of gene expressions during chondrogenic differentiation of MSCs in alginate hydrogel [46]. They identified various enriched gene clusters at different differentiation time points. In our study, several DEGs were picked up for RT-qPCR validation, such as DPT, COMP, and COL10A1. The biological functions of DPT were various, such as interacting with transforming growth factor-beta1 (TGF-*β*1), enhancing cell adhesion, and inhibiting cell proliferation [47]. In addition, cartilage oligomeric matrix protein (COMP) is a classical gene marker of chondrogenic differentiation and plays important roles in cell proliferation, adhesion, and differentiation [48–50]. It is noted that COL10A1 gene was upregulated after chondrogenic differentiation. Type X collagen is a marker of chondrocyte hypertrophy [51]. Currently, the widely used protocol for chondrogenic differentiation is the pellet culture system, which is put forward by Yoo and Johnstone et al. [52, 53]. This system uses serum-free chondrogenic medium containing TGF-*β* and dexamethasone to induce chondrogenic differentiation. There have been studies reporting the expression of type X collagen and other hypertrophy-related genes in chondrogenesis of MSCs, such as ALP, MMP13, and VEGF [52, 54, 55]. These genes indicate the hypertrophy stage of MCS chondrogenic differentiation. The chondrocyte hypertrophy could result in ossification, vascular invasion, and apoptosis eventually. The current chondrogenic induction system inevitably leads to hypertrophy, but the degree of hypertrophy could be controlled to a low level. Mueller and Tuan found that the withdrawal of TGF-*β* contributed to hypertrophy induction [56]. Some other measures have been taken to regulated chondrogenic hypertrophy, such as gene modification, growth factor addition, and signaling pathway interfering [57–59]. Besides altered gene expression patterns, the molecular function and pathway of DEGs were also analyzed. The GO analysis showed enrichment of many important biological processes, such as response to hypoxia, developmental process, and regulation of cell proliferation. Hypoxia is closely related to the chondrogenic differentiation process [60]. Because in biological process genes generally are enriched into functional pathways, it is essential to investigate the regulatory mechanisms of chondrogenic differentiation in terms of functional pathways involved. Some of the pathways were significantly enriched, such as focal adhesion, TGF-beta signaling pathway, and ECM-receptor interaction. Focal adhesion signaling is important for cell-matrix adhesions, which play vital roles in many biological processes, such as cell proliferation, cell differentiation, and regulated gene expressions [61–63]. In terms of TGF-beta signaling pathway, this pathway is involved in a great variety of cellular functions including migration, proliferation, differentiation, and apoptosis [64–66]. What is more, TGF-beta is usually added as an inducer of chondrogenic differentiation of stem cells [67].

Alternative splicing is a prevalent phenomenon in eukaryotic cells resulting in great diversities of protein categories and functions. To our knowledge, genome-wide analysis of alternative splicing in chondrogenic differentiation of stem cells has not been done before. We use HTA 2.0 to obtain a universal coverage of the genome to both identify evidence supported sequences and discover novel ASEs. Previously, many bioinformatics studies analyzed EST data in order to identify new ASEs [68]. Actually, for most genes not many ESTs have been sequenced, and this defect makes more ASEs undetectable in the available ESTs. In our study, we detected 4411 ASGs with 14061 ASEs during chondrogenic differentiation of CESCs. Furthermore, 8 ASGs were validated by RT-PCR successfully. These novel validated isoforms of ASGs have not been recorded in the NCBI Reference Sequences Database before; therefore further studies need to be done to investigate cellular and molecular functions of these isoforms. In terms of the molecular functions and pathways of these detected ASGs, GO analysis showed quantities of enriched GO terms, such as DNA-dependent regulation of transcription, signal transduction, and cell adhesion. The biological process of DNA-dependent regulation of transcription infers the role of AS in transcription regulation, since the complex correlation of transcription and splicing has been investigated before [69–71]. Besides, the enriched signal transduction process suggests that alternative splicing may modulate signaling pathways to exert its influence on cellular function networks. In addition, we performed KEGG pathway analysis and discovered many enriched signaling pathways, such as MAPK signaling pathway, ECM-receptor interaction, and p53 signaling pathway. The MAPK signaling cascade is a highly conserved module which plays roles in a great variety of cellular processes, such as cell differentiation, migration, and proliferation [72–74]. Since in the chondrogenic differentiation process, stem cell proliferation is usually inhibited and stem cells are induced toward differentiation, it is reasonable that the p53 signaling pathway is activated which generally results in cell cycle arrest, cell senescence, or apoptosis [75–77]. Thus the pathway analysis indicated that AS is highly involved in signaling pathways to regulate differentiation process.

Under many physiological and pathological circumstances, regulation of transcription and splicing generally coexists. It was reported that transcript splicing may affect gene expression through modifying transcription process and influencing RNA stability [69, 70]. In the other direction, transcription aberrance may also boost or impair splicing efficiency [71]. The over- or underexpression of splicing factors and their upstreams may affect* cis*-elements availability or spliceosome assembly to influence splicing machinery. In our study, we observed the overlapping of DEGs and ASGs; therefore the interregulation between transcription and splicing may be speculated to underline the chondrogenic differentiation of CESCs.

## 5. Conclusions

In our study, we use HTA 2.0 to investigate changed gene expression patterns and alternative splicing events during chondrogenic differentiation of CESCs. Various genes are found to be differentially expressed and/or alternatively spliced, and correlated molecular functions and pathways are also revealed. Further studies are needed to elucidate downstream mechanisms of AS regulation, and novel functional isoforms are potential targets in future functional researches.

## Supplementary Material

Table S1: Primers for DEGs validated in chondrogenic differentiation of CESCs by RT-qPCR.
Table S2: GO enrichment analysis of DEGs during chondrogenic differentiation of CESCs.
Table S3: KEGG pathway results in DEGs during chondrogenic differentiation of CESCs.
Table S4: ASGs detected during chondrogenic differentiation of CESCs.
Table S5: Primers for ASGs validated in chondrogenic differentiation of CESCs by RT-PCR.
Figure S6: RT-PCR result of GAPDH as internal control.
Table S7: GO enrichment analysis of ASGs during chondrogenic differentiation of CESCs.
Table S8: KEGG pathway results in ASGs during chondrogenic differentiation of CESCs.

## Figures and Tables

**Table 1:** Patients' information enrolled in our study.

Case number	Gender	Age (year)	Diagnosis	Degenerated disc level	Surgery type
1	Male	46	Disc herniation	L5-S1	MED^a^
2	Male	50	Spondylolisthesis	L4-L5	Quadrant assisted TLIF^b^
3	Male	65	Disc herniation	L4-L5	Quadrant assisted TLIF
4	Female	76	Spondylolisthesis	L4-L5	Quadrant assisted TLIF
5	Female	47	Disc herniation	L5-S1	TLIF
6	Male	37	Disc herniation	L5-S1	TLIF

^a^MED: microendoscopic discectomy; ^b^TLIF: transforaminal lumbar interbody fusion.

## References

[1] Andersson G. B. J. (1999). Epidemiological features of chronic low-back pain. *The Lancet*.

[2] Walker B. F. (2000). The prevalence of low back pain: a systematic review of the literature from 1966 to 1998. *Journal of Spinal Disorders*.

[3] Freemont A. J. (2009). The cellular pathobiology of the degenerate intervertebral disc and discogenic back pain. *Rheumatology*.

[4] Urban J. P. G., Smith S., Fairbank J. C. T. (2004). Nutrition of the intervertebral disc. *Spine*.

[5] Stokes I. A. F., Iatridis J. C. (2004). Mechanical conditions that accelerate intervertebral disc degeneration: overload versus immobilization. *Spine*.

[6] Battié M. C., Videman T. (2006). Lumbar disc degeneration: epidemiology and genetics. *The Journal of Bone and Joint Surgery A*.

[7] Zhao C.-Q., Wang L.-M., Jiang L.-S., Dai L.-Y. (2007). The cell biology of intervertebral disc aging and degeneration. *Ageing Research Reviews*.

[8] Boyd L. M., Carter A. J. (2006). Injectable biomaterials and vertebral endplate treatment for repair and regeneration of the intervertebral disc. *European Spine Journal*.

[9] Rajasekaran S., Venkatadass K., Naresh Babu J., Ganesh K., Shetty A. P. (2008). Pharmacological enhancement of disc diffusion and differentiation of healthy, ageing and degenerated discs: results from in-vivo serial post-contrast MRI studies in 365 human lumbar discs. *European Spine Journal*.

[10] Roberts S., Urban J. P. G., Evans H., Eisenstein S. M. (1996). Transport properties of the human cartilage endplate in relation to its composition and calcification. *Spine*.

[11] Peng B., Hou S., Shi Q., Jia L. (2001). The relationship between cartilage end-plate calcification and disc degeneration: an experimental study. *Chinese Medical Journal*.

[12] Antoniou J., Goudsouzian N. M., Heathfield T. F. (1996). The human lumbar endplate. Evidence of changes in biosynthesis and denaturation of the extracellular matrix with growth, maturation, aging, and degeneration. *Spine*.

[13] Pearce R. H., Grimmer B. J., Adams M. E. (1987). Degeneration and the chemical composition of the human lumbar intervertebral disc. *Journal of Orthopaedic Research*.

[14] Benneker L. M., Heini P. F., Alini M., Anderson S. E., Ito K. (2005). 2004 Young investigator award winner: vertebral endplate marrow contact channel occlusions and intervertebral disc degeneration. *Spine*.

[15] Smith L. J., Nerurkar N. L., Choi K.-S., Harfe B. D., Elliott D. M. (2011). Degeneration and regeneration of the intervertebral disc: Lessons from development. *DMM Disease Models and Mechanisms*.

[16] Pittenger M. F., Mackay A. M., Beck S. C. (1999). Multilineage potential of adult human mesenchymal stem cells. *Science*.

[17] Bajada S., Mazakova I., Richardson J. B., Ashammakhi N. (2008). Updates on stem cells and their applications in regenerative medicine. *Journal of Tissue Engineering and Regenerative Medicine*.

[18] Risbud M. V., Guttapalli A., Tsai T.-T. (2007). Evidence for skeletal progenitor cells in the degenerate human intervertebral disc. *Spine*.

[19] Sakaguchi Y., Sekiya I., Yagishita K., Muneta T. (2005). Comparison of human stem cells derived from various mesenchymal tissues: superiority of synovium as a cell source. *Arthritis and Rheumatism*.

[20] Bertolo A., Häfner S., Taddei A. R. (2015). Injectable microcarriers as human mesenchymal stem cell support and their application for cartilage and degenerated intervertebral disc repair. *European Cells and Materials*.

[21] Chung H. J., Kim I. K., Kim T. G., Park T. G. (2008). Highly open porous biodegradable microcarriers: in vitro cultivation of chondrocytes for injectable delivery. *Tissue Engineering Part A*.

[22] Hong Y., Gong Y., Gao C., Shen J. (2008). Collagen-coated polylactide microcarriers/chitosan hydrogel composite: injectable scaffold for cartilage regeneration. *Journal of Biomedical Materials Research A*.

[23] Declercq H. A., Gorski T. L., Tielens S. P., Schacht E. H., Cornelissen M. J. (2005). Encapsulation of osteoblast seeded microcarriers into injectable, photopolymerizable three-dimensional scaffolds based on D,L-lactide and *ε*-caprolactone. *Biomacromolecules*.

[24] Liu L.-T., Huang B., Li C.-Q., Zhuang Y., Wang J., Zhou Y. (2011). Characteristics of stem cells derived from the degenerated human intervertebral disc cartilage endplate. *PLoS ONE*.

[25] Blanco J. F., Graciani I. F., Sanchez-Guijo F. M. (2010). Isolation and characterization of mesenchymal stromal cells from human degenerated nucleus pulposus: comparison with bone marrow mesenchymal stromal cells from the same subjects. *Spine*.

[26] Black D. L. (2003). Mechanisms of alternative pre-messenger RNA splicing. *Annual Review of Biochemistry*.

[27] Blencowe B. J. (2006). Alternative splicing: new insights from global analyses. *Cell*.

[28] Pan Q., Shai O., Lee L. J., Frey B. J., Blencowe B. J. (2008). Deep surveying of alternative splicing complexity in the human transcriptome by high-throughput sequencing. *Nature Genetics*.

[29] Wang E. T., Sandberg R., Luo S. (2008). Alternative isoform regulation in human tissue transcriptomes. *Nature*.

[30] David C. J., Manley J. L. (2008). The search for alternative splicing regulators: new approaches offer a path to a splicing code. *Genes and Development*.

[31] Lukong K. E., Chang K.-W., Khandjian E. W., Richard S. (2008). RNA-binding proteins in human genetic disease. *Trends in Genetics*.

[32] Martinez-Contreras R., Cloutier P., Shkreta L., Fisette J. F., Revil T., Chabot B. (2007). hnRNP proteins and splicing control. *Advances in Experimental Medicine and Biology*.

[33] Ng B., Yang F., Huston D. P. (2004). Increased noncanonical splicing of autoantigen transcripts provides the structural basis for expression of untolerized epitopes. *The Journal of Allergy and Clinical Immunology*.

[34] Kazantseva J., Kivil A., Tints K., Kazantseva A., Neuman T., Palm K. (2013). Alternative splicing targeting the hTAF4-TAFH domain of TAF4 represses proliferation and accelerates chondrogenic differentiation of human mesenchymal stem cells. *PLoS ONE*.

[35] McAlinden A., Shim K.-H., Wirthlin L., Ravindran S., Hering T. M. (2012). Quantification of type II procollagen splice forms using alternative transcript-qPCR (AT-qPCR). *Matrix Biology*.

[36] Longo A., Librizzi M., Naselli F., Caradonna F., Tobiasch E., Luparello C. (2013). PTHrP in differentiating human mesenchymal stem cells: transcript isoform expression, promoter methylation, and protein accumulation. *Biochimie*.

[37] Thornemo M., Tallheden T., Jansson E. S. (2005). Clonal populations of chondrocytes with progenitor properties identified within human articular cartilage. *Cells Tissues Organs*.

[38] Purdom E., Simpson K. M., Robinson M. D., Conboy J. G., Lapuk A. V., Speed T. P. (2008). FIRMA: a method for detection of alternative splicing from exon array data. *Bioinformatics*.

[39] Srinivasan K., Shiue L., Hayes J. D. (2005). Detection and measurement of alternative splicing using splicing-sensitive microarrays. *Methods*.

[40] Clark T. A., Sugnet C. W., Ares M. (2002). Genomewide analysis of mRNA processing in yeast using splicing-specific microarrays. *Science*.

[41] Babadagli M. E., Tezcan B., Yilmaz S. T., Tufan A. C. (2014). Matrilin-3 as a putative effector of C-type natriuretic peptide signaling during TGF-beta induced chondrogenic differentiation of mesenchymal stem cells. *Molecular Biology Reports*.

[42] Fernandes A. M., Herlofsen S. R., Karlsen T. A., Küchler A. M., Fløisand Y., Brinchmann J. E. (2013). Similar properties of chondrocytes from osteoarthritis joints and mesenchymal stem cells from healthy donors for tissue engineering of articular cartilage. *PLoS ONE*.

[43] Herlofsen S. R., Bryne J. C., Høiby T. (2013). Genome-wide map of quantified epigenetic changes during in vitro chondrogenic differentiation of primary human mesenchymal stem cells. *BMC Genomics*.

[44] Weber M., Sotoca A. M., Kupfer P., Guthke R., van Zoelen E. J. (2013). Dynamic modelling of microRNA regulation during mesenchymal stem cell differentiation. *BMC Systems Biology*.

[45] Rodrigues R., Grosso A. R., Moita L. (2013). Genome-wide analysis of alternative splicing during dendritic cell response to a bacterial challenge. *PLoS ONE*.

[46] Herlofsen S. R., Küchler A. M., Melvik J. E., Brinchmann J. E. (2011). Chondrogenic differentiation of human bone marrow-derived mesenchymal stem cells in self-gelling alginate discs reveals novel chondrogenic signature gene clusters. *Tissue Engineering Part A*.

[47] Okamoto O., Fujiwara S. (2006). Dermatopontin, a novel player in the biology of the extracellular matrix. *Connective Tissue Research*.

[48] Rock M. J., Holden P., Horton W. A., Cohn D. H. (2010). Cartilage oligomeric matrix protein promotes cell attachment via two independent mechanisms involving CD47 and *α*v*β*3 integrin. *Molecular and Cellular Biochemistry*.

[49] Chen F. H., Herndon M. E., Patel N., Hecht J. T., Tuan R. S., Lawler J. (2007). Interaction of cartilage oligomeric matrix protein/thrombospondin 5 with aggrecan. *The Journal of Biological Chemistry*.

[50] Tan K., Duquette M., Joachimiak A., Lawler J. (2009). The crystal structure of the signature domain of cartilage oligomeric matrix protein: implications for collagen, glycosaminoglycan and integrin binding. *The FASEB Journal*.

[51] Pelttari K., Winter A., Steck E. (2006). Premature induction of hypertrophy during in vitro chondrogenesis of human mesenchymal stem cells correlates with calcification and vascular invasion after ectopic transplantation in SCID mice. *Arthritis & Rheumatism*.

[52] Yoo J. U., Barthel T. S., Nishimura K. (1998). The chondrogenic potential of human bone-marrow-derived mesenchymal progenitor cells. *The Journal of Bone and Joint Surgery A*.

[53] Johnstone B., Hering T. M., Caplan A. I., Goldberg V. M., Yoo J. U. (1998). In vitro chondrogenesis of bone marrow-derived mesenchymal progenitor cells. *Experimental Cell Research*.

[54] Sekiya I., Vuoristo J. T., Larson B. L., Prockop D. J. (2002). In vitro cartilage formation by human adult stem cells from bone marrow stroma defines the sequence of cellular and molecular events during chondrogenesis. *Proceedings of the National Academy of Sciences of the United States of America*.

[55] Mwale F., Girard-Lauriault P.-L., Wang H. T., Lerouge S., Antoniou J., Wertheimer M. R. (2006). Suppression of genes related to hypertrophy and osteogenesis in committed human mesenchymal stem cells cultured on novel nitrogen-rich plasma polymer coatings. *Tissue Engineering*.

[56] Mueller M. B., Tuan R. S. (2008). Functional characterization of hypertrophy in chondrogenesis of human mesenchymal stem cells. *Arthritis and Rheumatism*.

[57] Frisch J., Venkatesan J. K., Rey-Rico A., Schmitt G., Madry H., Cucchiarini M. (2014). Determination of the chondrogenic differentiation processes in human bone marrow-derived mesenchymal stem cells genetically modified to overexpress transforming growth factor-*β* via recombinant adeno-associated viral vectors. *Human Gene Therapy*.

[58] Handorf A. M., Chamberlain C. S., Li W. J. (2015). Endogenously produced Indian Hedgehog regulates TGF*β*-driven chondrogenesis of human bone marrow stromal/stem cells. *Stem Cells and Development*.

[59] Correa D., Somoza R. A., Lin P. (2015). Sequential exposure to fibroblast growth factors (FGF) 2, 9 and 18 enhances hMSC chondrogenic differentiation. *Osteoarthritis and Cartilage*.

[60] Shang J., Liu H., Li J., Zhou Y. (2014). Roles of hypoxia during the chondrogenic differentiation of mesenchymal stem cells. *Current Stem Cell Research & Therapy*.

[61] Petit V., Thiery J.-P. (2000). Focal adhesions: structure and dynamics. *Biology of the Cell*.

[62] Mitra S. K., Hanson D. A., Schlaepfer D. D. (2005). Focal adhesion kinase: in command and control of cell motility. *Nature Reviews Molecular Cell Biology*.

[63] Comoglio P. M., Boccaccio C., Trusolino L. (2003). Interactions between growth factor receptors and adhesion molecules: breaking the rules. *Current Opinion in Cell Biology*.

[64] Siegel P. M., Massagué J. (2003). Cytostatic and apoptotic actions of TGF-beta in homeostasis and cancer. *Nature Reviews Cancer*.

[65] Shi Y., Massagué J. (2003). Mechanisms of TGF-*β* signaling from cell membrane to the nucleus. *Cell*.

[66] Derynck R., Zhang Y. E. (2003). Smad-dependent and Smad-independent pathways in TGF-*β* family signalling. *Nature*.

[67] Li J., Wang J., Zou Y. (2012). The influence of delayed compressive stress on TGF-beta1-induced chondrogenic differentiation of rat BMSCs through Smad-dependent and Smad-independent pathways. *Biomaterials*.

[68] Kan Z., Rouchka E. C., Gish W. R., States D. J. (2001). Gene structure prediction and alternative splicing analysis using genomically aligned ESTs. *Genome Research*.

[69] Fujikake N., Nagai Y., Popiel H. A., Kano H., Yamaguchi M., Toda T. (2005). Alternative splicing regulates the transcriptional activity of *Drosophila* heat shock transcription factor in response to heat/cold stress. *FEBS Letters*.

[70] Sellers R. S., Luchin A. I., Richard V., Brena R. M., Lima D., Rosol T. J. (2004). Alternative splicing of parathyroid hormone-related protein mRNA: expression and stability. *Journal of Molecular Endocrinology*.

[71] Rosonina E., Bakowski M. A., McCracken S., Blencowe B. J. (2003). Transcriptional activators control splicing and 3′-end cleavage levels. *The Journal of Biological Chemistry*.

[72] Chen Z., Gibson T. B., Robinson F. (2001). MAP kinases. *Chemical Reviews*.

[73] Yang S.-H., Sharrocks A. D., Whitmarsh A. J. (2003). Transcriptional regulation by the MAP kinase signaling cascades. *Gene*.

[74] Tanoue T., Nishida E. (2002). Docking interactions in the mitogen-activated protein kinase cascades. *Pharmacology and Therapeutics*.

[75] Taylor W. R., Stark G. R. (2001). Regulation of the G2/M transition by p53. *Oncogene*.

[76] Levine A. J., Hu W., Feng Z. (2006). The P53 pathway: what questions remain to be explored?. *Cell Death & Differentiation*.

[77] Pietenpol J. A., Stewart Z. A. (2002). Cell cycle checkpoint signaling: cell cycle arrest versus apoptosis. *Toxicology*.

