# Supplementary material for: Global Gene Expression Profiling and Alternative Splicing Events during the Chondrogenic Differentiation of Human Cartilage Endplate-Derived Stem Cells
Source: Biomed Res Int. 2015 Nov 15;2015:604972. doi: 10.1155/2015/604972 (PMC4662983; doi:10.1155/2015/604972)
Supplement: Supplementary file 1 — Table S1: Primers for DEGs validated in chondrogenic differentiation of CESCs by RT-qPCR. Table S2: GO enrichment analysis of DEGs during chondrogenic differentiation of CESCs. Table S3: KEGG pathway results in DEGs during chondrogenic differentiation of CESCs. Table S4: ASGs detected during chondrogenic differentiation of CESCs. Table S5: Primers for ASGs validated in chondrogenic differentiation of CESCs by RT-PCR. Figure S6: RT-PCR result of GAPDH as internal control. Table S7: GO enrichment analysis of ASGs during chondrogenic differentiation of CESCs. Table S8: KEGG pathway results in ASGs during chondrogenic differentiation of CESCs. [file 604972.f1.pdf]

Gene symbol	Primers (5’ to 3’)
ANGPTL7	CTCCGCAAAGGTGGCTACT
	ACCCGTTTGAGGGAGTAGGT
DPT	ATGGAATGGTACCAGACGTGC
	CTGACTCGAAGTAGCGGCTC
RANBP3L	CCGTCACTAACCTCCCAACC
	ACAGGACCTTGTTCAGCACT
COL10A1	ACTCCCAGCACGCAGAATCC
	GGGCATTTGGTATCGTTCAGC
MMP7	AGTGGTCACCTACAGGATCG
	GGGATCTCTTTGCCCCACAT
COMP	GCTGTGGGTTACACTGCCT
	AAGCCCGCATAGTCGTCATC
CORIN	CAATCCCACAACAGAGCATCG
	CATCCTTGCAGTCCTCGTCA
CXCL14	TCATCACCACCAAGAGCGTG
	TTCCAGGCGTTGTACCACTT
ADAMTSL3	CCCTCCTGTGATAGAGACTGC
	TCAGCGACATGGGGAAAAGA
STC1	TGAAGTGGTTCGTTGCCTCA
	GACGAATGCTTTTCCCTGAGT
CP	CCGTTCTACAAAATGAAGACACCAA
	TTCCCCCACAAATGTACAAAGT
APLN	TCCGTCCAGTCCATCTTCCT
	AGATGAGACAGGCAGGGACT

Names	Genes_In_Term	DEG
Total	16490	671
GO:0001666//response to hypoxia	191	42
GO:0001944//vasculature development	355	57
GO:0007275//multicellular organismal development	3339	239
GO:0009653//anatomical structure morphogenesis	1573	150
GO:0032501//multicellular organismal process	5201	319
GO:0032502//developmental process	3759	262
GO:0036293//response to decreased oxygen levels	193	42
GO:0044707//single-multicellular organism process	4967	309
GO:0044767//single-organism developmental process	2824	207
GO:0048731//system development	2833	219
GO:0048856//anatomical structure development	3300	242
GO:0050793//regulation of developmental process	1333	124
GO:0051239//regulation of multicellular organismal process	1683	144
GO:0070482//response to oxygen levels	204	44
GO:0072358//cardiovascular system development	571	73
GO:0072359//circulatory system development	571	73
GO:0001568//blood vessel development	326	51
GO:0048513//organ development	2014	158
GO:2000026//regulation of multicellular organismal development	1024	99
GO:0009628//response to abiotic stimulus	722	79
GO:0042127//regulation of cell proliferation	1061	99
GO:0048514//blood vessel morphogenesis	268	43
GO:0050896//response to stimulus	6156	346
GO:0048468//cell development	1054	97
GO:0048646//anatomical structure formation involved in morphogenesis	604	68
GO:0009888//tissue development	1110	100
GO:0009605//response to external stimulus	885	86
GO:0030154//cell differentiation	2110	153
GO:0051094//positive regulation of developmental process	621	67
GO:0007155//cell adhesion	655	69
GO:0044699//single-organism process	10099	498
GO:0048519//negative regulation of biological process	2913	193
GO:0022610//biological adhesion	657	69
GO:0040011//locomotion	828	80
GO:0045595//regulation of cell differentiation	943	86
GO:0048869//cellular developmental process	2220	156
GO:0048518//positive regulation of biological process	3304	209
GO:0001501//skeletal system development	314	43
GO:0048583//regulation of response to stimulus	2414	164
GO:0040012//regulation of locomotion	429	51
GO:0048523//negative regulation of cellular process	2680	177
GO:0030334//regulation of cell migration	376	47
GO:2000145//regulation of cell motility	395	48
GO:0009719//response to endogenous stimulus	868	79
GO:0048522//positive regulation of cellular process	2907	187
GO:0030198//extracellular matrix organization	244	36
GO:0070887//cellular response to chemical stimulus	1508	115
GO:0043062//extracellular structure organization	245	36
GO:0008284//positive regulation of cell proliferation	602	61
GO:0051270//regulation of cellular component movement	439	50
GO:0007167//enzyme linked receptor protein signaling pathway	575	59
GO:0022603//regulation of anatomical structure morphogenesis	531	56
GO:0001503//ossification	139	26
GO:0048585//negative regulation of response to stimulus	758	70
GO:0030278//regulation of ossification	143	26
GO:0007399//nervous system development	1444	109

GO:1901700//response to oxygen-containing compound		836	74
GO:0006928//cellular component movement		923	79
GO:0065008//regulation of biological quality		2348	155
GO:0042221//response to chemical stimulus		2879	181
GO:0001525//angiogenesis		193	30
GO:0071453//cellular response to oxygen levels		62	17
GO:0009725//response to hormone stimulus		552	56
GO:0036294//cellular response to decreased oxygen levels		55	16
GO:0071456//cellular response to hypoxia		55	16
GO:0045597//positive regulation of cell differentiation		451	49
GO:0016477//cell migration		516	53
GO:0008015//blood circulation		236	33
GO:0032879//regulation of localization		1314	100
GO:0048705//skeletal system morphogenesis		143	25
GO:0003013//circulatory system process		238	33
GO:0009887//organ morphogenesis		614	59
GO:0032101//regulation of response to external stimulus		403	45
GO:0006950//response to stress		2592	165
GO:0010033//response to organic substance		1685	119
GO:0010906//regulation of glucose metabolic process		77	18
GO:0009790//embryo development		805	70
GO:0023051//regulation of signaling		1945	131
GO:0050727//regulation of inflammatory response		225	31
GO:0010646//regulation of cell communication		1949	131
GO:0022008//neurogenesis		959	78
GO:0007507//heart development		323	38
GO:0048736//appendage development		123	22
GO:0060173//limb development		123	22
GO:0071495//cellular response to endogenous stimulus		569	54
GO:0048870//cell motility		571	54
GO:0051674//localization of cell		571	54
GO:0060485//mesenchyme development		116	21
GO:0009966//regulation of signal transduction		1731	118
GO:0061448//connective tissue development		140	23
GO:0043067//regulation of programmed cell death		1022	80
GO:0010675//regulation of cellular carbohydrate metabolic process		98	19
GO:2000027//regulation of organ morphogenesis		130	22
GO:0051174//regulation of phosphorus metabolic process		1248	92
GO:0042981//regulation of apoptotic process		1011	79
GO:0048699//generation of neurons		890	72
GO:0033993//response to lipid		496	48
GO:0006109//regulation of carbohydrate metabolic process		102	19
GO:0008217//regulation of blood pressure		135	22
GO:0006935//chemotaxis		421	43
GO:0042330//taxis		421	43
GO:1901701//cellular response to oxygen-containing compound		485	47
GO:0010941//regulation of cell death		1055	80
GO:0010677//negative regulation of cellular carbohydrate metabolic proces		27	10
GO:1901342//regulation of vasculature development		154	23
GO:0019220//regulation of phosphate metabolic process		1236	89
GO:0071310//cellular response to organic substance		1181	86
GO:0001655//urogenital system development		246	30
GO:0000904//cell morphogenesis involved in differentiation		437	43
GO:0051246//regulation of protein metabolic process		1391	97
GO:0010243//response to organonitrogen compound		486	46
GO:0023056//positive regulation of signaling		805	65
GO:0010647//positive regulation of cell communication		806	65
GO:0009611//response to wounding		790	64

GO:0048660//regulation of smooth muscle cell proliferation	70	15
GO:0045667//regulation of osteoblast differentiation	90	17
GO:0010627//regulation of intracellular protein kinase cascade	654	56
GO:0042325//regulation of phosphorylation	815	65
GO:0048568//embryonic organ development	310	34
GO:0045912//negative regulation of carbohydrate metabolic process	30	10
GO:0032989//cellular component morphogenesis	647	55
GO:0061035//regulation of cartilage development	38	11
GO:0030155//regulation of cell adhesion	243	29
GO:0006957//complement activation, alternative pathway	18	8
GO:0043069//negative regulation of programmed cell death	550	49
GO:0051093//negative regulation of developmental process	518	47
GO:0072001//renal system development	217	27
GO:0003085//negative regulation of systemic arterial blood pressure	13	7
GO:0061298//retina vasculature development in camera-type eye	13	7
GO:0090287//regulation of cellular response to growth factor stimulus	129	20
GO:0045765//regulation of angiogenesis	142	21
GO:0007423//sensory organ development	380	38
GO:0001890//placenta development	119	19
GO:0043066//negative regulation of apoptotic process	544	48
GO:0051241//negative regulation of multicellular organismal process	323	34
GO:1901698//response to nitrogen compound	512	46
GO:0007169//transmembrane receptor protein tyrosine kinase signaling pat	432	41
GO:0060548//negative regulation of cell death	571	49
GO:0044763//single-organism cellular process	8996	427
GO:0001932//regulation of protein phosphorylation	763	60
GO:0042060//wound healing	457	42
GO:0043408//regulation of MAPK cascade	426	40
GO:0002347//response to tumor cell	10	6
GO:0045820//negative regulation of glycolysis	10	6
GO:0071363//cellular response to growth factor stimulus	364	36
GO:0008285//negative regulation of cell proliferation	476	43
GO:0048666//neuron development	543	47
GO:0048634//regulation of muscle organ development	82	15
GO:0035295//tube development	382	37
GO:0007411//axon guidance	247	28
GO:0097485//neuron projection guidance	247	28
GO:0043009//chordate embryonic development	462	42
GO:0035107//appendage morphogenesis	116	18
GO:0035108//limb morphogenesis	116	18
GO:0000902//cell morphogenesis	597	50
GO:0051147//regulation of muscle cell differentiation	84	15
GO:0080134//regulation of response to stress	723	57
GO:0043410//positive regulation of MAPK cascade	295	31
GO:0009792//embryo development ending in birth or egg hatching	468	42
GO:0050678//regulation of epithelial cell proliferation	182	23
GO:0048729//tissue morphogenesis	420	39
GO:0060284//regulation of cell development	420	39
GO:0032102//negative regulation of response to external stimulus	131	19
GO:0048812//neuron projection morphogenesis	358	35
GO:0051130//positive regulation of cellular component organization	454	41
GO:0007178//transmembrane receptor protein serine/threonine kinase sign	132	19
GO:0048661//positive regulation of smooth muscle cell proliferation	46	11
GO:0030182//neuron differentiation	659	53
GO:0009893//positive regulation of metabolic process	1809	113
GO:0009967//positive regulation of signal transduction	768	59
GO:0030449//regulation of complement activation	38	10
GO:2000257//regulation of protein activation cascade	38	10

GO:0044710//single-organism metabolic process	2708	156
GO:0070848//response to growth factor stimulus	378	36
GO:0048598//embryonic morphogenesis	427	39
GO:0023057//negative regulation of signaling	629	51
GO:0010648//negative regulation of cell communication	632	51
GO:0009968//negative regulation of signal transduction	599	49
GO:0010562//positive regulation of phosphorus metabolic process	600	49
GO:0045937//positive regulation of phosphate metabolic process	600	49
GO:0048667//cell morphogenesis involved in neuron differentiation	352	34
GO:0010604//positive regulation of macromolecule metabolic process	1664	105
GO:0002009//morphogenesis of an epithelium	322	32
GO:0060429//epithelium development	620	50
GO:0048771//tissue remodeling	79	14
GO:0030199//collagen fibril organization	40	10
GO:0045669//positive regulation of osteoblast differentiation	49	11
GO:0010740//positive regulation of intracellular protein kinase cascade	468	41
GO:0048608//reproductive structure development	220	25
GO:0061458//reproductive system development	220	25
GO:0070613//regulation of protein processing	59	12
GO:0061564//axon development	340	33
GO:0031175//neuron projection development	438	39
GO:0007409//axonogenesis	326	32
GO:0051247//positive regulation of protein metabolic process	804	60
GO:0006110//regulation of glycolysis	25	8
GO:0032330//regulation of chondrocyte differentiation	25	8
GO:0048584//positive regulation of response to stimulus	1201	81
GO:1901652//response to peptide	266	28
GO:0016202//regulation of striated muscle tissue development	81	14
GO:1901861//regulation of muscle tissue development	81	14
GO:0040007//growth	282	29
GO:0001822//kidney development	169	21
GO:0051128//regulation of cellular component organization	1113	76
GO:0001562//response to protozoan	19	7
GO:0051240//positive regulation of multicellular organismal process	498	42
GO:0050728//negative regulation of inflammatory response	73	13
GO:0001701//in utero embryonic development	288	29
GO:0001654//eye development	243	26
GO:0031667//response to nutrient levels	214	24
GO:0048762//mesenchymal cell differentiation	96	15
GO:0055123//digestive system development	96	15
GO:0045732//positive regulation of protein catabolic process	85	14
GO:0060541//respiratory system development	147	19
GO:0009991//response to extracellular stimulus	232	25
GO:0051716//cellular response to stimulus	4615	238
GO:0042327//positive regulation of phosphorylation	523	43
GO:0030323//respiratory tube development	136	18
GO:0048863//stem cell differentiation	205	23
GO:0048732//gland development	221	24
GO:0046683//response to organophosphorus	77	13
GO:0001958//endochondral ossification	21	7
GO:0036075//replacement ossification	21	7
GO:0001934//positive regulation of protein phosphorylation	512	42
GO:0048565//digestive tract development	89	14
GO:0006022//aminoglycan metabolic process	140	18
GO:0030574//collagen catabolic process	57	11
GO:2000736//regulation of stem cell differentiation	57	11
GO:0048878//chemical homeostasis	551	44
GO:0048844//artery morphogenesis	38	9

GO:0060322//head development	38	9
GO:0043434//response to peptide hormone stimulus	257	26
GO:0060324//face development	30	8
GO:0003073//regulation of systemic arterial blood pressure	69	12
GO:0044706//multi-multicellular organism process	170	20
GO:0010563//negative regulation of phosphorus metabolic process	258	26
GO:0045936//negative regulation of phosphate metabolic process	258	26
GO:0050679//positive regulation of epithelial cell proliferation	105	15
GO:0051216//cartilage development	105	15
GO:0042692//muscle cell differentiation	200	22
GO:0009607//response to biotic stimulus	524	42
GO:0031399//regulation of protein modification process	927	64
GO:0007417//central nervous system development	578	45
GO:0043470//regulation of carbohydrate catabolic process	31	8
GO:0043471//regulation of cellular carbohydrate catabolic process	31	8
GO:0042592//homeostatic process	873	61
GO:2000147//positive regulation of cell motility	231	24
GO:0044057//regulation of system process	390	34
GO:0090101//negative regulation of transmembrane receptor protein serine	71	12
GO:0050789//regulation of biological process	8567	400
GO:0030324//lung development	133	17
GO:0009892//negative regulation of metabolic process	1327	84
GO:0051272//positive regulation of cellular component movement	234	24
GO:0060350//endochondral bone morphogenesis	32	8
GO:0002376//immune system process	1675	101
GO:0014031//mesenchymal cell development	84	13
GO:0060349//bone morphogenesis	51	10
GO:0060325//face morphogenesis	24	7
GO:0045598//regulation of fat cell differentiation	62	11
GO:0090092//regulation of transmembrane receptor protein serine/threonin	149	18
GO:0071560//cellular response to transforming growth factor beta stimulus	97	14
GO:0065007//biological regulation	9063	419
GO:0005996//monosaccharide metabolic process	192	21
GO:0007568//aging	136	17
GO:0001957//intramembranous ossification	6	4
GO:0002418//immune response to tumor cell	6	4
GO:0036072//direct ossification	6	4
GO:0061299//retina vasculature morphogenesis in camera-type eye	6	4
GO:0044703//multi-organism reproductive process	178	20
GO:0042176//regulation of protein catabolic process	150	18
GO:0050730//regulation of peptidyl-tyrosine phosphorylation	150	18
GO:0042698//ovulation cycle	74	12
GO:0060348//bone development	74	12
GO:0040017//positive regulation of locomotion	238	24
GO:0071559//response to transforming growth factor beta stimulus	98	14
GO:0031325//positive regulation of cellular metabolic process	1708	102
GO:0043549//regulation of kinase activity	593	45
GO:0031347//regulation of defense response	436	36
GO:0044243//multicellular organismal catabolic process	64	11
GO:0048511//rhythmic process	139	17
GO:0048545//response to steroid hormone stimulus	241	24
GO:0060711//labyrinthine layer development	34	8
GO:0002673//regulation of acute inflammatory response	76	12
GO:0030335//positive regulation of cell migration	227	23
GO:0060537//muscle tissue development	212	22
GO:0051049//regulation of transport	974	65
GO:0045859//regulation of protein kinase activity	563	43
GO:0031324//negative regulation of cellular metabolic process	1232	78

GO:0043010//camera-type eye development	213	22
GO:0014070//response to organic cyclic compound	477	38
GO:0014706//striated muscle tissue development	199	21
GO:0007154//cell communication	4207	215
GO:0010171//body morphogenesis	35	8
GO:0070542//response to fatty acid	35	8
GO:0051153//regulation of striated muscle cell differentiation	55	10
GO:2000377//regulation of reactive oxygen species metabolic process	55	10
GO:0002920//regulation of humoral immune response	66	11
GO:0003006//developmental process involved in reproduction	375	32
GO:0071417//cellular response to organonitrogen compound	277	26
GO:0032332//positive regulation of chondrocyte differentiation	12	5
GO:0060840//artery development	45	9
GO:0032963//collagen metabolic process	78	12
GO:0045766//positive regulation of angiogenesis	78	12
GO:0006007//glucose catabolic process	56	10
GO:0060323//head morphogenesis	27	7
GO:0043583//ear development	158	18
GO:0042326//negative regulation of phosphorylation	202	21
GO:0065009//regulation of molecular function	1758	103
GO:0030203//glycosaminoglycan metabolic process	131	16
GO:0010605//negative regulation of macromolecule metabolic process	1226	77
GO:0007596//blood coagulation	380	32
GO:0050817//coagulation	380	32
GO:0060174//limb bud formation	7	4
GO:0022617//extracellular matrix disassembly	68	11
GO:0051338//regulation of transferase activity	613	45
GO:0050900//leukocyte migration	190	20
GO:0043065//positive regulation of apoptotic process	366	31
GO:0007599//hemostasis	383	32
GO:0022602//ovulation cycle process	69	11
GO:0009952//anterior/posterior pattern specification	147	17
GO:0070167//regulation of biomineral tissue development	58	10
GO:0031348//negative regulation of defense response	94	13
GO:0003002//regionalization	238	23
GO:0008585//female gonad development	70	11
GO:0045995//regulation of embryonic development	82	12
GO:0055114//oxidation-reduction process	767	53
GO:0031214//biomineral tissue development	48	9
GO:0051336//regulation of hydrolase activity	787	54
GO:0043068//positive regulation of programmed cell death	370	31
GO:0007584//response to nutrient	108	14
GO:0048701//embryonic cranial skeleton morphogenesis	29	7
GO:0061061//muscle structure development	321	28
GO:0021700//developmental maturation	136	16
GO:0014066//regulation of phosphatidylinositol 3-kinase cascade	49	9
GO:0050790//regulation of catalytic activity	1428	86
GO:0030168//platelet activation	151	17
GO:0006111//regulation of gluconeogenesis	21	6
GO:0040013//negative regulation of locomotion	137	16
GO:0044259//multicellular organismal macromolecule metabolic process	84	12
GO:0019318//hexose metabolic process	166	18
GO:0048858//cell projection morphogenesis	462	36
GO:0007565//female pregnancy	138	16
GO:0046849//bone remodeling	30	7
GO:2000278//regulation of DNA biosynthetic process	14	5
GO:0003231//cardiac ventricle development	73	11
GO:0001913//T cell mediated cytotoxicity	8	4

GO:2000573//positive regulation of DNA biosynthetic process	8	4
GO:NM_0059	8	4
GO:0009612//response to mechanical stimulus	125	15
GO:0016043//cellular component organization	3343	174
GO:0045860//positive regulation of protein kinase activity	361	30
GO:0048589//developmental growth	154	17
GO:1901699//cellular response to nitrogen compound	295	26
GO:0051707//response to other organism	503	38
GO:0023052//signaling	4097	207
GO:0044700//single organism signaling	4097	207
GO:0048641//regulation of skeletal muscle tissue development	51	9
GO:0030282//bone mineralization	31	7
GO:0045776//negative regulation of blood pressure	31	7
GO:0050819//negative regulation of coagulation	31	7
GO:0033674//positive regulation of kinase activity	381	31
GO:0006096//glycolysis	41	8
GO:0014068//positive regulation of phosphatidylinositol 3-kinase cascade	41	8
GO:0045807//positive regulation of endocytosis	63	10
GO:0010942//positive regulation of cell death	382	31
GO:0031401//positive regulation of protein modification process	635	45
GO:0030336//negative regulation of cell migration	114	14
GO:0006979//response to oxidative stress	233	22
GO:0045087//innate immune response	525	39
GO:0032844//regulation of homeostatic process	250	23
GO:0002076//osteoblast development	15	5
GO:0072012//glomerulus vasculature development	15	5
GO:0050731//positive regulation of peptidyl-tyrosine phosphorylation	115	14
GO:0006959//humoral immune response	129	15
GO:0007173//epidermal growth factor receptor signaling pathway	129	15
GO:0043589//skin morphogenesis	32	7
GO:0048546//digestive tract morphogenesis	42	8
GO:0032990//cell part morphogenesis	474	36
GO:0003205//cardiac chamber development	89	12
GO:0009743//response to carbohydrate stimulus	89	12
GO:0048562//embryonic organ morphogenesis	204	20
GO:0030500//regulation of bone mineralization	53	9
GO:0070555//response to interleukin-1	53	9
GO:0038127//ERBB signaling pathway	130	15
GO:0042445//hormone metabolic process	145	16
GO:0044236//multicellular organismal metabolic process	90	12
GO:0010817//regulation of hormone levels	190	19
GO:2000146//negative regulation of cell motility	117	14
GO:0044344//cellular response to fibroblast growth factor stimulus	131	15
GO:1901135//carbohydrate derivative metabolic process	1072	67
GO:0043467//regulation of generation of precursor metabolites and energy	54	9
GO:0090130//tissue migration	54	9
GO:0061138//morphogenesis of a branching epithelium	146	16
GO:0017015//regulation of transforming growth factor beta receptor signalir	78	11
GO:0048566//embryonic digestive tract development	33	7
GO:0071774//response to fibroblast growth factor stimulus	132	15
GO:0048469//cell maturation	91	12
GO:0060828//regulation of canonical Wnt receptor signaling pathway	118	14
GO:0048260//positive regulation of receptor-mediated endocytosis	24	6
GO:0051347//positive regulation of transferase activity	391	31
GO:0009896//positive regulation of catabolic process	147	16
GO:0061437//renal system vasculature development	16	5
GO:0061440//kidney vasculature development	16	5
GO:0008406//gonad development	162	17

GO:0048864//stem cell development	162	17
GO:0071900//regulation of protein serine/threonine kinase activity	340	28
GO:0005975//carbohydrate metabolic process	648	45
GO:0007292//female gamete generation	79	11
GO:0019216//regulation of lipid metabolic process	193	19
GO:0048706//embryonic skeletal system development	92	12
GO:0050878//regulation of body fluid levels	464	35
GO:0001892//embryonic placenta development	67	10
GO:0060191//regulation of lipase activity	106	13
GO:0051223//regulation of protein transport	291	25
GO:0006692//prostanoid metabolic process	25	6
GO:0006693//prostaglandin metabolic process	25	6
GO:0043666//regulation of phosphoprotein phosphatase activity	25	6
GO:0048008//platelet-derived growth factor receptor signaling pathway	25	6
GO:0035272//exocrine system development	45	8
GO:0010810//regulation of cell-substrate adhesion	94	12
GO:0030858//positive regulation of epithelial cell differentiation	35	7
GO:0071347//cellular response to interleukin-1	35	7
GO:0032870//cellular response to hormone stimulus	329	27
GO:0043588//skin development	213	20
GO:0019320//hexose catabolic process	69	10
GO:0033688//regulation of osteoblast proliferation	17	5
GO:0060445//branching involved in salivary gland morphogenesis	17	5
GO:0031646//positive regulation of neurological system process	57	9
GO:0043255//regulation of carbohydrate biosynthetic process	57	9
GO:0032880//regulation of protein localization	364	29
GO:0032270//positive regulation of cellular protein metabolic process	715	48
GO:0030638//polyketide metabolic process	10	4
GO:2000505//regulation of energy homeostasis	10	4
GO:0001763//morphogenesis of a branching structure	152	16
GO:0051146//striated muscle cell differentiation	152	16
GO:0010976//positive regulation of neuron projection development	46	8
GO:0051271//negative regulation of cellular component movement	123	14
GO:0015711//organic anion transport	263	23
GO:0001775//cell activation	491	36
GO:0014902//myotube differentiation	26	6
GO:0035567//non-canonical Wnt receptor signaling pathway	26	6
GO:0003007//heart morphogenesis	153	16
GO:0007435//salivary gland morphogenesis	36	7
GO:0048259//regulation of receptor-mediated endocytosis	36	7
GO:0044723//single-organism carbohydrate metabolic process	457	34
GO:0045596//negative regulation of cell differentiation	421	32
GO:0022414//reproductive process	898	57
GO:0019222//regulation of metabolic process	4734	231
GO:0045137//development of primary sexual characteristics	186	18
GO:0060137//maternal process involved in parturition	5	3
GO:0072103//glomerulus vasculature morphogenesis	5	3
GO:0072104//glomerular capillary formation	5	3
GO:0002053//positive regulation of mesenchymal cell proliferation	27	6
GO:0043405//regulation of MAP kinase activity	235	21
GO:0007179//transforming growth factor beta receptor signaling pathway	72	10
GO:0006721//terpenoid metabolic process	85	11
GO:0046545//development of primary female sexual characteristics	85	11
GO:0010631//epithelial cell migration	48	8
GO:0090132//epithelium migration	48	8
GO:0007165//signal transduction	3681	185
GO:0070201//regulation of establishment of protein localization	320	26
GO:0030326//embryonic limb morphogenesis	99	12

GO:0035113//embryonic appendage morphogenesis	99	12
GO:0045785//positive regulation of cell adhesion	113	13
GO:0006024//glycosaminoglycan biosynthetic process	86	11
GO:0051896//regulation of protein kinase B signaling cascade	86	11
GO:2000725//regulation of cardiac muscle cell differentiation	11	4
GO:0016101//diterpenoid metabolic process	73	10
GO:0001933//negative regulation of protein phosphorylation	190	18
GO:0030195//negative regulation of blood coagulation	28	6
GO:1900047//negative regulation of hemostasis	28	6
GO:0006023//aminoglycan biosynthetic process	87	11
GO:0060071//Wnt receptor signaling pathway, planar cell polarity pathway	19	5
GO:0090175//regulation of establishment of planar polarity	19	5
GO:0046365//monosaccharide catabolic process	74	10
GO:0072006//nephron development	74	10
GO:0032268//regulation of cellular protein metabolic process	1175	70
GO:0071902//positive regulation of protein serine/threonine kinase activity	207	19
GO:0010720//positive regulation of cell development	130	14
GO:0002088//lens development in camera-type eye	50	8
GO:0060688//regulation of morphogenesis of a branching structure	50	8
GO:0001942//hair follicle development	62	9
GO:0030856//regulation of epithelial cell differentiation	88	11
GO:0031960//response to corticosteroid stimulus	88	11
GO:0046660//female sex differentiation	88	11
GO:0042063//gliogenesis	116	13
GO:0007431//salivary gland development	39	7
GO:0043542//endothelial cell migration	39	7
GO:0001656//metanephros development	75	10
GO:0006690//icosanoid metabolic process	75	10
GO:1901568//fatty acid derivative metabolic process	75	10
GO:0045787//positive regulation of cell cycle	102	12
GO:0051222//positive regulation of protein transport	177	17
GO:0006357//regulation of transcription from RNA polymerase II promoter	1120	67
GO:0044246//regulation of multicellular organismal metabolic process	29	6
GO:0009895//negative regulation of catabolic process	89	11
GO:0010632//regulation of epithelial cell migration	89	11
GO:0022404//molting cycle process	63	9
GO:0022405//hair cycle process	63	9
GO:0006112//energy reserve metabolic process	103	12
GO:0071496//cellular response to external stimulus	147	15
GO:0016055//Wnt receptor signaling pathway	132	14
GO:0014032//neural crest cell development	51	8
GO:0051971//positive regulation of transmission of nerve impulse	51	8
GO:0034284//response to monosaccharide stimulus	76	10
GO:0061041//regulation of wound healing	76	10
GO:0080135//regulation of cellular response to stress	277	23
GO:0044281//small molecule metabolic process	2121	114
GO:0000122//negative regulation of transcription from RNA polymerase II p	455	33
GO:0050794//regulation of cellular process	8119	370
GO:0080090//regulation of primary metabolic process	4221	207
GO:0002228//natural killer cell mediated immunity	20	5
GO:0010660//regulation of muscle cell apoptotic process	20	5
GO:0030325//adrenal gland development	20	5
GO:0042267//natural killer cell mediated cytotoxicity	20	5
GO:0060412//ventricular septum morphogenesis	20	5
GO:0060612//adipose tissue development	20	5
GO:0007548//sex differentiation	211	19
GO:0048742//regulation of skeletal muscle fiber development	40	7
GO:0010463//mesenchymal cell proliferation	12	4

GO:0010656//negative regulation of muscle cell apoptotic process	12	4
GO:0090257//regulation of muscle system process	104	12
GO:0045216//cell-cell junction organization	119	13
GO:0051591//response to cAMP	52	8
GO:0071840//cellular component organization or biogenesis	3471	174
GO:0003281//ventricular septum development	30	6
GO:0015701//bicarbonate transport	30	6
GO:0007389//pattern specification process	350	27
GO:0014074//response to purine-containing compound	91	11
GO:0003151//outflow tract morphogenesis	41	7
GO:0003206//cardiac chamber morphogenesis	78	10
GO:0006091//generation of precursor metabolites and energy	316	25
GO:0060346//bone trabecula formation	6	3
GO:0060462//lung lobe development	6	3
GO:0060463//lung lobe morphogenesis	6	3
GO:0061438//renal system vasculature morphogenesis	6	3
GO:0061439//kidney vasculature morphogenesis	6	3
GO:2000543//positive regulation of gastrulation	6	3
GO:0030111//regulation of Wnt receptor signaling pathway	166	16
GO:0043406//positive regulation of MAP kinase activity	166	16
GO:0051046//regulation of secretion	425	31
GO:0055082//cellular chemical homeostasis	335	26
GO:0008543//fibroblast growth factor receptor signaling pathway	121	13
GO:0009987//cellular process	11733	512
GO:0003401//axis elongation	21	5
GO:0010718//positive regulation of epithelial to mesenchymal transition	21	5
GO:0032965//regulation of collagen biosynthetic process	21	5
GO:0048839//inner ear development	136	14
GO:0032868//response to insulin stimulus	167	16
GO:0051704//multi-organism process	1053	63
GO:0010517//regulation of phospholipase activity	79	10
GO:0042303//molting cycle	66	9
GO:0042633//hair cycle	66	9
GO:0001678//cellular glucose homeostasis	31	6
GO:0002040//sprouting angiogenesis	31	6
GO:0008544//epidermis development	184	17
GO:0051050//positive regulation of transport	446	32
GO:0042149//cellular response to glucose starvation	13	4
GO:0051154//negative regulation of striated muscle cell differentiation	13	4
GO:0001667//ameboidal cell migration	108	12
GO:0003018//vascular process in circulatory system	80	10
GO:0006937//regulation of muscle contraction	94	11
GO:0006820//anion transport	339	26
GO:1901564//organonitrogen compound metabolic process	1415	80
GO:0071396//cellular response to lipid	219	19
GO:0050795//regulation of behavior	139	14
GO:0003279//cardiac septum development	43	7
GO:0001570//vasculogenesis	55	8
GO:0007204//elevation of cytosolic calcium ion concentration	124	13
GO:0010712//regulation of collagen metabolic process	22	5
GO:0010464//regulation of mesenchymal cell proliferation	32	6
GO:0045429//positive regulation of nitric oxide biosynthetic process	32	6
GO:0055024//regulation of cardiac muscle tissue development	32	6
GO:0060411//cardiac septum morphogenesis	32	6
GO:0051384//response to glucocorticoid stimulus	82	10
GO:0030030//cell projection organization	644	42
GO:0034330//cell junction organization	141	14
GO:0043436//oxoacid metabolic process	865	53

GO:0002062//chondrocyte differentiation	33	6
GO:0016486//peptide hormone processing	33	6
GO:0019748//secondary metabolic process	33	6
GO:0035914//skeletal muscle cell differentiation	33	6
GO:0006952//defense response	1049	62
GO:0044724//single-organism carbohydrate catabolic process	97	11
GO:0031669//cellular response to nutrient levels	83	10
GO:0032314//regulation of Rac GTPase activity	14	4
GO:0038084//vascular endothelial growth factor signaling pathway	14	4
GO:0046629//gamma-delta T cell activation	14	4
GO:0048557//embryonic digestive tract morphogenesis	14	4
GO:0060713//labyrinthine layer morphogenesis	14	4
GO:0070168//negative regulation of biomineral tissue development	14	4
GO:0000003//reproduction	989	59
GO:0048010//vascular endothelial growth factor receptor signaling pathway	23	5
GO:0009891//positive regulation of biosynthetic process	1155	67
GO:0001523//retinoid metabolic process	70	9
GO:0010518//positive regulation of phospholipase activity	70	9
GO:0016337//cell-cell adhesion	293	23
GO:0010665//regulation of cardiac muscle cell apoptotic process	7	3
GO:0019511//peptidyl-proline hydroxylation	7	3
GO:0032317//regulation of Rap GTPase activity	7	3
GO:0032516//positive regulation of phosphoprotein phosphatase activity	7	3
GO:0042730//fibrinolysis	7	3
GO:0045091//regulation of retroviral genome replication	7	3
GO:0072102//glomerulus morphogenesis	7	3
GO:2000737//negative regulation of stem cell differentiation	7	3
GO:0035270//endocrine system development	98	11
GO:0010717//regulation of epithelial to mesenchymal transition	34	6
GO:0010863//positive regulation of phospholipase C activity	58	8
GO:0051179//localization	3502	173
GO:0033559//unsaturated fatty acid metabolic process	85	10
GO:0051248//negative regulation of protein metabolic process	424	30
GO:0051960//regulation of nervous system development	369	27
GO:0045600//positive regulation of fat cell differentiation	24	5
GO:0048599//oocyte development	24	5
GO:0007519//skeletal muscle tissue development	100	11
GO:0060326//cell chemotaxis	100	11
GO:0007166//cell surface receptor signaling pathway	2334	121
GO:0006956//complement activation	86	10
GO:0014033//neural crest cell differentiation	59	8
GO:0035239//tube morphogenesis	263	21
GO:0002686//negative regulation of leukocyte migration	15	4
GO:0071398//cellular response to fatty acid	15	4
GO:0006874//cellular calcium ion homeostasis	195	17
GO:0045685//regulation of glial cell differentiation	35	6
GO:0006006//glucose metabolic process	131	13
GO:0006720//isoprenoid metabolic process	101	11
GO:0019932//second-messenger-mediated signaling	101	11
GO:0031668//cellular response to extracellular stimulus	101	11
GO:0050708//regulation of protein secretion	147	14
GO:0019369//arachidonic acid metabolic process	47	7
GO:0030512//negative regulation of transforming growth factor beta recepto	47	7
GO:0030855//epithelial cell differentiation	372	27
GO:0006082//organic acid metabolic process	880	53
GO:0006898//receptor-mediated endocytosis	87	10
GO:0050818//regulation of coagulation	60	8
GO:0006869//lipid transport	164	15

GO:0016052//carbohydrate catabolic process	102	11
GO:0048011//neurotrophin TRK receptor signaling pathway	197	17
GO:0051173//positive regulation of nitrogen compound metabolic process	1068	62
GO:0009994//oocyte differentiation	25	5
GO:0072028//nephron morphogenesis	25	5
GO:0035051//cardiocyte differentiation	74	9
GO:0051480//cytosolic calcium ion homeostasis	133	13
GO:0048286//lung alveolus development	36	6
GO:0038179//neurotrophin signaling pathway	198	17
GO:0050806//positive regulation of synaptic transmission	48	7
GO:0072376//protein activation cascade	103	11
GO:0060255//regulation of macromolecule metabolic process	4012	194
GO:0060562//epithelial tube morphogenesis	250	20
GO:0003188//heart valve formation	8	3
GO:0003264//regulation of cardioblast proliferation	8	3
GO:0003266//regulation of secondary heart field cardioblast proliferation	8	3
GO:0010662//regulation of striated muscle cell apoptotic process	8	3
GO:0032487//regulation of Rap protein signal transduction	8	3
GO:0035413//positive regulation of catenin import into nucleus	8	3
GO:0045721//negative regulation of gluconeogenesis	8	3
GO:0060426//lung vasculature development	8	3
GO:0060484//lung-associated mesenchyme development	8	3
GO:0060670//branching involved in labyrinthine layer morphogenesis	8	3
GO:0061430//bone trabecula morphogenesis	8	3
GO:0045444//fat cell differentiation	75	9
GO:0090090//negative regulation of canonical Wnt receptor signaling pathw	75	9
GO:0031328//positive regulation of cellular biosynthetic process	1137	65
GO:0051150//regulation of smooth muscle cell differentiation	16	4
GO:0060669//embryonic placenta morphogenesis	16	4
GO:0072273//metanephric nephron morphogenesis	16	4
GO:0016051//carbohydrate biosynthetic process	104	11
GO:0043200//response to amino acid stimulus	62	8
GO:0048704//embryonic skeletal system morphogenesis	62	8
GO:1900274//regulation of phospholipase C activity	62	8
GO:0003044//regulation of systemic arterial blood pressure mediated by a c	37	6
GO:0050886//endocrine process	37	6
GO:0010628//positive regulation of gene expression	973	57
GO:0022411//cellular component disassembly	184	16
GO:0002682//regulation of immune system process	994	58
GO:0014910//regulation of smooth muscle cell migration	26	5
GO:0042517//positive regulation of tyrosine phosphorylation of Stat3 protei	26	5
GO:0072210//metanephric nephron development	26	5
GO:0070372//regulation of ERK1 and ERK2 cascade	120	12
GO:0019725//cellular homeostasis	417	29
GO:0030178//negative regulation of Wnt receptor signaling pathway	105	11
GO:0072503//cellular divalent inorganic cation homeostasis	202	17
GO:0044093//positive regulation of molecular function	1039	60
GO:0033500//carbohydrate homeostasis	91	10
GO:0034612//response to tumor necrosis factor	91	10
GO:0042593//glucose homeostasis	91	10
GO:0060041//retina development in camera-type eye	91	10
GO:0042493//response to drug	290	22
GO:0030100//regulation of endocytosis	106	11
GO:0050920//regulation of chemotaxis	106	11
GO:0001649//osteoblast differentiation	50	7
GO:0050654//chondroitin sulfate proteoglycan metabolic process	50	7
GO:0090183//regulation of kidney development	50	7
GO:0060193//positive regulation of lipase activity	77	9

GO:0030029//actin filament-based process	291	22
GO:0006081//cellular aldehyde metabolic process	38	6
GO:0008206//bile acid metabolic process	38	6
GO:0045840//positive regulation of mitosis	38	6
GO:0051785//positive regulation of nuclear division	38	6
GO:0032869//cellular response to insulin stimulus	138	13
GO:0060538//skeletal muscle organ development	107	11
GO:0001909//leukocyte mediated cytotoxicity	27	5
GO:0045445//myoblast differentiation	27	5
GO:0045604//regulation of epidermal cell differentiation	27	5
GO:0071322//cellular response to carbohydrate stimulus	27	5
GO:0001502//cartilage condensation	17	4
GO:0030728//ovulation	17	4
GO:0034694//response to prostaglandin stimulus	17	4
GO:0035909//aorta morphogenesis	17	4
GO:0045453//bone resorption	17	4
GO:0048710//regulation of astrocyte differentiation	17	4
GO:0006687//glycosphingolipid metabolic process	64	8
GO:0031323//regulation of cellular metabolic process	4250	203
GO:0006029//proteoglycan metabolic process	78	9
GO:0055074//calcium ion homeostasis	205	17
GO:0031330//negative regulation of cellular catabolic process	51	7
GO:0060341//regulation of cellular localization	637	40
GO:0007050//cell cycle arrest	108	11
GO:0010921//regulation of phosphatase activity	93	10
GO:0030036//actin cytoskeleton organization	258	20
GO:0001541//ovarian follicle development	39	6
GO:0010883//regulation of lipid storage	39	6
GO:0030509//BMP signaling pathway	39	6
GO:0010565//regulation of cellular ketone metabolic process	124	12
GO:0014812//muscle cell migration	9	3
GO:0014829//vascular smooth muscle contraction	9	3
GO:0019852//L-ascorbic acid metabolic process	9	3
GO:0030647//aminoglycoside antibiotic metabolic process	9	3
GO:0034383//low-density lipoprotein particle clearance	9	3
GO:0044597//daunorubicin metabolic process	9	3
GO:0044598//doxorubicin metabolic process	9	3
GO:0045986//negative regulation of smooth muscle contraction	9	3
GO:0048617//embryonic foregut morphogenesis	9	3
GO:0048711//positive regulation of astrocyte differentiation	9	3
GO:0048715//negative regulation of oligodendrocyte differentiation	9	3
GO:0061036//positive regulation of cartilage development	9	3
GO:NR_0014	9	3
GO:0010035//response to inorganic substance	259	20
GO:0051897//positive regulation of protein kinase B signaling cascade	65	8
GO:0031346//positive regulation of cell projection organization	109	11
GO:0045786//negative regulation of cell cycle	191	16
GO:0006940//regulation of smooth muscle contraction	40	6
GO:0051149//positive regulation of muscle cell differentiation	40	6
GO:0001516//prostaglandin biosynthetic process	18	4
GO:0002090//regulation of receptor internalization	18	4
GO:0010714//positive regulation of collagen metabolic process	18	4
GO:0032967//positive regulation of collagen biosynthetic process	18	4
GO:0046457//prostanoid biosynthetic process	18	4
GO:0048713//regulation of oligodendrocyte differentiation	18	4
GO:0060343//trabecula formation	18	4
GO:0006469//negative regulation of protein kinase activity	142	13
GO:0033554//cellular response to stress	951	55

GO:0000302//response to reactive oxygen species	111	11
GO:0009890//negative regulation of biosynthetic process	932	54
GO:0022612//gland morphogenesis	96	10
GO:0050714//positive regulation of protein secretion	96	10
GO:0001889//liver development	67	8
GO:0002697//regulation of immune effector process	264	20
GO:0010770//positive regulation of cell morphogenesis involved in different	29	5
GO:0042516//regulation of tyrosine phosphorylation of Stat3 protein	29	5
GO:2000243//positive regulation of reproductive process	29	5
GO:0002237//response to molecule of bacterial origin	194	16
GO:0035303//regulation of dephosphorylation	112	11
GO:0007162//negative regulation of cell adhesion	82	9
GO:0032526//response to retinoic acid	97	10
GO:0050707//regulation of cytokine secretion	97	10
GO:0055001//muscle cell development	97	10
GO:0046883//regulation of hormone secretion	161	14
GO:0072507//divalent inorganic cation homeostasis	213	17
GO:0006955//immune response	918	53
GO:0035904//aorta development	19	4
GO:0043618//regulation of transcription from RNA polymerase II promoter i	19	4
GO:0061178//regulation of insulin secretion involved in cellular response to	19	4
GO:0070306//lens fiber cell differentiation	19	4
GO:0001542//ovulation from ovarian follicle	10	3
GO:0007440//foregut morphogenesis	10	3
GO:0010866//regulation of triglyceride biosynthetic process	10	3
GO:0046033//AMP metabolic process	10	3
GO:1900115//extracellular regulation of signal transduction	10	3
GO:1900116//extracellular negative regulation of signal transduction	10	3
GO:0010001//glial cell differentiation	98	10
GO:0031327//negative regulation of cellular biosynthetic process	919	53
GO:0030193//regulation of blood coagulation	55	7
GO:1900046//regulation of hemostasis	55	7
GO:0032835//glomerulus development	42	6
GO:0045428//regulation of nitric oxide biosynthetic process	42	6
GO:0061097//regulation of protein tyrosine kinase activity	42	6
GO:0015758//glucose transport	30	5
GO:0010594//regulation of endothelial cell migration	69	8
GO:0061008//hepaticobiliary system development	69	8
GO:0071356//cellular response to tumor necrosis factor	69	8
GO:0016265//death	735	44
GO:0009617//response to bacterium	323	23
GO:0009894//regulation of catabolic process	535	34
GO:0008202//steroid metabolic process	215	17
GO:0050767//regulation of neurogenesis	324	23
GO:0034097//response to cytokine stimulus	458	30
GO:0010469//regulation of receptor activity	56	7
GO:0090288//negative regulation of cellular response to growth factor stimu	70	8
GO:0050801//ion homeostasis	382	26
GO:0008610//lipid biosynthetic process	459	30
GO:0046456//icosanoid biosynthetic process	43	6
GO:0048477//oogenesis	43	6
GO:1901570//fatty acid derivative biosynthetic process	43	6
GO:0042594//response to starvation	85	9
GO:0006790//sulfur compound metabolic process	217	17
GO:0006801//superoxide metabolic process	31	5
GO:0051148//negative regulation of muscle cell differentiation	31	5
GO:0003179//heart valve morphogenesis	20	4
GO:0007176//regulation of epidermal growth factor-activated receptor activi	20	4

GO:0035020//regulation of Rac protein signal transduction	20	4
GO:0035924//cellular response to vascular endothelial growth factor stimulu	20	4
GO:0007420//brain development	422	28
GO:1901214//regulation of neuron death	149	13
GO:0046903//secretion	403	27
GO:0006508//proteolysis	826	48
GO:0033673//negative regulation of kinase activity	150	13
GO:0032496//response to lipopolysaccharide	184	15
GO:0030166//proteoglycan biosynthetic process	44	6
GO:0033273//response to vitamin	44	6
GO:0042531//positive regulation of tyrosine phosphorylation of STAT protei	44	6
GO:0097006//regulation of plasma lipoprotein particle levels	44	6
GO:2000648//positive regulation of stem cell proliferation	44	6
GO:0002685//regulation of leukocyte migration	102	10
GO:0008016//regulation of heart contraction	102	10
GO:0009950//dorsal/ventral axis specification	11	3
GO:0010510//regulation of acetyl-CoA biosynthetic process from pyruvate	11	3
GO:0043116//negative regulation of vascular permeability	11	3
GO:0045606//positive regulation of epidermal cell differentiation	11	3
GO:0050812//regulation of acyl-CoA biosynthetic process	11	3
GO:0060841//venous blood vessel development	11	3
GO:0022604//regulation of cell morphogenesis	256	19
GO:0001974//blood vessel remodeling	32	5
GO:0001990//regulation of systemic arterial blood pressure by hormone	32	5
GO:0008645//hexose transport	32	5
GO:0030850//prostate gland development	32	5
GO:0045599//negative regulation of fat cell differentiation	32	5
GO:0046677//response to antibiotic	32	5
GO:0051145//smooth muscle cell differentiation	32	5
GO:0007517//muscle organ development	203	16
GO:0010558//negative regulation of macromolecule biosynthetic process	872	50
GO:0010557//positive regulation of macromolecule biosynthetic process	1062	59
GO:0010741//negative regulation of intracellular protein kinase cascade	119	11
GO:0034329//cell junction assembly	119	11
GO:0010769//regulation of cell morphogenesis involved in differentiation	152	13
GO:0055065//metal ion homeostasis	313	22
GO:0003170//heart valve development	21	4
GO:0003338//metanephros morphogenesis	21	4
GO:0008207//C21-steroid hormone metabolic process	21	4
GO:0023014//signal transduction by phosphorylation	21	4
GO:0035412//regulation of catenin import into nucleus	21	4
GO:0040036//regulation of fibroblast growth factor receptor signaling pathw	21	4
GO:0045687//positive regulation of glial cell differentiation	21	4
GO:0060674//placenta blood vessel development	21	4
GO:0090263//positive regulation of canonical Wnt receptor signaling pathwa	45	6
GO:0009746//response to hexose stimulus	73	8
GO:0055088//lipid homeostasis	88	9
GO:0043086//negative regulation of catalytic activity	507	32
GO:0010876//lipid localization	187	15
GO:0045944//positive regulation of transcription from RNA polymerase II pr	628	38
GO:1901566//organonitrogen compound biosynthetic process	508	32
GO:0042306//regulation of protein import into nucleus	120	11
GO:0048871//multicellular organismal homeostasis	120	11
GO:0001837//epithelial to mesenchymal transition	33	5
GO:0007566//embryo implantation	33	5
GO:0032147//activation of protein kinase activity	206	16
GO:0008219//cell death	733	43
GO:0045935//positive regulation of nucleobase-containing compound metal	1048	58

GO:0046364//monosaccharide biosynthetic process	46	6
GO:0048738//cardiac muscle tissue development	105	10
GO:0032787//monocarboxylic acid metabolic process	374	25
GO:0043523//regulation of neuron apoptotic process	138	12
GO:0050796//regulation of insulin secretion	122	11
GO:2001020//regulation of response to DNA damage stimulus	75	8
GO:0010574//regulation of vascular endothelial growth factor production	22	4
GO:0031952//regulation of protein autophosphorylation	22	4
GO:0042481//regulation of odontogenesis	22	4
GO:0043620//regulation of DNA-dependent transcription in response to stre	22	4
GO:0045661//regulation of myoblast differentiation	22	4
GO:0050873//brown fat cell differentiation	22	4
GO:0071326//cellular response to monosaccharide stimulus	22	4
GO:0072088//nephron epithelium morphogenesis	22	4
GO:0072676//lymphocyte migration	22	4
GO:0016331//morphogenesis of embryonic epithelium	106	10
GO:0044272//sulfur compound biosynthetic process	106	10
GO:0000303//response to superoxide	12	3
GO:0019371//cyclooxygenase pathway	12	3
GO:0019433//triglyceride catabolic process	12	3
GO:0030502//negative regulation of bone mineralization	12	3
GO:0031954//positive regulation of protein autophosphorylation	12	3
GO:0034204//lipid translocation	12	3
GO:0043584//nose development	12	3
GO:0045332//phospholipid translocation	12	3
GO:0051193//regulation of cofactor metabolic process	12	3
GO:0051196//regulation of coenzyme metabolic process	12	3
GO:0072075//metanephric mesenchyme development	12	3
GO:2000136//regulation of cell proliferation involved in heart morphogenesi	12	3
GO:0010812//negative regulation of cell-substrate adhesion	34	5
GO:0015749//monosaccharide transport	34	5
GO:0001756//somitogenesis	47	6
GO:0042058//regulation of epidermal growth factor receptor signaling pathv	47	6
GO:0050776//regulation of immune response	656	39
GO:0048520//positive regulation of behavior	91	9
GO:0070374//positive regulation of ERK1 and ERK2 cascade	91	9
GO:0001101//response to acid	76	8
GO:1901137//carbohydrate derivative biosynthetic process	476	30
GO:0051051//negative regulation of transport	265	19
GO:1900180//regulation of protein localization to nucleus	124	11
GO:0008203//cholesterol metabolic process	92	9
GO:0008283//cell proliferation	438	28
GO:0035282//segmentation	62	7
GO:0043085//positive regulation of catalytic activity	868	49
GO:0014013//regulation of gliogenesis	48	6
GO:0030204//chondroitin sulfate metabolic process	48	6
GO:0042509//regulation of tyrosine phosphorylation of STAT protein	48	6
GO:0045682//regulation of epidermis development	48	6
GO:1901184//regulation of ERBB signaling pathway	48	6
GO:0032269//negative regulation of cellular protein metabolic process	361	24
GO:0019229//regulation of vasoconstriction	35	5
GO:0045778//positive regulation of ossification	35	5
GO:0072009//nephron epithelium development	35	5
GO:0031069//hair follicle morphogenesis	23	4
GO:0061098//positive regulation of protein tyrosine kinase activity	23	4
GO:0002683//negative regulation of immune system process	194	15
GO:0071375//cellular response to peptide hormone stimulus	194	15
GO:1901653//cellular response to peptide	194	15

GO:0006875//cellular metal ion homeostasis	267	19
GO:0001894//tissue homeostasis	93	9
GO:0045892//negative regulation of transcription, DNA-dependent	745	43
GO:0001817//regulation of cytokine production	402	26
GO:0022857//transmembrane transporter activity	582	35
GO:0016266//O-glycan processing	49	6
GO:0002092//positive regulation of receptor internalization	13	3
GO:0007567//parturition	13	3
GO:0010611//regulation of cardiac muscle hypertrophy	13	3
GO:0032924//activin receptor signaling pathway	13	3
GO:0046461//neutral lipid catabolic process	13	3
GO:0046464//acylglycerol catabolic process	13	3
GO:0061418//regulation of transcription from RNA polymerase II promoter i	13	3
GO:1901215//negative regulation of neuron death	94	9
GO:0071345//cellular response to cytokine stimulus	384	25
GO:0051348//negative regulation of transferase activity	161	13
GO:0005215//transporter activity	585	35
GO:0018209//peptidyl-serine modification	64	7
GO:0019722//calcium-mediated signaling	64	7
GO:0010743//regulation of macrophage derived foam cell differentiation	24	4
GO:0018149//peptide cross-linking	24	4
GO:0042246//tissue regeneration	24	4
GO:0044060//regulation of endocrine process	24	4
GO:0044253//positive regulation of multicellular organismal metabolic proce	24	4
GO:0050650//chondroitin sulfate proteoglycan biosynthetic process	24	4
GO:0050769//positive regulation of neurogenesis	95	9
GO:0006629//lipid metabolic process	1005	55
GO:0003008//system process	1749	89
GO:0003337//mesenchymal to epithelial transition involved in metanephros	5	2
GO:0010667//negative regulation of cardiac muscle cell apoptotic process	5	2
GO:0014824//artery smooth muscle contraction	5	2
GO:0014909//smooth muscle cell migration	5	2
GO:0016553//base conversion or substitution editing	5	2
GO:0018401//peptidyl-proline hydroxylation to 4-hydroxy-L-proline	5	2
GO:0030859//polarized epithelial cell differentiation	5	2
GO:0030913//paranodal junction assembly	5	2
GO:0032430//positive regulation of phospholipase A2 activity	5	2
GO:0032486//Rap protein signal transduction	5	2
GO:0034144//negative regulation of toll-like receptor 4 signaling pathway	5	2
GO:0034392//negative regulation of smooth muscle cell apoptotic process	5	2
GO:0035581//sequestering of extracellular ligand from receptor	5	2
GO:0035989//tendon development	5	2
GO:0035990//tendon cell differentiation	5	2
GO:0035992//tendon formation	5	2
GO:0043619//regulation of transcription from RNA polymerase II promoter i	5	2
GO:0044252//negative regulation of multicellular organismal metabolic proc	5	2
GO:0045722//positive regulation of gluconeogenesis	5	2
GO:0046880//regulation of follicle-stimulating hormone secretion	5	2
GO:0060394//negative regulation of pathway-restricted SMAD protein phos	5	2
GO:0060501//positive regulation of epithelial cell proliferation involved in lur	5	2
GO:0060526//prostate glandular acinus morphogenesis	5	2
GO:0060527//prostate epithelial cord arborization involved in prostate glanc	5	2
GO:0060665//regulation of branching involved in salivary gland morphogen	5	2
GO:0060856//establishment of blood-brain barrier	5	2
GO:0061045//negative regulation of wound healing	5	2
GO:0061307//cardiac neural crest cell differentiation involved in heart devel	5	2
GO:0071318//cellular response to ATP	5	2
GO:0071637//regulation of monocyte chemotactic protein-1 production	5	2

GO:0072050//S-shaped body morphogenesis	5	2
GO:0097070//ductus arteriosus closure	5	2
GO:0097084//vascular smooth muscle cell development	5	2
GO:2000727//positive regulation of cardiac muscle cell differentiation	5	2
GO:0052547//regulation of peptidase activity	271	19
GO:0045893//positive regulation of transcription, DNA-dependent	899	50
GO:0051969//regulation of transmission of nerve impulse	180	14
GO:0032940//secretion by cell	329	22
GO:0052548//regulation of endopeptidase activity	235	17
GO:0055080//cation homeostasis	349	23
GO:1901615//organic hydroxy compound metabolic process	349	23
GO:0030177//positive regulation of Wnt receptor signaling pathway	65	7
GO:0042307//positive regulation of protein import into nucleus	65	7
GO:0044255//cellular lipid metabolic process	776	44
GO:0044765//single-organism transport	2250	111
GO:0032504//multicellular organism reproduction	590	35
GO:0008150//biological_process	14755	617
GO:0060627//regulation of vesicle-mediated transport	200	15
GO:0006811//ion transport	947	52
GO:0010629//negative regulation of gene expression	820	46
GO:0016125//sterol metabolic process	97	9
GO:0002456//T cell mediated immunity	25	4
GO:0007157//heterophilic cell-cell adhesion	25	4
GO:0043114//regulation of vascular permeability	25	4
GO:0045123//cellular extravasation	25	4
GO:0045684//positive regulation of epidermis development	25	4
GO:0002576//platelet degranulation	66	7
GO:0001935//endothelial cell proliferation	14	3
GO:0007520//myoblast fusion	14	3
GO:0008347//glial cell migration	14	3
GO:0010623//developmental programmed cell death	14	3
GO:0010955//negative regulation of protein processing	14	3
GO:0014743//regulation of muscle hypertrophy	14	3
GO:0014911//positive regulation of smooth muscle cell migration	14	3
GO:0016137//glycoside metabolic process	14	3
GO:0032148//activation of protein kinase B activity	14	3
GO:0032288//myelin assembly	14	3
GO:0033280//response to vitamin D	14	3
GO:0042448//progesterone metabolic process	14	3
GO:0042554//superoxide anion generation	14	3
GO:0045932//negative regulation of muscle contraction	14	3
GO:0046697//decidualization	14	3
GO:0048857//neural nucleus development	14	3
GO:0060037//pharyngeal system development	14	3
GO:0060571//morphogenesis of an epithelial fold	14	3
GO:0060716//labyrinthine layer blood vessel development	14	3
GO:0072074//kidney mesenchyme development	14	3
GO:0010676//positive regulation of cellular carbohydrate metabolic process	38	5
GO:0033628//regulation of cell adhesion mediated by integrin	38	5
GO:0050710//negative regulation of cytokine secretion	38	5
GO:0060993//kidney morphogenesis	38	5
GO:0050921//positive regulation of chemotaxis	82	8
GO:0008277//regulation of G-protein coupled receptor protein signaling pat	98	9
GO:0046427//positive regulation of JAK-STAT cascade	52	6
GO:0051090//regulation of sequence-specific DNA binding transcription fac	296	20
GO:0002688//regulation of leukocyte chemotaxis	67	7
GO:0006493//protein O-linked glycosylation	67	7
GO:0009267//cellular response to starvation	67	7

GO:0009953//dorsal/ventral pattern formation	67	7
GO:0009636//response to toxic substance	115	10
GO:0051048//negative regulation of secretion	132	11
GO:0090276//regulation of peptide hormone secretion	132	11
GO:0045664//regulation of neuron differentiation	259	18
GO:0043627//response to estrogen stimulus	99	9
GO:0046890//regulation of lipid biosynthetic process	99	9
GO:0001816//cytokine production	83	8
GO:0009791//post-embryonic development	83	8
GO:0002886//regulation of myeloid leukocyte mediated immunity	26	4
GO:0030279//negative regulation of ossification	26	4
GO:0030514//negative regulation of BMP signaling pathway	26	4
GO:0034381//plasma lipoprotein particle clearance	26	4
GO:0042312//regulation of vasodilation	26	4
GO:0090102//cochlea development	26	4
GO:0061053//somite development	53	6
GO:0072073//kidney epithelium development	53	6
GO:0046888//negative regulation of hormone secretion	39	5
GO:0031099//regeneration	68	7
GO:0072330//monocarboxylic acid biosynthetic process	151	12
GO:0006664//glycolipid metabolic process	100	9
GO:0048609//multicellular organismal reproductive process	579	34
GO:0055085//transmembrane transport	788	44
GO:0002791//regulation of peptide secretion	134	11
GO:0090087//regulation of peptide transport	134	11
GO:0000768//syncytium formation by plasma membrane fusion	15	3
GO:0001738//morphogenesis of a polarized epithelium	15	3
GO:0006688//glycosphingolipid biosynthetic process	15	3
GO:0010884//positive regulation of lipid storage	15	3
GO:0010922//positive regulation of phosphatase activity	15	3
GO:0019933//cAMP-mediated signaling	15	3
GO:0031670//cellular response to nutrient	15	3
GO:0033137//negative regulation of peptidyl-serine phosphorylation	15	3
GO:0050746//regulation of lipoprotein metabolic process	15	3
GO:0060291//long-term synaptic potentiation	15	3
GO:0060479//lung cell differentiation	15	3
GO:0070741//response to interleukin-6	15	3
GO:2000826//regulation of heart morphogenesis	15	3
GO:0002252//immune effector process	378	24
GO:0051346//negative regulation of hydrolase activity	262	18
GO:0022891//substrate-specific transmembrane transporter activity	581	34
GO:0051253//negative regulation of RNA metabolic process	769	43
GO:0007422//peripheral nervous system development	69	7
GO:0009266//response to temperature stimulus	101	9
GO:0044283//small molecule biosynthetic process	379	24
GO:0007603//phototransduction, visible light	54	6
GO:0060070//canonical Wnt receptor signaling pathway	54	6
GO:0001508//regulation of action potential	118	10
GO:0007160//cell-matrix adhesion	85	8
GO:0002138//retinoic acid biosynthetic process	6	2
GO:0007223//Wnt receptor signaling pathway, calcium modulating pathway	6	2
GO:0010761//fibroblast migration	6	2
GO:0010871//negative regulation of receptor biosynthetic process	6	2
GO:0010886//positive regulation of cholesterol storage	6	2
GO:0014820//tonic smooth muscle contraction	6	2
GO:0016264//gap junction assembly	6	2
GO:0019800//peptide cross-linking via chondroitin 4-sulfate glycosaminogly	6	2
GO:0030299//intestinal cholesterol absorption	6	2

GO:0032682//negative regulation of chemokine production	6	2
GO:0033004//negative regulation of mast cell activation	6	2
GO:0033690//positive regulation of osteoblast proliferation	6	2
GO:0043301//negative regulation of leukocyte degranulation	6	2
GO:0060059//embryonic retina morphogenesis in camera-type eye	6	2
GO:0060373//regulation of ventricular cardiac muscle cell membrane depol	6	2
GO:0060666//dichotomous subdivision of terminal units involved in salivary	6	2
GO:0070102//interleukin-6-mediated signaling pathway	6	2
GO:0070141//response to UV-A	6	2
GO:0072109//glomerular mesangium development	6	2
GO:0072141//renal interstitial cell development	6	2
GO:0072239//metanephric glomerulus vasculature development	6	2
GO:0086014//regulation of atrial cardiac muscle cell action potential	6	2
GO:0086026//atrial cardiac muscle cell to AV node cell signaling	6	2
GO:0086066//atrial cardiac muscle cell to AV node cell communication	6	2
GO:1900407//regulation of cellular response to oxidative stress	6	2
GO:1901031//regulation of response to reactive oxygen species	6	2
GO:2000794//regulation of epithelial cell proliferation involved in lung morpł	6	2
GO:0045165//cell fate commitment	189	14
GO:0010907//positive regulation of glucose metabolic process	27	4
GO:0042059//negative regulation of epidermal growth factor receptor signal	27	4
GO:0045933//positive regulation of muscle contraction	27	4
GO:0048730//epidermis morphogenesis	27	4
GO:0061383//trabecula morphogenesis	27	4
GO:1901185//negative regulation of ERBB signaling pathway	27	4
GO:0022892//substrate-specific transporter activity	583	34
GO:0071216//cellular response to biotic stimulus	102	9
GO:2000113//negative regulation of cellular macromolecule biosynthetic prc	837	46
GO:0010466//negative regulation of peptidase activity	154	12
GO:0016787//hydrolase activity	154	12
GO:0042471//ear morphogenesis	86	8
GO:0021675//nerve development	55	6
GO:0031644//regulation of neurological system process	191	14
GO:0051493//regulation of cytoskeleton organization	247	17
GO:0019319//hexose biosynthetic process	41	5
GO:0044092//negative regulation of molecular function	650	37
GO:0009749//response to glucose stimulus	71	7
GO:0042476//odontogenesis	71	7
GO:0034754//cellular hormone metabolic process	87	8
GO:0001991//regulation of systemic arterial blood pressure by circulatory re	16	3
GO:0010830//regulation of myotube differentiation	16	3
GO:0010869//regulation of receptor biosynthetic process	16	3
GO:0042474//middle ear morphogenesis	16	3
GO:0045686//negative regulation of glial cell differentiation	16	3
GO:0048247//lymphocyte chemotaxis	16	3
GO:0050892//intestinal absorption	16	3
GO:0060693//regulation of branching involved in salivary gland morphogen	16	3
GO:0072078//nephron tubule morphogenesis	16	3
GO:0097035//regulation of membrane lipid distribution	16	3
GO:0043551//regulation of phosphatidylinositol 3-kinase activity	28	4
GO:0048662//negative regulation of smooth muscle cell proliferation	28	4
GO:0050766//positive regulation of phagocytosis	28	4
GO:0061005//cell differentiation involved in kidney development	28	4
GO:0007043//cell-cell junction assembly	56	6
GO:0007613//memory	56	6
GO:0016525//negative regulation of angiogenesis	56	6
GO:0018958//phenol-containing compound metabolic process	56	6
GO:0019217//regulation of fatty acid metabolic process	56	6

GO:0050880//regulation of blood vessel size	56	6
GO:0034599//cellular response to oxidative stress	104	9
GO:0033043//regulation of organelle organization	487	29
GO:0046822//regulation of nucleocytoplasmic transport	139	11
GO:0043409//negative regulation of MAPK cascade	88	8
GO:0043524//negative regulation of neuron apoptotic process	88	8
GO:0001676//long-chain fatty acid metabolic process	72	7
GO:0051254//positive regulation of RNA metabolic process	931	50
GO:0030003//cellular cation homeostasis	289	19
GO:0010634//positive regulation of epithelial cell migration	57	6
GO:0035150//regulation of tube size	57	6
GO:0006805//xenobiotic metabolic process	140	11
GO:0044711//single-organism biosynthetic process	388	24
GO:0055002//striated muscle cell development	89	8
GO:0010951//negative regulation of endopeptidase activity	123	10
GO:0001657//ureteric bud development	73	7
GO:0046824//positive regulation of nucleocytoplasmic transport	73	7
GO:0003156//regulation of organ formation	29	4
GO:0003333//amino acid transmembrane transport	29	4
GO:0006584//catecholamine metabolic process	29	4
GO:0009712//catechol-containing compound metabolic process	29	4
GO:0009954//proximal/distal pattern formation	29	4
GO:0014015//positive regulation of gliogenesis	29	4
GO:0045168//cell-cell signaling involved in cell fate commitment	29	4
GO:0051004//regulation of lipoprotein lipase activity	29	4
GO:0061326//renal tubule development	29	4
GO:0071622//regulation of granulocyte chemotaxis	29	4
GO:0072080//nephron tubule development	29	4
GO:1901888//regulation of cell junction assembly	29	4
GO:0008286//insulin receptor signaling pathway	106	9
GO:0048015//phosphatidylinositol-mediated signaling	106	9
GO:0048017//inositol lipid-mediated signaling	106	9
GO:0051726//regulation of cell cycle	657	37
GO:0071466//cellular response to xenobiotic stimulus	141	11
GO:0032872//regulation of stress-activated MAPK cascade	159	12
GO:0001906//cell killing	43	5
GO:0006949//syncytium formation	17	3
GO:0042573//retinoic acid metabolic process	17	3
GO:0045909//positive regulation of vasodilation	17	3
GO:0046503//glycerolipid catabolic process	17	3
GO:0060441//epithelial tube branching involved in lung morphogenesis	17	3
GO:0072215//regulation of metanephros development	17	3
GO:0090103//cochlea morphogenesis	17	3
GO:2000209//regulation of anoikis	17	3
GO:0046470//phosphatidylcholine metabolic process	58	6
GO:0003256//regulation of transcription from RNA polymerase II promoter i	7	2
GO:0006030//chitin metabolic process	7	2
GO:0006032//chitin catabolic process	7	2
GO:0010042//response to manganese ion	7	2
GO:0010269//response to selenium ion	7	2
GO:0010613//positive regulation of cardiac muscle hypertrophy	7	2
GO:0010664//negative regulation of striated muscle cell apoptotic process	7	2
GO:0010832//negative regulation of myotube differentiation	7	2
GO:0014742//positive regulation of muscle hypertrophy	7	2
GO:0016102//diterpenoid biosynthetic process	7	2
GO:0032354//response to follicle-stimulating hormone stimulus	7	2
GO:0032909//regulation of transforming growth factor beta2 production	7	2
GO:0035089//establishment of apical/basal cell polarity	7	2

GO:0044320//cellular response to leptin stimulus	7	2
GO:0045603//positive regulation of endothelial cell differentiation	7	2
GO:0051890//regulation of cardioblast differentiation	7	2
GO:0060307//regulation of ventricular cardiac muscle cell membrane repola	7	2
GO:0060371//regulation of atrial cardiac muscle cell membrane depolarizati	7	2
GO:0060572//morphogenesis of an epithelial bud	7	2
GO:0070208//protein heterotrimerization	7	2
GO:0072071//renal interstitial cell differentiation	7	2
GO:0072077//renal vesicle morphogenesis	7	2
GO:0072283//metanephric renal vesicle morphogenesis	7	2
GO:2001015//negative regulation of skeletal muscle cell differentiation	7	2
GO:NM_0142	7	2
GO:0051172//negative regulation of nitrogen compound metabolic process	873	47
GO:0019752//carboxylic acid metabolic process	766	42
GO:0070302//regulation of stress-activated protein kinase signaling cascad	160	12
GO:0007229//integrin-mediated signaling pathway	74	7
GO:0031400//negative regulation of protein modification process	273	18
GO:0051345//positive regulation of hydrolase activity	473	28
GO:0048754//branching morphogenesis of an epithelial tube	125	10
GO:0009410//response to xenobiotic stimulus	143	11
GO:0019835//cytolysis	30	4
GO:0031076//embryonic camera-type eye development	30	4
GO:0060425//lung morphogenesis	30	4
GO:0042490//mechanoreceptor differentiation	44	5
GO:0048663//neuron fate commitment	44	5
GO:2000241//regulation of reproductive process	75	7
GO:0010038//response to metal ion	180	13
GO:0009100//glycoprotein metabolic process	315	20
GO:0007088//regulation of mitosis	92	8
GO:0051783//regulation of nuclear division	92	8
GO:0071219//cellular response to molecule of bacterial origin	92	8
GO:0007267//cell-cell signaling	729	40
GO:0032970//regulation of actin filament-based process	181	13
GO:0035637//multicellular organismal signaling	540	31
GO:0007010//cytoskeleton organization	561	32
GO:0002690//positive regulation of leukocyte chemotaxis	60	6
GO:0007623//circadian rhythm	60	6
GO:0019935//cyclic-nucleotide-mediated signaling	18	3
GO:0034405//response to fluid shear stress	18	3
GO:0046058//cAMP metabolic process	18	3
GO:0050829//defense response to Gram-negative bacterium	18	3
GO:0050918//positive chemotaxis	18	3
GO:0071634//regulation of transforming growth factor beta production	18	3
GO:0022407//regulation of cell-cell adhesion	76	7
GO:0042542//response to hydrogen peroxide	76	7
GO:0071901//negative regulation of protein serine/threonine kinase activity	76	7
GO:0009880//embryonic pattern specification	45	5
GO:0018108//peptidyl-tyrosine phosphorylation	45	5
GO:0045913//positive regulation of carbohydrate metabolic process	45	5
GO:0071300//cellular response to retinoic acid	45	5
GO:0009615//response to virus	201	14
GO:0045934//negative regulation of nucleobase-containing compound meta	861	46
GO:0042752//regulation of circadian rhythm	31	4
GO:2000401//regulation of lymphocyte migration	31	4
GO:0007346//regulation of mitotic cell cycle	259	17
GO:0002698//negative regulation of immune effector process	61	6
GO:0072091//regulation of stem cell proliferation	61	6
GO:0006954//inflammatory response	299	19

GO:0090316//positive regulation of intracellular protein transport	94	8
GO:0006636//unsaturated fatty acid biosynthetic process	46	5
GO:0007569//cell aging	46	5
GO:0046460//neutral lipid biosynthetic process	46	5
GO:0046463//acylglycerol biosynthetic process	46	5
GO:0016053//organic acid biosynthetic process	261	17
GO:0046394//carboxylic acid biosynthetic process	261	17
GO:0015075//ion transmembrane transporter activity	567	32
GO:0001764//neuron migration	78	7
GO:0001553//luteinization	8	2
GO:0002063//chondrocyte development	8	2
GO:0003161//cardiac conduction system development	8	2
GO:0007175//negative regulation of epidermal growth factor-activated rece	8	2
GO:0010755//regulation of plasminogen activation	8	2
GO:0010996//response to auditory stimulus	8	2
GO:0019471//4-hydroxyproline metabolic process	8	2
GO:0031659//positive regulation of cyclin-dependent protein serine/threonir	8	2
GO:0032276//regulation of gonadotropin secretion	8	2
GO:0032331//negative regulation of chondrocyte differentiation	8	2
GO:0032429//regulation of phospholipase A2 activity	8	2
GO:0033689//negative regulation of osteoblast proliferation	8	2
GO:0034505//tooth mineralization	8	2
GO:0038092//nodal signaling pathway	8	2
GO:0040034//regulation of development, heterochronic	8	2
GO:0043129//surfactant homeostasis	8	2
GO:0044321//response to leptin stimulus	8	2
GO:0045117//azole transport	8	2
GO:0045628//regulation of T-helper 2 cell differentiation	8	2
GO:0045663//positive regulation of myoblast differentiation	8	2
GO:0045844//positive regulation of striated muscle tissue development	8	2
GO:0048505//regulation of timing of cell differentiation	8	2
GO:0048636//positive regulation of muscle organ development	8	2
GO:0048875//chemical homeostasis within a tissue	8	2
GO:0060638//mesenchymal-epithelial cell signaling	8	2
GO:0061162//establishment of monopolar cell polarity	8	2
GO:0061339//establishment or maintenance of monopolar cell polarity	8	2
GO:0071276//cellular response to cadmium ion	8	2
GO:0071379//cellular response to prostaglandin stimulus	8	2
GO:0071850//mitotic cell cycle arrest	8	2
GO:0072087//renal vesicle development	8	2
GO:0072111//cell proliferation involved in kidney development	8	2
GO:0072203//cell proliferation involved in metanephros development	8	2
GO:1900015//regulation of cytokine production involved in inflammatory res	8	2
GO:1901072//glucosamine-containing compound catabolic process	8	2
GO:1901863//positive regulation of muscle tissue development	8	2
GO:0006040//amino sugar metabolic process	32	4
GO:0006730//one-carbon metabolic process	32	4
GO:0043550//regulation of lipid kinase activity	32	4
GO:0021782//glial cell development	62	6
GO:0015012//heparan sulfate proteoglycan biosynthetic process	19	3
GO:0043304//regulation of mast cell degranulation	19	3
GO:0043502//regulation of muscle adaptation	19	3
GO:0045823//positive regulation of heart contraction	19	3
GO:0045920//negative regulation of exocytosis	19	3
GO:0050716//positive regulation of interleukin-1 secretion	19	3
GO:0060740//prostate gland epithelium morphogenesis	19	3
GO:0061333//renal tubule morphogenesis	19	3
GO:0090207//regulation of triglyceride metabolic process	19	3

GO:2000404//regulation of T cell migration	19	3
GO:2001014//regulation of skeletal muscle cell differentiation	19	3
GO:0006873//cellular ion homeostasis	302	19
GO:0044708//single-organism behavior	302	19
GO:0071407//cellular response to organic cyclic compound	186	13
GO:0001708//cell fate specification	47	5
GO:0002027//regulation of heart rate	47	5
GO:0015748//organophosphate ester transport	47	5
GO:0018212//peptidyl-tyrosine modification	47	5
GO:0021954//central nervous system neuron development	47	5
GO:0035148//tube formation	96	8
GO:0015399//primary active transmembrane transporter activity	63	6
GO:0015405//P-P-bond-hydrolysis-driven transmembrane transporter activi	63	6
GO:0016462//pyrophosphatase activity	63	6
GO:0016817//hydrolase activity, acting on acid anhydrides	63	6
GO:0016818//hydrolase activity, acting on acid anhydrides, in phosphorus-c	63	6
GO:0016820//hydrolase activity, acting on acid anhydrides, catalyzing trans	63	6
GO:0016887//ATPase activity	63	6
GO:0017111//nucleoside-triphosphatase activity	63	6
GO:0031016//pancreas development	63	6
GO:0035050//embryonic heart tube development	63	6
GO:0042623//ATPase activity, coupled	63	6
GO:0042625//ATPase activity, coupled to transmembrane movement of ion:	63	6
GO:0042626//ATPase activity, coupled to transmembrane movement of sub	63	6
GO:0043492//ATPase activity, coupled to movement of substances	63	6
GO:0012501//programmed cell death	614	34
GO:0010470//regulation of gastrulation	33	4
GO:0015914//phospholipid transport	33	4
GO:0030595//leukocyte chemotaxis	80	7
GO:0097202//activation of cysteine-type endopeptidase activity	80	7
GO:0000187//activation of MAPK activity	115	9
GO:0006665//sphingolipid metabolic process	115	9
GO:0031589//cell-substrate adhesion	115	9
GO:0008643//carbohydrate transport	64	6
GO:0042552//myelination	64	6
GO:0050715//positive regulation of cytokine secretion	64	6
GO:0008045//motor neuron axon guidance	20	3
GO:0015179//L-amino acid transmembrane transporter activity	20	3
GO:0015301//anion:anion antiporter activity	20	3
GO:0017145//stem cell division	20	3
GO:0032925//regulation of activin receptor signaling pathway	20	3
GO:0033006//regulation of mast cell activation involved in immune respons	20	3
GO:0036151//phosphatidylcholine acyl-chain remodeling	20	3
GO:0045987//positive regulation of smooth muscle contraction	20	3
GO:0002520//immune system development	429	25
GO:0015980//energy derivation by oxidation of organic compounds	228	15
GO:0019228//regulation of action potential in neuron	81	7
GO:0031329//regulation of cellular catabolic process	471	27
GO:0001569//patterning of blood vessels	34	4
GO:0018210//peptidyl-threonine modification	34	4
GO:0045668//negative regulation of osteoblast differentiation	34	4
GO:0071230//cellular response to amino acid stimulus	34	4
GO:0003208//cardiac ventricle morphogenesis	49	5
GO:0006942//regulation of striated muscle contraction	49	5
GO:0050773//regulation of dendrite development	49	5
GO:0034765//regulation of ion transmembrane transport	153	11
GO:0016049//cell growth	65	6
GO:0060560//developmental growth involved in morphogenesis	65	6

GO:0007156//homophilic cell adhesion	99	8
GO:0043122//regulation of I-kappaB kinase/NF-kappaB cascade	191	13
GO:0003177//pulmonary valve development	9	2
GO:0003184//pulmonary valve morphogenesis	9	2
GO:0009404//toxin metabolic process	9	2
GO:0010544//negative regulation of platelet activation	9	2
GO:0016114//terpenoid biosynthetic process	9	2
GO:0019430//removal of superoxide radicals	9	2
GO:0031657//regulation of cyclin-dependent protein serine/threonine kinase	9	2
GO:0032495//response to muramyl dipeptide	9	2
GO:0032926//negative regulation of activin receptor signaling pathway	9	2
GO:0033629//negative regulation of cell adhesion mediated by integrin	9	2
GO:0035112//genitalia morphogenesis	9	2
GO:0035584//calcium-mediated signaling using intracellular calcium source	9	2
GO:0043931//ossification involved in bone maturation	9	2
GO:0045741//positive regulation of epidermal growth factor-activated recep	9	2
GO:0048368//lateral mesoderm development	9	2
GO:0055003//cardiac myofibril assembly	9	2
GO:0055089//fatty acid homeostasis	9	2
GO:0060192//negative regulation of lipase activity	9	2
GO:0060231//mesenchymal to epithelial transition	9	2
GO:0060379//cardiac muscle cell myoblast differentiation	9	2
GO:0060736//prostate gland growth	9	2
GO:0071451//cellular response to superoxide	9	2
GO:0072079//nephron tubule formation	9	2
GO:0086009//membrane repolarization	9	2
GO:0086011//membrane repolarization involved in regulation of action pote	9	2
GO:0086013//membrane repolarization involved in regulation of cardiac mu	9	2
GO:0090178//regulation of establishment of planar polarity involved in neur	9	2
GO:0090179//planar cell polarity pathway involved in neural tube closure	9	2
GO:0097150//neuronal stem cell maintenance	9	2
GO:1900006//positive regulation of dendrite development	9	2
GO:1901213//regulation of transcription from RNA polymerase II promoter i	9	2
GO:2000095//regulation of Wnt receptor signaling pathway, planar cell pola	9	2
GO:0022804//active transmembrane transporter activity	154	11
GO:0001936//regulation of endothelial cell proliferation	66	6
GO:0007272//ensheathment of neurons	66	6
GO:0008366//axon ensheathment	66	6
GO:0009408//response to heat	66	6
GO:0043507//positive regulation of JUN kinase activity	66	6
GO:0050673//epithelial cell proliferation	66	6
GO:0050866//negative regulation of cell activation	118	9
GO:0006699//bile acid biosynthetic process	21	3
GO:0014014//negative regulation of gliogenesis	21	3
GO:0030206//chondroitin sulfate biosynthetic process	21	3
GO:0030947//regulation of vascular endothelial growth factor receptor signa	21	3
GO:0042311//vasodilation	21	3
GO:0043388//positive regulation of DNA binding	21	3
GO:0045616//regulation of keratinocyte differentiation	21	3
GO:0046686//response to cadmium ion	21	3
GO:0060512//prostate gland morphogenesis	21	3
GO:0071331//cellular response to hexose stimulus	21	3
GO:0071333//cellular response to glucose stimulus	21	3
GO:0086001//regulation of cardiac muscle cell action potential	21	3
GO:0090022//regulation of neutrophil chemotaxis	21	3
GO:0090189//regulation of branching involved in ureteric bud morphogenes	21	3
GO:1901071//glucosamine-containing compound metabolic process	21	3
GO:0010822//positive regulation of mitochondrion organization	35	4

GO:0015807//L-amino acid transport	35	4
GO:0022409//positive regulation of cell-cell adhesion	35	4
GO:0048146//positive regulation of fibroblast proliferation	35	4
GO:0009584//detection of visible light	67	6
GO:0040014//regulation of multicellular organism growth	67	6
GO:0042391//regulation of membrane potential	214	14
GO:0048747//muscle fiber development	51	5
GO:0032956//regulation of actin cytoskeleton organization	157	11
GO:0019226//transmission of nerve impulse	523	29
GO:0008637//apoptotic mitochondrial changes	36	4
GO:0021545//cranial nerve development	36	4
GO:0071695//anatomical structure maturation	36	4
GO:0048872//homeostasis of number of cells	139	10
GO:0001838//embryonic epithelial tube formation	85	7
GO:0046425//regulation of JAK-STAT cascade	68	6
GO:0072593//reactive oxygen species metabolic process	68	6
GO:0015849//organic acid transport	177	12
GO:0046942//carboxylic acid transport	177	12
GO:0033157//regulation of intracellular protein transport	158	11
GO:0003081//regulation of systemic arterial blood pressure by renin-angiote	22	3
GO:0015171//amino acid transmembrane transporter activity	22	3
GO:0030212//hyaluronan metabolic process	22	3
GO:0030511//positive regulation of transforming growth factor beta receptor	22	3
GO:0034122//negative regulation of toll-like receptor signaling pathway	22	3
GO:0042036//negative regulation of cytokine biosynthetic process	22	3
GO:0042491//auditory receptor cell differentiation	22	3
GO:0043552//positive regulation of phosphatidylinositol 3-kinase activity	22	3
GO:0048645//organ formation	22	3
GO:0050704//regulation of interleukin-1 secretion	22	3
GO:0050974//detection of mechanical stimulus involved in sensory percepti	22	3
GO:0051893//regulation of focal adhesion assembly	22	3
GO:0090109//regulation of cell-substrate junction assembly	22	3
GO:0048592//eye morphogenesis	103	8
GO:0001952//regulation of cell-matrix adhesion	52	5
GO:0007202//activation of phospholipase C activity	52	5
GO:0042475//odontogenesis of dentin-containing tooth	52	5
GO:0050709//negative regulation of protein secretion	52	5
GO:0006198//cAMP catabolic process	10	2
GO:0006809//nitric oxide biosynthetic process	10	2
GO:0007096//regulation of exit from mitosis	10	2
GO:0010885//regulation of cholesterol storage	10	2
GO:0019372//lipoxygenase pathway	10	2
GO:0030219//megakaryocyte differentiation	10	2
GO:0033160//positive regulation of protein import into nucleus, translocation	10	2
GO:0034143//regulation of toll-like receptor 4 signaling pathway	10	2
GO:0035886//vascular smooth muscle cell differentiation	10	2
GO:0042574//retinal metabolic process	10	2
GO:0044241//lipid digestion	10	2
GO:0045618//positive regulation of keratinocyte differentiation	10	2
GO:0045821//positive regulation of glycolysis	10	2
GO:0046851//negative regulation of bone remodeling	10	2
GO:0060438//trachea development	10	2
GO:0060442//branching involved in prostate gland morphogenesis	10	2
GO:0060525//prostate glandular acinus development	10	2
GO:0060768//regulation of epithelial cell proliferation involved in prostate gl	10	2
GO:0070498//interleukin-1-mediated signaling pathway	10	2
GO:0070977//bone maturation	10	2
GO:0072182//regulation of nephron tubule epithelial cell differentiation	10	2

GO:0072189//ureter development	10	2
GO:0072224//metanephric glomerulus development	10	2
GO:0086010//membrane depolarization involved in regulation of action pote	10	2
GO:0086012//membrane depolarization involved in regulation of cardiac mu	10	2
GO:0090136//epithelial cell-cell adhesion	10	2
GO:0034762//regulation of transmembrane transport	159	11
GO:0071222//cellular response to lipopolysaccharide	86	7
GO:0072175//epithelial tube formation	86	7
GO:0042102//positive regulation of T cell proliferation	69	6
GO:0009889//regulation of biosynthetic process	3216	147
GO:0051224//negative regulation of protein transport	104	8
GO:0006094//gluconeogenesis	37	4
GO:0031623//receptor internalization	37	4
GO:0045069//regulation of viral genome replication	53	5
GO:0051101//regulation of DNA binding	53	5
GO:0044702//single organism reproductive process	658	35
GO:0006066//alcohol metabolic process	279	17
GO:0016485//protein processing	105	8
GO:0050804//regulation of synaptic transmission	161	11
GO:0060249//anatomical structure homeostasis	161	11
GO:0002828//regulation of type 2 immune response	23	3
GO:0008154//actin polymerization or depolymerization	23	3
GO:0032570//response to progesterone stimulus	23	3
GO:0042310//vasoconstriction	23	3
GO:0046513//ceramide biosynthetic process	23	3
GO:0051057//positive regulation of small GTPase mediated signal transduc	23	3
GO:0051966//regulation of synaptic transmission, glutamatergic	23	3
GO:0060428//lung epithelium development	23	3
GO:0086036//regulation of cardiac muscle cell membrane potential	23	3
GO:0090218//positive regulation of lipid kinase activity	23	3
GO:0042472//inner ear morphogenesis	70	6
GO:0072527//pyrimidine-containing compound metabolic process	70	6
GO:0002684//positive regulation of immune system process	638	34
GO:0043269//regulation of ion transport	321	19
GO:0051607//defense response to virus	124	9
GO:0032103//positive regulation of response to external stimulus	162	11
GO:0046328//regulation of JNK cascade	143	10
GO:0043506//regulation of JUN kinase activity	88	7
GO:0006865//amino acid transport	106	8
GO:0031532//actin cytoskeleton reorganization	38	4
GO:0050764//regulation of phagocytosis	38	4
GO:0071229//cellular response to acid	38	4
GO:0035556//intracellular signal transduction	1058	53
GO:0048534//hematopoietic or lymphoid organ development	405	23
GO:0002526//acute inflammatory response	54	5
GO:0007200//phospholipase C-activating G-protein coupled receptor signal	54	5
GO:0007610//behavior	364	21
GO:0009581//detection of external stimulus	144	10
GO:0002544//chronic inflammatory response	11	2
GO:0005355//glucose transmembrane transporter activity	11	2
GO:0006012//galactose metabolic process	11	2
GO:0010002//cardioblast differentiation	11	2
GO:0010745//negative regulation of macrophage derived foam cell different	11	2
GO:0021783//preganglionic parasympathetic nervous system development	11	2
GO:0030878//thyroid gland development	11	2
GO:0032793//positive regulation of CREB transcription factor activity	11	2
GO:0032878//regulation of establishment or maintenance of cell polarity	11	2
GO:0033363//secretory granule organization	11	2

GO:0034110//regulation of homotypic cell-cell adhesion	11	2
GO:0034442//regulation of lipoprotein oxidation	11	2
GO:0035810//positive regulation of urine volume	11	2
GO:0042487//regulation of odontogenesis of dentin-containing tooth	11	2
GO:0043403//skeletal muscle tissue regeneration	11	2
GO:0045187//regulation of circadian sleep/wake cycle, sleep	11	2
GO:0045197//establishment or maintenance of epithelial cell apical/basal p	11	2
GO:0045624//positive regulation of T-helper cell differentiation	11	2
GO:0046348//amino sugar catabolic process	11	2
GO:0048009//insulin-like growth factor receptor signaling pathway	11	2
GO:0060306//regulation of membrane repolarization	11	2
GO:0060351//cartilage development involved in endochondral bone morpho	11	2
GO:0060644//mammary gland epithelial cell differentiation	11	2
GO:0060742//epithelial cell differentiation involved in prostate gland develo	11	2
GO:0060976//coronary vasculature development	11	2
GO:0086019//cell-cell signaling involved in cardiac conduction	11	2
GO:0090162//establishment of epithelial cell polarity	11	2
GO:2000050//regulation of non-canonical Wnt receptor signaling pathway	11	2
GO:2000380//regulation of mesoderm development	11	2
GO:0008324//cation transmembrane transporter activity	471	26
GO:0006810//transport	2836	130
GO:0051234//establishment of localization	2883	132
GO:0001893//maternal placenta development	24	3
GO:0030201//heparan sulfate proteoglycan metabolic process	24	3
GO:0043043//peptide biosynthetic process	24	3
GO:0043300//regulation of leukocyte degranulation	24	3
GO:1901661//quinone metabolic process	24	3
GO:0008509//anion transmembrane transporter activity	145	10
GO:0030510//regulation of BMP signaling pathway	55	5
GO:0032355//response to estradiol stimulus	55	5
GO:0031032//actomyosin structure organization	39	4
GO:0034637//cellular carbohydrate biosynthetic process	39	4
GO:0042177//negative regulation of protein catabolic process	39	4
GO:0060113//inner ear receptor cell differentiation	39	4
GO:0060135//maternal process involved in female pregnancy	39	4
GO:0051047//positive regulation of secretion	205	13
GO:0005976//polysaccharide metabolic process	56	5
GO:0010821//regulation of mitochondrion organization	56	5
GO:0006766//vitamin metabolic process	91	7
GO:0034976//response to endoplasmic reticulum stress	91	7
GO:0051054//positive regulation of DNA metabolic process	91	7
GO:0046777//protein autophosphorylation	147	10
GO:0009582//detection of abiotic stimulus	128	9
GO:0006897//endocytosis	307	18
GO:0006939//smooth muscle contraction	40	4
GO:0032652//regulation of interleukin-1 production	40	4
GO:0046879//hormone secretion	40	4
GO:0001702//gastrulation with mouth forming second	25	3
GO:0009409//response to cold	25	3
GO:0015893//drug transport	25	3
GO:0018146//keratan sulfate biosynthetic process	25	3
GO:0034367//macromolecular complex remodeling	25	3
GO:0034368//protein-lipid complex remodeling	25	3
GO:0034369//plasma lipoprotein particle remodeling	25	3
GO:0007602//phototransduction	74	6
GO:0010638//positive regulation of organelle organization	207	13
GO:0009058//biosynthetic process	2149	100
GO:0042632//cholesterol homeostasis	57	5

GO:0010975//regulation of neuron projection development	168	11
GO:0001556//oocyte maturation	12	2
GO:0002003//angiotensin maturation	12	2
GO:0002042//cell migration involved in sprouting angiogenesis	12	2
GO:0006700//C21-steroid hormone biosynthetic process	12	2
GO:0009214//cyclic nucleotide catabolic process	12	2
GO:0015145//monosaccharide transmembrane transporter activity	12	2
GO:0015149//hexose transmembrane transporter activity	12	2
GO:0030949//positive regulation of vascular endothelial growth factor recep	12	2
GO:0032309//icosanoid secretion	12	2
GO:0034104//negative regulation of tissue remodeling	12	2
GO:0034391//regulation of smooth muscle cell apoptotic process	12	2
GO:0034394//protein localization to cell surface	12	2
GO:0036296//response to increased oxygen levels	12	2
GO:0042749//regulation of circadian sleep/wake cycle	12	2
GO:0045576//mast cell activation	12	2
GO:0046718//viral entry into host cell	12	2
GO:0048016//inositol phosphate-mediated signaling	12	2
GO:0048486//parasympathetic nervous system development	12	2
GO:0048635//negative regulation of muscle organ development	12	2
GO:0050482//arachidonic acid secretion	12	2
GO:0050748//negative regulation of lipoprotein metabolic process	12	2
GO:0051482//elevation of cytosolic calcium ion concentration involved in ph	12	2
GO:0051968//positive regulation of synaptic transmission, glutamatergic	12	2
GO:0055093//response to hyperoxia	12	2
GO:0060008//Sertoli cell differentiation	12	2
GO:0060602//branch elongation of an epithelium	12	2
GO:0060749//mammary gland alveolus development	12	2
GO:0061001//regulation of dendritic spine morphogenesis	12	2
GO:0061377//mammary gland lobule development	12	2
GO:0071354//cellular response to interleukin-6	12	2
GO:2000272//negative regulation of receptor activity	12	2
GO:2000696//regulation of epithelial cell differentiation involved in kidney de	12	2
GO:0010595//positive regulation of endothelial cell migration	41	4
GO:0042044//fluid transport	41	4
GO:0048709//oligodendrocyte differentiation	41	4
GO:0055117//regulation of cardiac muscle contraction	41	4
GO:0042439//ethanolamine-containing compound metabolic process	75	6
GO:0051091//positive regulation of sequence-specific DNA binding transcri	169	11
GO:0051098//regulation of binding	150	10
GO:0055092//sterol homeostasis	58	5
GO:0002675//positive regulation of acute inflammatory response	26	3
GO:0009167//purine ribonucleoside monophosphate metabolic process	26	3
GO:0032732//positive regulation of interleukin-1 production	26	3
GO:0033003//regulation of mast cell activation	26	3
GO:0035315//hair cell differentiation	26	3
GO:0042304//regulation of fatty acid biosynthetic process	26	3
GO:0046850//regulation of bone remodeling	26	3
GO:0048538//thymus development	26	3
GO:0050922//negative regulation of chemotaxis	26	3
GO:0051963//regulation of synapse assembly	26	3
GO:0019538//protein metabolic process	3191	144
GO:0050680//negative regulation of epithelial cell proliferation	76	6
GO:0016310//phosphorylation	812	41
GO:0030800//negative regulation of cyclic nucleotide metabolic process	42	4
GO:0030815//negative regulation of cAMP metabolic process	42	4
GO:0042787//protein ubiquitination involved in ubiquitin-dependent protein	42	4
GO:0006812//cation transport	592	31

GO:0001824//blastocyst development	59	5
GO:0006026//aminoglycan catabolic process	59	5
GO:0009101//glycoprotein biosynthetic process	273	16
GO:0097305//response to alcohol	172	11
GO:0031344//regulation of cell projection organization	212	13
GO:0001704//formation of primary germ layer	77	6
GO:0006919//activation of cysteine-type endopeptidase activity involved in	77	6
GO:0007369//gastrulation	114	8
GO:0051604//protein maturation	114	8
GO:0050865//regulation of cell activation	400	22
GO:0006643//membrane lipid metabolic process	153	10
GO:0001825//blastocyst formation	27	3
GO:0005342//organic acid transmembrane transporter activity	27	3
GO:0009126//purine nucleoside monophosphate metabolic process	27	3
GO:0014003//oligodendrocyte development	27	3
GO:0030239//myofibril assembly	27	3
GO:0046943//carboxylic acid transmembrane transporter activity	27	3
GO:0090278//negative regulation of peptide hormone secretion	27	3
GO:2000179//positive regulation of neural precursor cell proliferation	27	3
GO:0002253//activation of immune response	337	19
GO:0034220//ion transmembrane transport	337	19
GO:0001573//ganglioside metabolic process	13	2
GO:0002021//response to dietary excess	13	2
GO:0002548//monocyte chemotaxis	13	2
GO:0002701//negative regulation of production of molecular mediator of im	13	2
GO:0002719//negative regulation of cytokine production involved in immune	13	2
GO:0006098//pentose-phosphate shunt	13	2
GO:0010744//positive regulation of macrophage derived foam cell differenti	13	2
GO:0030041//actin filament polymerization	13	2
GO:0031290//retinal ganglion cell axon guidance	13	2
GO:0036148//phosphatidylglycerol acyl-chain remodeling	13	2
GO:0036150//phosphatidylserine acyl-chain remodeling	13	2
GO:0048103//somatic stem cell division	13	2
GO:0050919//negative chemotaxis	13	2
GO:0060026//convergent extension	13	2
GO:0071599//otic vesicle development	13	2
GO:0071887//leukocyte apoptotic process	13	2
GO:2000021//regulation of ion homeostasis	134	9
GO:0007189//adenylate cyclase-activating G-protein coupled receptor sign	43	4
GO:0043604//amide biosynthetic process	43	4
GO:0045665//negative regulation of neuron differentiation	43	4
GO:0043271//negative regulation of ion transport	60	5
GO:0045744//negative regulation of G-protein coupled receptor protein sigr	60	5
GO:0019827//stem cell maintenance	78	6
GO:0048593//camera-type eye morphogenesis	78	6
GO:0070663//regulation of leukocyte proliferation	154	10
GO:1901576//organic substance biosynthetic process	2080	96
GO:0046165//alcohol biosynthetic process	97	7
GO:0006576//cellular biogenic amine metabolic process	116	8
GO:0044106//cellular amine metabolic process	116	8
GO:0001707//mesoderm formation	61	5
GO:0048145//regulation of fibroblast proliferation	61	5
GO:0031326//regulation of cellular biosynthetic process	3188	143
GO:0048167//regulation of synaptic plasticity	79	6
GO:0002440//production of molecular mediator of immune response	44	4
GO:0019432//triglyceride biosynthetic process	44	4
GO:0072089//stem cell proliferation	44	4
GO:2000177//regulation of neural precursor cell proliferation	44	4

GO:0002792//negative regulation of peptide secretion	28	3
GO:0010633//negative regulation of epithelial cell migration	28	3
GO:0015662//ATPase activity, coupled to transmembrane movement of ions	28	3
GO:0030501//positive regulation of bone mineralization	28	3
GO:0031100//organ regeneration	28	3
GO:0031128//developmental induction	28	3
GO:0032436//positive regulation of proteasomal ubiquitin-dependent protein	28	3
GO:0035136//forelimb morphogenesis	28	3
GO:0048013//ephrin receptor signaling pathway	28	3
GO:0060416//response to growth hormone stimulus	28	3
GO:0070098//chemokine-mediated signaling pathway	28	3
GO:0050671//positive regulation of lymphocyte proliferation	98	7
GO:0010468//regulation of gene expression	3215	144
GO:0006631//fatty acid metabolic process	258	15
GO:0019221//cytokine-mediated signaling pathway	300	17
GO:0002687//positive regulation of leukocyte migration	80	6
GO:0045017//glycerolipid biosynthetic process	197	12
GO:0019829//cation-transporting ATPase activity	62	5
GO:0055007//cardiac muscle cell differentiation	62	5
GO:0006468//protein phosphorylation	559	29
GO:0022412//cellular process involved in reproduction in multicellular organ	157	10
GO:2000116//regulation of cysteine-type endopeptidase activity	157	10
GO:0006915//apoptotic process	603	31
GO:0032946//positive regulation of mononuclear cell proliferation	99	7
GO:0046546//development of primary male sexual characteristics	99	7
GO:0002002//regulation of angiotensin levels in blood	14	2
GO:0005452//inorganic anion exchanger activity	14	2
GO:0005978//glycogen biosynthetic process	14	2
GO:0006740//NADPH regeneration	14	2
GO:0006855//drug transmembrane transport	14	2
GO:0009250//glucan biosynthetic process	14	2
GO:0010460//positive regulation of heart rate	14	2
GO:0015695//organic cation transport	14	2
GO:0022410//circadian sleep/wake cycle process	14	2
GO:0030260//entry into host cell	14	2
GO:0033158//regulation of protein import into nucleus, translocation	14	2
GO:0035809//regulation of urine volume	14	2
GO:0044409//entry into host	14	2
GO:0050910//detection of mechanical stimulus involved in sensory percepti	14	2
GO:0051769//regulation of nitric-oxide synthase biosynthetic process	14	2
GO:0051806//entry into cell of other organism involved in symbiotic interact	14	2
GO:0051828//entry into other organism involved in symbiotic interaction	14	2
GO:0051917//regulation of fibrinolysis	14	2
GO:0052126//movement in host environment	14	2
GO:0052192//movement in environment of other organism involved in symb	14	2
GO:0060038//cardiac muscle cell proliferation	14	2
GO:0060177//regulation of angiotensin metabolic process	14	2
GO:0060384//innervation	14	2
GO:0060487//lung epithelial cell differentiation	14	2
GO:0060664//epithelial cell proliferation involved in salivary gland morphoge	14	2
GO:0071715//icosanoid transport	14	2
GO:1901571//fatty acid derivative transport	14	2
GO:2000351//regulation of endothelial cell apoptotic process	14	2
GO:0001776//leukocyte homeostasis	45	4
GO:0009914//hormone transport	45	4
GO:0015297//antiporter activity	45	4
GO:1900543//negative regulation of purine nucleotide metabolic process	45	4
GO:0050954//sensory perception of mechanical stimulus	119	8

GO:0007585//respiratory gaseous exchange	29	3
GO:0008088//axon cargo transport	29	3
GO:0010828//positive regulation of glucose transport	29	3
GO:0048814//regulation of dendrite morphogenesis	29	3
GO:0070169//positive regulation of biomineral tissue development	29	3
GO:0043112//receptor metabolic process	63	5
GO:0051099//positive regulation of binding	63	5
GO:0009247//glycolipid biosynthetic process	46	4
GO:0016050//vesicle organization	82	6
GO:0006767//water-soluble vitamin metabolic process	64	5
GO:0032387//negative regulation of intracellular transport	64	5
GO:0048332//mesoderm morphogenesis	64	5
GO:0032386//regulation of intracellular transport	263	15
GO:0003012//muscle system process	181	11
GO:0022600//digestive system process	30	3
GO:0033692//cellular polysaccharide biosynthetic process	30	3
GO:0042339//keratan sulfate metabolic process	30	3
GO:0060393//regulation of pathway-restricted SMAD protein phosphorylatic	30	3
GO:1901800//positive regulation of proteasomal protein catabolic process	30	3
GO:0009583//detection of light stimulus	83	6
GO:0006694//steroid biosynthetic process	102	7
GO:0070665//positive regulation of leukocyte proliferation	102	7
GO:2001056//positive regulation of cysteine-type endopeptidase activity	102	7
GO:0003215//cardiac right ventricle morphogenesis	15	2
GO:0009110//vitamin biosynthetic process	15	2
GO:0014855//striated muscle cell proliferation	15	2
GO:0031641//regulation of myelination	15	2
GO:0031650//regulation of heat generation	15	2
GO:0042523//positive regulation of tyrosine phosphorylation of Stat5 proteir	15	2
GO:0045070//positive regulation of viral genome replication	15	2
GO:0045601//regulation of endothelial cell differentiation	15	2
GO:0045742//positive regulation of epidermal growth factor receptor signali	15	2
GO:0046716//muscle cell cellular homeostasis	15	2
GO:0048070//regulation of developmental pigmentation	15	2
GO:0060998//regulation of dendritic spine development	15	2
GO:0061099//negative regulation of protein tyrosine kinase activity	15	2
GO:0061311//cell surface receptor signaling pathway involved in heart deve	15	2
GO:0070059//intrinsic apoptotic signaling pathway in response to endoplasi	15	2
GO:0080111//DNA demethylation	15	2
GO:0086002//regulation of cardiac muscle cell action potential involved in c	15	2
GO:2000811//negative regulation of anoikis	15	2
GO:0001938//positive regulation of endothelial cell proliferation	47	4
GO:0071260//cellular response to mechanical stimulus	47	4
GO:0002065//columnar/cuboidal epithelial cell differentiation	65	5
GO:0006633//fatty acid biosynthetic process	103	7
GO:0015850//organic hydroxy compound transport	84	6
GO:0007224//smoothened signaling pathway	48	4
GO:0021761//limbic system development	48	4
GO:0021987//cerebral cortex development	48	4
GO:0045980//negative regulation of nucleotide metabolic process	48	4
GO:0000271//polysaccharide biosynthetic process	31	3
GO:0005977//glycogen metabolic process	31	3
GO:0015909//long-chain fatty acid transport	31	3
GO:0055006//cardiac cell development	31	3
GO:0055081//anion homeostasis	31	3
GO:0072529//pyrimidine-containing compound catabolic process	31	3
GO:0030968//endoplasmic reticulum unfolded protein response	66	5
GO:0071241//cellular response to inorganic substance	66	5

GO:0046873//metal ion transmembrane transporter activity	310	17
GO:0008584//male gonad development	85	6
GO:0030902//hindbrain development	85	6
GO:0009308//amine metabolic process	124	8
GO:0044003//modification by symbiont of host morphology or physiology	247	14
GO:0018193//peptidyl-amino acid modification	441	23
GO:0021602//cranial nerve morphogenesis	16	2
GO:0022011//myelination in peripheral nervous system	16	2
GO:0032292//peripheral nervous system axon ensheathment	16	2
GO:0033630//positive regulation of cell adhesion mediated by integrin	16	2
GO:0033632//regulation of cell-cell adhesion mediated by integrin	16	2
GO:0042104//positive regulation of activated T cell proliferation	16	2
GO:0043372//positive regulation of CD4-positive, alpha-beta T cell different	16	2
GO:0045671//negative regulation of osteoclast differentiation	16	2
GO:0045736//negative regulation of cyclin-dependent protein serine/threoni	16	2
GO:0045745//positive regulation of G-protein coupled receptor protein signa	16	2
GO:0050901//leukocyte tethering or rolling	16	2
GO:0051155//positive regulation of striated muscle cell differentiation	16	2
GO:0055017//cardiac muscle tissue growth	16	2
GO:0071526//semaphorin-plexin signaling pathway	16	2
GO:0090023//positive regulation of neutrophil chemotaxis	16	2
GO:1901186//positive regulation of ERBB signaling pathway	16	2
GO:0034614//cellular response to reactive oxygen species	67	5
GO:0034620//cellular response to unfolded protein	67	5
GO:1901654//response to ketone	67	5
GO:0030099//myeloid cell differentiation	145	9
GO:0015918//sterol transport	49	4
GO:0018208//peptidyl-proline modification	49	4
GO:0030301//cholesterol transport	49	4
GO:0034332//adherens junction organization	49	4
GO:0046324//regulation of glucose import	49	4
GO:0006073//cellular glucan metabolic process	32	3
GO:0044042//glucan metabolic process	32	3
GO:0048521//negative regulation of behavior	32	3
GO:0070206//protein trimerization	32	3
GO:0043687//post-translational protein modification	166	10
GO:0006986//response to unfolded protein	106	7
GO:0010950//positive regulation of endopeptidase activity	106	7
GO:0042129//regulation of T cell proliferation	106	7
GO:0043433//negative regulation of sequence-specific DNA binding transcr	106	7
GO:0046661//male sex differentiation	106	7
GO:1901617//organic hydroxy compound biosynthetic process	126	8
GO:0009314//response to radiation	314	17
GO:0022836//gated channel activity	208	12
GO:0022839//ion gated channel activity	208	12
GO:0004721//phosphoprotein phosphatase activity	87	6
GO:0004725//protein tyrosine phosphatase activity	87	6
GO:0010959//regulation of metal ion transport	167	10
GO:0071704//organic substance metabolic process	6497	278
GO:0006672//ceramide metabolic process	50	4
GO:0010811//positive regulation of cell-substrate adhesion	50	4
GO:0030148//sphingolipid biosynthetic process	50	4
GO:0044264//cellular polysaccharide metabolic process	50	4
GO:0061180//mammary gland epithelium development	50	4
GO:0007586//digestion	88	6
GO:0042886//amide transport	69	5
GO:0045862//positive regulation of proteolysis	69	5
GO:0015077//monovalent inorganic cation transmembrane transporter activ	231	13

GO:0002790//peptide secretion	33	3
GO:0006953//acute-phase response	33	3
GO:0010332//response to gamma radiation	33	3
GO:0030593//neutrophil chemotaxis	33	3
GO:0030803//negative regulation of cyclic nucleotide biosynthetic process	33	3
GO:0030818//negative regulation of cAMP biosynthetic process	33	3
GO:0044273//sulfur compound catabolic process	33	3
GO:0071827//plasma lipoprotein particle organization	33	3
GO:0051171//regulation of nitrogen compound metabolic process	3364	148
GO:0043281//regulation of cysteine-type endopeptidase activity involved in	148	9
GO:0002089//lens morphogenesis in camera-type eye	17	2
GO:0006829//zinc ion transport	17	2
GO:0006972//hyperosmotic response	17	2
GO:0007250//activation of NF-kappaB-inducing kinase activity	17	2
GO:0010771//negative regulation of cell morphogenesis involved in differen	17	2
GO:0021795//cerebral cortex cell migration	17	2
GO:0034698//response to gonadotropin stimulus	17	2
GO:0042107//cytokine metabolic process	17	2
GO:0042522//regulation of tyrosine phosphorylation of Stat5 protein	17	2
GO:0042745//circadian sleep/wake cycle	17	2
GO:0045737//positive regulation of cyclin-dependent protein kinase activity	17	2
GO:0045747//positive regulation of Notch signaling pathway	17	2
GO:0046688//response to copper ion	17	2
GO:0086065//cell communication involved in cardiac conduction	17	2
GO:1901623//regulation of lymphocyte chemotaxis	17	2
GO:2000273//positive regulation of receptor activity	17	2
GO:0030814//regulation of cAMP metabolic process	108	7
GO:0048644//muscle organ morphogenesis	51	4
GO:0008152//metabolic process	7070	301
GO:0006796//phosphate-containing compound metabolic process	1781	81
GO:0002695//negative regulation of leukocyte activation	109	7
GO:0030808//regulation of nucleotide biosynthetic process	109	7
GO:1900371//regulation of purine nucleotide biosynthetic process	109	7
GO:0044249//cellular biosynthetic process	1995	90
GO:0006654//phosphatidic acid biosynthetic process	34	3
GO:0046473//phosphatidic acid metabolic process	34	3
GO:0071825//protein-lipid complex subunit organization	34	3
GO:0015103//inorganic anion transmembrane transporter activity	90	6
GO:0032319//regulation of Rho GTPase activity	90	6
GO:0021537//telencephalon development	130	8
GO:0016579//protein deubiquitination	52	4
GO:0042180//cellular ketone metabolic process	52	4
GO:0007605//sensory perception of sound	110	7
GO:0001759//organ induction	18	2
GO:0001953//negative regulation of cell-matrix adhesion	18	2
GO:0003254//regulation of membrane depolarization	18	2
GO:0010575//positive regulation vascular endothelial growth factor producti	18	2
GO:0014044//Schwann cell development	18	2
GO:0030539//male genitalia development	18	2
GO:0032410//negative regulation of transporter activity	18	2
GO:0033002//muscle cell proliferation	18	2
GO:0035088//establishment or maintenance of apical/basal cell polarity	18	2
GO:0035510//DNA dealkylation	18	2
GO:0035813//regulation of renal sodium excretion	18	2
GO:0036152//phosphatidylethanolamine acyl-chain remodeling	18	2
GO:0042417//dopamine metabolic process	18	2
GO:0042755//eating behavior	18	2
GO:0048745//smooth muscle tissue development	18	2

GO:0050718//positive regulation of interleukin-1 beta secretion	18	2
GO:0060419//heart growth	18	2
GO:0060600//dichotomous subdivision of an epithelial terminal unit	18	2
GO:0061245//establishment or maintenance of bipolar cell polarity	18	2
GO:0071624//positive regulation of granulocyte chemotaxis	18	2
GO:0086004//regulation of cardiac muscle cell contraction	18	2
GO:0090190//positive regulation of branching involved in ureteric bud morp	18	2
GO:0090279//regulation of calcium ion import	18	2
GO:1901222//regulation of NIK/NF-kappaB cascade	18	2
GO:0016788//hydrolase activity, acting on ester bonds	91	6
GO:0016791//phosphatase activity	91	6
GO:0042578//phosphoric ester hydrolase activity	91	6
GO:0043279//response to alkaloid	91	6
GO:0010952//positive regulation of peptidase activity	111	7
GO:0021915//neural tube development	111	7
GO:0006970//response to osmotic stress	53	4
GO:0016126//sterol biosynthetic process	35	3
GO:0030809//negative regulation of nucleotide biosynthetic process	35	3
GO:0043966//histone H3 acetylation	35	3
GO:0050982//detection of mechanical stimulus	35	3
GO:0051492//regulation of stress fiber assembly	35	3
GO:1900372//negative regulation of purine nucleotide biosynthetic process	35	3
GO:0006936//muscle contraction	153	9
GO:0007601//visual perception	153	9
GO:0009056//catabolic process	1631	74
GO:0032388//positive regulation of intracellular transport	133	8
GO:0050953//sensory perception of light stimulus	154	9
GO:0009306//protein secretion	54	4
GO:0032642//regulation of chemokine production	54	4
GO:0043407//negative regulation of MAP kinase activity	54	4
GO:0051701//interaction with host	282	15
GO:0002064//epithelial cell development	175	10
GO:0008360//regulation of cell shape	93	6
GO:0030817//regulation of cAMP biosynthetic process	93	6
GO:0019058//viral infectious cycle	36	3
GO:0032651//regulation of interleukin-1 beta production	36	3
GO:0034121//regulation of toll-like receptor signaling pathway	36	3
GO:0035094//response to nicotine	36	3
GO:0043535//regulation of blood vessel endothelial cell migration	36	3
GO:0001659//temperature homeostasis	19	2
GO:0002066//columnar/cuboidal epithelial cell development	19	2
GO:0006541//glutamine metabolic process	19	2
GO:0009084//glutamine family amino acid biosynthetic process	19	2
GO:0009268//response to pH	19	2
GO:0010226//response to lithium ion	19	2
GO:0015144//carbohydrate transmembrane transporter activity	19	2
GO:0015721//bile acid and bile salt transport	19	2
GO:0032873//negative regulation of stress-activated MAPK cascade	19	2
GO:0034333//adherens junction assembly	19	2
GO:0035850//epithelial cell differentiation involved in kidney development	19	2
GO:0043534//blood vessel endothelial cell migration	19	2
GO:0043536//positive regulation of blood vessel endothelial cell migration	19	2
GO:0046209//nitric oxide metabolic process	19	2
GO:0046320//regulation of fatty acid oxidation	19	2
GO:0051119//sugar transmembrane transporter activity	19	2
GO:0051602//response to electrical stimulus	19	2
GO:0051898//negative regulation of protein kinase B signaling cascade	19	2
GO:0060043//regulation of cardiac muscle cell proliferation	19	2

GO:0060968//regulation of gene silencing	19	2
GO:0070303//negative regulation of stress-activated protein kinase signalin	19	2
GO:1901476//carbohydrate transporter activity	19	2
GO:2000516//positive regulation of CD4-positive, alpha-beta T cell activatio	19	2
GO:0046474//glycerophospholipid biosynthetic process	176	10
GO:0021543//pallium development	74	5
GO:0010721//negative regulation of cell development	94	6
GO:0045834//positive regulation of lipid metabolic process	94	6
GO:0046887//positive regulation of hormone secretion	55	4
GO:0019048//modulation by virus of host morphology or physiology	241	13
GO:0001547//antral ovarian follicle growth	5	1
GO:0001554//luteolysis	5	1
GO:0001778//plasma membrane repair	5	1
GO:0001911//negative regulation of leukocyte mediated cytotoxicity	5	1
GO:0002467//germinal center formation	5	1
GO:0003032//detection of oxygen	5	1
GO:0003094//glomerular filtration	5	1
GO:0003100//regulation of systemic arterial blood pressure by endothelin	5	1
GO:0003174//mitral valve development	5	1
GO:0003307//regulation of Wnt receptor signaling pathway involved in hear	5	1
GO:0003308//negative regulation of Wnt receptor signaling pathway involve	5	1
GO:0003417//growth plate cartilage development	5	1
GO:0006003//fructose 2,6-bisphosphate metabolic process	5	1
GO:0006167//AMP biosynthetic process	5	1
GO:0006572//tyrosine catabolic process	5	1
GO:0006678//glucosylceramide metabolic process	5	1
GO:0006787//porphyrin-containing compound catabolic process	5	1
GO:0006857//oligopeptide transport	5	1
GO:0006925//inflammatory cell apoptotic process	5	1
GO:0007028//cytoplasm organization	5	1
GO:0007262//STAT protein import into nucleus	5	1
GO:0007442//hindgut morphogenesis	5	1
GO:0007501//mesodermal cell fate specification	5	1
GO:0007598//blood coagulation, extrinsic pathway	5	1
GO:0008216//spermidine metabolic process	5	1
GO:0009052//pentose-phosphate shunt, non-oxidative branch	5	1
GO:0009128//purine nucleoside monophosphate catabolic process	5	1
GO:0009133//nucleoside diphosphate biosynthetic process	5	1
GO:0009448//gamma-aminobutyric acid metabolic process	5	1
GO:0009744//response to sucrose stimulus	5	1
GO:0009912//auditory receptor cell fate commitment	5	1
GO:0010766//negative regulation of sodium ion transport	5	1
GO:0015106//bicarbonate transmembrane transporter activity	5	1
GO:0015180//L-alanine transmembrane transporter activity	5	1
GO:0015220//choline transmembrane transporter activity	5	1
GO:0015464//acetylcholine receptor activity	5	1
GO:0015665//alcohol transmembrane transporter activity	5	1
GO:0015840//urea transport	5	1
GO:0015865//purine nucleotide transport	5	1
GO:0015868//purine ribonucleotide transport	5	1
GO:0016907//G-protein coupled acetylcholine receptor activity	5	1
GO:0021557//oculomotor nerve development	5	1
GO:0021561//facial nerve development	5	1
GO:0021604//cranial nerve structural organization	5	1
GO:0021683//cerebellar granular layer morphogenesis	5	1
GO:0021869//forebrain ventricular zone progenitor cell division	5	1
GO:0022601//menstrual cycle phase	5	1
GO:0022614//membrane to membrane docking	5	1

GO:0022858//alanine transmembrane transporter activity	5	1
GO:0030042//actin filament depolymerization	5	1
GO:0031017//exocrine pancreas development	5	1
GO:0031115//negative regulation of microtubule polymerization	5	1
GO:0031223//auditory behavior	5	1
GO:0031342//negative regulation of cell killing	5	1
GO:0031392//regulation of prostaglandin biosynthetic process	5	1
GO:0031427//response to methotrexate	5	1
GO:0031915//positive regulation of synaptic plasticity	5	1
GO:0031936//negative regulation of chromatin silencing	5	1
GO:0032224//positive regulation of synaptic transmission, cholinergic	5	1
GO:0032277//negative regulation of gonadotropin secretion	5	1
GO:0032364//oxygen homeostasis	5	1
GO:0032747//positive regulation of interleukin-23 production	5	1
GO:0032854//positive regulation of Rap GTPase activity	5	1
GO:0032964//collagen biosynthetic process	5	1
GO:0033015//tetrapyrrole catabolic process	5	1
GO:0033633//negative regulation of cell-cell adhesion mediated by integrin	5	1
GO:0034285//response to disaccharide stimulus	5	1
GO:0034372//very-low-density lipoprotein particle remodeling	5	1
GO:0035330//regulation of hippo signaling cascade	5	1
GO:0035356//cellular triglyceride homeostasis	5	1
GO:0035624//receptor transactivation	5	1
GO:0036155//acylglycerol acyl-chain remodeling	5	1
GO:0042167//heme catabolic process	5	1
GO:0042723//thiamine-containing compound metabolic process	5	1
GO:0043476//pigment accumulation	5	1
GO:0043482//cellular pigment accumulation	5	1
GO:0043587//tongue morphogenesis	5	1
GO:0045714//regulation of low-density lipoprotein particle receptor biosynth	5	1
GO:0045743//positive regulation of fibroblast growth factor receptor signalir	5	1
GO:0045869//negative regulation of retroviral genome replication	5	1
GO:0046031//ADP metabolic process	5	1
GO:0046149//pigment catabolic process	5	1
GO:0046469//platelet activating factor metabolic process	5	1
GO:0046835//carbohydrate phosphorylation	5	1
GO:0048642//negative regulation of skeletal muscle tissue development	5	1
GO:0048664//neuron fate determination	5	1
GO:0051503//adenine nucleotide transport	5	1
GO:0051547//regulation of keratinocyte migration	5	1
GO:0051549//positive regulation of keratinocyte migration	5	1
GO:0051597//response to methylmercury	5	1
GO:0051608//histamine transport	5	1
GO:0051973//positive regulation of telomerase activity	5	1
GO:0060087//relaxation of vascular smooth muscle	5	1
GO:0060120//inner ear receptor cell fate commitment	5	1
GO:0060157//urinary bladder development	5	1
GO:0060297//regulation of sarcomere organization	5	1
GO:0060389//pathway-restricted SMAD protein phosphorylation	5	1
GO:0060452//positive regulation of cardiac muscle contraction	5	1
GO:0060605//tube lumen cavitation	5	1
GO:0060662//salivary gland cavitation	5	1
GO:0060687//regulation of branching involved in prostate gland morphogen	5	1
GO:0060696//regulation of phospholipid catabolic process	5	1
GO:0060710//chorio-allantoic fusion	5	1
GO:0060977//coronary vasculature morphogenesis	5	1
GO:0061037//negative regulation of cartilage development	5	1
GO:0061316//canonical Wnt receptor signaling pathway involved in heart d	5	1

GO:0061525//hindgut development	5	1
GO:0070120//ciliary neurotrophic factor-mediated signaling pathway	5	1
GO:0070295//renal water absorption	5	1
GO:0070383//DNA cytosine deamination	5	1
GO:0070424//regulation of nucleotide-binding oligomerization domain conta	5	1
GO:0071361//cellular response to ethanol	5	1
GO:0071380//cellular response to prostaglandin E stimulus	5	1
GO:0071503//response to heparin	5	1
GO:0071680//response to indole-3-methanol	5	1
GO:0071681//cellular response to indole-3-methanol	5	1
GO:0071731//response to nitric oxide	5	1
GO:0072008//glomerular mesangial cell differentiation	5	1
GO:0072124//regulation of glomerular mesangial cell proliferation	5	1
GO:0072143//mesangial cell development	5	1
GO:0072161//mesenchymal cell differentiation involved in kidney developm	5	1
GO:0072171//mesonephric tubule morphogenesis	5	1
GO:0072298//regulation of metanephric glomerulus development	5	1
GO:0072307//regulation of metanephric nephron tubule epithelial cell differe	5	1
GO:0072338//cellular lactam metabolic process	5	1
GO:0090232//positive regulation of spindle checkpoint	5	1
GO:0090244//Wnt receptor signaling pathway involved in somitogenesis	5	1
GO:0090381//regulation of heart induction	5	1
GO:0097205//renal filtration	5	1
GO:1900027//regulation of ruffle assembly	5	1
GO:1900107//regulation of nodal signaling pathway	5	1
GO:1901385//regulation of voltage-gated calcium channel activity	5	1
GO:1901618//organic hydroxy compound transmembrane transporter activi	5	1
GO:1901978//positive regulation of cell cycle checkpoint	5	1
GO:2000018//regulation of male gonad development	5	1
GO:2000048//negative regulation of cell-cell adhesion mediated by cadherir	5	1
GO:2000279//negative regulation of DNA biosynthetic process	5	1
GO:2000353//positive regulation of endothelial cell apoptotic process	5	1
GO:2000738//positive regulation of stem cell differentiation	5	1
GO:2000973//regulation of pro-B cell differentiation	5	1
GO:2001012//mesenchymal cell differentiation involved in renal system dev	5	1
GO:2001279//regulation of unsaturated fatty acid biosynthetic process	5	1
GO:NM_0032	5	1
GO:NM_0048	5	1
GO:NM_0062	5	1
GO:NM_0124	5	1
GO:NM_0230	5	1
GO:NR_0021	5	1
GO:0006464//cellular protein modification process	1902	85
GO:0036211//protein modification process	1902	85
GO:0007259//JAK-STAT cascade	37	3
GO:0009187//cyclic nucleotide metabolic process	37	3
GO:0034103//regulation of tissue remodeling	37	3
GO:0042993//positive regulation of transcription factor import into nucleus	37	3
GO:0048806//genitalia development	37	3
GO:0001818//negative regulation of cytokine production	136	8
GO:0030162//regulation of proteolysis	157	9
GO:0002694//regulation of leukocyte activation	375	19
GO:0051899//membrane depolarization	56	4
GO:0002026//regulation of the force of heart contraction	20	2
GO:0005242//inward rectifier potassium channel activity	20	2
GO:0006658//phosphatidylserine metabolic process	20	2
GO:0007220//Notch receptor processing	20	2
GO:0010453//regulation of cell fate commitment	20	2

GO:0010888//negative regulation of lipid storage	20	2
GO:0021952//central nervous system projection neuron axonogenesis	20	2
GO:0030101//natural killer cell activation	20	2
GO:0031102//neuron projection regeneration	20	2
GO:0033144//negative regulation of intracellular steroid hormone receptor s	20	2
GO:0033198//response to ATP	20	2
GO:0034308//primary alcohol metabolic process	20	2
GO:0044062//regulation of excretion	20	2
GO:0045073//regulation of chemokine biosynthetic process	20	2
GO:0046579//positive regulation of Ras protein signal transduction	20	2
GO:0048512//circadian behavior	20	2
GO:0048596//embryonic camera-type eye morphogenesis	20	2
GO:0050706//regulation of interleukin-1 beta secretion	20	2
GO:0051926//negative regulation of calcium ion transport	20	2
GO:0055021//regulation of cardiac muscle tissue growth	20	2
GO:0071320//cellular response to cAMP	20	2
GO:1900087//positive regulation of G1/S transition of mitotic cell cycle	20	2
GO:2001237//negative regulation of extrinsic apoptotic signaling pathway	20	2
GO:0050792//regulation of viral process	96	6
GO:0000079//regulation of cyclin-dependent protein serine/threonine kinase	76	5
GO:0006984//ER-nucleus signaling pathway	76	5
GO:0015718//monocarboxylic acid transport	76	5
GO:0050870//positive regulation of T cell activation	201	11
GO:0006833//water transport	38	3
GO:0014823//response to activity	38	3
GO:0015908//fatty acid transport	38	3
GO:0042308//negative regulation of protein import into nucleus	38	3
GO:0048741//skeletal muscle fiber development	38	3
GO:1901880//negative regulation of protein depolymerization	38	3
GO:0045833//negative regulation of lipid metabolic process	57	4
GO:0007188//adenylate cyclase-modulating G-protein coupled receptor sig	97	6
GO:0043280//positive regulation of cysteine-type endopeptidase activity inv	97	6
GO:0009913//epidermal cell differentiation	77	5
GO:0035967//cellular response to topologically incorrect protein	77	5
GO:0044087//regulation of cellular component biogenesis	312	16
GO:0051817//modification of morphology or physiology of other organism ir	268	14
GO:0015079//potassium ion transmembrane transporter activity	118	7
GO:0035966//response to topologically incorrect protein	118	7
GO:0005244//voltage-gated ion channel activity	139	8
GO:0022832//voltage-gated channel activity	139	8
GO:0006638//neutral lipid metabolic process	98	6
GO:0006639//acylglycerol metabolic process	98	6
GO:0001823//mesonephros development	21	2
GO:0003197//endocardial cushion development	21	2
GO:0005247//voltage-gated chloride channel activity	21	2
GO:0007622//rhythmic behavior	21	2
GO:0008308//voltage-gated anion channel activity	21	2
GO:0043113//receptor clustering	21	2
GO:0043124//negative regulation of I-kappaB kinase/NF-kappaB cascade	21	2
GO:0045879//negative regulation of smoothened signaling pathway	21	2
GO:0046135//pyrimidine nucleoside catabolic process	21	2
GO:0046717//acid secretion	21	2
GO:0048048//embryonic eye morphogenesis	21	2
GO:0001843//neural tube closure	58	4
GO:0042110//T cell activation	161	9
GO:0002260//lymphocyte homeostasis	39	3
GO:0009161//ribonucleoside monophosphate metabolic process	39	3
GO:0032720//negative regulation of tumor necrosis factor production	39	3

GO:0042345//regulation of NF-kappaB import into nucleus	39	3
GO:0045861//negative regulation of proteolysis	39	3
GO:0002262//myeloid cell homeostasis	78	5
GO:0050729//positive regulation of inflammatory response	78	5
GO:0009416//response to light stimulus	226	12
GO:0019219//regulation of nucleobase-containing compound metabolic pro	3304	143
GO:0006793//phosphorus metabolic process	1824	81
GO:1900542//regulation of purine nucleotide metabolic process	405	20
GO:0051924//regulation of calcium ion transport	120	7
GO:0003014//renal system process	59	4
GO:0009798//axis specification	59	4
GO:0060606//tube closure	59	4
GO:0030097//hemopoiesis	361	18
GO:0001514//selenocysteine incorporation	6	1
GO:0002072//optic cup morphogenesis involved in camera-type eye develo	6	1
GO:0002327//immature B cell differentiation	6	1
GO:0002866//positive regulation of acute inflammatory response to antigen	6	1
GO:0002885//positive regulation of hypersensitivity	6	1
GO:0003097//renal water transport	6	1
GO:0003306//Wnt receptor signaling pathway involved in heart developmer	6	1
GO:0006290//pyrimidine dimer repair	6	1
GO:0006451//translational readthrough	6	1
GO:0006477//protein sulfation	6	1
GO:0006537//glutamate biosynthetic process	6	1
GO:0006924//activation-induced cell death of T cells	6	1
GO:0008356//asymmetric cell division	6	1
GO:0009111//vitamin catabolic process	6	1
GO:0009186//deoxyribonucleoside diphosphate metabolic process	6	1
GO:0009996//negative regulation of cell fate specification	6	1
GO:0010614//negative regulation of cardiac muscle hypertrophy	6	1
GO:0010715//regulation of extracellular matrix disassembly	6	1
GO:0010867//positive regulation of triglyceride biosynthetic process	6	1
GO:0010890//positive regulation of sequestering of triglyceride	6	1
GO:0010944//negative regulation of transcription by competitive promoter b	6	1
GO:0014012//peripheral nervous system axon regeneration	6	1
GO:0014067//negative regulation of phosphatidylinositol 3-kinase cascade	6	1
GO:0014819//regulation of skeletal muscle contraction	6	1
GO:0015187//glycine transmembrane transporter activity	6	1
GO:0015808//L-alanine transport	6	1
GO:0015809//arginine transport	6	1
GO:0015816//glycine transport	6	1
GO:0016115//terpenoid catabolic process	6	1
GO:0021535//cell migration in hindbrain	6	1
GO:0021546//rhombomere development	6	1
GO:0021681//cerebellar granular layer development	6	1
GO:0021819//layer formation in cerebral cortex	6	1
GO:0021859//pyramidal neuron differentiation	6	1
GO:0021860//pyramidal neuron development	6	1
GO:0030300//regulation of intestinal cholesterol absorption	6	1
GO:0031639//plasminogen activation	6	1
GO:0031643//positive regulation of myelination	6	1
GO:0032222//regulation of synaptic transmission, cholinergic	6	1
GO:0032287//peripheral nervous system myelin maintenance	6	1
GO:0032623//interleukin-2 production	6	1
GO:0032667//regulation of interleukin-23 production	6	1
GO:0032811//negative regulation of epinephrine secretion	6	1
GO:0033147//negative regulation of intracellular estrogen receptor signaling	6	1
GO:0034350//regulation of glial cell apoptotic process	6	1

GO:0034351//negative regulation of glial cell apoptotic process	6	1
GO:0035728//response to hepatocyte growth factor stimulus	6	1
GO:0035729//cellular response to hepatocyte growth factor stimulus	6	1
GO:0035965//cardiolipin acyl-chain remodeling	6	1
GO:0040016//embryonic cleavage	6	1
GO:0042363//fat-soluble vitamin catabolic process	6	1
GO:0042482//positive regulation of odontogenesis	6	1
GO:0042661//regulation of mesodermal cell fate specification	6	1
GO:0043117//positive regulation of vascular permeability	6	1
GO:0043615//astrocyte cell migration	6	1
GO:0044557//relaxation of smooth muscle	6	1
GO:0045188//regulation of circadian sleep/wake cycle, non-REM sleep	6	1
GO:0045630//positive regulation of T-helper 2 cell differentiation	6	1
GO:0045634//regulation of melanocyte differentiation	6	1
GO:0045793//positive regulation of cell size	6	1
GO:0045916//negative regulation of complement activation	6	1
GO:0046596//regulation of viral entry into host cell	6	1
GO:0046628//positive regulation of insulin receptor signaling pathway	6	1
GO:0048643//positive regulation of skeletal muscle tissue development	6	1
GO:0048845//venous blood vessel morphogenesis	6	1
GO:0050942//positive regulation of pigment cell differentiation	6	1
GO:0051497//negative regulation of stress fiber assembly	6	1
GO:0055119//relaxation of cardiac muscle	6	1
GO:0060259//regulation of feeding behavior	6	1
GO:0060601//lateral sprouting from an epithelium	6	1
GO:0060969//negative regulation of gene silencing	6	1
GO:0060986//endocrine hormone secretion	6	1
GO:0060999//positive regulation of dendritic spine development	6	1
GO:0070166//enamel mineralization	6	1
GO:0070170//regulation of tooth mineralization	6	1
GO:0071071//regulation of phospholipid biosynthetic process	6	1
GO:0072007//mesangial cell differentiation	6	1
GO:0072173//metanephric tubule morphogenesis	6	1
GO:0072282//metanephric nephron tubule morphogenesis	6	1
GO:0086069//bundle of His cell to Purkinje myocyte communication	6	1
GO:0090280//positive regulation of calcium ion import	6	1
GO:0097090//presynaptic membrane organization	6	1
GO:1901016//regulation of potassium ion transmembrane transporter activi	6	1
GO:1901532//regulation of hematopoietic progenitor cell differentiation	6	1
GO:1901998//toxin transport	6	1
GO:2000188//regulation of cholesterol homeostasis	6	1
GO:2000258//negative regulation of protein activation cascade	6	1
GO:2001026//regulation of endothelial cell chemotaxis	6	1
GO:NM_0003	6	1
GO:NM_0013	6	1
GO:0031018//endocrine pancreas development	40	3
GO:0032231//regulation of actin filament bundle assembly	40	3
GO:0035265//organ growth	40	3
GO:0048385//regulation of retinoic acid receptor signaling pathway	40	3
GO:0050807//regulation of synapse organization	40	3
GO:0070988//demethylation	40	3
GO:0006813//potassium ion transport	121	7
GO:0043903//regulation of symbiosis, encompassing mutualism through pa	121	7
GO:0001937//negative regulation of endothelial cell proliferation	22	2
GO:0006706//steroid catabolic process	22	2
GO:0008299//isoprenoid biosynthetic process	22	2
GO:0030104//water homeostasis	22	2
GO:0032845//negative regulation of homeostatic process	22	2

GO:0035115//embryonic forelimb morphogenesis	22	2
GO:0035329//hippo signaling cascade	22	2
GO:0045622//regulation of T-helper cell differentiation	22	2
GO:0046006//regulation of activated T cell proliferation	22	2
GO:0048535//lymph node development	22	2
GO:0060119//inner ear receptor cell development	22	2
GO:0061337//cardiac conduction	22	2
GO:0070373//negative regulation of ERK1 and ERK2 cascade	22	2
GO:0006140//regulation of nucleotide metabolic process	408	20
GO:0042157//lipoprotein metabolic process	80	5
GO:0015833//peptide transport	60	4
GO:0010556//regulation of macromolecule biosynthetic process	3052	132
GO:0007276//gamete generation	454	22
GO:0046649//lymphocyte activation	252	13
GO:0055008//cardiac muscle tissue morphogenesis	41	3
GO:0071621//granulocyte chemotaxis	41	3
GO:0015081//sodium ion transmembrane transporter activity	81	5
GO:0046467//membrane lipid biosynthetic process	81	5
GO:0007281//germ cell development	123	7
GO:0005267//potassium channel activity	102	6
GO:0007498//mesoderm development	102	6
GO:0070646//protein modification by small protein removal	61	4
GO:0006887//exocytosis	188	10
GO:0030900//forebrain development	232	12
GO:0090068//positive regulation of cell cycle process	145	8
GO:0005979//regulation of glycogen biosynthetic process	23	2
GO:0010962//regulation of glucan biosynthetic process	23	2
GO:0014037//Schwann cell differentiation	23	2
GO:0015804//neutral amino acid transport	23	2
GO:0021955//central nervous system neuron axonogenesis	23	2
GO:0030049//muscle filament sliding	23	2
GO:0030835//negative regulation of actin filament depolymerization	23	2
GO:0032731//positive regulation of interleukin-1 beta production	23	2
GO:0038061//NIK/NF-kappaB cascade	23	2
GO:0050869//negative regulation of B cell activation	23	2
GO:0051496//positive regulation of stress fiber assembly	23	2
GO:0060420//regulation of heart growth	23	2
GO:0045321//leukocyte activation	322	16
GO:0030799//regulation of cyclic nucleotide metabolic process	124	7
GO:0010827//regulation of glucose transport	82	5
GO:0051052//regulation of DNA metabolic process	189	10
GO:0040008//regulation of growth	436	21
GO:0002028//regulation of sodium ion transport	42	3
GO:0008514//organic anion transmembrane transporter activity	42	3
GO:0048483//autonomic nervous system development	42	3
GO:0015276//ligand-gated ion channel activity	83	5
GO:0022834//ligand-gated channel activity	83	5
GO:0050808//synapse organization	83	5
GO:0006355//regulation of transcription, DNA-dependent	2732	118
GO:0035821//modification of morphology or physiology of other organism	280	14
GO:0000712//resolution of meiotic recombination intermediates	7	1
GO:0001574//ganglioside biosynthetic process	7	1
GO:0001779//natural killer cell differentiation	7	1
GO:0001955//blood vessel maturation	7	1
GO:0002279//mast cell activation involved in immune response	7	1
GO:0002367//cytokine production involved in immune response	7	1
GO:0002507//tolerance induction	7	1
GO:0002634//regulation of germinal center formation	7	1

GO:0003159//morphogenesis of an endothelium	7	1
GO:0005218//intracellular ligand-gated calcium channel activity	7	1
GO:0006002//fructose 6-phosphate metabolic process	7	1
GO:0006068//ethanol catabolic process	7	1
GO:0006171//cAMP biosynthetic process	7	1
GO:0006208//pyrimidine nucleobase catabolic process	7	1
GO:0006265//DNA topological change	7	1
GO:0006701//progesterone biosynthetic process	7	1
GO:0007183//SMAD protein complex assembly	7	1
GO:0007494//midgut development	7	1
GO:0008228//opsonization	7	1
GO:0008343//adult feeding behavior	7	1
GO:0009125//nucleoside monophosphate catabolic process	7	1
GO:0009265//2-deoxyribonucleotide biosynthetic process	7	1
GO:0009415//response to water stimulus	7	1
GO:0010172//embryonic body morphogenesis	7	1
GO:0010528//regulation of transposition	7	1
GO:0010529//negative regulation of transposition	7	1
GO:0010642//negative regulation of platelet-derived growth factor receptor	7	1
GO:0010700//negative regulation of norepinephrine secretion	7	1
GO:0010759//positive regulation of macrophage chemotaxis	7	1
GO:0014060//regulation of epinephrine secretion	7	1
GO:0014741//negative regulation of muscle hypertrophy	7	1
GO:0015271//outward rectifier potassium channel activity	7	1
GO:0016322//neuron remodeling	7	1
GO:0021801//cerebral cortex radial glia guided migration	7	1
GO:0030210//heparin biosynthetic process	7	1
GO:0030917//midbrain-hindbrain boundary development	7	1
GO:0033483//gas homeostasis	7	1
GO:0034135//regulation of toll-like receptor 2 signaling pathway	7	1
GO:0034370//triglyceride-rich lipoprotein particle remodeling	7	1
GO:0035458//cellular response to interferon-beta	7	1
GO:0035855//megakaryocyte development	7	1
GO:0042364//water-soluble vitamin biosynthetic process	7	1
GO:0042415//norepinephrine metabolic process	7	1
GO:0043303//mast cell degranulation	7	1
GO:0043353//enucleate erythrocyte differentiation	7	1
GO:0043371//negative regulation of CD4-positive, alpha-beta T cell differen	7	1
GO:0043517//positive regulation of DNA damage response, signal transduc	7	1
GO:0044030//regulation of DNA methylation	7	1
GO:0044068//modulation by symbiont of host cellular process	7	1
GO:0045623//negative regulation of T-helper cell differentiation	7	1
GO:0045719//negative regulation of glycogen biosynthetic process	7	1
GO:0046385//deoxyribose phosphate biosynthetic process	7	1
GO:0046500//S-adenosylmethionine metabolic process	7	1
GO:0048537//mucosal-associated lymphoid tissue development	7	1
GO:0048541//Peyers patch development	7	1
GO:0050667//homocysteine metabolic process	7	1
GO:0051024//positive regulation of immunoglobulin secretion	7	1
GO:0051938//L-glutamate import	7	1
GO:0060147//regulation of posttranscriptional gene silencing	7	1
GO:0060253//negative regulation of glial cell proliferation	7	1
GO:0060363//cranial suture morphogenesis	7	1
GO:0060561//apoptotic process involved in morphogenesis	7	1
GO:0060770//negative regulation of epithelial cell proliferation involved in pı	7	1
GO:0060911//cardiac cell fate commitment	7	1
GO:0060964//regulation of gene silencing by miRNA	7	1
GO:0060966//regulation of gene silencing by RNA	7	1

GO:0060973//cell migration involved in heart development	7	1
GO:0061154//endothelial tube morphogenesis	7	1
GO:0061314//Notch signaling involved in heart development	7	1
GO:0070231//T cell apoptotic process	7	1
GO:0070884//regulation of calcineurin-NFAT signaling cascade	7	1
GO:0071295//cellular response to vitamin	7	1
GO:0071875//adrenergic receptor signaling pathway	7	1
GO:0072070//loop of Henle development	7	1
GO:0090201//negative regulation of release of cytochrome c from mitochon	7	1
GO:1900017//positive regulation of cytokine production involved in inflamma	7	1
GO:1901722//regulation of cell proliferation involved in kidney development	7	1
GO:1901798//positive regulation of signal transduction by p53 class mediat	7	1
GO:2000482//regulation of interleukin-8 secretion	7	1
GO:2000810//regulation of tight junction assembly	7	1
GO:2001212//regulation of vasculogenesis	7	1
GO:NR_0268	7	1
GO:0014020//primary neural tube formation	63	4
GO:0071248//cellular response to metal ion	63	4
GO:0001709//cell fate determination	43	3
GO:0006835//dicarboxylic acid transport	43	3
GO:0008589//regulation of smoothened signaling pathway	43	3
GO:0019233//sensory perception of pain	43	3
GO:0043525//positive regulation of neuron apoptotic process	43	3
GO:0045446//endothelial cell differentiation	43	3
GO:0046823//negative regulation of nucleocytoplasmic transport	43	3
GO:0060675//ureteric bud morphogenesis	43	3
GO:0002244//hematopoietic progenitor cell differentiation	24	2
GO:0007266//Rho protein signal transduction	24	2
GO:0010543//regulation of platelet activation	24	2
GO:0010596//negative regulation of endothelial cell migration	24	2
GO:0033275//actin-myosin filament sliding	24	2
GO:0035116//embryonic hindlimb morphogenesis	24	2
GO:0046676//negative regulation of insulin secretion	24	2
GO:0051043//regulation of membrane protein ectodomain proteolysis	24	2
GO:0051180//vitamin transport	24	2
GO:0051953//negative regulation of amine transport	24	2
GO:0055090//acylglycerol homeostasis	24	2
GO:0070328//triglyceride homeostasis	24	2
GO:0050670//regulation of lymphocyte proliferation	148	8
GO:0001819//positive regulation of cytokine production	214	11
GO:0015267//channel activity	304	15
GO:0022803//passive transmembrane transporter activity	304	15
GO:0032944//regulation of mononuclear cell proliferation	149	8
GO:0032318//regulation of Ras GTPase activity	193	10
GO:0030802//regulation of cyclic nucleotide biosynthetic process	106	6
GO:0030810//positive regulation of nucleotide biosynthetic process	64	4
GO:0042990//regulation of transcription factor import into nucleus	64	4
GO:0051494//negative regulation of cytoskeleton organization	64	4
GO:1900373//positive regulation of purine nucleotide biosynthetic process	64	4
GO:0009855//determination of bilateral symmetry	85	5
GO:0032434//regulation of proteasomal ubiquitin-dependent protein catabo	44	3
GO:0097285//cell-type specific apoptotic process	44	3
GO:1901216//positive regulation of neuron death	44	3
GO:0002699//positive regulation of immune effector process	128	7
GO:2001141//regulation of RNA biosynthetic process	2744	118
GO:0016192//vesicle-mediated transport	768	35
GO:0007268//synaptic transmission	444	21
GO:0031331//positive regulation of cellular catabolic process	107	6

GO:0000578//embryonic axis specification	25	2
GO:0033344//cholesterol efflux	25	2
GO:0035019//somatic stem cell maintenance	25	2
GO:0035065//regulation of histone acetylation	25	2
GO:0045055//regulated secretory pathway	25	2
GO:0046326//positive regulation of glucose import	25	2
GO:0046471//phosphatidylglycerol metabolic process	25	2
GO:0072348//sulfur compound transport	25	2
GO:0090199//regulation of release of cytochrome c from mitochondria	25	2
GO:1901019//regulation of calcium ion transmembrane transporter activity	25	2
GO:2001234//negative regulation of apoptotic signaling pathway	25	2
GO:0033135//regulation of peptidyl-serine phosphorylation	65	4
GO:0090100//positive regulation of transmembrane receptor protein serine/	65	4
GO:0009799//specification of symmetry	86	5
GO:0048638//regulation of developmental growth	86	5
GO:0006650//glycerophospholipid metabolic process	218	11
GO:0045740//positive regulation of DNA replication	45	3
GO:0050803//regulation of synapse structure and activity	45	3
GO:0021953//central nervous system neuron differentiation	108	6
GO:0030879//mammary gland development	108	6
GO:0009118//regulation of nucleoside metabolic process	286	14
GO:0046486//glycerolipid metabolic process	286	14
GO:0008654//phospholipid biosynthetic process	196	10
GO:0071214//cellular response to abiotic stimulus	130	7
GO:0022890//inorganic cation transmembrane transporter activity	355	17
GO:0050863//regulation of T cell activation	264	13
GO:0002573//myeloid leukocyte differentiation	66	4
GO:0032846//positive regulation of homeostatic process	66	4
GO:0000729//DNA double-strand break processing	8	1
GO:0002448//mast cell mediated immunity	8	1
GO:0002883//regulation of hypersensitivity	8	1
GO:0002921//negative regulation of humoral immune response	8	1
GO:0003148//outflow tract septum morphogenesis	8	1
GO:0003339//regulation of mesenchymal to epithelial transition involved in	8	1
GO:0005391//sodium:potassium-exchanging ATPase activity	8	1
GO:0006216//cytidine catabolic process	8	1
GO:0006558//L-phenylalanine metabolic process	8	1
GO:0006559//L-phenylalanine catabolic process	8	1
GO:0006563//L-serine metabolic process	8	1
GO:0006707//cholesterol catabolic process	8	1
GO:0006817//phosphate ion transport	8	1
GO:0006853//carnitine shuttle	8	1
GO:0008300//isoprenoid catabolic process	8	1
GO:0009134//nucleoside diphosphate catabolic process	8	1
GO:0009135//purine nucleoside diphosphate metabolic process	8	1
GO:0009179//purine ribonucleoside diphosphate metabolic process	8	1
GO:0009972//cytidine deamination	8	1
GO:0010454//negative regulation of cell fate commitment	8	1
GO:0010661//positive regulation of muscle cell apoptotic process	8	1
GO:0010819//regulation of T cell chemotaxis	8	1
GO:0010820//positive regulation of T cell chemotaxis	8	1
GO:0014850//response to muscle activity	8	1
GO:0015810//aspartate transport	8	1
GO:0015884//folic acid transport	8	1
GO:0016127//sterol catabolic process	8	1
GO:0016540//protein autoprocessing	8	1
GO:0021873//forebrain neuroblast division	8	1
GO:0021903//rostrocaudal neural tube patterning	8	1

GO:0030202//heparin metabolic process	8	1
GO:0030916//otic vesicle formation	8	1
GO:0031935//regulation of chromatin silencing	8	1
GO:0031953//negative regulation of protein autophosphorylation	8	1
GO:0032048//cardiolipin metabolic process	8	1
GO:0032232//negative regulation of actin filament bundle assembly	8	1
GO:0032328//alanine transport	8	1
GO:0032703//negative regulation of interleukin-2 production	8	1
GO:0033089//positive regulation of T cell differentiation in thymus	8	1
GO:0033197//response to vitamin E	8	1
GO:0033623//regulation of integrin activation	8	1
GO:0033634//positive regulation of cell-cell adhesion mediated by integrin	8	1
GO:0033962//cytoplasmic mRNA processing body assembly	8	1
GO:0034331//cell junction maintenance	8	1
GO:0034379//very-low-density lipoprotein particle assembly	8	1
GO:0042135//neurotransmitter catabolic process	8	1
GO:0042362//fat-soluble vitamin biosynthetic process	8	1
GO:0042416//dopamine biosynthetic process	8	1
GO:0043092//L-amino acid import	8	1
GO:0045217//cell-cell junction maintenance	8	1
GO:0045540//regulation of cholesterol biosynthetic process	8	1
GO:0045605//negative regulation of epidermal cell differentiation	8	1
GO:0045625//regulation of T-helper 1 cell differentiation	8	1
GO:0045651//positive regulation of macrophage differentiation	8	1
GO:0045779//negative regulation of bone resorption	8	1
GO:0045989//positive regulation of striated muscle contraction	8	1
GO:0046085//adenosine metabolic process	8	1
GO:0046087//cytidine metabolic process	8	1
GO:0046548//retinal rod cell development	8	1
GO:0046599//regulation of centriole replication	8	1
GO:0048087//positive regulation of developmental pigmentation	8	1
GO:0048340//paraxial mesoderm morphogenesis	8	1
GO:0048532//anatomical structure arrangement	8	1
GO:0048569//post-embryonic organ development	8	1
GO:0048714//positive regulation of oligodendrocyte differentiation	8	1
GO:0050665//hydrogen peroxide biosynthetic process	8	1
GO:0050932//regulation of pigment cell differentiation	8	1
GO:0051151//negative regulation of smooth muscle cell differentiation	8	1
GO:0051156//glucose 6-phosphate metabolic process	8	1
GO:0051307//meiotic chromosome separation	8	1
GO:0051782//negative regulation of cell division	8	1
GO:0051895//negative regulation of focal adhesion assembly	8	1
GO:0052173//response to defenses of other organism involved in symbiotic	8	1
GO:0052200//response to host defenses	8	1
GO:0055091//phospholipid homeostasis	8	1
GO:0060055//angiogenesis involved in wound healing	8	1
GO:0060439//trachea morphogenesis	8	1
GO:0060575//intestinal epithelial cell differentiation	8	1
GO:0060592//mammary gland formation	8	1
GO:0060900//embryonic camera-type eye formation	8	1
GO:0061028//establishment of endothelial barrier	8	1
GO:0070233//negative regulation of T cell apoptotic process	8	1
GO:0070472//regulation of uterine smooth muscle contraction	8	1
GO:0070986//left/right axis specification	8	1
GO:0071236//cellular response to antibiotic	8	1
GO:0071480//cellular response to gamma radiation	8	1
GO:0071498//cellular response to fluid shear stress	8	1
GO:0071772//response to BMP stimulus	8	1

GO:0071773//cellular response to BMP stimulus	8	1
GO:0072015//glomerular visceral epithelial cell development	8	1
GO:0075136//response to host	8	1
GO:0090330//regulation of platelet aggregation	8	1
GO:0097186//amelogenesis	8	1
GO:1900024//regulation of substrate adhesion-dependent cell spreading	8	1
GO:1901379//regulation of potassium ion transmembrane transport	8	1
GO:1901889//negative regulation of cell junction assembly	8	1
GO:1902221//erythrose 4-phosphate/phosphoenolpyruvate family amino ac	8	1
GO:1902222//erythrose 4-phosphate/phosphoenolpyruvate family amino ac	8	1
GO:2000114//regulation of establishment of cell polarity	8	1
GO:2000310//regulation of N-methyl-D-aspartate selective glutamate recep	8	1
GO:2000651//positive regulation of sodium ion transmembrane transporter	8	1
GO:NM_0068	8	1
GO:0044242//cellular lipid catabolic process	109	6
GO:0007187//G-protein coupled receptor signaling pathway, coupled to cyc	131	7
GO:0001885//endothelial cell development	26	2
GO:0006775//fat-soluble vitamin metabolic process	26	2
GO:0009218//pyrimidine ribonucleotide metabolic process	26	2
GO:0022029//telencephalon cell migration	26	2
GO:0030901//midbrain development	26	2
GO:0042346//positive regulation of NF-kappaB import into nucleus	26	2
GO:0045923//positive regulation of fatty acid metabolic process	26	2
GO:0046626//regulation of insulin receptor signaling pathway	26	2
GO:0050805//negative regulation of synaptic transmission	26	2
GO:0052646//alditol phosphate metabolic process	26	2
GO:0070873//regulation of glycogen metabolic process	26	2
GO:2000756//regulation of peptidyl-lysine acetylation	26	2
GO:0048610//cellular process involved in reproduction	334	16
GO:0051249//regulation of lymphocyte activation	334	16
GO:0042446//hormone biosynthetic process	46	3
GO:0060415//muscle tissue morphogenesis	46	3
GO:1901879//regulation of protein depolymerization	46	3
GO:2000112//regulation of cellular macromolecule biosynthetic process	2977	127
GO:0032412//regulation of ion transmembrane transporter activity	67	4
GO:0034101//erythrocyte homeostasis	67	4
GO:0070588//calcium ion transmembrane transport	67	4
GO:1902106//negative regulation of leukocyte differentiation	67	4
GO:0044262//cellular carbohydrate metabolic process	110	6
GO:0043412//macromolecule modification	2017	87
GO:0015291//secondary active transmembrane transporter activity	89	5
GO:0005261//cation channel activity	245	12
GO:0097190//apoptotic signaling pathway	200	10
GO:0006213//pyrimidine nucleoside metabolic process	47	3
GO:0006987//activation of signaling protein activity involved in unfolded pro	47	3
GO:0009123//nucleoside monophosphate metabolic process	47	3
GO:0030216//keratinocyte differentiation	47	3
GO:0043154//negative regulation of cysteine-type endopeptidase activity in	47	3
GO:0043242//negative regulation of protein complex disassembly	47	3
GO:0050905//neuromuscular process	68	4
GO:0051251//positive regulation of lymphocyte activation	246	12
GO:0002067//glandular epithelial cell differentiation	27	2
GO:0002444//myeloid leukocyte mediated immunity	27	2
GO:0005251//delayed rectifier potassium channel activity	27	2
GO:0009132//nucleoside diphosphate metabolic process	27	2
GO:0010977//negative regulation of neuron projection development	27	2
GO:0021575//hindbrain morphogenesis	27	2
GO:0022408//negative regulation of cell-cell adhesion	27	2

GO:0032233//positive regulation of actin filament bundle assembly	27	2
GO:0032885//regulation of polysaccharide biosynthetic process	27	2
GO:0043266//regulation of potassium ion transport	27	2
GO:0043370//regulation of CD4-positive, alpha-beta T cell differentiation	27	2
GO:0045931//positive regulation of mitotic cell cycle	27	2
GO:0050732//negative regulation of peptidyl-tyrosine phosphorylation	27	2
GO:0042742//defense response to bacterium	156	8
GO:0071702//organic substance transport	1376	60
GO:0006367//transcription initiation from RNA polymerase II promoter	112	6
GO:0010923//negative regulation of phosphatase activity	69	4
GO:0007611//learning or memory	135	7
GO:0002443//leukocyte mediated immunity	180	9
GO:0006518//peptide metabolic process	91	5
GO:0000160//phosphorelay signal transduction system	9	1
GO:0001504//neurotransmitter uptake	9	1
GO:0001736//establishment of planar polarity	9	1
GO:0001840//neural plate development	9	1
GO:0002070//epithelial cell maturation	9	1
GO:0002320//lymphoid progenitor cell differentiation	9	1
GO:0002864//regulation of acute inflammatory response to antigenic stimul	9	1
GO:0003416//endochondral bone growth	9	1
GO:0005388//calcium-transporting ATPase activity	9	1
GO:0005513//detection of calcium ion	9	1
GO:0006266//DNA ligation	9	1
GO:0006516//glycoprotein catabolic process	9	1
GO:0006570//tyrosine metabolic process	9	1
GO:0007164//establishment of tissue polarity	9	1
GO:0007638//mechanosensory behavior	9	1
GO:0009649//entrainment of circadian clock	9	1
GO:0010640//regulation of platelet-derived growth factor receptor signaling	9	1
GO:0010758//regulation of macrophage chemotaxis	9	1
GO:0010870//positive regulation of receptor biosynthetic process	9	1
GO:0015280//ligand-gated sodium channel activity	9	1
GO:0015740//C4-dicarboxylate transport	9	1
GO:0015824//proline transport	9	1
GO:0019079//viral genome replication	9	1
GO:0019755//one-carbon compound transport	9	1
GO:0021940//positive regulation of cerebellar granule cell precursor prolifer	9	1
GO:0030220//platelet formation	9	1
GO:0032494//response to peptidoglycan	9	1
GO:0032691//negative regulation of interleukin-1 beta production	9	1
GO:0034764//positive regulation of transmembrane transport	9	1
GO:0034767//positive regulation of ion transmembrane transport	9	1
GO:0036344//platelet morphogenesis	9	1
GO:0040015//negative regulation of multicellular organism growth	9	1
GO:0042100//B cell proliferation	9	1
GO:0042511//positive regulation of tyrosine phosphorylation of Stat1 proteir	9	1
GO:0042640//anagen	9	1
GO:0043217//myelin maintenance	9	1
GO:0043508//negative regulation of JUN kinase activity	9	1
GO:0045006//DNA deamination	9	1
GO:0045176//apical protein localization	9	1
GO:0046113//nucleobase catabolic process	9	1
GO:0046133//pyrimidine ribonucleoside catabolic process	9	1
GO:0046689//response to mercury ion	9	1
GO:0050884//neuromuscular process controlling posture	9	1
GO:0050951//sensory perception of temperature stimulus	9	1
GO:0055057//neuroblast division	9	1

GO:0060272//embryonic skeletal joint morphogenesis	9	1
GO:0070874//negative regulation of glycogen metabolic process	9	1
GO:0071350//cellular response to interleukin-15	9	1
GO:0072017//distal tubule development	9	1
GO:0072202//cell differentiation involved in metanephros development	9	1
GO:0072310//glomerular epithelial cell development	9	1
GO:0072678//T cell migration	9	1
GO:0086005//regulation of ventricular cardiac muscle cell action potential	9	1
GO:0090049//regulation of cell migration involved in sprouting angiogenesis	9	1
GO:0090231//regulation of spindle checkpoint	9	1
GO:0097067//cellular response to thyroid hormone stimulus	9	1
GO:0097094//craniofacial suture morphogenesis	9	1
GO:0097237//cellular response to toxic substance	9	1
GO:0097267//omega-hydroxylase P450 pathway	9	1
GO:1900078//positive regulation of cellular response to insulin stimulus	9	1
GO:1901020//negative regulation of calcium ion transmembrane transporter	9	1
GO:2000036//regulation of stem cell maintenance	9	1
GO:2000352//negative regulation of endothelial cell apoptotic process	9	1
GO:2000515//negative regulation of CD4-positive, alpha-beta T cell activati	9	1
GO:2000772//regulation of cellular senescence	9	1
GO:0005245//voltage-gated calcium channel activity	28	2
GO:0006739//NADP metabolic process	28	2
GO:0007528//neuromuscular junction development	28	2
GO:0007628//adult walking behavior	28	2
GO:0021517//ventral spinal cord development	28	2
GO:0021885//forebrain cell migration	28	2
GO:0030834//regulation of actin filament depolymerization	28	2
GO:0030857//negative regulation of epithelial cell differentiation	28	2
GO:0048333//mesodermal cell differentiation	28	2
GO:0050663//cytokine secretion	28	2
GO:1901983//regulation of protein acetylation	28	2
GO:0045981//positive regulation of nucleotide metabolic process	70	4
GO:1900544//positive regulation of purine nucleotide metabolic process	70	4
GO:0034504//protein localization to nucleus	114	6
GO:0006641//triglyceride metabolic process	92	5
GO:0051495//positive regulation of cytoskeleton organization	92	5
GO:0003158//endothelium development	49	3
GO:0032075//positive regulation of nuclease activity	49	3
GO:0015108//chloride transmembrane transporter activity	71	4
GO:0022898//regulation of transmembrane transporter activity	71	4
GO:0007015//actin filament organization	93	5
GO:0006575//cellular modified amino acid metabolic process	183	9
GO:0050867//positive regulation of cell activation	275	13
GO:0006352//DNA-dependent transcription, initiation	138	7
GO:0002762//negative regulation of myeloid leukocyte differentiation	29	2
GO:0003382//epithelial cell morphogenesis	29	2
GO:0006206//pyrimidine nucleobase metabolic process	29	2
GO:0006656//phosphatidylcholine biosynthetic process	29	2
GO:0006695//cholesterol biosynthetic process	29	2
GO:0007044//cell-substrate junction assembly	29	2
GO:0007052//mitotic spindle organization	29	2
GO:0022406//membrane docking	29	2
GO:0034644//cellular response to UV	29	2
GO:0046638//positive regulation of alpha-beta T cell differentiation	29	2
GO:0051209//release of sequestered calcium ion into cytosol	29	2
GO:0051283//negative regulation of sequestering of calcium ion	29	2
GO:0055013//cardiac muscle cell development	29	2
GO:1901264//carbohydrate derivative transport	29	2

GO:1901989//positive regulation of cell cycle phase transition	29	2
GO:1901992//positive regulation of mitotic cell cycle phase transition	29	2
GO:0010970//microtubule-based transport	50	3
GO:0090317//negative regulation of intracellular protein transport	50	3
GO:0097479//synaptic vesicle localization	50	3
GO:2000117//negative regulation of cysteine-type endopeptidase activity	50	3
GO:0042451//purine nucleoside biosynthetic process	72	4
GO:0046129//purine ribonucleoside biosynthetic process	72	4
GO:0050768//negative regulation of neurogenesis	72	4
GO:0000738//DNA catabolic process, exonucleolytic	10	1
GO:0002251//organ or tissue specific immune response	10	1
GO:0003214//cardiac left ventricle morphogenesis	10	1
GO:0005451//monovalent cation:hydrogen antiporter activity	10	1
GO:0006000//fructose metabolic process	10	1
GO:0006465//signal peptide processing	10	1
GO:0006596//polyamine biosynthetic process	10	1
GO:0007063//regulation of sister chromatid cohesion	10	1
GO:0008631//intrinsic apoptotic signaling pathway in response to oxidative	10	1
GO:0009151//purine deoxyribonucleotide metabolic process	10	1
GO:0009185//ribonucleoside diphosphate metabolic process	10	1
GO:0009263//deoxyribonucleotide biosynthetic process	10	1
GO:0010693//negative regulation of alkaline phosphatase activity	10	1
GO:0010881//regulation of cardiac muscle contraction by regulation of the r	10	1
GO:0010935//regulation of macrophage cytokine production	10	1
GO:0015278//calcium-release channel activity	10	1
GO:0015802//basic amino acid transport	10	1
GO:0019067//viral assembly, maturation, egress, and release	10	1
GO:0021548//pons development	10	1
GO:0021781//glial cell fate commitment	10	1
GO:0021799//cerebral cortex radially oriented cell migration	10	1
GO:0021936//regulation of cerebellar granule cell precursor proliferation	10	1
GO:0030207//chondroitin sulfate catabolic process	10	1
GO:0032096//negative regulation of response to food	10	1
GO:0032099//negative regulation of appetite	10	1
GO:0032692//negative regulation of interleukin-1 production	10	1
GO:0033233//regulation of protein sumoylation	10	1
GO:0033327//Leydig cell differentiation	10	1
GO:0033604//negative regulation of catecholamine secretion	10	1
GO:0034374//low-density lipoprotein particle remodeling	10	1
GO:0034443//negative regulation of lipoprotein oxidation	10	1
GO:0035024//negative regulation of Rho protein signal transduction	10	1
GO:0035414//negative regulation of catenin import into nucleus	10	1
GO:0042347//negative regulation of NF-kappaB import into nucleus	10	1
GO:0043268//positive regulation of potassium ion transport	10	1
GO:0045683//negative regulation of epidermis development	10	1
GO:0046639//negative regulation of alpha-beta T cell differentiation	10	1
GO:0048268//clathrin coat assembly	10	1
GO:0048703//embryonic viscerocranium morphogenesis	10	1
GO:0051642//centrosome localization	10	1
GO:0051770//positive regulation of nitric-oxide synthase biosynthetic proce:	10	1
GO:0051918//negative regulation of fibrinolysis	10	1
GO:0055062//phosphate ion homeostasis	10	1
GO:0060117//auditory receptor cell development	10	1
GO:0060292//long term synaptic depression	10	1
GO:0060712//spongiotrophoblast layer development	10	1
GO:0060732//positive regulation of inositol phosphate biosynthetic process	10	1
GO:0060788//ectodermal placode formation	10	1
GO:0070227//lymphocyte apoptotic process	10	1

GO:0070307//lens fiber cell development	10	1
GO:0071600//otic vesicle morphogenesis	10	1
GO:0071696//ectodermal placode development	10	1
GO:0071697//ectodermal placode morphogenesis	10	1
GO:0072148//epithelial cell fate commitment	10	1
GO:0072506//trivalent inorganic anion homeostasis	10	1
GO:0090192//regulation of glomerulus development	10	1
GO:0090399//replicative senescence	10	1
GO:1901881//positive regulation of protein depolymerization	10	1
GO:1902001//fatty acid transmembrane transport	10	1
GO:2000047//regulation of cell-cell adhesion mediated by cadherin	10	1
GO:2000501//regulation of natural killer cell chemotaxis	10	1
GO:0045666//positive regulation of neuron differentiation	51	3
GO:0051250//negative regulation of lymphocyte activation	95	5
GO:0001841//neural tube formation	73	4
GO:0055072//iron ion homeostasis	73	4
GO:0006383//transcription from RNA polymerase III promoter	30	2
GO:0030317//sperm motility	30	2
GO:0032881//regulation of polysaccharide metabolic process	30	2
GO:0043392//negative regulation of DNA binding	30	2
GO:0045620//negative regulation of lymphocyte differentiation	30	2
GO:0050691//regulation of defense response to virus by host	30	2
GO:0051282//regulation of sequestering of calcium ion	30	2
GO:0051970//negative regulation of transmission of nerve impulse	30	2
GO:0060603//mammary gland duct morphogenesis	30	2
GO:0061077//chaperone-mediated protein folding	30	2
GO:0070252//actin-mediated cell contraction	30	2
GO:2000379//positive regulation of reactive oxygen species metabolic proc	30	2
GO:2000514//regulation of CD4-positive, alpha-beta T cell activation	30	2
GO:0022843//voltage-gated cation channel activity	118	6
GO:1901575//organic substance catabolic process	1470	63
GO:0005216//ion channel activity	303	14
GO:0022838//substrate-specific channel activity	303	14
GO:0005249//voltage-gated potassium channel activity	74	4
GO:0045471//response to ethanol	74	4
GO:0003143//embryonic heart tube morphogenesis	52	3
GO:0006027//glycosaminoglycan catabolic process	52	3
GO:0030010//establishment of cell polarity	52	3
GO:0030705//cytoskeleton-dependent intracellular transport	52	3
GO:0051291//protein heterooligomerization	52	3
GO:0044764//multi-organism cellular process	398	18
GO:0051252//regulation of RNA metabolic process	2804	118
GO:0051129//negative regulation of cellular component organization	281	13
GO:0030001//metal ion transport	446	20
GO:0003001//generation of a signal involved in cell-cell signaling	97	5
GO:0023061//signal release	97	5
GO:0030072//peptide hormone secretion	31	2
GO:0031663//lipopolysaccharide-mediated signaling pathway	31	2
GO:0043624//cellular protein complex disassembly	31	2
GO:0050931//pigment cell differentiation	31	2
GO:0051055//negative regulation of lipid biosynthetic process	31	2
GO:0055010//ventricular cardiac muscle tissue morphogenesis	31	2
GO:0060042//retina morphogenesis in camera-type eye	31	2
GO:0090066//regulation of anatomical structure size	212	10
GO:0051056//regulation of small GTPase mediated signal transduction	376	17
GO:1901607//alpha-amino acid biosynthetic process	75	4
GO:0061136//regulation of proteasomal protein catabolic process	53	3
GO:0000098//sulfur amino acid catabolic process	11	1

GO:0001833//inner cell mass cell proliferation	11	1
GO:0001946//lymphangiogenesis	11	1
GO:0002031//G-protein coupled receptor internalization	11	1
GO:0002532//production of molecular mediator involved in inflammatory res	11	1
GO:0003323//type B pancreatic cell development	11	1
GO:0006590//thyroid hormone generation	11	1
GO:0006895//Golgi to endosome transport	11	1
GO:0007213//G-protein coupled acetylcholine receptor signaling pathway	11	1
GO:0007350//blastoderm segmentation	11	1
GO:0007351//tripartite regional subdivision	11	1
GO:0008556//potassium-transporting ATPase activity	11	1
GO:0008595//anterior/posterior axis specification, embryo	11	1
GO:0010224//response to UV-B	11	1
GO:0010882//regulation of cardiac muscle contraction by calcium ion signal	11	1
GO:0010889//regulation of sequestering of triglyceride	11	1
GO:0014047//glutamate secretion	11	1
GO:0015114//phosphate ion transmembrane transporter activity	11	1
GO:0017187//peptidyl-glutamic acid carboxylation	11	1
GO:0018214//protein carboxylation	11	1
GO:0021520//spinal cord motor neuron cell fate specification	11	1
GO:0021756//striatum development	11	1
GO:0021884//forebrain neuron development	11	1
GO:0022010//central nervous system myelination	11	1
GO:0030214//hyaluronan catabolic process	11	1
GO:0031638//zymogen activation	11	1
GO:0032291//axon ensheathment in central nervous system	11	1
GO:0032922//circadian regulation of gene expression	11	1
GO:0033598//mammary gland epithelial cell proliferation	11	1
GO:0034310//primary alcohol catabolic process	11	1
GO:0035307//positive regulation of protein dephosphorylation	11	1
GO:0036092//phosphatidylinositol-3-phosphate biosynthetic process	11	1
GO:0036303//lymph vessel morphogenesis	11	1
GO:0042340//keratan sulfate catabolic process	11	1
GO:0042832//defense response to protozoan	11	1
GO:0042953//lipoprotein transport	11	1
GO:0043090//amino acid import	11	1
GO:0043568//positive regulation of insulin-like growth factor receptor signal	11	1
GO:0045815//positive regulation of gene expression, epigenetic	11	1
GO:0045843//negative regulation of striated muscle tissue development	11	1
GO:0046321//positive regulation of fatty acid oxidation	11	1
GO:0048934//peripheral nervous system neuron differentiation	11	1
GO:0048935//peripheral nervous system neuron development	11	1
GO:0051304//chromosome separation	11	1
GO:0051972//regulation of telomerase activity	11	1
GO:0060251//regulation of glial cell proliferation	11	1
GO:0060347//heart trabecula formation	11	1
GO:0060413//atrial septum morphogenesis	11	1
GO:0070293//renal absorption	11	1
GO:0071636//positive regulation of transforming growth factor beta producti	11	1
GO:0072164//mesonephric tubule development	11	1
GO:0090075//relaxation of muscle	11	1
GO:0097066//response to thyroid hormone stimulus	11	1
GO:1901862//negative regulation of muscle tissue development	11	1
GO:1901976//regulation of cell cycle checkpoint	11	1
GO:0033121//regulation of purine nucleotide catabolic process	283	13
GO:0046578//regulation of Ras protein signal transduction	307	14
GO:0030811//regulation of nucleotide catabolic process	284	13
GO:0016573//histone acetylation	76	4

GO:0043087//regulation of GTPase activity	261	12
GO:0035023//regulation of Rho protein signal transduction	168	8
GO:0003229//ventricular cardiac muscle tissue development	32	2
GO:0007205//protein kinase C-activating G-protein coupled receptor signali	32	2
GO:0009948//anterior/posterior axis specification	32	2
GO:0035137//hindlimb morphogenesis	32	2
GO:0043241//protein complex disassembly	32	2
GO:0044070//regulation of anion transport	54	3
GO:0072524//pyridine-containing compound metabolic process	54	3
GO:1901616//organic hydroxy compound catabolic process	54	3
GO:0044238//primary metabolic process	6263	259
GO:0010639//negative regulation of organelle organization	146	7
GO:0043123//positive regulation of I-kappaB kinase/NF-kappaB cascade	146	7
GO:0019953//sexual reproduction	524	23
GO:0045637//regulation of myeloid cell differentiation	123	6
GO:0060021//palate development	55	3
GO:0070664//negative regulation of leukocyte proliferation	55	3
GO:0033124//regulation of GTP catabolic process	264	12
GO:1901136//carbohydrate derivative catabolic process	478	21
GO:0005272//sodium channel activity	33	2
GO:0046131//pyrimidine ribonucleoside metabolic process	33	2
GO:0060795//cell fate commitment involved in formation of primary germ la	33	2
GO:0050864//regulation of B cell activation	78	4
GO:0071383//cellular response to steroid hormone stimulus	78	4
GO:0002710//negative regulation of T cell mediated immunity	12	1
GO:0006677//glycosylceramide metabolic process	12	1
GO:0006862//nucleotide transport	12	1
GO:0006978//DNA damage response, signal transduction by p53 class mec	12	1
GO:0010560//positive regulation of glycoprotein biosynthetic process	12	1
GO:0010837//regulation of keratinocyte proliferation	12	1
GO:0010880//regulation of release of sequestered calcium ion into cytosol I	12	1
GO:0010919//regulation of inositol phosphate biosynthetic process	12	1
GO:0014061//regulation of norepinephrine secretion	12	1
GO:0015370//solute:sodium symporter activity	12	1
GO:0015671//oxygen transport	12	1
GO:0015838//amino-acid betaine transport	12	1
GO:0015858//nucleoside transport	12	1
GO:0015879//carnitine transport	12	1
GO:0019373//epoxygenase P450 pathway	12	1
GO:0032225//regulation of synaptic transmission, dopaminergic	12	1
GO:0034695//response to prostaglandin E stimulus	12	1
GO:0035306//positive regulation of dephosphorylation	12	1
GO:0035815//positive regulation of renal sodium excretion	12	1
GO:0036342//post-anal tail morphogenesis	12	1
GO:0042219//cellular modified amino acid catabolic process	12	1
GO:0042510//regulation of tyrosine phosphorylation of Stat1 protein	12	1
GO:0042659//regulation of cell fate specification	12	1
GO:0043586//tongue development	12	1
GO:0043650//dicarboxylic acid biosynthetic process	12	1
GO:0045662//negative regulation of myoblast differentiation	12	1
GO:0045725//positive regulation of glycogen biosynthetic process	12	1
GO:0045955//negative regulation of calcium ion-dependent exocytosis	12	1
GO:0046339//diacylglycerol metabolic process	12	1
GO:0048246//macrophage chemotaxis	12	1
GO:0050435//beta-amyloid metabolic process	12	1
GO:0050651//dermatan sulfate proteoglycan biosynthetic process	12	1
GO:0050655//dermatan sulfate proteoglycan metabolic process	12	1
GO:0050930//induction of positive chemotaxis	12	1

GO:0051187//cofactor catabolic process	12	1
GO:0051261//protein depolymerization	12	1
GO:0060039//pericardium development	12	1
GO:0060065//uterus development	12	1
GO:0060122//inner ear receptor stereocilium organization	12	1
GO:0060766//negative regulation of androgen receptor signaling pathway	12	1
GO:0061046//regulation of branching involved in lung morphogenesis	12	1
GO:0061318//renal filtration cell differentiation	12	1
GO:0070672//response to interleukin-15	12	1
GO:0071385//cellular response to glucocorticoid stimulus	12	1
GO:0072112//glomerular visceral epithelial cell differentiation	12	1
GO:0072163//mesonephric epithelium development	12	1
GO:0072498//embryonic skeletal joint development	12	1
GO:0086091//regulation of heart rate by cardiac conduction	12	1
GO:0090303//positive regulation of wound healing	12	1
GO:NM_0024	12	1
GO:0002696//positive regulation of leukocyte activation	265	12
GO:0071705//nitrogen compound transport	337	15
GO:0007626//locomotory behavior	125	6
GO:0030819//positive regulation of cAMP biosynthetic process	56	3
GO:0032069//regulation of nuclease activity	56	3
GO:0033238//regulation of cellular amine metabolic process	56	3
GO:0045638//negative regulation of myeloid cell differentiation	56	3
GO:0032675//regulation of interleukin-6 production	79	4
GO:0050890//cognition	149	7
GO:0006644//phospholipid metabolic process	267	12
GO:0032006//regulation of TOR signaling cascade	34	2
GO:0048678//response to axon injury	34	2
GO:0050772//positive regulation of axonogenesis	34	2
GO:1900076//regulation of cellular response to insulin stimulus	34	2
GO:0016042//lipid catabolic process	197	9
GO:0009064//glutamine family amino acid metabolic process	57	3
GO:0050821//protein stabilization	57	3
GO:0071804//cellular potassium ion transport	57	3
GO:0071805//potassium ion transmembrane transport	57	3
GO:0017157//regulation of exocytosis	80	4
GO:0032409//regulation of transporter activity	80	4
GO:0046034//ATP metabolic process	174	8
GO:0010927//cellular component assembly involved in morphogenesis	127	6
GO:0000266//mitochondrial fission	13	1
GO:0002068//glandular epithelial cell development	13	1
GO:0003091//renal water homeostasis	13	1
GO:0003283//atrial septum development	13	1
GO:0003309//type B pancreatic cell differentiation	13	1
GO:0005217//intracellular ligand-gated ion channel activity	13	1
GO:0005980//glycogen catabolic process	13	1
GO:0006044//N-acetylglucosamine metabolic process	13	1
GO:0006278//RNA-dependent DNA replication	13	1
GO:0006927//transformed cell apoptotic process	13	1
GO:0007320//insemination	13	1
GO:0007342//fusion of sperm to egg plasma membrane	13	1
GO:0007413//axonal fasciculation	13	1
GO:0007512//adult heart development	13	1
GO:0010092//specification of organ identity	13	1
GO:0010954//positive regulation of protein processing	13	1
GO:0015101//organic cation transmembrane transporter activity	13	1
GO:0016254//preassembly of GPI anchor in ER membrane	13	1
GO:0019083//viral transcription	13	1

GO:0021984//adenohypophysis development	13	1
GO:0023019//signal transduction involved in regulation of gene expression	13	1
GO:0030224//monocyte differentiation	13	1
GO:0031620//regulation of fever generation	13	1
GO:0031622//positive regulation of fever generation	13	1
GO:0031665//negative regulation of lipopolysaccharide-mediated signaling	13	1
GO:0032413//negative regulation of ion transmembrane transporter activity	13	1
GO:0032438//melanosome organization	13	1
GO:0032469//endoplasmic reticulum calcium ion homeostasis	13	1
GO:0035456//response to interferon-beta	13	1
GO:0036149//phosphatidylinositol acyl-chain remodeling	13	1
GO:0042772//DNA damage response, signal transduction resulting in trans	13	1
GO:0043277//apoptotic cell clearance	13	1
GO:0043954//cellular component maintenance	13	1
GO:0045649//regulation of macrophage differentiation	13	1
GO:0045723//positive regulation of fatty acid biosynthetic process	13	1
GO:0045730//respiratory burst	13	1
GO:0046051//UTP metabolic process	13	1
GO:0048820//hair follicle maturation	13	1
GO:0051402//neuron apoptotic process	13	1
GO:0051923//sulfation	13	1
GO:0060045//positive regulation of cardiac muscle cell proliferation	13	1
GO:0070207//protein homotrimerization	13	1
GO:0070527//platelet aggregation	13	1
GO:0071285//cellular response to lithium ion	13	1
GO:0071384//cellular response to corticosteroid stimulus	13	1
GO:0072010//glomerular epithelium development	13	1
GO:0072234//metanephric nephron tubule development	13	1
GO:0072243//metanephric nephron epithelium development	13	1
GO:0072311//glomerular epithelial cell differentiation	13	1
GO:0090004//positive regulation of establishment of protein localization to	13	1
GO:0090208//positive regulation of triglyceride metabolic process	13	1
GO:0090322//regulation of superoxide metabolic process	13	1
GO:1901099//negative regulation of signal transduction in absence of ligan	13	1
GO:2001240//negative regulation of extrinsic apoptotic signaling pathway in	13	1
GO:0006473//protein acetylation	104	5
GO:0006144//purine nucleobase metabolic process	35	2
GO:0006691//leukotriene metabolic process	35	2
GO:0008593//regulation of Notch signaling pathway	35	2
GO:0031645//negative regulation of neurological system process	35	2
GO:0043967//histone H4 acetylation	35	2
GO:2001236//regulation of extrinsic apoptotic signaling pathway	35	2
GO:0007368//determination of left/right symmetry	81	4
GO:0050777//negative regulation of immune response	58	3
GO:0071478//cellular response to radiation	58	3
GO:0010564//regulation of cell cycle process	391	17
GO:0015672//monovalent inorganic cation transport	272	12
GO:0070507//regulation of microtubule cytoskeleton organization	82	4
GO:0002449//lymphocyte mediated immunity	153	7
GO:0050778//positive regulation of immune response	441	19
GO:0008344//adult locomotory behavior	59	3
GO:0009112//nucleobase metabolic process	59	3
GO:0030816//positive regulation of cAMP metabolic process	59	3
GO:0007041//lysosomal transport	36	2
GO:0021766//hippocampus development	36	2
GO:0032092//positive regulation of protein binding	36	2
GO:0033143//regulation of intracellular steroid hormone receptor signaling	36	2
GO:0060402//calcium ion transport into cytosol	36	2

GO:0097306//cellular response to alcohol	36	2
GO:2001022//positive regulation of response to DNA damage stimulus	36	2
GO:0030522//intracellular receptor signaling pathway	130	6
GO:0002460//adaptive immune response based on somatic recombination	154	7
GO:0002446//neutrophil mediated immunity	14	1
GO:0006067//ethanol metabolic process	14	1
GO:0006662//glycerol ether metabolic process	14	1
GO:0008272//sulfate transport	14	1
GO:0009070//serine family amino acid biosynthetic process	14	1
GO:0009251//glucan catabolic process	14	1
GO:0010692//regulation of alkaline phosphatase activity	14	1
GO:0010800//positive regulation of peptidyl-threonine phosphorylation	14	1
GO:0014912//negative regulation of smooth muscle cell migration	14	1
GO:0015175//neutral amino acid transmembrane transporter activity	14	1
GO:0015697//quaternary ammonium group transport	14	1
GO:0016572//histone phosphorylation	14	1
GO:0021544//subpallium development	14	1
GO:0030866//cortical actin cytoskeleton organization	14	1
GO:0031112//positive regulation of microtubule polymerization or depolyme	14	1
GO:0031652//positive regulation of heat generation	14	1
GO:0032095//regulation of response to food	14	1
GO:0032401//establishment of melanosome localization	14	1
GO:0032781//positive regulation of ATPase activity	14	1
GO:0035249//synaptic transmission, glutamatergic	14	1
GO:0035304//regulation of protein dephosphorylation	14	1
GO:0042089//cytokine biosynthetic process	14	1
GO:0042136//neurotransmitter biosynthetic process	14	1
GO:0042403//thyroid hormone metabolic process	14	1
GO:0044247//cellular polysaccharide catabolic process	14	1
GO:0045824//negative regulation of innate immune response	14	1
GO:0048753//pigment granule organization	14	1
GO:0051798//positive regulation of hair follicle development	14	1
GO:0051956//negative regulation of amino acid transport	14	1
GO:0060317//cardiac epithelial to mesenchymal transition	14	1
GO:0060972//left/right pattern formation	14	1
GO:0070076//histone lysine demethylation	14	1
GO:0070232//regulation of T cell apoptotic process	14	1
GO:0072170//metanephric tubule development	14	1
GO:0090026//positive regulation of monocyte chemotaxis	14	1
GO:0090181//regulation of cholesterol metabolic process	14	1
GO:2000178//negative regulation of neural precursor cell proliferation	14	1
GO:NM_0005	14	1
GO:0043244//regulation of protein complex disassembly	60	3
GO:0002377//immunoglobulin production	37	2
GO:0007193//adenylate cyclase-inhibiting G-protein coupled receptor signa	37	2
GO:0042733//embryonic digit morphogenesis	37	2
GO:0045071//negative regulation of viral genome replication	37	2
GO:0048066//developmental pigmentation	37	2
GO:0050885//neuromuscular process controlling balance	37	2
GO:0060401//cytosolic calcium ion transport	37	2
GO:0071482//cellular response to light stimulus	37	2
GO:0072522//purine-containing compound biosynthetic process	108	5
GO:0007631//feeding behavior	61	3
GO:0030218//erythrocyte differentiation	61	3
GO:0006486//protein glycosylation	229	10
GO:0043413//macromolecule glycosylation	229	10
GO:0016032//viral process	423	18
GO:0019637//organophosphate metabolic process	934	39

GO:0006816//calcium ion transport	157	7
GO:0008652//cellular amino acid biosynthetic process	109	5
GO:0007257//activation of JUN kinase activity	38	2
GO:0030521//androgen receptor signaling pathway	38	2
GO:0038032//termination of G-protein coupled receptor signaling pathway	38	2
GO:0001711//endodermal cell fate commitment	15	1
GO:0001832//blastocyst growth	15	1
GO:0001976//neurological system process involved in regulation of systemi	15	1
GO:0002029//desensitization of G-protein coupled receptor protein signalin	15	1
GO:0002052//positive regulation of neuroblast proliferation	15	1
GO:0002230//positive regulation of defense response to virus by host	15	1
GO:0002707//negative regulation of lymphocyte mediated immunity	15	1
GO:0006825//copper ion transport	15	1
GO:0007026//negative regulation of microtubule depolymerization	15	1
GO:0007045//cell-substrate adherens junction assembly	15	1
GO:0008105//asymmetric protein localization	15	1
GO:0009713//catechol-containing compound biosynthetic process	15	1
GO:0015813//L-glutamate transport	15	1
GO:0015949//nucleobase-containing small molecule interconversion	15	1
GO:0019321//pentose metabolic process	15	1
GO:0022401//negative adaptation of signaling pathway	15	1
GO:0030316//osteoclast differentiation	15	1
GO:0031103//axon regeneration	15	1
GO:0031113//regulation of microtubule polymerization	15	1
GO:0033146//regulation of intracellular estrogen receptor signaling pathwa	15	1
GO:0033574//response to testosterone stimulus	15	1
GO:0035066//positive regulation of histone acetylation	15	1
GO:0042119//neutrophil activation	15	1
GO:0042423//catecholamine biosynthetic process	15	1
GO:0045214//sarcomere organization	15	1
GO:0045648//positive regulation of erythrocyte differentiation	15	1
GO:0046329//negative regulation of JNK cascade	15	1
GO:0046939//nucleotide phosphorylation	15	1
GO:0048041//focal adhesion assembly	15	1
GO:0048251//elastic fiber assembly	15	1
GO:0048339//paraxial mesoderm development	15	1
GO:0048384//retinoic acid receptor signaling pathway	15	1
GO:0051647//nucleus localization	15	1
GO:0051905//establishment of pigment granule localization	15	1
GO:0051930//regulation of sensory perception of pain	15	1
GO:0051931//regulation of sensory perception	15	1
GO:0060004//reflex	15	1
GO:0070875//positive regulation of glycogen metabolic process	15	1
GO:0072207//metanephric epithelium development	15	1
GO:1901985//positive regulation of protein acetylation	15	1
GO:2000008//regulation of protein localization to cell surface	15	1
GO:2000249//regulation of actin cytoskeleton reorganization	15	1
GO:2000758//positive regulation of peptidyl-lysine acetylation	15	1
GO:0030804//positive regulation of cyclic nucleotide biosynthetic process	62	3
GO:0007163//establishment or maintenance of cell polarity	86	4
GO:0042455//ribonucleoside biosynthetic process	86	4
GO:0043393//regulation of protein binding	86	4
GO:0002521//leukocyte differentiation	208	9
GO:0006606//protein import into nucleus	87	4
GO:0009411//response to UV	87	4
GO:0015698//inorganic anion transport	87	4
GO:0044744//protein targeting to nucleus	87	4
GO:2001233//regulation of apoptotic signaling pathway	87	4

GO:0001755//neural crest cell migration	39	2
GO:0051017//actin filament bundle assembly	39	2
GO:0061572//actin filament bundle organization	39	2
GO:0070936//protein K48-linked ubiquitination	39	2
GO:0022037//metencephalon development	63	3
GO:0051100//negative regulation of binding	63	3
GO:0070085//glycosylation	233	10
GO:0003674//molecular_function	1016	42
GO:0022900//electron transport chain	112	5
GO:0007265//Ras protein signal transduction	88	4
GO:0000272//polysaccharide catabolic process	16	1
GO:0001836//release of cytochrome c from mitochondria	16	1
GO:0002483//antigen processing and presentation of endogenous peptide a	16	1
GO:0006415//translational termination	16	1
GO:0007620//copulation	16	1
GO:0009312//oligosaccharide biosynthetic process	16	1
GO:0010823//negative regulation of mitochondrion organization	16	1
GO:0015669//gas transport	16	1
GO:0016577//histone demethylation	16	1
GO:0019885//antigen processing and presentation of endogenous peptide a	16	1
GO:0023058//adaptation of signaling pathway	16	1
GO:0030865//cortical cytoskeleton organization	16	1
GO:0032098//regulation of appetite	16	1
GO:0034199//activation of protein kinase A activity	16	1
GO:0034375//high-density lipoprotein particle remodeling	16	1
GO:0035883//enteroendocrine cell differentiation	16	1
GO:0035987//endodermal cell differentiation	16	1
GO:0042572//retinol metabolic process	16	1
GO:0044058//regulation of digestive system process	16	1
GO:0045026//plasma membrane fusion	16	1
GO:0045907//positive regulation of vasoconstriction	16	1
GO:0051491//positive regulation of filopodium assembly	16	1
GO:0051881//regulation of mitochondrial membrane potential	16	1
GO:0051962//positive regulation of nervous system development	16	1
GO:0051965//positive regulation of synapse assembly	16	1
GO:0060314//regulation of ryanodine-sensitive calcium-release channel act	16	1
GO:0060395//SMAD protein signal transduction	16	1
GO:0070229//negative regulation of lymphocyte apoptotic process	16	1
GO:0070371//ERK1 and ERK2 cascade	16	1
GO:0072132//mesenchyme morphogenesis	16	1
GO:0090025//regulation of monocyte chemotaxis	16	1
GO:0090342//regulation of cell aging	16	1
GO:2001239//regulation of extrinsic apoptotic signaling pathway in absence	16	1
GO:0031345//negative regulation of cell projection organization	64	3
GO:0045639//positive regulation of myeloid cell differentiation	64	3
GO:0001558//regulation of cell growth	235	10
GO:0046637//regulation of alpha-beta T cell differentiation	40	2
GO:0006520//cellular amino acid metabolic process	407	17
GO:0051170//nuclear import	89	4
GO:2000045//regulation of G1/S transition of mitotic cell cycle	89	4
GO:0007254//JNK cascade	65	3
GO:0045582//positive regulation of T cell differentiation	65	3
GO:0006200//ATP catabolic process	139	6
GO:0018393//internal peptidyl-lysine acetylation	90	4
GO:0031647//regulation of protein stability	90	4
GO:0000186//activation of MAPKK activity	41	2
GO:0007034//vacuolar transport	41	2
GO:0007416//synapse assembly	41	2

GO:0023021//termination of signal transduction	41	2
GO:0034134//toll-like receptor 2 signaling pathway	41	2
GO:0002250//adaptive immune response	164	7
GO:0006917//induction of apoptosis	164	7
GO:0000718//nucleotide-excision repair, DNA damage removal	17	1
GO:0001782//B cell homeostasis	17	1
GO:0001945//lymph vessel development	17	1
GO:0002704//negative regulation of leukocyte mediated immunity	17	1
GO:0002823//negative regulation of adaptive immune response based on s	17	1
GO:0004889//acetylcholine-activated cation-selective channel activity	17	1
GO:0006595//polyamine metabolic process	17	1
GO:0007271//synaptic transmission, cholinergic	17	1
GO:0007616//long-term memory	17	1
GO:0009208//pyrimidine ribonucleoside triphosphate metabolic process	17	1
GO:0010824//regulation of centrosome duplication	17	1
GO:0014065//phosphatidylinositol 3-kinase cascade	17	1
GO:0015269//calcium-activated potassium channel activity	17	1
GO:0015299//solute:hydrogen antiporter activity	17	1
GO:0015800//acidic amino acid transport	17	1
GO:0031000//response to caffeine	17	1
GO:0031114//regulation of microtubule depolymerization	17	1
GO:0032105//negative regulation of response to extracellular stimulus	17	1
GO:0032108//negative regulation of response to nutrient levels	17	1
GO:0032365//intracellular lipid transport	17	1
GO:0032400//melanosome localization	17	1
GO:0043001//Golgi to plasma membrane protein transport	17	1
GO:0043206//extracellular fibril organization	17	1
GO:0043500//muscle adaptation	17	1
GO:0043537//negative regulation of blood vessel endothelial cell migration	17	1
GO:0044089//positive regulation of cellular component biogenesis	17	1
GO:0044349//DNA excision	17	1
GO:0045080//positive regulation of chemokine biosynthetic process	17	1
GO:0045930//negative regulation of mitotic cell cycle	17	1
GO:0050891//multicellular organismal water homeostasis	17	1
GO:0051205//protein insertion into membrane	17	1
GO:0051797//regulation of hair follicle development	17	1
GO:0060334//regulation of interferon-gamma-mediated signaling pathway	17	1
GO:0061384//heart trabecula morphogenesis	17	1
GO:0070536//protein K63-linked deubiquitination	17	1
GO:0070997//neuron death	17	1
GO:0071675//regulation of mononuclear cell migration	17	1
GO:0097435//fibril organization	17	1
GO:1900449//regulation of glutamate receptor signaling pathway	17	1
GO:1901655//cellular response to ketone	17	1
GO:2000406//positive regulation of T cell migration	17	1
GO:0012502//induction of programmed cell death	165	7
GO:0018394//peptidyl-lysine acetylation	91	4
GO:0007264//small GTPase mediated signal transduction	264	11
GO:0051259//protein oligomerization	289	12
GO:0000096//sulfur amino acid metabolic process	42	2
GO:0006220//pyrimidine nucleotide metabolic process	42	2
GO:0015293//symporter activity	42	2
GO:0015294//solute:cation symporter activity	42	2
GO:0035264//multicellular organism growth	42	2
GO:0045670//regulation of osteoclast differentiation	42	2
GO:0030801//positive regulation of cyclic nucleotide metabolic process	67	3
GO:1902275//regulation of chromatin organization	67	3
GO:0010212//response to ionizing radiation	92	4

GO:0045927//positive regulation of growth	117	5
GO:0043603//cellular amide metabolic process	142	6
GO:0016044//cellular membrane organization	365	15
GO:0003071//renal system process involved in regulation of systemic arteri	18	1
GO:0006482//protein demethylation	18	1
GO:0006611//protein export from nucleus	18	1
GO:0008089//anterograde axon cargo transport	18	1
GO:0008214//protein dealkylation	18	1
GO:0009074//aromatic amino acid family catabolic process	18	1
GO:0009303//rRNA transcription	18	1
GO:0014821//phasic smooth muscle contraction	18	1
GO:0016024//CDP-diacylglycerol biosynthetic process	18	1
GO:0019883//antigen processing and presentation of endogenous antigen	18	1
GO:0021696//cerebellar cortex morphogenesis	18	1
GO:0030194//positive regulation of blood coagulation	18	1
GO:0032007//negative regulation of TOR signaling cascade	18	1
GO:0042401//cellular biogenic amine biosynthetic process	18	1
GO:0042596//fear response	18	1
GO:0043491//protein kinase B signaling cascade	18	1
GO:0043516//regulation of DNA damage response, signal transduction by p	18	1
GO:0046325//negative regulation of glucose import	18	1
GO:0046636//negative regulation of alpha-beta T cell activation	18	1
GO:0048484//enteric nervous system development	18	1
GO:0050927//positive regulation of positive chemotaxis	18	1
GO:0051085//chaperone mediated protein folding requiring cofactor	18	1
GO:0051875//pigment granule localization	18	1
GO:0052652//cyclic purine nucleotide metabolic process	18	1
GO:0055012//ventricular cardiac muscle cell differentiation	18	1
GO:0060330//regulation of response to interferon-gamma	18	1
GO:0085029//extracellular matrix assembly	18	1
GO:0090003//regulation of establishment of protein localization to plasma n	18	1
GO:1900048//positive regulation of hemostasis	18	1
GO:1901796//regulation of signal transduction by p53 class mediator	18	1
GO:1902305//regulation of sodium ion transmembrane transport	18	1
GO:2000649//regulation of sodium ion transmembrane transporter activity	18	1
GO:0006885//regulation of pH	43	2
GO:0007159//leukocyte cell-cell adhesion	43	2
GO:0030048//actin filament-based movement	43	2
GO:0034162//toll-like receptor 9 signaling pathway	43	2
GO:0009163//nucleoside biosynthetic process	93	4
GO:0006470//protein dephosphorylation	144	6
GO:1901565//organonitrogen compound catabolic process	616	25
GO:0006475//internal protein amino acid acetylation	94	4
GO:0033044//regulation of chromosome organization	94	4
GO:0035335//peptidyl-tyrosine dephosphorylation	94	4
GO:0055076//transition metal ion homeostasis	94	4
GO:1901659//glycosyl compound biosynthetic process	94	4
GO:0003824//catalytic activity	294	12
GO:0006941//striated muscle contraction	44	2
GO:0032012//regulation of ARF protein signal transduction	44	2
GO:0046635//positive regulation of alpha-beta T cell activation	44	2
GO:0060443//mammary gland morphogenesis	44	2
GO:0030098//lymphocyte differentiation	145	6
GO:0043543//protein acylation	120	5
GO:0061024//membrane organization	370	15
GO:0030217//T cell differentiation	95	4
GO:0000188//inactivation of MAPK activity	19	1
GO:0006906//vesicle fusion	19	1

GO:0007405//neuroblast proliferation	19	1
GO:0009309//amine biosynthetic process	19	1
GO:0015491//cation:cation antiporter activity	19	1
GO:0019080//viral genome expression	19	1
GO:0033081//regulation of T cell differentiation in thymus	19	1
GO:0035162//embryonic hemopoiesis	19	1
GO:0036230//granulocyte activation	19	1
GO:0043462//regulation of ATPase activity	19	1
GO:0043567//regulation of insulin-like growth factor receptor signaling path	19	1
GO:0045577//regulation of B cell differentiation	19	1
GO:0046341//CDP-diacylglycerol metabolic process	19	1
GO:0046627//negative regulation of insulin receptor signaling pathway	19	1
GO:0048665//neuron fate specification	19	1
GO:0050926//regulation of positive chemotaxis	19	1
GO:0051023//regulation of immunoglobulin secretion	19	1
GO:0060397//JAK-STAT cascade involved in growth hormone signaling pat	19	1
GO:0060765//regulation of androgen receptor signaling pathway	19	1
GO:0072337//modified amino acid transport	19	1
GO:0072378//blood coagulation, fibrin clot formation	19	1
GO:0090200//positive regulation of release of cytochrome c from mitochonc	19	1
GO:0030307//positive regulation of cell growth	70	3
GO:0046164//alcohol catabolic process	45	2
GO:0046889//positive regulation of lipid biosynthetic process	45	2
GO:0050994//regulation of lipid catabolic process	45	2
GO:0070301//cellular response to hydrogen peroxide	45	2
GO:0044403//symbiosis, encompassing mutualism through parasitism	447	18
GO:0044419//interspecies interaction between organisms	447	18
GO:0050868//negative regulation of T cell activation	71	3
GO:0030534//adult behavior	97	4
GO:0032886//regulation of microtubule-based process	97	4
GO:0007243//intracellular protein kinase cascade	274	11
GO:0001947//heart looping	46	2
GO:0032984//macromolecular complex disassembly	46	2
GO:0048524//positive regulation of viral process	46	2
GO:0051781//positive regulation of cell division	46	2
GO:0070661//leukocyte proliferation	46	2
GO:0000726//non-recombinational repair	20	1
GO:0002825//regulation of T-helper 1 type immune response	20	1
GO:0003209//cardiac atrium morphogenesis	20	1
GO:0006501//C-terminal protein lipidation	20	1
GO:0006904//vesicle docking involved in exocytosis	20	1
GO:0007098//centrosome cycle	20	1
GO:0007530//sex determination	20	1
GO:0008227//G-protein coupled amine receptor activity	20	1
GO:0009067//aspartate family amino acid biosynthetic process	20	1
GO:0009127//purine nucleoside monophosphate biosynthetic process	20	1
GO:0009168//purine ribonucleoside monophosphate biosynthetic process	20	1
GO:0009395//phospholipid catabolic process	20	1
GO:0015296//anion:cation symporter activity	20	1
GO:0018200//peptidyl-glutamic acid modification	20	1
GO:0030032//lamellipodium assembly	20	1
GO:0031111//negative regulation of microtubule polymerization or depolyme	20	1
GO:0031664//regulation of lipopolysaccharide-mediated signaling pathway	20	1
GO:0034446//substrate adhesion-dependent cell spreading	20	1
GO:0042634//regulation of hair cycle	20	1
GO:0043299//leukocyte degranulation	20	1
GO:0043691//reverse cholesterol transport	20	1
GO:0046839//phospholipid dephosphorylation	20	1

GO:0048536//spleen development	20	1
GO:0050820//positive regulation of coagulation	20	1
GO:0051044//positive regulation of membrane protein ectodomain proteolys	20	1
GO:0060444//branching involved in mammary gland duct morphogenesis	20	1
GO:0002700//regulation of production of molecular mediator of immune res	72	3
GO:0097193//intrinsic apoptotic signaling pathway	98	4
GO:0090407//organophosphate biosynthetic process	376	15
GO:0070838//divalent metal ion transport	175	7
GO:0000041//transition metal ion transport	73	3
GO:0038093//Fc receptor signaling pathway	73	3
GO:0043473//pigmentation	73	3
GO:0007492//endoderm development	47	2
GO:0046620//regulation of organ growth	47	2
GO:0050830//defense response to Gram-positive bacterium	47	2
GO:0060491//regulation of cell projection assembly	47	2
GO:0044267//cellular protein metabolic process	2591	104
GO:1901990//regulation of mitotic cell cycle phase transition	151	6
GO:0002676//regulation of chronic inflammatory response	21	1
GO:0006071//glycerol metabolic process	21	1
GO:0006893//Golgi to plasma membrane transport	21	1
GO:0006998//nuclear envelope organization	21	1
GO:0008038//neuron recognition	21	1
GO:0009190//cyclic nucleotide biosynthetic process	21	1
GO:0010799//regulation of peptidyl-threonine phosphorylation	21	1
GO:0010829//negative regulation of glucose transport	21	1
GO:0021846//cell proliferation in forebrain	21	1
GO:0032757//positive regulation of interleukin-8 production	21	1
GO:0032891//negative regulation of organic acid transport	21	1
GO:0034377//plasma lipoprotein particle assembly	21	1
GO:0042551//neuron maturation	21	1
GO:0043243//positive regulation of protein complex disassembly	21	1
GO:0043330//response to exogenous dsRNA	21	1
GO:0046605//regulation of centrosome cycle	21	1
GO:0060048//cardiac muscle contraction	21	1
GO:0090398//cellular senescence	21	1
GO:1900077//negative regulation of cellular response to insulin stimulus	21	1
GO:1901657//glycosyl compound metabolic process	505	20
GO:0051302//regulation of cell division	74	3
GO:0072329//monocarboxylic acid catabolic process	74	3
GO:0050810//regulation of steroid biosynthetic process	48	2
GO:0061371//determination of heart left/right asymmetry	48	2
GO:0072511//divalent inorganic cation transport	178	7
GO:0043170//macromolecule metabolic process	4724	190
GO:0006814//sodium ion transport	101	4
GO:0031349//positive regulation of defense response	204	8
GO:0044237//cellular metabolic process	6082	245
GO:1901361//organic cyclic compound catabolic process	635	25
GO:0002718//regulation of cytokine production involved in immune respons	49	2
GO:0007589//body fluid secretion	49	2
GO:0017148//negative regulation of translation	49	2
GO:0019362//pyridine nucleotide metabolic process	49	2
GO:0043401//steroid hormone mediated signaling pathway	49	2
GO:0046496//nicotinamide nucleotide metabolic process	49	2
GO:0048489//synaptic vesicle transport	49	2
GO:0097480//establishment of synaptic vesicle localization	49	2
GO:0002011//morphogenesis of an epithelial sheet	22	1
GO:0002863//positive regulation of inflammatory response to antigenic stim	22	1
GO:0003230//cardiac atrium development	22	1

GO:0005227//calcium activated cation channel activity	22	1
GO:0006509//membrane protein ectodomain proteolysis	22	1
GO:0007398//ectoderm development	22	1
GO:0009225//nucleotide-sugar metabolic process	22	1
GO:0010043//response to zinc ion	22	1
GO:0018904//ether metabolic process	22	1
GO:0019400//alditol metabolic process	22	1
GO:0021522//spinal cord motor neuron differentiation	22	1
GO:0021772//olfactory bulb development	22	1
GO:0030261//chromosome condensation	22	1
GO:0030262//apoptotic nuclear changes	22	1
GO:0032369//negative regulation of lipid transport	22	1
GO:0040020//regulation of meiosis	22	1
GO:0042133//neurotransmitter metabolic process	22	1
GO:0042992//negative regulation of transcription factor import into nucleus	22	1
GO:0043030//regulation of macrophage activation	22	1
GO:0044275//cellular carbohydrate catabolic process	22	1
GO:0045124//regulation of bone resorption	22	1
GO:0045132//meiotic chromosome segregation	22	1
GO:0045581//negative regulation of T cell differentiation	22	1
GO:0046173//polyol biosynthetic process	22	1
GO:0048278//vesicle docking	22	1
GO:0051181//cofactor transport	22	1
GO:0051489//regulation of filopodium assembly	22	1
GO:0060396//growth hormone receptor signaling pathway	22	1
GO:0065005//protein-lipid complex assembly	22	1
GO:0072525//pyridine-containing compound biosynthetic process	22	1
GO:0002703//regulation of leukocyte mediated immunity	128	5
GO:0002822//regulation of adaptive immune response based on somatic re	102	4
GO:0015085//calcium ion transmembrane transporter activity	102	4
GO:0042035//regulation of cytokine biosynthetic process	102	4
GO:0045621//positive regulation of lymphocyte differentiation	76	3
GO:0051648//vesicle localization	103	4
GO:0019693//ribose phosphate metabolic process	487	19
GO:0032677//regulation of interleukin-8 production	50	2
GO:0033077//T cell differentiation in thymus	50	2
GO:0048525//negative regulation of viral process	50	2
GO:0002761//regulation of myeloid leukocyte differentiation	77	3
GO:0043900//regulation of multi-organism process	208	8
GO:0006536//glutamate metabolic process	23	1
GO:0009147//pyrimidine nucleoside triphosphate metabolic process	23	1
GO:0009220//pyrimidine ribonucleotide biosynthetic process	23	1
GO:0021988//olfactory lobe development	23	1
GO:0032480//negative regulation of type I interferon production	23	1
GO:0042181//ketone biosynthetic process	23	1
GO:0045646//regulation of erythrocyte differentiation	23	1
GO:0045739//positive regulation of DNA repair	23	1
GO:0046189//phenol-containing compound biosynthetic process	23	1
GO:0050995//negative regulation of lipid catabolic process	23	1
GO:0051668//localization within membrane	23	1
GO:0051955//regulation of amino acid transport	23	1
GO:0051983//regulation of chromosome segregation	23	1
GO:0071377//cellular response to glucagon stimulus	23	1
GO:0071378//cellular response to growth hormone stimulus	23	1
GO:0009154//purine ribonucleotide catabolic process	363	14
GO:0043902//positive regulation of multi-organism process	51	2
GO:0000165//MAPK cascade	131	5
GO:0018205//peptidyl-lysine modification	131	5

GO:0009261//ribonucleotide catabolic process	364	14
GO:1901605//alpha-amino acid metabolic process	210	8
GO:0045185//maintenance of protein location	79	3
GO:0051592//response to calcium ion	79	3
GO:0006879//cellular iron ion homeostasis	52	2
GO:0050871//positive regulation of B cell activation	52	2
GO:0046390//ribose phosphate biosynthetic process	106	4
GO:0016311//dephosphorylation	185	7
GO:0001914//regulation of T cell mediated cytotoxicity	24	1
GO:0001960//negative regulation of cytokine-mediated signaling pathway	24	1
GO:0007194//negative regulation of adenylate cyclase activity	24	1
GO:0009394//2-deoxyribonucleotide metabolic process	24	1
GO:0010259//multicellular organismal aging	24	1
GO:0010559//regulation of glycoprotein biosynthetic process	24	1
GO:0016339//calcium-dependent cell-cell adhesion	24	1
GO:0018410//C-terminal protein amino acid modification	24	1
GO:0019370//leukotriene biosynthetic process	24	1
GO:0021587//cerebellum morphogenesis	24	1
GO:0032414//positive regulation of ion transmembrane transporter activity	24	1
GO:0042220//response to cocaine	24	1
GO:0043029//T cell homeostasis	24	1
GO:0051445//regulation of meiotic cell cycle	24	1
GO:0002274//myeloid leukocyte activation	80	3
GO:0009150//purine ribonucleotide metabolic process	470	18
GO:0018105//peptidyl-serine phosphorylation	53	2
GO:0031333//negative regulation of protein complex assembly	53	2
GO:0042158//lipoprotein biosynthetic process	53	2
GO:0044728//DNA methylation or demethylation	53	2
GO:0045454//cell redox homeostasis	53	2
GO:0050770//regulation of axonogenesis	81	3
GO:0051403//stress-activated MAPK cascade	81	3
GO:0001706//endoderm formation	25	1
GO:0001710//mesodermal cell fate commitment	25	1
GO:0001961//positive regulation of cytokine-mediated signaling pathway	25	1
GO:0002820//negative regulation of adaptive immune response	25	1
GO:0008210//estrogen metabolic process	25	1
GO:0010508//positive regulation of autophagy	25	1
GO:0015298//solute:cation antiporter activity	25	1
GO:0031280//negative regulation of cyclase activity	25	1
GO:0032715//negative regulation of interleukin-6 production	25	1
GO:0050853//B cell receptor signaling pathway	25	1
GO:0050879//multicellular organismal movement	25	1
GO:0050881//musculoskeletal movement	25	1
GO:0060047//heart contraction	25	1
GO:0060760//positive regulation of response to cytokine stimulus	25	1
GO:0061515//myeloid cell development	25	1
GO:0071479//cellular response to ionizing radiation	25	1
GO:2000403//positive regulation of lymphocyte migration	25	1
GO:1901292//nucleoside phosphate catabolic process	396	15
GO:0021549//cerebellum development	54	2
GO:0030183//B cell differentiation	54	2
GO:0001959//regulation of cytokine-mediated signaling pathway	82	3
GO:0007612//learning	82	3
GO:0031098//stress-activated protein kinase signaling cascade	82	3
GO:0002832//negative regulation of response to biotic stimulus	26	1
GO:0002922//positive regulation of humoral immune response	26	1
GO:0003015//heart process	26	1
GO:0008333//endosome to lysosome transport	26	1

GO:0019692//deoxyribose phosphate metabolic process	26	1
GO:0021532//neural tube patterning	26	1
GO:0030513//positive regulation of BMP signaling pathway	26	1
GO:0032856//activation of Ras GTPase activity	26	1
GO:0040018//positive regulation of multicellular organism growth	26	1
GO:0042462//eye photoreceptor cell development	26	1
GO:0051350//negative regulation of lyase activity	26	1
GO:0060761//negative regulation of response to cytokine stimulus	26	1
GO:0070534//protein K63-linked ubiquitination	26	1
GO:2001257//regulation of cation channel activity	26	1
GO:0030518//intracellular steroid hormone receptor signaling pathway	55	2
GO:0071158//positive regulation of cell cycle arrest	55	2
GO:0032680//regulation of tumor necrosis factor production	83	3
GO:0051641//cellular localization	1390	54
GO:0006195//purine nucleotide catabolic process	374	14
GO:0051260//protein homooligomerization	191	7
GO:0071822//protein complex subunit organization	759	29
GO:0002224//toll-like receptor signaling pathway	84	3
GO:0022904//respiratory electron transport chain	84	3
GO:0006308//DNA catabolic process	56	2
GO:0006414//translational elongation	56	2
GO:0006921//cellular component disassembly involved in execution phase	56	2
GO:0002714//positive regulation of B cell mediated immunity	27	1
GO:0002891//positive regulation of immunoglobulin mediated immune resp	27	1
GO:0006312//mitotic recombination	27	1
GO:0006614//SRP-dependent cotranslational protein targeting to membran	27	1
GO:0009072//aromatic amino acid family metabolic process	27	1
GO:0009651//response to salt stress	27	1
GO:0030318//melanocyte differentiation	27	1
GO:0034109//homotypic cell-cell adhesion	27	1
GO:0035872//nucleotide-binding domain, leucine rich repeat containing rec	27	1
GO:0048147//negative regulation of fibroblast proliferation	27	1
GO:0046434//organophosphate catabolic process	429	16
GO:0055086//nucleobase-containing small molecule metabolic process	661	25
GO:0008064//regulation of actin polymerization or depolymerization	85	3
GO:0030832//regulation of actin filament length	85	3
GO:0060759//regulation of response to cytokine stimulus	85	3
GO:0072523//purine-containing compound catabolic process	378	14
GO:0005254//chloride channel activity	57	2
GO:0006892//post-Golgi vesicle-mediated transport	57	2
GO:0051952//regulation of amine transport	57	2
GO:0009259//ribonucleotide metabolic process	483	18
GO:0005262//calcium channel activity	86	3
GO:0007218//neuropeptide signaling pathway	86	3
GO:0043901//negative regulation of multi-organism process	86	3
GO:0030308//negative regulation of cell growth	114	4
GO:1901991//negative regulation of mitotic cell cycle phase transition	114	4
GO:1902107//positive regulation of leukocyte differentiation	114	4
GO:0000080//G1 phase of mitotic cell cycle	28	1
GO:0002275//myeloid cell activation involved in immune response	28	1
GO:0006613//cotranslational protein targeting to membrane	28	1
GO:0009262//deoxyribonucleotide metabolic process	28	1
GO:0014075//response to amine stimulus	28	1
GO:0033762//response to glucagon stimulus	28	1
GO:0046928//regulation of neurotransmitter secretion	28	1
GO:0050433//regulation of catecholamine secretion	28	1
GO:0070830//tight junction assembly	28	1
GO:0051649//establishment of localization in cell	1175	45

GO:0051258//protein polymerization	58	2
GO:0055067//monovalent inorganic cation homeostasis	58	2
GO:0006461//protein complex assembly	615	23
GO:0002819//regulation of adaptive immune response	115	4
GO:0017038//protein import	115	4
GO:0005253//anion channel activity	59	2
GO:0045761//regulation of adenylate cyclase activity	59	2
GO:0006163//purine nucleotide metabolic process	488	18
GO:0031295//T cell costimulation	88	3
GO:0042113//B cell activation	88	3
GO:0045926//negative regulation of growth	171	6
GO:0001578//microtubule bundle formation	29	1
GO:0006221//pyrimidine nucleotide biosynthetic process	29	1
GO:0006506//GPI anchor biosynthetic process	29	1
GO:0006901//vesicle coating	29	1
GO:0007190//activation of adenylate cyclase activity	29	1
GO:0017144//drug metabolic process	29	1
GO:0017158//regulation of calcium ion-dependent exocytosis	29	1
GO:0021695//cerebellar cortex development	29	1
GO:0021879//forebrain neuron differentiation	29	1
GO:0032312//regulation of ARF GTPase activity	29	1
GO:0042168//heme metabolic process	29	1
GO:0042743//hydrogen peroxide metabolic process	29	1
GO:0045047//protein targeting to ER	29	1
GO:0045777//positive regulation of blood pressure	29	1
GO:0050850//positive regulation of calcium-mediated signaling	29	1
GO:0051279//regulation of release of sequestered calcium ion into cytosol	29	1
GO:0051318//G1 phase	29	1
GO:2000107//negative regulation of leukocyte apoptotic process	29	1
GO:0006366//transcription from RNA polymerase II promoter	252	9
GO:0034341//response to interferon-gamma	144	5
GO:0009057//macromolecule catabolic process	645	24
GO:0070271//protein complex biogenesis	620	23
GO:0006733//oxidoreduction coenzyme metabolic process	60	2
GO:0015078//hydrogen ion transmembrane transporter activity	60	2
GO:0021510//spinal cord development	60	2
GO:0031056//regulation of histone modification	60	2
GO:0046634//regulation of alpha-beta T cell activation	60	2
GO:0031294//lymphocyte costimulation	89	3
GO:0005230//extracellular ligand-gated ion channel activity	30	1
GO:0005231//excitatory extracellular ligand-gated ion channel activity	30	1
GO:0007270//neuron-neuron synaptic transmission	30	1
GO:0031572//G2 DNA damage checkpoint	30	1
GO:0032411//positive regulation of transporter activity	30	1
GO:0032653//regulation of interleukin-10 production	30	1
GO:0033059//cellular pigmentation	30	1
GO:0033619//membrane protein proteolysis	30	1
GO:0043331//response to dsRNA	30	1
GO:0050999//regulation of nitric-oxide synthase activity	30	1
GO:0070228//regulation of lymphocyte apoptotic process	30	1
GO:0072599//establishment of protein localization to endoplasmic reticulum	30	1
GO:0000086//G2/M transition of mitotic cell cycle	90	3
GO:0006821//chloride transport	61	2
GO:0032368//regulation of lipid transport	61	2
GO:0034142//toll-like receptor 4 signaling pathway	61	2
GO:0050851//antigen receptor-mediated signaling pathway	119	4
GO:0016570//histone modification	229	8
GO:0009166//nucleotide catabolic process	389	14

GO:0042454//ribonucleoside catabolic process	363	13
GO:0072521//purine-containing compound metabolic process	521	19
GO:0009152//purine ribonucleotide biosynthetic process	91	3
GO:0051650//establishment of vesicle localization	91	3
GO:0015992//proton transport	62	2
GO:0030330//DNA damage response, signal transduction by p53 class med	62	2
GO:0002861//regulation of inflammatory response to antigenic stimulus	31	1
GO:0006505//GPI anchor metabolic process	31	1
GO:0007595//lactation	31	1
GO:0008209//androgen metabolic process	31	1
GO:0018107//peptidyl-threonine phosphorylation	31	1
GO:0032091//negative regulation of protein binding	31	1
GO:0032371//regulation of sterol transport	31	1
GO:0032374//regulation of cholesterol transport	31	1
GO:0035690//cellular response to drug	31	1
GO:0035725//sodium ion transmembrane transport	31	1
GO:0051865//protein autoubiquitination	31	1
GO:0050877//neurological system process	1396	53
GO:0002221//pattern recognition receptor signaling pathway	92	3
GO:0009119//ribonucleoside metabolic process	471	17
GO:0016569//covalent chromatin modification	232	8
GO:0006818//hydrogen transport	63	2
GO:0008361//regulation of cell size	63	2
GO:0019439//aromatic compound catabolic process	604	22
GO:0001754//eye photoreceptor cell differentiation	32	1
GO:0006900//membrane budding	32	1
GO:0007040//lysosome organization	32	1
GO:0030890//positive regulation of B cell proliferation	32	1
GO:0031058//positive regulation of histone modification	32	1
GO:0033209//tumor necrosis factor-mediated signaling pathway	32	1
GO:0042461//photoreceptor cell development	32	1
GO:0045762//positive regulation of adenylate cyclase activity	32	1
GO:0045839//negative regulation of mitosis	32	1
GO:0046580//negative regulation of Ras protein signal transduction	32	1
GO:0048639//positive regulation of developmental growth	32	1
GO:0051784//negative regulation of nuclear division	32	1
GO:0060338//regulation of type I interferon-mediated signaling pathway	32	1
GO:0045619//regulation of lymphocyte differentiation	122	4
GO:0071346//cellular response to interferon-gamma	122	4
GO:0046128//purine ribonucleoside metabolic process	448	16
GO:0050688//regulation of defense response to virus	64	2
GO:0051341//regulation of oxidoreductase activity	64	2
GO:0097194//execution phase of apoptosis	64	2
GO:0000082//G1/S transition of mitotic cell cycle	94	3
GO:0002758//innate immune response-activating signal transduction	94	3
GO:0046488//phosphatidylinositol metabolic process	123	4
GO:0007131//reciprocal meiotic recombination	33	1
GO:0007618//mating	33	1
GO:0009156//ribonucleoside monophosphate biosynthetic process	33	1
GO:0035825//reciprocal DNA recombination	33	1
GO:0050771//negative regulation of axonogenesis	33	1
GO:0051058//negative regulation of small GTPase mediated signal transdu	33	1
GO:0060563//neuroepithelial cell differentiation	33	1
GO:0090382//phagosome maturation	33	1
GO:1902115//regulation of organelle assembly	33	1
GO:0002768//immune response-regulating cell surface receptor signaling p	208	7
GO:0031279//regulation of cyclase activity	65	2
GO:0032321//positive regulation of Rho GTPase activity	65	2

GO:0050852//T cell receptor signaling pathway	95	3
GO:0042278//purine nucleoside metabolic process	451	16
GO:0032535//regulation of cellular component size	153	5
GO:0002764//immune response-regulating signaling pathway	291	10
GO:0072509//divalent inorganic cation transmembrane transporter activity	125	4
GO:0006164//purine nucleotide biosynthetic process	96	3
GO:0007005//mitochondrion organization	182	6
GO:0019218//regulation of steroid metabolic process	66	2
GO:0032874//positive regulation of stress-activated MAPK cascade	66	2
GO:0071156//regulation of cell cycle arrest	66	2
GO:0009069//serine family amino acid metabolic process	34	1
GO:0009620//response to fungus	34	1
GO:0021983//pituitary gland development	34	1
GO:0030433//ER-associated protein catabolic process	34	1
GO:0043297//apical junction assembly	34	1
GO:0048387//negative regulation of retinoic acid receptor signaling pathwa	34	1
GO:2001251//negative regulation of chromosome organization	34	1
GO:0009143//nucleoside triphosphate catabolic process	348	12
GO:0009164//nucleoside catabolic process	375	13
GO:0070304//positive regulation of stress-activated protein kinase signaling	67	2
GO:0006909//phagocytosis	127	4
GO:0031281//positive regulation of cyclase activity	35	1
GO:0032272//negative regulation of protein polymerization	35	1
GO:0072528//pyrimidine-containing compound biosynthetic process	35	1
GO:2000242//negative regulation of reproductive process	35	1
GO:0060333//interferon-gamma-mediated signaling pathway	98	3
GO:0002433//immune response-regulating cell surface receptor signaling p	68	2
GO:0008037//cell recognition	68	2
GO:0038094//Fc-gamma receptor signaling pathway	68	2
GO:0038096//Fc-gamma receptor signaling pathway involved in phagocyto	68	2
GO:0051339//regulation of lyase activity	68	2
GO:0009059//macromolecule biosynthetic process	1293	48
GO:0045580//regulation of T cell differentiation	99	3
GO:1901658//glycosyl compound catabolic process	379	13
GO:0000184//nuclear-transcribed mRNA catabolic process, nonsense-medi	36	1
GO:0030838//positive regulation of actin filament polymerization	36	1
GO:0032104//regulation of response to extracellular stimulus	36	1
GO:0032107//regulation of response to nutrient levels	36	1
GO:0032722//positive regulation of chemokine production	36	1
GO:0032755//positive regulation of interleukin-6 production	36	1
GO:0034146//toll-like receptor 5 signaling pathway	36	1
GO:0034166//toll-like receptor 10 signaling pathway	36	1
GO:0051353//positive regulation of oxidoreductase activity	36	1
GO:0006304//DNA modification	69	2
GO:0006944//cellular membrane fusion	69	2
GO:0007009//plasma membrane organization	69	2
GO:0044271//cellular nitrogen compound biosynthetic process	752	27
GO:0006275//regulation of DNA replication	100	3
GO:0006839//mitochondrial transport	100	3
GO:0009566//fertilization	100	3
GO:0009141//nucleoside triphosphate metabolic process	408	14
GO:0006152//purine nucleoside catabolic process	354	12
GO:0046130//purine ribonucleoside catabolic process	354	12
GO:0006753//nucleoside phosphate metabolic process	622	22
GO:0000910//cytokinesis	70	2
GO:0002431//Fc receptor mediated stimulatory signaling pathway	70	2
GO:0007219//Notch signaling pathway	70	2
GO:0042770//signal transduction in response to DNA damage	70	2

GO:0046916//cellular transition metal ion homeostasis	70	2
GO:0009116//nucleoside metabolic process	490	17
GO:0002757//immune response-activating signal transduction	273	9
GO:0001658//branching involved in ureteric bud morphogenesis	37	1
GO:0009066//aspartate family amino acid metabolic process	37	1
GO:0009124//nucleoside monophosphate biosynthetic process	37	1
GO:0021515//cell differentiation in spinal cord	37	1
GO:0051084//de novo posttranslational protein folding	37	1
GO:0051297//centrosome organization	37	1
GO:0051349//positive regulation of lyase activity	37	1
GO:0051588//regulation of neurotransmitter transport	37	1
GO:0051640//organelle localization	189	6
GO:1901987//regulation of cell cycle phase transition	246	8
GO:0030163//protein catabolic process	411	14
GO:0002218//activation of innate immune response	102	3
GO:0006958//complement activation, classical pathway	71	2
GO:0009451//RNA modification	71	2
GO:0009755//hormone-mediated signaling pathway	71	2
GO:0031110//regulation of microtubule polymerization or depolymerization	38	1
GO:0046530//photoreceptor cell differentiation	38	1
GO:0009260//ribonucleotide biosynthetic process	103	3
GO:0043270//positive regulation of ion transport	103	3
GO:0007283//spermatogenesis	359	12
GO:0046700//heterocycle catabolic process	602	21
GO:0002429//immune response-activating cell surface receptor signaling pa	192	6
GO:0048232//male gamete generation	360	12
GO:0051603//proteolysis involved in cellular protein catabolic process	360	12
GO:0044270//cellular nitrogen compound catabolic process	603	21
GO:0045088//regulation of innate immune response	193	6
GO:0006778//porphyrin-containing compound metabolic process	39	1
GO:0009311//oligosaccharide metabolic process	39	1
GO:0021872//forebrain generation of neurons	39	1
GO:0048284//organelle fusion	39	1
GO:0060359//response to ammonium ion	39	1
GO:NM_0010	250	8
GO:0007051//spindle organization	73	2
GO:0061025//membrane fusion	73	2
GO:0019941//modification-dependent protein catabolic process	334	11
GO:0009205//purine ribonucleoside triphosphate metabolic process	389	13
GO:0000209//protein polyubiquitination	135	4
GO:0051235//maintenance of location	105	3
GO:1902105//regulation of leukocyte differentiation	195	6
GO:0000413//protein peptidyl-prolyl isomerization	40	1
GO:0006458//de novo protein folding	40	1
GO:0032941//secretion by tissue	40	1
GO:0061351//neural precursor cell proliferation	40	1
GO:0006260//DNA replication	166	5
GO:0006913//nucleocytoplasmic transport	166	5
GO:0034613//cellular protein localization	688	24
GO:1901362//organic cyclic compound biosynthetic process	821	29
GO:0009144//purine nucleoside triphosphate metabolic process	392	13
GO:0043632//modification-dependent macromolecule catabolic process	337	11
GO:0072657//protein localization to membrane	75	2
GO:0009199//ribonucleoside triphosphate metabolic process	394	13
GO:0032320//positive regulation of Ras GTPase activity	138	4
GO:0002637//regulation of immunoglobulin production	41	1
GO:0006282//regulation of DNA repair	41	1
GO:0006521//regulation of cellular amino acid metabolic process	41	1

GO:0006626//protein targeting to mitochondrion	41	1
GO:0006888//ER to Golgi vesicle-mediated transport	41	1
GO:0032890//regulation of organic acid transport	41	1
GO:0050848//regulation of calcium-mediated signaling	41	1
GO:2001252//positive regulation of chromosome organization	41	1
GO:0070727//cellular macromolecule localization	692	24
GO:0000018//regulation of DNA recombination	42	1
GO:0002709//regulation of T cell mediated immunity	42	1
GO:0031023//microtubule organizing center organization	42	1
GO:0038123//toll-like receptor TLR1:TLR2 signaling pathway	42	1
GO:0038124//toll-like receptor TLR6:TLR2 signaling pathway	42	1
GO:0042130//negative regulation of T cell proliferation	42	1
GO:0046651//lymphocyte proliferation	42	1
GO:0070972//protein localization to endoplasmic reticulum	42	1
GO:0051169//nuclear transport	170	5
GO:0009203//ribonucleoside triphosphate catabolic process	342	11
GO:0009207//purine ribonucleoside triphosphate catabolic process	342	11
GO:0009117//nucleotide metabolic process	616	21
GO:0043623//cellular protein complex assembly	200	6
GO:0034655//nucleobase-containing compound catabolic process	563	19
GO:0043547//positive regulation of GTPase activity	201	6
GO:0033036//macromolecule localization	1357	49
GO:0009146//purine nucleoside triphosphate catabolic process	344	11
GO:0002763//positive regulation of myeloid leukocyte differentiation	43	1
GO:0032729//positive regulation of interferon-gamma production	43	1
GO:0032943//mononuclear cell proliferation	43	1
GO:0072655//establishment of protein localization to mitochondrion	43	1
GO:0044257//cellular protein catabolic process	373	12
GO:0033365//protein localization to organelle	289	9
GO:0002455//humoral immune response mediated by circulating immunogl	79	2
GO:0006749//glutathione metabolic process	44	1
GO:0010522//regulation of calcium ion transport into cytosol	44	1
GO:0030594//neurotransmitter receptor activity	44	1
GO:0044704//single-organism reproductive behavior	44	1
GO:0070585//protein localization to mitochondrion	44	1
GO:0006974//response to DNA damage stimulus	513	17
GO:0044770//cell cycle phase transition	174	5
GO:0044772//mitotic cell cycle phase transition	174	5
GO:0007588//excretion	45	1
GO:0032313//regulation of Rab GTPase activity	45	1
GO:0032483//regulation of Rab protein signal transduction	45	1
GO:0032663//regulation of interleukin-2 production	45	1
GO:0032851//positive regulation of Rab GTPase activity	45	1
GO:0033555//multicellular organismal response to stress	45	1
GO:0051289//protein homotetramerization	45	1
GO:0006661//phosphatidylinositol biosynthetic process	82	2
GO:0001910//regulation of leukocyte mediated cytotoxicity	46	1
GO:0030888//regulation of B cell proliferation	46	1
GO:0032768//regulation of monooxygenase activity	46	1
GO:0016054//organic acid catabolic process	178	5
GO:0046395//carboxylic acid catabolic process	178	5
GO:0032774//RNA biosynthetic process	438	14
GO:0032273//positive regulation of protein polymerization	47	1
GO:0001505//regulation of neurotransmitter levels	84	2
GO:0006511//ubiquitin-dependent protein catabolic process	328	10
GO:0002889//regulation of immunoglobulin mediated immune response	48	1
GO:0031341//regulation of cell killing	48	1
GO:0033013//tetrapyrrole metabolic process	48	1

GO:1901988//negative regulation of cell cycle phase transition	213	6
GO:0006351//transcription, DNA-dependent	416	13
GO:0002712//regulation of B cell mediated immunity	49	1
GO:0006497//protein lipidation	49	1
GO:0008630//intrinsic apoptotic signaling pathway in response to DNA dam	49	1
GO:0045727//positive regulation of translation	49	1
GO:0019882//antigen processing and presentation	274	8
GO:0010948//negative regulation of cell cycle process	245	7
GO:0046039//GTP metabolic process	215	6
GO:0033138//positive regulation of peptidyl-serine phosphorylation	50	1
GO:2000106//regulation of leukocyte apoptotic process	50	1
GO:0070647//protein modification by small protein conjugation or removal	505	16
GO:0021536//diencephalon development	51	1
GO:0045333//cellular respiration	123	3
GO:0048285//organelle fission	187	5
GO:0022607//cellular component assembly	1291	45
GO:0000819//sister chromatid segregation	52	1
GO:0002755//MyD88-dependent toll-like receptor signaling pathway	52	1
GO:0006997//nucleus organization	52	1
GO:0032945//negative regulation of mononuclear cell proliferation	52	1
GO:0042440//pigment metabolic process	52	1
GO:0050672//negative regulation of lymphocyte proliferation	52	1
GO:0032271//regulation of protein polymerization	90	2
GO:0072331//signal transduction by p53 class mediator	90	2
GO:0006605//protein targeting	250	7
GO:0042398//cellular modified amino acid biosynthetic process	53	1
GO:0051928//positive regulation of calcium ion transport	53	1
GO:0060337//type I interferon-mediated signaling pathway	53	1
GO:0071357//cellular response to type I interferon	53	1
GO:0044248//cellular catabolic process	1325	46
GO:0044265//cellular macromolecule catabolic process	540	17
GO:0016567//protein ubiquitination	427	13
GO:0002831//regulation of response to biotic stimulus	92	2
GO:0006836//neurotransmitter transport	92	2
GO:0000724//double-strand break repair via homologous recombination	54	1
GO:0006637//acyl-CoA metabolic process	54	1
GO:0010506//regulation of autophagy	54	1
GO:0035383//thioester metabolic process	54	1
GO:0090305//nucleic acid phosphodiester bond hydrolysis	93	2
GO:1901068//guanosine-containing compound metabolic process	224	6
GO:0000725//recombinational repair	55	1
GO:0007033//vacuole organization	55	1
GO:0034340//response to type I interferon	55	1
GO:0031334//positive regulation of protein complex assembly	94	2
GO:0016568//chromatin modification	346	10
GO:0006417//regulation of translation	195	5
GO:ENST000	130	3
GO:0006418//tRNA aminoacylation for protein translation	57	1
GO:0032088//negative regulation of NF-kappaB transcription factor activity	57	1
GO:0032479//regulation of type I interferon production	57	1
GO:0006996//organelle organization	1764	62
GO:0007093//mitotic cell cycle checkpoint	97	2
GO:0007269//neurotransmitter secretion	58	1
GO:0009062//fatty acid catabolic process	58	1
GO:0046330//positive regulation of JNK cascade	58	1
GO:0051053//negative regulation of DNA metabolic process	58	1
GO:0016482//cytoplasmic transport	410	12
GO:0007018//microtubule-based movement	134	3

GO:0006612//protein targeting to membrane	59	1
GO:0000280//nuclear division	168	4
GO:0007067//mitosis	168	4
GO:0034645//cellular macromolecule biosynthetic process	1244	42
GO:0045089//positive regulation of innate immune response	135	3
GO:0044282//small molecule catabolic process	233	6
GO:0044712//single-organism catabolic process	233	6
GO:0010608//posttranscriptional regulation of gene expression	325	9
GO:0019098//reproductive behavior	60	1
GO:0043038//amino acid activation	60	1
GO:0043039//tRNA aminoacylation	60	1
GO:0006184//GTP catabolic process	203	5
GO:0032446//protein modification by small protein conjugation	447	13
GO:0002706//regulation of lymphocyte mediated immunity	102	2
GO:0006914//autophagy	62	1
GO:2000134//negative regulation of G1/S transition of mitotic cell cycle	62	1
GO:0043933//macromolecular complex subunit organization	1038	34
GO:1901069//guanosine-containing compound catabolic process	206	5
GO:0009063//cellular amino acid catabolic process	104	2
GO:0051092//positive regulation of NF-kappaB transcription factor activity	104	2
GO:0051301//cell division	333	9
GO:0031397//negative regulation of protein ubiquitination	64	1
GO:NM_0012	64	1
GO:0034654//nucleobase-containing compound biosynthetic process	627	19
GO:0002474//antigen processing and presentation of peptide antigen via M	105	2
GO:0006807//nitrogen compound metabolic process	3073	111
GO:0043254//regulation of protein complex assembly	176	4
GO:0002824//positive regulation of adaptive immune response based on sc	65	1
GO:0051329//interphase of mitotic cell cycle	179	4
GO:0051656//establishment of organelle localization	145	3
GO:0006457//protein folding	180	4
GO:0002821//positive regulation of adaptive immune response	67	1
GO:0006289//nucleotide-excision repair	67	1
GO:0007338//single fertilization	67	1
GO:0048193//Golgi vesicle transport	146	3
GO:0006732//coenzyme metabolic process	181	4
GO:0051325//interphase	181	4
GO:0000226//microtubule cytoskeleton organization	215	5
GO:0051262//protein tetramerization	69	1
GO:0006886//intracellular protein transport	465	13
GO:0006968//cellular defense response	70	1
GO:0010498//proteasomal protein catabolic process	150	3
GO:0016064//immunoglobulin mediated immune response	113	2
GO:0072594//establishment of protein localization to organelle	113	2
GO:0051168//nuclear export	71	1
GO:0018130//heterocycle biosynthetic process	703	21
GO:0007127//meiosis I	72	1
GO:0070925//organelle assembly	222	5
GO:0019724//B cell mediated immunity	115	2
GO:0002708//positive regulation of lymphocyte mediated immunity	73	1
GO:0030833//regulation of actin filament polymerization	73	1
GO:0051186//cofactor metabolic process	224	5
GO:0007286//spermatid development	74	1
GO:0032649//regulation of interferon-gamma production	74	1
GO:0042108//positive regulation of cytokine biosynthetic process	74	1
GO:0040029//regulation of gene expression, epigenetic	117	2
GO:0002263//cell activation involved in immune response	75	1
GO:0002366//leukocyte activation involved in immune response	75	1

GO:0002705//positive regulation of leukocyte mediated immunity	75	1
GO:0008104//protein localization	1138	36
GO:0043648//dicarboxylic acid metabolic process	76	1
GO:0006413//translational initiation	77	1
GO:0000075//cell cycle checkpoint	196	4
GO:0009165//nucleotide biosynthetic process	160	3
GO:0006261//DNA-dependent DNA replication	78	1
GO:0048515//spermatid differentiation	78	1
GO:0065003//macromolecular complex assembly	866	26
GO:1901293//nucleoside phosphate biosynthetic process	162	3
GO:0006338//chromatin remodeling	80	1
GO:0032259//methylation	237	5
GO:0019438//aromatic compound biosynthetic process	702	20
GO:0000956//nuclear-transcribed mRNA catabolic process	82	1
GO:0002479//antigen processing and presentation of exogenous peptide ar	82	1
GO:0019751//polyol metabolic process	82	1
GO:0007126//meiosis	127	2
GO:1901606//alpha-amino acid catabolic process	83	1
GO:0006323//DNA packaging	169	3
GO:0051321//meiotic cell cycle	131	2
GO:0004930//G-protein coupled receptor activity	86	1
GO:0006479//protein methylation	86	1
GO:0008213//protein alkylation	86	1
GO:0042590//antigen processing and presentation of exogenous peptide ar	86	1
GO:0004888//transmembrane signaling receptor activity	87	1
GO:0044085//cellular component biogenesis	1423	45
GO:0006334//nucleosome assembly	133	2
GO:NM_0011	281	6
GO:0006325//chromatin organization	475	12
GO:0007049//cell cycle	921	27
GO:0004872//receptor activity	89	1
GO:0038023//signaling receptor activity	89	1
GO:0004871//signal transducer activity	90	1
GO:0006402//mRNA catabolic process	90	1
GO:0060089//molecular transducer activity	90	1
GO:0007017//microtubule-based process	353	8
GO:0000084//S phase of mitotic cell cycle	92	1
GO:0022618//ribonucleoprotein complex assembly	92	1
GO:0051320//S phase	92	1
GO:0006281//DNA repair	324	7
GO:0022402//cell cycle process	790	22
GO:0009108//coenzyme biosynthetic process	94	1
GO:0031396//regulation of protein ubiquitination	141	2
GO:0031497//chromatin assembly	142	2
GO:0071826//ribonucleoprotein complex subunit organization	96	1
GO:0046907//intracellular transport	855	24
GO:0043161//proteasomal ubiquitin-dependent protein catabolic process	145	2
GO:0034728//nucleosome organization	148	2
GO:0031398//positive regulation of protein ubiquitination	101	1
GO:0044260//cellular macromolecule metabolic process	4106	144
GO:0065004//protein-DNA complex assembly	149	2
GO:0060271//cilium morphogenesis	104	1
GO:0006302//double-strand break repair	105	1
GO:0006333//chromatin assembly or disassembly	155	2
GO:0030258//lipid modification	108	1
GO:0015931//nucleobase-containing compound transport	109	1
GO:0000077//DNA damage checkpoint	111	1
GO:0000090//mitotic anaphase	111	1

GO:0006401//RNA catabolic process	111	1
GO:0051322//anaphase	113	1
GO:0071103//DNA conformation change	206	3
GO:0006310//DNA recombination	163	2
GO:0008380//RNA splicing	163	2
GO:0045184//establishment of protein localization	868	23
GO:0071824//protein-DNA complex subunit organization	164	2
GO:0019886//antigen processing and presentation of exogenous peptide ar	117	1
GO:0031570//DNA integrity checkpoint	117	1
GO:0002495//antigen processing and presentation of peptide antigen via M	119	1
GO:0015031//protein transport	849	22
GO:0034622//cellular macromolecular complex assembly	435	9
GO:0007059//chromosome segregation	121	1
GO:0048002//antigen processing and presentation of peptide antigen	216	3
GO:0016197//endosomal transport	122	1
GO:0051188//cofactor biosynthetic process	124	1
GO:0002504//antigen processing and presentation of peptide or polysaccha	130	1
GO:0022403//cell cycle phase	316	5
GO:0000278//mitotic cell cycle	498	10
GO:0043414//macromolecule methylation	138	1
GO:0000087//M phase of mitotic cell cycle	139	1
GO:0006399//tRNA metabolic process	143	1
GO:0030031//cell projection assembly	143	1
GO:0002478//antigen processing and presentation of exogenous peptide ar	200	2
GO:0019884//antigen processing and presentation of exogenous antigen	202	2
GO:0007600//sensory perception	826	19
GO:0007186//G-protein coupled receptor signaling pathway	1047	26
GO:0051276//chromosome organization	637	13
GO:1901360//organic cyclic compound metabolic process	2697	81
GO:0000279//M phase	177	1
GO:0006397//mRNA processing	235	2
GO:0006259//DNA metabolic process	708	14
GO:0051606//detection of stimulus	645	12
GO:0006412//translation	341	4
GO:0022613//ribonucleoprotein complex biogenesis	215	1
GO:0016071//mRNA metabolic process	349	3
GO:0034641//cellular nitrogen compound metabolic process	2672	73
GO:0034660//ncRNA metabolic process	275	1
GO:0010467//gene expression	1324	26
GO:0006725//cellular aromatic compound metabolic process	2483	61
GO:0016070//RNA metabolic process	1058	17
GO:0006396//RNA processing	470	3
GO:0046483//heterocycle metabolic process	2465	59
GO:0050906//detection of stimulus involved in sensory perception	503	3
GO:0006139//nucleobase-containing compound metabolic process	2312	52
GO:0009593//detection of chemical stimulus	494	2
GO:0090304//nucleic acid metabolic process	1688	30
GO:0007608//sensory perception of smell	470	1
GO:0007606//sensory perception of chemical stimulus	520	1

Input_gene	Gene_Symbol	P_value	Q_value	Significant
-	-	-	-	-
TC01000904.hg.1	ENSG00000162692;	0	0	TRUE
TC01001805.hg.1	ENSG00000092969;	0	0	TRUE
TC01000728.hg.1	ENSG00000162433;	0	0	TRUE
TC01000904.hg.1	ENSG00000162692;	0	0	TRUE
TC01000394.hg.1	ENSG00000159023;	0	0	TRUE
TC01000728.hg.1	ENSG00000162433;	0	0	TRUE
TC01000904.hg.1	ENSG00000162692;	0	0	TRUE
TC01000394.hg.1	ENSG00000159023;	0	0	TRUE
TC01000728.hg.1	ENSG00000162433;	0	0	TRUE
TC01000728.hg.1	ENSG00000162433;	0	0	TRUE
TC01000728.hg.1	ENSG00000162433;	0	0	TRUE
TC01001188.hg.1;	ENSG00000143369;	0	0	TRUE
TC01000685.hg.1	ENSG00000162409;	0	0	TRUE
TC01000904.hg.1	ENSG00000162692;	0	0	TRUE
TC01000904.hg.1	ENSG00000162692;	0	0	TRUE
TC01000904.hg.1	ENSG00000162692;	0	0	TRUE
TC01001805.hg.1	ENSG00000092969;	1.11E-16	2.716E-14	TRUE
TC01000728.hg.1	ENSG00000162433;	1.11E-16	2.716E-14	TRUE
TC01001188.hg.1;	ENSG00000143369;	3.331E-16	7.505E-14	TRUE
TC01000904.hg.1	ENSG00000162692;	6.661E-16	1.426E-13	TRUE
TC01000904.hg.1	ENSG00000162692;	3.886E-15	7.921E-13	TRUE
TC01003129.hg.1	ENSG00000143140;	9.659E-15	1.88E-12	TRUE
TC01000100.hg.1	ENSG00000049246;	1.343E-14	2.5E-12	TRUE
TC01001704.hg.1	ENSG00000163531;	1.821E-14	3.248E-12	TRUE
TC01002284.hg.1	ENSG00000117122;	1.965E-14	3.365E-12	TRUE
TC01001090.hg.1	ENSG00000117289;	2.62E-14	4.314E-12	TRUE
TC01000685.hg.1	ENSG00000162409;	3.209E-14	5.087E-12	TRUE
TC01000904.hg.1	ENSG00000162692;	2.118E-13	3.239E-11	TRUE
TC01001188.hg.1;	ENSG00000143369;	2.389E-13	3.527E-11	TRUE
TC01000904.hg.1	ENSG00000162692;	3.234E-13	4.435E-11	TRUE
TC01000100.hg.1	ENSG00000049246;	3.256E-13	4.435E-11	TRUE
TC01000100.hg.1	ENSG00000049246;	3.315E-13	4.435E-11	TRUE
TC01000904.hg.1	ENSG00000162692;	3.744E-13	4.782E-11	TRUE
TC01000904.hg.1	ENSG00000162692;	3.798E-13	4.782E-11	TRUE
TC01001805.hg.1	ENSG00000092969;	1.118E-12	1.368E-10	TRUE
TC01000904.hg.1	ENSG00000162692;	1.519E-12	1.807E-10	TRUE
TC01000685.hg.1	ENSG00000162409;	2.272E-12	2.628E-10	TRUE
TC01001805.hg.1	ENSG00000092969;	2.599E-12	2.928E-10	TRUE
TC01000685.hg.1	ENSG00000162409;	5.561E-12	6.069E-10	TRUE
TC01000789.hg.1	ENSG00000162614;	5.67E-12	6.069E-10	TRUE
TC01000100.hg.1	ENSG00000049246;	6.655E-12	6.896E-10	TRUE
TC01000789.hg.1	ENSG00000162614;	6.765E-12	6.896E-10	TRUE
TC01000789.hg.1	ENSG00000162614;	1.115E-11	1.11E-09	TRUE
TC01000685.hg.1	ENSG00000162409;	1.147E-11	1.116E-09	TRUE
TC01000685.hg.1	ENSG00000162409;	1.374E-11	1.307E-09	TRUE
TC01001805.hg.1	ENSG00000092969;	1.872E-11	1.736E-09	TRUE
TC01000685.hg.1	ENSG00000162409;	1.906E-11	1.736E-09	TRUE
TC01001805.hg.1	ENSG00000092969;	2.112E-11	1.883E-09	TRUE
TC01000904.hg.1	ENSG00000162692;	4.274E-11	3.734E-09	TRUE
TC01000789.hg.1	ENSG00000162614;	4.435E-11	3.797E-09	TRUE
TC01000685.hg.1	ENSG00000162409;	5.498E-11	4.615E-09	TRUE
TC01001188.hg.1;	ENSG00000143369;	6.07E-11	4.916E-09	TRUE
TC01002954.hg.1	ENSG00000134243;	6.086E-11	4.916E-09	TRUE
TC01000685.hg.1	ENSG00000162409;	1.003E-10	7.955E-09	TRUE
TC01001188.hg.1;	ENSG00000143369;	1.176E-10	9.151E-09	TRUE
TC01000728.hg.1	ENSG00000162433;	1.314E-10	1.005E-08	TRUE

TC01000685.hg.1	ENSG00000162409;	1.99E-10	1.494E-08	TRUE
TC01000904.hg.1	ENSG00000162692;	2.296E-10	1.695E-08	TRUE
TC01000685.hg.1	ENSG00000162409;	2.346E-10	1.697E-08	TRUE
TC01000685.hg.1	ENSG00000162409;	2.379E-10	1.697E-08	TRUE
TC01003129.hg.1	ENSG00000143140;	2.505E-10	1.733E-08	TRUE
TC01003638.hg.1	ENSG00000073756;	2.51E-10	1.733E-08	TRUE
TC01000685.hg.1	ENSG00000162409;	2.686E-10	1.825E-08	TRUE
TC01003638.hg.1	ENSG00000073756;	3.202E-10	2.126E-08	TRUE
TC01003638.hg.1	ENSG00000073756;	3.202E-10	2.126E-08	TRUE
TC01001805.hg.1	ENSG00000092969;	3.642E-10	2.362E-08	TRUE
TC01000904.hg.1	ENSG00000162692;	5.307E-10	3.391E-08	TRUE
TC01000394.hg.1	ENSG00000159023;	5.495E-10	3.459E-08	TRUE
TC01000734.hg.1	ENSG00000118473;	5.632E-10	3.495E-08	TRUE
TC01001805.hg.1	ENSG00000092969;	6.362E-10	3.891E-08	TRUE
TC01000394.hg.1	ENSG00000159023;	6.863E-10	4.138E-08	TRUE
TC01001805.hg.1	ENSG00000092969;	7.283E-10	4.33E-08	TRUE
TC01001736.hg.1	ENSG00000196352;	8.147E-10	4.759E-08	TRUE
TC01000139.hg.1	ENSG00000171819;	8.226E-10	4.759E-08	TRUE
TC01000685.hg.1	ENSG00000162409;	1.008E-09	5.753E-08	TRUE
TC01000685.hg.1	ENSG00000162409;	1.287E-09	7.249E-08	TRUE
TC01000904.hg.1	ENSG00000162692;	1.378E-09	7.662E-08	TRUE
TC01000685.hg.1	ENSG00000162409;	2.529E-09	1.388E-07	TRUE
TC01001736.hg.1	ENSG00000196352;	2.651E-09	1.436E-07	TRUE
TC01000685.hg.1	ENSG00000162409;	2.879E-09	1.541E-07	TRUE
TC01001704.hg.1	ENSG00000163531;	3.142E-09	1.66E-07	TRUE
TC01000904.hg.1	ENSG00000162692;	4.118E-09	2.15E-07	TRUE
TC01003129.hg.1	ENSG00000143140;	4.365E-09	2.238E-07	TRUE
TC01003129.hg.1	ENSG00000143140;	4.365E-09	2.238E-07	TRUE
TC01000685.hg.1	ENSG00000162409;	6.152E-09	3.098E-07	TRUE
TC01000904.hg.1	ENSG00000162692;	6.951E-09	3.44E-07	TRUE
TC01000904.hg.1	ENSG00000162692;	6.951E-09	3.44E-07	TRUE
TC01001805.hg.1	ENSG00000092969;	7.834E-09	3.811E-07	TRUE
TC01000685.hg.1	ENSG00000162409;	9.602E-09	4.619E-07	TRUE
TC01001805.hg.1	ENSG00000092969;	1.063E-08	5.054E-07	TRUE
TC01000685.hg.1	ENSG00000162409;	1.09E-08	5.13E-07	TRUE
TC01000685.hg.1	ENSG00000162409;	1.255E-08	5.823E-07	TRUE
TC01002706.hg.1	ENSG00000184292;	1.265E-08	5.823E-07	TRUE
TC01001624.hg.1	ENSG00000116741;	1.47E-08	6.625E-07	TRUE
TC01000685.hg.1	ENSG00000162409;	1.47E-08	6.625E-07	TRUE
TC01001704.hg.1	ENSG00000163531;	1.68E-08	7.493E-07	TRUE
TC01001090.hg.1	ENSG00000117289;	2.412E-08	1.064E-06	TRUE
TC01000685.hg.1	ENSG00000162409;	2.491E-08	1.088E-06	TRUE
TC01003638.hg.1	ENSG00000073756;	2.58E-08	1.116E-06	TRUE
TC01001704.hg.1	ENSG00000163531;	2.82E-08	1.201E-06	TRUE
TC01001704.hg.1	ENSG00000163531;	2.82E-08	1.201E-06	TRUE
TC01000685.hg.1	ENSG00000162409;	3.25E-08	1.364E-06	TRUE
TC01000685.hg.1	ENSG00000162409;	4.371E-08	1.817E-06	TRUE
TC05003423.hg.1	ENSG00000082196;	5.241E-08	2.158E-06	TRUE
TC01001188.hg.1;	ENSG00000143369;	6.684E-08	2.725E-06	TRUE
TC01001624.hg.1	ENSG00000116741;	7.704E-08	3.111E-06	TRUE
TC01000685.hg.1	ENSG00000162409;	8.017E-08	3.202E-06	TRUE
TC02000041.hg.1	ENSG00000115738;	8.078E-08	3.202E-06	TRUE
TC01001704.hg.1	ENSG00000163531;	8.242E-08	3.237E-06	TRUE
TC01001624.hg.1	ENSG00000116741;	8.713E-08	3.391E-06	TRUE
TC01000685.hg.1	ENSG00000162409;	9.232E-08	3.561E-06	TRUE
TC01001188.hg.1;	ENSG00000143369;	9.46E-08	3.616E-06	TRUE
TC01001188.hg.1;	ENSG00000143369;	9.903E-08	3.752E-06	TRUE
TC01000904.hg.1	ENSG00000162692;	1.06E-07	3.98E-06	TRUE

TC01003638.hg.1	ENSG00000073756;	1.069E-07	3.98E-06	TRUE
TC02000041.hg.1	ENSG00000115738;	1.093E-07	4.034E-06	TRUE
TC01001188.hg.1;	ENSG00000143369;	1.139E-07	4.167E-06	TRUE
TC01001624.hg.1	ENSG00000116741;	1.488E-07	5.399E-06	TRUE
TC01000904.hg.1	ENSG00000162692;	1.522E-07	5.475E-06	TRUE
TC05003423.hg.1	ENSG00000082196;	1.671E-07	5.961E-06	TRUE
TC01001704.hg.1	ENSG00000163531;	1.883E-07	6.663E-06	TRUE
TC02001867.hg.1	ENSG00000115380;	2.057E-07	7.218E-06	TRUE
TC01001805.hg.1	ENSG00000092969;	2.119E-07	7.376E-06	TRUE
TC06004077.hg.1	ENSG00000243649;	2.197E-07	7.584E-06	TRUE
TC01000685.hg.1	ENSG00000162409;	2.245E-07	7.688E-06	TRUE
TC01001188.hg.1;	ENSG00000143369;	2.345E-07	7.969E-06	TRUE
TC02000041.hg.1	ENSG00000115738;	2.387E-07	8.046E-06	TRUE
TC06001510.hg.1	ENSG00000137331;	2.481E-07	8.264E-06	TRUE
TC02001750.hg.1	ENSG00000138061;	2.481E-07	8.264E-06	TRUE
TC02000219.hg.1	ENSG00000049323;	2.57E-07	8.463E-06	TRUE
TC01001188.hg.1;	ENSG00000143369;	2.939E-07	9.605E-06	TRUE
TC01001805.hg.1	ENSG00000092969;	3.141E-07	1.019E-05	TRUE
TC01000904.hg.1	ENSG00000162692;	3.193E-07	1.028E-05	TRUE
TC01000685.hg.1	ENSG00000162409;	3.975E-07	1.27E-05	TRUE
TC01001188.hg.1;	ENSG00000143369;	4.035E-07	1.28E-05	TRUE
TC01000685.hg.1	ENSG00000162409;	4.189E-07	1.319E-05	TRUE
TC01000685.hg.1	ENSG00000162409;	4.355E-07	1.361E-05	TRUE
TC01000685.hg.1	ENSG00000162409;	6.824E-07	2.117E-05	TRUE
TC01000100.hg.1	ENSG00000049246;	7.124E-07	2.194E-05	TRUE
TC01001624.hg.1	ENSG00000116741;	7.395E-07	2.256E-05	TRUE
TC01001473.hg.1	ENSG00000143153;	7.429E-07	2.256E-05	TRUE
TC01001624.hg.1	ENSG00000116741;	7.936E-07	2.392E-05	TRUE
TC01001090.hg.1	ENSG00000117289;	8.106E-07	2.418E-05	TRUE
TC06001510.hg.1	ENSG00000137331;	8.106E-07	2.418E-05	TRUE
TC01001805.hg.1	ENSG00000092969;	8.383E-07	2.475E-05	TRUE
TC01001805.hg.1	ENSG00000092969;	8.691E-07	2.548E-05	TRUE
TC01001704.hg.1	ENSG00000163531;	9.098E-07	2.649E-05	TRUE
TC03000577.hg.1	ENSG00000144857;	9.387E-07	2.715E-05	TRUE
TC01002166.hg.1	ENSG00000116285;	9.805E-07	2.786E-05	TRUE
TC01001704.hg.1	ENSG00000163531;	9.816E-07	2.786E-05	TRUE
TC01001704.hg.1	ENSG00000163531;	9.816E-07	2.786E-05	TRUE
TC01000904.hg.1	ENSG00000162692;	9.891E-07	2.786E-05	TRUE
TC01003129.hg.1	ENSG00000143140;	1.003E-06	2.796E-05	TRUE
TC01003129.hg.1	ENSG00000143140;	1.003E-06	2.796E-05	TRUE
TC01001704.hg.1	ENSG00000163531;	1.073E-06	2.964E-05	TRUE
TC03000577.hg.1	ENSG00000144857;	1.294E-06	3.55E-05	TRUE
TC01001736.hg.1	ENSG00000196352;	1.309E-06	3.569E-05	TRUE
TC01001805.hg.1	ENSG00000092969;	1.358E-06	3.678E-05	TRUE
TC01000904.hg.1	ENSG00000162692;	1.385E-06	3.728E-05	TRUE
TC01001188.hg.1;	ENSG00000143369;	1.409E-06	3.771E-05	TRUE
TC01001805.hg.1	ENSG00000092969;	1.438E-06	3.812E-05	TRUE
TC01001805.hg.1	ENSG00000092969;	1.438E-06	3.812E-05	TRUE
TC02002605.hg.1	ENSG00000064989;	1.452E-06	3.815E-05	TRUE
TC01001704.hg.1	ENSG00000163531;	1.555E-06	4.059E-05	TRUE
TC01000734.hg.1	ENSG00000118473;	1.579E-06	4.095E-05	TRUE
TC01001805.hg.1	ENSG00000092969;	1.633E-06	4.212E-05	TRUE
TC01003638.hg.1	ENSG00000073756;	1.695E-06	4.344E-05	TRUE
TC01001704.hg.1	ENSG00000163531;	1.733E-06	4.416E-05	TRUE
TC01000685.hg.1	ENSG00000162409;	1.906E-06	4.828E-05	TRUE
TC01001188.hg.1;	ENSG00000143369;	1.949E-06	4.851E-05	TRUE
TC01001736.hg.1	ENSG00000196352;	1.957E-06	4.851E-05	TRUE
TC01001736.hg.1	ENSG00000196352;	1.957E-06	4.851E-05	TRUE

TC01000643.hg.1	ENSG00000116157;	1.96E-06	4.851E-05	TRUE
TC01001805.hg.1	ENSG00000092969;	2.038E-06	5.015E-05	TRUE
TC01000904.hg.1	ENSG00000162692;	2.158E-06	5.261E-05	TRUE
TC01000685.hg.1	ENSG00000162409;	2.163E-06	5.261E-05	TRUE
TC01000685.hg.1	ENSG00000162409;	2.482E-06	6.002E-05	TRUE
TC01000685.hg.1	ENSG00000162409;	2.686E-06	6.461E-05	TRUE
TC01001805.hg.1	ENSG00000092969;	2.815E-06	6.713E-05	TRUE
TC01001805.hg.1	ENSG00000092969;	2.815E-06	6.713E-05	TRUE
TC01001704.hg.1	ENSG00000163531;	2.868E-06	6.783E-05	TRUE
TC01000685.hg.1	ENSG00000162409;	3.003E-06	7.065E-05	TRUE
TC03000214.hg.1	ENSG00000168036;	3.077E-06	7.198E-05	TRUE
TC01001090.hg.1	ENSG00000117289;	3.178E-06	7.394E-05	TRUE
TC01001805.hg.1	ENSG00000092969;	3.199E-06	7.403E-05	TRUE
TC01001805.hg.1	ENSG00000092969;	3.26E-06	7.503E-05	TRUE
TC03000214.hg.1	ENSG00000168036;	3.311E-06	7.58E-05	TRUE
TC01001188.hg.1;	ENSG00000143369;	3.388E-06	7.715E-05	TRUE
TC02000762.hg.1	ENSG00000163083;	3.555E-06	8.03E-05	TRUE
TC02000762.hg.1	ENSG00000163083;	3.555E-06	8.03E-05	TRUE
TC01001736.hg.1	ENSG00000196352;	3.609E-06	8.055E-05	TRUE
TC01001704.hg.1	ENSG00000163531;	3.613E-06	8.055E-05	TRUE
TC01001704.hg.1	ENSG00000163531;	3.992E-06	8.799E-05	TRUE
TC01001704.hg.1	ENSG00000163531;	3.997E-06	8.799E-05	TRUE
TC01001805.hg.1	ENSG00000092969;	4.008E-06	8.799E-05	TRUE
TC01000685.hg.1	ENSG00000162409;	4.214E-06	9.127E-05	TRUE
TC02001867.hg.1	ENSG00000115380;	4.214E-06	9.127E-05	TRUE
TC01000734.hg.1	ENSG00000118473;	4.228E-06	9.127E-05	TRUE
TC01000685.hg.1	ENSG00000162409;	4.242E-06	9.127E-05	TRUE
TC03000577.hg.1	ENSG00000144857;	4.349E-06	9.285E-05	TRUE
TC03000577.hg.1	ENSG00000144857;	4.349E-06	9.285E-05	TRUE
TC01001805.hg.1	ENSG00000092969;	4.557E-06	9.657E-05	TRUE
TC02000041.hg.1	ENSG00000115738;	5.26E-06	0.0001109	TRUE
TC01000734.hg.1	ENSG00000118473;	5.53E-06	0.0001161	TRUE
TC06001510.hg.1	ENSG00000137331;	5.879E-06	0.0001228	TRUE
TC01000734.hg.1	ENSG00000118473;	6.671E-06	0.0001386	TRUE
TC02002605.hg.1	ENSG00000064989;	6.773E-06	0.0001401	TRUE
TC01000904.hg.1	ENSG00000162692;	6.883E-06	0.0001417	TRUE
TC01001805.hg.1	ENSG00000092969;	6.917E-06	0.0001417	TRUE
TC01000685.hg.1	ENSG00000162409;	6.966E-06	0.000142	TRUE
TC01001805.hg.1	ENSG00000092969;	7.268E-06	0.0001471	TRUE
TC01001805.hg.1	ENSG00000092969;	7.268E-06	0.0001471	TRUE
TC02000082.hg.1	ENSG00000071575;	7.798E-06	0.0001567	TRUE
TC01002166.hg.1	ENSG00000116285;	8.253E-06	0.0001651	TRUE
TC01000685.hg.1	ENSG00000162409;	9.141E-06	0.000182	TRUE
TC01000100.hg.1	ENSG00000049246;	9.68E-06	0.0001919	TRUE
TC01001805.hg.1	ENSG00000092969;	9.78E-06	0.0001929	TRUE
TC01002166.hg.1	ENSG00000116285;	1.018E-05	0.0002	TRUE
TC01001805.hg.1	ENSG00000092969;	1.07E-05	0.0002092	TRUE
TC01001805.hg.1	ENSG00000092969;	1.206E-05	0.0002347	TRUE
TC01003638.hg.1	ENSG00000073756;	1.242E-05	0.0002406	TRUE
TC05000943.hg.1	ENSG00000156427;	1.263E-05	0.0002431	TRUE
TC05000943.hg.1	ENSG00000156427;	1.263E-05	0.0002431	TRUE
TC01001805.hg.1	ENSG00000092969;	1.311E-05	0.0002505	TRUE
TC01001805.hg.1	ENSG00000092969;	1.348E-05	0.0002565	TRUE
TC01000972.hg.1	ENSG00000064886;	1.527E-05	0.0002893	TRUE
TC01002932.hg.1	ENSG00000060718;	1.559E-05	0.0002934	TRUE
TC01001805.hg.1	ENSG00000092969;	1.559E-05	0.0002934	TRUE
TC01000685.hg.1	ENSG00000162409;	1.594E-05	0.000298	TRUE
TC01003129.hg.1	ENSG00000143140;	1.636E-05	0.0003039	TRUE

TC01001805.hg.1	ENSG00000092969;	1.636E-05	0.0003039	TRUE
TC01000685.hg.1	ENSG00000162409;	1.877E-05	0.0003464	TRUE
TC01001805.hg.1	ENSG00000092969;	1.904E-05	0.0003498	TRUE
TC03000846.hg.1	ENSG00000196549;	1.959E-05	0.0003584	TRUE
TC01001736.hg.1	ENSG00000196352;	1.988E-05	0.0003621	TRUE
TC01001624.hg.1	ENSG00000116741;	2.009E-05	0.0003637	TRUE
TC01001624.hg.1	ENSG00000116741;	2.009E-05	0.0003637	TRUE
TC01001188.hg.1;	ENSG00000143369;	2.204E-05	0.0003957	TRUE
TC01001805.hg.1	ENSG00000092969;	2.204E-05	0.0003957	TRUE
TC01002954.hg.1	ENSG00000134243;	2.258E-05	0.0004028	TRUE
TC01001090.hg.1	ENSG00000117289;	2.276E-05	0.0004042	TRUE
TC01001624.hg.1	ENSG00000116741;	2.285E-05	0.0004042	TRUE
TC01000728.hg.1	ENSG00000162433;	2.398E-05	0.0004225	TRUE
TC01000685.hg.1	ENSG00000162409;	2.475E-05	0.0004334	TRUE
TC01000685.hg.1	ENSG00000162409;	2.475E-05	0.0004334	TRUE
TC01000685.hg.1	ENSG00000162409;	2.516E-05	0.0004373	TRUE
TC01001805.hg.1	ENSG00000092969;	2.523E-05	0.0004373	TRUE
TC01001624.hg.1	ENSG00000116741;	2.562E-05	0.0004423	TRUE
TC02000219.hg.1	ENSG00000049323;	2.641E-05	0.0004541	TRUE
TC01000100.hg.1	ENSG00000049246;	2.794E-05	0.0004785	TRUE
TC01002166.hg.1	ENSG00000116285;	2.827E-05	0.0004821	TRUE
TC01000100.hg.1	ENSG00000049246;	2.989E-05	0.0005078	TRUE
TC01001805.hg.1	ENSG00000092969;	3.118E-05	0.0005275	TRUE
TC05000943.hg.1	ENSG00000156427;	3.184E-05	0.0005366	TRUE
TC01000904.hg.1	ENSG00000162692;	3.198E-05	0.0005368	TRUE
TC01001805.hg.1	ENSG00000092969;	3.258E-05	0.0005448	TRUE
TC05000943.hg.1	ENSG00000156427;	3.28E-05	0.0005463	TRUE
TC01001805.hg.1	ENSG00000092969;	3.38E-05	0.0005608	TRUE
TC01003638.hg.1	ENSG00000073756;	3.571E-05	0.0005898	TRUE
TC02000219.hg.1	ENSG00000049323;	3.582E-05	0.0005898	TRUE
TC01001805.hg.1	ENSG00000092969;	3.661E-05	0.0006004	TRUE
TC01000100.hg.1	ENSG00000049246;	3.702E-05	0.0006049	TRUE
TC01002578.hg.1	ENSG00000117394;	3.752E-05	0.0006108	TRUE
TC01000904.hg.1	ENSG00000162692;	3.78E-05	0.0006129	TRUE
TC01003211.hg.1;	ENSG00000143387;	3.818E-05	0.0006133	TRUE
TC06004073.hg.1	ENSG00000204520;	3.818E-05	0.0006133	TRUE
TC01003211.hg.1;	ENSG00000143387;	3.818E-05	0.0006133	TRUE
TC03001255.hg.1	ENSG00000033867;	3.818E-05	0.0006133	TRUE
TC01001736.hg.1	ENSG00000196352;	3.885E-05	0.0006182	TRUE
TC02000082.hg.1	ENSG00000071575;	3.92E-05	0.0006203	TRUE
TC01002166.hg.1	ENSG00000116285;	3.92E-05	0.0006203	TRUE
TC01001805.hg.1	ENSG00000092969;	4.05E-05	0.0006363	TRUE
TC05000943.hg.1	ENSG00000156427;	4.05E-05	0.0006363	TRUE
TC01001805.hg.1	ENSG00000092969;	4.106E-05	0.0006404	TRUE
TC01001805.hg.1	ENSG00000092969;	4.114E-05	0.0006404	TRUE
TC01000685.hg.1	ENSG00000162409;	4.284E-05	0.0006644	TRUE
TC01001624.hg.1	ENSG00000116741;	4.463E-05	0.0006898	TRUE
TC01001736.hg.1	ENSG00000196352;	4.727E-05	0.0007279	TRUE
TC01002932.hg.1	ENSG00000060718;	4.852E-05	0.0007446	TRUE
TC01000100.hg.1	ENSG00000049246;	5.007E-05	0.0007652	TRUE
TC01001090.hg.1	ENSG00000117289;	5.022E-05	0.0007652	TRUE
TC01000904.hg.1	ENSG00000162692;	5.113E-05	0.0007763	TRUE
TC01001736.hg.1	ENSG00000196352;	5.316E-05	0.0008042	TRUE
TC01001805.hg.1	ENSG00000092969;	5.483E-05	0.0008264	TRUE
TC01001805.hg.1	ENSG00000092969;	5.527E-05	0.0008275	TRUE
TC01000734.hg.1	ENSG00000118473;	5.528E-05	0.0008275	TRUE
TC01001624.hg.1	ENSG00000116741;	5.676E-05	0.0008467	TRUE
TC01000100.hg.1	ENSG00000049246;	5.865E-05	0.0008718	TRUE

TC02001750.hg.1	ENSG00000138061;	5.934E-05	0.000879	TRUE
TC01001090.hg.1	ENSG00000117289;	6.334E-05	0.0009327	TRUE
TC01001805.hg.1	ENSG00000092969;	6.357E-05	0.0009327	TRUE
TC01000100.hg.1	ENSG00000049246;	6.387E-05	0.0009327	TRUE
TC01001805.hg.1	ENSG00000092969;	6.394E-05	0.0009327	TRUE
TC01003638.hg.1	ENSG00000073756;	6.394E-05	0.0009327	TRUE
TC03000577.hg.1	ENSG00000144857;	6.483E-05	0.0009384	TRUE
TC05001933.hg.1	ENSG00000113721;	6.483E-05	0.0009384	TRUE
TC01001736.hg.1	ENSG00000196352;	6.51E-05	0.0009384	TRUE
TC01001805.hg.1	ENSG00000092969;	6.642E-05	0.0009542	TRUE
TC01000685.hg.1	ENSG00000162409;	6.759E-05	0.0009677	TRUE
TC05000943.hg.1	ENSG00000156427;	6.858E-05	0.000978	TRUE
TC01003129.hg.1	ENSG00000143140;	6.894E-05	0.000978	TRUE
TC01002932.hg.1	ENSG00000060718;	6.911E-05	0.000978	TRUE
TC01001188.hg.1;	ENSG00000143369;	6.911E-05	0.000978	TRUE
TC02000466.hg.1	ENSG00000159399;	7.609E-05	0.0010715	TRUE
TC01001805.hg.1	ENSG00000092969;	7.795E-05	0.0010917	TRUE
TC03001071.hg.1	ENSG00000114315;	7.804E-05	0.0010917	TRUE
TC01001624.hg.1	ENSG00000116741;	7.898E-05	0.0011013	TRUE
TC01000394.hg.1	ENSG00000159023;	8.32E-05	0.0011565	TRUE
TC02002292.hg.1	ENSG00000136720;	8.407E-05	0.0011647	TRUE
TC01000100.hg.1	ENSG00000049246;	8.526E-05	0.0011726	TRUE
TC01001473.hg.1	ENSG00000143153;	8.533E-05	0.0011726	TRUE
TC01001473.hg.1	ENSG00000143153;	8.533E-05	0.0011726	TRUE
TC01003778.hg.1	ENSG00000076356;	8.621E-05	0.0011769	TRUE
TC01002932.hg.1	ENSG00000060718;	8.632E-05	0.0011769	TRUE
TC01001624.hg.1	ENSG00000116741;	9.736E-05	0.0013231	TRUE
TC01000904.hg.1	ENSG00000162692;	9.766E-05	0.0013231	TRUE
TC01001090.hg.1	ENSG00000117289;	9.862E-05	0.0013282	TRUE
TC01001473.hg.1	ENSG00000143153;	9.887E-05	0.0013282	TRUE
TC01001805.hg.1	ENSG00000092969;	9.897E-05	0.0013282	TRUE
TC01003778.hg.1	ENSG00000076356;	0.0001016	0.0013586	TRUE
TC01001188.hg.1;	ENSG00000143369;	0.0001036	0.001382	TRUE
TC02002605.hg.1	ENSG00000064989;	0.000108	0.0014362	TRUE
TC01003778.hg.1	ENSG00000076356;	0.0001131	0.0014954	TRUE
TC02000762.hg.1	ENSG00000163083;	0.0001132	0.0014954	TRUE
TC03000214.hg.1	ENSG00000168036;	0.0001137	0.0014972	TRUE
TC01000643.hg.1	ENSG00000116157;	0.0001151	0.0015116	TRUE
TC01003638.hg.1	ENSG00000073756;	0.000117	0.0015319	TRUE
TC01001188.hg.1;	ENSG00000143369;	0.0001189	0.0015523	TRUE
TC01001090.hg.1	ENSG00000117289;	0.0001202	0.0015645	TRUE
TC01000904.hg.1	ENSG00000162692;	0.0001212	0.0015726	TRUE
TC01003042.hg.1	ENSG00000092607;	0.0001276	0.0016506	TRUE
TC01002954.hg.1	ENSG00000134243;	0.000129	0.0016638	TRUE
TC01003638.hg.1	ENSG00000073756;	0.0001316	0.0016913	TRUE
TC01001805.hg.1	ENSG00000092969;	0.0001382	0.0017667	TRUE
TC01001090.hg.1	ENSG00000117289;	0.0001382	0.0017667	TRUE
TC01001607.hg.1	ENSG00000116711;l	0.0001416	0.0018036	TRUE
TC04001084.hg.1	ENSG00000109819;	0.0001426	0.0018114	TRUE
TC01002706.hg.1	ENSG00000184292;	0.0001435	0.001817	TRUE
TC01002932.hg.1	ENSG00000060718;	0.000144	0.0018179	TRUE
TC02000245.hg.1	ENSG00000143891;	0.0001476	0.001858	TRUE
TC01001704.hg.1	ENSG00000163531;	0.0001526	0.0019155	TRUE
TC01003638.hg.1	ENSG00000073756;	0.0001563	0.0019564	TRUE
TC01003211.hg.1;	ENSG00000143387;	0.0001607	0.0020054	TRUE
TC05001933.hg.1	ENSG00000113721;	0.000162	0.0020156	TRUE
TC01003129.hg.1	ENSG00000143140;	0.0001667	0.0020585	TRUE
TC06004073.hg.1	ENSG00000204520;	0.0001669	0.0020585	TRUE

TC05001933.hg.1	ENSG00000113721;	0.0001669	0.0020585	TRUE
TC16000474.hg.1	ENSG00000205358;	0.0001669	0.0020585	TRUE
TC01001090.hg.1	ENSG00000117289;	0.0001695	0.0020789	TRUE
TC01000394.hg.1	ENSG00000159023;	0.0001749	0.0021396	TRUE
TC01001805.hg.1	ENSG00000092969;	0.0001777	0.0021672	TRUE
TC01003638.hg.1	ENSG00000073756;	0.00018	0.0021893	TRUE
TC01000685.hg.1	ENSG00000162409;	0.0001884	0.002283	TRUE
TC01002847.hg.1	ENSG00000117228;	0.0001898	0.002283	TRUE
TC01000100.hg.1	ENSG00000049246;	0.0001901	0.002283	TRUE
TC01000100.hg.1	ENSG00000049246;	0.0001901	0.002283	TRUE
TC03000577.hg.1	ENSG00000144857;	0.0001904	0.002283	TRUE
TC01003638.hg.1	ENSG00000073756;	0.0002003	0.0023887	TRUE
TC06001510.hg.1	ENSG00000137331;	0.0002003	0.0023887	TRUE
TC02002823.hg.1	ENSG00000135919;	0.0002003	0.0023887	TRUE
TC01001805.hg.1	ENSG00000092969;	0.0002032	0.0024101	TRUE
TC02000466.hg.1	ENSG00000159399;	0.0002093	0.0024713	TRUE
TC01001805.hg.1	ENSG00000092969;	0.0002093	0.0024713	TRUE
TC01000734.hg.1	ENSG00000118473;	0.0002114	0.0024864	TRUE
TC01001090.hg.1	ENSG00000117289;	0.0002129	0.0024967	TRUE
TC01001805.hg.1	ENSG00000092969;	0.0002163	0.0025287	TRUE
TC01002706.hg.1	ENSG00000184292;	0.0002168	0.0025287	TRUE
TC01000139.hg.1	ENSG00000171819;	0.0002206	0.0025666	TRUE
TC01000904.hg.1	ENSG00000162692;	0.0002213	0.0025673	TRUE
TC01000685.hg.1	ENSG00000162409;	0.0002345	0.0027061	TRUE
TC03000248.hg.1	ENSG00000144791;	0.0002348	0.0027061	TRUE
TC03001071.hg.1	ENSG00000114315;	0.0002348	0.0027061	TRUE
TC01002669.hg.1	ENSG00000157193;	0.0002378	0.0027292	TRUE
TC01001736.hg.1	ENSG00000196352;	0.0002407	0.0027519	TRUE
TC02001031.hg.1	ENSG00000152256;	0.0002407	0.0027519	TRUE
TC01001805.hg.1	ENSG00000092969;	0.0002475	0.0028179	TRUE
TC02000041.hg.1	ENSG00000115738;	0.0002494	0.002832	TRUE
TC01001704.hg.1	ENSG00000163531;	0.0002512	0.0028355	TRUE
TC01003129.hg.1	ENSG00000143140;	0.0002514	0.0028355	TRUE
TC01000904.hg.1	ENSG00000162692;	0.0002514	0.0028355	TRUE
TC01003042.hg.1	ENSG00000092607;	0.0002563	0.0028797	TRUE
TC01001188.hg.1;	ENSG00000143369;	0.0002579	0.002887	TRUE
TC01003718.hg.1	ENSG00000133048;	0.0002579	0.002887	TRUE
TC02001031.hg.1	ENSG00000152256;	0.0002621	0.0029224	TRUE
TC03000846.hg.1	ENSG00000196549;	0.0002775	0.0030858	TRUE
TC01002932.hg.1	ENSG00000060718;	0.0002795	0.0030999	TRUE
TC03000846.hg.1	ENSG00000196549;	0.0002817	0.0031163	TRUE
TC01002706.hg.1	ENSG00000184292;	0.0002851	0.0031382	TRUE
TC02001031.hg.1	ENSG00000152256;	0.0002852	0.0031382	TRUE
TC01000728.hg.1	ENSG00000162433;	0.0002884	0.003166	TRUE
TC01000685.hg.1	ENSG00000162409;	0.0002985	0.0032644	TRUE
TC02001750.hg.1	ENSG00000138061;	0.0002985	0.0032644	TRUE
TC03000214.hg.1	ENSG00000168036;	0.0003002	0.0032701	TRUE
TC02000219.hg.1	ENSG00000049323;	0.0003031	0.0032862	TRUE
TC01001805.hg.1	ENSG00000092969;	0.0003032	0.0032862	TRUE
TC02001031.hg.1	ENSG00000152256;	0.0003099	0.0033459	TRUE
TC02000041.hg.1	ENSG00000115738;	0.0003103	0.0033459	TRUE
TC03000248.hg.1	ENSG00000144791;	0.0003117	0.0033523	TRUE
TC01000734.hg.1	ENSG00000118473;	0.0003187	0.003419	TRUE
TC01001805.hg.1	ENSG00000092969;	0.0003197	0.0034215	TRUE
TC01000685.hg.1	ENSG00000162409;	0.0003245	0.0034638	TRUE
TC03001071.hg.1	ENSG00000114315;	0.0003302	0.003495	TRUE
TC03001071.hg.1	ENSG00000114315;	0.0003302	0.003495	TRUE
TC02000762.hg.1	ENSG00000163083;	0.0003302	0.003495	TRUE

TC01001805.hg.1	ENSG00000092969;	0.0003302	0.003495	TRUE
TC01001624.hg.1	ENSG00000116741;	0.0003347	0.0035294	TRUE
TC01000783.hg.1	ENSG00000117069;	0.0003374	0.0035488	TRUE
TC01003638.hg.1	ENSG00000073756;	0.0003395	0.003562	TRUE
TC01000685.hg.1	ENSG00000162409;	0.0003438	0.0035906	TRUE
TC01003042.hg.1	ENSG00000092607;	0.0003439	0.0035906	TRUE
TC01001473.hg.1	ENSG00000143153;	0.0003463	0.0036074	TRUE
TC01000904.hg.1	ENSG00000162692;	0.0003545	0.0036835	TRUE
TC01001624.hg.1	ENSG00000116741;	0.0003654	0.0037875	TRUE
TC01001805.hg.1	ENSG00000092969;	0.0003665	0.0037901	TRUE
TC01003638.hg.1	ENSG00000073756;	0.000405	0.0041626	TRUE
TC01003638.hg.1	ENSG00000073756;	0.000405	0.0041626	TRUE
TC05001933.hg.1	ENSG00000113721;	0.000405	0.0041626	TRUE
TC01001090.hg.1	ENSG00000117289;	0.000405	0.0041626	TRUE
TC01001805.hg.1	ENSG00000092969;	0.0004091	0.0041795	TRUE
TC01002706.hg.1	ENSG00000184292;	0.0004204	0.004285	TRUE
TC03000214.hg.1	ENSG00000168036;	0.0004447	0.0045168	TRUE
TC02000620.hg.1	ENSG00000115594;	0.0004447	0.0045168	TRUE
TC01000685.hg.1	ENSG00000162409;	0.0004483	0.0045268	TRUE
TC01001090.hg.1	ENSG00000117289;	0.0004508	0.0045268	TRUE
TC02000466.hg.1	ENSG00000159399;	0.0004519	0.0045268	TRUE
TC02001100.hg.1;	ENSG00000138448;	0.0004522	0.0045268	TRUE
TC07000137.hg.1	ENSG00000136244;	0.0004522	0.0045268	TRUE
TC01003638.hg.1	ENSG00000073756;	0.0004531	0.0045268	TRUE
TC03000262.hg.1	ENSG00000160801;	0.0004531	0.0045268	TRUE
TC01001805.hg.1	ENSG00000092969;	0.0004557	0.0045373	TRUE
TC01001805.hg.1	ENSG00000092969;	0.0004627	0.0045958	TRUE
TC01000728.hg.1	ENSG00000162433;	0.0004689	0.004641	TRUE
TC01000685.hg.1	ENSG00000162409;	0.0004689	0.004641	TRUE
TC03000214.hg.1	ENSG00000168036;	0.0004728	0.0046586	TRUE
TC01002954.hg.1	ENSG00000134243;	0.0004728	0.0046586	TRUE
TC01002669.hg.1	ENSG00000157193;	0.0004778	0.0046872	TRUE
TC01002706.hg.1	ENSG00000184292;	0.0004785	0.0046872	TRUE
TC01000685.hg.1	ENSG00000162409;	0.0004841	0.0047313	TRUE
TC01000904.hg.1	ENSG00000162692;	0.0004881	0.0047595	TRUE
TC01002954.hg.1	ENSG00000134243;	0.0005085	0.0049262	TRUE
TC06000595.hg.1	ENSG00000112655;	0.0005085	0.0049262	TRUE
TC01001805.hg.1	ENSG00000092969;	0.0005086	0.0049262	TRUE
TC01001805.hg.1	ENSG00000092969;	0.0005329	0.0051442	TRUE
TC01000734.hg.1	ENSG00000118473;	0.0005329	0.0051442	TRUE
TC01000783.hg.1	ENSG00000117069;	0.0005361	0.005157	TRUE
TC01002706.hg.1	ENSG00000184292;	0.0005378	0.0051625	TRUE
TC01001624.hg.1	ENSG00000116741;	0.0005668	0.0054281	TRUE
TC01000100.hg.1	ENSG00000049246;	0.0005789	0.0055321	TRUE
TC02000762.hg.1	ENSG00000163083;	0.0005983	0.0057043	TRUE
TC01001736.hg.1	ENSG00000196352;	0.0006308	0.0059683	TRUE
TC04002931.hg.1	ENSG00000134853;	0.0006308	0.0059683	TRUE
TC04002931.hg.1	ENSG00000134853;	0.0006308	0.0059683	TRUE
TC02002624.hg.1	ENSG00000115415;	0.0006315	0.0059683	TRUE
TC01001624.hg.1	ENSG00000116741;	0.0006332	0.0059708	TRUE
TC01001805.hg.1	ENSG00000092969;	0.0006388	0.0060063	TRUE
TC01002669.hg.1	ENSG00000157193;	0.0006431	0.0060063	TRUE
TC02000762.hg.1	ENSG00000163083;	0.0006431	0.0060063	TRUE
TC02001750.hg.1	ENSG00000138061;	0.0006433	0.0060063	TRUE
TC02001750.hg.1	ENSG00000138061;	0.0006433	0.0060063	TRUE
TC01000100.hg.1	ENSG00000049246;	0.0006505	0.0060536	TRUE
TC01001805.hg.1	ENSG00000092969;	0.0006594	0.006123	TRUE
TC01003129.hg.1	ENSG00000143140;	0.0006767	0.0062638	TRUE

TC01003129.hg.1	ENSG00000143140;	0.0006767	0.0062638	TRUE
TC01001805.hg.1	ENSG00000092969;	0.0006801	0.0062746	TRUE
TC02002292.hg.1	ENSG00000136720;	0.0007109	0.0065292	TRUE
TC01003718.hg.1	ENSG00000133048;	0.0007109	0.0065292	TRUE
TC10000350.hg.1	ENSG00000107984;	0.0007131	0.0065292	TRUE
TC01002669.hg.1	ENSG00000157193;	0.0007138	0.0065292	TRUE
TC01001624.hg.1	ENSG00000116741;	0.0007689	0.0070186	TRUE
TC02002823.hg.1	ENSG00000135919;	0.0007764	0.0070646	TRUE
TC02002823.hg.1	ENSG00000135919;	0.0007764	0.0070646	TRUE
TC02002292.hg.1	ENSG00000136720;	0.0007845	0.0071155	TRUE
TC06000595.hg.1	ENSG00000112655;	0.0007942	0.0071649	TRUE
TC06000595.hg.1	ENSG00000112655;	0.0007942	0.0071649	TRUE
TC02000466.hg.1	ENSG00000159399;	0.0007958	0.0071649	TRUE
TC03000214.hg.1	ENSG00000168036;	0.0007958	0.0071649	TRUE
TC01001624.hg.1	ENSG00000116741;	0.0008047	0.0072224	TRUE
TC01003718.hg.1	ENSG00000133048;	0.0008175	0.0073217	TRUE
TC01001805.hg.1	ENSG00000092969;	0.0008355	0.0074672	TRUE
TC03000214.hg.1	ENSG00000168036;	0.0008522	0.007576	TRUE
TC01002706.hg.1	ENSG00000184292;	0.0008522	0.007576	TRUE
TC01001805.hg.1	ENSG00000092969;	0.000853	0.007576	TRUE
TC01002166.hg.1	ENSG00000116285;	0.0008644	0.0076453	TRUE
TC01003638.hg.1	ENSG00000073756;	0.0008644	0.0076453	TRUE
TC02000762.hg.1	ENSG00000163083;	0.0008644	0.0076453	TRUE
TC01001805.hg.1	ENSG00000092969;	0.0008722	0.0076832	TRUE
TC01001805.hg.1	ENSG00000092969;	0.0008834	0.0077054	TRUE
TC02001750.hg.1	ENSG00000138061;	0.0008834	0.0077054	TRUE
TC02000041.hg.1	ENSG00000115738;	0.0008855	0.0077054	TRUE
TC01001607.hg.1	ENSG00000116711;I	0.0008855	0.0077054	TRUE
TC01001607.hg.1	ENSG00000116711;I	0.0008855	0.0077054	TRUE
TC01001805.hg.1	ENSG00000092969;	0.0008856	0.0077054	TRUE
TC01001805.hg.1	ENSG00000092969;	0.0009141	0.0079375	TRUE
TC01000100.hg.1	ENSG00000049246;	0.0009365	0.0081155	TRUE
TC05001590.hg.1	ENSG00000113369;	0.0009457	0.0081787	TRUE
TC02002823.hg.1	ENSG00000135919;	0.0009508	0.0081982	TRUE
TC01001805.hg.1	ENSG00000092969;	0.0009508	0.0081982	TRUE
TC01001805.hg.1	ENSG00000092969;	0.0009599	0.0082435	TRUE
TC01001805.hg.1	ENSG00000092969;	0.0009599	0.0082435	TRUE
TC01000685.hg.1	ENSG00000162409;	0.0009661	0.0082718	TRUE
TC01000685.hg.1	ENSG00000162409;	0.0009706	0.0082851	TRUE
TC01000685.hg.1	ENSG00000162409;	0.0009715	0.0082851	TRUE
TC02001206.hg.1	ENSG00000118257;	0.0009753	0.0082923	TRUE
TC01003638.hg.1	ENSG00000073756;	0.0009753	0.0082923	TRUE
TC01000904.hg.1	ENSG00000162692;	0.0009832	0.0083268	TRUE
TC02002823.hg.1	ENSG00000135919;	0.0009832	0.0083268	TRUE
TC01001805.hg.1	ENSG00000092969;	0.0009874	0.008337	TRUE
TC01000685.hg.1	ENSG00000162409;	0.0009897	0.00834	TRUE
TC01000100.hg.1	ENSG00000049246;	0.0009932	0.0083532	TRUE
TC01000100.hg.1	ENSG00000049246;	0.0010138	0.0084996	TRUE
TC01000100.hg.1	ENSG00000049246;	0.0010206	0.0084996	TRUE
TC06001089.hg.1	ENSG00000111981;I	0.0010237	0.0084996	TRUE
TC02001031.hg.1	ENSG00000152256;	0.0010237	0.0084996	TRUE
TC04002931.hg.1	ENSG00000134853;	0.0010237	0.0084996	TRUE
TC06001089.hg.1	ENSG00000111981;I	0.0010237	0.0084996	TRUE
TC01003129.hg.1	ENSG00000143140;	0.0010237	0.0084996	TRUE
TC02000041.hg.1	ENSG00000115738;	0.0010237	0.0084996	TRUE
TC02000762.hg.1	ENSG00000163083;	0.0010285	0.0084996	TRUE
TC03000577.hg.1	ENSG00000144857;	0.0010338	0.0085162	TRUE
TC02002624.hg.1	ENSG00000115415;	0.0010354	0.0085162	TRUE

TC02001031.hg.1	ENSG00000152256;	0.0010354	0.0085162	TRUE
TC01001624.hg.1	ENSG00000116741;	0.0010527	0.008633	TRUE
TC01000703.hg.1	ENSG00000132849;	0.001108	0.0090694	TRUE
TC02002624.hg.1	ENSG00000115415;	0.0011122	0.0090868	TRUE
TC01000394.hg.1	ENSG00000159023;	0.0011362	0.0092652	TRUE
TC01003129.hg.1	ENSG00000143140;	0.0011419	0.0092677	TRUE
TC03001255.hg.1	ENSG00000033867;	0.0011419	0.0092677	TRUE
TC01003778.hg.1	ENSG00000076356;	0.0011446	0.0092677	TRUE
TC01003638.hg.1	ENSG00000073756;	0.0011452	0.0092677	TRUE
TC01003129.hg.1	ENSG00000143140;	0.0012035	0.0097175	TRUE
TC01003129.hg.1	ENSG00000143140;	0.0012053	0.0097175	TRUE
TC01000685.hg.1	ENSG00000162409;	0.001211	0.0097447	TRUE
TC05001752.hg.1	ENSG00000138829;	0.0012233	0.0097699	TRUE
TC10001711.hg.1;	ENSG00000066468;	0.0012233	0.0097699	TRUE
TC10001711.hg.1;	ENSG00000066468;	0.0012233	0.0097699	TRUE
TC04002931.hg.1	ENSG00000134853;	0.0012233	0.0097699	TRUE
TC04002931.hg.1	ENSG00000134853;	0.0012233	0.0097699	TRUE
TC08000845.hg.1	ENSG00000187786;	0.0012233	0.0097699	TRUE
TC03000248.hg.1	ENSG00000144791;	0.0012312	0.0097699	TRUE
TC02001963.hg.1	ENSG00000163235;	0.0012312	0.0097699	TRUE
TC01001805.hg.1	ENSG00000092969;	0.0012757	0.0100951	TRUE
TC01000904.hg.1	ENSG00000162692;	0.0012857	0.010155	TRUE
TC02001031.hg.1	ENSG00000152256;	0.001293	0.0101552	TRUE
TC01000100.hg.1	ENSG00000049246;	0.0012936	0.0101552	TRUE
TC06000595.hg.1	ENSG00000112655;	0.0012991	0.0101552	TRUE
TC01001805.hg.1	ENSG00000092969;	0.0012991	0.0101552	TRUE
TC05001933.hg.1	ENSG00000113721;	0.0012991	0.0101552	TRUE
TC03001071.hg.1	ENSG00000114315;	0.0012999	0.0101552	TRUE
TC01000685.hg.1	ENSG00000162409;	0.0013117	0.0102288	TRUE
TC01000904.hg.1	ENSG00000162692;	0.0013238	0.0103042	TRUE
TC01001624.hg.1	ENSG00000116741;	0.0013308	0.0103398	TRUE
TC01001805.hg.1	ENSG00000092969;	0.0013475	0.0104409	TRUE
TC01001805.hg.1	ENSG00000092969;	0.0013475	0.0104409	TRUE
TC01000904.hg.1	ENSG00000162692;	0.0013679	0.0105609	TRUE
TC06000608.hg.1	ENSG00000112715;	0.0013679	0.0105609	TRUE
TC01001090.hg.1	ENSG00000117289;	0.0014025	0.0107798	TRUE
TC01000734.hg.1	ENSG00000118473;	0.0014026	0.0107798	TRUE
TC01000685.hg.1	ENSG00000162409;	0.0014477	0.0110972	TRUE
TC10000350.hg.1	ENSG00000107984;	0.0014477	0.0110972	TRUE
TC02001206.hg.1	ENSG00000118257;	0.0014659	0.0111927	TRUE
TC01001624.hg.1	ENSG00000116741;	0.0014667	0.0111927	TRUE
TC01001624.hg.1	ENSG00000116741;	0.0014972	0.0114049	TRUE
TC01000685.hg.1	ENSG00000162409;	0.0015216	0.01157	TRUE
TC01000728.hg.1	ENSG00000162433;	0.0015636	0.0118684	TRUE
TC02001628.hg.1	ENSG00000084674;	0.0015925	0.0120667	TRUE
TC01000734.hg.1	ENSG00000118473;	0.0016032	0.0121256	TRUE
TC01003129.hg.1	ENSG00000143140;	0.001608	0.0121407	TRUE
TC02001437.hg.1	ENSG00000144476;	0.0016172	0.0121623	TRUE
TC01001736.hg.1	ENSG00000196352;	0.0016181	0.0121623	TRUE
TC05001933.hg.1	ENSG00000113721;	0.0016255	0.0121623	TRUE
TC02002624.hg.1	ENSG00000115415;	0.0016265	0.0121623	TRUE
TC01003638.hg.1	ENSG00000073756;	0.0016265	0.0121623	TRUE
TC10000350.hg.1	ENSG00000107984;	0.0016265	0.0121623	TRUE
TC01003129.hg.1	ENSG00000143140;	0.0016265	0.0121623	TRUE
TC01003638.hg.1	ENSG00000073756;	0.0017724	0.0131959	TRUE
TC01001704.hg.1	ENSG00000163531;	0.0017925	0.0133226	TRUE
TC01000703.hg.1	ENSG00000132849;	0.0018363	0.0136246	TRUE
TC01001607.hg.1	ENSG00000116711;	0.0019133	0.0141602	TRUE

TC03000262.hg.1	ENSG00000160801;	0.0019205	0.0141602	TRUE
TC03000846.hg.1	ENSG00000196549;	0.0019205	0.0141602	TRUE
TC01000728.hg.1	ENSG00000162433;	0.0019205	0.0141602	TRUE
TC01002954.hg.1	ENSG00000134243;	0.0019205	0.0141602	TRUE
TC01000904.hg.1	ENSG00000162692;	0.0019284	0.0141602	TRUE
TC02000466.hg.1	ENSG00000159399;	0.0019332	0.0141716	TRUE
TC01000685.hg.1	ENSG00000162409;	0.0019435	0.0142222	TRUE
TC02002808.hg.1	ENSG00000116106;	0.0019621	0.0142729	TRUE
TC02001206.hg.1	ENSG00000118257;	0.0019621	0.0142729	TRUE
TC06004073.hg.1	ENSG00000204520;	0.0019621	0.0142729	TRUE
TC02000041.hg.1	ENSG00000115738;	0.0019621	0.0142729	TRUE
TC01000904.hg.1	ENSG00000162692;	0.0019621	0.0142729	TRUE
TC01001188.hg.1;	ENSG00000143369;	0.0019621	0.0142729	TRUE
TC01001624.hg.1	ENSG00000116741;	0.0020021	0.0144777	TRUE
TC02001206.hg.1	ENSG00000118257;	0.0020082	0.0144979	TRUE
TC01001805.hg.1	ENSG00000092969;	0.0020325	0.0146487	TRUE
TC01002669.hg.1	ENSG00000157193;	0.0020504	0.0147401	TRUE
TC03000013.hg.1	ENSG00000150995;	0.0020504	0.0147401	TRUE
TC01000904.hg.1	ENSG00000162692;	0.0020648	0.0147767	TRUE
TC02001031.hg.1	ENSG00000152256;	0.0020762	0.0147767	TRUE
TC03002106.hg.1	ENSG00000090530;	0.0020762	0.0147767	TRUE
TC02002808.hg.1	ENSG00000116106;	0.0020762	0.0147767	TRUE
TC05001933.hg.1	ENSG00000113721;	0.0020762	0.0147767	TRUE
TC03001590.hg.1	ENSG00000184500;	0.0020762	0.0147767	TRUE
TC04000408.hg.1	ENSG00000169429;	0.0020762	0.0147767	TRUE
TC04002931.hg.1	ENSG00000134853;	0.0020762	0.0147767	TRUE
TC03001071.hg.1	ENSG00000114315;	0.0020762	0.0147767	TRUE
TC02001031.hg.1	ENSG00000152256;	0.0020997	0.0148328	TRUE
TC01001805.hg.1	ENSG00000092969;	0.0022531	0.0158904	TRUE
TC03000013.hg.1	ENSG00000150995;	0.0022884	0.0161127	TRUE
TC01000685.hg.1	ENSG00000162409;	0.0022934	0.0161215	TRUE
TC01001607.hg.1	ENSG00000116711;l	0.0023255	0.0163208	TRUE
TC01001624.hg.1	ENSG00000116741;	0.0024059	0.0168573	TRUE
TC01002669.hg.1	ENSG00000157193;	0.0024433	0.0170908	TRUE
TC01003638.hg.1	ENSG00000073756;	0.0024529	0.0171161	TRUE
TC02000762.hg.1	ENSG00000163083;	0.0024529	0.0171161	TRUE
TC01002954.hg.1	ENSG00000134243;	0.0024674	0.0171617	TRUE
TC04000313.hg.1	ENSG00000157404;	0.0024674	0.0171617	TRUE
TC01000685.hg.1	ENSG00000162409;	0.0025115	0.0174256	TRUE
TC01001736.hg.1	ENSG00000196352;	0.002538	0.0175812	TRUE
TC02001206.hg.1	ENSG00000118257;	0.002555	0.0176702	TRUE
TC03000214.hg.1	ENSG00000168036;	0.0025845	0.0178414	TRUE
TC03002146.hg.1	ENSG00000189058;	0.0025901	0.0178414	TRUE
TC02001628.hg.1	ENSG00000084674;	0.0025901	0.0178414	TRUE
TC01001736.hg.1	ENSG00000196352;	0.0026066	0.0179115	TRUE
TC02000041.hg.1	ENSG00000115738;	0.0026272	0.0180244	TRUE
TC02000466.hg.1	ENSG00000159399;	0.002646	0.0181238	TRUE
TC01002669.hg.1	ENSG00000157193;	0.0026699	0.0182295	TRUE
TC03000013.hg.1	ENSG00000150995;	0.0026699	0.0182295	TRUE
TC01000685.hg.1	ENSG00000162409;	0.0026699	0.0182295	TRUE
TC01001805.hg.1	ENSG00000092969;	0.0027106	0.0184483	TRUE
TC01001607.hg.1	ENSG00000116711;l	0.0027276	0.0185201	TRUE
TC02000219.hg.1	ENSG00000049323;	0.0027276	0.0185201	TRUE
TC01001090.hg.1	ENSG00000117289;	0.0027344	0.018522	TRUE
TC01001607.hg.1	ENSG00000116711;l	0.0027476	0.0185819	TRUE
TC02001628.hg.1	ENSG00000084674;	0.0027657	0.0186752	TRUE
TC02002823.hg.1	ENSG00000135919;	0.0028453	0.019182	TRUE
TC01000685.hg.1	ENSG00000162409;	0.0028812	0.0193929	TRUE

TC02000466.hg.1	ENSG00000159399;	0.0028856	0.0193929	TRUE
TC01002954.hg.1	ENSG00000134243;	0.0028988	0.0194508	TRUE
TC01003638.hg.1	ENSG00000073756;	0.0029173	0.0195445	TRUE
TC02000762.hg.1	ENSG00000163083;	0.0029648	0.0198165	TRUE
TC03000214.hg.1	ENSG00000168036;	0.0029648	0.0198165	TRUE
TC03001071.hg.1	ENSG00000114315;	0.0030188	0.02012	TRUE
TC01001736.hg.1	ENSG00000196352;	0.003022	0.02012	TRUE
TC01002166.hg.1	ENSG00000116285;	0.0030461	0.020249	TRUE
TC01002954.hg.1	ENSG00000134243;	0.0030548	0.020275	TRUE
TC01003638.hg.1	ENSG00000073756;	0.0030835	0.020434	TRUE
TC01001736.hg.1	ENSG00000196352;	0.0031151	0.0206117	TRUE
TC01000100.hg.1	ENSG00000049246;	0.0031247	0.0206431	TRUE
TC03000214.hg.1	ENSG00000168036;	0.003161	0.0208508	TRUE
TC01003129.hg.1	ENSG00000143140;	0.0032218	0.0210573	TRUE
TC03000214.hg.1	ENSG00000168036;	0.0032218	0.0210573	TRUE
TC03000214.hg.1	ENSG00000168036;	0.0032218	0.0210573	TRUE
TC02001031.hg.1	ENSG00000152256;	0.0032218	0.0210573	TRUE
TC02002808.hg.1	ENSG00000116106;	0.0032218	0.0210573	TRUE
TC07000328.hg.1	ENSG00000146648;	0.0032218	0.0210573	TRUE
TC05003423.hg.1	ENSG00000082196;	0.0032218	0.0210573	TRUE
TC01002166.hg.1	ENSG00000116285;	0.0032218	0.0210573	TRUE
TC03000214.hg.1	ENSG00000168036;	0.0032218	0.0210573	TRUE
TC06000968.hg.1	ENSG00000146374;	0.0032218	0.0210573	TRUE
TC05001752.hg.1	ENSG00000138829;	0.0032218	0.0210573	TRUE
TC01001624.hg.1	ENSG00000116741;	0.0033097	0.021419	TRUE
TC03000248.hg.1	ENSG00000144791;	0.0033097	0.021419	TRUE
TC01003638.hg.1	ENSG00000073756;	0.0033287	0.0214936	TRUE
TC10001711.hg.1;	ENSG00000066468;	0.0033435	0.0215239	TRUE
TC01000904.hg.1	ENSG00000162692;	0.0033435	0.0215239	TRUE
TC03000214.hg.1	ENSG00000168036;	0.0033435	0.0215239	TRUE
TC01000783.hg.1	ENSG00000117069;	0.0033591	0.0215594	TRUE
TC04002931.hg.1	ENSG00000134853;	0.0035025	0.0223699	TRUE
TC01003042.hg.1	ENSG00000092607;	0.0035025	0.0223699	TRUE
TC03000013.hg.1	ENSG00000150995;	0.0035025	0.0223699	TRUE
TC03000846.hg.1	ENSG00000196549;	0.0035129	0.0223699	TRUE
TC03000846.hg.1	ENSG00000196549;	0.0035129	0.0223699	TRUE
TC01001805.hg.1	ENSG00000092969;	0.0035167	0.0223699	TRUE
TC01002932.hg.1	ENSG00000060718;	0.0035221	0.0223714	TRUE
TC01000904.hg.1	ENSG00000162692;	0.0035396	0.0224463	TRUE
TC05001933.hg.1	ENSG00000113721;	0.0035497	0.0224463	TRUE
TC03001071.hg.1	ENSG00000114315;	0.0035497	0.0224463	TRUE
TC03000214.hg.1	ENSG00000168036;	0.0035497	0.0224463	TRUE
TC01003718.hg.1	ENSG00000133048;	0.0035691	0.0225024	TRUE
TC01000904.hg.1	ENSG00000162692;	0.0036069	0.0227077	TRUE
TC03000248.hg.1	ENSG00000144791;	0.0036181	0.0227445	TRUE
TC01001736.hg.1	ENSG00000196352;	0.00375	0.0235394	TRUE
TC01000394.hg.1	ENSG00000159023;	0.0038074	0.0238644	TRUE
TC01000685.hg.1	ENSG00000162409;	0.003845	0.0240122	TRUE
TC01003638.hg.1	ENSG00000073756;	0.003845	0.0240122	TRUE
TC01000685.hg.1	ENSG00000162409;	0.003845	0.0240122	TRUE
TC02001750.hg.1	ENSG00000138061;	0.003845	0.0240122	TRUE
TC01000728.hg.1	ENSG00000162433;	0.0038609	0.0240242	TRUE
TC01000734.hg.1	ENSG00000118473;	0.0038928	0.0241303	TRUE
TC04000408.hg.1	ENSG00000169429;	0.0038928	0.0241303	TRUE
TC03000248.hg.1	ENSG00000144791;	0.0039005	0.0241303	TRUE
TC04001509.hg.1	ENSG00000173376;	0.0039005	0.0241303	TRUE
TC01002706.hg.1	ENSG00000184292;	0.0039005	0.0241303	TRUE
TC03000013.hg.1	ENSG00000150995;	0.0039574	0.0244113	TRUE

TC01000394.hg.1	ENSG00000159023;	0.0040221	0.0247393	TRUE
TC03001466.hg.1	ENSG00000163931;	0.0040307	0.0247393	TRUE
TC06001772.hg.1	ENSG00000146233;	0.0040307	0.0247393	TRUE
TC02001963.hg.1	ENSG00000163235;	0.0040307	0.0247393	TRUE
TC02001963.hg.1	ENSG00000163235;	0.0040307	0.0247393	TRUE
TC01000685.hg.1	ENSG00000162409;	0.0041554	0.0254132	TRUE
TC01002954.hg.1	ENSG00000134243;	0.004184	0.0255517	TRUE
TC06001089.hg.1	ENSG00000111981;I	0.0042129	0.0255777	TRUE
TC02000281.hg.1	ENSG00000116016;	0.0042129	0.0255777	TRUE
TC01002166.hg.1	ENSG00000116285;	0.0042129	0.0255777	TRUE
TC01000904.hg.1	ENSG00000162692;	0.0042129	0.0255777	TRUE
TC01001805.hg.1	ENSG00000092969;	0.0042331	0.0255777	TRUE
TC01003638.hg.1	ENSG00000073756;	0.0042331	0.0255777	TRUE
TC02001628.hg.1	ENSG00000084674;	0.0042331	0.0255777	TRUE
TC03001071.hg.1	ENSG00000114315;	0.0042331	0.0255777	TRUE
TC01003211.hg.1;	ENSG00000143387;	0.0042331	0.0255777	TRUE
TC02000041.hg.1	ENSG00000115738;	0.0042331	0.0255777	TRUE
TC01000783.hg.1	ENSG00000117069;	0.0042716	0.0256837	TRUE
TC01000100.hg.1	ENSG00000049246;	0.0043093	0.0258737	TRUE
TC02002292.hg.1	ENSG00000136720;	0.0043163	0.0258798	TRUE
TC01001736.hg.1	ENSG00000196352;	0.0043531	0.0260635	TRUE
TC06001510.hg.1	ENSG00000137331;	0.0043659	0.0261039	TRUE
TC01001805.hg.1	ENSG00000092969;	0.0043986	0.0262629	TRUE
TC01000685.hg.1	ENSG00000162409;	0.0044922	0.026776	TRUE
TC01001805.hg.1	ENSG00000092969;	0.0044971	0.026776	TRUE
TC01000394.hg.1	ENSG00000159023;	0.004504	0.0267803	TRUE
TC02000762.hg.1	ENSG00000163083;	0.004603	0.0272375	TRUE
TC02001100.hg.1;	ENSG00000138448;	0.004603	0.0272375	TRUE
TC04000503.hg.1	ENSG00000138696;	0.004603	0.0272375	TRUE
TC01000685.hg.1	ENSG00000162409;	0.0046573	0.0272375	TRUE
TC05001933.hg.1	ENSG00000113721;	0.0046874	0.0272375	TRUE
TC04000729.hg.1	ENSG00000151617;	0.0046874	0.0272375	TRUE
TC01002578.hg.1	ENSG00000117394;	0.0046874	0.0272375	TRUE
TC07001883.hg.1	ENSG00000085662;	0.0046874	0.0272375	TRUE
TC02001628.hg.1	ENSG00000084674;	0.0046874	0.0272375	TRUE
TC07001883.hg.1	ENSG00000085662;	0.0046874	0.0272375	TRUE
TC07001883.hg.1	ENSG00000085662;	0.0046874	0.0272375	TRUE
TC01001624.hg.1	ENSG00000116741;	0.0046874	0.0272375	TRUE
TC03000214.hg.1	ENSG00000168036;	0.0046874	0.0272375	TRUE
TC02000041.hg.1	ENSG00000115738;	0.0046874	0.0272375	TRUE
TC02000041.hg.1	ENSG00000115738;	0.0046874	0.0272375	TRUE
TC08000845.hg.1	ENSG00000187786;	0.0046874	0.0272375	TRUE
TC06004066.hg.1	NR_001434;NR_001	0.0046874	0.0272375	TRUE
TC01001090.hg.1	ENSG00000117289;	0.004701	0.0272375	TRUE
TC01003718.hg.1	ENSG00000133048;	0.0047018	0.0272375	TRUE
TC01002669.hg.1	ENSG00000157193;	0.0048182	0.0278742	TRUE
TC01000685.hg.1	ENSG00000162409;	0.0050605	0.0292364	TRUE
TC01001624.hg.1	ENSG00000116741;	0.0052329	0.0301569	TRUE
TC03000577.hg.1	ENSG00000144857;	0.0052329	0.0301569	TRUE
TC01003638.hg.1	ENSG00000073756;	0.0052696	0.0301569	TRUE
TC07001293.hg.1	ENSG00000106483;	0.0052696	0.0301569	TRUE
TC05001933.hg.1	ENSG00000113721;	0.0052696	0.0301569	TRUE
TC05001933.hg.1	ENSG00000113721;	0.0052696	0.0301569	TRUE
TC01003638.hg.1	ENSG00000073756;	0.0052696	0.0301569	TRUE
TC02000041.hg.1	ENSG00000115738;	0.0052696	0.0301569	TRUE
TC05001752.hg.1	ENSG00000138829;	0.0052696	0.0301569	TRUE
TC01001624.hg.1	ENSG00000116741;	0.0052903	0.0301569	TRUE
TC01000685.hg.1	ENSG00000162409;	0.0053389	0.0303935	TRUE

TC01001090.hg.1	ENSG00000117289;	0.0055264	0.0314189	TRUE
TC01000100.hg.1	ENSG00000049246;	0.0055449	0.0314826	TRUE
TC01001805.hg.1	ENSG00000092969;	0.005635	0.0319305	TRUE
TC01001805.hg.1	ENSG00000092969;	0.005635	0.0319305	TRUE
TC01000728.hg.1	ENSG00000162433;	0.0056612	0.0320151	TRUE
TC01001736.hg.1	ENSG00000196352;	0.0057957	0.0326497	TRUE
TC01001805.hg.1	ENSG00000092969;	0.0057962	0.0326497	TRUE
TC03001071.hg.1	ENSG00000114315;	0.0057962	0.0326497	TRUE
TC02000762.hg.1	ENSG00000163083;	0.0057962	0.0326497	TRUE
TC01003638.hg.1	ENSG00000073756;	0.0058689	0.0329724	TRUE
TC01001805.hg.1	ENSG00000092969;	0.0059099	0.0331591	TRUE
TC01002706.hg.1	ENSG00000184292;	0.0060125	0.0336903	TRUE
TC05001933.hg.1	ENSG00000113721;	0.0060605	0.0338708	TRUE
TC05003423.hg.1	ENSG00000082196;	0.0060605	0.0338708	TRUE
TC03001071.hg.1	ENSG00000114315;	0.0060605	0.0338708	TRUE
TC01002578.hg.1	ENSG00000117394;	0.0061171	0.0340983	TRUE
TC01001736.hg.1	ENSG00000196352;	0.0063592	0.0354012	TRUE
TC01000904.hg.1	ENSG00000162692;	0.0063986	0.0355748	TRUE
TC03001071.hg.1	ENSG00000114315;	0.006463	0.0356875	TRUE
TC02000281.hg.1	ENSG00000116016;	0.006463	0.0356875	TRUE
TC04001245.hg.1	ENSG00000145242;	0.006463	0.0356875	TRUE
TC03000917.hg.1	ENSG00000136603;	0.006463	0.0356875	TRUE
TC07000768.hg.1	ENSG00000174697;	0.0064953	0.0356875	TRUE
TC03000214.hg.1	ENSG00000168036;	0.0064953	0.0356875	TRUE
TC19000191.hg.1	ENSG00000130164;	0.0064953	0.0356875	TRUE
TC01000728.hg.1	ENSG00000162433;	0.0064953	0.0356875	TRUE
TC02000219.hg.1	ENSG00000049323;	0.0064953	0.0356875	TRUE
TC02000219.hg.1	ENSG00000049323;	0.0064953	0.0356875	TRUE
TC02000041.hg.1	ENSG00000115738;	0.0065106	0.0356875	TRUE
TC01000100.hg.1	ENSG00000049246;	0.0065339	0.0357695	TRUE
TC02002823.hg.1	ENSG00000135919;	0.0066565	0.036371	TRUE
TC02002823.hg.1	ENSG00000135919;	0.0066565	0.036371	TRUE
TC03001071.hg.1	ENSG00000114315;	0.0066785	0.0363748	TRUE
TC01003638.hg.1	ENSG00000073756;	0.0066785	0.0363748	TRUE
TC01002166.hg.1	ENSG00000116285;	0.0066785	0.0363748	TRUE
TC01002578.hg.1	ENSG00000117394;	0.0067271	0.0365467	TRUE
TC01003638.hg.1	ENSG00000073756;	0.0067627	0.0366066	TRUE
TC01000728.hg.1	ENSG00000162433;	0.0067627	0.0366066	TRUE
TC01003718.hg.1	ENSG00000133048;	0.0067627	0.0366066	TRUE
TC01001805.hg.1	ENSG00000092969;	0.0067724	0.0366066	TRUE
TC01003638.hg.1	ENSG00000073756;	0.0068054	0.0367391	TRUE
TC01000685.hg.1	ENSG00000162409;	0.00694	0.0374185	TRUE
TC01000685.hg.1	ENSG00000162409;	0.0069629	0.0374948	TRUE
TC01002669.hg.1	ENSG00000157193;	0.0070544	0.0379394	TRUE
TC01000904.hg.1	ENSG00000162692;	0.0072179	0.0387702	TRUE
TC01002166.hg.1	ENSG00000116285;	0.0073474	0.0394161	TRUE
TC02000219.hg.1	ENSG00000049323;	0.0073708	0.0394922	TRUE
TC01000685.hg.1	ENSG00000162409;	0.0074328	0.039734	TRUE
TC01000685.hg.1	ENSG00000162409;	0.0074345	0.039734	TRUE
TC01001607.hg.1	ENSG00000116711;l	0.0075007	0.0399883	TRUE
TC02000762.hg.1	ENSG00000163083;	0.0075007	0.0399883	TRUE
TC01001607.hg.1	ENSG00000116711;l	0.0075007	0.0399883	TRUE
TC01000685.hg.1	ENSG00000162409;	0.0075904	0.0403656	TRUE
TC01003497.hg.1	ENSG00000117479;	0.0076122	0.0404316	TRUE
TC04000173.hg.1	ENSG00000109610;	0.0077577	0.041128	TRUE
TC10000350.hg.1	ENSG00000107984;	0.0077577	0.041128	TRUE
TC01003129.hg.1	ENSG00000143140;	0.0078229	0.0412581	TRUE
TC01002166.hg.1	ENSG00000116285;	0.0078229	0.0412581	TRUE

TC02002808.hg.1	ENSG00000116106;	0.0078229	0.0412581	TRUE
TC02001206.hg.1	ENSG00000118257;	0.0078229	0.0412581	TRUE
TC01000728.hg.1	ENSG00000162433;	0.0078353	0.0412581	TRUE
TC01001805.hg.1	ENSG00000092969;	0.007863	0.0413533	TRUE
TC01001607.hg.1	ENSG00000116711;I	0.0078947	0.0414688	TRUE
TC01002669.hg.1	ENSG00000157193;	0.0082279	0.0431662	TRUE
TC01001624.hg.1	ENSG00000116741;	0.0082996	0.0434893	TRUE
TC01003638.hg.1	ENSG00000073756;	0.0083099	0.0434897	TRUE
TC02002292.hg.1	ENSG00000136720;	0.0083935	0.043767	TRUE
TC01003638.hg.1	ENSG00000073756;	0.0083935	0.043767	TRUE
TC03001071.hg.1	ENSG00000114315;	0.0083935	0.043767	TRUE
TC02001628.hg.1	ENSG00000084674;	0.0083935	0.043767	TRUE
TC02002624.hg.1	ENSG00000115415;	0.0083935	0.043767	TRUE
TC01001188.hg.1;	ENSG00000143369;	0.0085757	0.0445269	TRUE
TC01001624.hg.1	ENSG00000116741;	0.0085757	0.0445269	TRUE
TC02002714.hg.1	ENSG00000163251;	0.0086636	0.0447486	TRUE
TC02001031.hg.1	ENSG00000152256;	0.0086636	0.0447486	TRUE
TC11000182.hg.1;	ENSG00000148926;	0.0086636	0.0447486	TRUE
TC07001293.hg.1	ENSG00000106483;	0.0086636	0.0447486	TRUE
TC02001031.hg.1	ENSG00000152256;	0.0086636	0.0447486	TRUE
TC07001559.hg.1	ENSG00000075223;	0.0086636	0.0447486	TRUE
TC01001805.hg.1	ENSG00000092969;	0.0086968	0.0447486	TRUE
TC01001805.hg.1	ENSG00000092969;	0.0088931	0.0455402	TRUE
TC03000846.hg.1	ENSG00000196549;	0.0088931	0.0455402	TRUE
TC01002578.hg.1	ENSG00000117394;	0.0088931	0.0455402	TRUE
TC03000214.hg.1	ENSG00000168036;	0.0088931	0.0455402	TRUE
TC02000082.hg.1	ENSG00000071575;	0.0088931	0.0455402	TRUE
TC01001607.hg.1	ENSG00000116711;I	0.0088931	0.0455402	TRUE
TC03000214.hg.1	ENSG00000168036;	0.0088931	0.0455402	TRUE
TC01002954.hg.1	ENSG00000134243;	0.008939	0.0455571	TRUE
TC01000100.hg.1	ENSG00000049246;	0.0090221	0.0459257	TRUE
TC01001805.hg.1	ENSG00000092969;	0.0090896	0.0462147	TRUE
TC01001624.hg.1	ENSG00000116741;	0.0092144	0.0467616	TRUE
TC01000703.hg.1	ENSG00000132849;	0.0092144	0.0467616	TRUE
TC01001805.hg.1	ENSG00000092969;	0.00923	0.0467616	TRUE
TC01001736.hg.1	ENSG00000196352;	0.0092557	0.0468366	TRUE
TC01003129.hg.1	ENSG00000143140;	0.0093579	0.0468661	TRUE
TC03000214.hg.1	ENSG00000168036;	0.0093579	0.0468661	TRUE
TC07001883.hg.1	ENSG00000085662;	0.0093579	0.0468661	TRUE
TC01001805.hg.1	ENSG00000092969;	0.0093579	0.0468661	TRUE
TC07000328.hg.1	ENSG00000146648;	0.0093579	0.0468661	TRUE
TC03000214.hg.1	ENSG00000168036;	0.0093579	0.0468661	TRUE
TC02000041.hg.1	ENSG00000115738;	0.0093579	0.0468661	TRUE
TC02002292.hg.1	ENSG00000136720;	0.0093579	0.0468661	TRUE
TC07001293.hg.1	ENSG00000106483;	0.0093601	0.0468661	TRUE
TC01000904.hg.1	ENSG00000162692;	0.0094429	0.0472257	TRUE
TC01000685.hg.1	ENSG00000162409;	0.0094661	0.0472861	TRUE
TC01001090.hg.1	ENSG00000117289;	0.0095115	0.0474575	TRUE
TC01000685.hg.1	ENSG00000162409;	0.0095733	0.0477103	TRUE
TC02000281.hg.1	ENSG00000116016;	0.0096748	0.0481603	TRUE
TC01000783.hg.1	ENSG00000117069;	0.0097717	0.0485634	TRUE
TC01003638.hg.1	ENSG00000073756;	0.0097841	0.0485634	TRUE
TC01003211.hg.1;	ENSG00000143387;	0.0097841	0.0485634	TRUE
TC01001805.hg.1	ENSG00000092969;	0.0101383	0.0502046	FALSE
TC01003638.hg.1	ENSG00000073756;	0.0101383	0.0502046	FALSE
TC01001805.hg.1	ENSG00000092969;	0.0102077	0.050461	FALSE
TC01001805.hg.1	ENSG00000092969;	0.0103089	0.0509024	FALSE
TC02000041.hg.1	ENSG00000115738;	0.0103981	0.0512512	FALSE

TC03001466.hg.1	ENSG00000163931;	0.0104035	0.0512512	FALSE
TC01001805.hg.1	ENSG00000092969;	0.0104289	0.0513174	FALSE
TC01001607.hg.1	ENSG00000116711;I	0.0106025	0.0521115	FALSE
TC01001805.hg.1	ENSG00000092969;	0.0106894	0.0524786	FALSE
TC01002578.hg.1	ENSG00000117394;	0.0110046	0.0537714	FALSE
TC06001510.hg.1	ENSG00000137331;	0.0110469	0.0537714	FALSE
TC01003638.hg.1	ENSG00000073756;	0.0110762	0.0537714	FALSE
TC01002166.hg.1	ENSG00000116285;	0.0110762	0.0537714	FALSE
TC06000087.hg.1	ENSG00000078401;	0.0110762	0.0537714	FALSE
TC02000281.hg.1	ENSG00000116016;	0.0110762	0.0537714	FALSE
TC03000577.hg.1	ENSG00000144857;	0.0110762	0.0537714	FALSE
TC01001624.hg.1	ENSG00000116741;	0.0110762	0.0537714	FALSE
TC01000904.hg.1	ENSG00000162692;	0.0110762	0.0537714	FALSE
TC03000214.hg.1	ENSG00000168036;	0.0110762	0.0537714	FALSE
TC04000432.hg.1	ENSG00000156234;	0.0110762	0.0537714	FALSE
TC03000214.hg.1	ENSG00000168036;	0.0111097	0.0537714	FALSE
TC02000511.hg.1;	ENSG00000168906;	0.0111097	0.0537714	FALSE
TC04000173.hg.1	ENSG00000109610;	0.0112063	0.053813	FALSE
TC01003638.hg.1	ENSG00000073756;	0.0112063	0.053813	FALSE
TC02001628.hg.1	ENSG00000084674;	0.0112063	0.053813	FALSE
TC01001188.hg.1;	ENSG00000143369;	0.0112063	0.053813	FALSE
TC06000608.hg.1	ENSG00000112715;	0.0112063	0.053813	FALSE
TC04000274.hg.1	ENSG00000145246;	0.0112063	0.053813	FALSE
TC07001627.hg.1	ENSG00000105880;	0.0112063	0.053813	FALSE
TC04000274.hg.1	ENSG00000145246;	0.0112063	0.053813	FALSE
TC02001031.hg.1	ENSG00000152256;	0.0112063	0.053813	FALSE
TC02001031.hg.1	ENSG00000152256;	0.0112063	0.053813	FALSE
TC02002624.hg.1	ENSG00000115415;	0.0112063	0.053813	FALSE
TC03000214.hg.1	ENSG00000168036;	0.0112063	0.053813	FALSE
TC01002706.hg.1	ENSG00000184292;	0.0114978	0.0547824	FALSE
TC01002578.hg.1	ENSG00000117394;	0.0114978	0.0547824	FALSE
TC01003778.hg.1	ENSG00000076356;	0.0115268	0.0547985	FALSE
TC01002166.hg.1	ENSG00000116285;	0.0115268	0.0547985	FALSE
TC01000904.hg.1	ENSG00000162692;	0.0115809	0.0549641	FALSE
TC01000734.hg.1	ENSG00000118473;	0.0116722	0.0553057	FALSE
TC01003718.hg.1	ENSG00000133048;	0.0116722	0.0553057	FALSE
TC04002931.hg.1	ENSG00000134853;	0.0119204	0.0563882	FALSE
TC01000783.hg.1	ENSG00000117069;	0.0120196	0.0567946	FALSE
TC01003638.hg.1	ENSG00000073756;	0.0122717	0.057922	FALSE
TC01003638.hg.1	ENSG00000073756;	0.0123382	0.0581716	FALSE
TC01000685.hg.1	ENSG00000162409;	0.0124867	0.0588068	FALSE
TC01001603.hg.1	ENSG00000116690;	0.0125316	0.0589534	FALSE
TC01003778.hg.1	ENSG00000076356;	0.0126525	0.0594571	FALSE
TC01001624.hg.1	ENSG00000116741;	0.0126933	0.0595735	FALSE
TC02000041.hg.1	ENSG00000115738;	0.012733	0.0595735	FALSE
TC04001509.hg.1	ENSG00000173376;	0.012733	0.0595735	FALSE
TC03001071.hg.1	ENSG00000114315;	0.012733	0.0595735	FALSE
TC01001805.hg.1	ENSG00000092969;	0.012733	0.0595735	FALSE
TC01002166.hg.1	ENSG00000116285;	0.012733	0.0595735	FALSE
TC01001624.hg.1	ENSG00000116741;	0.0128817	0.0600724	FALSE
TC01003638.hg.1	ENSG00000073756;	0.0129762	0.0602601	FALSE
TC01001805.hg.1	ENSG00000092969;	0.0129762	0.0602601	FALSE
TC03000214.hg.1	ENSG00000168036;	0.0129762	0.0602601	FALSE
TC01001805.hg.1	ENSG00000092969;	0.0129853	0.0602601	FALSE
TC01002669.hg.1	ENSG00000157193;	0.0129853	0.0602601	FALSE
TC01001736.hg.1	ENSG00000196352;	0.0131129	0.0606879	FALSE
TC01000685.hg.1	ENSG00000162409;	0.0131129	0.0606879	FALSE
TC01000685.hg.1	ENSG00000162409;	0.0131129	0.0606879	FALSE

TC01001736.hg.1	ENSG00000196352;	0.0132058	0.060986	FALSE
TC01003211.hg.1;	ENSG00000143387;	0.0133427	0.0615518	FALSE
TC01000100.hg.1	ENSG00000049246;	0.0133587	0.0615594	FALSE
TC01001805.hg.1	ENSG00000092969;	0.0137459	0.0632757	FALSE
TC01001473.hg.1	ENSG00000143153;	0.0138358	0.0636211	FALSE
TC01001907.hg.1	ENSG00000143641;	0.0140249	0.0644214	FALSE
TC07001293.hg.1	ENSG00000106483;	0.0141339	0.0646445	FALSE
TC01001736.hg.1	ENSG00000196352;	0.0141339	0.0646445	FALSE
TC01001624.hg.1	ENSG00000116741;	0.0141339	0.0646445	FALSE
TC02000762.hg.1	ENSG00000163083;	0.0141339	0.0646445	FALSE
TC02001628.hg.1	ENSG00000084674;	0.0141339	0.0646445	FALSE
TC02001628.hg.1	ENSG00000084674;	0.0141339	0.0646445	FALSE
TC02000281.hg.1	ENSG00000116016;	0.0141339	0.0646445	FALSE
TC04001084.hg.1	ENSG00000109819;	0.0142415	0.0648593	FALSE
TC01000904.hg.1	ENSG00000162692;	0.0143728	0.0653879	FALSE
TC01001624.hg.1	ENSG00000116741;	0.0144673	0.065748	FALSE
TC01001473.hg.1	ENSG00000143153;	0.0148553	0.065977	FALSE
TC01000685.hg.1	ENSG00000162409;	0.0149141	0.065977	FALSE
TC04000408.hg.1	ENSG00000169429;	0.0149141	0.065977	FALSE
TC02001100.hg.1;	ENSG00000138448;	0.0150917	0.065977	FALSE
TC04001509.hg.1	ENSG00000173376;	0.0150917	0.065977	FALSE
TC03002146.hg.1	ENSG00000189058;	0.0150917	0.065977	FALSE
TC01003129.hg.1	ENSG00000143140;	0.0150917	0.065977	FALSE
TC05001933.hg.1	ENSG00000113721;	0.0150917	0.065977	FALSE
TC05000619.hg.1	ENSG00000198108;	0.0150917	0.065977	FALSE
TC02000041.hg.1	ENSG00000115738;	0.0151842	0.065977	FALSE
TC01000685.hg.1	ENSG00000162409;	0.0152106	0.065977	FALSE
TC01000394.hg.1	ENSG00000159023;	0.0152217	0.065977	FALSE
TC03000214.hg.1	ENSG00000168036;	0.0152328	0.065977	FALSE
TC02001031.hg.1	ENSG00000152256;	0.0152328	0.065977	FALSE
TC04000729.hg.1	ENSG00000151617;	0.0152328	0.065977	FALSE
TC05001933.hg.1	ENSG00000113721;	0.0152328	0.065977	FALSE
TC21000241.hg.1	ENSG00000197381;	0.0152328	0.065977	FALSE
TC10001398.hg.1	ENSG00000122884;	0.0152328	0.065977	FALSE
TC06000595.hg.1	ENSG00000112655;	0.0152328	0.065977	FALSE
TC12000076.hg.1	ENSG00000010278;	0.0152328	0.065977	FALSE
TC07000328.hg.1	ENSG00000146648;	0.0152328	0.065977	FALSE
TC05001385.hg.1	ENSG00000145632;	0.0152328	0.065977	FALSE
TC06001027.hg.1	ENSG00000118503;	0.0152328	0.065977	FALSE
TC06000087.hg.1	ENSG00000078401;	0.0152328	0.065977	FALSE
TC02000219.hg.1	ENSG00000049323;	0.0152328	0.065977	FALSE
TC08000845.hg.1	ENSG00000187786;	0.0152328	0.065977	FALSE
TC08000845.hg.1	ENSG00000187786;	0.0152328	0.065977	FALSE
TC08000845.hg.1	ENSG00000187786;	0.0152328	0.065977	FALSE
TC02000281.hg.1	ENSG00000116016;	0.0152328	0.065977	FALSE
TC05001590.hg.1	ENSG00000113369;	0.0152328	0.065977	FALSE
TC04001084.hg.1	ENSG00000109819;	0.0152328	0.065977	FALSE
TC02000762.hg.1	ENSG00000163083;	0.0152328	0.065977	FALSE
TC10000350.hg.1	ENSG00000107984;	0.0152328	0.065977	FALSE
TC10001711.hg.1;	ENSG00000066468;	0.0152328	0.065977	FALSE
TC10001711.hg.1;	ENSG00000066468;	0.0152328	0.065977	FALSE
TC10001711.hg.1;	ENSG00000066468;	0.0152328	0.065977	FALSE
TC07001562.hg.1	ENSG00000019991;	0.0152328	0.065977	FALSE
TC03000214.hg.1	ENSG00000168036;	0.0152328	0.065977	FALSE
TC07000643.hg.1	ENSG00000106366;	0.0152328	0.065977	FALSE
TC03001071.hg.1	ENSG00000114315;	0.0152328	0.065977	FALSE
TC01003638.hg.1	ENSG00000073756;	0.0152328	0.065977	FALSE
TC03002146.hg.1	ENSG00000189058;	0.0152328	0.065977	FALSE

TC03001071.hg.1	ENSG00000114315;	0.0152328	0.065977	FALSE
TC16000668.hg.1	ENSG00000103241;	0.0152328	0.065977	FALSE
TC03001071.hg.1	ENSG00000114315;	0.0152328	0.065977	FALSE
TC15000226.hg.1	ENSG00000166923;	0.0152328	0.065977	FALSE
TC01001188.hg.1;	ENSG00000143369;	0.0152421	0.065977	FALSE
TC02000041.hg.1	ENSG00000115738;	0.015349	0.0663726	FALSE
TC01002669.hg.1	ENSG00000157193;	0.0154553	0.0667652	FALSE
TC01003718.hg.1	ENSG00000133048;	0.015784	0.0681164	FALSE
TC02002606.hg.1	ENSG00000003436;	0.0159106	0.0685934	FALSE
TC01001736.hg.1	ENSG00000196352;	0.016136	0.0693663	FALSE
TC01000685.hg.1	ENSG00000162409;	0.016136	0.0693663	FALSE
TC07001293.hg.1	ENSG00000106483;	0.0161466	0.0693663	FALSE
TC01003638.hg.1	ENSG00000073756;	0.0161466	0.0693663	FALSE
TC01000783.hg.1	ENSG00000117069;	0.0164486	0.0705574	FALSE
TC01000685.hg.1	ENSG00000162409;	0.0165015	0.0707138	FALSE
TC01001624.hg.1	ENSG00000116741;	0.0166889	0.071445	FALSE
TC01000100.hg.1	ENSG00000049246;	0.0167649	0.0716988	FALSE
TC01000734.hg.1	ENSG00000118473;	0.0168928	0.0721737	FALSE
TC01000685.hg.1	ENSG00000162409;	0.016969	0.0724268	FALSE
TC01000100.hg.1	ENSG00000049246;	0.0170824	0.0728386	FALSE
TC01000685.hg.1	ENSG00000162409;	0.0172062	0.0731458	FALSE
TC06004073.hg.1	ENSG00000204520;	0.0174016	0.0731458	FALSE
TC01000904.hg.1	ENSG00000162692;	0.0174016	0.0731458	FALSE
TC11000182.hg.1;	ENSG00000148926;	0.0174016	0.0731458	FALSE
TC01000904.hg.1	ENSG00000162692;	0.0174016	0.0731458	FALSE
TC01001805.hg.1	ENSG00000092969;	0.0174016	0.0731458	FALSE
TC03001590.hg.1	ENSG00000184500;	0.0174495	0.0731458	FALSE
TC07000220.hg.1	ENSG00000164619;	0.0174535	0.0731458	FALSE
TC10001744.hg.1	ENSG00000148848;	0.0174535	0.0731458	FALSE
TC01001805.hg.1	ENSG00000092969;	0.0174535	0.0731458	FALSE
TC02002714.hg.1	ENSG00000163251;	0.0174535	0.0731458	FALSE
TC01001736.hg.1	ENSG00000196352;	0.0174535	0.0731458	FALSE
TC01001624.hg.1	ENSG00000116741;	0.0174535	0.0731458	FALSE
TC05001933.hg.1	ENSG00000113721;	0.0174535	0.0731458	FALSE
TC07001883.hg.1	ENSG00000085662;	0.0174535	0.0731458	FALSE
TC02001031.hg.1	ENSG00000152256;	0.0174535	0.0731458	FALSE
TC11002140.hg.1;	ENSG00000149256;	0.0174535	0.0731458	FALSE
TC01003638.hg.1	ENSG00000073756;	0.0174535	0.0731458	FALSE
TC10000986.hg.1	ENSG00000151632;	0.0174535	0.0731458	FALSE
TC06000087.hg.1	ENSG00000078401;	0.0174535	0.0731458	FALSE
TC01001624.hg.1	ENSG00000116741;	0.0174535	0.0731458	FALSE
TC01003638.hg.1	ENSG00000073756;	0.0174535	0.0731458	FALSE
TC03001255.hg.1	ENSG00000033867;	0.0174535	0.0731458	FALSE
TC01003778.hg.1	ENSG00000076356;	0.0174535	0.0731458	FALSE
TC07000328.hg.1	ENSG00000146648;	0.0174535	0.0731458	FALSE
TC02002292.hg.1	ENSG00000136720;	0.0174535	0.0731458	FALSE
TC02002624.hg.1	ENSG00000115415;	0.0174535	0.0731458	FALSE
TC01000685.hg.1	ENSG00000162409;	0.0181667	0.0752508	FALSE
TC01001805.hg.1	ENSG00000092969;	0.0181667	0.0752508	FALSE
TC05003423.hg.1	ENSG00000082196;	0.0181667	0.0752508	FALSE
TC03000214.hg.1	ENSG00000168036;	0.0181667	0.0752508	FALSE
TC04000408.hg.1	ENSG00000169429;	0.0182598	0.0754537	FALSE
TC01001624.hg.1	ENSG00000116741;	0.0182878	0.0754965	FALSE
TC03001071.hg.1	ENSG00000114315;	0.0184434	0.0760658	FALSE
TC01002669.hg.1	ENSG00000157193;	0.0187258	0.0771562	FALSE
TC04000408.hg.1	ENSG00000169429;	0.0188248	0.0772724	FALSE
TC01001907.hg.1	ENSG00000143641;	0.0188248	0.0772724	FALSE
TC01000685.hg.1	ENSG00000162409;	0.0188248	0.0772724	FALSE

TC02002714.hg.1	ENSG00000163251;	0.0188248	0.0772724	FALSE
TC01001607.hg.1	ENSG00000116711;I	0.0188443	0.0772724	FALSE
TC02000762.hg.1	ENSG00000163083;	0.0189308	0.0775157	FALSE
TC01002578.hg.1	ENSG00000117394;	0.0189308	0.0775157	FALSE
TC01002669.hg.1	ENSG00000157193;	0.0193341	0.0790539	FALSE
TC01001090.hg.1	ENSG00000117289;	0.0194179	0.0792828	FALSE
TC01000685.hg.1	ENSG00000162409;	0.0194179	0.0792828	FALSE
TC01003638.hg.1	ENSG00000073756;	0.0195134	0.0795209	FALSE
TC02001628.hg.1	ENSG00000084674;	0.0195134	0.0795209	FALSE
TC02001031.hg.1	ENSG00000152256;	0.0199202	0.0808708	FALSE
TC01001188.hg.1;	ENSG00000143369;	0.0199202	0.0808708	FALSE
TC07000220.hg.1	ENSG00000164619;	0.0199202	0.0808708	FALSE
TC02001628.hg.1	ENSG00000084674;	0.0199202	0.0808708	FALSE
TC01003129.hg.1	ENSG00000143140;	0.0199202	0.0808708	FALSE
TC03001071.hg.1	ENSG00000114315;	0.0199202	0.0808708	FALSE
TC01003778.hg.1	ENSG00000076356;	0.0201058	0.0813161	FALSE
TC03000214.hg.1	ENSG00000168036;	0.0201058	0.0813161	FALSE
TC02000762.hg.1	ENSG00000163083;	0.0201614	0.0814253	FALSE
TC03002146.hg.1	ENSG00000189058;	0.0202744	0.0818044	FALSE
TC01003638.hg.1	ENSG00000073756;	0.0205289	0.0827536	FALSE
TC01000783.hg.1	ENSG00000117069;	0.0205978	0.082953	FALSE
TC01001624.hg.1	ENSG00000116741;	0.0207258	0.0833902	FALSE
TC01000685.hg.1	ENSG00000162409;	0.0208084	0.0836439	FALSE
TC01002578.hg.1	ENSG00000117394;	0.0209245	0.0839925	FALSE
TC01002578.hg.1	ENSG00000117394;	0.0209245	0.0839925	FALSE
TC10001744.hg.1	ENSG00000148848;	0.0211693	0.0841914	FALSE
TC06000595.hg.1	ENSG00000112655;	0.0211693	0.0841914	FALSE
TC01000783.hg.1	ENSG00000117069;	0.0211693	0.0841914	FALSE
TC02001628.hg.1	ENSG00000084674;	0.0211693	0.0841914	FALSE
TC05001933.hg.1	ENSG00000113721;	0.0211693	0.0841914	FALSE
TC04001245.hg.1	ENSG00000145242;	0.0211693	0.0841914	FALSE
TC07000768.hg.1	ENSG00000174697;	0.0211693	0.0841914	FALSE
TC0X000943.hg.1	ENSG00000198947;	0.0211693	0.0841914	FALSE
TC02001100.hg.1;	ENSG00000138448;	0.0211693	0.0841914	FALSE
TC02002823.hg.1	ENSG00000135919;	0.0211693	0.0841914	FALSE
TC03000214.hg.1	ENSG00000168036;	0.0211693	0.0841914	FALSE
TC01003718.hg.1	ENSG00000133048;	0.0211693	0.0841914	FALSE
TC03000214.hg.1	ENSG00000168036;	0.0211693	0.0841914	FALSE
TC01001736.hg.1	ENSG00000196352;	0.0212593	0.0841914	FALSE
TC01001188.hg.1;	ENSG00000143369;	0.0214355	0.0848111	FALSE
TC01001473.hg.1	ENSG00000143153;	0.0216729	0.0856177	FALSE
TC01000100.hg.1	ENSG00000049246;	0.0216794	0.0856177	FALSE
TC01001704.hg.1	ENSG00000163531;	0.0217999	0.0860022	FALSE
TC06004073.hg.1	ENSG00000204520;	0.0218284	0.0860022	FALSE
TC01000685.hg.1	ENSG00000162409;	0.0218601	0.0860022	FALSE
TC01002669.hg.1	ENSG00000157193;	0.0218672	0.0860022	FALSE
TC02002714.hg.1	ENSG00000163251;	0.0218672	0.0860022	FALSE
TC01001704.hg.1	ENSG00000163531;	0.0221389	0.0861295	FALSE
TC01001095.hg.1	ENSG00000143127;	0.0222023	0.0861295	FALSE
TC08000482.hg.1	ENSG00000121039;	0.0222375	0.0861295	FALSE
TC09001326.hg.1	ENSG00000169071;	0.0222375	0.0861295	FALSE
TC02002765.hg.1	ENSG00000079308;	0.0222375	0.0861295	FALSE
TC02001100.hg.1;	ENSG00000138448;	0.0222375	0.0861295	FALSE
TC02001628.hg.1	ENSG00000084674;	0.0222375	0.0861295	FALSE
TC04000729.hg.1	ENSG00000151617;	0.0222375	0.0861295	FALSE
TC01003129.hg.1	ENSG00000143140;	0.0222375	0.0861295	FALSE
TC04001509.hg.1	ENSG00000173376;	0.0222375	0.0861295	FALSE
TC10002924.hg.1	ENSG00000187134;	0.0222375	0.0861295	FALSE

TC03002146.hg.1	ENSG00000189058;	0.0222375	0.0861295	FALSE
TC16000668.hg.1	ENSG00000103241;	0.0222375	0.0861295	FALSE
TC02001100.hg.1;	ENSG00000138448;	0.0222375	0.0861295	FALSE
TC16000668.hg.1	ENSG00000103241;	0.0222375	0.0861295	FALSE
TC11000707.hg.1;	ENSG00000162337;	0.0222375	0.0861295	FALSE
TC01003129.hg.1	ENSG00000143140;	0.0222375	0.0861295	FALSE
TC07001559.hg.1	ENSG00000075223;	0.0222375	0.0861295	FALSE
TC04000699.hg.1	ENSG00000109458;	0.0222375	0.0861295	FALSE
TC03000846.hg.1	ENSG00000196549;	0.0222375	0.0861295	FALSE
TC05001933.hg.1	ENSG00000113721;	0.0222375	0.0861295	FALSE
TC03001071.hg.1	ENSG00000114315;	0.0222375	0.0861295	FALSE
TC04002931.hg.1	ENSG00000134853;	0.0222375	0.0861295	FALSE
TC01003129.hg.1	ENSG00000143140;	0.0222375	0.0861295	FALSE
TC01003129.hg.1	ENSG00000143140;	0.0222375	0.0861295	FALSE
TC01003129.hg.1	ENSG00000143140;	0.0222375	0.0861295	FALSE
TC07001562.hg.1	ENSG00000019991;	0.0222375	0.0861295	FALSE
TC07001562.hg.1	ENSG00000019991;	0.0222375	0.0861295	FALSE
TC10001711.hg.1;	ENSG00000066468;	0.0222375	0.0861295	FALSE
TC01001805.hg.1	ENSG00000092969;	0.0225857	0.0861295	FALSE
TC01000685.hg.1	ENSG00000162409;	0.0226519	0.0861295	FALSE
TC01002166.hg.1	ENSG00000116285;	0.0226519	0.0861295	FALSE
TC01001624.hg.1	ENSG00000116741;	0.0226519	0.0861295	FALSE
TC01001805.hg.1	ENSG00000092969;	0.0226519	0.0861295	FALSE
TC05001752.hg.1	ENSG00000138829;	0.0226519	0.0861295	FALSE
TC01002166.hg.1	ENSG00000116285;	0.0226519	0.0861295	FALSE
TC01001473.hg.1	ENSG00000143153;	0.022654	0.0861295	FALSE
TC01001090.hg.1	ENSG00000117289;	0.0231109	0.0877887	FALSE
TC01000100.hg.1	ENSG00000049246;	0.0235084	0.0892029	FALSE
TC01001188.hg.1;	ENSG00000143369;	0.0235353	0.0892029	FALSE
TC01001473.hg.1	ENSG00000143153;	0.0235353	0.0892029	FALSE
TC06000087.hg.1	ENSG00000078401;	0.0236404	0.0894824	FALSE
TC02002823.hg.1	ENSG00000135919;	0.0237297	0.089741	FALSE
TC01002669.hg.1	ENSG00000157193;	0.0244593	0.0924185	FALSE
TC01000789.hg.1	ENSG00000162614;	0.024522	0.092574	FALSE
TC07001332.hg.1	ENSG00000164708;	0.0245677	0.0926646	FALSE
TC01001090.hg.1	ENSG00000117289;	0.0249912	0.094179	FALSE
TC01000904.hg.1	ENSG00000162692;	0.0250857	0.0944105	FALSE
TC03000214.hg.1	ENSG00000168036;	0.0250857	0.0944105	FALSE
TC04002931.hg.1	ENSG00000134853;	0.0251427	0.0945002	FALSE
TC03000846.hg.1	ENSG00000196549;	0.025283	0.0945711	FALSE
TC12001324.hg.1	ENSG00000123095;	0.025283	0.0945711	FALSE
TC02001100.hg.1;	ENSG00000138448;	0.025283	0.0945711	FALSE
TC06000087.hg.1	ENSG00000078401;	0.025283	0.0945711	FALSE
TC02000041.hg.1	ENSG00000115738;	0.025283	0.0945711	FALSE
TC04000432.hg.1	ENSG00000156234;	0.025283	0.0945711	FALSE
TC10000811.hg.1;	ENSG00000150594;	0.025283	0.0945711	FALSE
TC07001562.hg.1	ENSG00000019991;	0.025283	0.0945711	FALSE
TC03000214.hg.1	ENSG00000168036;	0.025283	0.0945711	FALSE
TC04000274.hg.1	ENSG00000145246;	0.025283	0.0945711	FALSE
TC01002616.hg.1	ENSG00000117461;	0.0256005	0.095117	FALSE
TC03002146.hg.1	ENSG00000189058;	0.0256005	0.095117	FALSE
TC02001100.hg.1;	ENSG00000138448;	0.0256005	0.095117	FALSE
TC02002624.hg.1	ENSG00000115415;	0.0256005	0.095117	FALSE
TC01000703.hg.1	ENSG00000132849;	0.0256956	0.095117	FALSE
TC01003638.hg.1	ENSG00000073756;	0.0256956	0.095117	FALSE
TC02002624.hg.1	ENSG00000115415;	0.0256956	0.095117	FALSE
TC01000728.hg.1	ENSG00000162433;	0.0256956	0.095117	FALSE
TC01000685.hg.1	ENSG00000162409;	0.0256956	0.095117	FALSE

TC01001624.hg.1	ENSG00000116741;	0.0256956	0.095117	FALSE
TC02000281.hg.1	ENSG00000116016;	0.0258358	0.0953475	FALSE
TC01000789.hg.1	ENSG00000162614;	0.0264071	0.0973718	FALSE
TC01003638.hg.1	ENSG00000073756;	0.0265798	0.0979245	FALSE
TC01001624.hg.1	ENSG00000116741;	0.0267103	0.0982784	FALSE
TC04001084.hg.1	ENSG00000109819;	0.0267103	0.0982784	FALSE
TC01001607.hg.1	ENSG00000116711;I	0.0268493	0.0986627	FALSE
TC02000041.hg.1	ENSG00000115738;	0.0271333	0.0996207	FALSE
TC01001736.hg.1	ENSG00000196352;	0.0276075	0.101275	FALSE
TC01001805.hg.1	ENSG00000092969;	0.0277668	0.1017286	FALSE
TC01001624.hg.1	ENSG00000116741;	0.0277668	0.1017286	FALSE
TC01000728.hg.1	ENSG00000162433;	0.0278316	0.1018353	FALSE
TC01000685.hg.1	ENSG00000162409;	0.0278795	0.1019233	FALSE
TC04000253.hg.1	ENSG00000154277;	0.0283446	0.1034553	FALSE
TC02002606.hg.1	ENSG00000003436;	0.0285281	0.1034553	FALSE
TC03000214.hg.1	ENSG00000168036;	0.0286955	0.1034553	FALSE
TC01003638.hg.1	ENSG00000073756;	0.0286955	0.1034553	FALSE
TC03000214.hg.1	ENSG00000168036;	0.0287692	0.1034553	FALSE
TC01000685.hg.1	ENSG00000162409;	0.0287692	0.1034553	FALSE
TC01001805.hg.1	ENSG00000092969;	0.0287692	0.1034553	FALSE
TC01001805.hg.1	ENSG00000092969;	0.0287692	0.1034553	FALSE
TC03000214.hg.1	ENSG00000168036;	0.0287692	0.1034553	FALSE
TC02000041.hg.1	ENSG00000115738;	0.0287692	0.1034553	FALSE
TC02002714.hg.1	ENSG00000163251;	0.0287692	0.1034553	FALSE
TC01002954.hg.1	ENSG00000134243;	0.0287692	0.1034553	FALSE
TC03000214.hg.1	ENSG00000168036;	0.0287692	0.1034553	FALSE
TC04000408.hg.1	ENSG00000169429;	0.0287692	0.1034553	FALSE
TC03000214.hg.1	ENSG00000168036;	0.0287692	0.1034553	FALSE
TC03002146.hg.1	ENSG00000189058;	0.0287692	0.1034553	FALSE
TC01000685.hg.1	ENSG00000162409;	0.0287805	0.1034553	FALSE
TC02001031.hg.1	ENSG00000152256;	0.0287805	0.1034553	FALSE
TC02001031.hg.1	ENSG00000152256;	0.0287805	0.1034553	FALSE
TC01000685.hg.1	ENSG00000162409;	0.0287819	0.1034553	FALSE
TC01000728.hg.1	ENSG00000162433;	0.0291253	0.104602	FALSE
TC01001805.hg.1	ENSG00000092969;	0.0292476	0.1049531	FALSE
TC06001089.hg.1	ENSG00000111981;I	0.0295489	0.1058384	FALSE
TC10001744.hg.1	ENSG00000148848;	0.0297937	0.1058384	FALSE
TC08000482.hg.1	ENSG00000121039;	0.0297937	0.1058384	FALSE
TC10000811.hg.1;	ENSG00000150594;	0.0297937	0.1058384	FALSE
TC02001628.hg.1	ENSG00000084674;	0.0297937	0.1058384	FALSE
TC08000482.hg.1	ENSG00000121039;	0.0297937	0.1058384	FALSE
TC02002624.hg.1	ENSG00000115415;	0.0297937	0.1058384	FALSE
TC06000595.hg.1	ENSG00000112655;	0.0297937	0.1058384	FALSE
TC07001618.hg.1	ENSG00000004799;	0.0297937	0.1058384	FALSE
TC01000873.hg.1	ENSG00000143036;	0.0299454	0.1058384	FALSE
TC15000226.hg.1	ENSG00000166923;	0.030302	0.1058384	FALSE
TC01000972.hg.1	ENSG00000064886;	0.030302	0.1058384	FALSE
TC01000972.hg.1	ENSG00000064886;	0.030302	0.1058384	FALSE
TC01003638.hg.1	ENSG00000073756;	0.030302	0.1058384	FALSE
TC02001628.hg.1	ENSG00000084674;	0.030302	0.1058384	FALSE
TC06000087.hg.1	ENSG00000078401;	0.030302	0.1058384	FALSE
TC02001031.hg.1	ENSG00000152256;	0.030302	0.1058384	FALSE
TC12001324.hg.1	ENSG00000123095;	0.030302	0.1058384	FALSE
TC06000087.hg.1	ENSG00000078401;	0.030302	0.1058384	FALSE
TC08000482.hg.1	ENSG00000121039;	0.030302	0.1058384	FALSE
TC07001630.hg.1	ENSG00000070669;	0.030302	0.1058384	FALSE
TC01001805.hg.1	ENSG00000092969;	0.030302	0.1058384	FALSE
TC06000595.hg.1	ENSG00000112655;	0.030302	0.1058384	FALSE

TC02000762.hg.1	ENSG00000163083;	0.030302	0.1058384	FALSE
TC03000214.hg.1	ENSG00000168036;	0.030302	0.1058384	FALSE
TC01001805.hg.1	ENSG00000092969;	0.030302	0.1058384	FALSE
TC01003129.hg.1	ENSG00000143140;	0.030302	0.1058384	FALSE
TC01003129.hg.1	ENSG00000143140;	0.030302	0.1058384	FALSE
TC08000482.hg.1	ENSG00000121039;	0.030302	0.1058384	FALSE
TC03000917.hg.1	ENSG00000136603;	0.030302	0.1058384	FALSE
TC03001071.hg.1	ENSG00000114315;	0.030302	0.1058384	FALSE
TC03000214.hg.1	ENSG00000168036;	0.030302	0.1058384	FALSE
TC03000214.hg.1	ENSG00000168036;	0.030302	0.1058384	FALSE
TC12001324.hg.1	ENSG00000123095;	0.030302	0.1058384	FALSE
TC03001888.hg.1	ENSG00000169908;	0.030302	0.1058384	FALSE
TC01000100.hg.1	ENSG00000049246;	0.0303843	0.1058384	FALSE
TC01001607.hg.1	ENSG00000116711;I	0.030497	0.106071	FALSE
TC01001805.hg.1	ENSG00000092969;	0.0305007	0.106071	FALSE
TC01001095.hg.1	ENSG00000143127;	0.0306256	0.1064189	FALSE
TC01001624.hg.1	ENSG00000116741;	0.0306605	0.1064537	FALSE
TC01001624.hg.1	ENSG00000116741;	0.0307185	0.1065689	FALSE
TC03000214.hg.1	ENSG00000168036;	0.0314178	0.1089065	FALSE
TC01000728.hg.1	ENSG00000162433;	0.031841	0.1102842	FALSE
TC06004073.hg.1	ENSG00000204520;	0.0321603	0.1112102	FALSE
TC02002714.hg.1	ENSG00000163251;	0.0321603	0.1112102	FALSE
TC08000482.hg.1	ENSG00000121039;	0.0321603	0.1112102	FALSE
TC03001071.hg.1	ENSG00000114315;	0.0322611	0.111334	FALSE
TC01001805.hg.1	ENSG00000092969;	0.0322611	0.111334	FALSE
TC02000762.hg.1	ENSG00000163083;	0.032641	0.1124337	FALSE
TC01001090.hg.1	ENSG00000117289;	0.0326454	0.1124337	FALSE
TC01000783.hg.1	ENSG00000117069;	0.0333739	0.1148501	FALSE
TC02001963.hg.1	ENSG00000163235;	0.0336588	0.1156448	FALSE
TC02001963.hg.1	ENSG00000163235;	0.0336588	0.1156448	FALSE
TC02002624.hg.1	ENSG00000115415;	0.0336588	0.1156448	FALSE
TC01003129.hg.1	ENSG00000143140;	0.0338114	0.1159829	FALSE
TC01002706.hg.1	ENSG00000184292;	0.033922	0.1162689	FALSE
TC01001704.hg.1	ENSG00000163531;	0.0343412	0.1176117	FALSE
TC01000394.hg.1	ENSG00000159023;	0.0344442	0.1178701	FALSE
TC04000408.hg.1	ENSG00000169429;	0.0346312	0.1179503	FALSE
TC01000100.hg.1	ENSG00000049246;	0.0346312	0.1179503	FALSE
TC04001245.hg.1	ENSG00000145242;	0.0346985	0.1179503	FALSE
TC05001933.hg.1	ENSG00000113721;	0.0346985	0.1179503	FALSE
TC05000054.hg.1	ENSG00000078295;	0.0346985	0.1179503	FALSE
TC07000137.hg.1	ENSG00000136244;	0.0346985	0.1179503	FALSE
TC06000608.hg.1	ENSG00000112715;	0.0346985	0.1179503	FALSE
TC01001805.hg.1	ENSG00000092969;	0.0346985	0.1179503	FALSE
TC02002823.hg.1	ENSG00000135919;	0.0347431	0.1179503	FALSE
TC01001090.hg.1	ENSG00000117289;	0.0347431	0.1179503	FALSE
TC01001624.hg.1	ENSG00000116741;	0.0347431	0.1179503	FALSE
TC02002714.hg.1	ENSG00000163251;	0.0351238	0.1189124	FALSE
TC02001867.hg.1	ENSG00000115380;	0.0351238	0.1189124	FALSE
TC01000685.hg.1	ENSG00000162409;	0.0351238	0.1189124	FALSE
TC06000595.hg.1	ENSG00000112655;	0.0351238	0.1189124	FALSE
TC01002847.hg.1	ENSG00000117228;	0.0355975	0.1202784	FALSE
TC01000100.hg.1	ENSG00000049246;	0.0357061	0.1205503	FALSE
TC01000685.hg.1	ENSG00000162409;	0.0357755	0.1206419	FALSE
TC01001188.hg.1;	ENSG00000143369;	0.0357755	0.1206419	FALSE
TC02000041.hg.1	ENSG00000115738;	0.0362605	0.1221332	FALSE
TC01001736.hg.1	ENSG00000196352;	0.0371416	0.1248064	FALSE
TC02002624.hg.1	ENSG00000115415;	0.0371416	0.1248064	FALSE
TC01000904.hg.1	ENSG00000162692;	0.0371416	0.1248064	FALSE

TC01003638.hg.1	ENSG00000073756;	0.0375551	0.1260967	FALSE
TC01003638.hg.1	ENSG00000073756;	0.0381384	0.1278048	FALSE
TC02000041.hg.1	ENSG00000115738;	0.0381384	0.1278048	FALSE
TC05001110.hg.1;	ENSG00000153395;	0.0381384	0.1278048	FALSE
TC05001110.hg.1;	ENSG00000153395;	0.0381384	0.1278048	FALSE
TC01001607.hg.1	ENSG00000116711;l	0.0385591	0.1283841	FALSE
TC01001607.hg.1	ENSG00000116711;l	0.0385591	0.1283841	FALSE
TC01001473.hg.1	ENSG00000143153;	0.0389593	0.1283841	FALSE
TC03000214.hg.1	ENSG00000168036;	0.039212	0.1283841	FALSE
TC04002931.hg.1	ENSG00000134853;	0.0393287	0.1283841	FALSE
TC05000943.hg.1	ENSG00000156427;	0.0393287	0.1283841	FALSE
TC01003129.hg.1	ENSG00000143140;	0.0393287	0.1283841	FALSE
TC01002166.hg.1	ENSG00000116285;	0.0393287	0.1283841	FALSE
TC02002823.hg.1	ENSG00000135919;	0.0393287	0.1283841	FALSE
TC05000165.hg.1	ENSG00000079215;	0.0393287	0.1283841	FALSE
TC10001398.hg.1	ENSG00000122884;	0.0393287	0.1283841	FALSE
TC07000328.hg.1	ENSG00000146648;	0.0393287	0.1283841	FALSE
TC02000762.hg.1	ENSG00000163083;	0.0393287	0.1283841	FALSE
TC02001867.hg.1	ENSG00000115380;	0.0393287	0.1283841	FALSE
TC07000328.hg.1	ENSG00000146648;	0.0393287	0.1283841	FALSE
TC15000226.hg.1	ENSG00000166923;	0.0393287	0.1283841	FALSE
TC07000004.hg.1	ENSG00000177706;	0.0393287	0.1283841	FALSE
TC06002168.hg.1	ENSG00000164442;	0.0393287	0.1283841	FALSE
TC02002823.hg.1	ENSG00000135919;	0.0393287	0.1283841	FALSE
TC02000281.hg.1	ENSG00000116016;	0.0393287	0.1283841	FALSE
TC02000762.hg.1	ENSG00000163083;	0.0393287	0.1283841	FALSE
TC01003497.hg.1	ENSG00000117479;	0.0393287	0.1283841	FALSE
TC02000286.hg.1	ENSG00000171150;	0.0393287	0.1283841	FALSE
TC03000577.hg.1	ENSG00000144857;	0.0393287	0.1283841	FALSE
TC06000945.hg.1	ENSG00000152661;	0.0393287	0.1283841	FALSE
TC02002823.hg.1	ENSG00000135919;	0.0393287	0.1283841	FALSE
TC06000945.hg.1	ENSG00000152661;	0.0393287	0.1283841	FALSE
TC02000281.hg.1	ENSG00000116016;	0.0393287	0.1283841	FALSE
TC07001562.hg.1	ENSG00000019991;	0.0393287	0.1283841	FALSE
TC06000595.hg.1	ENSG00000112655;	0.0393287	0.1283841	FALSE
TC06000595.hg.1	ENSG00000112655;	0.0393287	0.1283841	FALSE
TC16000473.hg.1	ENSG00000198417;	0.0393287	0.1283841	FALSE
TC02001628.hg.1	ENSG00000084674;	0.0393287	0.1283841	FALSE
TC07001552.hg.1	ENSG00000187391;	0.0393287	0.1283841	FALSE
TC03000214.hg.1	ENSG00000168036;	0.0393287	0.1283841	FALSE
TC02002624.hg.1	ENSG00000115415;	0.0393287	0.1283841	FALSE
TC02002624.hg.1	ENSG00000115415;	0.0393287	0.1283841	FALSE
TC03002146.hg.1	ENSG00000189058;	0.0393287	0.1283841	FALSE
TC01000972.hg.1	ENSG00000064886;	0.0393287	0.1283841	FALSE
TC06000945.hg.1	ENSG00000152661;	0.0393287	0.1283841	FALSE
TC01000972.hg.1	ENSG00000064886;	0.0396158	0.1283841	FALSE
TC02000511.hg.1;	ENSG00000168906;	0.0396158	0.1283841	FALSE
TC01002616.hg.1	ENSG00000117461;	0.0396158	0.1283841	FALSE
TC02000041.hg.1	ENSG00000115738;	0.0397654	0.1286741	FALSE
TC02002292.hg.1	ENSG00000136720;	0.0399927	0.1288252	FALSE
TC02001031.hg.1	ENSG00000152256;	0.0399927	0.1288252	FALSE
TC01001624.hg.1	ENSG00000116741;	0.0399927	0.1288252	FALSE
TC01001805.hg.1	ENSG00000092969;	0.0399927	0.1288252	FALSE
TC10000811.hg.1;	ENSG00000150594;	0.0399927	0.1288252	FALSE
TC11000238.hg.1;	ENSG00000173432;	0.0399927	0.1288252	FALSE
TC08001275.hg.1	ENSG00000172817;	0.0399927	0.1288252	FALSE
TC03000214.hg.1	ENSG00000168036;	0.0399927	0.1288252	FALSE
TC19000191.hg.1	ENSG00000130164;	0.0399927	0.1288252	FALSE

TC01001188.hg.1;	ENSG00000143369;	0.0399927	0.1288252	FALSE
TC12001324.hg.1	ENSG00000123095;	0.0399927	0.1288252	FALSE
TC01001736.hg.1	ENSG00000196352;	0.0404248	0.1295832	FALSE
TC01003638.hg.1	ENSG00000073756;	0.0404248	0.1295832	FALSE
TC01003638.hg.1	ENSG00000073756;	0.0408458	0.1307859	FALSE
TC03000214.hg.1	ENSG00000168036;	0.0413065	0.1319649	FALSE
TC01003129.hg.1	ENSG00000143140;	0.0413065	0.1319649	FALSE
TC04000274.hg.1	ENSG00000145246;	0.0413065	0.1319649	FALSE
TC02001867.hg.1	ENSG00000115380;	0.0413065	0.1319649	FALSE
TC02002808.hg.1	ENSG00000116106;	0.0413065	0.1319649	FALSE
TC03000214.hg.1	ENSG00000168036;	0.0417431	0.1330619	FALSE
TC01001473.hg.1	ENSG00000143153;	0.0425039	0.1347347	FALSE
TC01001473.hg.1	ENSG00000143153;	0.0425039	0.1347347	FALSE
TC01001473.hg.1	ENSG00000143153;	0.0425039	0.1347347	FALSE
TC01001473.hg.1	ENSG00000143153;	0.0425039	0.1347347	FALSE
TC01001473.hg.1	ENSG00000143153;	0.0425039	0.1347347	FALSE
TC01001473.hg.1	ENSG00000143153;	0.0425039	0.1347347	FALSE
TC01001473.hg.1	ENSG00000143153;	0.0425039	0.1347347	FALSE
TC01001473.hg.1	ENSG00000143153;	0.0425039	0.1347347	FALSE
TC02001031.hg.1	ENSG00000152256;	0.0425039	0.1347347	FALSE
TC01003129.hg.1	ENSG00000143140;	0.0425039	0.1347347	FALSE
TC01001473.hg.1	ENSG00000143153;	0.0425039	0.1347347	FALSE
TC01001473.hg.1	ENSG00000143153;	0.0425039	0.1347347	FALSE
TC01001473.hg.1	ENSG00000143153;	0.0425039	0.1347347	FALSE
TC01001473.hg.1	ENSG00000143153;	0.0425039	0.1347347	FALSE
TC01001805.hg.1	ENSG00000092969;	0.0427868	0.1348824	FALSE
TC08000845.hg.1	ENSG00000187786;	0.0436817	0.1375516	FALSE
TC04000274.hg.1	ENSG00000145246;	0.0436817	0.1375516	FALSE
TC04000313.hg.1	ENSG00000157404;	0.0440409	0.1384789	FALSE
TC02002624.hg.1	ENSG00000115415;	0.0440409	0.1384789	FALSE
TC02001963.hg.1	ENSG00000163235;	0.0449483	0.141073	FALSE
TC01000783.hg.1	ENSG00000117069;	0.0449483	0.141073	FALSE
TC01001095.hg.1	ENSG00000143127;	0.0449483	0.141073	FALSE
TC01002578.hg.1	ENSG00000117394;	0.045358	0.1420466	FALSE
TC01001704.hg.1	ENSG00000163531;	0.045358	0.1420466	FALSE
TC05003423.hg.1	ENSG00000082196;	0.045358	0.1420466	FALSE
TC02002808.hg.1	ENSG00000116106;	0.0456697	0.1424496	FALSE
TC05000165.hg.1	ENSG00000079215;	0.0456697	0.1424496	FALSE
TC03001255.hg.1	ENSG00000033867;	0.0456697	0.1424496	FALSE
TC01001805.hg.1	ENSG00000092969;	0.0456697	0.1424496	FALSE
TC07001552.hg.1	ENSG00000187391;	0.0456697	0.1424496	FALSE
TC02001031.hg.1	ENSG00000152256;	0.0456697	0.1424496	FALSE
TC01001607.hg.1	ENSG00000116711;I	0.0456697	0.1424496	FALSE
TC01003638.hg.1	ENSG00000073756;	0.0456697	0.1424496	FALSE
TC01000904.hg.1	ENSG00000162692;	0.0461124	0.1433603	FALSE
TC01000685.hg.1	ENSG00000162409;	0.0463345	0.1439463	FALSE
TC01001704.hg.1	ENSG00000163531;	0.0465927	0.1446212	FALSE
TC01000685.hg.1	ENSG00000162409;	0.0466193	0.1446212	FALSE
TC03000214.hg.1	ENSG00000168036;	0.0479729	0.1484879	FALSE
TC01001907.hg.1	ENSG00000143641;	0.0479729	0.1484879	FALSE
TC02000041.hg.1	ENSG00000115738;	0.0479729	0.1484879	FALSE
TC04002931.hg.1	ENSG00000134853;	0.0479729	0.1484879	FALSE
TC03001362.hg.1	ENSG00000160808;	0.0481067	0.1484879	FALSE
TC01001624.hg.1	ENSG00000116741;	0.0481067	0.1484879	FALSE
TC01002669.hg.1	ENSG00000157193;	0.0481067	0.1484879	FALSE
TC01003129.hg.1	ENSG00000143140;	0.0481433	0.1484879	FALSE
TC02000286.hg.1	ENSG00000171150;	0.0483287	0.1488989	FALSE
TC06000595.hg.1	ENSG00000112655;	0.0483287	0.1488989	FALSE

TC01006336.hg.1	ENSG00000162706;	0.0485867	0.1495324	FALSE
TC01001188.hg.1;	ENSG00000143369;	0.0487105	0.1496183	FALSE
TC01003129.hg.1	ENSG00000143140;	0.0492262	0.1496183	FALSE
TC01003129.hg.1	ENSG00000143140;	0.0492262	0.1496183	FALSE
TC02001750.hg.1	ENSG00000138061;	0.0492262	0.1496183	FALSE
TC02002823.hg.1	ENSG00000135919;	0.0492262	0.1496183	FALSE
TC08000482.hg.1	ENSG00000121039;	0.0492262	0.1496183	FALSE
TC04000173.hg.1	ENSG00000109610;	0.0492262	0.1496183	FALSE
TC07000328.hg.1	ENSG00000146648;	0.0492262	0.1496183	FALSE
TC06001027.hg.1	ENSG00000118503;	0.0492262	0.1496183	FALSE
TC07001552.hg.1	ENSG00000187391;	0.0492262	0.1496183	FALSE
TC07000643.hg.1	ENSG00000106366;	0.0492262	0.1496183	FALSE
TC03000214.hg.1	ENSG00000168036;	0.0492262	0.1496183	FALSE
TC05000914.hg.1	ENSG00000145934;	0.0492262	0.1496183	FALSE
TC07000768.hg.1	ENSG00000174697;	0.0492262	0.1496183	FALSE
TC02001963.hg.1	ENSG00000163235;	0.0492262	0.1496183	FALSE
TC06002168.hg.1	ENSG00000164442;	0.0492262	0.1496183	FALSE
TC04002931.hg.1	ENSG00000134853;	0.0492262	0.1496183	FALSE
TC01000685.hg.1	ENSG00000162409;	0.0492262	0.1496183	FALSE
TC01001624.hg.1	ENSG00000116741;	0.0492262	0.1496183	FALSE
TC03000214.hg.1	ENSG00000168036;	0.0492262	0.1496183	FALSE
TC15000226.hg.1	ENSG00000166923;	0.0492262	0.1496183	FALSE
TC10001711.hg.1;	ENSG00000066468;	0.0492262	0.1496183	FALSE
TC04000173.hg.1	ENSG00000109610;	0.0492262	0.1496183	FALSE
TC03000214.hg.1	ENSG00000168036;	0.0492262	0.1496183	FALSE
TC01003129.hg.1	ENSG00000143140;	0.0492262	0.1496183	FALSE
TC01003129.hg.1	ENSG00000143140;	0.0492262	0.1496183	FALSE
TC01003129.hg.1	ENSG00000143140;	0.0492262	0.1496183	FALSE
TC06000595.hg.1	ENSG00000112655;	0.0492262	0.1496183	FALSE
TC06000595.hg.1	ENSG00000112655;	0.0492262	0.1496183	FALSE
TC03001071.hg.1	ENSG00000114315;	0.0492262	0.1496183	FALSE
TC01002669.hg.1	ENSG00000157193;	0.0492262	0.1496183	FALSE
TC15000226.hg.1	ENSG00000166923;	0.0492262	0.1496183	FALSE
TC10001571.hg.1	ENSG00000120057;	0.0492262	0.1496183	FALSE
TC01001473.hg.1	ENSG00000143153;	0.0500362	0.1503192	FALSE
TC01001188.hg.1;	ENSG00000143369;	0.0514166	0.1537643	FALSE
TC01001704.hg.1	ENSG00000163531;	0.0514166	0.1537643	FALSE
TC01001704.hg.1	ENSG00000163531;	0.0514166	0.1537643	FALSE
TC06004073.hg.1	ENSG00000204520;	0.0514166	0.1537643	FALSE
TC02002714.hg.1	ENSG00000163251;	0.0514166	0.1537643	FALSE
TC02000041.hg.1	ENSG00000115738;	0.0514166	0.1537643	FALSE
TC02000041.hg.1	ENSG00000115738;	0.0514633	0.1537643	FALSE
TC06001772.hg.1	ENSG00000146233;	0.0517217	0.1537643	FALSE
TC02000041.hg.1	ENSG00000115738;	0.0517217	0.1537643	FALSE
TC05000619.hg.1	ENSG00000198108;	0.0517217	0.1537643	FALSE
TC05000943.hg.1	ENSG00000156427;	0.0517217	0.1537643	FALSE
TC01001624.hg.1	ENSG00000116741;	0.0517217	0.1537643	FALSE
TC03001071.hg.1	ENSG00000114315;	0.0517217	0.1537643	FALSE
TC01002166.hg.1	ENSG00000116285;	0.0517217	0.1537643	FALSE
TC06004141.hg.1	ENSG00000112096;	0.0517217	0.1537643	FALSE
TC08001275.hg.1	ENSG00000172817;	0.0517217	0.1537643	FALSE
TC01000904.hg.1	ENSG00000162692;	0.0517217	0.1537643	FALSE
TC01000904.hg.1	ENSG00000162692;	0.0517217	0.1537643	FALSE
TC01003129.hg.1	ENSG00000143140;	0.0517217	0.1537643	FALSE
TC04000408.hg.1	ENSG00000169429;	0.0517217	0.1537643	FALSE
TC01002706.hg.1	ENSG00000184292;	0.0517217	0.1537643	FALSE
TC01000972.hg.1	ENSG00000064886;	0.0517217	0.1537643	FALSE
TC03000631.hg.1	ENSG00000114023;	0.0524886	0.1550215	FALSE

TC05000165.hg.1	ENSG00000079215;	0.0524886	0.1550215	FALSE
TC04000432.hg.1	ENSG00000156234;	0.0524886	0.1550215	FALSE
TC04002931.hg.1	ENSG00000134853;	0.0524886	0.1550215	FALSE
TC01002669.hg.1	ENSG00000157193;	0.0546223	0.1609902	FALSE
TC01000734.hg.1	ENSG00000118473;	0.0546223	0.1609902	FALSE
TC01001704.hg.1	ENSG00000163531;	0.0550008	0.1619383	FALSE
TC04000253.hg.1	ENSG00000154277;	0.0555296	0.163383	FALSE
TC01002706.hg.1	ENSG00000184292;	0.0560145	0.1646965	FALSE
TC01001704.hg.1	ENSG00000163531;	0.0571235	0.167842	FALSE
TC02000466.hg.1	ENSG00000159399;	0.0572274	0.1679168	FALSE
TC03001071.hg.1	ENSG00000114315;	0.0572274	0.1679168	FALSE
TC01003638.hg.1	ENSG00000073756;	0.0572274	0.1679168	FALSE
TC01001805.hg.1	ENSG00000092969;	0.0575457	0.1686197	FALSE
TC03000214.hg.1	ENSG00000168036;	0.0577324	0.1688567	FALSE
TC03001071.hg.1	ENSG00000114315;	0.0579463	0.1688567	FALSE
TC04000173.hg.1	ENSG00000109610;	0.0579463	0.1688567	FALSE
TC01000685.hg.1	ENSG00000162409;	0.0580585	0.1688567	FALSE
TC01000685.hg.1	ENSG00000162409;	0.0580585	0.1688567	FALSE
TC01003638.hg.1	ENSG00000073756;	0.0581084	0.1688567	FALSE
TC03000846.hg.1	ENSG00000196549;	0.0581394	0.1688567	FALSE
TC05000165.hg.1	ENSG00000079215;	0.0581394	0.1688567	FALSE
TC08001567.hg.1	ENSG00000170961;	0.0581394	0.1688567	FALSE
TC06002168.hg.1	ENSG00000164442;	0.0581394	0.1688567	FALSE
TC02001031.hg.1	ENSG00000152256;	0.0581394	0.1688567	FALSE
TC02000082.hg.1	ENSG00000071575;	0.0581394	0.1688567	FALSE
TC03001071.hg.1	ENSG00000114315;	0.0581394	0.1688567	FALSE
TC04000313.hg.1	ENSG00000157404;	0.0581394	0.1688567	FALSE
TC03000214.hg.1	ENSG00000168036;	0.0581394	0.1688567	FALSE
TC11000238.hg.1;	ENSG00000173432;	0.0581394	0.1688567	FALSE
TC01002932.hg.1	ENSG00000060718;	0.0581394	0.1688567	FALSE
TC03002146.hg.1	ENSG00000189058;	0.0581394	0.1688567	FALSE
TC03002146.hg.1	ENSG00000189058;	0.0581394	0.1688567	FALSE
TC03000214.hg.1	ENSG00000168036;	0.058788	0.1699333	FALSE
TC03002146.hg.1	ENSG00000189058;	0.0594749	0.1705987	FALSE
TC03000013.hg.1	ENSG00000150995;	0.0594749	0.1705987	FALSE
TC03000214.hg.1	ENSG00000168036;	0.0594749	0.1705987	FALSE
TC05003423.hg.1	ENSG00000082196;	0.0594749	0.1705987	FALSE
TC06001015.hg.1	ENSG00000171408;	0.0599089	0.1705987	FALSE
TC08000127.hg.1	ENSG00000003989;	0.0599089	0.1705987	FALSE
TC07000235.hg.1	ENSG00000011426;	0.0599089	0.1705987	FALSE
TC02001628.hg.1	ENSG00000084674;	0.0599089	0.1705987	FALSE
TC01003638.hg.1	ENSG00000073756;	0.0599089	0.1705987	FALSE
TC04000313.hg.1	ENSG00000157404;	0.0599089	0.1705987	FALSE
TC07000137.hg.1	ENSG00000136244;	0.0599089	0.1705987	FALSE
TC06001027.hg.1	ENSG00000118503;	0.0599089	0.1705987	FALSE
TC03001071.hg.1	ENSG00000114315;	0.0599089	0.1705987	FALSE
TC10002924.hg.1	ENSG00000187134;	0.0599089	0.1705987	FALSE
TC10002924.hg.1	ENSG00000187134;	0.0599089	0.1705987	FALSE
TC17000220.hg.1	ENSG00000108448;	0.0599089	0.1705987	FALSE
TC01000685.hg.1	ENSG00000162409;	0.0599089	0.1705987	FALSE
TC06001027.hg.1	ENSG00000118503;	0.0599089	0.1705987	FALSE
TC03000214.hg.1	ENSG00000168036;	0.0599089	0.1705987	FALSE
TC10001711.hg.1;	ENSG00000066468;	0.0599089	0.1705987	FALSE
TC10001711.hg.1;	ENSG00000066468;	0.0599089	0.1705987	FALSE
TC03000214.hg.1	ENSG00000168036;	0.0599089	0.1705987	FALSE
TC02000620.hg.1	ENSG00000115594;	0.0599089	0.1705987	FALSE
TC07000768.hg.1	ENSG00000174697;	0.0599089	0.1705987	FALSE
TC02002624.hg.1	ENSG00000115415;	0.0599089	0.1705987	FALSE

TC14001188.hg.1;	ENSG00000126778;	0.0599089	0.1705987	FALSE
TC04002931.hg.1	ENSG00000134853;	0.0599089	0.1705987	FALSE
TC01003129.hg.1	ENSG00000143140;	0.0599089	0.1705987	FALSE
TC01003129.hg.1	ENSG00000143140;	0.0599089	0.1705987	FALSE
TC02001750.hg.1	ENSG00000138061;	0.0599089	0.1705987	FALSE
TC01003129.hg.1	ENSG00000143140;	0.0602535	0.1705987	FALSE
TC02002624.hg.1	ENSG00000115415;	0.0607532	0.171843	FALSE
TC03000214.hg.1	ENSG00000168036;	0.0607532	0.171843	FALSE
TC01000904.hg.1	ENSG00000162692;	0.0613889	0.1733608	FALSE
TC01000100.hg.1	ENSG00000049246;	0.061391	0.1733608	FALSE
TC03002146.hg.1	ENSG00000189058;	0.061534	0.1736499	FALSE
TC07001332.hg.1	ENSG00000164708;	0.0621875	0.1753209	FALSE
TC02002605.hg.1	ENSG00000064989;	0.0621875	0.1753209	FALSE
TC04000408.hg.1	ENSG00000169429;	0.0635759	0.1789993	FALSE
TC02000041.hg.1	ENSG00000115738;	0.0635759	0.1789993	FALSE
TC01001624.hg.1	ENSG00000116741;	0.0640945	0.1802815	FALSE
TC01000685.hg.1	ENSG00000162409;	0.0641715	0.1803795	FALSE
TC03000846.hg.1	ENSG00000196549;	0.064359	0.180788	FALSE
TC01002669.hg.1	ENSG00000157193;	0.0646983	0.1808462	FALSE
TC01003211.hg.1;	ENSG00000143387;	0.0646983	0.1808462	FALSE
TC01001188.hg.1;	ENSG00000143369;	0.0649125	0.1808462	FALSE
TC11000198.hg.1;	ENSG00000133816;	0.0649125	0.1808462	FALSE
TC01001090.hg.1	ENSG00000117289;	0.0649125	0.1808462	FALSE
TC04000729.hg.1	ENSG00000151617;	0.0649125	0.1808462	FALSE
TC02000996.hg.1	ENSG00000172292;	0.0649125	0.1808462	FALSE
TC07001897.hg.1	ENSG00000157680;	0.0649125	0.1808462	FALSE
TC01003638.hg.1	ENSG00000073756;	0.0649125	0.1808462	FALSE
TC01002166.hg.1	ENSG00000116285;	0.0649125	0.1808462	FALSE
TC01003129.hg.1	ENSG00000143140;	0.0649125	0.1808462	FALSE
TC04000313.hg.1	ENSG00000157404;	0.0649125	0.1808462	FALSE
TC06000595.hg.1	ENSG00000112655;	0.06495	0.1808462	FALSE
TC01000728.hg.1	ENSG00000162433;	0.06495	0.1808462	FALSE
TC01000904.hg.1	ENSG00000162692;	0.0658989	0.1833095	FALSE
TC01003129.hg.1	ENSG00000143140;	0.0662471	0.1841207	FALSE
TC01002847.hg.1	ENSG00000117228;	0.0662766	0.1841207	FALSE
TC01003638.hg.1	ENSG00000073756;	0.0669985	0.1859916	FALSE
TC02002714.hg.1	ENSG00000163251;	0.0670394	0.1859916	FALSE
TC02002714.hg.1	ENSG00000163251;	0.0670804	0.1859916	FALSE
TC01000685.hg.1	ENSG00000162409;	0.0672634	0.1863784	FALSE
TC04000313.hg.1	ENSG00000157404;	0.0673664	0.1864213	FALSE
TC02001100.hg.1;	ENSG00000138448;	0.0673664	0.1864213	FALSE
TC04002931.hg.1	ENSG00000134853;	0.0673664	0.1864213	FALSE
TC01000115.hg.1;	ENSG00000171621;	0.0674531	0.1864213	FALSE
TC01000904.hg.1	ENSG00000162692;	0.067831	0.1871661	FALSE
TC01000904.hg.1	ENSG00000162692;	0.0678319	0.1871661	FALSE
TC03000262.hg.1	ENSG00000160801;	0.0678319	0.1871661	FALSE
TC01003638.hg.1	ENSG00000073756;	0.0689193	0.1899829	FALSE
TC01002669.hg.1	ENSG00000157193;	0.06956	0.1916256	FALSE
TC01000904.hg.1	ENSG00000162692;	0.0712968	0.1936373	FALSE
TC01002578.hg.1	ENSG00000117394;	0.0712968	0.1936373	FALSE
TC11001589.hg.1;	ENSG00000175264;	0.0712968	0.1936373	FALSE
TC15000226.hg.1	ENSG00000166923;	0.0712968	0.1936373	FALSE
TC02001100.hg.1;	ENSG00000138448;	0.0712968	0.1936373	FALSE
TC03001071.hg.1	ENSG00000114315;	0.0712968	0.1936373	FALSE
TC14001188.hg.1;	ENSG00000126778;	0.0712968	0.1936373	FALSE
TC01002669.hg.1	ENSG00000157193;	0.0712968	0.1936373	FALSE
TC12000601.hg.1	ENSG00000127314;	0.0712968	0.1936373	FALSE
TC02002823.hg.1	ENSG00000135919;	0.0712968	0.1936373	FALSE

TC02002823.hg.1	ENSG00000135919;	0.0712968	0.1936373	FALSE
TC03002146.hg.1	ENSG00000189058;	0.0712968	0.1936373	FALSE
TC06000087.hg.1	ENSG00000078401;	0.0712968	0.1936373	FALSE
TC06000621.hg.1	ENSG00000124813;	0.0712968	0.1936373	FALSE
TC06000945.hg.1	ENSG00000152661;	0.0712968	0.1936373	FALSE
TC07000137.hg.1	ENSG00000136244;	0.0712968	0.1936373	FALSE
TC06000595.hg.1	ENSG00000112655;	0.0712968	0.1936373	FALSE
TC02000286.hg.1	ENSG00000171150;	0.0712968	0.1936373	FALSE
TC01000972.hg.1	ENSG00000064886;	0.0712968	0.1936373	FALSE
TC02000230.hg.1	ENSG00000150938;	0.0712968	0.1936373	FALSE
TC01003129.hg.1	ENSG00000143140;	0.0712968	0.1936373	FALSE
TC17001682.hg.1	ENSG00000108821;	0.0712968	0.1936373	FALSE
TC02000041.hg.1	ENSG00000115738;	0.0712968	0.1936373	FALSE
TC03000214.hg.1	ENSG00000168036;	0.0712968	0.1936373	FALSE
TC05001933.hg.1	ENSG00000113721;	0.0712968	0.1936373	FALSE
TC01003129.hg.1	ENSG00000143140;	0.0712968	0.1936373	FALSE
TC06000595.hg.1	ENSG00000112655;	0.0712968	0.1936373	FALSE
TC10001571.hg.1	ENSG00000120057;	0.0712968	0.1936373	FALSE
TC0X001186.hg.1	ENSG00000072133;	0.0712968	0.1936373	FALSE
TC01001473.hg.1	ENSG00000143153;	0.0716472	0.1936373	FALSE
TC01000685.hg.1	ENSG00000162409;	0.0719708	0.193989	FALSE
TC01000685.hg.1	ENSG00000162409;	0.0720085	0.193989	FALSE
TC01003638.hg.1	ENSG00000073756;	0.0720298	0.193989	FALSE
TC02002292.hg.1	ENSG00000136720;	0.0720298	0.193989	FALSE
TC05001614.hg.1	ENSG00000175426;	0.0720298	0.193989	FALSE
TC02001031.hg.1	ENSG00000152256;	0.0720298	0.193989	FALSE
TC07001883.hg.1	ENSG00000085662;	0.0720298	0.193989	FALSE
TC02000416.hg.1	ENSG00000116005;	0.0721398	0.193989	FALSE
TC03001071.hg.1	ENSG00000114315;	0.0722421	0.1940812	FALSE
TC01001090.hg.1	ENSG00000117289;	0.0722421	0.1940812	FALSE
TC04002931.hg.1	ENSG00000134853;	0.0727613	0.1950477	FALSE
TC03001575.hg.1	ENSG00000114480;	0.0727613	0.1950477	FALSE
TC02002823.hg.1	ENSG00000135919;	0.0727613	0.1950477	FALSE
TC03001071.hg.1	ENSG00000114315;	0.0727613	0.1950477	FALSE
TC01003638.hg.1	ENSG00000073756;	0.0727613	0.1950477	FALSE
TC01001805.hg.1	ENSG00000092969;	0.0761067	0.2036329	FALSE
TC03001575.hg.1	ENSG00000114480;	0.0768055	0.2053102	FALSE
TC03000631.hg.1	ENSG00000114023;	0.0768055	0.2053102	FALSE
TC01002578.hg.1	ENSG00000117394;	0.0772869	0.2062751	FALSE
TC03000013.hg.1	ENSG00000150995;	0.0772869	0.2062751	FALSE
TC04002931.hg.1	ENSG00000134853;	0.0772869	0.2062751	FALSE
TC02001031.hg.1	ENSG00000152256;	0.0774781	0.2064271	FALSE
TC01002669.hg.1	ENSG00000157193;	0.0775037	0.2064271	FALSE
TC01002669.hg.1	ENSG00000157193;	0.0775367	0.2064271	FALSE
TC04000729.hg.1	ENSG00000151617;	0.0783688	0.2083831	FALSE
TC06001027.hg.1	ENSG00000118503;	0.0783688	0.2083831	FALSE
TC06000087.hg.1	ENSG00000078401;	0.0783688	0.2083831	FALSE
TC03000214.hg.1	ENSG00000168036;	0.0794791	0.210681	FALSE
TC07000137.hg.1	ENSG00000136244;	0.0794791	0.210681	FALSE
TC01003497.hg.1	ENSG00000117479;	0.0794791	0.210681	FALSE
TC09001335.hg.1	ENSG00000127083;	0.0794791	0.210681	FALSE
TC02001628.hg.1	ENSG00000084674;	0.0794791	0.210681	FALSE
TC02001628.hg.1	ENSG00000084674;	0.0794791	0.210681	FALSE
TC02001628.hg.1	ENSG00000084674;	0.0794791	0.210681	FALSE
TC01002669.hg.1	ENSG00000157193;	0.0803784	0.2125385	FALSE
TC02001963.hg.1	ENSG00000163235;	0.0806954	0.2132452	FALSE
TC01000685.hg.1	ENSG00000162409;	0.0808688	0.2135714	FALSE
TC02001628.hg.1	ENSG00000084674;	0.0815207	0.2151604	FALSE

TC01002669.hg.1	ENSG00000157193;	0.0819034	0.2160374	FALSE
TC12000227.hg.1	ENSG00000172572;	0.0833149	0.2174338	FALSE
TC03000846.hg.1	ENSG00000196549;	0.0833149	0.2174338	FALSE
TC06000608.hg.1	ENSG00000112715;	0.0833149	0.2174338	FALSE
TC07001883.hg.1	ENSG00000085662;	0.0833149	0.2174338	FALSE
TC06001015.hg.1	ENSG00000171408;	0.0833149	0.2174338	FALSE
TC01002578.hg.1	ENSG00000117394;	0.0833149	0.2174338	FALSE
TC01002578.hg.1	ENSG00000117394;	0.0833149	0.2174338	FALSE
TC05000943.hg.1	ENSG00000156427;	0.0833149	0.2174338	FALSE
TC01001607.hg.1	ENSG00000116711;I	0.0833149	0.2174338	FALSE
TC06001027.hg.1	ENSG00000118503;	0.0833149	0.2174338	FALSE
TC06000087.hg.1	ENSG00000078401;	0.0833149	0.2174338	FALSE
TC14001443.hg.1	ENSG00000140092;	0.0833149	0.2174338	FALSE
TC05001933.hg.1	ENSG00000113721;	0.0833149	0.2174338	FALSE
TC07000137.hg.1	ENSG00000136244;	0.0833149	0.2174338	FALSE
TC04000313.hg.1	ENSG00000157404;	0.0833149	0.2174338	FALSE
TC02001100.hg.1;	ENSG00000138448;	0.0833149	0.2174338	FALSE
TC03000013.hg.1	ENSG00000150995;	0.0833149	0.2174338	FALSE
TC03001071.hg.1	ENSG00000114315;	0.0833149	0.2174338	FALSE
TC07000137.hg.1	ENSG00000136244;	0.0833149	0.2174338	FALSE
TC01001607.hg.1	ENSG00000116711;I	0.0833149	0.2174338	FALSE
TC02001100.hg.1;	ENSG00000138448;	0.0833149	0.2174338	FALSE
TC06000087.hg.1	ENSG00000078401;	0.0833149	0.2174338	FALSE
TC01003638.hg.1	ENSG00000073756;	0.0833149	0.2174338	FALSE
TC05001933.hg.1	ENSG00000113721;	0.0833149	0.2174338	FALSE
TC08000845.hg.1	ENSG00000187786;	0.0833149	0.2174338	FALSE
TC08000482.hg.1	ENSG00000121039;	0.0833149	0.2174338	FALSE
TC02000041.hg.1	ENSG00000115738;	0.0833149	0.2174338	FALSE
TC01002669.hg.1	ENSG00000157193;	0.0833149	0.2174338	FALSE
TC02000041.hg.1	ENSG00000115738;	0.0833149	0.2174338	FALSE
TC04000699.hg.1	ENSG00000109458;	0.0833149	0.2174338	FALSE
TC01002166.hg.1	ENSG00000116285;	0.0833149	0.2174338	FALSE
TC02002624.hg.1	ENSG00000115415;	0.0833149	0.2174338	FALSE
TC01003638.hg.1	ENSG00000073756;	0.0841851	0.2174338	FALSE
TC05000054.hg.1	ENSG00000078295;	0.0841851	0.2174338	FALSE
TC02000041.hg.1	ENSG00000115738;	0.0841851	0.2174338	FALSE
TC01001624.hg.1	ENSG00000116741;	0.0841851	0.2174338	FALSE
TC01000873.hg.1	ENSG00000143036;	0.0845295	0.2179749	FALSE
TC01002669.hg.1	ENSG00000157193;	0.0845728	0.2179749	FALSE
TC01000394.hg.1	ENSG00000159023;	0.0859337	0.2213491	FALSE
TC02001628.hg.1	ENSG00000084674;	0.0863863	0.2223812	FALSE
TC01003638.hg.1	ENSG00000073756;	0.0872476	0.223858	FALSE
TC01000728.hg.1	ENSG00000162433;	0.0872476	0.223858	FALSE
TC11000238.hg.1;	ENSG00000173432;	0.0872476	0.223858	FALSE
TC02001031.hg.1	ENSG00000152256;	0.0872476	0.223858	FALSE
TC03001071.hg.1	ENSG00000114315;	0.0872476	0.223858	FALSE
TC01000685.hg.1	ENSG00000162409;	0.0872476	0.223858	FALSE
TC06000945.hg.1	ENSG00000152661;	0.0872476	0.223858	FALSE
TC03000214.hg.1	ENSG00000168036;	0.0872476	0.223858	FALSE
TC04000432.hg.1	ENSG00000156234;	0.0872476	0.223858	FALSE
TC04001245.hg.1	ENSG00000145242;	0.0872476	0.223858	FALSE
TC01000115.hg.1;	ENSG00000171621;	0.0877371	0.2243744	FALSE
TC01001805.hg.1	ENSG00000092969;	0.0887969	0.2269489	FALSE
TC01000685.hg.1	ENSG00000162409;	0.0900537	0.2300238	FALSE
TC04000729.hg.1	ENSG00000151617;	0.0902062	0.2301386	FALSE
TC04000729.hg.1	ENSG00000151617;	0.0902062	0.2301386	FALSE
TC06000120.hg.1	ENSG00000137393;	0.0902062	0.2301386	FALSE
TC01000685.hg.1	ENSG00000162409;	0.0906208	0.2309211	FALSE

TC03000917.hg.1	ENSG00000136603;	0.0914005	0.2327004	FALSE
TC01000972.hg.1	ENSG00000064886;	0.0914005	0.2327004	FALSE
TC01000783.hg.1	ENSG00000117069;	0.0922479	0.2346485	FALSE
TC01001090.hg.1	ENSG00000117289;	0.0929	0.2360878	FALSE
TC01002669.hg.1	ENSG00000157193;	0.0929241	0.2360878	FALSE
TC03000214.hg.1	ENSG00000168036;	0.0931797	0.2365268	FALSE
TC02002624.hg.1	ENSG00000115415;	0.0931797	0.2365268	FALSE
TC03000214.hg.1	ENSG00000168036;	0.0933652	0.2367169	FALSE
TC03000846.hg.1	ENSG00000196549;	0.0933652	0.2367169	FALSE
TC01000904.hg.1	ENSG00000162692;	0.0943156	0.2389143	FALSE
TC01000783.hg.1	ENSG00000117069;	0.0949278	0.2399879	FALSE
TC03000917.hg.1	ENSG00000136603;	0.0953221	0.2399879	FALSE
TC05000165.hg.1	ENSG00000079215;	0.0953221	0.2399879	FALSE
TC01000728.hg.1	ENSG00000162433;	0.0953221	0.2399879	FALSE
TC02000041.hg.1	ENSG00000115738;	0.0953221	0.2399879	FALSE
TC04002931.hg.1	ENSG00000134853;	0.0953221	0.2399879	FALSE
TC05000165.hg.1	ENSG00000079215;	0.0953221	0.2399879	FALSE
TC02000762.hg.1	ENSG00000163083;	0.0953221	0.2399879	FALSE
TC03000214.hg.1	ENSG00000168036;	0.0953221	0.2399879	FALSE
TC01001736.hg.1	ENSG00000196352;	0.0953308	0.2399879	FALSE
TC01000685.hg.1	ENSG00000162409;	0.0953308	0.2399879	FALSE
TC02002049.hg.1	ENSG00000115525;	0.0958932	0.2399879	FALSE
TC01000734.hg.1	ENSG00000118473;	0.0958932	0.2399879	FALSE
TC07000137.hg.1	ENSG00000136244;	0.0958932	0.2399879	FALSE
TC01001805.hg.1	ENSG00000092969;	0.0958932	0.2399879	FALSE
TC01001805.hg.1	ENSG00000092969;	0.0958932	0.2399879	FALSE
TC03001466.hg.1	ENSG00000163931;	0.0958932	0.2399879	FALSE
TC02001628.hg.1	ENSG00000084674;	0.0958932	0.2399879	FALSE
TC14000067.hg.1	ENSG00000181784;	0.0958932	0.2399879	FALSE
TC04000503.hg.1	ENSG00000138696;	0.0958932	0.2399879	FALSE
TC01001607.hg.1	ENSG00000116711;I	0.0958932	0.2399879	FALSE
TC01001607.hg.1	ENSG00000116711;I	0.0958932	0.2399879	FALSE
TC01001805.hg.1	ENSG00000092969;	0.0958932	0.2399879	FALSE
TC02001206.hg.1	ENSG00000118257;	0.0958932	0.2399879	FALSE
TC06000595.hg.1	ENSG00000112655;	0.0958932	0.2399879	FALSE
TC10001711.hg.1;	ENSG00000066468;	0.0958932	0.2399879	FALSE
TC01001805.hg.1	ENSG00000092969;	0.0958932	0.2399879	FALSE
TC01003129.hg.1	ENSG00000143140;	0.096394	0.2399879	FALSE
TC02002605.hg.1	ENSG00000064989;	0.0964274	0.2399879	FALSE
TC05001614.hg.1	ENSG00000175426;	0.0964274	0.2399879	FALSE
TC02000041.hg.1	ENSG00000115738;	0.0964274	0.2399879	FALSE
TC01003638.hg.1	ENSG00000073756;	0.0965614	0.2399879	FALSE
TC01001624.hg.1	ENSG00000116741;	0.0965614	0.2399879	FALSE
TC03001071.hg.1	ENSG00000114315;	0.097677	0.2424792	FALSE
TC03000214.hg.1	ENSG00000168036;	0.097677	0.2424792	FALSE
TC01000904.hg.1	ENSG00000162692;	0.0980453	0.2431819	FALSE
TC01000685.hg.1	ENSG00000162409;	0.1000378	0.2479801	FALSE
TC01000685.hg.1	ENSG00000162409;	0.1002618	0.2483915	FALSE
TC01000873.hg.1	ENSG00000143036;	0.1006837	0.2492206	FALSE
TC01000873.hg.1	ENSG00000143036;	0.1006837	0.2492206	FALSE
TC08000845.hg.1	ENSG00000187786;	0.1018667	0.2518576	FALSE
TC04002931.hg.1	ENSG00000134853;	0.1018667	0.2518576	FALSE
TC01000100.hg.1	ENSG00000049246;	0.1020951	0.2522039	FALSE
TC01003638.hg.1	ENSG00000073756;	0.1022877	0.2525338	FALSE
TC01001907.hg.1	ENSG00000143641;	0.1028441	0.2535419	FALSE
TC05001110.hg.1;	ENSG00000153395;	0.1028441	0.2535419	FALSE
TC02002624.hg.1	ENSG00000115415;	0.1028441	0.2535419	FALSE
TC02000041.hg.1	ENSG00000115738;	0.1028441	0.2535419	FALSE

TC02000762.hg.1	ENSG00000163083;	0.1036887	0.2545247	FALSE
TC01002706.hg.1	ENSG00000184292;	0.1036887	0.2545247	FALSE
TC01001473.hg.1	ENSG00000143153;	0.1036887	0.2545247	FALSE
TC04000503.hg.1	ENSG00000138696;	0.1036887	0.2545247	FALSE
TC07001562.hg.1	ENSG00000019991;	0.1036887	0.2545247	FALSE
TC02002714.hg.1	ENSG00000163251;	0.1036887	0.2545247	FALSE
TC02000082.hg.1	ENSG00000071575;	0.1036887	0.2545247	FALSE
TC03000214.hg.1	ENSG00000168036;	0.1036887	0.2545247	FALSE
TC02002808.hg.1	ENSG00000116106;	0.1036887	0.2545247	FALSE
TC02002624.hg.1	ENSG00000115415;	0.1036887	0.2545247	FALSE
TC02001437.hg.1	ENSG00000144476;	0.1036887	0.2545247	FALSE
TC01000904.hg.1	ENSG00000162692;	0.1044181	0.2554364	FALSE
TC01000100.hg.1	ENSG00000049246;	0.104528	0.2555593	FALSE
TC01001607.hg.1	ENSG00000116711;|	0.1057285	0.2583468	FALSE
TC01000904.hg.1	ENSG00000162692;	0.106341	0.2596952	FALSE
TC04000408.hg.1	ENSG00000169429;	0.1070104	0.261181	FALSE
TC01000873.hg.1	ENSG00000143036;	0.1072746	0.2612542	FALSE
TC01001473.hg.1	ENSG00000143153;	0.1073142	0.2612542	FALSE
TC04002931.hg.1	ENSG00000134853;	0.1073142	0.2612542	FALSE
TC01000685.hg.1	ENSG00000162409;	0.1077213	0.2612542	FALSE
TC02000762.hg.1	ENSG00000163083;	0.1077542	0.2612542	FALSE
TC02002624.hg.1	ENSG00000115415;	0.1077542	0.2612542	FALSE
TC01001805.hg.1	ENSG00000092969;	0.1077891	0.2612542	FALSE
TC01000904.hg.1	ENSG00000162692;	0.1086661	0.2612542	FALSE
TC03000214.hg.1	ENSG00000168036;	0.1086661	0.2612542	FALSE
TC03000846.hg.1	ENSG00000196549;	0.1089665	0.2612542	FALSE
TC03001255.hg.1	ENSG00000033867;	0.1089665	0.2612542	FALSE
TC03001575.hg.1	ENSG00000114480;	0.1089665	0.2612542	FALSE
TC03001466.hg.1	ENSG00000163931;	0.1089665	0.2612542	FALSE
TC01003497.hg.1	ENSG00000117479;	0.1089665	0.2612542	FALSE
TC03001575.hg.1	ENSG00000114480;	0.1089665	0.2612542	FALSE
TC06000087.hg.1	ENSG00000078401;	0.1089665	0.2612542	FALSE
TC14000951.hg.1	ENSG00000092068;	0.1089665	0.2612542	FALSE
TC07000137.hg.1	ENSG00000136244;	0.1089665	0.2612542	FALSE
TC02001100.hg.1;	ENSG00000138448;	0.1089665	0.2612542	FALSE
TC07000137.hg.1	ENSG00000136244;	0.1089665	0.2612542	FALSE
TC06000087.hg.1	ENSG00000078401;	0.1089665	0.2612542	FALSE
TC02001100.hg.1;	ENSG00000138448;	0.1089665	0.2612542	FALSE
TC01002932.hg.1	ENSG00000060718;	0.1089665	0.2612542	FALSE
TC06000087.hg.1	ENSG00000078401;	0.1089665	0.2612542	FALSE
TC02001100.hg.1;	ENSG00000138448;	0.1089665	0.2612542	FALSE
TC02001100.hg.1;	ENSG00000138448;	0.1089665	0.2612542	FALSE
TC02002480.hg.1	ENSG00000078098;	0.1089665	0.2612542	FALSE
TC02001100.hg.1;	ENSG00000138448;	0.1089665	0.2612542	FALSE
TC02001100.hg.1;	ENSG00000138448;	0.1089665	0.2612542	FALSE
TC01001805.hg.1	ENSG00000092969;	0.1089665	0.2612542	FALSE
TC03000846.hg.1	ENSG00000196549;	0.1089665	0.2612542	FALSE
TC02002823.hg.1	ENSG00000135919;	0.1089665	0.2612542	FALSE
TC12001890.hg.1	ENSG00000017427;	0.1089665	0.2612542	FALSE
TC07000137.hg.1	ENSG00000136244;	0.1089665	0.2612542	FALSE
TC01001607.hg.1	ENSG00000116711;|	0.1089665	0.2612542	FALSE
TC01001607.hg.1	ENSG00000116711;|	0.1089665	0.2612542	FALSE
TC06001027.hg.1	ENSG00000118503;	0.1089665	0.2612542	FALSE
TC01001805.hg.1	ENSG00000092969;	0.1094509	0.2612542	FALSE
TC06000087.hg.1	ENSG00000078401;	0.1094509	0.2612542	FALSE
TC03001255.hg.1	ENSG00000033867;	0.1094509	0.2612542	FALSE
TC04000729.hg.1	ENSG00000151617;	0.1094509	0.2612542	FALSE
TC01002932.hg.1	ENSG00000060718;	0.1122462	0.2673143	FALSE

TC02000891.hg.1	ENSG00000150540;	0.1123332	0.2673143	FALSE
TC04000253.hg.1	ENSG00000154277;	0.1123332	0.2673143	FALSE
TC12001890.hg.1	ENSG00000017427;	0.1123332	0.2673143	FALSE
TC01002669.hg.1	ENSG00000157193;	0.1123332	0.2673143	FALSE
TC04000503.hg.1	ENSG00000138696;	0.1123332	0.2673143	FALSE
TC02002605.hg.1	ENSG00000064989;	0.1129013	0.2681445	FALSE
TC01000394.hg.1	ENSG00000159023;	0.1129013	0.2681445	FALSE
TC01000783.hg.1	ENSG00000117069;	0.1162426	0.2758506	FALSE
TC01002954.hg.1	ENSG00000134243;	0.1167865	0.2769877	FALSE
TC01002578.hg.1	ENSG00000117394;	0.1186253	0.2810375	FALSE
TC03002146.hg.1	ENSG00000189058;	0.1186253	0.2810375	FALSE
TC08000845.hg.1	ENSG00000187786;	0.1186253	0.2810375	FALSE
TC01003638.hg.1	ENSG00000073756;	0.1187908	0.2811184	FALSE
TC01001624.hg.1	ENSG00000116741;	0.120739	0.2855711	FALSE
TC10000811.hg.1;	ENSG00000150594;	0.1212413	0.2862846	FALSE
TC03001575.hg.1	ENSG00000114480;	0.1212413	0.2862846	FALSE
TC09001335.hg.1	ENSG00000127083;	0.1212413	0.2862846	FALSE
TC07000220.hg.1	ENSG00000164619;	0.1212413	0.2862846	FALSE
TC02000082.hg.1	ENSG00000071575;	0.1212413	0.2862846	FALSE
TC01002669.hg.1	ENSG00000157193;	0.1218367	0.2867433	FALSE
TC01000685.hg.1	ENSG00000162409;	0.1219517	0.2867433	FALSE
TC01000904.hg.1	ENSG00000162692;	0.1219517	0.2867433	FALSE
TC02002624.hg.1	ENSG00000115415;	0.1219517	0.2867433	FALSE
TC07001559.hg.1	ENSG00000075223;	0.1224738	0.2867433	FALSE
TC09000358.hg.1	ENSG00000135069;	0.1224738	0.2867433	FALSE
TC01001805.hg.1	ENSG00000092969;	0.1224738	0.2867433	FALSE
TC03000214.hg.1	ENSG00000168036;	0.1224738	0.2867433	FALSE
TC01003638.hg.1	ENSG00000073756;	0.1224738	0.2867433	FALSE
TC04000313.hg.1	ENSG00000157404;	0.1224738	0.2867433	FALSE
TC17001462.hg.1	ENSG00000131747;	0.1224738	0.2867433	FALSE
TC03000214.hg.1	ENSG00000168036;	0.1224738	0.2867433	FALSE
TC02001963.hg.1	ENSG00000163235;	0.1224738	0.2867433	FALSE
TC07000137.hg.1	ENSG00000136244;	0.1224738	0.2867433	FALSE
TC03001527.hg.1	ENSG00000163638;	0.1224738	0.2867433	FALSE
TC01002669.hg.1	ENSG00000157193;	0.1224738	0.2867433	FALSE
TC01002166.hg.1	ENSG00000116285;	0.1224738	0.2867433	FALSE
TC03000214.hg.1	ENSG00000168036;	0.1224738	0.2867433	FALSE
TC03000013.hg.1	ENSG00000150995;	0.1224738	0.2867433	FALSE
TC10000406.hg.1	ENSG00000138336;	0.1224738	0.2867433	FALSE
TC01003129.hg.1	ENSG00000143140;	0.1224738	0.2867433	FALSE
TC07001618.hg.1	ENSG00000004799;	0.1224738	0.2867433	FALSE
TC01001188.hg.1;	ENSG00000143369;	0.1232134	0.2869059	FALSE
TC01003638.hg.1	ENSG00000073756;	0.1232134	0.2869059	FALSE
TC02001031.hg.1	ENSG00000152256;	0.1244833	0.2896267	FALSE
TC01003638.hg.1	ENSG00000073756;	0.1265574	0.2942924	FALSE
TC01000685.hg.1	ENSG00000162409;	0.1269929	0.2951447	FALSE
TC03000577.hg.1	ENSG00000144857;	0.1303574	0.3013501	FALSE
TC01002669.hg.1	ENSG00000157193;	0.1303574	0.3013501	FALSE
TC01002669.hg.1	ENSG00000157193;	0.1303574	0.3013501	FALSE
TC04000729.hg.1	ENSG00000151617;	0.1303574	0.3013501	FALSE
TC03001575.hg.1	ENSG00000114480;	0.1303984	0.3013501	FALSE
TC03001575.hg.1	ENSG00000114480;	0.1303984	0.3013501	FALSE
TC01000685.hg.1	ENSG00000162409;	0.1303984	0.3013501	FALSE
TC04002931.hg.1	ENSG00000134853;	0.1303984	0.3013501	FALSE
TC01000685.hg.1	ENSG00000162409;	0.1303984	0.3013501	FALSE
TC06000779.hg.1	ENSG00000135318;	0.1303984	0.3013501	FALSE
TC04000408.hg.1	ENSG00000169429;	0.1304724	0.3013501	FALSE
TC02000041.hg.1	ENSG00000115738;	0.1304724	0.3013501	FALSE

TC01001473.hg.1	ENSG00000143153;	0.1313122	0.3030446	FALSE
TC04000313.hg.1	ENSG00000157404;	0.1322532	0.3049695	FALSE
TC01003778.hg.1	ENSG00000076356;	0.1322532	0.3049695	FALSE
TC01000873.hg.1	ENSG00000143036;	0.133038	0.3063719	FALSE
TC01000904.hg.1	ENSG00000162692;	0.1330403	0.3063719	FALSE
TC01000685.hg.1	ENSG00000162409;	0.1341074	0.3086634	FALSE
TC0X000943.hg.1	ENSG00000198947;	0.1363581	0.3113626	FALSE
TC07000525.hg.1	ENSG00000008277;	0.1363581	0.3113626	FALSE
TC07000525.hg.1	ENSG00000008277;	0.1363581	0.3113626	FALSE
TC01001805.hg.1	ENSG00000092969;	0.1363581	0.3113626	FALSE
TC04000432.hg.1	ENSG00000156234;	0.1363581	0.3113626	FALSE
TC10001663.hg.1	ENSG00000119927;	0.1363581	0.3113626	FALSE
TC02000286.hg.1	ENSG00000171150;	0.1363581	0.3113626	FALSE
TC03000214.hg.1	ENSG00000168036;	0.1363581	0.3113626	FALSE
TC06001027.hg.1	ENSG00000118503;	0.1363581	0.3113626	FALSE
TC05001590.hg.1	ENSG00000113369;	0.1363581	0.3113626	FALSE
TC01000904.hg.1	ENSG00000162692;	0.1363581	0.3113626	FALSE
TC15000226.hg.1	ENSG00000166923;	0.1363581	0.3113626	FALSE
TC01001805.hg.1	ENSG00000092969;	0.1363581	0.3113626	FALSE
TC01003778.hg.1	ENSG00000076356;	0.1363581	0.3113626	FALSE
TC04000408.hg.1	ENSG00000169429;	0.1363581	0.3113626	FALSE
TC02001963.hg.1	ENSG00000163235;	0.1363581	0.3113626	FALSE
TC04000173.hg.1	ENSG00000109610;	0.1365894	0.3113626	FALSE
TC04000408.hg.1	ENSG00000169429;	0.1365894	0.3113626	FALSE
TC01001090.hg.1	ENSG00000117289;	0.1365894	0.3113626	FALSE
TC01002284.hg.1	ENSG00000117122;	0.137306	0.3126632	FALSE
TC02001628.hg.1	ENSG00000084674;	0.1376686	0.3129895	FALSE
TC03002106.hg.1	ENSG00000090530;	0.1376686	0.3129895	FALSE
TC02001628.hg.1	ENSG00000084674;	0.1376686	0.3129895	FALSE
TC01006336.hg.1	ENSG00000162706;	0.1376686	0.3129895	FALSE
TC02000466.hg.1	ENSG00000159399;	0.1376686	0.3129895	FALSE
TC03001575.hg.1	ENSG00000114480;	0.1397897	0.3170541	FALSE
TC03001575.hg.1	ENSG00000114480;	0.1397897	0.3170541	FALSE
TC04000432.hg.1	ENSG00000156234;	0.1397897	0.3170541	FALSE
TC03000917.hg.1	ENSG00000136603;	0.1397897	0.3170541	FALSE
TC01001907.hg.1	ENSG00000143641;	0.1400262	0.3171705	FALSE
TC04000408.hg.1	ENSG00000169429;	0.1408899	0.318621	FALSE
TC02002624.hg.1	ENSG00000115415;	0.1408899	0.318621	FALSE
TC01000904.hg.1	ENSG00000162692;	0.1408899	0.318621	FALSE
TC02000041.hg.1	ENSG00000115738;	0.1408899	0.318621	FALSE
TC03000214.hg.1	ENSG00000168036;	0.1408899	0.318621	FALSE
TC01000685.hg.1	ENSG00000162409;	0.1418698	0.3203294	FALSE
TC01000904.hg.1	ENSG00000162692;	0.1421771	0.320854	FALSE
TC01003791.hg.1	ENSG00000143473;	0.1424096	0.3211248	FALSE
TC01003791.hg.1	ENSG00000143473;	0.1424096	0.3211248	FALSE
TC04000469.hg.1	ENSG00000163629;	0.1430784	0.3222934	FALSE
TC04000469.hg.1	ENSG00000163629;	0.1430784	0.3222934	FALSE
TC01003638.hg.1	ENSG00000073756;	0.143895	0.3238771	FALSE
TC01000115.hg.1;	ENSG00000171621;	0.1448698	0.3258999	FALSE
TC02000996.hg.1	ENSG00000172292;	0.1451406	0.3259953	FALSE
TC04001509.hg.1	ENSG00000173376;	0.1451406	0.3259953	FALSE
TC01000783.hg.1	ENSG00000117069;	0.1451406	0.3259953	FALSE
TC03001575.hg.1	ENSG00000114480;	0.1451406	0.3259953	FALSE
TC02000041.hg.1	ENSG00000115738;	0.1451406	0.3259953	FALSE
TC04001084.hg.1	ENSG00000109819;	0.1486392	0.3330774	FALSE
TC06000087.hg.1	ENSG00000078401;	0.1491935	0.3330774	FALSE
TC02000082.hg.1	ENSG00000071575;	0.1491935	0.3330774	FALSE
TC01001473.hg.1	ENSG00000143153;	0.1492091	0.3330774	FALSE

TC06000087.hg.1	ENSG00000078401;	0.1494005	0.3330774	FALSE
TC07000137.hg.1	ENSG00000136244;	0.1494005	0.3330774	FALSE
TC06004141.hg.1	ENSG00000112096;	0.1494005	0.3330774	FALSE
TC05003439.hg.1	ENSG00000213949;	0.1494005	0.3330774	FALSE
TC04000729.hg.1	ENSG00000151617;	0.1494005	0.3330774	FALSE
TC04000729.hg.1	ENSG00000151617;	0.1494005	0.3330774	FALSE
TC02000416.hg.1	ENSG00000116005;	0.1494005	0.3330774	FALSE
TC02001628.hg.1	ENSG00000084674;	0.1494005	0.3330774	FALSE
TC01000100.hg.1	ENSG00000049246;	0.149715	0.3330774	FALSE
TC02002624.hg.1	ENSG00000115415;	0.1498143	0.3330774	FALSE
TC03000214.hg.1	ENSG00000168036;	0.1505667	0.3330774	FALSE
TC02001139.hg.1;	ENSG00000196950;	0.1505667	0.3330774	FALSE
TC02000891.hg.1	ENSG00000150540;	0.1505667	0.3330774	FALSE
TC01003718.hg.1	ENSG00000133048;	0.1505667	0.3330774	FALSE
TC01002706.hg.1	ENSG00000184292;	0.1505667	0.3330774	FALSE
TC01002669.hg.1	ENSG00000157193;	0.1505667	0.3330774	FALSE
TC07001630.hg.1	ENSG00000070669;	0.1505667	0.3330774	FALSE
TC09000347.hg.1	ENSG00000099139;	0.1505667	0.3330774	FALSE
TC04000313.hg.1	ENSG00000157404;	0.1505667	0.3330774	FALSE
TC07000137.hg.1	ENSG00000136244;	0.1505667	0.3330774	FALSE
TC07000328.hg.1	ENSG00000146648;	0.1505667	0.3330774	FALSE
TC03001071.hg.1	ENSG00000114315;	0.1505667	0.3330774	FALSE
TC04000173.hg.1	ENSG00000109610;	0.1505667	0.3330774	FALSE
TC01003129.hg.1	ENSG00000143140;	0.1505667	0.3330774	FALSE
TC04000432.hg.1	ENSG00000156234;	0.1505667	0.3330774	FALSE
TC02001963.hg.1	ENSG00000163235;	0.1505667	0.3330774	FALSE
TC02002605.hg.1	ENSG00000064989;	0.1508613	0.3330774	FALSE
TC03001362.hg.1	ENSG00000160808;	0.1527671	0.3371114	FALSE
TC01000115.hg.1;	ENSG00000171621;	0.1537829	0.339178	FALSE
TC01000685.hg.1	ENSG00000162409;	0.1540255	0.3395383	FALSE
TC02000041.hg.1	ENSG00000115738;	0.1559676	0.3434657	FALSE
TC02002605.hg.1	ENSG00000064989;	0.1559676	0.3434657	FALSE
TC02002605.hg.1	ENSG00000064989;	0.1559676	0.3434657	FALSE
TC01000783.hg.1	ENSG00000117069;	0.1572475	0.3459282	FALSE
TC01001607.hg.1	ENSG00000116711;I	0.159216	0.3498993	FALSE
TC01001607.hg.1	ENSG00000116711;I	0.159216	0.3498993	FALSE
TC02001628.hg.1	ENSG00000084674;	0.159216	0.3498993	FALSE
TC02000416.hg.1	ENSG00000116005;	0.1600465	0.3512735	FALSE
TC01002166.hg.1	ENSG00000116285;	0.1600465	0.3512735	FALSE
TC01002669.hg.1	ENSG00000157193;	0.1603756	0.3517254	FALSE
TC04000253.hg.1	ENSG00000154277;	0.1605415	0.3518188	FALSE
TC07001883.hg.1	ENSG00000085662;	0.1605415	0.3518188	FALSE
TC01002932.hg.1	ENSG00000060718;	0.1611524	0.3528867	FALSE
TC03000214.hg.1	ENSG00000168036;	0.1650503	0.3583992	FALSE
TC03002146.hg.1	ENSG00000189058;	0.1650503	0.3583992	FALSE
TC01003129.hg.1	ENSG00000143140;	0.1650503	0.3583992	FALSE
TC01003638.hg.1	ENSG00000073756;	0.1650503	0.3583992	FALSE
TC07000525.hg.1	ENSG00000008277;	0.1650503	0.3583992	FALSE
TC03000214.hg.1	ENSG00000168036;	0.1650503	0.3583992	FALSE
TC10000811.hg.1;	ENSG00000150594;	0.1650503	0.3583992	FALSE
TC01001805.hg.1	ENSG00000092969;	0.1650503	0.3583992	FALSE
TC06000595.hg.1	ENSG00000112655;	0.1650503	0.3583992	FALSE
TC10000406.hg.1	ENSG00000138336;	0.1650503	0.3583992	FALSE
TC04001169.hg.1;	ENSG00000145244;	0.1650503	0.3583992	FALSE
TC01001607.hg.1	ENSG00000116711;I	0.1650503	0.3583992	FALSE
TC01001805.hg.1	ENSG00000092969;	0.1650503	0.3583992	FALSE
TC04000253.hg.1	ENSG00000154277;	0.1650503	0.3583992	FALSE
TC05001933.hg.1	ENSG00000113721;	0.1650503	0.3583992	FALSE

TC17000220.hg.1	ENSG00000108448;	0.1650503	0.3583992	FALSE
TC01001805.hg.1	ENSG00000092969;	0.1650503	0.3583992	FALSE
TC07001559.hg.1	ENSG00000075223;	0.1650503	0.3583992	FALSE
TC06000595.hg.1	ENSG00000112655;	0.1650503	0.3583992	FALSE
TC04000408.hg.1	ENSG00000169429;	0.1650503	0.3583992	FALSE
TC01003129.hg.1	ENSG00000143140;	0.1650503	0.3583992	FALSE
TC14001188.hg.1;	ENSG00000126778;	0.1650503	0.3583992	FALSE
TC03000214.hg.1	ENSG00000168036;	0.1650503	0.3583992	FALSE
TC01003718.hg.1	ENSG00000133048;	0.1650503	0.3583992	FALSE
TC04000469.hg.1	ENSG00000163629;	0.1658883	0.3583992	FALSE
TC04000469.hg.1	ENSG00000163629;	0.1658883	0.3583992	FALSE
TC04000469.hg.1	ENSG00000163629;	0.1658883	0.3583992	FALSE
TC02000891.hg.1	ENSG00000150540;	0.1658883	0.3583992	FALSE
TC02002624.hg.1	ENSG00000115415;	0.166414	0.3589914	FALSE
TC01003778.hg.1	ENSG00000076356;	0.166414	0.3589914	FALSE
TC01002578.hg.1	ENSG00000117394;	0.168457	0.3631241	FALSE
TC01000685.hg.1	ENSG00000162409;	0.1692217	0.3641308	FALSE
TC04000729.hg.1	ENSG00000151617;	0.1692217	0.3641308	FALSE
TC03000986.hg.1	ENSG00000163872;	0.1692217	0.3641308	FALSE
TC01002932.hg.1	ENSG00000060718;	0.1692217	0.3641308	FALSE
TC01002706.hg.1	ENSG00000184292;	0.1692217	0.3641308	FALSE
TC04000729.hg.1	ENSG00000151617;	0.1692217	0.3641308	FALSE
TC01003697.hg.1	ENSG00000163431;	0.1718617	0.3690694	FALSE
TC01002706.hg.1	ENSG00000184292;	0.1718617	0.3690694	FALSE
TC01000972.hg.1	ENSG00000064886;	0.1726373	0.3704563	FALSE
TC01003638.hg.1	ENSG00000073756;	0.1749577	0.3706089	FALSE
TC01002706.hg.1	ENSG00000184292;	0.1764434	0.3706089	FALSE
TC01003718.hg.1	ENSG00000133048;	0.176507	0.3706089	FALSE
TC03002146.hg.1	ENSG00000189058;	0.176507	0.3706089	FALSE
TC01001624.hg.1	ENSG00000116741;	0.176507	0.3706089	FALSE
TC01000904.hg.1	ENSG00000162692;	0.1765376	0.3706089	FALSE
TC02001031.hg.1	ENSG00000152256;	0.1767644	0.3706089	FALSE
TC03000248.hg.1	ENSG00000144791;	0.1778365	0.3706089	FALSE
TC02002605.hg.1	ENSG00000064989;	0.1778365	0.3706089	FALSE
TC09000347.hg.1	ENSG00000099139;	0.179403	0.3706089	FALSE
TC06001027.hg.1	ENSG00000118503;	0.179403	0.3706089	FALSE
TC02001031.hg.1	ENSG00000152256;	0.179403	0.3706089	FALSE
TC06000087.hg.1	ENSG00000078401;	0.179403	0.3706089	FALSE
TC01003638.hg.1	ENSG00000073756;	0.179403	0.3706089	FALSE
TC04001084.hg.1	ENSG00000109819;	0.1797629	0.3706089	FALSE
TC02001031.hg.1	ENSG00000152256;	0.1797629	0.3706089	FALSE
TC01003605.hg.1	ENSG00000135821;	0.1797629	0.3706089	FALSE
TC01003605.hg.1	ENSG00000135821;	0.1797629	0.3706089	FALSE
TC05001519.hg.1	ENSG00000113273;	0.1797629	0.3706089	FALSE
TC01003638.hg.1	ENSG00000073756;	0.1797629	0.3706089	FALSE
TC01002578.hg.1	ENSG00000117394;	0.1797629	0.3706089	FALSE
TC10002924.hg.1	ENSG00000187134;	0.1797629	0.3706089	FALSE
TC13001719.hg.1	ENSG00000150907;	0.1797629	0.3706089	FALSE
TC02001031.hg.1	ENSG00000152256;	0.1797629	0.3706089	FALSE
TC07001552.hg.1	ENSG00000187391;	0.1797629	0.3706089	FALSE
TC06000608.hg.1	ENSG00000112715;	0.1797629	0.3706089	FALSE
TC01003638.hg.1	ENSG00000073756;	0.1797629	0.3706089	FALSE
TC08000127.hg.1	ENSG00000003989;	0.1797629	0.3706089	FALSE
TC04001084.hg.1	ENSG00000109819;	0.1797629	0.3706089	FALSE
TC01002578.hg.1	ENSG00000117394;	0.1797629	0.3706089	FALSE
TC06004141.hg.1	ENSG00000112096;	0.1797629	0.3706089	FALSE
TC07001552.hg.1	ENSG00000187391;	0.1797629	0.3706089	FALSE
TC10001711.hg.1;	ENSG00000066468;	0.1797629	0.3706089	FALSE

TC03000248.hg.1	ENSG00000144791;	0.1797629	0.3706089	FALSE
TC13001719.hg.1	ENSG00000150907;	0.1797629	0.3706089	FALSE
TC01002578.hg.1	ENSG00000117394;	0.1797629	0.3706089	FALSE
TC02000286.hg.1	ENSG00000171150;	0.1797629	0.3706089	FALSE
TC01000873.hg.1	ENSG00000143036;	0.1811029	0.3706089	FALSE
TC01002669.hg.1	ENSG00000157193;	0.1826968	0.3706089	FALSE
TC01002706.hg.1	ENSG00000184292;	0.1839378	0.3706089	FALSE
TC01003638.hg.1	ENSG00000073756;	0.1839378	0.3706089	FALSE
TC02000762.hg.1	ENSG00000163083;	0.1846847	0.3706089	FALSE
TC01000904.hg.1	ENSG00000162692;	0.1847822	0.3706089	FALSE
TC04000503.hg.1	ENSG00000138696	0.1875799	0.3706089	FALSE
TC12001587.hg.1	ENSG00000123342	0.1875799	0.3706089	FALSE
TC02000432.hg.1	ENSG00000135636	0.1875799	0.3706089	FALSE
TC05000159.hg.1	ENSG00000168685	0.1875799	0.3706089	FALSE
TC04000432.hg.1	ENSG00000156234	0.1875799	0.3706089	FALSE
TC06004141.hg.1	ENSG00000112096	0.1875799	0.3706089	FALSE
TC11002366.hg.1	ENSG00000076706	0.1875799	0.3706089	FALSE
TC06000087.hg.1	ENSG00000078401	0.1875799	0.3706089	FALSE
TC01003129.hg.1	ENSG00000143140	0.1875799	0.3706089	FALSE
TC10000350.hg.1	ENSG00000107984	0.1875799	0.3706089	FALSE
TC10000350.hg.1	ENSG00000107984	0.1875799	0.3706089	FALSE
TC19001293.hg.1	ENSG00000105664	0.1875799	0.3706089	FALSE
TC10000053.hg.1	ENSG00000170525	0.1875799	0.3706089	FALSE
TC14000819.hg.1	ENSG00000185100	0.1875799	0.3706089	FALSE
TC12002066.hg.1	ENSG00000158104	0.1875799	0.3706089	FALSE
TC09000560.hg.1	ENSG00000148154	0.1875799	0.3706089	FALSE
TC22000259.hg.1	ENSG00000100292	0.1875799	0.3706089	FALSE
TC12002122.hg.1	ENSG00000139370	0.1875799	0.3706089	FALSE
TC07000137.hg.1	ENSG00000136244	0.1875799	0.3706089	FALSE
TC10000565.hg.1	ENSG00000108175	0.1875799	0.3706089	FALSE
TC03001071.hg.1	ENSG00000114315	0.1875799	0.3706089	FALSE
TC14000353.hg.1	ENSG00000165617	0.1875799	0.3706089	FALSE
TC20000368.hg.1	ENSG00000064655	0.1875799	0.3706089	FALSE
TC02002606.hg.1	ENSG00000003436	0.1875799	0.3706089	FALSE
TC01002195.hg.1	ENSG00000116649	0.1875799	0.3706089	FALSE
TC03001466.hg.1	ENSG00000163931	0.1875799	0.3706089	FALSE
TC06000779.hg.1	ENSG00000135318	0.1875799	0.3706089	FALSE
TC01000785.hg.1	ENSG00000154027	0.1875799	0.3706089	FALSE
TC05000165.hg.1	ENSG00000079215	0.1875799	0.3706089	FALSE
TC02002605.hg.1	ENSG00000064989	0.1875799	0.3706089	FALSE
TC03001071.hg.1	ENSG00000114315	0.1875799	0.3706089	FALSE
TC02002823.hg.1	ENSG00000135919	0.1875799	0.3706089	FALSE
TC03001255.hg.1	ENSG00000033867	0.1875799	0.3706089	FALSE
TC05000840.hg.1	ENSG00000123643	0.1875799	0.3706089	FALSE
TC09000535.hg.1	ENSG00000070214	0.1875799	0.3706089	FALSE
TC07000852.hg.1	ENSG00000181072	0.1875799	0.3706089	FALSE
TC09000535.hg.1	ENSG00000070214	0.1875799	0.3706089	FALSE
TC18000164.hg.1	ENSG00000141469	0.1875799	0.3706089	FALSE
TC06000945.hg.1	ENSG00000152661	0.1875799	0.3706089	FALSE
TC06000945.hg.1	ENSG00000152661	0.1875799	0.3706089	FALSE
TC07000852.hg.1	ENSG00000181072	0.1875799	0.3706089	FALSE
TC03001071.hg.1	ENSG00000114315	0.1875799	0.3706089	FALSE
TC14001188.hg.1	ENSG00000126778	0.1875799	0.3706089	FALSE
TC0X000943.hg.1	ENSG00000198947	0.1875799	0.3706089	FALSE
TC02002823.hg.1	ENSG00000135919	0.1875799	0.3706089	FALSE
TC10001711.hg.1	ENSG00000066468	0.1875799	0.3706089	FALSE
TC01001805.hg.1	ENSG00000092969	0.1875799	0.3706089	FALSE
TC01000904.hg.1	ENSG00000162692	0.1875799	0.3706089	FALSE

TC05000840.hg.1	ENSG00000123643	0.1875799	0.3706089	FALSE
TC11000198.hg.1	ENSG00000133816	0.1875799	0.3706089	FALSE
TC12001890.hg.1	ENSG00000017427	0.1875799	0.3706089	FALSE
TC08000508.hg.1	ENSG00000104435	0.1875799	0.3706089	FALSE
TC05000165.hg.1	ENSG00000079215	0.1875799	0.3706089	FALSE
TC05000159.hg.1	ENSG00000168685	0.1875799	0.3706089	FALSE
TC01003638.hg.1	ENSG00000073756	0.1875799	0.3706089	FALSE
TC07001630.hg.1	ENSG00000070669	0.1875799	0.3706089	FALSE
TC01003638.hg.1	ENSG00000073756	0.1875799	0.3706089	FALSE
TC10000406.hg.1	ENSG00000138336	0.1875799	0.3706089	FALSE
TC06000976.hg.1	ENSG00000196569	0.1875799	0.3706089	FALSE
TC02000762.hg.1	ENSG00000163083	0.1875799	0.3706089	FALSE
TC06004141.hg.1	ENSG00000112096	0.1875799	0.3706089	FALSE
TC22000019.hg.1	ENSG00000177663	0.1875799	0.3706089	FALSE
TC04000432.hg.1	ENSG00000156234	0.1875799	0.3706089	FALSE
TC17001682.hg.1	ENSG00000108821	0.1875799	0.3706089	FALSE
TC22000259.hg.1	ENSG00000100292	0.1875799	0.3706089	FALSE
TC13000147.hg.1	ENSG00000102760	0.1875799	0.3706089	FALSE
TC02002605.hg.1	ENSG00000064989	0.1875799	0.3706089	FALSE
TC08000149.hg.1	ENSG00000175445	0.1875799	0.3706089	FALSE
TC03000248.hg.1	ENSG00000144791	0.1875799	0.3706089	FALSE
TC05003423.hg.1	ENSG00000082196	0.1875799	0.3706089	FALSE
TC10000811.hg.1	ENSG00000150594	0.1875799	0.3706089	FALSE
TC22001477.hg.1	ENSG00000100344	0.1875799	0.3706089	FALSE
TC22000259.hg.1	ENSG00000100292	0.1875799	0.3706089	FALSE
TC01003497.hg.1	ENSG00000117479	0.1875799	0.3706089	FALSE
TC0X000034.hg.1	ENSG00000146950	0.1875799	0.3706089	FALSE
TC0X000034.hg.1	ENSG00000146950	0.1875799	0.3706089	FALSE
TC03000214.hg.1	ENSG00000168036	0.1875799	0.3706089	FALSE
TC02001100.hg.1	ENSG00000138448	0.1875799	0.3706089	FALSE
TC03000214.hg.1	ENSG00000168036	0.1875799	0.3706089	FALSE
TC22001467.hg.1	ENSG00000128394	0.1875799	0.3706089	FALSE
TC01000785.hg.1	ENSG00000154027	0.1875799	0.3706089	FALSE
TC22000259.hg.1	ENSG00000100292	0.1875799	0.3706089	FALSE
TC01001607.hg.1	ENSG00000116711	0.1875799	0.3706089	FALSE
TC15000386.hg.1	ENSG00000140285	0.1875799	0.3706089	FALSE
TC10000350.hg.1	ENSG00000107984	0.1875799	0.3706089	FALSE
TC03001071.hg.1	ENSG00000114315	0.1875799	0.3706089	FALSE
TC06000945.hg.1	ENSG00000152661	0.1875799	0.3706089	FALSE
TC15000386.hg.1	ENSG00000140285	0.1875799	0.3706089	FALSE
TC15000386.hg.1	ENSG00000140285	0.1875799	0.3706089	FALSE
TC05001519.hg.1	ENSG00000113273	0.1875799	0.3706089	FALSE
TC06000087.hg.1	ENSG00000078401	0.1875799	0.3706089	FALSE
TC15000226.hg.1	ENSG00000166923	0.1875799	0.3706089	FALSE
TC01001624.hg.1	ENSG00000116741	0.1875799	0.3706089	FALSE
TC03001071.hg.1	ENSG00000114315	0.1875799	0.3706089	FALSE
TC17000158.hg.1	ENSG00000141052	0.1875799	0.3706089	FALSE
TC06000087.hg.1	ENSG00000078401	0.1875799	0.3706089	FALSE
TC01001805.hg.1	ENSG00000092969	0.1875799	0.3706089	FALSE
TC01001624.hg.1	ENSG00000116741	0.1875799	0.3706089	FALSE
TC20000833.hg.1	ENSG00000198959	0.1875799	0.3706089	FALSE
TC20000833.hg.1	ENSG00000198959	0.1875799	0.3706089	FALSE
TC10001711.hg.1	ENSG00000066468	0.1875799	0.3706089	FALSE
TC19000191.hg.1	ENSG00000130164	0.1875799	0.3706089	FALSE
TC01000904.hg.1	ENSG00000162692	0.1875799	0.3706089	FALSE
TC05001933.hg.1	ENSG00000113721	0.1875799	0.3706089	FALSE
TC07000768.hg.1	ENSG00000174697	0.1875799	0.3706089	FALSE
TC03000214.hg.1	ENSG00000168036	0.1875799	0.3706089	FALSE

TC14000353.hg.1	ENSG00000165617	0.1875799	0.3706089	FALSE
TC05001289.hg.1	ENSG00000113594	0.1875799	0.3706089	FALSE
TC08001567.hg.1	ENSG00000170961	0.1875799	0.3706089	FALSE
TC22001467.hg.1	ENSG00000128394	0.1875799	0.3706089	FALSE
TC06001027.hg.1	ENSG00000118503	0.1875799	0.3706089	FALSE
TC06004141.hg.1	ENSG00000112096	0.1875799	0.3706089	FALSE
TC14000306.hg.1	ENSG00000125384	0.1875799	0.3706089	FALSE
TC05000701.hg.1	ENSG00000120738	0.1875799	0.3706089	FALSE
TC03000214.hg.1	ENSG00000168036	0.1875799	0.3706089	FALSE
TC03000214.hg.1	ENSG00000168036	0.1875799	0.3706089	FALSE
TC13001719.hg.1	ENSG00000150907	0.1875799	0.3706089	FALSE
TC10001498.hg.1	ENSG00000107796	0.1875799	0.3706089	FALSE
TC05000701.hg.1	ENSG00000120738	0.1875799	0.3706089	FALSE
TC10001498.hg.1	ENSG00000107796	0.1875799	0.3706089	FALSE
TC02002624.hg.1	ENSG00000115415	0.1875799	0.3706089	FALSE
TC14001188.hg.1	ENSG00000126778	0.1875799	0.3706089	FALSE
TC05000701.hg.1	ENSG00000120738	0.1875799	0.3706089	FALSE
TC02002624.hg.1	ENSG00000115415	0.1875799	0.3706089	FALSE
TC03000846.hg.1	ENSG00000196549	0.1875799	0.3706089	FALSE
TC08001659.hg.1	ENSG00000104419	0.1875799	0.3706089	FALSE
TC10000350.hg.1	ENSG00000107984	0.1875799	0.3706089	FALSE
TC10000350.hg.1	ENSG00000107984	0.1875799	0.3706089	FALSE
TC11002366.hg.1	ENSG00000076706	0.1875799	0.3706089	FALSE
TC01002706.hg.1	ENSG00000184292	0.1875799	0.3706089	FALSE
TC14000353.hg.1	ENSG00000165617	0.1875799	0.3706089	FALSE
TC0X000943.hg.1	ENSG00000198947	0.1875799	0.3706089	FALSE
TC09000535.hg.1	ENSG00000070214	0.1875799	0.3706089	FALSE
TC08001659.hg.1	ENSG00000104419	0.1875799	0.3706089	FALSE
TC06002168.hg.1	ENSG00000164442	0.1875799	0.3706089	FALSE
TC13000147.hg.1	ENSG00000102760	0.1875799	0.3706089	FALSE
TC10001517.hg.1	ENSG00000148677	0.1875799	0.3706089	FALSE
TC13000147.hg.1	ENSG00000102760	0.1875799	0.3706089	FALSE
TC01001805.hg.1	ENSG00000092969	0.1875799	0.3706089	FALSE
TC03001071.hg.1	ENSG00000114315	0.1875799	0.3706089	FALSE
TC02002624.hg.1	ENSG00000115415	0.1875799	0.3706089	FALSE
TC01003638.hg.1	ENSG00000073756	0.1875799	0.3706089	FALSE
TC09001058.hg.1	ENSG00000198467	0.1875799	0.3706089	FALSE
TC15001761.hg.1	ENSG00000103942	0.1875799	0.3706089	FALSE
TC01001258.hg.1	ENSG00000160678	0.1875799	0.3706089	FALSE
TC17001886.hg.1	ENSG00000132471	0.1875799	0.3706089	FALSE
TC13000109.hg.1	ENSG00000073910	0.1875799	0.3706089	FALSE
TC06001332.hg.1	ENSG00000168405	0.1875799	0.3706089	FALSE
TC01000115.hg.1;	ENSG00000171621;	0.1893732	0.3706089	FALSE
TC01000115.hg.1;	ENSG00000171621;	0.1893732	0.3706089	FALSE
TC02002624.hg.1	ENSG00000115415;	0.1897456	0.3707443	FALSE
TC05000054.hg.1	ENSG00000078295;	0.1897456	0.3707443	FALSE
TC06000945.hg.1	ENSG00000152661;	0.1897456	0.3707443	FALSE
TC01003638.hg.1	ENSG00000073756;	0.1897456	0.3707443	FALSE
TC03000214.hg.1	ENSG00000168036;	0.1897456	0.3707443	FALSE
TC01001805.hg.1	ENSG00000092969;	0.1901057	0.37094	FALSE
TC02000082.hg.1	ENSG00000071575;	0.1905157	0.3715707	FALSE
TC01000904.hg.1	ENSG00000162692;	0.1926417	0.375546	FALSE
TC01003129.hg.1	ENSG00000143140;	0.1929831	0.3760403	FALSE
TC03001362.hg.1	ENSG00000160808;	0.1946622	0.3772516	FALSE
TC06000728.hg.1	ENSG00000185760;	0.1946622	0.3772516	FALSE
TC01001607.hg.1	ENSG00000116711;I	0.1946622	0.3772516	FALSE
TC19001250.hg.1	ENSG00000074181;	0.1946622	0.3772516	FALSE
TC10000350.hg.1	ENSG00000107984;	0.1946622	0.3772516	FALSE

TC02001100.hg.1;	ENSG00000138448;	0.1946622	0.3772516	FALSE
TC02002808.hg.1	ENSG00000116106;	0.1946622	0.3772516	FALSE
TC02000041.hg.1	ENSG00000115738;	0.1946622	0.3772516	FALSE
TC03002146.hg.1	ENSG00000189058;	0.1946622	0.3772516	FALSE
TC08001275.hg.1	ENSG00000172817;	0.1946622	0.3772516	FALSE
TC01003638.hg.1	ENSG00000073756;	0.1946622	0.3772516	FALSE
TC09000222.hg.1	ENSG00000137124;	0.1946622	0.3772516	FALSE
TC04001169.hg.1;	ENSG00000145244;	0.1946622	0.3772516	FALSE
TC07000137.hg.1	ENSG00000136244;	0.1946622	0.3772516	FALSE
TC07001897.hg.1	ENSG00000157680;	0.1946622	0.3772516	FALSE
TC07000137.hg.1	ENSG00000136244;	0.1946622	0.3772516	FALSE
TC11000707.hg.1;	ENSG00000162337;	0.1946622	0.3772516	FALSE
TC17000220.hg.1	ENSG00000108448;	0.1946622	0.3772516	FALSE
TC01003638.hg.1	ENSG00000073756;	0.1946622	0.3772516	FALSE
TC10001711.hg.1;	ENSG00000066468;	0.1946622	0.3772516	FALSE
TC08001062.hg.1	ENSG00000159167;	0.1946622	0.3772516	FALSE
TC07000328.hg.1	ENSG00000146648;	0.1946622	0.3772516	FALSE
TC10000438.hg.1	ENSG00000107731;	0.1946622	0.3772516	FALSE
TC04000408.hg.1	ENSG00000169429;	0.1963821	0.3785285	FALSE
TC06001027.hg.1	ENSG00000118503;	0.1968152	0.379022	FALSE
TC04000408.hg.1	ENSG00000169429;	0.1968152	0.379022	FALSE
TC01000685.hg.1	ENSG00000162409;	0.1968152	0.379022	FALSE
TC01000904.hg.1	ENSG00000162692;	0.1971093	0.3792472	FALSE
TC05000054.hg.1	ENSG00000078295;	0.2002352	0.3846564	FALSE
TC04001084.hg.1	ENSG00000109819;	0.2002352	0.3846564	FALSE
TC01000685.hg.1	ENSG00000162409;	0.2002352	0.3846564	FALSE
TC03002146.hg.1	ENSG00000189058;	0.2002352	0.3846564	FALSE
TC10001711.hg.1;	ENSG00000066468;	0.2002352	0.3846564	FALSE
TC02000270.hg.1	ENSG00000152527;	0.2002352	0.3846564	FALSE
TC03002146.hg.1	ENSG00000189058;	0.2013953	0.3862784	FALSE
TC02002605.hg.1	ENSG00000064989;	0.2027197	0.3885574	FALSE
TC02002624.hg.1	ENSG00000115415;	0.2027197	0.3885574	FALSE
TC01001090.hg.1	ENSG00000117289;	0.2040121	0.3906848	FALSE
TC04000408.hg.1	ENSG00000169429;	0.2040121	0.3906848	FALSE
TC01002706.hg.1	ENSG00000184292;	0.2041544	0.3906952	FALSE
TC01000904.hg.1	ENSG00000162692;	0.2044028	0.3909958	FALSE
TC01001473.hg.1	ENSG00000143153;	0.2052516	0.3923563	FALSE
TC04000408.hg.1	ENSG00000169429;	0.2052516	0.3923563	FALSE
TC01003791.hg.1	ENSG00000143473;	0.2057834	0.393022	FALSE
TC01003791.hg.1	ENSG00000143473;	0.2057834	0.393022	FALSE
TC02001628.hg.1	ENSG00000084674;	0.2091307	0.3983264	FALSE
TC02001628.hg.1	ENSG00000084674;	0.2091307	0.3983264	FALSE
TC11001026.hg.1;	ENSG00000109906;	0.2097085	0.3983264	FALSE
TC16000668.hg.1	ENSG00000103241;	0.2097085	0.3983264	FALSE
TC0X000038.hg.1	ENSG00000073464;	0.2097085	0.3983264	FALSE
TC07000137.hg.1	ENSG00000136244;	0.2097085	0.3983264	FALSE
TC0X000038.hg.1	ENSG00000073464;	0.2097085	0.3983264	FALSE
TC07001552.hg.1	ENSG00000187391;	0.2097085	0.3983264	FALSE
TC02002624.hg.1	ENSG00000115415;	0.2097085	0.3983264	FALSE
TC02002823.hg.1	ENSG00000135919;	0.2097085	0.3983264	FALSE
TC06000779.hg.1	ENSG00000135318;	0.2097085	0.3983264	FALSE
TC01001607.hg.1	ENSG00000116711;I	0.2097085	0.3983264	FALSE
TC11000707.hg.1;	ENSG00000162337;	0.2097085	0.3983264	FALSE
TC08000646.hg.1	ENSG00000164932;	0.2099145	0.3983264	FALSE
TC02002714.hg.1	ENSG00000163251;	0.210003	0.3983264	FALSE
TC01001805.hg.1	ENSG00000092969;	0.210858	0.3993703	FALSE
TC01000728.hg.1	ENSG00000162433;	0.210858	0.3993703	FALSE
TC06001027.hg.1	ENSG00000118503;	0.210858	0.3993703	FALSE

TC01003638.hg.1	ENSG00000073756;	0.210858	0.3993703	FALSE
TC02002823.hg.1	ENSG00000135919;	0.210858	0.3993703	FALSE
TC02000041.hg.1	ENSG00000115738;	0.2112954	0.3993703	FALSE
TC01003638.hg.1	ENSG00000073756;	0.2112954	0.3993703	FALSE
TC01002669.hg.1	ENSG00000157193;	0.2112996	0.3993703	FALSE
TC01000100.hg.1	ENSG00000049246;	0.2128369	0.4008753	FALSE
TC01000685.hg.1	ENSG00000162409;	0.2133716	0.4008753	FALSE
TC01001624.hg.1	ENSG00000116741;	0.216297	0.4008753	FALSE
TC01003638.hg.1	ENSG00000073756;	0.2169376	0.4008753	FALSE
TC06000087.hg.1	ENSG00000078401;	0.2185337	0.4008753	FALSE
TC02002714.hg.1	ENSG00000163251;	0.2185337	0.4008753	FALSE
TC08000646.hg.1	ENSG00000164932;	0.2185337	0.4008753	FALSE
TC01000904.hg.1	ENSG00000162692;	0.219337	0.4008753	FALSE
TC14001364.hg.1	ENSG00000211448	0.2206483	0.4008753	FALSE
TC15000971.hg.1	ENSG00000184254	0.2206483	0.4008753	FALSE
TC04000313.hg.1	ENSG00000157404	0.2206483	0.4008753	FALSE
TC19001103.hg.1	ENSG00000125730	0.2206483	0.4008753	FALSE
TC19001103.hg.1	ENSG00000125730	0.2206483	0.4008753	FALSE
TC08001567.hg.1	ENSG00000170961	0.2206483	0.4008753	FALSE
TC03000214.hg.1	ENSG00000168036	0.2206483	0.4008753	FALSE
TC11000404.hg.1	ENSG00000134574	0.2206483	0.4008753	FALSE
TC14001364.hg.1	ENSG00000211448	0.2206483	0.4008753	FALSE
TC06002036.hg.1	ENSG00000249853	0.2206483	0.4008753	FALSE
TC05000165.hg.1	ENSG00000079215	0.2206483	0.4008753	FALSE
TC01001805.hg.1	ENSG00000092969	0.2206483	0.4008753	FALSE
TC11001026.hg.1	ENSG00000109906	0.2206483	0.4008753	FALSE
TC10000663.hg.1	ENSG00000095596	0.2206483	0.4008753	FALSE
TC01000785.hg.1	ENSG00000154027	0.2206483	0.4008753	FALSE
TC10000350.hg.1	ENSG00000107984	0.2206483	0.4008753	FALSE
TC01001624.hg.1	ENSG00000116741	0.2206483	0.4008753	FALSE
TC02002480.hg.1	ENSG00000078098	0.2206483	0.4008753	FALSE
TC19000191.hg.1	ENSG00000130164	0.2206483	0.4008753	FALSE
TC08000149.hg.1	ENSG00000175445	0.2206483	0.4008753	FALSE
TC12001324.hg.1	ENSG00000123095	0.2206483	0.4008753	FALSE
TC03002146.hg.1	ENSG00000189058	0.2206483	0.4008753	FALSE
TC02002823.hg.1	ENSG00000135919	0.2206483	0.4008753	FALSE
TC0X000943.hg.1	ENSG00000198947	0.2206483	0.4008753	FALSE
TC05000840.hg.1	ENSG00000123643	0.2206483	0.4008753	FALSE
TC05000840.hg.1	ENSG00000123643	0.2206483	0.4008753	FALSE
TC08000127.hg.1	ENSG00000003989	0.2206483	0.4008753	FALSE
TC05000840.hg.1	ENSG00000123643	0.2206483	0.4008753	FALSE
TC10000663.hg.1	ENSG00000095596	0.2206483	0.4008753	FALSE
TC01003778.hg.1	ENSG00000076356	0.2206483	0.4008753	FALSE
TC10001327.hg.1	ENSG00000122877	0.2206483	0.4008753	FALSE
TC02002823.hg.1	ENSG00000135919	0.2206483	0.4008753	FALSE
TC01002669.hg.1	ENSG00000157193	0.2206483	0.4008753	FALSE
TC10001711.hg.1	ENSG00000066468	0.2206483	0.4008753	FALSE
TC10001711.hg.1	ENSG00000066468	0.2206483	0.4008753	FALSE
TC07000768.hg.1	ENSG00000174697	0.2206483	0.4008753	FALSE
TC08001175.hg.1	ENSG00000104368	0.2206483	0.4008753	FALSE
TC11002140.hg.1	ENSG00000149256	0.2206483	0.4008753	FALSE
TC06000976.hg.1	ENSG00000196569	0.2206483	0.4008753	FALSE
TC08001659.hg.1	ENSG00000104419	0.2206483	0.4008753	FALSE
TC10001663.hg.1	ENSG00000119927	0.2206483	0.4008753	FALSE
TC22000019.hg.1	ENSG00000177663	0.2206483	0.4008753	FALSE
TC10000811.hg.1	ENSG00000150594	0.2206483	0.4008753	FALSE
TC08001275.hg.1	ENSG00000172817	0.2206483	0.4008753	FALSE
TC17000383.hg.1	ENSG00000108691	0.2206483	0.4008753	FALSE

TC17000383.hg.1	ENSG00000108691	0.2206483	0.4008753	FALSE
TC07001562.hg.1	ENSG00000019991	0.2206483	0.4008753	FALSE
TC07001562.hg.1	ENSG00000019991	0.2206483	0.4008753	FALSE
TC01001607.hg.1	ENSG00000116711	0.2206483	0.4008753	FALSE
TC17001462.hg.1	ENSG00000131747	0.2206483	0.4008753	FALSE
TC10000663.hg.1	ENSG00000095596	0.2206483	0.4008753	FALSE
TC06000087.hg.1	ENSG00000078401	0.2206483	0.4008753	FALSE
TC10000350.hg.1	ENSG00000107984	0.2206483	0.4008753	FALSE
TC12000227.hg.1	ENSG00000172572	0.2206483	0.4008753	FALSE
TC17000383.hg.1	ENSG00000108691	0.2206483	0.4008753	FALSE
TC01001624.hg.1	ENSG00000116741	0.2206483	0.4008753	FALSE
TC07000137.hg.1	ENSG00000136244	0.2206483	0.4008753	FALSE
TC07000137.hg.1	ENSG00000136244	0.2206483	0.4008753	FALSE
TC03001527.hg.1	ENSG00000163638	0.2206483	0.4008753	FALSE
TC06000087.hg.1	ENSG00000078401	0.2206483	0.4008753	FALSE
TC01001736.hg.1	ENSG00000196352	0.2206483	0.4008753	FALSE
TC06002036.hg.1	ENSG00000249853	0.2206483	0.4008753	FALSE
TC07000768.hg.1	ENSG00000174697	0.2206483	0.4008753	FALSE
TC11002427.hg.1	ENSG00000064309	0.2206483	0.4008753	FALSE
TC18000548.hg.1	ENSG00000183287	0.2206483	0.4008753	FALSE
TC03001527.hg.1	ENSG00000163638	0.2206483	0.4008753	FALSE
TC01002706.hg.1	ENSG00000184292	0.2206483	0.4008753	FALSE
TC01001624.hg.1	ENSG00000116741	0.2206483	0.4008753	FALSE
TC01000734.hg.1	ENSG00000118473	0.2206483	0.4008753	FALSE
TC10001711.hg.1	ENSG00000066468	0.2206483	0.4008753	FALSE
TC10000406.hg.1	ENSG00000138336	0.2206483	0.4008753	FALSE
TC11002170.hg.1	ENSG00000174804	0.2206483	0.4008753	FALSE
TC01002669.hg.1	ENSG00000157193	0.2206483	0.4008753	FALSE
TC07000004.hg.1	ENSG00000177706	0.2206483	0.4008753	FALSE
TC09001336.hg.1	ENSG00000106819	0.2206483	0.4008753	FALSE
TC05001110.hg.1	ENSG00000153395	0.2206483	0.4008753	FALSE
TC10001498.hg.1	ENSG00000107796	0.2206483	0.4008753	FALSE
TC03001071.hg.1	ENSG00000114315	0.2206483	0.4008753	FALSE
TC03001071.hg.1	ENSG00000114315	0.2206483	0.4008753	FALSE
TC01003129.hg.1	ENSG00000143140	0.2206483	0.4008753	FALSE
TC05001933.hg.1	ENSG00000113721	0.2206483	0.4008753	FALSE
TC20000162.hg.1	ENSG00000101463	0.2206483	0.4008753	FALSE
TC16000571.hg.1	ENSG00000198373	0.2206483	0.4008753	FALSE
TC03001071.hg.1	ENSG00000114315	0.2206483	0.4008753	FALSE
TC14000951.hg.1	ENSG00000092068	0.2206483	0.4008753	FALSE
TC17001459.hg.1	ENSG00000126368	0.2206483	0.4008753	FALSE
TC01001736.hg.1	ENSG00000196352	0.2206483	0.4008753	FALSE
TC04000432.hg.1	ENSG00000156234	0.2206483	0.4008753	FALSE
TC17000819.hg.1	ENSG00000125398	0.2206483	0.4008753	FALSE
TC12001755.hg.1	ENSG00000175183	0.2206483	0.4008753	FALSE
TC02001031.hg.1	ENSG00000152256;	0.2216004	0.4008753	FALSE
TC01002706.hg.1	ENSG00000184292;	0.2216004	0.4008753	FALSE
TC01001805.hg.1	ENSG00000092969;	0.2216004	0.4008753	FALSE
TC10000663.hg.1	ENSG00000095596;	0.2216004	0.4008753	FALSE
TC04001245.hg.1	ENSG00000145242;	0.2216004	0.4008753	FALSE
TC0Y000185.hg.1	ENSG00000012817;	0.2216004	0.4008753	FALSE
TC01001473.hg.1	ENSG00000143153;	0.2228692	0.4024902	FALSE
TC02001100.hg.1;	ENSG00000138448;	0.2228692	0.4024902	FALSE
TC02002624.hg.1	ENSG00000115415;	0.2248654	0.4048145	FALSE
TC06001772.hg.1	ENSG00000146233;	0.2248654	0.4048145	FALSE
TC08000482.hg.1	ENSG00000121039;	0.2248654	0.4048145	FALSE
TC08001567.hg.1	ENSG00000170961;	0.2248654	0.4048145	FALSE
TC05001590.hg.1	ENSG00000113369;	0.2248654	0.4048145	FALSE

TC03000214.hg.1	ENSG00000168036;	0.2248654	0.4048145	FALSE
TC14001122.hg.1;	ENSG00000151748;	0.2248654	0.4048145	FALSE
TC02000286.hg.1	ENSG00000171150;	0.2248654	0.4048145	FALSE
TC10001663.hg.1	ENSG00000119927;	0.2248654	0.4048145	FALSE
TC04000432.hg.1	ENSG00000156234;	0.2248654	0.4048145	FALSE
TC03001255.hg.1	ENSG00000033867;	0.2248654	0.4048145	FALSE
TC01003129.hg.1	ENSG00000143140;	0.2248654	0.4048145	FALSE
TC0X001186.hg.1	ENSG00000072133;	0.2248654	0.4048145	FALSE
TC01001624.hg.1	ENSG00000116741;	0.2258162	0.4053329	FALSE
TC02001628.hg.1	ENSG00000084674;	0.2261041	0.4056796	FALSE
TC06000087.hg.1	ENSG00000078401;	0.227246	0.4075577	FALSE
TC01000100.hg.1	ENSG00000049246;	0.2277468	0.4082848	FALSE
TC01001624.hg.1	ENSG00000116741;	0.2279473	0.4084731	FALSE
TC01000904.hg.1	ENSG00000162692;	0.2280942	0.4085654	FALSE
TC03001362.hg.1	ENSG00000160808;	0.232449	0.4161046	FALSE
TC05003439.hg.1	ENSG00000213949;	0.232449	0.4161046	FALSE
TC01001473.hg.1	ENSG00000143153;	0.2336212	0.4178535	FALSE
TC01000783.hg.1	ENSG00000117069;	0.2336212	0.4178535	FALSE
TC02000762.hg.1	ENSG00000163083;	0.2348991	0.4198761	FALSE
TC01001792.hg.1	ENSG00000082482;	0.2354526	0.4206019	FALSE
TC06002168.hg.1	ENSG00000164442;	0.2354526	0.4206019	FALSE
TC04000253.hg.1	ENSG00000154277;	0.2360446	0.4213957	FALSE
TC03001590.hg.1	ENSG00000184500;	0.2367021	0.4223934	FALSE
TC01002669.hg.1	ENSG00000157193;	0.2369971	0.4227436	FALSE
TC02000041.hg.1	ENSG00000115738;	0.2385711	0.4253739	FALSE
TC07001374.hg.1	ENSG00000106070;	0.2400991	0.4259918	FALSE
TC07001374.hg.1	ENSG00000106070;	0.2400991	0.4259918	FALSE
TC07000525.hg.1	ENSG00000008277;	0.2400991	0.4259918	FALSE
TC05000840.hg.1	ENSG00000123643;	0.2400991	0.4259918	FALSE
TC02002808.hg.1	ENSG00000116106;	0.2400991	0.4259918	FALSE
TC03001362.hg.1	ENSG00000160808;	0.2400991	0.4259918	FALSE
TC02000270.hg.1	ENSG00000152527;	0.2400991	0.4259918	FALSE
TC17000220.hg.1	ENSG00000108448;	0.2400991	0.4259918	FALSE
TC01003718.hg.1	ENSG00000133048;	0.2400991	0.4259918	FALSE
TC02000041.hg.1	ENSG00000115738;	0.2400991	0.4259918	FALSE
TC11002178.hg.1;	ENSG00000086991;	0.2400991	0.4259918	FALSE
TC10001711.hg.1;	ENSG00000066468;	0.2400991	0.4259918	FALSE
TC01000904.hg.1	ENSG00000162692;	0.2402112	0.4259918	FALSE
TC02002605.hg.1	ENSG00000064989;	0.2409934	0.4272019	FALSE
TC02000466.hg.1	ENSG00000159399;	0.2412079	0.4274052	FALSE
TC04002931.hg.1	ENSG00000134853;	0.241602	0.4279264	FALSE
TC01000734.hg.1	ENSG00000118473;	0.2428513	0.4299613	FALSE
TC02002823.hg.1	ENSG00000135919;	0.2433907	0.4305602	FALSE
TC05000165.hg.1	ENSG00000079215;	0.2433907	0.4305602	FALSE
TC03001071.hg.1	ENSG00000114315;	0.2433907	0.4305602	FALSE
TC03000013.hg.1	ENSG00000150995;	0.2488599	0.4323241	FALSE
TC03000013.hg.1	ENSG00000150995;	0.2488599	0.4323241	FALSE
TC01001704.hg.1	ENSG00000163531;	0.2488599	0.4323241	FALSE
TC01000100.hg.1	ENSG00000049246;	0.249158	0.4323241	FALSE
TC01000904.hg.1	ENSG00000162692;	0.2516292	0.4323241	FALSE
TC17001462.hg.1	ENSG00000131747	0.2523727	0.4323241	FALSE
TC02002049.hg.1	ENSG00000115525	0.2523727	0.4323241	FALSE
TC02000041.hg.1	ENSG00000115738	0.2523727	0.4323241	FALSE
TC16000454.hg.1	ENSG00000087245	0.2523727	0.4323241	FALSE
TC04000313.hg.1	ENSG00000157404	0.2523727	0.4323241	FALSE
TC04000313.hg.1	ENSG00000157404	0.2523727	0.4323241	FALSE
TC06001027.hg.1	ENSG00000118503	0.2523727	0.4323241	FALSE
TC06001027.hg.1	ENSG00000118503	0.2523727	0.4323241	FALSE

TC03000214.hg.1	ENSG00000168036	0.2523727	0.4323241	FALSE
TC03000013.hg.1	ENSG00000150995	0.2523727	0.4323241	FALSE
TC10000022.hg.1	ENSG00000067057	0.2523727	0.4323241	FALSE
TC09000222.hg.1	ENSG00000137124	0.2523727	0.4323241	FALSE
TC05000054.hg.1	ENSG00000078295	0.2523727	0.4323241	FALSE
TC08000203.hg.1	ENSG00000092964	0.2523727	0.4323241	FALSE
TC17001462.hg.1	ENSG00000131747	0.2523727	0.4323241	FALSE
TC11000182.hg.1	ENSG00000148926	0.2523727	0.4323241	FALSE
TC15000622.hg.1	ENSG00000166949	0.2523727	0.4323241	FALSE
TC16000668.hg.1	ENSG00000103241	0.2523727	0.4323241	FALSE
TC03000862.hg.1	ENSG00000163661	0.2523727	0.4323241	FALSE
TC07000768.hg.1	ENSG00000174697	0.2523727	0.4323241	FALSE
TC06000779.hg.1	ENSG00000135318	0.2523727	0.4323241	FALSE
TC01000785.hg.1	ENSG00000154027	0.2523727	0.4323241	FALSE
TC12000076.hg.1	ENSG00000010278	0.2523727	0.4323241	FALSE
TC11002427.hg.1	ENSG00000064309	0.2523727	0.4323241	FALSE
TC22001467.hg.1	ENSG00000128394	0.2523727	0.4323241	FALSE
TC22001467.hg.1	ENSG00000128394	0.2523727	0.4323241	FALSE
TC03002146.hg.1	ENSG00000189058	0.2523727	0.4323241	FALSE
TC10000811.hg.1	ENSG00000150594	0.2523727	0.4323241	FALSE
TC17000383.hg.1	ENSG00000108691	0.2523727	0.4323241	FALSE
TC10000811.hg.1	ENSG00000150594	0.2523727	0.4323241	FALSE
TC01001624.hg.1	ENSG00000116741	0.2523727	0.4323241	FALSE
TC20000926.hg.1	ENSG00000158445	0.2523727	0.4323241	FALSE
TC12001837.hg.1	ENSG00000074527	0.2523727	0.4323241	FALSE
TC01002669.hg.1	ENSG00000157193	0.2523727	0.4323241	FALSE
TC08001022.hg.1	ENSG00000147408	0.2523727	0.4323241	FALSE
TC03001071.hg.1	ENSG00000114315	0.2523727	0.4323241	FALSE
TC06004141.hg.1	ENSG00000112096	0.2523727	0.4323241	FALSE
TC06001027.hg.1	ENSG00000118503	0.2523727	0.4323241	FALSE
TC08000149.hg.1	ENSG00000175445	0.2523727	0.4323241	FALSE
TC02002624.hg.1	ENSG00000115415	0.2523727	0.4323241	FALSE
TC04000313.hg.1	ENSG00000157404	0.2523727	0.4323241	FALSE
TC09000358.hg.1	ENSG00000135069	0.2523727	0.4323241	FALSE
TC02000281.hg.1	ENSG00000116016	0.2523727	0.4323241	FALSE
TC04000313.hg.1	ENSG00000157404	0.2523727	0.4323241	FALSE
TC02000041.hg.1	ENSG00000115738	0.2523727	0.4323241	FALSE
TC02000286.hg.1	ENSG00000171150	0.2523727	0.4323241	FALSE
TC10001517.hg.1	ENSG00000148677	0.2523727	0.4323241	FALSE
TC10000406.hg.1	ENSG00000138336	0.2523727	0.4323241	FALSE
TC02001100.hg.1	ENSG00000138448	0.2523727	0.4323241	FALSE
TC02000286.hg.1	ENSG00000171150	0.2523727	0.4323241	FALSE
TC07001374.hg.1	ENSG00000106070	0.2523727	0.4323241	FALSE
TC01000785.hg.1	ENSG00000154027	0.2523727	0.4323241	FALSE
TC02000511.hg.1	ENSG00000168906	0.2523727	0.4323241	FALSE
TC02000041.hg.1	ENSG00000115738	0.2523727	0.4323241	FALSE
TC02000041.hg.1	ENSG00000115738	0.2523727	0.4323241	FALSE
TC11002178.hg.1	ENSG00000086991	0.2523727	0.4323241	FALSE
TC07000137.hg.1	ENSG00000136244	0.2523727	0.4323241	FALSE
TC05000165.hg.1	ENSG00000079215	0.2523727	0.4323241	FALSE
TC03000248.hg.1	ENSG00000144791	0.2523727	0.4323241	FALSE
TC03001071.hg.1	ENSG00000114315	0.2523727	0.4323241	FALSE
TC10001711.hg.1	ENSG00000066468	0.2523727	0.4323241	FALSE
TC02002714.hg.1	ENSG00000163251	0.2523727	0.4323241	FALSE
TC17000015.hg.1	ENSG00000132386	0.2523727	0.4323241	FALSE
TC11002140.hg.1	ENSG00000149256	0.2523727	0.4323241	FALSE
TC03000248.hg.1	ENSG00000144791	0.2523727	0.4323241	FALSE
TC03000248.hg.1	ENSG00000144791	0.2523727	0.4323241	FALSE

TC05001933.hg.1	ENSG00000113721	0.2523727	0.4323241	FALSE
TC03000214.hg.1	ENSG00000168036	0.2523727	0.4323241	FALSE
TC20000621.hg.1	ENSG00000101384	0.2523727	0.4323241	FALSE
TC01001805.hg.1	ENSG00000092969	0.2523727	0.4323241	FALSE
TC12001890.hg.1	ENSG00000017427	0.2523727	0.4323241	FALSE
TC07000768.hg.1	ENSG00000174697	0.2523727	0.4323241	FALSE
TC10000811.hg.1	ENSG00000150594	0.2523727	0.4323241	FALSE
TC20000621.hg.1	ENSG00000101384	0.2523727	0.4323241	FALSE
TC07001562.hg.1	ENSG00000019991	0.2523727	0.4323241	FALSE
TC22000019.hg.1	ENSG00000177663	0.2523727	0.4323241	FALSE
TC05000701.hg.1	ENSG00000120738	0.2523727	0.4323241	FALSE
TC10001517.hg.1	ENSG00000148677	0.2523727	0.4323241	FALSE
TC19000677.hg.1	ENSG00000134830	0.2523727	0.4323241	FALSE
TC06000945.hg.1	ENSG00000152661	0.2523727	0.4323241	FALSE
TC11000182.hg.1	ENSG00000148926	0.2523727	0.4323241	FALSE
TC06002302.hg.1	ENSG00000223414	0.2523727	0.4323241	FALSE
TC08000646.hg.1	ENSG00000164932;	0.2538731	0.4323241	FALSE
TC02000041.hg.1	ENSG00000115738;	0.2538731	0.4323241	FALSE
TC03000214.hg.1	ENSG00000168036;	0.254413	0.4323241	FALSE
TC01003497.hg.1	ENSG00000117479;	0.254413	0.4323241	FALSE
TC02002823.hg.1	ENSG00000135919;	0.254413	0.4323241	FALSE
TC01003638.hg.1	ENSG00000073756;	0.254413	0.4323241	FALSE
TC01001805.hg.1	ENSG00000092969;	0.254413	0.4323241	FALSE
TC03000214.hg.1	ENSG00000168036;	0.254413	0.4323241	FALSE
TC03002146.hg.1	ENSG00000189058;	0.254413	0.4323241	FALSE
TC03000214.hg.1	ENSG00000168036;	0.254413	0.4323241	FALSE
TC04000313.hg.1	ENSG00000157404;	0.2553781	0.4323241	FALSE
TC07000559.hg.1	ENSG00000164692;	0.2553781	0.4323241	FALSE
TC02002823.hg.1	ENSG00000135919;	0.2553781	0.4323241	FALSE
TC04000432.hg.1	ENSG00000156234;	0.2553781	0.4323241	FALSE
TC03001362.hg.1	ENSG00000160808;	0.2553781	0.4323241	FALSE
TC03000214.hg.1	ENSG00000168036;	0.2553781	0.4323241	FALSE
TC02000762.hg.1	ENSG00000163083;	0.2553781	0.4323241	FALSE
TC10000811.hg.1;	ENSG00000150594;	0.2553781	0.4323241	FALSE
TC01002578.hg.1	ENSG00000117394;	0.2553781	0.4323241	FALSE
TC07000768.hg.1	ENSG00000174697;	0.2553781	0.4323241	FALSE
TC05003423.hg.1	ENSG00000082196;	0.2553781	0.4323241	FALSE
TC05003423.hg.1	ENSG00000082196;	0.2553781	0.4323241	FALSE
TC01000904.hg.1	ENSG00000162692;	0.2555974	0.4323241	FALSE
TC01003638.hg.1	ENSG00000073756;	0.2558734	0.4326201	FALSE
TC01001792.hg.1	ENSG00000082482;	0.2576541	0.4353728	FALSE
TC01001792.hg.1	ENSG00000082482;	0.2576541	0.4353728	FALSE
TC01000904.hg.1	ENSG00000162692;	0.2613557	0.4413664	FALSE
TC01002166.hg.1	ENSG00000116285;	0.2615546	0.4415281	FALSE
TC02002605.hg.1	ENSG00000064989;	0.2627223	0.4431702	FALSE
TC02002605.hg.1	ENSG00000064989;	0.2628897	0.4431702	FALSE
TC01003638.hg.1	ENSG00000073756;	0.2628897	0.4431702	FALSE
TC01002706.hg.1	ENSG00000184292;	0.2628897	0.4431702	FALSE
TC02002605.hg.1	ENSG00000064989;	0.2628897	0.4431702	FALSE
TC06000945.hg.1	ENSG00000152661;	0.264343	0.4451819	FALSE
TC02000082.hg.1	ENSG00000071575;	0.2655034	0.4467846	FALSE
TC01001805.hg.1	ENSG00000092969;	0.2655034	0.4467846	FALSE
TC01001805.hg.1	ENSG00000092969;	0.2655034	0.4467846	FALSE
TC02000286.hg.1	ENSG00000171150;	0.2658496	0.4470158	FALSE
TC01000100.hg.1	ENSG00000049246;	0.2659893	0.447075	FALSE
TC01002669.hg.1	ENSG00000157193;	0.2662879	0.4474012	FALSE
TC02001031.hg.1	ENSG00000152256;	0.2691606	0.4520505	FALSE
TC01000685.hg.1	ENSG00000162409;	0.2696661	0.4527218	FALSE

TC02002714.hg.1	ENSG00000163251;	0.2706736	0.4529859	FALSE
TC02001628.hg.1	ENSG00000084674;	0.2706736	0.4529859	FALSE
TC03001071.hg.1	ENSG00000114315;	0.2706736	0.4529859	FALSE
TC04001084.hg.1	ENSG00000109819;	0.2706736	0.4529859	FALSE
TC04000313.hg.1	ENSG00000157404;	0.2706736	0.4529859	FALSE
TC12001890.hg.1	ENSG00000017427;	0.2706736	0.4529859	FALSE
TC01001607.hg.1	ENSG00000116711;I	0.2706736	0.4529859	FALSE
TC01003497.hg.1	ENSG00000117479;	0.2706736	0.4529859	FALSE
TC03000631.hg.1	ENSG00000114023;	0.2706736	0.4529859	FALSE
TC0X000943.hg.1	ENSG00000198947;	0.2706736	0.4529859	FALSE
TC10000438.hg.1	ENSG00000107731;	0.2706736	0.4529859	FALSE
TC07000137.hg.1	ENSG00000136244;	0.2719655	0.4529859	FALSE
TC03001071.hg.1	ENSG00000114315;	0.2719655	0.4529859	FALSE
TC06000945.hg.1	ENSG00000152661;	0.2721657	0.4529859	FALSE
TC07000768.hg.1	ENSG00000174697;	0.2721657	0.4529859	FALSE
TC01000873.hg.1	ENSG00000143036;	0.2750844	0.4529859	FALSE
TC04002931.hg.1	ENSG00000134853;	0.27665	0.4529859	FALSE
TC04001245.hg.1	ENSG00000145242;	0.27665	0.4529859	FALSE
TC02002808.hg.1	ENSG00000116106;	0.2766546	0.4529859	FALSE
TC02000041.hg.1	ENSG00000115738;	0.2766546	0.4529859	FALSE
TC01001624.hg.1	ENSG00000116741;	0.2766652	0.4529859	FALSE
TC01000873.hg.1	ENSG00000143036;	0.2766652	0.4529859	FALSE
TC01000873.hg.1	ENSG00000143036;	0.2768598	0.4529859	FALSE
TC01003638.hg.1	ENSG00000073756;	0.2785352	0.4529859	FALSE
TC01001473.hg.1	ENSG00000143153;	0.2791627	0.4529859	FALSE
TC01000904.hg.1	ENSG00000162692;	0.2795189	0.4529859	FALSE
TC04000313.hg.1	ENSG00000157404;	0.2810941	0.4529859	FALSE
TC01000734.hg.1	ENSG00000118473;	0.2810941	0.4529859	FALSE
TC18000097.hg.1	ENSG00000101773	0.2828076	0.4529859	FALSE
TC04000313.hg.1	ENSG00000157404	0.2828076	0.4529859	FALSE
TC19001103.hg.1	ENSG00000125730	0.2828076	0.4529859	FALSE
TC01001736.hg.1	ENSG00000196352	0.2828076	0.4529859	FALSE
TC10001711.hg.1	ENSG00000066468	0.2828076	0.4529859	FALSE
TC02002624.hg.1	ENSG00000115415	0.2828076	0.4529859	FALSE
TC01001473.hg.1	ENSG00000143153	0.2828076	0.4529859	FALSE
TC22001467.hg.1	ENSG00000128394	0.2828076	0.4529859	FALSE
TC12002066.hg.1	ENSG00000158104	0.2828076	0.4529859	FALSE
TC12002066.hg.1	ENSG00000158104	0.2828076	0.4529859	FALSE
TC09000358.hg.1	ENSG00000135069	0.2828076	0.4529859	FALSE
TC06001772.hg.1	ENSG00000146233	0.2828076	0.4529859	FALSE
TC05001172.hg.1	ENSG00000154122	0.2828076	0.4529859	FALSE
TC01000685.hg.1	ENSG00000162409	0.2828076	0.4529859	FALSE
TC10000663.hg.1	ENSG00000095596	0.2828076	0.4529859	FALSE
TC03000208.hg.1	ENSG00000168032	0.2828076	0.4529859	FALSE
TC01000785.hg.1	ENSG00000154027	0.2828076	0.4529859	FALSE
TC01000785.hg.1	ENSG00000154027	0.2828076	0.4529859	FALSE
TC22001467.hg.1	ENSG00000128394	0.2828076	0.4529859	FALSE
TC10000350.hg.1	ENSG00000107984	0.2828076	0.4529859	FALSE
TC06001142.hg.1	ENSG00000164694	0.2828076	0.4529859	FALSE
TC04000432.hg.1	ENSG00000156234	0.2828076	0.4529859	FALSE
TC04000432.hg.1	ENSG00000156234	0.2828076	0.4529859	FALSE
TC04001084.hg.1	ENSG00000109819	0.2828076	0.4529859	FALSE
TC05000165.hg.1	ENSG00000079215	0.2828076	0.4529859	FALSE
TC01003497.hg.1	ENSG00000117479	0.2828076	0.4529859	FALSE
TC06001772.hg.1	ENSG00000146233	0.2828076	0.4529859	FALSE
TC20000108.hg.1	ENSG00000125851	0.2828076	0.4529859	FALSE
TC10001711.hg.1	ENSG00000066468	0.2828076	0.4529859	FALSE
TC03001071.hg.1	ENSG00000114315	0.2828076	0.4529859	FALSE

TC08001022.hg.1	ENSG00000147408	0.2828076	0.4529859	FALSE
TC10001711.hg.1	ENSG00000066468	0.2828076	0.4529859	FALSE
TC10000406.hg.1	ENSG00000138336	0.2828076	0.4529859	FALSE
TC01002166.hg.1	ENSG00000116285	0.2828076	0.4529859	FALSE
TC01001607.hg.1	ENSG00000116711	0.2828076	0.4529859	FALSE
TC01002706.hg.1	ENSG00000184292	0.2828076	0.4529859	FALSE
TC05000840.hg.1	ENSG00000123643	0.2828076	0.4529859	FALSE
TC06001027.hg.1	ENSG00000118503	0.2828076	0.4529859	FALSE
TC05000159.hg.1	ENSG00000168685	0.2828076	0.4529859	FALSE
TC07000768.hg.1	ENSG00000174697	0.2828076	0.4529859	FALSE
TC04000432.hg.1	ENSG00000156234	0.2828076	0.4529859	FALSE
TC04000432.hg.1	ENSG00000156234	0.2828076	0.4529859	FALSE
TC03000248.hg.1	ENSG00000144791	0.2828076	0.4529859	FALSE
TC0X000034.hg.1	ENSG00000146950	0.2828076	0.4529859	FALSE
TC02001628.hg.1	ENSG00000084674	0.2828076	0.4529859	FALSE
TC0X000207.hg.1	ENSG00000189221	0.2828076	0.4529859	FALSE
TC20000893.hg.1	ENSG00000100979	0.2828076	0.4529859	FALSE
TC01001805.hg.1	ENSG00000092969	0.2828076	0.4529859	FALSE
TC05000165.hg.1	ENSG00000079215	0.2828076	0.4529859	FALSE
TC0X000034.hg.1	ENSG00000146950	0.2828076	0.4529859	FALSE
TC02001628.hg.1	ENSG00000084674	0.2828076	0.4529859	FALSE
TC03001071.hg.1	ENSG00000114315	0.2828076	0.4529859	FALSE
TC02000286.hg.1	ENSG00000171150	0.2828076	0.4529859	FALSE
TC02000041.hg.1	ENSG00000115738	0.2828076	0.4529859	FALSE
TC06001027.hg.1	ENSG00000118503	0.2828076	0.4529859	FALSE
TC01001624.hg.1	ENSG00000116741	0.2828076	0.4529859	FALSE
TC06000779.hg.1	ENSG00000135318	0.2828076	0.4529859	FALSE
TC22001467.hg.1	ENSG00000128394	0.2828076	0.4529859	FALSE
TC09000388.hg.1	ENSG00000148053	0.2828076	0.4529859	FALSE
TC05001385.hg.1	ENSG00000145632	0.2828076	0.4529859	FALSE
TC03001527.hg.1	ENSG00000163638	0.2828076	0.4529859	FALSE
TC15000622.hg.1	ENSG00000166949	0.2828076	0.4529859	FALSE
TC0X000943.hg.1	ENSG00000198947	0.2828076	0.4529859	FALSE
TC02002714.hg.1	ENSG00000163251	0.2828076	0.4529859	FALSE
TC11002140.hg.1	ENSG00000149256	0.2828076	0.4529859	FALSE
TC06004141.hg.1	ENSG00000112096	0.2828076	0.4529859	FALSE
TC03001527.hg.1	ENSG00000163638	0.2828076	0.4529859	FALSE
TC21000420.hg.1	ENSG00000159200	0.2828076	0.4529859	FALSE
TC02000466.hg.1	ENSG00000159399	0.2828076	0.4529859	FALSE
TC17001462.hg.1	ENSG00000131747	0.2828076	0.4529859	FALSE
TC01001090.hg.1	ENSG00000117289	0.2828076	0.4529859	FALSE
TC03002146.hg.1	ENSG00000189058	0.2828076	0.4529859	FALSE
TC15000622.hg.1	ENSG00000166949	0.2828076	0.4529859	FALSE
TC15000622.hg.1	ENSG00000166949	0.2828076	0.4529859	FALSE
TC10001663.hg.1	ENSG00000119927	0.2828076	0.4529859	FALSE
TC11002366.hg.1	ENSG00000076706	0.2828076	0.4529859	FALSE
TC03000214.hg.1	ENSG00000168036	0.2828076	0.4529859	FALSE
TC14001122.hg.1	ENSG00000151748	0.2828076	0.4529859	FALSE
TC10001711.hg.1	ENSG00000066468	0.2828076	0.4529859	FALSE
TC15000971.hg.1	ENSG00000184254	0.2828076	0.4529859	FALSE
TC03000214.hg.1	ENSG00000168036	0.2828076	0.4529859	FALSE
TC10001663.hg.1	ENSG00000119927	0.2828076	0.4529859	FALSE
TC10000811.hg.1	ENSG00000150594	0.2828076	0.4529859	FALSE
TC06002168.hg.1	ENSG00000164442	0.2828076	0.4529859	FALSE
TC01001607.hg.1	ENSG00000116711	0.2828076	0.4529859	FALSE
TC11002178.hg.1	ENSG00000086991	0.2828076	0.4529859	FALSE
TC08001567.hg.1	ENSG00000170961	0.2828076	0.4529859	FALSE
TC07001627.hg.1	ENSG00000105880	0.2828076	0.4529859	FALSE

TC07001627.hg.1	ENSG00000105880	0.2828076	0.4529859	FALSE
TC07001552.hg.1	ENSG00000187391	0.2828076	0.4529859	FALSE
TC15000622.hg.1	ENSG00000166949	0.2828076	0.4529859	FALSE
TC02002823.hg.1	ENSG00000135919	0.2828076	0.4529859	FALSE
TC07000004.hg.1	ENSG00000177706	0.2828076	0.4529859	FALSE
TC01002706.hg.1	ENSG00000184292	0.2828076	0.4529859	FALSE
TC16000571.hg.1	ENSG00000198373	0.2828076	0.4529859	FALSE
TC03002146.hg.1	ENSG00000189058	0.2828076	0.4529859	FALSE
TC12002066.hg.1	ENSG00000158104	0.2828076	0.4529859	FALSE
TC12002066.hg.1	ENSG00000158104	0.2828076	0.4529859	FALSE
TC12000601.hg.1	ENSG00000127314	0.2828076	0.4529859	FALSE
TC04001084.hg.1	ENSG00000109819	0.2828076	0.4529859	FALSE
TC0X000943.hg.1	ENSG00000198947	0.2828076	0.4529859	FALSE
TC10002938.hg.1	ENSG00000148671	0.2828076	0.4529859	FALSE
TC01001607.hg.1	ENSG00000116711;I	0.2836849	0.4529859	FALSE
TC02002605.hg.1	ENSG00000064989;	0.2849347	0.4548119	FALSE
TC03000214.hg.1	ENSG00000168036;	0.2859591	0.4551149	FALSE
TC10000663.hg.1	ENSG00000095596;	0.2859591	0.4551149	FALSE
TC01000728.hg.1	ENSG00000162433;	0.2859591	0.4551149	FALSE
TC01002669.hg.1	ENSG00000157193;	0.2859591	0.4551149	FALSE
TC03001071.hg.1	ENSG00000114315;	0.2859591	0.4551149	FALSE
TC01003638.hg.1	ENSG00000073756;	0.2859591	0.4551149	FALSE
TC01003638.hg.1	ENSG00000073756;	0.2859591	0.4551149	FALSE
TC07000768.hg.1	ENSG00000174697;	0.2859591	0.4551149	FALSE
TC01003638.hg.1	ENSG00000073756;	0.2859591	0.4551149	FALSE
TC01001607.hg.1	ENSG00000116711;I	0.2859591	0.4551149	FALSE
TC07001374.hg.1	ENSG00000106070;	0.2859591	0.4551149	FALSE
TC04001084.hg.1	ENSG00000109819;	0.2859591	0.4551149	FALSE
TC02000762.hg.1	ENSG00000163083;	0.2865597	0.4551149	FALSE
TC01000904.hg.1	ENSG00000162692;	0.2865597	0.4551149	FALSE
TC07001883.hg.1	ENSG00000085662;	0.2878412	0.4567265	FALSE
TC03001362.hg.1	ENSG00000160808;	0.2878412	0.4567265	FALSE
TC02000270.hg.1	ENSG00000152527;	0.2878412	0.4567265	FALSE
TC01000100.hg.1	ENSG00000049246;	0.2889668	0.458173	FALSE
TC04001084.hg.1	ENSG00000109819;	0.290269	0.4598118	FALSE
TC02000041.hg.1	ENSG00000115738;	0.290269	0.4598118	FALSE
TC01001688.hg.1	ENSG00000058668;	0.290269	0.4598118	FALSE
TC02000041.hg.1	ENSG00000115738;	0.290269	0.4598118	FALSE
TC03001575.hg.1	ENSG00000114480;	0.2907541	0.4601547	FALSE
TC01000115.hg.1;	ENSG00000171621;	0.2938112	0.4648211	FALSE
TC03001255.hg.1	ENSG00000033867;	0.2959081	0.4679655	FALSE
TC01001792.hg.1	ENSG00000082482;	0.296348	0.4684881	FALSE
TC01002954.hg.1	ENSG00000134243;	0.2976644	0.4703955	FALSE
TC01000728.hg.1	ENSG00000162433;	0.2990658	0.4720003	FALSE
TC04000408.hg.1	ENSG00000169429;	0.2990658	0.4720003	FALSE
TC01000728.hg.1	ENSG00000162433;	0.2990658	0.4720003	FALSE
TC01001090.hg.1	ENSG00000117289;	0.2990658	0.4720003	FALSE
TC06001027.hg.1	ENSG00000118503;	0.2990658	0.4720003	FALSE
TC02000270.hg.1	ENSG00000152527;	0.2990658	0.4720003	FALSE
TC04000253.hg.1	ENSG00000154277;	0.2994838	0.4720509	FALSE
TC01000904.hg.1	ENSG00000162692;	0.3010857	0.4733776	FALSE
TC02001031.hg.1	ENSG00000152256;	0.3012101	0.4733776	FALSE
TC04000313.hg.1	ENSG00000157404;	0.3012101	0.4733776	FALSE
TC06000728.hg.1	ENSG00000185760;	0.3012101	0.4733776	FALSE
TC01000785.hg.1	ENSG00000154027;	0.3012101	0.4733776	FALSE
TC02002808.hg.1	ENSG00000116106;	0.3012101	0.4733776	FALSE
TC02002823.hg.1	ENSG00000135919;	0.3012101	0.4733776	FALSE
TC02002823.hg.1	ENSG00000135919;	0.3012101	0.4733776	FALSE

TC11002178.hg.1;	ENSG00000086991;	0.3012101	0.4733776	FALSE
TC07001374.hg.1	ENSG00000106070;	0.3012101	0.4733776	FALSE
TC10000811.hg.1;	ENSG00000150594;	0.3012101	0.4733776	FALSE
TC02000286.hg.1	ENSG00000171150;	0.3012101	0.4733776	FALSE
TC03001071.hg.1	ENSG00000114315;	0.3012101	0.4733776	FALSE
TC01002166.hg.1	ENSG00000116285;	0.3012101	0.4733776	FALSE
TC04000432.hg.1	ENSG00000156234;	0.3026541	0.4744278	FALSE
TC01000685.hg.1	ENSG00000162409;	0.3037578	0.4759836	FALSE
TC03000917.hg.1	ENSG00000136603;	0.3049975	0.4777513	FALSE
TC01001805.hg.1	ENSG00000092969;	0.3087325	0.4822002	FALSE
TC01003638.hg.1	ENSG00000073756;	0.3108608	0.4822002	FALSE
TC01001736.hg.1	ENSG00000196352;	0.3115961	0.4822002	FALSE
TC03000846.hg.1	ENSG00000196549;	0.3119239	0.4822002	FALSE
TC01003791.hg.1	ENSG00000143473	0.3120053	0.4822002	FALSE
TC05000165.hg.1	ENSG00000079215	0.3120053	0.4822002	FALSE
TC08000646.hg.1	ENSG00000164932	0.3120053	0.4822002	FALSE
TC03000214.hg.1	ENSG00000168036	0.3120053	0.4822002	FALSE
TC06000945.hg.1	ENSG00000152661	0.3120053	0.4822002	FALSE
TC04000313.hg.1	ENSG00000157404	0.3120053	0.4822002	FALSE
TC19001103.hg.1	ENSG00000125730	0.3120053	0.4822002	FALSE
TC19001293.hg.1	ENSG00000105664	0.3120053	0.4822002	FALSE
TC01001688.hg.1	ENSG00000058668	0.3120053	0.4822002	FALSE
TC12000621.hg.1	ENSG00000135643	0.3120053	0.4822002	FALSE
TC17001462.hg.1	ENSG00000131747	0.3120053	0.4822002	FALSE
TC03001527.hg.1	ENSG00000163638	0.3120053	0.4822002	FALSE
TC12002066.hg.1	ENSG00000158104	0.3120053	0.4822002	FALSE
TC08000646.hg.1	ENSG00000164932	0.3120053	0.4822002	FALSE
TC05000165.hg.1	ENSG00000079215	0.3120053	0.4822002	FALSE
TC02000041.hg.1	ENSG00000115738	0.3120053	0.4822002	FALSE
TC03002146.hg.1	ENSG00000189058	0.3120053	0.4822002	FALSE
TC17000383.hg.1	ENSG00000108691	0.3120053	0.4822002	FALSE
TC06000087.hg.1	ENSG00000078401	0.3120053	0.4822002	FALSE
TC0X000034.hg.1	ENSG00000146950	0.3120053	0.4822002	FALSE
TC05000165.hg.1	ENSG00000079215	0.3120053	0.4822002	FALSE
TC05000840.hg.1	ENSG00000123643	0.3120053	0.4822002	FALSE
TC17000383.hg.1	ENSG00000108691	0.3120053	0.4822002	FALSE
TC18000164.hg.1	ENSG00000141469	0.3120053	0.4822002	FALSE
TC12001890.hg.1	ENSG00000017427	0.3120053	0.4822002	FALSE
TC01002284.hg.1	ENSG00000117122	0.3120053	0.4822002	FALSE
TC07000137.hg.1	ENSG00000136244	0.3120053	0.4822002	FALSE
TC06001027.hg.1	ENSG00000118503	0.3120053	0.4822002	FALSE
TC05001933.hg.1	ENSG00000113721	0.3120053	0.4822002	FALSE
TC05001933.hg.1	ENSG00000113721	0.3120053	0.4822002	FALSE
TC01002284.hg.1	ENSG00000117122	0.3120053	0.4822002	FALSE
TC05002075.hg.1	ENSG00000113739	0.3120053	0.4822002	FALSE
TC05000159.hg.1	ENSG00000168685	0.3120053	0.4822002	FALSE
TC04000313.hg.1	ENSG00000157404	0.3120053	0.4822002	FALSE
TC01003638.hg.1	ENSG00000073756	0.3120053	0.4822002	FALSE
TC08001659.hg.1	ENSG00000104419	0.3120053	0.4822002	FALSE
TC10001571.hg.1	ENSG00000120057	0.3120053	0.4822002	FALSE
TC22001467.hg.1	ENSG00000128394	0.3120053	0.4822002	FALSE
TC0X000034.hg.1	ENSG00000146950	0.3120053	0.4822002	FALSE
TC08000203.hg.1	ENSG00000092964	0.3120053	0.4822002	FALSE
TC22001467.hg.1	ENSG00000128394	0.3120053	0.4822002	FALSE
TC07001332.hg.1	ENSG00000164708	0.3120053	0.4822002	FALSE
TC17000533.hg.1	ENSG00000108797	0.3120053	0.4822002	FALSE
TC10000811.hg.1	ENSG00000150594	0.3120053	0.4822002	FALSE
TC10001711.hg.1	ENSG00000066468	0.3120053	0.4822002	FALSE

TC03000214.hg.1	ENSG00000168036	0.3120053	0.4822002	FALSE
TC07001374.hg.1	ENSG00000106070	0.3120053	0.4822002	FALSE
TC05000159.hg.1	ENSG00000168685	0.3120053	0.4822002	FALSE
TC20000621.hg.1	ENSG00000101384	0.3120053	0.4822002	FALSE
TC02002624.hg.1	ENSG00000115415	0.3120053	0.4822002	FALSE
TC07001552.hg.1	ENSG00000187391	0.3120053	0.4822002	FALSE
TC17000383.hg.1	ENSG00000108691	0.3120053	0.4822002	FALSE
TC01003129.hg.1	ENSG00000143140	0.3120053	0.4822002	FALSE
TC01003638.hg.1	ENSG00000073756	0.3120053	0.4822002	FALSE
TC08001659.hg.1	ENSG00000104419	0.3120053	0.4822002	FALSE
TC04000313.hg.1	ENSG00000157404	0.3120053	0.4822002	FALSE
TC10001711.hg.1	ENSG00000066468	0.3120053	0.4822002	FALSE
TC01001607.hg.1	ENSG00000116711	0.3120053	0.4822002	FALSE
TC02001750.hg.1	ENSG00000138061	0.3120053	0.4822002	FALSE
TC07000768.hg.1	ENSG00000174697	0.3120053	0.4822002	FALSE
TC10000811.hg.1	ENSG00000150594	0.3120053	0.4822002	FALSE
TC14001122.hg.1	ENSG00000151748	0.3120053	0.4822002	FALSE
TC06001027.hg.1	ENSG00000118503	0.3120053	0.4822002	FALSE
TC02000286.hg.1	ENSG00000171150	0.3120053	0.4822002	FALSE
TC12001916.hg.1	ENSG00000074590	0.3120053	0.4822002	FALSE
TC02001100.hg.1;	ENSG00000138448;	0.3164041	0.482381	FALSE
TC03001466.hg.1	ENSG00000163931;	0.3164041	0.482381	FALSE
TC10001711.hg.1;	ENSG00000066468;	0.3164041	0.482381	FALSE
TC02002808.hg.1	ENSG00000116106;	0.3164041	0.482381	FALSE
TC09000015.hg.1	ENSG00000147852;	0.3164041	0.482381	FALSE
TC01002669.hg.1	ENSG00000157193;	0.3164041	0.482381	FALSE
TC02000270.hg.1	ENSG00000152527;	0.3164041	0.482381	FALSE
TC02002624.hg.1	ENSG00000115415;	0.3164041	0.482381	FALSE
TC16000668.hg.1	ENSG00000103241;	0.3164041	0.482381	FALSE
TC01003718.hg.1	ENSG00000133048;	0.3164041	0.482381	FALSE
TC04001084.hg.1	ENSG00000109819;	0.3164041	0.482381	FALSE
TC02002605.hg.1	ENSG00000064989;	0.3180088	0.4837078	FALSE
TC02002605.hg.1	ENSG00000064989;	0.3180088	0.4837078	FALSE
TC01001090.hg.1	ENSG00000117289;	0.3193616	0.4855067	FALSE
TC02001628.hg.1	ENSG00000084674;	0.3199759	0.4861816	FALSE
TC05000243.hg.1	ENSG00000095015;	0.3199759	0.4861816	FALSE
TC03000214.hg.1	ENSG00000168036;	0.3215725	0.4882609	FALSE
TC04000408.hg.1	ENSG00000169429;	0.3215725	0.4882609	FALSE
TC02000416.hg.1	ENSG00000116005;	0.3273067	0.4966153	FALSE
TC04001084.hg.1	ENSG00000109819;	0.3273067	0.4966153	FALSE
TC0X000034.hg.1	ENSG00000146950;	0.328052	0.4974816	FALSE
TC01001607.hg.1	ENSG00000116711;I	0.3286263	0.4981761	FALSE
TC01000904.hg.1	ENSG00000162692;	0.3295551	0.4994073	FALSE
TC03000917.hg.1	ENSG00000136603;	0.3305888	0.5007017	FALSE
TC03000214.hg.1	ENSG00000168036;	0.3315204	0.5007017	FALSE
TC03000214.hg.1	ENSG00000168036;	0.3315204	0.5007017	FALSE
TC06000779.hg.1	ENSG00000135318;	0.3315204	0.5007017	FALSE
TC01000873.hg.1	ENSG00000143036;	0.3315204	0.5007017	FALSE
TC01000685.hg.1	ENSG00000162409;	0.3315204	0.5007017	FALSE
TC02001031.hg.1	ENSG00000152256;	0.3315204	0.5007017	FALSE
TC05001385.hg.1	ENSG00000145632;	0.3315204	0.5007017	FALSE
TC01000904.hg.1	ENSG00000162692;	0.3315204	0.5007017	FALSE
TC01003638.hg.1	ENSG00000073756;	0.3315204	0.5007017	FALSE
TC02000286.hg.1	ENSG00000171150;	0.3315204	0.5007017	FALSE
TC03000013.hg.1	ENSG00000150995;	0.3315204	0.5007017	FALSE
TC03000013.hg.1	ENSG00000150995;	0.3315204	0.5007017	FALSE
TC04002931.hg.1	ENSG00000134853;	0.3315204	0.5007017	FALSE
TC06000945.hg.1	ENSG00000152661;	0.3315204	0.5007017	FALSE

TC07000328.hg.1	ENSG00000146648;	0.3315204	0.5007017	FALSE
TC07000328.hg.1	ENSG00000146648;	0.3315204	0.5007017	FALSE
TC04000253.hg.1	ENSG00000154277;	0.3328341	0.5009185	FALSE
TC03002146.hg.1	ENSG00000189058;	0.3328341	0.5009185	FALSE
TC03000214.hg.1	ENSG00000168036;	0.3328341	0.5009185	FALSE
TC06001027.hg.1	ENSG00000118503;	0.3328341	0.5009185	FALSE
TC01000785.hg.1	ENSG00000154027;	0.3366205	0.5054196	FALSE
TC01000785.hg.1	ENSG00000154027;	0.3366205	0.5054196	FALSE
TC02000041.hg.1	ENSG00000115738;	0.3366205	0.5054196	FALSE
TC18000097.hg.1	ENSG00000101773	0.340016	0.5054196	FALSE
TC07000137.hg.1	ENSG00000136244	0.340016	0.5054196	FALSE
TC16000668.hg.1	ENSG00000103241	0.340016	0.5054196	FALSE
TC17000241.hg.1	ENSG00000142494	0.340016	0.5054196	FALSE
TC10000053.hg.1	ENSG00000170525	0.340016	0.5054196	FALSE
TC09000347.hg.1	ENSG00000099139	0.340016	0.5054196	FALSE
TC01002195.hg.1	ENSG00000116649	0.340016	0.5054196	FALSE
TC03000214.hg.1	ENSG00000168036	0.340016	0.5054196	FALSE
TC02001031.hg.1	ENSG00000152256	0.340016	0.5054196	FALSE
TC01000785.hg.1	ENSG00000154027	0.340016	0.5054196	FALSE
TC01000785.hg.1	ENSG00000154027	0.340016	0.5054196	FALSE
TC01000785.hg.1	ENSG00000154027	0.340016	0.5054196	FALSE
TC01001805.hg.1	ENSG00000092969	0.340016	0.5054196	FALSE
TC0X000943.hg.1	ENSG00000198947	0.340016	0.5054196	FALSE
TC01001805.hg.1	ENSG00000092969	0.340016	0.5054196	FALSE
TC03000013.hg.1	ENSG00000150995	0.340016	0.5054196	FALSE
TC08000127.hg.1	ENSG00000003989	0.340016	0.5054196	FALSE
TC09000347.hg.1	ENSG00000099139	0.340016	0.5054196	FALSE
TC03001255.hg.1	ENSG00000033867	0.340016	0.5054196	FALSE
TC03000214.hg.1	ENSG00000168036	0.340016	0.5054196	FALSE
TC01002669.hg.1	ENSG00000157193	0.340016	0.5054196	FALSE
TC12001890.hg.1	ENSG00000017427	0.340016	0.5054196	FALSE
TC05001519.hg.1	ENSG00000113273	0.340016	0.5054196	FALSE
TC07000768.hg.1	ENSG00000174697	0.340016	0.5054196	FALSE
TC07000768.hg.1	ENSG00000174697	0.340016	0.5054196	FALSE
TC06001027.hg.1	ENSG00000118503	0.340016	0.5054196	FALSE
TC05000701.hg.1	ENSG00000120738	0.340016	0.5054196	FALSE
TC04002931.hg.1	ENSG00000134853	0.340016	0.5054196	FALSE
TC10000811.hg.1	ENSG00000150594	0.340016	0.5054196	FALSE
TC02001628.hg.1	ENSG00000084674	0.340016	0.5054196	FALSE
TC03002146.hg.1	ENSG00000189058	0.340016	0.5054196	FALSE
TC09001585.hg.1	ENSG00000173611	0.340016	0.5054196	FALSE
TC10001571.hg.1	ENSG00000120057	0.340016	0.5054196	FALSE
TC05003423.hg.1	ENSG00000082196	0.340016	0.5054196	FALSE
TC10000811.hg.1	ENSG00000150594	0.340016	0.5054196	FALSE
TC03001071.hg.1	ENSG00000114315	0.340016	0.5054196	FALSE
TC02000286.hg.1	ENSG00000171150	0.340016	0.5054196	FALSE
TC07001534.hg.1	ENSG00000127946	0.340016	0.5054196	FALSE
TC08000482.hg.1	ENSG00000121039	0.340016	0.5054196	FALSE
TC01003778.hg.1	ENSG00000076356	0.340016	0.5054196	FALSE
TC17000383.hg.1	ENSG00000108691	0.340016	0.5054196	FALSE
TC07000643.hg.1	ENSG00000106366	0.340016	0.5054196	FALSE
TC07001293.hg.1	ENSG00000106483	0.340016	0.5054196	FALSE
TC03001255.hg.1	ENSG00000033867	0.340016	0.5054196	FALSE
TC05001385.hg.1	ENSG00000145632	0.340016	0.5054196	FALSE
TC11000182.hg.1	ENSG00000148926	0.340016	0.5054196	FALSE
TC03000262.hg.1	ENSG00000160801	0.340016	0.5054196	FALSE
TC03000214.hg.1	ENSG00000168036	0.340016	0.5054196	FALSE
TC01001805.hg.1	ENSG00000092969	0.340016	0.5054196	FALSE

TC10001711.hg.1	ENSG00000066468	0.340016	0.5054196	FALSE
TC10001711.hg.1	ENSG00000066468	0.340016	0.5054196	FALSE
TC03000214.hg.1	ENSG00000168036	0.340016	0.5054196	FALSE
TC03000214.hg.1	ENSG00000168036	0.340016	0.5054196	FALSE
TC03001071.hg.1	ENSG00000114315	0.340016	0.5054196	FALSE
TC07001293.hg.1	ENSG00000106483	0.340016	0.5054196	FALSE
TC05000701.hg.1	ENSG00000120738	0.340016	0.5054196	FALSE
TC03000846.hg.1	ENSG00000196549	0.340016	0.5054196	FALSE
TC08000508.hg.1	ENSG00000104435	0.340016	0.5054196	FALSE
TC01000685.hg.1	ENSG00000162409	0.340016	0.5054196	FALSE
TC13000147.hg.1	ENSG00000102760	0.340016	0.5054196	FALSE
TC17000383.hg.1	ENSG00000108691	0.340016	0.5054196	FALSE
TC07000137.hg.1	ENSG00000136244;	0.3440882	0.506026	FALSE
TC02000041.hg.1	ENSG00000115738;	0.3442609	0.5061061	FALSE
TC08000646.hg.1	ENSG00000164932;	0.3459444	0.5078875	FALSE
TC03001884.hg.1	ENSG00000047457;	0.3459444	0.5078875	FALSE
TC01003629.hg.1	ENSG00000116679;	0.3465404	0.5078875	FALSE
TC02001628.hg.1	ENSG00000084674;	0.3465404	0.5078875	FALSE
TC07001374.hg.1	ENSG00000106070;	0.3465404	0.5078875	FALSE
TC02000041.hg.1	ENSG00000115738;	0.3465404	0.5078875	FALSE
TC02000041.hg.1	ENSG00000115738;	0.3465404	0.5078875	FALSE
TC06001027.hg.1	ENSG00000118503;	0.3465404	0.5078875	FALSE
TC03000013.hg.1	ENSG00000150995;	0.3465404	0.5078875	FALSE
TC01003638.hg.1	ENSG00000073756;	0.3465404	0.5078875	FALSE
TC10001711.hg.1;	ENSG00000066468;	0.3465404	0.5078875	FALSE
TC06004150.hg.1	ENSG00000096060;	0.3465404	0.5078875	FALSE
TC03001362.hg.1	ENSG00000160808;	0.3465404	0.5078875	FALSE
TC05001933.hg.1	ENSG00000113721;	0.3465404	0.5078875	FALSE
TC02000286.hg.1	ENSG00000171150;	0.3465404	0.5078875	FALSE
TC01003791.hg.1	ENSG00000143473;	0.348361	0.5093352	FALSE
TC01000972.hg.1	ENSG00000064886;	0.3496982	0.5111158	FALSE
TC01001792.hg.1	ENSG00000082482;	0.3514038	0.5133457	FALSE
TC01001792.hg.1	ENSG00000082482;	0.3514038	0.5133457	FALSE
TC01003791.hg.1	ENSG00000143473;	0.3552728	0.5181028	FALSE
TC06004141.hg.1	ENSG00000112096;	0.3552728	0.5181028	FALSE
TC03001071.hg.1	ENSG00000114315;	0.3553258	0.5181028	FALSE
TC05001519.hg.1	ENSG00000113273;	0.3553258	0.5181028	FALSE
TC06000595.hg.1	ENSG00000112655;	0.3553258	0.5181028	FALSE
TC04000253.hg.1	ENSG00000154277;	0.3553258	0.5181028	FALSE
TC03000917.hg.1	ENSG00000136603;	0.3553258	0.5181028	FALSE
TC01000904.hg.1	ENSG00000162692;	0.3563706	0.5190958	FALSE
TC01000100.hg.1	ENSG00000049246;	0.3571532	0.5200588	FALSE
TC01002706.hg.1	ENSG00000184292;	0.3576652	0.5206273	FALSE
TC01001473.hg.1	ENSG00000143153;	0.3595512	0.5219753	FALSE
TC06000087.hg.1	ENSG00000078401;	0.3605202	0.5219753	FALSE
TC06000087.hg.1	ENSG00000078401;	0.3605202	0.5219753	FALSE
TC06000087.hg.1	ENSG00000078401;	0.3614467	0.5219753	FALSE
TC02002624.hg.1	ENSG00000115415;	0.3614467	0.5219753	FALSE
TC0Y000351.hg.1	ENSG00000129824;	0.3614467	0.5219753	FALSE
TC04000313.hg.1	ENSG00000157404;	0.3614467	0.5219753	FALSE
TC05001110.hg.1;	ENSG00000153395;	0.3614467	0.5219753	FALSE
TC03001362.hg.1	ENSG00000160808;	0.3614467	0.5219753	FALSE
TC11000707.hg.1;	ENSG00000162337;	0.3614467	0.5219753	FALSE
TC01001624.hg.1	ENSG00000116741;	0.3621145	0.5219753	FALSE
TC01002166.hg.1	ENSG00000116285;	0.3626312	0.5219753	FALSE
TC01003605.hg.1	ENSG00000135821;	0.3646004	0.5219753	FALSE
TC02000082.hg.1	ENSG00000071575;	0.3665379	0.5219753	FALSE
TC02000416.hg.1	ENSG00000116005	0.3668879	0.5219753	FALSE

TC20000945.hg.1	ENSG00000101115	0.3668879	0.5219753	FALSE
TC18000548.hg.1	ENSG00000183287	0.3668879	0.5219753	FALSE
TC11000182.hg.1	ENSG00000148926	0.3668879	0.5219753	FALSE
TC08000127.hg.1	ENSG00000003989	0.3668879	0.5219753	FALSE
TC02001031.hg.1	ENSG00000152256	0.3668879	0.5219753	FALSE
TC14001364.hg.1	ENSG00000211448	0.3668879	0.5219753	FALSE
TC01002954.hg.1	ENSG00000134243	0.3668879	0.5219753	FALSE
TC07000852.hg.1	ENSG00000181072	0.3668879	0.5219753	FALSE
TC02002714.hg.1	ENSG00000163251	0.3668879	0.5219753	FALSE
TC02002714.hg.1	ENSG00000163251	0.3668879	0.5219753	FALSE
TC01001473.hg.1	ENSG00000143153	0.3668879	0.5219753	FALSE
TC02002714.hg.1	ENSG00000163251	0.3668879	0.5219753	FALSE
TC03000846.hg.1	ENSG00000196549	0.3668879	0.5219753	FALSE
TC0X000943.hg.1	ENSG00000198947	0.3668879	0.5219753	FALSE
TC08000149.hg.1	ENSG00000175445	0.3668879	0.5219753	FALSE
TC09000388.hg.1	ENSG00000148053	0.3668879	0.5219753	FALSE
TC05001172.hg.1	ENSG00000154122	0.3668879	0.5219753	FALSE
TC03001590.hg.1	ENSG00000184500	0.3668879	0.5219753	FALSE
TC03001590.hg.1	ENSG00000184500	0.3668879	0.5219753	FALSE
TC12003293.hg.1	ENSG00000180818	0.3668879	0.5219753	FALSE
TC15000971.hg.1	ENSG00000184254	0.3668879	0.5219753	FALSE
TC10001711.hg.1	ENSG00000066468	0.3668879	0.5219753	FALSE
TC11002140.hg.1	ENSG00000149256	0.3668879	0.5219753	FALSE
TC11001415.hg.1	ENSG00000133800	0.3668879	0.5219753	FALSE
TC08001175.hg.1	ENSG00000104368	0.3668879	0.5219753	FALSE
TC11002140.hg.1	ENSG00000149256	0.3668879	0.5219753	FALSE
TC12001324.hg.1	ENSG00000123095	0.3668879	0.5219753	FALSE
TC02000041.hg.1	ENSG00000115738	0.3668879	0.5219753	FALSE
TC09000222.hg.1	ENSG00000137124	0.3668879	0.5219753	FALSE
TC12002155.hg.1	ENSG00000176915	0.3668879	0.5219753	FALSE
TC01002616.hg.1	ENSG00000117461	0.3668879	0.5219753	FALSE
TC18000548.hg.1	ENSG00000183287	0.3668879	0.5219753	FALSE
TC09001335.hg.1	ENSG00000127083	0.3668879	0.5219753	FALSE
TC07000137.hg.1	ENSG00000136244	0.3668879	0.5219753	FALSE
TC02001628.hg.1	ENSG00000084674	0.3668879	0.5219753	FALSE
TC05000165.hg.1	ENSG00000079215	0.3668879	0.5219753	FALSE
TC12001890.hg.1	ENSG00000017427	0.3668879	0.5219753	FALSE
TC10000406.hg.1	ENSG00000138336	0.3668879	0.5219753	FALSE
TC10000350.hg.1	ENSG00000107984	0.3668879	0.5219753	FALSE
TC04001084.hg.1	ENSG00000109819	0.3668879	0.5219753	FALSE
TC09000388.hg.1	ENSG00000148053	0.3668879	0.5219753	FALSE
TC09000388.hg.1	ENSG00000148053	0.3668879	0.5219753	FALSE
TC17001462.hg.1	ENSG00000131747	0.3668879	0.5219753	FALSE
TC15000226.hg.1	ENSG00000166923	0.3668879	0.5219753	FALSE
TC03001071.hg.1	ENSG00000114315	0.3668879	0.5219753	FALSE
TC21000345.hg.1	ENSG00000154734	0.3668879	0.5219753	FALSE
TC01003129.hg.1	ENSG00000143140	0.3668879	0.5219753	FALSE
TC08001567.hg.1	ENSG00000170961	0.3668879	0.5219753	FALSE
TC01003638.hg.1	ENSG00000073756	0.3668879	0.5219753	FALSE
TC14001188.hg.1	ENSG00000126778	0.3668879	0.5219753	FALSE
TC01001624.hg.1	ENSG00000116741	0.3668879	0.5219753	FALSE
TC04000313.hg.1	ENSG00000157404	0.3668879	0.5219753	FALSE
TC10000350.hg.1	ENSG00000107984	0.3668879	0.5219753	FALSE
TC08001659.hg.1	ENSG00000104419	0.3668879	0.5219753	FALSE
TC01001624.hg.1	ENSG00000116741;	0.3671263	0.5219753	FALSE
TC01002166.hg.1	ENSG00000116285;	0.3695662	0.5252699	FALSE
TC01001624.hg.1	ENSG00000116741;	0.3718705	0.5283697	FALSE
TC03000986.hg.1	ENSG00000163872;	0.3739218	0.5311079	FALSE

TC01001624.hg.1	ENSG00000116741;	0.3741503	0.5312562	FALSE
TC01002166.hg.1	ENSG00000116285;	0.3761929	0.5334927	FALSE
TC03001362.hg.1	ENSG00000160808;	0.3762239	0.5334927	FALSE
TC06000087.hg.1	ENSG00000078401;	0.3762239	0.5334927	FALSE
TC02002714.hg.1	ENSG00000163251;	0.3762239	0.5334927	FALSE
TC03000214.hg.1	ENSG00000168036;	0.3762239	0.5334927	FALSE
TC0Y000351.hg.1	ENSG00000129824;	0.3762239	0.5334927	FALSE
TC06004055.hg.1	ENSG00000047936;	0.3777162	0.5349001	FALSE
TC03001466.hg.1	ENSG00000163931;	0.3777162	0.5349001	FALSE
TC06001772.hg.1	ENSG00000146233;	0.3777162	0.5349001	FALSE
TC01000115.hg.1;	ENSG00000171621;	0.3824195	0.5412026	FALSE
TC01002706.hg.1	ENSG00000184292;	0.3839145	0.543049	FALSE
TC01001188.hg.1;	ENSG00000143369;	0.3839145	0.543049	FALSE
TC01001624.hg.1	ENSG00000116741;	0.3841881	0.5431669	FALSE
TC02000041.hg.1	ENSG00000115738;	0.3848911	0.5439812	FALSE
TC04002931.hg.1	ENSG00000134853;	0.3888527	0.5461141	FALSE
TC06001027.hg.1	ENSG00000118503;	0.3888527	0.5461141	FALSE
TC01001624.hg.1	ENSG00000116741;	0.3890693	0.5461141	FALSE
TC01000972.hg.1	ENSG00000064886;	0.3903905	0.5461141	FALSE
TC0X000034.hg.1	ENSG00000146950;	0.3908576	0.5461141	FALSE
TC01000728.hg.1	ENSG00000162433;	0.3908576	0.5461141	FALSE
TC03000214.hg.1	ENSG00000168036;	0.3908576	0.5461141	FALSE
TC02000041.hg.1	ENSG00000115738;	0.3925258	0.5461141	FALSE
TC03000131.hg.1	ENSG00000077092;	0.3925258	0.5461141	FALSE
TC05000159.hg.1	ENSG00000168685	0.3926673	0.5461141	FALSE
TC09000560.hg.1	ENSG00000148154	0.3926673	0.5461141	FALSE
TC06000945.hg.1	ENSG00000152661	0.3926673	0.5461141	FALSE
TC0X001186.hg.1	ENSG00000072133	0.3926673	0.5461141	FALSE
TC03000214.hg.1	ENSG00000168036	0.3926673	0.5461141	FALSE
TC15000386.hg.1	ENSG00000140285	0.3926673	0.5461141	FALSE
TC0X000943.hg.1	ENSG00000198947	0.3926673	0.5461141	FALSE
TC03000262.hg.1	ENSG00000160801	0.3926673	0.5461141	FALSE
TC10000811.hg.1	ENSG00000150594	0.3926673	0.5461141	FALSE
TC05000165.hg.1	ENSG00000079215	0.3926673	0.5461141	FALSE
TC17001902.hg.1	ENSG00000161544	0.3926673	0.5461141	FALSE
TC01000685.hg.1	ENSG00000162409	0.3926673	0.5461141	FALSE
TC06000945.hg.1	ENSG00000152661	0.3926673	0.5461141	FALSE
TC01000685.hg.1	ENSG00000162409	0.3926673	0.5461141	FALSE
TC02001750.hg.1	ENSG00000138061	0.3926673	0.5461141	FALSE
TC01003638.hg.1	ENSG00000073756	0.3926673	0.5461141	FALSE
TC14000306.hg.1	ENSG00000125384	0.3926673	0.5461141	FALSE
TC12002155.hg.1	ENSG00000176915	0.3926673	0.5461141	FALSE
TC06000087.hg.1	ENSG00000078401	0.3926673	0.5461141	FALSE
TC10001571.hg.1	ENSG00000120057	0.3926673	0.5461141	FALSE
TC02000416.hg.1	ENSG00000116005	0.3926673	0.5461141	FALSE
TC04000313.hg.1	ENSG00000157404	0.3926673	0.5461141	FALSE
TC10000350.hg.1	ENSG00000107984	0.3926673	0.5461141	FALSE
TC03000214.hg.1	ENSG00000168036	0.3926673	0.5461141	FALSE
TC05000165.hg.1	ENSG00000079215	0.3926673	0.5461141	FALSE
TC12001405.hg.1	ENSG00000139174	0.3926673	0.5461141	FALSE
TC12001890.hg.1	ENSG00000017427	0.3926673	0.5461141	FALSE
TC10000811.hg.1	ENSG00000150594	0.3926673	0.5461141	FALSE
TC14000067.hg.1	ENSG00000181784	0.3926673	0.5461141	FALSE
TC11000238.hg.1	ENSG00000173432	0.3926673	0.5461141	FALSE
TC03000846.hg.1	ENSG00000196549	0.3926673	0.5461141	FALSE
TC08001022.hg.1	ENSG00000147408	0.3926673	0.5461141	FALSE
TC08001022.hg.1	ENSG00000147408	0.3926673	0.5461141	FALSE
TC04000408.hg.1	ENSG00000169429	0.3926673	0.5461141	FALSE

TC22000259.hg.1	ENSG00000100292	0.3926673	0.5461141	FALSE
TC11000198.hg.1	ENSG00000133816	0.3926673	0.5461141	FALSE
TC15000622.hg.1	ENSG00000166949	0.3926673	0.5461141	FALSE
TC17000158.hg.1	ENSG00000141052	0.3926673	0.5461141	FALSE
TC08000646.hg.1	ENSG00000164932	0.3926673	0.5461141	FALSE
TC12001890.hg.1	ENSG00000017427	0.3926673	0.5461141	FALSE
TC03000214.hg.1	ENSG00000168036	0.3926673	0.5461141	FALSE
TC07001552.hg.1	ENSG00000187391	0.3926673	0.5461141	FALSE
TC05000159.hg.1	ENSG00000168685	0.3926673	0.5461141	FALSE
TC08001062.hg.1	ENSG00000159167	0.3926673	0.5461141	FALSE
TC07001552.hg.1	ENSG00000187391	0.3926673	0.5461141	FALSE
TC14001188.hg.1	ENSG00000126778	0.3926673	0.5461141	FALSE
TC03000214.hg.1	ENSG00000168036	0.3926673	0.5461141	FALSE
TC01003129.hg.1	ENSG00000143140	0.3926673	0.5461141	FALSE
TC10000811.hg.1	ENSG00000150594	0.3926673	0.5461141	FALSE
TC22000620.hg.1	ENSG00000169184	0.3926673	0.5461141	FALSE
TC01000904.hg.1	ENSG00000162692;	0.3940543	0.5461141	FALSE
TC01000685.hg.1	ENSG00000162409;	0.3981497	0.5516113	FALSE
TC02002808.hg.1	ENSG00000116106;	0.3995224	0.5533341	FALSE
TC02002605.hg.1	ENSG00000064989;	0.3999396	0.5534642	FALSE
TC04000408.hg.1	ENSG00000169429;	0.3999396	0.5534642	FALSE
TC05001110.hg.1;	ENSG00000153395;	0.3999396	0.5534642	FALSE
TC03000214.hg.1	ENSG00000168036;	0.3999396	0.5534642	FALSE
TC05003423.hg.1	ENSG00000082196;	0.4017986	0.5555877	FALSE
TC01003638.hg.1	ENSG00000073756;	0.4040217	0.558323	FALSE
TC01000873.hg.1	ENSG00000143036;	0.4040375	0.558323	FALSE
TC01000685.hg.1	ENSG00000162409;	0.4053352	0.5596646	FALSE
TC03002146.hg.1	ENSG00000189058;	0.4053352	0.5596646	FALSE
TC03000917.hg.1	ENSG00000136603;	0.4053352	0.5596646	FALSE
TC07000768.hg.1	ENSG00000174697;	0.4053352	0.5596646	FALSE
TC01001607.hg.1	ENSG00000116711;I	0.4097176	0.5644098	FALSE
TC01003605.hg.1	ENSG00000135821;	0.4109699	0.5644098	FALSE
TC04001084.hg.1	ENSG00000109819;	0.4109699	0.5644098	FALSE
TC01001792.hg.1	ENSG00000082482;	0.4109699	0.5644098	FALSE
TC01001792.hg.1	ENSG00000082482;	0.4109699	0.5644098	FALSE
TC02001031.hg.1	ENSG00000152256;	0.4110456	0.5644098	FALSE
TC04001084.hg.1	ENSG00000109819;	0.4110456	0.5644098	FALSE
TC01000728.hg.1	ENSG00000162433;	0.4135085	0.5644098	FALSE
TC04002931.hg.1	ENSG00000134853;	0.4141344	0.5644098	FALSE
TC10001769.hg.1	ENSG00000176171	0.4173985	0.5644098	FALSE
TC02001031.hg.1	ENSG00000152256	0.4173985	0.5644098	FALSE
TC08001567.hg.1	ENSG00000170961	0.4173985	0.5644098	FALSE
TC01003129.hg.1	ENSG00000143140	0.4173985	0.5644098	FALSE
TC02001031.hg.1	ENSG00000152256	0.4173985	0.5644098	FALSE
TC03000013.hg.1	ENSG00000150995	0.4173985	0.5644098	FALSE
TC14001124.hg.1	ENSG00000100504	0.4173985	0.5644098	FALSE
TC16001281.hg.1	ENSG00000183196	0.4173985	0.5644098	FALSE
TC11002175.hg.1	ENSG00000109861	0.4173985	0.5644098	FALSE
TC03000631.hg.1	ENSG00000114023	0.4173985	0.5644098	FALSE
TC02002823.hg.1	ENSG00000135919	0.4173985	0.5644098	FALSE
TC12000076.hg.1	ENSG00000010278	0.4173985	0.5644098	FALSE
TC02002808.hg.1	ENSG00000116106	0.4173985	0.5644098	FALSE
TC06000945.hg.1	ENSG00000152661	0.4173985	0.5644098	FALSE
TC10001711.hg.1	ENSG00000066468	0.4173985	0.5644098	FALSE
TC19001103.hg.1	ENSG00000125730	0.4173985	0.5644098	FALSE
TC14000951.hg.1	ENSG00000092068	0.4173985	0.5644098	FALSE
TC18000552.hg.1	ENSG00000197563	0.4173985	0.5644098	FALSE
TC0Y000351.hg.1	ENSG00000129824	0.4173985	0.5644098	FALSE

TC03001071.hg.1	ENSG00000114315	0.4173985	0.5644098	FALSE
TC04002931.hg.1	ENSG00000134853	0.4173985	0.5644098	FALSE
TC0X002327.hg.1	ENSG00000087842	0.4173985	0.5644098	FALSE
TC01003638.hg.1	ENSG00000073756	0.4173985	0.5644098	FALSE
TC01003638.hg.1	ENSG00000073756	0.4173985	0.5644098	FALSE
TC06001027.hg.1	ENSG00000118503	0.4173985	0.5644098	FALSE
TC10000811.hg.1	ENSG00000150594	0.4173985	0.5644098	FALSE
TC0X000034.hg.1	ENSG00000146950	0.4173985	0.5644098	FALSE
TC03000013.hg.1	ENSG00000150995	0.4173985	0.5644098	FALSE
TC02002624.hg.1	ENSG00000115415	0.4173985	0.5644098	FALSE
TC01001607.hg.1	ENSG00000116711	0.4173985	0.5644098	FALSE
TC0X001186.hg.1	ENSG00000072133	0.4173985	0.5644098	FALSE
TC20000833.hg.1	ENSG00000198959	0.4173985	0.5644098	FALSE
TC0X000034.hg.1	ENSG00000146950	0.4173985	0.5644098	FALSE
TC02000041.hg.1	ENSG00000115738	0.4173985	0.5644098	FALSE
TC01003638.hg.1	ENSG00000073756	0.4173985	0.5644098	FALSE
TC01001736.hg.1	ENSG00000196352	0.4173985	0.5644098	FALSE
TC01000728.hg.1	ENSG00000162433	0.4173985	0.5644098	FALSE
TC01003638.hg.1	ENSG00000073756	0.4173985	0.5644098	FALSE
TC03000631.hg.1	ENSG00000114023	0.4173985	0.5644098	FALSE
TC06002036.hg.1	ENSG00000249853	0.4173985	0.5644098	FALSE
TC10001711.hg.1	ENSG00000066468	0.4173985	0.5644098	FALSE
TC03000917.hg.1	ENSG00000136603	0.4173985	0.5644098	FALSE
TC04002931.hg.1	ENSG00000134853	0.4173985	0.5644098	FALSE
TC02000041.hg.1	ENSG00000115738	0.4173985	0.5644098	FALSE
TC08001062.hg.1	ENSG00000159167	0.4173985	0.5644098	FALSE
TC07001552.hg.1	ENSG00000187391	0.4173985	0.5644098	FALSE
TC03001071.hg.1	ENSG00000114315	0.4173985	0.5644098	FALSE
TC03001071.hg.1	ENSG00000114315	0.4173985	0.5644098	FALSE
TC07001552.hg.1	ENSG00000187391	0.4173985	0.5644098	FALSE
TC02001031.hg.1	ENSG00000152256	0.4173985	0.5644098	FALSE
TC19000191.hg.1	ENSG00000130164	0.4173985	0.5644098	FALSE
TC14001443.hg.1	ENSG00000140092	0.4173985	0.5644098	FALSE
TC10000438.hg.1	ENSG00000107731	0.4173985	0.5644098	FALSE
TC10000438.hg.1	ENSG00000107731	0.4173985	0.5644098	FALSE
TC03000986.hg.1	ENSG00000163872;	0.4174075	0.5644098	FALSE
TC06000779.hg.1	ENSG00000135318;	0.4196451	0.5668089	FALSE
TC16000193.hg.1	ENSG00000103222;	0.4196451	0.5668089	FALSE
TC03001071.hg.1	ENSG00000114315;	0.4196451	0.5668089	FALSE
TC01003638.hg.1	ENSG00000073756;	0.4196451	0.5668089	FALSE
TC17000220.hg.1	ENSG00000108448;	0.4196451	0.5668089	FALSE
TC10000438.hg.1	ENSG00000107731;	0.4196451	0.5668089	FALSE
TC06000945.hg.1	ENSG00000152661;	0.4202625	0.5670166	FALSE
TC01001736.hg.1	ENSG00000196352;	0.4219365	0.5690062	FALSE
TC01003638.hg.1	ENSG00000073756;	0.4219365	0.5690062	FALSE
TC02000041.hg.1	ENSG00000115738;	0.4247745	0.5725629	FALSE
TC01001473.hg.1	ENSG00000143153;	0.429034	0.5781222	FALSE
TC03000214.hg.1	ENSG00000168036;	0.4294448	0.5784937	FALSE
TC01001736.hg.1	ENSG00000196352;	0.4307872	0.5801195	FALSE
TC01001736.hg.1	ENSG00000196352;	0.4323436	0.5820323	FALSE
TC02002808.hg.1	ENSG00000116106;	0.4328331	0.5823251	FALSE
TC06000779.hg.1	ENSG00000135318;	0.4328331	0.5823251	FALSE
TC02002605.hg.1	ENSG00000064989;	0.4328331	0.5823251	FALSE
TC01002954.hg.1	ENSG00000134243;	0.433777	0.5826795	FALSE
TC01002669.hg.1	ENSG00000157193;	0.433777	0.5826795	FALSE
TC01000394.hg.1	ENSG00000159023;	0.433777	0.5826795	FALSE
TC08001275.hg.1	ENSG00000172817;	0.433777	0.5826795	FALSE
TC03000013.hg.1	ENSG00000150995;	0.433777	0.5826795	FALSE

TC03000214.hg.1	ENSG00000168036;	0.433777	0.5826795	FALSE
TC07000328.hg.1	ENSG00000146648;	0.433777	0.5826795	FALSE
TC01001090.hg.1	ENSG00000117289;	0.4359742	0.5848968	FALSE
TC01001736.hg.1	ENSG00000196352;	0.4374583	0.5867039	FALSE
TC07000137.hg.1	ENSG00000136244	0.441124	0.5878305	FALSE
TC09000222.hg.1	ENSG00000137124	0.441124	0.5878305	FALSE
TC18000565.hg.1	ENSG00000166479	0.441124	0.5878305	FALSE
TC03003381.hg.1	ENSG00000225697	0.441124	0.5878305	FALSE
TC09000358.hg.1	ENSG00000135069	0.441124	0.5878305	FALSE
TC14001124.hg.1	ENSG00000100504	0.441124	0.5878305	FALSE
TC01001805.hg.1	ENSG00000092969	0.441124	0.5878305	FALSE
TC01003718.hg.1	ENSG00000133048	0.441124	0.5878305	FALSE
TC07000643.hg.1	ENSG00000106366	0.441124	0.5878305	FALSE
TC05000840.hg.1	ENSG00000123643	0.441124	0.5878305	FALSE
TC01000685.hg.1	ENSG00000162409	0.441124	0.5878305	FALSE
TC01000685.hg.1	ENSG00000162409	0.441124	0.5878305	FALSE
TC15000971.hg.1	ENSG00000184254	0.441124	0.5878305	FALSE
TC01000394.hg.1	ENSG00000159023	0.441124	0.5878305	FALSE
TC08000508.hg.1	ENSG00000104435	0.441124	0.5878305	FALSE
TC01003638.hg.1	ENSG00000073756	0.441124	0.5878305	FALSE
TC07000768.hg.1	ENSG00000174697	0.441124	0.5878305	FALSE
TC0X000034.hg.1	ENSG00000146950	0.441124	0.5878305	FALSE
TC03001362.hg.1	ENSG00000160808	0.441124	0.5878305	FALSE
TC08001175.hg.1	ENSG00000104368	0.441124	0.5878305	FALSE
TC12002155.hg.1	ENSG00000176915	0.441124	0.5878305	FALSE
TC09000347.hg.1	ENSG00000099139	0.441124	0.5878305	FALSE
TC0X000207.hg.1	ENSG00000189221	0.441124	0.5878305	FALSE
TC14001364.hg.1	ENSG00000211448	0.441124	0.5878305	FALSE
TC14001124.hg.1	ENSG00000100504	0.441124	0.5878305	FALSE
TC06001027.hg.1	ENSG00000118503	0.441124	0.5878305	FALSE
TC0X000034.hg.1	ENSG00000146950	0.441124	0.5878305	FALSE
TC01001805.hg.1	ENSG00000092969	0.441124	0.5878305	FALSE
TC07000768.hg.1	ENSG00000174697	0.441124	0.5878305	FALSE
TC01001805.hg.1	ENSG00000092969	0.441124	0.5878305	FALSE
TC06002168.hg.1	ENSG00000164442	0.441124	0.5878305	FALSE
TC0Y000185.hg.1	ENSG00000012817	0.441124	0.5878305	FALSE
TC10001663.hg.1	ENSG00000119927	0.441124	0.5878305	FALSE
TC03001071.hg.1	ENSG00000114315	0.441124	0.5878305	FALSE
TC07000643.hg.1	ENSG00000106366	0.441124	0.5878305	FALSE
TC02001628.hg.1	ENSG00000084674	0.441124	0.5878305	FALSE
TC02000041.hg.1	ENSG00000115738	0.441124	0.5878305	FALSE
TC07000449.hg.1	ENSG00000049540	0.441124	0.5878305	FALSE
TC02000270.hg.1	ENSG00000152527;	0.4436535	0.5878305	FALSE
TC01001907.hg.1	ENSG00000143641;	0.4477217	0.5923958	FALSE
TC05000054.hg.1	ENSG00000078295;	0.4477217	0.5923958	FALSE
TC03000214.hg.1	ENSG00000168036;	0.4477217	0.5923958	FALSE
TC20000876.hg.1	ENSG00000124107;	0.4477217	0.5923958	FALSE
TC04000313.hg.1	ENSG00000157404;	0.4477217	0.5923958	FALSE
TC05000165.hg.1	ENSG00000079215;	0.4477217	0.5923958	FALSE
TC03000013.hg.1	ENSG00000150995;	0.4477217	0.5923958	FALSE
TC01003638.hg.1	ENSG00000073756;	0.4477217	0.5923958	FALSE
TC01000785.hg.1	ENSG00000154027;	0.4495834	0.5940328	FALSE
TC04000253.hg.1	ENSG00000154277;	0.4543918	0.6001084	FALSE
TC02000041.hg.1	ENSG00000115738;	0.4543918	0.6001084	FALSE
TC01000783.hg.1	ENSG00000117069;	0.45557	0.6012935	FALSE
TC01000783.hg.1	ENSG00000117069;	0.45557	0.6012935	FALSE
TC01000904.hg.1	ENSG00000162692;	0.4570526	0.6029714	FALSE
TC01000728.hg.1	ENSG00000162433;	0.4572812	0.6030506	FALSE

TC01001688.hg.1	ENSG00000058668;	0.4573944	0.6030506	FALSE
TC01003605.hg.1	ENSG00000135821;	0.4575592	0.6030822	FALSE
TC04000699.hg.1	ENSG00000109458;	0.4614709	0.6042028	FALSE
TC04001084.hg.1	ENSG00000109819;	0.4614709	0.6042028	FALSE
TC01001624.hg.1	ENSG00000116741;	0.4614709	0.6042028	FALSE
TC03000214.hg.1	ENSG00000168036	0.4638847	0.6042028	FALSE
TC20000945.hg.1	ENSG00000101115	0.4638847	0.6042028	FALSE
TC06004141.hg.1	ENSG00000112096	0.4638847	0.6042028	FALSE
TC11000182.hg.1	ENSG00000148926	0.4638847	0.6042028	FALSE
TC03000214.hg.1	ENSG00000168036	0.4638847	0.6042028	FALSE
TC22001467.hg.1	ENSG00000128394	0.4638847	0.6042028	FALSE
TC05000159.hg.1	ENSG00000168685	0.4638847	0.6042028	FALSE
TC03001884.hg.1	ENSG00000047457	0.4638847	0.6042028	FALSE
TC08000508.hg.1	ENSG00000104435	0.4638847	0.6042028	FALSE
TC02001031.hg.1	ENSG00000152256	0.4638847	0.6042028	FALSE
TC0X000034.hg.1	ENSG00000146950	0.4638847	0.6042028	FALSE
TC01001805.hg.1	ENSG00000092969	0.4638847	0.6042028	FALSE
TC05000165.hg.1	ENSG00000079215	0.4638847	0.6042028	FALSE
TC01000785.hg.1	ENSG00000154027	0.4638847	0.6042028	FALSE
TC03001466.hg.1	ENSG00000163931	0.4638847	0.6042028	FALSE
TC11000182.hg.1	ENSG00000148926	0.4638847	0.6042028	FALSE
TC07000004.hg.1	ENSG00000177706	0.4638847	0.6042028	FALSE
TC03002146.hg.1	ENSG00000189058	0.4638847	0.6042028	FALSE
TC08000508.hg.1	ENSG00000104435	0.4638847	0.6042028	FALSE
TC08001275.hg.1	ENSG00000172817	0.4638847	0.6042028	FALSE
TC06000087.hg.1	ENSG00000078401	0.4638847	0.6042028	FALSE
TC04001084.hg.1	ENSG00000109819	0.4638847	0.6042028	FALSE
TC04000408.hg.1	ENSG00000169429	0.4638847	0.6042028	FALSE
TC01001805.hg.1	ENSG00000092969	0.4638847	0.6042028	FALSE
TC10001517.hg.1	ENSG00000148677	0.4638847	0.6042028	FALSE
TC02000041.hg.1	ENSG00000115738	0.4638847	0.6042028	FALSE
TC14000353.hg.1	ENSG00000165617	0.4638847	0.6042028	FALSE
TC01000728.hg.1	ENSG00000162433	0.4638847	0.6042028	FALSE
TC02001031.hg.1	ENSG00000152256	0.4638847	0.6042028	FALSE
TC14001443.hg.1	ENSG00000140092	0.4638847	0.6042028	FALSE
TC15000622.hg.1	ENSG00000166949	0.4638847	0.6042028	FALSE
TC03000131.hg.1	ENSG00000077092	0.4638847	0.6042028	FALSE
TC0X000943.hg.1	ENSG00000198947	0.4638847	0.6042028	FALSE
TC0X000034.hg.1	ENSG00000146950	0.4638847	0.6042028	FALSE
TC06000087.hg.1	ENSG00000078401	0.4638847	0.6042028	FALSE
TC06000087.hg.1	ENSG00000078401	0.4638847	0.6042028	FALSE
TC06000945.hg.1	ENSG00000152661	0.4638847	0.6042028	FALSE
TC12001890.hg.1	ENSG00000017427	0.4638847	0.6042028	FALSE
TC03001071.hg.1	ENSG00000114315	0.4638847	0.6042028	FALSE
TC04001084.hg.1	ENSG00000109819	0.4638847	0.6042028	FALSE
TC03000214.hg.1	ENSG00000168036	0.4638847	0.6042028	FALSE
TC04002931.hg.1	ENSG00000134853	0.4638847	0.6042028	FALSE
TC04001084.hg.1	ENSG00000109819	0.4638847	0.6042028	FALSE
TC02002605.hg.1	ENSG00000064989;	0.4650428	0.6042028	FALSE
TC06000595.hg.1	ENSG00000112655;	0.465747	0.6047506	FALSE
TC01000785.hg.1	ENSG00000154027;	0.465747	0.6047506	FALSE
TC01000394.hg.1	ENSG00000159023;	0.465747	0.6047506	FALSE
TC01000904.hg.1	ENSG00000162692;	0.473611	0.6145888	FALSE
TC01001090.hg.1	ENSG00000117289;	0.4746969	0.6150159	FALSE
TC01003638.hg.1	ENSG00000073756;	0.4746969	0.6150159	FALSE
TC03003381.hg.1	ENSG00000225697;	0.4746969	0.6150159	FALSE
TC01001090.hg.1	ENSG00000117289;	0.4746969	0.6150159	FALSE
TC03000631.hg.1	ENSG00000114023;	0.4746969	0.6150159	FALSE

TC02001206.hg.1	ENSG00000118257;	0.4750176	0.6150159	FALSE
TC0X000034.hg.1	ENSG00000146950;	0.4750176	0.6150159	FALSE
TC0X000034.hg.1	ENSG00000146950;	0.4750176	0.6150159	FALSE
TC06001027.hg.1	ENSG00000118503;	0.4750176	0.6150159	FALSE
TC02002823.hg.1	ENSG00000135919;	0.4756013	0.6152135	FALSE
TC02000041.hg.1	ENSG00000115738;	0.4756013	0.6152135	FALSE
TC01000783.hg.1	ENSG00000117069;	0.4774247	0.6172924	FALSE
TC01001473.hg.1	ENSG00000143153;	0.4809076	0.6214226	FALSE
TC04001084.hg.1	ENSG00000109819;	0.4812798	0.6214226	FALSE
TC05001385.hg.1	ENSG00000145632;	0.4835895	0.6214226	FALSE
TC14001124.hg.1	ENSG00000100504	0.4857198	0.6214226	FALSE
TC06004141.hg.1	ENSG00000112096	0.4857198	0.6214226	FALSE
TC05001617.hg.1	ENSG00000164307	0.4857198	0.6214226	FALSE
TC0Y000351.hg.1	ENSG00000129824	0.4857198	0.6214226	FALSE
TC02002823.hg.1	ENSG00000135919	0.4857198	0.6214226	FALSE
TC01000783.hg.1	ENSG00000117069	0.4857198	0.6214226	FALSE
TC07001562.hg.1	ENSG00000019991	0.4857198	0.6214226	FALSE
TC17001902.hg.1	ENSG00000161544	0.4857198	0.6214226	FALSE
TC0Y000185.hg.1	ENSG00000012817	0.4857198	0.6214226	FALSE
TC05001617.hg.1	ENSG00000164307	0.4857198	0.6214226	FALSE
TC11000182.hg.1	ENSG00000148926	0.4857198	0.6214226	FALSE
TC01000394.hg.1	ENSG00000159023	0.4857198	0.6214226	FALSE
TC07000768.hg.1	ENSG00000174697	0.4857198	0.6214226	FALSE
TC05000054.hg.1	ENSG00000078295	0.4857198	0.6214226	FALSE
TC17001830.hg.1	ENSG00000154265	0.4857198	0.6214226	FALSE
TC02001031.hg.1	ENSG00000152256	0.4857198	0.6214226	FALSE
TC03000214.hg.1	ENSG00000168036	0.4857198	0.6214226	FALSE
TC15000971.hg.1	ENSG00000184254	0.4857198	0.6214226	FALSE
TC07000768.hg.1	ENSG00000174697	0.4857198	0.6214226	FALSE
TC12000076.hg.1	ENSG00000010278	0.4857198	0.6214226	FALSE
TC01003638.hg.1	ENSG00000073756	0.4857198	0.6214226	FALSE
TC05000914.hg.1	ENSG00000145934	0.4857198	0.6214226	FALSE
TC06004141.hg.1	ENSG00000112096	0.4857198	0.6214226	FALSE
TC20000162.hg.1	ENSG00000101463	0.4857198	0.6214226	FALSE
TC20000162.hg.1	ENSG00000101463	0.4857198	0.6214226	FALSE
TC0X000943.hg.1	ENSG00000198947	0.4857198	0.6214226	FALSE
TC07001552.hg.1	ENSG00000187391	0.4857198	0.6214226	FALSE
TC10001663.hg.1	ENSG00000119927	0.4857198	0.6214226	FALSE
TC02001100.hg.1	ENSG00000138448	0.4857198	0.6214226	FALSE
TC16000668.hg.1	ENSG00000103241	0.4857198	0.6214226	FALSE
TC07000643.hg.1	ENSG00000106366	0.4857198	0.6214226	FALSE
TC12001916.hg.1	ENSG00000074590	0.4857198	0.6214226	FALSE
TC10000438.hg.1	ENSG00000107731	0.4857198	0.6214226	FALSE
TC01002706.hg.1	ENSG00000184292;	0.4860625	0.6214226	FALSE
TC02000041.hg.1	ENSG00000115738;	0.4860625	0.6214226	FALSE
TC01001805.hg.1	ENSG00000092969;	0.4882867	0.6238881	FALSE
TC02000286.hg.1	ENSG00000171150;	0.4883553	0.6238881	FALSE
TC01001607.hg.1	ENSG00000116711;I	0.4913523	0.6275296	FALSE
TC01001090.hg.1	ENSG00000117289;	0.4924215	0.6286139	FALSE
TC02000041.hg.1	ENSG00000115738;	0.4924215	0.6286139	FALSE
TC04000699.hg.1	ENSG00000109458;	0.4964221	0.6333431	FALSE
TC02000286.hg.1	ENSG00000171150;	0.4964221	0.6333431	FALSE
TC01001112.hg.1;	ENSG00000131778;	0.5003767	0.6381033	FALSE
TC03000986.hg.1	ENSG00000163872;	0.50119	0.6385575	FALSE
TC04001084.hg.1	ENSG00000109819;	0.50119	0.6385575	FALSE
TC05000243.hg.1	ENSG00000095015;	0.5014787	0.6385575	FALSE
TC01002954.hg.1	ENSG00000134243;	0.5014787	0.6385575	FALSE
TC05000746.hg.1	ENSG00000113209;	0.5014787	0.6385575	FALSE

TC01001624.hg.1	ENSG00000116741;	0.5014787	0.6385575	FALSE
TC05000243.hg.1	ENSG00000095015;	0.5014787	0.6385575	FALSE
TC01001736.hg.1	ENSG00000196352;	0.5032505	0.6401472	FALSE
TC01001805.hg.1	ENSG00000092969;	0.5032505	0.6401472	FALSE
TC11000404.hg.1	ENSG00000134574	0.5066669	0.6402697	FALSE
TC06001027.hg.1	ENSG00000118503	0.5066669	0.6402697	FALSE
TC18000548.hg.1	ENSG00000183287	0.5066669	0.6402697	FALSE
TC05000159.hg.1	ENSG00000168685	0.5066669	0.6402697	FALSE
TC05000159.hg.1	ENSG00000168685	0.5066669	0.6402697	FALSE
TC15001136.hg.1	ENSG00000166664	0.5066669	0.6402697	FALSE
TC01002195.hg.1	ENSG00000116649	0.5066669	0.6402697	FALSE
TC16001225.hg.1	ENSG00000181019	0.5066669	0.6402697	FALSE
TC09000388.hg.1	ENSG00000148053	0.5066669	0.6402697	FALSE
TC01000728.hg.1	ENSG00000162433	0.5066669	0.6402697	FALSE
TC05001385.hg.1	ENSG00000145632	0.5066669	0.6402697	FALSE
TC06000087.hg.1	ENSG00000078401	0.5066669	0.6402697	FALSE
TC12000621.hg.1	ENSG00000135643	0.5066669	0.6402697	FALSE
TC17000241.hg.1	ENSG00000142494	0.5066669	0.6402697	FALSE
TC05000165.hg.1	ENSG00000079215	0.5066669	0.6402697	FALSE
TC07000137.hg.1	ENSG00000136244	0.5066669	0.6402697	FALSE
TC08000508.hg.1	ENSG00000104435	0.5066669	0.6402697	FALSE
TC07000768.hg.1	ENSG00000174697	0.5066669	0.6402697	FALSE
TC07000768.hg.1	ENSG00000174697	0.5066669	0.6402697	FALSE
TC01000685.hg.1	ENSG00000162409	0.5066669	0.6402697	FALSE
TC0X000034.hg.1	ENSG00000146950	0.5066669	0.6402697	FALSE
TC18000047.hg.1	ENSG00000168461	0.5066669	0.6402697	FALSE
TC12001181.hg.1	ENSG00000197614	0.5066669	0.6402697	FALSE
TC22000259.hg.1	ENSG00000100292	0.5066669	0.6402697	FALSE
TC13000147.hg.1	ENSG00000102760	0.5066669	0.6402697	FALSE
TC20000162.hg.1	ENSG00000101463	0.5066669	0.6402697	FALSE
TC11000404.hg.1	ENSG00000134574	0.5066669	0.6402697	FALSE
TC22000259.hg.1	ENSG00000100292	0.5066669	0.6402697	FALSE
TC15000622.hg.1	ENSG00000166949	0.5066669	0.6402697	FALSE
TC08001567.hg.1	ENSG00000170961	0.5066669	0.6402697	FALSE
TC07000328.hg.1	ENSG00000146648	0.5066669	0.6402697	FALSE
TC01001805.hg.1	ENSG00000092969	0.5066669	0.6402697	FALSE
TC02002624.hg.1	ENSG00000115415	0.5066669	0.6402697	FALSE
TC21000345.hg.1	ENSG00000154734	0.5066669	0.6402697	FALSE
TC06001027.hg.1	ENSG00000118503	0.5066669	0.6402697	FALSE
TC03000631.hg.1	ENSG00000114023	0.5066669	0.6402697	FALSE
TC17000383.hg.1	ENSG00000108691	0.5066669	0.6402697	FALSE
TC12001181.hg.1	ENSG00000197614	0.5066669	0.6402697	FALSE
TC04001084.hg.1	ENSG00000109819	0.5066669	0.6402697	FALSE
TC14000306.hg.1	ENSG00000125384	0.5066669	0.6402697	FALSE
TC04000432.hg.1	ENSG00000156234	0.5066669	0.6402697	FALSE
TC01001805.hg.1	ENSG00000092969;	0.5097031	0.6402697	FALSE
TC03000986.hg.1	ENSG00000163872;	0.5098919	0.6403189	FALSE
TC02002419.hg.1	ENSG00000115963;	0.5116257	0.6423078	FALSE
TC03000917.hg.1	ENSG00000136603;	0.5132705	0.6441839	FALSE
TC02000416.hg.1	ENSG00000116005;	0.5143831	0.6449185	FALSE
TC01000728.hg.1	ENSG00000162433;	0.5143831	0.6449185	FALSE
TC03001255.hg.1	ENSG00000033867;	0.5143831	0.6449185	FALSE
TC03001255.hg.1	ENSG00000033867;	0.5143831	0.6449185	FALSE
TC0X000116.hg.1	ENSG00000005889;	0.5143831	0.6449185	FALSE
TC03000214.hg.1	ENSG00000168036;	0.5143831	0.6449185	FALSE
TC02002605.hg.1	ENSG00000064989;	0.5168199	0.6472154	FALSE
TC04001084.hg.1	ENSG00000109819;	0.5168199	0.6472154	FALSE
TC01000904.hg.1	ENSG00000162692;	0.5185244	0.6490652	FALSE

TC01001805.hg.1	ENSG00000092969;	0.5199765	0.6506926	FALSE
TC03000846.hg.1	ENSG00000196549;	0.5212938	0.6521504	FALSE
TC01000685.hg.1	ENSG00000162409;	0.521943	0.6521534	FALSE
TC09000347.hg.1	ENSG00000099139	0.526762	0.6521534	FALSE
TC0Y000185.hg.1	ENSG00000012817	0.526762	0.6521534	FALSE
TC10001327.hg.1	ENSG00000122877	0.526762	0.6521534	FALSE
TC04002948.hg.1	ENSG00000137463	0.526762	0.6521534	FALSE
TC0Y000185.hg.1	ENSG00000012817	0.526762	0.6521534	FALSE
TC12002066.hg.1	ENSG00000158104	0.526762	0.6521534	FALSE
TC14000067.hg.1	ENSG00000181784	0.526762	0.6521534	FALSE
TC06000087.hg.1	ENSG00000078401	0.526762	0.6521534	FALSE
TC10001663.hg.1	ENSG00000119927	0.526762	0.6521534	FALSE
TC05001617.hg.1	ENSG00000164307	0.526762	0.6521534	FALSE
TC02002823.hg.1	ENSG00000135919	0.526762	0.6521534	FALSE
TC07000643.hg.1	ENSG00000106366	0.526762	0.6521534	FALSE
TC01000685.hg.1	ENSG00000162409	0.526762	0.6521534	FALSE
TC01002195.hg.1	ENSG00000116649	0.526762	0.6521534	FALSE
TC10000811.hg.1	ENSG00000150594	0.526762	0.6521534	FALSE
TC17000383.hg.1	ENSG00000108691	0.526762	0.6521534	FALSE
TC10001517.hg.1	ENSG00000148677	0.526762	0.6521534	FALSE
TC07001374.hg.1	ENSG00000106070	0.526762	0.6521534	FALSE
TC02000286.hg.1	ENSG00000171150	0.526762	0.6521534	FALSE
TC04000729.hg.1	ENSG00000151617	0.526762	0.6521534	FALSE
TC04000408.hg.1	ENSG00000169429	0.526762	0.6521534	FALSE
TC14001141.hg.1	ENSG00000197930	0.526762	0.6521534	FALSE
TC0X000034.hg.1	ENSG00000146950	0.526762	0.6521534	FALSE
TC05000054.hg.1	ENSG00000078295	0.526762	0.6521534	FALSE
TC17000158.hg.1	ENSG00000141052	0.526762	0.6521534	FALSE
TC02002624.hg.1	ENSG00000115415	0.526762	0.6521534	FALSE
TC14001443.hg.1	ENSG00000140092	0.526762	0.6521534	FALSE
TC02001031.hg.1	ENSG00000152256	0.526762	0.6521534	FALSE
TC07000643.hg.1	ENSG00000106366	0.526762	0.6521534	FALSE
TC10001517.hg.1	ENSG00000148677	0.526762	0.6521534	FALSE
TC0X000943.hg.1	ENSG00000198947	0.526762	0.6521534	FALSE
TC0X000943.hg.1	ENSG00000198947	0.526762	0.6521534	FALSE
TC06000087.hg.1	ENSG00000078401;	0.5270646	0.6521534	FALSE
TC01000904.hg.1	ENSG00000162692;	0.5270646	0.6521534	FALSE
TC03001362.hg.1	ENSG00000160808;	0.5270646	0.6521534	FALSE
TC05000243.hg.1	ENSG00000095015;	0.5270646	0.6521534	FALSE
TC01000785.hg.1	ENSG00000154027;	0.527085	0.6521534	FALSE
TC03002008.hg.1	ENSG00000144959;	0.5350448	0.6616969	FALSE
TC01000972.hg.1	ENSG00000064886;	0.5351795	0.6616969	FALSE
TC03000986.hg.1	ENSG00000163872;	0.5355711	0.6616969	FALSE
TC03000214.hg.1	ENSG00000168036;	0.5355711	0.6616969	FALSE
TC04000469.hg.1	ENSG00000163629;	0.5355711	0.6616969	FALSE
TC03001884.hg.1	ENSG00000047457;	0.5355711	0.6616969	FALSE
TC01000785.hg.1	ENSG00000154027;	0.5355711	0.6616969	FALSE
TC01001473.hg.1	ENSG00000143153;	0.537468	0.6634661	FALSE
TC03001362.hg.1	ENSG00000160808;	0.5395199	0.6655193	FALSE
TC06001029.hg.1	ENSG00000112379;	0.5395199	0.6655193	FALSE
TC02000286.hg.1	ENSG00000171150;	0.5395199	0.6655193	FALSE
TC10001711.hg.1;	ENSG00000066468;	0.5395199	0.6655193	FALSE
TC01000904.hg.1	ENSG00000162692;	0.5418575	0.6679217	FALSE
TC03000986.hg.1	ENSG00000163872;	0.5425956	0.668639	FALSE
TC01000685.hg.1	ENSG00000162409;	0.5434981	0.6687124	FALSE
TC02002714.hg.1	ENSG00000163251;	0.5439803	0.6687124	FALSE
TC06000004.hg.1	ENSG00000112679	0.5460397	0.6687124	FALSE
TC02000432.hg.1	ENSG00000135636	0.5460397	0.6687124	FALSE

TC10001711.hg.1	ENSG00000066468	0.5460397	0.6687124	FALSE
TC01002195.hg.1	ENSG00000116649	0.5460397	0.6687124	FALSE
TC17000241.hg.1	ENSG00000142494	0.5460397	0.6687124	FALSE
TC0Y000351.hg.1	ENSG00000129824	0.5460397	0.6687124	FALSE
TC05000159.hg.1	ENSG00000168685	0.5460397	0.6687124	FALSE
TC04000313.hg.1	ENSG00000157404	0.5460397	0.6687124	FALSE
TC04000408.hg.1	ENSG00000169429	0.5460397	0.6687124	FALSE
TC03001362.hg.1	ENSG00000160808	0.5460397	0.6687124	FALSE
TC12001890.hg.1	ENSG00000017427	0.5460397	0.6687124	FALSE
TC02000041.hg.1	ENSG00000115738	0.5460397	0.6687124	FALSE
TC10001663.hg.1	ENSG00000119927	0.5460397	0.6687124	FALSE
TC07001374.hg.1	ENSG00000106070	0.5460397	0.6687124	FALSE
TC12003293.hg.1	ENSG00000180818	0.5460397	0.6687124	FALSE
TC04000408.hg.1	ENSG00000169429	0.5460397	0.6687124	FALSE
TC07000137.hg.1	ENSG00000136244	0.5460397	0.6687124	FALSE
TC02002624.hg.1	ENSG00000115415	0.5460397	0.6687124	FALSE
TC12001890.hg.1	ENSG00000017427	0.5460397	0.6687124	FALSE
TC01003497.hg.1	ENSG00000117479	0.5460397	0.6687124	FALSE
TC02002606.hg.1	ENSG00000003436	0.5460397	0.6687124	FALSE
TC03000631.hg.1	ENSG00000114023	0.5460397	0.6687124	FALSE
TC01001805.hg.1	ENSG00000092969;	0.5465603	0.6687124	FALSE
TC06001772.hg.1	ENSG00000146233;	0.5517464	0.6745755	FALSE
TC01003638.hg.1	ENSG00000073756;	0.5517464	0.6745755	FALSE
TC10000811.hg.1;	ENSG00000150594;	0.5517464	0.6745755	FALSE
TC06001027.hg.1	ENSG00000118503;	0.5517464	0.6745755	FALSE
TC01000904.hg.1	ENSG00000162692;	0.5527353	0.6752061	FALSE
TC01000904.hg.1	ENSG00000162692;	0.5527353	0.6752061	FALSE
TC02000286.hg.1	ENSG00000171150;	0.5562332	0.6791884	FALSE
TC02002808.hg.1	ENSG00000116106;	0.5605595	0.6840189	FALSE
TC03000214.hg.1	ENSG00000168036;	0.5605595	0.6840189	FALSE
TC02001100.hg.1;	ENSG00000138448;	0.5617592	0.6840189	FALSE
TC06000945.hg.1	ENSG00000152661;	0.5637421	0.6840189	FALSE
TC0Y000351.hg.1	ENSG00000129824;	0.5637421	0.6840189	FALSE
TC17001462.hg.1	ENSG00000131747;	0.5637421	0.6840189	FALSE
TC10001711.hg.1;	ENSG00000066468;	0.5637421	0.6840189	FALSE
TC04000313.hg.1	ENSG00000157404;	0.5637421	0.6840189	FALSE
TC18000097.hg.1	ENSG00000101773	0.5645333	0.6840189	FALSE
TC02000286.hg.1	ENSG00000171150	0.5645333	0.6840189	FALSE
TC01003129.hg.1	ENSG00000143140	0.5645333	0.6840189	FALSE
TC18000552.hg.1	ENSG00000197563	0.5645333	0.6840189	FALSE
TC11002162.hg.1	ENSG00000137501	0.5645333	0.6840189	FALSE
TC03000214.hg.1	ENSG00000168036	0.5645333	0.6840189	FALSE
TC06002168.hg.1	ENSG00000164442	0.5645333	0.6840189	FALSE
TC07000852.hg.1	ENSG00000181072	0.5645333	0.6840189	FALSE
TC07001630.hg.1	ENSG00000070669	0.5645333	0.6840189	FALSE
TC14000819.hg.1	ENSG00000185100	0.5645333	0.6840189	FALSE
TC14000819.hg.1	ENSG00000185100	0.5645333	0.6840189	FALSE
TC01001607.hg.1	ENSG00000116711	0.5645333	0.6840189	FALSE
TC03001255.hg.1	ENSG00000033867	0.5645333	0.6840189	FALSE
TC03001590.hg.1	ENSG00000184500	0.5645333	0.6840189	FALSE
TC04000313.hg.1	ENSG00000157404	0.5645333	0.6840189	FALSE
TC08000508.hg.1	ENSG00000104435	0.5645333	0.6840189	FALSE
TC06001027.hg.1	ENSG00000118503	0.5645333	0.6840189	FALSE
TC11002170.hg.1	ENSG00000174804	0.5645333	0.6840189	FALSE
TC01001805.hg.1	ENSG00000092969	0.5645333	0.6840189	FALSE
TC04000313.hg.1	ENSG00000157404	0.5645333	0.6840189	FALSE
TC17001830.hg.1	ENSG00000154265	0.5645333	0.6840189	FALSE
TC10000874.hg.1	ENSG00000203805	0.5645333	0.6840189	FALSE

TC06002168.hg.1	ENSG00000164442	0.5645333	0.6840189	FALSE
TC07000643.hg.1	ENSG00000106366	0.5645333	0.6840189	FALSE
TC10000811.hg.1	ENSG00000150594	0.5645333	0.6840189	FALSE
TC11000707.hg.1	ENSG00000162337	0.5645333	0.6840189	FALSE
TC01001805.hg.1	ENSG00000092969;	0.5657816	0.6840189	FALSE
TC02001031.hg.1	ENSG00000152256;	0.5687255	0.6873839	FALSE
TC01000785.hg.1	ENSG00000154027;	0.56894	0.6874491	FALSE
TC01001688.hg.1	ENSG00000058668;	0.5724334	0.691475	FALSE
TC02001139.hg.1;	ENSG00000196950;	0.5752032	0.6941087	FALSE
TC03000013.hg.1	ENSG00000150995;	0.5752032	0.6941087	FALSE
TC04000313.hg.1	ENSG00000157404;	0.5752032	0.6941087	FALSE
TC03000214.hg.1	ENSG00000168036;	0.5755055	0.6941087	FALSE
TC10001711.hg.1;	ENSG00000066468;	0.5755055	0.6941087	FALSE
TC07000137.hg.1	ENSG00000136244;	0.5755055	0.6941087	FALSE
TC01002706.hg.1	ENSG00000184292;	0.5755055	0.6941087	FALSE
TC01000115.hg.1;	ENSG00000171621;	0.5785654	0.6973082	FALSE
TC02000041.hg.1	ENSG00000115738;	0.5817685	0.6980253	FALSE
TC06001027.hg.1	ENSG00000118503	0.5822745	0.6980253	FALSE
TC07000768.hg.1	ENSG00000174697	0.5822745	0.6980253	FALSE
TC18000047.hg.1	ENSG00000168461	0.5822745	0.6980253	FALSE
TC12002155.hg.1	ENSG00000176915	0.5822745	0.6980253	FALSE
TC02002808.hg.1	ENSG00000116106	0.5822745	0.6980253	FALSE
TC05000054.hg.1	ENSG00000078295	0.5822745	0.6980253	FALSE
TC01003718.hg.1	ENSG00000133048	0.5822745	0.6980253	FALSE
TC07001374.hg.1	ENSG00000106070	0.5822745	0.6980253	FALSE
TC10001711.hg.1	ENSG00000066468	0.5822745	0.6980253	FALSE
TC07000643.hg.1	ENSG00000106366	0.5822745	0.6980253	FALSE
TC07000768.hg.1	ENSG00000174697	0.5822745	0.6980253	FALSE
TC02001628.hg.1	ENSG00000084674	0.5822745	0.6980253	FALSE
TC12001837.hg.1	ENSG00000074527	0.5822745	0.6980253	FALSE
TC08000508.hg.1	ENSG00000104435	0.5822745	0.6980253	FALSE
TC02002624.hg.1	ENSG00000115415	0.5822745	0.6980253	FALSE
TC05001385.hg.1	ENSG00000145632	0.5822745	0.6980253	FALSE
TC03001362.hg.1	ENSG00000160808	0.5822745	0.6980253	FALSE
TC02000041.hg.1	ENSG00000115738	0.5822745	0.6980253	FALSE
TC07001374.hg.1	ENSG00000106070	0.5822745	0.6980253	FALSE
TC01000728.hg.1	ENSG00000162433;	0.5825845	0.6980253	FALSE
TC01001090.hg.1	ENSG00000117289;	0.584496	0.7000217	FALSE
TC06001772.hg.1	ENSG00000146233;	0.584496	0.7000217	FALSE
TC02001628.hg.1	ENSG00000084674;	0.5870356	0.70267	FALSE
TC06000945.hg.1	ENSG00000152661;	0.5870356	0.70267	FALSE
TC01001688.hg.1	ENSG00000058668;	0.5905109	0.7065336	FALSE
TC01000115.hg.1;	ENSG00000171621;	0.5918158	0.707897	FALSE
TC01001473.hg.1	ENSG00000143153;	0.5927074	0.7087654	FALSE
TC01003211.hg.1;	ENSG00000143387;	0.5929109	0.7088108	FALSE
TC01000115.hg.1;	ENSG00000171621;	0.5951068	0.7093939	FALSE
TC01001112.hg.1;	ENSG00000131778;	0.5975873	0.7093939	FALSE
TC01001805.hg.1	ENSG00000092969;	0.5983319	0.7093939	FALSE
TC02000466.hg.1	ENSG00000159399;	0.5983319	0.7093939	FALSE
TC05001481.hg.1	ENSG00000171617;	0.5983319	0.7093939	FALSE
TC03001466.hg.1	ENSG00000163931;	0.5983319	0.7093939	FALSE
TC03000131.hg.1	ENSG00000077092;	0.5983319	0.7093939	FALSE
TC03001466.hg.1	ENSG00000163931;	0.5983319	0.7093939	FALSE
TC03000214.hg.1	ENSG00000168036;	0.5983319	0.7093939	FALSE
TC03000214.hg.1	ENSG00000168036;	0.5983319	0.7093939	FALSE
TC20000621.hg.1	ENSG00000101384	0.599294	0.7093939	FALSE
TC19001103.hg.1	ENSG00000125730	0.599294	0.7093939	FALSE
TC01003129.hg.1	ENSG00000143140	0.599294	0.7093939	FALSE

TC12000621.hg.1	ENSG00000135643	0.599294	0.7093939	FALSE
TC05001617.hg.1	ENSG00000164307	0.599294	0.7093939	FALSE
TC03000214.hg.1	ENSG00000168036	0.599294	0.7093939	FALSE
TC08001022.hg.1	ENSG00000147408	0.599294	0.7093939	FALSE
TC06004141.hg.1	ENSG00000112096	0.599294	0.7093939	FALSE
TC18000565.hg.1	ENSG00000166479	0.599294	0.7093939	FALSE
TC07000768.hg.1	ENSG00000174697	0.599294	0.7093939	FALSE
TC12003293.hg.1	ENSG00000180818	0.599294	0.7093939	FALSE
TC02000041.hg.1	ENSG00000115738	0.599294	0.7093939	FALSE
TC17001462.hg.1	ENSG00000131747	0.599294	0.7093939	FALSE
TC17001462.hg.1	ENSG00000131747	0.599294	0.7093939	FALSE
TC02001100.hg.1	ENSG00000138448	0.599294	0.7093939	FALSE
TC12000227.hg.1	ENSG00000172572	0.599294	0.7093939	FALSE
TC0X000207.hg.1	ENSG00000189221	0.599294	0.7093939	FALSE
TC05003423.hg.1	ENSG00000082196	0.599294	0.7093939	FALSE
TC08000127.hg.1	ENSG00000003989	0.599294	0.7093939	FALSE
TC14001124.hg.1	ENSG00000100504	0.599294	0.7093939	FALSE
TC06001027.hg.1	ENSG00000118503	0.599294	0.7093939	FALSE
TC17001462.hg.1	ENSG00000131747	0.599294	0.7093939	FALSE
TC02000286.hg.1	ENSG00000171150	0.599294	0.7093939	FALSE
TC07000768.hg.1	ENSG00000174697	0.599294	0.7093939	FALSE
TC11002162.hg.1	ENSG00000137501	0.599294	0.7093939	FALSE
TC01003497.hg.1	ENSG00000117479	0.599294	0.7093939	FALSE
TC05000914.hg.1	ENSG00000145934	0.599294	0.7093939	FALSE
TC02002624.hg.1	ENSG00000115415	0.599294	0.7093939	FALSE
TC02001628.hg.1	ENSG00000084674	0.599294	0.7093939	FALSE
TC09000358.hg.1	ENSG00000135069	0.599294	0.7093939	FALSE
TC02001031.hg.1	ENSG00000152256;	0.6002963	0.7093939	FALSE
TC02000286.hg.1	ENSG00000171150;	0.6005241	0.7093939	FALSE
TC01001688.hg.1	ENSG00000058668;	0.6005241	0.7093939	FALSE
TC02000082.hg.1	ENSG00000071575;	0.6005241	0.7093939	FALSE
TC02000286.hg.1	ENSG00000171150;	0.6026886	0.7115582	FALSE
TC03000214.hg.1	ENSG00000168036;	0.6082499	0.7179261	FALSE
TC01000728.hg.1	ENSG00000162433;	0.6085642	0.7180991	FALSE
TC07000643.hg.1	ENSG00000106366;	0.6093943	0.7186823	FALSE
TC02002714.hg.1	ENSG00000163251;	0.6093943	0.7186823	FALSE
TC20000876.hg.1	ENSG00000124107;	0.6093943	0.7186823	FALSE
TC02000041.hg.1	ENSG00000115738;	0.6115855	0.7208694	FALSE
TC02001100.hg.1;	ENSG00000138448;	0.6148757	0.7238324	FALSE
TC05000165.hg.1	ENSG00000079215	0.615621	0.7238324	FALSE
TC01000728.hg.1	ENSG00000162433	0.615621	0.7238324	FALSE
TC01000785.hg.1	ENSG00000154027	0.615621	0.7238324	FALSE
TC02000041.hg.1	ENSG00000115738	0.615621	0.7238324	FALSE
TC06001027.hg.1	ENSG00000118503	0.615621	0.7238324	FALSE
TC11000182.hg.1	ENSG00000148926	0.615621	0.7238324	FALSE
TC02000041.hg.1	ENSG00000115738	0.615621	0.7238324	FALSE
TC07000328.hg.1	ENSG00000146648	0.615621	0.7238324	FALSE
TC01001805.hg.1	ENSG00000092969	0.615621	0.7238324	FALSE
TC10000811.hg.1	ENSG00000150594	0.615621	0.7238324	FALSE
TC07000328.hg.1	ENSG00000146648	0.615621	0.7238324	FALSE
TC07000768.hg.1	ENSG00000174697	0.615621	0.7238324	FALSE
TC03000214.hg.1	ENSG00000168036	0.615621	0.7238324	FALSE
TC05000054.hg.1	ENSG00000078295	0.615621	0.7238324	FALSE
TC02002624.hg.1	ENSG00000115415	0.615621	0.7238324	FALSE
TC01001112.hg.1;	ENSG00000131778;	0.6201293	0.7274452	FALSE
TC17001462.hg.1	ENSG00000131747;	0.6202231	0.7274452	FALSE
TC02001100.hg.1;	ENSG00000138448;	0.6208442	0.7278745	FALSE
TC03000986.hg.1	ENSG00000163872;	0.6208442	0.7278745	FALSE

TC01001112.hg.1;	ENSG00000131778;	0.6242305	0.7315442	FALSE
TC01003605.hg.1	ENSG00000135821;	0.6256224	0.7329747	FALSE
TC02000219.hg.1	ENSG00000049323;	0.6289749	0.7366	FALSE
TC01001090.hg.1	ENSG00000117289;	0.6289749	0.7366	FALSE
TC03001884.hg.1	ENSG00000047457;	0.630819	0.736885	FALSE
TC07000137.hg.1	ENSG00000136244;	0.630819	0.736885	FALSE
TC01000785.hg.1	ENSG00000154027;	0.6308704	0.736885	FALSE
TC03002008.hg.1	ENSG00000144959;	0.6311382	0.736885	FALSE
TC05000159.hg.1	ENSG00000168685	0.6312838	0.736885	FALSE
TC01001188.hg.1	ENSG00000143369	0.6312838	0.736885	FALSE
TC10000811.hg.1	ENSG00000150594	0.6312838	0.736885	FALSE
TC01000785.hg.1	ENSG00000154027	0.6312838	0.736885	FALSE
TC06000087.hg.1	ENSG00000078401	0.6312838	0.736885	FALSE
TC03000214.hg.1	ENSG00000168036	0.6312838	0.736885	FALSE
TC05000746.hg.1	ENSG00000113209	0.6312838	0.736885	FALSE
TC18000552.hg.1	ENSG00000197563	0.6312838	0.736885	FALSE
TC17000124.hg.1	ENSG00000179593	0.6312838	0.736885	FALSE
TC02002823.hg.1	ENSG00000135919	0.6312838	0.736885	FALSE
TC0X000943.hg.1	ENSG00000198947	0.6312838	0.736885	FALSE
TC02000891.hg.1	ENSG00000150540	0.6312838	0.736885	FALSE
TC01001805.hg.1	ENSG00000092969	0.6312838	0.736885	FALSE
TC12000227.hg.1	ENSG00000172572	0.6312838	0.736885	FALSE
TC04000313.hg.1	ENSG00000157404;	0.6374656	0.7425824	FALSE
TC01000728.hg.1	ENSG00000162433;	0.6387501	0.7438763	FALSE
TC01000685.hg.1	ENSG00000162409;	0.641183	0.7461006	FALSE
TC08000508.hg.1	ENSG00000104435;	0.641183	0.7461006	FALSE
TC02001628.hg.1	ENSG00000084674;	0.641183	0.7461006	FALSE
TC10000406.hg.1	ENSG00000138336;	0.641183	0.7461006	FALSE
TC07000137.hg.1	ENSG00000136244;	0.641183	0.7461006	FALSE
TC02002808.hg.1	ENSG00000116106;	0.6458196	0.7494176	FALSE
TC04000699.hg.1	ENSG00000109458;	0.6458196	0.7494176	FALSE
TC03000214.hg.1	ENSG00000168036	0.6463092	0.7494176	FALSE
TC20000368.hg.1	ENSG00000064655	0.6463092	0.7494176	FALSE
TC06000087.hg.1	ENSG00000078401	0.6463092	0.7494176	FALSE
TC05000159.hg.1	ENSG00000168685	0.6463092	0.7494176	FALSE
TC04002931.hg.1	ENSG00000134853	0.6463092	0.7494176	FALSE
TC01000685.hg.1	ENSG00000162409	0.6463092	0.7494176	FALSE
TC17000241.hg.1	ENSG00000142494	0.6463092	0.7494176	FALSE
TC10000811.hg.1	ENSG00000150594	0.6463092	0.7494176	FALSE
TC06001027.hg.1	ENSG00000118503	0.6463092	0.7494176	FALSE
TC20000942.hg.1	ENSG00000101096	0.6463092	0.7494176	FALSE
TC03000013.hg.1	ENSG00000150995	0.6463092	0.7494176	FALSE
TC03000013.hg.1	ENSG00000150995	0.6463092	0.7494176	FALSE
TC03001362.hg.1	ENSG00000160808	0.6463092	0.7494176	FALSE
TC06000087.hg.1	ENSG00000078401	0.6463092	0.7494176	FALSE
TC04000313.hg.1	ENSG00000157404	0.6463092	0.7494176	FALSE
TC11002178.hg.1	ENSG00000086991	0.6463092	0.7494176	FALSE
TC04000432.hg.1	ENSG00000156234	0.6463092	0.7494176	FALSE
TC01001112.hg.1;	ENSG00000131778;	0.6493773	0.7511441	FALSE
TC02002823.hg.1	ENSG00000135919;	0.6513163	0.7530817	FALSE
TC01000904.hg.1	ENSG00000162692;	0.6513163	0.7530817	FALSE
TC01001188.hg.1;	ENSG00000143369;	0.6540364	0.7557165	FALSE
TC01003638.hg.1	ENSG00000073756;	0.6540364	0.7557165	FALSE
TC04000699.hg.1	ENSG00000109458;	0.6540364	0.7557165	FALSE
TC06001027.hg.1	ENSG00000118503	0.6607232	0.7606302	FALSE
TC19001103.hg.1	ENSG00000125730	0.6607232	0.7606302	FALSE
TC03001362.hg.1	ENSG00000160808	0.6607232	0.7606302	FALSE
TC01002954.hg.1	ENSG00000134243	0.6607232	0.7606302	FALSE

TC01000785.hg.1	ENSG00000154027	0.6607232	0.7606302	FALSE
TC03001071.hg.1	ENSG00000114315	0.6607232	0.7606302	FALSE
TC03001071.hg.1	ENSG00000114315	0.6607232	0.7606302	FALSE
TC04000432.hg.1	ENSG00000156234	0.6607232	0.7606302	FALSE
TC10001663.hg.1	ENSG00000119927	0.6607232	0.7606302	FALSE
TC09000388.hg.1	ENSG00000148053	0.6607232	0.7606302	FALSE
TC10000811.hg.1	ENSG00000150594	0.6607232	0.7606302	FALSE
TC01001188.hg.1	ENSG00000143369	0.6607232	0.7606302	FALSE
TC16000571.hg.1	ENSG00000198373	0.6607232	0.7606302	FALSE
TC0X000943.hg.1	ENSG00000198947	0.6607232	0.7606302	FALSE
TC04001084.hg.1	ENSG00000109819;	0.6612206	0.7606302	FALSE
TC02000041.hg.1	ENSG00000115738;	0.6612206	0.7606302	FALSE
TC06001027.hg.1	ENSG00000118503;	0.6621157	0.761353	FALSE
TC01000685.hg.1	ENSG00000162409;	0.6625448	0.7616418	FALSE
TC01001112.hg.1;	ENSG00000131778;	0.6640183	0.7629988	FALSE
TC03000917.hg.1	ENSG00000136603;	0.6640816	0.7629988	FALSE
TC01002578.hg.1	ENSG00000117394;	0.6654265	0.7643389	FALSE
TC01003211.hg.1;	ENSG00000143387;	0.6700574	0.7693484	FALSE
TC04001084.hg.1	ENSG00000109819;	0.6700574	0.7693484	FALSE
TC11000404.hg.1;	ENSG00000134574;	0.6708977	0.7697971	FALSE
TC0Y000351.hg.1	ENSG00000129824;	0.6708977	0.7697971	FALSE
TC15001110.hg.1;	ENSG00000104067;	0.6708977	0.7697971	FALSE
TC19001103.hg.1	ENSG00000125730	0.6745506	0.7726425	FALSE
TC19001103.hg.1	ENSG00000125730	0.6745506	0.7726425	FALSE
TC17001462.hg.1	ENSG00000131747	0.6745506	0.7726425	FALSE
TC0Y000351.hg.1	ENSG00000129824	0.6745506	0.7726425	FALSE
TC12002066.hg.1	ENSG00000158104	0.6745506	0.7726425	FALSE
TC14002310.hg.1	ENSG00000168398	0.6745506	0.7726425	FALSE
TC04000313.hg.1	ENSG00000157404	0.6745506	0.7726425	FALSE
TC04002931.hg.1	ENSG00000134853	0.6745506	0.7726425	FALSE
TC01001090.hg.1	ENSG00000117289	0.6745506	0.7726425	FALSE
TC06004141.hg.1	ENSG00000112096	0.6745506	0.7726425	FALSE
TC01001112.hg.1;	ENSG00000131778;	0.6759078	0.7730594	FALSE
TC01000728.hg.1	ENSG00000162433;	0.6770613	0.7741719	FALSE
TC02000270.hg.1	ENSG00000152527;	0.6778616	0.7746731	FALSE
TC02000270.hg.1	ENSG00000152527;	0.6778616	0.7746731	FALSE
TC01001188.hg.1;	ENSG00000143369;	0.6778616	0.7746731	FALSE
TC01001112.hg.1;	ENSG00000131778;	0.6792726	0.7758714	FALSE
TC0X000038.hg.1	ENSG00000073464;	0.6803496	0.7766871	FALSE
TC01002954.hg.1	ENSG00000134243;	0.6803496	0.7766871	FALSE
TC07000768.hg.1	ENSG00000174697;	0.6803496	0.7766871	FALSE
TC01000728.hg.1	ENSG00000162433;	0.6835885	0.7799687	FALSE
TC02001100.hg.1;	ENSG00000138448;	0.6855283	0.7817653	FALSE
TC01002954.hg.1	ENSG00000134243;	0.6855283	0.7817653	FALSE
TC02001100.hg.1;	ENSG00000138448;	0.6855283	0.7817653	FALSE
TC01001805.hg.1	ENSG00000092969;	0.6869639	0.7824973	FALSE
TC05001385.hg.1	ENSG00000145632;	0.6869639	0.7824973	FALSE
TC02000041.hg.1	ENSG00000115738;	0.6869639	0.7824973	FALSE
TC09000965.hg.1	ENSG00000147883	0.6878153	0.7824973	FALSE
TC04000313.hg.1	ENSG00000157404	0.6878153	0.7824973	FALSE
TC0Y000351.hg.1	ENSG00000129824	0.6878153	0.7824973	FALSE
TC01000785.hg.1	ENSG00000154027	0.6878153	0.7824973	FALSE
TC02000891.hg.1	ENSG00000150540	0.6878153	0.7824973	FALSE
TC05000054.hg.1	ENSG00000078295	0.6878153	0.7824973	FALSE
TC09000388.hg.1	ENSG00000148053	0.6878153	0.7824973	FALSE
TC10000811.hg.1	ENSG00000150594	0.6878153	0.7824973	FALSE
TC01000703.hg.1	ENSG00000132849	0.6878153	0.7824973	FALSE
TC01000685.hg.1	ENSG00000162409;	0.6887687	0.7825422	FALSE

TC14000067.hg.1	ENSG00000181784;	0.6895786	0.7831506	FALSE
TC06000087.hg.1	ENSG00000078401;	0.6895786	0.7831506	FALSE
TC01002578.hg.1	ENSG00000117394;	0.692699	0.7863815	FALSE
TC02000286.hg.1	ENSG00000171150;	0.6935343	0.7870166	FALSE
TC01001090.hg.1	ENSG00000117289;	0.6935343	0.7870166	FALSE
TC0X000038.hg.1	ENSG00000073464;	0.6985872	0.7911873	FALSE
TC09000388.hg.1	ENSG00000148053;	0.6985872	0.7911873	FALSE
TC01000728.hg.1	ENSG00000162433;	0.6999827	0.7911873	FALSE
TC02001031.hg.1	ENSG00000152256;	0.7004511	0.7911873	FALSE
TC01000904.hg.1	ENSG00000162692;	0.7004511	0.7911873	FALSE
TC01001805.hg.1	ENSG00000092969;	0.7004978	0.7911873	FALSE
TC0X001279.hg.1	ENSG00000077274	0.7005401	0.7911873	FALSE
TC01000785.hg.1	ENSG00000154027	0.7005401	0.7911873	FALSE
TC18000552.hg.1	ENSG00000197563	0.7005401	0.7911873	FALSE
TC07001534.hg.1	ENSG00000127946	0.7005401	0.7911873	FALSE
TC09000388.hg.1	ENSG00000148053	0.7005401	0.7911873	FALSE
TC01000728.hg.1	ENSG00000162433	0.7005401	0.7911873	FALSE
TC10000811.hg.1	ENSG00000150594	0.7005401	0.7911873	FALSE
TC02002823.hg.1	ENSG00000135919	0.7005401	0.7911873	FALSE
TC10001711.hg.1	ENSG00000066468	0.7005401	0.7911873	FALSE
TC10002939.hg.1	ENSG00000151303	0.7005401	0.7911873	FALSE
TC22000259.hg.1	ENSG00000100292	0.7005401	0.7911873	FALSE
TC06004141.hg.1	ENSG00000112096	0.7005401	0.7911873	FALSE
TC0Y000351.hg.1	ENSG00000129824	0.7005401	0.7911873	FALSE
TC02000041.hg.1	ENSG00000115738	0.7005401	0.7911873	FALSE
TC12001890.hg.1	ENSG00000017427	0.7005401	0.7911873	FALSE
TC0X000943.hg.1	ENSG00000198947	0.7005401	0.7911873	FALSE
TC09000965.hg.1	ENSG00000147883	0.7005401	0.7911873	FALSE
TC10001663.hg.1	ENSG00000119927	0.7005401	0.7911873	FALSE
TC03000917.hg.1	ENSG00000136603;	0.7019223	0.7911873	FALSE
TC01000904.hg.1	ENSG00000162692;	0.7022539	0.7913527	FALSE
TC01000972.hg.1	ENSG00000064886;	0.7047679	0.7939767	FALSE
TC01002578.hg.1	ENSG00000117394;	0.7071436	0.7958228	FALSE
TC03001466.hg.1	ENSG00000163931;	0.707378	0.7958228	FALSE
TC05000840.hg.1	ENSG00000123643;	0.707378	0.7958228	FALSE
TC09000015.hg.1	ENSG00000147852;	0.707378	0.7958228	FALSE
TC04001084.hg.1	ENSG00000109819;	0.707378	0.7958228	FALSE
TC02000286.hg.1	ENSG00000171150;	0.707378	0.7958228	FALSE
TC02001031.hg.1	ENSG00000152256;	0.7077079	0.7958228	FALSE
TC15001136.hg.1	ENSG00000166664	0.712747	0.8001232	FALSE
TC15001136.hg.1	ENSG00000166664	0.712747	0.8001232	FALSE
TC08001175.hg.1	ENSG00000104368	0.712747	0.8001232	FALSE
TC18000097.hg.1	ENSG00000101773	0.712747	0.8001232	FALSE
TC0X000943.hg.1	ENSG00000198947	0.712747	0.8001232	FALSE
TC02000082.hg.1	ENSG00000071575	0.712747	0.8001232	FALSE
TC0X000034.hg.1	ENSG00000146950	0.712747	0.8001232	FALSE
TC05001617.hg.1	ENSG00000164307	0.712747	0.8001232	FALSE
TC02002624.hg.1	ENSG00000115415	0.712747	0.8001232	FALSE
TC07000328.hg.1	ENSG00000146648	0.712747	0.8001232	FALSE
TC10001663.hg.1	ENSG00000119927	0.712747	0.8001232	FALSE
TC0Y000351.hg.1	ENSG00000129824	0.712747	0.8001232	FALSE
TC03001823.hg.1	ENSG00000114107;	0.7148292	0.8010952	FALSE
TC0X000038.hg.1	ENSG00000073464;	0.7159538	0.8019357	FALSE
TC02001100.hg.1;	ENSG00000138448;	0.7159538	0.8019357	FALSE
TC05000243.hg.1	ENSG00000095015;	0.7159538	0.8019357	FALSE
TC02001031.hg.1	ENSG00000152256;	0.7188279	0.8046493	FALSE
TC01000685.hg.1	ENSG00000162409;	0.719038	0.8046493	FALSE
TC01001112.hg.1;	ENSG00000131778;	0.7191282	0.8046493	FALSE

TC01001112.hg.1;	ENSG00000131778;	0.7206468	0.8061377	FALSE
TC01000728.hg.1	ENSG00000162433;	0.7216372	0.8069181	FALSE
TC01000785.hg.1	ENSG00000154027;	0.7218157	0.8069181	FALSE
TC03000214.hg.1	ENSG00000168036;	0.7218157	0.8069181	FALSE
TC05000840.hg.1	ENSG00000123643;	0.7243175	0.8075559	FALSE
TC08001659.hg.1	ENSG00000104419;	0.7243175	0.8075559	FALSE
TC19001103.hg.1	ENSG00000125730	0.724457	0.8075559	FALSE
TC18000552.hg.1	ENSG00000197563	0.724457	0.8075559	FALSE
TC02000466.hg.1	ENSG00000159399	0.724457	0.8075559	FALSE
TC11000182.hg.1	ENSG00000148926	0.724457	0.8075559	FALSE
TC02001031.hg.1	ENSG00000152256	0.724457	0.8075559	FALSE
TC20000876.hg.1	ENSG00000124107	0.724457	0.8075559	FALSE
TC07000768.hg.1	ENSG00000174697	0.724457	0.8075559	FALSE
TC07000768.hg.1	ENSG00000174697	0.724457	0.8075559	FALSE
TC10001517.hg.1	ENSG00000148677	0.724457	0.8075559	FALSE
TC13000845.hg.1	ENSG00000102452	0.724457	0.8075559	FALSE
TC11000404.hg.1	ENSG00000134574	0.724457	0.8075559	FALSE
TC01001704.hg.1	ENSG00000163531;	0.7251215	0.8075559	FALSE
TC01003211.hg.1;	ENSG00000143387;	0.7286682	0.8112948	FALSE
TC01000728.hg.1	ENSG00000162433;	0.7290034	0.811457	FALSE
TC01000685.hg.1	ENSG00000162409;	0.7322642	0.8147885	FALSE
TC05000840.hg.1	ENSG00000123643;	0.7324722	0.8147885	FALSE
TC05000159.hg.1	ENSG00000168685;	0.7324722	0.8147885	FALSE
TC01001112.hg.1;	ENSG00000131778;	0.7350474	0.8161702	FALSE
TC09000388.hg.1	ENSG00000148053	0.7356903	0.8161702	FALSE
TC07001534.hg.1	ENSG00000127946	0.7356903	0.8161702	FALSE
TC05001519.hg.1	ENSG00000113273	0.7356903	0.8161702	FALSE
TC20000942.hg.1	ENSG00000101096	0.7356903	0.8161702	FALSE
TC04001084.hg.1	ENSG00000109819	0.7356903	0.8161702	FALSE
TC02002624.hg.1	ENSG00000115415	0.7356903	0.8161702	FALSE
TC09000388.hg.1	ENSG00000148053	0.7356903	0.8161702	FALSE
TC09000388.hg.1	ENSG00000148053	0.7356903	0.8161702	FALSE
TC10001711.hg.1	ENSG00000066468	0.7356903	0.8161702	FALSE
TC09001585.hg.1	ENSG00000173611	0.7356903	0.8161702	FALSE
TC07000768.hg.1	ENSG00000174697	0.7356903	0.8161702	FALSE
TC10001711.hg.1	ENSG00000066468	0.7356903	0.8161702	FALSE
TC02002624.hg.1	ENSG00000115415	0.7356903	0.8161702	FALSE
TC02000041.hg.1	ENSG00000115738;	0.7367647	0.8161702	FALSE
TC01000904.hg.1	ENSG00000162692;	0.7367647	0.8161702	FALSE
TC01000728.hg.1	ENSG00000162433;	0.7394894	0.8188707	FALSE
TC06001027.hg.1	ENSG00000118503;	0.7404209	0.8194782	FALSE
TC06000087.hg.1	ENSG00000078401;	0.7404209	0.8194782	FALSE
TC15001110.hg.1;	ENSG00000104067;	0.7404209	0.8194782	FALSE
TC05001385.hg.1	ENSG00000145632;	0.741975	0.8206679	FALSE
TC01003211.hg.1;	ENSG00000143387;	0.741975	0.8206679	FALSE
TC01001607.hg.1	ENSG00000116711;l	0.7425483	0.8209838	FALSE
TC17001462.hg.1	ENSG00000131747	0.7464664	0.8239226	FALSE
TC02002823.hg.1	ENSG00000135919	0.7464664	0.8239226	FALSE
TC14000819.hg.1	ENSG00000185100	0.7464664	0.8239226	FALSE
TC17001462.hg.1	ENSG00000131747	0.7464664	0.8239226	FALSE
TC02002808.hg.1	ENSG00000116106	0.7464664	0.8239226	FALSE
TC09001585.hg.1	ENSG00000173611	0.7464664	0.8239226	FALSE
TC04000313.hg.1	ENSG00000157404	0.7464664	0.8239226	FALSE
TC18000047.hg.1	ENSG00000168461	0.7464664	0.8239226	FALSE
TC06000087.hg.1	ENSG00000078401	0.7464664	0.8239226	FALSE
TC02001031.hg.1	ENSG00000152256;	0.7471309	0.8239226	FALSE
TC09000388.hg.1	ENSG00000148053;	0.7481669	0.8247194	FALSE
TC01002166.hg.1	ENSG00000116285;	0.7481669	0.8247194	FALSE

TC02001031.hg.1	ENSG00000152256;	0.7484314	0.8247194	FALSE
TC01000728.hg.1	ENSG00000162433;	0.7487583	0.8248673	FALSE
TC02000270.hg.1	ENSG00000152527;	0.7510204	0.8270339	FALSE
TC01003211.hg.1;	ENSG00000143387;	0.7511114	0.8270339	FALSE
TC01001688.hg.1	ENSG00000058668;	0.753825	0.8298083	FALSE
TC01000785.hg.1	ENSG00000154027;	0.7547578	0.8306217	FALSE
TC02000281.hg.1	ENSG00000116016;	0.7552306	0.8309283	FALSE
TC02001628.hg.1	ENSG00000084674;	0.7557135	0.8310325	FALSE
TC01001805.hg.1	ENSG00000092969;	0.7557135	0.8310325	FALSE
TC02000041.hg.1	ENSG00000115738;	0.7557135	0.8310325	FALSE
TC09000358.hg.1	ENSG00000135069	0.7568037	0.8311638	FALSE
TC03000862.hg.1	ENSG00000163661	0.7568037	0.8311638	FALSE
TC03001071.hg.1	ENSG00000114315	0.7568037	0.8311638	FALSE
TC12000803.hg.1	ENSG00000166598	0.7568037	0.8311638	FALSE
TC01000703.hg.1	ENSG00000132849	0.7568037	0.8311638	FALSE
TC10000663.hg.1	ENSG00000095596	0.7568037	0.8311638	FALSE
TC10000406.hg.1	ENSG00000138336	0.7568037	0.8311638	FALSE
TC01001112.hg.1;	ENSG00000131778;	0.7613622	0.835313	FALSE
TC01001112.hg.1;	ENSG00000131778;	0.7615698	0.8353268	FALSE
TC01001805.hg.1	ENSG00000092969;	0.763064	0.8367513	FALSE
TC03000013.hg.1	ENSG00000150995;	0.7647178	0.83835	FALSE
TC09000388.hg.1	ENSG00000148053	0.7667201	0.8398053	FALSE
TC08000508.hg.1	ENSG00000104435	0.7667201	0.8398053	FALSE
TC01000785.hg.1	ENSG00000154027	0.7667201	0.8398053	FALSE
TC17000015.hg.1	ENSG00000132386	0.7667201	0.8398053	FALSE
TC01000904.hg.1	ENSG00000162692;	0.7670261	0.8398053	FALSE
TC03000013.hg.1	ENSG00000150995;	0.770222	0.8426579	FALSE
TC02002808.hg.1	ENSG00000116106;	0.770222	0.8426579	FALSE
TC03000013.hg.1	ENSG00000150995;	0.770222	0.8426579	FALSE
TC03000013.hg.1	ENSG00000150995;	0.770222	0.8426579	FALSE
TC09000388.hg.1	ENSG00000148053;	0.770222	0.8426579	FALSE
TC01000783.hg.1	ENSG00000117069;	0.7708161	0.8426618	FALSE
TC02000286.hg.1	ENSG00000171150;	0.7729705	0.8448013	FALSE
TC01001112.hg.1;	ENSG00000131778;	0.7742666	0.8460018	FALSE
TC0Y000351.hg.1	ENSG00000129824	0.7762328	0.8468196	FALSE
TC05000243.hg.1	ENSG00000095015	0.7762328	0.8468196	FALSE
TC07000768.hg.1	ENSG00000174697	0.7762328	0.8468196	FALSE
TC07000768.hg.1	ENSG00000174697	0.7762328	0.8468196	FALSE
TC07000137.hg.1	ENSG00000136244	0.7762328	0.8468196	FALSE
TC07000137.hg.1	ENSG00000136244	0.7762328	0.8468196	FALSE
TC05000243.hg.1	ENSG00000095015	0.7762328	0.8468196	FALSE
TC05000243.hg.1	ENSG00000095015	0.7762328	0.8468196	FALSE
TC06000087.hg.1	ENSG00000078401	0.7762328	0.8468196	FALSE
TC10000406.hg.1	ENSG00000138336;	0.7771909	0.8468196	FALSE
TC02000432.hg.1	ENSG00000135636;	0.7771909	0.8468196	FALSE
TC02000432.hg.1	ENSG00000135636;	0.7771909	0.8468196	FALSE
TC01000785.hg.1	ENSG00000154027;	0.7776165	0.8468523	FALSE
TC04002931.hg.1	ENSG00000134853;	0.7787901	0.8476991	FALSE
TC01000685.hg.1	ENSG00000162409;	0.7787901	0.8476991	FALSE
TC02001628.hg.1	ENSG00000084674;	0.7787901	0.8476991	FALSE
TC01000728.hg.1	ENSG00000162433;	0.7803238	0.8489368	FALSE
TC01001112.hg.1;	ENSG00000131778;	0.7808938	0.8492331	FALSE
TC01001112.hg.1;	ENSG00000131778;	0.7808938	0.8492331	FALSE
TC01000728.hg.1	ENSG00000162433;	0.7815108	0.8495804	FALSE
TC07000235.hg.1	ENSG00000011426;	0.7839741	0.8510556	FALSE
TC03000013.hg.1	ENSG00000150995;	0.7839741	0.8510556	FALSE
TC19001250.hg.1	ENSG00000074181;	0.7839741	0.8510556	FALSE
TC08001659.hg.1	ENSG00000104419;	0.7839741	0.8510556	FALSE

TC03001884.hg.1	ENSG00000047457;	0.7839741	0.8510556	FALSE
TC01000728.hg.1	ENSG00000162433;	0.7841697	0.8510556	FALSE
TC01003211.hg.1;	ENSG00000143387;	0.7853071	0.8510556	FALSE
TC03000214.hg.1	ENSG00000168036	0.7853581	0.8510556	FALSE
TC07001630.hg.1	ENSG00000070669	0.7853581	0.8510556	FALSE
TC14000819.hg.1	ENSG00000185100	0.7853581	0.8510556	FALSE
TC12003293.hg.1	ENSG00000180818	0.7853581	0.8510556	FALSE
TC14001141.hg.1	ENSG00000197930	0.7853581	0.8510556	FALSE
TC03000214.hg.1	ENSG00000168036	0.7853581	0.8510556	FALSE
TC09000388.hg.1	ENSG00000148053	0.7853581	0.8510556	FALSE
TC09000388.hg.1	ENSG00000148053	0.7853581	0.8510556	FALSE
TC01003778.hg.1	ENSG00000076356;	0.7860485	0.8510556	FALSE
TC02000041.hg.1	ENSG00000115738;	0.7883088	0.853287	FALSE
TC02000416.hg.1	ENSG00000116005;	0.7890783	0.853904	FALSE
TC01003211.hg.1;	ENSG00000143387;	0.7900612	0.8547515	FALSE
TC01001736.hg.1	ENSG00000196352;	0.7905753	0.8548757	FALSE
TC21000241.hg.1	ENSG00000197381;	0.7905753	0.8548757	FALSE
TC03000131.hg.1	ENSG00000077092;	0.7905753	0.8548757	FALSE
TC08000508.hg.1	ENSG00000104435	0.7941118	0.8581579	FALSE
TC09000388.hg.1	ENSG00000148053	0.7941118	0.8581579	FALSE
TC01000785.hg.1	ENSG00000154027;	0.7955155	0.859241	FALSE
TC05001933.hg.1	ENSG00000113721;	0.7955155	0.859241	FALSE
TC01001624.hg.1	ENSG00000116741;	0.7962902	0.8597524	FALSE
TC01001112.hg.1;	ENSG00000131778;	0.7971364	0.8604491	FALSE
TC02001031.hg.1	ENSG00000152256;	0.7983199	0.8615093	FALSE
TC01001624.hg.1	ENSG00000116741;	0.799274	0.8621049	FALSE
TC02000416.hg.1	ENSG00000116005;	0.799274	0.8621049	FALSE
TC01001112.hg.1;	ENSG00000131778;	0.7994759	0.8621049	FALSE
TC01003211.hg.1;	ENSG00000143387;	0.8022878	0.8639694	FALSE
TC22000259.hg.1	ENSG00000100292	0.802509	0.8639694	FALSE
TC01000783.hg.1	ENSG00000117069	0.802509	0.8639694	FALSE
TC10001711.hg.1	ENSG00000066468	0.802509	0.8639694	FALSE
TC02000432.hg.1	ENSG00000135636	0.802509	0.8639694	FALSE
TC02000891.hg.1	ENSG00000150540	0.802509	0.8639694	FALSE
TC03003365.hg.1	ENSG00000176945;	0.8026177	0.8639694	FALSE
TC05001385.hg.1	ENSG00000145632;	0.8032457	0.8643195	FALSE
TC02000432.hg.1	ENSG00000135636;	0.8032457	0.8643195	FALSE
TC02000416.hg.1	ENSG00000116005;	0.8038284	0.8645182	FALSE
TC01000728.hg.1	ENSG00000162433;	0.8039353	0.8645182	FALSE
TC06001027.hg.1	ENSG00000118503;	0.8045559	0.8649683	FALSE
TC02000219.hg.1	ENSG00000049323;	0.8060695	0.866378	FALSE
TC02000041.hg.1	ENSG00000115738;	0.8100418	0.8696946	FALSE
TC06004150.hg.1	ENSG00000096060	0.8105642	0.8696946	FALSE
TC14001141.hg.1	ENSG00000197930	0.8105642	0.8696946	FALSE
TC02000466.hg.1	ENSG00000159399	0.8105642	0.8696946	FALSE
TC10001711.hg.1	ENSG00000066468	0.8105642	0.8696946	FALSE
TC03002111.hg.1;	ENSG00000205835;	0.8105917	0.8696946	FALSE
TC01001090.hg.1	ENSG00000117289;	0.8105917	0.8696946	FALSE
TC01001090.hg.1	ENSG00000117289;	0.8107804	0.8696946	FALSE
TC01000685.hg.1	ENSG00000162409;	0.8117976	0.8705676	FALSE
TC01000728.hg.1	ENSG00000162433;	0.8122622	0.8708476	FALSE
TC02000416.hg.1	ENSG00000116005;	0.812763	0.8711663	FALSE
TC07001552.hg.1	ENSG00000187391;	0.8152307	0.8735927	FALSE
TC01000728.hg.1	ENSG00000162433;	0.8176681	0.8753288	FALSE
TC01002166.hg.1	ENSG00000116285;	0.8180122	0.8753288	FALSE
TC07000137.hg.1	ENSG00000136244	0.8182914	0.8753288	FALSE
TC07000328.hg.1	ENSG00000146648	0.8182914	0.8753288	FALSE
TC16001225.hg.1	ENSG00000181019	0.8182914	0.8753288	FALSE

TC04002948.hg.1	ENSG00000137463	0.8182914	0.8753288	FALSE
TC07001898.hg.1	ENSG00000182158	0.8182914	0.8753288	FALSE
TC07000768.hg.1	ENSG00000174697	0.8182914	0.8753288	FALSE
TC12001890.hg.1	ENSG00000017427	0.8182914	0.8753288	FALSE
TC04001084.hg.1	ENSG00000109819	0.8182914	0.8753288	FALSE
TC01001090.hg.1	ENSG00000117289;	0.8191	0.8753288	FALSE
TC05000159.hg.1	ENSG00000168685	0.8257037	0.8812117	FALSE
TC05000159.hg.1	ENSG00000168685	0.8257037	0.8812117	FALSE
TC03000214.hg.1	ENSG00000168036	0.8257037	0.8812117	FALSE
TC05000243.hg.1	ENSG00000095015	0.8257037	0.8812117	FALSE
TC05000243.hg.1	ENSG00000095015	0.8257037	0.8812117	FALSE
TC09000038.hg.1	ENSG00000197646	0.8257037	0.8812117	FALSE
TC05000159.hg.1	ENSG00000168685	0.8257037	0.8812117	FALSE
TC0Y000351.hg.1	ENSG00000129824	0.8257037	0.8812117	FALSE
TC01001090.hg.1	ENSG00000117289;	0.8264576	0.8812117	FALSE
TC01001112.hg.1;	ENSG00000131778;	0.826977	0.8814362	FALSE
TC01001112.hg.1;	ENSG00000131778;	0.826977	0.8814362	FALSE
TC01000728.hg.1	ENSG00000162433;	0.828191	0.8823894	FALSE
TC07000235.hg.1	ENSG00000011426;	0.8283866	0.8823894	FALSE
TC01001112.hg.1;	ENSG00000131778;	0.8303454	0.8842559	FALSE
TC01001624.hg.1	ENSG00000116741;	0.8318808	0.8856706	FALSE
TC01000685.hg.1	ENSG00000162409;	0.8321376	0.8856732	FALSE
TC01001112.hg.1;	ENSG00000131778;	0.8324288	0.8856732	FALSE
TC02000041.hg.1	ENSG00000115738	0.8328142	0.8856732	FALSE
TC02002714.hg.1	ENSG00000163251	0.8328142	0.8856732	FALSE
TC05000159.hg.1	ENSG00000168685	0.8328142	0.8856732	FALSE
TC04002948.hg.1	ENSG00000137463	0.8328142	0.8856732	FALSE
TC02000416.hg.1	ENSG00000116005;	0.8352109	0.8876708	FALSE
TC01001090.hg.1	ENSG00000117289;	0.8364346	0.8887507	FALSE
TC01001736.hg.1	ENSG00000196352;	0.8372584	0.8894053	FALSE
TC06004141.hg.1	ENSG00000112096	0.839635	0.8909352	FALSE
TC0X000943.hg.1	ENSG00000198947	0.839635	0.8909352	FALSE
TC07000852.hg.1	ENSG00000181072	0.839635	0.8909352	FALSE
TC02002823.hg.1	ENSG00000135919	0.839635	0.8909352	FALSE
TC04002948.hg.1	ENSG00000137463	0.839635	0.8909352	FALSE
TC01001112.hg.1;	ENSG00000131778;	0.8399473	0.8909352	FALSE
TC03001823.hg.1	ENSG00000114107;	0.8412302	0.8919644	FALSE
TC03001823.hg.1	ENSG00000114107;	0.8412302	0.8919644	FALSE
TC22000259.hg.1	ENSG00000100292	0.8461779	0.8962117	FALSE
TC02002143.hg.1	ENSG00000204634	0.8461779	0.8962117	FALSE
TC02002143.hg.1	ENSG00000204634	0.8461779	0.8962117	FALSE
TC06001027.hg.1	ENSG00000118503	0.8461779	0.8962117	FALSE
TC02002143.hg.1	ENSG00000204634	0.8461779	0.8962117	FALSE
TC10000811.hg.1	ENSG00000150594	0.8461779	0.8962117	FALSE
TC06004141.hg.1	ENSG00000112096	0.8461779	0.8962117	FALSE
TC01002616.hg.1	ENSG00000117461;	0.8521906	0.9015209	FALSE
TC05000159.hg.1	ENSG00000168685	0.8524543	0.9015209	FALSE
TC20000942.hg.1	ENSG00000101096	0.8524543	0.9015209	FALSE
TC07000328.hg.1	ENSG00000146648	0.8524543	0.9015209	FALSE
TC02000416.hg.1	ENSG00000116005;	0.8549538	0.9036063	FALSE
TC02000416.hg.1	ENSG00000116005;	0.8549538	0.9036063	FALSE
TC01003629.hg.1	ENSG00000116679;	0.8569069	0.9053352	FALSE
TC05000243.hg.1	ENSG00000095015	0.8584749	0.9067681	FALSE
TC05000165.hg.1	ENSG00000079215;	0.8614405	0.9096761	FALSE
TC04000101.hg.1	ENSG00000249811;	0.8637447	0.9118844	FALSE
TC19001103.hg.1	ENSG00000125730	0.8642502	0.9119682	FALSE
TC05000159.hg.1	ENSG00000168685	0.8642502	0.9119682	FALSE
TC22000259.hg.1	ENSG00000100292	0.8642502	0.9119682	FALSE

TC05001385.hg.1	ENSG00000145632;	0.8695023	0.9165715	FALSE
TC01003629.hg.1	ENSG00000116679;	0.8697067	0.9165715	FALSE
TC19001103.hg.1	ENSG00000125730	0.8697902	0.9165715	FALSE
TC18000552.hg.1	ENSG00000197563	0.8697902	0.9165715	FALSE
TC22000259.hg.1	ENSG00000100292	0.8697902	0.9165715	FALSE
TC07000137.hg.1	ENSG00000136244	0.8697902	0.9165715	FALSE
TC02001100.hg.1;	ENSG00000138448;	0.8735124	0.9199278	FALSE
TC05001385.hg.1	ENSG00000145632;	0.8749965	0.9208116	FALSE
TC01000728.hg.1	ENSG00000162433;	0.8750376	0.9208116	FALSE
TC07000137.hg.1	ENSG00000136244	0.8751044	0.9208116	FALSE
TC10001663.hg.1	ENSG00000119927	0.8751044	0.9208116	FALSE
TC01000115.hg.1;	ENSG00000171621;	0.8788584	0.9244208	FALSE
TC03001071.hg.1	ENSG00000114315	0.880202	0.9256067	FALSE
TC04001084.hg.1	ENSG00000109819;	0.8818783	0.9271417	FALSE
TC07001562.hg.1	ENSG00000019991;	0.8822433	0.9272489	FALSE
TC01000703.hg.1	ENSG00000132849;	0.8824135	0.9272489	FALSE
TC17001462.hg.1	ENSG00000131747	0.8850919	0.9292651	FALSE
TC05000243.hg.1	ENSG00000095015	0.8850919	0.9292651	FALSE
TC12002155.hg.1	ENSG00000176915	0.8850919	0.9292651	FALSE
TC09000038.hg.1	ENSG00000197646	0.8850919	0.9292651	FALSE
TC22000259.hg.1	ENSG00000100292	0.8850919	0.9292651	FALSE
TC09000038.hg.1	ENSG00000197646	0.8850919	0.9292651	FALSE
TC05000243.hg.1	ENSG00000095015;	0.8860977	0.9294093	FALSE
TC08001659.hg.1	ENSG00000104419;	0.8860977	0.9294093	FALSE
TC01001090.hg.1	ENSG00000117289;	0.887267	0.9302939	FALSE
TC02000511.hg.1	ENSG00000168906	0.8897824	0.9323604	FALSE
TC05001933.hg.1	ENSG00000113721	0.8897824	0.9323604	FALSE
TC05000701.hg.1	ENSG00000120738	0.8897824	0.9323604	FALSE
TC05000701.hg.1	ENSG00000120738	0.8897824	0.9323604	FALSE
TC01001112.hg.1;	ENSG00000131778;	0.8907853	0.9325313	FALSE
TC02000416.hg.1	ENSG00000116005;	0.8907958	0.9325313	FALSE
TC01000115.hg.1;	ENSG00000171621;	0.8909257	0.9325313	FALSE
TC06001027.hg.1	ENSG00000118503;	0.8933699	0.9347468	FALSE
TC05000165.hg.1	ENSG00000079215;	0.8933699	0.9347468	FALSE
TC18000097.hg.1	ENSG00000101773	0.8942818	0.9350153	FALSE
TC10001663.hg.1	ENSG00000119927	0.8942818	0.9350153	FALSE
TC01000685.hg.1	ENSG00000162409	0.8942818	0.9350153	FALSE
TC10001663.hg.1	ENSG00000119927	0.8942818	0.9350153	FALSE
TC14000067.hg.1	ENSG00000181784;	0.896842	0.93712	FALSE
TC01000728.hg.1	ENSG00000162433;	0.8975685	0.9376503	FALSE
TC18000097.hg.1	ENSG00000101773	0.8985977	0.9382675	FALSE
TC05001519.hg.1	ENSG00000113273	0.8985977	0.9382675	FALSE
TC05000701.hg.1	ENSG00000120738	0.8985977	0.9382675	FALSE
TC04000432.hg.1	ENSG00000156234;	0.9002087	0.9394913	FALSE
TC01000685.hg.1	ENSG00000162409;	0.9015188	0.9406292	FALSE
TC01001624.hg.1	ENSG00000116741;	0.9026982	0.9416303	FALSE
TC08000255.hg.1	ENSG00000157168;	0.9033844	0.9421166	FALSE
TC12000531.hg.1	ENSG00000166986	0.9067089	0.9450749	FALSE
TC06001027.hg.1	ENSG00000118503	0.9067089	0.9450749	FALSE
TC06001027.hg.1	ENSG00000118503	0.9067089	0.9450749	FALSE
TC01000394.hg.1	ENSG00000159023;	0.9071042	0.9450749	FALSE
TC05001385.hg.1	ENSG00000145632;	0.9097038	0.9475528	FALSE
TC0X000207.hg.1	ENSG00000189221	0.9105183	0.9475659	FALSE
TC07000768.hg.1	ENSG00000174697	0.9105183	0.9475659	FALSE
TC06000004.hg.1	ENSG00000112679	0.9105183	0.9475659	FALSE
TC10001517.hg.1	ENSG00000148677	0.9105183	0.9475659	FALSE
TC01001090.hg.1	ENSG00000117289;	0.9108231	0.9475659	FALSE
TC04000253.hg.1	ENSG00000154277;	0.9140234	0.9505881	FALSE

TC0Y000351.hg.1	ENSG00000129824	0.9141722	0.9505881	FALSE
TC07001562.hg.1	ENSG00000019991;	0.9148664	0.9509635	FALSE
TC07001562.hg.1	ENSG00000019991;	0.9148664	0.9509635	FALSE
TC01000783.hg.1	ENSG00000117069;	0.9161876	0.9515819	FALSE
TC01003211.hg.1;	ENSG00000143387;	0.9165113	0.9515819	FALSE
TC02000416.hg.1	ENSG00000116005;	0.9165328	0.9515819	FALSE
TC02000416.hg.1	ENSG00000116005;	0.9165328	0.9515819	FALSE
TC01001624.hg.1	ENSG00000116741;	0.9166839	0.9515819	FALSE
TC02002823.hg.1	ENSG00000135919	0.9176772	0.9521513	FALSE
TC12000531.hg.1	ENSG00000166986	0.9176772	0.9521513	FALSE
TC12000531.hg.1	ENSG00000166986	0.9176772	0.9521513	FALSE
TC01002847.hg.1	ENSG00000117228;	0.9199917	0.9540902	FALSE
TC01000115.hg.1;	ENSG00000171621;	0.9222857	0.9562376	FALSE
TC05000159.hg.1	ENSG00000168685;	0.923672	0.9574431	FALSE
TC05001519.hg.1	ENSG00000113273	0.9242642	0.9577091	FALSE
TC14000353.hg.1	ENSG00000165617	0.9242642	0.9577091	FALSE
TC01002578.hg.1	ENSG00000117394;	0.9255253	0.9586601	FALSE
TC01002847.hg.1	ENSG00000117228;	0.9257419	0.9586601	FALSE
TC02000416.hg.1	ENSG00000116005;	0.9286678	0.9613413	FALSE
TC07000137.hg.1	ENSG00000136244;	0.9286678	0.9613413	FALSE
TC01001805.hg.1	ENSG00000092969;	0.9290517	0.96139	FALSE
TC06001027.hg.1	ENSG00000118503	0.9303249	0.9623586	FALSE
TC08001590.hg.1	ENSG00000156804	0.9303249	0.9623586	FALSE
TC01000785.hg.1	ENSG00000154027;	0.9310407	0.9623654	FALSE
TC02001100.hg.1;	ENSG00000138448;	0.9310484	0.9623654	FALSE
TC01000728.hg.1	ENSG00000162433;	0.9311537	0.9623654	FALSE
TC04000432.hg.1	ENSG00000156234;	0.9313431	0.9623654	FALSE
TC19001103.hg.1	ENSG00000125730	0.9331711	0.9640216	FALSE
TC05001385.hg.1	ENSG00000145632;	0.9367394	0.9674744	FALSE
TC03000214.hg.1	ENSG00000168036;	0.9380105	0.9683789	FALSE
TC04000643.hg.1	ENSG00000164070;	0.9384508	0.9683789	FALSE
TC19001103.hg.1	ENSG00000125730	0.93852	0.9683789	FALSE
TC11000404.hg.1	ENSG00000134574	0.93852	0.9683789	FALSE
TC12000076.hg.1	ENSG00000010278	0.93852	0.9683789	FALSE
TC01002954.hg.1	ENSG00000134243;	0.9398527	0.9692123	FALSE
TC02000511.hg.1;	ENSG00000168906;	0.9401201	0.9692123	FALSE
TC05001385.hg.1	ENSG00000145632;	0.9401201	0.9692123	FALSE
TC03000214.hg.1	ENSG00000168036;	0.9408515	0.9696161	FALSE
TC06004141.hg.1	ENSG00000112096	0.9434413	0.9720457	FALSE
TC01001090.hg.1	ENSG00000117289;	0.9436631	0.9720457	FALSE
TC11001948.hg.1	ENSG00000175592	0.9457524	0.9739635	FALSE
TC05001481.hg.1	ENSG00000171617;	0.946726	0.9747316	FALSE
TC01001736.hg.1	ENSG00000196352;	0.9475599	0.9752383	FALSE
TC04002948.hg.1	ENSG00000137463;	0.9475599	0.9752383	FALSE
TC10001327.hg.1	ENSG00000122877	0.9479692	0.9753079	FALSE
TC01000785.hg.1	ENSG00000154027;	0.9484894	0.9756086	FALSE
TC17001462.hg.1	ENSG00000131747	0.9500956	0.977026	FALSE
TC03000248.hg.1	ENSG00000144791;	0.9506272	0.9773379	FALSE
TC01001736.hg.1	ENSG00000196352;	0.9510568	0.9775448	FALSE
TC19001103.hg.1	ENSG00000125730	0.9521352	0.9783009	FALSE
TC05000243.hg.1	ENSG00000095015	0.9521352	0.9783009	FALSE
TC02000511.hg.1;	ENSG00000168906;	0.9531377	0.9789785	FALSE
TC04000313.hg.1	ENSG00000157404	0.9540915	0.9792639	FALSE
TC02002714.hg.1	ENSG00000163251	0.9540915	0.9792639	FALSE
TC22000259.hg.1	ENSG00000100292	0.9540915	0.9792639	FALSE
TC10000406.hg.1	ENSG00000138336;	0.9543305	0.9792639	FALSE
TC04000313.hg.1	ENSG00000157404	0.955968	0.9804742	FALSE
TC04000313.hg.1	ENSG00000157404	0.955968	0.9804742	FALSE

TC19001103.hg.1	ENSG00000125730	0.955968	0.9804742	FALSE
TC01001090.hg.1	ENSG00000117289;	0.9575892	0.9816147	FALSE
TC05000165.hg.1	ENSG00000079215	0.9577679	0.9816147	FALSE
TC0Y000351.hg.1	ENSG00000129824	0.9594944	0.9831487	FALSE
TC05001385.hg.1	ENSG00000145632;	0.9606689	0.9840212	FALSE
TC01000785.hg.1	ENSG00000154027;	0.960843	0.9840212	FALSE
TC17001462.hg.1	ENSG00000131747	0.9611503	0.9840212	FALSE
TC04000313.hg.1	ENSG00000157404	0.9611503	0.9840212	FALSE
TC01002578.hg.1	ENSG00000117394;	0.9621864	0.9847286	FALSE
TC01000785.hg.1	ENSG00000154027;	0.9632082	0.9855388	FALSE
TC01001112.hg.1	ENSG00000131778	0.9642622	0.9863815	FALSE
TC02000891.hg.1	ENSG00000150540;	0.9668146	0.9883654	FALSE
TC01000785.hg.1	ENSG00000154027;	0.9671223	0.9883654	FALSE
TC0Y000351.hg.1	ENSG00000129824	0.9671251	0.9883654	FALSE
TC02001100.hg.1	ENSG00000138448	0.9671251	0.9883654	FALSE
TC07000768.hg.1	ENSG00000174697	0.9671251	0.9883654	FALSE
TC17001462.hg.1	ENSG00000131747;	0.9677926	0.9885755	FALSE
TC12002066.hg.1	ENSG00000158104	0.9684696	0.989031	FALSE
TC01003172.hg.1	ENSG00000203818;	0.9704699	0.9908375	FALSE
TC17001462.hg.1	ENSG00000131747;	0.9720292	0.9917579	FALSE
TC07000852.hg.1	ENSG00000181072	0.9721822	0.9917579	FALSE
TC12001807.hg.1	ENSG00000133639	0.9721822	0.9917579	FALSE
TC12001807.hg.1	ENSG00000133639	0.9721822	0.9917579	FALSE
TC02001100.hg.1	ENSG00000138448	0.9721822	0.9917579	FALSE
TC07000852.hg.1	ENSG00000181072	0.9733201	0.9921434	FALSE
TC01000703.hg.1	ENSG00000132849;	0.9733713	0.9921434	FALSE
TC01003172.hg.1	ENSG00000203818;	0.9739407	0.9924203	FALSE
TC02000448.hg.1	ENSG00000163017;	0.9743378	0.9924203	FALSE
TC01000685.hg.1	ENSG00000162409;	0.9743384	0.9924203	FALSE
TC01000685.hg.1	ENSG00000162409;	0.974668	0.99252	FALSE
TC07000852.hg.1	ENSG00000181072	0.9754583	0.9929704	FALSE
TC07000852.hg.1	ENSG00000181072	0.9754583	0.9929704	FALSE
TC07000852.hg.1	ENSG00000181072	0.9764624	0.993402	FALSE
TC0Y000351.hg.1	ENSG00000129824	0.9764624	0.993402	FALSE
TC07000852.hg.1	ENSG00000181072	0.9764624	0.993402	FALSE
TC03000214.hg.1	ENSG00000168036;	0.977383	0.9938662	FALSE
TC11000174.hg.1	ENSG00000166483	0.9783491	0.9943762	FALSE
TC03000248.hg.1	ENSG00000144791	0.9783491	0.9943762	FALSE
TC11000174.hg.1	ENSG00000166483	0.9783491	0.9943762	FALSE
TC01001112.hg.1;	ENSG00000131778;	0.9796572	0.9951952	FALSE
TC01000685.hg.1	ENSG00000162409;	0.979958	0.9951952	FALSE
TC02000511.hg.1	ENSG00000168906	0.9800848	0.9951952	FALSE
TC06001027.hg.1	ENSG00000118503;	0.9803999	0.9952791	FALSE
TC01003172.hg.1	ENSG00000203818;	0.981089	0.9957425	FALSE
TC03000248.hg.1	ENSG00000144791	0.9816815	0.9961078	FALSE
TC01000685.hg.1	ENSG00000162409;	0.9819491	0.9961431	FALSE
TC12000803.hg.1	ENSG00000166598;	0.9830181	0.9969913	FALSE
TC01003172.hg.1	ENSG00000203818;	0.9847556	0.998355	FALSE
TC12001405.hg.1	ENSG00000139174	0.9851362	0.998355	FALSE
TC01000115.hg.1;	ENSG00000171621;	0.9852478	0.998355	FALSE
TC01003172.hg.1	ENSG00000203818;	0.9852954	0.998355	FALSE
TC09000455.hg.1	ENSG00000158079	0.9868882	0.9997323	FALSE
TC18000097.hg.1	ENSG00000101773	0.9874251	1	FALSE
TC01003172.hg.1	ENSG00000203818;	0.9881646	1	FALSE
TC10000874.hg.1	ENSG00000203805	0.9889077	1	FALSE
TC06000945.hg.1	ENSG00000152661	0.9893621	1	FALSE
TC18000097.hg.1	ENSG00000101773	0.9902157	1	FALSE
TC12002155.hg.1	ENSG00000176915	0.9902157	1	FALSE

TC0Y000351.hg.1	ENSG00000129824	0.9902157	1	FALSE
TC12002155.hg.1	ENSG00000176915	0.991001	1	FALSE
TC01003172.hg.1	ENSG00000203818;	0.9911297	1	FALSE
TC17001462.hg.1	ENSG00000131747;	0.9911556	1	FALSE
TC01003629.hg.1	ENSG00000116679;	0.9911556	1	FALSE
TC01001090.hg.1	ENSG00000117289;	0.9914466	1	FALSE
TC01003172.hg.1	ENSG00000203818;	0.991473	1	FALSE
TC06000408.hg.1	ENSG00000223865	0.9923878	1	FALSE
TC18000097.hg.1	ENSG00000101773	0.9923878	1	FALSE
TC06000408.hg.1	ENSG00000223865	0.9929989	1	FALSE
TC01001090.hg.1	ENSG00000117289;	0.9930557	1	FALSE
TC01003172.hg.1	ENSG00000203818;	0.9931003	1	FALSE
TC17001462.hg.1	ENSG00000131747	0.9935611	1	FALSE
TC02001100.hg.1;	ENSG00000138448;	0.9936553	1	FALSE
TC01002954.hg.1	ENSG00000134243	0.993825	1	FALSE
TC02000511.hg.1	ENSG00000168906	0.994321	1	FALSE
TC06000408.hg.1	ENSG00000223865	0.9955826	1	FALSE
TC05001385.hg.1	ENSG00000145632;	0.9966005	1	FALSE
TC03001823.hg.1	ENSG00000114107;	0.99665	1	FALSE
TC12001807.hg.1	ENSG00000133639	0.9968404	1	FALSE
TC12002155.hg.1	ENSG00000176915	0.99697	1	FALSE
TC12000531.hg.1	ENSG00000166986	0.9974377	1	FALSE
TC04000313.hg.1	ENSG00000157404	0.9974377	1	FALSE
TC02001100.hg.1;	ENSG00000138448;	0.9977554	1	FALSE
TC02001100.hg.1;	ENSG00000138448;	0.9979178	1	FALSE
TC01002706.hg.1	ENSG00000184292;	0.9983396	1	FALSE
TC01002954.hg.1	ENSG00000134243;	0.9983741	1	FALSE
TC01000685.hg.1	ENSG00000162409;	0.9986429	1	FALSE
TC01000685.hg.1	ENSG00000162409;	0.9993735	1	FALSE
TC12002155.hg.1	ENSG00000176915	0.9993847	1	FALSE
TC04001084.hg.1	ENSG00000109819;	0.999404	1	FALSE
TC01001112.hg.1;	ENSG00000131778;	0.9994547	1	FALSE
TC01002669.hg.1	ENSG00000157193;	0.9995416	1	FALSE
TC0Y000351.hg.1	ENSG00000129824;	0.999603	1	FALSE
TC03000248.hg.1	ENSG00000144791	0.9998755	1	FALSE
TC04001084.hg.1	ENSG00000109819;	0.9999439	1	FALSE
TC01000728.hg.1	ENSG00000162433;	0.9999755	1	FALSE
TC12000531.hg.1	ENSG00000166986	0.9999901	1	FALSE
TC01003629.hg.1	ENSG00000116679;	0.9999968	1	FALSE
TC01000728.hg.1	ENSG00000162433;	0.999999	1	FALSE
TC01003629.hg.1	ENSG00000116679;	0.9999992	1	FALSE
TC01003629.hg.1	ENSG00000116679;	0.9999994	1	FALSE
TC01000728.hg.1	ENSG00000162433;	0.9999996	1	FALSE
TC01002932.hg.1	ENSG00000060718;	0.9999998	1	FALSE
TC01000728.hg.1	ENSG00000162433;	0.9999999	1	FALSE
TC06004141.hg.1	ENSG00000112096;	1	1	FALSE
TC01001112.hg.1;	ENSG00000131778;	1	1	FALSE
TC07001256.hg.1	ENSG00000154678	1	1	FALSE
TC07001256.hg.1	ENSG00000154678	1	1	FALSE

Names	Genes_In_Term	DEG	Input_gene
Total	18153	702	-
GO:0005576//extracellular region	2049	186	TC01000139.hg.1;
GO:0005578//proteinaceous extracellular matrix	325	54	TC01001188.hg.1;
GO:0031012//extracellular matrix	395	70	TC01001188.hg.1;
GO:0044421//extracellular region part	1093	123	TC01000904.hg.1;
GO:0005615//extracellular space	820	84	TC01000904.hg.1;
GO:0044420//extracellular matrix part	152	23	TC01002284.hg.1;
GO:0005886//plasma membrane	4087	214	TC01000394.hg.1;
GO:0016020//membrane	7384	349	TC01000394.hg.1;
GO:0071944//cell periphery	4180	216	TC01000394.hg.1;
GO:0044425//membrane part	5713	276	TC01000394.hg.1;
GO:0031224//intrinsic to membrane	5004	245	TC01000723.hg.1;
GO:NM_0059	10	5	TC02000511.hg.1;
GO:0016021//integral to membrane	4884	237	TC01000723.hg.1;
GO:0044459//plasma membrane part	1883	106	TC01000703.hg.1;
GO:0009986//cell surface	494	38	TC01000904.hg.1;
GO:0043025//neuronal cell body	216	21	TC01001805.hg.1;
GO:0031226//intrinsic to plasma membrane	1212	73	TC01000723.hg.1;
GO:0043205//fibril	8	4	TC01002284.hg.1;
GO:0005604//basement membrane	75	11	TC03002106.hg.1;
GO:0005887//integral to plasma membrane	1162	70	TC01000723.hg.1;
GO:0005783//endoplasmic reticulum	1218	72	TC01001607.hg.1;
GO:0044297//cell body	244	22	TC01001805.hg.1;
GO:0043005//neuron projection	464	34	TC01001805.hg.1;
GO:0097458//neuron part	598	41	TC01001805.hg.1;
GO:0016323//basolateral plasma membrane	146	15	TC01001473.hg.1;
GO:0005575//cellular_component	15844	640	TC01000100.hg.1;
GO:0005737//cytoplasm	8351	364	TC01000100.hg.1;
GO:0001527//microfibril	6	3	TC01002284.hg.1;
GO:0043235//receptor complex	161	15	TC01001095.hg.1;
GO:0031983//vesicle lumen	62	8	TC02001628.hg.1;
GO:0002116//semaphorin receptor complex	8	3	TC01003778.hg.1;
GO:0005788//endoplasmic reticulum lumen	141	13	TC01002932.hg.1;
GO:0031091//platelet alpha granule	51	7	TC02002823.hg.1;
GO:0045177//apical part of cell	264	20	TC01000703.hg.1;
GO:0008305//integrin complex	27	5	TC01001095.hg.1;
GO:0034358//plasma lipoprotein particle	39	6	TC02000416.hg.1;
GO:0005911//cell-cell junction	249	19	TC01000703.hg.1;
GO:NR_0014	9	3	TC06004066.hg.1;
GO:0032994//protein-lipid complex	40	6	TC02000416.hg.1;
GO:0042995//cell projection	1055	58	TC01000904.hg.1;
GO:0034774//secretory granule lumen	54	7	TC03001590.hg.1;
GO:0030424//axon	207	16	TC01001805.hg.1;
GO:0005905//coated pit	45	6	TC01000734.hg.1;
GO:0044432//endoplasmic reticulum part	871	48	TC01001607.hg.1;
GO:0060205//cytoplasmic membrane-bounded vesicle lumen	61	7	TC03001590.hg.1;
GO:0030132//clathrin coat of coated pit	12	3	TC01000734.hg.1;
GO:0009897//external side of plasma membrane	200	15	TC01000904.hg.1;
GO:0012505//endomembrane system	1516	76	TC01000734.hg.1;
GO:0060076//excitatory synapse	13	3	TC09000388.hg.1;
GO:0034361//very-low-density lipoprotein particle	24	4	TC02000416.hg.1;
GO:0034385//triglyceride-rich lipoprotein particle	24	4	TC02000416.hg.1;
GO:0042827//platelet dense granule	5	2	TC03000013.hg.1;
GO:0048471//perinuclear region of cytoplasm	387	24	TC01000703.hg.1;
GO:0005741//mitochondrial outer membrane	101	9	TC02000466.hg.1;
GO:0005913//cell-cell adherens junction	39	5	TC03000214.hg.1;
GO:0031093//platelet alpha granule lumen	39	5	TC03001590.hg.1;

GO:0005581//collagen	69	7	TC01002932.hg.1;
GO:0030426//growth cone	69	7	TC02002808.hg.1;
GO:0030125//clathrin vesicle coat	15	3	TC01000734.hg.1;
GO:0030425//dendrite	194	14	TC01002954.hg.1;
GO:0005794//Golgi apparatus	1093	56	TC01000394.hg.1;
GO:0014704//intercalated disc	27	4	TC01003129.hg.1;
GO:0030864//cortical actin cytoskeleton	27	4	TC01000394.hg.1;
GO:0030122//AP-2 adaptor complex	6	2	TC01000734.hg.1;
GO:0030173//integral to Golgi membrane	41	5	TC01000783.hg.1;
GO:0016324//apical plasma membrane	196	14	TC01000703.hg.1;
GO:0000139//Golgi membrane	523	30	TC01000783.hg.1;
GO:0031988//membrane-bounded vesicle	875	46	TC01000734.hg.1;
GO:0019867//outer membrane	124	10	TC02000466.hg.1;
GO:0005583//fibrillar collagen	16	3	TC01002932.hg.1;
GO:0030427//site of polarized growth	73	7	TC02002808.hg.1;
GO:0031228//intrinsic to Golgi membrane	43	5	TC01000783.hg.1;
GO:0005789//endoplasmic reticulum membrane	750	40	TC01001607.hg.1;
GO:0030141//secretory granule	223	15	TC02002823.hg.1;
GO:0044291//cell-cell contact zone	30	4	TC01003129.hg.1;
GO:0005606//laminin-1 complex	7	2	TC06000976.hg.1;
GO:0030128//clathrin coat of endocytic vesicle	7	2	TC01000734.hg.1;
GO:0032580//Golgi cisterna membrane	62	6	TC01001907.hg.1;
GO:0042175//nuclear outer membrane-endoplasmic reticulum r	764	40	TC01001607.hg.1;
GO:0031982//vesicle	949	48	TC01000734.hg.1;
GO:0005605//basal lamina	19	3	TC06000976.hg.1;
GO:0043204//perikaryon	33	4	TC02002808.hg.1;
GO:0045121//membrane raft	174	12	TC01001473.hg.1;
GO:0043292//contractile fiber	136	10	TC01002530.hg.1;
GO:0031968//organelle outer membrane	118	9	TC02000466.hg.1;
GO:0044306//neuron projection terminus	49	5	TC02002808.hg.1;
GO:0070161//anchoring junction	196	13	TC02001031.hg.1;
GO:0044449//contractile fiber part	120	9	TC01002530.hg.1;
GO:0044444//cytoplasmic part	6139	259	TC01000394.hg.1;
GO:0030877//beta-catenin destruction complex	9	2	TC03000214.hg.1;
GO:0044295//axonal growth cone	9	2	TC02002808.hg.1;
GO:0005637//nuclear inner membrane	35	4	TC01003791.hg.1;
GO:0070062//extracellular vesicular exosome	69	6	TC06000779.hg.1;
GO:0043230//extracellular organelle	70	6	TC06000779.hg.1;
GO:0065010//extracellular membrane-bounded organelle	70	6	TC06000779.hg.1;
GO:0043256//laminin complex	10	2	TC06000976.hg.1;
GO:0030017//sarcomere	107	8	TC01002530.hg.1;
GO:0030118//clathrin coat	38	4	TC01000734.hg.1;
GO:0031674//I band	72	6	TC01002530.hg.1;
GO:0031985//Golgi cisterna	72	6	TC01001907.hg.1;
GO:0042612//MHC class I protein complex	55	5	TC06001089.hg.1;
GO:0015629//actin cytoskeleton	335	19	TC01000394.hg.1;
GO:0044431//Golgi apparatus part	623	32	TC01000783.hg.1;
GO:0005916//fascia adherens	11	2	TC03000214.hg.1;
GO:0042627//chylomicron	11	2	TC02001628.hg.1;
GO:0031984//organelle subcompartment	74	6	TC01001907.hg.1;
GO:0031225//anchored to membrane	131	9	TC01001736.hg.1;
GO:0030054//cell junction	651	33	TC01000703.hg.1;
GO:0031904//endosome lumen	12	2	TC01003211.hg.1;
GO:0031090//organelle membrane	2205	98	TC01000734.hg.1;
GO:0016605//PML body	77	6	TC03000917.hg.1;
GO:0030131//clathrin adaptor complex	26	3	TC01000734.hg.1;
GO:0043296//apical junction complex	97	7	TC01000703.hg.1;
GO:0030175//filopodium	43	4	TC01000904.hg.1;

GO:0045178//basal part of cell	27	3	TC01002706.hg.1;
GO:0030669//clathrin-coated endocytic vesicle membrane	61	5	TC01000734.hg.1;
GO:0031528//microvillus membrane	13	2	TC03000214.hg.1;
GO:0005921//gap junction	28	3	TC01003129.hg.1;
GO:0016328//lateral plasma membrane	28	3	TC01002706.hg.1;
GO:0005923//tight junction	82	6	TC01000703.hg.1;
GO:0070160//occluding junction	82	6	TC01000703.hg.1;
GO:0005912//adherens junction	183	11	TC02001031.hg.1;
GO:0045334//clathrin-coated endocytic vesicle	64	5	TC01000734.hg.1;
GO:0030863//cortical cytoskeleton	47	4	TC01000394.hg.1;
GO:0030016//myofibril	124	8	TC01002530.hg.1;
GO:0030057//desmosome	15	2	TC06002157.hg.1;
GO:0030027//lamellipodium	106	7	TC02000270.hg.1;
GO:0030120//vesicle coat	32	3	TC01000734.hg.1;
GO:0005922//connexon complex	16	2	TC01003129.hg.1;
GO:0016023//cytoplasmic membrane-bounded vesicle	811	38	TC01000734.hg.1;
GO:0030119//AP-type membrane coat adaptor complex	33	3	TC01000734.hg.1;
GO:0031526//brush border membrane	33	3	TC03000262.hg.1;
GO:0046658//anchored to plasma membrane	33	3	TC05000478.hg.1;
GO:0030055//cell-substrate junction	132	8	TC02001031.hg.1;
GO:0031410//cytoplasmic vesicle	867	40	TC01000734.hg.1;
GO:0005903//brush border	53	4	TC03000262.hg.1;
GO:0005614//interstitial matrix	18	2	TC06001189.hg.1;
GO:0030140//trans-Golgi network transport vesicle	18	2	TC01002954.hg.1;
GO:0042383//sarcolemma	74	5	TC01000904.hg.1;
GO:0016010//dystrophin-associated glycoprotein complex	19	2	TC05000860.hg.1;
GO:0034362//low-density lipoprotein particle	19	2	TC02001628.hg.1;
GO:0005667//transcription factor complex	249	13	TC01003629.hg.1;
GO:0014069//postsynaptic density	76	5	TC02002808.hg.1;
GO:0005791//rough endoplasmic reticulum	37	3	TC04001245.hg.1;
GO:0005902//microvillus	57	4	TC01000904.hg.1;
GO:0000015//phosphopyruvate hydratase complex	5	1	TC12000099.hg.1
GO:0005593//FACIT collagen	5	1	TC08000700.hg.1
GO:0005826//actomyosin contractile ring	5	1	TC07000235.hg.1
GO:0036021//endolysosome lumen	5	1	TC01003211.hg.1
GO:0044453//nuclear membrane part	5	1	TC01003887.hg.1
GO:0071437//invadopodium	5	1	TC02002480.hg.1
GO:NM_0032	5	1	TC09001058.hg.1
GO:NM_0041	5	1	TC09001345.hg.1
GO:NM_0149	5	1	TC12000656.hg.1
GO:NM_1985	5	1	TC02000705.hg.1
GO:0044433//cytoplasmic vesicle part	413	20	TC01000734.hg.1;
GO:0005795//Golgi stack	100	6	TC01001907.hg.1;
GO:0009925//basal plasma membrane	21	2	TC01002706.hg.1;
GO:0043198//dendritic shaft	21	2	TC11002435.hg.1;
GO:0005901//caveola	60	4	TC01001473.hg.1;
GO:0008076//voltage-gated potassium channel complex	60	4	TC06000728.hg.1;
GO:0034705//potassium channel complex	60	4	TC06000728.hg.1;
GO:0045202//synapse	400	19	TC02002808.hg.1;
GO:0030018//Z disc	61	4	TC01002530.hg.1;
GO:0014731//spectrin-associated cytoskeleton	6	1	TC01000394.hg.1
GO:0016012//sarcoglycan complex	6	1	TC05000860.hg.1
GO:0016342//catenin complex	6	1	TC03000214.hg.1
GO:0019907//cyclin-dependent protein kinase activating kinase	6	1	TC12003293.hg.1
GO:0031092//platelet alpha granule membrane	6	1	TC12000076.hg.1
GO:0032809//neuronal cell body membrane	6	1	TC20000926.hg.1
GO:0033270//paranode region of axon	6	1	TC17000533.hg.1
GO:0044298//cell body membrane	6	1	TC20000926.hg.1

GO:0070382//exocytic vesicle	6	1	TC11002162.hg.1
GO:NR_0021	6	1	TC06001332.hg.1
GO:0030665//clathrin-coated vesicle membrane	126	7	TC01000734.hg.1;
GO:0005924//cell-substrate adherens junction	128	7	TC02001031.hg.1;
GO:0044448//cell cortex part	86	5	TC01000394.hg.1;
GO:0001939//female pronucleus	7	1	TC01002578.hg.1
GO:0008091//spectrin	7	1	TC01000394.hg.1
GO:0016011//dystroglycan complex	7	1	TC05000860.hg.1
GO:0016327//apicolateral plasma membrane	7	1	TC15001110.hg.1
GO:0016442//RNA-induced silencing complex	7	1	TC03000248.hg.1
GO:0030130//clathrin coat of trans-Golgi network vesicle	7	1	TC22000487.hg.1
GO:0031232//extrinsic to external side of plasma membrane	7	1	TC02002823.hg.1
GO:0031332//RNAi effector complex	7	1	TC03000248.hg.1
GO:0032590//dendrite membrane	7	1	TC20000926.hg.1
GO:0036056//filtration diaphragm	7	1	TC07001552.hg.1
GO:0036057//slit diaphragm	7	1	TC07001552.hg.1
GO:0046581//intercellular canaliculus	7	1	TC15001110.hg.1
GO:NM_0124	7	1	TC17001886.hg.1
GO:0000323//lytic vacuole	364	17	TC01002441.hg.1;
GO:0005764//lysosome	364	17	TC01002441.hg.1;
GO:0032589//neuron projection membrane	25	2	TC17001353.hg.1;
GO:0055038//recycling endosome membrane	25	2	TC08001659.hg.1;
GO:0045211//postsynaptic membrane	155	8	TC02002808.hg.1;
GO:0030667//secretory granule membrane	46	3	TC03000013.hg.1;
GO:0012510//trans-Golgi network transport vesicle membrane	8	1	TC22000487.hg.1
GO:0070938//contractile ring	8	1	TC07000235.hg.1
GO:NM_0068	8	1	TC10002938.hg.1
GO:0030136//clathrin-coated vesicle	209	10	TC01000734.hg.1;
GO:0019897//extrinsic to plasma membrane	71	4	TC01002166.hg.1;
GO:0030117//membrane coat	71	4	TC01000734.hg.1;
GO:0043202//lysosomal lumen	71	4	TC01003211.hg.1;
GO:0048475//coated membrane	71	4	TC01000734.hg.1;
GO:0031252//cell leading edge	234	11	TC02000270.hg.1;
GO:0030673//axolemma	9	1	TC17001353.hg.1
GO:0030934//anchoring collagen	9	1	TC08000700.hg.1
GO:0031095//platelet dense tubular network membrane	9	1	TC03000013.hg.1
GO:0036019//endolysosome	9	1	TC01003211.hg.1
GO:0042588//zymogen granule	9	1	TC10000415.hg.1
GO:0019898//extrinsic to membrane	119	6	TC01000394.hg.1;
GO:0005769//early endosome	190	9	TC01002954.hg.1;
GO:0033267//axon part	97	5	TC02002808.hg.1;
GO:0005890//sodium:potassium-exchanging ATPase complex	10	1	TC01001473.hg.1
GO:0043020//NADPH oxidase complex	10	1	TC11002178.hg.1
GO:0043034//costamere	10	1	TC0X000943.hg.1
GO:NM_0024	10	1	TC22000620.hg.1
GO:NR_0268	10	1	TC21000425.hg.1
GO:0044463//cell projection part	534	23	TC02002480.hg.1;
GO:0031965//nuclear membrane	194	9	TC01002954.hg.1;
GO:0005775//vacuolar lumen	76	4	TC01003211.hg.1;
GO:0005925//focal adhesion	124	6	TC02001031.hg.1;
GO:0005665//DNA-directed RNA polymerase II, core complex	11	1	TC04001084.hg.1
GO:0031094//platelet dense tubular network	11	1	TC03000013.hg.1
GO:0031307//integral to mitochondrial outer membrane	11	1	TC04002948.hg.1
GO:0033276//transcription factor TFTC complex	11	1	TC0X001176.hg.1
GO:NM_0140	11	1	TC02000286.hg.1
GO:0005793//endoplasmic reticulum-Golgi intermediate compai	55	3	TC02002637.hg.1;
GO:0005798//Golgi-associated vesicle	55	3	TC01002954.hg.1;
GO:0017053//transcriptional repressor complex	55	3	TC01003042.hg.1;

GO:0042611//MHC protein complex	126	6	TC06000408.hg.1;
GO:0030660//Golgi-associated vesicle membrane	33	2	TC06000945.hg.1;
GO:0031594//neuromuscular junction	33	2	TC02002808.hg.1;
GO:0030666//endocytic vesicle membrane	127	6	TC01000734.hg.1;
GO:0031306//intrinsic to mitochondrial outer membrane	12	1	TC04002948.hg.1
GO:0048786//presynaptic active zone	12	1	TC09000388.hg.1
GO:0005938//cell cortex	177	8	TC01000394.hg.1;
GO:0030133//transport vesicle	177	8	TC01002954.hg.1;
GO:0001725//stress fiber	34	2	TC01002530.hg.1;
GO:0097060//synaptic membrane	179	8	TC02002808.hg.1;
GO:0044304//main axon	35	2	TC17000533.hg.1;
GO:0031253//cell projection membrane	181	8	TC02002480.hg.1;
GO:0002102//podosome	13	1	TC01000904.hg.1
GO:0005790//smooth endoplasmic reticulum	13	1	TC17001353.hg.1
GO:0005942//phosphatidylinositol 3-kinase complex	13	1	TC01002616.hg.1
GO:0031258//lamellipodium membrane	13	1	TC02002480.hg.1
GO:0032432//actin filament bundle	37	2	TC01002530.hg.1;
GO:NM_0005	14	1	TC07000449.hg.1
GO:0005671//Ada2/Gcn5/Ada3 transcription activator complex	15	1	TC03000986.hg.1
GO:0031362//anchored to external side of plasma membrane	15	1	TC05001657.hg.1
GO:0005876//spindle microtubule	39	2	TC0X001279.hg.1
GO:0005829//cytosol	1983	78	TC01000685.hg.1;
GO:0009295//nucleoid	40	2	TC06004141.hg.1;
GO:0005796//Golgi lumen	65	3	TC03001590.hg.1;
GO:0034703//cation channel complex	116	5	TC06000728.hg.1;
GO:0000159//protein phosphatase type 2A complex	16	1	TC04001001.hg.1
GO:0005669//transcription factor TFIID complex	16	1	TC0X001176.hg.1
GO:0005859//muscle myosin complex	16	1	TC03001362.hg.1
GO:0045120//pronucleus	16	1	TC01002578.hg.1
GO:0072686//mitotic spindle	16	1	TC03000986.hg.1
GO:0008287//protein serine/threonine phosphatase complex	41	2	TC03000013.hg.1;
GO:0005773//vacuole	427	17	TC01002441.hg.1;
GO:0042641//actomyosin	42	2	TC01002530.hg.1;
GO:0043679//axon terminus	42	2	TC02002808.hg.1;
GO:0005892//acetylcholine-gated channel complex	17	1	TC15001136.hg.1
GO:0022627//cytosolic small ribosomal subunit	17	1	TC0Y000351.hg.1
GO:0044456//synapse part	301	12	TC02002808.hg.1;
GO:0055037//recycling endosome	69	3	TC02001437.hg.1;
GO:0034702//ion channel complex	173	7	TC06000728.hg.1;
GO:0031941//filamentous actin	18	1	TC0X000034.hg.1
GO:0000932//cytoplasmic mRNA processing body	44	2	TC03000248.hg.1;
GO:0005811//lipid particle	44	2	TC12000399.hg.1;
GO:0044464//cell part	13780	533	TC01000100.hg.1;
GO:0005623//cell	13781	533	TC01000100.hg.1;
GO:0044440//endosomal part	332	13	TC01002954.hg.1;
GO:0031233//intrinsic to external side of plasma membrane	19	1	TC05001657.hg.1
GO:0031463//Cul3-RING ubiquitin ligase complex	20	1	TC05001481.hg.1
GO:0030662//coated vesicle membrane	180	7	TC01000734.hg.1;
GO:0022626//cytosolic ribosome	48	2	TC03002146.hg.1;
GO:0030315//T-tubule	21	1	TC02000432.hg.1
GO:0031672//A band	21	1	TC03001362.hg.1
GO:0032420//stereocilium	21	1	TC03001255.hg.1
GO:0042470//melanosome	76	3	TC01002578.hg.1;
GO:0048770//pigment granule	76	3	TC01002578.hg.1;
GO:0001917//photoreceptor inner segment	22	1	TC12000099.hg.1
GO:0016460//myosin II complex	22	1	TC03001362.hg.1
GO:0030659//cytoplasmic vesicle membrane	347	13	TC01000734.hg.1;
GO:0015935//small ribosomal subunit	51	2	TC0Y000351.hg.1

GO:0010008//endosome membrane	322	12	TC01002954.hg.1;
GO:0030139//endocytic vesicle	189	7	TC01000734.hg.1;
GO:0030135//coated vesicle	270	10	TC01000734.hg.1;
GO:0000790//nuclear chromatin	135	5	TC02002624.hg.1;
GO:0005884//actin filament	52	2	TC0X000034.hg.1
GO:0070461//SAGA-type complex	25	1	TC0X001176.hg.1
GO:0012506//vesicle membrane	357	13	TC01000734.hg.1;
GO:0005844//polysome	26	1	TC0Y000351.hg.1
GO:0005891//voltage-gated calcium channel complex	26	1	TC19001220.hg.1
GO:0032592//integral to mitochondrial membrane	26	1	TC04002948.hg.1
GO:0043195//terminal bouton	26	1	TC09000388.hg.1
GO:0000795//synaptonemal complex	27	1	TC17001462.hg.1
GO:0043209//myelin sheath	27	1	TC01003017.hg.1
GO:0031301//integral to organelle membrane	255	9	TC01000783.hg.1;
GO:0005740//mitochondrial envelope	475	17	TC01001090.hg.1;
GO:0031966//mitochondrial membrane	449	16	TC01001607.hg.1;
GO:0034364//high-density lipoprotein particle	28	1	TC11000238.hg.1
GO:0005814//centriole	59	2	TC05001385.hg.1;
GO:0005802//trans-Golgi network	147	5	TC04000173.hg.1;
GO:0032588//trans-Golgi network membrane	60	2	TC06000408.hg.1;
GO:0044437//vacuolar part	288	10	TC01002441.hg.1;
GO:0032421//stereocilium bundle	29	1	TC03001255.hg.1
GO:0044445//cytosolic part	148	5	TC01002616.hg.1;
GO:0016459//myosin complex	61	2	TC03001362.hg.1;
GO:0005771//multivesicular body	30	1	TC06000945.hg.1
GO:0031256//leading edge membrane	93	3	TC02002480.hg.1;
GO:0031901//early endosome membrane	93	3	TC02002808.hg.1;
GO:0005765//lysosomal membrane	182	6	TC01002441.hg.1;
GO:NM_0010	324	11	TC01001704.hg.1;
GO:0016604//nuclear body	212	7	TC03000917.hg.1;
GO:0043197//dendritic spine	32	1	TC05000914.hg.1
GO:0044309//neuron spine	32	1	TC05000914.hg.1
GO:0044451//nucleoplasm part	690	24	TC01003042.hg.1;
GO:0031300//intrinsic to organelle membrane	275	9	TC01000783.hg.1;
GO:0000123//histone acetyltransferase complex	69	2	TC03000986.hg.1;
GO:0032153//cell division site	36	1	TC07000235.hg.1
GO:0032155//cell division site part	36	1	TC07000235.hg.1
GO:0005768//endosome	564	19	TC01002954.hg.1;
GO:0042645//mitochondrial nucleoid	37	1	TC06004141.hg.1
GO:0035770//ribonucleoprotein granule	72	2	TC03000248.hg.1;
GO:0034704//calcium channel complex	38	1	TC19001220.hg.1
GO:0000118//histone deacetylase complex	40	1	TC04000856.hg.1
GO:0005758//mitochondrial intermembrane space	40	1	TC01001090.hg.1
GO:0034707//chloride channel complex	40	1	TC21000138.hg.1
GO:0000151//ubiquitin ligase complex	142	4	TC05001481.hg.1;
GO:0031967//organelle envelope	779	26	TC01001090.hg.1;
GO:0005881//cytoplasmic microtubule	42	1	TC05000601.hg.1
GO:0031234//extrinsic to internal side of plasma membrane	42	1	TC01002166.hg.1
GO:0045335//phagocytic vesicle	80	2	TC02001100.hg.1;
GO:0042734//presynaptic membrane	44	1	TC13000741.hg.1
GO:0030496//midbody	81	2	TC01002578.hg.1;
GO:0016591//DNA-directed RNA polymerase II, holoenzyme	82	2	TC04001084.hg.1;
GO:0031975//envelope	792	26	TC01001090.hg.1;
GO:0005856//cytoskeleton	1539	53	TC01000394.hg.1;
GO:0001750//photoreceptor outer segment	48	1	TC09000614.hg.1
GO:0016607//nuclear speck	86	2	TC10000565.hg.1;
GO:0005635//nuclear envelope	310	9	TC01002954.hg.1;
GO:0000922//spindle pole	87	2	TC13000109.hg.1;

GO:0009898//internal side of plasma membrane	87	2	TC01001624.hg.1;
GO:0016529//sarcoplasmic reticulum	49	1	TC03000013.hg.1
GO:0045095//keratin filament	49	1	TC17001485.hg.1
GO:0005882//intermediate filament	123	3	TC06000945.hg.1;
GO:NM_0011	405	12	TC04001459.hg.1;
GO:0044424//intracellular part	11420	428	TC01000100.hg.1;
GO:0005774//vacuolar membrane	225	6	TC01002441.hg.1;
GO:0000792//heterochromatin	52	1	TC20000945.hg.1
GO:0019866//organelle inner membrane	352	10	TC01001607.hg.1;
GO:0000428//DNA-directed RNA polymerase complex	93	2	TC04001084.hg.1;
GO:0055029//nuclear DNA-directed RNA polymerase complex	93	2	TC04001084.hg.1;
GO:0016528//sarcoplasm	54	1	TC03000013.hg.1
GO:0031970//organelle envelope lumen	54	1	TC01001090.hg.1
GO:0030880//RNA polymerase complex	95	2	TC04001084.hg.1;
GO:0005819//spindle	200	5	TC03000986.hg.1;
GO:0030670//phagocytic vesicle membrane	57	1	TC18000047.hg.1
GO:0044450//microtubule organizing center part	98	2	TC05001385.hg.1;
GO:0044454//nuclear chromosome part	237	6	TC02002624.hg.1;
GO:0001669//acrosomal vesicle	59	1	TC05003439.hg.1
GO:0044391//ribosomal subunit	101	2	TC0Y000351.hg.1
GO:0044429//mitochondrial part	698	21	TC01000728.hg.1;
GO:0016363//nuclear matrix	63	1	TC05001481.hg.1
GO:0005770//late endosome	143	3	TC06000945.hg.1;
GO:0000794//condensed nuclear chromosome	64	1	TC17001462.hg.1
GO:0005739//mitochondrion	1457	47	TC01000728.hg.1;
GO:0097223//sperm part	66	1	TC05003439.hg.1
GO:0005654//nucleoplasm	1186	37	TC01000685.hg.1;
GO:0000785//chromatin	290	7	TC01003172.hg.1;
GO:0042613//MHC class II protein complex	71	1	TC06000408.hg.1
GO:0005777//peroxisome	118	2	TC03001466.hg.1;
GO:0042579//microbody	118	2	TC03001466.hg.1;
GO:0045111//intermediate filament cytoskeleton	158	3	TC06000945.hg.1;
GO:0000228//nuclear chromosome	267	6	TC02002624.hg.1;
GO:0030658//transport vesicle membrane	120	2	TC06000408.hg.1;
GO:ENST000	160	3	TC08000255.hg.1;
GO:0043227//membrane-bounded organelle	8945	325	TC01000100.hg.1;
GO:0005622//intracellular	11692	432	TC01000100.hg.1;
GO:0034399//nuclear periphery	77	1	TC05001481.hg.1
GO:0001726//ruffle	122	2	TC05002110.hg.1;
GO:0031902//late endosome membrane	79	1	TC17001830.hg.1
GO:0044430//cytoskeletal part	1112	33	TC01000394.hg.1;
GO:0032993//protein-DNA complex	129	2	TC01003172.hg.1;
GO:0005813//centrosome	356	8	TC01000785.hg.1;
GO:0008021//synaptic vesicle	87	1	TC03000846.hg.1
GO:0031513//nonmotile primary cilium	88	1	TC09000614.hg.1
GO:0000786//nucleosome	89	1	TC01003172.hg.1
GO:0012507//ER to Golgi transport vesicle membrane	89	1	TC06000408.hg.1
GO:0071556//integral to lumenal side of endoplasmic reticulum	89	1	TC06000408.hg.1
GO:0005759//mitochondrial matrix	291	6	TC01000728.hg.1;
GO:0031461//cullin-RING ubiquitin ligase complex	90	1	TC05001481.hg.1
GO:0043231//intracellular membrane-bounded organelle	8909	320	TC01000100.hg.1;
GO:0043233//organelle lumen	2913	95	TC01000685.hg.1;
GO:0030134//ER to Golgi transport vesicle	92	1	TC06000408.hg.1
GO:0043226//organelle	9838	355	TC01000100.hg.1;
GO:0031974//membrane-enclosed lumen	2959	96	TC01000685.hg.1;
GO:0044422//organelle part	6109	212	TC01000394.hg.1;
GO:0072372//primary cilium	104	1	TC09000614.hg.1
GO:0005743//mitochondrial inner membrane	317	6	TC01001607.hg.1;

GO:0030176//integral to endoplasmic reticulum membrane	163	2	TC06000408.hg.1;
GO:0005840//ribosome	208	3	TC03002146.hg.1;
GO:0005815//microtubule organizing center	440	9	TC01000785.hg.1;
GO:0005681//spliceosomal complex	117	1	TC01003629.hg.1
GO:0044455//mitochondrial membrane part	121	1	TC04002948.hg.1
GO:0070013//intracellular organelle lumen	2868	89	TC01000685.hg.1;
GO:0044446//intracellular organelle part	6023	204	TC01000394.hg.1;
GO:0043229//intracellular organelle	9803	348	TC01000100.hg.1;
GO:0005874//microtubule	308	5	TC05000601.hg.1;
GO:0031227//intrinsic to endoplasmic reticulum membrane	179	2	TC06000408.hg.1;
GO:0005730//nucleolus	1315	35	TC01001112.hg.1;
GO:0000793//condensed chromosome	139	1	TC17001462.hg.1
GO:0005929//cilium	254	3	TC07001256.hg.1;
GO:0031981//nuclear lumen	2338	67	TC01000685.hg.1;
GO:0015630//microtubule cytoskeleton	803	17	TC01000785.hg.1;
GO:0043234//protein complex	3150	93	TC01000394.hg.1;
GO:0005634//nucleus	5342	171	TC01000100.hg.1;
GO:0044427//chromosomal part	517	8	TC01003172.hg.1;
GO:0044428//nuclear part	2702	75	TC01000685.hg.1;
GO:0043228//non-membrane-bounded organelle	3387	96	TC01000394.hg.1;
GO:0043232//intracellular non-membrane-bounded organelle	3387	96	TC01000394.hg.1;
GO:0005694//chromosome	611	9	TC01003172.hg.1;
GO:0030529//ribonucleoprotein complex	531	7	TC01003629.hg.1;
GO:0032991//macromolecular complex	3724	104	TC01000394.hg.1;

Gene_Symbol	P_value	Q_value	Significant
-	-	-	-
ENSG00000171819;	0	0	TRUE
ENSG00000143369;	0	0	TRUE
ENSG00000143369;	0	0	TRUE
ENSG00000162692;	0	0	TRUE
ENSG00000162692;	2.22E-16	1.901E-14	TRUE
ENSG00000117122;	2.096E-08	1.495E-06	TRUE
ENSG00000159023;	3.818E-07	2.334E-05	TRUE
ENSG00000159023;	4.94E-07	2.643E-05	TRUE
ENSG00000159023;	9.046E-07	4.302E-05	TRUE
ENSG00000159023;	4.396E-06	0.0001882	TRUE
ENSG00000185483;	8.602E-06	0.0003347	TRUE
ENSG00000168906;	1.828E-05	0.0006518	TRUE
ENSG00000185483;	2.585E-05	0.0008512	TRUE
ENSG00000132849;	4.434E-05	0.0013317	TRUE
ENSG00000162692;	4.667E-05	0.0013317	TRUE
ENSG00000092969;	0.0001022	0.0026242	TRUE
ENSG00000185483;	0.0001042	0.0026242	TRUE
ENSG00000117122;	0.0001371	0.0030949	TRUE
ENSG00000090530;	0.0001374	0.0030949	TRUE
ENSG00000185483;	0.0001447	0.0030966	TRUE
ENSG00000116711;	0.0002045	0.0041069	TRUE
ENSG00000092969;	0.0002111	0.0041069	TRUE
ENSG00000092969;	0.0002882	0.0052062	TRUE
ENSG00000092969;	0.0002919	0.0052062	TRUE
ENSG00000143153;	0.0005411	0.0092636	TRUE
ENSG00000049246;	0.0006227	0.0102498	TRUE
ENSG00000049246;	0.0008828	0.0139947	TRUE
ENSG00000117122;	0.0010551	0.0161281	TRUE
ENSG00000143127;	0.0014815	0.0218651	TRUE
ENSG00000084674;	0.0025696	0.0366599	TRUE
ENSG00000076356;	0.0027872	0.0384819	TRUE
ENSG00000060718;	0.0032679	0.0414688	TRUE
ENSG00000135919;	0.003306	0.0414688	TRUE
ENSG00000132849;	0.0033375	0.0414688	TRUE
ENSG00000143127;	0.0033911	0.0414688	TRUE
ENSG00000116005;	0.0035927	0.0427133	TRUE
ENSG00000132849;	0.003854	0.0445814	TRUE
NR_001434;NR_001	0.0040613	0.0448978	TRUE
ENSG00000116005;	0.0040912	0.0448978	TRUE
ENSG00000162692;	0.0043435	0.0464749	TRUE
ENSG00000184500;	0.004578	0.0477902	TRUE
ENSG00000092969;	0.0067636	0.068924	FALSE
ENSG00000118473;	0.0073789	0.0734455	FALSE
ENSG00000116711;	0.0086771	0.0844045	FALSE
ENSG00000184500;	0.0089478	0.0851035	FALSE
ENSG00000118473;	0.0097534	0.0907493	FALSE
ENSG00000162692;	0.0111207	0.1012695	FALSE
ENSG00000118473;	0.0113897	0.1015583	FALSE
ENSG00000148053;	0.0123198	0.1076096	FALSE
ENSG00000116005;	0.0127175	0.1077836	FALSE
ENSG00000116005;	0.0127175	0.1077836	FALSE
ENSG00000150995;	0.0138153	0.1137102	FALSE
ENSG00000132849;	0.0159801	0.1266208	FALSE
ENSG00000159399;	0.0162798	0.1266208	FALSE
ENSG00000168036;	0.0165465	0.1266208	FALSE
ENSG00000184500;	0.0165465	0.1266208	FALSE

ENSG00000060718;	0.017011	0.1266208	FALSE
ENSG00000116106;	0.017011	0.1266208	FALSE
ENSG00000118473;	0.0185069	0.1313246	FALSE
ENSG00000134243;	0.0188017	0.1313246	FALSE
ENSG00000159023;	0.0190795	0.1313246	FALSE
ENSG00000143140;	0.0191771	0.1313246	FALSE
ENSG00000159023;	0.0191771	0.1313246	FALSE
ENSG00000118473;	0.0201953	0.1319739	FALSE
ENSG00000117069;	0.0202268	0.1319739	FALSE
ENSG00000132849;	0.0203511	0.1319739	FALSE
ENSG00000117069;	0.0208164	0.1329761	FALSE
ENSG00000118473;	0.0215158	0.1353439	FALSE
ENSG00000159399;	0.0221048	0.1353439	FALSE
ENSG00000060718;	0.0221357	0.1353439	FALSE
ENSG00000116106;	0.0225386	0.1358663	FALSE
ENSG00000117069;	0.0244047	0.1450725	FALSE
ENSG00000116711;	0.0251192	0.1472743	FALSE
ENSG00000135919;	0.0270042	0.15417	FALSE
ENSG00000143140;	0.0273523	0.15417	FALSE
ENSG00000196569;	0.0275561	0.15417	FALSE
ENSG00000118473;	0.0275561	0.15417	FALSE
ENSG00000143641;	0.032223	0.175137	FALSE
ENSG00000116711;	0.0323267	0.175137	FALSE
ENSG00000118473;	0.0346507	0.1853813	FALSE
ENSG00000196569;	0.0351674	0.1858229	FALSE
ENSG00000116106;	0.0373204	0.1947944	FALSE
ENSG00000143153;	0.0380859	0.1957754	FALSE
ENSG00000183386;	0.0384232	0.1957754	FALSE
ENSG00000159399;	0.0394726	0.1987561	FALSE
ENSG00000116106;	0.0401047	0.1995907	FALSE
ENSG00000152256;	0.0414589	0.2039588	FALSE
ENSG00000183386;	0.0431947	0.2092526	FALSE
ENSG00000159023;	0.043702	0.2092526	FALSE
ENSG00000168036;	0.0448841	0.2092526	FALSE
ENSG00000116106;	0.0448841	0.2092526	FALSE
ENSG00000143473;	0.0449795	0.2092526	FALSE
ENSG00000135318;	0.0502947	0.2314636	FALSE
ENSG00000135318;	0.0532949	0.241378	FALSE
ENSG00000135318;	0.0532949	0.241378	FALSE
ENSG00000196569;	0.0546961	0.2438533	FALSE
ENSG00000183386;	0.0555258	0.2450006	FALSE
ENSG00000118473;	0.0579867	0.253248	FALSE
ENSG00000183386;	0.059615	0.2564343	FALSE
ENSG00000143641;	0.059615	0.2564343	FALSE
ENSG00000111981;	0.0607797	0.2575616	FALSE
ENSG00000159023;	0.0624124	0.2618872	FALSE
ENSG00000117069;	0.0631092	0.2622403	FALSE
ENSG00000168036;	0.0651776	0.2669476	FALSE
ENSG00000084674;	0.0651776	0.2669476	FALSE
ENSG00000143641;	0.0663625	0.2679541	FALSE
ENSG00000196352;	0.0678526	0.2714104	FALSE
ENSG00000132849;	0.068921	0.2731313	FALSE
ENSG00000143387;	0.0762625	0.2961081	FALSE
ENSG00000118473;	0.0767003	0.2961081	FALSE
ENSG00000136603;	0.0772842	0.2961081	FALSE
ENSG00000118473;	0.0774862	0.2961081	FALSE
ENSG00000132849;	0.081795	0.3098076	FALSE
ENSG00000162692;	0.0836096	0.3139027	FALSE

ENSG00000184292;	0.0847749	0.31551	FALSE
ENSG00000118473;	0.0864764	0.3190682	FALSE
ENSG00000168036;	0.0878884	0.3215063	FALSE
ENSG00000143140;	0.0923431	0.333526	FALSE
ENSG00000184292;	0.0923431	0.333526	FALSE
ENSG00000132849;	0.0975958	0.3464113	FALSE
ENSG00000132849;	0.0975958	0.3464113	FALSE
ENSG00000152256;	0.0987434	0.3464113	FALSE
ENSG00000118473;	0.1011484	0.3519637	FALSE
ENSG00000159023;	0.1074485	0.3696817	FALSE
ENSG00000183386;	0.1079678	0.3696817	FALSE
ENSG00000112378;	0.1125341	0.3822587	FALSE
ENSG00000152527;	0.1165151	0.3926651	FALSE
ENSG00000118473;	0.125173	0.4162156	FALSE
ENSG00000143140;	0.1254482	0.4162156	FALSE
ENSG00000118473;	0.1277051	0.4204446	FALSE
ENSG00000118473;	0.1339583	0.4343497	FALSE
ENSG00000160801;	0.1339583	0.4343497	FALSE
ENSG00000174136;	0.1339583	0.4343497	FALSE
ENSG00000152256;	0.1396567	0.4460677	FALSE
ENSG00000118473;	0.1411494	0.4474959	FALSE
ENSG00000160801;	0.1481409	0.4662081	FALSE
ENSG00000112562;	0.1522195	0.4738177	FALSE
ENSG00000134243;	0.1522195	0.4738177	FALSE
ENSG00000162692;	0.1580296	0.4865949	FALSE
ENSG00000170624;	0.1659903	0.4913501	FALSE
ENSG00000084674;	0.1659903	0.4913501	FALSE
ENSG00000116679;	0.1690855	0.4913501	FALSE
ENSG00000116106;	0.1707184	0.4913501	FALSE
ENSG00000145242;	0.1710336	0.4913501	FALSE
ENSG00000162692;	0.1780756	0.4913501	FALSE
ENSG00000111674	0.1789872	0.4913501	FALSE
ENSG00000187955	0.1789872	0.4913501	FALSE
ENSG00000011426	0.1789872	0.4913501	FALSE
ENSG00000143387	0.1789872	0.4913501	FALSE
ENSG00000143815	0.1789872	0.4913501	FALSE
ENSG00000078098	0.1789872	0.4913501	FALSE
ENSG00000198467	0.1789872	0.4913501	FALSE
ENSG00000131669	0.1789872	0.4913501	FALSE
ENSG00000067798	0.1789872	0.4913501	FALSE
ENSG00000188177	0.1789872	0.4913501	FALSE
ENSG00000118473;	0.1790902	0.4913501	FALSE
ENSG00000143641;	0.190552	0.5194665	FALSE
ENSG00000184292;	0.1941075	0.5241514	FALSE
ENSG00000149571;	0.1941075	0.5241514	FALSE
ENSG00000143153;	0.2017417	0.5273861	FALSE
ENSG00000185760;	0.2017417	0.5273861	FALSE
ENSG00000185760;	0.2017417	0.5273861	FALSE
ENSG00000116106;	0.2090053	0.5273861	FALSE
ENSG00000183386;	0.2098305	0.5273861	FALSE
ENSG00000159023	0.2107456	0.5273861	FALSE
ENSG00000170624	0.2107456	0.5273861	FALSE
ENSG00000168036	0.2107456	0.5273861	FALSE
ENSG00000180818	0.2107456	0.5273861	FALSE
ENSG00000010278	0.2107456	0.5273861	FALSE
ENSG00000158445	0.2107456	0.5273861	FALSE
ENSG00000108797	0.2107456	0.5273861	FALSE
ENSG00000158445	0.2107456	0.5273861	FALSE

ENSG00000137501	0.2107456	0.5273861	FALSE
ENSG00000168405	0.2107456	0.5273861	FALSE
ENSG00000118473;	0.2156369	0.5273861	FALSE
ENSG00000152256;	0.2269221	0.548914	FALSE
ENSG00000159023;	0.2393096	0.548914	FALSE
ENSG00000117394	0.2412772	0.548914	FALSE
ENSG00000159023	0.2412772	0.548914	FALSE
ENSG00000170624	0.2412772	0.548914	FALSE
ENSG00000104067	0.2412772	0.548914	FALSE
ENSG00000144791	0.2412772	0.548914	FALSE
ENSG00000070371	0.2412772	0.548914	FALSE
ENSG00000135919	0.2412772	0.548914	FALSE
ENSG00000144791	0.2412772	0.548914	FALSE
ENSG00000158445	0.2412772	0.548914	FALSE
ENSG00000187391	0.2412772	0.548914	FALSE
ENSG00000187391	0.2412772	0.548914	FALSE
ENSG00000104067	0.2412772	0.548914	FALSE
ENSG00000132471	0.2412772	0.548914	FALSE
ENSG00000162511;	0.2456006	0.548914	FALSE
ENSG00000162511;	0.2456006	0.548914	FALSE
ENSG00000176658;	0.2517121	0.5526738	FALSE
ENSG00000104419;	0.2517121	0.5526738	FALSE
ENSG00000116106;	0.2518023	0.5526738	FALSE
ENSG00000150995;	0.2624249	0.5730503	FALSE
ENSG00000070371	0.2706293	0.5849966	FALSE
ENSG00000011426	0.2706293	0.5849966	FALSE
ENSG00000148671	0.2706293	0.5849966	FALSE
ENSG00000118473;	0.2906239	0.614936	FALSE
ENSG00000116285;	0.2947157	0.614936	FALSE
ENSG00000118473;	0.2947157	0.614936	FALSE
ENSG00000143387;	0.2947157	0.614936	FALSE
ENSG00000118473;	0.2947157	0.614936	FALSE
ENSG00000152527;	0.296793	0.614936	FALSE
ENSG00000176658	0.2988474	0.614936	FALSE
ENSG00000187955	0.2988474	0.614936	FALSE
ENSG00000150995	0.2988474	0.614936	FALSE
ENSG00000143387	0.2988474	0.614936	FALSE
ENSG00000122862	0.2988474	0.614936	FALSE
ENSG00000159023;	0.31292	0.6347382	FALSE
ENSG00000134243;	0.3149278	0.6357976	FALSE
ENSG00000116106;	0.3215284	0.6389967	FALSE
ENSG00000143153	0.3259753	0.6389967	FALSE
ENSG00000086991	0.3259753	0.6389967	FALSE
ENSG00000198947	0.3259753	0.6389967	FALSE
ENSG00000169184	0.3259753	0.6389967	FALSE
NR_026812	0.3259753	0.6389967	FALSE
ENSG00000078098;	0.3269633	0.6389967	FALSE
ENSG00000134243;	0.3365536	0.6547497	FALSE
ENSG00000143387;	0.3387589	0.6560579	FALSE
ENSG00000152256;	0.3472764	0.669092	FALSE
ENSG00000109819	0.3520551	0.669092	FALSE
ENSG00000150995	0.3520551	0.669092	FALSE
ENSG00000137463	0.3520551	0.669092	FALSE
ENSG00000187325	0.3520551	0.669092	FALSE
ENSG00000171150	0.3520551	0.669092	FALSE
ENSG00000081320;	0.358318	0.669092	FALSE
ENSG00000134243;	0.358318	0.669092	FALSE
ENSG00000092607;	0.358318	0.669092	FALSE

ENSG00000223865;	0.3611221	0.669092	FALSE
ENSG00000152661;	0.3667042	0.6731995	FALSE
ENSG00000116106;	0.3667042	0.6731995	FALSE
ENSG00000118473;	0.3680577	0.6731995	FALSE
ENSG00000137463	0.3771272	0.6803889	FALSE
ENSG00000148053	0.3771272	0.6803889	FALSE
ENSG00000159023;	0.3775522	0.6803889	FALSE
ENSG00000134243;	0.3775522	0.6803889	FALSE
ENSG00000183386;	0.3806888	0.6817357	FALSE
ENSG00000116106;	0.3893503	0.6943413	FALSE
ENSG00000108797;	0.3945406	0.700678	FALSE
ENSG00000078098;	0.4011604	0.7023583	FALSE
ENSG00000162692	0.4012304	0.7023583	FALSE
ENSG00000176658	0.4012304	0.7023583	FALSE
ENSG00000117461	0.4012304	0.7023583	FALSE
ENSG00000078098	0.4012304	0.7023583	FALSE
ENSG00000183386;	0.4218041	0.7308994	FALSE
ENSG00000049540	0.4244022	0.732436	FALSE
ENSG00000163872	0.4466785	0.7646386	FALSE
ENSG00000184349	0.4466785	0.7646386	FALSE
ENSG00000077274;	0.4484213	0.7646386	FALSE
ENSG00000162409;	0.4541341	0.7713072	FALSE
ENSG00000112096;	0.4614686	0.7765278	FALSE
ENSG00000184500;	0.462488	0.7765278	FALSE
ENSG00000185760;	0.4673064	0.7765278	FALSE
ENSG00000074211	0.4680938	0.7765278	FALSE
ENSG00000187325	0.4680938	0.7765278	FALSE
ENSG00000160808	0.4680938	0.7765278	FALSE
ENSG00000117394	0.4680938	0.7765278	FALSE
ENSG00000163872	0.4680938	0.7765278	FALSE
ENSG00000150995;	0.4743333	0.777834	FALSE
ENSG00000162511;	0.4863217	0.7877803	FALSE
ENSG00000183386;	0.4870101	0.7877803	FALSE
ENSG00000116106;	0.4870101	0.7877803	FALSE
ENSG00000166664	0.4886815	0.7877803	FALSE
ENSG00000129824	0.4886815	0.7877803	FALSE
ENSG00000116106;	0.4987648	0.7995181	FALSE
ENSG00000144476;	0.5021405	0.8019259	FALSE
ENSG00000185760;	0.5057767	0.8047302	FALSE
ENSG00000146950	0.5084734	0.8060245	FALSE
ENSG00000144791;	0.5117813	0.8067859	FALSE
ENSG00000185432;	0.5117813	0.8067859	FALSE
ENSG00000049246;	0.5168746	0.8095496	FALSE
ENSG00000049246;	0.5182631	0.8095496	FALSE
ENSG00000134243;	0.5217575	0.8120444	FALSE
ENSG00000184349	0.5275002	0.8180076	FALSE
ENSG00000171617	0.5457915	0.8433169	FALSE
ENSG00000118473;	0.5479845	0.8436596	FALSE
ENSG00000189058;	0.558901	0.8570368	FALSE
ENSG00000135636	0.5633757	0.8570368	FALSE
ENSG00000160808	0.5633757	0.8570368	FALSE
ENSG00000033867	0.5633757	0.8570368	FALSE
ENSG00000117394;	0.5676868	0.8570368	FALSE
ENSG00000117394;	0.5676868	0.8570368	FALSE
ENSG00000111674	0.5802801	0.869912	FALSE
ENSG00000160808	0.5802801	0.869912	FALSE
ENSG00000118473;	0.5865385	0.8746985	FALSE
ENSG00000129824;	0.5920565	0.8790702	FALSE

ENSG00000134243;	0.5935777	0.8790702	FALSE
ENSG00000118473;	0.599857	0.8803759	FALSE
ENSG00000118473;	0.6005803	0.8803759	FALSE
ENSG00000115415;	0.6021167	0.8803759	FALSE
ENSG00000146950;	0.6026872	0.8803759	FALSE
ENSG00000187325	0.6271718	0.9105164	FALSE
ENSG00000118473;	0.6275756	0.9105164	FALSE
ENSG00000129824	0.6416094	0.9230549	FALSE
ENSG00000141837	0.6416094	0.9230549	FALSE
ENSG00000137463	0.6416094	0.9230549	FALSE
ENSG00000148053	0.6416094	0.9230549	FALSE
ENSG00000131747	0.6554887	0.9312685	FALSE
ENSG00000134198	0.6554887	0.9312685	FALSE
ENSG00000117069;	0.65711	0.9312685	FALSE
ENSG00000117289;	0.6634908	0.9372081	FALSE
ENSG00000116711;	0.6673635	0.9385566	FALSE
ENSG00000173432	0.6688313	0.9385566	FALSE
ENSG00000145632;	0.6712418	0.9388545	FALSE
ENSG00000109610;	0.6764479	0.9388545	FALSE
ENSG00000223865;	0.6802118	0.9388545	FALSE
ENSG00000162511;	0.6804801	0.9388545	FALSE
ENSG00000033867	0.6816578	0.9388545	FALSE
ENSG00000117461;	0.682205	0.9388545	FALSE
ENSG00000160808;	0.6889809	0.9451404	FALSE
ENSG00000152661	0.6939882	0.9489678	FALSE
ENSG00000078098;	0.7029179	0.9565942	FALSE
ENSG00000116106;	0.7029179	0.9565942	FALSE
ENSG00000162511;	0.7105121	0.9608052	FALSE
ENSG00000163531;	0.7126543	0.9608052	FALSE
ENSG00000136603;	0.7167686	0.9608052	FALSE
ENSG00000145934	0.7172366	0.9608052	FALSE
ENSG00000145934	0.7172366	0.9608052	FALSE
ENSG00000092607;	0.7335951	0.9781268	FALSE
ENSG00000117069;	0.7409858	0.9849128	FALSE
ENSG00000163872;	0.7521791	0.9966955	FALSE
ENSG00000011426	0.7585753	0.9999686	FALSE
ENSG00000011426	0.7585753	0.9999686	FALSE
ENSG00000134243;	0.7641625	0.9999686	FALSE
ENSG00000112096	0.7679301	0.9999686	FALSE
ENSG00000144791;	0.7728647	0.9999686	FALSE
ENSG00000141837	0.7769228	0.9999686	FALSE
ENSG00000164105	0.7938779	0.9999686	FALSE
ENSG00000117289	0.7938779	0.9999686	FALSE
ENSG00000159212	0.7938779	0.9999686	FALSE
ENSG00000171617;	0.8039824	0.9999686	FALSE
ENSG00000117289;	0.808629	0.9999686	FALSE
ENSG00000155324	0.8095459	0.9999686	FALSE
ENSG00000116285	0.8095459	0.9999686	FALSE
ENSG00000138448;	0.8208036	0.9999686	FALSE
ENSG00000178695	0.8240245	0.9999686	FALSE
ENSG00000117394;	0.8261121	0.9999686	FALSE
ENSG00000109819;	0.8312787	0.9999686	FALSE
ENSG00000117289;	0.8328319	0.9999686	FALSE
ENSG00000159023;	0.8336702	0.9999686	FALSE
ENSG00000095303	0.8497674	0.9999686	FALSE
ENSG00000108175;	0.8505868	0.9999686	FALSE
ENSG00000134243;	0.8515674	0.9999686	FALSE
ENSG00000073910;	0.855089	0.9999686	FALSE

ENSG00000116741;	0.855089	0.9999686	FALSE
ENSG00000150995	0.8555925	0.9999686	FALSE
ENSG00000212724	0.8555925	0.9999686	FALSE
ENSG00000152661;	0.8594255	0.9999686	FALSE
ENSG00000138795;	0.8632113	0.9999686	FALSE
ENSG00000049246;	0.8696915	0.9999686	FALSE
ENSG00000162511;	0.8714885	0.9999686	FALSE
ENSG00000101115	0.871749	0.9999686	FALSE
ENSG00000116711;	0.8782325	0.9999686	FALSE
ENSG00000109819;	0.879594	0.9999686	FALSE
ENSG00000109819;	0.879594	0.9999686	FALSE
ENSG00000150995	0.8815041	0.9999686	FALSE
ENSG00000117289	0.8815041	0.9999686	FALSE
ENSG00000109819;	0.8868729	0.9999686	FALSE
ENSG00000163872;	0.8901852	0.9999686	FALSE
ENSG00000168461	0.8947651	0.9999686	FALSE
ENSG00000145632;	0.8970297	0.9999686	FALSE
ENSG00000115415;	0.9000617	0.9999686	FALSE
ENSG00000213949	0.9027718	0.9999686	FALSE
ENSG00000129824;	0.9063324	0.9999686	FALSE
ENSG00000162433;	0.9071465	0.9999686	FALSE
ENSG00000171617	0.9170061	0.9999686	FALSE
ENSG00000152661;	0.9184416	0.9999686	FALSE
ENSG00000131747	0.9202267	0.9999686	FALSE
ENSG00000162433;	0.9213458	0.9999686	FALSE
ENSG00000213949	0.9262984	0.9999686	FALSE
ENSG00000162409;	0.9312996	0.9999686	FALSE
ENSG00000203818;	0.9350653	0.9999686	FALSE
ENSG00000223865	0.9395345	0.9999686	FALSE
ENSG00000163931;	0.9457935	0.9999686	FALSE
ENSG00000163931;	0.9457935	0.9999686	FALSE
ENSG00000152661;	0.9467756	0.9999686	FALSE
ENSG00000115415;	0.9487792	0.9999686	FALSE
ENSG00000223865;	0.9492247	0.9999686	FALSE
ENSG00000157168;	0.9497726	0.9999686	FALSE
ENSG00000049246;	0.9504739	0.9999686	FALSE
ENSG00000049246;	0.9509358	0.9999686	FALSE
ENSG00000171617	0.9523225	0.9999686	FALSE
ENSG00000196923;	0.9524483	0.9999686	FALSE
ENSG00000154265	0.9559539	0.9999686	FALSE
ENSG00000159023;	0.9583629	0.9999686	FALSE
ENSG00000203818;	0.9622589	0.9999686	FALSE
ENSG00000154027;	0.967861	0.9999686	FALSE
ENSG00000196549	0.9679193	0.9999686	FALSE
ENSG00000095303	0.9691658	0.9999686	FALSE
ENSG00000203818	0.9703641	0.9999686	FALSE
ENSG00000223865	0.9703641	0.9999686	FALSE
ENSG00000223865	0.9703641	0.9999686	FALSE
ENSG00000162433;	0.9710537	0.9999686	FALSE
ENSG00000171617	0.9715158	0.9999686	FALSE
ENSG00000049246;	0.973069	0.9999686	FALSE
ENSG00000162409;	0.9734987	0.9999686	FALSE
ENSG00000223865	0.9736868	0.9999686	FALSE
ENSG00000049246;	0.9773815	0.9999686	FALSE
ENSG00000162409;	0.9776181	0.9999686	FALSE
ENSG00000159023;	0.9787857	0.9999686	FALSE
ENSG00000095303	0.9836505	0.9999686	FALSE
ENSG00000116711;	0.9848794	0.9999686	FALSE

ENSG00000223865;	0.9880546	0.9999686	FALSE
ENSG00000189058;	0.9882352	0.9999686	FALSE
ENSG00000154027;	0.9894429	0.9999686	FALSE
ENSG00000116679	0.9902399	0.9999686	FALSE
ENSG00000137463	0.9916731	0.9999686	FALSE
ENSG00000162409;	0.9922804	0.9999686	FALSE
ENSG00000159023;	0.9923917	0.9999686	FALSE
ENSG00000049246;	0.9925928	0.9999686	FALSE
ENSG00000155324;	0.9930982	0.9999686	FALSE
ENSG00000223865;	0.9931389	0.9999686	FALSE
ENSG00000131778;	0.9944889	0.9999686	FALSE
ENSG00000131747	0.995927	0.9999686	FALSE
ENSG00000154678;	0.9973086	0.9999686	FALSE
ENSG00000162409;	0.997792	0.9999686	FALSE
ENSG00000154027;	0.9983761	0.9999686	FALSE
ENSG00000159023;	0.9989339	0.9999686	FALSE
ENSG00000049246;	0.9990239	0.9999686	FALSE
ENSG00000203818;	0.9994238	0.9999686	FALSE
ENSG00000162409;	0.9996278	0.9999686	FALSE
ENSG00000159023;	0.9998584	0.9999686	FALSE
ENSG00000159023;	0.9998584	0.9999686	FALSE
ENSG00000203818;	0.9998702	0.9999686	FALSE
ENSG00000116679;	0.9998806	0.9999686	FALSE
ENSG00000159023;	0.9999686	0.9999686	FALSE

Names
Total
GO:0005102//receptor binding
GO:0097367//carbohydrate derivative binding
GO:0019838//growth factor binding
GO:0005539//glycosaminoglycan binding
GO:0019199//transmembrane receptor protein kinase activity
GO:0004714//transmembrane receptor protein tyrosine kinase activity
GO:0005515//protein binding
GO:0008201//heparin binding
GO:0001848//complement binding
GO:0005488//binding
GO:1901681//sulfur compound binding
GO:0017147//Wnt-protein binding
GO:0001664//G-protein coupled receptor binding
GO:0046332//SMAD binding
GO:0005201//extracellular matrix structural constituent
GO:0038024//cargo receptor activity
GO:0008237//metallopeptidase activity
GO:0030881//beta-2-microglobulin binding
GO:NM_0059
GO:0005509//calcium ion binding
GO:0004175//endopeptidase activity
GO:0004713//protein tyrosine kinase activity
GO:0048407//platelet-derived growth factor binding
GO:0008376//acetylgalactosaminyltransferase activity
GO:0070011//peptidase activity, acting on L-amino acid peptides
GO:0004033//aldo-keto reductase (NADP) activity
GO:0004222//metalloendopeptidase activity
GO:0008106//alcohol dehydrogenase (NADP+) activity
GO:0008233//peptidase activity
GO:0016705//oxidoreductase activity, acting on paired donors, with incorporation or reduction of molecular o
GO:0070851//growth factor receptor binding
GO:0070412//R-SMAD binding
GO:0004029//aldehyde dehydrogenase (NAD) activity
GO:0004653//polypeptide N-acetylgalactosaminyltransferase activity
GO:0004784//superoxide dismutase activity
GO:0016721//oxidoreductase activity, acting on superoxide radicals as acceptor
GO:0004252//serine-type endopeptidase activity
GO:0031690//adrenergic receptor binding
GO:0046703//natural killer cell lectin-like receptor binding
GO:0005044//scavenger receptor activity
GO:0008083//growth factor activity
GO:0017171//serine hydrolase activity
GO:0016773//phosphotransferase activity, alcohol group as acceptor
GO:0003824//catalytic activity
GO:0032052//bile acid binding
GO:0019955//cytokine binding
GO:0051213//dioxygenase activity
GO:0030228//lipoprotein particle receptor activity
GO:0016491//oxidoreductase activity
GO:0019899//enzyme binding
GO:0016641//oxidoreductase activity, acting on the CH-NH2 group of donors, oxygen as acceptor
GO:0004030//aldehyde dehydrogenase [NAD(P)+] activity
GO:0004032//alditol:NADP+ 1-oxidoreductase activity
GO:0016301//kinase activity
GO:0016740//transferase activity
GO:0008236//serine-type peptidase activity

GO:0004672//protein kinase activity
GO:0035035//histone acetyltransferase binding
GO:0016702//oxidoreductase activity, acting on single donors with incorporation of molecular oxygen, incorpo
GO:0005507//copper ion binding
GO:0016701//oxidoreductase activity, acting on single donors with incorporation of molecular oxygen
GO:0016638//oxidoreductase activity, acting on the CH-NH2 group of donors
GO:0005021//vascular endothelial growth factor-activated receptor activity
GO:0031543//peptidyl-proline dioxygenase activity
GO:NR_0014
GO:0008484//sulfuric ester hydrolase activity
GO:0043169//cation binding
GO:0046872//metal ion binding
GO:0042802//identical protein binding
GO:0005126//cytokine receptor binding
GO:0042813//Wnt-activated receptor activity
GO:0017154//semaphorin receptor activity
GO:0005518//collagen binding
GO:0019200//carbohydrate kinase activity
GO:0043167//ion binding
GO:0042803//protein homodimerization activity
GO:0005041//low-density lipoprotein receptor activity
GO:0008194//UDP-glycosyltransferase activity
GO:0043425//bHLH transcription factor binding
GO:0019902//phosphatase binding
GO:0001085//RNA polymerase II transcription factor binding
GO:0019825//oxygen binding
GO:0004866//endopeptidase inhibitor activity
GO:0005161//platelet-derived growth factor receptor binding
GO:0050750//low-density lipoprotein particle receptor binding
GO:0046983//protein dimerization activity
GO:0005520//insulin-like growth factor binding
GO:0061135//endopeptidase regulator activity
GO:0044212//transcription regulatory region DNA binding
GO:0003674//molecular_function
GO:0002020//protease binding
GO:0001191//RNA polymerase II transcription factor binding transcription factor activity involved in negative r
GO:0030414//peptidase inhibitor activity
GO:0004958//prostaglandin F receptor activity
GO:0005004//GPI-linked ephrin receptor activity
GO:0005127//ciliary neurotrophic factor receptor binding
GO:0005536//glucose binding
GO:0008061//chitin binding
GO:0019966//interleukin-1 binding
GO:0045118//azole transporter activity
GO:1901474//azole transmembrane transporter activity
GO:0005540//hyaluronic acid binding
GO:0019903//protein phosphatase binding
GO:0001106//RNA polymerase II transcription corepressor activity
GO:0004089//carbonate dehydratase activity
GO:0000975//regulatory region DNA binding
GO:0001067//regulatory region nucleic acid binding
GO:0003779//actin binding
GO:0016709//oxidoreductase activity, acting on paired donors, with incorporation or reduction of molecular ox
GO:0004012//phospholipid-translocating ATPase activity
GO:0005160//transforming growth factor beta receptor binding
GO:0016209//antioxidant activity
GO:0016772//transferase activity, transferring phosphorus-containing groups
GO:0001102//RNA polymerase II activating transcription factor binding

GO:0005544//calcium-dependent phospholipid binding
GO:0042826//histone deacetylase binding
GO:0005138//interleukin-6 receptor binding
GO:0030215//semaphorin receptor binding
GO:0031545//peptidyl-proline 4-dioxygenase activity
GO:0043121//neurotrophin binding
GO:0045236//CXCR chemokine receptor binding
GO:0050656//3-phosphoadenosine 5-phosphosulfate binding
GO:0016903//oxidoreductase activity, acting on the aldehyde or oxo group of donors
GO:0004867//serine-type endopeptidase inhibitor activity
GO:0000287//magnesium ion binding
GO:0008395//steroid hydroxylase activity
GO:0070325//lipoprotein particle receptor binding
GO:0004497//monooxygenase activity
GO:0004857//enzyme inhibitor activity
GO:0034185//apolipoprotein binding
GO:0048029//monosaccharide binding
GO:0004017//adenylate kinase activity
GO:0004115//3,5-cyclic-AMP phosphodiesterase activity
GO:0004568//chitinase activity
GO:0008443//phosphofructokinase activity
GO:0019798//procollagen-proline dioxygenase activity
GO:0035251//UDP-glucosyltransferase activity
GO:0016706//oxidoreductase activity, acting on paired donors, with incorporation or reduction of molecular ox
GO:0016620//oxidoreductase activity, acting on the aldehyde or oxo group of donors, NAD or NADP as accep
GO:0043168//anion binding
GO:0008009//chemokine activity
GO:0005154//epidermal growth factor receptor binding
GO:0090484//drug transporter activity
GO:0005109//frizzled binding
GO:0004872//receptor activity
GO:0031406//carboxylic acid binding
GO:0020037//heme binding
GO:0016757//transferase activity, transferring glycosyl groups
GO:0046982//protein heterodimerization activity
GO:0001076//RNA polymerase II transcription factor binding transcription factor activity
GO:0004716//receptor signaling protein tyrosine kinase activity
GO:0005172//vascular endothelial growth factor receptor binding
GO:0003714//transcription corepressor activity
GO:0061134//peptidase regulator activity
GO:0030674//protein binding, bridging
GO:0031418//L-ascorbic acid binding
GO:0033293//monocarboxylic acid binding
GO:0008146//sulfotransferase activity
GO:0003810//protein-glutamine gamma-glutamyltransferase activity
GO:0005005//transmembrane-ephrin receptor activity
GO:0019534//toxin transporter activity
GO:0046527//glucosyltransferase activity
GO:0016758//transferase activity, transferring hexosyl groups
GO:0030145//manganese ion binding
GO:0008373//sialyltransferase activity
GO:0046906//tetrapyrrole binding
GO:0008092//cytoskeletal protein binding
GO:0042379//chemokine receptor binding
GO:0008307//structural constituent of muscle
GO:0004708//MAP kinase kinase activity
GO:0004955//prostaglandin receptor activity
GO:0005243//gap junction channel activity

GO:0070410//co-SMAD binding
GO:0000976//transcription regulatory region sequence-specific DNA binding
GO:0005506//iron ion binding
GO:0004114//3,5-cyclic-nucleotide phosphodiesterase activity
GO:0019843//rRNA binding
GO:0005057//receptor signaling protein activity
GO:0001540//beta-amyloid binding
GO:0004112//cyclic-nucleotide phosphodiesterase activity
GO:0004623//phospholipase A2 activity
GO:0016628//oxidoreductase activity, acting on the CH-CH group of donors, NAD or NADP as acceptor
GO:0004954//prostanoid receptor activity
GO:0042301//phosphate ion binding
GO:0005178//integrin binding
GO:0000987//core promoter proximal region sequence-specific DNA binding
GO:0060090//binding, bridging
GO:0005158//insulin receptor binding
GO:0046875//ephrin receptor binding
GO:0004221//ubiquitin thiolesterase activity
GO:0008528//G-protein coupled peptide receptor activity
GO:0001159//core promoter proximal region DNA binding
GO:0004601//peroxidase activity
GO:0016684//oxidoreductase activity, acting on peroxide as acceptor
GO:0033613//activating transcription factor binding
GO:0004065//arylsulfatase activity
GO:0008191//metalloendopeptidase inhibitor activity
GO:0010576//metalloenzyme regulator activity
GO:0048018//receptor agonist activity
GO:0048551//metalloenzyme inhibitor activity
GO:0019904//protein domain specific binding
GO:0070888//E-box binding
GO:0001104//RNA polymerase II transcription cofactor activity
GO:0004702//receptor signaling protein serine/threonine kinase activity
GO:0005070//SH3/SH2 adaptor activity
GO:0000982//RNA polymerase II core promoter proximal region sequence-specific DNA binding transcription
GO:0001653//peptide receptor activity
GO:0016836//hydro-lyase activity
GO:0000978//RNA polymerase II core promoter proximal region sequence-specific DNA binding
GO:0043621//protein self-association
GO:0005516//calmodulin binding
GO:0004622//lysophospholipase activity
GO:0022829//wide pore channel activity
GO:0051721//protein phosphatase 2A binding
GO:0005548//phospholipid transporter activity
GO:0016782//transferase activity, transferring sulfur-containing groups
GO:0004896//cytokine receptor activity
GO:0050839//cell adhesion molecule binding
GO:0003682//chromatin binding
GO:0004953//icosanoid receptor activity
GO:0008601//protein phosphatase type 2A regulator activity
GO:0048020//CCR chemokine receptor binding
GO:0004871//signal transducer activity
GO:0060089//molecular transducer activity
GO:0005179//hormone activity
GO:0001078//RNA polymerase II core promoter proximal region sequence-specific DNA binding transcription
GO:0005080//protein kinase C binding
GO:0004620//phospholipase activity
GO:0030246//carbohydrate binding
GO:0001871//pattern binding

GO:0003756//protein disulfide isomerase activity
GO:0016864//intramolecular oxidoreductase activity, transposing S-S bonds
GO:0030247//polysaccharide binding
GO:0043492//ATPase activity, coupled to movement of substances
GO:0016860//intramolecular oxidoreductase activity
GO:0030165//PDZ domain binding
GO:0004675//transmembrane receptor protein serine/threonine kinase activity
GO:0004709//MAP kinase kinase kinase activity
GO:0005024//transforming growth factor beta-activated receptor activity
GO:0016862//intramolecular oxidoreductase activity, interconverting keto- and enol-groups
GO:0043548//phosphatidylinositol 3-kinase binding
GO:0001227//RNA polymerase II transcription regulatory region sequence-specific DNA binding transcription
GO:0030545//receptor regulator activity
GO:0050840//extracellular matrix binding
GO:0004806//triglyceride lipase activity
GO:0005003//ephrin receptor activity
GO:0017134//fibroblast growth factor binding
GO:0000988//protein binding transcription factor activity
GO:0001948//glycoprotein binding
GO:0019900//kinase binding
GO:0019842//vitamin binding
GO:0042056//chemoattractant activity
GO:0051183//vitamin transporter activity
GO:0032403//protein complex binding
GO:0000989//transcription factor binding transcription factor activity
GO:0070491//repressing transcription factor binding
GO:0015291//secondary active transmembrane transporter activity
GO:0035591//signaling adaptor activity
GO:0043130//ubiquitin binding
GO:0019201//nucleotide kinase activity
GO:0031490//chromatin DNA binding
GO:0042277//peptide binding
GO:0003712//transcription cofactor activity
GO:0016835//carbon-oxygen lyase activity
GO:0000014//single-stranded DNA specific endodeoxyribonuclease activity
GO:0001517//N-acetylglucosamine 6-O-sulfotransferase activity
GO:0001846//opsonin binding
GO:0004028//3-chloroallyl aldehyde dehydrogenase activity
GO:0004340//glucokinase activity
GO:0004366//glycerol-3-phosphate O-acyltransferase activity
GO:0004396//hexokinase activity
GO:0004630//phospholipase D activity
GO:0004634//phosphopyruvate hydratase activity
GO:0004679//AMP-activated protein kinase activity
GO:0004936//alpha-adrenergic receptor activity
GO:0005007//fibroblast growth factor-activated receptor activity
GO:0008131//primary amine oxidase activity
GO:0008467//[heparan sulfate]-glucosamine 3-sulfotransferase 1 activity
GO:0008517//folic acid transporter activity
GO:0008865//fructokinase activity
GO:0015174//basic amino acid transmembrane transporter activity
GO:0016413//O-acetyltransferase activity
GO:0019158//mannokinase activity
GO:0031694//alpha-2A adrenergic receptor binding
GO:0032036//myosin heavy chain binding
GO:0032453//histone demethylase activity (H3-K4 specific)
GO:0035252//UDP-xylosyltransferase activity
GO:0035259//glucocorticoid receptor binding

GO:0042285//xylosyltransferase activity
GO:0045499//chemorepellent activity
GO:0048406//nerve growth factor binding
GO:0050998//nitric-oxide synthase binding
GO:0055103//ligase regulator activity
GO:NM_0145
GO:NM_0211
GO:NR_0021
GO:0008238//exopeptidase activity
GO:0001968//fibronectin binding
GO:0019956//chemokine binding
GO:0045296//cadherin binding
GO:0003690//double-stranded DNA binding
GO:0033218//amide binding
GO:0046943//carboxylic acid transmembrane transporter activity
GO:0004843//ubiquitin-specific protease activity
GO:0005125//cytokine activity
GO:0071813//lipoprotein particle binding
GO:0071814//protein-lipid complex binding
GO:1901682//sulfur compound transmembrane transporter activity
GO:0005319//lipid transporter activity
GO:0008301//DNA binding, bending
GO:0016298//lipase activity
GO:0017124//SH3 domain binding
GO:0005342//organic acid transmembrane transporter activity
GO:0015179//L-amino acid transmembrane transporter activity
GO:0016755//transferase activity, transferring amino-acyl groups
GO:0003916//DNA topoisomerase activity
GO:0004035//alkaline phosphatase activity
GO:0004128//cytochrome-b5 reductase activity, acting on NAD(P)H
GO:0004322//ferroxidase activity
GO:0004331//fructose-2,6-bisphosphate 2-phosphatase activity
GO:0004645//phosphorylase activity
GO:0005072//transforming growth factor beta receptor, cytoplasmic mediator activity
GO:0005114//type II transforming growth factor beta receptor binding
GO:0008035//high-density lipoprotein particle binding
GO:0008140//cAMP response element binding protein binding
GO:0008179//adenylate cyclase binding
GO:0016165//linoleate 13S-lipoxygenase activity
GO:0016174//NAD(P)H oxidase activity
GO:0016595//glutamate binding
GO:0016670//oxidoreductase activity, acting on a sulfur group of donors, oxygen as acceptor
GO:0016724//oxidoreductase activity, oxidizing metal ions, oxygen as acceptor
GO:0017081//chloride channel regulator activity
GO:0030955//potassium ion binding
GO:0031702//type 1 angiotensin receptor binding
GO:0031994//insulin-like growth factor I binding
GO:0035014//phosphatidylinositol 3-kinase regulator activity
GO:0043237//laminin-1 binding
GO:0043426//MRF binding
GO:0050291//sphingosine N-acyltransferase activity
GO:0050780//dopamine receptor binding
GO:0070087//chromo shadow domain binding
GO:0070097//delta-catenin binding
GO:0070492//oligosaccharide binding
GO:NM_0018
GO:NM_0048
GO:NM_0066

GO:NM_0203
GO:0008234//cysteine-type peptidase activity
GO:0008514//organic anion transmembrane transporter activity
GO:0015026//coreceptor activity
GO:0032559//adenyl ribonucleotide binding
GO:0072341//modified amino acid binding
GO:0016790//thiolester hydrolase activity
GO:0030554//adenyl nucleotide binding
GO:0001637//G-protein coupled chemoattractant receptor activity
GO:0004950//chemokine receptor activity
GO:0030546//receptor activator activity
GO:0000155//phosphorelay sensor kinase activity
GO:0004908//interleukin-1 receptor activity
GO:0008046//axon guidance receptor activity
GO:0016208//AMP binding
GO:0016494//C-X-C chemokine receptor activity
GO:0016671//oxidoreductase activity, acting on a sulfur group of donors, disulfide as acceptor
GO:0030898//actin-dependent ATPase activity
GO:0031701//angiotensin receptor binding
GO:0031996//thioesterase binding
GO:0045294//alpha-catenin binding
GO:0047498//calcium-dependent phospholipase A2 activity
GO:NM_0003
GO:0004674//protein serine/threonine kinase activity
GO:0019783//small conjugating protein-specific protease activity
GO:0019901//protein kinase binding
GO:0022804//active transmembrane transporter activity
GO:0030295//protein kinase activator activity
GO:0043236//laminin binding
GO:0008374//O-acyltransferase activity
GO:0032182//small conjugating protein binding
GO:0008081//phosphoric diester hydrolase activity
GO:0008134//transcription factor binding
GO:0009055//electron carrier activity
GO:0016614//oxidoreductase activity, acting on CH-OH group of donors
GO:0051015//actin filament binding
GO:0016655//oxidoreductase activity, acting on NAD(P)H, quinone or similar compound as acceptor
GO:0015020//glucuronosyltransferase activity
GO:0017022//myosin binding
GO:0038023//signaling receptor activity
GO:0000983//RNA polymerase II core promoter sequence-specific DNA binding transcription factor activity
GO:0003964//RNA-directed DNA polymerase activity
GO:0004126//cytidine deaminase activity
GO:0004673//protein histidine kinase activity
GO:0005095//GTPase inhibitor activity
GO:0008253//5-nucleotidase activity
GO:0008271//secondary active sulfate transmembrane transporter activity
GO:0016175//superoxide-generating NADPH oxidase activity
GO:0016832//aldehyde-lyase activity
GO:0019206//nucleoside kinase activity
GO:0030296//protein tyrosine kinase activator activity
GO:0030547//receptor inhibitor activity
GO:0048019//receptor antagonist activity
GO:0050786//RAGE receptor binding
GO:0070411//I-SMAD binding
GO:0071949//FAD binding
GO:NM_0032
GO:NM_0142

GO:0005215//transporter activity
GO:0015171//amino acid transmembrane transporter activity
GO:0016853//isomerase activity
GO:0004888//transmembrane signaling receptor activity
GO:0004712//protein serine/threonine/tyrosine kinase activity
GO:0019239//deaminase activity
GO:0043566//structure-specific DNA binding
GO:0004197//cysteine-type endopeptidase activity
GO:0005496//steroid binding
GO:0004000//adenosine deaminase activity
GO:0004551//nucleotide diphosphatase activity
GO:0004935//adrenergic receptor activity
GO:0005123//death receptor binding
GO:0005521//lamin binding
GO:0008195//phosphatidate phosphatase activity
GO:0008430//selenium binding
GO:0015643//toxic substance binding
GO:0016857//racemase and epimerase activity, acting on carbohydrates and derivatives
GO:0030507//spectrin binding
GO:0070696//transmembrane receptor protein serine/threonine kinase binding
GO:0071837//HMG box domain binding
GO:NM_0007
GO:NM_0022
GO:NM_0024
GO:0001077//RNA polymerase II core promoter proximal region sequence-specific DNA binding transcription
GO:0001046//core promoter sequence-specific DNA binding
GO:0004177//aminopeptidase activity
GO:0005501//retinoid binding
GO:0019840//isoprenoid binding
GO:0033764//steroid dehydrogenase activity, acting on the CH-OH group of donors, NAD or NADP as accep
GO:0031625//ubiquitin protein ligase binding
GO:0044389//small conjugating protein ligase binding
GO:0005524//ATP binding
GO:0022892//substrate-specific transporter activity
GO:0008013//beta-catenin binding
GO:0016667//oxidoreductase activity, acting on a sulfur group of donors
GO:0035258//steroid hormone receptor binding
GO:0016616//oxidoreductase activity, acting on the CH-OH group of donors, NAD or NADP as acceptor
GO:0004982//N-formyl peptide receptor activity
GO:0008239//dipeptidyl-peptidase activity
GO:0008252//nucleotidase activity
GO:0015175//neutral amino acid transmembrane transporter activity
GO:0015238//drug transmembrane transporter activity
GO:0015296//anion:cation symporter activity
GO:0016303//1-phosphatidylinositol-3-kinase activity
GO:0016812//hydrolase activity, acting on carbon-nitrogen (but not peptide) bonds, in cyclic amides
GO:0017153//sodium:dicarboxylate symporter activity
GO:0019203//carbohydrate phosphatase activity
GO:0031420//alkali metal ion binding
GO:0033612//receptor serine/threonine kinase binding
GO:0050308//sugar-phosphatase activity
GO:0051428//peptide hormone receptor binding
GO:NM_0047
GO:0022857//transmembrane transporter activity
GO:0019209//kinase activator activity
GO:0030971//receptor tyrosine kinase binding
GO:0048306//calcium-dependent protein binding
GO:0019207//kinase regulator activity

GO:0004016//adenylate cyclase activity
GO:0004522//pancreatic ribonuclease activity
GO:0005112//Notch binding
GO:0005159//insulin-like growth factor receptor binding
GO:0005344//oxygen transporter activity
GO:0005372//water transmembrane transporter activity
GO:0015116//sulfate transmembrane transporter activity
GO:0016722//oxidoreductase activity, oxidizing metal ions
GO:0016775//phosphotransferase activity, nitrogenous group as acceptor
GO:0016868//intramolecular transferase activity, phosphotransferases
GO:0016884//carbon-nitrogen ligase activity, with glutamine as amido-N-donor
GO:0017025//TBP-class protein binding
GO:0017166//vinculin binding
GO:0031432//titin binding
GO:0035004//phosphatidylinositol 3-kinase activity
GO:0042166//acetylcholine binding
GO:0045295//gamma-catenin binding
GO:0050431//transforming growth factor beta binding
GO:0070008//serine-type exopeptidase activity
GO:0070403//NAD+ binding
GO:0071855//neuropeptide receptor binding
GO:NM_0037
GO:0000977//RNA polymerase II regulatory region sequence-specific DNA binding
GO:0016229//steroid dehydrogenase activity
GO:0016814//hydrolase activity, acting on carbon-nitrogen (but not peptide) bonds, in cyclic amidines
GO:0043565//sequence-specific DNA binding
GO:0016787//hydrolase activity
GO:0001228//RNA polymerase II transcription regulatory region sequence-specific DNA binding transcription
GO:0016627//oxidoreductase activity, acting on the CH-CH group of donors
GO:0005198//structural molecule activity
GO:0016776//phosphotransferase activity, phosphate group as acceptor
GO:0019003//GDP binding
GO:0001012//RNA polymerase II regulatory region DNA binding
GO:0003708//retinoic acid receptor activity
GO:0004861//cyclin-dependent protein serine/threonine kinase inhibitor activity
GO:0005542//folic acid binding
GO:0016653//oxidoreductase activity, acting on NAD(P)H, heme protein as acceptor
GO:0030159//receptor signaling complex scaffold activity
GO:0034483//heparan sulfate sulfotransferase activity
GO:0035173//histone kinase activity
GO:0048185//activin binding
GO:0071889//14-3-3 protein binding
GO:NM_0068
GO:0005100//Rho GTPase activator activity
GO:0016788//hydrolase activity, acting on ester bonds
GO:0008289//lipid binding
GO:0008509//anion transmembrane transporter activity
GO:0019887//protein kinase regulator activity
GO:0003785//actin monomer binding
GO:0008093//cytoskeletal adaptor activity
GO:0008170//N-methyltransferase activity
GO:0016861//intramolecular oxidoreductase activity, interconverting aldoses and ketoses
GO:0017110//nucleoside-diphosphatase activity
GO:0017127//cholesterol transporter activity
GO:0042043//neurexin family protein binding
GO:0050664//oxidoreductase activity, acting on NAD(P)H, oxygen as acceptor
GO:0051184//cofactor transporter activity
GO:0052742//phosphatidylinositol kinase activity

GO:0005200//structural constituent of cytoskeleton
GO:0005149//interleukin-1 receptor binding
GO:0005545//1-phosphatidylinositol binding
GO:0015248//sterol transporter activity
GO:0015464//acetylcholine receptor activity
GO:0016854//racemase and epimerase activity
GO:0016892//endoribonuclease activity, producing 3-phosphomonoesters
GO:0050253//retinyl-palmitate esterase activity
GO:1901338//catecholamine binding
GO:1901677//phosphate transmembrane transporter activity
GO:0008757//S-adenosylmethionine-dependent methyltransferase activity
GO:0015293//symporter activity
GO:0008022//protein C-terminus binding
GO:0016651//oxidoreductase activity, acting on NAD(P)H
GO:0000981//sequence-specific DNA binding RNA polymerase II transcription factor activity
GO:0004143//diacylglycerol kinase activity
GO:0032452//histone demethylase activity
GO:0043395//heparan sulfate proteoglycan binding
GO:0004860//protein kinase inhibitor activity
GO:0008235//metalloexopeptidase activity
GO:0030234//enzyme regulator activity
GO:0016597//amino acid binding
GO:0001786//phosphatidylserine binding
GO:0001972//retinoic acid binding
GO:0004198//calcium-dependent cysteine-type endopeptidase activity
GO:0005104//fibroblast growth factor receptor binding
GO:0016922//ligand-dependent nuclear receptor binding
GO:0030552//cAMP binding
GO:0050699//WW domain binding
GO:0017137//Rab GTPase binding
GO:0019205//nucleobase-containing compound kinase activity
GO:0032555//purine ribonucleotide binding
GO:0003713//transcription coactivator activity
GO:0032550//purine ribonucleoside binding
GO:0052689//carboxylic ester hydrolase activity
GO:0008324//cation transmembrane transporter activity
GO:0050660//flavin adenine dinucleotide binding
GO:0001103//RNA polymerase II repressing transcription factor binding
GO:0005527//macrolide binding
GO:0005528//FK506 binding
GO:0008242//omega peptidase activity
GO:0008603//cAMP-dependent protein kinase regulator activity
GO:0030276//clathrin binding
GO:0042165//neurotransmitter binding
GO:0043027//cysteine-type endopeptidase inhibitor activity involved in apoptotic process
GO:0097110//scaffold protein binding
GO:0001883//purine nucleoside binding
GO:0019210//kinase inhibitor activity
GO:0044325//ion channel binding
GO:0032549//ribonucleoside binding
GO:0017076//purine nucleotide binding
GO:0005543//phospholipid binding
GO:0022891//substrate-specific transmembrane transporter activity
GO:0042578//phosphoric ester hydrolase activity
GO:0004602//glutathione peroxidase activity
GO:0005086//ARF guanyl-nucleotide exchange factor activity
GO:0005537//mannose binding
GO:0030331//estrogen receptor binding

GO:0043015//gamma-tubulin binding
GO:0043394//proteoglycan binding
GO:0032553//ribonucleotide binding
GO:0001882//nucleoside binding
GO:0001047//core promoter binding
GO:0005164//tumor necrosis factor receptor binding
GO:0008138//protein tyrosine/serine/threonine phosphatase activity
GO:0019888//protein phosphatase regulator activity
GO:0001105//RNA polymerase II transcription coactivator activity
GO:0048487//beta-tubulin binding
GO:0003705//RNA polymerase II distal enhancer sequence-specific DNA binding transcription factor activity
GO:0003684//damaged DNA binding
GO:0000146//microfilament motor activity
GO:0015103//inorganic anion transmembrane transporter activity
GO:0051018//protein kinase A binding
GO:1901505//carbohydrate derivative transporter activity
GO:0008144//drug binding
GO:0004879//ligand-activated sequence-specific DNA binding RNA polymerase II transcription factor activity
GO:0046873//metal ion transmembrane transporter activity
GO:0042562//hormone binding
GO:0000979//RNA polymerase II core promoter sequence-specific DNA binding
GO:0005310//dicarboxylic acid transmembrane transporter activity
GO:0008227//G-protein coupled amine receptor activity
GO:0008483//transaminase activity
GO:0016538//cyclin-dependent protein serine/threonine kinase regulator activity
GO:0016799//hydrolase activity, hydrolyzing N-glycosyl compounds
GO:0016849//phosphorus-oxygen lyase activity
GO:0030291//protein serine/threonine kinase inhibitor activity
GO:NM_0252
GO:0008168//methyltransferase activity
GO:0035639//purine ribonucleoside triphosphate binding
GO:0016829//lyase activity
GO:0016746//transferase activity, transferring acyl groups
GO:0002039//p53 binding
GO:0003707//steroid hormone receptor activity
GO:0008017//microtubule binding
GO:0009975//cyclase activity
GO:0016411//acylglycerol O-acyltransferase activity
GO:0031683//G-protein beta/gamma-subunit complex binding
GO:0032451//demethylase activity
GO:0035254//glutamate receptor binding
GO:0042887//amide transmembrane transporter activity
GO:0070330//aromatase activity
GO:0015075//ion transmembrane transporter activity
GO:0016763//transferase activity, transferring pentosyl groups
GO:0042626//ATPase activity, coupled to transmembrane movement of substances
GO:0005343//organic acid:sodium symporter activity
GO:0016769//transferase activity, transferring nitrogenous groups
GO:0022890//inorganic cation transmembrane transporter activity
GO:0016741//transferase activity, transferring one-carbon groups
GO:0004842//ubiquitin-protein ligase activity
GO:0032813//tumor necrosis factor receptor superfamily binding
GO:0004129//cytochrome-c oxidase activity
GO:0004181//metallocarboxypeptidase activity
GO:0005504//fatty acid binding
GO:0015002//heme-copper terminal oxidase activity
GO:0016675//oxidoreductase activity, acting on a heme group of donors
GO:0016676//oxidoreductase activity, acting on a heme group of donors, oxygen as acceptor

GO:0016894//endonuclease activity, active with either ribo- or deoxyribonucleic acids and producing 3-phosp
GO:0016765//transferase activity, transferring alkyl or aryl (other than methyl) groups
GO:0019208//phosphatase regulator activity
GO:0016747//transferase activity, transferring acyl groups other than amino-acyl groups
GO:0001190//RNA polymerase II transcription factor binding transcription factor activity involved in positive re
GO:0003950//NAD+ ADP-ribosyltransferase activity
GO:0005244//voltage-gated ion channel activity
GO:0008028//monocarboxylic acid transmembrane transporter activity
GO:0022832//voltage-gated channel activity
GO:0050997//quaternary ammonium group binding
GO:0017016//Ras GTPase binding
GO:0030170//pyridoxal phosphate binding
GO:0048037//cofactor binding
GO:0016879//ligase activity, forming carbon-nitrogen bonds
GO:0004693//cyclin-dependent protein serine/threonine kinase activity
GO:0016712//oxidoreductase activity, acting on paired donors, with incorporation or reduction of molecular ox
GO:0030594//neurotransmitter receptor activity
GO:0097472//cyclin-dependent protein kinase activity
GO:0015399//primary active transmembrane transporter activity
GO:0015405//P-P-bond-hydrolysis-driven transmembrane transporter activity
GO:0035091//phosphatidylinositol binding
GO:0005546//phosphatidylinositol-4,5-bisphosphate binding
GO:0015035//protein disulfide oxidoreductase activity
GO:0030551//cyclic nucleotide binding
GO:0042605//peptide antigen binding
GO:NM_0010
GO:0004435//phosphatidylinositol phospholipase C activity
GO:0019787//small conjugating protein ligase activity
GO:0016866//intramolecular transferase activity
GO:0016820//hydrolase activity, acting on acid anhydrides, catalyzing transmembrane movement of substanc
GO:0004629//phospholipase C activity
GO:0008060//ARF GTPase activator activity
GO:0017046//peptide hormone binding
GO:0019905//syntaxin binding
GO:0015267//channel activity
GO:0022803//passive transmembrane transporter activity
GO:0042623//ATPase activity, coupled
GO:0000049//tRNA binding
GO:0042169//SH2 domain binding
GO:0031267//small GTPase binding
GO:0015631//tubulin binding
GO:0016791//phosphatase activity
GO:0004520//endodeoxyribonuclease activity
GO:0015036//disulfide oxidoreductase activity
GO:0015078//hydrogen ion transmembrane transporter activity
GO:0015485//cholesterol binding
GO:0046914//transition metal ion binding
GO:0016798//hydrolase activity, acting on glycosyl bonds
GO:0051427//hormone receptor binding
GO:0050681//androgen receptor binding
GO:0004091//carboxylesterase activity
GO:0043028//cysteine-type endopeptidase regulator activity involved in apoptotic process
GO:0004553//hydrolase activity, hydrolyzing O-glycosyl compounds
GO:0016887//ATPase activity
GO:0030374//ligand-dependent nuclear receptor transcription coactivator activity
GO:0051020//GTPase binding
GO:0004003//ATP-dependent DNA helicase activity
GO:0035064//methylated histone residue binding

GO:0004180//carboxypeptidase activity
GO:0032947//protein complex scaffold
GO:0034061//DNA polymerase activity
GO:0005089//Rho guanyl-nucleotide exchange factor activity
GO:0032934//sterol binding
GO:0004519//endonuclease activity
GO:0008565//protein transporter activity
GO:0005096//GTPase activator activity
GO:0003725//double-stranded RNA binding
GO:0051087//chaperone binding
GO:0008094//DNA-dependent ATPase activity
GO:0005099//Ras GTPase activator activity
GO:0000149//SNARE binding
GO:0036094//small molecule binding
GO:0015077//monovalent inorganic cation transmembrane transporter activity
GO:0003755//peptidyl-prolyl cis-trans isomerase activity
GO:0016881//acid-amino acid ligase activity
GO:0000166//nucleotide binding
GO:1901265//nucleoside phosphate binding
GO:0035257//nuclear hormone receptor binding
GO:0004715//non-membrane spanning protein tyrosine kinase activity
GO:0016859//cis-trans isomerase activity
GO:0051287//NAD binding
GO:0003823//antigen binding
GO:0004536//deoxyribonuclease activity
GO:0004869//cysteine-type endopeptidase inhibitor activity
GO:0015081//sodium ion transmembrane transporter activity
GO:0015294//solute:cation symporter activity
GO:0015370//solute:sodium symporter activity
GO:0016830//carbon-carbon lyase activity
GO:0003700//sequence-specific DNA binding transcription factor activity
GO:0004521//endoribonuclease activity
GO:0022836//gated channel activity
GO:0022839//ion gated channel activity
GO:0001071//nucleic acid binding transcription factor activity
GO:0015276//ligand-gated ion channel activity
GO:0022834//ligand-gated channel activity
GO:0031072//heat shock protein binding
GO:NM_0011
GO:0016407//acetyltransferase activity
GO:0016410//N-acyltransferase activity
GO:0016810//hydrolase activity, acting on carbon-nitrogen (but not peptide) bonds
GO:0003678//DNA helicase activity
GO:0017048//Rho GTPase binding
GO:0051082//unfolded protein binding
GO:0005097//Rab GTPase activator activity
GO:NM_0012
GO:0005083//small GTPase regulator activity
GO:0004812//aminoacyl-tRNA ligase activity
GO:0016875//ligase activity, forming carbon-oxygen bonds
GO:0016876//ligase activity, forming aminoacyl-tRNA and related compounds
GO:0005216//ion channel activity
GO:0030695//GTPase regulator activity
GO:1901981//phosphatidylinositol phosphate binding
GO:0060589//nucleoside-triphosphatase regulator activity
GO:ENST000
GO:0016247//channel regulator activity
GO:0003924//GTPase activity

GO:0042393//histone binding
GO:0050662//coenzyme binding
GO:0022838//substrate-specific channel activity
GO:0016874//ligase activity
GO:0043178//alcohol binding
GO:0005088//Ras guanyl-nucleotide exchange factor activity
GO:0005525//GTP binding
GO:0008047//enzyme activator activity
GO:0003774//motor activity
GO:0019001//guanyl nucleotide binding
GO:0032561//guanyl ribonucleotide binding
GO:0004721//phosphoprotein phosphatase activity
GO:0004540//ribonuclease activity
GO:0047485//protein N-terminus binding
GO:0003729//mRNA binding
GO:0008026//ATP-dependent helicase activity
GO:0070035//purine NTP-dependent helicase activity
GO:0017111//nucleoside-triphosphatase activity
GO:0005085//guanyl-nucleotide exchange factor activity
GO:0016462//pyrophosphatase activity
GO:0016818//hydrolase activity, acting on acid anhydrides, in phosphorus-containing anhydrides
GO:0004518//nuclease activity
GO:0008270//zinc ion binding
GO:0003735//structural constituent of ribosome
GO:0016817//hydrolase activity, acting on acid anhydrides
GO:0004386//helicase activity
GO:0004930//G-protein coupled receptor activity
GO:0016779//nucleotidyltransferase activity
GO:0003677//DNA binding
GO:0003723//RNA binding
GO:0097159//organic cyclic compound binding
GO:1901363//heterocyclic compound binding
GO:0003676//nucleic acid binding

Genes_In_Term	DEG	Input_gene	Gene_Symbol	P_value	Q_value	Significant
16391	645	-	-	-	-	-
1140	87	TC01000904.hg.1	ENSG00000162692;	1.484E-09	8.419E-07	TRUE
180	27	TC01000972.hg.1	ENSG00000064886;	2.145E-09	8.419E-07	TRUE
94	19	TC01002954.hg.1	ENSG00000134243;	3.515E-09	9.198E-07	TRUE
163	25	TC02000704.hg.1	ENSG00000144152;	5.315E-09	1.043E-06	TRUE
81	17	TC01000723.hg.1	ENSG00000185483;	1.291E-08	2.027E-06	TRUE
65	15	TC01000723.hg.1	ENSG00000185483;	2.382E-08	3.117E-06	TRUE
6571	324	TC01000394.hg.1	ENSG00000159023;	6.73E-08	7.548E-06	TRUE
121	19	TC02000704.hg.1	ENSG00000144152;	2.498E-07	2.451E-05	TRUE
14	7	TC06004077.hg.1	ENSG00000243649;	3.814E-07	3.327E-05	TRUE
11232	496	TC01000394.hg.1	ENSG00000159023;	1.019E-06	8.001E-05	TRUE
160	21	TC02000704.hg.1	ENSG00000144152;	1.278E-06	9.122E-05	TRUE
27	8	TC01000723.hg.1	ENSG00000185483;	6.29E-06	0.0004115	TRUE
157	19	TC04000253.hg.1	ENSG00000154277;	1.341E-05	0.0008099	TRUE
51	10	TC02002166.hg.1	ENSG00000135966;	2.463E-05	0.001381	TRUE
54	10	TC01003718.hg.1	ENSG00000133048;	4.147E-05	0.0021705	TRUE
56	10	TC01001603.hg.1	ENSG00000116690;I	5.749E-05	0.0028207	TRUE
175	19	TC02002480.hg.1	ENSG00000078098;	6.157E-05	0.0028432	TRUE
7	4	TC06004073.hg.1	ENSG00000204520;	7.562E-05	0.0032087	TRUE
7	4	TC16000474.hg.1	ENSG00000205358;	7.562E-05	0.0032087	TRUE
614	44	TC01001607.hg.1	ENSG00000116711;I	9.38E-05	0.0036818	TRUE
398	32	TC01003211.hg.1	ENSG00000143387;	0.0001088	0.0040674	TRUE
139	16	TC01000723.hg.1	ENSG00000185483;	0.0001158	0.004133	TRUE
8	4	TC04002931.hg.1	ENSG00000134853;	0.0001465	0.0050009	TRUE
31	7	TC01001907.hg.1	ENSG00000143641;	0.0001629	0.0053273	TRUE
576	41	TC01003211.hg.1	ENSG00000143387;	0.0001867	0.0058625	TRUE
15	5	TC07001883.hg.1	ENSG00000085662;	0.0002008	0.0060624	TRUE
104	13	TC02002480.hg.1	ENSG00000078098;	0.0002187	0.0063582	TRUE
9	4	TC07001883.hg.1	ENSG00000085662;	0.0002555	0.0067764	TRUE
604	42	TC01003211.hg.1	ENSG00000143387;	0.0002572	0.0067764	TRUE
164	17	TC01003638.hg.1	ENSG00000073756;	0.000259	0.0067764	TRUE
93	12	TC01001188.hg.1	ENSG00000143369;	0.0002808	0.0071118	TRUE
17	5	TC03000214.hg.1	ENSG00000168036;	0.0003875	0.0095062	TRUE
10	4	TC09000222.hg.1	ENSG00000137124;	0.0004126	0.009816	TRUE
18	5	TC01001907.hg.1	ENSG00000143641;	0.0005193	0.0119896	TRUE
5	3	TC04000173.hg.1	ENSG00000109610;	0.0005715	0.0126382	TRUE
5	3	TC04000173.hg.1	ENSG00000109610;	0.0005715	0.0126382	TRUE
161	16	TC02002480.hg.1	ENSG00000078098;	0.0006218	0.0129796	TRUE
11	4	TC04000253.hg.1	ENSG00000154277;	0.0006283	0.0129796	TRUE
19	5	TC06001089.hg.1	ENSG00000111981;I	0.0006821	0.0137295	TRUE
39	7	TC01001603.hg.1	ENSG00000116690;I	0.0007251	0.0142294	TRUE
134	14	TC01001805.hg.1	ENSG00000092969;	0.000816	0.0156229	TRUE
185	17	TC02002480.hg.1	ENSG00000078098;	0.0010366	0.0193567	TRUE
708	45	TC01000685.hg.1	ENSG00000162409;	0.0010653	0.0193567	TRUE
5273	244	TC01000643.hg.1	ENSG00000116157;I	0.0011056	0.0193567	TRUE
6	3	TC10000986.hg.1	ENSG00000151632;	0.0011096	0.0193567	TRUE
67	9	TC02001100.hg.1	ENSG00000138448;	0.0011899	0.0203054	TRUE
81	10	TC01003638.hg.1	ENSG00000073756;	0.0012568	0.0208922	TRUE
13	4	TC01002669.hg.1	ENSG00000157193;	0.0012783	0.0208922	TRUE
695	44	TC01000643.hg.1	ENSG00000116157;I	0.0013041	0.0208922	TRUE
973	57	TC01001090.hg.1	ENSG00000117289;I	0.0016809	0.0263901	TRUE
14	4	TC02002002.hg.1	ENSG00000115318;I	0.0017343	0.0266944	TRUE
7	3	TC15000971.hg.1	ENSG00000184254;	0.0018851	0.0278388	TRUE
7	3	TC07001883.hg.1	ENSG00000085662;	0.0018851	0.0278388	TRUE
853	51	TC01000685.hg.1	ENSG00000162409;	0.001915	0.0278388	TRUE
1747	92	TC01000685.hg.1	ENSG00000162409;	0.0021454	0.0306209	TRUE
182	16	TC02002480.hg.1	ENSG00000078098;	0.0022708	0.03102	TRUE

594	38	TC01000685.hg.1	ENSG00000162409;	0.0022784	0.03102	TRUE
15	4	TC02000281.hg.1	ENSG00000116016;l	0.0022919	0.03102	TRUE
74	9	TC01003638.hg.1	ENSG00000073756;	0.002412	0.0320917	TRUE
48	7	TC02002002.hg.1	ENSG00000115318;l	0.0025573	0.0334581	TRUE
75	9	TC01003638.hg.1	ENSG00000073756;	0.0026475	0.0340707	TRUE
17	4	TC02002002.hg.1	ENSG00000115318;l	0.0037537	0.0475271	TRUE
9	3	TC02001206.hg.1	ENSG00000118257;l	0.0042646	0.0523086	FALSE
9	3	TC03002106.hg.1	ENSG00000090530;	0.0042646	0.0523086	FALSE
9	3	TC06004066.hg.1	NR_001434;NR_001	0.0042646	0.0523086	FALSE
18	4	TC04001487.hg.1	ENSG00000180801;	0.0046779	0.0556381	FALSE
3759	176	TC01000685.hg.1	ENSG00000162409;	0.0047628	0.0558031	FALSE
3693	173	TC01000685.hg.1	ENSG00000162409;	0.0050803	0.0586478	FALSE
791	46	TC01001258.hg.1	ENSG00000160678;	0.0052427	0.0596447	FALSE
217	17	TC01001188.hg.1	ENSG00000143369;	0.0055164	0.0618627	FALSE
19	4	TC02002714.hg.1	ENSG00000163251;	0.0057434	0.0635009	FALSE
10	3	TC01003778.hg.1	ENSG00000076356;	0.0059155	0.0644954	FALSE
56	7	TC01001095.hg.1	ENSG00000143127;	0.0061495	0.0661282	FALSE
20	4	TC02000466.hg.1	ENSG00000159399;	0.0069592	0.0738236	FALSE
5776	257	TC01000394.hg.1	ENSG00000159023;	0.0073625	0.0770612	FALSE
516	32	TC01001805.hg.1	ENSG00000092969;	0.0076324	0.0788347	FALSE
11	3	TC09000015.hg.1	ENSG00000147852;	0.0078982	0.0805206	FALSE
121	11	TC01001907.hg.1	ENSG00000143641;	0.0081961	0.0819863	FALSE
21	4	TC06000621.hg.1	ENSG00000124813;	0.0083335	0.0819863	FALSE
105	10	TC03000214.hg.1	ENSG00000168036;	0.0083553	0.0819863	FALSE
61	7	TC03000214.hg.1	ENSG00000168036;	0.0097801	0.0947823	FALSE
34	5	TC02001750.hg.1	ENSG00000138061;	0.0100403	0.0950037	FALSE
159	13	TC02000230.hg.1	ENSG00000150938;	0.0101538	0.0950037	FALSE
12	3	TC02000620.hg.1	ENSG00000115594;l	0.0102265	0.0950037	FALSE
12	3	TC02001628.hg.1	ENSG00000084674;	0.0102265	0.0950037	FALSE
867	48	TC01001805.hg.1	ENSG00000092969;	0.0106527	0.0972371	FALSE
23	4	TC02000230.hg.1	ENSG00000150938;	0.0115881	0.1045592	FALSE
162	13	TC02000230.hg.1	ENSG00000150938;	0.0117582	0.1048884	FALSE
312	21	TC01003042.hg.1	ENSG00000092607;	0.0120373	0.105138	FALSE
14259	580	TC01000100.hg.1	ENSG00000049246;	0.012054	0.105138	FALSE
64	7	TC01001188.hg.1	ENSG00000143369;	0.0125953	0.1086521	FALSE
24	4	TC01003042.hg.1	ENSG00000092607;	0.013482	0.114392	FALSE
165	13	TC02000230.hg.1	ENSG00000150938;	0.0135522	0.114392	FALSE
5	2	TC10000986.hg.1	ENSG00000151632;	0.0142839	0.1150034	FALSE
5	2	TC02002808.hg.1	ENSG00000116106;l	0.0142839	0.1150034	FALSE
5	2	TC05001289.hg.1	ENSG00000113594;l	0.0142839	0.1150034	FALSE
5	2	TC02000466.hg.1	ENSG00000159399;	0.0142839	0.1150034	FALSE
5	2	TC01000972.hg.1	ENSG00000064886;	0.0142839	0.1150034	FALSE
5	2	TC17000220.hg.1	ENSG00000108448;	0.0142839	0.1150034	FALSE
5	2	TC01003497.hg.1	ENSG00000117479;l	0.0142839	0.1150034	FALSE
5	2	TC01003497.hg.1	ENSG00000117479;l	0.0142839	0.1150034	FALSE
25	4	TC02000937.hg.1	ENSG00000123610;	0.0155617	0.1187553	FALSE
67	7	TC03000214.hg.1	ENSG00000168036;	0.0159512	0.1187553	FALSE
14	3	TC01003042.hg.1	ENSG00000092607;	0.0159592	0.1187553	FALSE
14	3	TC09000191.hg.1	ENSG00000107159;	0.0159592	0.1187553	FALSE
321	21	TC01003042.hg.1	ENSG00000092607;	0.0161114	0.1187553	FALSE
321	21	TC01003042.hg.1	ENSG00000092607;	0.0161114	0.1187553	FALSE
303	20	TC01000394.hg.1	ENSG00000159023;	0.0170092	0.1236319	FALSE
39	5	TC06001772.hg.1	ENSG00000146233;	0.0177004	0.1274756	FALSE
15	3	TC04000274.hg.1	ENSG00000145246;	0.019376	0.1371023	FALSE
15	3	TC01001805.hg.1	ENSG00000092969;	0.019376	0.1371023	FALSE
70	7	TC01000643.hg.1	ENSG00000116157;l	0.0198964	0.1371023	FALSE
989	52	TC01000685.hg.1	ENSG00000162409;	0.0200008	0.1371023	FALSE
27	4	TC03000214.hg.1	ENSG00000168036;	0.0202988	0.1371023	FALSE

27	4	TC01001607.hg.1	ENSG00000116711;I	0.0202988	0.1371023	FALSE
55	6	TC03001071.hg.1	ENSG00000114315;I	0.0205079	0.1371023	FALSE
6	2	TC05001617.hg.1	ENSG00000164307;	0.020871	0.1371023	FALSE
6	2	TC07001559.hg.1	ENSG00000075223;	0.020871	0.1371023	FALSE
6	2	TC10001398.hg.1	ENSG00000122884;	0.020871	0.1371023	FALSE
6	2	TC01002954.hg.1	ENSG00000134243;	0.020871	0.1371023	FALSE
6	2	TC04000408.hg.1	ENSG00000169429;	0.020871	0.1371023	FALSE
6	2	TC06002036.hg.1	ENSG00000249853;	0.020871	0.1371023	FALSE
41	5	TC07001883.hg.1	ENSG00000085662;	0.0216148	0.1379482	FALSE
88	8	TC02000230.hg.1	ENSG00000150938;	0.0224193	0.1419284	FALSE
177	13	TC01003745.hg.1	ENSG00000163545;	0.0228966	0.1437444	FALSE
16	3	TC06001772.hg.1	ENSG00000146233;	0.0231639	0.1437444	FALSE
16	3	TC02001628.hg.1	ENSG00000084674;	0.0231639	0.1437444	FALSE
106	9	TC02001750.hg.1	ENSG00000138061;	0.0238561	0.1463053	FALSE
276	18	TC01001090.hg.1	ENSG00000117289;I	0.0252912	0.1539041	FALSE
17	3	TC01002669.hg.1	ENSG00000157193;	0.0273232	0.1619689	FALSE
59	6	TC02000466.hg.1	ENSG00000159399;	0.0279771	0.1619689	FALSE
7	2	TC01000728.hg.1	ENSG00000162433;	0.0284653	0.1619689	FALSE
7	2	TC06001015.hg.1	ENSG00000171408;	0.0284653	0.1619689	FALSE
7	2	TC01000972.hg.1	ENSG00000064886;	0.0284653	0.1619689	FALSE
7	2	TC10000022.hg.1	ENSG00000067057;	0.0284653	0.1619689	FALSE
7	2	TC03002106.hg.1	ENSG00000090530;	0.0284653	0.1619689	FALSE
7	2	TC09000560.hg.1	ENSG00000148154;	0.0284653	0.1619689	FALSE
44	5	TC03002106.hg.1	ENSG00000090530;	0.0284735	0.1619689	FALSE
30	4	TC09000222.hg.1	ENSG00000137124;	0.0289083	0.1632589	FALSE
2670	123	TC01000394.hg.1	ENSG00000159023;	0.0307618	0.1724858	FALSE
45	5	TC04000408.hg.1	ENSG00000169429;	0.0310326	0.17277	FALSE
18	3	TC02000286.hg.1	ENSG00000171150;I	0.0318523	0.1754671	FALSE
18	3	TC01003497.hg.1	ENSG00000117479;I	0.0318523	0.1754671	FALSE
31	4	TC06000968.hg.1	ENSG00000146374;	0.0321906	0.1754836	FALSE
1477	72	TC01000723.hg.1	ENSG00000185483;	0.0330889	0.1791362	FALSE
168	12	TC01003497.hg.1	ENSG00000117479;I	0.0338294	0.1818911	FALSE
131	10	TC01003638.hg.1	ENSG00000073756;	0.0340891	0.1820405	FALSE
266	17	TC01000783.hg.1	ENSG00000117069;I	0.0343756	0.1823302	FALSE
328	20	TC01003042.hg.1	ENSG00000092607;	0.0355465	0.1868235	FALSE
79	7	TC01003042.hg.1	ENSG00000092607;	0.0356988	0.1868235	FALSE
8	2	TC04000313.hg.1	ENSG00000157404;	0.0369777	0.1916006	FALSE
8	2	TC06000608.hg.1	ENSG00000112715;I	0.0369777	0.1916006	FALSE
153	11	TC01003042.hg.1	ENSG00000092607;	0.0394259	0.2022834	FALSE
192	13	TC02000230.hg.1	ENSG00000150938;	0.040444	0.2061593	FALSE
99	8	TC04000699.hg.1	ENSG00000109458;	0.0412761	0.2090433	FALSE
20	3	TC03002106.hg.1	ENSG00000090530;	0.0420045	0.2113689	FALSE
49	5	TC09002910.hg.1	ENSG00000107317;	0.0426805	0.2134023	FALSE
50	5	TC02002292.hg.1	ENSG00000136720;	0.0459514	0.2265385	FALSE
9	2	TC06001246.hg.1	ENSG00000124491;	0.0463243	0.2265385	FALSE
9	2	TC02002808.hg.1	ENSG00000116106;I	0.0463243	0.2265385	FALSE
9	2	TC11000707.hg.1	ENSG00000162337;	0.0463243	0.2265385	FALSE
9	2	TC09000560.hg.1	ENSG00000148154;	0.0463243	0.2265385	FALSE
177	12	TC01001907.hg.1	ENSG00000143641;	0.0472984	0.2265385	FALSE
35	4	TC01001907.hg.1	ENSG00000143641;	0.0474197	0.2265385	FALSE
21	3	TC01000783.hg.1	ENSG00000117069;I	0.0476164	0.2265385	FALSE
140	10	TC01003638.hg.1	ENSG00000073756;	0.0498647	0.2358061	FALSE
557	30	TC01000394.hg.1	ENSG00000159023;	0.0512914	0.2411002	FALSE
52	5	TC04000408.hg.1	ENSG00000169429;	0.0529293	0.2473187	FALSE
37	4	TC01000789.hg.1	ENSG00000162614;	0.0562939	0.2582786	FALSE
10	2	TC02001963.hg.1	ENSG00000163235;	0.0564265	0.2582786	FALSE
10	2	TC10000986.hg.1	ENSG00000151632;	0.0564265	0.2582786	FALSE
10	2	TC01003129.hg.1	ENSG00000143140;	0.0564265	0.2582786	FALSE

10	2	TC0Y000052.hg.1	ENSG00000114374;I	0.0564265	0.2582786	FALSE
125	9	TC01003042.hg.1	ENSG00000092607;	0.0583826	0.2633928	FALSE
164	11	TC02001750.hg.1	ENSG00000138061;	0.0594795	0.2662943	FALSE
23	3	TC06001015.hg.1	ENSG00000171408;	0.0598738	0.2662943	FALSE
23	3	TC0Y000351.hg.1	ENSG00000129824;	0.0598738	0.2662943	FALSE
90	7	TC01001805.hg.1	ENSG00000092969;	0.0640957	0.2826694	FALSE
24	3	TC01001805.hg.1	ENSG00000092969;	0.0665012	0.2875219	FALSE
24	3	TC06001015.hg.1	ENSG00000171408;	0.0665012	0.2875219	FALSE
24	3	TC01001607.hg.1	ENSG00000116711;I	0.0665012	0.2875219	FALSE
24	3	TC01003887.hg.1	ENSG00000143815;	0.0665012	0.2875219	FALSE
11	2	TC10000986.hg.1	ENSG00000151632;	0.0672105	0.2875219	FALSE
11	2	TC03002008.hg.1	ENSG00000144959;	0.0672105	0.2875219	FALSE
74	6	TC01000904.hg.1	ENSG00000162692;	0.0708724	0.2997439	FALSE
40	4	TC02002624.hg.1	ENSG00000115415;I	0.0711515	0.2997439	FALSE
111	8	TC04000699.hg.1	ENSG00000109458;	0.071404	0.2997439	FALSE
25	3	TC02001031.hg.1	ENSG00000152256;	0.0734475	0.3058689	FALSE
25	3	TC02002808.hg.1	ENSG00000116106;I	0.0734475	0.3058689	FALSE
75	6	TC04000101.hg.1	ENSG00000249811;I	0.074609	0.3074438	FALSE
75	6	TC02001437.hg.1	ENSG00000144476;	0.074609	0.3074438	FALSE
41	4	TC02002624.hg.1	ENSG00000115415;I	0.0765068	0.3103766	FALSE
41	4	TC01000643.hg.1	ENSG00000116157;I	0.0765068	0.3103766	FALSE
41	4	TC01000643.hg.1	ENSG00000116157;I	0.0765068	0.3103766	FALSE
41	4	TC03000214.hg.1	ENSG00000168036;	0.0765068	0.3103766	FALSE
12	2	TC04001487.hg.1	ENSG00000180801;	0.0786073	0.3116503	FALSE
12	2	TC03001169.hg.1	ENSG00000157150;	0.0786073	0.3116503	FALSE
12	2	TC03001169.hg.1	ENSG00000157150;	0.0786073	0.3116503	FALSE
12	2	TC04000432.hg.1	ENSG00000156234;	0.0786073	0.3116503	FALSE
12	2	TC03001169.hg.1	ENSG00000157150;	0.0786073	0.3116503	FALSE
449	24	TC01000734.hg.1	ENSG00000118473;I	0.0801141	0.3128834	FALSE
26	3	TC08000845.hg.1	ENSG00000187786;	0.0807016	0.3136178	FALSE
59	5	TC01003042.hg.1	ENSG00000092607;	0.0819183	0.314992	FALSE
42	4	TC01001805.hg.1	ENSG00000092969;	0.0820584	0.314992	FALSE
42	4	TC04000699.hg.1	ENSG00000109458;	0.0820584	0.314992	FALSE
78	6	TC02000281.hg.1	ENSG00000116016;I	0.0864771	0.3287387	FALSE
78	6	TC02001437.hg.1	ENSG00000144476;	0.0864771	0.3287387	FALSE
43	4	TC09000191.hg.1	ENSG00000107159;	0.0878026	0.3288826	FALSE
27	3	TC02002624.hg.1	ENSG00000115415;I	0.088252	0.3288826	FALSE
27	3	TC01002578.hg.1	ENSG00000117394;I	0.088252	0.3288826	FALSE
136	9	TC01000394.hg.1	ENSG00000159023;	0.0884003	0.3288826	FALSE
13	2	TC01001607.hg.1	ENSG00000116711;I	0.0905521	0.333725	FALSE
13	2	TC01003129.hg.1	ENSG00000143140;	0.0905521	0.333725	FALSE
13	2	TC12002155.hg.1	ENSG00000176915;	0.0905521	0.333725	FALSE
28	3	TC04000274.hg.1	ENSG00000145246;	0.0960865	0.3505876	FALSE
62	5	TC02002292.hg.1	ENSG00000136720;	0.0964674	0.3505876	FALSE
81	6	TC02000620.hg.1	ENSG00000115594;I	0.0993193	0.3592885	FALSE
45	4	TC01000904.hg.1	ENSG00000162692;	0.0998529	0.3595621	FALSE
287	16	TC01000685.hg.1	ENSG00000162409;	0.1027814	0.3658039	FALSE
14	2	TC10000986.hg.1	ENSG00000151632;	0.1029843	0.3658039	FALSE
14	2	TC04001001.hg.1	ENSG00000074211;I	0.1029843	0.3658039	FALSE
14	2	TC04000432.hg.1	ENSG00000156234;	0.1029843	0.3658039	FALSE
1559	71	TC01000100.hg.1	ENSG00000049246;	0.1066324	0.3745255	FALSE
1559	71	TC01000100.hg.1	ENSG00000049246;	0.1066324	0.3745255	FALSE
103	7	TC02000762.hg.1	ENSG00000163083;	0.1114107	0.3886994	FALSE
30	3	TC03001071.hg.1	ENSG00000114315;I	0.1125575	0.3894349	FALSE
30	3	TC02001100.hg.1	ENSG00000138448;	0.1125575	0.3894349	FALSE
84	6	TC01001607.hg.1	ENSG00000116711;I	0.1131098	0.3894349	FALSE
206	12	TC01001603.hg.1	ENSG00000116690;I	0.113991	0.3907552	FALSE
15	2	TC01001603.hg.1	ENSG00000116690;I	0.1158471	0.3928293	FALSE

15	2	TC14001141.hg.1	ENSG00000197930;	0.1158471	0.3928293	FALSE
15	2	TC14001141.hg.1	ENSG00000197930;	0.1158471	0.3928293	FALSE
15	2	TC01001603.hg.1	ENSG00000116690;I	0.1158471	0.3928293	FALSE
66	5	TC04000274.hg.1	ENSG00000145246;	0.1177444	0.3949972	FALSE
48	4	TC09002910.hg.1	ENSG00000107317;	0.1192653	0.3983967	FALSE
67	5	TC02002714.hg.1	ENSG00000163251;	0.1233839	0.4104085	FALSE
16	2	TC02000219.hg.1	ENSG00000049323;	0.1290876	0.4217318	FALSE
16	2	TC05000243.hg.1	ENSG00000095015;	0.1290876	0.4217318	FALSE
16	2	TC02000219.hg.1	ENSG00000049323;	0.1290876	0.4217318	FALSE
16	2	TC14001141.hg.1	ENSG00000197930;	0.1290876	0.4217318	FALSE
16	2	TC05001933.hg.1	ENSG00000113721;I	0.1290876	0.4217318	FALSE
32	3	TC03001071.hg.1	ENSG00000114315;I	0.1300116	0.4217318	FALSE
33	3	TC04000432.hg.1	ENSG00000156234;	0.1390743	0.4483505	FALSE
33	3	TC01001188.hg.1	ENSG00000143369;	0.1390743	0.4483505	FALSE
17	2	TC08000149.hg.1	ENSG00000175445;	0.1426561	0.4549289	FALSE
17	2	TC02002808.hg.1	ENSG00000116106;I	0.1426561	0.4549289	FALSE
17	2	TC04000432.hg.1	ENSG00000156234;	0.1426561	0.4549289	FALSE
415	21	TC01003042.hg.1	ENSG00000092607;	0.1437228	0.4549289	FALSE
52	4	TC09000015.hg.1	ENSG00000147852;	0.1474302	0.46479	FALSE
350	18	TC01002166.hg.1	ENSG00000116285;I	0.1501968	0.4716178	FALSE
72	5	TC01003497.hg.1	ENSG00000117479;I	0.1533672	0.4796545	FALSE
18	2	TC07001562.hg.1	ENSG00000019991;	0.1565066	0.4832773	FALSE
18	2	TC01002578.hg.1	ENSG00000117394;I	0.1565066	0.4832773	FALSE
286	15	TC01000904.hg.1	ENSG00000162692;	0.1588692	0.4832773	FALSE
401	20	TC01003042.hg.1	ENSG00000092607;	0.1656003	0.4832773	FALSE
36	3	TC03000214.hg.1	ENSG00000168036;	0.1674458	0.4832773	FALSE
115	7	TC01003497.hg.1	ENSG00000117479;I	0.1676473	0.4832773	FALSE
55	4	TC04000699.hg.1	ENSG00000109458;	0.1700687	0.4832773	FALSE
55	4	TC04000253.hg.1	ENSG00000154277;	0.1700687	0.4832773	FALSE
19	2	TC01000728.hg.1	ENSG00000162433;	0.1705959	0.4832773	FALSE
19	2	TC04001084.hg.1	ENSG00000109819;	0.1705959	0.4832773	FALSE
137	8	TC01001805.hg.1	ENSG00000092969;	0.1725285	0.4832773	FALSE
382	19	TC01003042.hg.1	ENSG00000092607;	0.1759922	0.4832773	FALSE
56	4	TC09000191.hg.1	ENSG00000107159;	0.1778716	0.4832773	FALSE
5	1	TC18000097.hg.1	ENSG00000101773	0.1818873	0.4832773	FALSE
5	1	TC16001281.hg.1	ENSG00000183196	0.1818873	0.4832773	FALSE
5	1	TC02001100.hg.1	ENSG00000138448	0.1818873	0.4832773	FALSE
5	1	TC17001250.hg.1	ENSG00000108602	0.1818873	0.4832773	FALSE
5	1	TC02000466.hg.1	ENSG00000159399	0.1818873	0.4832773	FALSE
5	1	TC10001663.hg.1	ENSG00000119927	0.1818873	0.4832773	FALSE
5	1	TC02000466.hg.1	ENSG00000159399	0.1818873	0.4832773	FALSE
5	1	TC22000259.hg.1	ENSG00000100292	0.1818873	0.4832773	FALSE
5	1	TC12000099.hg.1	ENSG00000111674	0.1818873	0.4832773	FALSE
5	1	TC01000685.hg.1	ENSG00000162409	0.1818873	0.4832773	FALSE
5	1	TC10000811.hg.1	ENSG00000150594	0.1818873	0.4832773	FALSE
5	1	TC10001711.hg.1	ENSG00000066468	0.1818873	0.4832773	FALSE
5	1	TC0X000207.hg.1	ENSG00000189221	0.1818873	0.4832773	FALSE
5	1	TC06002036.hg.1	ENSG00000249853	0.1818873	0.4832773	FALSE
5	1	TC01003497.hg.1	ENSG00000117479	0.1818873	0.4832773	FALSE
5	1	TC02000466.hg.1	ENSG00000159399	0.1818873	0.4832773	FALSE
5	1	TC12002122.hg.1	ENSG00000139370	0.1818873	0.4832773	FALSE
5	1	TC05001110.hg.1	ENSG00000153395	0.1818873	0.4832773	FALSE
5	1	TC02000466.hg.1	ENSG00000159399	0.1818873	0.4832773	FALSE
5	1	TC04000253.hg.1	ENSG00000154277	0.1818873	0.4832773	FALSE
5	1	TC03001362.hg.1	ENSG00000160808	0.1818873	0.4832773	FALSE
5	1	TC0Y000185.hg.1	ENSG00000012817	0.1818873	0.4832773	FALSE
5	1	TC03003359.hg.1	ENSG00000172986	0.1818873	0.4832773	FALSE
5	1	TC21000169.hg.1	ENSG00000157557	0.1818873	0.4832773	FALSE

5	1	TC03003359.hg.1	ENSG00000172986	0.1818873	0.4832773	FALSE
5	1	TC05001657.hg.1	ENSG00000184349	0.1818873	0.4832773	FALSE
5	1	TC01002954.hg.1	ENSG00000134243	0.1818873	0.4832773	FALSE
5	1	TC0X000943.hg.1	ENSG00000198947	0.1818873	0.4832773	FALSE
5	1	TC02000082.hg.1	ENSG00000071575	0.1818873	0.4832773	FALSE
5	1	TC12000621.hg.1	ENSG00000135643	0.1818873	0.4832773	FALSE
5	1	TC01000139.hg.1	ENSG00000171819	0.1818873	0.4832773	FALSE
5	1	TC06001332.hg.1	ENSG00000168405	0.1818873	0.4832773	FALSE
97	6	TC02002480.hg.1	ENSG00000078098;	0.182845	0.4832773	FALSE
20	2	TC01002284.hg.1	ENSG00000117122;I	0.1848838	0.4853974	FALSE
20	2	TC02001437.hg.1	ENSG00000144476;	0.1848838	0.4853974	FALSE
20	2	TC08001659.hg.1	ENSG00000104419;	0.1848838	0.4853974	FALSE
98	6	TC02002624.hg.1	ENSG00000115415;I	0.1888052	0.492399	FALSE
141	8	TC01001805.hg.1	ENSG00000092969;	0.1920339	0.499161	FALSE
78	5	TC03003381.hg.1	ENSG00000225697;	0.1928574	0.4996471	FALSE
39	3	TC06001027.hg.1	ENSG00000118503;I	0.1973158	0.5028079	FALSE
208	11	TC02000762.hg.1	ENSG00000163083;	0.1978293	0.5028079	FALSE
21	2	TC09000015.hg.1	ENSG00000147852;	0.199333	0.5028079	FALSE
21	2	TC09000015.hg.1	ENSG00000147852;	0.199333	0.5028079	FALSE
21	2	TC01003497.hg.1	ENSG00000117479;I	0.199333	0.5028079	FALSE
80	5	TC02001628.hg.1	ENSG00000084674;	0.206752	0.5028079	FALSE
40	3	TC13001719.hg.1	ENSG00000150907;	0.2075454	0.5028079	FALSE
102	6	TC01001607.hg.1	ENSG00000116711;I	0.2133629	0.5028079	FALSE
102	6	TC01000734.hg.1	ENSG00000118473;I	0.2133629	0.5028079	FALSE
81	5	TC03003381.hg.1	ENSG00000225697;	0.21382	0.5028079	FALSE
22	2	TC12002122.hg.1	ENSG00000139370;	0.2139088	0.5028079	FALSE
22	2	TC06001246.hg.1	ENSG00000124491;	0.2139088	0.5028079	FALSE
6	1	TC17001462.hg.1	ENSG00000131747	0.2140906	0.5028079	FALSE
6	1	TC19000377.hg.1	ENSG00000160161	0.2140906	0.5028079	FALSE
6	1	TC16001225.hg.1	ENSG00000181019	0.2140906	0.5028079	FALSE
6	1	TC03001884.hg.1	ENSG00000047457	0.2140906	0.5028079	FALSE
6	1	TC10000053.hg.1	ENSG00000170525	0.2140906	0.5028079	FALSE
6	1	TC14001124.hg.1	ENSG00000100504	0.2140906	0.5028079	FALSE
6	1	TC15000622.hg.1	ENSG00000166949	0.2140906	0.5028079	FALSE
6	1	TC01001805.hg.1	ENSG00000092969	0.2140906	0.5028079	FALSE
6	1	TC22000702.hg.1	ENSG00000128335	0.2140906	0.5028079	FALSE
6	1	TC21000506.hg.1	ENSG00000142178	0.2140906	0.5028079	FALSE
6	1	TC05000054.hg.1	ENSG00000078295	0.2140906	0.5028079	FALSE
6	1	TC17000124.hg.1	ENSG00000179593	0.2140906	0.5028079	FALSE
6	1	TC11002178.hg.1	ENSG00000086991	0.2140906	0.5028079	FALSE
6	1	TC05000165.hg.1	ENSG00000079215	0.2140906	0.5028079	FALSE
6	1	TC02000416.hg.1	ENSG00000116005	0.2140906	0.5028079	FALSE
6	1	TC03001884.hg.1	ENSG00000047457	0.2140906	0.5028079	FALSE
6	1	TC15001136.hg.1	ENSG00000166664	0.2140906	0.5028079	FALSE
6	1	TC15002776.hg.1	ENSG00000067225	0.2140906	0.5028079	FALSE
6	1	TC14002310.hg.1	ENSG00000168398	0.2140906	0.5028079	FALSE
6	1	TC02001100.hg.1	ENSG00000138448	0.2140906	0.5028079	FALSE
6	1	TC01002616.hg.1	ENSG00000117461	0.2140906	0.5028079	FALSE
6	1	TC12001837.hg.1	ENSG00000074527	0.2140906	0.5028079	FALSE
6	1	TC12001324.hg.1	ENSG00000123095	0.2140906	0.5028079	FALSE
6	1	TC02000996.hg.1	ENSG00000172292	0.2140906	0.5028079	FALSE
6	1	TC21000138.hg.1	ENSG00000159212	0.2140906	0.5028079	FALSE
6	1	TC01003887.hg.1	ENSG00000143815	0.2140906	0.5028079	FALSE
6	1	TC14000353.hg.1	ENSG00000165617	0.2140906	0.5028079	FALSE
6	1	TC08002613.hg.1	ENSG00000134013	0.2140906	0.5028079	FALSE
6	1	TC0X000038.hg.1	ENSG00000073464	0.2140906	0.5028079	FALSE
6	1	TC15001761.hg.1	ENSG00000103942	0.2140906	0.5028079	FALSE
6	1	TC17001590.hg.1	ENSG00000131094	0.2140906	0.5028079	FALSE

6	1	TC02001139.hg.1	ENSG00000196950	0.2140906	0.5028079	FALSE
147	8	TC01003211.hg.1	ENSG00000143387;	0.2229008	0.5028079	FALSE
83	5	TC03003381.hg.1	ENSG00000225697;	0.2281783	0.5126696	FALSE
23	2	TC02001437.hg.1	ENSG00000144476;	0.2285788	0.5126696	FALSE
1483	64	TC01000685.hg.1	ENSG00000162409;	0.2330876	0.5212928	FALSE
43	3	TC01003497.hg.1	ENSG00000117479;I	0.2388794	0.5268378	FALSE
106	6	TC04000101.hg.1	ENSG00000249811;I	0.2389483	0.5268378	FALSE
1487	64	TC01000685.hg.1	ENSG00000162409;	0.2399816	0.5268378	FALSE
24	2	TC02001437.hg.1	ENSG00000144476;	0.2433131	0.5268378	FALSE
24	2	TC02001437.hg.1	ENSG00000144476;	0.2433131	0.5268378	FALSE
24	2	TC04000432.hg.1	ENSG00000156234;	0.2433131	0.5268378	FALSE
7	1	TC01003791.hg.1	ENSG00000143473	0.2450281	0.5268378	FALSE
7	1	TC02000620.hg.1	ENSG00000115594	0.2450281	0.5268378	FALSE
7	1	TC05001151.hg.1	ENSG00000112902	0.2450281	0.5268378	FALSE
7	1	TC14001124.hg.1	ENSG00000100504	0.2450281	0.5268378	FALSE
7	1	TC02001437.hg.1	ENSG00000144476	0.2450281	0.5268378	FALSE
7	1	TC14001141.hg.1	ENSG00000197930	0.2450281	0.5268378	FALSE
7	1	TC17001353.hg.1	ENSG00000176658	0.2450281	0.5268378	FALSE
7	1	TC14002310.hg.1	ENSG00000168398	0.2450281	0.5268378	FALSE
7	1	TC10000811.hg.1	ENSG00000150594	0.2450281	0.5268378	FALSE
7	1	TC03000214.hg.1	ENSG00000168036	0.2450281	0.5268378	FALSE
7	1	TC01001607.hg.1	ENSG00000116711	0.2450281	0.5268378	FALSE
7	1	TC17000819.hg.1	ENSG00000125398	0.2450281	0.5268378	FALSE
429	20	TC01000685.hg.1	ENSG00000162409;	0.2483185	0.5268378	FALSE
44	3	TC06001027.hg.1	ENSG00000118503;I	0.2494996	0.527917	FALSE
314	15	TC01002166.hg.1	ENSG00000116285;I	0.2563396	0.5340341	FALSE
176	9	TC01003497.hg.1	ENSG00000117479;I	0.2576671	0.5340341	FALSE
25	2	TC13000147.hg.1	ENSG00000102760;	0.258084	0.5340341	FALSE
25	2	TC01001188.hg.1	ENSG00000143369;	0.258084	0.5340341	FALSE
45	3	TC05001110.hg.1;	ENSG00000153395;	0.2601887	0.5340341	FALSE
66	4	TC04000253.hg.1	ENSG00000154277;	0.2613724	0.5340341	FALSE
88	5	TC06001015.hg.1	ENSG00000171408;	0.2651945	0.5340341	FALSE
387	18	TC02000082.hg.1	ENSG00000071575;	0.2657491	0.5340341	FALSE
155	8	TC02001750.hg.1	ENSG00000138061;	0.2666037	0.5340341	FALSE
133	7	TC07001883.hg.1	ENSG00000085662;	0.2698227	0.5340341	FALSE
67	4	TC01000789.hg.1	ENSG00000162614;	0.2701336	0.5340341	FALSE
46	3	TC10000986.hg.1	ENSG00000151632;	0.2709356	0.5340341	FALSE
26	2	TC05000619.hg.1	ENSG00000198108;	0.2728659	0.5340341	FALSE
26	2	TC03001362.hg.1	ENSG00000160808;	0.2728659	0.5340341	FALSE
1285	55	TC01000723.hg.1	ENSG00000185483;	0.2740541	0.5340341	FALSE
8	1	TC02002624.hg.1	ENSG00000115415	0.2747496	0.5340341	FALSE
8	1	TC11002175.hg.1	ENSG00000109861	0.2747496	0.5340341	FALSE
8	1	TC22001467.hg.1	ENSG00000128394	0.2747496	0.5340341	FALSE
8	1	TC01003791.hg.1	ENSG00000143473	0.2747496	0.5340341	FALSE
8	1	TC07001897.hg.1	ENSG00000157680	0.2747496	0.5340341	FALSE
8	1	TC06000779.hg.1	ENSG00000135318	0.2747496	0.5340341	FALSE
8	1	TC03003381.hg.1	ENSG00000225697	0.2747496	0.5340341	FALSE
8	1	TC11002178.hg.1	ENSG00000086991	0.2747496	0.5340341	FALSE
8	1	TC17001301.hg.1	ENSG00000109107	0.2747496	0.5340341	FALSE
8	1	TC01000785.hg.1	ENSG00000154027	0.2747496	0.5340341	FALSE
8	1	TC15000226.hg.1	ENSG00000166923	0.2747496	0.5340341	FALSE
8	1	TC10000350.hg.1	ENSG00000107984	0.2747496	0.5340341	FALSE
8	1	TC10000350.hg.1	ENSG00000107984	0.2747496	0.5340341	FALSE
8	1	TC01003266.hg.1	ENSG00000196154	0.2747496	0.5340341	FALSE
8	1	TC03000214.hg.1	ENSG00000168036	0.2747496	0.5340341	FALSE
8	1	TC11000198.hg.1	ENSG00000133816	0.2747496	0.5340341	FALSE
8	1	TC09001058.hg.1	ENSG00000198467	0.2747496	0.5340341	FALSE
8	1	TC03001888.hg.1	ENSG00000169908	0.2747496	0.5340341	FALSE

677	30	TC01002578.hg.1	ENSG00000117394;I	0.2755208	0.5340341	FALSE
47	3	TC12001429.hg.1	ENSG00000139209;	0.2817296	0.5447235	FALSE
158	8	TC02000245.hg.1	ENSG00000143891;	0.2836068	0.5470058	FALSE
1192	51	TC01000723.hg.1	ENSG00000185483;	0.2843646	0.547123	FALSE
27	2	TC02001963.hg.1	ENSG00000163235;	0.287635	0.5513882	FALSE
27	2	TC21000241.hg.1	ENSG00000197381;	0.287635	0.5513882	FALSE
160	8	TC02002624.hg.1	ENSG00000115415;I	0.2950958	0.5552063	FALSE
70	4	TC01003211.hg.1	ENSG00000143387;	0.296697	0.5552063	FALSE
70	4	TC03002146.hg.1	ENSG00000189058;	0.296697	0.5552063	FALSE
9	1	TC21000241.hg.1	ENSG00000197381	0.3033028	0.5552063	FALSE
9	1	TC19000377.hg.1	ENSG00000160161	0.3033028	0.5552063	FALSE
9	1	TC10000811.hg.1	ENSG00000150594	0.3033028	0.5552063	FALSE
9	1	TC09001515.hg.1	ENSG00000181634	0.3033028	0.5552063	FALSE
9	1	TC01003887.hg.1	ENSG00000143815	0.3033028	0.5552063	FALSE
9	1	TC10000874.hg.1	ENSG00000203805	0.3033028	0.5552063	FALSE
9	1	TC14001364.hg.1	ENSG00000211448	0.3033028	0.5552063	FALSE
9	1	TC15001136.hg.1	ENSG00000166664	0.3033028	0.5552063	FALSE
9	1	TC02000245.hg.1	ENSG00000143891	0.3033028	0.5552063	FALSE
9	1	TC01000394.hg.1	ENSG00000159023	0.3033028	0.5552063	FALSE
9	1	TC07001552.hg.1	ENSG00000187391	0.3033028	0.5552063	FALSE
9	1	TC07001627.hg.1	ENSG00000105880	0.3033028	0.5552063	FALSE
9	1	TC07000852.hg.1	ENSG00000181072	0.3033028	0.5552063	FALSE
9	1	TC21000443.hg.1	ENSG00000157542	0.3033028	0.5552063	FALSE
9	1	TC22000620.hg.1	ENSG00000169184	0.3033028	0.5552063	FALSE
49	3	TC02000281.hg.1	ENSG00000116016;I	0.3034185	0.5552063	FALSE
29	2	TC02002624.hg.1	ENSG00000115415;I	0.3170493	0.5754815	FALSE
29	2	TC05001617.hg.1	ENSG00000164307;	0.3170493	0.5754815	FALSE
29	2	TC09002910.hg.1	ENSG00000107317;	0.3170493	0.5754815	FALSE
29	2	TC09002910.hg.1	ENSG00000107317;	0.3170493	0.5754815	FALSE
29	2	TC10000986.hg.1	ENSG00000151632;	0.3170493	0.5754815	FALSE
141	7	TC01001090.hg.1	ENSG00000117289;I	0.3197865	0.5754815	FALSE
141	7	TC01001090.hg.1	ENSG00000117289;I	0.3197865	0.5754815	FALSE
1456	61	TC01000685.hg.1	ENSG00000162409;	0.3203636	0.5754815	FALSE
451	20	TC01003497.hg.1	ENSG00000117479;I	0.3229983	0.5769462	FALSE
51	3	TC0X000034.hg.1	ENSG00000146950;	0.325177	0.5769462	FALSE
51	3	TC02000416.hg.1	ENSG00000116005;I	0.325177	0.5769462	FALSE
51	3	TC03000214.hg.1	ENSG00000168036;	0.325177	0.5769462	FALSE
119	6	TC07001883.hg.1	ENSG00000085662;	0.3271924	0.5769462	FALSE
10	1	TC19000677.hg.1	ENSG00000134830	0.3307335	0.5769462	FALSE
10	1	TC02002480.hg.1	ENSG00000078098	0.3307335	0.5769462	FALSE
10	1	TC06000779.hg.1	ENSG00000135318	0.3307335	0.5769462	FALSE
10	1	TC14000951.hg.1	ENSG00000092068	0.3307335	0.5769462	FALSE
10	1	TC17000241.hg.1	ENSG00000142494	0.3307335	0.5769462	FALSE
10	1	TC05000165.hg.1	ENSG00000079215	0.3307335	0.5769462	FALSE
10	1	TC01002616.hg.1	ENSG00000117461	0.3307335	0.5769462	FALSE
10	1	TC08000203.hg.1	ENSG00000092964	0.3307335	0.5769462	FALSE
10	1	TC05000165.hg.1	ENSG00000079215	0.3307335	0.5769462	FALSE
10	1	TC10000053.hg.1	ENSG00000170525	0.3307335	0.5769462	FALSE
10	1	TC15002776.hg.1	ENSG00000067225	0.3307335	0.5769462	FALSE
10	1	TC07001552.hg.1	ENSG00000187391	0.3307335	0.5769462	FALSE
10	1	TC10000053.hg.1	ENSG00000170525	0.3307335	0.5769462	FALSE
10	1	TC07000768.hg.1	ENSG00000174697	0.3307335	0.5769462	FALSE
10	1	TC13000364.hg.1	ENSG00000198542	0.3307335	0.5769462	FALSE
407	18	TC01003129.hg.1	ENSG00000143140;	0.3390184	0.5810687	FALSE
31	2	TC13000147.hg.1	ENSG00000102760;	0.3461722	0.5818245	FALSE
31	2	TC02000286.hg.1	ENSG00000171150;I	0.3461722	0.5818245	FALSE
31	2	TC09000015.hg.1	ENSG00000147852;	0.3461722	0.5818245	FALSE
122	6	TC01000785.hg.1	ENSG00000154027;	0.3482442	0.5818245	FALSE

11	1	TC05000054.hg.1	ENSG00000078295	0.3570858	0.5818245	FALSE
11	1	TC14000067.hg.1	ENSG00000181784	0.3570858	0.5818245	FALSE
11	1	TC20000621.hg.1	ENSG00000101384	0.3570858	0.5818245	FALSE
11	1	TC12001890.hg.1	ENSG00000017427	0.3570858	0.5818245	FALSE
11	1	TC17001902.hg.1	ENSG00000161544	0.3570858	0.5818245	FALSE
11	1	TC18000164.hg.1	ENSG00000141469	0.3570858	0.5818245	FALSE
11	1	TC03003381.hg.1	ENSG00000225697	0.3570858	0.5818245	FALSE
11	1	TC03001884.hg.1	ENSG00000047457	0.3570858	0.5818245	FALSE
11	1	TC01003791.hg.1	ENSG00000143473	0.3570858	0.5818245	FALSE
11	1	TC07001332.hg.1	ENSG00000164708	0.3570858	0.5818245	FALSE
11	1	TC07001630.hg.1	ENSG00000070669	0.3570858	0.5818245	FALSE
11	1	TC03000986.hg.1	ENSG00000163872	0.3570858	0.5818245	FALSE
11	1	TC0X000943.hg.1	ENSG00000198947	0.3570858	0.5818245	FALSE
11	1	TC10001517.hg.1	ENSG00000148677	0.3570858	0.5818245	FALSE
11	1	TC01002616.hg.1	ENSG00000117461	0.3570858	0.5818245	FALSE
11	1	TC15001136.hg.1	ENSG00000166664	0.3570858	0.5818245	FALSE
11	1	TC18000435.hg.1	ENSG00000134762	0.3570858	0.5818245	FALSE
11	1	TC02001100.hg.1	ENSG00000138448	0.3570858	0.5818245	FALSE
11	1	TC04001169.hg.1	ENSG00000145244	0.3570858	0.5818245	FALSE
11	1	TC15000971.hg.1	ENSG00000184254	0.3570858	0.5818245	FALSE
11	1	TC06000087.hg.1	ENSG00000078401	0.3570858	0.5818245	FALSE
11	1	TC04000396.hg.1	ENSG00000080493	0.3570858	0.5818245	FALSE
77	4	TC02002624.hg.1	ENSG00000115415;I	0.3595939	0.5818245	FALSE
32	2	TC10000986.hg.1	ENSG00000151632;	0.3605829	0.5818245	FALSE
32	2	TC21000241.hg.1	ENSG00000197381;	0.3605829	0.5818245	FALSE
560	24	TC01003042.hg.1	ENSG00000092607;	0.3624767	0.5830824	FALSE
2304	94	TC01000972.hg.1	ENSG00000064886;	0.3672768	0.5895957	FALSE
55	3	TC02000281.hg.1	ENSG00000116016;I	0.3686179	0.589939	FALSE
55	3	TC01003887.hg.1	ENSG00000143815;	0.3686179	0.589939	FALSE
539	23	TC01000394.hg.1	ENSG00000159023;	0.3744703	0.5963038	FALSE
33	2	TC01000728.hg.1	ENSG00000162433;	0.3748738	0.5963038	FALSE
33	2	TC12000601.hg.1	ENSG00000127314;	0.3748738	0.5963038	FALSE
79	4	TC02002624.hg.1	ENSG00000115415;I	0.3776116	0.5988386	FALSE
12	1	TC03000131.hg.1	ENSG00000077092	0.382402	0.5997713	FALSE
12	1	TC09000965.hg.1	ENSG00000147883	0.382402	0.5997713	FALSE
12	1	TC01003497.hg.1	ENSG00000117479	0.382402	0.5997713	FALSE
12	1	TC16001225.hg.1	ENSG00000181019	0.382402	0.5997713	FALSE
12	1	TC07001552.hg.1	ENSG00000187391	0.382402	0.5997713	FALSE
12	1	TC06002036.hg.1	ENSG00000249853	0.382402	0.5997713	FALSE
12	1	TC01000685.hg.1	ENSG00000162409	0.382402	0.5997713	FALSE
12	1	TC19000012.hg.1	ENSG00000070404	0.382402	0.5997713	FALSE
12	1	TC21000506.hg.1	ENSG00000142178	0.382402	0.5997713	FALSE
12	1	TC10002938.hg.1	ENSG00000148671	0.382402	0.5997713	FALSE
34	2	TC01002166.hg.1	ENSG00000116285;I	0.389032	0.6035377	FALSE
718	30	TC01001607.hg.1	ENSG00000116711;I	0.3934404	0.6091729	FALSE
695	29	TC01000394.hg.1	ENSG00000159023;	0.3989891	0.6165481	FALSE
106	5	TC03003381.hg.1	ENSG00000225697;	0.4053033	0.6193548	FALSE
106	5	TC01000785.hg.1	ENSG00000154027;	0.4053033	0.6193548	FALSE
13	1	TC03001362.hg.1	ENSG00000160808	0.4067228	0.6193548	FALSE
13	1	TC04001820.hg.1	ENSG00000154556	0.4067228	0.6193548	FALSE
13	1	TC02000891.hg.1	ENSG00000150540	0.4067228	0.6193548	FALSE
13	1	TC12000096.hg.1	ENSG00000111669	0.4067228	0.6193548	FALSE
13	1	TC03000208.hg.1	ENSG00000168032	0.4067228	0.6193548	FALSE
13	1	TC02001628.hg.1	ENSG00000084674	0.4067228	0.6193548	FALSE
13	1	TC11002162.hg.1	ENSG00000137501	0.4067228	0.6193548	FALSE
13	1	TC11002178.hg.1	ENSG00000086991	0.4067228	0.6193548	FALSE
13	1	TC01003497.hg.1	ENSG00000117479	0.4067228	0.6193548	FALSE
13	1	TC01002616.hg.1	ENSG00000117461	0.4067228	0.6193548	FALSE

84	4	TC01000394.hg.1	ENSG00000159023;	0.4223643	0.6363093	FALSE
14	1	TC05001617.hg.1	ENSG00000164307	0.4300873	0.6363093	FALSE
14	1	TC01000394.hg.1	ENSG00000159023	0.4300873	0.6363093	FALSE
14	1	TC02001628.hg.1	ENSG00000084674	0.4300873	0.6363093	FALSE
14	1	TC15001136.hg.1	ENSG00000166664	0.4300873	0.6363093	FALSE
14	1	TC02000245.hg.1	ENSG00000143891	0.4300873	0.6363093	FALSE
14	1	TC14000067.hg.1	ENSG00000181784	0.4300873	0.6363093	FALSE
14	1	TC22001477.hg.1	ENSG00000100344	0.4300873	0.6363093	FALSE
14	1	TC10000811.hg.1	ENSG00000150594	0.4300873	0.6363093	FALSE
14	1	TC05001172.hg.1	ENSG00000154122	0.4300873	0.6363093	FALSE
37	2	TC02000891.hg.1	ENSG00000150540;	0.4305989	0.6363093	FALSE
85	4	TC05000165.hg.1	ENSG00000079215;	0.4312313	0.6363093	FALSE
134	6	TC01001188.hg.1	ENSG00000143369;	0.432941	0.6376336	FALSE
86	4	TC10000986.hg.1	ENSG00000151632;	0.4400614	0.6469067	FALSE
236	10	TC02000281.hg.1	ENSG00000116016;I	0.4513077	0.6615241	FALSE
15	1	TC07001897.hg.1	ENSG00000157680	0.452533	0.6615241	FALSE
15	1	TC0Y000185.hg.1	ENSG00000012817	0.452533	0.6615241	FALSE
15	1	TC19001293.hg.1	ENSG00000105664	0.452533	0.6615241	FALSE
39	2	TC02000082.hg.1	ENSG00000071575;	0.4574611	0.6656292	FALSE
39	2	TC05001617.hg.1	ENSG00000164307;	0.4574611	0.6656292	FALSE
891	36	TC01000785.hg.1	ENSG00000154027;	0.4594551	0.6666771	FALSE
89	4	TC01003497.hg.1	ENSG00000117479;I	0.4662947	0.675353	FALSE
16	1	TC11002162.hg.1	ENSG00000137501	0.4740961	0.6816216	FALSE
16	1	TC10000663.hg.1	ENSG00000095596	0.4740961	0.6816216	FALSE
16	1	TC0X001279.hg.1	ENSG00000077274	0.4740961	0.6816216	FALSE
16	1	TC05000943.hg.1	ENSG00000156427	0.4740961	0.6816216	FALSE
16	1	TC04001084.hg.1	ENSG00000109819	0.4740961	0.6816216	FALSE
16	1	TC12000227.hg.1	ENSG00000172572	0.4740961	0.6816216	FALSE
16	1	TC20000980.hg.1	ENSG00000124225	0.4740961	0.6816216	FALSE
41	2	TC08001659.hg.1	ENSG00000104419;	0.4835747	0.6853547	FALSE
41	2	TC01000728.hg.1	ENSG00000162433;	0.4835747	0.6853547	FALSE
1839	73	TC01000685.hg.1	ENSG00000162409;	0.487155	0.6853547	FALSE
218	9	TC03000214.hg.1	ENSG00000168036;	0.4897523	0.6853547	FALSE
1815	72	TC01000685.hg.1	ENSG00000162409;	0.4898931	0.6853547	FALSE
117	5	TC01001607.hg.1	ENSG00000116711;I	0.4901835	0.6853547	FALSE
92	4	TC03000013.hg.1	ENSG00000150995;	0.4920741	0.6853547	FALSE
67	3	TC11000198.hg.1	ENSG00000133816;	0.4941868	0.6853547	FALSE
17	1	TC18000097.hg.1	ENSG00000101773	0.4948111	0.6853547	FALSE
17	1	TC06004150.hg.1	ENSG00000096060	0.4948111	0.6853547	FALSE
17	1	TC06004150.hg.1	ENSG00000096060	0.4948111	0.6853547	FALSE
17	1	TC04000253.hg.1	ENSG00000154277	0.4948111	0.6853547	FALSE
17	1	TC01000785.hg.1	ENSG00000154027	0.4948111	0.6853547	FALSE
17	1	TC07001534.hg.1	ENSG00000127946	0.4948111	0.6853547	FALSE
17	1	TC15001136.hg.1	ENSG00000166664	0.4948111	0.6853547	FALSE
17	1	TC06001027.hg.1	ENSG00000118503	0.4948111	0.6853547	FALSE
17	1	TC06000945.hg.1	ENSG00000152661	0.4948111	0.6853547	FALSE
1818	72	TC01000685.hg.1	ENSG00000162409;	0.4959245	0.6853547	FALSE
42	2	TC02000082.hg.1	ENSG00000071575;	0.4963365	0.6853547	FALSE
42	2	TC02000041.hg.1	ENSG00000115738;I	0.4963365	0.6853547	FALSE
1819	72	TC01000685.hg.1	ENSG00000162409;	0.497934	0.6857511	FALSE
1845	73	TC01000685.hg.1	ENSG00000162409;	0.4991441	0.6862139	FALSE
477	19	TC01000394.hg.1	ENSG00000159023;	0.5119147	0.6986398	FALSE
299	12	TC01003497.hg.1	ENSG00000117479;I	0.5139725	0.6986398	FALSE
299	12	TC06000004.hg.1	ENSG00000112679;I	0.5139725	0.6986398	FALSE
18	1	TC01000643.hg.1	ENSG00000116157	0.5147113	0.6986398	FALSE
18	1	TC06001029.hg.1	ENSG00000112379	0.5147113	0.6986398	FALSE
18	1	TC08000694.hg.1	ENSG00000184374	0.5147113	0.6986398	FALSE
18	1	TC03000214.hg.1	ENSG00000168036	0.5147113	0.6986398	FALSE

18	1	TC08001659.hg.1	ENSG00000104419	0.5147113	0.6986398	FALSE
18	1	TC19001293.hg.1	ENSG00000105664	0.5147113	0.6986398	FALSE
1854	73	TC01000685.hg.1	ENSG00000162409;	0.5170825	0.6986398	FALSE
1829	72	TC01000685.hg.1	ENSG00000162409;	0.5179866	0.6986589	FALSE
44	2	TC02002624.hg.1	ENSG00000115415;I	0.5212476	0.7000502	FALSE
44	2	TC02002624.hg.1	ENSG00000115415;I	0.5212476	0.7000502	FALSE
44	2	TC06000004.hg.1	ENSG00000112679;I	0.5212476	0.7000502	FALSE
44	2	TC04001001.hg.1	ENSG00000074211;I	0.5212476	0.7000502	FALSE
19	1	TC10001517.hg.1	ENSG00000148677	0.5338288	0.7132862	FALSE
19	1	TC06000945.hg.1	ENSG00000152661	0.5338288	0.7132862	FALSE
72	3	TC12001324.hg.1	ENSG00000123095;	0.5428113	0.7234412	FALSE
46	2	TC11000404.hg.1	ENSG00000134574;	0.5453196	0.7255524	FALSE
20	1	TC17001353.hg.1	ENSG00000176658	0.5521944	0.728543	FALSE
20	1	TC03003381.hg.1	ENSG00000225697	0.5521944	0.728543	FALSE
20	1	TC14000353.hg.1	ENSG00000165617	0.5521944	0.728543	FALSE
20	1	TC09000015.hg.1	ENSG00000147852	0.5521944	0.728543	FALSE
73	3	TC06004150.hg.1	ENSG00000096060;	0.5522078	0.728543	FALSE
47	2	TC03000131.hg.1	ENSG00000077092;	0.5570347	0.7330633	FALSE
47	2	TC03000013.hg.1	ENSG00000150995;	0.5570347	0.7330633	FALSE
48	2	TC03000262.hg.1	ENSG00000160801;	0.5685336	0.7363078	FALSE
21	1	TC02002624.hg.1	ENSG00000115415	0.5698374	0.7363078	FALSE
21	1	TC05000165.hg.1	ENSG00000079215	0.5698374	0.7363078	FALSE
21	1	TC10000811.hg.1	ENSG00000150594	0.5698374	0.7363078	FALSE
21	1	TC09000358.hg.1	ENSG00000135069	0.5698374	0.7363078	FALSE
21	1	TC09000965.hg.1	ENSG00000147883	0.5698374	0.7363078	FALSE
21	1	TC04002929.hg.1	ENSG00000109743	0.5698374	0.7363078	FALSE
21	1	TC05000054.hg.1	ENSG00000078295	0.5698374	0.7363078	FALSE
21	1	TC09000965.hg.1	ENSG00000147883	0.5698374	0.7363078	FALSE
21	1	TC09000038.hg.1	ENSG00000197646	0.5698374	0.7363078	FALSE
128	5	TC02000891.hg.1	ENSG00000150540;	0.5702868	0.7363078	FALSE
1806	70	TC01000685.hg.1	ENSG00000162409;	0.5738923	0.7392547	FALSE
155	6	TC05000054.hg.1	ENSG00000078295;	0.5744527	0.7392547	FALSE
182	7	TC02000996.hg.1	ENSG00000172292;	0.5784464	0.7431099	FALSE
49	2	TC10001517.hg.1	ENSG00000148677;	0.5798151	0.7431099	FALSE
49	2	TC03000131.hg.1	ENSG00000077092;	0.5798151	0.7431099	FALSE
103	4	TC01000734.hg.1	ENSG00000118473;I	0.5815518	0.7435149	FALSE
22	1	TC05000054.hg.1	ENSG00000078295	0.5867864	0.7453517	FALSE
22	1	TC22001477.hg.1	ENSG00000100344	0.5867864	0.7453517	FALSE
22	1	TC05000054.hg.1	ENSG00000078295	0.5867864	0.7453517	FALSE
22	1	TC0Y000185.hg.1	ENSG00000012817	0.5867864	0.7453517	FALSE
22	1	TC20000162.hg.1	ENSG00000101463	0.5867864	0.7453517	FALSE
22	1	TC18000164.hg.1	ENSG00000141469	0.5867864	0.7453517	FALSE
22	1	TC02001750.hg.1	ENSG00000138061	0.5867864	0.7453517	FALSE
239	9	TC03000013.hg.1	ENSG00000150995;	0.6011868	0.7568487	FALSE
51	2	TC03000634.hg.1	ENSG00000173193;	0.6017234	0.7568487	FALSE
51	2	TC13000799.hg.1	ENSG00000125257;	0.6017234	0.7568487	FALSE
23	1	TC05000165.hg.1	ENSG00000079215	0.6030686	0.7568487	FALSE
23	1	TC09000358.hg.1	ENSG00000135069	0.6030686	0.7568487	FALSE
79	3	TC03000013.hg.1	ENSG00000150995;	0.6060967	0.7588293	FALSE
134	5	TC02000891.hg.1	ENSG00000150540;	0.6111218	0.7627676	FALSE
241	9	TC06000120.hg.1	ENSG00000137393;	0.6111857	0.7627676	FALSE
52	2	TC02002624.hg.1	ENSG00000115415;I	0.61235	0.7630075	FALSE
24	1	TC18000574.hg.1	ENSG00000166347	0.6187101	0.7656467	FALSE
24	1	TC07000810.hg.1	ENSG00000128510	0.6187101	0.7656467	FALSE
24	1	TC09002910.hg.1	ENSG00000107317	0.6187101	0.7656467	FALSE
24	1	TC18000574.hg.1	ENSG00000166347	0.6187101	0.7656467	FALSE
24	1	TC18000574.hg.1	ENSG00000166347	0.6187101	0.7656467	FALSE
24	1	TC18000574.hg.1	ENSG00000166347	0.6187101	0.7656467	FALSE

24	1	TC14000067.hg.1	ENSG00000181784	0.6187101	0.7656467	FALSE
53	2	TC01002195.hg.1	ENSG00000116649;I	0.6227585	0.7656467	FALSE
53	2	TC04001001.hg.1	ENSG00000074211;I	0.6227585	0.7656467	FALSE
137	5	TC02000996.hg.1	ENSG00000172292;	0.6306727	0.7730892	FALSE
25	1	TC10001517.hg.1	ENSG00000148677	0.6337362	0.7730892	FALSE
25	1	TC03000634.hg.1	ENSG00000173193	0.6337362	0.7730892	FALSE
25	1	TC13000845.hg.1	ENSG00000102452	0.6337362	0.7730892	FALSE
25	1	TC03003381.hg.1	ENSG00000225697	0.6337362	0.7730892	FALSE
25	1	TC13000845.hg.1	ENSG00000102452	0.6337362	0.7730892	FALSE
25	1	TC15001136.hg.1	ENSG00000166664	0.6337362	0.7730892	FALSE
111	4	TC02002827.hg.1	ENSG00000135905;	0.6405871	0.7772192	FALSE
55	2	TC09000358.hg.1	ENSG00000135069;	0.6429245	0.7788514	FALSE
248	9	TC03001466.hg.1	ENSG00000163931;	0.6451525	0.779792	FALSE
329	12	TC01003605.hg.1	ENSG00000135821;	0.6474052	0.779792	FALSE
26	1	TC01001710.hg.1	ENSG00000117266	0.648171	0.779792	FALSE
26	1	TC02001750.hg.1	ENSG00000138061	0.648171	0.779792	FALSE
26	1	TC15001136.hg.1	ENSG00000166664	0.648171	0.779792	FALSE
26	1	TC01001710.hg.1	ENSG00000117266	0.648171	0.779792	FALSE
56	2	TC13000799.hg.1	ENSG00000125257;	0.6526841	0.7816278	FALSE
56	2	TC13000799.hg.1	ENSG00000125257;	0.6526841	0.7816278	FALSE
142	5	TC01000394.hg.1	ENSG00000159023;	0.6618972	0.7880207	FALSE
27	1	TC11002162.hg.1	ENSG00000137501	0.6620378	0.7880207	FALSE
27	1	TC18000565.hg.1	ENSG00000166479	0.6620378	0.7880207	FALSE
27	1	TC12000227.hg.1	ENSG00000172572	0.6620378	0.7880207	FALSE
27	1	TC14000951.hg.1	ENSG00000092068	0.6620378	0.7880207	FALSE
281	10	TC01001792.hg.1	ENSG00000082482;	0.6720614	0.7969308	FALSE
28	1	TC14002310.hg.1	ENSG00000168398	0.6753588	0.799633	FALSE
256	9	TC06000120.hg.1	ENSG00000137393;	0.6818658	0.8061214	FALSE
29	1	TC07001332.hg.1	ENSG00000164708	0.6881556	0.8123341	FALSE
60	2	TC13000799.hg.1	ENSG00000125257;	0.6896032	0.8128206	FALSE
30	1	TC14002310.hg.1	ENSG00000168398	0.7004487	0.8225164	FALSE
30	1	TC10002939.hg.1	ENSG00000151303	0.7004487	0.8225164	FALSE
30	1	TC03000262.hg.1	ENSG00000160801	0.7004487	0.8225164	FALSE
30	1	TC19001220.hg.1	ENSG00000141837	0.7004487	0.8225164	FALSE
121	4	TC01003129.hg.1	ENSG00000143140;	0.7063168	0.8256279	FALSE
121	4	TC01003129.hg.1	ENSG00000143140;	0.7063168	0.8256279	FALSE
262	9	TC01001112.hg.1;	ENSG00000131778;	0.7078313	0.8256279	FALSE
31	1	TC12000531.hg.1	ENSG00000166986	0.7122579	0.8289436	FALSE
31	1	TC08001361.hg.1	ENSG00000076641	0.7122579	0.8289436	FALSE
123	4	TC02002827.hg.1	ENSG00000135905;	0.7183583	0.8311831	FALSE
152	5	TC01000734.hg.1	ENSG00000118473;I	0.7190286	0.8311831	FALSE
209	7	TC06000004.hg.1	ENSG00000112679;I	0.7200363	0.8311831	FALSE
32	1	TC18000097.hg.1	ENSG00000101773	0.7236023	0.8311831	FALSE
32	1	TC18000565.hg.1	ENSG00000166479	0.7236023	0.8311831	FALSE
32	1	TC18000574.hg.1	ENSG00000166347	0.7236023	0.8311831	FALSE
32	1	TC03002146.hg.1	ENSG00000189058	0.7236023	0.8311831	FALSE
2097	78	TC01001907.hg.1	ENSG00000143641;	0.7240093	0.8311831	FALSE
124	4	TC01000972.hg.1	ENSG00000064886;	0.724241	0.8311831	FALSE
125	4	TC03000214.hg.1	ENSG00000168036;	0.7300321	0.8366061	FALSE
33	1	TC04001084.hg.1	ENSG00000109819	0.7345	0.8404993	FALSE
34	1	TC03002008.hg.1	ENSG00000144959	0.7449688	0.8506189	FALSE
34	1	TC06001027.hg.1	ENSG00000118503	0.7449688	0.8506189	FALSE
98	3	TC01000972.hg.1	ENSG00000064886;	0.7467998	0.8508532	FALSE
328	11	TC01001112.hg.1;	ENSG00000131778;	0.7479485	0.850927	FALSE
35	1	TC04001084.hg.1	ENSG00000109819	0.7550253	0.857735	FALSE
131	4	TC02002827.hg.1	ENSG00000135905;	0.762872	0.8653967	FALSE
36	1	TC01001112.hg.1	ENSG00000131778	0.7646859	0.8655782	FALSE
36	1	TC08002613.hg.1	ENSG00000134013	0.7646859	0.8655782	FALSE

37	1	TC07000810.hg.1	ENSG00000128510	0.7739661	0.8729359	FALSE
37	1	TC07001552.hg.1	ENSG00000187391	0.7739661	0.8729359	FALSE
37	1	TC11002175.hg.1	ENSG00000109861	0.7739661	0.8729359	FALSE
72	2	TC02002827.hg.1	ENSG00000135905;	0.7812884	0.8786696	FALSE
38	1	TC03002146.hg.1	ENSG00000189058	0.7828809	0.879201	FALSE
105	3	TC11002175.hg.1	ENSG00000109861;	0.787352	0.8821354	FALSE
73	2	TC02002605.hg.1	ENSG00000064989;	0.7877413	0.8821354	FALSE
226	7	TC01001624.hg.1	ENSG00000116741;I	0.7906755	0.8831329	FALSE
39	1	TC21000241.hg.1	ENSG00000197381	0.7914445	0.8831329	FALSE
39	1	TC03001884.hg.1	ENSG00000047457	0.7914445	0.8831329	FALSE
74	2	TC01001112.hg.1;	ENSG00000131778;	0.7940261	0.8841284	FALSE
107	3	TC01002166.hg.1	ENSG00000116285;I	0.7979015	0.8871851	FALSE
40	1	TC19001220.hg.1	ENSG00000141837	0.799671	0.8878949	FALSE
2389	87	TC01000685.hg.1	ENSG00000162409;	0.8028006	0.8901109	FALSE
76	2	TC05000165.hg.1	ENSG00000079215;	0.806104	0.8925129	FALSE
41	1	TC06004150.hg.1	ENSG00000096060	0.8075734	0.8928804	FALSE
293	9	TC06000120.hg.1	ENSG00000137393;	0.8194728	0.9017157	FALSE
2246	81	TC01000685.hg.1	ENSG00000162409;	0.8209483	0.9017157	FALSE
2246	81	TC01000685.hg.1	ENSG00000162409;	0.8209483	0.9017157	FALSE
112	3	TC03000214.hg.1	ENSG00000168036;	0.822371	0.9017157	FALSE
43	1	TC11000174.hg.1	ENSG00000166483	0.8224566	0.9017157	FALSE
43	1	TC06004150.hg.1	ENSG00000096060	0.8224566	0.9017157	FALSE
43	1	TC15000971.hg.1	ENSG00000184254	0.8224566	0.9017157	FALSE
80	2	TC05000159.hg.1	ENSG00000168685;	0.8283836	0.9024633	FALSE
44	1	TC18000097.hg.1	ENSG00000101773	0.8294615	0.9024633	FALSE
44	1	TC06001027.hg.1	ENSG00000118503	0.8294615	0.9024633	FALSE
44	1	TC05000165.hg.1	ENSG00000079215	0.8294615	0.9024633	FALSE
44	1	TC05000165.hg.1	ENSG00000079215	0.8294615	0.9024633	FALSE
44	1	TC05000165.hg.1	ENSG00000079215	0.8294615	0.9024633	FALSE
44	1	TC17001301.hg.1	ENSG00000109107	0.8294615	0.9024633	FALSE
861	29	TC01003042.hg.1	ENSG00000092607;	0.8335435	0.9025264	FALSE
45	1	TC14000067.hg.1	ENSG00000181784	0.8361904	0.9041453	FALSE
82	2	TC03000013.hg.1	ENSG00000150995;	0.8386365	0.9048829	FALSE
82	2	TC03000013.hg.1	ENSG00000150995;	0.8386365	0.9048829	FALSE
865	29	TC01003042.hg.1	ENSG00000092607;	0.8403308	0.9048829	FALSE
46	1	TC03000013.hg.1	ENSG00000150995	0.8426542	0.9049022	FALSE
46	1	TC03000013.hg.1	ENSG00000150995	0.8426542	0.9049022	FALSE
46	1	TC06004150.hg.1	ENSG00000096060	0.8426542	0.9049022	FALSE
335	10	TC01006414.hg.1	ENSG00000143248;	0.8537786	0.9132602	FALSE
48	1	TC05001110.hg.1	ENSG00000153395	0.8548278	0.9132602	FALSE
48	1	TC02000996.hg.1	ENSG00000172292	0.8548278	0.9132602	FALSE
120	3	TC08000203.hg.1	ENSG00000092964;	0.8562541	0.9132602	FALSE
49	1	TC01001112.hg.1	ENSG00000131778	0.8605572	0.9155331	FALSE
49	1	TC02002827.hg.1	ENSG00000135905	0.8605572	0.9155331	FALSE
87	2	TC12000803.hg.1	ENSG00000166598;	0.861884	0.9155331	FALSE
50	1	TC02002143.hg.1	ENSG00000204634	0.8660608	0.9187267	FALSE
91	2	TC16001088.hg.1	ENSG00000171208;	0.8782297	0.9303783	FALSE
288	8	TC01002166.hg.1	ENSG00000116285;I	0.8841088	0.9348149	FALSE
54	1	TC12000531.hg.1	ENSG00000166986	0.8859901	0.9348149	FALSE
54	1	TC12000531.hg.1	ENSG00000166986	0.8859901	0.9348149	FALSE
54	1	TC12000531.hg.1	ENSG00000166986	0.8859901	0.9348149	FALSE
94	2	TC03000013.hg.1	ENSG00000150995;	0.8892972	0.9357886	FALSE
414	12	TC01001624.hg.1	ENSG00000116741;I	0.8945557	0.9400619	FALSE
59	1	TC11002162.hg.1	ENSG00000137501	0.9067902	0.9513874	FALSE
422	12	TC01001624.hg.1	ENSG00000116741;I	0.9077569	0.9513874	FALSE
137	3	TC08000255.hg.1	ENSG00000157168;	0.9100148	0.9516911	FALSE
60	1	TC15001136.hg.1	ENSG00000166664	0.9104714	0.9516911	FALSE
207	5	TC01002847.hg.1	ENSG00000117228;I	0.914138	0.9532678	FALSE

102	2	TC08002613.hg.1	ENSG00000134013;	0.9144085	0.9532678	FALSE
175	4	TC11000198.hg.1	ENSG00000133816;	0.9175198	0.9552428	FALSE
104	2	TC03000013.hg.1	ENSG00000150995;	0.919794	0.9563421	FALSE
496	14	TC01003605.hg.1	ENSG00000135821;	0.9268748	0.9624296	FALSE
66	1	TC03002146.hg.1	ENSG00000189058	0.9297021	0.9640901	FALSE
109	2	TC02002827.hg.1	ENSG00000135905;	0.9318956	0.9641218	FALSE
378	10	TC01000728.hg.1	ENSG00000162433;	0.9321891	0.9641218	FALSE
357	9	TC01001624.hg.1	ENSG00000116741;I	0.9448571	0.9737847	FALSE
116	2	TC03001362.hg.1	ENSG00000160808;	0.9459658	0.9737847	FALSE
390	10	TC01000728.hg.1	ENSG00000162433;	0.9462138	0.9737847	FALSE
390	10	TC01000728.hg.1	ENSG00000162433;	0.9462138	0.9737847	FALSE
117	2	TC06000004.hg.1	ENSG00000112679;I	0.9477344	0.9737847	FALSE
74	1	TC14000067.hg.1	ENSG00000181784	0.949084	0.9738966	FALSE
80	1	TC20000926.hg.1	ENSG00000158445	0.9600292	0.9838419	FALSE
81	1	TC21000241.hg.1	ENSG00000197381	0.9616098	0.9841769	FALSE
129	2	TC01001112.hg.1;	ENSG00000131778;	0.9650793	0.9858	FALSE
129	2	TC01001112.hg.1;	ENSG00000131778;	0.9650793	0.9858	FALSE
698	19	TC01001112.hg.1;	ENSG00000131778;	0.9684109	0.9872761	FALSE
178	3	TC02002827.hg.1	ENSG00000135905;	0.9732134	0.9905258	FALSE
744	20	TC01001112.hg.1;	ENSG00000131778;	0.9756081	0.9905258	FALSE
746	20	TC01001112.hg.1;	ENSG00000131778;	0.9764131	0.9905258	FALSE
184	3	TC11002175.hg.1	ENSG00000109861;	0.9777408	0.9905258	FALSE
1861	58	TC01002530.hg.1	ENSG00000183386;	0.9794401	0.9905258	FALSE
145	2	TC0Y000351.hg.1	ENSG00000129824;	0.9798048	0.9905258	FALSE
757	20	TC01001112.hg.1;	ENSG00000131778;	0.9804313	0.9905258	FALSE
167	2	TC01001112.hg.1;	ENSG00000131778;	0.9906341	0.9986897	FALSE
801	20	TC02001437.hg.1	ENSG00000144476;	0.9910564	0.9986897	FALSE
124	1	TC11002175.hg.1	ENSG00000109861	0.9932436	0.9996105	FALSE
2075	58	TC01001112.hg.1;	ENSG00000131778;	0.9988159	0.9999999	FALSE
612	11	TC04001084.hg.1	ENSG00000109819;	0.9992695	0.9999999	FALSE
5012	157	TC01000685.hg.1	ENSG00000162409;	0.9998529	0.9999999	FALSE
4941	153	TC01000685.hg.1	ENSG00000162409;	0.9999108	0.9999999	FALSE
2991	70	TC01001112.hg.1;	ENSG00000131778;	0.9999999	0.9999999	FALSE

Names	Genes_In_Term	DEG
Total	9927	415
ko04610 Complement and coagulation cascades	157	24
ko05200 Pathways in cancer	439	44
ko04510 Focal adhesion	377	34
ko05410 Hypertrophic cardiomyopathy (HCM)	127	16
ko04350 TGF-beta signaling pathway	116	15
ko05211 Renal cell carcinoma	95	13
ko00010 Glycolysis / Gluconeogenesis	96	13
ko04512 ECM-receptor interaction	166	18
ko05412 Arrhythmogenic right ventricular cardiomyopathy (ARVC)	114	14
ko05215 Prostate cancer	118	14
ko00340 Histidine metabolism	26	6
ko00910 Nitrogen metabolism	33	6
ko04060 Cytokine-cytokine receptor interaction	309	24
ko00561 Glycerolipid metabolism	86	10
ko00051 Fructose and mannose metabolism	47	7
ko05222 Small cell lung cancer	119	12
ko04930 Type II diabetes mellitus	77	9
ko05218 Melanoma	92	10
ko04360 Axon guidance	244	19
ko00512 Mucin type O-Glycan biosynthesis	30	5
ko04640 Hematopoietic cell lineage	161	14
ko04976 Bile secretion	100	10
ko00410 beta-Alanine metabolism	46	6
ko04810 Regulation of actin cytoskeleton	332	23
ko00710 Carbon fixation in photosynthetic organisms	35	5
ko05414 Dilated cardiomyopathy	178	14
ko00360 Phenylalanine metabolism	26	4
ko04270 Vascular smooth muscle contraction	198	14
ko04710 Circadian rhythm - mammal	31	4
ko00903 Limonene and pinene degradation	8	2
ko00350 Tyrosine metabolism	47	5
ko04974 Protein digestion and absorption	167	12
ko04115 p53 signaling pathway	97	8
ko04150 mTOR signaling pathway	99	8
ko05212 Pancreatic cancer	101	8
ko00120 Primary bile acid biosynthesis	23	3
ko00625 Chloroalkane and chloroalkene degradation	11	2
ko00330 Arginine and proline metabolism	88	7
ko04310 Wnt signaling pathway	240	15
ko05144 Malaria	126	9
ko00532 Glycosaminoglycan biosynthesis - chondroitin sulfate	25	3
ko04614 Renin-angiotensin system	25	3
ko04973 Carbohydrate digestion and absorption	91	7
ko04013 MAPK signaling pathway - fly	26	3
ko05219 Bladder cancer	58	5
ko00620 Pyruvate metabolism	59	5
ko05210 Colorectal cancer	97	7
ko04972 Pancreatic secretion	154	10
ko05100 Bacterial invasion of epithelial cells	155	10
ko00564 Glycerophospholipid metabolism	120	8
ko04670 Leukocyte transendothelial migration	220	13
ko05146 Amoebiasis	241	14
ko05213 Endometrial cancer	84	6
ko04920 Adipocytokine signaling pathway	103	7
ko04520 Adherens junction	142	9
ko00533 Glycosaminoglycan biosynthesis - keratan sulfate	16	2

ko04711	Circadian rhythm - fly	16	2
ko03320	PPAR signaling pathway	123	8
ko05214	Glioma	126	8
ko00053	Ascorbate and aldarate metabolism	33	3
ko00590	Arachidonic acid metabolism	89	6
ko04960	Aldosterone-regulated sodium reabsorption	89	6
ko00680	Methane metabolism	52	4
ko04010	MAPK signaling pathway	384	20
ko00040	Pentose and glucuronate interconversions	36	3
ko00524	Butirosin and neomycin biosynthesis	5	1
ko00140	Steroid hormone biosynthesis	74	5
ko04961	Endocrine and other factor-regulated calcium reabsorptior	94	6
ko00030	Pentose phosphate pathway	37	3
ko05160	Hepatitis C	197	11
ko05142	Chagas disease (American trypanosomiasis)	198	11
ko00604	Glycosphingolipid biosynthesis - ganglio series	21	2
ko04380	Osteoclast differentiation	180	10
ko00052	Galactose metabolism	39	3
ko04964	Proximal tubule bicarbonate reclamation	58	4
ko04260	Cardiac muscle contraction	100	6
ko02010	ABC transporters	60	4
ko00730	Thiamine metabolism	7	1
ko04112	Cell cycle - Caulobacter	7	1
ko00380	Tryptophan metabolism	62	4
ko04540	Gap junction	124	7
ko04978	Mineral absorption	63	4
ko05217	Basal cell carcinoma	63	4
ko04630	Jak-STAT signaling pathway	190	10
ko04370	VEGF signaling pathway	126	7
ko00250	Alanine, aspartate and glutamate metabolism	44	3
ko00500	Starch and sucrose metabolism	85	5
ko04062	Chemokine signaling pathway	263	13
ko05120	Epithelial cell signaling in Helicobacter pylori infection	88	5
ko04910	Insulin signaling pathway	243	12
ko00750	Vitamin B6 metabolism	9	1
ko05145	Toxoplasmosis	266	13
ko05221	Acute myeloid leukemia	90	5
ko05223	Non-small cell lung cancer	90	5
ko04724	Glutamatergic synapse	158	8
ko00534	Glycosaminoglycan biosynthesis - heparan sulfate	29	2
ko00520	Amino sugar and nucleotide sugar metabolism	93	5
ko04012	ErbB signaling pathway	139	7
ko04142	Lysosome	163	8
ko00565	Ether lipid metabolism	53	3
ko00230	Purine metabolism	257	12
ko04971	Gastric acid secretion	143	7
ko04916	Melanogenesis	145	7
ko00760	Nicotinate and nicotinamide metabolism	33	2
ko05143	African trypanosomiasis	101	5
ko00130	Ubiquinone and other terpenoid-quinone biosynthesis	13	1
ko00950	Isoquinoline alkaloid biosynthesis	13	1
ko04977	Vitamin digestion and absorption	13	1
ko04530	Tight junction	288	13
ko05020	Prion diseases	58	3
ko02020	Two-component system	14	1
ko00480	Glutathione metabolism	83	4
ko04912	GnRH signaling pathway	154	7
ko04914	Progesterone-mediated oocyte maturation	131	6

ko04621 NOD-like receptor signaling pathway	108	5
ko00514 Other types of O-glycan biosynthesis	62	3
ko04722 Neurotrophin signaling pathway	228	10
ko00521 Streptomycin biosynthesis	16	1
ko04330 Notch signaling pathway	89	4
ko00100 Steroid biosynthesis	41	2
ko05216 Thyroid cancer	41	2
ko04144 Endocytosis	380	16
ko00640 Propanoate metabolism	43	2
ko05131 Shigellosis	117	5
ko05220 Chronic myeloid leukemia	118	5
ko04320 Dorso-ventral axis formation	69	3
ko04020 Calcium signaling pathway	364	15
ko04514 Cell adhesion molecules (CAMs)	340	14
ko05150 Staphylococcus aureus infection	268	11
ko00270 Cysteine and methionine metabolism	71	3
ko00860 Porphyrin and chlorophyll metabolism	46	2
ko00600 Sphingolipid metabolism	72	3
ko05140 Leishmaniasis	223	9
ko04730 Long-term depression	100	4
ko00450 Selenocompound metabolism	22	1
ko00531 Glycosaminoglycan degradation	22	1
ko04620 Toll-like receptor signaling pathway	152	6
ko00920 Sulfur metabolism	23	1
ko00980 Metabolism of xenobiotics by cytochrome P450	77	3
ko04742 Taste transduction	51	2
ko04080 Neuroactive ligand-receptor interaction	357	14
ko00260 Glycine, serine and threonine metabolism	52	2
ko04970 Salivary secretion	183	7
ko00982 Drug metabolism - cytochrome P450	81	3
ko05016 Huntingtons disease	239	9
ko04110 Cell cycle	162	6
ko00563 Glycosylphosphatidylinositol(GPI)-anchor biosynthesis	27	1
ko00071 Fatty acid metabolism	56	2
ko04070 Phosphatidylinositol signaling system	164	6
ko04975 Fat digestion and absorption	58	2
ko04950 Maturity onset diabetes of the young	29	1
ko04622 RIG-I-like receptor signaling pathway	90	3
ko00280 Valine, leucine and isoleucine degradation	63	2
ko00592 alpha-Linolenic acid metabolism	33	1
ko04720 Long-term potentiation	129	4
ko00591 Linoleic acid metabolism	38	1
ko00830 Retinol metabolism	72	2
ko05340 Primary immunodeficiency	106	3
ko04662 B cell receptor signaling pathway	166	5
ko04650 Natural killer cell mediated cytotoxicity	307	10
ko04664 Fc epsilon RI signaling pathway	169	5
ko03410 Base excision repair	43	1
ko04140 Regulation of autophagy	43	1
ko00310 Lysine degradation	80	2
ko04120 Ubiquitin mediated proteolysis	205	6
ko04666 Fc gamma R-mediated phagocytosis	206	6
ko05110 Vibrio cholerae infection	120	3
ko04114 Oocyte meiosis	187	5
ko05323 Rheumatoid arthritis	281	8
ko04962 Vasopressin-regulated water reabsorption	58	1
ko04113 Meiosis - yeast	97	2
ko04210 Apoptosis	133	3

ko04626 Plant-pathogen interaction	99	2
ko03022 Basal transcription factors	61	1
ko05010 Alzheimers disease	233	6
ko00562 Inositol phosphate metabolism	106	2
ko05130 Pathogenic Escherichia coli infection	108	2
ko00970 Aminoacyl-tRNA biosynthesis	69	1
ko04111 Cell cycle - yeast	113	2
ko03420 Nucleotide excision repair	74	1
ko04623 Cytosolic DNA-sensing pathway	74	1
ko03015 mRNA surveillance pathway	85	1
ko00240 Pyrimidine metabolism	135	2
ko04660 T cell receptor signaling pathway	221	4
ko04672 Intestinal immune network for IgA production	221	4
ko05012 Parkinsons disease	159	2
ko04146 Peroxisome	116	1
ko05162 Measles	291	5
ko03018 RNA degradation	126	1
ko03050 Proteasome	646	15
ko04145 Phagosome	423	8
ko00190 Oxidative phosphorylation	144	1
ko05152 Tuberculosis	405	7
ko05416 Viral myocarditis	369	6
ko03013 RNA transport	252	3
ko05332 Graft-versus-host disease	211	2
ko03010 Ribosome	158	1
ko04141 Protein processing in endoplasmic reticulum	262	3
ko05322 Systemic lupus erythematosus	380	5
ko05310 Asthma	206	1
ko04940 Type I diabetes mellitus	227	1
ko05330 Allograft rejection	261	1
ko05320 Autoimmune thyroid disease	272	1
ko04612 Antigen processing and presentation	295	1

Input_gene	Gene_Symbol	P_value	Q_value	Significant
-	-	-	-	-
TC01001736.hg.1;	ENSG00000196352;	3.132E-08	5.258E-06	TRUE
TC01001805.hg.1;	ENSG00000092969;	5.155E-08	5.258E-06	TRUE
TC01000789.hg.1;	ENSG00000162614;	1.849E-05	0.0012576	TRUE
TC01000685.hg.1;	ENSG00000162409;	7.495E-05	0.0037846	TRUE
TC01001805.hg.1;	ENSG00000092969;	9.276E-05	0.0037846	TRUE
TC01001805.hg.1;	ENSG00000092969;	0.0001516	0.0049199	TRUE
TC02000245.hg.1;	ENSG00000143891;	0.0001688	0.0049199	TRUE
TC01001095.hg.1;	ENSG00000143127;	0.0001937	0.0049396	TRUE
TC01001095.hg.1;	ENSG00000143127;	0.0002741	0.006214	TRUE
TC01002616.hg.1;	ENSG00000117461;	0.0003931	0.0080186	TRUE
TC02000891.hg.1;	ENSG00000150540;	0.0005809	0.0107729	TRUE
TC01003605.hg.1;	ENSG00000135821;	0.0021819	0.0370916	TRUE
TC01001805.hg.1;	ENSG00000092969;	0.0025558	0.0401056	TRUE
TC06001313.hg.1;	ENSG00000172197;	0.0030242	0.0426504	TRUE
TC02000466.hg.1;	ENSG00000159399;	0.0031361	0.0426504	TRUE
TC01002616.hg.1;	ENSG00000117461;	0.0040387	0.0514934	FALSE
TC01002503.hg.1;	ENSG00000171812;	0.0046473	0.0557681	FALSE
TC01002616.hg.1;	ENSG00000117461;	0.0049361	0.0559419	FALSE
TC01001624.hg.1;	ENSG00000116741;	0.0067209	0.0721607	FALSE
TC01001907.hg.1;	ENSG00000143641;	0.0074829	0.0727476	FALSE
TC01001736.hg.1;	ENSG00000196352;	0.0074887	0.0727476	FALSE
TC01001473.hg.1;	ENSG00000143153;	0.0088304	0.0818819	FALSE
TC01002195.hg.1;	ENSG00000116649;	0.0117002	0.1022221	FALSE
TC01000789.hg.1;	ENSG00000162614;	0.0120261	0.1022221	FALSE
TC03001466.hg.1;	ENSG00000163931;	0.0143782	0.117326	FALSE
TC01001095.hg.1;	ENSG00000143127;	0.0170988	0.1341596	FALSE
TC0X000207.hg.1	ENSG00000189221;	0.0216996	0.1639527	FALSE
TC01000789.hg.1;	ENSG00000162614;	0.0380705	0.273107	FALSE
TC01000100.hg.1;	ENSG00000049246;	0.038824	0.273107	FALSE
TC09000222.hg.1;	ENSG00000137124;	0.0413009	0.2808458	FALSE
TC06002113.hg.1;	ENSG00000079931;	0.0453512	0.2984403	FALSE
TC01001473.hg.1;	ENSG00000143153;	0.0472497	0.3012169	FALSE
TC02002765.hg.1;	ENSG00000079308;	0.0497355	0.307456	FALSE
TC01000685.hg.1;	ENSG00000162409;	0.0549016	0.3294098	FALSE
TC01001805.hg.1;	ENSG00000092969;	0.0603985	0.3520372	FALSE
TC06001772.hg.1;	ENSG00000146233;	0.0691616	0.3919157	FALSE
TC09000222.hg.1;	ENSG00000137124;	0.0747195	0.4012834	FALSE
TC01002195.hg.1;	ENSG00000116649;	0.0747489	0.4012834	FALSE
TC02002714.hg.1;	ENSG00000163251;	0.0782154	0.4076661	FALSE
TC01000904.hg.1;	ENSG00000162692;	0.0812165	0.4076661	FALSE
TC05000619.hg.1;	ENSG00000198108;	0.0845605	0.4076661	FALSE
TC03000846.hg.1;	ENSG00000196549;	0.0845605	0.4076661	FALSE
TC01001473.hg.1;	ENSG00000143153;	0.0859296	0.4076661	FALSE
TC06004055.hg.1;	ENSG00000047936;	0.0927597	0.4264469	FALSE
TC04000408.hg.1;	ENSG00000169429;	0.0940692	0.4264469	FALSE
TC07001883.hg.1;	ENSG00000085662;	0.0994561	0.4410661	FALSE
TC01001805.hg.1;	ENSG00000092969;	0.1109792	0.4720244	FALSE
TC01001473.hg.1;	ENSG00000143153;	0.1110646	0.4720244	FALSE
TC01002530.hg.1;	ENSG00000183386;	0.1145794	0.4770243	FALSE
TC01001607.hg.1;	ENSG00000116711;I	0.1293312	0.5026062	FALSE
TC01000904.hg.1;	ENSG00000162692;	0.1319086	0.5026062	FALSE
TC01001805.hg.1;	ENSG00000092969;	0.1336722	0.5026062	FALSE
TC01002616.hg.1;	ENSG00000117461;	0.1387917	0.5026062	FALSE
TC01000685.hg.1;	ENSG00000162409;	0.139471	0.5026062	FALSE
TC03000214.hg.1;	ENSG00000168036;	0.1402341	0.5026062	FALSE
TC11001589.hg.1;	ENSG00000175264;	0.1424328	0.5026062	FALSE

TC01000100.hg.1;	ENSG00000049246;	0.1424328	0.5026062	FALSE
TC01002503.hg.1;	ENSG00000171812;	0.1428978	0.5026062	FALSE
TC01002616.hg.1;	ENSG00000117461;	0.1571398	0.5374235	FALSE
TC04002948.hg.1;	ENSG00000137463;	0.1580657	0.5374235	FALSE
TC01000643.hg.1;	ENSG00000116157;	0.1680238	0.555594	FALSE
TC01001473.hg.1;	ENSG00000143153;	0.1680238	0.555594	FALSE
TC07001332.hg.1;	ENSG00000164708;	0.1715805	0.555594	FALSE
TC01001607.hg.1;	ENSG00000116711;I	0.1827953	0.5826601	FALSE
TC07001883.hg.1;	ENSG00000085662;	0.189422	0.5894088	FALSE
TC02000466.hg.1	ENSG00000159399	0.1923001	0.5894088	FALSE
TC02001750.hg.1;	ENSG00000138061;	0.1966654	0.5894088	FALSE
TC01001473.hg.1;	ENSG00000143153;	0.1995905	0.5894088	FALSE
TC03001466.hg.1;	ENSG00000163931;	0.2002076	0.5894088	FALSE
TC01002616.hg.1;	ENSG00000117461;	0.2022481	0.5894088	FALSE
TC01001805.hg.1;	ENSG00000092969;	0.2067261	0.5939736	FALSE
TC01000783.hg.1;	ENSG00000117069;	0.2183551	0.6077033	FALSE
TC01001805.hg.1;	ENSG00000092969;	0.2211572	0.6077033	FALSE
TC02000466.hg.1;	ENSG00000159399;	0.2221879	0.6077033	FALSE
TC01001473.hg.1;	ENSG00000143153;	0.2234203	0.6077033	FALSE
TC01001473.hg.1;	ENSG00000143153;	0.240055	0.6399211	FALSE
TC13000799.hg.1;	ENSG00000125257;	0.2415388	0.6399211	FALSE
TC12000656.hg.1	ENSG00000067798	0.2584565	0.6589568	FALSE
TC19000377.hg.1	ENSG00000160161	0.2584565	0.6589568	FALSE
TC02001750.hg.1;	ENSG00000138061;	0.2599773	0.6589568	FALSE
TC03000013.hg.1;	ENSG00000150995;	0.2616446	0.6589568	FALSE
TC01001473.hg.1;	ENSG00000143153;	0.2692983	0.6591813	FALSE
TC02002714.hg.1;	ENSG00000163251;	0.2692983	0.6591813	FALSE
TC01002616.hg.1;	ENSG00000117461;	0.2721614	0.6591813	FALSE
TC01001607.hg.1;	ENSG00000116711;I	0.2746589	0.6591813	FALSE
TC01003605.hg.1;	ENSG00000135821;	0.2788585	0.6614784	FALSE
TC02000466.hg.1;	ENSG00000159399;	0.2824088	0.6621999	FALSE
TC01002530.hg.1;	ENSG00000183386;	0.30676	0.7037198	FALSE
TC03000743.hg.1;	ENSG00000114098;	0.307015	0.7037198	FALSE
TC01000685.hg.1;	ENSG00000162409;	0.3179919	0.7060143	FALSE
TC09000358.hg.1	ENSG00000135069	0.3192062	0.7060143	FALSE
TC01001607.hg.1;	ENSG00000116711;I	0.3209821	0.7060143	FALSE
TC01002616.hg.1;	ENSG00000117461;	0.3235899	0.7060143	FALSE
TC01002616.hg.1;	ENSG00000117461;	0.3235899	0.7060143	FALSE
TC01001607.hg.1;	ENSG00000116711;I	0.3408766	0.7300014	FALSE
TC02002292.hg.1;	ENSG00000136720;	0.34353	0.7300014	FALSE
TC01000972.hg.1;	ENSG00000064886;	0.348622	0.7331844	FALSE
TC01002616.hg.1;	ENSG00000117461;	0.362409	0.7544023	FALSE
TC01002441.hg.1;	ENSG00000162511;	0.3727217	0.7680326	FALSE
TC01001607.hg.1;	ENSG00000116711;I	0.3828222	0.7800143	FALSE
TC01000728.hg.1;	ENSG00000162433;	0.3890343	0.7800143	FALSE
TC01000789.hg.1;	ENSG00000162614;	0.3900071	0.7800143	FALSE
TC02002714.hg.1;	ENSG00000163251;	0.4038261	0.7926469	FALSE
TC04002929.hg.1;	ENSG00000109743;	0.4040945	0.7926469	FALSE
TC01000904.hg.1;	ENSG00000162692;	0.4155965	0.8022759	FALSE
TC12002066.hg.1	ENSG00000158104	0.4262131	0.8022759	FALSE
TC0X000207.hg.1	ENSG00000189221	0.4262131	0.8022759	FALSE
TC16000193.hg.1	ENSG00000103222	0.4262131	0.8022759	FALSE
TC01000394.hg.1;	ENSG00000159023;	0.428667	0.8022759	FALSE
TC05000701.hg.1;	ENSG00000120738;	0.4393785	0.8148474	FALSE
TC01003605.hg.1	ENSG00000135821	0.4502318	0.827453	FALSE
TC01000643.hg.1;	ENSG00000116157;	0.4594076	0.8367781	FALSE
TC01001607.hg.1;	ENSG00000116711;I	0.4656617	0.8396159	FALSE
TC01002616.hg.1;	ENSG00000117461;	0.469293	0.8396159	FALSE

TC03000248.hg.1;	ENSG00000144791;	0.4733129	0.8396159	FALSE
TC03003359.hg.1;	ENSG00000172986;	0.4831603	0.8447682	FALSE
TC01002616.hg.1;	ENSG00000117461;	0.4844994	0.8447682	FALSE
TC02000466.hg.1	ENSG00000159399	0.4953017	0.8562843	FALSE
TC03000743.hg.1;	ENSG00000114098;	0.5140016	0.8739698	FALSE
TC01003887.hg.1;	ENSG00000143815;	0.516242	0.8739698	FALSE
TC03000214.hg.1;	ENSG00000168036;	0.516242	0.8739698	FALSE
TC01001805.hg.1;	ENSG00000092969;	0.5254819	0.8786746	FALSE
TC09000222.hg.1;	ENSG00000137124;	0.5420302	0.8958753	FALSE
TC02002827.hg.1;	ENSG00000135905;	0.5445517	0.8958753	FALSE
TC01001805.hg.1;	ENSG00000092969;	0.5521917	0.897151	FALSE
TC07000328.hg.1;	ENSG00000146648;	0.5555498	0.897151	FALSE
TC01000789.hg.1;	ENSG00000162614;	0.5613118	0.897151	FALSE
TC01000904.hg.1;	ENSG00000162692;	0.5629183	0.897151	FALSE
TC02000937.hg.1;	ENSG00000123610;	0.5691238	0.9000097	FALSE
TC01002195.hg.1;	ENSG00000116649;	0.5750958	0.9014796	FALSE
TC03001884.hg.1;	ENSG00000047457;	0.5788913	0.9014796	FALSE
TC02000996.hg.1;	ENSG00000172292;	0.5846656	0.9035742	FALSE
TC01001805.hg.1;	ENSG00000092969;	0.5914901	0.907248	FALSE
TC01001607.hg.1;	ENSG00000116711;I	0.6067767	0.9162838	FALSE
TC12000531.hg.1	ENSG00000166986	0.6095706	0.9162838	FALSE
TC05001519.hg.1	ENSG00000113273	0.6095706	0.9162838	FALSE
TC01002616.hg.1;	ENSG00000117461;	0.6153474	0.9162838	FALSE
TC01002847.hg.1	ENSG00000117228	0.6259289	0.9214912	FALSE
TC02001750.hg.1;	ENSG00000138061;	0.6304133	0.9214912	FALSE
TC19001220.hg.1;	ENSG00000141837;	0.6353936	0.9214912	FALSE
TC02002605.hg.1;	ENSG00000064989;	0.636913	0.9214912	FALSE
TC09000358.hg.1;	ENSG00000135069;	0.6459559	0.9255479	FALSE
TC01001473.hg.1;	ENSG00000143153;	0.6487909	0.9255479	FALSE
TC0X000207.hg.1	ENSG00000189221;	0.6644246	0.9412681	FALSE
TC03000013.hg.1;	ENSG00000150995;	0.6737554	0.9417541	FALSE
TC01001805.hg.1;	ENSG00000092969;	0.6764135	0.9417541	FALSE
TC18000552.hg.1	ENSG00000197563	0.6848031	0.9417541	FALSE
TC09000222.hg.1;	ENSG00000137124;	0.6857916	0.9417541	FALSE
TC01002616.hg.1;	ENSG00000117461;	0.6878498	0.9417541	FALSE
TC01001607.hg.1;	ENSG00000116711;I	0.7042933	0.9578389	FALSE
TC03001071.hg.1	ENSG00000114315	0.7106761	0.9601187	FALSE
TC04000408.hg.1;	ENSG00000169429;	0.7324664	0.983047	FALSE
TC09000222.hg.1;	ENSG00000137124;	0.7466015	0.9954687	FALSE
TC01001607.hg.1	ENSG00000116711	0.756238	0.9999972	FALSE
TC02000184.hg.1;	ENSG00000163803;	0.7937541	0.9999972	FALSE
TC01001607.hg.1	ENSG00000116711	0.8032567	0.9999972	FALSE
TC08000482.hg.1;	ENSG00000121039;	0.8097263	0.9999972	FALSE
TC01002284.hg.1;	ENSG00000117122;	0.8260767	0.9999972	FALSE
TC01002616.hg.1;	ENSG00000117461;	0.8295643	0.9999972	FALSE
TC01002616.hg.1;	ENSG00000117461;	0.8327549	0.9999972	FALSE
TC01001607.hg.1;	ENSG00000116711;I	0.8409307	0.9999972	FALSE
TC11001132.hg.1	ENSG00000110002	0.8412237	0.9999972	FALSE
TC01000685.hg.1	ENSG00000162409	0.8412237	0.9999972	FALSE
TC09000222.hg.1;	ENSG00000137124;	0.8536688	0.9999972	FALSE
TC02000432.hg.1;	ENSG00000135636;	0.8637991	0.9999972	FALSE
TC01002616.hg.1;	ENSG00000117461;	0.8667901	0.9999972	FALSE
TC07000220.hg.1;	ENSG00000164619;	0.8835443	0.9999972	FALSE
TC02000184.hg.1;	ENSG00000163803;	0.8966996	0.9999972	FALSE
TC01001805.hg.1;	ENSG00000092969;	0.9069193	0.9999972	FALSE
TC07001898.hg.1	ENSG00000182158	0.9166023	0.9999972	FALSE
TC02000184.hg.1;	ENSG00000163803;	0.9179061	0.9999972	FALSE
TC01002616.hg.1;	ENSG00000117461;	0.9210981	0.9999972	FALSE

TC09000202.hg.1;	ENSG00000122694;	0.9234202	0.9999972	FALSE
TC0X001176.hg.1	ENSG00000187325	0.9266879	0.9999972	FALSE
TC03000013.hg.1;	ENSG00000150995;	0.9290561	0.9999972	FALSE
TC02002765.hg.1;	ENSG00000079308;	0.9400939	0.9999972	FALSE
TC03000214.hg.1;	ENSG00000168036;	0.9441861	0.9999972	FALSE
TC12000531.hg.1	ENSG00000166986	0.9480226	0.9999972	FALSE
TC04001001.hg.1;	ENSG00000074211;	0.9532856	0.9999972	FALSE
TC11000404.hg.1	ENSG00000134574	0.958082	0.9999972	FALSE
TC07000137.hg.1	ENSG00000136244	0.958082	0.9999972	FALSE
TC04001001.hg.1	ENSG00000074211	0.9738952	0.9999972	FALSE
TC03000208.hg.1;	ENSG00000168032;	0.9789948	0.9999972	FALSE
TC01002616.hg.1;	ENSG00000117461;	0.9845502	0.9999972	FALSE
TC01002284.hg.1;	ENSG00000117122;	0.9845502	0.9999972	FALSE
TC04000253.hg.1;	ENSG00000154277;	0.9914308	0.9999972	FALSE
TC06004141.hg.1	ENSG00000112096	0.9931481	0.9999972	FALSE
TC01002616.hg.1;	ENSG00000117461;	0.9945281	0.9999972	FALSE
TC12000099.hg.1	ENSG00000111674	0.9955535	0.9999972	FALSE
TC01002725.hg.1;	ENSG00000132854;	0.9968736	0.9999972	FALSE
TC02001100.hg.1;	ENSG00000138448;	0.9973809	0.9999972	FALSE
TC12001622.hg.1	ENSG00000185633	0.9979606	0.9999972	FALSE
TC01001805.hg.1;	ENSG00000092969;	0.9984299	0.9999972	FALSE
TC01001736.hg.1;	ENSG00000196352;	0.9984673	0.9999972	FALSE
TC05001271.hg.1;	ENSG00000164188;	0.9986078	0.9999972	FALSE
TC06000408.hg.1;	ENSG00000223865;	0.998848	0.9999972	FALSE
TC0Y000351.hg.1	ENSG00000129824	0.9988889	0.9999972	FALSE
TC04000643.hg.1;	ENSG00000164070;	0.999034	0.9999972	FALSE
TC01003172.hg.1;	ENSG00000203818;	0.9997019	0.9999972	FALSE
TC06000408.hg.1	ENSG00000223865	0.9998624	0.9999972	FALSE
TC06000408.hg.1	ENSG00000223865	0.999945	0.9999972	FALSE
TC06000408.hg.1	ENSG00000223865	0.9999876	0.9999972	FALSE
TC06000408.hg.1	ENSG00000223865	0.9999923	0.9999972	FALSE
TC06000408.hg.1	ENSG00000223865	0.9999972	0.9999972	FALSE

Transcript Cluster	Splicing Index	PSR/Junction ID	Gene Symbo
TC02001616.hg.1	352.89	PSR02025915.hg.1	LAPTM4A
TC02001616.hg.1	-3.84	JUC02013480.hg.1	LAPTM4A
TC01002980.hg.1	159.1	JUC01024887.hg.1	DRAM2
TC01002980.hg.1	12.18	JUC01024882.hg.1	DRAM2
TC01002980.hg.1	5.02	JUC01024886.hg.1	DRAM2
TC01002980.hg.1	4.16	PSR01046254.hg.1	DRAM2
TC01002980.hg.1	-4.21	JUC01024866.hg.1	DRAM2
TC01002980.hg.1	-4.66	JUC01024864.hg.1	DRAM2
TC01002980.hg.1	-7.17	JUC01024868.hg.1	DRAM2
TC17000371.hg.1	94.15	PSR17004672.hg.1	LRRC37B
TC17000371.hg.1	2.41	JUC17002672.hg.1	LRRC37B
TC17000371.hg.1	-2.16	JUC17002669.hg.1	LRRC37B
TC18000239.hg.1	75.39	JUC18001901.hg.1	CCDC102B
TC18000239.hg.1	27.58	PSR18002816.hg.1	CCDC102B
TC18000239.hg.1	16.81	JUC18001900.hg.1	CCDC102B
TC18000239.hg.1	15.44	PSR18002819.hg.1	CCDC102B
TC18000239.hg.1	14.68	PSR18002814.hg.1	CCDC102B
TC18000239.hg.1	14.3	PSR18002834.hg.1	CCDC102B
TC18000239.hg.1	12.26	PSR18002813.hg.1	CCDC102B
TC18000239.hg.1	10.74	PSR18002817.hg.1	CCDC102B
TC18000239.hg.1	8.73	JUC18001903.hg.1	CCDC102B
TC18000239.hg.1	7.41	JUC18001899.hg.1	CCDC102B
TC18000239.hg.1	5.33	PSR18002830.hg.1	CCDC102B
TC18000239.hg.1	4.44	PSR18002818.hg.1	CCDC102B
TC18000239.hg.1	4.1	JUC18001907.hg.1	CCDC102B
TC18000239.hg.1	3.61	PSR18002820.hg.1	CCDC102B
TC18000239.hg.1	3.45	JUC18001913.hg.1	CCDC102B
TC18000239.hg.1	2.32	PSR18002821.hg.1	CCDC102B
TC18000239.hg.1	-2.35	JUC18001906.hg.1	CCDC102B
TC18000239.hg.1	-2.36	JUC18001908.hg.1	CCDC102B
TC03000214.hg.1	60.47	JUC03002262.hg.1	CTNNB1
TC03000214.hg.1	4.21	PSR03004264.hg.1	CTNNB1
TC03000214.hg.1	3.71	PSR03004296.hg.1	CTNNB1
TC03000214.hg.1	3.27	PSR03004299.hg.1	CTNNB1
TC03000214.hg.1	3.27	JUC03002273.hg.1	CTNNB1
TC03000214.hg.1	3.23	PSR03004311.hg.1	CTNNB1
TC03000214.hg.1	2.76	PSR03004298.hg.1	CTNNB1
TC03000214.hg.1	2.49	JUC03002263.hg.1	CTNNB1
TC03000214.hg.1	2.19	JUC03002271.hg.1	CTNNB1
TC03000214.hg.1	2.16	JUC03002282.hg.1	CTNNB1
TC03000214.hg.1	2.14	JUC03002275.hg.1	CTNNB1
TC03000214.hg.1	2.06	PSR03004265.hg.1	CTNNB1
TC03000214.hg.1	-2.9	JUC03002269.hg.1	CTNNB1
TC03000214.hg.1	-3.1	PSR03004261.hg.1	CTNNB1
TC03000214.hg.1	-3.23	PSR03004254.hg.1	CTNNB1
TC03000214.hg.1	-3.5	JUC03002264.hg.1	CTNNB1
TC03000214.hg.1	-3.69	PSR03004316.hg.1	CTNNB1
TC14001022.hg.1	58.92	JUC14005823.hg.1	EGLN3
TC14001022.hg.1	36.08	PSR14011679.hg.1	EGLN3
TC14001022.hg.1	31.52	JUC14005824.hg.1	EGLN3
TC14001022.hg.1	28.29	PSR14011681.hg.1	EGLN3
TC14001022.hg.1	21.36	PSR14011673.hg.1	EGLN3
TC14001022.hg.1	13.34	PSR14011672.hg.1	EGLN3
TC14001022.hg.1	9.86	PSR14011658.hg.1	EGLN3
TC14001022.hg.1	4.98	JUC14005825.hg.1	EGLN3
TC14001022.hg.1	4.17	PSR14011668.hg.1	EGLN3
TC14001022.hg.1	4.04	PSR14011669.hg.1	EGLN3

TC14001022.hg.1	2.48	PSR14011670.hg.1	EGLN3
TC14001022.hg.1	2.19	PSR14011660.hg.1	EGLN3
TC14001022.hg.1	2.06	PSR14011659.hg.1	EGLN3
TC14001022.hg.1	-2.06	PSR14011662.hg.1	EGLN3
TC14001022.hg.1	-2.3	JUC14005834.hg.1	EGLN3
TC14001022.hg.1	-2.66	PSR14011664.hg.1	EGLN3
TC14001022.hg.1	-2.79	PSR14011663.hg.1	EGLN3
TC14001022.hg.1	-3.38	PSR14011666.hg.1	EGLN3
TC14001022.hg.1	-4.28	JUC14005831.hg.1	EGLN3
TC14001022.hg.1	-9.09	JUC14005828.hg.1	EGLN3
TC11002084.hg.1	54.03	JUC11013212.hg.1	CHRDL2
TC11002084.hg.1	22.76	JUC11013201.hg.1	CHRDL2
TC11002084.hg.1	14.72	PSR11024992.hg.1	CHRDL2
TC11002084.hg.1	13.75	JUC11013199.hg.1	CHRDL2
TC11002084.hg.1	11.08	PSR11024998.hg.1	CHRDL2
TC11002084.hg.1	9.71	PSR11025005.hg.1	CHRDL2
TC11002084.hg.1	8.37	PSR11025000.hg.1	CHRDL2
TC11002084.hg.1	3.14	PSR11025011.hg.1	CHRDL2
TC11002084.hg.1	2.44	PSR11025006.hg.1	CHRDL2
TC11002084.hg.1	2.1	PSR11025007.hg.1	CHRDL2
TC11002084.hg.1	2.07	PSR11024993.hg.1	CHRDL2
TC11002084.hg.1	-2.2	PSR11024995.hg.1	CHRDL2
TC11002084.hg.1	-3.03	PSR11024991.hg.1	CHRDL2
TC11002084.hg.1	-4.04	PSR11024999.hg.1	CHRDL2
TC11002084.hg.1	-4.45	JUC11013202.hg.1	CHRDL2
TC11002084.hg.1	-4.49	JUC11013204.hg.1	CHRDL2
TC11002084.hg.1	-4.57	JUC11013200.hg.1	CHRDL2
TC11002084.hg.1	-4.61	JUC11013197.hg.1	CHRDL2
TC11002084.hg.1	-5.57	JUC11013210.hg.1	CHRDL2
TC11002084.hg.1	-7.27	JUC11013203.hg.1	CHRDL2
TC11002084.hg.1	-11.26	JUC11013205.hg.1	CHRDL2
TC09000388.hg.1	50.28	JUC09002263.hg.1	NTRK2
TC09000388.hg.1	46.64	PSR09004159.hg.1	NTRK2
TC09000388.hg.1	39.67	PSR09004128.hg.1	NTRK2
TC09000388.hg.1	35.42	JUC09002271.hg.1	NTRK2
TC09000388.hg.1	35.21	PSR09004158.hg.1	NTRK2
TC09000388.hg.1	33.98	PSR09004137.hg.1	NTRK2
TC09000388.hg.1	32.8	PSR09004130.hg.1	NTRK2
TC09000388.hg.1	29.95	PSR09004163.hg.1	NTRK2
TC09000388.hg.1	29	JUC09002274.hg.1	NTRK2
TC09000388.hg.1	28.05	PSR09004132.hg.1	NTRK2
TC09000388.hg.1	25.84	PSR09004131.hg.1	NTRK2
TC09000388.hg.1	25.25	PSR09004155.hg.1	NTRK2
TC09000388.hg.1	25.01	PSR09004161.hg.1	NTRK2
TC09000388.hg.1	23.49	PSR09004162.hg.1	NTRK2
TC09000388.hg.1	21.93	PSR09004156.hg.1	NTRK2
TC09000388.hg.1	21.31	JUC09002277.hg.1	NTRK2
TC09000388.hg.1	19.19	PSR09004154.hg.1	NTRK2
TC09000388.hg.1	17.47	JUC09002272.hg.1	NTRK2
TC09000388.hg.1	16.24	JUC09002266.hg.1	NTRK2
TC09000388.hg.1	12.79	PSR09004151.hg.1	NTRK2
TC09000388.hg.1	12.31	PSR09004146.hg.1	NTRK2
TC09000388.hg.1	9.63	JUC09002256.hg.1	NTRK2
TC09000388.hg.1	7.31	JUC09002257.hg.1	NTRK2
TC09000388.hg.1	7.07	PSR09004129.hg.1	NTRK2
TC09000388.hg.1	4.74	PSR09004135.hg.1	NTRK2
TC09000388.hg.1	3.99	JUC09002270.hg.1	NTRK2
TC09000388.hg.1	3.13	PSR09004147.hg.1	NTRK2

TC09000388.hg.1	2.39	PSR09004150.hg.1	NTRK2
TC09000388.hg.1	-2.07	PSR09004143.hg.1	NTRK2
TC09000388.hg.1	-2.1	JUC09002275.hg.1	NTRK2
TC09000388.hg.1	-2.18	PSR09004142.hg.1	NTRK2
TC09000388.hg.1	-4.4	JUC09002254.hg.1	NTRK2
TC09000388.hg.1	-4.41	JUC09002265.hg.1	NTRK2
TC09000388.hg.1	-5.22	PSR09004139.hg.1	NTRK2
TC09000388.hg.1	-7.79	JUC09002255.hg.1	NTRK2
TC09000388.hg.1	-9.32	JUC09002253.hg.1	NTRK2
TC05000394.hg.1	43.13	JUC05003038.hg.1	THBS4
TC05000394.hg.1	33.3	JUC05003027.hg.1	THBS4
TC05000394.hg.1	6.97	JUC05003021.hg.1	THBS4
TC05000394.hg.1	6.8	JUC05003012.hg.1	THBS4
TC05000394.hg.1	6.42	JUC05003011.hg.1	THBS4
TC05000394.hg.1	2.34	PSR05005595.hg.1	THBS4
TC05000394.hg.1	-2.08	JUC05003041.hg.1	THBS4
TC11002235.hg.1	41.07	PSR11026723.hg.1	MMP3
TC11002235.hg.1	32.66	JUC11014228.hg.1	MMP3
TC11002235.hg.1	31.17	PSR11026709.hg.1	MMP3
TC11002235.hg.1	25.63	JUC11014227.hg.1	MMP3
TC11002235.hg.1	21.17	JUC11014222.hg.1	MMP3
TC11002235.hg.1	16.21	PSR11026704.hg.1	MMP3
TC11002235.hg.1	13.49	PSR11026702.hg.1	MMP3
TC11002235.hg.1	4.53	JUC11014230.hg.1	MMP3
TC11002235.hg.1	2.42	PSR11026713.hg.1	MMP3
TC11002235.hg.1	-2.09	JUC11014225.hg.1	MMP3
TC11002235.hg.1	-2.24	JUC11014220.hg.1	MMP3
TC04001084.hg.1	41.02	JUC04007928.hg.1	PPARGC1A
TC04001084.hg.1	36.61	JUC04007926.hg.1	PPARGC1A
TC04001084.hg.1	22.3	JUC04007929.hg.1	PPARGC1A
TC04001084.hg.1	21.93	PSR04014895.hg.1	PPARGC1A
TC04001084.hg.1	21.34	JUC04007930.hg.1	PPARGC1A
TC04001084.hg.1	21.15	PSR04014913.hg.1	PPARGC1A
TC04001084.hg.1	18.79	JUC04007945.hg.1	PPARGC1A
TC04001084.hg.1	18.64	PSR04014887.hg.1	PPARGC1A
TC04001084.hg.1	18	PSR04014881.hg.1	PPARGC1A
TC04001084.hg.1	15.87	JUC04007939.hg.1	PPARGC1A
TC04001084.hg.1	15.3	JUC04007942.hg.1	PPARGC1A
TC04001084.hg.1	15.12	PSR04014896.hg.1	PPARGC1A
TC04001084.hg.1	14.09	PSR04014892.hg.1	PPARGC1A
TC04001084.hg.1	13.99	JUC04007943.hg.1	PPARGC1A
TC04001084.hg.1	12.95	PSR04014897.hg.1	PPARGC1A
TC04001084.hg.1	12.53	JUC04007937.hg.1	PPARGC1A
TC04001084.hg.1	12.44	PSR04014898.hg.1	PPARGC1A
TC04001084.hg.1	11.72	JUC04007927.hg.1	PPARGC1A
TC04001084.hg.1	11.61	PSR04014865.hg.1	PPARGC1A
TC04001084.hg.1	10.27	PSR04014875.hg.1	PPARGC1A
TC04001084.hg.1	8.54	PSR04014899.hg.1	PPARGC1A
TC04001084.hg.1	7.69	PSR04014879.hg.1	PPARGC1A
TC04001084.hg.1	5.72	PSR04014905.hg.1	PPARGC1A
TC04001084.hg.1	5.15	JUC04007924.hg.1	PPARGC1A
TC04001084.hg.1	4.99	PSR04014886.hg.1	PPARGC1A
TC04001084.hg.1	4.44	PSR04014873.hg.1	PPARGC1A
TC04001084.hg.1	3.4	PSR04014880.hg.1	PPARGC1A
TC04001084.hg.1	3.32	PSR04014904.hg.1	PPARGC1A
TC04001084.hg.1	3.07	JUC04007933.hg.1	PPARGC1A
TC04001084.hg.1	2.44	PSR04014911.hg.1	PPARGC1A
TC04001084.hg.1	2.34	PSR04014877.hg.1	PPARGC1A

TC04001084.hg.1	2	PSR04014882.hg.1	PPARGC1A
TC04001084.hg.1	-2.2	JUC04007922.hg.1	PPARGC1A
TC07000852.hg.1	40.31	PSR07013412.hg.1	CHRM2
TC07000852.hg.1	34.13	JUC07006418.hg.1	CHRM2
TC07000852.hg.1	32.62	PSR07013408.hg.1	CHRM2
TC07000852.hg.1	29.54	JUC07006416.hg.1	CHRM2
TC07000852.hg.1	26.19	PSR07013413.hg.1	CHRM2
TC07000852.hg.1	22.33	PSR07013415.hg.1	CHRM2
TC07000852.hg.1	15.28	PSR07013411.hg.1	CHRM2
TC07000852.hg.1	14.46	PSR07013414.hg.1	CHRM2
TC07000852.hg.1	10.42	JUC07006421.hg.1	CHRM2
TC07000852.hg.1	5.65	JUC07006423.hg.1	CHRM2
TC07000852.hg.1	5.54	JUC07006424.hg.1	CHRM2
TC07000852.hg.1	3.66	PSR07013405.hg.1	CHRM2
TC07000852.hg.1	3.31	PSR07013404.hg.1	CHRM2
TC07000852.hg.1	2.33	JUC07006422.hg.1	CHRM2
TC07000852.hg.1	-2.89	PSR07013417.hg.1	CHRM2
TC03001884.hg.1	34.24	JUC03016874.hg.1	CP
TC03001884.hg.1	33.66	JUC03016880.hg.1	CP
TC03001884.hg.1	29.99	JUC03016868.hg.1	CP
TC03001884.hg.1	20.8	PSR03033932.hg.1	CP
TC03001884.hg.1	20.49	PSR03033946.hg.1	CP
TC03001884.hg.1	12.84	JUC03016888.hg.1	CP
TC03001884.hg.1	9.86	JUC03016870.hg.1	CP
TC03001884.hg.1	8.84	PSR03033980.hg.1	CP
TC03001884.hg.1	8.78	PSR03033934.hg.1	CP
TC03001884.hg.1	8.56	PSR03033933.hg.1	CP
TC03001884.hg.1	8.02	JUC03016866.hg.1	CP
TC03001884.hg.1	7.11	PSR03033984.hg.1	CP
TC03001884.hg.1	5.6	PSR03033962.hg.1	CP
TC03001884.hg.1	5.56	PSR03033953.hg.1	CP
TC03001884.hg.1	5.46	PSR03033970.hg.1	CP
TC03001884.hg.1	5.1	JUC03016890.hg.1	CP
TC03001884.hg.1	4.98	PSR03033952.hg.1	CP
TC03001884.hg.1	2.83	JUC03016882.hg.1	CP
TC03001884.hg.1	2.49	JUC03016876.hg.1	CP
TC03001884.hg.1	2.2	JUC03016867.hg.1	CP
TC03001884.hg.1	2.16	PSR03033956.hg.1	CP
TC03001884.hg.1	2.14	PSR03033960.hg.1	CP
TC03001884.hg.1	2	JUC03016881.hg.1	CP
TC03001884.hg.1	-2.15	PSR03033964.hg.1	CP
TC03001884.hg.1	-2.35	PSR03033950.hg.1	CP
TC03001884.hg.1	-2.42	PSR03033966.hg.1	CP
TC03001884.hg.1	-2.45	PSR03033944.hg.1	CP
TC03001884.hg.1	-3.22	JUC03016884.hg.1	CP
TC07000261.hg.1	33.91	JUC07001943.hg.1	HECW1
TC07000261.hg.1	16.27	JUC07001934.hg.1	HECW1
TC07000261.hg.1	11.08	PSR07003973.hg.1	HECW1
TC07000261.hg.1	10.64	JUC07001924.hg.1	HECW1
TC07000261.hg.1	7.67	JUC07001923.hg.1	HECW1
TC07000261.hg.1	6.73	PSR07003974.hg.1	HECW1
TC07000261.hg.1	5.04	JUC07001929.hg.1	HECW1
TC07000261.hg.1	3.75	JUC07001937.hg.1	HECW1
TC07000261.hg.1	3.46	JUC07001914.hg.1	HECW1
TC07000261.hg.1	3.4	JUC07001935.hg.1	HECW1
TC07000261.hg.1	3.39	PSR07004013.hg.1	HECW1
TC07000261.hg.1	3.38	JUC07001911.hg.1	HECW1
TC07000261.hg.1	3.34	PSR07004022.hg.1	HECW1

TC07000261.hg.1	3.25	PSR07004025.hg.1	HECW1
TC07000261.hg.1	2.84	JUC07001947.hg.1	HECW1
TC07000261.hg.1	2.76	JUC07001927.hg.1	HECW1
TC07000261.hg.1	2.72	PSR07004008.hg.1	HECW1
TC07000261.hg.1	2.64	PSR07004010.hg.1	HECW1
TC07000261.hg.1	2.51	PSR07003972.hg.1	HECW1
TC07000261.hg.1	2.51	PSR07004017.hg.1	HECW1
TC07000261.hg.1	2.46	JUC07001932.hg.1	HECW1
TC07000261.hg.1	2.24	PSR07003987.hg.1	HECW1
TC07000261.hg.1	2.22	PSR07003969.hg.1	HECW1
TC07000261.hg.1	2.21	PSR07004005.hg.1	HECW1
TC07000261.hg.1	2.18	JUC07001930.hg.1	HECW1
TC07000261.hg.1	2.13	PSR07004003.hg.1	HECW1
TC07000261.hg.1	2.11	PSR07004001.hg.1	HECW1
TC07000261.hg.1	2.09	PSR07003999.hg.1	HECW1
TC07000261.hg.1	2.01	PSR07004014.hg.1	HECW1
TC07000261.hg.1	-2.11	JUC07001915.hg.1	HECW1
TC07000261.hg.1	-2.13	JUC07001913.hg.1	HECW1
TC07000261.hg.1	-2.25	PSR07003989.hg.1	HECW1
TC07000261.hg.1	-2.34	PSR07003979.hg.1	HECW1
TC07000261.hg.1	-2.43	PSR07004015.hg.1	HECW1
TC07000261.hg.1	-2.48	JUC07001928.hg.1	HECW1
TC07000261.hg.1	-2.52	JUC07001950.hg.1	HECW1
TC12001622.hg.1	32.88	PSR12021838.hg.1	NDUFA4L2
TC12001622.hg.1	29.74	JUC12012057.hg.1	NDUFA4L2
TC12001622.hg.1	21.87	PSR12021841.hg.1	NDUFA4L2
TC12001622.hg.1	18.72	PSR12021836.hg.1	NDUFA4L2
TC12001622.hg.1	5.75	JUC12012055.hg.1	NDUFA4L2
TC12001622.hg.1	5.64	PSR12021828.hg.1	NDUFA4L2
TC12001622.hg.1	3.55	PSR12021835.hg.1	NDUFA4L2
TC12001622.hg.1	2.66	PSR12021832.hg.1	NDUFA4L2
TC12001622.hg.1	2.37	PSR12021830.hg.1	NDUFA4L2
TC12001622.hg.1	-3.05	JUC12012058.hg.1	NDUFA4L2
TC07000815.hg.1	31.18	PSR07012856.hg.1	MEST
TC07000815.hg.1	25.53	PSR07012861.hg.1	MEST
TC07000815.hg.1	21.81	JUC07006103.hg.1	MEST
TC07000815.hg.1	21.6	PSR07012889.hg.1	MEST
TC07000815.hg.1	21.48	PSR07012862.hg.1	MEST
TC07000815.hg.1	20.82	PSR07012846.hg.1	MEST
TC07000815.hg.1	20.8	PSR07012857.hg.1	MEST
TC07000815.hg.1	20.28	PSR07012860.hg.1	MEST
TC07000815.hg.1	17.82	JUC07006105.hg.1	MEST
TC07000815.hg.1	17.31	JUC07006092.hg.1	MEST
TC07000815.hg.1	16.8	JUC07006110.hg.1	MEST
TC07000815.hg.1	16.45	JUC07006094.hg.1	MEST
TC07000815.hg.1	16	PSR07012863.hg.1	MEST
TC07000815.hg.1	15.73	JUC07006107.hg.1	MEST
TC07000815.hg.1	15.69	PSR07012883.hg.1	MEST
TC07000815.hg.1	15.62	PSR07012852.hg.1	MEST
TC07000815.hg.1	14.48	PSR07012892.hg.1	MEST
TC07000815.hg.1	14.44	PSR07012879.hg.1	MEST
TC07000815.hg.1	13.21	PSR07012847.hg.1	MEST
TC07000815.hg.1	11.23	JUC07006091.hg.1	MEST
TC07000815.hg.1	6.69	PSR07012875.hg.1	MEST
TC07000815.hg.1	5.36	JUC07006095.hg.1	MEST
TC07000815.hg.1	5.1	JUC07006101.hg.1	MEST
TC07000815.hg.1	4.48	PSR07012866.hg.1	MEST
TC07000815.hg.1	2.17	JUC07006102.hg.1	MEST

TC07000815.hg.1	2.02	PSR07012884.hg.1	MEST
TC07000815.hg.1	-2.01	PSR07012897.hg.1	MEST
TC07000815.hg.1	-2.03	PSR07012870.hg.1	MEST
TC07000815.hg.1	-2.43	JUC07006108.hg.1	MEST
TC07000815.hg.1	-2.52	JUC07006086.hg.1	MEST
TC07000815.hg.1	-3.94	JUC07006096.hg.1	MEST
TC18000164.hg.1	30.46	JUC18001343.hg.1	SLC14A1
TC18000164.hg.1	10.3	JUC18001344.hg.1	SLC14A1
TC18000164.hg.1	4.83	PSR18001904.hg.1	SLC14A1
TC18000164.hg.1	2.71	PSR18001908.hg.1	SLC14A1
TC18000164.hg.1	2.53	PSR18001909.hg.1	SLC14A1
TC09001533.hg.1	27.39	JUC09010372.hg.1	DBC1
TC09001533.hg.1	8.01	PSR09019143.hg.1	DBC1
TC09001533.hg.1	7.81	PSR09019127.hg.1	DBC1
TC09001533.hg.1	4.03	PSR09019129.hg.1	DBC1
TC09001533.hg.1	3.84	JUC09010375.hg.1	DBC1
TC09001533.hg.1	2.41	PSR09019142.hg.1	DBC1
TC09001533.hg.1	2.06	JUC09010373.hg.1	DBC1
TC09001533.hg.1	-3.29	JUC09010371.hg.1	DBC1
TC09001533.hg.1	-3.37	JUC09010377.hg.1	DBC1
TC13000576.hg.1	26.18	JUC13003856.hg.1	POSTN
TC13000576.hg.1	4.65	PSR13006602.hg.1	POSTN
TC13000576.hg.1	3.95	PSR13006607.hg.1	POSTN
TC13000576.hg.1	3.17	JUC13003844.hg.1	POSTN
TC13000576.hg.1	2.72	PSR13006629.hg.1	POSTN
TC13000576.hg.1	2.63	PSR13006600.hg.1	POSTN
TC13000576.hg.1	2.48	JUC13003855.hg.1	POSTN
TC13000576.hg.1	2.45	JUC13003846.hg.1	POSTN
TC13000576.hg.1	2.34	PSR13006595.hg.1	POSTN
TC13000576.hg.1	2.29	PSR13006594.hg.1	POSTN
TC13000576.hg.1	2.24	JUC13003850.hg.1	POSTN
TC13000576.hg.1	2.12	JUC13003862.hg.1	POSTN
TC13000576.hg.1	-2.02	PSR13006604.hg.1	POSTN
TC13000576.hg.1	-2.28	JUC13003842.hg.1	POSTN
TC13000576.hg.1	-2.53	PSR13006606.hg.1	POSTN
TC13000576.hg.1	-2.59	PSR13006598.hg.1	POSTN
TC13000576.hg.1	-7.66	PSR13006635.hg.1	POSTN
TC13000576.hg.1	-7.79	JUC13003859.hg.1	POSTN
TC13000576.hg.1	-8.44	PSR13006593.hg.1	POSTN
TC05001614.hg.1	25.85	JUC05011357.hg.1	PCSK1
TC05001614.hg.1	14.22	JUC05011367.hg.1	PCSK1
TC05001614.hg.1	12.63	PSR05022045.hg.1	PCSK1
TC05001614.hg.1	11.73	PSR05022020.hg.1	PCSK1
TC05001614.hg.1	11.38	PSR05022043.hg.1	PCSK1
TC05001614.hg.1	10.18	PSR05022042.hg.1	PCSK1
TC05001614.hg.1	9.62	PSR05022039.hg.1	PCSK1
TC05001614.hg.1	9.09	PSR05022032.hg.1	PCSK1
TC05001614.hg.1	8.16	PSR05022041.hg.1	PCSK1
TC05001614.hg.1	7.43	JUC05011365.hg.1	PCSK1
TC05001614.hg.1	6.91	PSR05022038.hg.1	PCSK1
TC05001614.hg.1	6.59	JUC05011355.hg.1	PCSK1
TC05001614.hg.1	6.19	PSR05022029.hg.1	PCSK1
TC05001614.hg.1	3.5	JUC05011361.hg.1	PCSK1
TC05001614.hg.1	2.92	JUC05011363.hg.1	PCSK1
TC05001614.hg.1	2.53	PSR05022036.hg.1	PCSK1
TC05001614.hg.1	2.48	PSR05022034.hg.1	PCSK1
TC05001614.hg.1	2.35	PSR05022030.hg.1	PCSK1
TC05001614.hg.1	2.35	JUC05011369.hg.1	PCSK1

TC05001614.hg.1	2.24	JUC05011358.hg.1	PCSK1
TC05001614.hg.1	-2.12	JUC05011354.hg.1	PCSK1
TC05001614.hg.1	-2.15	PSR05022027.hg.1	PCSK1
TC05001614.hg.1	-2.4	PSR05022026.hg.1	PCSK1
TC05001614.hg.1	-4.48	JUC05011353.hg.1	PCSK1
TC08001062.hg.1	24.46	JUC08006956.hg.1	STC1
TC08001062.hg.1	10.67	PSR08013517.hg.1	STC1
TC08001062.hg.1	8.09	JUC08006955.hg.1	STC1
TC08001062.hg.1	2.37	PSR08013512.hg.1	STC1
TC08001062.hg.1	-2.34	PSR08013516.hg.1	STC1
TC08001062.hg.1	-3	JUC08006958.hg.1	STC1
TC08001062.hg.1	-3	JUC08006959.hg.1	STC1
TC09000560.hg.1	23.99	JUC09003458.hg.1	UGCG
TC09000560.hg.1	13.37	PSR09006240.hg.1	UGCG
TC09000560.hg.1	11.64	PSR09006242.hg.1	UGCG
TC09000560.hg.1	10.72	PSR09006239.hg.1	UGCG
TC09000560.hg.1	9.14	PSR09006253.hg.1	UGCG
TC09000560.hg.1	3.16	JUC09003451.hg.1	UGCG
TC09000560.hg.1	2.27	PSR09006246.hg.1	UGCG
TC09000560.hg.1	2.22	PSR09006247.hg.1	UGCG
TC09000560.hg.1	-2.32	JUC09003455.hg.1	UGCG
TC02002747.hg.1	22.83	PSR02044128.hg.1	FN1
TC02002747.hg.1	21.37	PSR02044122.hg.1	FN1
TC02002747.hg.1	20.45	PSR02044190.hg.1	FN1
TC02002747.hg.1	20.3	PSR02044115.hg.1	FN1
TC02002747.hg.1	19.89	PSR02044168.hg.1	FN1
TC02002747.hg.1	19.24	PSR02044182.hg.1	FN1
TC02002747.hg.1	18.9	PSR02044123.hg.1	FN1
TC02002747.hg.1	18.38	PSR02044185.hg.1	FN1
TC02002747.hg.1	17.73	PSR02044151.hg.1	FN1
TC02002747.hg.1	17.56	PSR02044152.hg.1	FN1
TC02002747.hg.1	15.93	PSR02044165.hg.1	FN1
TC02002747.hg.1	15.79	PSR02044157.hg.1	FN1
TC02002747.hg.1	14.91	PSR02044164.hg.1	FN1
TC02002747.hg.1	14.18	PSR02044146.hg.1	FN1
TC02002747.hg.1	13.29	PSR02044148.hg.1	FN1
TC02002747.hg.1	12.86	PSR02044150.hg.1	FN1
TC02002747.hg.1	12.81	PSR02044117.hg.1	FN1
TC02002747.hg.1	12.24	PSR02044149.hg.1	FN1
TC02002747.hg.1	11.27	PSR02044134.hg.1	FN1
TC02002747.hg.1	11.01	PSR02044129.hg.1	FN1
TC02002747.hg.1	10.01	PSR02044116.hg.1	FN1
TC02002747.hg.1	8.91	PSR02044135.hg.1	FN1
TC02002747.hg.1	5.59	PSR02044121.hg.1	FN1
TC02002747.hg.1	4.32	JUC02023158.hg.1	FN1
TC02002747.hg.1	4.31	PSR02044108.hg.1	FN1
TC02002747.hg.1	3.16	PSR02044126.hg.1	FN1
TC02002747.hg.1	2.69	JUC02023167.hg.1	FN1
TC02002747.hg.1	2.64	JUC02023178.hg.1	FN1
TC02002747.hg.1	2.31	JUC02023171.hg.1	FN1
TC02002747.hg.1	2.22	JUC02023201.hg.1	FN1
TC02002747.hg.1	2.21	PSR02044127.hg.1	FN1
TC02002747.hg.1	2.2	PSR02044200.hg.1	FN1
TC02002747.hg.1	2.04	PSR02044131.hg.1	FN1
TC6_mcf_hap5000	22.29	PSR6_mcf_hap5000	HLA-A
TC6_mcf_hap5000	5.09	PSR6_mcf_hap5000	HLA-A
TC6_mcf_hap5000	3.33	PSR6_mcf_hap5000	HLA-A
TC6_mcf_hap5000	2.72	PSR6_mcf_hap5000	HLA-A

TC6_mcf_hap5000	2.71	JUC6_mcf_hap50017	HLA-A
TC6_mcf_hap5000	2.21	PSR6_mcf_hap5000	HLA-A
TC6_mcf_hap5000	2.14	JUC6_mcf_hap50017	HLA-A
TC6_mcf_hap5000	-4.21	JUC6_mcf_hap50017	HLA-A
TC01000733.hg.1	21.68	PSR01011722.hg.1	PDE4B
TC01000733.hg.1	16.45	JUC01006093.hg.1	PDE4B
TC01000733.hg.1	16.34	PSR01011733.hg.1	PDE4B
TC01000733.hg.1	16.1	PSR01011723.hg.1	PDE4B
TC01000733.hg.1	15.77	JUC01006078.hg.1	PDE4B
TC01000733.hg.1	13.55	PSR01011734.hg.1	PDE4B
TC01000733.hg.1	12.27	PSR01011727.hg.1	PDE4B
TC01000733.hg.1	10.51	PSR01011735.hg.1	PDE4B
TC01000733.hg.1	10.28	PSR01011726.hg.1	PDE4B
TC01000733.hg.1	10.11	JUC01006077.hg.1	PDE4B
TC01000733.hg.1	9.64	JUC01006080.hg.1	PDE4B
TC01000733.hg.1	9.37	JUC01006088.hg.1	PDE4B
TC01000733.hg.1	9.26	JUC01006075.hg.1	PDE4B
TC01000733.hg.1	9.21	PSR01011724.hg.1	PDE4B
TC01000733.hg.1	8.83	JUC01006079.hg.1	PDE4B
TC01000733.hg.1	8.81	PSR01011725.hg.1	PDE4B
TC01000733.hg.1	8.38	PSR01011736.hg.1	PDE4B
TC01000733.hg.1	7.84	PSR01011721.hg.1	PDE4B
TC01000733.hg.1	7.04	PSR01011737.hg.1	PDE4B
TC01000733.hg.1	5.58	PSR01011743.hg.1	PDE4B
TC01000733.hg.1	4.75	PSR01011757.hg.1	PDE4B
TC01000733.hg.1	4.15	PSR01011744.hg.1	PDE4B
TC01000733.hg.1	3.8	PSR01011761.hg.1	PDE4B
TC01000733.hg.1	-2.13	PSR01011746.hg.1	PDE4B
TC01000733.hg.1	-2.27	PSR01011741.hg.1	PDE4B
TC01000733.hg.1	-2.39	PSR01011739.hg.1	PDE4B
TC01000733.hg.1	-2.41	JUC01006092.hg.1	PDE4B
TC01000733.hg.1	-2.56	JUC01006082.hg.1	PDE4B
TC01000733.hg.1	-2.74	JUC01006083.hg.1	PDE4B
TC01000733.hg.1	-2.75	JUC01006097.hg.1	PDE4B
TC01000733.hg.1	-3.19	JUC01006091.hg.1	PDE4B
TC03001459.hg.1	21.46	JUC03012921.hg.1	GLT8D1
TC03001459.hg.1	2.05	PSR03026478.hg.1	GLT8D1
TC6_dbb_hap3000	21.22	PSR6_dbb_hap3000	HLA-A
TC6_dbb_hap3000	4.84	PSR6_dbb_hap3000	HLA-A
TC6_dbb_hap3000	3.17	PSR6_dbb_hap3000	HLA-A
TC6_dbb_hap3000	2.59	PSR6_dbb_hap3000	HLA-A
TC6_dbb_hap3000	2.58	JUC6_dbb_hap3001	HLA-A
TC6_dbb_hap3000	2.33	PSR6_dbb_hap3000	HLA-A
TC6_dbb_hap3000	2.1	PSR6_dbb_hap3000	HLA-A
TC6_dbb_hap3000	2.06	PSR6_dbb_hap3000	HLA-A
TC6_dbb_hap3000	2.04	JUC6_dbb_hap3001	HLA-A
TC6_dbb_hap3000	-4.42	JUC6_dbb_hap3001	HLA-A
TC13000563.hg.1	20.81	JUC13003717.hg.1	DCLK1
TC13000563.hg.1	11.33	PSR13006338.hg.1	DCLK1
TC13000563.hg.1	6.29	PSR13006349.hg.1	DCLK1
TC13000563.hg.1	4.07	JUC13003732.hg.1	DCLK1
TC13000563.hg.1	3.78	PSR13006350.hg.1	DCLK1
TC13000563.hg.1	3.38	JUC13003735.hg.1	DCLK1
TC13000563.hg.1	2.25	JUC13003741.hg.1	DCLK1
TC13000563.hg.1	2.14	JUC13003738.hg.1	DCLK1
TC13000563.hg.1	2.11	PSR13006341.hg.1	DCLK1
TC13000563.hg.1	2.08	PSR13006342.hg.1	DCLK1
TC13000563.hg.1	2.02	PSR13006348.hg.1	DCLK1

TC0Y000163.hg.1	20.73	JUC0Y001069.hg.1	UTY
TC0Y000163.hg.1	11.86	JUC0Y000994.hg.1	UTY
TC0Y000163.hg.1	6.08	PSR0Y001638.hg.1	UTY
TC0Y000163.hg.1	5.71	PSR0Y001641.hg.1	UTY
TC0Y000163.hg.1	5.62	PSR0Y001637.hg.1	UTY
TC0Y000163.hg.1	4.14	JUC0Y001001.hg.1	UTY
TC0Y000163.hg.1	3.95	PSR0Y001645.hg.1	UTY
TC0Y000163.hg.1	3.55	PSR0Y001647.hg.1	UTY
TC0Y000163.hg.1	3.53	JUC0Y001064.hg.1	UTY
TC0Y000163.hg.1	2.6	PSR0Y001635.hg.1	UTY
TC0Y000163.hg.1	2.21	JUC0Y001041.hg.1	UTY
TC0Y000163.hg.1	-2.01	JUC0Y001059.hg.1	UTY
TC0Y000163.hg.1	-2.04	PSR0Y001636.hg.1	UTY
TC0Y000163.hg.1	-2.16	PSR0Y001634.hg.1	UTY
TC0Y000163.hg.1	-2.19	JUC0Y001013.hg.1	UTY
TC0Y000163.hg.1	-2.25	JUC0Y001009.hg.1	UTY
TC0Y000163.hg.1	-2.26	PSR0Y001624.hg.1	UTY
TC0Y000163.hg.1	-2.29	JUC0Y000999.hg.1	UTY
TC0Y000163.hg.1	-2.31	PSR0Y001621.hg.1	UTY
TC0Y000163.hg.1	-2.34	PSR0Y001632.hg.1	UTY
TC0Y000163.hg.1	-2.4	PSR0Y001648.hg.1	UTY
TC0Y000163.hg.1	-2.46	PSR0Y001674.hg.1	UTY
TC0Y000163.hg.1	-2.46	JUC0Y001050.hg.1	UTY
TC0Y000163.hg.1	-2.47	PSR0Y001626.hg.1	UTY
TC0Y000163.hg.1	-2.48	PSR0Y001666.hg.1	UTY
TC0Y000163.hg.1	-2.51	PSR0Y001669.hg.1	UTY
TC0Y000163.hg.1	-2.68	PSR0Y001616.hg.1	UTY
TC0Y000163.hg.1	-2.78	PSR0Y001682.hg.1	UTY
TC0Y000163.hg.1	-2.86	PSR0Y001670.hg.1	UTY
TC0Y000163.hg.1	-2.9	PSR0Y001617.hg.1	UTY
TC0Y000163.hg.1	-2.96	JUC0Y001088.hg.1	UTY
TC0Y000163.hg.1	-2.97	PSR0Y001619.hg.1	UTY
TC0Y000163.hg.1	-2.99	JUC0Y001023.hg.1	UTY
TC0Y000163.hg.1	-3.04	JUC0Y001063.hg.1	UTY
TC0Y000163.hg.1	-3.15	JUC0Y001081.hg.1	UTY
TC0Y000163.hg.1	-3.38	PSR0Y001650.hg.1	UTY
TC0Y000163.hg.1	-3.39	PSR0Y001685.hg.1	UTY
TC0Y000163.hg.1	-3.41	PSR0Y001679.hg.1	UTY
TC0Y000163.hg.1	-3.45	JUC0Y001040.hg.1	UTY
TC0Y000163.hg.1	-3.47	PSR0Y001676.hg.1	UTY
TC0Y000163.hg.1	-3.59	PSR0Y001663.hg.1	UTY
TC0Y000163.hg.1	-3.71	PSR0Y001654.hg.1	UTY
TC0Y000163.hg.1	-3.71	PSR0Y001665.hg.1	UTY
TC0Y000163.hg.1	-3.74	PSR0Y001620.hg.1	UTY
TC0Y000163.hg.1	-3.78	JUC0Y001042.hg.1	UTY
TC0Y000163.hg.1	-3.87	PSR0Y001656.hg.1	UTY
TC0Y000163.hg.1	-3.88	JUC0Y001077.hg.1	UTY
TC0Y000163.hg.1	-3.93	PSR0Y001678.hg.1	UTY
TC0Y000163.hg.1	-3.93	PSR0Y001681.hg.1	UTY
TC0Y000163.hg.1	-4	JUC0Y001016.hg.1	UTY
TC0Y000163.hg.1	-4.03	PSR0Y001671.hg.1	UTY
TC0Y000163.hg.1	-4.26	JUC0Y001047.hg.1	UTY
TC0Y000163.hg.1	-4.36	PSR0Y001668.hg.1	UTY
TC0Y000163.hg.1	-4.43	JUC0Y000990.hg.1	UTY
TC0Y000163.hg.1	-4.59	JUC0Y001027.hg.1	UTY
TC0Y000163.hg.1	-5.03	JUC0Y001038.hg.1	UTY
TC0Y000163.hg.1	-5.19	JUC0Y001039.hg.1	UTY
TC0Y000163.hg.1	-5.21	JUC0Y001035.hg.1	UTY

TC0Y000163.hg.1	-5.41	JUC0Y001074.hg.1	UTY
TC0Y000163.hg.1	-5.55	PSR0Y001653.hg.1	UTY
TC0Y000163.hg.1	-5.56	PSR0Y001680.hg.1	UTY
TC0Y000163.hg.1	-6.22	PSR0Y001630.hg.1	UTY
TC0Y000163.hg.1	-6.22	JUC0Y001036.hg.1	UTY
TC0Y000163.hg.1	-6.32	PSR0Y001675.hg.1	UTY
TC0Y000163.hg.1	-6.34	JUC0Y000998.hg.1	UTY
TC0Y000163.hg.1	-6.42	PSR0Y001657.hg.1	UTY
TC0Y000163.hg.1	-6.43	JUC0Y001054.hg.1	UTY
TC0Y000163.hg.1	-6.5	PSR0Y001659.hg.1	UTY
TC0Y000163.hg.1	-6.9	PSR0Y001633.hg.1	UTY
TC0Y000163.hg.1	-7.05	PSR0Y001677.hg.1	UTY
TC0Y000163.hg.1	-7.3	JUC0Y001072.hg.1	UTY
TC0Y000163.hg.1	-7.33	JUC0Y001003.hg.1	UTY
TC0Y000163.hg.1	-7.79	JUC0Y001037.hg.1	UTY
TC0Y000163.hg.1	-8.4	JUC0Y001026.hg.1	UTY
TC0Y000163.hg.1	-8.56	JUC0Y001031.hg.1	UTY
TC0Y000163.hg.1	-8.85	JUC0Y001057.hg.1	UTY
TC0Y000163.hg.1	-9.01	JUC0Y001052.hg.1	UTY
TC0Y000163.hg.1	-9.15	JUC0Y001067.hg.1	UTY
TC0Y000163.hg.1	-9.53	JUC0Y001049.hg.1	UTY
TC0Y000163.hg.1	-10.76	JUC0Y001062.hg.1	UTY
TC0Y000163.hg.1	-11.68	JUC0Y000996.hg.1	UTY
TC0Y000163.hg.1	-13.93	JUC0Y000995.hg.1	UTY
TC08000531.hg.1	20.49	PSR08007210.hg.1	CA2
TC08000531.hg.1	19.71	PSR08007200.hg.1	CA2
TC08000531.hg.1	16.18	PSR08007208.hg.1	CA2
TC08000531.hg.1	13.45	JUC08003565.hg.1	CA2
TC08000531.hg.1	9.6	JUC08003560.hg.1	CA2
TC08000531.hg.1	7.8	PSR08007204.hg.1	CA2
TC08000531.hg.1	7.52	PSR08007218.hg.1	CA2
TC08000531.hg.1	5.98	JUC08003562.hg.1	CA2
TC08000531.hg.1	2.43	PSR08007205.hg.1	CA2
TC08000531.hg.1	2.04	JUC08003561.hg.1	CA2
TC08000531.hg.1	-3.14	JUC08003564.hg.1	CA2
TC08000531.hg.1	-4.25	PSR08007212.hg.1	CA2
TC03002106.hg.1	20.26	PSR03037284.hg.1	LEPREL1
TC03002106.hg.1	15.77	PSR03037252.hg.1	LEPREL1
TC03002106.hg.1	14.36	PSR03037273.hg.1	LEPREL1
TC03002106.hg.1	13.92	PSR03037285.hg.1	LEPREL1
TC03002106.hg.1	12.88	PSR03037266.hg.1	LEPREL1
TC03002106.hg.1	9.33	PSR03037282.hg.1	LEPREL1
TC03002106.hg.1	5.52	PSR03037283.hg.1	LEPREL1
TC03002106.hg.1	5.47	PSR03037277.hg.1	LEPREL1
TC03002106.hg.1	4.61	JUC03018433.hg.1	LEPREL1
TC03002106.hg.1	4.56	PSR03037268.hg.1	LEPREL1
TC03002106.hg.1	3.97	PSR03037262.hg.1	LEPREL1
TC03002106.hg.1	3.84	JUC03018423.hg.1	LEPREL1
TC03002106.hg.1	-2.12	JUC03018420.hg.1	LEPREL1
TC03002106.hg.1	-2.53	JUC03018434.hg.1	LEPREL1
TC10000053.hg.1	20.02	JUC10000311.hg.1	PFKFB3
TC10000053.hg.1	8.92	JUC10000316.hg.1	PFKFB3
TC10000053.hg.1	7.95	PSR10000608.hg.1	PFKFB3
TC10000053.hg.1	7.94	PSR10000620.hg.1	PFKFB3
TC10000053.hg.1	6.68	PSR10000634.hg.1	PFKFB3
TC10000053.hg.1	6.29	PSR10000601.hg.1	PFKFB3
TC10000053.hg.1	5.77	PSR10000631.hg.1	PFKFB3
TC10000053.hg.1	5.63	PSR10000626.hg.1	PFKFB3

TC10000053.hg.1	5.45	JUC10000335.hg.1	PFKFB3
TC10000053.hg.1	5.31	JUC10000324.hg.1	PFKFB3
TC10000053.hg.1	4.97	PSR10000611.hg.1	PFKFB3
TC10000053.hg.1	4.11	JUC10000309.hg.1	PFKFB3
TC10000053.hg.1	3.9	JUC10000307.hg.1	PFKFB3
TC10000053.hg.1	2.97	JUC10000323.hg.1	PFKFB3
TC10000053.hg.1	2.77	JUC10000325.hg.1	PFKFB3
TC10000053.hg.1	2.44	PSR10000639.hg.1	PFKFB3
TC10000053.hg.1	2.03	PSR10000638.hg.1	PFKFB3
TC10000053.hg.1	2.02	JUC10000320.hg.1	PFKFB3
TC10000053.hg.1	-2.03	JUC10000330.hg.1	PFKFB3
TC10000053.hg.1	-3.6	JUC10000304.hg.1	PFKFB3
TC10000053.hg.1	-4.09	JUC10000319.hg.1	PFKFB3
TC10002924.hg.1	19.88	JUC10016959.hg.1	AKR1C1
TC10002924.hg.1	13.55	PSR10000320.hg.1	AKR1C1
TC10002924.hg.1	13.09	PSR10000319.hg.1	AKR1C1
TC10002924.hg.1	12.34	PSR10000321.hg.1	AKR1C1
TC10002924.hg.1	11.92	PSR10000318.hg.1	AKR1C1
TC10002924.hg.1	9.98	JUC10016964.hg.1	AKR1C1
TC10002924.hg.1	6.25	JUC10016957.hg.1	AKR1C1
TC10002924.hg.1	5.46	JUC10016970.hg.1	AKR1C1
TC10002924.hg.1	4.64	PSR10000324.hg.1	AKR1C1
TC10002924.hg.1	4.47	PSR10000329.hg.1	AKR1C1
TC10002924.hg.1	3.72	PSR10000334.hg.1	AKR1C1
TC10002924.hg.1	3.5	PSR10000335.hg.1	AKR1C1
TC10002924.hg.1	3.38	PSR10000346.hg.1	AKR1C1
TC10002924.hg.1	3.36	PSR10000338.hg.1	AKR1C1
TC10002924.hg.1	2.75	JUC10016969.hg.1	AKR1C1
TC10002924.hg.1	2.55	PSR10000328.hg.1	AKR1C1
TC10002924.hg.1	2.11	PSR10000322.hg.1	AKR1C1
TC10002924.hg.1	-2.28	PSR10000332.hg.1	AKR1C1
TC08002613.hg.1	19.78	JUC08015206.hg.1	LOXL2
TC08002613.hg.1	10.35	JUC08015204.hg.1	LOXL2
TC08002613.hg.1	6.8	JUC08015186.hg.1	LOXL2
TC08002613.hg.1	5.5	JUC08015200.hg.1	LOXL2
TC08002613.hg.1	4.85	JUC08015208.hg.1	LOXL2
TC08002613.hg.1	4.73	PSR08013437.hg.1	LOXL2
TC08002613.hg.1	4.71	JUC08015202.hg.1	LOXL2
TC08002613.hg.1	4.2	PSR08013449.hg.1	LOXL2
TC08002613.hg.1	3.35	PSR08013420.hg.1	LOXL2
TC08002613.hg.1	3.23	PSR08013422.hg.1	LOXL2
TC08002613.hg.1	2.83	PSR08013401.hg.1	LOXL2
TC08002613.hg.1	2.54	JUC08015201.hg.1	LOXL2
TC08002613.hg.1	-2.02	PSR08013435.hg.1	LOXL2
TC08002613.hg.1	-2.08	JUC08015191.hg.1	LOXL2
TC08002613.hg.1	-4.33	PSR08013421.hg.1	LOXL2
TC01001427.hg.1	19.49	JUC01011775.hg.1	RGS4
TC01001427.hg.1	9.83	JUC01011772.hg.1	RGS4
TC01001427.hg.1	4.27	PSR01022322.hg.1	RGS4
TC01001427.hg.1	3.65	PSR01022315.hg.1	RGS4
TC01001427.hg.1	3.51	JUC01011765.hg.1	RGS4
TC01001427.hg.1	2.82	PSR01022320.hg.1	RGS4
TC01001427.hg.1	2.62	PSR01022325.hg.1	RGS4
TC01001427.hg.1	2.59	PSR01022323.hg.1	RGS4
TC01001427.hg.1	2.39	PSR01022321.hg.1	RGS4
TC01001427.hg.1	2.1	PSR01022328.hg.1	RGS4
TC01001427.hg.1	-2.14	PSR01022339.hg.1	RGS4
TC01001427.hg.1	-2.34	JUC01011764.hg.1	RGS4

TC03001370.hg.1	19.17	PSR03023647.hg.1	SMARCC1
TC03001370.hg.1	-3.68	JUC03011797.hg.1	SMARCC1
TC20000833.hg.1	18.83	JUC20005932.hg.1	TGM2
TC20000833.hg.1	15.75	JUC20005940.hg.1	TGM2
TC20000833.hg.1	14.11	PSR20011484.hg.1	TGM2
TC20000833.hg.1	13.14	PSR20011483.hg.1	TGM2
TC20000833.hg.1	10.59	PSR20011481.hg.1	TGM2
TC20000833.hg.1	9.41	PSR20011476.hg.1	TGM2
TC20000833.hg.1	7.4	PSR20011465.hg.1	TGM2
TC20000833.hg.1	7.31	JUC20005942.hg.1	TGM2
TC20000833.hg.1	6.88	JUC20005935.hg.1	TGM2
TC20000833.hg.1	5.29	PSR20011459.hg.1	TGM2
TC20000833.hg.1	2.85	JUC20005931.hg.1	TGM2
TC20000833.hg.1	2.22	JUC20005924.hg.1	TGM2
TC20000833.hg.1	2	JUC20005941.hg.1	TGM2
TC20000833.hg.1	-2.25	JUC20005927.hg.1	TGM2
TC20000833.hg.1	-2.49	JUC20005930.hg.1	TGM2
TC20000833.hg.1	-4.16	JUC20005925.hg.1	TGM2
TC06000608.hg.1	18.71	JUC06003213.hg.1	VEGFA
TC06000608.hg.1	13.95	PSR06007279.hg.1	VEGFA
TC06000608.hg.1	11.74	PSR06007282.hg.1	VEGFA
TC06000608.hg.1	8.82	PSR06007285.hg.1	VEGFA
TC06000608.hg.1	8.1	PSR06007295.hg.1	VEGFA
TC06000608.hg.1	7.79	PSR06007309.hg.1	VEGFA
TC06000608.hg.1	7.21	PSR06007290.hg.1	VEGFA
TC06000608.hg.1	7.1	PSR06007286.hg.1	VEGFA
TC06000608.hg.1	6.18	JUC06003202.hg.1	VEGFA
TC06000608.hg.1	4.24	JUC06003203.hg.1	VEGFA
TC06000608.hg.1	4.21	PSR06007297.hg.1	VEGFA
TC06000608.hg.1	2.87	PSR06007293.hg.1	VEGFA
TC06000608.hg.1	2.25	JUC06003210.hg.1	VEGFA
TC06000608.hg.1	-2.05	PSR06007307.hg.1	VEGFA
TC06000608.hg.1	-2.23	JUC06003201.hg.1	VEGFA
TC06000608.hg.1	-2.29	PSR06007313.hg.1	VEGFA
TC06000608.hg.1	-2.42	JUC06003198.hg.1	VEGFA
TC06000608.hg.1	-4.32	PSR06007303.hg.1	VEGFA
TC06000608.hg.1	-5.09	JUC06003199.hg.1	VEGFA
TC19002613.hg.1	18.26	PSR19021613.hg.1	PSG4
TC19002613.hg.1	16.16	JUC19017113.hg.1	PSG4
TC19002613.hg.1	15.07	PSR19021609.hg.1	PSG4
TC19002613.hg.1	14.53	PSR19021618.hg.1	PSG4
TC19002613.hg.1	12.41	JUC19017116.hg.1	PSG4
TC19002613.hg.1	11.13	PSR19021607.hg.1	PSG4
TC19002613.hg.1	10.38	PSR19021614.hg.1	PSG4
TC19002613.hg.1	6.86	PSR19021615.hg.1	PSG4
TC19002613.hg.1	5.66	JUC19017120.hg.1	PSG4
TC19002613.hg.1	4.76	JUC19017119.hg.1	PSG4
TC19002613.hg.1	2.82	JUC19017115.hg.1	PSG4
TC19002613.hg.1	2.42	PSR19021610.hg.1	PSG4
TC09000253.hg.1	18.12	JUC09001493.hg.1	CNTNAP3B, [illegible]
TC09000253.hg.1	4.33	JUC09001475.hg.1	CNTNAP3B, [illegible]
TC09000253.hg.1	3.9	PSR09002824.hg.1	CNTNAP3B, [illegible]
TC09000253.hg.1	3.81	JUC09001471.hg.1	CNTNAP3B, [illegible]
TC09000253.hg.1	3.73	PSR09002825.hg.1	CNTNAP3B, [illegible]
TC09000253.hg.1	3.6	PSR09002867.hg.1	CNTNAP3B, [illegible]
TC09000253.hg.1	3.56	PSR09002842.hg.1	CNTNAP3B, [illegible]
TC09000253.hg.1	3.24	PSR09002837.hg.1	CNTNAP3B, [illegible]
TC09000253.hg.1	3.17	JUC09001498.hg.1	CNTNAP3B, [illegible]

TC09000253.hg.1	2.99	JUC09001500.hg.1	CNTNAP3B, (
TC09000253.hg.1	2.5	PSR09002853.hg.1	CNTNAP3B, (
TC09000253.hg.1	2.4	PSR09002852.hg.1	CNTNAP3B, (
TC09000253.hg.1	2.36	PSR09002855.hg.1	CNTNAP3B, (
TC09000253.hg.1	2.31	PSR09002828.hg.1	CNTNAP3B, (
TC09000253.hg.1	2.27	JUC09001476.hg.1	CNTNAP3B, (
TC09000253.hg.1	2.24	JUC09001483.hg.1	CNTNAP3B, (
TC09000253.hg.1	2.18	PSR09002850.hg.1	CNTNAP3B, (
TC09000253.hg.1	2.17	PSR09002862.hg.1	CNTNAP3B, (
TC09000253.hg.1	2.14	PSR09002868.hg.1	CNTNAP3B, (
TC09000253.hg.1	2.1	PSR09002840.hg.1	CNTNAP3B, (
TC09000253.hg.1	2.01	PSR09002826.hg.1	CNTNAP3B, (
TC09000253.hg.1	2	PSR09002857.hg.1	CNTNAP3B, (
TC09000253.hg.1	-2.21	PSR09002831.hg.1	CNTNAP3B, (
TC09000253.hg.1	-2.31	JUC09001482.hg.1	CNTNAP3B, (
TC09000253.hg.1	-2.68	PSR09002833.hg.1	CNTNAP3B, (
TC09000253.hg.1	-3.65	JUC09001499.hg.1	CNTNAP3B, (
TC09000253.hg.1	-3.95	JUC09001484.hg.1	CNTNAP3B, (
TC6_qbl_hap60002	17.91	PSR6_qbl_hap60002	HLA-A
TC6_qbl_hap60002	3.76	PSR6_qbl_hap60002	HLA-A
TC6_qbl_hap60002	2.72	PSR6_qbl_hap60002	HLA-A
TC6_qbl_hap60002	2.23	JUC6_qbl_hap60018	HLA-A
TC6_qbl_hap60002	-2.05	PSR6_qbl_hap60002	HLA-A
TC6_qbl_hap60002	-2.61	JUC6_qbl_hap60018	HLA-A
TC6_qbl_hap60002	-5.13	JUC6_qbl_hap60018	HLA-A
TC05001900.hg.1	17.6	JUC05013360.hg.1	LARS
TC05001900.hg.1	-2	JUC05013374.hg.1	LARS
TC05001900.hg.1	-2.04	PSR05026207.hg.1	LARS
TC05001900.hg.1	-2.29	JUC05013399.hg.1	LARS
TC05001900.hg.1	-2.46	JUC05013366.hg.1	LARS
TC05001900.hg.1	-3.9	PSR05026192.hg.1	LARS
TC01006319.hg.1	17.48	PSR01007334.hg.1	SH3D21
TC01006319.hg.1	3.98	PSR01007333.hg.1	SH3D21
TC01006319.hg.1	3.77	PSR01007304.hg.1	SH3D21
TC01006319.hg.1	3.49	JUC01043939.hg.1	SH3D21
TC01006319.hg.1	2.88	PSR01007303.hg.1	SH3D21
TC01006319.hg.1	2.78	JUC01043945.hg.1	SH3D21
TC01006319.hg.1	2.54	JUC01043934.hg.1	SH3D21
TC01006319.hg.1	2.44	PSR01007328.hg.1	SH3D21
TC01006319.hg.1	2.24	PSR01007326.hg.1	SH3D21
TC01006319.hg.1	2.21	PSR01007330.hg.1	SH3D21
TC01006319.hg.1	2.09	PSR01007329.hg.1	SH3D21
TC01006319.hg.1	-2.8	JUC01043937.hg.1	SH3D21
TC01006319.hg.1	-2.9	PSR01007315.hg.1	SH3D21
TC01006319.hg.1	-3.06	JUC01043930.hg.1	SH3D21
TC10001030.hg.1	17.3	PSR10012789.hg.1	SFTA1P
TC10001030.hg.1	4.47	PSR10012796.hg.1	SFTA1P
TC10001030.hg.1	3.17	PSR10012792.hg.1	SFTA1P
TC10001030.hg.1	2.22	JUC10007406.hg.1	SFTA1P
TC10001030.hg.1	-3.17	JUC10007402.hg.1	SFTA1P
TC10001030.hg.1	-3.95	PSR10012794.hg.1	SFTA1P
TC12000406.hg.1	17.2	JUC12002822.hg.1	SLC4A8
TC12000406.hg.1	5.5	JUC12002820.hg.1	SLC4A8
TC12000406.hg.1	4.45	JUC12002829.hg.1	SLC4A8
TC12000406.hg.1	3.5	PSR12005113.hg.1	SLC4A8
TC12000406.hg.1	3.3	PSR12005107.hg.1	SLC4A8
TC12000406.hg.1	2.47	PSR12005105.hg.1	SLC4A8
TC12000406.hg.1	2.42	PSR12005109.hg.1	SLC4A8

TC12000406.hg.1	2.37	PSR12005104.hg.1	SLC4A8
TC12000406.hg.1	2.36	PSR12005108.hg.1	SLC4A8
TC12000406.hg.1	2.34	JUC12002837.hg.1	SLC4A8
TC12000406.hg.1	2.31	PSR12005117.hg.1	SLC4A8
TC12000406.hg.1	2.3	PSR12005138.hg.1	SLC4A8
TC12000406.hg.1	2.26	PSR12005114.hg.1	SLC4A8
TC12000406.hg.1	2.23	PSR12005100.hg.1	SLC4A8
TC12000406.hg.1	2.13	PSR12005101.hg.1	SLC4A8
TC12000406.hg.1	2.13	PSR12005110.hg.1	SLC4A8
TC12000406.hg.1	2.09	PSR12005102.hg.1	SLC4A8
TC12000406.hg.1	-2.06	JUC12002835.hg.1	SLC4A8
TC12000406.hg.1	-2.45	JUC12002826.hg.1	SLC4A8
TC12000406.hg.1	-4.24	JUC12002811.hg.1	SLC4A8
TC01001710.hg.1	16.41	JUC01014286.hg.1	CDK18
TC01001710.hg.1	15.53	JUC01014300.hg.1	CDK18
TC01001710.hg.1	13.94	JUC01014291.hg.1	CDK18
TC01001710.hg.1	12.2	JUC01014289.hg.1	CDK18
TC01001710.hg.1	12.17	JUC01014310.hg.1	CDK18
TC01001710.hg.1	11.93	PSR01026626.hg.1	CDK18
TC01001710.hg.1	9.7	PSR01026639.hg.1	CDK18
TC01001710.hg.1	9.58	JUC01014307.hg.1	CDK18
TC01001710.hg.1	9.08	PSR01026631.hg.1	CDK18
TC01001710.hg.1	8.55	PSR01026666.hg.1	CDK18
TC01001710.hg.1	8.18	PSR01026655.hg.1	CDK18
TC01001710.hg.1	7.91	PSR01026632.hg.1	CDK18
TC01001710.hg.1	7.83	PSR01026680.hg.1	CDK18
TC01001710.hg.1	7.46	PSR01026685.hg.1	CDK18
TC01001710.hg.1	6.81	PSR01026677.hg.1	CDK18
TC01001710.hg.1	6.61	PSR01026654.hg.1	CDK18
TC01001710.hg.1	6.52	PSR01026694.hg.1	CDK18
TC01001710.hg.1	6.21	PSR01026690.hg.1	CDK18
TC01001710.hg.1	6.01	PSR01026670.hg.1	CDK18
TC01001710.hg.1	5.72	PSR01026645.hg.1	CDK18
TC01001710.hg.1	5.69	PSR01026683.hg.1	CDK18
TC01001710.hg.1	5.44	JUC01014311.hg.1	CDK18
TC01001710.hg.1	5.39	JUC01014295.hg.1	CDK18
TC01001710.hg.1	5.17	PSR01026678.hg.1	CDK18
TC01001710.hg.1	5	JUC01014287.hg.1	CDK18
TC01001710.hg.1	4.82	PSR01026658.hg.1	CDK18
TC01001710.hg.1	4.24	PSR01026636.hg.1	CDK18
TC01001710.hg.1	4.14	JUC01014305.hg.1	CDK18
TC01001710.hg.1	3.6	PSR01026641.hg.1	CDK18
TC01001710.hg.1	3.35	PSR01026696.hg.1	CDK18
TC01001710.hg.1	3.08	PSR01026671.hg.1	CDK18
TC01001710.hg.1	3.02	PSR01026644.hg.1	CDK18
TC01001710.hg.1	2.83	PSR01026643.hg.1	CDK18
TC01001710.hg.1	2.32	JUC01014283.hg.1	CDK18
TC01001710.hg.1	2.28	JUC01014285.hg.1	CDK18
TC01001710.hg.1	2.25	JUC01014297.hg.1	CDK18
TC01001710.hg.1	2.01	PSR01026651.hg.1	CDK18
TC01001710.hg.1	-2.19	PSR01026665.hg.1	CDK18
TC01001710.hg.1	-2.33	JUC01014298.hg.1	CDK18
TC01001710.hg.1	-2.62	PSR01026667.hg.1	CDK18
TC01001710.hg.1	-2.71	PSR01026646.hg.1	CDK18
TC01001710.hg.1	-2.75	JUC01014308.hg.1	CDK18
TC01001710.hg.1	-3.04	PSR01026692.hg.1	CDK18
TC01001710.hg.1	-3.05	PSR01026702.hg.1	CDK18
TC01001710.hg.1	-3.12	JUC01014296.hg.1	CDK18

TC01001710.hg.1	-3.74	PSR01026647.hg.1	CDK18
TC01001710.hg.1	-4.06	JUC01014294.hg.1	CDK18
TC01001710.hg.1	-7.64	PSR01026662.hg.1	CDK18
TC01001710.hg.1	-8.51	JUC01014302.hg.1	CDK18
TC06001029.hg.1	16.4	JUC06005887.hg.1	KIAA1244
TC06001029.hg.1	7.54	PSR06012058.hg.1	KIAA1244
TC06001029.hg.1	6.31	JUC06005875.hg.1	KIAA1244
TC06001029.hg.1	5.15	JUC06005888.hg.1	KIAA1244
TC06001029.hg.1	4.75	JUC06005896.hg.1	KIAA1244
TC06001029.hg.1	4.21	JUC06005866.hg.1	KIAA1244
TC06001029.hg.1	3.45	PSR06012056.hg.1	KIAA1244
TC06001029.hg.1	3.35	PSR06012055.hg.1	KIAA1244
TC06001029.hg.1	2.33	PSR06012057.hg.1	KIAA1244
TC06001029.hg.1	2	PSR06012060.hg.1	KIAA1244
TC06001029.hg.1	-2.01	JUC06005869.hg.1	KIAA1244
TC06001029.hg.1	-2.07	JUC06005899.hg.1	KIAA1244
TC06001029.hg.1	-2.12	PSR06012076.hg.1	KIAA1244
TC06001029.hg.1	-2.18	PSR06012043.hg.1	KIAA1244
TC06001029.hg.1	-2.41	PSR06012066.hg.1	KIAA1244
TC06001029.hg.1	-2.54	JUC06005895.hg.1	KIAA1244
TC06001029.hg.1	-2.58	JUC06005878.hg.1	KIAA1244
TC06001029.hg.1	-2.65	JUC06005871.hg.1	KIAA1244
TC06001029.hg.1	-2.74	PSR06012063.hg.1	KIAA1244
TC06001029.hg.1	-2.85	JUC06005873.hg.1	KIAA1244
TC06001029.hg.1	-2.88	JUC06005876.hg.1	KIAA1244
TC06001029.hg.1	-3.01	PSR06012061.hg.1	KIAA1244
TC06001029.hg.1	-3.05	JUC06005868.hg.1	KIAA1244
TC06001029.hg.1	-3.55	PSR06012077.hg.1	KIAA1244
TC06001029.hg.1	-3.58	JUC06005894.hg.1	KIAA1244
TC06001029.hg.1	-3.76	JUC06005885.hg.1	KIAA1244
TC06001029.hg.1	-3.87	JUC06005892.hg.1	KIAA1244
TC06001029.hg.1	-4.36	JUC06005877.hg.1	KIAA1244
TC09002932.hg.1	16.2	JUC09016215.hg.1	ANKRD20A1,
TC09002932.hg.1	2.37	PSR09003017.hg.1	ANKRD20A1,
TC09002932.hg.1	2.3	JUC09016214.hg.1	ANKRD20A1,
TC09002932.hg.1	2.19	JUC09016225.hg.1	ANKRD20A1,
TC09002932.hg.1	-2.37	JUC09016222.hg.1	ANKRD20A1,
TC6_cox_hap2000	16.15	JUC6_cox_hap2002(	CFB
TC6_cox_hap2000	14.43	PSR6_cox_hap2001:	CFB
TC6_cox_hap2000	13.4	PSR6_cox_hap2001:	CFB
TC6_cox_hap2000	12.99	PSR6_cox_hap2001:	CFB
TC6_cox_hap2000	12.23	PSR6_cox_hap2001	CFB
TC6_cox_hap2000	11.75	PSR6_cox_hap2001	CFB
TC6_cox_hap2000	10.73	PSR6_cox_hap2001:	CFB
TC6_cox_hap2000	7.56	PSR6_cox_hap2001:	CFB
TC6_cox_hap2000	6.81	PSR6_cox_hap2001:	CFB
TC6_cox_hap2000	5.66	PSR6_cox_hap2001:	CFB
TC6_cox_hap2000	5.64	PSR6_cox_hap2001:	CFB
TC6_cox_hap2000	5.35	PSR6_cox_hap2001	CFB
TC6_cox_hap2000	4.07	PSR6_cox_hap2001:	CFB
TC6_cox_hap2000	3.93	PSR6_cox_hap2001:	CFB
TC6_cox_hap2000	3.38	PSR6_cox_hap2001:	CFB
TC6_cox_hap2000	3.31	PSR6_cox_hap2001:	CFB
TC6_cox_hap2000	2.99	PSR6_cox_hap2001:	CFB
TC6_cox_hap2000	2.87	JUC6_cox_hap2002(	CFB
TC6_cox_hap2000	2.8	PSR6_cox_hap2001:	CFB
TC6_cox_hap2000	2.65	PSR6_cox_hap2001:	CFB
TC6_cox_hap2000	2.52	PSR6_cox_hap2001:	CFB

TC6_cox_hap2000	2.32	JUC6_cox_hap2002(	CFB
TC6_cox_hap2000	2.01	JUC6_cox_hap2002(	CFB
TC6_cox_hap2000	-2.46	JUC6_cox_hap2001(	CFB
TC6_dbb_hap3000	16.15	JUC6_dbb_hap3001	CFB
TC6_dbb_hap3000	14.43	PSR6_dbb_hap3001:	CFB
TC6_dbb_hap3000	13.4	PSR6_dbb_hap3001:	CFB
TC6_dbb_hap3000	12.99	PSR6_dbb_hap3001:	CFB
TC6_dbb_hap3000	12.23	PSR6_dbb_hap3001:	CFB
TC6_dbb_hap3000	11.14	PSR6_dbb_hap3001:	CFB
TC6_dbb_hap3000	10.73	PSR6_dbb_hap3001:	CFB
TC6_dbb_hap3000	7.56	PSR6_dbb_hap3001:	CFB
TC6_dbb_hap3000	6.81	PSR6_dbb_hap3001:	CFB
TC6_dbb_hap3000	5.66	PSR6_dbb_hap3001:	CFB
TC6_dbb_hap3000	5.64	PSR6_dbb_hap3001:	CFB
TC6_dbb_hap3000	5.35	PSR6_dbb_hap3001:	CFB
TC6_dbb_hap3000	4.07	PSR6_dbb_hap3001:	CFB
TC6_dbb_hap3000	3.93	PSR6_dbb_hap3001:	CFB
TC6_dbb_hap3000	3.38	PSR6_dbb_hap3001:	CFB
TC6_dbb_hap3000	3.31	PSR6_dbb_hap3001:	CFB
TC6_dbb_hap3000	2.99	PSR6_dbb_hap3001:	CFB
TC6_dbb_hap3000	2.87	JUC6_dbb_hap3001(	CFB
TC6_dbb_hap3000	2.8	PSR6_dbb_hap3001:	CFB
TC6_dbb_hap3000	2.65	PSR6_dbb_hap3001:	CFB
TC6_dbb_hap3000	2.52	PSR6_dbb_hap3001:	CFB
TC6_dbb_hap3000	2.32	JUC6_dbb_hap3001	CFB
TC6_dbb_hap3000	2.01	JUC6_dbb_hap3001	CFB
TC6_dbb_hap3000	-2.46	JUC6_dbb_hap3001	CFB
TC6_mcf_hap5000	16.15	JUC6_mcf_hap5001(	CFB
TC6_mcf_hap5000	14.43	PSR6_mcf_hap5001	CFB
TC6_mcf_hap5000	13.4	PSR6_mcf_hap5001	CFB
TC6_mcf_hap5000	12.99	PSR6_mcf_hap5001	CFB
TC6_mcf_hap5000	12.23	PSR6_mcf_hap5001	CFB
TC6_mcf_hap5000	11.14	PSR6_mcf_hap5001	CFB
TC6_mcf_hap5000	10.73	PSR6_mcf_hap5001	CFB
TC6_mcf_hap5000	8.04	PSR6_mcf_hap5001	CFB
TC6_mcf_hap5000	7.56	PSR6_mcf_hap5001	CFB
TC6_mcf_hap5000	6.81	PSR6_mcf_hap5001	CFB
TC6_mcf_hap5000	5.66	PSR6_mcf_hap5001	CFB
TC6_mcf_hap5000	5.64	PSR6_mcf_hap5001	CFB
TC6_mcf_hap5000	5.35	PSR6_mcf_hap5001	CFB
TC6_mcf_hap5000	4.07	PSR6_mcf_hap5001	CFB
TC6_mcf_hap5000	3.93	PSR6_mcf_hap5001	CFB
TC6_mcf_hap5000	3.38	PSR6_mcf_hap5001	CFB
TC6_mcf_hap5000	3.31	PSR6_mcf_hap5001	CFB
TC6_mcf_hap5000	2.99	PSR6_mcf_hap5001	CFB
TC6_mcf_hap5000	2.87	JUC6_mcf_hap5001(	CFB
TC6_mcf_hap5000	2.8	PSR6_mcf_hap5001	CFB
TC6_mcf_hap5000	2.65	PSR6_mcf_hap5001	CFB
TC6_mcf_hap5000	2.52	PSR6_mcf_hap5001	CFB
TC6_mcf_hap5000	2.32	JUC6_mcf_hap5001(	CFB
TC6_mcf_hap5000	2.01	JUC6_mcf_hap5001(	CFB
TC6_mcf_hap5000	-2.46	JUC6_mcf_hap5001(	CFB
TC6_qbl_hap6000	16.15	JUC6_qbl_hap60019	CFB
TC6_qbl_hap6000	14.43	PSR6_qbl_hap60013	CFB
TC6_qbl_hap6000	13.4	PSR6_qbl_hap60013	CFB
TC6_qbl_hap6000	12.99	PSR6_qbl_hap60013	CFB
TC6_qbl_hap6000	12.23	PSR6_qbl_hap60013	CFB
TC6_qbl_hap6000	11.14	PSR6_qbl_hap60014	CFB

TC6_qbl_hap6000‌2	10.73	PSR6_qbl_hap60013	CFB
TC6_qbl_hap6000‌2	7.56	PSR6_qbl_hap60013	CFB
TC6_qbl_hap6000‌2	6.81	PSR6_qbl_hap60013	CFB
TC6_qbl_hap6000‌2	5.66	PSR6_qbl_hap60013	CFB
TC6_qbl_hap6000‌2	5.64	PSR6_qbl_hap60013	CFB
TC6_qbl_hap6000‌2	5.35	PSR6_qbl_hap60013	CFB
TC6_qbl_hap6000‌2	4.07	PSR6_qbl_hap60013	CFB
TC6_qbl_hap6000‌2	3.93	PSR6_qbl_hap60013	CFB
TC6_qbl_hap6000‌2	3.38	PSR6_qbl_hap60013	CFB
TC6_qbl_hap6000‌2	3.31	PSR6_qbl_hap60013	CFB
TC6_qbl_hap6000‌2	2.99	PSR6_qbl_hap60013	CFB
TC6_qbl_hap6000‌2	2.87	JUC6_qbl_hap60019	CFB
TC6_qbl_hap6000‌2	2.8	PSR6_qbl_hap60013	CFB
TC6_qbl_hap6000‌2	2.65	PSR6_qbl_hap60013	CFB
TC6_qbl_hap6000‌2	2.52	PSR6_qbl_hap60013	CFB
TC6_qbl_hap6000‌2	2.32	JUC6_qbl_hap60019	CFB
TC6_qbl_hap6000‌2	2.01	JUC6_qbl_hap60019	CFB
TC6_qbl_hap6000‌2	-2.46	JUC6_qbl_hap60019	CFB
TC6_ssto_hap700(	16.15	JUC6_ssto_hap7001	CFB
TC6_ssto_hap700(	14.43	PSR6_ssto_hap7001	CFB
TC6_ssto_hap700(	13.4	PSR6_ssto_hap7001	CFB
TC6_ssto_hap700(	12.99	PSR6_ssto_hap7001	CFB
TC6_ssto_hap700(	12.23	PSR6_ssto_hap7001	CFB
TC6_ssto_hap700(	11.14	PSR6_ssto_hap7001	CFB
TC6_ssto_hap700(	10.73	PSR6_ssto_hap7001	CFB
TC6_ssto_hap700(	8.04	PSR6_ssto_hap7001	CFB
TC6_ssto_hap700(	7.56	PSR6_ssto_hap7001	CFB
TC6_ssto_hap700(	6.81	PSR6_ssto_hap7001	CFB
TC6_ssto_hap700(	5.66	PSR6_ssto_hap7001	CFB
TC6_ssto_hap700(	5.64	PSR6_ssto_hap7001	CFB
TC6_ssto_hap700(	5.35	PSR6_ssto_hap7001	CFB
TC6_ssto_hap700(	4.07	PSR6_ssto_hap7001	CFB
TC6_ssto_hap700(	3.93	PSR6_ssto_hap7001	CFB
TC6_ssto_hap700(	3.38	PSR6_ssto_hap7001	CFB
TC6_ssto_hap700(	3.31	PSR6_ssto_hap7001	CFB
TC6_ssto_hap700(	2.99	PSR6_ssto_hap7001	CFB
TC6_ssto_hap700(	2.87	JUC6_ssto_hap7001	CFB
TC6_ssto_hap700(	2.8	PSR6_ssto_hap7001	CFB
TC6_ssto_hap700(	2.65	PSR6_ssto_hap7001	CFB
TC6_ssto_hap700(	2.52	PSR6_ssto_hap7001	CFB
TC6_ssto_hap700(	2.32	JUC6_ssto_hap7001	CFB
TC6_ssto_hap700(	2.01	JUC6_ssto_hap7001	CFB
TC6_ssto_hap700(	-2.46	JUC6_ssto_hap7001	CFB
TC15000930.hg.1	15.75	JUC15004593.hg.1	MCTP2
TC15000930.hg.1	2.19	PSR15008655.hg.1	MCTP2
TC15001541.hg.1	15.73	JUC15007849.hg.1	CA12
TC15001541.hg.1	10.99	PSR15014514.hg.1	CA12
TC15001541.hg.1	10.68	PSR15014515.hg.1	CA12
TC15001541.hg.1	9.57	PSR15014492.hg.1	CA12
TC15001541.hg.1	4.54	PSR15014511.hg.1	CA12
TC15001541.hg.1	4.36	JUC15007842.hg.1	CA12
TC15001541.hg.1	3.67	PSR15014503.hg.1	CA12
TC15001541.hg.1	3.65	PSR15014502.hg.1	CA12
TC15001541.hg.1	3.07	JUC15007843.hg.1	CA12
TC15001541.hg.1	2.32	JUC15007840.hg.1	CA12
TC15001541.hg.1	-3.17	JUC15007846.hg.1	CA12
TC15001541.hg.1	-3.65	JUC15007839.hg.1	CA12
TC11001589.hg.1	15.57	JUC11010566.hg.1	CHST1

TC11001589.hg.1	3.67	PSR11019775.hg.1	CHST1
TC11001589.hg.1	3.35	PSR11019785.hg.1	CHST1
TC11001589.hg.1	3.35	PSR11019787.hg.1	CHST1
TC11001589.hg.1	3.07	JUC11010567.hg.1	CHST1
TC11001589.hg.1	3.04	JUC11010564.hg.1	CHST1
TC11001589.hg.1	2.59	PSR11019786.hg.1	CHST1
TC11001589.hg.1	2.5	JUC11010563.hg.1	CHST1
TC11001589.hg.1	2.46	PSR11019773.hg.1	CHST1
TC11001589.hg.1	-2.06	PSR11019784.hg.1	CHST1
TC03000716.hg.1	15.38	JUC03006529.hg.1	CCRL1
TC03000716.hg.1	14.11	PSR03012884.hg.1	CCRL1
TC03000716.hg.1	7.45	PSR03012883.hg.1	CCRL1
TC03000716.hg.1	2.59	JUC03006527.hg.1	CCRL1
TC03000716.hg.1	-2.17	PSR03012890.hg.1	CCRL1
TC03000716.hg.1	-2.26	PSR03012886.hg.1	CCRL1
TC02002143.hg.1	15.28	JUC02018085.hg.1	TBC1D8
TC02002143.hg.1	8.64	PSR02035035.hg.1	TBC1D8
TC02002143.hg.1	8.55	JUC02018071.hg.1	TBC1D8
TC02002143.hg.1	8.11	PSR02035037.hg.1	TBC1D8
TC02002143.hg.1	7.76	JUC02018062.hg.1	TBC1D8
TC02002143.hg.1	6.19	PSR02035040.hg.1	TBC1D8
TC02002143.hg.1	5.93	PSR02035042.hg.1	TBC1D8
TC02002143.hg.1	5.68	PSR02035027.hg.1	TBC1D8
TC02002143.hg.1	5.6	JUC02018077.hg.1	TBC1D8
TC02002143.hg.1	5.58	PSR02035039.hg.1	TBC1D8
TC02002143.hg.1	5.48	PSR02035036.hg.1	TBC1D8
TC02002143.hg.1	5.33	PSR02035015.hg.1	TBC1D8
TC02002143.hg.1	5.22	PSR02035041.hg.1	TBC1D8
TC02002143.hg.1	4.71	PSR02035033.hg.1	TBC1D8
TC02002143.hg.1	4.49	JUC02018078.hg.1	TBC1D8
TC02002143.hg.1	4.23	JUC02018068.hg.1	TBC1D8
TC02002143.hg.1	3.88	PSR02035013.hg.1	TBC1D8
TC02002143.hg.1	3.84	PSR02035002.hg.1	TBC1D8
TC02002143.hg.1	3.53	PSR02035026.hg.1	TBC1D8
TC02002143.hg.1	3.35	PSR02035019.hg.1	TBC1D8
TC02002143.hg.1	3.33	PSR02035004.hg.1	TBC1D8
TC02002143.hg.1	3.04	PSR02035025.hg.1	TBC1D8
TC02002143.hg.1	2.91	PSR02035012.hg.1	TBC1D8
TC02002143.hg.1	2.74	JUC02018079.hg.1	TBC1D8
TC02002143.hg.1	2.67	JUC02018076.hg.1	TBC1D8
TC02002143.hg.1	2.25	PSR02035007.hg.1	TBC1D8
TC02002143.hg.1	2.25	PSR02035017.hg.1	TBC1D8
TC02002143.hg.1	2.2	JUC02018083.hg.1	TBC1D8
TC02002143.hg.1	2.05	PSR02035016.hg.1	TBC1D8
TC02002143.hg.1	-2.01	JUC02018070.hg.1	TBC1D8
TC02002143.hg.1	-2.05	JUC02018074.hg.1	TBC1D8
TC02002143.hg.1	-2.51	JUC02018088.hg.1	TBC1D8
TC07000220.hg.1	15.03	JUC07001632.hg.1	BMPER
TC07000220.hg.1	7.58	PSR07003442.hg.1	BMPER
TC07000220.hg.1	6.33	JUC07001633.hg.1	BMPER
TC07000220.hg.1	6.32	PSR07003465.hg.1	BMPER
TC07000220.hg.1	5.17	JUC07001637.hg.1	BMPER
TC07000220.hg.1	4.93	PSR07003471.hg.1	BMPER
TC07000220.hg.1	3.36	PSR07003452.hg.1	BMPER
TC07000220.hg.1	2.8	JUC07001641.hg.1	BMPER
TC07000220.hg.1	2.78	PSR07003459.hg.1	BMPER
TC07000220.hg.1	2.76	JUC07001640.hg.1	BMPER
TC07000220.hg.1	2.46	PSR07003448.hg.1	BMPER

TC07000220.hg.1	-2.09	PSR07003460.hg.1	BMPER
TC07000220.hg.1	-2.5	JUC07001630.hg.1	BMPER
TC07000220.hg.1	-2.72	PSR07003464.hg.1	BMPER
TC07000220.hg.1	-2.76	JUC07001627.hg.1	BMPER
TC07000220.hg.1	-3.54	JUC07001634.hg.1	BMPER
TC01001030.hg.1	14.85	JUC01008564.hg.1	FAM46C
TC01001030.hg.1	14.85	JUC01008566.hg.1	FAM46C
TC01001030.hg.1	3.2	PSR01016356.hg.1	FAM46C
TC01001030.hg.1	2.9	PSR01016353.hg.1	FAM46C
TC01001030.hg.1	2.29	PSR01016355.hg.1	FAM46C
TC01001030.hg.1	2.17	JUC01008565.hg.1	FAM46C
TC07001751.hg.1	14.77	JUC07013421.hg.1	NRCAM
TC07001751.hg.1	3.64	JUC07013464.hg.1	NRCAM
TC07001751.hg.1	2.89	JUC07013434.hg.1	NRCAM
TC07001751.hg.1	2.82	JUC07013432.hg.1	NRCAM
TC07001751.hg.1	2.45	JUC07013451.hg.1	NRCAM
TC07001751.hg.1	2.41	JUC07013462.hg.1	NRCAM
TC07001751.hg.1	2.31	JUC07013424.hg.1	NRCAM
TC07001751.hg.1	2.28	PSR07027358.hg.1	NRCAM
TC07001751.hg.1	2.14	PSR07027362.hg.1	NRCAM
TC07001751.hg.1	2.06	PSR07027301.hg.1	NRCAM
TC07001751.hg.1	2.05	JUC07013445.hg.1	NRCAM
TC07001751.hg.1	-2.06	JUC07013420.hg.1	NRCAM
TC07001751.hg.1	-2.17	JUC07013422.hg.1	NRCAM
TC07001751.hg.1	-2.22	PSR07027367.hg.1	NRCAM
TC07001751.hg.1	-2.24	PSR07027336.hg.1	NRCAM
TC07001751.hg.1	-2.28	PSR07027330.hg.1	NRCAM
TC07001751.hg.1	-2.3	PSR07027295.hg.1	NRCAM
TC07001751.hg.1	-2.33	PSR07027331.hg.1	NRCAM
TC07001751.hg.1	-2.42	JUC07013469.hg.1	NRCAM
TC07001751.hg.1	-2.46	PSR07027335.hg.1	NRCAM
TC07001751.hg.1	-2.52	PSR07027329.hg.1	NRCAM
TC07001751.hg.1	-2.67	PSR07027312.hg.1	NRCAM
TC07001751.hg.1	-2.73	PSR07027372.hg.1	NRCAM
TC07001751.hg.1	-2.82	PSR07027296.hg.1	NRCAM
TC07001751.hg.1	-2.82	JUC07013435.hg.1	NRCAM
TC07001751.hg.1	-2.94	PSR07027365.hg.1	NRCAM
TC07001751.hg.1	-3.1	JUC07013449.hg.1	NRCAM
TC07001751.hg.1	-3.15	PSR07027317.hg.1	NRCAM
TC07001751.hg.1	-3.15	PSR07027369.hg.1	NRCAM
TC07001751.hg.1	-3.16	PSR07027332.hg.1	NRCAM
TC07001751.hg.1	-3.35	JUC07013417.hg.1	NRCAM
TC07001751.hg.1	-3.64	JUC07013456.hg.1	NRCAM
TC07001751.hg.1	-3.88	PSR07027303.hg.1	NRCAM
TC07001751.hg.1	-4.15	PSR07027363.hg.1	NRCAM
TC07001751.hg.1	-4.2	PSR07027360.hg.1	NRCAM
TC07001751.hg.1	-5.1	JUC07013465.hg.1	NRCAM
TC07001751.hg.1	-5.68	PSR07027322.hg.1	NRCAM
TC07001751.hg.1	-6.62	JUC07013467.hg.1	NRCAM
TC07001751.hg.1	-7.39	JUC07013459.hg.1	NRCAM
TC07001751.hg.1	-8.58	JUC07013452.hg.1	NRCAM
TC11001027.hg.1	14.75	PSR11012850.hg.1	NNMT
TC11001027.hg.1	4.46	PSR11012851.hg.1	NNMT
TC11001027.hg.1	3.31	JUC11006915.hg.1	NNMT
TC11001027.hg.1	2.36	PSR11012848.hg.1	NNMT
TC11001027.hg.1	2.04	PSR11012863.hg.1	NNMT
TC11001027.hg.1	-4.04	PSR11012853.hg.1	NNMT
TC01006400.hg.1	14.74	JUC01046627.hg.1	GSTM1

TC01006400.hg.1	12.81	JUC01046631.hg.1	GSTM1
TC01006400.hg.1	10.9	PSR01015155.hg.1	GSTM1
TC18000077.hg.1	14.71	JUC18000529.hg.1	ANKRD20A5I
TC18000077.hg.1	2.4	JUC18000533.hg.1	ANKRD20A5I
TC18000077.hg.1	2.18	JUC18000530.hg.1	ANKRD20A5I
TC18000077.hg.1	2.14	JUC18000538.hg.1	ANKRD20A5I
TC18000077.hg.1	-2.28	PSR18000886.hg.1	ANKRD20A5I
TC09001097.hg.1	14.66	JUC09007862.hg.1	
TC09001097.hg.1	2.69	PSR09014647.hg.1	
TC09001097.hg.1	2.16	PSR09014646.hg.1	
TC09001097.hg.1	-2.66	PSR09014643.hg.1	
TC09001097.hg.1	-2.86	JUC09007858.hg.1	
TC09001097.hg.1	-4.51	JUC09007859.hg.1	
TC09001097.hg.1	-4.88	JUC09007861.hg.1	
TC09000086.hg.1	14.56	JUC09000563.hg.1	ADAMTSL1
TC09000086.hg.1	6.07	JUC09000567.hg.1	ADAMTSL1
TC09000086.hg.1	4.63	PSR09001021.hg.1	ADAMTSL1
TC09000086.hg.1	3.79	JUC09000560.hg.1	ADAMTSL1
TC09000086.hg.1	3.73	PSR09001028.hg.1	ADAMTSL1
TC09000086.hg.1	3.63	PSR09001034.hg.1	ADAMTSL1
TC09000086.hg.1	3.55	JUC09000568.hg.1	ADAMTSL1
TC09000086.hg.1	3.44	JUC09000576.hg.1	ADAMTSL1
TC09000086.hg.1	3.23	PSR09001059.hg.1	ADAMTSL1
TC09000086.hg.1	3.18	PSR09001069.hg.1	ADAMTSL1
TC09000086.hg.1	3.12	PSR09001048.hg.1	ADAMTSL1
TC09000086.hg.1	2.93	PSR09001023.hg.1	ADAMTSL1
TC09000086.hg.1	2.74	PSR09001075.hg.1	ADAMTSL1
TC09000086.hg.1	2.71	PSR09001080.hg.1	ADAMTSL1
TC09000086.hg.1	2.71	JUC09000558.hg.1	ADAMTSL1
TC09000086.hg.1	2.53	JUC09000573.hg.1	ADAMTSL1
TC09000086.hg.1	2.34	PSR09001070.hg.1	ADAMTSL1
TC09000086.hg.1	2.25	PSR09001057.hg.1	ADAMTSL1
TC09000086.hg.1	2.21	JUC09000566.hg.1	ADAMTSL1
TC09000086.hg.1	2.11	JUC09000577.hg.1	ADAMTSL1
TC09000086.hg.1	2.1	JUC09000557.hg.1	ADAMTSL1
TC09000086.hg.1	2.07	PSR09001073.hg.1	ADAMTSL1
TC09000086.hg.1	2.01	PSR09001078.hg.1	ADAMTSL1
TC09000086.hg.1	-2.01	PSR09001077.hg.1	ADAMTSL1
TC09000086.hg.1	-2.19	JUC09000571.hg.1	ADAMTSL1
TC09000086.hg.1	-2.24	JUC09000561.hg.1	ADAMTSL1
TC09000086.hg.1	-2.35	PSR09001058.hg.1	ADAMTSL1
TC09000086.hg.1	-2.88	JUC09000559.hg.1	ADAMTSL1
TC09000086.hg.1	-2.92	JUC09000574.hg.1	ADAMTSL1
TC09000086.hg.1	-3.22	PSR09001039.hg.1	ADAMTSL1
TC09000086.hg.1	-3.38	JUC09000556.hg.1	ADAMTSL1
TC09000086.hg.1	-3.59	PSR09001041.hg.1	ADAMTSL1
TC09000086.hg.1	-3.65	PSR09001038.hg.1	ADAMTSL1
TC09000086.hg.1	-3.82	PSR09001033.hg.1	ADAMTSL1
TC09000086.hg.1	-4.38	JUC09000565.hg.1	ADAMTSL1
TC09000086.hg.1	-4.83	PSR09001040.hg.1	ADAMTSL1
TC09000086.hg.1	-4.97	PSR09001030.hg.1	ADAMTSL1
TC09000086.hg.1	-5.17	JUC09000582.hg.1	ADAMTSL1
TC09000086.hg.1	-5.27	PSR09001037.hg.1	ADAMTSL1
TC09000086.hg.1	-5.94	PSR09001031.hg.1	ADAMTSL1
TC09000086.hg.1	-7.72	JUC09000585.hg.1	ADAMTSL1
TC09000086.hg.1	-8.45	JUC09000583.hg.1	ADAMTSL1
TC09000086.hg.1	-9.37	JUC09000570.hg.1	ADAMTSL1
TC09000086.hg.1	-10.12	PSR09001036.hg.1	ADAMTSL1

TC08001013.hg.1	14.4	JUC08006601.hg.1	MTUS1
TC08001013.hg.1	3.8	JUC08006614.hg.1	MTUS1
TC08001013.hg.1	3.66	JUC08006626.hg.1	MTUS1
TC08001013.hg.1	3.04	PSR08012784.hg.1	MTUS1
TC08001013.hg.1	2.94	PSR08012772.hg.1	MTUS1
TC08001013.hg.1	2.88	JUC08006609.hg.1	MTUS1
TC08001013.hg.1	2.67	JUC08006606.hg.1	MTUS1
TC08001013.hg.1	2.5	PSR08012762.hg.1	MTUS1
TC08001013.hg.1	2.45	PSR08012746.hg.1	MTUS1
TC08001013.hg.1	2.33	JUC08006627.hg.1	MTUS1
TC08001013.hg.1	2.24	PSR08012756.hg.1	MTUS1
TC08001013.hg.1	2.23	PSR08012742.hg.1	MTUS1
TC08001013.hg.1	2.21	PSR08012731.hg.1	MTUS1
TC08001013.hg.1	2.21	PSR08012764.hg.1	MTUS1
TC08001013.hg.1	2.21	JUC08006600.hg.1	MTUS1
TC08001013.hg.1	2.18	PSR08012768.hg.1	MTUS1
TC08001013.hg.1	2.11	PSR08012788.hg.1	MTUS1
TC08001013.hg.1	2.03	PSR08012736.hg.1	MTUS1
TC08001013.hg.1	-2.02	JUC08006619.hg.1	MTUS1
TC01001607.hg.1	14.34	PSR01024936.hg.1	PLA2G4A
TC01001607.hg.1	9.21	JUC01013350.hg.1	PLA2G4A
TC01001607.hg.1	6.75	PSR01024943.hg.1	PLA2G4A
TC01001607.hg.1	6.01	JUC01013348.hg.1	PLA2G4A
TC01001607.hg.1	5.2	JUC01013363.hg.1	PLA2G4A
TC01001607.hg.1	4.88	JUC01013354.hg.1	PLA2G4A
TC01001607.hg.1	3.52	JUC01013347.hg.1	PLA2G4A
TC01001607.hg.1	3.33	JUC01013366.hg.1	PLA2G4A
TC01001607.hg.1	2.43	PSR01024940.hg.1	PLA2G4A
TC01001607.hg.1	2.2	JUC01013357.hg.1	PLA2G4A
TC01001607.hg.1	2.16	JUC01013361.hg.1	PLA2G4A
TC01001607.hg.1	2.1	JUC01013360.hg.1	PLA2G4A
TC01001607.hg.1	-2.06	PSR01024959.hg.1	PLA2G4A
TC01001607.hg.1	-2.42	PSR01024946.hg.1	PLA2G4A
TC01001607.hg.1	-2.54	JUC01013351.hg.1	PLA2G4A
TC06002113.hg.1	14.22	JUC06013133.hg.1	MOXD1
TC06002113.hg.1	9.35	PSR06026518.hg.1	MOXD1
TC06002113.hg.1	8.53	PSR06026525.hg.1	MOXD1
TC06002113.hg.1	8.47	PSR06026528.hg.1	MOXD1
TC06002113.hg.1	4.22	PSR06026526.hg.1	MOXD1
TC06002113.hg.1	2.93	JUC06013131.hg.1	MOXD1
TC06002113.hg.1	2.55	PSR06026524.hg.1	MOXD1
TC06002113.hg.1	2.41	JUC06013142.hg.1	MOXD1
TC06002113.hg.1	2.06	JUC06013135.hg.1	MOXD1
TC06002113.hg.1	2.02	PSR06026517.hg.1	MOXD1
TC06002113.hg.1	-2.04	JUC06013138.hg.1	MOXD1
TC01006269.hg.1	14	JUC01038991.hg.1	
TC01006269.hg.1	8.34	PSR01009162.hg.1	
TC01006269.hg.1	7.24	PSR01009171.hg.1	
TC01006269.hg.1	6.28	JUC01038993.hg.1	
TC01006269.hg.1	6.18	JUC01038995.hg.1	
TC01006269.hg.1	6.06	JUC01038996.hg.1	
TC01006269.hg.1	5.64	JUC01038992.hg.1	
TC01006269.hg.1	4.69	PSR01009175.hg.1	
TC01006269.hg.1	4.4	JUC01038998.hg.1	
TC01006269.hg.1	4.29	PSR01009177.hg.1	
TC01006269.hg.1	3.82	JUC01039001.hg.1	
TC01006269.hg.1	3.76	PSR01009176.hg.1	
TC01006269.hg.1	3.74	PSR01009178.hg.1	

TC01006269.hg.1	2.69	PSR01009172.hg.1	
TC01006269.hg.1	-2.23	JUC01038999.hg.1	
TC11000173.hg.1	14	JUC11001057.hg.1	ZNF143
TC11000173.hg.1	2.28	JUC11001055.hg.1	ZNF143
TC11000173.hg.1	2.02	PSR11002346.hg.1	ZNF143
TC11000173.hg.1	-3.53	JUC11001062.hg.1	ZNF143
TC01001258.hg.1	13.94	JUC01010429.hg.1	S100A1
TC01001258.hg.1	8.51	PSR01019177.hg.1	S100A1
TC01001258.hg.1	8.48	PSR01019176.hg.1	S100A1
TC01001258.hg.1	8.42	JUC01010428.hg.1	S100A1
TC01001258.hg.1	7.66	JUC01010425.hg.1	S100A1
TC01001258.hg.1	7.17	PSR01019170.hg.1	S100A1
TC01001258.hg.1	6.61	PSR01019179.hg.1	S100A1
TC05001590.hg.1	13.9	PSR05021639.hg.1	ARRDC3
TC05001590.hg.1	10.16	PSR05021635.hg.1	ARRDC3
TC05001590.hg.1	7.85	PSR05021634.hg.1	ARRDC3
TC05001590.hg.1	6.73	JUC05011125.hg.1	ARRDC3
TC05001590.hg.1	6.12	JUC05011116.hg.1	ARRDC3
TC05001590.hg.1	5.18	PSR05021632.hg.1	ARRDC3
TC05001590.hg.1	3.84	JUC05011118.hg.1	ARRDC3
TC05001590.hg.1	2.49	PSR05021631.hg.1	ARRDC3
TC05001590.hg.1	2.08	PSR05021629.hg.1	ARRDC3
TC05001590.hg.1	-2.07	PSR05021605.hg.1	ARRDC3
TC11000182.hg.1	13.79	PSR11002447.hg.1	ADM
TC11000182.hg.1	8.16	PSR11002452.hg.1	ADM
TC11000182.hg.1	7.72	JUC11001098.hg.1	ADM
TC11000182.hg.1	6.86	PSR11002449.hg.1	ADM
TC11000182.hg.1	5.1	PSR11002446.hg.1	ADM
TC11000182.hg.1	5.06	JUC11001100.hg.1	ADM
TC11000182.hg.1	4.6	JUC11001101.hg.1	ADM
TC11000182.hg.1	3.82	PSR11002453.hg.1	ADM
TC11000182.hg.1	-2.55	PSR11002461.hg.1	ADM
TC11000182.hg.1	-2.74	JUC11001103.hg.1	ADM
TC08001022.hg.1	13.67	JUC08006752.hg.1	CSGALNACT
TC08001022.hg.1	11.67	JUC08006748.hg.1	CSGALNACT
TC08001022.hg.1	11.64	JUC08006744.hg.1	CSGALNACT
TC08001022.hg.1	10.39	JUC08006725.hg.1	CSGALNACT
TC08001022.hg.1	9.47	JUC08006726.hg.1	CSGALNACT
TC08001022.hg.1	8.89	PSR08013006.hg.1	CSGALNACT
TC08001022.hg.1	8.77	JUC08006751.hg.1	CSGALNACT
TC08001022.hg.1	8.66	PSR08012998.hg.1	CSGALNACT
TC08001022.hg.1	8.45	JUC08006733.hg.1	CSGALNACT
TC08001022.hg.1	8.37	PSR08012996.hg.1	CSGALNACT
TC08001022.hg.1	8.32	PSR08012995.hg.1	CSGALNACT
TC08001022.hg.1	8.29	JUC08006735.hg.1	CSGALNACT
TC08001022.hg.1	8.29	JUC08006736.hg.1	CSGALNACT
TC08001022.hg.1	8.1	PSR08012973.hg.1	CSGALNACT
TC08001022.hg.1	7.93	PSR08013003.hg.1	CSGALNACT
TC08001022.hg.1	7.93	JUC08006747.hg.1	CSGALNACT
TC08001022.hg.1	7.71	JUC08006750.hg.1	CSGALNACT
TC08001022.hg.1	7.48	JUC08006727.hg.1	CSGALNACT
TC08001022.hg.1	7.35	JUC08006730.hg.1	CSGALNACT
TC08001022.hg.1	6.91	PSR08012993.hg.1	CSGALNACT
TC08001022.hg.1	6.88	PSR08012997.hg.1	CSGALNACT
TC08001022.hg.1	6.65	PSR08012994.hg.1	CSGALNACT
TC08001022.hg.1	6.46	PSR08012988.hg.1	CSGALNACT
TC08001022.hg.1	6.16	JUC08006740.hg.1	CSGALNACT
TC08001022.hg.1	5.76	PSR08012989.hg.1	CSGALNACT

TC08001022.hg.1	5.23	PSR08012987.hg.1	CSGALNACT
TC08001022.hg.1	5.14	PSR08012971.hg.1	CSGALNACT
TC08001022.hg.1	5.11	PSR08012992.hg.1	CSGALNACT
TC08001022.hg.1	4.75	JUC08006742.hg.1	CSGALNACT
TC08001022.hg.1	4.68	JUC08006728.hg.1	CSGALNACT
TC08001022.hg.1	4.17	JUC08006741.hg.1	CSGALNACT
TC08001022.hg.1	3.96	PSR08013000.hg.1	CSGALNACT
TC08001022.hg.1	3.55	JUC08006753.hg.1	CSGALNACT
TC08001022.hg.1	3.43	PSR08012985.hg.1	CSGALNACT
TC08001022.hg.1	3.41	JUC08006737.hg.1	CSGALNACT
TC08001022.hg.1	3.36	PSR08012983.hg.1	CSGALNACT
TC08001022.hg.1	3.31	PSR08013001.hg.1	CSGALNACT
TC08001022.hg.1	2.88	JUC08006749.hg.1	CSGALNACT
TC08001022.hg.1	2.67	PSR08012984.hg.1	CSGALNACT
TC08001022.hg.1	2.51	PSR08012991.hg.1	CSGALNACT
TC08001022.hg.1	-3.13	JUC08006731.hg.1	CSGALNACT
TC04002929.hg.1	13.55	JUC04017042.hg.1	BST1
TC04002929.hg.1	5.05	JUC04017031.hg.1	BST1
TC04002929.hg.1	5.02	PSR04002285.hg.1	BST1
TC04002929.hg.1	4.91	PSR04002301.hg.1	BST1
TC04002929.hg.1	4.73	JUC04017038.hg.1	BST1
TC04002929.hg.1	4.61	PSR04002302.hg.1	BST1
TC04002929.hg.1	4.19	PSR04002303.hg.1	BST1
TC04002929.hg.1	3.83	PSR04002282.hg.1	BST1
TC04002929.hg.1	3.79	PSR04002297.hg.1	BST1
TC04002929.hg.1	2.54	JUC04017034.hg.1	BST1
TC04002929.hg.1	2.25	PSR04002284.hg.1	BST1
TC02002819.hg.1	13.41	JUC02023745.hg.1	AP1S3
TC02002819.hg.1	12.69	JUC02023750.hg.1	AP1S3
TC02002819.hg.1	11.85	JUC02023753.hg.1	AP1S3
TC02002819.hg.1	11.23	JUC02023748.hg.1	AP1S3
TC02002819.hg.1	10.96	JUC02023741.hg.1	AP1S3
TC02002819.hg.1	4.96	PSR02045505.hg.1	AP1S3
TC02002819.hg.1	3.96	PSR02045504.hg.1	AP1S3
TC02002819.hg.1	3.88	PSR02045495.hg.1	AP1S3
TC02002819.hg.1	3.86	PSR02045498.hg.1	AP1S3
TC02002819.hg.1	3.78	JUC02023752.hg.1	AP1S3
TC02002819.hg.1	3.72	PSR02045490.hg.1	AP1S3
TC02002819.hg.1	3.41	PSR02045479.hg.1	AP1S3
TC02002819.hg.1	3.19	PSR02045499.hg.1	AP1S3
TC02002819.hg.1	2.98	PSR02045480.hg.1	AP1S3
TC02002819.hg.1	2.42	JUC02023743.hg.1	AP1S3
TC02002819.hg.1	2.02	PSR02045484.hg.1	AP1S3
TC02002819.hg.1	-2.18	JUC02023749.hg.1	AP1S3
TC01001625.hg.1	13.35	PSR01025117.hg.1	TROVE2
TC01001625.hg.1	2.24	PSR01025086.hg.1	TROVE2
TC01001625.hg.1	-5.11	JUC01013440.hg.1	TROVE2
TC08000500.hg.1	13.1	PSR08006842.hg.1	
TC08000500.hg.1	7.61	JUC08003377.hg.1	
TC08000500.hg.1	3.6	JUC08003381.hg.1	
TC08000500.hg.1	-2.18	PSR08006844.hg.1	
TC08000500.hg.1	-3.18	JUC08003378.hg.1	
TC17001937.hg.1	13.08	JUC17014859.hg.1	NPTX1
TC17001937.hg.1	4.38	PSR17026383.hg.1	NPTX1
TC17001937.hg.1	2.79	PSR17026384.hg.1	NPTX1
TC17001937.hg.1	2.77	JUC17014862.hg.1	NPTX1
TC17001937.hg.1	-2.51	PSR17026387.hg.1	NPTX1
TC06000408.hg.1	13	JUC06001845.hg.1	HLA-DPB1

TC06000408.hg.1	6.28	PSR06004790.hg.1	HLA-DPB1
TC06000408.hg.1	6.17	JUC06001841.hg.1	HLA-DPB1
TC06000408.hg.1	5.82	PSR06004789.hg.1	HLA-DPB1
TC06000408.hg.1	5.81	JUC06001846.hg.1	HLA-DPB1
TC06000408.hg.1	5.5	JUC06001855.hg.1	HLA-DPB1
TC06000408.hg.1	5.02	PSR06004787.hg.1	HLA-DPB1
TC06000408.hg.1	4.65	PSR06004808.hg.1	HLA-DPB1
TC06000408.hg.1	4.28	JUC06001842.hg.1	HLA-DPB1
TC06000408.hg.1	3.7	JUC06001848.hg.1	HLA-DPB1
TC06000408.hg.1	3.45	PSR06004779.hg.1	HLA-DPB1
TC06000408.hg.1	3.4	PSR06004783.hg.1	HLA-DPB1
TC06000408.hg.1	3.33	PSR06004794.hg.1	HLA-DPB1
TC06000408.hg.1	3.29	PSR06004793.hg.1	HLA-DPB1
TC06000408.hg.1	3.02	JUC06001850.hg.1	HLA-DPB1
TC06000408.hg.1	2.38	PSR06004785.hg.1	HLA-DPB1
TC06000408.hg.1	2.29	PSR06004806.hg.1	HLA-DPB1
TC06000408.hg.1	2.05	PSR06004782.hg.1	HLA-DPB1
TC06000408.hg.1	-2.11	JUC06001849.hg.1	HLA-DPB1
TC06004077.hg.1	12.82	JUC06021027.hg.1	CFB
TC06004077.hg.1	11.45	PSR06004095.hg.1	CFB
TC06004077.hg.1	11.17	PSR06004107.hg.1	CFB
TC06004077.hg.1	10.91	JUC06021031.hg.1	CFB
TC06004077.hg.1	10.42	PSR06004082.hg.1	CFB
TC06004077.hg.1	9.7	PSR06004117.hg.1	CFB
TC06004077.hg.1	9.32	PSR06004127.hg.1	CFB
TC06004077.hg.1	8.51	PSR06004113.hg.1	CFB
TC06004077.hg.1	6.99	PSR06004084.hg.1	CFB
TC06004077.hg.1	6.45	PSR06004091.hg.1	CFB
TC06004077.hg.1	6	PSR06004097.hg.1	CFB
TC06004077.hg.1	5.4	PSR06004108.hg.1	CFB
TC06004077.hg.1	4.24	PSR06004118.hg.1	CFB
TC06004077.hg.1	3.45	PSR06004085.hg.1	CFB
TC06004077.hg.1	3.23	PSR06004101.hg.1	CFB
TC06004077.hg.1	3.12	PSR06004087.hg.1	CFB
TC06004077.hg.1	2.68	PSR06004096.hg.1	CFB
TC06004077.hg.1	2.63	PSR06004094.hg.1	CFB
TC06004077.hg.1	2.28	JUC06021022.hg.1	CFB
TC06004077.hg.1	2.23	PSR06004105.hg.1	CFB
TC06004077.hg.1	2.22	PSR06004089.hg.1	CFB
TC06004077.hg.1	2.1	PSR06004098.hg.1	CFB
TC06004077.hg.1	2	PSR06004088.hg.1	CFB
TC06004077.hg.1	-2.02	JUC06021021.hg.1	CFB
TC06004077.hg.1	-2.03	JUC06021024.hg.1	CFB
TC06004077.hg.1	-2.07	PSR06004126.hg.1	CFB
TC06004077.hg.1	-2.23	JUC06021032.hg.1	CFB
TC06004077.hg.1	-3.1	JUC06021026.hg.1	CFB
TC01000545.hg.1	12.78	JUC01004478.hg.1	CDC20
TC01000545.hg.1	2.37	PSR01008666.hg.1	CDC20
TC01000545.hg.1	2.33	PSR01008667.hg.1	CDC20
TC01000545.hg.1	2.26	PSR01008681.hg.1	CDC20
TC01000545.hg.1	2.16	PSR01008662.hg.1	CDC20
TC01000545.hg.1	2.07	PSR01008672.hg.1	CDC20
TC01000545.hg.1	2.05	PSR01008663.hg.1	CDC20
TC01000545.hg.1	2.04	PSR01008671.hg.1	CDC20
TC01000545.hg.1	-2.2	JUC01004472.hg.1	CDC20
TC17000124.hg.1	12.71	PSR17001843.hg.1	ALOX15B
TC17000124.hg.1	7.91	JUC17001078.hg.1	ALOX15B
TC17000124.hg.1	4.67	JUC17001084.hg.1	ALOX15B

TC17000124.hg.1	4.07	PSR17001844.hg.1	ALOX15B
TC17000124.hg.1	2.05	PSR17001841.hg.1	ALOX15B
TC17000124.hg.1	-2.3	PSR17001832.hg.1	ALOX15B
TC17000124.hg.1	-2.61	PSR17001833.hg.1	ALOX15B
TC17000124.hg.1	-3.04	JUC17001074.hg.1	ALOX15B
TC17000124.hg.1	-3.09	JUC17001080.hg.1	ALOX15B
TC17000124.hg.1	-3.28	PSR17001840.hg.1	ALOX15B
TC17000124.hg.1	-5.39	JUC17001081.hg.1	ALOX15B
TC17000124.hg.1	-6.27	PSR17001829.hg.1	ALOX15B
TC08002617.hg.1	12.7	JUC08015262.hg.1	TPD52
TC08002617.hg.1	8.85	JUC08015265.hg.1	TPD52
TC08002617.hg.1	5.33	JUC08015282.hg.1	TPD52
TC08002617.hg.1	4.61	JUC08015270.hg.1	TPD52
TC08002617.hg.1	4.3	JUC08015264.hg.1	TPD52
TC08002617.hg.1	4.2	PSR08017513.hg.1	TPD52
TC08002617.hg.1	3.87	PSR08017500.hg.1	TPD52
TC08002617.hg.1	3.81	JUC08015275.hg.1	TPD52
TC08002617.hg.1	3.69	JUC08015285.hg.1	TPD52
TC08002617.hg.1	3.47	JUC08015272.hg.1	TPD52
TC08002617.hg.1	3.4	PSR08017517.hg.1	TPD52
TC08002617.hg.1	3.16	PSR08017503.hg.1	TPD52
TC08002617.hg.1	3.04	JUC08015288.hg.1	TPD52
TC08002617.hg.1	2.99	PSR08017522.hg.1	TPD52
TC08002617.hg.1	2.94	PSR08017523.hg.1	TPD52
TC08002617.hg.1	2.92	PSR08017456.hg.1	TPD52
TC08002617.hg.1	2.87	PSR08017475.hg.1	TPD52
TC08002617.hg.1	2.8	PSR08017511.hg.1	TPD52
TC08002617.hg.1	2.76	PSR08017501.hg.1	TPD52
TC08002617.hg.1	2.72	PSR08017507.hg.1	TPD52
TC08002617.hg.1	2.72	PSR08017521.hg.1	TPD52
TC08002617.hg.1	2.72	JUC08015290.hg.1	TPD52
TC08002617.hg.1	2.68	PSR08017508.hg.1	TPD52
TC08002617.hg.1	2.57	PSR08017478.hg.1	TPD52
TC08002617.hg.1	2.55	PSR08017457.hg.1	TPD52
TC08002617.hg.1	2.55	PSR08017516.hg.1	TPD52
TC08002617.hg.1	2.41	PSR08017520.hg.1	TPD52
TC08002617.hg.1	2.35	PSR08017458.hg.1	TPD52
TC08002617.hg.1	2.35	PSR08017488.hg.1	TPD52
TC08002617.hg.1	2.31	PSR08017506.hg.1	TPD52
TC08002617.hg.1	2.25	PSR08017505.hg.1	TPD52
TC08002617.hg.1	2.24	JUC08015261.hg.1	TPD52
TC08002617.hg.1	2.22	PSR08017499.hg.1	TPD52
TC08002617.hg.1	2.09	PSR08017474.hg.1	TPD52
TC08002617.hg.1	2.07	PSR08017487.hg.1	TPD52
TC08002617.hg.1	2.04	PSR08017510.hg.1	TPD52
TC15000585.hg.1	12.49	JUC15002316.hg.1	FBXL22
TC15000585.hg.1	2.93	PSR15004731.hg.1	FBXL22
TC01003718.hg.1	12.2	PSR01057083.hg.1	CHI3L1
TC01003718.hg.1	7.71	PSR01057064.hg.1	CHI3L1
TC01003718.hg.1	2.71	PSR01057073.hg.1	CHI3L1
TC01003718.hg.1	2.57	PSR01057081.hg.1	CHI3L1
TC01003718.hg.1	2.42	PSR01057068.hg.1	CHI3L1
TC01003718.hg.1	-2.56	PSR01057067.hg.1	CHI3L1
TC01003718.hg.1	-2.67	JUC01029778.hg.1	CHI3L1
TC01003718.hg.1	-2.76	JUC01029775.hg.1	CHI3L1
TC01003718.hg.1	-3.06	PSR01057058.hg.1	CHI3L1
TC01003718.hg.1	-4	JUC01029780.hg.1	CHI3L1
TC06000087.hg.1	12.17	PSR06000920.hg.1	EDN1

TC06000087.hg.1	9.91	PSR06000919.hg.1	EDN1
TC06000087.hg.1	9.23	PSR06000926.hg.1	EDN1
TC06000087.hg.1	9.08	PSR06000929.hg.1	EDN1
TC06000087.hg.1	7.46	PSR06000921.hg.1	EDN1
TC06000087.hg.1	4.46	PSR06000925.hg.1	EDN1
TC06000087.hg.1	3.25	PSR06000932.hg.1	EDN1
TC06000087.hg.1	-2.95	JUC06000412.hg.1	EDN1
TC11002162.hg.1	12.06	PSR11025898.hg.1	SYTL2
TC11002162.hg.1	8.19	PSR11025902.hg.1	SYTL2
TC11002162.hg.1	7.55	PSR11025913.hg.1	SYTL2
TC11002162.hg.1	7.43	PSR11025895.hg.1	SYTL2
TC11002162.hg.1	7.2	PSR11025897.hg.1	SYTL2
TC11002162.hg.1	7.06	JUC11013740.hg.1	SYTL2
TC11002162.hg.1	6.87	PSR11025879.hg.1	SYTL2
TC11002162.hg.1	6.6	JUC11013751.hg.1	SYTL2
TC11002162.hg.1	6.05	JUC11013732.hg.1	SYTL2
TC11002162.hg.1	6.03	JUC11013730.hg.1	SYTL2
TC11002162.hg.1	5.96	PSR11025865.hg.1	SYTL2
TC11002162.hg.1	5.87	PSR11025896.hg.1	SYTL2
TC11002162.hg.1	5.53	JUC11013729.hg.1	SYTL2
TC11002162.hg.1	5.43	PSR11025912.hg.1	SYTL2
TC11002162.hg.1	5.22	JUC11013746.hg.1	SYTL2
TC11002162.hg.1	5.09	PSR11025910.hg.1	SYTL2
TC11002162.hg.1	5.04	JUC11013743.hg.1	SYTL2
TC11002162.hg.1	5.02	JUC11013750.hg.1	SYTL2
TC11002162.hg.1	4.82	PSR11025892.hg.1	SYTL2
TC11002162.hg.1	4.44	JUC11013747.hg.1	SYTL2
TC11002162.hg.1	4.29	PSR11025900.hg.1	SYTL2
TC11002162.hg.1	4.16	JUC11013753.hg.1	SYTL2
TC11002162.hg.1	4.11	PSR11025887.hg.1	SYTL2
TC11002162.hg.1	4.03	PSR11025907.hg.1	SYTL2
TC11002162.hg.1	3.72	PSR11025908.hg.1	SYTL2
TC11002162.hg.1	3.7	PSR11025903.hg.1	SYTL2
TC11002162.hg.1	3.69	PSR11025909.hg.1	SYTL2
TC11002162.hg.1	3.67	JUC11013756.hg.1	SYTL2
TC11002162.hg.1	3.64	PSR11025867.hg.1	SYTL2
TC11002162.hg.1	3.62	JUC11013734.hg.1	SYTL2
TC11002162.hg.1	3.52	PSR11025888.hg.1	SYTL2
TC11002162.hg.1	3.47	JUC11013749.hg.1	SYTL2
TC11002162.hg.1	3.27	JUC11013754.hg.1	SYTL2
TC11002162.hg.1	3.26	PSR11025906.hg.1	SYTL2
TC11002162.hg.1	3.07	PSR11025881.hg.1	SYTL2
TC11002162.hg.1	2.84	PSR11025904.hg.1	SYTL2
TC11002162.hg.1	2.73	JUC11013742.hg.1	SYTL2
TC11002162.hg.1	2.69	JUC11013733.hg.1	SYTL2
TC11002162.hg.1	2.59	JUC11013736.hg.1	SYTL2
TC11002162.hg.1	2.1	JUC11013738.hg.1	SYTL2
TC11002162.hg.1	-2.08	JUC11013744.hg.1	SYTL2
TC10002952.hg.1	12.02	JUC10017425.hg.1	ARHGAP19
TC10002952.hg.1	6.14	JUC10017418.hg.1	ARHGAP19
TC10002952.hg.1	-2.09	PSR10019899.hg.1	ARHGAP19
TC07001611.hg.1	11.96	JUC07012180.hg.1	TFPI2
TC07001611.hg.1	3.98	JUC07012179.hg.1	TFPI2
TC07001611.hg.1	3.69	PSR07024439.hg.1	TFPI2
TC07001611.hg.1	3.01	PSR07024432.hg.1	TFPI2
TC07001611.hg.1	2.79	PSR07024431.hg.1	TFPI2
TC07001611.hg.1	-2.25	PSR07024441.hg.1	TFPI2
TC07001611.hg.1	-2.72	PSR07024443.hg.1	TFPI2

TC15001219.hg.1	11.95	JUC15005791.hg.1	
TC15001219.hg.1	6.23	PSR15010956.hg.1	
TC15001219.hg.1	5.86	JUC15005790.hg.1	
TC15001219.hg.1	3.29	PSR15010954.hg.1	
TC15001219.hg.1	2.81	PSR15010957.hg.1	
TC15001219.hg.1	2.67	PSR15010955.hg.1	
TC15001219.hg.1	-2.22	PSR15010951.hg.1	
TC15001219.hg.1	-2.54	PSR15010952.hg.1	
TC15001219.hg.1	-2.98	PSR15010953.hg.1	
TC15001219.hg.1	-4.65	PSR15010958.hg.1	
TC15001219.hg.1	-13.7	JUC15005792.hg.1	
TC03001038.hg.1	11.92	JUC03009289.hg.1	LPP
TC03001038.hg.1	6.04	JUC03009315.hg.1	LPP
TC03001038.hg.1	4.18	JUC03009293.hg.1	LPP
TC03001038.hg.1	2.93	PSR03018388.hg.1	LPP
TC03001038.hg.1	2.9	PSR03018355.hg.1	LPP
TC03001038.hg.1	2.14	PSR03018394.hg.1	LPP
TC03001038.hg.1	-2.66	JUC03009297.hg.1	LPP
TC17000861.hg.1	11.9	JUC17006242.hg.1	ITGB4
TC17000861.hg.1	5.4	JUC17006231.hg.1	ITGB4
TC17000861.hg.1	4.28	JUC17006239.hg.1	ITGB4
TC17000861.hg.1	3.82	JUC17006237.hg.1	ITGB4
TC17000861.hg.1	3.23	PSR17011214.hg.1	ITGB4
TC17000861.hg.1	3.16	JUC17006233.hg.1	ITGB4
TC17000861.hg.1	2.99	JUC17006257.hg.1	ITGB4
TC17000861.hg.1	2.46	JUC17006240.hg.1	ITGB4
TC17000861.hg.1	2.13	JUC17006267.hg.1	ITGB4
TC17000861.hg.1	-2.09	JUC17006236.hg.1	ITGB4
TC01002075.hg.1	11.77	JUC01017039.hg.1	FAM41C
TC01002075.hg.1	2.45	PSR01031378.hg.1	FAM41C
TC6_cox_hap2000	11.75	JUC6_cox_hap20017	HLA-DPB1
TC6_cox_hap2000	5.68	PSR6_cox_hap20018	HLA-DPB1
TC6_cox_hap2000	5.57	JUC6_cox_hap20017	HLA-DPB1
TC6_cox_hap2000	5.26	PSR6_cox_hap20018	HLA-DPB1
TC6_cox_hap2000	4.97	JUC6_cox_hap20017	HLA-DPB1
TC6_cox_hap2000	4.53	PSR6_cox_hap20018	HLA-DPB1
TC6_cox_hap2000	4.34	PSR6_cox_hap20018	HLA-DPB1
TC6_cox_hap2000	3.76	PSR6_cox_hap20018	HLA-DPB1
TC6_cox_hap2000	3.11	PSR6_cox_hap20018	HLA-DPB1
TC6_cox_hap2000	2.72	JUC6_cox_hap20017	HLA-DPB1
TC6_qbl_hap60002	11.7	JUC6_qbl_hap60017	HLA-DPB1
TC6_qbl_hap60002	5.65	PSR6_qbl_hap60018	HLA-DPB1
TC6_qbl_hap60002	5.55	JUC6_qbl_hap60017	HLA-DPB1
TC6_qbl_hap60002	5.24	PSR6_qbl_hap60018	HLA-DPB1
TC6_qbl_hap60002	5.23	JUC6_qbl_hap60017	HLA-DPB1
TC6_qbl_hap60002	4.95	JUC6_qbl_hap60017	HLA-DPB1
TC6_qbl_hap60002	4.52	PSR6_qbl_hap60018	HLA-DPB1
TC6_qbl_hap60002	4.3	PSR6_qbl_hap60018	HLA-DPB1
TC6_qbl_hap60002	3.33	JUC6_qbl_hap60017	HLA-DPB1
TC6_qbl_hap60002	3.1	PSR6_qbl_hap60018	HLA-DPB1
TC6_qbl_hap60002	3.06	PSR6_qbl_hap60018	HLA-DPB1
TC6_qbl_hap60002	3	PSR6_qbl_hap60018	HLA-DPB1
TC6_qbl_hap60002	2.96	PSR6_qbl_hap60018	HLA-DPB1
TC6_qbl_hap60002	2.71	JUC6_qbl_hap60017	HLA-DPB1
TC6_qbl_hap60002	2.06	PSR6_qbl_hap60018	HLA-DPB1
TC6_qbl_hap60002	2.04	PSR6_qbl_hap60018	HLA-DPB1
TC6_qbl_hap60002	-2.35	JUC6_qbl_hap60017	HLA-DPB1
TC10000022.hg.1	11.67	JUC10000125.hg.1	PFKP

TC10000022.hg.1	7.77	JUC10000130.hg.1	PFKP
TC10000022.hg.1	6.94	PSR10000188.hg.1	PFKP
TC10000022.hg.1	5.62	PSR10000197.hg.1	PFKP
TC10000022.hg.1	5.25	PSR10000189.hg.1	PFKP
TC10000022.hg.1	5.05	PSR10000212.hg.1	PFKP
TC10000022.hg.1	4.99	PSR10000185.hg.1	PFKP
TC10000022.hg.1	4.47	JUC10000124.hg.1	PFKP
TC10000022.hg.1	4.38	PSR10000237.hg.1	PFKP
TC10000022.hg.1	4.34	PSR10000203.hg.1	PFKP
TC10000022.hg.1	4.03	JUC10000133.hg.1	PFKP
TC10000022.hg.1	3.88	PSR10000204.hg.1	PFKP
TC10000022.hg.1	3.83	JUC10000117.hg.1	PFKP
TC10000022.hg.1	3.81	PSR10000187.hg.1	PFKP
TC10000022.hg.1	3.61	JUC10000134.hg.1	PFKP
TC10000022.hg.1	3.6	PSR10000192.hg.1	PFKP
TC10000022.hg.1	3.55	PSR10000205.hg.1	PFKP
TC10000022.hg.1	2.86	PSR10000222.hg.1	PFKP
TC10000022.hg.1	2.78	PSR10000225.hg.1	PFKP
TC10000022.hg.1	2.61	PSR10000207.hg.1	PFKP
TC10000022.hg.1	2.41	PSR10000226.hg.1	PFKP
TC10000022.hg.1	2.07	PSR10000194.hg.1	PFKP
TC0Y000185.hg.1	11.65	JUC0Y001153.hg.1	KDM5D
TC0Y000185.hg.1	11.12	PSR0Y001862.hg.1	KDM5D
TC0Y000185.hg.1	9.66	JUC0Y001180.hg.1	KDM5D
TC0Y000185.hg.1	9.48	PSR0Y001860.hg.1	KDM5D
TC0Y000185.hg.1	6.88	JUC0Y001164.hg.1	KDM5D
TC0Y000185.hg.1	5.93	PSR0Y001818.hg.1	KDM5D
TC0Y000185.hg.1	5.34	PSR0Y001859.hg.1	KDM5D
TC0Y000185.hg.1	3.56	JUC0Y001158.hg.1	KDM5D
TC0Y000185.hg.1	3.44	PSR0Y001817.hg.1	KDM5D
TC0Y000185.hg.1	3.18	PSR0Y001816.hg.1	KDM5D
TC0Y000185.hg.1	2.07	PSR0Y001856.hg.1	KDM5D
TC0Y000185.hg.1	2.03	JUC0Y001186.hg.1	KDM5D
TC0Y000185.hg.1	2.01	PSR0Y001857.hg.1	KDM5D
TC0Y000185.hg.1	-2.06	PSR0Y001835.hg.1	KDM5D
TC0Y000185.hg.1	-2.12	PSR0Y001825.hg.1	KDM5D
TC0Y000185.hg.1	-2.34	PSR0Y001854.hg.1	KDM5D
TC0Y000185.hg.1	-2.34	JUC0Y001178.hg.1	KDM5D
TC0Y000185.hg.1	-2.41	JUC0Y001166.hg.1	KDM5D
TC0Y000185.hg.1	-2.58	PSR0Y001840.hg.1	KDM5D
TC0Y000185.hg.1	-2.71	PSR0Y001838.hg.1	KDM5D
TC0Y000185.hg.1	-2.94	PSR0Y001830.hg.1	KDM5D
TC0Y000185.hg.1	-2.98	JUC0Y001183.hg.1	KDM5D
TC0Y000185.hg.1	-3.01	PSR0Y001837.hg.1	KDM5D
TC0Y000185.hg.1	-3.15	JUC0Y001173.hg.1	KDM5D
TC0Y000185.hg.1	-3.4	JUC0Y001184.hg.1	KDM5D
TC0Y000185.hg.1	-3.43	PSR0Y001841.hg.1	KDM5D
TC0Y000185.hg.1	-3.49	PSR0Y001820.hg.1	KDM5D
TC0Y000185.hg.1	-3.56	JUC0Y001155.hg.1	KDM5D
TC0Y000185.hg.1	-3.57	PSR0Y001849.hg.1	KDM5D
TC0Y000185.hg.1	-3.68	JUC0Y001181.hg.1	KDM5D
TC0Y000185.hg.1	-3.85	PSR0Y001842.hg.1	KDM5D
TC0Y000185.hg.1	-3.97	PSR0Y001836.hg.1	KDM5D
TC0Y000185.hg.1	-4	PSR0Y001851.hg.1	KDM5D
TC0Y000185.hg.1	-4.14	JUC0Y001182.hg.1	KDM5D
TC0Y000185.hg.1	-4.47	PSR0Y001824.hg.1	KDM5D
TC0Y000185.hg.1	-4.59	JUC0Y001154.hg.1	KDM5D
TC0Y000185.hg.1	-4.78	PSR0Y001826.hg.1	KDM5D

TC0Y000185.hg.1	-5.01	JUC0Y001188.hg.1	KDM5D
TC0Y000185.hg.1	-6.18	JUC0Y001159.hg.1	KDM5D
TC0Y000185.hg.1	-6.88	JUC0Y001176.hg.1	KDM5D
TC0Y000185.hg.1	-7.57	JUC0Y001157.hg.1	KDM5D
TC0Y000185.hg.1	-7.69	JUC0Y001185.hg.1	KDM5D
TC6_dbb_hap3000	11.64	JUC6_dbb_hap3000(	HLA-DPB1
TC6_dbb_hap3000	5.63	PSR6_dbb_hap3001	HLA-DPB1
TC6_dbb_hap3000	5.53	JUC6_dbb_hap3000(	HLA-DPB1
TC6_dbb_hap3000	5.21	PSR6_dbb_hap3001	HLA-DPB1
TC6_dbb_hap3000	5.2	JUC6_dbb_hap3000(	HLA-DPB1
TC6_dbb_hap3000	4.92	JUC6_dbb_hap3000(	HLA-DPB1
TC6_dbb_hap3000	4.49	PSR6_dbb_hap3001	HLA-DPB1
TC6_dbb_hap3000	4.17	PSR6_dbb_hap3001	HLA-DPB1
TC6_dbb_hap3000	3.32	JUC6_dbb_hap3000(	HLA-DPB1
TC6_dbb_hap3000	3.09	PSR6_dbb_hap3001	HLA-DPB1
TC6_dbb_hap3000	3.05	PSR6_dbb_hap3001	HLA-DPB1
TC6_dbb_hap3000	2.98	PSR6_dbb_hap3001	HLA-DPB1
TC6_dbb_hap3000	2.94	PSR6_dbb_hap3001	HLA-DPB1
TC6_dbb_hap3000	2.7	JUC6_dbb_hap3000(	HLA-DPB1
TC6_dbb_hap3000	2.13	PSR6_dbb_hap3001	HLA-DPB1
TC6_dbb_hap3000	2.05	PSR6_dbb_hap3001	HLA-DPB1
TC6_dbb_hap3000	-2.36	JUC6_dbb_hap3000(	HLA-DPB1
TC6_ssto_hap700(	11.64	JUC6_ssto_hap7000	HLA-DPB1
TC6_ssto_hap700(	5.63	PSR6_ssto_hap7001	HLA-DPB1
TC6_ssto_hap700(	5.53	JUC6_ssto_hap7000	HLA-DPB1
TC6_ssto_hap700(	5.21	PSR6_ssto_hap7001	HLA-DPB1
TC6_ssto_hap700(	5.2	JUC6_ssto_hap7000	HLA-DPB1
TC6_ssto_hap700(	4.92	JUC6_ssto_hap7000	HLA-DPB1
TC6_ssto_hap700(	4.49	PSR6_ssto_hap7001	HLA-DPB1
TC6_ssto_hap700(	4.17	PSR6_ssto_hap7001	HLA-DPB1
TC6_ssto_hap700(	3.32	JUC6_ssto_hap7000	HLA-DPB1
TC6_ssto_hap700(	3.09	PSR6_ssto_hap7001	HLA-DPB1
TC6_ssto_hap700(	3.05	PSR6_ssto_hap7001	HLA-DPB1
TC6_ssto_hap700(	2.98	PSR6_ssto_hap7001	HLA-DPB1
TC6_ssto_hap700(	2.94	PSR6_ssto_hap7001	HLA-DPB1
TC6_ssto_hap700(	2.7	JUC6_ssto_hap7000	HLA-DPB1
TC6_ssto_hap700(	2.13	PSR6_ssto_hap7001	HLA-DPB1
TC6_ssto_hap700(	2.05	PSR6_ssto_hap7001	HLA-DPB1
TC6_ssto_hap700(	-2.36	JUC6_ssto_hap7000	HLA-DPB1
TC04000584.hg.1	11.59	JUC04004492.hg.1	ANK2
TC04000584.hg.1	9.78	JUC04004498.hg.1	ANK2
TC04000584.hg.1	7.48	JUC04004543.hg.1	ANK2
TC04000584.hg.1	7.22	JUC04004513.hg.1	ANK2
TC04000584.hg.1	6.36	PSR04008544.hg.1	ANK2
TC04000584.hg.1	6.16	JUC04004558.hg.1	ANK2
TC04000584.hg.1	5.83	JUC04004509.hg.1	ANK2
TC04000584.hg.1	5.38	JUC04004552.hg.1	ANK2
TC04000584.hg.1	5.25	JUC04004544.hg.1	ANK2
TC04000584.hg.1	5.19	JUC04004522.hg.1	ANK2
TC04000584.hg.1	5.13	PSR04008543.hg.1	ANK2
TC04000584.hg.1	5.06	PSR04008553.hg.1	ANK2
TC04000584.hg.1	4.95	PSR04008549.hg.1	ANK2
TC04000584.hg.1	4.88	JUC04004551.hg.1	ANK2
TC04000584.hg.1	4.77	PSR04008561.hg.1	ANK2
TC04000584.hg.1	4.76	PSR04008559.hg.1	ANK2
TC04000584.hg.1	4.64	JUC04004508.hg.1	ANK2
TC04000584.hg.1	4.39	PSR04008532.hg.1	ANK2
TC04000584.hg.1	4.17	PSR04008541.hg.1	ANK2

TC04000584.hg.1	4.05	PSR04008546.hg.1	ANK2
TC04000584.hg.1	3.88	JUC04004531.hg.1	ANK2
TC04000584.hg.1	3.87	JUC04004521.hg.1	ANK2
TC04000584.hg.1	3.82	JUC04004512.hg.1	ANK2
TC04000584.hg.1	3.76	PSR04008555.hg.1	ANK2
TC04000584.hg.1	3.69	PSR04008547.hg.1	ANK2
TC04000584.hg.1	3.64	PSR04008552.hg.1	ANK2
TC04000584.hg.1	3.62	PSR04008557.hg.1	ANK2
TC04000584.hg.1	3.54	PSR04008560.hg.1	ANK2
TC04000584.hg.1	3.43	PSR04008551.hg.1	ANK2
TC04000584.hg.1	3.38	JUC04004519.hg.1	ANK2
TC04000584.hg.1	3.33	PSR04008554.hg.1	ANK2
TC04000584.hg.1	3.09	PSR04008550.hg.1	ANK2
TC04000584.hg.1	3.05	PSR04008526.hg.1	ANK2
TC04000584.hg.1	3	PSR04008542.hg.1	ANK2
TC04000584.hg.1	2.88	JUC04004538.hg.1	ANK2
TC04000584.hg.1	2.78	PSR04008558.hg.1	ANK2
TC04000584.hg.1	2.76	JUC04004523.hg.1	ANK2
TC04000584.hg.1	2.65	PSR04008556.hg.1	ANK2
TC04000584.hg.1	2.45	JUC04004532.hg.1	ANK2
TC04000584.hg.1	2.4	JUC04004524.hg.1	ANK2
TC04000584.hg.1	-2.07	PSR04008595.hg.1	ANK2
TC04000584.hg.1	-2.18	PSR04008618.hg.1	ANK2
TC04000584.hg.1	-2.87	JUC04004496.hg.1	ANK2
TC6_mann_hap40	11.56	JUC6_mann_hap400	HLA-DPB1
TC6_mann_hap40	5.59	PSR6_mann_hap40	HLA-DPB1
TC6_mann_hap40	5.48	JUC6_mann_hap400	HLA-DPB1
TC6_mann_hap40	5.17	PSR6_mann_hap40	HLA-DPB1
TC6_mann_hap40	5.17	JUC6_mann_hap400	HLA-DPB1
TC6_mann_hap40	4.89	JUC6_mann_hap400	HLA-DPB1
TC6_mann_hap40	4.46	PSR6_mann_hap40	HLA-DPB1
TC6_mann_hap40	4.24	PSR6_mann_hap40	HLA-DPB1
TC6_mann_hap40	3.29	JUC6_mann_hap400	HLA-DPB1
TC6_mann_hap40	3.06	PSR6_mann_hap40	HLA-DPB1
TC6_mann_hap40	3.02	PSR6_mann_hap40	HLA-DPB1
TC6_mann_hap40	2.96	PSR6_mann_hap40	HLA-DPB1
TC6_mann_hap40	2.92	PSR6_mann_hap40	HLA-DPB1
TC6_mann_hap40	2.68	JUC6_mann_hap400	HLA-DPB1
TC6_mann_hap40	2.39	PSR6_mann_hap40	HLA-DPB1
TC6_mann_hap40	2.04	PSR6_mann_hap40	HLA-DPB1
TC6_mann_hap40	-2.38	JUC6_mann_hap400	HLA-DPB1
TC6_apd_hap1000	11.51	JUC6_apd_hap1000	HLA-DPB1
TC6_apd_hap1000	5.56	PSR6_apd_hap1000	HLA-DPB1
TC6_apd_hap1000	5.46	JUC6_apd_hap1000	HLA-DPB1
TC6_apd_hap1000	5.15	PSR6_apd_hap1000	HLA-DPB1
TC6_apd_hap1000	5.15	JUC6_apd_hap1000	HLA-DPB1
TC6_apd_hap1000	4.87	JUC6_apd_hap1000	HLA-DPB1
TC6_apd_hap1000	4.44	PSR6_apd_hap1000	HLA-DPB1
TC6_apd_hap1000	4.23	PSR6_apd_hap1000	HLA-DPB1
TC6_apd_hap1000	3.28	JUC6_apd_hap1000	HLA-DPB1
TC6_apd_hap1000	3.05	PSR6_apd_hap1000	HLA-DPB1
TC6_apd_hap1000	3.01	PSR6_apd_hap1000	HLA-DPB1
TC6_apd_hap1000	2.95	PSR6_apd_hap1000	HLA-DPB1
TC6_apd_hap1000	2.91	PSR6_apd_hap1000	HLA-DPB1
TC6_apd_hap1000	2.67	JUC6_apd_hap1000	HLA-DPB1
TC6_apd_hap1000	2.56	PSR6_apd_hap1000	HLA-DPB1
TC6_apd_hap1000	2.03	PSR6_apd_hap1000	HLA-DPB1
TC6_apd_hap1000	-2.39	JUC6_apd_hap1000	HLA-DPB1

TC6_mcf_hap5000	11.51	JUC6_mcf_hap5001	HLA-DPB1
TC6_mcf_hap5000	5.56	PSR6_mcf_hap5001	HLA-DPB1
TC6_mcf_hap5000	5.46	JUC6_mcf_hap5001	HLA-DPB1
TC6_mcf_hap5000	5.15	PSR6_mcf_hap5001	HLA-DPB1
TC6_mcf_hap5000	5.15	JUC6_mcf_hap5001	HLA-DPB1
TC6_mcf_hap5000	4.87	JUC6_mcf_hap5001	HLA-DPB1
TC6_mcf_hap5000	4.44	PSR6_mcf_hap5001	HLA-DPB1
TC6_mcf_hap5000	4.23	PSR6_mcf_hap5001	HLA-DPB1
TC6_mcf_hap5000	3.28	JUC6_mcf_hap5001	HLA-DPB1
TC6_mcf_hap5000	3.05	PSR6_mcf_hap5001	HLA-DPB1
TC6_mcf_hap5000	3.01	PSR6_mcf_hap5001	HLA-DPB1
TC6_mcf_hap5000	2.95	PSR6_mcf_hap5001	HLA-DPB1
TC6_mcf_hap5000	2.91	PSR6_mcf_hap5001	HLA-DPB1
TC6_mcf_hap5000	2.67	JUC6_mcf_hap5001	HLA-DPB1
TC6_mcf_hap5000	2.56	PSR6_mcf_hap5001	HLA-DPB1
TC6_mcf_hap5000	2.03	PSR6_mcf_hap5001	HLA-DPB1
TC6_mcf_hap5000	-2.39	JUC6_mcf_hap5001	HLA-DPB1
TC14000371.hg.1	11.39	JUC14002036.hg.1	PRKCH
TC14000371.hg.1	2.45	PSR14004091.hg.1	PRKCH
TC01006414.hg.1	11.35	PSR01053860.hg.1	RGS5
TC01006414.hg.1	10.95	JUC01046795.hg.1	RGS5
TC01006414.hg.1	5.7	PSR01053864.hg.1	RGS5
TC01006414.hg.1	5.32	JUC01046803.hg.1	RGS5
TC01006414.hg.1	3.84	PSR01053854.hg.1	RGS5
TC01006414.hg.1	3.01	PSR01053857.hg.1	RGS5
TC01006414.hg.1	2.96	JUC01046799.hg.1	RGS5
TC01006414.hg.1	2.28	PSR01053861.hg.1	RGS5
TC01006414.hg.1	-2.19	PSR01053852.hg.1	RGS5
TC16000099.hg.1	11.33	JUC16000854.hg.1	IL32
TC16000099.hg.1	7.86	PSR16001819.hg.1	IL32
TC16000099.hg.1	6.01	PSR16001753.hg.1	IL32
TC16000099.hg.1	5.71	PSR16001820.hg.1	IL32
TC16000099.hg.1	5.02	PSR16001806.hg.1	IL32
TC16000099.hg.1	4.8	PSR16001804.hg.1	IL32
TC16000099.hg.1	4.74	PSR16001834.hg.1	IL32
TC16000099.hg.1	4.67	PSR16001801.hg.1	IL32
TC16000099.hg.1	4.55	PSR16001759.hg.1	IL32
TC16000099.hg.1	4.38	PSR16001810.hg.1	IL32
TC16000099.hg.1	4.33	JUC16000855.hg.1	IL32
TC16000099.hg.1	4.07	PSR16001780.hg.1	IL32
TC16000099.hg.1	3.94	PSR16001835.hg.1	IL32
TC16000099.hg.1	3.25	JUC16000858.hg.1	IL32
TC16000099.hg.1	3.18	PSR16001831.hg.1	IL32
TC16000099.hg.1	3.11	JUC16000850.hg.1	IL32
TC16000099.hg.1	3.1	PSR16001798.hg.1	IL32
TC16000099.hg.1	3.05	PSR16001800.hg.1	IL32
TC16000099.hg.1	2.56	PSR16001760.hg.1	IL32
TC16000099.hg.1	2.34	JUC16000867.hg.1	IL32
TC16000099.hg.1	2.16	JUC16000849.hg.1	IL32
TC08000700.hg.1	11.22	PSR08009448.hg.1	COL14A1
TC08000700.hg.1	10.86	JUC08004851.hg.1	COL14A1
TC08000700.hg.1	8.88	PSR08009444.hg.1	COL14A1
TC08000700.hg.1	8.26	PSR08009445.hg.1	COL14A1
TC08000700.hg.1	6.33	PSR08009467.hg.1	COL14A1
TC08000700.hg.1	5.14	JUC08004872.hg.1	COL14A1
TC08000700.hg.1	4.09	JUC08004864.hg.1	COL14A1
TC08000700.hg.1	3.7	JUC08004871.hg.1	COL14A1
TC08000700.hg.1	3.1	JUC08004823.hg.1	COL14A1

TC08000700.hg.1	2.22	JUC08004833.hg.1	COL14A1
TC08000700.hg.1	-2.39	PSR08009513.hg.1	COL14A1
TC08000700.hg.1	-2.62	JUC08004845.hg.1	COL14A1
TC08000700.hg.1	-2.78	JUC08004856.hg.1	COL14A1
TC08000700.hg.1	-3.27	PSR08009485.hg.1	COL14A1
TC08000700.hg.1	-8.26	JUC08004854.hg.1	COL14A1
TC10001699.hg.1	11.21	PSR10022383.hg.1	EIF3A, SNOR
TC10001699.hg.1	2.23	JUC10012980.hg.1	EIF3A, SNOR
TC09001092.hg.1	11.16	JUC09007810.hg.1	CNTNAP3
TC09001092.hg.1	3.24	JUC09007819.hg.1	CNTNAP3
TC09001092.hg.1	2.89	JUC09007841.hg.1	CNTNAP3
TC09001092.hg.1	2.73	JUC09007812.hg.1	CNTNAP3
TC09001092.hg.1	2.72	JUC09007820.hg.1	CNTNAP3
TC09001092.hg.1	2.71	PSR09014558.hg.1	CNTNAP3
TC09001092.hg.1	2.67	JUC09007827.hg.1	CNTNAP3
TC09001092.hg.1	2.6	PSR09014553.hg.1	CNTNAP3
TC09001092.hg.1	2.31	JUC09007831.hg.1	CNTNAP3
TC09001092.hg.1	2.12	PSR09014592.hg.1	CNTNAP3
TC09001092.hg.1	2.09	PSR09014609.hg.1	CNTNAP3
TC09001092.hg.1	2.03	PSR09014557.hg.1	CNTNAP3
TC09001092.hg.1	2.02	JUC09007813.hg.1	CNTNAP3
TC09001092.hg.1	2	PSR09014585.hg.1	CNTNAP3
TC09001092.hg.1	-2.44	JUC09007815.hg.1	CNTNAP3
TC09001092.hg.1	-2.6	PSR09014584.hg.1	CNTNAP3
TC09001092.hg.1	-2.63	JUC09007826.hg.1	CNTNAP3
TC09001092.hg.1	-2.98	JUC09007834.hg.1	CNTNAP3
TC09001092.hg.1	-3.3	PSR09014598.hg.1	CNTNAP3
TC09001092.hg.1	-3.76	JUC09007836.hg.1	CNTNAP3
TC09001092.hg.1	-5.93	JUC09007817.hg.1	CNTNAP3
TC09001092.hg.1	-6.41	JUC09007840.hg.1	CNTNAP3
TC01006284.hg.1	11.11	JUC01039220.hg.1	LOC1002890
TC01006284.hg.1	10.04	JUC01039205.hg.1	LOC1002890
TC01006284.hg.1	8.31	JUC01039217.hg.1	LOC1002890
TC01006284.hg.1	5.85	JUC01039218.hg.1	LOC1002890
TC01006284.hg.1	5.02	JUC01039213.hg.1	LOC1002890
TC01006284.hg.1	4.49	PSR01016703.hg.1	LOC1002890
TC01006284.hg.1	3.91	PSR01016696.hg.1	LOC1002890
TC01006284.hg.1	3.24	PSR01016698.hg.1	LOC1002890
TC01006284.hg.1	3.21	PSR01016697.hg.1	LOC1002890
TC01006284.hg.1	3.14	PSR01016701.hg.1	LOC1002890
TC01006284.hg.1	3.07	PSR01016694.hg.1	LOC1002890
TC01006284.hg.1	2.99	PSR01016693.hg.1	LOC1002890
TC01006284.hg.1	2.73	JUC01039216.hg.1	LOC1002890
TC01006284.hg.1	2.54	PSR01016685.hg.1	LOC1002890
TC01006284.hg.1	2.21	PSR01016687.hg.1	LOC1002890
TC01006284.hg.1	2.21	PSR01016695.hg.1	LOC1002890
TC01006284.hg.1	2.07	PSR01016690.hg.1	LOC1002890
TC03000013.hg.1	11.09	PSR03000336.hg.1	ITPR1
TC03000013.hg.1	8.48	PSR03000269.hg.1	ITPR1
TC03000013.hg.1	7.03	JUC03000164.hg.1	ITPR1
TC03000013.hg.1	6.93	JUC03000200.hg.1	ITPR1
TC03000013.hg.1	6.26	JUC03000191.hg.1	ITPR1
TC03000013.hg.1	6.06	PSR03000355.hg.1	ITPR1
TC03000013.hg.1	5.88	PSR03000259.hg.1	ITPR1
TC03000013.hg.1	5.72	JUC03000182.hg.1	ITPR1
TC03000013.hg.1	5.6	JUC03000174.hg.1	ITPR1
TC03000013.hg.1	5.19	JUC03000179.hg.1	ITPR1
TC03000013.hg.1	5.1	PSR03000285.hg.1	ITPR1

TC03000013.hg.1	5.08	PSR03000332.hg.1	ITPR1
TC03000013.hg.1	5.02	PSR03000335.hg.1	ITPR1
TC03000013.hg.1	4.91	PSR03000327.hg.1	ITPR1
TC03000013.hg.1	4.69	PSR03000356.hg.1	ITPR1
TC03000013.hg.1	4.55	PSR03000345.hg.1	ITPR1
TC03000013.hg.1	4.36	JUC03000210.hg.1	ITPR1
TC03000013.hg.1	4.29	JUC03000152.hg.1	ITPR1
TC03000013.hg.1	4.13	PSR03000314.hg.1	ITPR1
TC03000013.hg.1	3.43	JUC03000180.hg.1	ITPR1
TC03000013.hg.1	3.32	PSR03000342.hg.1	ITPR1
TC03000013.hg.1	3.23	JUC03000216.hg.1	ITPR1
TC03000013.hg.1	3.01	JUC03000186.hg.1	ITPR1
TC03000013.hg.1	2.78	PSR03000312.hg.1	ITPR1
TC03000013.hg.1	2.77	JUC03000176.hg.1	ITPR1
TC03000013.hg.1	2.75	JUC03000148.hg.1	ITPR1
TC03000013.hg.1	2.74	JUC03000166.hg.1	ITPR1
TC03000013.hg.1	2.7	PSR03000274.hg.1	ITPR1
TC03000013.hg.1	2.53	PSR03000307.hg.1	ITPR1
TC03000013.hg.1	2.53	JUC03000212.hg.1	ITPR1
TC03000013.hg.1	2.43	PSR03000295.hg.1	ITPR1
TC03000013.hg.1	2.35	PSR03000354.hg.1	ITPR1
TC03000013.hg.1	2.29	JUC03000190.hg.1	ITPR1
TC03000013.hg.1	2.25	JUC03000208.hg.1	ITPR1
TC03000013.hg.1	2.22	JUC03000155.hg.1	ITPR1
TC03000013.hg.1	2.15	PSR03000311.hg.1	ITPR1
TC03000013.hg.1	2.14	PSR03000258.hg.1	ITPR1
TC03000013.hg.1	2.13	PSR03000337.hg.1	ITPR1
TC03000013.hg.1	-2.05	JUC03000196.hg.1	ITPR1
TC03000013.hg.1	-2.18	PSR03000346.hg.1	ITPR1
TC03000013.hg.1	-2.4	JUC03000163.hg.1	ITPR1
TC03000013.hg.1	-2.83	JUC03000202.hg.1	ITPR1
TC03000013.hg.1	-2.99	JUC03000170.hg.1	ITPR1
TC01000054.hg.1	10.77	JUC01000374.hg.1	PRKCZ
TC01000054.hg.1	3.66	PSR01000795.hg.1	PRKCZ
TC01000054.hg.1	3.55	PSR01000759.hg.1	PRKCZ
TC01000054.hg.1	3.51	JUC01000404.hg.1	PRKCZ
TC01000054.hg.1	3.5	PSR01000768.hg.1	PRKCZ
TC01000054.hg.1	3.49	PSR01000798.hg.1	PRKCZ
TC01000054.hg.1	3.47	JUC01000378.hg.1	PRKCZ
TC01000054.hg.1	3.41	PSR01000758.hg.1	PRKCZ
TC01000054.hg.1	3.39	PSR01000769.hg.1	PRKCZ
TC01000054.hg.1	3.33	PSR01000748.hg.1	PRKCZ
TC01000054.hg.1	3.24	JUC01000390.hg.1	PRKCZ
TC01000054.hg.1	3.21	PSR01000752.hg.1	PRKCZ
TC01000054.hg.1	3.12	PSR01000760.hg.1	PRKCZ
TC01000054.hg.1	3.04	PSR01000779.hg.1	PRKCZ
TC01000054.hg.1	3.01	PSR01000767.hg.1	PRKCZ
TC01000054.hg.1	2.9	PSR01000753.hg.1	PRKCZ
TC01000054.hg.1	2.85	PSR01000757.hg.1	PRKCZ
TC01000054.hg.1	2.79	PSR01000751.hg.1	PRKCZ
TC01000054.hg.1	2.6	PSR01000806.hg.1	PRKCZ
TC01000054.hg.1	2.59	PSR01000754.hg.1	PRKCZ
TC01000054.hg.1	2.52	JUC01000405.hg.1	PRKCZ
TC01000054.hg.1	2.5	PSR01000796.hg.1	PRKCZ
TC01000054.hg.1	2.44	PSR01000749.hg.1	PRKCZ
TC01000054.hg.1	2.42	PSR01000761.hg.1	PRKCZ
TC01000054.hg.1	2.36	PSR01000756.hg.1	PRKCZ
TC01000054.hg.1	2.35	PSR01000811.hg.1	PRKCZ

TC01000054.hg.1	2.29	PSR01000772.hg.1	PRKCZ
TC01000054.hg.1	2.24	PSR01000809.hg.1	PRKCZ
TC01000054.hg.1	2.2	PSR01000799.hg.1	PRKCZ
TC01000054.hg.1	2.2	PSR01000800.hg.1	PRKCZ
TC01000054.hg.1	2.15	PSR01000805.hg.1	PRKCZ
TC01000054.hg.1	2.15	JUC01000409.hg.1	PRKCZ
TC01000054.hg.1	2.13	PSR01000810.hg.1	PRKCZ
TC01000054.hg.1	2.12	PSR01000808.hg.1	PRKCZ
TC01000054.hg.1	2.09	PSR01000755.hg.1	PRKCZ
TC01000054.hg.1	2.08	PSR01000807.hg.1	PRKCZ
TC01000054.hg.1	2.03	PSR01000765.hg.1	PRKCZ
TC01000054.hg.1	2	PSR01000771.hg.1	PRKCZ
TC6_mann_hap40(	10.75	JUC6_mann_hap400	CFB
TC6_mann_hap40(	9.61	PSR6_mann_hap40(	CFB
TC6_mann_hap40(	8.92	PSR6_mann_hap40(	CFB
TC6_mann_hap40(	8.65	PSR6_mann_hap40(	CFB
TC6_mann_hap40(	8.14	PSR6_mann_hap40(	CFB
TC6_mann_hap40(	7.14	PSR6_mann_hap40(	CFB
TC6_mann_hap40(	5.03	PSR6_mann_hap40(	CFB
TC6_mann_hap40(	4.47	PSR6_mann_hap40(	CFB
TC6_mann_hap40(	3.77	PSR6_mann_hap40(	CFB
TC6_mann_hap40(	3.76	PSR6_mann_hap40(	CFB
TC6_mann_hap40(	3.56	PSR6_mann_hap40(	CFB
TC6_mann_hap40(	2.62	PSR6_mann_hap40(	CFB
TC6_mann_hap40(	2.55	PSR6_mann_hap40(	CFB
TC6_mann_hap40(	2.25	PSR6_mann_hap40(	CFB
TC6_mann_hap40(	2.2	PSR6_mann_hap40(	CFB
TC6_mann_hap40(	-2.4	JUC6_mann_hap400	CFB
TC6_mann_hap40(	-2.42	JUC6_mann_hap400	CFB
TC6_mann_hap40(	-2.66	JUC6_mann_hap400	CFB
TC08000250.hg.1	10.74	JUC08001743.hg.1	NRG1
TC08000250.hg.1	5.63	JUC08001769.hg.1	NRG1
TC08000250.hg.1	5.11	PSR08003572.hg.1	NRG1
TC08000250.hg.1	3.44	JUC08001752.hg.1	NRG1
TC08000250.hg.1	3.33	PSR08003601.hg.1	NRG1
TC08000250.hg.1	2.87	PSR08003602.hg.1	NRG1
TC08000250.hg.1	2.61	PSR08003577.hg.1	NRG1
TC08000250.hg.1	2.4	JUC08001760.hg.1	NRG1
TC08000250.hg.1	2.37	JUC08001754.hg.1	NRG1
TC08000250.hg.1	2.22	PSR08003576.hg.1	NRG1
TC08000250.hg.1	2.06	JUC08001758.hg.1	NRG1
TC08000250.hg.1	-2.82	JUC08001742.hg.1	NRG1
TC08000250.hg.1	-2.83	PSR08003562.hg.1	NRG1
TC08000250.hg.1	-2.99	PSR08003597.hg.1	NRG1
TC08000250.hg.1	-3.8	PSR08003569.hg.1	NRG1
TC08000250.hg.1	-3.9	PSR08003567.hg.1	NRG1
TC08000250.hg.1	-4.13	PSR08003551.hg.1	NRG1
TC08000250.hg.1	-4.15	PSR08003565.hg.1	NRG1
TC08000250.hg.1	-4.28	PSR08003553.hg.1	NRG1
TC08000250.hg.1	-4.56	PSR08003549.hg.1	NRG1
TC08000250.hg.1	-4.76	JUC08001759.hg.1	NRG1
TC08000250.hg.1	-4.78	PSR08003563.hg.1	NRG1
TC08000250.hg.1	-5.04	PSR08003561.hg.1	NRG1
TC08000250.hg.1	-5.11	PSR08003568.hg.1	NRG1
TC08000250.hg.1	-5.18	PSR08003566.hg.1	NRG1
TC08000250.hg.1	-5.41	PSR08003598.hg.1	NRG1
TC08000250.hg.1	-5.7	PSR08003547.hg.1	NRG1
TC08000250.hg.1	-6.14	PSR08003550.hg.1	NRG1

TC08000250.hg.1	-6.57	JUC08001756.hg.1	NRG1
TC08000250.hg.1	-6.9	PSR08003559.hg.1	NRG1
TC08000250.hg.1	-7.1	PSR08003552.hg.1	NRG1
TC08000250.hg.1	-7.17	JUC08001767.hg.1	NRG1
TC08000250.hg.1	-7.22	PSR08003564.hg.1	NRG1
TC08000250.hg.1	-7.32	PSR08003560.hg.1	NRG1
TC08000250.hg.1	-7.36	PSR08003557.hg.1	NRG1
TC08000250.hg.1	-7.85	PSR08003556.hg.1	NRG1
TC08000250.hg.1	-8.52	JUC08001746.hg.1	NRG1
TC08000250.hg.1	-14.8	JUC08001762.hg.1	NRG1
TC02000466.hg.1	10.7	JUC02003726.hg.1	HK2
TC02000466.hg.1	5.79	PSR02007337.hg.1	HK2
TC02000466.hg.1	5.08	PSR02007342.hg.1	HK2
TC02000466.hg.1	4.86	PSR02007346.hg.1	HK2
TC02000466.hg.1	4.19	PSR02007353.hg.1	HK2
TC02000466.hg.1	4.05	JUC02003722.hg.1	HK2
TC02000466.hg.1	3.15	PSR02007354.hg.1	HK2
TC02000466.hg.1	3.02	JUC02003716.hg.1	HK2
TC02000466.hg.1	2.77	JUC02003719.hg.1	HK2
TC02000466.hg.1	2.26	PSR02007367.hg.1	HK2
TC02000466.hg.1	2.17	PSR02007366.hg.1	HK2
TC02000466.hg.1	2.06	JUC02003723.hg.1	HK2
TC02000466.hg.1	2	JUC02003727.hg.1	HK2
TC02000466.hg.1	-2	PSR02007349.hg.1	HK2
TC02000466.hg.1	-2.09	JUC02003731.hg.1	HK2
TC02000466.hg.1	-2.16	PSR02007359.hg.1	HK2
TC02000466.hg.1	-2.63	PSR02007357.hg.1	HK2
TC02000466.hg.1	-3.97	JUC02003734.hg.1	HK2
TC02000466.hg.1	-4.54	JUC02003720.hg.1	HK2
TC19001103.hg.1	10.7	PSR19015185.hg.1	C3
TC19001103.hg.1	9.07	PSR19015175.hg.1	C3
TC19001103.hg.1	8.9	PSR19015164.hg.1	C3
TC19001103.hg.1	8.29	PSR19015139.hg.1	C3
TC19001103.hg.1	7.97	PSR19015126.hg.1	C3
TC19001103.hg.1	7.61	PSR19015184.hg.1	C3
TC19001103.hg.1	6.65	PSR19015130.hg.1	C3
TC19001103.hg.1	6.53	PSR19015157.hg.1	C3
TC19001103.hg.1	6.32	PSR19015177.hg.1	C3
TC19001103.hg.1	6.12	PSR19015151.hg.1	C3
TC19001103.hg.1	6.03	PSR19015186.hg.1	C3
TC19001103.hg.1	5.08	JUC19008614.hg.1	C3
TC19001103.hg.1	4.71	PSR19015159.hg.1	C3
TC19001103.hg.1	4.14	JUC19008609.hg.1	C3
TC19001103.hg.1	4.08	JUC19008607.hg.1	C3
TC19001103.hg.1	3.01	JUC19008618.hg.1	C3
TC19001103.hg.1	2.51	JUC19008621.hg.1	C3
TC19001103.hg.1	2.46	PSR19015160.hg.1	C3
TC19001103.hg.1	2.33	PSR19015166.hg.1	C3
TC19001103.hg.1	2.27	PSR19015145.hg.1	C3
TC19001103.hg.1	2.26	PSR19015181.hg.1	C3
TC19001103.hg.1	2.18	PSR19015163.hg.1	C3
TC19001103.hg.1	2.1	JUC19008590.hg.1	C3
TC19001103.hg.1	2.01	JUC19008581.hg.1	C3
TC19001103.hg.1	-2.01	PSR19015174.hg.1	C3
TC19001103.hg.1	-2.02	JUC19008583.hg.1	C3
TC19001103.hg.1	-2.06	PSR19015135.hg.1	C3
TC19001103.hg.1	-2.08	JUC19008591.hg.1	C3
TC19001103.hg.1	-2.12	JUC19008617.hg.1	C3

TC19001103.hg.1	-2.16	PSR19015127.hg.1	C3
TC19001103.hg.1	-2.26	PSR19015176.hg.1	C3
TC19001103.hg.1	-2.45	PSR19015153.hg.1	C3
TC19001103.hg.1	-2.48	JUC19008595.hg.1	C3
TC19001103.hg.1	-2.61	PSR19015128.hg.1	C3
TC19001103.hg.1	-2.91	JUC19008615.hg.1	C3
TC19001103.hg.1	-3.03	JUC19008603.hg.1	C3
TC19001103.hg.1	-3.41	JUC19008620.hg.1	C3
TC19001103.hg.1	-5.99	JUC19008588.hg.1	C3
TC22000583.hg.1	10.68	JUC22004128.hg.1	GSTT1
TC22000583.hg.1	9.18	JUC22004121.hg.1	GSTT1
TC22000583.hg.1	8.63	JUC22004129.hg.1	GSTT1
TC22000583.hg.1	8.51	PSR22010293.hg.1	GSTT1
TC22000583.hg.1	8.2	PSR22010306.hg.1	GSTT1
TC22000583.hg.1	8.13	JUC22004120.hg.1	GSTT1
TC22000583.hg.1	6.68	PSR22010294.hg.1	GSTT1
TC22000583.hg.1	6.33	PSR22010295.hg.1	GSTT1
TC22000583.hg.1	6.3	PSR22010289.hg.1	GSTT1
TC22000583.hg.1	5.31	JUC22004127.hg.1	GSTT1
TC22000583.hg.1	5.26	PSR22010296.hg.1	GSTT1
TC22000583.hg.1	5.03	PSR22010288.hg.1	GSTT1
TC22000583.hg.1	4.73	PSR22010292.hg.1	GSTT1
TC22000583.hg.1	4.05	PSR22010280.hg.1	GSTT1
TC22000583.hg.1	3.09	JUC22004126.hg.1	GSTT1
TC22000583.hg.1	-2.85	JUC22004133.hg.1	GSTT1
TC12000516.hg.1	10.64	JUC12003519.hg.1	HSD17B6
TC12000516.hg.1	4.75	PSR12006776.hg.1	HSD17B6
TC12000516.hg.1	3.45	PSR12006777.hg.1	HSD17B6
TC12000516.hg.1	-2.45	JUC12003516.hg.1	HSD17B6
TC12000516.hg.1	-2.47	PSR12006763.hg.1	HSD17B6
TC12000516.hg.1	-2.94	PSR12006771.hg.1	HSD17B6
TC01001792.hg.1	10.6	PSR01028033.hg.1	KCNK2
TC01001792.hg.1	9.39	JUC01015113.hg.1	KCNK2
TC01001792.hg.1	9.24	PSR01028031.hg.1	KCNK2
TC01001792.hg.1	8.36	JUC01015106.hg.1	KCNK2
TC01001792.hg.1	6.65	PSR01028038.hg.1	KCNK2
TC01001792.hg.1	6.56	JUC01015105.hg.1	KCNK2
TC01001792.hg.1	6.25	PSR01028030.hg.1	KCNK2
TC01001792.hg.1	5.7	JUC01015110.hg.1	KCNK2
TC01001792.hg.1	5.42	PSR01028041.hg.1	KCNK2
TC01001792.hg.1	3.14	JUC01015102.hg.1	KCNK2
TC01001792.hg.1	2.9	PSR01028061.hg.1	KCNK2
TC01001792.hg.1	2.87	JUC01015114.hg.1	KCNK2
TC01001792.hg.1	2.61	PSR01028040.hg.1	KCNK2
TC01001792.hg.1	2.45	PSR01028044.hg.1	KCNK2
TC01001792.hg.1	-2.04	PSR01028057.hg.1	KCNK2
TC01001792.hg.1	-2.55	PSR01028059.hg.1	KCNK2
TC01001792.hg.1	-3.22	JUC01015112.hg.1	KCNK2
TC06002020.hg.1	10.53	PSR06025200.hg.1	TRAF3IP2
TC06002020.hg.1	3.2	PSR06025203.hg.1	TRAF3IP2
TC06002020.hg.1	2.97	PSR06025199.hg.1	TRAF3IP2
TC06002020.hg.1	2.53	PSR06025204.hg.1	TRAF3IP2
TC06002020.hg.1	2.41	PSR06025207.hg.1	TRAF3IP2
TC06002020.hg.1	2.36	PSR06025197.hg.1	TRAF3IP2
TC06002020.hg.1	2.16	PSR06025205.hg.1	TRAF3IP2
TC06002020.hg.1	2.07	JUC06012426.hg.1	TRAF3IP2
TC06002020.hg.1	2.06	PSR06025209.hg.1	TRAF3IP2
TC05003409.hg.1	10.42	JUC05019376.hg.1	SH3TC2

TC05003409.hg.1	6.38	PSR05026503.hg.1	SH3TC2
TC05003409.hg.1	3.69	JUC05019388.hg.1	SH3TC2
TC05003409.hg.1	2.31	JUC05019385.hg.1	SH3TC2
TC05003409.hg.1	2.25	JUC05019405.hg.1	SH3TC2
TC05003409.hg.1	2.04	PSR05026501.hg.1	SH3TC2
TC05003409.hg.1	-2.08	PSR05026534.hg.1	SH3TC2
TC05003409.hg.1	-2.26	JUC05019401.hg.1	SH3TC2
TC05003409.hg.1	-5.29	JUC05019386.hg.1	SH3TC2
TC04001030.hg.1	10.4	JUC04007534.hg.1	SLC2A9
TC04001030.hg.1	8.66	JUC04007514.hg.1	SLC2A9
TC04001030.hg.1	2.78	JUC04007520.hg.1	SLC2A9
TC04001030.hg.1	2.36	JUC04007522.hg.1	SLC2A9
TC04001030.hg.1	2.19	PSR04014095.hg.1	SLC2A9
TC04001030.hg.1	2.19	PSR04014119.hg.1	SLC2A9
TC04001030.hg.1	2.19	JUC04007516.hg.1	SLC2A9
TC04001030.hg.1	2.18	PSR04014127.hg.1	SLC2A9
TC04001030.hg.1	2.03	PSR04014121.hg.1	SLC2A9
TC04001030.hg.1	-2.13	JUC04007528.hg.1	SLC2A9
TC04001030.hg.1	-2.42	JUC04007513.hg.1	SLC2A9
TC04001030.hg.1	-2.46	JUC04007535.hg.1	SLC2A9
TC03002093.hg.1	10.26	JUC03018366.hg.1	MASP1
TC03002093.hg.1	8.64	JUC03018375.hg.1	MASP1
TC03002093.hg.1	8.2	PSR03037133.hg.1	MASP1
TC03002093.hg.1	7.03	PSR03037136.hg.1	MASP1
TC03002093.hg.1	6.78	PSR03037131.hg.1	MASP1
TC03002093.hg.1	6.22	JUC03018384.hg.1	MASP1
TC03002093.hg.1	5.93	PSR03037157.hg.1	MASP1
TC03002093.hg.1	5.79	JUC03018392.hg.1	MASP1
TC03002093.hg.1	5.29	PSR03037149.hg.1	MASP1
TC03002093.hg.1	5.07	PSR03037171.hg.1	MASP1
TC03002093.hg.1	4.93	PSR03037152.hg.1	MASP1
TC03002093.hg.1	4.87	PSR03037188.hg.1	MASP1
TC03002093.hg.1	4.86	PSR03037138.hg.1	MASP1
TC03002093.hg.1	4.81	JUC03018382.hg.1	MASP1
TC03002093.hg.1	4.58	JUC03018371.hg.1	MASP1
TC03002093.hg.1	4.28	PSR03037154.hg.1	MASP1
TC03002093.hg.1	4.19	JUC03018367.hg.1	MASP1
TC03002093.hg.1	4.01	PSR03037177.hg.1	MASP1
TC03002093.hg.1	3.78	PSR03037134.hg.1	MASP1
TC03002093.hg.1	3.62	PSR03037155.hg.1	MASP1
TC03002093.hg.1	3.6	PSR03037170.hg.1	MASP1
TC03002093.hg.1	3.2	PSR03037135.hg.1	MASP1
TC03002093.hg.1	3.2	PSR03037180.hg.1	MASP1
TC03002093.hg.1	3.09	JUC03018378.hg.1	MASP1
TC03002093.hg.1	2.81	PSR03037175.hg.1	MASP1
TC03002093.hg.1	2.79	PSR03037184.hg.1	MASP1
TC03002093.hg.1	2.7	JUC03018389.hg.1	MASP1
TC03002093.hg.1	2.66	PSR03037132.hg.1	MASP1
TC03002093.hg.1	2.42	PSR03037137.hg.1	MASP1
TC03002093.hg.1	2.29	PSR03037182.hg.1	MASP1
TC03002093.hg.1	2.22	PSR03037168.hg.1	MASP1
TC03002093.hg.1	2.15	PSR03037159.hg.1	MASP1
TC03002093.hg.1	-2.01	PSR03037150.hg.1	MASP1
TC03002093.hg.1	-2.29	PSR03037164.hg.1	MASP1
TC03002093.hg.1	-3.4	JUC03018380.hg.1	MASP1
TC03002093.hg.1	-3.5	JUC03018383.hg.1	MASP1
TC03002093.hg.1	-4.63	JUC03018388.hg.1	MASP1
TC03002093.hg.1	-4.81	JUC03018379.hg.1	MASP1

TC0X000230.hg.1	10.12	JUC0X001461.hg.1	CDK16
TC0X000230.hg.1	4.47	JUC0X001462.hg.1	CDK16
TC0X000230.hg.1	3.38	PSR0X002664.hg.1	CDK16
TC0X000230.hg.1	2.64	PSR0X002673.hg.1	CDK16
TC0X000230.hg.1	2.55	JUC0X001459.hg.1	CDK16
TC0X000230.hg.1	2.52	PSR0X002641.hg.1	CDK16
TC0X000230.hg.1	2.45	PSR0X002681.hg.1	CDK16
TC0X000230.hg.1	2.44	PSR0X002667.hg.1	CDK16
TC0X000230.hg.1	2.4	PSR0X002642.hg.1	CDK16
TC0X000230.hg.1	2.29	PSR0X002617.hg.1	CDK16
TC0X000230.hg.1	2.18	PSR0X002666.hg.1	CDK16
TC0X000230.hg.1	2.13	PSR0X002630.hg.1	CDK16
TC0X000230.hg.1	2.04	PSR0X002616.hg.1	CDK16
TC01002725.hg.1	10.11	PSR01042730.hg.1	KANK4
TC01002725.hg.1	3.77	PSR01042731.hg.1	KANK4
TC01002725.hg.1	3.01	PSR01042733.hg.1	KANK4
TC01002725.hg.1	2.7	JUC01022827.hg.1	KANK4
TC01002725.hg.1	2.64	PSR01042732.hg.1	KANK4
TC01002725.hg.1	2.26	PSR01042740.hg.1	KANK4
TC01002725.hg.1	2.08	JUC01022823.hg.1	KANK4
TC01002725.hg.1	-2.32	JUC01022829.hg.1	KANK4
TC01002725.hg.1	-2.34	PSR01042739.hg.1	KANK4
TC01002725.hg.1	-2.36	PSR01042742.hg.1	KANK4
TC01002725.hg.1	-2.54	JUC01022828.hg.1	KANK4
TC01002725.hg.1	-2.63	PSR01042744.hg.1	KANK4
TC01002725.hg.1	-3.04	PSR01042736.hg.1	KANK4
TC01002725.hg.1	-3.74	PSR01042738.hg.1	KANK4
TC01002725.hg.1	-4.41	PSR01042745.hg.1	KANK4
TC01002725.hg.1	-4.48	JUC01022821.hg.1	KANK4
TC07000425.hg.1	10.1	JUC07002734.hg.1	WBSCR17
TC07000425.hg.1	6.83	JUC07002727.hg.1	WBSCR17
TC07000425.hg.1	3.12	PSR07005728.hg.1	WBSCR17
TC07000425.hg.1	3.01	PSR07005716.hg.1	WBSCR17
TC07000425.hg.1	2.64	JUC07002726.hg.1	WBSCR17
TC07000425.hg.1	2.26	PSR07005714.hg.1	WBSCR17
TC07000425.hg.1	-2.06	JUC07002728.hg.1	WBSCR17
TC07000425.hg.1	-2.08	JUC07002724.hg.1	WBSCR17
TC07000425.hg.1	-2.2	PSR07005711.hg.1	WBSCR17
TC07000425.hg.1	-2.35	PSR07005712.hg.1	WBSCR17
TC07000425.hg.1	-2.77	JUC07002736.hg.1	WBSCR17
TC07000425.hg.1	-3	PSR07005713.hg.1	WBSCR17
TC03001382.hg.1	10.07	JUC03012047.hg.1	COL7A1
TC03001382.hg.1	10	JUC03012054.hg.1	COL7A1
TC03001382.hg.1	9.39	JUC03012000.hg.1	COL7A1
TC03001382.hg.1	7.5	PSR03024171.hg.1	COL7A1
TC03001382.hg.1	6.18	PSR03024170.hg.1	COL7A1
TC03001382.hg.1	6.15	JUC03012063.hg.1	COL7A1
TC03001382.hg.1	5.22	PSR03024044.hg.1	COL7A1
TC03001382.hg.1	5.13	PSR03024168.hg.1	COL7A1
TC03001382.hg.1	5.1	JUC03012030.hg.1	COL7A1
TC03001382.hg.1	5.01	JUC03012001.hg.1	COL7A1
TC03001382.hg.1	4.56	PSR03024172.hg.1	COL7A1
TC03001382.hg.1	4.44	PSR03024158.hg.1	COL7A1
TC03001382.hg.1	4.43	PSR03024157.hg.1	COL7A1
TC03001382.hg.1	4.31	PSR03024173.hg.1	COL7A1
TC03001382.hg.1	3.91	JUC03012040.hg.1	COL7A1
TC03001382.hg.1	3.65	PSR03024057.hg.1	COL7A1
TC03001382.hg.1	3.61	PSR03024169.hg.1	COL7A1

TC03001382.hg.1	3.46	PSR03024161.hg.1	COL7A1
TC03001382.hg.1	3.19	PSR03024164.hg.1	COL7A1
TC03001382.hg.1	3.16	JUC03012013.hg.1	COL7A1
TC03001382.hg.1	3.06	PSR03024153.hg.1	COL7A1
TC03001382.hg.1	3.04	PSR03024166.hg.1	COL7A1
TC03001382.hg.1	2.95	PSR03024122.hg.1	COL7A1
TC03001382.hg.1	2.73	PSR03024162.hg.1	COL7A1
TC03001382.hg.1	2.53	PSR03024165.hg.1	COL7A1
TC03001382.hg.1	2.49	PSR03024043.hg.1	COL7A1
TC03001382.hg.1	2.39	PSR03024150.hg.1	COL7A1
TC03001382.hg.1	2.34	JUC03011993.hg.1	COL7A1
TC03001382.hg.1	2.23	JUC03011980.hg.1	COL7A1
TC03001382.hg.1	2.04	JUC03012037.hg.1	COL7A1
TC03001382.hg.1	2.03	JUC03011990.hg.1	COL7A1
TC03001382.hg.1	-2	JUC03011971.hg.1	COL7A1
TC03001382.hg.1	-2.06	PSR03024104.hg.1	COL7A1
TC03001382.hg.1	-2.07	PSR03024098.hg.1	COL7A1
TC03001382.hg.1	-2.1	PSR03024092.hg.1	COL7A1
TC03001382.hg.1	-2.32	JUC03011979.hg.1	COL7A1
TC03001382.hg.1	-2.59	PSR03024146.hg.1	COL7A1
TC17001250.hg.1	10	PSR17016769.hg.1	ALDH3A1
TC17001250.hg.1	9.72	PSR17016802.hg.1	ALDH3A1
TC17001250.hg.1	9.59	PSR17016786.hg.1	ALDH3A1
TC17001250.hg.1	8.27	JUC17009527.hg.1	ALDH3A1
TC17001250.hg.1	8.13	PSR17016794.hg.1	ALDH3A1
TC17001250.hg.1	8.12	PSR17016782.hg.1	ALDH3A1
TC17001250.hg.1	8.11	PSR17016790.hg.1	ALDH3A1
TC17001250.hg.1	8.07	JUC17009523.hg.1	ALDH3A1
TC17001250.hg.1	7.69	PSR17016781.hg.1	ALDH3A1
TC17001250.hg.1	7.69	PSR17016784.hg.1	ALDH3A1
TC17001250.hg.1	7.4	PSR17016789.hg.1	ALDH3A1
TC17001250.hg.1	7.26	JUC17009526.hg.1	ALDH3A1
TC17001250.hg.1	6.92	PSR17016780.hg.1	ALDH3A1
TC17001250.hg.1	6.53	PSR17016795.hg.1	ALDH3A1
TC17001250.hg.1	6.33	PSR17016785.hg.1	ALDH3A1
TC17001250.hg.1	6.24	PSR17016776.hg.1	ALDH3A1
TC17001250.hg.1	5.48	PSR17016800.hg.1	ALDH3A1
TC17001250.hg.1	5.02	JUC17009532.hg.1	ALDH3A1
TC17001250.hg.1	4.61	PSR17016765.hg.1	ALDH3A1
TC17001250.hg.1	4.38	PSR17016771.hg.1	ALDH3A1
TC17001250.hg.1	4.35	JUC17009525.hg.1	ALDH3A1
TC17001250.hg.1	4.08	JUC17009524.hg.1	ALDH3A1
TC17001250.hg.1	3.56	JUC17009522.hg.1	ALDH3A1
TC17001250.hg.1	3.08	JUC17009521.hg.1	ALDH3A1
TC17001250.hg.1	2.99	PSR17016763.hg.1	ALDH3A1
TC17001250.hg.1	2.72	PSR17016787.hg.1	ALDH3A1
TC17001250.hg.1	2.7	PSR17016761.hg.1	ALDH3A1
TC17001250.hg.1	2.53	PSR17016804.hg.1	ALDH3A1
TC17001250.hg.1	-2.07	PSR17016767.hg.1	ALDH3A1
TC17001250.hg.1	-2.9	JUC17009533.hg.1	ALDH3A1
TC17001250.hg.1	-4.09	PSR17016783.hg.1	ALDH3A1
TC17001250.hg.1	-4.72	JUC17009535.hg.1	ALDH3A1
TC17001250.hg.1	-7.44	PSR17016774.hg.1	ALDH3A1
TC17001250.hg.1	-9.14	JUC17009519.hg.1	ALDH3A1
TC17001250.hg.1	-19.92	JUC17009518.hg.1	ALDH3A1
TC17001250.hg.1	-23.73	JUC17009530.hg.1	ALDH3A1
TC01001704.hg.1	9.98	JUC01014213.hg.1	NFASC
TC01001704.hg.1	6.52	PSR01026480.hg.1	NFASC

TC01001704.hg.1	5.61	JUC01014185.hg.1	NFASC
TC01001704.hg.1	5.35	JUC01014238.hg.1	NFASC
TC01001704.hg.1	4.92	JUC01014186.hg.1	NFASC
TC01001704.hg.1	4.8	JUC01014205.hg.1	NFASC
TC01001704.hg.1	4.47	JUC01014191.hg.1	NFASC
TC01001704.hg.1	4.29	JUC01014222.hg.1	NFASC
TC01001704.hg.1	4.14	JUC01014183.hg.1	NFASC
TC01001704.hg.1	4.01	PSR01026475.hg.1	NFASC
TC01001704.hg.1	3.62	JUC01014240.hg.1	NFASC
TC01001704.hg.1	3.61	JUC01014189.hg.1	NFASC
TC01001704.hg.1	3.45	JUC01014230.hg.1	NFASC
TC01001704.hg.1	3.38	JUC01014193.hg.1	NFASC
TC01001704.hg.1	3.35	PSR01026476.hg.1	NFASC
TC01001704.hg.1	3.32	PSR01026474.hg.1	NFASC
TC01001704.hg.1	3.31	PSR01026499.hg.1	NFASC
TC01001704.hg.1	3.16	PSR01026507.hg.1	NFASC
TC01001704.hg.1	3.16	JUC01014201.hg.1	NFASC
TC01001704.hg.1	3.13	PSR01026532.hg.1	NFASC
TC01001704.hg.1	3.06	PSR01026506.hg.1	NFASC
TC01001704.hg.1	3.06	JUC01014209.hg.1	NFASC
TC01001704.hg.1	3.02	PSR01026547.hg.1	NFASC
TC01001704.hg.1	2.98	JUC01014211.hg.1	NFASC
TC01001704.hg.1	2.89	JUC01014225.hg.1	NFASC
TC01001704.hg.1	2.83	PSR01026497.hg.1	NFASC
TC01001704.hg.1	2.7	PSR01026496.hg.1	NFASC
TC01001704.hg.1	2.66	JUC01014231.hg.1	NFASC
TC01001704.hg.1	2.47	JUC01014218.hg.1	NFASC
TC01001704.hg.1	2.38	JUC01014199.hg.1	NFASC
TC01001704.hg.1	2.26	PSR01026524.hg.1	NFASC
TC01001704.hg.1	2.24	PSR01026515.hg.1	NFASC
TC01001704.hg.1	2.13	JUC01014180.hg.1	NFASC
TC01001704.hg.1	2.09	PSR01026548.hg.1	NFASC
TC01001704.hg.1	2	PSR01026510.hg.1	NFASC
TC01001704.hg.1	-2.1	JUC01014219.hg.1	NFASC
TC01001704.hg.1	-2.34	JUC01014202.hg.1	NFASC
TC01001704.hg.1	-2.36	JUC01014217.hg.1	NFASC
TC01001704.hg.1	-2.58	JUC01014241.hg.1	NFASC
TC01001704.hg.1	-2.77	PSR01026541.hg.1	NFASC
TC08001552.hg.1	9.97	PSR08020447.hg.1	TNFRSF11B
TC08001552.hg.1	9.36	PSR08020448.hg.1	TNFRSF11B
TC08001552.hg.1	7.09	JUC08010437.hg.1	TNFRSF11B
TC08001552.hg.1	5.06	PSR08020443.hg.1	TNFRSF11B
TC08001552.hg.1	2.39	JUC08010439.hg.1	TNFRSF11B
TC14001466.hg.1	9.96	JUC14008515.hg.1	SERPINA1
TC14001466.hg.1	6.98	PSR14016504.hg.1	SERPINA1
TC14001466.hg.1	6.67	JUC14008529.hg.1	SERPINA1
TC14001466.hg.1	6.23	PSR14016508.hg.1	SERPINA1
TC14001466.hg.1	6.22	JUC14008517.hg.1	SERPINA1
TC14001466.hg.1	6.11	JUC14008516.hg.1	SERPINA1
TC14001466.hg.1	5.56	PSR14016499.hg.1	SERPINA1
TC14001466.hg.1	5.3	PSR14016525.hg.1	SERPINA1
TC14001466.hg.1	5.28	JUC14008514.hg.1	SERPINA1
TC14001466.hg.1	5.06	JUC14008521.hg.1	SERPINA1
TC14001466.hg.1	4.73	JUC14008525.hg.1	SERPINA1
TC14001466.hg.1	4.62	JUC14008518.hg.1	SERPINA1
TC14001466.hg.1	4.35	JUC14008523.hg.1	SERPINA1
TC14001466.hg.1	4.19	PSR14016526.hg.1	SERPINA1
TC14001466.hg.1	3.49	JUC14008522.hg.1	SERPINA1

TC14001466.hg.1	3.31	JUC14008519.hg.1	SERPINA1
TC14001466.hg.1	-2.27	JUC14008526.hg.1	SERPINA1
TC14001466.hg.1	-2.48	JUC14008513.hg.1	SERPINA1
TC17000654.hg.1	9.95	JUC17004532.hg.1	ITGA3
TC17000654.hg.1	7.77	JUC17004527.hg.1	ITGA3
TC17000654.hg.1	5.09	PSR17008176.hg.1	ITGA3
TC17000654.hg.1	4.96	JUC17004502.hg.1	ITGA3
TC17000654.hg.1	4.86	PSR17008158.hg.1	ITGA3
TC17000654.hg.1	4.77	PSR17008182.hg.1	ITGA3
TC17000654.hg.1	4.27	PSR17008169.hg.1	ITGA3
TC17000654.hg.1	4.11	PSR17008143.hg.1	ITGA3
TC17000654.hg.1	4.03	PSR17008186.hg.1	ITGA3
TC17000654.hg.1	3.55	PSR17008153.hg.1	ITGA3
TC17000654.hg.1	3.19	PSR17008147.hg.1	ITGA3
TC17000654.hg.1	3.06	PSR17008188.hg.1	ITGA3
TC17000654.hg.1	2.93	PSR17008178.hg.1	ITGA3
TC17000654.hg.1	2.79	JUC17004514.hg.1	ITGA3
TC17000654.hg.1	2.74	JUC17004509.hg.1	ITGA3
TC17000654.hg.1	2.66	PSR17008179.hg.1	ITGA3
TC17000654.hg.1	-2.19	JUC17004519.hg.1	ITGA3
TC17000654.hg.1	-2.45	JUC17004511.hg.1	ITGA3
TC17000654.hg.1	-2.83	JUC17004517.hg.1	ITGA3
TC03000791.hg.1	9.94	PSR03014276.hg.1	ZIC1
TC03000791.hg.1	8.51	JUC03007207.hg.1	ZIC1
TC03000791.hg.1	4.98	PSR03014275.hg.1	ZIC1
TC03000791.hg.1	2.68	PSR03014278.hg.1	ZIC1
TC03000791.hg.1	2.24	JUC03007205.hg.1	ZIC1
TC14002338.hg.1	9.89	JUC14012318.hg.1	PPP1R3E
TC14002338.hg.1	6.26	JUC14012324.hg.1	PPP1R3E
TC14002338.hg.1	3.65	PSR14010571.hg.1	PPP1R3E
TC14002338.hg.1	3.45	PSR14010574.hg.1	PPP1R3E
TC14002338.hg.1	3.34	JUC14012322.hg.1	PPP1R3E
TC14002338.hg.1	3.17	PSR14010573.hg.1	PPP1R3E
TC14002338.hg.1	3.04	JUC14012321.hg.1	PPP1R3E
TC14002338.hg.1	2.31	PSR14010568.hg.1	PPP1R3E
TC03001255.hg.1	9.87	JUC03010703.hg.1	SLC4A7
TC03001255.hg.1	4.64	JUC03010715.hg.1	SLC4A7
TC03001255.hg.1	3.49	PSR03021509.hg.1	SLC4A7
TC03001255.hg.1	3.16	PSR03021530.hg.1	SLC4A7
TC03001255.hg.1	3.16	PSR03021534.hg.1	SLC4A7
TC03001255.hg.1	3.14	PSR03021502.hg.1	SLC4A7
TC03001255.hg.1	3.11	JUC03010709.hg.1	SLC4A7
TC03001255.hg.1	3.1	PSR03021523.hg.1	SLC4A7
TC03001255.hg.1	2.61	JUC03010717.hg.1	SLC4A7
TC03001255.hg.1	2.58	PSR03021522.hg.1	SLC4A7
TC03001255.hg.1	2.53	PSR03021518.hg.1	SLC4A7
TC03001255.hg.1	-2.21	PSR03021496.hg.1	SLC4A7
TC03001255.hg.1	-2.29	JUC03010705.hg.1	SLC4A7
TC03001255.hg.1	-2.31	JUC03010692.hg.1	SLC4A7
TC03001255.hg.1	-3.06	JUC03010694.hg.1	SLC4A7
TC07001327.hg.1	9.81	JUC07010013.hg.1	COA1
TC07001327.hg.1	5.17	PSR07020475.hg.1	COA1
TC07001327.hg.1	4.66	JUC07010025.hg.1	COA1
TC07001327.hg.1	2.94	JUC07010015.hg.1	COA1
TC07001327.hg.1	-2.62	PSR07020459.hg.1	COA1
TC02000281.hg.1	9.8	PSR02004258.hg.1	EPAS1, LOC[illegible]
TC02000281.hg.1	6.5	PSR02004249.hg.1	EPAS1, LOC[illegible]
TC02000281.hg.1	4.35	PSR02004254.hg.1	EPAS1, LOC[illegible]

TC02000281.hg.1	4.03	JUC02002226.hg.1	EPAS1, LOC1
TC02000281.hg.1	3.05	PSR02004248.hg.1	EPAS1, LOC1
TC02000281.hg.1	2.86	PSR02004274.hg.1	EPAS1, LOC1
TC02000281.hg.1	-2.29	JUC02002237.hg.1	EPAS1, LOC1
TC02000281.hg.1	-2.39	JUC02002239.hg.1	EPAS1, LOC1
TC02000281.hg.1	-2.62	PSR02004276.hg.1	EPAS1, LOC1
TC02000281.hg.1	-3.02	JUC02002222.hg.1	EPAS1, LOC1
TC02000281.hg.1	-3.08	PSR02004264.hg.1	EPAS1, LOC1
TC02000281.hg.1	-3.78	JUC02002238.hg.1	EPAS1, LOC1
TC02000281.hg.1	-5.14	JUC02002231.hg.1	EPAS1, LOC1
TC12000099.hg.1	9.75	PSR12001255.hg.1	ENO2
TC12000099.hg.1	7.68	PSR12001256.hg.1	ENO2
TC12000099.hg.1	7.45	PSR12001267.hg.1	ENO2
TC12000099.hg.1	5.68	PSR12001272.hg.1	ENO2
TC12000099.hg.1	5.36	PSR12001259.hg.1	ENO2
TC12000099.hg.1	4.29	JUC12000653.hg.1	ENO2
TC12000099.hg.1	4.27	JUC12000645.hg.1	ENO2
TC12000099.hg.1	3.68	JUC12000648.hg.1	ENO2
TC12000099.hg.1	3.5	JUC12000646.hg.1	ENO2
TC12000099.hg.1	3.44	PSR12001265.hg.1	ENO2
TC12000099.hg.1	2.83	JUC12000655.hg.1	ENO2
TC12000099.hg.1	2.82	PSR12001260.hg.1	ENO2
TC12000099.hg.1	2.62	JUC12000656.hg.1	ENO2
TC12000099.hg.1	2.59	JUC12000647.hg.1	ENO2
TC12000099.hg.1	2.05	PSR12001268.hg.1	ENO2
TC10002927.hg.1	9.69	JUC10017022.hg.1	LDB3
TC10002927.hg.1	3.11	JUC10017010.hg.1	LDB3
TC10002927.hg.1	2.53	JUC10017009.hg.1	LDB3
TC10002927.hg.1	2.04	PSR10006610.hg.1	LDB3
TC10002927.hg.1	-2.02	PSR10006634.hg.1	LDB3
TC01001313.hg.1	9.59	JUC01010885.hg.1	LMNA
TC01001313.hg.1	3.63	JUC01010877.hg.1	LMNA
TC01001313.hg.1	3.63	JUC01010886.hg.1	LMNA
TC01001313.hg.1	2.1	PSR01020426.hg.1	LMNA
TC01001313.hg.1	2.03	JUC01010896.hg.1	LMNA
TC01001313.hg.1	-2.83	PSR01020388.hg.1	LMNA
TC06000532.hg.1	9.58	JUC06002389.hg.1	CDKN1A
TC06000532.hg.1	2.21	PSR06005802.hg.1	CDKN1A
TC05001389.hg.1	9.55	JUC05009966.hg.1	PDE4D
TC05001389.hg.1	9.07	PSR05019394.hg.1	PDE4D
TC05001389.hg.1	3.61	PSR05019387.hg.1	PDE4D
TC05001389.hg.1	3.43	PSR05019385.hg.1	PDE4D
TC05001389.hg.1	3.11	PSR05019388.hg.1	PDE4D
TC05001389.hg.1	3	PSR05019386.hg.1	PDE4D
TC05001389.hg.1	2.47	PSR05019374.hg.1	PDE4D
TC05001389.hg.1	2.45	JUC05010011.hg.1	PDE4D
TC05001389.hg.1	2.37	PSR05019382.hg.1	PDE4D
TC05001389.hg.1	2.36	PSR05019373.hg.1	PDE4D
TC05001389.hg.1	2.31	PSR05019368.hg.1	PDE4D
TC05001389.hg.1	2.23	JUC05010000.hg.1	PDE4D
TC05001389.hg.1	2.12	PSR05019367.hg.1	PDE4D
TC05001389.hg.1	-2.02	PSR05019362.hg.1	PDE4D
TC05001389.hg.1	-2.07	PSR05019355.hg.1	PDE4D
TC05001389.hg.1	-2.07	PSR05019410.hg.1	PDE4D
TC05001389.hg.1	-2.08	PSR05019401.hg.1	PDE4D
TC05001389.hg.1	-2.15	PSR05019421.hg.1	PDE4D
TC05001389.hg.1	-2.21	JUC05009969.hg.1	PDE4D
TC05001389.hg.1	-2.33	PSR05019429.hg.1	PDE4D

TC05001389.hg.1	-2.38	PSR05019414.hg.1	PDE4D
TC05001389.hg.1	-2.52	JUC05009962.hg.1	PDE4D
TC05001389.hg.1	-2.62	PSR05019404.hg.1	PDE4D
TC05001389.hg.1	-2.66	PSR05019425.hg.1	PDE4D
TC05001389.hg.1	-2.7	PSR05019363.hg.1	PDE4D
TC05001389.hg.1	-2.74	PSR05019417.hg.1	PDE4D
TC05001389.hg.1	-3.02	JUC05009956.hg.1	PDE4D
TC05001389.hg.1	-3.72	JUC05009982.hg.1	PDE4D
TC05001389.hg.1	-4.23	PSR05019413.hg.1	PDE4D
TC05001389.hg.1	-4.27	JUC05010007.hg.1	PDE4D
TC05001389.hg.1	-4.6	JUC05009996.hg.1	PDE4D
TC05001389.hg.1	-4.75	PSR05019407.hg.1	PDE4D
TC05001389.hg.1	-4.95	PSR05019411.hg.1	PDE4D
TC05001389.hg.1	-5.79	PSR05019418.hg.1	PDE4D
TC05001389.hg.1	-6.28	JUC05010005.hg.1	PDE4D
TC05001389.hg.1	-6.97	PSR05019409.hg.1	PDE4D
TC17001040.hg.1	9.55	JUC17007991.hg.1	INCA1
TC17001040.hg.1	-2.03	PSR17014000.hg.1	INCA1
TC05000401.hg.1	9.49	JUC05003141.hg.1	RASGRF2
TC05000401.hg.1	7.11	JUC05003146.hg.1	RASGRF2
TC05000401.hg.1	7.01	JUC05003120.hg.1	RASGRF2
TC05000401.hg.1	5.21	JUC05003118.hg.1	RASGRF2
TC05000401.hg.1	4.26	JUC05003143.hg.1	RASGRF2
TC05000401.hg.1	3.99	PSR05005724.hg.1	RASGRF2
TC05000401.hg.1	3.9	PSR05005739.hg.1	RASGRF2
TC05000401.hg.1	2.97	JUC05003145.hg.1	RASGRF2
TC05000401.hg.1	2.77	JUC05003131.hg.1	RASGRF2
TC05000401.hg.1	2.68	PSR05005740.hg.1	RASGRF2
TC05000401.hg.1	2.46	JUC05003135.hg.1	RASGRF2
TC05000401.hg.1	2.12	PSR05005736.hg.1	RASGRF2
TC05000401.hg.1	-2	PSR05005728.hg.1	RASGRF2
TC05000401.hg.1	-2.1	PSR05005731.hg.1	RASGRF2
TC05000401.hg.1	-2.37	PSR05005733.hg.1	RASGRF2
TC05000401.hg.1	-2.38	JUC05003136.hg.1	RASGRF2
TC05000401.hg.1	-2.52	JUC05003139.hg.1	RASGRF2
TC05000401.hg.1	-2.55	PSR05005729.hg.1	RASGRF2
TC05000401.hg.1	-2.61	PSR05005752.hg.1	RASGRF2
TC05000401.hg.1	-2.66	PSR05005743.hg.1	RASGRF2
TC05000401.hg.1	-2.81	PSR05005748.hg.1	RASGRF2
TC05000401.hg.1	-2.86	JUC05003138.hg.1	RASGRF2
TC05000401.hg.1	-2.96	PSR05005749.hg.1	RASGRF2
TC05000401.hg.1	-2.99	PSR05005745.hg.1	RASGRF2
TC05000401.hg.1	-3	PSR05005747.hg.1	RASGRF2
TC05000401.hg.1	-4.36	JUC05003124.hg.1	RASGRF2
TC05000401.hg.1	-5.05	PSR05005751.hg.1	RASGRF2
TC05000401.hg.1	-5.32	PSR05005721.hg.1	RASGRF2
TC05000401.hg.1	-6	PSR05005753.hg.1	RASGRF2
TC05000401.hg.1	-6.21	PSR05005742.hg.1	RASGRF2
TC05000401.hg.1	-6.71	JUC05003128.hg.1	RASGRF2
TC05000401.hg.1	-6.81	JUC05003123.hg.1	RASGRF2
TC05000401.hg.1	-9.23	JUC05003144.hg.1	RASGRF2
TC05000401.hg.1	-9.93	JUC05003129.hg.1	RASGRF2
TC05000401.hg.1	-11.08	PSR05005725.hg.1	RASGRF2
TC05000401.hg.1	-12.99	JUC05003116.hg.1	RASGRF2
TC01003638.hg.1	9.47	PSR01055959.hg.1	PTGS2
TC01003638.hg.1	7.14	JUC01029200.hg.1	PTGS2
TC01003638.hg.1	5.79	PSR01055969.hg.1	PTGS2
TC01003638.hg.1	5.48	JUC01029201.hg.1	PTGS2

TC01003638.hg.1	4.96	PSR01055954.hg.1	PTGS2
TC01003638.hg.1	3.14	PSR01055964.hg.1	PTGS2
TC01003638.hg.1	2.87	JUC01029207.hg.1	PTGS2
TC01001954.hg.1	9.37	PSR01030172.hg.1	LGALS8
TC01001954.hg.1	3.24	JUC01016317.hg.1	LGALS8
TC01001954.hg.1	2.92	JUC01016322.hg.1	LGALS8
TC01001954.hg.1	2.82	PSR01030169.hg.1	LGALS8
TC01001954.hg.1	2.82	JUC01016309.hg.1	LGALS8
TC01001954.hg.1	2.35	PSR01030163.hg.1	LGALS8
TC01001954.hg.1	2.23	JUC01016319.hg.1	LGALS8
TC01001954.hg.1	2.06	JUC01016325.hg.1	LGALS8
TC01001954.hg.1	2.06	JUC01016326.hg.1	LGALS8
TC01001954.hg.1	-3.1	PSR01030181.hg.1	LGALS8
TC02002624.hg.1	9.37	JUC02021911.hg.1	STAT1
TC02002624.hg.1	4.41	JUC02021918.hg.1	STAT1
TC02002624.hg.1	3.7	JUC02021937.hg.1	STAT1
TC02002624.hg.1	2.69	PSR02042058.hg.1	STAT1
TC02002624.hg.1	2.6	PSR02042023.hg.1	STAT1
TC02002624.hg.1	2.41	PSR02042052.hg.1	STAT1
TC02002624.hg.1	2.36	PSR02042006.hg.1	STAT1
TC02002624.hg.1	-2.16	PSR02042033.hg.1	STAT1
TC02002624.hg.1	-4.74	JUC02021932.hg.1	STAT1
TC06001717.hg.1	9.28	JUC06009702.hg.1	TREM1
TC06001717.hg.1	7.03	PSR06020463.hg.1	TREM1
TC06001717.hg.1	5.24	PSR06020461.hg.1	TREM1
TC06001717.hg.1	4.44	PSR06020460.hg.1	TREM1
TC06001717.hg.1	3.88	PSR06020466.hg.1	TREM1
TC15000800.hg.1	9.26	JUC15003821.hg.1	ADAMTSL3
TC15000800.hg.1	4.01	JUC15003803.hg.1	ADAMTSL3
TC15000800.hg.1	3.58	JUC15003812.hg.1	ADAMTSL3
TC15000800.hg.1	3.44	JUC15003816.hg.1	ADAMTSL3
TC15000800.hg.1	3.22	JUC15003794.hg.1	ADAMTSL3
TC15000800.hg.1	2.75	JUC15003813.hg.1	ADAMTSL3
TC15000800.hg.1	2.66	PSR15007321.hg.1	ADAMTSL3
TC15000800.hg.1	2.65	JUC15003804.hg.1	ADAMTSL3
TC15000800.hg.1	2.64	PSR15007320.hg.1	ADAMTSL3
TC15000800.hg.1	2.42	PSR15007322.hg.1	ADAMTSL3
TC15000800.hg.1	2.32	PSR15007302.hg.1	ADAMTSL3
TC15000800.hg.1	2.31	JUC15003805.hg.1	ADAMTSL3
TC15000800.hg.1	-2.25	JUC15003811.hg.1	ADAMTSL3
TC15000800.hg.1	-2.32	JUC15003795.hg.1	ADAMTSL3
TC15000800.hg.1	-2.45	PSR15007315.hg.1	ADAMTSL3
TC15000800.hg.1	-2.58	JUC15003817.hg.1	ADAMTSL3
TC15000800.hg.1	-2.74	JUC15003820.hg.1	ADAMTSL3
TC15000800.hg.1	-2.77	PSR15007316.hg.1	ADAMTSL3
TC15000800.hg.1	-2.78	JUC15003808.hg.1	ADAMTSL3
TC15000800.hg.1	-2.88	PSR15007299.hg.1	ADAMTSL3
TC15000800.hg.1	-2.91	PSR15007324.hg.1	ADAMTSL3
TC15000800.hg.1	-2.98	PSR15007314.hg.1	ADAMTSL3
TC15000800.hg.1	-3.11	JUC15003810.hg.1	ADAMTSL3
TC15000800.hg.1	-3.43	PSR15007298.hg.1	ADAMTSL3
TC15000800.hg.1	-3.88	JUC15003807.hg.1	ADAMTSL3
TC15000800.hg.1	-4.22	PSR15007331.hg.1	ADAMTSL3
TC15000800.hg.1	-4.93	JUC15003823.hg.1	ADAMTSL3
TC15000800.hg.1	-6.39	PSR15007327.hg.1	ADAMTSL3
TC15000800.hg.1	-6.87	PSR15007330.hg.1	ADAMTSL3
TC15000800.hg.1	-8.65	JUC15003799.hg.1	ADAMTSL3
TC15000800.hg.1	-9.37	PSR15007328.hg.1	ADAMTSL3

TC15000800.hg.1	-10.99	PSR15007332.hg.1	ADAMTSL3
TC15000800.hg.1	-19.84	PSR15007306.hg.1	ADAMTSL3
TC15000800.hg.1	-23.54	JUC15003822.hg.1	ADAMTSL3
TC21000559.hg.1	9.26	PSR21006827.hg.1	S100B
TC21000559.hg.1	8	PSR21006829.hg.1	S100B
TC21000559.hg.1	7.11	PSR21006830.hg.1	S100B
TC21000559.hg.1	6.38	PSR21006828.hg.1	S100B
TC21000559.hg.1	6.15	PSR21006822.hg.1	S100B
TC21000559.hg.1	5.74	PSR21006825.hg.1	S100B
TC21000559.hg.1	4.22	JUC21003510.hg.1	S100B
TC03000944.hg.1	9.2	JUC03008302.hg.1	LINC00578
TC03000944.hg.1	-2.02	PSR03016306.hg.1	LINC00578
TC03000944.hg.1	-5.38	PSR03016301.hg.1	LINC00578
TC21000420.hg.1	9.2	PSR21004944.hg.1	RCAN1
TC21000420.hg.1	8.72	PSR21004941.hg.1	RCAN1
TC21000420.hg.1	8.35	JUC21002514.hg.1	RCAN1
TC21000420.hg.1	8.1	JUC21002516.hg.1	RCAN1
TC21000420.hg.1	8.07	PSR21004939.hg.1	RCAN1
TC21000420.hg.1	6.5	PSR21004942.hg.1	RCAN1
TC21000420.hg.1	5.86	PSR21004940.hg.1	RCAN1
TC21000420.hg.1	5.36	PSR21004921.hg.1	RCAN1
TC21000420.hg.1	4.63	PSR21004938.hg.1	RCAN1
TC21000420.hg.1	3.48	PSR21004927.hg.1	RCAN1
TC21000420.hg.1	3.24	JUC21002525.hg.1	RCAN1
TC21000420.hg.1	2.59	PSR21004936.hg.1	RCAN1
TC21000420.hg.1	2.45	JUC21002522.hg.1	RCAN1
TC07000328.hg.1	9.17	JUC07002401.hg.1	EGFR
TC07000328.hg.1	4.01	JUC07002372.hg.1	EGFR
TC07000328.hg.1	2.58	PSR07004855.hg.1	EGFR
TC07000328.hg.1	2.58	PSR07004858.hg.1	EGFR
TC07000328.hg.1	2.41	PSR07004880.hg.1	EGFR
TC07000328.hg.1	2.29	PSR07004851.hg.1	EGFR
TC07000328.hg.1	2.17	JUC07002375.hg.1	EGFR
TC07000328.hg.1	2	PSR07004874.hg.1	EGFR
TC09001070.hg.1	9.09	JUC09007729.hg.1	RNF38
TC09001070.hg.1	7.83	JUC09007723.hg.1	RNF38
TC09001070.hg.1	4.02	PSR09014342.hg.1	RNF38
TC10000773.hg.1	9.08	JUC10005116.hg.1	PDCD11
TC10000773.hg.1	2.6	JUC10005118.hg.1	PDCD11
TC10000773.hg.1	2.22	PSR10009205.hg.1	PDCD11
TC10000773.hg.1	2.1	JUC10005138.hg.1	PDCD11
TC10000773.hg.1	2.01	JUC10005125.hg.1	PDCD11
TC10000773.hg.1	-2.05	JUC10005144.hg.1	PDCD11
TC10000773.hg.1	-2.21	PSR10009224.hg.1	PDCD11
TC17001240.hg.1	9.08	JUC17009475.hg.1	B9D1
TC17001240.hg.1	4.88	JUC17009459.hg.1	B9D1
TC17001240.hg.1	3.91	JUC17009463.hg.1	B9D1
TC17001240.hg.1	2.61	PSR17016660.hg.1	B9D1
TC17001240.hg.1	2.45	PSR17016683.hg.1	B9D1
TC17001240.hg.1	2.39	PSR17016691.hg.1	B9D1
TC17001240.hg.1	2.33	PSR17016689.hg.1	B9D1
TC17001240.hg.1	2.32	PSR17016687.hg.1	B9D1
TC17001240.hg.1	2.29	PSR17016662.hg.1	B9D1
TC17001240.hg.1	2.21	PSR17016686.hg.1	B9D1
TC17001240.hg.1	2.18	PSR17016664.hg.1	B9D1
TC17001240.hg.1	2.12	JUC17009467.hg.1	B9D1
TC17001240.hg.1	2.11	PSR17016667.hg.1	B9D1
TC17001240.hg.1	2.06	PSR17016669.hg.1	B9D1

TC17001240.hg.1	-2.18	JUC17009469.hg.1	B9D1
TC17001240.hg.1	-2.21	JUC17009472.hg.1	B9D1
TC19001210.hg.1	9.04	JUC19009834.hg.1	PRDX2
TC19001210.hg.1	2.65	PSR19017006.hg.1	PRDX2
TC19001210.hg.1	2.57	PSR19017002.hg.1	PRDX2
TC19001210.hg.1	2.16	PSR19017009.hg.1	PRDX2
TC02002174.hg.1	8.99	JUC02018260.hg.1	ST6GAL2
TC02002174.hg.1	7.47	JUC02018263.hg.1	ST6GAL2
TC02002174.hg.1	6.76	JUC02018267.hg.1	ST6GAL2
TC02002174.hg.1	4.3	PSR02035394.hg.1	ST6GAL2
TC02002174.hg.1	3.36	PSR02035392.hg.1	ST6GAL2
TC02002174.hg.1	2.46	PSR02035393.hg.1	ST6GAL2
TC02002174.hg.1	-2.92	PSR02035396.hg.1	ST6GAL2
TC02002174.hg.1	-4.01	JUC02018266.hg.1	ST6GAL2
TC02002174.hg.1	-4.42	PSR02035395.hg.1	ST6GAL2
TC02002174.hg.1	-7.09	PSR02035388.hg.1	ST6GAL2
TC02002174.hg.1	-9.02	JUC02018264.hg.1	ST6GAL2
TC02002174.hg.1	-10.79	JUC02018268.hg.1	ST6GAL2
TC02002174.hg.1	-11.48	PSR02035397.hg.1	ST6GAL2
TC02002174.hg.1	-12.91	PSR02035399.hg.1	ST6GAL2
TC02002174.hg.1	-14.75	PSR02035398.hg.1	ST6GAL2
TC02002174.hg.1	-17.04	PSR02035402.hg.1	ST6GAL2
TC02002174.hg.1	-17.45	PSR02035390.hg.1	ST6GAL2
TC02002174.hg.1	-20.33	PSR02035400.hg.1	ST6GAL2
TC6_ssto_hap700(	8.97	JUC6_ssto_hap7001	HLA-B
TC6_ssto_hap700(	3.89	JUC6_ssto_hap7001	HLA-B
TC6_ssto_hap700(	3.58	PSR6_ssto_hap7002	HLA-B
TC6_ssto_hap700(	3	JUC6_ssto_hap7001	HLA-B
TC6_ssto_hap700(	2.92	JUC6_ssto_hap7001	HLA-B
TC6_ssto_hap700(	2.91	PSR6_ssto_hap7002	HLA-B
TC6_ssto_hap700(	2.68	PSR6_ssto_hap7002	HLA-B
TC6_ssto_hap700(	2.56	PSR6_ssto_hap7002	HLA-B
TC6_ssto_hap700(	2.5	PSR6_ssto_hap7002	HLA-B
TC6_ssto_hap700(	2.34	PSR6_ssto_hap7002	HLA-B
TC6_ssto_hap700(	2.34	PSR6_ssto_hap7002	HLA-B
TC6_ssto_hap700(	2.31	PSR6_ssto_hap7002	HLA-B
TC6_ssto_hap700(	2.01	PSR6_ssto_hap7002	HLA-B
TC03000495.hg.1	8.84	JUC03004682.hg.1	ST3GAL6
TC03000495.hg.1	4.52	PSR03009403.hg.1	ST3GAL6
TC03000495.hg.1	4.42	PSR03009401.hg.1	ST3GAL6
TC03000495.hg.1	3.95	PSR03009402.hg.1	ST3GAL6
TC03000495.hg.1	3.34	PSR03009345.hg.1	ST3GAL6
TC03000495.hg.1	3.33	JUC03004664.hg.1	ST3GAL6
TC03000495.hg.1	3.16	PSR03009358.hg.1	ST3GAL6
TC03000495.hg.1	3.15	PSR03009339.hg.1	ST3GAL6
TC03000495.hg.1	3.11	PSR03009344.hg.1	ST3GAL6
TC03000495.hg.1	3.05	PSR03009363.hg.1	ST3GAL6
TC03000495.hg.1	2.99	PSR03009354.hg.1	ST3GAL6
TC03000495.hg.1	2.96	PSR03009346.hg.1	ST3GAL6
TC03000495.hg.1	2.9	PSR03009392.hg.1	ST3GAL6
TC03000495.hg.1	2.76	PSR03009380.hg.1	ST3GAL6
TC03000495.hg.1	2.69	PSR03009334.hg.1	ST3GAL6
TC03000495.hg.1	2.67	PSR03009398.hg.1	ST3GAL6
TC03000495.hg.1	2.55	PSR03009371.hg.1	ST3GAL6
TC03000495.hg.1	2.54	PSR03009367.hg.1	ST3GAL6
TC03000495.hg.1	2.53	PSR03009337.hg.1	ST3GAL6
TC03000495.hg.1	2.37	JUC03004681.hg.1	ST3GAL6
TC03000495.hg.1	2.35	PSR03009396.hg.1	ST3GAL6

TC03000495.hg.1	2.3	PSR03009378.hg.1	ST3GAL6
TC03000495.hg.1	2.28	JUC03004691.hg.1	ST3GAL6
TC03000495.hg.1	2.25	JUC03004678.hg.1	ST3GAL6
TC03000495.hg.1	2.22	PSR03009400.hg.1	ST3GAL6
TC03000495.hg.1	2.2	PSR03009347.hg.1	ST3GAL6
TC03000495.hg.1	2.1	JUC03004663.hg.1	ST3GAL6
TC03000495.hg.1	2.03	JUC03004694.hg.1	ST3GAL6
TC03000561.hg.1	8.78	JUC03005064.hg.1	PVRL3
TC03000561.hg.1	3.06	JUC03005062.hg.1	PVRL3
TC03000561.hg.1	2.49	PSR03010133.hg.1	PVRL3
TC03000561.hg.1	2.44	PSR03010138.hg.1	PVRL3
TC03000561.hg.1	2.31	PSR03010140.hg.1	PVRL3
TC03000561.hg.1	2.19	PSR03010135.hg.1	PVRL3
TC03000561.hg.1	2.16	PSR03010144.hg.1	PVRL3
TC15000368.hg.1	8.78	JUC15001592.hg.1	SEMA6D
TC15000368.hg.1	5.72	PSR15003354.hg.1	SEMA6D
TC15000368.hg.1	4.13	PSR15003350.hg.1	SEMA6D
TC15000368.hg.1	4.04	PSR15003353.hg.1	SEMA6D
TC15000368.hg.1	3.49	PSR15003349.hg.1	SEMA6D
TC15000368.hg.1	3.28	PSR15003356.hg.1	SEMA6D
TC15000368.hg.1	3.1	PSR15003378.hg.1	SEMA6D
TC15000368.hg.1	3	PSR15003362.hg.1	SEMA6D
TC15000368.hg.1	2.92	PSR15003360.hg.1	SEMA6D
TC15000368.hg.1	2.78	PSR15003381.hg.1	SEMA6D
TC15000368.hg.1	2.59	PSR15003357.hg.1	SEMA6D
TC15000368.hg.1	2.58	JUC15001609.hg.1	SEMA6D
TC15000368.hg.1	2.57	PSR15003364.hg.1	SEMA6D
TC15000368.hg.1	-2.01	PSR15003371.hg.1	SEMA6D
TC15000368.hg.1	-2.03	PSR15003377.hg.1	SEMA6D
TC15000368.hg.1	-2.68	JUC15001590.hg.1	SEMA6D
TC04000706.hg.1	8.72	JUC04005392.hg.1	HHIP
TC04000706.hg.1	5.52	PSR04010020.hg.1	HHIP
TC04000706.hg.1	5.32	PSR04010021.hg.1	HHIP
TC04000706.hg.1	5.19	PSR04010034.hg.1	HHIP
TC04000706.hg.1	5.04	PSR04010037.hg.1	HHIP
TC04000706.hg.1	4.81	PSR04010019.hg.1	HHIP
TC04000706.hg.1	4.81	PSR04010022.hg.1	HHIP
TC04000706.hg.1	4.47	PSR04010045.hg.1	HHIP
TC04000706.hg.1	4.34	PSR04010032.hg.1	HHIP
TC04000706.hg.1	4.04	JUC04005393.hg.1	HHIP
TC04000706.hg.1	4.02	PSR04010040.hg.1	HHIP
TC04000706.hg.1	3.94	PSR04010043.hg.1	HHIP
TC04000706.hg.1	3.81	PSR04010052.hg.1	HHIP
TC04000706.hg.1	3.49	JUC04005372.hg.1	HHIP
TC04000706.hg.1	3.3	PSR04010033.hg.1	HHIP
TC04000706.hg.1	3.27	PSR04010044.hg.1	HHIP
TC04000706.hg.1	3.18	PSR04010051.hg.1	HHIP
TC04000706.hg.1	3.13	PSR04010049.hg.1	HHIP
TC04000706.hg.1	2.97	JUC04005383.hg.1	HHIP
TC04000706.hg.1	2.88	JUC04005384.hg.1	HHIP
TC04000706.hg.1	2.52	PSR04010029.hg.1	HHIP
TC04000706.hg.1	2.36	JUC04005375.hg.1	HHIP
TC04000706.hg.1	2.04	PSR04010048.hg.1	HHIP
TC10002938.hg.1	8.7	PSR10006674.hg.1	C10orf116
TC10002938.hg.1	5.49	PSR10006676.hg.1	C10orf116
TC10002938.hg.1	4.76	JUC10017204.hg.1	C10orf116
TC10002938.hg.1	4.16	PSR10006680.hg.1	C10orf116
TC10002938.hg.1	3.37	PSR10006683.hg.1	C10orf116

TC10002938.hg.1	-2.4	PSR10006679.hg.1	C10orf116
TC11002427.hg.1	8.7	JUC11015481.hg.1	CDON
TC11002427.hg.1	8.29	JUC11015494.hg.1	CDON
TC11002427.hg.1	7.59	PSR11029100.hg.1	CDON
TC11002427.hg.1	7.21	PSR11029091.hg.1	CDON
TC11002427.hg.1	7.15	PSR11029101.hg.1	CDON
TC11002427.hg.1	6.43	PSR11029087.hg.1	CDON
TC11002427.hg.1	6.33	JUC11015495.hg.1	CDON
TC11002427.hg.1	6.08	PSR11029095.hg.1	CDON
TC11002427.hg.1	5.24	PSR11029086.hg.1	CDON
TC11002427.hg.1	5.11	JUC11015488.hg.1	CDON
TC11002427.hg.1	3.78	JUC11015489.hg.1	CDON
TC11002427.hg.1	2.92	PSR11029099.hg.1	CDON
TC11002427.hg.1	2.41	JUC11015491.hg.1	CDON
TC11002427.hg.1	2.26	JUC11015480.hg.1	CDON
TC11002427.hg.1	2.1	JUC11015477.hg.1	CDON
TC11002427.hg.1	-2.1	PSR11029073.hg.1	CDON
TC11002427.hg.1	-2.14	JUC11015482.hg.1	CDON
TC11002427.hg.1	-2.69	JUC11015493.hg.1	CDON
TC11002427.hg.1	-6.42	JUC11015499.hg.1	CDON
TC10001676.hg.1	8.69	JUC10012801.hg.1	ABLIM1
TC10001676.hg.1	6.19	JUC10012833.hg.1	ABLIM1
TC10001676.hg.1	5.13	JUC10012831.hg.1	ABLIM1
TC10001676.hg.1	3.75	PSR10022098.hg.1	ABLIM1
TC10001676.hg.1	3.64	PSR10022061.hg.1	ABLIM1
TC10001676.hg.1	3.55	PSR10022092.hg.1	ABLIM1
TC10001676.hg.1	3.55	JUC10012822.hg.1	ABLIM1
TC10001676.hg.1	3.41	JUC10012802.hg.1	ABLIM1
TC10001676.hg.1	2.98	PSR10022063.hg.1	ABLIM1
TC10001676.hg.1	2.95	JUC10012804.hg.1	ABLIM1
TC10001676.hg.1	2.93	JUC10012798.hg.1	ABLIM1
TC10001676.hg.1	2.91	JUC10012830.hg.1	ABLIM1
TC10001676.hg.1	2.88	PSR10022094.hg.1	ABLIM1
TC10001676.hg.1	2.85	PSR10022075.hg.1	ABLIM1
TC10001676.hg.1	2.85	JUC10012824.hg.1	ABLIM1
TC10001676.hg.1	2.83	JUC10012826.hg.1	ABLIM1
TC10001676.hg.1	2.82	JUC10012796.hg.1	ABLIM1
TC10001676.hg.1	2.78	PSR10022095.hg.1	ABLIM1
TC10001676.hg.1	2.73	PSR10022096.hg.1	ABLIM1
TC10001676.hg.1	2.64	PSR10022091.hg.1	ABLIM1
TC10001676.hg.1	2.56	PSR10022077.hg.1	ABLIM1
TC10001676.hg.1	2.52	PSR10022062.hg.1	ABLIM1
TC10001676.hg.1	2.42	PSR10022101.hg.1	ABLIM1
TC10001676.hg.1	2.41	PSR10022039.hg.1	ABLIM1
TC10001676.hg.1	2.4	PSR10022081.hg.1	ABLIM1
TC10001676.hg.1	2.34	PSR10022100.hg.1	ABLIM1
TC10001676.hg.1	2.27	PSR10022088.hg.1	ABLIM1
TC10001676.hg.1	2.21	JUC10012803.hg.1	ABLIM1
TC10001676.hg.1	2.21	JUC10012815.hg.1	ABLIM1
TC10001676.hg.1	2.05	PSR10022069.hg.1	ABLIM1
TC10001676.hg.1	2.02	PSR10022046.hg.1	ABLIM1
TC10001676.hg.1	-2.7	PSR10022060.hg.1	ABLIM1
TC10001676.hg.1	-3.43	JUC10012837.hg.1	ABLIM1
TC06001046.hg.1	8.67	PSR06012235.hg.1	GPR126
TC06001046.hg.1	7.4	PSR06012251.hg.1	GPR126
TC06001046.hg.1	6.54	JUC06006011.hg.1	GPR126
TC06001046.hg.1	6.38	PSR06012240.hg.1	GPR126
TC06001046.hg.1	6.13	JUC06005992.hg.1	GPR126

TC06001046.hg.1	5.83	PSR06012237.hg.1	GPR126
TC06001046.hg.1	5.82	PSR06012275.hg.1	GPR126
TC06001046.hg.1	5.6	PSR06012258.hg.1	GPR126
TC06001046.hg.1	5.49	PSR06012271.hg.1	GPR126
TC06001046.hg.1	5.44	PSR06012249.hg.1	GPR126
TC06001046.hg.1	5.43	JUC06005988.hg.1	GPR126
TC06001046.hg.1	5.29	JUC06005999.hg.1	GPR126
TC06001046.hg.1	4.45	JUC06006005.hg.1	GPR126
TC06001046.hg.1	3.99	PSR06012267.hg.1	GPR126
TC06001046.hg.1	2.69	JUC06005993.hg.1	GPR126
TC06001046.hg.1	2.18	JUC06006003.hg.1	GPR126
TC06001046.hg.1	2.07	PSR06012265.hg.1	GPR126
TC06001046.hg.1	2.03	JUC06006010.hg.1	GPR126
TC06001046.hg.1	-2.45	JUC06006013.hg.1	GPR126
TC06001046.hg.1	-2.66	JUC06006000.hg.1	GPR126
TC06001046.hg.1	-3.33	JUC06005995.hg.1	GPR126
TC07003293.hg.1	8.66	JUC07020378.hg.1	HILPDA
TC07003293.hg.1	7.23	JUC07020373.hg.1	HILPDA
TC07003293.hg.1	4.41	PSR07012185.hg.1	HILPDA
TC07003293.hg.1	3.36	PSR07012201.hg.1	HILPDA
TC07003293.hg.1	3.12	JUC07020374.hg.1	HILPDA
TC07003293.hg.1	2.39	JUC07020376.hg.1	HILPDA
TC07003293.hg.1	-2.7	PSR07012203.hg.1	HILPDA
TC07003293.hg.1	-3.21	PSR07012198.hg.1	HILPDA
TC02002002.hg.1	8.65	PSR02032182.hg.1	LOXL3
TC02002002.hg.1	8.1	JUC02016631.hg.1	LOXL3
TC02002002.hg.1	7.79	JUC02016626.hg.1	LOXL3
TC02002002.hg.1	4.58	PSR02032162.hg.1	LOXL3
TC02002002.hg.1	4.16	PSR02032187.hg.1	LOXL3
TC02002002.hg.1	4.16	JUC02016623.hg.1	LOXL3
TC02002002.hg.1	3.78	PSR02032178.hg.1	LOXL3
TC02002002.hg.1	3.55	PSR02032190.hg.1	LOXL3
TC02002002.hg.1	3.09	PSR02032158.hg.1	LOXL3
TC02002002.hg.1	3.04	JUC02016628.hg.1	LOXL3
TC02002002.hg.1	2.67	PSR02032159.hg.1	LOXL3
TC02002002.hg.1	2.59	JUC02016636.hg.1	LOXL3
TC02002002.hg.1	2.53	PSR02032160.hg.1	LOXL3
TC02002002.hg.1	2.11	JUC02016622.hg.1	LOXL3
TC05001851.hg.1	8.6	PSR05025367.hg.1	NRG2
TC05001851.hg.1	3	JUC05012988.hg.1	NRG2
TC05001851.hg.1	2.92	JUC05012983.hg.1	NRG2
TC05001851.hg.1	2.6	JUC05013004.hg.1	NRG2
TC05001851.hg.1	-3.28	JUC05012989.hg.1	NRG2
TC10002925.hg.1	8.6	PSR10000348.hg.1	AKR1C3
TC10002925.hg.1	8.43	PSR10000356.hg.1	AKR1C3
TC10002925.hg.1	7.48	PSR10000354.hg.1	AKR1C3
TC10002925.hg.1	7.39	PSR10000353.hg.1	AKR1C3
TC10002925.hg.1	6.71	PSR10000351.hg.1	AKR1C3
TC10002925.hg.1	6.53	PSR10000352.hg.1	AKR1C3
TC10002925.hg.1	6.23	JUC10016973.hg.1	AKR1C3
TC10002925.hg.1	6.11	PSR10000349.hg.1	AKR1C3
TC10002925.hg.1	4.89	PSR10000370.hg.1	AKR1C3
TC10002925.hg.1	4.53	PSR10000364.hg.1	AKR1C3
TC10002925.hg.1	3.83	JUC10016983.hg.1	AKR1C3
TC10002925.hg.1	2.58	PSR10000357.hg.1	AKR1C3
TC10002925.hg.1	2.4	JUC10016972.hg.1	AKR1C3
TC10002925.hg.1	2.34	JUC10016977.hg.1	AKR1C3
TC10002925.hg.1	2.29	JUC10016981.hg.1	AKR1C3

TC10002925.hg.1	-2.21	JUC10016984.hg.1	AKR1C3
TC10002925.hg.1	-2.21	JUC10016985.hg.1	AKR1C3
TC10002925.hg.1	-2.23	PSR10000369.hg.1	AKR1C3
TC01001106.hg.1	8.58	JUC01008923.hg.1	LOC1005060
TC01001106.hg.1	2.59	JUC01008898.hg.1	LOC1005060
TC01001106.hg.1	2.21	PSR01017522.hg.1	LOC1005060
TC01001106.hg.1	2.14	JUC01008910.hg.1	LOC1005060
TC01001106.hg.1	-2.18	JUC01008901.hg.1	LOC1005060
TC01001106.hg.1	-2.5	JUC01008914.hg.1	LOC1005060
TC02001557.hg.1	8.48	PSR02025178.hg.1	ITGB1BP1
TC02001557.hg.1	6.51	PSR02025177.hg.1	ITGB1BP1
TC02001557.hg.1	3.03	JUC02013066.hg.1	ITGB1BP1
TC02001557.hg.1	2.09	JUC02013053.hg.1	ITGB1BP1
TC07001276.hg.1	8.48	JUC07009693.hg.1	TBX20
TC07001276.hg.1	2.06	PSR07019866.hg.1	TBX20
TC07001276.hg.1	-2.32	PSR07019862.hg.1	TBX20
TC07001276.hg.1	-2.7	PSR07019861.hg.1	TBX20
TC07001276.hg.1	-4.19	JUC07009695.hg.1	TBX20
TC07001276.hg.1	-4.59	JUC07009696.hg.1	TBX20
TC13000561.hg.1	8.45	PSR13006310.hg.1	MAB21L1, MI
TC13000561.hg.1	5.83	PSR13006309.hg.1	MAB21L1, MI
TC13000561.hg.1	3.98	JUC13003711.hg.1	MAB21L1, MI
TC13000561.hg.1	3.49	PSR13006303.hg.1	MAB21L1, MI
TC6_mann_hap40(	8.45	JUC6_mann_hap400	HLA-B
TC6_mann_hap40(	4.95	PSR6_mann_hap40C	HLA-B
TC6_mann_hap40(	3.66	JUC6_mann_hap400	HLA-B
TC6_mann_hap40(	3.37	PSR6_mann_hap40C	HLA-B
TC6_mann_hap40(	2.82	JUC6_mann_hap400	HLA-B
TC6_mann_hap40(	2.75	JUC6_mann_hap400	HLA-B
TC6_mann_hap40(	2.74	PSR6_mann_hap40C	HLA-B
TC6_mann_hap40(	2.53	PSR6_mann_hap40C	HLA-B
TC6_mann_hap40(	2.52	PSR6_mann_hap40C	HLA-B
TC6_mann_hap40(	2.42	PSR6_mann_hap40C	HLA-B
TC6_mann_hap40(	2.35	PSR6_mann_hap40C	HLA-B
TC6_mann_hap40(	2.21	PSR6_mann_hap40C	HLA-B
TC6_mann_hap40(	2.21	PSR6_mann_hap40C	HLA-B
TC6_cox_hap2000	8.44	JUC6_cox_hap2001ε	HLA-B
TC6_cox_hap2000	5.57	PSR6_cox_hap2002	HLA-B
TC6_cox_hap2000	3.66	JUC6_cox_hap2001ε	HLA-B
TC6_cox_hap2000	3.18	PSR6_cox_hap2002	HLA-B
TC6_cox_hap2000	2.75	JUC6_cox_hap2001ε	HLA-B
TC6_cox_hap2000	2.3	PSR6_cox_hap2002	HLA-B
TC6_cox_hap2000	2.07	PSR6_cox_hap2002	HLA-B
TC6_cox_hap2000	2	PSR6_cox_hap2002	HLA-B
TC07001163.hg.1	8.41	JUC07008885.hg.1	AGR2
TC07001163.hg.1	3.46	PSR07018203.hg.1	AGR2
TC07001163.hg.1	2.36	PSR07018194.hg.1	AGR2
TC07001163.hg.1	2.23	PSR07018198.hg.1	AGR2
TC07001163.hg.1	2.22	PSR07018196.hg.1	AGR2
TC07001163.hg.1	2.21	PSR07018189.hg.1	AGR2
TC07001163.hg.1	2.03	PSR07018213.hg.1	AGR2
TC10001216.hg.1	8.41	JUC10008791.hg.1	C10orf10
TC10001216.hg.1	4.89	PSR10015195.hg.1	C10orf10
TC10001216.hg.1	4.45	PSR10015181.hg.1	C10orf10
TC01000785.hg.1	8.4	JUC01006579.hg.1	AK5
TC01000785.hg.1	6.85	JUC01006573.hg.1	AK5
TC01000785.hg.1	4.26	PSR01012502.hg.1	AK5
TC01000785.hg.1	3.83	PSR01012501.hg.1	AK5

TC01000785.hg.1	3.34	PSR01012526.hg.1	AK5
TC01000785.hg.1	3.33	JUC01006574.hg.1	AK5
TC01000785.hg.1	3.09	PSR01012500.hg.1	AK5
TC01000785.hg.1	2.99	PSR01012515.hg.1	AK5
TC01000785.hg.1	2.97	PSR01012506.hg.1	AK5
TC01000785.hg.1	2.48	JUC01006575.hg.1	AK5
TC01000785.hg.1	2.31	PSR01012510.hg.1	AK5
TC01000785.hg.1	-2.03	PSR01012522.hg.1	AK5
TC01000785.hg.1	-2.07	JUC01006567.hg.1	AK5
TC01000785.hg.1	-2.19	JUC01006565.hg.1	AK5
TC01000785.hg.1	-2.24	JUC01006571.hg.1	AK5
TC01000785.hg.1	-2.35	PSR01012518.hg.1	AK5
TC01000785.hg.1	-2.36	PSR01012521.hg.1	AK5
TC01000785.hg.1	-2.77	PSR01012517.hg.1	AK5
TC01000785.hg.1	-3.84	PSR01012520.hg.1	AK5
TC01000785.hg.1	-3.96	JUC01006566.hg.1	AK5
TC01000785.hg.1	-4.5	JUC01006564.hg.1	AK5
TC12001960.hg.1	8.4	PSR12025515.hg.1	C12orf76
TC12001960.hg.1	5.03	JUC12014254.hg.1	C12orf76
TC12001960.hg.1	4.05	PSR12025514.hg.1	C12orf76
TC12001960.hg.1	3.81	PSR12025504.hg.1	C12orf76
TC12001960.hg.1	3.35	JUC12014240.hg.1	C12orf76
TC12001960.hg.1	3.29	PSR12025507.hg.1	C12orf76
TC12001960.hg.1	3.26	PSR12025492.hg.1	C12orf76
TC12001960.hg.1	3.25	PSR12025511.hg.1	C12orf76
TC12001960.hg.1	3.13	PSR12025501.hg.1	C12orf76
TC12001960.hg.1	3.02	JUC12014248.hg.1	C12orf76
TC12001960.hg.1	2.73	JUC12014249.hg.1	C12orf76
TC12001960.hg.1	2.72	PSR12025506.hg.1	C12orf76
TC12001960.hg.1	2.64	PSR12025510.hg.1	C12orf76
TC12001960.hg.1	2.54	JUC12014255.hg.1	C12orf76
TC12001960.hg.1	2.52	PSR12025502.hg.1	C12orf76
TC12001960.hg.1	2.5	PSR12025491.hg.1	C12orf76
TC12001960.hg.1	2.46	PSR12025508.hg.1	C12orf76
TC12001960.hg.1	2.25	PSR12025493.hg.1	C12orf76
TC12001960.hg.1	2.25	PSR12025498.hg.1	C12orf76
TC10000593.hg.1	8.36	JUC10003740.hg.1	CDHR1
TC10000593.hg.1	2.17	JUC10003739.hg.1	CDHR1
TC10000593.hg.1	-2.3	JUC10003733.hg.1	CDHR1
TC10000593.hg.1	-4.36	PSR10006459.hg.1	CDHR1
TC21000400.hg.1	8.32	JUC21002358.hg.1	SYNJ1
TC21000400.hg.1	4.38	JUC21002366.hg.1	SYNJ1
TC21000400.hg.1	3.46	JUC21002374.hg.1	SYNJ1
TC21000400.hg.1	2.88	JUC21002357.hg.1	SYNJ1
TC21000400.hg.1	2.77	PSR21004525.hg.1	SYNJ1
TC21000400.hg.1	2.47	PSR21004489.hg.1	SYNJ1
TC21000400.hg.1	2.43	JUC21002376.hg.1	SYNJ1
TC21000400.hg.1	2.41	PSR21004487.hg.1	SYNJ1
TC21000400.hg.1	2.37	PSR21004488.hg.1	SYNJ1
TC21000400.hg.1	2.26	PSR21004491.hg.1	SYNJ1
TC21000400.hg.1	2.19	JUC21002391.hg.1	SYNJ1
TC21000400.hg.1	2.14	PSR21004524.hg.1	SYNJ1
TC21000400.hg.1	-2.81	JUC21002399.hg.1	SYNJ1
TC21000400.hg.1	-2.99	JUC21002377.hg.1	SYNJ1
TC21000400.hg.1	-4.31	JUC21002386.hg.1	SYNJ1
TC02004994.hg.1	8.31	JUC02031859.hg.1	IL1RL1
TC02004994.hg.1	6.37	PSR02009898.hg.1	IL1RL1
TC02004994.hg.1	4.52	PSR02009907.hg.1	IL1RL1

TC02004994.hg.1	4.45	PSR02009906.hg.1	IL1RL1
TC02004994.hg.1	3.84	PSR02009905.hg.1	IL1RL1
TC02004994.hg.1	3.79	PSR02009877.hg.1	IL1RL1
TC02004994.hg.1	3.76	PSR02009904.hg.1	IL1RL1
TC02004994.hg.1	3.49	PSR02009908.hg.1	IL1RL1
TC02004994.hg.1	3.4	PSR02009885.hg.1	IL1RL1
TC02004994.hg.1	2.79	PSR02009879.hg.1	IL1RL1
TC02004994.hg.1	2.43	PSR02009874.hg.1	IL1RL1
TC02004994.hg.1	2.02	PSR02009897.hg.1	IL1RL1
TC02004994.hg.1	-2.71	JUC02031874.hg.1	IL1RL1
TC17000636.hg.1	8.27	JUC17004403.hg.1	IGF2BP1
TC17000636.hg.1	3.13	JUC17004414.hg.1	IGF2BP1
TC17000636.hg.1	2.4	JUC17004408.hg.1	IGF2BP1
TC17000636.hg.1	-2.06	PSR17007948.hg.1	IGF2BP1
TC17000636.hg.1	-2.24	PSR17007961.hg.1	IGF2BP1
TC17000636.hg.1	-2.28	PSR17007952.hg.1	IGF2BP1
TC01002884.hg.1	8.26	JUC01024205.hg.1	ARHGAP29
TC01002884.hg.1	6.62	PSR01045040.hg.1	ARHGAP29
TC01002884.hg.1	6.2	PSR01045074.hg.1	ARHGAP29
TC01002884.hg.1	5.4	PSR01045039.hg.1	ARHGAP29
TC01002884.hg.1	4.98	PSR01045073.hg.1	ARHGAP29
TC01002884.hg.1	4.21	PSR01045046.hg.1	ARHGAP29
TC01002884.hg.1	4.06	PSR01045038.hg.1	ARHGAP29
TC01002884.hg.1	3.57	JUC01024220.hg.1	ARHGAP29
TC01002884.hg.1	2.4	JUC01024209.hg.1	ARHGAP29
TC01002884.hg.1	2.26	JUC01024213.hg.1	ARHGAP29
TC01002884.hg.1	-2.25	JUC01024221.hg.1	ARHGAP29
TC01000972.hg.1	8.25	JUC01008101.hg.1	CHI3L2
TC01000972.hg.1	5.99	PSR01015486.hg.1	CHI3L2
TC01000972.hg.1	5.29	PSR01015483.hg.1	CHI3L2
TC01000972.hg.1	5.11	JUC01008091.hg.1	CHI3L2
TC01000972.hg.1	5.05	PSR01015473.hg.1	CHI3L2
TC01000972.hg.1	5.01	PSR01015500.hg.1	CHI3L2
TC01000972.hg.1	4.27	JUC01008095.hg.1	CHI3L2
TC01000972.hg.1	4.14	PSR01015504.hg.1	CHI3L2
TC01000972.hg.1	3.98	JUC01008105.hg.1	CHI3L2
TC01000972.hg.1	3.71	PSR01015481.hg.1	CHI3L2
TC01000972.hg.1	2.66	PSR01015484.hg.1	CHI3L2
TC01000972.hg.1	-2.74	JUC01008096.hg.1	CHI3L2
TC03001427.hg.1	8.24	PSR03025664.hg.1	HYAL1
TC03001427.hg.1	3.75	JUC03012572.hg.1	HYAL1
TC03001427.hg.1	3.29	JUC03012577.hg.1	HYAL1
TC03001427.hg.1	2.57	PSR03025665.hg.1	HYAL1
TC03001427.hg.1	2.53	PSR03025666.hg.1	HYAL1
TC03001427.hg.1	2.39	PSR03025658.hg.1	HYAL1
TC03001427.hg.1	2.34	PSR03025651.hg.1	HYAL1
TC03001427.hg.1	2.29	PSR03025659.hg.1	HYAL1
TC03001427.hg.1	2.14	PSR03025667.hg.1	HYAL1
TC03001427.hg.1	2.08	PSR03025668.hg.1	HYAL1
TC03001427.hg.1	2.01	PSR03025652.hg.1	HYAL1
TC03001427.hg.1	-3.09	PSR03025649.hg.1	HYAL1
TC03001427.hg.1	-6.97	JUC03012573.hg.1	HYAL1
TC02000230.hg.1	8.23	JUC02001778.hg.1	CRIM1
TC02000230.hg.1	3.35	JUC02001789.hg.1	CRIM1
TC02000230.hg.1	3.33	JUC02001791.hg.1	CRIM1
TC02000230.hg.1	3.21	JUC02001787.hg.1	CRIM1
TC02000230.hg.1	2.23	PSR02003383.hg.1	CRIM1
TC02000230.hg.1	-2.06	JUC02001784.hg.1	CRIM1

TC02000230.hg.1	-2.71	PSR02003414.hg.1	CRIM1
TC02000230.hg.1	-2.96	JUC02001771.hg.1	CRIM1
TC06001313.hg.1	8.2	PSR06015735.hg.1	MBOAT1
TC06001313.hg.1	6.64	PSR06015716.hg.1	MBOAT1
TC06001313.hg.1	5.84	PSR06015734.hg.1	MBOAT1
TC06001313.hg.1	5.79	JUC06007851.hg.1	MBOAT1
TC06001313.hg.1	5.4	JUC06007857.hg.1	MBOAT1
TC06001313.hg.1	5.37	JUC06007860.hg.1	MBOAT1
TC06001313.hg.1	5.37	JUC06007861.hg.1	MBOAT1
TC06001313.hg.1	5.34	JUC06007849.hg.1	MBOAT1
TC06001313.hg.1	3.22	JUC06007845.hg.1	MBOAT1
TC06001313.hg.1	3.2	JUC06007846.hg.1	MBOAT1
TC06001313.hg.1	2.33	JUC06007859.hg.1	MBOAT1
TC06001313.hg.1	2.25	JUC06007850.hg.1	MBOAT1
TC06001313.hg.1	2.16	PSR06015721.hg.1	MBOAT1
TC06001313.hg.1	2.12	PSR06015715.hg.1	MBOAT1
TC01003266.hg.1	8.19	PSR01050262.hg.1	S100A4
TC01003266.hg.1	7.09	PSR01050254.hg.1	S100A4
TC01003266.hg.1	6.08	JUC01026573.hg.1	S100A4
TC01003266.hg.1	5.85	PSR01050261.hg.1	S100A4
TC01003266.hg.1	5.69	PSR01050255.hg.1	S100A4
TC01003266.hg.1	3.43	PSR01050256.hg.1	S100A4
TC01000323.hg.1	8.14	JUC01002639.hg.1	LDLRAP1
TC01000323.hg.1	2.79	PSR01004937.hg.1	LDLRAP1
TC01000323.hg.1	2.76	PSR01004938.hg.1	LDLRAP1
TC01000323.hg.1	2.49	JUC01002638.hg.1	LDLRAP1
TC01000323.hg.1	2.19	PSR01004944.hg.1	LDLRAP1
TC01000323.hg.1	2.05	PSR01004942.hg.1	LDLRAP1
TC06001355.hg.1	8.1	JUC06008083.hg.1	HIST1H4H, H
TC06001355.hg.1	2.82	PSR06016180.hg.1	HIST1H4H, H
TC06001355.hg.1	2.18	PSR06016179.hg.1	HIST1H4H, H
TC08001659.hg.1	8.03	JUC08011053.hg.1	NDRG1
TC08001659.hg.1	5.17	PSR08021681.hg.1	NDRG1
TC08001659.hg.1	5.08	PSR08021677.hg.1	NDRG1
TC08001659.hg.1	5.05	PSR08021688.hg.1	NDRG1
TC08001659.hg.1	4	PSR08021689.hg.1	NDRG1
TC08001659.hg.1	3.94	JUC08011041.hg.1	NDRG1
TC08001659.hg.1	3.76	PSR08021680.hg.1	NDRG1
TC08001659.hg.1	3.74	PSR08021691.hg.1	NDRG1
TC08001659.hg.1	3.63	JUC08011051.hg.1	NDRG1
TC08001659.hg.1	3.45	PSR08021675.hg.1	NDRG1
TC08001659.hg.1	3.38	JUC08011042.hg.1	NDRG1
TC08001659.hg.1	3.35	JUC08011052.hg.1	NDRG1
TC08001659.hg.1	3.34	JUC08011054.hg.1	NDRG1
TC08001659.hg.1	3.3	JUC08011074.hg.1	NDRG1
TC08001659.hg.1	3.28	PSR08021690.hg.1	NDRG1
TC08001659.hg.1	3.02	JUC08011047.hg.1	NDRG1
TC08001659.hg.1	2.86	PSR08021679.hg.1	NDRG1
TC08001659.hg.1	2.47	JUC08011067.hg.1	NDRG1
TC08001659.hg.1	2.43	JUC08011048.hg.1	NDRG1
TC08001659.hg.1	2.42	JUC08011043.hg.1	NDRG1
TC08001659.hg.1	2.29	JUC08011070.hg.1	NDRG1
TC08001659.hg.1	2.09	PSR08021674.hg.1	NDRG1
TC08001659.hg.1	2.03	JUC08011049.hg.1	NDRG1
TC08001659.hg.1	-2	PSR08021612.hg.1	NDRG1
TC08001659.hg.1	-2.02	PSR08021630.hg.1	NDRG1
TC08001659.hg.1	-2.38	PSR08021664.hg.1	NDRG1
TC08001659.hg.1	-2.75	PSR08021633.hg.1	NDRG1

TC03001673.hg.1	7.96	JUC03015000.hg.1	LSAMP
TC03001673.hg.1	5.56	JUC03015005.hg.1	LSAMP
TC03001673.hg.1	4.92	PSR03030207.hg.1	LSAMP
TC03001673.hg.1	4.88	JUC03015006.hg.1	LSAMP
TC03001673.hg.1	4.63	PSR03030206.hg.1	LSAMP
TC03001673.hg.1	4.07	PSR03030212.hg.1	LSAMP
TC03001673.hg.1	3.83	PSR03030208.hg.1	LSAMP
TC03001673.hg.1	3.48	PSR03030202.hg.1	LSAMP
TC03001673.hg.1	3.32	PSR03030210.hg.1	LSAMP
TC03001673.hg.1	3.2	PSR03030191.hg.1	LSAMP
TC03001673.hg.1	2.99	PSR03030204.hg.1	LSAMP
TC03001673.hg.1	2.77	JUC03015010.hg.1	LSAMP
TC03001673.hg.1	2.65	PSR03030213.hg.1	LSAMP
TC03001673.hg.1	2.51	PSR03030211.hg.1	LSAMP
TC03001673.hg.1	2.39	JUC03015003.hg.1	LSAMP
TC03001673.hg.1	2.17	PSR03030214.hg.1	LSAMP
TC03001673.hg.1	-2.54	JUC03015007.hg.1	LSAMP
TC16001225.hg.1	7.95	PSR16016942.hg.1	NQO1
TC16001225.hg.1	5.97	PSR16016943.hg.1	NQO1
TC16001225.hg.1	2.85	JUC16009538.hg.1	NQO1
TC16001225.hg.1	2.32	PSR16016940.hg.1	NQO1
TC16001225.hg.1	2.29	JUC16009545.hg.1	NQO1
TC16001225.hg.1	-2.04	JUC16009540.hg.1	NQO1
TC16001225.hg.1	-2.16	JUC16009544.hg.1	NQO1
TC16001225.hg.1	-3.37	JUC16009543.hg.1	NQO1
TC17002889.hg.1	7.94	JUC17018847.hg.1	TRIM16
TC17002889.hg.1	6.89	PSR17015793.hg.1	TRIM16
TC17002889.hg.1	5.35	PSR17015791.hg.1	TRIM16
TC17002889.hg.1	4.94	JUC17018853.hg.1	TRIM16
TC17002889.hg.1	4.41	PSR17015804.hg.1	TRIM16
TC17002889.hg.1	4.27	JUC17018857.hg.1	TRIM16
TC17002889.hg.1	4.25	PSR17015798.hg.1	TRIM16
TC17002889.hg.1	4.18	PSR17015799.hg.1	TRIM16
TC17002889.hg.1	3.74	PSR17015800.hg.1	TRIM16
TC17002889.hg.1	3.42	PSR17015801.hg.1	TRIM16
TC17002889.hg.1	3.32	JUC17018848.hg.1	TRIM16
TC17002889.hg.1	2.93	PSR17015795.hg.1	TRIM16
TC17002889.hg.1	2.66	PSR17015802.hg.1	TRIM16
TC17002889.hg.1	2.63	PSR17015803.hg.1	TRIM16
TC17002889.hg.1	2.36	PSR17015796.hg.1	TRIM16
TC17002889.hg.1	2.19	PSR17015797.hg.1	TRIM16
TC17002889.hg.1	2.19	JUC17018844.hg.1	TRIM16
TC17002889.hg.1	2.15	PSR17015790.hg.1	TRIM16
TC17002889.hg.1	-2.24	PSR17015784.hg.1	TRIM16
TC10000381.hg.1	7.92	JUC10002504.hg.1	ZNF365
TC10000381.hg.1	5.08	JUC10002496.hg.1	ZNF365
TC10000381.hg.1	4.17	PSR10004319.hg.1	ZNF365
TC10000381.hg.1	3.29	JUC10002495.hg.1	ZNF365
TC10000381.hg.1	2.77	PSR10004316.hg.1	ZNF365
TC10000381.hg.1	-3.45	PSR10004310.hg.1	ZNF365
TC01006305.hg.1	7.91	JUC01039746.hg.1	YY1AP1
TC01006305.hg.1	2.88	JUC01039752.hg.1	YY1AP1
TC01006305.hg.1	2.84	PSR01051582.hg.1	YY1AP1
TC01006305.hg.1	2.66	PSR01051516.hg.1	YY1AP1
TC01006305.hg.1	2.41	JUC01039759.hg.1	YY1AP1
TC01006305.hg.1	2.27	JUC01039731.hg.1	YY1AP1
TC01006305.hg.1	-2.37	JUC01039738.hg.1	YY1AP1
TC01006305.hg.1	-2.96	PSR01051544.hg.1	YY1AP1

TC17000241.hg.1	7.91	JUC17001949.hg.1	SLC47A1
TC17000241.hg.1	6.24	PSR17003384.hg.1	SLC47A1
TC17000241.hg.1	5.9	PSR17003370.hg.1	SLC47A1
TC17000241.hg.1	4.87	PSR17003386.hg.1	SLC47A1
TC17000241.hg.1	4.62	JUC17001927.hg.1	SLC47A1
TC17000241.hg.1	4.16	PSR17003381.hg.1	SLC47A1
TC17000241.hg.1	4.08	JUC17001940.hg.1	SLC47A1
TC17000241.hg.1	3.55	PSR17003385.hg.1	SLC47A1
TC17000241.hg.1	3.46	JUC17001928.hg.1	SLC47A1
TC17000241.hg.1	3.43	PSR17003401.hg.1	SLC47A1
TC17000241.hg.1	3.43	JUC17001953.hg.1	SLC47A1
TC17000241.hg.1	3.17	JUC17001933.hg.1	SLC47A1
TC17000241.hg.1	3.02	PSR17003374.hg.1	SLC47A1
TC17000241.hg.1	2.91	PSR17003389.hg.1	SLC47A1
TC17000241.hg.1	2.79	JUC17001929.hg.1	SLC47A1
TC17000241.hg.1	2.74	PSR17003405.hg.1	SLC47A1
TC17000241.hg.1	2.26	PSR17003379.hg.1	SLC47A1
TC17000241.hg.1	-2.11	JUC17001936.hg.1	SLC47A1
TC17000241.hg.1	-2.12	PSR17003375.hg.1	SLC47A1
TC17000241.hg.1	-2.52	PSR17003398.hg.1	SLC47A1
TC17000241.hg.1	-2.57	PSR17003397.hg.1	SLC47A1
TC17000241.hg.1	-2.59	PSR17003399.hg.1	SLC47A1
TC17000241.hg.1	-2.87	JUC17001931.hg.1	SLC47A1
TC17000241.hg.1	-3.12	PSR17003378.hg.1	SLC47A1
TC17000241.hg.1	-3.45	JUC17001938.hg.1	SLC47A1
TC17000241.hg.1	-4.79	JUC17001946.hg.1	SLC47A1
TC19001582.hg.1	7.88	JUC19012346.hg.1	PSG5
TC19001582.hg.1	6.14	JUC19012349.hg.1	PSG5
TC19001582.hg.1	5.91	PSR19021660.hg.1	PSG5
TC19001582.hg.1	5.54	PSR19021659.hg.1	PSG5
TC19001582.hg.1	5.53	JUC19012352.hg.1	PSG5
TC19001582.hg.1	5.34	PSR19021668.hg.1	PSG5
TC19001582.hg.1	5.1	PSR19021669.hg.1	PSG5
TC19001582.hg.1	5.03	PSR19021675.hg.1	PSG5
TC19001582.hg.1	4.44	JUC19012350.hg.1	PSG5
TC19001582.hg.1	4.37	PSR19021667.hg.1	PSG5
TC19001582.hg.1	4.25	PSR19021670.hg.1	PSG5
TC19001582.hg.1	3.13	PSR19021663.hg.1	PSG5
TC19001582.hg.1	2.78	JUC19012359.hg.1	PSG5
TC19001582.hg.1	-2.03	JUC19012357.hg.1	PSG5
TC16000186.hg.1	7.81	JUC16001430.hg.1	PDXDC1
TC16000186.hg.1	4.65	JUC16001414.hg.1	PDXDC1
TC16000186.hg.1	2.02	PSR16002809.hg.1	PDXDC1
TC16000186.hg.1	-2.17	JUC16001412.hg.1	PDXDC1
TC16000186.hg.1	-2.48	JUC16001418.hg.1	PDXDC1
TC03000297.hg.1	7.78	JUC03003040.hg.1	APEH
TC03000297.hg.1	-2.11	PSR03006060.hg.1	APEH
TC03000297.hg.1	-2.52	JUC03003035.hg.1	APEH
TC06004059.hg.1	7.78	JUC06020787.hg.1	HLA-C
TC06004059.hg.1	2.69	PSR06017338.hg.1	HLA-C
TC06004059.hg.1	2.68	PSR06017314.hg.1	HLA-C
TC06004059.hg.1	2.55	JUC06020796.hg.1	HLA-C
TC06004059.hg.1	2.37	JUC06020793.hg.1	HLA-C
TC06004059.hg.1	2.02	PSR06017311.hg.1	HLA-C
TC06004059.hg.1	-3.2	JUC06020786.hg.1	HLA-C
TC04002924.hg.1	7.77	PSR04019961.hg.1	SLC39A8
TC04002924.hg.1	4.15	PSR04019957.hg.1	SLC39A8
TC04002924.hg.1	3.66	PSR04019939.hg.1	SLC39A8

TC04002924.hg.1	3.57	PSR04019955.hg.1	SLC39A8
TC04002924.hg.1	3.54	PSR04019945.hg.1	SLC39A8
TC04002924.hg.1	2.95	PSR04019938.hg.1	SLC39A8
TC04002924.hg.1	2.58	JUC04016969.hg.1	SLC39A8
TC04002924.hg.1	2.21	PSR04019956.hg.1	SLC39A8
TC04002924.hg.1	2.05	PSR04019959.hg.1	SLC39A8
TC08002614.hg.1	7.76	JUC08015219.hg.1	ZNF395
TC08002614.hg.1	4.93	PSR08014006.hg.1	ZNF395
TC08002614.hg.1	4.91	JUC08015216.hg.1	ZNF395
TC08002614.hg.1	4.83	JUC08015211.hg.1	ZNF395
TC08002614.hg.1	4.72	JUC08015224.hg.1	ZNF395
TC08002614.hg.1	4.35	JUC08015222.hg.1	ZNF395
TC08002614.hg.1	4.33	PSR08013999.hg.1	ZNF395
TC08002614.hg.1	3.84	JUC08015212.hg.1	ZNF395
TC08002614.hg.1	3.79	PSR08014002.hg.1	ZNF395
TC08002614.hg.1	3.71	JUC08015215.hg.1	ZNF395
TC08002614.hg.1	3.6	JUC08015223.hg.1	ZNF395
TC08002614.hg.1	3.52	JUC08015229.hg.1	ZNF395
TC08002614.hg.1	3.42	PSR08014005.hg.1	ZNF395
TC08002614.hg.1	3.39	JUC08015230.hg.1	ZNF395
TC08002614.hg.1	3.36	PSR08013994.hg.1	ZNF395
TC08002614.hg.1	3.03	PSR08013985.hg.1	ZNF395
TC08002614.hg.1	3.01	JUC08015218.hg.1	ZNF395
TC08002614.hg.1	2.62	JUC08015214.hg.1	ZNF395
TC08002614.hg.1	2.35	PSR08013997.hg.1	ZNF395
TC08002614.hg.1	2.24	JUC08015231.hg.1	ZNF395
TC08002614.hg.1	-2.11	JUC08015225.hg.1	ZNF395
TC08002614.hg.1	-2.16	PSR08013978.hg.1	ZNF395
TC08002614.hg.1	-2.35	JUC08015226.hg.1	ZNF395
TC08002614.hg.1	-2.94	PSR08013981.hg.1	ZNF395
TC08002614.hg.1	-3.44	JUC08015228.hg.1	ZNF395
TC08002614.hg.1	-4.12	JUC08015232.hg.1	ZNF395
TC01002179.hg.1	7.75	JUC01017995.hg.1	SLC2A5
TC01002179.hg.1	4.21	JUC01018000.hg.1	SLC2A5
TC01002179.hg.1	3.84	JUC01018016.hg.1	SLC2A5
TC01002179.hg.1	2.7	JUC01018010.hg.1	SLC2A5
TC01002179.hg.1	2.18	PSR01033568.hg.1	SLC2A5
TC01002179.hg.1	2.1	PSR01033545.hg.1	SLC2A5
TC01002179.hg.1	2.07	PSR01033588.hg.1	SLC2A5
TC01002179.hg.1	2.02	JUC01017997.hg.1	SLC2A5
TC01002179.hg.1	2.01	JUC01017998.hg.1	SLC2A5
TC02000184.hg.1	7.74	JUC02001312.hg.1	PLB1
TC02000184.hg.1	6.68	PSR02002638.hg.1	PLB1
TC02000184.hg.1	4.12	PSR02002644.hg.1	PLB1
TC02000184.hg.1	4.06	PSR02002633.hg.1	PLB1
TC02000184.hg.1	3.71	JUC02001293.hg.1	PLB1
TC02000184.hg.1	3.5	JUC02001316.hg.1	PLB1
TC02000184.hg.1	3.08	JUC02001315.hg.1	PLB1
TC02000184.hg.1	2.94	PSR02002642.hg.1	PLB1
TC02000184.hg.1	2.79	JUC02001327.hg.1	PLB1
TC02000184.hg.1	2.41	JUC02001289.hg.1	PLB1
TC02000184.hg.1	2.31	PSR02002643.hg.1	PLB1
TC02000184.hg.1	2.24	JUC02001307.hg.1	PLB1
TC02000184.hg.1	2.17	JUC02001313.hg.1	PLB1
TC02000184.hg.1	2.04	PSR02002607.hg.1	PLB1
TC02000184.hg.1	-2.05	PSR02002651.hg.1	PLB1
TC02000184.hg.1	-2.36	PSR02002630.hg.1	PLB1
TC02000184.hg.1	-2.38	JUC02001296.hg.1	PLB1

TC02000184.hg.1	-2.43	PSR02002623.hg.1	PLB1
TC02000184.hg.1	-2.44	PSR02002617.hg.1	PLB1
TC02000184.hg.1	-2.44	PSR02002652.hg.1	PLB1
TC02000184.hg.1	-2.61	PSR02002625.hg.1	PLB1
TC02000184.hg.1	-2.68	PSR02002597.hg.1	PLB1
TC02000184.hg.1	-2.71	PSR02002596.hg.1	PLB1
TC02000184.hg.1	-2.71	JUC02001328.hg.1	PLB1
TC02000184.hg.1	-2.77	PSR02002606.hg.1	PLB1
TC02000184.hg.1	-2.77	PSR02002628.hg.1	PLB1
TC02000184.hg.1	-2.82	JUC02001317.hg.1	PLB1
TC02000184.hg.1	-2.86	JUC02001283.hg.1	PLB1
TC02000184.hg.1	-2.88	PSR02002593.hg.1	PLB1
TC02000184.hg.1	-2.95	PSR02002586.hg.1	PLB1
TC02000184.hg.1	-3	PSR02002624.hg.1	PLB1
TC02000184.hg.1	-3	JUC02001290.hg.1	PLB1
TC02000184.hg.1	-3.01	PSR02002611.hg.1	PLB1
TC02000184.hg.1	-3.11	JUC02001311.hg.1	PLB1
TC02000184.hg.1	-3.16	JUC02001303.hg.1	PLB1
TC02000184.hg.1	-3.23	PSR02002582.hg.1	PLB1
TC02000184.hg.1	-3.59	PSR02002631.hg.1	PLB1
TC02000184.hg.1	-3.75	PSR02002635.hg.1	PLB1
TC02000184.hg.1	-3.81	PSR02002626.hg.1	PLB1
TC02000184.hg.1	-3.81	JUC02001338.hg.1	PLB1
TC02000184.hg.1	-3.85	JUC02001319.hg.1	PLB1
TC02000184.hg.1	-3.87	PSR02002573.hg.1	PLB1
TC02000184.hg.1	-3.92	PSR02002584.hg.1	PLB1
TC02000184.hg.1	-4.01	PSR02002629.hg.1	PLB1
TC02000184.hg.1	-4.08	JUC02001331.hg.1	PLB1
TC02000184.hg.1	-4.18	PSR02002616.hg.1	PLB1
TC02000184.hg.1	-4.23	JUC02001286.hg.1	PLB1
TC02000184.hg.1	-4.23	JUC02001298.hg.1	PLB1
TC02000184.hg.1	-4.27	JUC02001282.hg.1	PLB1
TC02000184.hg.1	-4.28	JUC02001285.hg.1	PLB1
TC02000184.hg.1	-4.33	PSR02002571.hg.1	PLB1
TC02000184.hg.1	-4.36	PSR02002581.hg.1	PLB1
TC02000184.hg.1	-4.39	PSR02002632.hg.1	PLB1
TC02000184.hg.1	-4.48	PSR02002609.hg.1	PLB1
TC02000184.hg.1	-4.49	PSR02002570.hg.1	PLB1
TC02000184.hg.1	-4.63	PSR02002591.hg.1	PLB1
TC02000184.hg.1	-4.77	PSR02002575.hg.1	PLB1
TC02000184.hg.1	-5.02	PSR02002602.hg.1	PLB1
TC02000184.hg.1	-5.3	JUC02001278.hg.1	PLB1
TC02000184.hg.1	-5.37	PSR02002569.hg.1	PLB1
TC02000184.hg.1	-5.52	PSR02002587.hg.1	PLB1
TC02000184.hg.1	-5.67	JUC02001329.hg.1	PLB1
TC02000184.hg.1	-6.02	JUC02001310.hg.1	PLB1
TC02000184.hg.1	-6.4	PSR02002590.hg.1	PLB1
TC02000184.hg.1	-6.55	PSR02002576.hg.1	PLB1
TC02000184.hg.1	-6.89	PSR02002579.hg.1	PLB1
TC02000184.hg.1	-6.95	PSR02002577.hg.1	PLB1
TC02000184.hg.1	-6.97	PSR02002585.hg.1	PLB1
TC02000184.hg.1	-7.67	PSR02002574.hg.1	PLB1
TC02000184.hg.1	-8.11	PSR02002589.hg.1	PLB1
TC0Y000052.hg.1	7.73	PSR0Y000610.hg.1	USP9Y
TC0Y000052.hg.1	4.84	PSR0Y000611.hg.1	USP9Y
TC0Y000052.hg.1	3.55	JUC0Y000264.hg.1	USP9Y
TC0Y000052.hg.1	2.81	JUC0Y000252.hg.1	USP9Y
TC0Y000052.hg.1	2.63	JUC0Y000254.hg.1	USP9Y

TC0Y000052.hg.1	2.39	JUC0Y000263.hg.1	USP9Y
TC0Y000052.hg.1	2.28	PSR0Y000596.hg.1	USP9Y
TC0Y000052.hg.1	2.08	JUC0Y000235.hg.1	USP9Y
TC0Y000052.hg.1	2	JUC0Y000273.hg.1	USP9Y
TC0Y000052.hg.1	-2.14	PSR0Y000606.hg.1	USP9Y
TC0Y000052.hg.1	-2.16	JUC0Y000246.hg.1	USP9Y
TC0Y000052.hg.1	-2.18	PSR0Y000583.hg.1	USP9Y
TC0Y000052.hg.1	-2.2	PSR0Y000585.hg.1	USP9Y
TC0Y000052.hg.1	-2.34	JUC0Y000245.hg.1	USP9Y
TC0Y000052.hg.1	-2.4	PSR0Y000576.hg.1	USP9Y
TC0Y000052.hg.1	-2.5	PSR0Y000584.hg.1	USP9Y
TC0Y000052.hg.1	-2.63	JUC0Y000269.hg.1	USP9Y
TC0Y000052.hg.1	-2.72	PSR0Y000575.hg.1	USP9Y
TC0Y000052.hg.1	-2.98	JUC0Y000281.hg.1	USP9Y
TC0Y000052.hg.1	-3.16	JUC0Y000259.hg.1	USP9Y
TC0Y000052.hg.1	-3.17	PSR0Y000605.hg.1	USP9Y
TC0Y000052.hg.1	-3.44	JUC0Y000241.hg.1	USP9Y
TC0Y000052.hg.1	-3.53	JUC0Y000238.hg.1	USP9Y
TC0Y000052.hg.1	-3.63	PSR0Y000582.hg.1	USP9Y
TC0Y000052.hg.1	-3.75	JUC0Y000247.hg.1	USP9Y
TC0Y000052.hg.1	-3.79	JUC0Y000278.hg.1	USP9Y
TC0Y000052.hg.1	-3.8	PSR0Y000580.hg.1	USP9Y
TC0Y000052.hg.1	-4.03	JUC0Y000262.hg.1	USP9Y
TC0Y000052.hg.1	-4.07	JUC0Y000240.hg.1	USP9Y
TC0Y000052.hg.1	-4.07	JUC0Y000268.hg.1	USP9Y
TC0Y000052.hg.1	-4.09	JUC0Y000266.hg.1	USP9Y
TC0Y000052.hg.1	-4.32	JUC0Y000242.hg.1	USP9Y
TC0Y000052.hg.1	-4.56	JUC0Y000275.hg.1	USP9Y
TC0Y000052.hg.1	-4.69	PSR0Y000598.hg.1	USP9Y
TC0Y000052.hg.1	-4.73	JUC0Y000279.hg.1	USP9Y
TC0Y000052.hg.1	-4.91	PSR0Y000617.hg.1	USP9Y
TC0Y000052.hg.1	-5.21	PSR0Y000568.hg.1	USP9Y
TC17002858.hg.1	7.73	PSR17007738.hg.1	HOXB-AS3
TC17002858.hg.1	5.01	JUC17018423.hg.1	HOXB-AS3
TC17002858.hg.1	2.59	JUC17018421.hg.1	HOXB-AS3
TC17002858.hg.1	2.13	PSR17007739.hg.1	HOXB-AS3
TC17002858.hg.1	-2.01	PSR17007734.hg.1	HOXB-AS3
TC17002858.hg.1	-2.31	PSR17007735.hg.1	HOXB-AS3
TC17002858.hg.1	-2.76	JUC17018413.hg.1	HOXB-AS3
TC17002858.hg.1	-2.81	JUC17018411.hg.1	HOXB-AS3
TC17002858.hg.1	-4.43	PSR17007736.hg.1	HOXB-AS3
TC10000826.hg.1	7.67	JUC10005569.hg.1	PLEKHS1
TC10000826.hg.1	5.95	JUC10005556.hg.1	PLEKHS1
TC10000826.hg.1	4.28	PSR10009968.hg.1	PLEKHS1
TC10000826.hg.1	2.77	JUC10005566.hg.1	PLEKHS1
TC10000826.hg.1	2.67	PSR10009984.hg.1	PLEKHS1
TC10000826.hg.1	2.33	JUC10005563.hg.1	PLEKHS1
TC10000826.hg.1	2.12	PSR10009958.hg.1	PLEKHS1
TC10000826.hg.1	-2.06	PSR10009974.hg.1	PLEKHS1
TC10000826.hg.1	-2.19	JUC10005560.hg.1	PLEKHS1
TC10000826.hg.1	-2.3	JUC10005562.hg.1	PLEKHS1
TC10000826.hg.1	-2.37	JUC10005567.hg.1	PLEKHS1
TC10000826.hg.1	-3.46	JUC10005557.hg.1	PLEKHS1
TC19001250.hg.1	7.64	JUC19010219.hg.1	NOTCH3
TC19001250.hg.1	6.59	JUC19010198.hg.1	NOTCH3
TC19001250.hg.1	5.02	JUC19010217.hg.1	NOTCH3
TC19001250.hg.1	4.54	JUC19010201.hg.1	NOTCH3
TC19001250.hg.1	4.25	PSR19017572.hg.1	NOTCH3

TC19001250.hg.1	3.84	JUC19010206.hg.1	NOTCH3
TC19001250.hg.1	3.59	PSR19017555.hg.1	NOTCH3
TC19001250.hg.1	3.57	PSR19017554.hg.1	NOTCH3
TC19001250.hg.1	3.2	PSR19017553.hg.1	NOTCH3
TC19001250.hg.1	3.07	JUC19010199.hg.1	NOTCH3
TC19001250.hg.1	3.03	JUC19010200.hg.1	NOTCH3
TC19001250.hg.1	2.76	JUC19010203.hg.1	NOTCH3
TC19001250.hg.1	2.59	PSR19017548.hg.1	NOTCH3
TC19001250.hg.1	2.54	PSR19017549.hg.1	NOTCH3
TC19001250.hg.1	2.46	JUC19010212.hg.1	NOTCH3
TC19001250.hg.1	2.42	PSR19017550.hg.1	NOTCH3
TC19001250.hg.1	2.41	PSR19017558.hg.1	NOTCH3
TC19001250.hg.1	2.36	JUC19010192.hg.1	NOTCH3
TC19001250.hg.1	2.34	PSR19017556.hg.1	NOTCH3
TC19001250.hg.1	2.23	PSR19017551.hg.1	NOTCH3
TC19001250.hg.1	2.01	PSR19017546.hg.1	NOTCH3
TC19001250.hg.1	-2	JUC19010215.hg.1	NOTCH3
TC15000270.hg.1	7.63	PSR15001691.hg.1	THBS1
TC15000270.hg.1	6.82	PSR15001722.hg.1	THBS1
TC15000270.hg.1	5.39	PSR15001698.hg.1	THBS1
TC15000270.hg.1	5.35	PSR15001712.hg.1	THBS1
TC15000270.hg.1	4.11	PSR15001697.hg.1	THBS1
TC15000270.hg.1	3.4	PSR15001696.hg.1	THBS1
TC15000270.hg.1	3.32	PSR15001721.hg.1	THBS1
TC15000270.hg.1	3.27	PSR15001685.hg.1	THBS1
TC15000270.hg.1	2.56	PSR15001677.hg.1	THBS1
TC15000270.hg.1	2.43	JUC15000740.hg.1	THBS1
TC15000270.hg.1	2.12	JUC15000735.hg.1	THBS1
TC12001984.hg.1	7.62	JUC12014552.hg.1	DDX54
TC12001984.hg.1	2.1	PSR12026080.hg.1	DDX54
TC12001984.hg.1	-2.12	JUC12014562.hg.1	DDX54
TC11000316.hg.1	7.61	JUC11002135.hg.1	HIPK3
TC11000316.hg.1	-2.01	PSR11004039.hg.1	HIPK3
TC12001277.hg.1	7.6	JUC12009119.hg.1	ERP27
TC12001277.hg.1	5.01	PSR12016635.hg.1	ERP27
TC12001277.hg.1	4.21	PSR12016640.hg.1	ERP27
TC12001277.hg.1	3.55	PSR12016637.hg.1	ERP27
TC12001277.hg.1	3.5	PSR12016638.hg.1	ERP27
TC12001277.hg.1	-2.12	JUC12009116.hg.1	ERP27
TC12001277.hg.1	-2.16	PSR12016628.hg.1	ERP27
TC12001277.hg.1	-2.49	PSR12016630.hg.1	ERP27
TC12001277.hg.1	-4.42	JUC12009115.hg.1	ERP27
TC15000622.hg.1	7.59	JUC15002502.hg.1	SMAD3
TC15000622.hg.1	4.72	JUC15002500.hg.1	SMAD3
TC15000622.hg.1	3.72	PSR15005089.hg.1	SMAD3
TC15000622.hg.1	3.17	JUC15002501.hg.1	SMAD3
TC15000622.hg.1	3.11	PSR15005085.hg.1	SMAD3
TC15000622.hg.1	2.77	PSR15005093.hg.1	SMAD3
TC15000622.hg.1	2.04	PSR15005097.hg.1	SMAD3
TC17000536.hg.1	7.57	JUC17003668.hg.1	WNK4
TC17000536.hg.1	5.7	JUC17003676.hg.1	WNK4
TC17000536.hg.1	4.95	PSR17006514.hg.1	WNK4
TC17000536.hg.1	3.75	JUC17003670.hg.1	WNK4
TC17000536.hg.1	3.44	PSR17006532.hg.1	WNK4
TC17000536.hg.1	3.38	JUC17003662.hg.1	WNK4
TC17000536.hg.1	3.1	PSR17006508.hg.1	WNK4
TC17000536.hg.1	3.06	PSR17006510.hg.1	WNK4
TC17000536.hg.1	2.95	PSR17006523.hg.1	WNK4

TC17000536.hg.1	2.87	JUC17003656.hg.1	WNK4
TC17000536.hg.1	2.66	PSR17006534.hg.1	WNK4
TC17000536.hg.1	2.49	PSR17006507.hg.1	WNK4
TC17000536.hg.1	2.36	PSR17006533.hg.1	WNK4
TC17000536.hg.1	2.34	JUC17003661.hg.1	WNK4
TC17000536.hg.1	2.33	PSR17006511.hg.1	WNK4
TC17000536.hg.1	2.21	JUC17003671.hg.1	WNK4
TC17000536.hg.1	-2.12	JUC17003664.hg.1	WNK4
TC17000536.hg.1	-2.23	PSR17006524.hg.1	WNK4
TC17000536.hg.1	-3.29	PSR17006525.hg.1	WNK4
TC17000536.hg.1	-3.57	PSR17006536.hg.1	WNK4
TC17000536.hg.1	-4.46	JUC17003677.hg.1	WNK4
TC17000536.hg.1	-4.47	JUC17003665.hg.1	WNK4
TC17000536.hg.1	-5.01	JUC17003674.hg.1	WNK4
TC17000536.hg.1	-5.58	PSR17006518.hg.1	WNK4
TC17000536.hg.1	-5.89	PSR17006520.hg.1	WNK4
TC17000536.hg.1	-6.22	PSR17006521.hg.1	WNK4
TC17000536.hg.1	-6.64	PSR17006535.hg.1	WNK4
TC17000536.hg.1	-6.73	JUC17003658.hg.1	WNK4
TC17000536.hg.1	-9.43	JUC17003659.hg.1	WNK4
TC17000536.hg.1	-23.96	JUC17003675.hg.1	WNK4
TC08000208.hg.1	7.53	JUC08001473.hg.1	EPHX2
TC08000208.hg.1	3.26	JUC08001479.hg.1	EPHX2
TC08000208.hg.1	3.06	JUC08001494.hg.1	EPHX2
TC08000208.hg.1	2.4	JUC08001483.hg.1	EPHX2
TC08000208.hg.1	2.01	JUC08001489.hg.1	EPHX2
TC08000208.hg.1	-2.17	PSR08003028.hg.1	EPHX2
TC08000208.hg.1	-2.62	PSR08003009.hg.1	EPHX2
TC08000208.hg.1	-2.81	JUC08001469.hg.1	EPHX2
TC01003745.hg.1	7.52	JUC01030000.hg.1	NUAK2
TC01003745.hg.1	4.75	JUC01030002.hg.1	NUAK2
TC01003745.hg.1	2.91	JUC01029999.hg.1	NUAK2
TC01003745.hg.1	2.77	PSR01057454.hg.1	NUAK2
TC01003745.hg.1	-2.86	JUC01030001.hg.1	NUAK2
TC01003745.hg.1	-2.91	PSR01057457.hg.1	NUAK2
TC01003745.hg.1	-5.35	JUC01029997.hg.1	NUAK2
TC6_cox_hap2000	7.52	JUC6_cox_hap2001{	HLA-C
TC6_cox_hap2000	2.59	PSR6_cox_hap2002(	HLA-C
TC6_cox_hap2000	2.56	PSR6_cox_hap2002	HLA-C
TC6_cox_hap2000	2.46	JUC6_cox_hap2001{	HLA-C
TC6_cox_hap2000	2.29	JUC6_cox_hap2001{	HLA-C
TC6_cox_hap2000	2.03	JUC6_cox_hap2001{	HLA-C
TC6_cox_hap2000	-2.02	JUC6_cox_hap2001{	HLA-C
TC6_cox_hap2000	-3.31	JUC6_cox_hap2001{	HLA-C
TC02002201.hg.1	7.5	PSR02035635.hg.1	LIMS3L, LIMS
TC02002201.hg.1	6.48	JUC02018392.hg.1	LIMS3L, LIMS
TC02002201.hg.1	5.52	PSR02035631.hg.1	LIMS3L, LIMS
TC02002201.hg.1	4.87	PSR02035634.hg.1	LIMS3L, LIMS
TC02002201.hg.1	4.6	PSR02035629.hg.1	LIMS3L, LIMS
TC02002201.hg.1	4.57	PSR02035630.hg.1	LIMS3L, LIMS
TC02002201.hg.1	4.2	JUC02018410.hg.1	LIMS3L, LIMS
TC02002201.hg.1	4.18	PSR02035633.hg.1	LIMS3L, LIMS
TC02002201.hg.1	3.46	PSR02035632.hg.1	LIMS3L, LIMS
TC02002201.hg.1	3.31	JUC02018409.hg.1	LIMS3L, LIMS
TC02002201.hg.1	2.95	JUC02018407.hg.1	LIMS3L, LIMS
TC02002201.hg.1	2.67	PSR02035636.hg.1	LIMS3L, LIMS
TC02002201.hg.1	-2.17	JUC02018400.hg.1	LIMS3L, LIMS
TC02002645.hg.1	7.47	JUC02022204.hg.1	ANKRD44

TC02002645.hg.1	5.02	JUC02022190.hg.1	ANKRD44
TC02002645.hg.1	2.24	JUC02022203.hg.1	ANKRD44
TC02002645.hg.1	2.12	JUC02022212.hg.1	ANKRD44
TC02002645.hg.1	2.09	PSR02042446.hg.1	ANKRD44
TC02002645.hg.1	2.09	JUC02022219.hg.1	ANKRD44
TC02002645.hg.1	2.07	PSR02042419.hg.1	ANKRD44
TC02002645.hg.1	-2.14	PSR02042417.hg.1	ANKRD44
TC02002645.hg.1	-2.18	JUC02022199.hg.1	ANKRD44
TC02002645.hg.1	-2.26	JUC02022225.hg.1	ANKRD44
TC02002645.hg.1	-2.68	PSR02042425.hg.1	ANKRD44
TC02002645.hg.1	-2.71	PSR02042443.hg.1	ANKRD44
TC02002645.hg.1	-2.79	JUC02022218.hg.1	ANKRD44
TC02002645.hg.1	-2.85	JUC02022221.hg.1	ANKRD44
TC02002645.hg.1	-2.88	PSR02042440.hg.1	ANKRD44
TC02002645.hg.1	-3.07	JUC02022201.hg.1	ANKRD44
TC02002645.hg.1	-3.46	JUC02022211.hg.1	ANKRD44
TC02002645.hg.1	-3.8	PSR02042453.hg.1	ANKRD44
TC12002066.hg.1	7.46	PSR12027178.hg.1	HPD
TC12002066.hg.1	6.86	JUC12015248.hg.1	HPD
TC12002066.hg.1	6.06	PSR12027180.hg.1	HPD
TC12002066.hg.1	4.4	PSR12027156.hg.1	HPD
TC12002066.hg.1	3.52	JUC12015240.hg.1	HPD
TC12002066.hg.1	3.42	JUC12015250.hg.1	HPD
TC12002066.hg.1	3.03	PSR12027177.hg.1	HPD
TC12002066.hg.1	2.69	PSR12027171.hg.1	HPD
TC12002066.hg.1	2.41	PSR12027176.hg.1	HPD
TC12002066.hg.1	-2	PSR12027158.hg.1	HPD
TC12002066.hg.1	-2.11	JUC12015243.hg.1	HPD
TC12002066.hg.1	-2.22	JUC12015242.hg.1	HPD
TC14001451.hg.1	7.45	PSR14016287.hg.1	LGMN
TC14001451.hg.1	-2.04	JUC14008413.hg.1	LGMN
TC01001985.hg.1	7.44	JUC01016734.hg.1	ZNF238
TC01001985.hg.1	3.06	PSR01030750.hg.1	ZNF238
TC01001985.hg.1	2.7	JUC01016730.hg.1	ZNF238
TC01001985.hg.1	2.52	JUC01016733.hg.1	ZNF238
TC01001985.hg.1	2.43	PSR01030745.hg.1	ZNF238
TC01001985.hg.1	2.38	PSR01030744.hg.1	ZNF238
TC01003334.hg.1	7.42	JUC01027218.hg.1	SMG5
TC01003334.hg.1	2.32	JUC01027206.hg.1	SMG5
TC01003334.hg.1	-2.28	JUC01027201.hg.1	SMG5
TC01003334.hg.1	-2.37	JUC01027198.hg.1	SMG5
TC01003334.hg.1	-2.67	PSR01051948.hg.1	SMG5
TC07003315.hg.1	7.42	JUC07020730.hg.1	SUN1
TC07003315.hg.1	4.34	PSR07000120.hg.1	SUN1
TC07003315.hg.1	3.65	PSR07000093.hg.1	SUN1
TC07003315.hg.1	3.2	PSR07000101.hg.1	SUN1
TC07003315.hg.1	3.19	PSR07000100.hg.1	SUN1
TC07003315.hg.1	3.12	PSR07000102.hg.1	SUN1
TC07003315.hg.1	3.05	JUC07020749.hg.1	SUN1
TC07003315.hg.1	3.02	PSR07000115.hg.1	SUN1
TC07003315.hg.1	2.79	PSR07000151.hg.1	SUN1
TC07003315.hg.1	2.75	JUC07020735.hg.1	SUN1
TC07003315.hg.1	2.65	PSR07000103.hg.1	SUN1
TC07003315.hg.1	2.61	PSR07000110.hg.1	SUN1
TC07003315.hg.1	2.56	PSR07000109.hg.1	SUN1
TC07003315.hg.1	2.51	JUC07020726.hg.1	SUN1
TC07003315.hg.1	2.42	PSR07000119.hg.1	SUN1
TC07003315.hg.1	2.33	PSR07000160.hg.1	SUN1

TC07003315.hg.1	2.31	JUC07020757.hg.1	SUN1
TC07003315.hg.1	2.24	PSR07000108.hg.1	SUN1
TC07003315.hg.1	2.21	JUC07020746.hg.1	SUN1
TC07003315.hg.1	2.1	PSR07000107.hg.1	SUN1
TC07003315.hg.1	2.08	PSR07000177.hg.1	SUN1
TC07003315.hg.1	2.07	PSR07000150.hg.1	SUN1
TC07003315.hg.1	2.05	PSR07000148.hg.1	SUN1
TC07003315.hg.1	2.01	PSR07000127.hg.1	SUN1
TC03001800.hg.1	7.4	JUC03016184.hg.1	SLCO2A1
TC03001800.hg.1	3.06	PSR03032636.hg.1	SLCO2A1
TC03001800.hg.1	2.26	JUC03016198.hg.1	SLCO2A1
TC03001800.hg.1	2	JUC03016186.hg.1	SLCO2A1
TC03001800.hg.1	-2.54	PSR03032642.hg.1	SLCO2A1
TC03001800.hg.1	-2.72	PSR03032653.hg.1	SLCO2A1
TC03001800.hg.1	-2.74	JUC03016189.hg.1	SLCO2A1
TC03001800.hg.1	-2.75	JUC03016185.hg.1	SLCO2A1
TC03001800.hg.1	-2.79	PSR03032652.hg.1	SLCO2A1
TC03001800.hg.1	-2.88	PSR03032654.hg.1	SLCO2A1
TC03001800.hg.1	-2.98	PSR03032627.hg.1	SLCO2A1
TC03001800.hg.1	-3.02	PSR03032631.hg.1	SLCO2A1
TC03001800.hg.1	-3.2	PSR03032638.hg.1	SLCO2A1
TC03001800.hg.1	-3.49	JUC03016195.hg.1	SLCO2A1
TC03001800.hg.1	-5.21	JUC03016182.hg.1	SLCO2A1
TC03001800.hg.1	-7.26	JUC03016191.hg.1	SLCO2A1
TC11000903.hg.1	7.39	JUC11005972.hg.1	FAT3
TC11000903.hg.1	6.18	JUC11005965.hg.1	FAT3
TC11000903.hg.1	4.69	JUC11005970.hg.1	FAT3
TC11000903.hg.1	3.67	PSR11011316.hg.1	FAT3
TC11000903.hg.1	2.28	PSR11011319.hg.1	FAT3
TC11000903.hg.1	2.09	JUC11005955.hg.1	FAT3
TC11000903.hg.1	-2.03	PSR11011321.hg.1	FAT3
TC02001769.hg.1	7.37	JUC02014965.hg.1	SLC8A1
TC02001769.hg.1	3.77	PSR02028578.hg.1	SLC8A1
TC02001769.hg.1	2.9	PSR02028579.hg.1	SLC8A1
TC02001769.hg.1	2.7	PSR02028577.hg.1	SLC8A1
TC02001769.hg.1	2.66	PSR02028574.hg.1	SLC8A1
TC02001769.hg.1	2.32	PSR02028560.hg.1	SLC8A1
TC02001769.hg.1	2.21	PSR02028576.hg.1	SLC8A1
TC02001769.hg.1	2.17	PSR02028583.hg.1	SLC8A1
TC02001769.hg.1	2.12	JUC02014963.hg.1	SLC8A1
TC03000816.hg.1	7.37	JUC03007408.hg.1	SELT
TC03000816.hg.1	-2.59	PSR03014668.hg.1	SELT
TC03000816.hg.1	-4.31	JUC03007410.hg.1	SELT
TC17000119.hg.1	7.35	JUC17000979.hg.1	CYB5D1
TC17000119.hg.1	2.12	PSR17001706.hg.1	CYB5D1
TC02000041.hg.1	7.33	JUC02000259.hg.1	ID2
TC02000041.hg.1	7.07	PSR02000477.hg.1	ID2
TC02000041.hg.1	6.37	PSR02000478.hg.1	ID2
TC02000041.hg.1	6.32	PSR02000476.hg.1	ID2
TC02000041.hg.1	-2.57	PSR02000480.hg.1	ID2
TC02004964.hg.1	7.31	JUC02031711.hg.1	LOC1001342
TC02004964.hg.1	5.88	PSR02004376.hg.1	LOC1001342
TC02004964.hg.1	4.3	JUC02031710.hg.1	LOC1001342
TC02004964.hg.1	4.26	PSR02004375.hg.1	LOC1001342
TC02004964.hg.1	2.97	PSR02004378.hg.1	LOC1001342
TC11001450.hg.1	7.3	JUC11009728.hg.1	PLEKHA7
TC11001450.hg.1	4.99	PSR11018211.hg.1	PLEKHA7
TC11001450.hg.1	4.28	JUC11009717.hg.1	PLEKHA7

TC11001450.hg.1	3.38	JUC11009723.hg.1	PLEKHA7
TC11001450.hg.1	2.9	JUC11009722.hg.1	PLEKHA7
TC11001450.hg.1	2.71	JUC11009740.hg.1	PLEKHA7
TC11001450.hg.1	2.71	JUC11009745.hg.1	PLEKHA7
TC11001450.hg.1	2.57	PSR11018200.hg.1	PLEKHA7
TC11001450.hg.1	2.37	JUC11009741.hg.1	PLEKHA7
TC11001450.hg.1	2.35	PSR11018212.hg.1	PLEKHA7
TC11001450.hg.1	2.04	JUC11009729.hg.1	PLEKHA7
TC11001450.hg.1	-2.33	PSR11018195.hg.1	PLEKHA7
TC11001450.hg.1	-2.35	PSR11018218.hg.1	PLEKHA7
TC11001450.hg.1	-2.43	PSR11018205.hg.1	PLEKHA7
TC11001450.hg.1	-2.5	PSR11018206.hg.1	PLEKHA7
TC11001450.hg.1	-2.5	PSR11018209.hg.1	PLEKHA7
TC11001450.hg.1	-2.54	PSR11018219.hg.1	PLEKHA7
TC11001450.hg.1	-2.93	PSR11018197.hg.1	PLEKHA7
TC11001450.hg.1	-3.04	JUC11009724.hg.1	PLEKHA7
TC11001450.hg.1	-3.13	JUC11009720.hg.1	PLEKHA7
TC11001450.hg.1	-3.27	PSR11018228.hg.1	PLEKHA7
TC11001450.hg.1	-3.45	JUC11009739.hg.1	PLEKHA7
TC11001450.hg.1	-3.65	JUC11009731.hg.1	PLEKHA7
TC11001450.hg.1	-4.23	JUC11009736.hg.1	PLEKHA7
TC01001423.hg.1	7.27	PSR01022235.hg.1	DDR2
TC01001423.hg.1	4.12	JUC01011721.hg.1	DDR2
TC01001423.hg.1	-2.01	PSR01022258.hg.1	DDR2
TC03000146.hg.1	7.26	PSR03002686.hg.1	RBMS3
TC03000146.hg.1	2	JUC03001422.hg.1	RBMS3
TC01002669.hg.1	7.23	PSR01041948.hg.1	LRP8
TC01002669.hg.1	7.12	JUC01022419.hg.1	LRP8
TC01002669.hg.1	7.05	JUC01022429.hg.1	LRP8
TC01002669.hg.1	6.53	JUC01022411.hg.1	LRP8
TC01002669.hg.1	5.66	JUC01022420.hg.1	LRP8
TC01002669.hg.1	5.4	PSR01041953.hg.1	LRP8
TC01002669.hg.1	5.24	PSR01041939.hg.1	LRP8
TC01002669.hg.1	5.02	PSR01041949.hg.1	LRP8
TC01002669.hg.1	4.94	PSR01041950.hg.1	LRP8
TC01002669.hg.1	4.75	PSR01041942.hg.1	LRP8
TC01002669.hg.1	4.74	PSR01041951.hg.1	LRP8
TC01002669.hg.1	4.44	JUC01022423.hg.1	LRP8
TC01002669.hg.1	4.43	JUC01022424.hg.1	LRP8
TC01002669.hg.1	4.38	JUC01022414.hg.1	LRP8
TC01002669.hg.1	4.29	PSR01041944.hg.1	LRP8
TC01002669.hg.1	4.28	JUC01022412.hg.1	LRP8
TC01002669.hg.1	3.72	JUC01022422.hg.1	LRP8
TC01002669.hg.1	3.71	PSR01041935.hg.1	LRP8
TC01002669.hg.1	3.61	PSR01041943.hg.1	LRP8
TC01002669.hg.1	3.39	JUC01022435.hg.1	LRP8
TC01002669.hg.1	3.31	PSR01041918.hg.1	LRP8
TC01002669.hg.1	3.03	JUC01022425.hg.1	LRP8
TC01002669.hg.1	2.91	JUC01022408.hg.1	LRP8
TC01002669.hg.1	2.75	JUC01022413.hg.1	LRP8
TC01002669.hg.1	2.7	PSR01041934.hg.1	LRP8
TC01002669.hg.1	2.69	JUC01022421.hg.1	LRP8
TC01002669.hg.1	2.37	JUC01022415.hg.1	LRP8
TC01002669.hg.1	2.23	PSR01041945.hg.1	LRP8
TC01002669.hg.1	2	PSR01041920.hg.1	LRP8
TC01002669.hg.1	2	PSR01041940.hg.1	LRP8
TC01002669.hg.1	-2.24	JUC01022434.hg.1	LRP8
TC04001286.hg.1	7.23	PSR04017583.hg.1	CXCL2

TC04001286.hg.1	6.67	PSR04017580.hg.1	CXCL2
TC04001286.hg.1	6.31	PSR04017579.hg.1	CXCL2
TC04001286.hg.1	5.69	PSR04017577.hg.1	CXCL2
TC04001286.hg.1	5.42	PSR04017571.hg.1	CXCL2
TC04001286.hg.1	4.68	PSR04017570.hg.1	CXCL2
TC04001286.hg.1	4.12	JUC04009393.hg.1	CXCL2
TC04001286.hg.1	3.87	PSR04017575.hg.1	CXCL2
TC04001286.hg.1	2.48	PSR04017574.hg.1	CXCL2
TC04001286.hg.1	-2.2	JUC04009392.hg.1	CXCL2
TC04000514.hg.1	7.14	JUC04003940.hg.1	LOC1005070
TC04000514.hg.1	2.6	JUC04003941.hg.1	LOC1005070
TC04000514.hg.1	2.51	PSR04007563.hg.1	LOC1005070
TC04000514.hg.1	2.36	JUC04003925.hg.1	LOC1005070
TC04000514.hg.1	2.25	PSR04007558.hg.1	LOC1005070
TC04000514.hg.1	2.23	PSR04007555.hg.1	LOC1005070
TC04000514.hg.1	-2.68	PSR04007556.hg.1	LOC1005070
TC10002941.hg.1	7.14	JUC10017226.hg.1	STAMBPL1
TC10002941.hg.1	3.7	JUC10017233.hg.1	STAMBPL1
TC10002941.hg.1	3.29	JUC10017232.hg.1	STAMBPL1
TC10002941.hg.1	2.96	PSR10006942.hg.1	STAMBPL1
TC10002941.hg.1	2.91	PSR10006933.hg.1	STAMBPL1
TC10002941.hg.1	2.74	PSR10006934.hg.1	STAMBPL1
TC10002941.hg.1	2.59	PSR10006930.hg.1	STAMBPL1
TC10002941.hg.1	2.44	PSR10006939.hg.1	STAMBPL1
TC10002941.hg.1	2.13	JUC10017229.hg.1	STAMBPL1
TC10002941.hg.1	2.06	PSR10006932.hg.1	STAMBPL1
TC10002941.hg.1	2.01	JUC10017227.hg.1	STAMBPL1
TC10002941.hg.1	-3.51	JUC10017223.hg.1	STAMBPL1
TC18000567.hg.1	7.1	JUC18003951.hg.1	RTTN
TC18000567.hg.1	4.33	JUC18004000.hg.1	RTTN
TC18000567.hg.1	3.01	JUC18003990.hg.1	RTTN
TC18000567.hg.1	2.49	JUC18003974.hg.1	RTTN
TC18000567.hg.1	2.43	JUC18003985.hg.1	RTTN
TC18000567.hg.1	2.03	JUC18003999.hg.1	RTTN
TC18000567.hg.1	2	PSR18005929.hg.1	RTTN
TC18000567.hg.1	-2.02	PSR18005928.hg.1	RTTN
TC18000567.hg.1	-2.31	JUC18003978.hg.1	RTTN
TC18000567.hg.1	-2.8	JUC18003983.hg.1	RTTN
TC05001752.hg.1	7.06	JUC05012198.hg.1	FBN2
TC05001752.hg.1	6.96	JUC05012163.hg.1	FBN2
TC05001752.hg.1	6.38	JUC05012157.hg.1	FBN2
TC05001752.hg.1	5.34	JUC05012214.hg.1	FBN2
TC05001752.hg.1	4.87	JUC05012181.hg.1	FBN2
TC05001752.hg.1	4.48	JUC05012170.hg.1	FBN2
TC05001752.hg.1	3.81	JUC05012171.hg.1	FBN2
TC05001752.hg.1	3.52	PSR05023556.hg.1	FBN2
TC05001752.hg.1	3.44	PSR05023548.hg.1	FBN2
TC05001752.hg.1	3.43	JUC05012164.hg.1	FBN2
TC05001752.hg.1	3.19	PSR05023526.hg.1	FBN2
TC05001752.hg.1	2.9	PSR05023539.hg.1	FBN2
TC05001752.hg.1	2.9	JUC05012165.hg.1	FBN2
TC05001752.hg.1	2.81	PSR05023549.hg.1	FBN2
TC05001752.hg.1	2.69	JUC05012158.hg.1	FBN2
TC05001752.hg.1	2.67	PSR05023551.hg.1	FBN2
TC05001752.hg.1	2.64	PSR05023518.hg.1	FBN2
TC05001752.hg.1	2.61	PSR05023547.hg.1	FBN2
TC05001752.hg.1	2.57	JUC05012189.hg.1	FBN2
TC05001752.hg.1	2.5	PSR05023501.hg.1	FBN2

TC05001752.hg.1	2.5	PSR05023550.hg.1	FBN2
TC05001752.hg.1	2.4	JUC05012229.hg.1	FBN2
TC05001752.hg.1	2.36	PSR05023529.hg.1	FBN2
TC05001752.hg.1	2.21	JUC05012207.hg.1	FBN2
TC05001752.hg.1	2.2	JUC05012166.hg.1	FBN2
TC05001752.hg.1	2.18	PSR05023546.hg.1	FBN2
TC05001752.hg.1	2.17	JUC05012196.hg.1	FBN2
TC05001752.hg.1	2.14	JUC05012179.hg.1	FBN2
TC05001752.hg.1	2.1	JUC05012227.hg.1	FBN2
TC05001752.hg.1	2.09	PSR05023522.hg.1	FBN2
TC05001752.hg.1	2.07	JUC05012183.hg.1	FBN2
TC05001752.hg.1	2.06	PSR05023542.hg.1	FBN2
TC05001752.hg.1	-2.02	PSR05023475.hg.1	FBN2
TC05001752.hg.1	-2.19	PSR05023493.hg.1	FBN2
TC05001752.hg.1	-2.27	JUC05012175.hg.1	FBN2
TC05001752.hg.1	-2.28	JUC05012212.hg.1	FBN2
TC05001752.hg.1	-2.29	JUC05012204.hg.1	FBN2
TC05001752.hg.1	-2.3	JUC05012225.hg.1	FBN2
TC05001752.hg.1	-2.32	PSR05023510.hg.1	FBN2
TC05001752.hg.1	-2.38	JUC05012186.hg.1	FBN2
TC05001752.hg.1	-2.8	JUC05012221.hg.1	FBN2
TC05001752.hg.1	-2.84	JUC05012184.hg.1	FBN2
TC05001752.hg.1	-2.91	PSR05023545.hg.1	FBN2
TC05001752.hg.1	-2.94	PSR05023525.hg.1	FBN2
TC05001752.hg.1	-3.3	JUC05012202.hg.1	FBN2
TC05001752.hg.1	-3.65	PSR05023534.hg.1	FBN2
TC05001752.hg.1	-3.71	JUC05012203.hg.1	FBN2
TC05001752.hg.1	-4.22	JUC05012156.hg.1	FBN2
TC09001282.hg.1	7.06	JUC09008605.hg.1	KIF27
TC09001282.hg.1	3.1	JUC09008602.hg.1	KIF27
TC09001282.hg.1	2.77	PSR09015980.hg.1	KIF27
TC09001282.hg.1	2.31	PSR09016001.hg.1	KIF27
TC09001282.hg.1	2.12	JUC09008598.hg.1	KIF27
TC09001282.hg.1	2	JUC09008594.hg.1	KIF27
TC09001282.hg.1	-2.79	JUC09008592.hg.1	KIF27
TC10000486.hg.1	7.04	PSR10005813.hg.1	ZNF503-AS1
TC10000486.hg.1	6.79	JUC10003414.hg.1	ZNF503-AS1
TC10000486.hg.1	6.54	JUC10003419.hg.1	ZNF503-AS1
TC10000486.hg.1	5.58	PSR10005811.hg.1	ZNF503-AS1
TC10000486.hg.1	4.48	PSR10005823.hg.1	ZNF503-AS1
TC10000486.hg.1	3.81	PSR10005810.hg.1	ZNF503-AS1
TC10000486.hg.1	3.72	PSR10005819.hg.1	ZNF503-AS1
TC10000486.hg.1	3.71	PSR10005826.hg.1	ZNF503-AS1
TC10000486.hg.1	3.04	PSR10005816.hg.1	ZNF503-AS1
TC10000486.hg.1	2.86	PSR10005822.hg.1	ZNF503-AS1
TC10000486.hg.1	2.83	PSR10005832.hg.1	ZNF503-AS1
TC10000486.hg.1	2.78	JUC10003409.hg.1	ZNF503-AS1
TC10000486.hg.1	2.68	PSR10005821.hg.1	ZNF503-AS1
TC10000486.hg.1	2.18	JUC10003417.hg.1	ZNF503-AS1
TC10000486.hg.1	-2.99	JUC10003423.hg.1	ZNF503-AS1
TC01002153.hg.1	7.03	JUC01017818.hg.1	PLEKHG5, TI
TC01002153.hg.1	5.57	JUC01017827.hg.1	PLEKHG5, TI
TC01002153.hg.1	4.97	JUC01017791.hg.1	PLEKHG5, TI
TC01002153.hg.1	4.79	JUC01017792.hg.1	PLEKHG5, TI
TC01002153.hg.1	4.68	PSR01033181.hg.1	PLEKHG5, TI
TC01002153.hg.1	4.5	JUC01017820.hg.1	PLEKHG5, TI
TC01002153.hg.1	4.23	JUC01017811.hg.1	PLEKHG5, TI
TC01002153.hg.1	4.05	PSR01033184.hg.1	PLEKHG5, TI

TC01002153.hg.1	4	PSR01033183.hg.1	PLEKHG5, TI
TC01002153.hg.1	3.89	PSR01033187.hg.1	PLEKHG5, TI
TC01002153.hg.1	3.79	PSR01033217.hg.1	PLEKHG5, TI
TC01002153.hg.1	3.53	JUC01017816.hg.1	PLEKHG5, TI
TC01002153.hg.1	3.42	JUC01017823.hg.1	PLEKHG5, TI
TC01002153.hg.1	3.32	JUC01017793.hg.1	PLEKHG5, TI
TC01002153.hg.1	3.13	PSR01033218.hg.1	PLEKHG5, TI
TC01002153.hg.1	3.02	PSR01033233.hg.1	PLEKHG5, TI
TC01002153.hg.1	2.99	PSR01033207.hg.1	PLEKHG5, TI
TC01002153.hg.1	2.85	JUC01017803.hg.1	PLEKHG5, TI
TC01002153.hg.1	2.81	PSR01033189.hg.1	PLEKHG5, TI
TC01002153.hg.1	2.81	PSR01033225.hg.1	PLEKHG5, TI
TC01002153.hg.1	2.68	PSR01033238.hg.1	PLEKHG5, TI
TC01002153.hg.1	2.66	JUC01017821.hg.1	PLEKHG5, TI
TC01002153.hg.1	2.62	PSR01033179.hg.1	PLEKHG5, TI
TC01002153.hg.1	2.62	PSR01033226.hg.1	PLEKHG5, TI
TC01002153.hg.1	2.56	PSR01033177.hg.1	PLEKHG5, TI
TC01002153.hg.1	2.55	PSR01033212.hg.1	PLEKHG5, TI
TC01002153.hg.1	2.54	PSR01033175.hg.1	PLEKHG5, TI
TC01002153.hg.1	2.53	PSR01033234.hg.1	PLEKHG5, TI
TC01002153.hg.1	2.51	JUC01017813.hg.1	PLEKHG5, TI
TC01002153.hg.1	2.5	PSR01033231.hg.1	PLEKHG5, TI
TC01002153.hg.1	2.44	PSR01033195.hg.1	PLEKHG5, TI
TC01002153.hg.1	2.42	PSR01033222.hg.1	PLEKHG5, TI
TC01002153.hg.1	2.41	JUC01017831.hg.1	PLEKHG5, TI
TC01002153.hg.1	2.35	PSR01033220.hg.1	PLEKHG5, TI
TC01002153.hg.1	2.27	PSR01033208.hg.1	PLEKHG5, TI
TC01002153.hg.1	2.27	PSR01033228.hg.1	PLEKHG5, TI
TC01002153.hg.1	2.26	PSR01033192.hg.1	PLEKHG5, TI
TC01002153.hg.1	2.23	PSR01033230.hg.1	PLEKHG5, TI
TC01002153.hg.1	2.18	PSR01033172.hg.1	PLEKHG5, TI
TC01002153.hg.1	2.16	JUC01017810.hg.1	PLEKHG5, TI
TC01002153.hg.1	2.12	PSR01033251.hg.1	PLEKHG5, TI
TC01002153.hg.1	2.05	PSR01033215.hg.1	PLEKHG5, TI
TC01002153.hg.1	-2.18	JUC01017790.hg.1	PLEKHG5, TI
TC01000904.hg.1	7.02	JUC01007530.hg.1	VCAM1
TC01000904.hg.1	5.39	PSR01014244.hg.1	VCAM1
TC01000904.hg.1	5.2	JUC01007529.hg.1	VCAM1
TC01000904.hg.1	5.07	JUC01007534.hg.1	VCAM1
TC01000904.hg.1	2.84	PSR01014256.hg.1	VCAM1
TC01000904.hg.1	2.84	JUC01007533.hg.1	VCAM1
TC01000904.hg.1	2.41	JUC01007531.hg.1	VCAM1
TC01001557.hg.1	7.02	JUC01012726.hg.1	TOR1AIP1
TC01001557.hg.1	2.3	PSR01024024.hg.1	TOR1AIP1
TC01001557.hg.1	-2.46	PSR01024030.hg.1	TOR1AIP1
TC01001557.hg.1	-2.5	PSR01024037.hg.1	TOR1AIP1
TC01001557.hg.1	-4.62	JUC01012727.hg.1	TOR1AIP1
TC0X001278.hg.1	7.02	PSR0X017319.hg.1	CHRDL1
TC0X001278.hg.1	5.99	PSR0X017318.hg.1	CHRDL1
TC0X001278.hg.1	5.15	JUC0X008797.hg.1	CHRDL1
TC0X001278.hg.1	3.45	PSR0X017314.hg.1	CHRDL1
TC0X001278.hg.1	2.06	JUC0X008802.hg.1	CHRDL1
TC0X001278.hg.1	-2.03	JUC0X008803.hg.1	CHRDL1
TC0X001278.hg.1	-2.06	JUC0X008799.hg.1	CHRDL1
TC0X001278.hg.1	-2.23	JUC0X008800.hg.1	CHRDL1
TC11000245.hg.1	7.01	JUC11001617.hg.1	SPTY2D1-AS
TC11000245.hg.1	3.18	PSR11003257.hg.1	SPTY2D1-AS
TC11000245.hg.1	2.35	PSR11003252.hg.1	SPTY2D1-AS

TC11000245.hg.1	2.28	PSR11003258.hg.1	SPTY2D1-AS
TC17000311.hg.1	7.01	JUC17002134.hg.1	KSR1
TC17000311.hg.1	6.77	JUC17002130.hg.1	KSR1
TC17000311.hg.1	3.82	JUC17002109.hg.1	KSR1
TC17000311.hg.1	3.36	JUC17002115.hg.1	KSR1
TC17000311.hg.1	2.42	PSR17003731.hg.1	KSR1
TC17000311.hg.1	2.36	JUC17002123.hg.1	KSR1
TC17000311.hg.1	2.14	PSR17003725.hg.1	KSR1
TC17000311.hg.1	-2.04	JUC17002128.hg.1	KSR1
TC17000311.hg.1	-2.79	JUC17002131.hg.1	KSR1
TC08000453.hg.1	7	JUC08003156.hg.1	C8orf34
TC08000453.hg.1	3.84	JUC08003151.hg.1	C8orf34
TC08000453.hg.1	3.58	PSR08006438.hg.1	C8orf34
TC08000453.hg.1	3.38	JUC08003158.hg.1	C8orf34
TC08000453.hg.1	3.37	JUC08003152.hg.1	C8orf34
TC08000453.hg.1	3.2	PSR08006449.hg.1	C8orf34
TC08000453.hg.1	2.85	PSR08006441.hg.1	C8orf34
TC08000453.hg.1	2.32	PSR08006445.hg.1	C8orf34
TC08000453.hg.1	2.3	JUC08003162.hg.1	C8orf34
TC08000453.hg.1	2.06	PSR08006444.hg.1	C8orf34
TC08000453.hg.1	-2.91	JUC08003153.hg.1	C8orf34
TC22000129.hg.1	7	JUC22000666.hg.1	BCR
TC22000129.hg.1	2.4	JUC22000686.hg.1	BCR
TC22000129.hg.1	2.09	JUC22000694.hg.1	BCR
TC22000129.hg.1	2.04	PSR22002285.hg.1	BCR
TC11001174.hg.1	6.98	JUC11008040.hg.1	ST3GAL4
TC11001174.hg.1	-2	PSR11014846.hg.1	ST3GAL4
TC11001174.hg.1	-2.24	PSR11014847.hg.1	ST3GAL4
TC20000481.hg.1	6.98	JUC20003543.hg.1	CDH4
TC20000481.hg.1	2.12	PSR20006696.hg.1	CDH4
TC20000481.hg.1	-2.85	JUC20003539.hg.1	CDH4
TC02000235.hg.1	6.97	JUC02001842.hg.1	C2orf56
TC02000235.hg.1	3.52	JUC02001830.hg.1	C2orf56
TC02000235.hg.1	2.18	JUC02001834.hg.1	C2orf56
TC02000235.hg.1	2.15	PSR02003524.hg.1	C2orf56
TC02000235.hg.1	2.07	PSR02003497.hg.1	C2orf56
TC02000235.hg.1	-2.02	PSR02003508.hg.1	C2orf56
TC02000235.hg.1	-2.64	JUC02001848.hg.1	C2orf56
TC02000235.hg.1	-3.13	JUC02001832.hg.1	C2orf56
TC13000790.hg.1	6.97	PSR13008880.hg.1	DCT
TC13000790.hg.1	2.76	JUC13005209.hg.1	DCT
TC13000790.hg.1	2.57	PSR13008881.hg.1	DCT
TC13000790.hg.1	2.33	PSR13008894.hg.1	DCT
TC13000790.hg.1	2.28	JUC13005210.hg.1	DCT
TC19001471.hg.1	6.97	JUC19011510.hg.1	ZNF529
TC19001471.hg.1	2.82	JUC19011506.hg.1	ZNF529
TC19001471.hg.1	2.55	JUC19011508.hg.1	ZNF529
TC19001471.hg.1	2.22	PSR19019996.hg.1	ZNF529
TC19001471.hg.1	2.15	PSR19020006.hg.1	ZNF529
TC12000076.hg.1	6.95	PSR12000741.hg.1	CD9, LOC10(
TC12000076.hg.1	5.67	JUC12000421.hg.1	CD9, LOC10(
TC12000076.hg.1	4.11	PSR12000750.hg.1	CD9, LOC10(
TC12000076.hg.1	3.9	JUC12000427.hg.1	CD9, LOC10(
TC12000076.hg.1	3.73	PSR12000751.hg.1	CD9, LOC10(
TC12000076.hg.1	3.34	PSR12000763.hg.1	CD9, LOC10(
TC12000076.hg.1	3.14	PSR12000749.hg.1	CD9, LOC10(
TC12000076.hg.1	2.97	PSR12000755.hg.1	CD9, LOC10(
TC12000076.hg.1	2.77	JUC12000419.hg.1	CD9, LOC10(

TC12000076.hg.1	2.39	JUC12000429.hg.1	CD9, LOC10(
TC12000076.hg.1	2.11	PSR12000748.hg.1	CD9, LOC10(
TC17000220.hg.1	6.94	PSR17003122.hg.1	TRIM16L
TC17000220.hg.1	6.58	JUC17001777.hg.1	TRIM16L
TC17000220.hg.1	6.55	JUC17001784.hg.1	TRIM16L
TC17000220.hg.1	5.89	PSR17003117.hg.1	TRIM16L
TC17000220.hg.1	5.56	JUC17001770.hg.1	TRIM16L
TC17000220.hg.1	5.31	JUC17001781.hg.1	TRIM16L
TC17000220.hg.1	5.22	JUC17001771.hg.1	TRIM16L
TC17000220.hg.1	4.96	JUC17001775.hg.1	TRIM16L
TC17000220.hg.1	4.78	PSR17003120.hg.1	TRIM16L
TC17000220.hg.1	4.54	PSR17003119.hg.1	TRIM16L
TC17000220.hg.1	4.51	PSR17003121.hg.1	TRIM16L
TC17000220.hg.1	4.45	PSR17003147.hg.1	TRIM16L
TC17000220.hg.1	4.42	PSR17003126.hg.1	TRIM16L
TC17000220.hg.1	4.39	PSR17003123.hg.1	TRIM16L
TC17000220.hg.1	4.33	JUC17001780.hg.1	TRIM16L
TC17000220.hg.1	4.25	JUC17001787.hg.1	TRIM16L
TC17000220.hg.1	2.81	PSR17003124.hg.1	TRIM16L
TC17000220.hg.1	2.54	PSR17003150.hg.1	TRIM16L
TC17000220.hg.1	2.54	PSR17003151.hg.1	TRIM16L
TC17000220.hg.1	2.36	PSR17003132.hg.1	TRIM16L
TC17000220.hg.1	2.26	JUC17001776.hg.1	TRIM16L
TC17000220.hg.1	-2.28	PSR17003133.hg.1	TRIM16L
TC17000220.hg.1	-2.28	PSR17003140.hg.1	TRIM16L
TC06002124.hg.1	6.93	JUC06013213.hg.1	SLC2A12
TC06002124.hg.1	3.32	PSR06026678.hg.1	SLC2A12
TC06002124.hg.1	2.15	PSR06026679.hg.1	SLC2A12
TC11000148.hg.1	6.91	JUC11000910.hg.1	SYT9
TC11000148.hg.1	2.66	PSR11002086.hg.1	SYT9
TC11000148.hg.1	2.54	JUC11000913.hg.1	SYT9
TC11000148.hg.1	2.21	PSR11002081.hg.1	SYT9
TC11000148.hg.1	-3.19	JUC11000912.hg.1	SYT9
TC05000004.hg.1	6.9	JUC05000027.hg.1	SDHA
TC05000004.hg.1	3.39	JUC05000036.hg.1	SDHA
TC05000004.hg.1	2.76	JUC05000035.hg.1	SDHA
TC05000004.hg.1	2.5	PSR05000087.hg.1	SDHA
TC05000004.hg.1	2.42	PSR05000086.hg.1	SDHA
TC05000004.hg.1	2.32	JUC05000051.hg.1	SDHA
TC05000004.hg.1	2.13	PSR05000053.hg.1	SDHA
TC05000004.hg.1	2.12	JUC05000034.hg.1	SDHA
TC05000004.hg.1	2.09	JUC05000023.hg.1	SDHA
TC08000778.hg.1	6.9	JUC08005388.hg.1	KHDRBS3
TC08000778.hg.1	6.84	JUC08005375.hg.1	KHDRBS3
TC08000778.hg.1	6.36	JUC08005377.hg.1	KHDRBS3
TC08000778.hg.1	4.14	PSR08010416.hg.1	KHDRBS3
TC08000778.hg.1	4.05	PSR08010420.hg.1	KHDRBS3
TC08000778.hg.1	3.33	PSR08010445.hg.1	KHDRBS3
TC08000778.hg.1	3.3	PSR08010415.hg.1	KHDRBS3
TC08000778.hg.1	3.21	PSR08010440.hg.1	KHDRBS3
TC08000778.hg.1	3.04	PSR08010450.hg.1	KHDRBS3
TC08000778.hg.1	2.78	PSR08010417.hg.1	KHDRBS3
TC08000778.hg.1	2.74	JUC08005383.hg.1	KHDRBS3
TC08000778.hg.1	2.73	PSR08010454.hg.1	KHDRBS3
TC08000778.hg.1	2.69	PSR08010439.hg.1	KHDRBS3
TC08000778.hg.1	2.68	PSR08010424.hg.1	KHDRBS3
TC08000778.hg.1	2.52	PSR08010429.hg.1	KHDRBS3
TC08000778.hg.1	2.48	PSR08010458.hg.1	KHDRBS3

TC08000778.hg.1	2.41	JUC08005390.hg.1	KHDRBS3
TC08000778.hg.1	2.39	PSR08010418.hg.1	KHDRBS3
TC08000778.hg.1	2.37	JUC08005378.hg.1	KHDRBS3
TC12000357.hg.1	6.88	JUC12002424.hg.1	CACNB3
TC12000357.hg.1	2.74	PSR12004431.hg.1	CACNB3
TC12000357.hg.1	-2.06	JUC12002422.hg.1	CACNB3
TC09002907.hg.1	6.86	PSR09006630.hg.1	ORM1
TC09002907.hg.1	3.21	PSR09006631.hg.1	ORM1
TC09002907.hg.1	2.76	PSR09006628.hg.1	ORM1
TC09002907.hg.1	2.32	JUC09015877.hg.1	ORM1
TC09002907.hg.1	-2	PSR09006624.hg.1	ORM1
TC09002907.hg.1	-2.52	PSR09006633.hg.1	ORM1
TC09002907.hg.1	-2.65	PSR09006637.hg.1	ORM1
TC01000755.hg.1	6.84	JUC01006326.hg.1	SRSF11
TC01000755.hg.1	3.28	JUC01006313.hg.1	SRSF11
TC01000755.hg.1	3.08	JUC01006315.hg.1	SRSF11
TC01000755.hg.1	2.46	PSR01012085.hg.1	SRSF11
TC01000755.hg.1	2.27	JUC01006335.hg.1	SRSF11
TC01000755.hg.1	-2.24	JUC01006314.hg.1	SRSF11
TC01000755.hg.1	-2.28	JUC01006328.hg.1	SRSF11
TC01003476.hg.1	6.84	JUC01028176.hg.1	ILDR2
TC01003476.hg.1	3.27	PSR01054065.hg.1	ILDR2
TC01003476.hg.1	-2.07	JUC01028167.hg.1	ILDR2
TC09001515.hg.1	6.84	PSR09018975.hg.1	TNFSF15
TC09001515.hg.1	4.63	PSR09018967.hg.1	TNFSF15
TC09001515.hg.1	3.59	PSR09018973.hg.1	TNFSF15
TC09001515.hg.1	2.34	PSR09018971.hg.1	TNFSF15
TC09001515.hg.1	2.28	PSR09018969.hg.1	TNFSF15
TC09001515.hg.1	2.17	PSR09018974.hg.1	TNFSF15
TC09001515.hg.1	-2.53	PSR09018970.hg.1	TNFSF15
TC09001515.hg.1	-2.64	JUC09010268.hg.1	TNFSF15
TC09001515.hg.1	-3.43	PSR09018972.hg.1	TNFSF15
TC09001515.hg.1	-5.01	JUC09010266.hg.1	TNFSF15
TC09001515.hg.1	-5.49	JUC09010267.hg.1	TNFSF15
TC20000245.hg.1	6.83	JUC20001792.hg.1	ACSS2
TC20000245.hg.1	3.3	JUC20001760.hg.1	ACSS2
TC20000245.hg.1	3.11	JUC20001775.hg.1	ACSS2
TC20000245.hg.1	2.94	JUC20001798.hg.1	ACSS2
TC20000245.hg.1	2.93	JUC20001797.hg.1	ACSS2
TC20000245.hg.1	2.89	JUC20001774.hg.1	ACSS2
TC20000245.hg.1	2.47	JUC20001767.hg.1	ACSS2
TC20000245.hg.1	2.44	JUC20001768.hg.1	ACSS2
TC20000245.hg.1	2.31	PSR20003183.hg.1	ACSS2
TC20000245.hg.1	2.2	PSR20003180.hg.1	ACSS2
TC20000245.hg.1	2.15	PSR20003148.hg.1	ACSS2
TC20000245.hg.1	2.11	PSR20003150.hg.1	ACSS2
TC20000245.hg.1	2.07	PSR20003199.hg.1	ACSS2
TC20000245.hg.1	2.06	PSR20003169.hg.1	ACSS2
TC20000245.hg.1	-2.17	JUC20001793.hg.1	ACSS2
TC20000245.hg.1	-2.91	JUC20001773.hg.1	ACSS2
TC01000217.hg.1	6.8	JUC01001941.hg.1	CROCC
TC01000217.hg.1	2.67	PSR01003527.hg.1	CROCC
TC01000217.hg.1	2.11	JUC01001933.hg.1	CROCC
TC01000217.hg.1	2.08	PSR01003582.hg.1	CROCC
TC01000217.hg.1	-2.07	JUC01001932.hg.1	CROCC
TC01000217.hg.1	-2.15	JUC01001920.hg.1	CROCC
TC01000217.hg.1	-2.43	PSR01003541.hg.1	CROCC
TC01000217.hg.1	-2.74	JUC01001921.hg.1	CROCC

TC04000291.hg.1	6.79	JUC04002095.hg.1	DCUN1D4
TC04000291.hg.1	3.73	PSR04004186.hg.1	DCUN1D4
TC04000291.hg.1	3.14	JUC04002089.hg.1	DCUN1D4
TC04000291.hg.1	-2.45	JUC04002075.hg.1	DCUN1D4
TC04000291.hg.1	-2.52	PSR04004194.hg.1	DCUN1D4
TC04000291.hg.1	-2.55	PSR04004211.hg.1	DCUN1D4
TC04000291.hg.1	-3.14	PSR04004212.hg.1	DCUN1D4
TC06000350.hg.1	6.79	PSR06003120.hg.1	DDR1, MIR46
TC06000350.hg.1	4.97	PSR06003238.hg.1	DDR1, MIR46
TC06000350.hg.1	3.79	JUC06001373.hg.1	DDR1, MIR46
TC06000350.hg.1	3.39	JUC06001411.hg.1	DDR1, MIR46
TC06000350.hg.1	3.32	PSR06003178.hg.1	DDR1, MIR46
TC06000350.hg.1	3.25	PSR06003151.hg.1	DDR1, MIR46
TC06000350.hg.1	3.22	PSR06003128.hg.1	DDR1, MIR46
TC06000350.hg.1	3.14	JUC06001398.hg.1	DDR1, MIR46
TC06000350.hg.1	3.11	JUC06001386.hg.1	DDR1, MIR46
TC06000350.hg.1	3.08	JUC06001363.hg.1	DDR1, MIR46
TC06000350.hg.1	3.05	PSR06003191.hg.1	DDR1, MIR46
TC06000350.hg.1	3.03	PSR06003207.hg.1	DDR1, MIR46
TC06000350.hg.1	3.02	PSR06003112.hg.1	DDR1, MIR46
TC06000350.hg.1	3	JUC06001382.hg.1	DDR1, MIR46
TC06000350.hg.1	2.94	JUC06001393.hg.1	DDR1, MIR46
TC06000350.hg.1	2.89	PSR06003099.hg.1	DDR1, MIR46
TC06000350.hg.1	2.84	JUC06001415.hg.1	DDR1, MIR46
TC06000350.hg.1	2.8	JUC06001405.hg.1	DDR1, MIR46
TC06000350.hg.1	2.77	JUC06001379.hg.1	DDR1, MIR46
TC06000350.hg.1	2.75	PSR06003226.hg.1	DDR1, MIR46
TC06000350.hg.1	2.75	JUC06001377.hg.1	DDR1, MIR46
TC06000350.hg.1	2.73	PSR06003125.hg.1	DDR1, MIR46
TC06000350.hg.1	2.71	PSR06003102.hg.1	DDR1, MIR46
TC06000350.hg.1	2.67	PSR06003097.hg.1	DDR1, MIR46
TC06000350.hg.1	2.67	PSR06003164.hg.1	DDR1, MIR46
TC06000350.hg.1	2.67	JUC06001412.hg.1	DDR1, MIR46
TC06000350.hg.1	2.66	JUC06001390.hg.1	DDR1, MIR46
TC06000350.hg.1	2.65	PSR06003158.hg.1	DDR1, MIR46
TC06000350.hg.1	2.64	PSR06003122.hg.1	DDR1, MIR46
TC06000350.hg.1	2.64	PSR06003126.hg.1	DDR1, MIR46
TC06000350.hg.1	2.57	JUC06001385.hg.1	DDR1, MIR46
TC06000350.hg.1	2.55	JUC06001366.hg.1	DDR1, MIR46
TC06000350.hg.1	2.53	JUC06001387.hg.1	DDR1, MIR46
TC06000350.hg.1	2.49	PSR06003193.hg.1	DDR1, MIR46
TC06000350.hg.1	2.48	PSR06003150.hg.1	DDR1, MIR46
TC06000350.hg.1	2.48	JUC06001374.hg.1	DDR1, MIR46
TC06000350.hg.1	2.44	PSR06003149.hg.1	DDR1, MIR46
TC06000350.hg.1	2.43	PSR06003105.hg.1	DDR1, MIR46
TC06000350.hg.1	2.43	PSR06003142.hg.1	DDR1, MIR46
TC06000350.hg.1	2.43	PSR06003144.hg.1	DDR1, MIR46
TC06000350.hg.1	2.42	JUC06001372.hg.1	DDR1, MIR46
TC06000350.hg.1	2.41	PSR06003248.hg.1	DDR1, MIR46
TC06000350.hg.1	2.39	PSR06003134.hg.1	DDR1, MIR46
TC06000350.hg.1	2.39	PSR06003140.hg.1	DDR1, MIR46
TC06000350.hg.1	2.39	JUC06001408.hg.1	DDR1, MIR46
TC06000350.hg.1	2.36	JUC06001370.hg.1	DDR1, MIR46
TC06000350.hg.1	2.34	PSR06003179.hg.1	DDR1, MIR46
TC06000350.hg.1	2.31	PSR06003172.hg.1	DDR1, MIR46
TC06000350.hg.1	2.3	JUC06001410.hg.1	DDR1, MIR46
TC06000350.hg.1	2.29	PSR06003113.hg.1	DDR1, MIR46
TC06000350.hg.1	2.13	JUC06001375.hg.1	DDR1, MIR46

TC06000350.hg.1	2.1	PSR06003098.hg.1	DDR1, MIR46
TC06000350.hg.1	2.07	PSR06003135.hg.1	DDR1, MIR46
TC06000350.hg.1	2.06	PSR06003115.hg.1	DDR1, MIR46
TC06000350.hg.1	2.06	PSR06003124.hg.1	DDR1, MIR46
TC06000350.hg.1	2.06	PSR06003194.hg.1	DDR1, MIR46
TC06000350.hg.1	2.04	JUC06001414.hg.1	DDR1, MIR46
TC06000350.hg.1	2.03	PSR06003127.hg.1	DDR1, MIR46
TC06000350.hg.1	2.03	PSR06003146.hg.1	DDR1, MIR46
TC06000350.hg.1	2	PSR06003243.hg.1	DDR1, MIR46
TC06000350.hg.1	-2.41	PSR06003212.hg.1	DDR1, MIR46
TC09000663.hg.1	6.74	JUC09004301.hg.1	GARNL3
TC09000663.hg.1	6.72	PSR09007701.hg.1	GARNL3
TC09000663.hg.1	6.5	PSR09007780.hg.1	GARNL3
TC09000663.hg.1	6.05	PSR09007710.hg.1	GARNL3
TC09000663.hg.1	6.05	JUC09004298.hg.1	GARNL3
TC09000663.hg.1	6.04	JUC09004321.hg.1	GARNL3
TC09000663.hg.1	6.03	JUC09004322.hg.1	GARNL3
TC09000663.hg.1	5.6	JUC09004297.hg.1	GARNL3
TC09000663.hg.1	5.56	PSR09007722.hg.1	GARNL3
TC09000663.hg.1	5.51	PSR09007720.hg.1	GARNL3
TC09000663.hg.1	5.28	JUC09004326.hg.1	GARNL3
TC09000663.hg.1	5.11	PSR09007774.hg.1	GARNL3
TC09000663.hg.1	4.9	JUC09004315.hg.1	GARNL3
TC09000663.hg.1	4.56	PSR09007778.hg.1	GARNL3
TC09000663.hg.1	4.5	PSR09007703.hg.1	GARNL3
TC09000663.hg.1	4.5	PSR09007782.hg.1	GARNL3
TC09000663.hg.1	4.23	PSR09007755.hg.1	GARNL3
TC09000663.hg.1	4.2	PSR09007714.hg.1	GARNL3
TC09000663.hg.1	4.16	PSR09007779.hg.1	GARNL3
TC09000663.hg.1	4.08	PSR09007730.hg.1	GARNL3
TC09000663.hg.1	4.06	PSR09007773.hg.1	GARNL3
TC09000663.hg.1	3.91	PSR09007702.hg.1	GARNL3
TC09000663.hg.1	3.91	PSR09007776.hg.1	GARNL3
TC09000663.hg.1	3.85	PSR09007747.hg.1	GARNL3
TC09000663.hg.1	3.82	PSR09007769.hg.1	GARNL3
TC09000663.hg.1	3.82	JUC09004306.hg.1	GARNL3
TC09000663.hg.1	3.72	PSR09007751.hg.1	GARNL3
TC09000663.hg.1	3.66	PSR09007767.hg.1	GARNL3
TC09000663.hg.1	3.58	PSR09007752.hg.1	GARNL3
TC09000663.hg.1	3.55	PSR09007761.hg.1	GARNL3
TC09000663.hg.1	3.48	JUC09004314.hg.1	GARNL3
TC09000663.hg.1	3.44	PSR09007765.hg.1	GARNL3
TC09000663.hg.1	3.41	JUC09004329.hg.1	GARNL3
TC09000663.hg.1	3.37	PSR09007781.hg.1	GARNL3
TC09000663.hg.1	3.35	JUC09004325.hg.1	GARNL3
TC09000663.hg.1	3.28	PSR09007715.hg.1	GARNL3
TC09000663.hg.1	3.23	PSR09007708.hg.1	GARNL3
TC09000663.hg.1	3.17	PSR09007743.hg.1	GARNL3
TC09000663.hg.1	3.16	PSR09007740.hg.1	GARNL3
TC09000663.hg.1	3.1	PSR09007742.hg.1	GARNL3
TC09000663.hg.1	3.01	PSR09007766.hg.1	GARNL3
TC09000663.hg.1	3	PSR09007754.hg.1	GARNL3
TC09000663.hg.1	2.95	PSR09007711.hg.1	GARNL3
TC09000663.hg.1	2.95	PSR09007768.hg.1	GARNL3
TC09000663.hg.1	2.81	PSR09007771.hg.1	GARNL3
TC09000663.hg.1	2.81	JUC09004290.hg.1	GARNL3
TC09000663.hg.1	2.68	PSR09007729.hg.1	GARNL3
TC09000663.hg.1	2.65	PSR09007739.hg.1	GARNL3

TC09000663.hg.1	2.65	PSR09007775.hg.1	GARNL3
TC09000663.hg.1	2.62	JUC09004313.hg.1	GARNL3
TC09000663.hg.1	2.52	PSR09007744.hg.1	GARNL3
TC09000663.hg.1	2.49	PSR09007759.hg.1	GARNL3
TC09000663.hg.1	2.49	JUC09004323.hg.1	GARNL3
TC09000663.hg.1	2.48	JUC09004319.hg.1	GARNL3
TC09000663.hg.1	2.39	PSR09007712.hg.1	GARNL3
TC09000663.hg.1	2.36	JUC09004337.hg.1	GARNL3
TC09000663.hg.1	2.35	JUC09004305.hg.1	GARNL3
TC09000663.hg.1	2.3	JUC09004304.hg.1	GARNL3
TC09000663.hg.1	2.28	PSR09007777.hg.1	GARNL3
TC09000663.hg.1	2.26	PSR09007764.hg.1	GARNL3
TC09000663.hg.1	2.07	PSR09007749.hg.1	GARNL3
TC09000663.hg.1	2.02	PSR09007724.hg.1	GARNL3
TC09000663.hg.1	2	JUC09004317.hg.1	GARNL3
TC01001851.hg.1	6.73	JUC01015595.hg.1	DNAH14
TC01001851.hg.1	3.96	JUC01015507.hg.1	DNAH14
TC01001851.hg.1	3.94	JUC01015557.hg.1	DNAH14
TC01001851.hg.1	3.11	JUC01015499.hg.1	DNAH14
TC01001851.hg.1	2.9	JUC01015496.hg.1	DNAH14
TC01001851.hg.1	2.64	PSR01028846.hg.1	DNAH14
TC01001851.hg.1	2.5	PSR01028803.hg.1	DNAH14
TC01001851.hg.1	2.44	PSR01028850.hg.1	DNAH14
TC01001851.hg.1	2.39	JUC01015484.hg.1	DNAH14
TC01001851.hg.1	2.33	PSR01028776.hg.1	DNAH14
TC01001851.hg.1	2.3	JUC01015488.hg.1	DNAH14
TC01001851.hg.1	2.28	JUC01015585.hg.1	DNAH14
TC01001851.hg.1	2.25	JUC01015494.hg.1	DNAH14
TC01001851.hg.1	2.17	JUC01015570.hg.1	DNAH14
TC01001851.hg.1	2.05	PSR01028820.hg.1	DNAH14
TC01001851.hg.1	2.02	JUC01015538.hg.1	DNAH14
TC08001175.hg.1	6.73	JUC08007764.hg.1	PLAT
TC08001175.hg.1	5.03	JUC08007770.hg.1	PLAT
TC08001175.hg.1	4.37	PSR08015132.hg.1	PLAT
TC08001175.hg.1	2.39	JUC08007758.hg.1	PLAT
TC08001175.hg.1	2.02	PSR08015125.hg.1	PLAT
TC08001175.hg.1	2.02	PSR08015126.hg.1	PLAT
TC08001175.hg.1	-2.48	PSR08015136.hg.1	PLAT
TC08001175.hg.1	-2.6	PSR08015134.hg.1	PLAT
TC08001175.hg.1	-2.65	PSR08015147.hg.1	PLAT
TC08001175.hg.1	-2.88	JUC08007765.hg.1	PLAT
TC08001175.hg.1	-2.91	JUC08007771.hg.1	PLAT
TC08001175.hg.1	-3.04	JUC08007768.hg.1	PLAT
TC08001175.hg.1	-3.21	PSR08015151.hg.1	PLAT
TC08001175.hg.1	-3.63	PSR08015144.hg.1	PLAT
TC08001175.hg.1	-3.82	PSR08015146.hg.1	PLAT
TC08001175.hg.1	-4.05	JUC08007749.hg.1	PLAT
TC08001175.hg.1	-4.36	PSR08015161.hg.1	PLAT
TC08001175.hg.1	-4.49	PSR08015160.hg.1	PLAT
TC08001175.hg.1	-4.99	PSR08015141.hg.1	PLAT
TC08001175.hg.1	-5.03	PSR08015140.hg.1	PLAT
TC08001175.hg.1	-5.13	PSR08015135.hg.1	PLAT
TC08001175.hg.1	-5.48	JUC08007763.hg.1	PLAT
TC08001175.hg.1	-5.67	JUC08007754.hg.1	PLAT
TC08001175.hg.1	-6.78	JUC08007761.hg.1	PLAT
TC08001175.hg.1	-6.89	PSR08015150.hg.1	PLAT
TC08001175.hg.1	-6.92	PSR08015131.hg.1	PLAT
TC08001175.hg.1	-6.93	PSR08015155.hg.1	PLAT

TC08001175.hg.1	-7.84	PSR08015156.hg.1	PLAT
TC08001175.hg.1	-9.95	JUC08007759.hg.1	PLAT
TC17000246.hg.1	6.72	PSR17003422.hg.1	ALDH3A2
TC17000246.hg.1	6.33	PSR17003424.hg.1	ALDH3A2
TC17000246.hg.1	6.33	PSR17003426.hg.1	ALDH3A2
TC17000246.hg.1	5.95	PSR17003421.hg.1	ALDH3A2
TC17000246.hg.1	2.87	PSR17003434.hg.1	ALDH3A2
TC17000246.hg.1	2.75	PSR17003428.hg.1	ALDH3A2
TC17000246.hg.1	-2.57	PSR17003441.hg.1	ALDH3A2
TC17000246.hg.1	-2.88	JUC17001972.hg.1	ALDH3A2
TC17000246.hg.1	-3.18	JUC17001965.hg.1	ALDH3A2
TC6_qbl_hap6000	6.71	JUC6_qbl_hap60009	HLA-C
TC6_qbl_hap6000	3.45	PSR6_qbl_hap60026	HLA-C
TC6_qbl_hap6000	2.91	JUC6_qbl_hap60010	HLA-C
TC6_qbl_hap6000	2.89	JUC6_qbl_hap60009	HLA-C
TC6_qbl_hap6000	2.17	PSR6_qbl_hap60026	HLA-C
TC6_qbl_hap6000	2.16	PSR6_qbl_hap60026	HLA-C
TC6_qbl_hap6000	-2.39	JUC6_qbl_hap60009	HLA-C
TC6_ssto_hap700	6.71	JUC6_ssto_hap7001	HLA-C
TC6_ssto_hap700	3.45	PSR6_ssto_hap7002	HLA-C
TC6_ssto_hap700	2.89	JUC6_ssto_hap7001	HLA-C
TC6_ssto_hap700	2.17	PSR6_ssto_hap7002	HLA-C
TC6_ssto_hap700	2.16	PSR6_ssto_hap7002	HLA-C
TC6_ssto_hap700	-2.39	JUC6_ssto_hap7001	HLA-C
TC10002948.hg.1	6.68	JUC10017333.hg.1	AS3MT
TC10002948.hg.1	2.15	PSR10009125.hg.1	AS3MT
TC10002948.hg.1	2.04	PSR10009138.hg.1	AS3MT
TC16001244.hg.1	6.68	JUC16009717.hg.1	HYDIN
TC16001244.hg.1	6.06	JUC16009796.hg.1	HYDIN
TC16001244.hg.1	3.6	JUC16009748.hg.1	HYDIN
TC16001244.hg.1	3.21	JUC16009736.hg.1	HYDIN
TC16001244.hg.1	2.93	JUC16009747.hg.1	HYDIN
TC16001244.hg.1	2.55	JUC16009740.hg.1	HYDIN
TC16001244.hg.1	2.42	JUC16009789.hg.1	HYDIN
TC16001244.hg.1	2.22	PSR16017189.hg.1	HYDIN
TC16001244.hg.1	2.22	JUC16009725.hg.1	HYDIN
TC16001244.hg.1	2.21	JUC16009750.hg.1	HYDIN
TC16001244.hg.1	2.09	JUC16009726.hg.1	HYDIN
TC16001244.hg.1	-2.26	JUC16009795.hg.1	HYDIN
TC16001244.hg.1	-2.93	JUC16009710.hg.1	HYDIN
TC16001244.hg.1	-3.07	JUC16009768.hg.1	HYDIN
TC17001479.hg.1	6.68	JUC17011095.hg.1	KRTAP1-5
TC17001479.hg.1	4.81	PSR17019665.hg.1	KRTAP1-5
TC17001479.hg.1	3.82	PSR17019666.hg.1	KRTAP1-5
TC17001479.hg.1	3.62	PSR17019661.hg.1	KRTAP1-5
TC17001479.hg.1	2.31	PSR17019668.hg.1	KRTAP1-5
TC17001479.hg.1	-2.31	PSR17019664.hg.1	KRTAP1-5
TC17001479.hg.1	-3.57	PSR17019662.hg.1	KRTAP1-5
TC13000049.hg.1	6.67	JUC13000185.hg.1	TNFRSF19
TC13000049.hg.1	-2.07	PSR13000342.hg.1	TNFRSF19
TC13000049.hg.1	-2.17	PSR13000349.hg.1	TNFRSF19
TC13000049.hg.1	-2.2	JUC13000189.hg.1	TNFRSF19
TC13000049.hg.1	-2.44	PSR13000350.hg.1	TNFRSF19
TC03000966.hg.1	6.65	PSR03016756.hg.1	SOX2-OT
TC03000966.hg.1	2.16	JUC03008507.hg.1	SOX2-OT
TC03000966.hg.1	2.09	JUC03008519.hg.1	SOX2-OT
TC03000966.hg.1	2.05	PSR03016757.hg.1	SOX2-OT
TC02005003.hg.1	6.64	JUC02032002.hg.1	PHOSPHO2

TC02005003.hg.1	3.19	JUC02031999.hg.1	PHOSPHO2
TC02005003.hg.1	2.83	PSR02014741.hg.1	PHOSPHO2
TC02005003.hg.1	2.06	PSR02014753.hg.1	PHOSPHO2
TC22001468.hg.1	6.64	PSR22005835.hg.1	APOBEC3G
TC22001468.hg.1	6.53	PSR22005843.hg.1	APOBEC3G
TC22001468.hg.1	6.29	PSR22005842.hg.1	APOBEC3G
TC22001468.hg.1	6.26	PSR22005827.hg.1	APOBEC3G
TC22001468.hg.1	5.81	PSR22005838.hg.1	APOBEC3G
TC22001468.hg.1	5.04	PSR22005844.hg.1	APOBEC3G
TC22001468.hg.1	4.76	PSR22005833.hg.1	APOBEC3G
TC22001468.hg.1	4.46	PSR22005830.hg.1	APOBEC3G
TC22001468.hg.1	3.95	PSR22005848.hg.1	APOBEC3G
TC22001468.hg.1	3.57	JUC22009310.hg.1	APOBEC3G
TC22001468.hg.1	3.25	PSR22005837.hg.1	APOBEC3G
TC22001468.hg.1	2.96	PSR22005847.hg.1	APOBEC3G
TC22001468.hg.1	2.56	PSR22005839.hg.1	APOBEC3G
TC22001468.hg.1	2.22	JUC22009309.hg.1	APOBEC3G
TC22001468.hg.1	2.04	PSR22005831.hg.1	APOBEC3G
TC22001468.hg.1	-2.05	PSR22005840.hg.1	APOBEC3G
TC22001468.hg.1	-2.42	JUC22009313.hg.1	APOBEC3G
TC15001730.hg.1	6.63	PSR15016911.hg.1	STARD5
TC15001730.hg.1	4.22	PSR15016909.hg.1	STARD5
TC15001730.hg.1	4.06	PSR15016908.hg.1	STARD5
TC15001730.hg.1	3.48	PSR15016910.hg.1	STARD5
TC15001730.hg.1	2.65	PSR15016925.hg.1	STARD5
TC15001730.hg.1	2.59	PSR15016923.hg.1	STARD5
TC15001730.hg.1	2.54	PSR15016912.hg.1	STARD5
TC15001730.hg.1	2.21	PSR15016922.hg.1	STARD5
TC15001730.hg.1	-2.23	JUC15009223.hg.1	STARD5
TC21000423.hg.1	6.63	PSR21004982.hg.1	RUNX1, LOC
TC21000423.hg.1	6.02	PSR21004981.hg.1	RUNX1, LOC
TC21000423.hg.1	3.89	JUC21002535.hg.1	RUNX1, LOC
TC21000423.hg.1	3.68	JUC21002548.hg.1	RUNX1, LOC
TC21000423.hg.1	2.67	JUC21002533.hg.1	RUNX1, LOC
TC21000423.hg.1	2.53	PSR21004983.hg.1	RUNX1, LOC
TC21000423.hg.1	2.23	PSR21004990.hg.1	RUNX1, LOC
TC21000423.hg.1	2.2	JUC21002547.hg.1	RUNX1, LOC
TC21000423.hg.1	2.18	JUC21002554.hg.1	RUNX1, LOC
TC21000423.hg.1	-2.69	JUC21002552.hg.1	RUNX1, LOC
TC02000082.hg.1	6.62	PSR02001045.hg.1	TRIB2
TC02000082.hg.1	4.27	JUC02000586.hg.1	TRIB2
TC02000082.hg.1	3.86	JUC02000590.hg.1	TRIB2
TC02000082.hg.1	2.35	PSR02001048.hg.1	TRIB2
TC02000082.hg.1	2.25	PSR02001046.hg.1	TRIB2
TC02004931.hg.1	6.62	JUC02031433.hg.1	ZNF514
TC02004931.hg.1	2.3	PSR02033774.hg.1	ZNF514
TC02004931.hg.1	2.16	PSR02033778.hg.1	ZNF514
TC18000103.hg.1	6.61	JUC18000847.hg.1	LAMA3
TC18000103.hg.1	3.59	JUC18000786.hg.1	LAMA3
TC18000103.hg.1	3.29	JUC18000818.hg.1	LAMA3
TC18000103.hg.1	2.98	JUC18000803.hg.1	LAMA3
TC18000103.hg.1	2.57	JUC18000864.hg.1	LAMA3
TC18000103.hg.1	2.03	JUC18000789.hg.1	LAMA3
TC18000103.hg.1	2.03	JUC18000815.hg.1	LAMA3
TC18000103.hg.1	2.03	JUC18000835.hg.1	LAMA3
TC18000103.hg.1	2.02	JUC18000834.hg.1	LAMA3
TC18000103.hg.1	-2.02	JUC18000799.hg.1	LAMA3
TC18000103.hg.1	-2.05	JUC18000819.hg.1	LAMA3

TC18000103.hg.1	-2.19	PSR18001178.hg.1	LAMA3
TC18000103.hg.1	-2.28	PSR18001162.hg.1	LAMA3
TC18000103.hg.1	-3.08	JUC18000839.hg.1	LAMA3
TC05000408.hg.1	6.58	PSR05005897.hg.1	ATP6AP1L, F
TC05000408.hg.1	4.06	PSR05005896.hg.1	ATP6AP1L, F
TC05000408.hg.1	3.5	JUC05003201.hg.1	ATP6AP1L, F
TC05000408.hg.1	3.19	PSR05005893.hg.1	ATP6AP1L, F
TC05000408.hg.1	2.74	JUC05003202.hg.1	ATP6AP1L, F
TC05000408.hg.1	2.62	JUC05003198.hg.1	ATP6AP1L, F
TC05000408.hg.1	2.42	PSR05005895.hg.1	ATP6AP1L, F
TC05000408.hg.1	2.37	JUC05003213.hg.1	ATP6AP1L, F
TC05000408.hg.1	2.12	PSR05005891.hg.1	ATP6AP1L, F
TC05000408.hg.1	2.1	PSR05005892.hg.1	ATP6AP1L, F
TC05000408.hg.1	2.05	PSR05005879.hg.1	ATP6AP1L, F
TC05000408.hg.1	-3.16	JUC05003212.hg.1	ATP6AP1L, F
TC02001119.hg.1	6.57	JUC02009081.hg.1	MFSD6
TC02001119.hg.1	2.63	PSR02016942.hg.1	MFSD6
TC02001119.hg.1	2.55	PSR02016917.hg.1	MFSD6
TC02001119.hg.1	2.48	PSR02016915.hg.1	MFSD6
TC02001119.hg.1	2.04	PSR02016946.hg.1	MFSD6
TC02001119.hg.1	2.02	PSR02016916.hg.1	MFSD6
TC02001119.hg.1	2.02	PSR02016944.hg.1	MFSD6
TC02001119.hg.1	-2.05	JUC02009078.hg.1	MFSD6
TC02001119.hg.1	-2.96	JUC02009074.hg.1	MFSD6
TC06002044.hg.1	6.56	JUC06012603.hg.1	COL10A1
TC06002044.hg.1	-6.36	JUC06012602.hg.1	COL10A1
TC06002044.hg.1	-7.12	PSR06025544.hg.1	COL10A1
TC06002044.hg.1	-10.31	PSR06025553.hg.1	COL10A1
TC06002044.hg.1	-11.35	JUC06012600.hg.1	COL10A1
TC06002044.hg.1	-16.54	PSR06025549.hg.1	COL10A1
TC06002044.hg.1	-28.24	PSR06025551.hg.1	COL10A1
TC03001750.hg.1	6.55	JUC03015833.hg.1	MGLL
TC03001750.hg.1	3.75	JUC03015832.hg.1	MGLL
TC03001750.hg.1	3.62	PSR03031775.hg.1	MGLL
TC03001750.hg.1	3.21	PSR03031784.hg.1	MGLL
TC03001750.hg.1	3.07	JUC03015831.hg.1	MGLL
TC03001750.hg.1	2.53	PSR03031765.hg.1	MGLL
TC03001750.hg.1	2.5	PSR03031785.hg.1	MGLL
TC03001750.hg.1	2.44	PSR03031779.hg.1	MGLL
TC03001750.hg.1	2.4	PSR03031782.hg.1	MGLL
TC03001750.hg.1	-2.46	JUC03015825.hg.1	MGLL
TC0X001303.hg.1	6.53	PSR0X017526.hg.1	KLHL13
TC0X001303.hg.1	4.21	PSR0X017536.hg.1	KLHL13
TC0X001303.hg.1	3.92	PSR0X017528.hg.1	KLHL13
TC0X001303.hg.1	3.61	PSR0X017527.hg.1	KLHL13
TC0X001303.hg.1	3.5	JUC0X008931.hg.1	KLHL13
TC0X001303.hg.1	3.16	JUC0X008925.hg.1	KLHL13
TC0X001303.hg.1	2.83	PSR0X017523.hg.1	KLHL13
TC0X001303.hg.1	2.74	PSR0X017512.hg.1	KLHL13
TC0X001303.hg.1	2.61	PSR0X017530.hg.1	KLHL13
TC0X001303.hg.1	2.45	JUC0X008921.hg.1	KLHL13
TC0X001303.hg.1	-2.07	PSR0X017517.hg.1	KLHL13
TC0X001303.hg.1	-3.02	JUC0X008924.hg.1	KLHL13
TC02002217.hg.1	6.52	JUC02018636.hg.1	CKAP2L
TC02002217.hg.1	3.43	JUC02018633.hg.1	CKAP2L
TC02002217.hg.1	2.55	PSR02036026.hg.1	CKAP2L
TC02002217.hg.1	-2.23	JUC02018640.hg.1	CKAP2L
TC11000721.hg.1	6.52	JUC11004882.hg.1	ANO1

TC11000721.hg.1	5.16	JUC11004885.hg.1	ANO1
TC11000721.hg.1	3.85	JUC11004880.hg.1	ANO1
TC11000721.hg.1	3.64	JUC11004860.hg.1	ANO1
TC11000721.hg.1	2.71	JUC11004873.hg.1	ANO1
TC11000721.hg.1	2.24	PSR11009420.hg.1	ANO1
TC11000721.hg.1	2.21	PSR11009443.hg.1	ANO1
TC11000721.hg.1	2.02	JUC11004878.hg.1	ANO1
TC11000721.hg.1	-2.23	PSR11009419.hg.1	ANO1
TC11000721.hg.1	-2.83	PSR11009413.hg.1	ANO1
TC11000721.hg.1	-4.55	JUC11004871.hg.1	ANO1
TC11000721.hg.1	-5.58	JUC11004874.hg.1	ANO1
TC11000211.hg.1	6.51	JUC11001366.hg.1	SPON1
TC11000211.hg.1	5.31	PSR11002835.hg.1	SPON1
TC11000211.hg.1	5.12	PSR11002843.hg.1	SPON1
TC11000211.hg.1	4.79	JUC11001354.hg.1	SPON1
TC11000211.hg.1	4.06	PSR11002840.hg.1	SPON1
TC11000211.hg.1	3.58	JUC11001363.hg.1	SPON1
TC11000211.hg.1	3.54	JUC11001364.hg.1	SPON1
TC11000211.hg.1	2.75	PSR11002841.hg.1	SPON1
TC11000211.hg.1	2.57	PSR11002845.hg.1	SPON1
TC11000211.hg.1	2.44	PSR11002836.hg.1	SPON1
TC11000211.hg.1	-2.22	JUC11001355.hg.1	SPON1
TC11000211.hg.1	-2.35	JUC11001360.hg.1	SPON1
TC11000211.hg.1	-2.57	JUC11001353.hg.1	SPON1
TC11000211.hg.1	-3.57	PSR11002851.hg.1	SPON1
TC11000211.hg.1	-3.67	PSR11002847.hg.1	SPON1
TC11000211.hg.1	-3.7	JUC11001352.hg.1	SPON1
TC11000211.hg.1	-3.91	PSR11002849.hg.1	SPON1
TC11000211.hg.1	-3.92	PSR11002850.hg.1	SPON1
TC11000211.hg.1	-5.05	PSR11002832.hg.1	SPON1
TC11000211.hg.1	-5.76	PSR11002846.hg.1	SPON1
TC11000211.hg.1	-5.92	JUC11001359.hg.1	SPON1
TC11000211.hg.1	-6.43	PSR11002848.hg.1	SPON1
TC11000211.hg.1	-6.84	PSR11002853.hg.1	SPON1
TC11000211.hg.1	-6.84	JUC11001356.hg.1	SPON1
TC11000211.hg.1	-7.14	PSR11002831.hg.1	SPON1
TC11000211.hg.1	-8.08	JUC11001357.hg.1	SPON1
TC6_dbb_hap3000	6.51	JUC6_dbb_hap3000[illegible]	HLA-C
TC6_dbb_hap3000	3.35	PSR6_dbb_hap3002[illegible]	HLA-C
TC6_dbb_hap3000	2.82	JUC6_dbb_hap3000[illegible]	HLA-C
TC6_dbb_hap3000	2.8	JUC6_dbb_hap3000[illegible]	HLA-C
TC6_dbb_hap3000	2.21	PSR6_dbb_hap3002[illegible]	HLA-C
TC6_dbb_hap3000	2.17	JUC6_dbb_hap3000[illegible]	HLA-C
TC6_dbb_hap3000	2.14	PSR6_dbb_hap3002[illegible]	HLA-C
TC6_dbb_hap3000	-2.17	JUC6_dbb_hap3001[illegible]	HLA-C
TC6_dbb_hap3000	-2.47	JUC6_dbb_hap3000[illegible]	HLA-C
TC03001227.hg.1	6.48	JUC03010478.hg.1	SGOL1
TC03001227.hg.1	4.69	JUC03010481.hg.1	SGOL1
TC03001227.hg.1	2.48	PSR03021130.hg.1	SGOL1
TC03001227.hg.1	2.42	JUC03010477.hg.1	SGOL1
TC03001227.hg.1	2.4	JUC03010480.hg.1	SGOL1
TC03001227.hg.1	2.28	JUC03010487.hg.1	SGOL1
TC03001227.hg.1	2.04	PSR03021136.hg.1	SGOL1
TC01000999.hg.1	6.47	JUC01008315.hg.1	MAGI3
TC01000999.hg.1	3.97	JUC01008312.hg.1	MAGI3
TC01000999.hg.1	2.63	PSR01015920.hg.1	MAGI3
TC01000999.hg.1	2.12	JUC01008323.hg.1	MAGI3
TC01000999.hg.1	-2.53	JUC01008303.hg.1	MAGI3

TC01000999.hg.1	-3.44	JUC01008307.hg.1	MAGI3
TC02005026.hg.1	6.46	JUC02032391.hg.1	ASB3, GPR7
TC02005026.hg.1	2.73	PSR02029421.hg.1	ASB3, GPR7
TC02005026.hg.1	2.7	PSR02029425.hg.1	ASB3, GPR7
TC02005026.hg.1	-2.01	JUC02032392.hg.1	ASB3, GPR7
TC03001562.hg.1	6.46	JUC03013999.hg.1	CNTN3
TC03001562.hg.1	2.97	JUC03013994.hg.1	CNTN3
TC03001562.hg.1	2.26	PSR03028408.hg.1	CNTN3
TC03001562.hg.1	-2	PSR03028405.hg.1	CNTN3
TC03001562.hg.1	-2.04	PSR03028401.hg.1	CNTN3
TC03001562.hg.1	-2.1	JUC03013988.hg.1	CNTN3
TC03001562.hg.1	-2.57	JUC03013989.hg.1	CNTN3
TC03001562.hg.1	-2.66	JUC03013995.hg.1	CNTN3
TC03001562.hg.1	-2.71	PSR03028400.hg.1	CNTN3
TC03001562.hg.1	-3.27	JUC03013985.hg.1	CNTN3
TC03001562.hg.1	-7.82	JUC03013996.hg.1	CNTN3
TC08001016.hg.1	6.46	JUC08006694.hg.1	PSD3
TC08001016.hg.1	2.42	PSR08012927.hg.1	PSD3
TC08001016.hg.1	2.4	PSR08012926.hg.1	PSD3
TC08001016.hg.1	-2	PSR08012929.hg.1	PSD3
TC08001016.hg.1	-2.07	PSR08012940.hg.1	PSD3
TC08001016.hg.1	-2.08	JUC08006711.hg.1	PSD3
TC08001016.hg.1	-2.13	JUC08006706.hg.1	PSD3
TC08001584.hg.1	6.44	JUC08010627.hg.1	ATAD2
TC08001584.hg.1	3.38	JUC08010603.hg.1	ATAD2
TC08001584.hg.1	2.98	JUC08010594.hg.1	ATAD2
TC08001584.hg.1	2.89	JUC08010613.hg.1	ATAD2
TC08001584.hg.1	2.52	JUC08010606.hg.1	ATAD2
TC08001584.hg.1	2.26	PSR08020799.hg.1	ATAD2
TC08001584.hg.1	2.24	JUC08010607.hg.1	ATAD2
TC08001584.hg.1	2.21	PSR08020790.hg.1	ATAD2
TC08001584.hg.1	2.04	PSR08020803.hg.1	ATAD2
TC08001584.hg.1	-2.13	JUC08010614.hg.1	ATAD2
TC17000830.hg.1	6.43	JUC17005912.hg.1	GPRC5C
TC17000830.hg.1	3.31	PSR17010711.hg.1	GPRC5C
TC05001793.hg.1	6.42	JUC05012544.hg.1	SAR1B
TC05001793.hg.1	-2.05	PSR05024435.hg.1	SAR1B
TC05001793.hg.1	-2.36	PSR05024420.hg.1	SAR1B
TC05001793.hg.1	-2.37	PSR05024419.hg.1	SAR1B
TC05001793.hg.1	-2.41	PSR05024418.hg.1	SAR1B
TC05001793.hg.1	-2.54	PSR05024415.hg.1	SAR1B
TC19001061.hg.1	6.42	JUC19008187.hg.1	STAP2
TC19001061.hg.1	2.4	PSR19014501.hg.1	STAP2
TC19001061.hg.1	-2	PSR19014494.hg.1	STAP2
TC19001061.hg.1	-2.24	JUC19008177.hg.1	STAP2
TC14000554.hg.1	6.41	JUC14003432.hg.1	CALM1, CALI
TC14000554.hg.1	-3.02	PSR14006760.hg.1	CALM1, CALI
TC21000134.hg.1	6.4	JUC21000604.hg.1	MRPS6, SLC
TC21000134.hg.1	5.3	PSR21001123.hg.1	MRPS6, SLC
TC21000134.hg.1	2.97	JUC21000618.hg.1	MRPS6, SLC
TC21000134.hg.1	2.95	JUC21000617.hg.1	MRPS6, SLC
TC21000134.hg.1	2.64	PSR21001147.hg.1	MRPS6, SLC
TC21000134.hg.1	2.15	JUC21000609.hg.1	MRPS6, SLC
TC21000134.hg.1	2	PSR21001149.hg.1	MRPS6, SLC
TC09001585.hg.1	6.36	JUC09010789.hg.1	SCAI
TC09001585.hg.1	3.31	JUC09010783.hg.1	SCAI
TC09001585.hg.1	3.21	PSR09019933.hg.1	SCAI
TC09001585.hg.1	2.56	PSR09019911.hg.1	SCAI

TC09001585.hg.1	2.53	PSR09019928.hg.1	SCAI
TC09001585.hg.1	2.5	JUC09010782.hg.1	SCAI
TC09001585.hg.1	2.27	PSR09019910.hg.1	SCAI
TC09001585.hg.1	2.08	JUC09010781.hg.1	SCAI
TC09001585.hg.1	-2.09	JUC09010798.hg.1	SCAI
TC09001585.hg.1	-2.31	JUC09010784.hg.1	SCAI
TC09001585.hg.1	-4.19	JUC09010787.hg.1	SCAI
TC12001129.hg.1	6.36	JUC12008133.hg.1	ANO2
TC12001129.hg.1	4.52	JUC12008121.hg.1	ANO2
TC12001129.hg.1	4.13	JUC12008124.hg.1	ANO2
TC12001129.hg.1	3.86	PSR12014757.hg.1	ANO2
TC12001129.hg.1	3.23	PSR12014754.hg.1	ANO2
TC12001129.hg.1	2.41	PSR12014755.hg.1	ANO2
TC12001129.hg.1	2.26	PSR12014753.hg.1	ANO2
TC12001129.hg.1	-2.2	JUC12008135.hg.1	ANO2
TC12001129.hg.1	-2.24	PSR12014747.hg.1	ANO2
TC12001129.hg.1	-2.27	PSR12014780.hg.1	ANO2
TC12001129.hg.1	-2.32	PSR12014766.hg.1	ANO2
TC12001129.hg.1	-2.4	PSR12014779.hg.1	ANO2
TC12001129.hg.1	-2.41	PSR12014762.hg.1	ANO2
TC12001129.hg.1	-2.59	JUC12008123.hg.1	ANO2
TC12001129.hg.1	-2.74	PSR12014765.hg.1	ANO2
TC12001129.hg.1	-2.93	PSR12014772.hg.1	ANO2
TC12001129.hg.1	-3.93	JUC12008107.hg.1	ANO2
TC22000318.hg.1	6.36	JUC22002199.hg.1	CACNA1I
TC22000318.hg.1	5.64	JUC22002184.hg.1	CACNA1I
TC22000318.hg.1	4.76	JUC22002180.hg.1	CACNA1I
TC22000318.hg.1	3.37	JUC22002191.hg.1	CACNA1I
TC22000318.hg.1	3.24	JUC22002202.hg.1	CACNA1I
TC22000318.hg.1	2.79	JUC22002206.hg.1	CACNA1I
TC22000318.hg.1	2.35	PSR22006053.hg.1	CACNA1I
TC22000318.hg.1	2.34	JUC22002181.hg.1	CACNA1I
TC22000318.hg.1	2.2	JUC22002176.hg.1	CACNA1I
TC22000318.hg.1	2.09	JUC22002186.hg.1	CACNA1I
TC22000318.hg.1	2.08	PSR22006054.hg.1	CACNA1I
TC22000318.hg.1	-2.76	JUC22002178.hg.1	CACNA1I
TC14000277.hg.1	6.35	PSR14002948.hg.1	KLHDC1
TC14000277.hg.1	2	JUC14001366.hg.1	KLHDC1
TC02004920.hg.1	6.33	JUC02031214.hg.1	LRRFIP1
TC02004920.hg.1	3.63	JUC02031203.hg.1	LRRFIP1
TC02004920.hg.1	3.37	JUC02031184.hg.1	LRRFIP1
TC02004920.hg.1	3.08	JUC02031192.hg.1	LRRFIP1
TC02004920.hg.1	2.78	JUC02031178.hg.1	LRRFIP1
TC02004920.hg.1	2.5	JUC02031198.hg.1	LRRFIP1
TC02004920.hg.1	2.09	PSR02023125.hg.1	LRRFIP1
TC02004920.hg.1	2.02	JUC02031210.hg.1	LRRFIP1
TC02004920.hg.1	2	JUC02031206.hg.1	LRRFIP1
TC02004920.hg.1	-2.07	PSR02023155.hg.1	LRRFIP1
TC02004920.hg.1	-2.44	PSR02023150.hg.1	LRRFIP1
TC02004920.hg.1	-3.74	JUC02031218.hg.1	LRRFIP1
TC05000988.hg.1	6.33	JUC05007364.hg.1	FAM153B, LC
TC05000988.hg.1	2.79	JUC05007387.hg.1	FAM153B, LC
TC05000988.hg.1	2	JUC05007405.hg.1	FAM153B, LC
TC05000988.hg.1	2	JUC05007407.hg.1	FAM153B, LC
TC05000988.hg.1	-2.25	PSR05014238.hg.1	FAM153B, LC
TC05000988.hg.1	-2.68	PSR05014221.hg.1	FAM153B, LC
TC08001282.hg.1	6.33	JUC08008502.hg.1	PDE7A
TC08001282.hg.1	2.69	JUC08008494.hg.1	PDE7A

TC08001282.hg.1	2.19	JUC08008490.hg.1	PDE7A
TC08001282.hg.1	2.13	PSR08016509.hg.1	PDE7A
TC08001282.hg.1	-2.11	JUC08008497.hg.1	PDE7A
TC08001282.hg.1	-2.57	JUC08008492.hg.1	PDE7A
TC08001282.hg.1	-2.85	PSR08016494.hg.1	PDE7A
TC08001282.hg.1	-3.06	PSR08016489.hg.1	PDE7A
TC12001837.hg.1	6.33	PSR12024172.hg.1	NTN4
TC12001837.hg.1	5.81	PSR12024177.hg.1	NTN4
TC12001837.hg.1	5.28	JUC12013425.hg.1	NTN4
TC12001837.hg.1	3.8	JUC12013421.hg.1	NTN4
TC12001837.hg.1	3.3	JUC12013426.hg.1	NTN4
TC12001837.hg.1	3.21	JUC12013418.hg.1	NTN4
TC12001837.hg.1	3.01	PSR12024173.hg.1	NTN4
TC12001837.hg.1	2.83	JUC12013416.hg.1	NTN4
TC12001837.hg.1	-4.19	JUC12013417.hg.1	NTN4
TC01000734.hg.1	6.32	JUC01006111.hg.1	SGIP1
TC01000734.hg.1	5.43	PSR01011767.hg.1	SGIP1
TC01000734.hg.1	4.91	PSR01011825.hg.1	SGIP1
TC01000734.hg.1	4.89	JUC01006115.hg.1	SGIP1
TC01000734.hg.1	4.89	JUC01006139.hg.1	SGIP1
TC01000734.hg.1	4.86	PSR01011823.hg.1	SGIP1
TC01000734.hg.1	4.31	PSR01011773.hg.1	SGIP1
TC01000734.hg.1	4.12	JUC01006106.hg.1	SGIP1
TC01000734.hg.1	3.84	JUC01006099.hg.1	SGIP1
TC01000734.hg.1	3.74	PSR01011801.hg.1	SGIP1
TC01000734.hg.1	3.66	PSR01011783.hg.1	SGIP1
TC01000734.hg.1	3.66	PSR01011818.hg.1	SGIP1
TC01000734.hg.1	3.39	PSR01011775.hg.1	SGIP1
TC01000734.hg.1	3.06	JUC01006134.hg.1	SGIP1
TC01000734.hg.1	3.03	PSR01011776.hg.1	SGIP1
TC01000734.hg.1	2.97	PSR01011812.hg.1	SGIP1
TC01000734.hg.1	2.76	JUC01006098.hg.1	SGIP1
TC01000734.hg.1	2.65	JUC01006141.hg.1	SGIP1
TC01000734.hg.1	2.46	JUC01006127.hg.1	SGIP1
TC01000734.hg.1	2.37	PSR01011779.hg.1	SGIP1
TC01000734.hg.1	2.05	PSR01011814.hg.1	SGIP1
TC01000734.hg.1	2.04	JUC01006116.hg.1	SGIP1
TC01000734.hg.1	2.02	PSR01011799.hg.1	SGIP1
TC01000734.hg.1	-2.13	PSR01011806.hg.1	SGIP1
TC01000734.hg.1	-2.21	PSR01011778.hg.1	SGIP1
TC01000734.hg.1	-2.37	PSR01011774.hg.1	SGIP1
TC01000734.hg.1	-2.44	JUC01006136.hg.1	SGIP1
TC01000734.hg.1	-2.7	JUC01006120.hg.1	SGIP1
TC01000734.hg.1	-2.74	PSR01011795.hg.1	SGIP1
TC01000734.hg.1	-2.86	JUC01006125.hg.1	SGIP1
TC01000734.hg.1	-2.88	JUC01006105.hg.1	SGIP1
TC01000734.hg.1	-2.94	JUC01006130.hg.1	SGIP1
TC01000734.hg.1	-3.19	JUC01006123.hg.1	SGIP1
TC01000734.hg.1	-3.86	JUC01006102.hg.1	SGIP1
TC07003392.hg.1	6.32	JUC07021812.hg.1	FAM188B, AC
TC07003392.hg.1	5.27	PSR07003132.hg.1	FAM188B, AC
TC07003392.hg.1	4.32	PSR07003135.hg.1	FAM188B, AC
TC07003392.hg.1	4.06	PSR07003122.hg.1	FAM188B, AC
TC07003392.hg.1	3.88	JUC07021796.hg.1	FAM188B, AC
TC07003392.hg.1	3.2	JUC07021795.hg.1	FAM188B, AC
TC07003392.hg.1	2.49	PSR07003131.hg.1	FAM188B, AC
TC07003392.hg.1	2.06	PSR07003120.hg.1	FAM188B, AC
TC07003392.hg.1	-2.06	PSR07003108.hg.1	FAM188B, AC

TC07003392.hg.1	-2.06	PSR07003110.hg.1	FAM188B, AC
TC07003392.hg.1	-2.08	PSR07003109.hg.1	FAM188B, AC
TC07003392.hg.1	-2.09	PSR07003128.hg.1	FAM188B, AC
TC07003392.hg.1	-2.09	JUC07021794.hg.1	FAM188B, AC
TC07003392.hg.1	-2.11	PSR07003090.hg.1	FAM188B, AC
TC07003392.hg.1	-2.13	PSR07003129.hg.1	FAM188B, AC
TC07003392.hg.1	-2.13	JUC07021798.hg.1	FAM188B, AC
TC07003392.hg.1	-2.21	JUC07021800.hg.1	FAM188B, AC
TC07003392.hg.1	-2.24	PSR07003098.hg.1	FAM188B, AC
TC07003392.hg.1	-2.27	JUC07021793.hg.1	FAM188B, AC
TC07003392.hg.1	-2.3	JUC07021818.hg.1	FAM188B, AC
TC07003392.hg.1	-2.32	JUC07021813.hg.1	FAM188B, AC
TC07003392.hg.1	-2.38	PSR07003114.hg.1	FAM188B, AC
TC07003392.hg.1	-2.42	PSR07003103.hg.1	FAM188B, AC
TC07003392.hg.1	-2.49	PSR07003111.hg.1	FAM188B, AC
TC07003392.hg.1	-2.53	PSR07003124.hg.1	FAM188B, AC
TC07003392.hg.1	-2.54	JUC07021805.hg.1	FAM188B, AC
TC07003392.hg.1	-2.56	JUC07021814.hg.1	FAM188B, AC
TC07003392.hg.1	-2.63	JUC07021803.hg.1	FAM188B, AC
TC07003392.hg.1	-2.71	PSR07003099.hg.1	FAM188B, AC
TC07003392.hg.1	-2.76	JUC07021810.hg.1	FAM188B, AC
TC07003392.hg.1	-2.82	PSR07003093.hg.1	FAM188B, AC
TC07003392.hg.1	-2.83	PSR07003100.hg.1	FAM188B, AC
TC07003392.hg.1	-2.93	PSR07003095.hg.1	FAM188B, AC
TC07003392.hg.1	-3.02	JUC07021806.hg.1	FAM188B, AC
TC07003392.hg.1	-3.33	PSR07003094.hg.1	FAM188B, AC
TC07003392.hg.1	-3.34	JUC07021802.hg.1	FAM188B, AC
TC07003392.hg.1	-3.38	JUC07021815.hg.1	FAM188B, AC
TC07003392.hg.1	-3.4	PSR07003092.hg.1	FAM188B, AC
TC07003392.hg.1	-3.43	JUC07021819.hg.1	FAM188B, AC
TC07003392.hg.1	-3.48	PSR07003091.hg.1	FAM188B, AC
TC07003392.hg.1	-3.51	JUC07021807.hg.1	FAM188B, AC
TC07003392.hg.1	-3.63	PSR07003113.hg.1	FAM188B, AC
TC07003392.hg.1	-3.86	JUC07021820.hg.1	FAM188B, AC
TC07003392.hg.1	-3.88	PSR07003104.hg.1	FAM188B, AC
TC07003392.hg.1	-4.21	PSR07003101.hg.1	FAM188B, AC
TC07003392.hg.1	-4.26	JUC07021799.hg.1	FAM188B, AC
TC07003392.hg.1	-4.62	PSR07003102.hg.1	FAM188B, AC
TC07003392.hg.1	-4.72	PSR07003107.hg.1	FAM188B, AC
TC07003392.hg.1	-5.06	JUC07021788.hg.1	FAM188B, AC
TC07003392.hg.1	-5.28	JUC07021789.hg.1	FAM188B, AC
TC0Y000073.hg.1	6.32	PSR0Y000841.hg.1	RPS4Y2
TC0Y000073.hg.1	3.09	JUC0Y000372.hg.1	RPS4Y2
TC0Y000073.hg.1	2.67	PSR0Y000840.hg.1	RPS4Y2
TC11000332.hg.1	6.31	JUC11002277.hg.1	CD44
TC11000332.hg.1	4.67	JUC11002270.hg.1	CD44
TC11000332.hg.1	3.31	JUC11002276.hg.1	CD44
TC11000332.hg.1	3.18	JUC11002269.hg.1	CD44
TC11000332.hg.1	2.97	JUC11002280.hg.1	CD44
TC11000332.hg.1	2.83	JUC11002283.hg.1	CD44
TC11000332.hg.1	2.15	JUC11002281.hg.1	CD44
TC11000332.hg.1	-2.37	PSR11004275.hg.1	CD44
TC11000332.hg.1	-2.53	JUC11002273.hg.1	CD44
TC11000332.hg.1	-2.68	PSR11004279.hg.1	CD44
TC11000332.hg.1	-2.68	JUC11002295.hg.1	CD44
TC11000332.hg.1	-3.24	JUC11002296.hg.1	CD44
TC11000332.hg.1	-3.56	PSR11004280.hg.1	CD44
TC11000332.hg.1	-7.87	JUC11002292.hg.1	CD44

TC08001248.hg.1	6.3	PSR08016170.hg.1	TOX
TC08001248.hg.1	4.38	JUC08008328.hg.1	TOX
TC08001248.hg.1	3.44	PSR08016174.hg.1	TOX
TC08001248.hg.1	2.09	JUC08008331.hg.1	TOX
TC05001030.hg.1	6.29	JUC05007779.hg.1	FAM153C
TC05001030.hg.1	2.78	JUC05007778.hg.1	FAM153C
TC05001030.hg.1	-2.7	PSR05014979.hg.1	FAM153C
TC05001030.hg.1	-2.88	JUC05007775.hg.1	FAM153C
TC04000766.hg.1	6.28	JUC04005640.hg.1	ARFIP1
TC04000766.hg.1	3.96	JUC04005637.hg.1	ARFIP1
TC04000766.hg.1	3.41	JUC04005654.hg.1	ARFIP1
TC04000766.hg.1	2.61	PSR04010524.hg.1	ARFIP1
TC04000766.hg.1	2.49	JUC04005639.hg.1	ARFIP1
TC04000766.hg.1	2.17	PSR04010522.hg.1	ARFIP1
TC04000766.hg.1	2.02	PSR04010531.hg.1	ARFIP1
TC17000015.hg.1	6.28	JUC17000060.hg.1	SERPINF1
TC17000015.hg.1	2.24	PSR17000142.hg.1	SERPINF1
TC17000015.hg.1	2.06	JUC17000065.hg.1	SERPINF1
TC17000015.hg.1	-2.71	JUC17000062.hg.1	SERPINF1
TC17000015.hg.1	-3.08	PSR17000138.hg.1	SERPINF1
TC17000015.hg.1	-4.18	JUC17000061.hg.1	SERPINF1
TC17000015.hg.1	-7.34	PSR17000137.hg.1	SERPINF1
TC17000015.hg.1	-7.38	PSR17000135.hg.1	SERPINF1
TC18000150.hg.1	6.27	JUC18001214.hg.1	FHOD3
TC18000150.hg.1	4.07	JUC18001207.hg.1	FHOD3
TC18000150.hg.1	3.64	PSR18001741.hg.1	FHOD3
TC18000150.hg.1	3.47	PSR18001739.hg.1	FHOD3
TC18000150.hg.1	3.03	JUC18001229.hg.1	FHOD3
TC18000150.hg.1	2.9	JUC18001197.hg.1	FHOD3
TC18000150.hg.1	2.74	PSR18001737.hg.1	FHOD3
TC18000150.hg.1	2.56	PSR18001740.hg.1	FHOD3
TC18000150.hg.1	2.51	JUC18001228.hg.1	FHOD3
TC18000150.hg.1	2.15	PSR18001747.hg.1	FHOD3
TC18000150.hg.1	-2.09	PSR18001735.hg.1	FHOD3
TC18000150.hg.1	-2.14	PSR18001756.hg.1	FHOD3
TC18000150.hg.1	-2.17	PSR18001733.hg.1	FHOD3
TC18000150.hg.1	-2.17	PSR18001765.hg.1	FHOD3
TC18000150.hg.1	-2.21	JUC18001221.hg.1	FHOD3
TC18000150.hg.1	-2.22	PSR18001762.hg.1	FHOD3
TC18000150.hg.1	-2.26	PSR18001743.hg.1	FHOD3
TC18000150.hg.1	-2.27	JUC18001200.hg.1	FHOD3
TC18000150.hg.1	-2.33	PSR18001738.hg.1	FHOD3
TC18000150.hg.1	-2.44	PSR18001745.hg.1	FHOD3
TC18000150.hg.1	-2.44	PSR18001758.hg.1	FHOD3
TC18000150.hg.1	-2.45	PSR18001744.hg.1	FHOD3
TC18000150.hg.1	-2.74	PSR18001755.hg.1	FHOD3
TC18000150.hg.1	-3.03	PSR18001752.hg.1	FHOD3
TC18000150.hg.1	-3.03	JUC18001213.hg.1	FHOD3
TC18000150.hg.1	-3.61	JUC18001202.hg.1	FHOD3
TC18000150.hg.1	-3.77	PSR18001753.hg.1	FHOD3
TC18000150.hg.1	-4.09	JUC18001223.hg.1	FHOD3
TC18000150.hg.1	-4.65	PSR18001750.hg.1	FHOD3
TC18000150.hg.1	-4.96	JUC18001203.hg.1	FHOD3
TC18000150.hg.1	-6.59	JUC18001224.hg.1	FHOD3
TC18000150.hg.1	-7.02	JUC18001210.hg.1	FHOD3
TC12001307.hg.1	6.25	JUC12009447.hg.1	SOX5
TC12001307.hg.1	4.31	JUC12009427.hg.1	SOX5
TC12001307.hg.1	3.49	JUC12009448.hg.1	SOX5

TC12001307.hg.1	2.89	JUC12009431.hg.1	SOX5
TC12001307.hg.1	2.85	JUC12009444.hg.1	SOX5
TC12001307.hg.1	2.67	JUC12009453.hg.1	SOX5
TC12001307.hg.1	2.4	PSR12017165.hg.1	SOX5
TC12001307.hg.1	2.33	JUC12009443.hg.1	SOX5
TC12001307.hg.1	2.29	PSR12017171.hg.1	SOX5
TC12001307.hg.1	2.02	PSR12017158.hg.1	SOX5
TC12001307.hg.1	-2.15	PSR12017146.hg.1	SOX5
TC01001586.hg.1	6.23	JUC01013116.hg.1	SMG7
TC01001586.hg.1	3.37	JUC01013091.hg.1	SMG7
TC01001586.hg.1	2.6	JUC01013115.hg.1	SMG7
TC01001586.hg.1	-2.4	PSR01024601.hg.1	SMG7
TC13001724.hg.1	6.22	JUC13008471.hg.1	SOHLH2, CC
TC13001724.hg.1	2.41	PSR13006364.hg.1	SOHLH2, CC
TC05001617.hg.1	6.2	JUC05011398.hg.1	ERAP1
TC05001617.hg.1	3.53	PSR05022094.hg.1	ERAP1
TC05001617.hg.1	3.18	PSR05022078.hg.1	ERAP1
TC05001617.hg.1	2.99	PSR05022067.hg.1	ERAP1
TC05001617.hg.1	2.92	PSR05022095.hg.1	ERAP1
TC05001617.hg.1	2.85	PSR05022051.hg.1	ERAP1
TC05001617.hg.1	2.66	PSR05022089.hg.1	ERAP1
TC05001617.hg.1	2.61	PSR05022070.hg.1	ERAP1
TC05001617.hg.1	2.13	PSR05022093.hg.1	ERAP1
TC05001617.hg.1	2.12	PSR05022090.hg.1	ERAP1
TC05001617.hg.1	-2.03	JUC05011378.hg.1	ERAP1
TC05001617.hg.1	-2.39	JUC05011397.hg.1	ERAP1
TC05001617.hg.1	-2.49	JUC05011390.hg.1	ERAP1
TC05001617.hg.1	-2.8	JUC05011399.hg.1	ERAP1
TC05001617.hg.1	-3.56	JUC05011392.hg.1	ERAP1
TC14000819.hg.1	6.2	JUC14004565.hg.1	ADSSL1
TC14000819.hg.1	5.33	PSR14008952.hg.1	ADSSL1
TC14000819.hg.1	5.25	PSR14008953.hg.1	ADSSL1
TC14000819.hg.1	5.15	PSR14008967.hg.1	ADSSL1
TC14000819.hg.1	5.14	PSR14008970.hg.1	ADSSL1
TC14000819.hg.1	4.07	JUC14004561.hg.1	ADSSL1
TC14000819.hg.1	4	PSR14008968.hg.1	ADSSL1
TC14000819.hg.1	3.57	PSR14008951.hg.1	ADSSL1
TC14000819.hg.1	2.61	PSR14008966.hg.1	ADSSL1
TC14000819.hg.1	2.45	PSR14008954.hg.1	ADSSL1
TC14000819.hg.1	-2.03	JUC14004564.hg.1	ADSSL1
TC14000819.hg.1	-2.31	PSR14008965.hg.1	ADSSL1
TC14000819.hg.1	-3.13	JUC14004555.hg.1	ADSSL1
TC14000819.hg.1	-4.02	JUC14004563.hg.1	ADSSL1
TC18000161.hg.1	6.2	JUC18001326.hg.1	SLC14A2
TC18000161.hg.1	2.43	JUC18001313.hg.1	SLC14A2
TC18000161.hg.1	2.2	PSR18001880.hg.1	SLC14A2
TC18000161.hg.1	2.19	PSR18001881.hg.1	SLC14A2
TC18000161.hg.1	2.05	PSR18001878.hg.1	SLC14A2
TC18000161.hg.1	-2.11	PSR18001871.hg.1	SLC14A2
TC18000161.hg.1	-2.14	JUC18001325.hg.1	SLC14A2
TC18000161.hg.1	-2.25	JUC18001321.hg.1	SLC14A2
TC18000161.hg.1	-2.43	PSR18001889.hg.1	SLC14A2
TC18000161.hg.1	-3.31	PSR18001873.hg.1	SLC14A2
TC19000530.hg.1	6.2	JUC19004295.hg.1	RYR1
TC19000530.hg.1	2.47	JUC19004290.hg.1	RYR1
TC19000530.hg.1	2.24	JUC19004195.hg.1	RYR1
TC19000530.hg.1	2.18	PSR19007127.hg.1	RYR1
TC19000530.hg.1	2.08	JUC19004240.hg.1	RYR1

TC20000176.hg.1	6.2	JUC20001177.hg.1	FAM182A
TC20000176.hg.1	3.19	JUC20001178.hg.1	FAM182A
TC20000176.hg.1	2.88	JUC20001184.hg.1	FAM182A
TC20000176.hg.1	2.45	JUC20001175.hg.1	FAM182A
TC20000176.hg.1	2.12	PSR20002201.hg.1	FAM182A
TC20000176.hg.1	2.03	JUC20001185.hg.1	FAM182A
TC20000176.hg.1	2.03	JUC20001186.hg.1	FAM182A
TC20000176.hg.1	-2.23	PSR20002187.hg.1	FAM182A
TC20000176.hg.1	-3.37	PSR20002184.hg.1	FAM182A
TC08000769.hg.1	6.19	JUC08005285.hg.1	TG
TC08000769.hg.1	5.27	JUC08005334.hg.1	TG
TC08000769.hg.1	4.44	JUC08005304.hg.1	TG
TC08000769.hg.1	4.37	JUC08005284.hg.1	TG
TC08000769.hg.1	4.28	JUC08005295.hg.1	TG
TC08000769.hg.1	3.92	JUC08005305.hg.1	TG
TC08000769.hg.1	3.76	JUC08005337.hg.1	TG
TC08000769.hg.1	3.66	JUC08005298.hg.1	TG
TC08000769.hg.1	3.06	JUC08005327.hg.1	TG
TC08000769.hg.1	2.79	PSR08010284.hg.1	TG
TC08000769.hg.1	2.68	PSR08010308.hg.1	TG
TC08000769.hg.1	2.65	JUC08005300.hg.1	TG
TC08000769.hg.1	2.65	JUC08005326.hg.1	TG
TC08000769.hg.1	2.43	JUC08005277.hg.1	TG
TC08000769.hg.1	2.39	PSR08010310.hg.1	TG
TC08000769.hg.1	2.26	PSR08010295.hg.1	TG
TC19001156.hg.1	6.19	JUC19009319.hg.1	COL5A3
TC19001156.hg.1	2.31	JUC19009333.hg.1	COL5A3
TC19001156.hg.1	2.02	JUC19009321.hg.1	COL5A3
TC19001156.hg.1	-2.02	PSR19016017.hg.1	COL5A3
TC19001156.hg.1	-2.03	PSR19016015.hg.1	COL5A3
TC19001156.hg.1	-2.08	PSR19015953.hg.1	COL5A3
TC19001156.hg.1	-2.15	PSR19016013.hg.1	COL5A3
TC19001156.hg.1	-2.17	PSR19016014.hg.1	COL5A3
TC19001156.hg.1	-2.17	JUC19009322.hg.1	COL5A3
TC19001156.hg.1	-2.27	JUC19009303.hg.1	COL5A3
TC19001156.hg.1	-2.35	JUC19009329.hg.1	COL5A3
TC19001156.hg.1	-3.16	PSR19015957.hg.1	COL5A3
TC19001156.hg.1	-3.49	JUC19009305.hg.1	COL5A3
TC19001156.hg.1	-3.56	JUC19009332.hg.1	COL5A3
TC19001156.hg.1	-3.7	PSR19016018.hg.1	COL5A3
TC19001156.hg.1	-3.78	PSR19015952.hg.1	COL5A3
TC19001156.hg.1	-4.38	JUC19009336.hg.1	COL5A3
TC19001156.hg.1	-4.92	JUC19009280.hg.1	COL5A3
TC19001156.hg.1	-5.82	JUC19009285.hg.1	COL5A3
TC19001156.hg.1	-6.68	PSR19016016.hg.1	COL5A3
TC19001156.hg.1	-6.79	JUC19009315.hg.1	COL5A3
TC19001156.hg.1	-9.79	JUC19009277.hg.1	COL5A3
TC19001156.hg.1	-15.02	JUC19009314.hg.1	COL5A3
TC17000924.hg.1	6.18	JUC17006882.hg.1	BAIAP2
TC17000924.hg.1	2.51	PSR17012168.hg.1	BAIAP2
TC17000924.hg.1	2.31	PSR17012173.hg.1	BAIAP2
TC17000924.hg.1	-2.3	JUC17006867.hg.1	BAIAP2
TC17000924.hg.1	-2.35	JUC17006875.hg.1	BAIAP2
TC01001204.hg.1	6.17	JUC01010211.hg.1	PIP5K1A
TC01001204.hg.1	4.5	JUC01010194.hg.1	PIP5K1A
TC01001204.hg.1	2.72	JUC01010193.hg.1	PIP5K1A
TC01001204.hg.1	2.53	JUC01010199.hg.1	PIP5K1A
TC01001204.hg.1	-2.12	JUC01010200.hg.1	PIP5K1A

TC01001204.hg.1	-3.71	PSR01018613.hg.1	PIP5K1A
TC04001487.hg.1	6.17	JUC04011077.hg.1	ARSJ
TC04001487.hg.1	3.87	PSR04021107.hg.1	ARSJ
TC04001487.hg.1	2.54	JUC04011086.hg.1	ARSJ
TC04001487.hg.1	2.44	PSR04021106.hg.1	ARSJ
TC04001487.hg.1	2.36	PSR04021082.hg.1	ARSJ
TC04001487.hg.1	-2.06	JUC04011084.hg.1	ARSJ
TC01003311.hg.1	6.16	PSR01051374.hg.1	CLK2
TC01003311.hg.1	4.71	PSR01051372.hg.1	CLK2
TC01003311.hg.1	2.49	PSR01051400.hg.1	CLK2
TC01003311.hg.1	2.11	JUC01027006.hg.1	CLK2
TC01003311.hg.1	2.09	PSR01051387.hg.1	CLK2
TC01003311.hg.1	2.05	PSR01051401.hg.1	CLK2
TC01003311.hg.1	2.03	PSR01051402.hg.1	CLK2
TC01003311.hg.1	-3.98	JUC01027012.hg.1	CLK2
TC0X000821.hg.1	6.16	JUC0X005468.hg.1	NLGN4X
TC0X000821.hg.1	3.72	JUC0X005472.hg.1	NLGN4X
TC0X000821.hg.1	3.38	PSR0X011188.hg.1	NLGN4X
TC0X000821.hg.1	3.11	PSR0X011197.hg.1	NLGN4X
TC0X000821.hg.1	3.07	PSR0X011193.hg.1	NLGN4X
TC0X000821.hg.1	2.65	PSR0X011194.hg.1	NLGN4X
TC0X000821.hg.1	2.61	JUC0X005470.hg.1	NLGN4X
TC0X000821.hg.1	2.61	JUC0X005483.hg.1	NLGN4X
TC0X000821.hg.1	2.39	JUC0X005471.hg.1	NLGN4X
TC0X000821.hg.1	2.08	PSR0X011195.hg.1	NLGN4X
TC0X000821.hg.1	-2.06	PSR0X011181.hg.1	NLGN4X
TC0X000821.hg.1	-2.59	PSR0X011191.hg.1	NLGN4X
TC08001497.hg.1	6.15	JUC08009991.hg.1	UBR5
TC08001497.hg.1	4.5	JUC08009971.hg.1	UBR5
TC08001497.hg.1	2.91	PSR08019644.hg.1	UBR5
TC08001497.hg.1	2.9	PSR08019697.hg.1	UBR5
TC08001497.hg.1	2.29	PSR08019705.hg.1	UBR5
TC08001497.hg.1	2.15	JUC08009988.hg.1	UBR5
TC08001497.hg.1	2.13	PSR08019706.hg.1	UBR5
TC08001497.hg.1	2.11	JUC08009961.hg.1	UBR5
TC08001497.hg.1	2.04	JUC08009985.hg.1	UBR5
TC08001497.hg.1	2.03	JUC08009993.hg.1	UBR5
TC08001497.hg.1	-3	JUC08009957.hg.1	UBR5
TC20000368.hg.1	6.15	JUC20002922.hg.1	EYA2
TC20000368.hg.1	5	JUC20002909.hg.1	EYA2
TC20000368.hg.1	3.78	PSR20005361.hg.1	EYA2
TC20000368.hg.1	3.75	JUC20002920.hg.1	EYA2
TC20000368.hg.1	3.49	JUC20002914.hg.1	EYA2
TC20000368.hg.1	2.88	JUC20002910.hg.1	EYA2
TC20000368.hg.1	2.82	JUC20002931.hg.1	EYA2
TC20000368.hg.1	2.6	JUC20002924.hg.1	EYA2
TC20000368.hg.1	2.3	JUC20002908.hg.1	EYA2
TC20000368.hg.1	2.29	PSR20005374.hg.1	EYA2
TC20000368.hg.1	2.22	PSR20005375.hg.1	EYA2
TC20000368.hg.1	2.17	JUC20002926.hg.1	EYA2
TC20000368.hg.1	-2.12	PSR20005363.hg.1	EYA2
TC20000368.hg.1	-2.14	PSR20005372.hg.1	EYA2
TC20000368.hg.1	-2.24	PSR20005382.hg.1	EYA2
TC20000368.hg.1	-2.39	PSR20005379.hg.1	EYA2
TC20000368.hg.1	-2.41	JUC20002927.hg.1	EYA2
TC20000368.hg.1	-2.81	JUC20002912.hg.1	EYA2
TC20000368.hg.1	-3.07	JUC20002930.hg.1	EYA2
TC20000368.hg.1	-3.09	PSR20005384.hg.1	EYA2

TC20000368.hg.1	-3.35	PSR20005358.hg.1	EYA2
TC20000368.hg.1	-4.45	PSR20005356.hg.1	EYA2
TC20000368.hg.1	-4.46	PSR20005357.hg.1	EYA2
TC20000368.hg.1	-4.58	PSR20005355.hg.1	EYA2
TC20000368.hg.1	-6.17	JUC20002907.hg.1	EYA2
TC04002931.hg.1	6.14	JUC04017072.hg.1	PDGFRA
TC04002931.hg.1	5.16	JUC04017075.hg.1	PDGFRA
TC04002931.hg.1	3.98	JUC04017073.hg.1	PDGFRA
TC04002931.hg.1	3.64	JUC04017092.hg.1	PDGFRA
TC04002931.hg.1	3.41	PSR04004383.hg.1	PDGFRA
TC04002931.hg.1	3.26	JUC04017077.hg.1	PDGFRA
TC04002931.hg.1	3.01	PSR04004360.hg.1	PDGFRA
TC04002931.hg.1	3.01	PSR04004362.hg.1	PDGFRA
TC04002931.hg.1	2.3	PSR04004363.hg.1	PDGFRA
TC04002931.hg.1	-2.44	JUC04017102.hg.1	PDGFRA
TC15000846.hg.1	6.14	JUC15004137.hg.1	FANCI
TC15000846.hg.1	2.49	JUC15004147.hg.1	FANCI
TC15000846.hg.1	2.35	PSR15007805.hg.1	FANCI
TC15000846.hg.1	-2.41	JUC15004131.hg.1	FANCI
TC01003532.hg.1	6.11	JUC01028596.hg.1	SNORD47, S
TC01003532.hg.1	2.5	JUC01028600.hg.1	SNORD47, S
TC01003532.hg.1	2.38	PSR01054836.hg.1	SNORD47, S
TC01003532.hg.1	2.06	PSR01054799.hg.1	SNORD47, S
TC01003532.hg.1	-4.38	JUC01028606.hg.1	SNORD47, S
TC01003532.hg.1	-4.9	JUC01028597.hg.1	SNORD47, S
TC07000178.hg.1	6.1	PSR07002787.hg.1	CREB5, LOC
TC07000178.hg.1	5.67	PSR07002771.hg.1	CREB5, LOC
TC07000178.hg.1	4.8	PSR07002779.hg.1	CREB5, LOC
TC07000178.hg.1	4.24	PSR07002793.hg.1	CREB5, LOC
TC07000178.hg.1	4.15	PSR07002770.hg.1	CREB5, LOC
TC07000178.hg.1	3.6	PSR07002789.hg.1	CREB5, LOC
TC07000178.hg.1	3.26	PSR07002772.hg.1	CREB5, LOC
TC07000178.hg.1	3.23	JUC07001341.hg.1	CREB5, LOC
TC07000178.hg.1	2.91	PSR07002773.hg.1	CREB5, LOC
TC07000178.hg.1	2.77	PSR07002798.hg.1	CREB5, LOC
TC07000178.hg.1	2.67	PSR07002799.hg.1	CREB5, LOC
TC07000178.hg.1	2.57	PSR07002769.hg.1	CREB5, LOC
TC07000178.hg.1	2.43	PSR07002797.hg.1	CREB5, LOC
TC07000178.hg.1	2.38	PSR07002785.hg.1	CREB5, LOC
TC07000178.hg.1	2.25	PSR07002803.hg.1	CREB5, LOC
TC07000178.hg.1	2.17	PSR07002781.hg.1	CREB5, LOC
TC07000178.hg.1	2.17	PSR07002783.hg.1	CREB5, LOC
TC07000178.hg.1	-2.71	JUC07001349.hg.1	CREB5, LOC
TC10001398.hg.1	6.1	JUC10010247.hg.1	P4HA1
TC10001398.hg.1	5.35	PSR10017612.hg.1	P4HA1
TC10001398.hg.1	4.75	PSR10017588.hg.1	P4HA1
TC10001398.hg.1	3.95	JUC10010265.hg.1	P4HA1
TC10001398.hg.1	3.77	JUC10010262.hg.1	P4HA1
TC10001398.hg.1	3.45	JUC10010252.hg.1	P4HA1
TC10001398.hg.1	2.95	JUC10010266.hg.1	P4HA1
TC10001398.hg.1	2.57	PSR10017613.hg.1	P4HA1
TC10001398.hg.1	2.27	JUC10010264.hg.1	P4HA1
TC10001398.hg.1	-3.17	PSR10017611.hg.1	P4HA1
TC20000391.hg.1	6.1	JUC20003114.hg.1	SLC9A8
TC20000391.hg.1	2.11	PSR20005637.hg.1	SLC9A8
TC22000700.hg.1	6.09	PSR22012322.hg.1	APOL3
TC22000700.hg.1	6.08	JUC22005104.hg.1	APOL3
TC22000700.hg.1	3.83	PSR22012313.hg.1	APOL3

TC22000700.hg.1	3.21	PSR22012309.hg.1	APOL3
TC22000700.hg.1	3.17	JUC22005094.hg.1	APOL3
TC22000700.hg.1	2.98	PSR22012321.hg.1	APOL3
TC22000700.hg.1	2.93	PSR22012311.hg.1	APOL3
TC22000700.hg.1	2.83	JUC22005111.hg.1	APOL3
TC22000700.hg.1	2.59	PSR22012306.hg.1	APOL3
TC22000700.hg.1	2.58	PSR22012308.hg.1	APOL3
TC22000700.hg.1	2.46	PSR22012307.hg.1	APOL3
TC22000700.hg.1	2.26	PSR22012324.hg.1	APOL3
TC22000700.hg.1	2.12	PSR22012299.hg.1	APOL3
TC22000700.hg.1	-2.21	PSR22012302.hg.1	APOL3
TC0X001024.hg.1	6.08	JUC0X006839.hg.1	GRIPAP1
TC0X001024.hg.1	2.01	JUC0X006841.hg.1	GRIPAP1
TC0X001024.hg.1	2	PSR0X013634.hg.1	GRIPAP1
TC0X001024.hg.1	-2.09	PSR0X013632.hg.1	GRIPAP1
TC0X001024.hg.1	-2.28	JUC0X006821.hg.1	GRIPAP1
TC13000051.hg.1	6.08	PSR13000426.hg.1	C1QTNF9, SI
TC13000051.hg.1	5.17	PSR13000377.hg.1	C1QTNF9, SI
TC13000051.hg.1	4.26	JUC13000224.hg.1	C1QTNF9, SI
TC13000051.hg.1	4.14	JUC13000222.hg.1	C1QTNF9, SI
TC13000051.hg.1	3.76	PSR13000379.hg.1	C1QTNF9, SI
TC13000051.hg.1	3.52	PSR13000378.hg.1	C1QTNF9, SI
TC13000051.hg.1	3.35	PSR13000372.hg.1	C1QTNF9, SI
TC13000051.hg.1	3.31	PSR13000427.hg.1	C1QTNF9, SI
TC13000051.hg.1	3.26	PSR13000392.hg.1	C1QTNF9, SI
TC13000051.hg.1	3.23	PSR13000394.hg.1	C1QTNF9, SI
TC13000051.hg.1	3.2	PSR13000423.hg.1	C1QTNF9, SI
TC13000051.hg.1	3.15	PSR13000371.hg.1	C1QTNF9, SI
TC13000051.hg.1	3.12	PSR13000419.hg.1	C1QTNF9, SI
TC13000051.hg.1	2.94	PSR13000424.hg.1	C1QTNF9, SI
TC13000051.hg.1	2.71	PSR13000376.hg.1	C1QTNF9, SI
TC13000051.hg.1	2.58	PSR13000373.hg.1	C1QTNF9, SI
TC13000051.hg.1	2.58	PSR13000420.hg.1	C1QTNF9, SI
TC13000051.hg.1	2.57	JUC13000216.hg.1	C1QTNF9, SI
TC13000051.hg.1	2.53	JUC13000202.hg.1	C1QTNF9, SI
TC13000051.hg.1	2.48	PSR13000380.hg.1	C1QTNF9, SI
TC13000051.hg.1	2.43	JUC13000205.hg.1	C1QTNF9, SI
TC13000051.hg.1	2.32	PSR13000390.hg.1	C1QTNF9, SI
TC13000051.hg.1	2.27	JUC13000229.hg.1	C1QTNF9, SI
TC13000051.hg.1	2.01	PSR13000425.hg.1	C1QTNF9, SI
TC13000051.hg.1	2	PSR13000375.hg.1	C1QTNF9, SI
TC13000051.hg.1	-2.43	JUC13000214.hg.1	C1QTNF9, SI
TC13000051.hg.1	-2.65	JUC13000221.hg.1	C1QTNF9, SI
TC13000051.hg.1	-4.48	JUC13000227.hg.1	C1QTNF9, SI
TC03000577.hg.1	6.06	JUC03005245.hg.1	BOC
TC03000577.hg.1	4.98	JUC03005224.hg.1	BOC
TC03000577.hg.1	4.03	JUC03005233.hg.1	BOC
TC03000577.hg.1	3.95	JUC03005229.hg.1	BOC
TC03000577.hg.1	3.86	JUC03005235.hg.1	BOC
TC03000577.hg.1	3.76	PSR03010503.hg.1	BOC
TC03000577.hg.1	3.63	PSR03010518.hg.1	BOC
TC03000577.hg.1	3.54	PSR03010506.hg.1	BOC
TC03000577.hg.1	3.42	JUC03005246.hg.1	BOC
TC03000577.hg.1	3.21	JUC03005238.hg.1	BOC
TC03000577.hg.1	3.17	PSR03010530.hg.1	BOC
TC03000577.hg.1	2.82	JUC03005239.hg.1	BOC
TC03000577.hg.1	2.76	PSR03010521.hg.1	BOC
TC03000577.hg.1	2.63	PSR03010487.hg.1	BOC

TC03000577.hg.1	2.58	PSR03010537.hg.1	BOC
TC03000577.hg.1	2.36	PSR03010484.hg.1	BOC
TC03000577.hg.1	2.31	PSR03010488.hg.1	BOC
TC03000577.hg.1	2.31	PSR03010509.hg.1	BOC
TC03000577.hg.1	2.08	PSR03010489.hg.1	BOC
TC03000577.hg.1	2.07	PSR03010508.hg.1	BOC
TC04001425.hg.1	6.04	JUC04010455.hg.1	PPP3CA
TC04001425.hg.1	2.06	PSR04019922.hg.1	PPP3CA
TC04001425.hg.1	-2.09	JUC04010458.hg.1	PPP3CA
TC04001425.hg.1	-2.28	JUC04010448.hg.1	PPP3CA
TC08000149.hg.1	6.04	PSR08001499.hg.1	LPL
TC08000149.hg.1	2.87	JUC08000818.hg.1	LPL
TC08000149.hg.1	2.77	JUC08000813.hg.1	LPL
TC08000149.hg.1	2.67	JUC08000810.hg.1	LPL
TC08000149.hg.1	2.63	PSR08001507.hg.1	LPL
TC08000149.hg.1	2.33	PSR08001496.hg.1	LPL
TC08000149.hg.1	2.29	PSR08001503.hg.1	LPL
TC08000149.hg.1	2.2	PSR08001508.hg.1	LPL
TC08000149.hg.1	2.15	PSR08001513.hg.1	LPL
TC08000149.hg.1	-2.02	JUC08000816.hg.1	LPL
TC08000149.hg.1	-2.08	PSR08001525.hg.1	LPL
TC08000149.hg.1	-2.33	JUC08000814.hg.1	LPL
TC08000149.hg.1	-2.93	PSR08001521.hg.1	LPL
TC08000149.hg.1	-3.06	PSR08001515.hg.1	LPL
TC08000149.hg.1	-3.11	PSR08001522.hg.1	LPL
TC08000149.hg.1	-3.11	PSR08001526.hg.1	LPL
TC08000149.hg.1	-3.15	PSR08001520.hg.1	LPL
TC08000149.hg.1	-3.88	PSR08001527.hg.1	LPL
TC08000149.hg.1	-4.03	JUC08000804.hg.1	LPL
TC08000149.hg.1	-4.98	PSR08001524.hg.1	LPL
TC08000149.hg.1	-7.22	PSR08001528.hg.1	LPL
TC08000149.hg.1	-8.52	JUC08000807.hg.1	LPL
TC08000149.hg.1	-11.02	JUC08000817.hg.1	LPL
TC02001069.hg.1	6.03	PSR02015873.hg.1	RBM45
TC02001069.hg.1	4.04	JUC02008426.hg.1	RBM45
TC02001069.hg.1	2.74	JUC02008432.hg.1	RBM45
TC02001069.hg.1	2.44	JUC02008438.hg.1	RBM45
TC02001069.hg.1	2.35	JUC02008428.hg.1	RBM45
TC02001069.hg.1	2.26	JUC02008435.hg.1	RBM45
TC02001069.hg.1	2.03	JUC02008427.hg.1	RBM45
TC02001069.hg.1	-2.2	JUC02008433.hg.1	RBM45
TC02001069.hg.1	-2.22	PSR02015881.hg.1	RBM45
TC05001166.hg.1	6.03	JUC05008458.hg.1	CTNND2
TC05001166.hg.1	4.67	JUC05008474.hg.1	CTNND2
TC05001166.hg.1	4.35	PSR05016680.hg.1	CTNND2
TC05001166.hg.1	3.95	PSR05016692.hg.1	CTNND2
TC05001166.hg.1	3.16	JUC05008466.hg.1	CTNND2
TC05001166.hg.1	3.1	JUC05008463.hg.1	CTNND2
TC05001166.hg.1	3.02	JUC05008490.hg.1	CTNND2
TC05001166.hg.1	2.73	PSR05016688.hg.1	CTNND2
TC05001166.hg.1	2.61	PSR05016676.hg.1	CTNND2
TC05001166.hg.1	2.6	JUC05008478.hg.1	CTNND2
TC05001166.hg.1	2.53	PSR05016655.hg.1	CTNND2
TC05001166.hg.1	2.44	PSR05016637.hg.1	CTNND2
TC05001166.hg.1	2.43	JUC05008468.hg.1	CTNND2
TC05001166.hg.1	2.4	JUC05008457.hg.1	CTNND2
TC05001166.hg.1	2.34	PSR05016671.hg.1	CTNND2
TC05001166.hg.1	2.33	PSR05016670.hg.1	CTNND2

TC05001166.hg.1	2.29	JUC05008485.hg.1	CTNND2
TC05001166.hg.1	2.27	PSR05016689.hg.1	CTNND2
TC05001166.hg.1	2.27	PSR05016697.hg.1	CTNND2
TC05001166.hg.1	2.22	PSR05016684.hg.1	CTNND2
TC05001166.hg.1	2.21	JUC05008489.hg.1	CTNND2
TC05001166.hg.1	2.15	PSR05016677.hg.1	CTNND2
TC05001166.hg.1	2.09	JUC05008484.hg.1	CTNND2
TC05001166.hg.1	2.06	PSR05016643.hg.1	CTNND2
TC05001166.hg.1	2.05	PSR05016687.hg.1	CTNND2
TC05001166.hg.1	2.04	PSR05016683.hg.1	CTNND2
TC05001166.hg.1	2.03	PSR05016672.hg.1	CTNND2
TC05001166.hg.1	-2.33	JUC05008472.hg.1	CTNND2
TC05001166.hg.1	-3.88	JUC05008480.hg.1	CTNND2
TC05001166.hg.1	-4.68	PSR05016649.hg.1	CTNND2
TC05003407.hg.1	6.03	JUC05019343.hg.1	CDKL3
TC05003407.hg.1	2.46	JUC05019349.hg.1	CDKL3
TC05003407.hg.1	2.44	PSR05024365.hg.1	CDKL3
TC05003407.hg.1	2.44	JUC05019322.hg.1	CDKL3
TC05003407.hg.1	2.26	JUC05019344.hg.1	CDKL3
TC05003407.hg.1	-2.44	JUC05019341.hg.1	CDKL3
TC05003407.hg.1	-2.45	JUC05019336.hg.1	CDKL3
TC04000038.hg.1	6.01	JUC04000373.hg.1	SH3BP2
TC04000038.hg.1	5.04	PSR04000918.hg.1	SH3BP2
TC04000038.hg.1	4.47	PSR04000928.hg.1	SH3BP2
TC04000038.hg.1	4.44	JUC04000384.hg.1	SH3BP2
TC04000038.hg.1	4.12	PSR04000954.hg.1	SH3BP2
TC04000038.hg.1	3.72	JUC04000366.hg.1	SH3BP2
TC04000038.hg.1	3.65	PSR04000924.hg.1	SH3BP2
TC04000038.hg.1	3.63	PSR04000929.hg.1	SH3BP2
TC04000038.hg.1	3.55	PSR04000931.hg.1	SH3BP2
TC04000038.hg.1	3.48	JUC04000383.hg.1	SH3BP2
TC04000038.hg.1	3.47	JUC04000371.hg.1	SH3BP2
TC04000038.hg.1	3.41	PSR04000955.hg.1	SH3BP2
TC04000038.hg.1	3.39	JUC04000362.hg.1	SH3BP2
TC04000038.hg.1	3.36	JUC04000360.hg.1	SH3BP2
TC04000038.hg.1	3.26	PSR04000969.hg.1	SH3BP2
TC04000038.hg.1	3.23	PSR04000908.hg.1	SH3BP2
TC04000038.hg.1	2.97	JUC04000372.hg.1	SH3BP2
TC04000038.hg.1	2.89	PSR04000934.hg.1	SH3BP2
TC04000038.hg.1	2.86	PSR04000921.hg.1	SH3BP2
TC04000038.hg.1	2.86	PSR04000932.hg.1	SH3BP2
TC04000038.hg.1	2.84	PSR04000910.hg.1	SH3BP2
TC04000038.hg.1	2.8	PSR04000914.hg.1	SH3BP2
TC04000038.hg.1	2.74	PSR04000947.hg.1	SH3BP2
TC04000038.hg.1	2.69	JUC04000374.hg.1	SH3BP2
TC04000038.hg.1	2.65	PSR04000973.hg.1	SH3BP2
TC04000038.hg.1	2.56	PSR04000926.hg.1	SH3BP2
TC04000038.hg.1	2.52	PSR04000917.hg.1	SH3BP2
TC04000038.hg.1	2.48	PSR04000919.hg.1	SH3BP2
TC04000038.hg.1	2.44	PSR04000949.hg.1	SH3BP2
TC04000038.hg.1	2.44	JUC04000353.hg.1	SH3BP2
TC04000038.hg.1	2.43	JUC04000379.hg.1	SH3BP2
TC04000038.hg.1	2.42	PSR04000963.hg.1	SH3BP2
TC04000038.hg.1	2.4	PSR04000939.hg.1	SH3BP2
TC04000038.hg.1	2.35	PSR04000956.hg.1	SH3BP2
TC04000038.hg.1	2.3	PSR04000920.hg.1	SH3BP2
TC04000038.hg.1	2.24	PSR04000964.hg.1	SH3BP2
TC04000038.hg.1	2.15	JUC04000376.hg.1	SH3BP2

TC04000038.hg.1	2.09	PSR04000967.hg.1	SH3BP2
TC04000038.hg.1	2.04	PSR04000966.hg.1	SH3BP2
TC04000038.hg.1	2.01	JUC04000357.hg.1	SH3BP2
TC04000038.hg.1	-2.31	JUC04000356.hg.1	SH3BP2
TC10000865.hg.1	6.01	JUC10005880.hg.1	GRK5
TC10000865.hg.1	4.75	PSR10010451.hg.1	GRK5
TC10000865.hg.1	4.03	PSR10010443.hg.1	GRK5
TC10000865.hg.1	3.53	JUC10005867.hg.1	GRK5
TC10000865.hg.1	3.49	PSR10010448.hg.1	GRK5
TC10000865.hg.1	3.08	JUC10005875.hg.1	GRK5
TC10000865.hg.1	3.02	JUC10005869.hg.1	GRK5
TC10000865.hg.1	2.75	PSR10010467.hg.1	GRK5
TC10000865.hg.1	2.66	JUC10005872.hg.1	GRK5
TC10000865.hg.1	2.6	PSR10010473.hg.1	GRK5
TC10000865.hg.1	2.51	JUC10005864.hg.1	GRK5
TC10000865.hg.1	2.34	PSR10010449.hg.1	GRK5
TC10000865.hg.1	2.16	PSR10010463.hg.1	GRK5
TC10000865.hg.1	2.16	JUC10005863.hg.1	GRK5
TC10000865.hg.1	-2.01	PSR10010459.hg.1	GRK5
TC10000865.hg.1	-2.17	PSR10010457.hg.1	GRK5
TC02001362.hg.1	6	JUC02011363.hg.1	MFF
TC02001362.hg.1	-2.15	PSR02021299.hg.1	MFF
TC02001362.hg.1	-2.19	PSR02021271.hg.1	MFF
TC04001057.hg.1	6	PSR04014446.hg.1	FBXL5
TC04001057.hg.1	3.3	JUC04007728.hg.1	FBXL5
TC04001057.hg.1	3	JUC04007711.hg.1	FBXL5
TC04001057.hg.1	2.72	JUC04007714.hg.1	FBXL5
TC04001057.hg.1	2.06	JUC04007729.hg.1	FBXL5
TC04001057.hg.1	2.04	JUC04007724.hg.1	FBXL5
TC08000291.hg.1	6	JUC08002001.hg.1	LETM2
TC08000291.hg.1	3.62	PSR08004051.hg.1	LETM2
TC08000291.hg.1	3.36	PSR08004066.hg.1	LETM2
TC08000291.hg.1	3.32	PSR08004042.hg.1	LETM2
TC08000291.hg.1	3.23	PSR08004033.hg.1	LETM2
TC08000291.hg.1	2.99	PSR08004064.hg.1	LETM2
TC08000291.hg.1	2.94	PSR08004053.hg.1	LETM2
TC08000291.hg.1	2.7	PSR08004039.hg.1	LETM2
TC08000291.hg.1	2.66	PSR08004034.hg.1	LETM2
TC08000291.hg.1	2.66	PSR08004049.hg.1	LETM2
TC08000291.hg.1	2.37	JUC08002002.hg.1	LETM2
TC08000291.hg.1	2.34	PSR08004046.hg.1	LETM2
TC08000291.hg.1	2.27	JUC08001997.hg.1	LETM2
TC08000291.hg.1	2.26	PSR08004052.hg.1	LETM2
TC08000291.hg.1	2.25	PSR08004060.hg.1	LETM2
TC08000291.hg.1	-2.06	PSR08004058.hg.1	LETM2
TC08000291.hg.1	-4.68	JUC08001999.hg.1	LETM2
TC18000040.hg.1	6	JUC18000252.hg.1	SOGA2
TC18000040.hg.1	-2.28	PSR18000378.hg.1	SOGA2
TC18000040.hg.1	-2.37	PSR18000396.hg.1	SOGA2
TC18000040.hg.1	-2.69	PSR18000391.hg.1	SOGA2
TC01001188.hg.1	5.99	PSR01018298.hg.1	ECM1
TC01001188.hg.1	5.64	PSR01018304.hg.1	ECM1
TC01001188.hg.1	5.36	PSR01018303.hg.1	ECM1
TC01001188.hg.1	5.34	PSR01018308.hg.1	ECM1
TC01001188.hg.1	4.47	JUC01010046.hg.1	ECM1
TC01001188.hg.1	4.06	PSR01018291.hg.1	ECM1
TC01001188.hg.1	3.51	JUC01010051.hg.1	ECM1
TC01001188.hg.1	3.33	PSR01018312.hg.1	ECM1

TC01001188.hg.1	2.97	JUC01010050.hg.1	ECM1
TC01001188.hg.1	2.78	PSR01018315.hg.1	ECM1
TC17000885.hg.1	5.99	JUC17006451.hg.1	9-Sep
TC17000885.hg.1	5.22	JUC17006454.hg.1	9-Sep
TC17000885.hg.1	3.5	PSR17011529.hg.1	9-Sep
TC17000885.hg.1	2.87	PSR17011525.hg.1	9-Sep
TC17000885.hg.1	2.73	PSR17011512.hg.1	9-Sep
TC17000885.hg.1	2.71	PSR17011511.hg.1	9-Sep
TC17000885.hg.1	2.49	PSR17011518.hg.1	9-Sep
TC17000885.hg.1	2.36	PSR17011515.hg.1	9-Sep
TC17000885.hg.1	2.31	PSR17011526.hg.1	9-Sep
TC17000885.hg.1	2.3	PSR17011527.hg.1	9-Sep
TC17000885.hg.1	2.27	PSR17011513.hg.1	9-Sep
TC17000885.hg.1	2.26	JUC17006452.hg.1	9-Sep
TC17000885.hg.1	2.22	PSR17011523.hg.1	9-Sep
TC17000885.hg.1	2.09	PSR17011524.hg.1	9-Sep
TC17000885.hg.1	2.02	JUC17006444.hg.1	9-Sep
TC17000885.hg.1	2.01	PSR17011516.hg.1	9-Sep
TC17000885.hg.1	-3.57	JUC17006455.hg.1	9-Sep
TC02002928.hg.1	5.98	JUC02024562.hg.1	NDUFA10
TC02002928.hg.1	2.48	PSR02046941.hg.1	NDUFA10
TC02002928.hg.1	2.26	JUC02024582.hg.1	NDUFA10
TC02002928.hg.1	-2.24	JUC02024578.hg.1	NDUFA10
TC11000184.hg.1	5.98	JUC11001116.hg.1	AMPD3
TC11000184.hg.1	5.52	JUC11001109.hg.1	AMPD3
TC11000184.hg.1	3.27	PSR11002474.hg.1	AMPD3
TC11000184.hg.1	3.18	PSR11002481.hg.1	AMPD3
TC11000184.hg.1	3.14	JUC11001110.hg.1	AMPD3
TC11000184.hg.1	3.05	PSR11002473.hg.1	AMPD3
TC11000184.hg.1	3.04	PSR11002501.hg.1	AMPD3
TC11000184.hg.1	2.81	PSR11002476.hg.1	AMPD3
TC11000184.hg.1	2.74	JUC11001127.hg.1	AMPD3
TC11000184.hg.1	2.73	PSR11002479.hg.1	AMPD3
TC11000184.hg.1	2.65	JUC11001113.hg.1	AMPD3
TC11000184.hg.1	2.52	PSR11002496.hg.1	AMPD3
TC11000184.hg.1	2.31	JUC11001115.hg.1	AMPD3
TC11000184.hg.1	2.21	PSR11002493.hg.1	AMPD3
TC11000184.hg.1	2.2	PSR11002495.hg.1	AMPD3
TC11000184.hg.1	2.14	PSR11002498.hg.1	AMPD3
TC11000184.hg.1	2.04	PSR11002492.hg.1	AMPD3
TC11000184.hg.1	-2.13	PSR11002505.hg.1	AMPD3
TC11000184.hg.1	-2.39	PSR11002491.hg.1	AMPD3
TC11000184.hg.1	-3.16	PSR11002489.hg.1	AMPD3
TC11000184.hg.1	-3.96	JUC11001131.hg.1	AMPD3
TC11000184.hg.1	-5.56	JUC11001126.hg.1	AMPD3
TC11000184.hg.1	-8.04	JUC11001123.hg.1	AMPD3
TC0X000413.hg.1	5.96	PSR0X005524.hg.1	UPRT
TC0X000413.hg.1	-2.04	JUC0X002732.hg.1	UPRT
TC0X000987.hg.1	5.94	JUC0X006596.hg.1	
TC0X000987.hg.1	2.62	PSR0X013117.hg.1	
TC01001996.hg.1	5.93	JUC01016826.hg.1	KIF26B
TC01001996.hg.1	4.25	JUC01016830.hg.1	KIF26B
TC01001996.hg.1	2.95	JUC01016822.hg.1	KIF26B
TC01001996.hg.1	2.76	JUC01016827.hg.1	KIF26B
TC01001996.hg.1	-2.46	PSR01030904.hg.1	KIF26B
TC01001996.hg.1	-2.53	JUC01016816.hg.1	KIF26B
TC01001996.hg.1	-2.54	PSR01030923.hg.1	KIF26B
TC01001996.hg.1	-2.68	JUC01016820.hg.1	KIF26B

TC01001996.hg.1	-2.76	PSR01030907.hg.1	KIF26B
TC01001996.hg.1	-3.15	PSR01030908.hg.1	KIF26B
TC01001996.hg.1	-3.75	PSR01030920.hg.1	KIF26B
TC01001996.hg.1	-3.84	JUC01016821.hg.1	KIF26B
TC01001996.hg.1	-3.9	JUC01016814.hg.1	KIF26B
TC01001996.hg.1	-3.97	PSR01030918.hg.1	KIF26B
TC01001996.hg.1	-4.56	JUC01016818.hg.1	KIF26B
TC05000158.hg.1	5.93	JUC05000976.hg.1	SPEF2
TC05000158.hg.1	5.26	JUC05000992.hg.1	SPEF2
TC05000158.hg.1	4.27	JUC05000950.hg.1	SPEF2
TC05000158.hg.1	4.21	PSR05001774.hg.1	SPEF2
TC05000158.hg.1	3.75	JUC05000995.hg.1	SPEF2
TC05000158.hg.1	3.61	JUC05000990.hg.1	SPEF2
TC05000158.hg.1	3.53	PSR05001754.hg.1	SPEF2
TC05000158.hg.1	3.41	JUC05000964.hg.1	SPEF2
TC05000158.hg.1	3.36	JUC05000979.hg.1	SPEF2
TC05000158.hg.1	3.23	PSR05001769.hg.1	SPEF2
TC05000158.hg.1	3.21	JUC05000969.hg.1	SPEF2
TC05000158.hg.1	3.13	PSR05001767.hg.1	SPEF2
TC05000158.hg.1	3.07	JUC05000963.hg.1	SPEF2
TC05000158.hg.1	3.05	PSR05001763.hg.1	SPEF2
TC05000158.hg.1	2.97	PSR05001770.hg.1	SPEF2
TC05000158.hg.1	2.96	PSR05001777.hg.1	SPEF2
TC05000158.hg.1	2.91	PSR05001778.hg.1	SPEF2
TC05000158.hg.1	2.89	PSR05001748.hg.1	SPEF2
TC05000158.hg.1	2.82	PSR05001762.hg.1	SPEF2
TC05000158.hg.1	2.78	PSR05001772.hg.1	SPEF2
TC05000158.hg.1	2.74	JUC05000949.hg.1	SPEF2
TC05000158.hg.1	2.74	JUC05000965.hg.1	SPEF2
TC05000158.hg.1	2.63	PSR05001764.hg.1	SPEF2
TC05000158.hg.1	2.58	JUC05000994.hg.1	SPEF2
TC05000158.hg.1	2.47	PSR05001776.hg.1	SPEF2
TC05000158.hg.1	2.41	PSR05001714.hg.1	SPEF2
TC05000158.hg.1	2.38	JUC05000975.hg.1	SPEF2
TC05000158.hg.1	2.36	PSR05001765.hg.1	SPEF2
TC05000158.hg.1	2.32	PSR05001773.hg.1	SPEF2
TC05000158.hg.1	2.26	PSR05001757.hg.1	SPEF2
TC05000158.hg.1	2.21	PSR05001775.hg.1	SPEF2
TC05000158.hg.1	2.2	PSR05001755.hg.1	SPEF2
TC05000158.hg.1	2.18	PSR05001752.hg.1	SPEF2
TC05000158.hg.1	2.16	JUC05000947.hg.1	SPEF2
TC05000158.hg.1	2.13	JUC05000982.hg.1	SPEF2
TC05000158.hg.1	2.03	PSR05001760.hg.1	SPEF2
TC05000158.hg.1	2	JUC05000967.hg.1	SPEF2
TC05000158.hg.1	-2.12	PSR05001722.hg.1	SPEF2
TC04001431.hg.1	5.91	JUC04010514.hg.1	UBE2D3
TC04001431.hg.1	2	JUC04010522.hg.1	UBE2D3
TC04001431.hg.1	-3.02	PSR04020054.hg.1	UBE2D3
TC07003376.hg.1	5.9	JUC07021649.hg.1	POLR2J3, UF
TC07003376.hg.1	3.8	JUC07021640.hg.1	POLR2J3, UF
TC07003376.hg.1	-2.19	PSR07026340.hg.1	POLR2J3, UF
TC07003376.hg.1	-2.47	JUC07021655.hg.1	POLR2J3, UF
TC07003376.hg.1	-2.78	PSR07026373.hg.1	POLR2J3, UF
TC02000271.hg.1	5.89	JUC02002101.hg.1	DYNC2LI1
TC02000271.hg.1	2.01	PSR02004043.hg.1	DYNC2LI1
TC02001687.hg.1	5.89	JUC02014267.hg.1	IFT172
TC02001687.hg.1	2.53	JUC02014223.hg.1	IFT172
TC02001687.hg.1	2.17	JUC02014251.hg.1	IFT172

TC02001687.hg.1	2.04	PSR02027297.hg.1	IFT172
TC02001687.hg.1	2.04	JUC02014269.hg.1	IFT172
TC02001687.hg.1	-2.01	PSR02027305.hg.1	IFT172
TC02001687.hg.1	-2.3	JUC02014268.hg.1	IFT172
TC02001687.hg.1	-2.33	JUC02014244.hg.1	IFT172
TC02001687.hg.1	-2.63	JUC02014264.hg.1	IFT172
TC02001687.hg.1	-2.66	JUC02014225.hg.1	IFT172
TC02001687.hg.1	-2.68	JUC02014273.hg.1	IFT172
TC02001687.hg.1	-4.36	JUC02014263.hg.1	IFT172
TC17000819.hg.1	5.89	JUC17005808.hg.1	SOX9
TC17000819.hg.1	-2.24	PSR17010551.hg.1	SOX9
TC19001076.hg.1	5.89	JUC19008307.hg.1	PTPRS
TC19001076.hg.1	2.71	JUC19008316.hg.1	PTPRS
TC19001076.hg.1	2.46	JUC19008306.hg.1	PTPRS
TC19001076.hg.1	2.4	JUC19008289.hg.1	PTPRS
TC19001076.hg.1	2.39	JUC19008303.hg.1	PTPRS
TC19001076.hg.1	2.27	PSR19014711.hg.1	PTPRS
TC19001076.hg.1	2.17	JUC19008297.hg.1	PTPRS
TC19001076.hg.1	2.14	JUC19008310.hg.1	PTPRS
TC19001076.hg.1	2.04	PSR19014724.hg.1	PTPRS
TC19001076.hg.1	-2.3	JUC19008291.hg.1	PTPRS
TC07000784.hg.1	5.88	JUC07005794.hg.1	CCDC136
TC07000784.hg.1	3.46	JUC07005813.hg.1	CCDC136
TC07000784.hg.1	-2.3	PSR07012381.hg.1	CCDC136
TC07000784.hg.1	-6.61	JUC07005791.hg.1	CCDC136
TC11000773.hg.1	5.87	JUC11005159.hg.1	P2RY6
TC11000773.hg.1	2.67	JUC11005160.hg.1	P2RY6
TC11000773.hg.1	2.51	PSR11009934.hg.1	P2RY6
TC11000773.hg.1	2.43	PSR11009935.hg.1	P2RY6
TC11000773.hg.1	2.42	PSR11009949.hg.1	P2RY6
TC11000773.hg.1	2.19	PSR11009938.hg.1	P2RY6
TC11000773.hg.1	2.06	PSR11009944.hg.1	P2RY6
TC11000773.hg.1	-3.23	JUC11005164.hg.1	P2RY6
TC06000726.hg.1	5.86	JUC06003900.hg.1	RIMS1
TC06000726.hg.1	4.3	JUC06003956.hg.1	RIMS1
TC06000726.hg.1	3.71	JUC06003952.hg.1	RIMS1
TC06000726.hg.1	3.5	JUC06003932.hg.1	RIMS1
TC06000726.hg.1	3.13	JUC06003919.hg.1	RIMS1
TC06000726.hg.1	3.05	JUC06003905.hg.1	RIMS1
TC06000726.hg.1	2.93	PSR06008665.hg.1	RIMS1
TC06000726.hg.1	2.92	JUC06003948.hg.1	RIMS1
TC06000726.hg.1	2.8	JUC06003904.hg.1	RIMS1
TC06000726.hg.1	2.65	JUC06003950.hg.1	RIMS1
TC06000726.hg.1	2.46	JUC06003933.hg.1	RIMS1
TC06000726.hg.1	2.31	JUC06003942.hg.1	RIMS1
TC06000726.hg.1	2.3	PSR06008653.hg.1	RIMS1
TC06000726.hg.1	2.25	PSR06008631.hg.1	RIMS1
TC06000726.hg.1	2.15	PSR06008688.hg.1	RIMS1
TC06000726.hg.1	2.12	PSR06008635.hg.1	RIMS1
TC06000726.hg.1	2.07	PSR06008684.hg.1	RIMS1
TC06000726.hg.1	2.05	PSR06008614.hg.1	RIMS1
TC06000726.hg.1	2.01	PSR06008671.hg.1	RIMS1
TC06000726.hg.1	2	JUC06003939.hg.1	RIMS1
TC01000347.hg.1	5.85	JUC01002825.hg.1	RPS6KA1
TC01000347.hg.1	2.24	PSR01005382.hg.1	RPS6KA1
TC01000347.hg.1	2.07	JUC01002824.hg.1	RPS6KA1
TC01000347.hg.1	2.01	PSR01005353.hg.1	RPS6KA1
TC01000347.hg.1	-2.08	PSR01005371.hg.1	RPS6KA1

TC01000347.hg.1	-2.52	PSR01005361.hg.1	RPS6KA1
TC01000347.hg.1	-3	PSR01005384.hg.1	RPS6KA1
TC01000347.hg.1	-3.67	JUC01002794.hg.1	RPS6KA1
TC01000347.hg.1	-3.73	JUC01002809.hg.1	RPS6KA1
TC01000347.hg.1	-3.84	JUC01002816.hg.1	RPS6KA1
TC01000347.hg.1	-3.9	JUC01002800.hg.1	RPS6KA1
TC01000347.hg.1	-5.49	JUC01002813.hg.1	RPS6KA1
TC12000606.hg.1	5.84	JUC12004259.hg.1	MDM2
TC12000606.hg.1	-2.04	PSR12008030.hg.1	MDM2
TC12000606.hg.1	-2.13	JUC12004252.hg.1	MDM2
TC12000606.hg.1	-2.43	PSR12008016.hg.1	MDM2
TC02002074.hg.1	5.83	JUC02017114.hg.1	EIF2AK3
TC02002074.hg.1	2.79	PSR02033226.hg.1	EIF2AK3
TC02002074.hg.1	2.5	PSR02033231.hg.1	EIF2AK3
TC02002074.hg.1	2.32	PSR02033229.hg.1	EIF2AK3
TC02002074.hg.1	2.01	PSR02033233.hg.1	EIF2AK3
TC12001168.hg.1	5.83	PSR12015538.hg.1	SLC2A14
TC12001168.hg.1	5.31	PSR12015537.hg.1	SLC2A14
TC12001168.hg.1	5.3	JUC12008558.hg.1	SLC2A14
TC12001168.hg.1	5.23	JUC12008559.hg.1	SLC2A14
TC12001168.hg.1	5.18	PSR12015551.hg.1	SLC2A14
TC12001168.hg.1	4.47	JUC12008564.hg.1	SLC2A14
TC12001168.hg.1	4.35	PSR12015539.hg.1	SLC2A14
TC12001168.hg.1	4.04	PSR12015552.hg.1	SLC2A14
TC12001168.hg.1	3.91	JUC12008563.hg.1	SLC2A14
TC12001168.hg.1	3.8	JUC12008560.hg.1	SLC2A14
TC12001168.hg.1	3.72	PSR12015540.hg.1	SLC2A14
TC12001168.hg.1	3.61	JUC12008568.hg.1	SLC2A14
TC12001168.hg.1	3.23	PSR12015534.hg.1	SLC2A14
TC12001168.hg.1	3.18	PSR12015529.hg.1	SLC2A14
TC12001168.hg.1	2.46	PSR12015521.hg.1	SLC2A14
TC12001168.hg.1	2.02	JUC12008571.hg.1	SLC2A14
TC04001783.hg.1	5.82	JUC04012944.hg.1	MGC45800
TC04001783.hg.1	3.78	PSR04024409.hg.1	MGC45800
TC04001783.hg.1	3.48	JUC04012945.hg.1	MGC45800
TC04001783.hg.1	2.54	PSR04024410.hg.1	MGC45800
TC04001783.hg.1	-2	PSR04024423.hg.1	MGC45800
TC04001783.hg.1	-2.08	PSR04024414.hg.1	MGC45800
TC04001783.hg.1	-2.19	JUC04012953.hg.1	MGC45800
TC04001783.hg.1	-2.37	PSR04024412.hg.1	MGC45800
TC08000695.hg.1	5.82	JUC08004786.hg.1	MAL2
TC08000695.hg.1	4.06	PSR08009382.hg.1	MAL2
TC08000695.hg.1	3.67	JUC08004793.hg.1	MAL2
TC08000695.hg.1	3.4	PSR08009397.hg.1	MAL2
TC08000695.hg.1	3.09	JUC08004791.hg.1	MAL2
TC08000695.hg.1	3.06	PSR08009390.hg.1	MAL2
TC08000695.hg.1	2.99	PSR08009383.hg.1	MAL2
TC08000695.hg.1	2.56	PSR08009384.hg.1	MAL2
TC08000695.hg.1	2.13	PSR08009380.hg.1	MAL2
TC08000695.hg.1	2.04	PSR08009388.hg.1	MAL2
TC08000695.hg.1	2.04	PSR08009396.hg.1	MAL2
TC08000695.hg.1	-2.14	JUC08004790.hg.1	MAL2
TC10002961.hg.1	5.82	JUC10017501.hg.1	NSUN6
TC10002961.hg.1	-2.67	PSR10013654.hg.1	NSUN6
TC10002961.hg.1	-2.67	JUC10017500.hg.1	NSUN6
TC02001126.hg.1	5.81	JUC02009174.hg.1	MYO1B
TC02001126.hg.1	4.01	JUC02009165.hg.1	MYO1B
TC02001126.hg.1	2.35	PSR02017114.hg.1	MYO1B

TC02001126.hg.1	2.32	PSR02017102.hg.1	MYO1B
TC02001126.hg.1	2.04	PSR02017060.hg.1	MYO1B
TC02001628.hg.1	5.81	JUC02013582.hg.1	APOB
TC02001628.hg.1	5.79	JUC02013572.hg.1	APOB
TC02001628.hg.1	3.75	PSR02026065.hg.1	APOB
TC02001628.hg.1	3.28	JUC02013561.hg.1	APOB
TC02001628.hg.1	3	JUC02013563.hg.1	APOB
TC02001628.hg.1	2.89	JUC02013581.hg.1	APOB
TC02001628.hg.1	2.85	PSR02026060.hg.1	APOB
TC02001628.hg.1	2.79	JUC02013580.hg.1	APOB
TC02001628.hg.1	2.74	JUC02013564.hg.1	APOB
TC02001628.hg.1	2.3	PSR02026068.hg.1	APOB
TC02001628.hg.1	2.22	JUC02013570.hg.1	APOB
TC02001628.hg.1	2.18	PSR02026090.hg.1	APOB
TC02001628.hg.1	2.16	JUC02013568.hg.1	APOB
TC02001628.hg.1	2.15	PSR02026087.hg.1	APOB
TC02001628.hg.1	-2.05	JUC02013573.hg.1	APOB
TC02001628.hg.1	-2.06	JUC02013578.hg.1	APOB
TC02001628.hg.1	-2.13	JUC02013566.hg.1	APOB
TC02001628.hg.1	-2.33	JUC02013585.hg.1	APOB
TC02001628.hg.1	-2.43	PSR02026083.hg.1	APOB
TC02001628.hg.1	-2.46	JUC02013558.hg.1	APOB
TC02001628.hg.1	-2.57	PSR02026079.hg.1	APOB
TC02001628.hg.1	-2.67	PSR02026077.hg.1	APOB
TC02001628.hg.1	-2.79	PSR02026094.hg.1	APOB
TC02001628.hg.1	-3.01	PSR02026063.hg.1	APOB
TC02001628.hg.1	-3.33	JUC02013586.hg.1	APOB
TC02001628.hg.1	-3.53	PSR02026059.hg.1	APOB
TC02001628.hg.1	-3.86	PSR02026092.hg.1	APOB
TC02001628.hg.1	-4.22	PSR02026075.hg.1	APOB
TC15001107.hg.1	5.81	PSR15010083.hg.1	FAM189A1
TC15001107.hg.1	2.9	PSR15010085.hg.1	FAM189A1
TC15001107.hg.1	-2.48	JUC15005335.hg.1	FAM189A1
TC01002832.hg.1	5.8	JUC01023653.hg.1	COL24A1
TC01002832.hg.1	4.84	PSR01044149.hg.1	COL24A1
TC01002832.hg.1	2.76	JUC01023668.hg.1	COL24A1
TC01002832.hg.1	2.16	JUC01023649.hg.1	COL24A1
TC01002832.hg.1	2.1	PSR01044144.hg.1	COL24A1
TC01002832.hg.1	2.1	PSR01044145.hg.1	COL24A1
TC01002832.hg.1	2.01	JUC01023699.hg.1	COL24A1
TC01002832.hg.1	-2.05	PSR01044135.hg.1	COL24A1
TC01002832.hg.1	-2.43	JUC01023691.hg.1	COL24A1
TC01002832.hg.1	-2.44	JUC01023697.hg.1	COL24A1
TC01002832.hg.1	-2.48	PSR01044090.hg.1	COL24A1
TC01002832.hg.1	-4.61	JUC01023713.hg.1	COL24A1
TC0X000298.hg.1	5.79	JUC0X001944.hg.1	MAGED4, MA
TC0X000298.hg.1	3.55	JUC0X001951.hg.1	MAGED4, MA
TC0X000298.hg.1	3.47	PSR0X003995.hg.1	MAGED4, MA
TC0X000298.hg.1	2.87	JUC0X001943.hg.1	MAGED4, MA
TC0X000298.hg.1	2.75	PSR0X003963.hg.1	MAGED4, MA
TC0X000298.hg.1	2.4	JUC0X001941.hg.1	MAGED4, MA
TC0X000298.hg.1	2.26	JUC0X001932.hg.1	MAGED4, MA
TC0X000298.hg.1	2.07	PSR0X003990.hg.1	MAGED4, MA
TC03001500.hg.1	5.78	PSR03027397.hg.1	ACOX2
TC03001500.hg.1	3.67	PSR03027355.hg.1	ACOX2
TC03001500.hg.1	3.3	JUC03013379.hg.1	ACOX2
TC03001500.hg.1	3.06	PSR03027384.hg.1	ACOX2
TC03001500.hg.1	2.52	PSR03027374.hg.1	ACOX2

TC03001500.hg.1	2.46	PSR03027381.hg.1	ACOX2
TC03001500.hg.1	2.06	JUC03013389.hg.1	ACOX2
TC03001500.hg.1	2.03	PSR03027363.hg.1	ACOX2
TC03001500.hg.1	2.02	JUC03013381.hg.1	ACOX2
TC03001500.hg.1	-2.2	JUC03013382.hg.1	ACOX2
TC03001500.hg.1	-2.23	PSR03027378.hg.1	ACOX2
TC03001500.hg.1	-2.25	PSR03027383.hg.1	ACOX2
TC03001500.hg.1	-2.33	PSR03027386.hg.1	ACOX2
TC03001500.hg.1	-2.4	PSR03027354.hg.1	ACOX2
TC03001500.hg.1	-3.36	JUC03013374.hg.1	ACOX2
TC10000289.hg.1	5.78	JUC10001861.hg.1	RASSF4
TC10000289.hg.1	5.17	JUC10001845.hg.1	RASSF4
TC10000289.hg.1	2.66	PSR10003233.hg.1	RASSF4
TC10000289.hg.1	2.51	PSR10003246.hg.1	RASSF4
TC10000289.hg.1	2.51	PSR10003257.hg.1	RASSF4
TC10000289.hg.1	2.24	PSR10003219.hg.1	RASSF4
TC10000289.hg.1	2.15	JUC10001848.hg.1	RASSF4
TC10000289.hg.1	2.08	PSR10003241.hg.1	RASSF4
TC10000289.hg.1	2.04	JUC10001864.hg.1	RASSF4
TC10000289.hg.1	2	PSR10003254.hg.1	RASSF4
TC10000289.hg.1	-2	JUC10001851.hg.1	RASSF4
TC10000289.hg.1	-2.26	JUC10001850.hg.1	RASSF4
TC10000289.hg.1	-2.36	PSR10003253.hg.1	RASSF4
TC10000289.hg.1	-2.43	JUC10001855.hg.1	RASSF4
TC10000289.hg.1	-2.57	PSR10003238.hg.1	RASSF4
TC10000289.hg.1	-2.59	PSR10003249.hg.1	RASSF4
TC10000289.hg.1	-2.8	JUC10001853.hg.1	RASSF4
TC10000289.hg.1	-2.9	PSR10003225.hg.1	RASSF4
TC10000289.hg.1	-2.92	PSR10003255.hg.1	RASSF4
TC10000289.hg.1	-4.06	JUC10001866.hg.1	RASSF4
TC21001058.hg.1	5.78	JUC21005084.hg.1	DONSON
TC21001058.hg.1	3.5	JUC21005089.hg.1	DONSON
TC21001058.hg.1	-2.67	PSR21004740.hg.1	DONSON
TC0Y000353.hg.1	5.77	PSR0Y000796.hg.1	TXLNG2P
TC0Y000353.hg.1	4.45	PSR0Y000795.hg.1	TXLNG2P
TC0Y000353.hg.1	3.57	PSR0Y000791.hg.1	TXLNG2P
TC0Y000353.hg.1	-2.29	PSR0Y000797.hg.1	TXLNG2P
TC0Y000353.hg.1	-2.69	JUC0Y001894.hg.1	TXLNG2P
TC0Y000353.hg.1	-2.78	PSR0Y000788.hg.1	TXLNG2P
TC0Y000353.hg.1	-2.78	PSR0Y000804.hg.1	TXLNG2P
TC0Y000353.hg.1	-3.62	PSR0Y000789.hg.1	TXLNG2P
TC0Y000353.hg.1	-4.69	PSR0Y000790.hg.1	TXLNG2P
TC0Y000353.hg.1	-4.79	JUC0Y001898.hg.1	TXLNG2P
TC0Y000353.hg.1	-5.3	PSR0Y000800.hg.1	TXLNG2P
TC0Y000353.hg.1	-6.21	PSR0Y000799.hg.1	TXLNG2P
TC11000043.hg.1	5.77	JUC11000425.hg.1	BRSK2
TC11000043.hg.1	5.62	JUC11000436.hg.1	BRSK2
TC11000043.hg.1	4.08	JUC11000440.hg.1	BRSK2
TC11000043.hg.1	2.54	JUC11000437.hg.1	BRSK2
TC11000043.hg.1	2	PSR11000878.hg.1	BRSK2
TC11000043.hg.1	-2.1	JUC11000421.hg.1	BRSK2
TC11000043.hg.1	-3.3	JUC11000439.hg.1	BRSK2
TC02001658.hg.1	5.76	JUC02013820.hg.1	DNMT3A
TC02001658.hg.1	2.5	JUC02013807.hg.1	DNMT3A
TC02001658.hg.1	2.09	PSR02026486.hg.1	DNMT3A
TC02001658.hg.1	2.07	JUC02013806.hg.1	DNMT3A
TC02001658.hg.1	-2.01	JUC02013832.hg.1	DNMT3A
TC02001658.hg.1	-2.21	PSR02026457.hg.1	DNMT3A

TC02001963.hg.1	5.74	JUC02016322.hg.1	TGFA
TC02001963.hg.1	4.85	PSR02031288.hg.1	TGFA
TC02001963.hg.1	4.2	JUC02016328.hg.1	TGFA
TC02001963.hg.1	4.17	PSR02031287.hg.1	TGFA
TC02001963.hg.1	4.14	PSR02031284.hg.1	TGFA
TC02001963.hg.1	3.79	PSR02031290.hg.1	TGFA
TC02001963.hg.1	3.6	JUC02016319.hg.1	TGFA
TC02001963.hg.1	3.39	PSR02031278.hg.1	TGFA
TC02001963.hg.1	2.78	PSR02031280.hg.1	TGFA
TC02001963.hg.1	2.4	PSR02031283.hg.1	TGFA
TC02001963.hg.1	2.22	PSR02031285.hg.1	TGFA
TC02001963.hg.1	-2.04	JUC02016323.hg.1	TGFA
TC02001963.hg.1	-2.09	JUC02016320.hg.1	TGFA
TC02001963.hg.1	-2.5	PSR02031273.hg.1	TGFA
TC02001963.hg.1	-2.76	PSR02031270.hg.1	TGFA
TC02001963.hg.1	-3.63	PSR02031281.hg.1	TGFA
TC02001963.hg.1	-3.74	PSR02031271.hg.1	TGFA
TC02001963.hg.1	-3.88	PSR02031272.hg.1	TGFA
TC02001963.hg.1	-4.17	PSR02031274.hg.1	TGFA
TC02001963.hg.1	-5.9	JUC02016326.hg.1	TGFA
TC05000776.hg.1	5.74	JUC05005730.hg.1	RNF14
TC05000776.hg.1	3.52	JUC05005727.hg.1	RNF14
TC05000776.hg.1	3.05	JUC05005716.hg.1	RNF14
TC05000776.hg.1	2.12	PSR05011056.hg.1	RNF14
TC06000866.hg.1	5.73	JUC06004739.hg.1	LACE1
TC06000866.hg.1	2.93	JUC06004748.hg.1	LACE1
TC06000866.hg.1	-2.17	PSR06010111.hg.1	LACE1
TC12001300.hg.1	5.73	JUC12009376.hg.1	ABCC9
TC12001300.hg.1	2.52	PSR12016996.hg.1	ABCC9
TC12001300.hg.1	2.27	JUC12009368.hg.1	ABCC9
TC12001300.hg.1	2.25	JUC12009351.hg.1	ABCC9
TC12001300.hg.1	2.2	JUC12009336.hg.1	ABCC9
TC12001300.hg.1	2.13	PSR12016993.hg.1	ABCC9
TC12001300.hg.1	-2.32	JUC12009340.hg.1	ABCC9
TC12001300.hg.1	-2.86	JUC12009369.hg.1	ABCC9
TC12001300.hg.1	-2.92	JUC12009374.hg.1	ABCC9
TC12001300.hg.1	-2.95	JUC12009343.hg.1	ABCC9
TC12001300.hg.1	-2.96	JUC12009354.hg.1	ABCC9
TC12001300.hg.1	-3.3	JUC12009370.hg.1	ABCC9
TC16001012.hg.1	5.72	JUC16008127.hg.1	SEZ6L2
TC16001012.hg.1	2.86	JUC16008116.hg.1	SEZ6L2
TC16001012.hg.1	2.63	JUC16008129.hg.1	SEZ6L2
TC16001012.hg.1	2.59	JUC16008126.hg.1	SEZ6L2
TC16001012.hg.1	2.45	PSR16014481.hg.1	SEZ6L2
TC16001012.hg.1	2.35	JUC16008125.hg.1	SEZ6L2
TC16001012.hg.1	2.31	PSR16014478.hg.1	SEZ6L2
TC16001012.hg.1	2.31	JUC16008128.hg.1	SEZ6L2
TC16001012.hg.1	2.2	PSR16014480.hg.1	SEZ6L2
TC16001012.hg.1	2.07	JUC16008123.hg.1	SEZ6L2
TC16001012.hg.1	-2.1	PSR16014497.hg.1	SEZ6L2
TC16001012.hg.1	-3.09	JUC16008122.hg.1	SEZ6L2
TC02001031.hg.1	5.71	PSR02015345.hg.1	PDK1
TC02001031.hg.1	5.58	PSR02015344.hg.1	PDK1
TC02001031.hg.1	4.12	PSR02015358.hg.1	PDK1
TC02001031.hg.1	3.86	JUC02008197.hg.1	PDK1
TC02001031.hg.1	3.73	PSR02015353.hg.1	PDK1
TC02001031.hg.1	3.63	PSR02015342.hg.1	PDK1
TC02001031.hg.1	3.49	JUC02008190.hg.1	PDK1

TC02001031.hg.1	3.34	JUC02008194.hg.1	PDK1
TC02001031.hg.1	3.17	PSR02015372.hg.1	PDK1
TC02001031.hg.1	2.34	JUC02008199.hg.1	PDK1
TC02001031.hg.1	2.08	PSR02015347.hg.1	PDK1
TC02001031.hg.1	-2.24	PSR02015369.hg.1	PDK1
TC02001031.hg.1	-3.32	JUC02008185.hg.1	PDK1
TC02001031.hg.1	-3.81	JUC02008189.hg.1	PDK1
TC02001031.hg.1	-4.52	JUC02008180.hg.1	PDK1
TC06002238.hg.1	5.71	JUC06013931.hg.1	SYNE1
TC06002238.hg.1	3.65	PSR06027938.hg.1	SYNE1
TC06002238.hg.1	2.55	JUC06014103.hg.1	SYNE1
TC06002238.hg.1	2.54	JUC06014008.hg.1	SYNE1
TC06002238.hg.1	2.34	JUC06014070.hg.1	SYNE1
TC06002238.hg.1	2.33	PSR06028108.hg.1	SYNE1
TC06002238.hg.1	2.31	PSR06027918.hg.1	SYNE1
TC06002238.hg.1	2.3	PSR06028154.hg.1	SYNE1
TC06002238.hg.1	2.2	PSR06027936.hg.1	SYNE1
TC06002238.hg.1	2.1	PSR06027924.hg.1	SYNE1
TC06002238.hg.1	2.03	PSR06027985.hg.1	SYNE1
TC06002238.hg.1	2	PSR06028044.hg.1	SYNE1
TC06002238.hg.1	-2	JUC06013988.hg.1	SYNE1
TC06002238.hg.1	-2.02	JUC06014090.hg.1	SYNE1
TC06002238.hg.1	-2.09	PSR06028083.hg.1	SYNE1
TC06002238.hg.1	-2.16	PSR06028145.hg.1	SYNE1
TC06002238.hg.1	-2.17	PSR06028137.hg.1	SYNE1
TC06002238.hg.1	-2.23	JUC06013970.hg.1	SYNE1
TC06002238.hg.1	-2.27	JUC06014050.hg.1	SYNE1
TC06002238.hg.1	-2.33	JUC06014021.hg.1	SYNE1
TC06002238.hg.1	-2.39	PSR06028149.hg.1	SYNE1
TC06002238.hg.1	-2.4	JUC06013964.hg.1	SYNE1
TC06002238.hg.1	-2.4	JUC06014056.hg.1	SYNE1
TC06002238.hg.1	-2.46	PSR06028139.hg.1	SYNE1
TC06002238.hg.1	-2.46	PSR06028148.hg.1	SYNE1
TC06002238.hg.1	-2.62	JUC06014032.hg.1	SYNE1
TC06002238.hg.1	-2.65	JUC06014098.hg.1	SYNE1
TC06002238.hg.1	-2.84	JUC06013973.hg.1	SYNE1
TC06002238.hg.1	-2.89	PSR06028142.hg.1	SYNE1
TC06002238.hg.1	-2.9	JUC06013976.hg.1	SYNE1
TC06002238.hg.1	-2.98	PSR06028084.hg.1	SYNE1
TC06002238.hg.1	-3.67	JUC06014033.hg.1	SYNE1
TC06002238.hg.1	-3.83	JUC06014002.hg.1	SYNE1
TC06002238.hg.1	-4.03	JUC06013934.hg.1	SYNE1
TC06002238.hg.1	-4.06	PSR06028140.hg.1	SYNE1
TC06002238.hg.1	-4.69	PSR06028146.hg.1	SYNE1
TC06002238.hg.1	-4.73	JUC06013987.hg.1	SYNE1
TC07000735.hg.1	5.69	JUC07005509.hg.1	CPED1
TC07000735.hg.1	5.11	JUC07005518.hg.1	CPED1
TC07000735.hg.1	3.97	JUC07005500.hg.1	CPED1
TC07000735.hg.1	3.63	PSR07011743.hg.1	CPED1
TC07000735.hg.1	3.34	PSR07011742.hg.1	CPED1
TC07000735.hg.1	3.04	PSR07011764.hg.1	CPED1
TC07000735.hg.1	2.99	PSR07011741.hg.1	CPED1
TC07000735.hg.1	2.96	JUC07005510.hg.1	CPED1
TC07000735.hg.1	2.54	PSR07011735.hg.1	CPED1
TC07000735.hg.1	-2.52	JUC07005508.hg.1	CPED1
TC07000735.hg.1	-3	JUC07005513.hg.1	CPED1
TC02002666.hg.1	5.68	JUC02022400.hg.1	CLK1
TC02002666.hg.1	4.53	JUC02022398.hg.1	CLK1

TC02002666.hg.1	4.03	JUC02022392.hg.1	CLK1
TC02002666.hg.1	2.36	JUC02022390.hg.1	CLK1
TC02002666.hg.1	-2.06	PSR02042811.hg.1	CLK1
TC02002666.hg.1	-2.15	PSR02042813.hg.1	CLK1
TC02002666.hg.1	-2.35	PSR02042814.hg.1	CLK1
TC02002666.hg.1	-2.52	PSR02042810.hg.1	CLK1
TC02002666.hg.1	-3.63	PSR02042807.hg.1	CLK1
TC07001883.hg.1	5.68	JUC07014436.hg.1	AKR1B1
TC07001883.hg.1	3.99	PSR07029203.hg.1	AKR1B1
TC07001883.hg.1	2.55	PSR07029200.hg.1	AKR1B1
TC07001883.hg.1	2.45	PSR07029214.hg.1	AKR1B1
TC07001883.hg.1	2.43	PSR07029199.hg.1	AKR1B1
TC07001883.hg.1	2.39	PSR07029192.hg.1	AKR1B1
TC07001883.hg.1	2.03	PSR07029201.hg.1	AKR1B1
TC0Y000181.hg.1	5.68	PSR0Y001782.hg.1	NCRNA0018[illegible]
TC0Y000181.hg.1	4.99	JUC0Y001135.hg.1	NCRNA0018[illegible]
TC0Y000181.hg.1	4.29	JUC0Y001125.hg.1	NCRNA0018[illegible]
TC0Y000181.hg.1	2.52	PSR0Y001781.hg.1	NCRNA0018[illegible]
TC0Y000181.hg.1	2.09	PSR0Y001775.hg.1	NCRNA0018[illegible]
TC0Y000181.hg.1	2.02	JUC0Y001127.hg.1	NCRNA0018[illegible]
TC10000869.hg.1	5.68	PSR10010519.hg.1	INPP5F
TC10000869.hg.1	5.68	JUC10005913.hg.1	INPP5F
TC10000869.hg.1	4.91	PSR10010518.hg.1	INPP5F
TC10000869.hg.1	3.8	PSR10010521.hg.1	INPP5F
TC10000869.hg.1	3.5	JUC10005912.hg.1	INPP5F
TC10000869.hg.1	3.36	PSR10010526.hg.1	INPP5F
TC10000869.hg.1	3.34	JUC10005916.hg.1	INPP5F
TC10000869.hg.1	2.95	JUC10005903.hg.1	INPP5F
TC10000869.hg.1	2.93	PSR10010520.hg.1	INPP5F
TC10000869.hg.1	2.37	PSR10010523.hg.1	INPP5F
TC10000869.hg.1	2.24	PSR10010524.hg.1	INPP5F
TC10000869.hg.1	2.1	JUC10005905.hg.1	INPP5F
TC10000869.hg.1	-2.25	JUC10005899.hg.1	INPP5F
TC10000869.hg.1	-2.58	JUC10005901.hg.1	INPP5F
TC03001466.hg.1	5.67	JUC03013014.hg.1	TKT
TC03001466.hg.1	4.2	PSR03026747.hg.1	TKT
TC03001466.hg.1	4.08	PSR03026725.hg.1	TKT
TC03001466.hg.1	3.96	PSR03026720.hg.1	TKT
TC03001466.hg.1	3.86	PSR03026722.hg.1	TKT
TC03001466.hg.1	3.43	PSR03026750.hg.1	TKT
TC03001466.hg.1	2.86	PSR03026710.hg.1	TKT
TC03001466.hg.1	2.85	PSR03026737.hg.1	TKT
TC03001466.hg.1	2.84	JUC03013026.hg.1	TKT
TC03001466.hg.1	2.74	PSR03026735.hg.1	TKT
TC03001466.hg.1	2.68	PSR03026757.hg.1	TKT
TC03001466.hg.1	2.63	PSR03026763.hg.1	TKT
TC03001466.hg.1	2.46	JUC03013021.hg.1	TKT
TC03001466.hg.1	2.4	JUC03013027.hg.1	TKT
TC03001466.hg.1	2.29	PSR03026740.hg.1	TKT
TC03001466.hg.1	2.28	JUC03013009.hg.1	TKT
TC03001466.hg.1	2.25	PSR03026736.hg.1	TKT
TC03001466.hg.1	2.17	JUC03013030.hg.1	TKT
TC03001466.hg.1	2.01	JUC03013010.hg.1	TKT
TC0X001048.hg.1	5.67	JUC0X007050.hg.1	MAGED4, M[illegible]
TC0X001048.hg.1	3.47	JUC0X007058.hg.1	MAGED4, M[illegible]
TC0X001048.hg.1	3.22	PSR0X014088.hg.1	MAGED4, M[illegible]
TC0X001048.hg.1	2.88	PSR0X014056.hg.1	MAGED4, M[illegible]
TC0X001048.hg.1	2.81	JUC0X007049.hg.1	MAGED4, M[illegible]

TC0X001048.hg.1	2.42	PSR0X014061.hg.1	MAGED4, MA
TC0X001048.hg.1	2.35	JUC0X007047.hg.1	MAGED4, MA
TC0X001048.hg.1	2.23	JUC0X007057.hg.1	MAGED4, MA
TC0X001048.hg.1	2.2	PSR0X014051.hg.1	MAGED4, MA
TC0X001482.hg.1	5.66	JUC0X009765.hg.1	TMEM185A
TC0X001482.hg.1	2.33	JUC0X009769.hg.1	TMEM185A
TC0X001482.hg.1	2.26	JUC0X009768.hg.1	TMEM185A
TC0X001482.hg.1	-2.32	PSR0X019270.hg.1	TMEM185A
TC01006309.hg.1	5.64	JUC01040506.hg.1	NBPF10, LOC
TC01006309.hg.1	2.26	PSR01017103.hg.1	NBPF10, LOC
TC01006309.hg.1	2.17	JUC01039833.hg.1	NBPF10, LOC
TC10000169.hg.1	5.64	JUC10001084.hg.1	KIAA1217
TC10000169.hg.1	2.51	PSR10002017.hg.1	KIAA1217
TC10000169.hg.1	2.33	JUC10001066.hg.1	KIAA1217
TC10000169.hg.1	2.02	JUC10001080.hg.1	KIAA1217
TC10000169.hg.1	-2.12	PSR10001984.hg.1	KIAA1217
TC10000169.hg.1	-2.17	JUC10001069.hg.1	KIAA1217
TC10000169.hg.1	-2.31	JUC10001067.hg.1	KIAA1217
TC10000169.hg.1	-2.31	JUC10001077.hg.1	KIAA1217
TC10000169.hg.1	-2.42	JUC10001073.hg.1	KIAA1217
TC10000169.hg.1	-2.6	JUC10001072.hg.1	KIAA1217
TC10000169.hg.1	-2.74	JUC10001074.hg.1	KIAA1217
TC10000169.hg.1	-3.01	PSR10002026.hg.1	KIAA1217
TC10000169.hg.1	-3.09	JUC10001088.hg.1	KIAA1217
TC10000169.hg.1	-3.23	JUC10001081.hg.1	KIAA1217
TC10000169.hg.1	-3.29	JUC10001087.hg.1	KIAA1217
TC10000169.hg.1	-3.31	JUC10001082.hg.1	KIAA1217
TC10000169.hg.1	-3.38	PSR10001979.hg.1	KIAA1217
TC10000169.hg.1	-3.38	PSR10001981.hg.1	KIAA1217
TC10000169.hg.1	-3.39	PSR10001982.hg.1	KIAA1217
TC10000169.hg.1	-4.26	PSR10002027.hg.1	KIAA1217
TC10000169.hg.1	-4.28	PSR10001987.hg.1	KIAA1217
TC10000169.hg.1	-5.97	JUC10001089.hg.1	KIAA1217
TC10000169.hg.1	-6.16	PSR10001986.hg.1	KIAA1217
TC01002327.hg.1	5.62	JUC01019262.hg.1	USP48
TC01002327.hg.1	3.26	JUC01019266.hg.1	USP48
TC01002327.hg.1	2.36	PSR01035799.hg.1	USP48
TC01002327.hg.1	2.29	JUC01019265.hg.1	USP48
TC01002327.hg.1	-2.06	PSR01035821.hg.1	USP48
TC01002327.hg.1	-2.22	JUC01019258.hg.1	USP48
TC01002327.hg.1	-2.32	JUC01019278.hg.1	USP48
TC01002327.hg.1	-2.64	JUC01019277.hg.1	USP48
TC04001585.hg.1	5.62	JUC04011637.hg.1	MAML3
TC04001585.hg.1	-2.12	PSR04022103.hg.1	MAML3
TC04001585.hg.1	-2.32	JUC04011648.hg.1	MAML3
TC04001585.hg.1	-2.32	JUC04011649.hg.1	MAML3
TC04001585.hg.1	-3.12	JUC04011645.hg.1	MAML3
TC17001249.hg.1	5.61	JUC17009494.hg.1	SLC47A2
TC17001249.hg.1	4.6	JUC17009499.hg.1	SLC47A2
TC17001249.hg.1	3.54	PSR17016757.hg.1	SLC47A2
TC17001249.hg.1	3.52	PSR17016739.hg.1	SLC47A2
TC17001249.hg.1	3.39	PSR17016746.hg.1	SLC47A2
TC17001249.hg.1	3.31	PSR17016731.hg.1	SLC47A2
TC17001249.hg.1	3.1	PSR17016747.hg.1	SLC47A2
TC17001249.hg.1	3.08	JUC17009512.hg.1	SLC47A2
TC17001249.hg.1	2.94	JUC17009510.hg.1	SLC47A2
TC17001249.hg.1	2.93	JUC17009511.hg.1	SLC47A2
TC17001249.hg.1	2.92	PSR17016732.hg.1	SLC47A2

TC17001249.hg.1	2.88	JUC17009503.hg.1	SLC47A2
TC17001249.hg.1	2.72	PSR17016750.hg.1	SLC47A2
TC17001249.hg.1	2.69	PSR17016730.hg.1	SLC47A2
TC17001249.hg.1	2.57	PSR17016756.hg.1	SLC47A2
TC17001249.hg.1	2.56	JUC17009497.hg.1	SLC47A2
TC17001249.hg.1	2.47	PSR17016740.hg.1	SLC47A2
TC17001249.hg.1	2.45	PSR17016755.hg.1	SLC47A2
TC17001249.hg.1	2.39	PSR17016738.hg.1	SLC47A2
TC17001249.hg.1	2.29	JUC17009496.hg.1	SLC47A2
TC17001249.hg.1	2.21	PSR17016744.hg.1	SLC47A2
TC17001249.hg.1	2.15	JUC17009504.hg.1	SLC47A2
TC17001249.hg.1	2.01	PSR17016722.hg.1	SLC47A2
TC17001249.hg.1	-2.63	JUC17009506.hg.1	SLC47A2
TC17001249.hg.1	-2.99	JUC17009515.hg.1	SLC47A2
TC17001249.hg.1	-3.13	PSR17016741.hg.1	SLC47A2
TC17001249.hg.1	-3.37	PSR17016729.hg.1	SLC47A2
TC17001249.hg.1	-3.43	JUC17009509.hg.1	SLC47A2
TC17001249.hg.1	-6.49	JUC17009502.hg.1	SLC47A2
TC17001249.hg.1	-6.88	PSR17016745.hg.1	SLC47A2
TC17001249.hg.1	-6.95	JUC17009492.hg.1	SLC47A2
TC17001249.hg.1	-14.24	JUC17009501.hg.1	SLC47A2
TC6_mann_hap40(	5.61	JUC6_mann_hap400	HLA-C
TC6_mann_hap40(	2.94	PSR6_mann_hap40(	HLA-C
TC6_mann_hap40(	2.43	JUC6_mann_hap400	HLA-C
TC6_mann_hap40(	2.42	JUC6_mann_hap400	HLA-C
TC6_mann_hap40(	-2.86	JUC6_mann_hap400	HLA-C
TC6_mann_hap40(	-3.05	JUC6_mann_hap400	HLA-C
TC01001264.hg.1	5.6	JUC01010511.hg.1	SLC27A3
TC01001264.hg.1	2.1	PSR01019328.hg.1	SLC27A3
TC06004055.hg.1	5.6	JUC06020692.hg.1	ROS1
TC06004055.hg.1	4.19	JUC06020681.hg.1	ROS1
TC06004055.hg.1	4.19	JUC06020683.hg.1	ROS1
TC06004055.hg.1	3.73	JUC06020665.hg.1	ROS1
TC06004055.hg.1	3.53	JUC06020667.hg.1	ROS1
TC06004055.hg.1	3.21	JUC06020661.hg.1	ROS1
TC06004055.hg.1	3.19	JUC06020666.hg.1	ROS1
TC06004055.hg.1	3.17	PSR06025669.hg.1	ROS1
TC06004055.hg.1	3.16	PSR06025666.hg.1	ROS1
TC06004055.hg.1	2.98	JUC06020671.hg.1	ROS1
TC06004055.hg.1	2.96	JUC06020685.hg.1	ROS1
TC06004055.hg.1	2.94	PSR06025645.hg.1	ROS1
TC06004055.hg.1	2.58	JUC06020684.hg.1	ROS1
TC06004055.hg.1	2.37	PSR06025671.hg.1	ROS1
TC06004055.hg.1	2.33	PSR06025646.hg.1	ROS1
TC06004055.hg.1	2.32	JUC06020698.hg.1	ROS1
TC06004055.hg.1	2.23	JUC06020668.hg.1	ROS1
TC06004055.hg.1	-2.01	JUC06020688.hg.1	ROS1
TC06004055.hg.1	-2.05	JUC06020674.hg.1	ROS1
TC06004055.hg.1	-2.11	PSR06025650.hg.1	ROS1
TC06004055.hg.1	-2.19	PSR06025629.hg.1	ROS1
TC06004055.hg.1	-2.22	JUC06020659.hg.1	ROS1
TC06004055.hg.1	-2.23	JUC06020675.hg.1	ROS1
TC06004055.hg.1	-2.24	PSR06025665.hg.1	ROS1
TC06004055.hg.1	-2.36	PSR06025636.hg.1	ROS1
TC06004055.hg.1	-2.49	JUC06020697.hg.1	ROS1
TC06004055.hg.1	-2.51	JUC06020696.hg.1	ROS1
TC06004055.hg.1	-2.58	PSR06025622.hg.1	ROS1
TC06004055.hg.1	-2.91	JUC06020673.hg.1	ROS1

TC06004055.hg.1	-2.95	PSR06025620.hg.1	ROS1
TC06004055.hg.1	-3.18	PSR06025624.hg.1	ROS1
TC06004055.hg.1	-3.21	JUC06020654.hg.1	ROS1
TC06004055.hg.1	-3.36	JUC06020662.hg.1	ROS1
TC06004055.hg.1	-3.43	PSR06025625.hg.1	ROS1
TC06004055.hg.1	-3.49	JUC06020676.hg.1	ROS1
TC06004055.hg.1	-3.8	PSR06025633.hg.1	ROS1
TC06004055.hg.1	-3.81	JUC06020690.hg.1	ROS1
TC06004055.hg.1	-3.88	PSR06025630.hg.1	ROS1
TC06004055.hg.1	-3.98	JUC06020699.hg.1	ROS1
TC06004055.hg.1	-4.28	PSR06025627.hg.1	ROS1
TC06004055.hg.1	-4.87	PSR06025632.hg.1	ROS1
TC06004055.hg.1	-5	PSR06025635.hg.1	ROS1
TC06004055.hg.1	-5.34	PSR06025621.hg.1	ROS1
TC06004055.hg.1	-5.42	PSR06025623.hg.1	ROS1
TC06004055.hg.1	-6.09	JUC06020656.hg.1	ROS1
TC07000782.hg.1	5.6	PSR07012309.hg.1	CALU
TC07000782.hg.1	-2.13	JUC07005775.hg.1	CALU
TC22000223.hg.1	5.6	JUC22001505.hg.1	RNF185
TC22000223.hg.1	2.76	PSR22004215.hg.1	RNF185
TC22000223.hg.1	2.4	JUC22001501.hg.1	RNF185
TC06000185.hg.1	5.58	JUC06000897.hg.1	BTN2A2
TC06000185.hg.1	-2.38	PSR06001836.hg.1	BTN2A2
TC02002194.hg.1	5.57	PSR02035538.hg.1	MALL
TC02002194.hg.1	4.85	JUC02018342.hg.1	MALL
TC02002194.hg.1	3.32	PSR02035540.hg.1	MALL
TC02002194.hg.1	3.11	PSR02035541.hg.1	MALL
TC02002194.hg.1	3	PSR02035542.hg.1	MALL
TC02002194.hg.1	-2.12	PSR02035539.hg.1	MALL
TC02002194.hg.1	-5.65	JUC02018338.hg.1	MALL
TC05000384.hg.1	5.57	JUC05002928.hg.1	SCAMP1
TC05000384.hg.1	3.05	JUC05002917.hg.1	SCAMP1
TC05000384.hg.1	2.59	PSR05005405.hg.1	SCAMP1
TC05000384.hg.1	2.38	PSR05005377.hg.1	SCAMP1
TC05000384.hg.1	2.27	JUC05002918.hg.1	SCAMP1
TC05000384.hg.1	2.26	PSR05005410.hg.1	SCAMP1
TC05000384.hg.1	2.17	PSR05005404.hg.1	SCAMP1
TC05000384.hg.1	2.11	PSR05005384.hg.1	SCAMP1
TC05000384.hg.1	2.02	PSR05005409.hg.1	SCAMP1
TC6_apd_hap1000	5.56	JUC6_apd_hap1000	HLA-DPA1
TC6_apd_hap1000	4.03	JUC6_apd_hap1000	HLA-DPA1
TC6_apd_hap1000	2.67	PSR6_apd_hap1001	HLA-DPA1
TC6_apd_hap1000	2.49	PSR6_apd_hap1001	HLA-DPA1
TC6_apd_hap1000	2.32	PSR6_apd_hap1001	HLA-DPA1
TC6_apd_hap1000	2.31	PSR6_apd_hap1001	HLA-DPA1
TC6_apd_hap1000	2.25	PSR6_apd_hap1001	HLA-DPA1
TC6_apd_hap1000	2.08	PSR6_apd_hap1001	HLA-DPA1
TC6_apd_hap1000	2.06	PSR6_apd_hap1001	HLA-DPA1
TC6_apd_hap1000	-2.23	PSR6_apd_hap1001	HLA-DPA1
TC6_apd_hap1000	-4.18	JUC6_apd_hap1000	HLA-DPA1
TC6_dbb_hap3000	5.56	JUC6_dbb_hap3001	HLA-DPA1
TC6_dbb_hap3000	4.03	JUC6_dbb_hap3001	HLA-DPA1
TC6_dbb_hap3000	3.09	PSR6_dbb_hap3003	HLA-DPA1
TC6_dbb_hap3000	2.67	PSR6_dbb_hap3003	HLA-DPA1
TC6_dbb_hap3000	2.32	PSR6_dbb_hap3003	HLA-DPA1
TC6_dbb_hap3000	2.31	PSR6_dbb_hap3003	HLA-DPA1
TC6_dbb_hap3000	2.25	PSR6_dbb_hap3003	HLA-DPA1
TC6_dbb_hap3000	2.08	PSR6_dbb_hap3003	HLA-DPA1

TC6_dbb_hap3000	2.06	PSR6_dbb_hap3003	HLA-DPA1
TC6_dbb_hap3000	-2.23	PSR6_dbb_hap3003	HLA-DPA1
TC6_dbb_hap3000	-4.18	JUC6_dbb_hap3001	HLA-DPA1
TC6_mann_hap40	5.56	JUC6_mann_hap400	HLA-DPA1
TC6_mann_hap40	4.03	JUC6_mann_hap400	HLA-DPA1
TC6_mann_hap40	3.33	PSR6_mann_hap400	HLA-DPA1
TC6_mann_hap40	3.09	PSR6_mann_hap400	HLA-DPA1
TC6_mann_hap40	2.67	PSR6_mann_hap400	HLA-DPA1
TC6_mann_hap40	2.32	PSR6_mann_hap400	HLA-DPA1
TC6_mann_hap40	2.31	PSR6_mann_hap400	HLA-DPA1
TC6_mann_hap40	2.08	PSR6_mann_hap400	HLA-DPA1
TC6_mann_hap40	2.06	PSR6_mann_hap400	HLA-DPA1
TC6_mann_hap40	-2.23	PSR6_mann_hap400	HLA-DPA1
TC6_mann_hap40	-4.18	JUC6_mann_hap400	HLA-DPA1
TC6_mcf_hap5000	5.56	JUC6_mcf_hap5001	HLA-DPA1
TC6_mcf_hap5000	4.03	JUC6_mcf_hap5001	HLA-DPA1
TC6_mcf_hap5000	2.67	PSR6_mcf_hap5003	HLA-DPA1
TC6_mcf_hap5000	2.49	PSR6_mcf_hap5003	HLA-DPA1
TC6_mcf_hap5000	2.32	PSR6_mcf_hap5003	HLA-DPA1
TC6_mcf_hap5000	2.31	PSR6_mcf_hap5003	HLA-DPA1
TC6_mcf_hap5000	2.25	PSR6_mcf_hap5003	HLA-DPA1
TC6_mcf_hap5000	2.08	PSR6_mcf_hap5003	HLA-DPA1
TC6_mcf_hap5000	2.06	PSR6_mcf_hap5003	HLA-DPA1
TC6_mcf_hap5000	-2.23	PSR6_mcf_hap5003	HLA-DPA1
TC6_mcf_hap5000	-4.18	JUC6_mcf_hap5001	HLA-DPA1
TC6_mcf_hap5000	5.56	JUC6_mcf_hap5001	HLA-C
TC6_mcf_hap5000	2.41	JUC6_mcf_hap5001	HLA-C
TC6_mcf_hap5000	2.39	JUC6_mcf_hap5001	HLA-C
TC6_mcf_hap5000	2.16	PSR6_mcf_hap5002	HLA-C
TC6_mcf_hap5000	-2.89	JUC6_mcf_hap5001	HLA-C
TC6_qbl_hap6000	5.56	JUC6_qbl_hap60015	HLA-DPA1
TC6_qbl_hap6000	4.03	JUC6_qbl_hap60014	HLA-DPA1
TC6_qbl_hap6000	3.33	PSR6_qbl_hap60038	HLA-DPA1
TC6_qbl_hap6000	3.09	PSR6_qbl_hap60038	HLA-DPA1
TC6_qbl_hap6000	2.67	PSR6_qbl_hap60038	HLA-DPA1
TC6_qbl_hap6000	2.32	PSR6_qbl_hap60038	HLA-DPA1
TC6_qbl_hap6000	2.31	PSR6_qbl_hap60038	HLA-DPA1
TC6_qbl_hap6000	2.08	PSR6_qbl_hap60038	HLA-DPA1
TC6_qbl_hap6000	2.06	PSR6_qbl_hap60038	HLA-DPA1
TC6_qbl_hap6000	-2.23	PSR6_qbl_hap60038	HLA-DPA1
TC6_qbl_hap6000	-4.18	JUC6_qbl_hap60015	HLA-DPA1
TC6_ssto_hap700	5.56	JUC6_ssto_hap7001	HLA-DPA1
TC6_ssto_hap700	4.03	JUC6_ssto_hap7001	HLA-DPA1
TC6_ssto_hap700	3.09	PSR6_ssto_hap7003	HLA-DPA1
TC6_ssto_hap700	2.67	PSR6_ssto_hap7003	HLA-DPA1
TC6_ssto_hap700	2.32	PSR6_ssto_hap7003	HLA-DPA1
TC6_ssto_hap700	2.31	PSR6_ssto_hap7003	HLA-DPA1
TC6_ssto_hap700	2.25	PSR6_ssto_hap7003	HLA-DPA1
TC6_ssto_hap700	2.08	PSR6_ssto_hap7003	HLA-DPA1
TC6_ssto_hap700	2.06	PSR6_ssto_hap7003	HLA-DPA1
TC6_ssto_hap700	-2.23	PSR6_ssto_hap7003	HLA-DPA1
TC6_ssto_hap700	-4.18	JUC6_ssto_hap7001	HLA-DPA1
TC07001047.hg.1	5.55	JUC07007831.hg.1	RNF32
TC07001047.hg.1	2.46	PSR07016158.hg.1	RNF32
TC07001047.hg.1	2.22	PSR07016120.hg.1	RNF32
TC07001047.hg.1	2.04	PSR07016145.hg.1	RNF32
TC0X001291.hg.1	5.55	JUC0X008899.hg.1	LRCH2
TC0X001291.hg.1	2.66	JUC0X008891.hg.1	LRCH2

TC0X001291.hg.1	2.32	PSR0X017483.hg.1	LRCH2
TC0X001291.hg.1	-2.16	JUC0X008896.hg.1	LRCH2
TC0X001291.hg.1	-2.2	JUC0X008902.hg.1	LRCH2
TC0X001291.hg.1	-2.99	JUC0X008887.hg.1	LRCH2
TC07000116.hg.1	5.54	JUC07000847.hg.1	HDAC9
TC07000116.hg.1	5.49	PSR07001771.hg.1	HDAC9
TC07000116.hg.1	2.63	PSR07001793.hg.1	HDAC9
TC07000116.hg.1	2.56	JUC07000846.hg.1	HDAC9
TC07000116.hg.1	2.41	JUC07000801.hg.1	HDAC9
TC07000116.hg.1	2.32	PSR07001767.hg.1	HDAC9
TC07000116.hg.1	2.08	PSR07001777.hg.1	HDAC9
TC07000116.hg.1	2.07	PSR07001785.hg.1	HDAC9
TC07000116.hg.1	-2.08	JUC07000797.hg.1	HDAC9
TC07000116.hg.1	-2.1	PSR07001856.hg.1	HDAC9
TC07000116.hg.1	-2.21	PSR07001847.hg.1	HDAC9
TC07000116.hg.1	-2.31	PSR07001858.hg.1	HDAC9
TC07000116.hg.1	-2.59	PSR07001852.hg.1	HDAC9
TC07000116.hg.1	-2.65	JUC07000802.hg.1	HDAC9
TC07000116.hg.1	-2.72	JUC07000823.hg.1	HDAC9
TC07000116.hg.1	-2.79	PSR07001851.hg.1	HDAC9
TC07000116.hg.1	-2.86	PSR07001859.hg.1	HDAC9
TC07000116.hg.1	-4	JUC07000810.hg.1	HDAC9
TC07000116.hg.1	-4.06	PSR07001821.hg.1	HDAC9
TC07000116.hg.1	-4.16	PSR07001860.hg.1	HDAC9
TC07000116.hg.1	-6.51	JUC07000793.hg.1	HDAC9
TC03001666.hg.1	5.53	JUC03014914.hg.1	KIAA1407
TC03001666.hg.1	3.32	JUC03014926.hg.1	KIAA1407
TC03001666.hg.1	3.03	PSR03030084.hg.1	KIAA1407
TC03001666.hg.1	2.98	PSR03030071.hg.1	KIAA1407
TC03001666.hg.1	2.11	JUC03014908.hg.1	KIAA1407
TC10001576.hg.1	5.52	JUC10011746.hg.1	LOXL4
TC10001576.hg.1	3.66	JUC10011749.hg.1	LOXL4
TC10001576.hg.1	3.16	JUC10011742.hg.1	LOXL4
TC10001576.hg.1	3.13	JUC10011751.hg.1	LOXL4
TC10001576.hg.1	2.78	JUC10011753.hg.1	LOXL4
TC10001576.hg.1	2.64	JUC10011755.hg.1	LOXL4
TC10001576.hg.1	2.58	JUC10011750.hg.1	LOXL4
TC10001576.hg.1	2.54	JUC10011743.hg.1	LOXL4
TC10001576.hg.1	2.49	PSR10020182.hg.1	LOXL4
TC10001576.hg.1	2.37	PSR10020192.hg.1	LOXL4
TC10001576.hg.1	-2.27	JUC10011745.hg.1	LOXL4
TC10001576.hg.1	-2.93	PSR10020196.hg.1	LOXL4
TC11001132.hg.1	5.52	JUC11007750.hg.1	VWA5A
TC11001132.hg.1	3.38	JUC11007747.hg.1	VWA5A
TC11001132.hg.1	3.23	JUC11007759.hg.1	VWA5A
TC11001132.hg.1	3.13	PSR11014315.hg.1	VWA5A
TC11001132.hg.1	2.63	PSR11014338.hg.1	VWA5A
TC11001132.hg.1	2.45	JUC11007753.hg.1	VWA5A
TC11001132.hg.1	2.38	PSR11014342.hg.1	VWA5A
TC11001132.hg.1	2.24	JUC11007757.hg.1	VWA5A
TC11001132.hg.1	2.13	PSR11014339.hg.1	VWA5A
TC11001132.hg.1	-2.31	JUC11007763.hg.1	VWA5A
TC11001132.hg.1	-2.59	JUC11007755.hg.1	VWA5A
TC02001347.hg.1	5.51	JUC02011201.hg.1	SGPP2
TC02001347.hg.1	2.14	JUC02011198.hg.1	SGPP2
TC02001347.hg.1	-2.25	PSR02021024.hg.1	SGPP2
TC0X001186.hg.1	5.51	JUC0X008128.hg.1	RPS6KA6
TC0X001186.hg.1	4.17	JUC0X008123.hg.1	RPS6KA6

TC0X001186.hg.1	4.13	JUC0X008109.hg.1	RPS6KA6
TC0X001186.hg.1	3.34	PSR0X016023.hg.1	RPS6KA6
TC0X001186.hg.1	3.3	PSR0X016025.hg.1	RPS6KA6
TC0X001186.hg.1	3.22	PSR0X016020.hg.1	RPS6KA6
TC0X001186.hg.1	2.87	PSR0X016022.hg.1	RPS6KA6
TC0X001186.hg.1	2.66	JUC0X008124.hg.1	RPS6KA6
TC0X001186.hg.1	2.41	PSR0X016024.hg.1	RPS6KA6
TC0X001186.hg.1	2.4	JUC0X008111.hg.1	RPS6KA6
TC0X001186.hg.1	2.22	PSR0X016015.hg.1	RPS6KA6
TC0X001186.hg.1	-2.26	PSR0X016011.hg.1	RPS6KA6
TC0X001186.hg.1	-3.05	JUC0X008131.hg.1	RPS6KA6
TC0X001186.hg.1	-3.73	PSR0X016006.hg.1	RPS6KA6
TC02000231.hg.1	5.5	JUC02001805.hg.1	VIT
TC02000231.hg.1	2.16	PSR02003442.hg.1	VIT
TC02000231.hg.1	2.09	PSR02003420.hg.1	VIT
TC02000231.hg.1	2.07	PSR02003440.hg.1	VIT
TC02000231.hg.1	-2.18	PSR02003424.hg.1	VIT
TC02000231.hg.1	-2.54	JUC02001798.hg.1	VIT
TC02000231.hg.1	-2.62	PSR02003435.hg.1	VIT
TC02000231.hg.1	-2.77	PSR02003426.hg.1	VIT
TC22000863.hg.1	5.5	JUC22006061.hg.1	EFCAB6
TC22000863.hg.1	3.87	JUC22006072.hg.1	EFCAB6
TC22000863.hg.1	3.44	JUC22006074.hg.1	EFCAB6
TC22000863.hg.1	3.09	PSR22014593.hg.1	EFCAB6
TC22000863.hg.1	2.93	PSR22014594.hg.1	EFCAB6
TC22000863.hg.1	2.77	JUC22006041.hg.1	EFCAB6
TC22000863.hg.1	2.71	PSR22014617.hg.1	EFCAB6
TC22000863.hg.1	2.67	JUC22006044.hg.1	EFCAB6
TC22000863.hg.1	2.42	JUC22006085.hg.1	EFCAB6
TC22000863.hg.1	2.2	PSR22014599.hg.1	EFCAB6
TC22000863.hg.1	2.11	PSR22014622.hg.1	EFCAB6
TC22000863.hg.1	2.1	PSR22014597.hg.1	EFCAB6
TC10000738.hg.1	5.49	JUC10004847.hg.1	BTRC
TC10000738.hg.1	2.98	PSR10008705.hg.1	BTRC
TC10000738.hg.1	2.48	PSR10008697.hg.1	BTRC
TC10000738.hg.1	2.28	JUC10004835.hg.1	BTRC
TC10000738.hg.1	-2.96	JUC10004838.hg.1	BTRC
TC01000702.hg.1	5.48	JUC01005776.hg.1	NFIA
TC01000702.hg.1	2.73	JUC01005781.hg.1	NFIA
TC01000702.hg.1	2.43	JUC01005779.hg.1	NFIA
TC01000702.hg.1	2.36	JUC01005764.hg.1	NFIA
TC01000702.hg.1	-2.62	PSR01011201.hg.1	NFIA
TC01000702.hg.1	-2.64	PSR01011193.hg.1	NFIA
TC06001579.hg.1	5.47	JUC06008998.hg.1	COL11A2
TC06001579.hg.1	2.7	JUC06009011.hg.1	COL11A2
TC06001579.hg.1	2.54	JUC06008991.hg.1	COL11A2
TC06001579.hg.1	2.49	JUC06009017.hg.1	COL11A2
TC06001579.hg.1	2.22	JUC06009039.hg.1	COL11A2
TC06001579.hg.1	2.1	JUC06009031.hg.1	COL11A2
TC06001579.hg.1	-2.14	PSR06018959.hg.1	COL11A2
TC06001579.hg.1	-2.18	JUC06009022.hg.1	COL11A2
TC06001579.hg.1	-2.21	PSR06018978.hg.1	COL11A2
TC06001579.hg.1	-2.46	JUC06008992.hg.1	COL11A2
TC06001579.hg.1	-2.77	JUC06009063.hg.1	COL11A2
TC06001579.hg.1	-2.85	JUC06009047.hg.1	COL11A2
TC06001579.hg.1	-5.6	JUC06009032.hg.1	COL11A2
TC18000548.hg.1	5.47	PSR18005703.hg.1	CCBE1
TC18000548.hg.1	5.07	PSR18005702.hg.1	CCBE1

TC18000548.hg.1	3.9	PSR18005696.hg.1	CCBE1
TC18000548.hg.1	3.38	JUC18003841.hg.1	CCBE1
TC18000548.hg.1	2.91	PSR18005693.hg.1	CCBE1
TC18000548.hg.1	2.13	PSR18005689.hg.1	CCBE1
TC18000548.hg.1	-2.41	PSR18005699.hg.1	CCBE1
TC18000548.hg.1	-2.93	JUC18003840.hg.1	CCBE1
TC08000383.hg.1	5.46	JUC08002692.hg.1	LYN
TC08000383.hg.1	2.9	PSR08005437.hg.1	LYN
TC08000383.hg.1	2.75	PSR08005436.hg.1	LYN
TC08000383.hg.1	2.64	JUC08002695.hg.1	LYN
TC08000383.hg.1	2.55	PSR08005438.hg.1	LYN
TC08000383.hg.1	2.27	PSR08005439.hg.1	LYN
TC08000383.hg.1	2.14	PSR08005454.hg.1	LYN
TC08000383.hg.1	-2.76	JUC08002705.hg.1	LYN
TC0X000606.hg.1	5.46	JUC0X003998.hg.1	STAG2
TC0X000606.hg.1	2.75	PSR0X007952.hg.1	STAG2
TC0X000606.hg.1	2.67	JUC0X003994.hg.1	STAG2
TC0X000606.hg.1	2.05	PSR0X007974.hg.1	STAG2
TC0X000606.hg.1	-2	JUC0X004020.hg.1	STAG2
TC0X000606.hg.1	-2.22	JUC0X003996.hg.1	STAG2
TC0X000606.hg.1	-2.23	JUC0X003995.hg.1	STAG2
TC10001340.hg.1	5.46	JUC10009865.hg.1	DNAJC12
TC10001340.hg.1	4	JUC10009872.hg.1	DNAJC12
TC10001340.hg.1	3.69	JUC10009869.hg.1	DNAJC12
TC10001340.hg.1	2.03	PSR10016887.hg.1	DNAJC12
TC10001340.hg.1	-2.09	JUC10009871.hg.1	DNAJC12
TC10001340.hg.1	-2.66	PSR10016885.hg.1	DNAJC12
TC10001340.hg.1	-3.5	PSR10016902.hg.1	DNAJC12
TC10001340.hg.1	-3.85	PSR10016897.hg.1	DNAJC12
TC10001340.hg.1	-3.97	PSR10016891.hg.1	DNAJC12
TC21000249.hg.1	5.45	JUC21001614.hg.1	COL6A1
TC21000249.hg.1	2.43	PSR21003049.hg.1	COL6A1
TC21000249.hg.1	2.3	PSR21003011.hg.1	COL6A1
TC21000249.hg.1	2.18	PSR21003041.hg.1	COL6A1
TC21000249.hg.1	2.16	PSR21003013.hg.1	COL6A1
TC21000476.hg.1	5.44	JUC21002950.hg.1	RIPK4
TC21000476.hg.1	3.47	PSR21005724.hg.1	RIPK4
TC21000476.hg.1	2.33	PSR21005721.hg.1	RIPK4
TC21000476.hg.1	2.13	JUC21002943.hg.1	RIPK4
TC21000476.hg.1	-2.13	JUC21002948.hg.1	RIPK4
TC21000476.hg.1	-2.42	PSR21005730.hg.1	RIPK4
TC21000476.hg.1	-3.08	PSR21005729.hg.1	RIPK4
TC21000476.hg.1	-3.25	JUC21002952.hg.1	RIPK4
TC21000476.hg.1	-6.2	PSR21005723.hg.1	RIPK4
TC21000476.hg.1	-12.8	JUC21002955.hg.1	RIPK4
TC0X000362.hg.1	5.42	JUC0X002276.hg.1	EFNB1
TC0X000362.hg.1	5	PSR0X004705.hg.1	EFNB1
TC0X000362.hg.1	3.16	JUC0X002274.hg.1	EFNB1
TC0X000362.hg.1	3.11	JUC0X002279.hg.1	EFNB1
TC0X000362.hg.1	3.1	PSR0X004708.hg.1	EFNB1
TC0X000362.hg.1	-3.25	JUC0X002273.hg.1	EFNB1
TC0X000362.hg.1	-6.3	JUC0X002275.hg.1	EFNB1
TC16001088.hg.1	5.41	PSR16015289.hg.1	NETO2
TC16001088.hg.1	5.15	PSR16015288.hg.1	NETO2
TC16001088.hg.1	2.54	JUC16008539.hg.1	NETO2
TC16001088.hg.1	2.08	JUC16008532.hg.1	NETO2
TC19001584.hg.1	5.41	JUC19012371.hg.1	PSG9
TC19001584.hg.1	3.09	PSR19021695.hg.1	PSG9

TC19001584.hg.1	3.04	JUC19012370.hg.1	PSG9
TC19001584.hg.1	2.94	JUC19012369.hg.1	PSG9
TC19001584.hg.1	2.79	JUC19012372.hg.1	PSG9
TC19001584.hg.1	2.52	PSR19021693.hg.1	PSG9
TC19001584.hg.1	2.14	PSR19021706.hg.1	PSG9
TC19001584.hg.1	2.05	PSR19021699.hg.1	PSG9
TC19001584.hg.1	-2.72	PSR19021698.hg.1	PSG9
TC19001584.hg.1	-3.19	PSR19021701.hg.1	PSG9
TC19001584.hg.1	-4.73	JUC19012376.hg.1	PSG9
TC19001584.hg.1	-5.41	JUC19012377.hg.1	PSG9
TC6_mann_hap40(	5.41	PSR6_mann_hap40(	MICA
TC6_mann_hap40(	3.21	PSR6_mann_hap40(	MICA
TC6_mann_hap40(	3.01	JUC6_mann_hap400	MICA
TC6_mann_hap40(	2.82	PSR6_mann_hap40(	MICA
TC6_mann_hap40(	2.74	JUC6_mann_hap400	MICA
TC6_mann_hap40(	2.47	JUC6_mann_hap400	MICA
TC6_mann_hap40(	2.46	JUC6_mann_hap400	MICA
TC6_mann_hap40(	2.44	PSR6_mann_hap40(	MICA
TC6_mann_hap40(	2.11	JUC6_mann_hap400	MICA
TC6_mann_hap40(	2.1	JUC6_mann_hap400	MICA
TC11002275.hg.1	5.4	JUC11014533.hg.1	ARHGAP20
TC11002275.hg.1	2.82	PSR11027288.hg.1	ARHGAP20
TC11002275.hg.1	2.19	PSR11027291.hg.1	ARHGAP20
TC11002275.hg.1	2.08	PSR11027289.hg.1	ARHGAP20
TC01000794.hg.1	5.39	JUC01006636.hg.1	IFI44L
TC01000794.hg.1	3.11	JUC01006645.hg.1	IFI44L
TC01000794.hg.1	-2.03	PSR01012681.hg.1	IFI44L
TC06004058.hg.1	5.39	PSR06028998.hg.1	RNASET2
TC06004058.hg.1	4.18	JUC06020771.hg.1	RNASET2
TC06004058.hg.1	3.27	PSR06029026.hg.1	RNASET2
TC06004058.hg.1	3.19	PSR06029015.hg.1	RNASET2
TC06004058.hg.1	3.01	PSR06029016.hg.1	RNASET2
TC06004058.hg.1	2.75	PSR06029029.hg.1	RNASET2
TC06004058.hg.1	2.74	JUC06020749.hg.1	RNASET2
TC06004058.hg.1	2.67	PSR06029006.hg.1	RNASET2
TC06004058.hg.1	2.66	PSR06028982.hg.1	RNASET2
TC06004058.hg.1	2.54	PSR06029027.hg.1	RNASET2
TC06004058.hg.1	2.44	PSR06029018.hg.1	RNASET2
TC06004058.hg.1	2.42	JUC06020773.hg.1	RNASET2
TC06004058.hg.1	2.36	PSR06028977.hg.1	RNASET2
TC06004058.hg.1	2.35	PSR06028983.hg.1	RNASET2
TC06004058.hg.1	2.35	JUC06020776.hg.1	RNASET2
TC06004058.hg.1	2.26	PSR06029004.hg.1	RNASET2
TC06004058.hg.1	2.21	PSR06028985.hg.1	RNASET2
TC06004058.hg.1	2.19	JUC06020750.hg.1	RNASET2
TC06004058.hg.1	2.19	JUC06020770.hg.1	RNASET2
TC06004058.hg.1	2.12	JUC06020761.hg.1	RNASET2
TC06004058.hg.1	2.03	JUC06020751.hg.1	RNASET2
TC01002815.hg.1	5.38	JUC01023457.hg.1	TTLL7
TC01002815.hg.1	2.84	PSR01043773.hg.1	TTLL7
TC01002815.hg.1	2.61	JUC01023465.hg.1	TTLL7
TC01002815.hg.1	2.53	PSR01043780.hg.1	TTLL7
TC01002815.hg.1	2.46	PSR01043821.hg.1	TTLL7
TC01002815.hg.1	2.39	PSR01043786.hg.1	TTLL7
TC01002815.hg.1	2.39	PSR01043795.hg.1	TTLL7
TC01002815.hg.1	2.25	PSR01043789.hg.1	TTLL7
TC01002815.hg.1	2.12	PSR01043820.hg.1	TTLL7
TC01002815.hg.1	2.11	PSR01043802.hg.1	TTLL7

TC01002815.hg.1	2.03	PSR01043797.hg.1	TTLL7
TC01002815.hg.1	2.01	PSR01043809.hg.1	TTLL7
TC01002815.hg.1	-2.12	JUC01023455.hg.1	TTLL7
TC01002815.hg.1	-2.18	JUC01023464.hg.1	TTLL7
TC02000723.hg.1	5.38	JUC02005935.hg.1	LOC654433
TC02000723.hg.1	-2.01	PSR02011238.hg.1	LOC654433
TC02000723.hg.1	-2.02	JUC02005937.hg.1	LOC654433
TC02000723.hg.1	-2.06	PSR02011250.hg.1	LOC654433
TC02000723.hg.1	-2.28	JUC02005946.hg.1	LOC654433
TC02000723.hg.1	-2.37	PSR02011239.hg.1	LOC654433
TC02000723.hg.1	-2.52	PSR02011221.hg.1	LOC654433
TC02000723.hg.1	-2.72	PSR02011235.hg.1	LOC654433
TC02000723.hg.1	-2.98	JUC02005943.hg.1	LOC654433
TC02000723.hg.1	-3.24	JUC02005934.hg.1	LOC654433
TC02000723.hg.1	-3.88	JUC02005944.hg.1	LOC654433
TC06000544.hg.1	5.38	PSR06005959.hg.1	RNF8
TC06000544.hg.1	2.3	JUC06002482.hg.1	RNF8
TC06000544.hg.1	2.25	JUC06002478.hg.1	RNF8
TC19000796.hg.1	5.36	JUC19006555.hg.1	ZNF610
TC19000796.hg.1	-2.27	PSR19011194.hg.1	ZNF610
TC06000999.hg.1	5.35	PSR06011772.hg.1	EYA4
TC06000999.hg.1	5.29	JUC06005697.hg.1	EYA4
TC06000999.hg.1	4.95	JUC06005713.hg.1	EYA4
TC06000999.hg.1	3.91	JUC06005716.hg.1	EYA4
TC06000999.hg.1	3.33	PSR06011773.hg.1	EYA4
TC06000999.hg.1	3.25	JUC06005703.hg.1	EYA4
TC06000999.hg.1	2.46	PSR06011803.hg.1	EYA4
TC06000999.hg.1	2.3	JUC06005706.hg.1	EYA4
TC06000999.hg.1	2.23	PSR06011769.hg.1	EYA4
TC06000999.hg.1	2.15	PSR06011806.hg.1	EYA4
TC06000999.hg.1	2.02	JUC06005698.hg.1	EYA4
TC06000999.hg.1	-2.22	PSR06011779.hg.1	EYA4
TC06000999.hg.1	-2.46	PSR06011788.hg.1	EYA4
TC06000999.hg.1	-2.5	PSR06011774.hg.1	EYA4
TC06000999.hg.1	-2.69	JUC06005714.hg.1	EYA4
TC06000999.hg.1	-2.75	PSR06011787.hg.1	EYA4
TC06000999.hg.1	-3.09	JUC06005721.hg.1	EYA4
TC06000999.hg.1	-3.39	PSR06011794.hg.1	EYA4
TC06000999.hg.1	-3.71	PSR06011771.hg.1	EYA4
TC06000999.hg.1	-3.84	PSR06011799.hg.1	EYA4
TC06000999.hg.1	-3.92	PSR06011763.hg.1	EYA4
TC06000999.hg.1	-4.07	PSR06011798.hg.1	EYA4
TC06000999.hg.1	-4.27	PSR06011792.hg.1	EYA4
TC06000999.hg.1	-4.31	JUC06005694.hg.1	EYA4
TC06000999.hg.1	-4.33	PSR06011795.hg.1	EYA4
TC06000999.hg.1	-4.52	PSR06011764.hg.1	EYA4
TC06000999.hg.1	-5	PSR06011793.hg.1	EYA4
TC06000999.hg.1	-6.62	PSR06011768.hg.1	EYA4
TC06000999.hg.1	-6.93	JUC06005708.hg.1	EYA4
TC06000999.hg.1	-7.6	JUC06005723.hg.1	EYA4
TC6_mann_hap40(	5.35	JUC6_mann_hap400	COL11A2
TC6_mann_hap40(	2.64	JUC6_mann_hap400	COL11A2
TC6_mann_hap40(	2.49	JUC6_mann_hap400	COL11A2
TC6_mann_hap40(	2.43	JUC6_mann_hap400	COL11A2
TC6_mann_hap40(	2.17	JUC6_mann_hap400	COL11A2
TC6_mann_hap40(	2.06	JUC6_mann_hap400	COL11A2
TC6_mann_hap40(	-2.02	JUC6_mann_hap400	COL11A2
TC6_mann_hap40(	-2.09	PSR6_mann_hap40(	COL11A2

TC6_mann_hap40(	-2.19	PSR6_mann_hap40(	COL11A2
TC6_mann_hap40(	-2.23	JUC6_mann_hap400	COL11A2
TC6_mann_hap40(	-2.26	PSR6_mann_hap40(	COL11A2
TC6_mann_hap40(	-2.52	JUC6_mann_hap400	COL11A2
TC6_mann_hap40(	-2.83	JUC6_mann_hap400	COL11A2
TC6_mann_hap40(	-2.91	JUC6_mann_hap400	COL11A2
TC6_mann_hap40(	-5.73	JUC6_mann_hap400	COL11A2
TC02000952.hg.1	5.34	JUC02007459.hg.1	GPD2
TC02000952.hg.1	3.55	JUC02007473.hg.1	GPD2
TC02000952.hg.1	3.06	PSR02013883.hg.1	GPD2
TC02000952.hg.1	2.67	PSR02013922.hg.1	GPD2
TC02000952.hg.1	2.52	PSR02013878.hg.1	GPD2
TC02000952.hg.1	2.3	PSR02013893.hg.1	GPD2
TC02000952.hg.1	2.18	PSR02013885.hg.1	GPD2
TC02000952.hg.1	-2.59	JUC02007451.hg.1	GPD2
TC02000952.hg.1	-2.74	JUC02007462.hg.1	GPD2
TC02000952.hg.1	-3.75	JUC02007455.hg.1	GPD2
TC07001617.hg.1	5.34	PSR07024581.hg.1	PON2
TC07001617.hg.1	4.89	JUC07012234.hg.1	PON2
TC07001617.hg.1	2.49	PSR07024589.hg.1	PON2
TC07001617.hg.1	2.22	JUC07012228.hg.1	PON2
TC07001617.hg.1	2.02	PSR07024573.hg.1	PON2
TC07001617.hg.1	-2.16	PSR07024583.hg.1	PON2
TC07001617.hg.1	-2.17	JUC07012236.hg.1	PON2
TC6_ssto_hap700(	5.34	JUC6_ssto_hap7001	COL11A2
TC6_ssto_hap700(	2.63	JUC6_ssto_hap7001	COL11A2
TC6_ssto_hap700(	2.48	JUC6_ssto_hap7001	COL11A2
TC6_ssto_hap700(	2.42	JUC6_ssto_hap7001	COL11A2
TC6_ssto_hap700(	2.17	JUC6_ssto_hap7001	COL11A2
TC6_ssto_hap700(	2.05	JUC6_ssto_hap7001	COL11A2
TC6_ssto_hap700(	-2.02	JUC6_ssto_hap7001	COL11A2
TC6_ssto_hap700(	-2.19	PSR6_ssto_hap7003	COL11A2
TC6_ssto_hap700(	-2.23	JUC6_ssto_hap7001	COL11A2
TC6_ssto_hap700(	-2.26	PSR6_ssto_hap7003	COL11A2
TC6_ssto_hap700(	-2.52	JUC6_ssto_hap7001	COL11A2
TC6_ssto_hap700(	-2.84	JUC6_ssto_hap7001	COL11A2
TC6_ssto_hap700(	-2.92	JUC6_ssto_hap7001	COL11A2
TC6_ssto_hap700(	-5.74	JUC6_ssto_hap7001	COL11A2
TC07000525.hg.1	5.32	PSR07007506.hg.1	ADAM22
TC07000525.hg.1	5.24	JUC07003618.hg.1	ADAM22
TC07000525.hg.1	4.96	JUC07003612.hg.1	ADAM22
TC07000525.hg.1	4.65	PSR07007501.hg.1	ADAM22
TC07000525.hg.1	3.23	JUC07003631.hg.1	ADAM22
TC07000525.hg.1	3.15	PSR07007551.hg.1	ADAM22
TC07000525.hg.1	2.98	PSR07007505.hg.1	ADAM22
TC07000525.hg.1	2.47	JUC07003607.hg.1	ADAM22
TC07000525.hg.1	2.11	PSR07007538.hg.1	ADAM22
TC07000525.hg.1	2.09	JUC07003594.hg.1	ADAM22
TC07000525.hg.1	2.06	JUC07003611.hg.1	ADAM22
TC07000525.hg.1	2.05	JUC07003627.hg.1	ADAM22
TC07000525.hg.1	2.01	PSR07007502.hg.1	ADAM22
TC07000525.hg.1	2	PSR07007548.hg.1	ADAM22
TC07000525.hg.1	-2.12	JUC07003630.hg.1	ADAM22
TC07000525.hg.1	-2.32	JUC07003623.hg.1	ADAM22
TC07000525.hg.1	-2.35	JUC07003621.hg.1	ADAM22
TC07000525.hg.1	-2.38	PSR07007529.hg.1	ADAM22
TC07000525.hg.1	-2.7	PSR07007542.hg.1	ADAM22
TC07000525.hg.1	-3.05	PSR07007519.hg.1	ADAM22

TC07000525.hg.1	-3.06	JUC07003605.hg.1	ADAM22
TC07000525.hg.1	-3.16	JUC07003593.hg.1	ADAM22
TC07000525.hg.1	-3.3	PSR07007518.hg.1	ADAM22
TC07000525.hg.1	-3.41	JUC07003616.hg.1	ADAM22
TC07000525.hg.1	-3.85	PSR07007522.hg.1	ADAM22
TC07000525.hg.1	-3.98	JUC07003628.hg.1	ADAM22
TC07000525.hg.1	-4	PSR07007513.hg.1	ADAM22
TC07000525.hg.1	-4.12	JUC07003615.hg.1	ADAM22
TC07000525.hg.1	-4.38	PSR07007499.hg.1	ADAM22
TC07000525.hg.1	-5.11	JUC07003619.hg.1	ADAM22
TC07000525.hg.1	-5.53	JUC07003590.hg.1	ADAM22
TC07000525.hg.1	-5.55	JUC07003614.hg.1	ADAM22
TC07000525.hg.1	-5.93	PSR07007536.hg.1	ADAM22
TC07000525.hg.1	-6.87	JUC07003591.hg.1	ADAM22
TC07000525.hg.1	-6.93	JUC07003602.hg.1	ADAM22
TC07000525.hg.1	-7.09	PSR07007544.hg.1	ADAM22
TC07000525.hg.1	-7.13	PSR07007545.hg.1	ADAM22
TC07000525.hg.1	-7.25	PSR07007496.hg.1	ADAM22
TC07000525.hg.1	-7.85	PSR07007504.hg.1	ADAM22
TC07000525.hg.1	-7.93	PSR07007495.hg.1	ADAM22
TC07000525.hg.1	-8.6	PSR07007497.hg.1	ADAM22
TC07000525.hg.1	-9.2	PSR07007535.hg.1	ADAM22
TC07000525.hg.1	-11.27	JUC07003617.hg.1	ADAM22
TC07000525.hg.1	-11.34	JUC07003596.hg.1	ADAM22
TC07000525.hg.1	-11.57	PSR07007490.hg.1	ADAM22
TC07000525.hg.1	-11.96	PSR07007493.hg.1	ADAM22
TC07000525.hg.1	-12.86	PSR07007503.hg.1	ADAM22
TC07000525.hg.1	-13.57	JUC07003625.hg.1	ADAM22
TC07000525.hg.1	-14.87	JUC07003599.hg.1	ADAM22
TC22000195.hg.1	5.32	JUC22001254.hg.1	EWSR1
TC22000195.hg.1	-2.23	PSR22003585.hg.1	EWSR1
TC10000986.hg.1	5.31	PSR10012373.hg.1	AKR1C2, LO(
TC10000986.hg.1	3.45	PSR10012376.hg.1	AKR1C2, LO(
TC10000986.hg.1	3.18	PSR10012355.hg.1	AKR1C2, LO(
TC10000986.hg.1	3.04	PSR10012356.hg.1	AKR1C2, LO(
TC10000986.hg.1	2.65	JUC10007165.hg.1	AKR1C2, LO(
TC10000986.hg.1	2.47	PSR10012375.hg.1	AKR1C2, LO(
TC05000943.hg.1	5.29	PSR05013796.hg.1	FGF18
TC05000943.hg.1	4.88	PSR05013794.hg.1	FGF18
TC05000943.hg.1	2.9	PSR05013795.hg.1	FGF18
TC05000943.hg.1	-2.07	JUC05007157.hg.1	FGF18
TC05000943.hg.1	-2.55	PSR05013790.hg.1	FGF18
TC05000943.hg.1	-2.55	PSR05013791.hg.1	FGF18
TC12000452.hg.1	5.29	JUC12003184.hg.1	HOXC8
TC12000452.hg.1	3.66	PSR12005914.hg.1	HOXC8
TC12000452.hg.1	2.84	PSR12005921.hg.1	HOXC8
TC6_cox_hap2000	5.29	JUC6_cox_hap2001	COL11A2
TC6_cox_hap2000	2.61	JUC6_cox_hap2001	COL11A2
TC6_cox_hap2000	2.46	JUC6_cox_hap2001	COL11A2
TC6_cox_hap2000	2.4	JUC6_cox_hap2001	COL11A2
TC6_cox_hap2000	2.15	JUC6_cox_hap2001	COL11A2
TC6_cox_hap2000	2.03	JUC6_cox_hap2001	COL11A2
TC6_cox_hap2000	-2.04	JUC6_cox_hap2001	COL11A2
TC6_cox_hap2000	-2.21	PSR6_cox_hap2004	COL11A2
TC6_cox_hap2000	-2.25	JUC6_cox_hap2001	COL11A2
TC6_cox_hap2000	-2.28	PSR6_cox_hap2004	COL11A2
TC6_cox_hap2000	-2.55	JUC6_cox_hap2001	COL11A2
TC6_cox_hap2000	-2.86	JUC6_cox_hap2001	COL11A2

TC6_cox_hap2000	-2.94	JUC6_cox_hap20015	COL11A2
TC6_cox_hap2000	-5.79	JUC6_cox_hap20015	COL11A2
TC6_dbb_hap3000	5.29	JUC6_dbb_hap30014	COL11A2
TC6_dbb_hap3000	2.61	JUC6_dbb_hap30014	COL11A2
TC6_dbb_hap3000	2.46	JUC6_dbb_hap30013	COL11A2
TC6_dbb_hap3000	2.4	JUC6_dbb_hap30014	COL11A2
TC6_dbb_hap3000	2.15	JUC6_dbb_hap30014	COL11A2
TC6_dbb_hap3000	2.03	JUC6_dbb_hap30014	COL11A2
TC6_dbb_hap3000	-2.04	JUC6_dbb_hap30014	COL11A2
TC6_dbb_hap3000	-2.21	PSR6_dbb_hap30038	COL11A2
TC6_dbb_hap3000	-2.25	JUC6_dbb_hap30014	COL11A2
TC6_dbb_hap3000	-2.28	PSR6_dbb_hap30038	COL11A2
TC6_dbb_hap3000	-2.55	JUC6_dbb_hap30013	COL11A2
TC6_dbb_hap3000	-2.86	JUC6_dbb_hap30014	COL11A2
TC6_dbb_hap3000	-2.94	JUC6_dbb_hap30014	COL11A2
TC6_dbb_hap3000	-5.79	JUC6_dbb_hap30014	COL11A2
TC6_mcf_hap5000	5.29	JUC6_mcf_hap50012	COL11A2
TC6_mcf_hap5000	2.61	JUC6_mcf_hap50013	COL11A2
TC6_mcf_hap5000	2.46	JUC6_mcf_hap50012	COL11A2
TC6_mcf_hap5000	2.4	JUC6_mcf_hap50013	COL11A2
TC6_mcf_hap5000	2.15	JUC6_mcf_hap50013	COL11A2
TC6_mcf_hap5000	2.03	JUC6_mcf_hap50013	COL11A2
TC6_mcf_hap5000	-2.04	JUC6_mcf_hap50013	COL11A2
TC6_mcf_hap5000	-2.21	PSR6_mcf_hap50037	COL11A2
TC6_mcf_hap5000	-2.25	JUC6_mcf_hap50013	COL11A2
TC6_mcf_hap5000	-2.28	PSR6_mcf_hap50037	COL11A2
TC6_mcf_hap5000	-2.55	JUC6_mcf_hap50012	COL11A2
TC6_mcf_hap5000	-2.86	JUC6_mcf_hap50013	COL11A2
TC6_mcf_hap5000	-2.94	JUC6_mcf_hap50013	COL11A2
TC6_mcf_hap5000	-5.79	JUC6_mcf_hap50013	COL11A2
TC6_qbl_hap6000	5.29	JUC6_qbl_hap60015	COL11A2
TC6_qbl_hap6000	2.61	JUC6_qbl_hap60015	COL11A2
TC6_qbl_hap6000	2.46	JUC6_qbl_hap60015	COL11A2
TC6_qbl_hap6000	2.4	JUC6_qbl_hap60015	COL11A2
TC6_qbl_hap6000	2.15	JUC6_qbl_hap60015	COL11A2
TC6_qbl_hap6000	2.03	JUC6_qbl_hap60015	COL11A2
TC6_qbl_hap6000	-2.04	JUC6_qbl_hap60015	COL11A2
TC6_qbl_hap6000	-2.21	PSR6_qbl_hap60039	COL11A2
TC6_qbl_hap6000	-2.25	JUC6_qbl_hap60015	COL11A2
TC6_qbl_hap6000	-2.28	PSR6_qbl_hap60039	COL11A2
TC6_qbl_hap6000	-2.55	JUC6_qbl_hap60015	COL11A2
TC6_qbl_hap6000	-2.86	JUC6_qbl_hap60015	COL11A2
TC6_qbl_hap6000	-2.94	JUC6_qbl_hap60015	COL11A2
TC6_qbl_hap6000	-5.79	JUC6_qbl_hap60015	COL11A2
TC03000076.hg.1	5.27	JUC03000873.hg.1	HDAC11
TC03000076.hg.1	4.69	JUC03000892.hg.1	HDAC11
TC03000076.hg.1	3.63	JUC03000890.hg.1	HDAC11
TC03000076.hg.1	3.03	JUC03000880.hg.1	HDAC11
TC03000076.hg.1	2.83	PSR03001655.hg.1	HDAC11
TC03000076.hg.1	2.79	PSR03001675.hg.1	HDAC11
TC03000076.hg.1	2.5	PSR03001656.hg.1	HDAC11
TC03000076.hg.1	2.47	PSR03001707.hg.1	HDAC11
TC03000076.hg.1	2.39	PSR03001665.hg.1	HDAC11
TC03000076.hg.1	2.39	PSR03001667.hg.1	HDAC11
TC03000076.hg.1	2.36	PSR03001705.hg.1	HDAC11
TC03000076.hg.1	2.28	JUC03000895.hg.1	HDAC11
TC03000076.hg.1	2.1	PSR03001706.hg.1	HDAC11
TC03000076.hg.1	2.09	PSR03001708.hg.1	HDAC11

TC03000076.hg.1	2.06	PSR03001682.hg.1	HDAC11
TC03000076.hg.1	2.06	JUC03000889.hg.1	HDAC11
TC03000076.hg.1	-2.78	JUC03000886.hg.1	HDAC11
TC03000076.hg.1	-3.24	PSR03001694.hg.1	HDAC11
TC05001385.hg.1	5.27	PSR05019320.hg.1	PLK2
TC05001385.hg.1	5.13	PSR05019301.hg.1	PLK2
TC05001385.hg.1	5.12	PSR05019305.hg.1	PLK2
TC05001385.hg.1	3.95	PSR05019306.hg.1	PLK2
TC05001385.hg.1	3.89	JUC05009929.hg.1	PLK2
TC05001385.hg.1	3.68	PSR05019290.hg.1	PLK2
TC05001385.hg.1	3.48	PSR05019267.hg.1	PLK2
TC05001385.hg.1	3.41	PSR05019308.hg.1	PLK2
TC05001385.hg.1	2.97	PSR05019313.hg.1	PLK2
TC05001385.hg.1	2.92	JUC05009933.hg.1	PLK2
TC05001385.hg.1	2.91	PSR05019302.hg.1	PLK2
TC05001385.hg.1	2.82	PSR05019295.hg.1	PLK2
TC05001385.hg.1	2.82	JUC05009946.hg.1	PLK2
TC05001385.hg.1	2.69	PSR05019298.hg.1	PLK2
TC05001385.hg.1	2.61	PSR05019277.hg.1	PLK2
TC05001385.hg.1	2.42	PSR05019315.hg.1	PLK2
TC05001385.hg.1	2.22	PSR05019263.hg.1	PLK2
TC05001385.hg.1	2.19	PSR05019276.hg.1	PLK2
TC05001385.hg.1	2.12	PSR05019316.hg.1	PLK2
TC07001501.hg.1	5.27	JUC07011080.hg.1	TBL2
TC07001501.hg.1	2.39	PSR07022522.hg.1	TBL2
TC07001501.hg.1	2.34	JUC07011069.hg.1	TBL2
TC07001501.hg.1	2.31	PSR07022555.hg.1	TBL2
TC07001501.hg.1	2.17	PSR07022509.hg.1	TBL2
TC07001501.hg.1	2.16	PSR07022534.hg.1	TBL2
TC07001501.hg.1	2.11	JUC07011074.hg.1	TBL2
TC07001501.hg.1	2.06	PSR07022504.hg.1	TBL2
TC07001501.hg.1	-2.22	JUC07011063.hg.1	TBL2
TC14000948.hg.1	5.27	PSR14010481.hg.1	ACIN1
TC14000948.hg.1	2.54	JUC14005313.hg.1	ACIN1
TC14000948.hg.1	-2.05	JUC14005302.hg.1	ACIN1
TC14000948.hg.1	-2.97	JUC14005325.hg.1	ACIN1
TC16000381.hg.1	5.27	JUC16003130.hg.1	KIAA0664L3
TC16000381.hg.1	4.83	JUC16003132.hg.1	KIAA0664L3
TC16000381.hg.1	2.74	JUC16003138.hg.1	KIAA0664L3
TC16000381.hg.1	2.55	PSR16005676.hg.1	KIAA0664L3
TC16000381.hg.1	2.27	PSR16005672.hg.1	KIAA0664L3
TC16000381.hg.1	-2.08	PSR16005678.hg.1	KIAA0664L3
TC6_dbb_hap3000	5.27	PSR6_dbb_hap3000	HLA-H
TC6_dbb_hap3000	3.53	PSR6_dbb_hap3000	HLA-H
TC6_dbb_hap3000	2.6	JUC6_dbb_hap3001(	HLA-H
TC6_dbb_hap3000	2.3	JUC6_dbb_hap3001(	HLA-H
TC6_dbb_hap3000	2.1	PSR6_dbb_hap3000:	HLA-H
TC6_dbb_hap3000	2.06	PSR6_dbb_hap3000	HLA-H
TC01002163.hg.1	5.26	JUC01017910.hg.1	TNFRSF9
TC01002163.hg.1	5.14	PSR01033393.hg.1	TNFRSF9
TC01002163.hg.1	4.86	JUC01017913.hg.1	TNFRSF9
TC01002163.hg.1	4.17	PSR01033391.hg.1	TNFRSF9
TC01002163.hg.1	3.52	PSR01033383.hg.1	TNFRSF9
TC01002163.hg.1	2.53	PSR01033390.hg.1	TNFRSF9
TC01002163.hg.1	-2.05	JUC01017908.hg.1	TNFRSF9
TC01002163.hg.1	-3.14	PSR01033386.hg.1	TNFRSF9
TC02001495.hg.1	5.26	JUC02012693.hg.1	SH3YL1
TC02001495.hg.1	3.67	JUC02012660.hg.1	SH3YL1

TC02001495.hg.1	3.53	PSR02024531.hg.1	SH3YL1
TC02001495.hg.1	3.1	PSR02024528.hg.1	SH3YL1
TC02001495.hg.1	2.81	PSR02024524.hg.1	SH3YL1
TC02001495.hg.1	2.81	PSR02024529.hg.1	SH3YL1
TC02001495.hg.1	2.67	JUC02012694.hg.1	SH3YL1
TC02001495.hg.1	2.64	PSR02024469.hg.1	SH3YL1
TC02001495.hg.1	2.56	PSR02024468.hg.1	SH3YL1
TC02001495.hg.1	2.49	PSR02024472.hg.1	SH3YL1
TC02001495.hg.1	2.43	PSR02024527.hg.1	SH3YL1
TC02001495.hg.1	2.39	PSR02024534.hg.1	SH3YL1
TC02001495.hg.1	2.35	JUC02012683.hg.1	SH3YL1
TC02001495.hg.1	2.27	JUC02012689.hg.1	SH3YL1
TC02001495.hg.1	2.17	PSR02024518.hg.1	SH3YL1
TC02001495.hg.1	2.17	PSR02024530.hg.1	SH3YL1
TC02001495.hg.1	2.09	PSR02024455.hg.1	SH3YL1
TC02001495.hg.1	2.04	JUC02012681.hg.1	SH3YL1
TC02001495.hg.1	2.01	PSR02024476.hg.1	SH3YL1
TC02001495.hg.1	-2.19	PSR02024509.hg.1	SH3YL1
TC02001495.hg.1	-2.2	PSR02024499.hg.1	SH3YL1
TC02001495.hg.1	-5.35	JUC02012669.hg.1	SH3YL1
TC02001495.hg.1	-8.96	JUC02012658.hg.1	SH3YL1
TC14000440.hg.1	5.26	JUC14002843.hg.1	PAPLN
TC14000440.hg.1	3.7	PSR14005411.hg.1	PAPLN
TC14000440.hg.1	3.51	PSR14005444.hg.1	PAPLN
TC14000440.hg.1	2.77	JUC14002842.hg.1	PAPLN
TC14000440.hg.1	2.11	PSR14005442.hg.1	PAPLN
TC01006404.hg.1	5.25	PSR01016601.hg.1	
TC01006404.hg.1	5.09	JUC01046653.hg.1	
TC01006404.hg.1	4.14	JUC01046654.hg.1	
TC01006404.hg.1	3.42	JUC01046658.hg.1	
TC01006404.hg.1	3.01	PSR01016603.hg.1	
TC01006404.hg.1	2.68	JUC01046657.hg.1	
TC01006404.hg.1	2.21	PSR01016609.hg.1	
TC01006404.hg.1	2.14	PSR01016610.hg.1	
TC02002529.hg.1	5.25	JUC02021160.hg.1	
TC02002529.hg.1	4.22	PSR02040131.hg.1	
TC02002529.hg.1	2.95	PSR02040135.hg.1	
TC02002529.hg.1	2.73	PSR02040139.hg.1	
TC02002529.hg.1	2.64	PSR02040130.hg.1	
TC02002529.hg.1	2.42	PSR02040138.hg.1	
TC02002529.hg.1	2.09	PSR02040136.hg.1	
TC02002529.hg.1	2.07	PSR02040129.hg.1	
TC02002529.hg.1	-2.09	PSR02040132.hg.1	
TC02002529.hg.1	-2.58	JUC02021156.hg.1	
TC10000081.hg.1	5.25	JUC10000441.hg.1	CELF2
TC10000081.hg.1	3.59	JUC10000449.hg.1	CELF2
TC10000081.hg.1	3.46	JUC10000453.hg.1	CELF2
TC10000081.hg.1	2.61	PSR10000864.hg.1	CELF2
TC10000081.hg.1	2.56	PSR10000854.hg.1	CELF2
TC10000081.hg.1	2.5	JUC10000456.hg.1	CELF2
TC10000081.hg.1	2.27	PSR10000853.hg.1	CELF2
TC10000081.hg.1	2.23	JUC10000450.hg.1	CELF2
TC10000081.hg.1	2.17	PSR10000842.hg.1	CELF2
TC10000081.hg.1	2.15	PSR10000839.hg.1	CELF2
TC10000081.hg.1	2.06	JUC10000454.hg.1	CELF2
TC10000081.hg.1	2.03	PSR10000871.hg.1	CELF2
TC10000081.hg.1	-2.22	PSR10000866.hg.1	CELF2
TC10000081.hg.1	-2.29	JUC10000452.hg.1	CELF2

TC10000081.hg.1	-2.33	PSR10000843.hg.1	CELF2
TC10000081.hg.1	-3.78	JUC10000457.hg.1	CELF2
TC15001136.hg.1	5.24	JUC15005382.hg.1	CHRFAM7A
TC15001136.hg.1	3.19	PSR15010181.hg.1	CHRFAM7A
TC15001136.hg.1	2.61	PSR15010183.hg.1	CHRFAM7A
TC15001136.hg.1	2.52	JUC15005388.hg.1	CHRFAM7A
TC15001136.hg.1	2.37	PSR15010184.hg.1	CHRFAM7A
TC15001136.hg.1	2	PSR15010182.hg.1	CHRFAM7A
TC10001448.hg.1	5.23	JUC10010737.hg.1	LOC283050
TC10001448.hg.1	4.27	JUC10010739.hg.1	LOC283050
TC10001448.hg.1	3.94	JUC10010742.hg.1	LOC283050
TC10001448.hg.1	2.41	JUC10010744.hg.1	LOC283050
TC10001448.hg.1	2.35	JUC10010740.hg.1	LOC283050
TC10001448.hg.1	2.21	PSR10018392.hg.1	LOC283050
TC10001448.hg.1	2.06	JUC10010738.hg.1	LOC283050
TC10001448.hg.1	2.02	PSR10018408.hg.1	LOC283050
TC01003226.hg.1	5.22	JUC01026389.hg.1	SELENBP1
TC01003226.hg.1	2.43	PSR01049721.hg.1	SELENBP1
TC01003226.hg.1	2.23	PSR01049710.hg.1	SELENBP1
TC01003226.hg.1	2.16	PSR01049730.hg.1	SELENBP1
TC01003226.hg.1	2.1	PSR01049723.hg.1	SELENBP1
TC01003226.hg.1	2	JUC01026386.hg.1	SELENBP1
TC03000208.hg.1	5.22	JUC03002206.hg.1	ENTPD3
TC03000208.hg.1	3.65	JUC03002200.hg.1	ENTPD3
TC03000208.hg.1	-2.15	PSR03004121.hg.1	ENTPD3
TC03000208.hg.1	-2.54	JUC03002199.hg.1	ENTPD3
TC03000208.hg.1	-3.37	PSR03004126.hg.1	ENTPD3
TC03000208.hg.1	-4.83	PSR03004119.hg.1	ENTPD3
TC03000208.hg.1	-5.58	PSR03004123.hg.1	ENTPD3
TC03000208.hg.1	-6.42	JUC03002201.hg.1	ENTPD3
TC03000208.hg.1	-7.28	PSR03004120.hg.1	ENTPD3
TC04001169.hg.1	5.22	PSR04016020.hg.1	CORIN
TC04001169.hg.1	2.81	PSR04016032.hg.1	CORIN
TC04001169.hg.1	2.78	PSR04016054.hg.1	CORIN
TC04001169.hg.1	2.41	PSR04016030.hg.1	CORIN
TC04001169.hg.1	2.41	JUC04008509.hg.1	CORIN
TC04001169.hg.1	2.13	JUC04008491.hg.1	CORIN
TC04001169.hg.1	-2.45	JUC04008496.hg.1	CORIN
TC04001169.hg.1	-2.48	PSR04016026.hg.1	CORIN
TC04001169.hg.1	-2.78	PSR04016023.hg.1	CORIN
TC04001169.hg.1	-2.85	PSR04016039.hg.1	CORIN
TC04001169.hg.1	-2.86	JUC04008506.hg.1	CORIN
TC04001169.hg.1	-3.75	JUC04008505.hg.1	CORIN
TC04001169.hg.1	-3.81	JUC04008517.hg.1	CORIN
TC04001169.hg.1	-3.82	PSR04016028.hg.1	CORIN
TC04001169.hg.1	-3.83	PSR04016043.hg.1	CORIN
TC04001169.hg.1	-5	JUC04008499.hg.1	CORIN
TC04001169.hg.1	-5.75	JUC04008514.hg.1	CORIN
TC04001169.hg.1	-6.37	PSR04016041.hg.1	CORIN
TC04001169.hg.1	-6.45	JUC04008490.hg.1	CORIN
TC04001169.hg.1	-6.69	JUC04008498.hg.1	CORIN
TC04001169.hg.1	-7.47	JUC04008503.hg.1	CORIN
TC04001169.hg.1	-13.29	JUC04008495.hg.1	CORIN
TC04001169.hg.1	-16.48	JUC04008516.hg.1	CORIN
TC04001169.hg.1	-20.18	PSR04016053.hg.1	CORIN
TC04001169.hg.1	-22.4	PSR04016050.hg.1	CORIN
TC04001169.hg.1	-24.14	PSR04016057.hg.1	CORIN
TC04001169.hg.1	-27.5	PSR04016027.hg.1	CORIN

TC01001399.hg.1	5.21	JUC01011642.hg.1	FCGR2A
TC01001399.hg.1	3.49	PSR01021959.hg.1	FCGR2A
TC01001399.hg.1	3.15	PSR01021960.hg.1	FCGR2A
TC01001399.hg.1	2.65	PSR01021958.hg.1	FCGR2A
TC01001399.hg.1	2.65	PSR01021987.hg.1	FCGR2A
TC01001399.hg.1	2.59	PSR01021965.hg.1	FCGR2A
TC01001399.hg.1	2.54	JUC01011634.hg.1	FCGR2A
TC01001399.hg.1	2.43	PSR01021982.hg.1	FCGR2A
TC01001399.hg.1	2.41	JUC01011639.hg.1	FCGR2A
TC01001399.hg.1	2.26	PSR01021967.hg.1	FCGR2A
TC01001399.hg.1	2.14	JUC01011636.hg.1	FCGR2A
TC01001399.hg.1	-2.54	PSR01021970.hg.1	FCGR2A
TC01001399.hg.1	-4.66	PSR01021972.hg.1	FCGR2A
TC01001399.hg.1	-5.28	JUC01011643.hg.1	FCGR2A
TC01001399.hg.1	-10.33	PSR01021973.hg.1	FCGR2A
TC01001399.hg.1	-10.69	PSR01021977.hg.1	FCGR2A
TC02001904.hg.1	5.21	JUC02016053.hg.1	WDPCP
TC02001904.hg.1	2.72	JUC02016047.hg.1	WDPCP
TC02001904.hg.1	2.34	JUC02016040.hg.1	WDPCP
TC02001904.hg.1	2.03	PSR02030494.hg.1	WDPCP
TC02001904.hg.1	2.03	PSR02030511.hg.1	WDPCP
TC07000559.hg.1	5.21	PSR07008216.hg.1	COL1A2
TC07000559.hg.1	3.43	PSR07008241.hg.1	COL1A2
TC07000559.hg.1	3.1	PSR07008234.hg.1	COL1A2
TC07000559.hg.1	2.86	PSR07008221.hg.1	COL1A2
TC07000559.hg.1	2.81	PSR07008247.hg.1	COL1A2
TC07000559.hg.1	2.68	PSR07008232.hg.1	COL1A2
TC07000559.hg.1	2.66	PSR07008242.hg.1	COL1A2
TC07000559.hg.1	2.59	PSR07008249.hg.1	COL1A2
TC07000559.hg.1	2.14	PSR07008230.hg.1	COL1A2
TC07000559.hg.1	2.07	PSR07008227.hg.1	COL1A2
TC07000559.hg.1	2.06	PSR07008243.hg.1	COL1A2
TC07000559.hg.1	2.03	PSR07008254.hg.1	COL1A2
TC07000559.hg.1	-2	JUC07003932.hg.1	COL1A2
TC07000559.hg.1	-2.01	JUC07003952.hg.1	COL1A2
TC07000559.hg.1	-2.03	PSR07008244.hg.1	COL1A2
TC07000559.hg.1	-2.1	PSR07008253.hg.1	COL1A2
TC07000559.hg.1	-2.16	JUC07003924.hg.1	COL1A2
TC07000559.hg.1	-2.21	PSR07008256.hg.1	COL1A2
TC07000559.hg.1	-2.26	PSR07008250.hg.1	COL1A2
TC07000559.hg.1	-2.29	JUC07003915.hg.1	COL1A2
TC07000559.hg.1	-2.37	PSR07008255.hg.1	COL1A2
TC07000559.hg.1	-2.39	PSR07008251.hg.1	COL1A2
TC07000559.hg.1	-2.41	PSR07008252.hg.1	COL1A2
TC07000559.hg.1	-2.71	JUC07003935.hg.1	COL1A2
TC07000559.hg.1	-2.75	PSR07008173.hg.1	COL1A2
TC02001610.hg.1	5.2	JUC02013413.hg.1	
TC02001610.hg.1	3.67	JUC02013414.hg.1	
TC02001610.hg.1	3.09	PSR02025810.hg.1	
TC02001610.hg.1	2.65	JUC02013415.hg.1	
TC02001610.hg.1	2.48	PSR02025809.hg.1	
TC02001610.hg.1	2.44	PSR02025805.hg.1	
TC02001610.hg.1	2.09	JUC02013412.hg.1	
TC01000184.hg.1	5.19	JUC01001610.hg.1	KAZN
TC01000184.hg.1	2.71	JUC01001611.hg.1	KAZN
TC01000184.hg.1	2.6	PSR01002917.hg.1	KAZN
TC01000184.hg.1	2.56	PSR01002938.hg.1	KAZN
TC01000184.hg.1	2.33	PSR01002912.hg.1	KAZN

TC01000184.hg.1	2.15	JUC01001612.hg.1	KAZN
TC01000184.hg.1	2.06	PSR01002916.hg.1	KAZN
TC01000184.hg.1	2.04	JUC01001621.hg.1	KAZN
TC01000184.hg.1	2.04	JUC01001624.hg.1	KAZN
TC01000184.hg.1	2	PSR01002935.hg.1	KAZN
TC01006369.hg.1	5.19	JUC01045125.hg.1	NBPF10, LO(
TC01006369.hg.1	2.47	PSR01048679.hg.1	NBPF10, LO(
TC01006369.hg.1	2.15	PSR01048654.hg.1	NBPF10, LO(
TC01006369.hg.1	2.15	PSR01048706.hg.1	NBPF10, LO(
TC01006369.hg.1	2.15	PSR01048715.hg.1	NBPF10, LO(
TC01006369.hg.1	2.09	PSR01048708.hg.1	NBPF10, LO(
TC01006369.hg.1	2.09	PSR01048717.hg.1	NBPF10, LO(
TC01006369.hg.1	2.06	PSR01048690.hg.1	NBPF10, LO(
TC04001820.hg.1	5.18	PSR04024888.hg.1	SORBS2
TC04001820.hg.1	4.26	PSR04024903.hg.1	SORBS2
TC04001820.hg.1	4.09	JUC04013189.hg.1	SORBS2
TC04001820.hg.1	3.89	JUC04013236.hg.1	SORBS2
TC04001820.hg.1	3.87	PSR04025012.hg.1	SORBS2
TC04001820.hg.1	3.64	PSR04025026.hg.1	SORBS2
TC04001820.hg.1	3.58	PSR04025034.hg.1	SORBS2
TC04001820.hg.1	3.56	JUC04013212.hg.1	SORBS2
TC04001820.hg.1	3.41	JUC04013248.hg.1	SORBS2
TC04001820.hg.1	3.34	JUC04013204.hg.1	SORBS2
TC04001820.hg.1	3.02	JUC04013247.hg.1	SORBS2
TC04001820.hg.1	2.99	JUC04013221.hg.1	SORBS2
TC04001820.hg.1	2.9	JUC04013217.hg.1	SORBS2
TC04001820.hg.1	2.87	JUC04013195.hg.1	SORBS2
TC04001820.hg.1	2.81	PSR04025040.hg.1	SORBS2
TC04001820.hg.1	2.67	PSR04025027.hg.1	SORBS2
TC04001820.hg.1	2.48	PSR04024935.hg.1	SORBS2
TC04001820.hg.1	2.47	PSR04025035.hg.1	SORBS2
TC04001820.hg.1	2.44	JUC04013229.hg.1	SORBS2
TC04001820.hg.1	2.35	PSR04025015.hg.1	SORBS2
TC04001820.hg.1	2.28	PSR04025038.hg.1	SORBS2
TC04001820.hg.1	2.25	PSR04025019.hg.1	SORBS2
TC04001820.hg.1	2.23	PSR04024894.hg.1	SORBS2
TC04001820.hg.1	2.2	PSR04025010.hg.1	SORBS2
TC04001820.hg.1	2.17	PSR04024913.hg.1	SORBS2
TC04001820.hg.1	2.14	PSR04024994.hg.1	SORBS2
TC04001820.hg.1	2.1	PSR04024904.hg.1	SORBS2
TC04001820.hg.1	2.1	PSR04025017.hg.1	SORBS2
TC04001820.hg.1	2.07	PSR04024997.hg.1	SORBS2
TC04001820.hg.1	2.03	JUC04013200.hg.1	SORBS2
TC04001820.hg.1	2	JUC04013214.hg.1	SORBS2
TC04001820.hg.1	-4.02	JUC04013224.hg.1	SORBS2
TC06001332.hg.1	5.18	JUC06008028.hg.1	CMAHP
TC06001332.hg.1	-2.03	PSR06015966.hg.1	CMAHP
TC06001332.hg.1	-2.2	PSR06015985.hg.1	CMAHP
TC06001332.hg.1	-2.2	PSR06016004.hg.1	CMAHP
TC06001332.hg.1	-2.46	PSR06016001.hg.1	CMAHP
TC06001332.hg.1	-2.51	PSR06016003.hg.1	CMAHP
TC06001332.hg.1	-3.33	PSR06015972.hg.1	CMAHP
TC06001332.hg.1	-3.63	PSR06015981.hg.1	CMAHP
TC06001332.hg.1	-4.02	PSR06015975.hg.1	CMAHP
TC06001332.hg.1	-4.06	PSR06015999.hg.1	CMAHP
TC06001332.hg.1	-4.15	PSR06016000.hg.1	CMAHP
TC06001332.hg.1	-4.37	PSR06015973.hg.1	CMAHP
TC06001332.hg.1	-4.76	PSR06015995.hg.1	CMAHP

TC06001332.hg.1	-5.25	PSR06015965.hg.1	CMAHP
TC06001332.hg.1	-5.65	PSR06015974.hg.1	CMAHP
TC06001332.hg.1	-6.74	PSR06015984.hg.1	CMAHP
TC06001332.hg.1	-7.33	JUC06008018.hg.1	CMAHP
TC08000452.hg.1	5.18	JUC08003116.hg.1	PREX2
TC08000452.hg.1	5.06	JUC08003104.hg.1	PREX2
TC08000452.hg.1	4.32	JUC08003110.hg.1	PREX2
TC08000452.hg.1	3.8	JUC08003140.hg.1	PREX2
TC08000452.hg.1	3.69	JUC08003127.hg.1	PREX2
TC08000452.hg.1	3.45	JUC08003128.hg.1	PREX2
TC08000452.hg.1	3.42	PSR08006423.hg.1	PREX2
TC08000452.hg.1	3.08	PSR08006404.hg.1	PREX2
TC08000452.hg.1	2.99	PSR08006424.hg.1	PREX2
TC08000452.hg.1	2.65	PSR08006433.hg.1	PREX2
TC08000452.hg.1	2.46	PSR08006422.hg.1	PREX2
TC08000452.hg.1	2.37	JUC08003119.hg.1	PREX2
TC08000452.hg.1	2.28	PSR08006421.hg.1	PREX2
TC08000452.hg.1	2.08	PSR08006425.hg.1	PREX2
TC08000452.hg.1	2.07	JUC08003099.hg.1	PREX2
TC08000452.hg.1	2.06	PSR08006426.hg.1	PREX2
TC08000452.hg.1	-2.4	JUC08003142.hg.1	PREX2
TC08000452.hg.1	-4.1	JUC08003134.hg.1	PREX2
TC15001636.hg.1	5.17	JUC15008577.hg.1	LOXL1-AS1
TC15001636.hg.1	2.53	PSR15015878.hg.1	LOXL1-AS1
TC15001636.hg.1	2.21	PSR15015877.hg.1	LOXL1-AS1
TC15001636.hg.1	2.08	PSR15015875.hg.1	LOXL1-AS1
TC15001636.hg.1	2.06	JUC15008578.hg.1	LOXL1-AS1
TC21000039.hg.1	5.17	JUC21000081.hg.1	USP25
TC21000039.hg.1	-2.06	PSR21000147.hg.1	USP25
TC17000914.hg.1	5.16	JUC17006637.hg.1	CCDC40
TC17000914.hg.1	3.55	PSR17011848.hg.1	CCDC40
TC17000914.hg.1	3.32	JUC17006646.hg.1	CCDC40
TC6_qbl_hap6000´	5.16	PSR6_qbl_hap60022	ZNRD1-AS1
TC6_qbl_hap6000´	4.45	PSR6_qbl_hap60021	ZNRD1-AS1
TC6_qbl_hap6000´	3.58	PSR6_qbl_hap60021	ZNRD1-AS1
TC6_qbl_hap6000´	3.4	PSR6_qbl_hap60021	ZNRD1-AS1
TC6_qbl_hap6000´	3.29	PSR6_qbl_hap60021	ZNRD1-AS1
TC6_qbl_hap6000´	3.11	PSR6_qbl_hap60021	ZNRD1-AS1
TC6_qbl_hap6000´	3.06	PSR6_qbl_hap60021	ZNRD1-AS1
TC6_qbl_hap6000´	2.92	PSR6_qbl_hap60021	ZNRD1-AS1
TC6_qbl_hap6000´	2.83	PSR6_qbl_hap60021	ZNRD1-AS1
TC6_qbl_hap6000´	2.65	JUC6_qbl_hap60007	ZNRD1-AS1
TC6_qbl_hap6000´	2.58	PSR6_qbl_hap60021	ZNRD1-AS1
TC6_qbl_hap6000´	2.58	JUC6_qbl_hap60007	ZNRD1-AS1
TC6_qbl_hap6000´	2.36	PSR6_qbl_hap60021	ZNRD1-AS1
TC6_qbl_hap6000´	-2.55	JUC6_qbl_hap60007	ZNRD1-AS1
TC0X000724.hg.1	5.15	JUC0X004713.hg.1	MTMR1
TC0X000724.hg.1	2.47	PSR0X009344.hg.1	MTMR1
TC0X000724.hg.1	2.46	PSR0X009314.hg.1	MTMR1
TC0X000724.hg.1	2.33	PSR0X009319.hg.1	MTMR1
TC0X000724.hg.1	2.33	PSR0X009338.hg.1	MTMR1
TC0X000724.hg.1	2.22	PSR0X009337.hg.1	MTMR1
TC0X000724.hg.1	2.22	JUC0X004738.hg.1	MTMR1
TC0X000724.hg.1	2.11	PSR0X009349.hg.1	MTMR1
TC07000424.hg.1	5.14	JUC07002697.hg.1	AUTS2
TC07000424.hg.1	2.45	PSR07005666.hg.1	AUTS2
TC07000424.hg.1	2	JUC07002703.hg.1	AUTS2
TC22000019.hg.1	5.14	JUC22000038.hg.1	IL17RA

TC22000019.hg.1	4.45	PSR22000087.hg.1	IL17RA
TC22000019.hg.1	3.1	JUC22000039.hg.1	IL17RA
TC22000019.hg.1	2.81	PSR22000070.hg.1	IL17RA
TC22000019.hg.1	2.56	PSR22000072.hg.1	IL17RA
TC05000258.hg.1	5.13	JUC05001746.hg.1	PART1
TC05000258.hg.1	4.64	JUC05001745.hg.1	PART1
TC05000258.hg.1	4.04	PSR05003151.hg.1	PART1
TC05000258.hg.1	3.38	PSR05003152.hg.1	PART1
TC05000258.hg.1	3.12	PSR05003153.hg.1	PART1
TC07003396.hg.1	5.13	JUC07021873.hg.1	TRRAP
TC07003396.hg.1	2.19	PSR07008540.hg.1	TRRAP
TC07003396.hg.1	2.13	PSR07008577.hg.1	TRRAP
TC07003396.hg.1	2.09	JUC07021897.hg.1	TRRAP
TC07003396.hg.1	-2.01	JUC07021910.hg.1	TRRAP
TC07003396.hg.1	-2.15	JUC07021902.hg.1	TRRAP
TC11002170.hg.1	5.13	JUC11013816.hg.1	FZD4
TC11002170.hg.1	3.52	PSR11026010.hg.1	FZD4
TC05000849.hg.1	5.12	JUC05006395.hg.1	GRIA1
TC05000849.hg.1	3.36	JUC05006399.hg.1	GRIA1
TC05000849.hg.1	2.79	PSR05012232.hg.1	GRIA1
TC05000849.hg.1	2.64	PSR05012238.hg.1	GRIA1
TC05000849.hg.1	2.43	PSR05012225.hg.1	GRIA1
TC05000849.hg.1	2.4	JUC05006389.hg.1	GRIA1
TC05000849.hg.1	2.32	JUC05006392.hg.1	GRIA1
TC05000849.hg.1	2.16	JUC05006384.hg.1	GRIA1
TC05000849.hg.1	2.15	PSR05012243.hg.1	GRIA1
TC05000849.hg.1	2.04	PSR05012235.hg.1	GRIA1
TC05000849.hg.1	2.03	PSR05012261.hg.1	GRIA1
TC05000849.hg.1	-2.08	PSR05012270.hg.1	GRIA1
TC05000849.hg.1	-2.45	JUC05006404.hg.1	GRIA1
TC05000849.hg.1	-2.57	PSR05012272.hg.1	GRIA1
TC05000849.hg.1	-2.68	JUC05006397.hg.1	GRIA1
TC07000296.hg.1	5.11	PSR07004546.hg.1	
TC07000296.hg.1	3.75	JUC07002181.hg.1	
TC02000704.hg.1	5.1	JUC02005753.hg.1	FBLN7
TC02000704.hg.1	4.62	PSR02010875.hg.1	FBLN7
TC02000704.hg.1	4	PSR02010887.hg.1	FBLN7
TC02000704.hg.1	3.69	JUC02005764.hg.1	FBLN7
TC02000704.hg.1	3.34	PSR02010886.hg.1	FBLN7
TC02000704.hg.1	2.94	PSR02010868.hg.1	FBLN7
TC02000704.hg.1	2.64	JUC02005766.hg.1	FBLN7
TC02000704.hg.1	2.4	PSR02010874.hg.1	FBLN7
TC02000704.hg.1	2.18	PSR02010873.hg.1	FBLN7
TC02000704.hg.1	2	JUC02005761.hg.1	FBLN7
TC02000704.hg.1	-2	JUC02005765.hg.1	FBLN7
TC02000704.hg.1	-2.04	JUC02005760.hg.1	FBLN7
TC02000704.hg.1	-2.08	PSR02010880.hg.1	FBLN7
TC02000704.hg.1	-2.64	PSR02010883.hg.1	FBLN7
TC03001849.hg.1	5.1	JUC03016541.hg.1	TFDP2
TC03001849.hg.1	-2.08	JUC03016524.hg.1	TFDP2
TC03001849.hg.1	-2.14	JUC03016539.hg.1	TFDP2
TC03001849.hg.1	-2.47	PSR03033311.hg.1	TFDP2
TC20000802.hg.1	5.1	JUC20005685.hg.1	FER1L4
TC20000802.hg.1	2.58	JUC20005675.hg.1	FER1L4
TC20000802.hg.1	2.34	PSR20010821.hg.1	FER1L4
TC20000802.hg.1	2.22	PSR20010829.hg.1	FER1L4
TC20000802.hg.1	2.18	JUC20005647.hg.1	FER1L4
TC20000802.hg.1	2.17	PSR20010818.hg.1	FER1L4

TC20000802.hg.1	2.09	PSR20010845.hg.1	FER1L4
TC20000802.hg.1	2.04	JUC20005645.hg.1	FER1L4
TC20000802.hg.1	-2.19	JUC20005662.hg.1	FER1L4
TC20000802.hg.1	-2.25	PSR20010824.hg.1	FER1L4
TC20000802.hg.1	-2.37	JUC20005654.hg.1	FER1L4
TC20000802.hg.1	-2.38	PSR20010838.hg.1	FER1L4
TC20000802.hg.1	-2.44	PSR20010814.hg.1	FER1L4
TC20000802.hg.1	-2.51	PSR20010849.hg.1	FER1L4
TC20000802.hg.1	-2.51	JUC20005669.hg.1	FER1L4
TC20000802.hg.1	-2.77	JUC20005664.hg.1	FER1L4
TC20000802.hg.1	-2.84	JUC20005678.hg.1	FER1L4
TC20000802.hg.1	-3.08	JUC20005663.hg.1	FER1L4
TC20000802.hg.1	-3.1	JUC20005671.hg.1	FER1L4
TC20000802.hg.1	-3.21	JUC20005677.hg.1	FER1L4
TC20000802.hg.1	-3.33	JUC20005642.hg.1	FER1L4
TC20000802.hg.1	-4.36	PSR20010839.hg.1	FER1L4
TC20000802.hg.1	-5.35	PSR20010812.hg.1	FER1L4
TC20000802.hg.1	-6.08	JUC20005659.hg.1	FER1L4
TC19002651.hg.1	5.1	JUC19017503.hg.1	ZNF765
TC19002651.hg.1	-2	PSR19011361.hg.1	ZNF765
TC19002651.hg.1	-3.56	JUC19017499.hg.1	ZNF765
TC01006324.hg.1	5.09	PSR01013873.hg.1	TMEM56
TC01006324.hg.1	4.51	PSR01013874.hg.1	TMEM56
TC01006324.hg.1	3.68	JUC01044057.hg.1	TMEM56
TC01006324.hg.1	3.27	PSR01013872.hg.1	TMEM56
TC01006324.hg.1	2.64	PSR01013869.hg.1	TMEM56
TC01006324.hg.1	-2.06	JUC01044056.hg.1	TMEM56
TC01006324.hg.1	-2.16	JUC01044054.hg.1	TMEM56
TC01006324.hg.1	-2.27	PSR01013886.hg.1	TMEM56
TC07001065.hg.1	5.09	JUC07007952.hg.1	WDR60
TC07001065.hg.1	2.22	JUC07007944.hg.1	WDR60
TC07001065.hg.1	2.15	PSR07016344.hg.1	WDR60
TC07001552.hg.1	5.09	JUC07011580.hg.1	MAGI2
TC07001552.hg.1	3.45	JUC07011566.hg.1	MAGI2
TC07001552.hg.1	2.64	PSR07023362.hg.1	MAGI2
TC07001552.hg.1	2.55	PSR07023355.hg.1	MAGI2
TC07001552.hg.1	2.45	JUC07011572.hg.1	MAGI2
TC07001552.hg.1	2	PSR07023347.hg.1	MAGI2
TC07001552.hg.1	-2.11	PSR07023380.hg.1	MAGI2
TC07001552.hg.1	-2.12	JUC07011591.hg.1	MAGI2
TC07001552.hg.1	-2.18	JUC07011561.hg.1	MAGI2
TC07001552.hg.1	-2.24	PSR07023342.hg.1	MAGI2
TC07001552.hg.1	-2.25	PSR07023345.hg.1	MAGI2
TC07001552.hg.1	-2.45	JUC07011583.hg.1	MAGI2
TC07001552.hg.1	-2.57	PSR07023370.hg.1	MAGI2
TC07001552.hg.1	-2.87	JUC07011567.hg.1	MAGI2
TC07001552.hg.1	-3.14	JUC07011571.hg.1	MAGI2
TC07001552.hg.1	-3.24	JUC07011562.hg.1	MAGI2
TC07001552.hg.1	-3.48	PSR07023372.hg.1	MAGI2
TC07001552.hg.1	-3.62	JUC07011559.hg.1	MAGI2
TC07001552.hg.1	-3.83	PSR07023382.hg.1	MAGI2
TC07001552.hg.1	-3.84	PSR07023357.hg.1	MAGI2
TC07001552.hg.1	-3.93	PSR07023341.hg.1	MAGI2
TC07001552.hg.1	-4.18	PSR07023338.hg.1	MAGI2
TC07001552.hg.1	-4.22	PSR07023374.hg.1	MAGI2
TC07001552.hg.1	-4.61	PSR07023383.hg.1	MAGI2
TC07001552.hg.1	-4.7	JUC07011576.hg.1	MAGI2
TC07001552.hg.1	-5.61	PSR07023373.hg.1	MAGI2

TC07001552.hg.1	-5.62	PSR07023364.hg.1	MAGI2
TC07001552.hg.1	-6.28	PSR07023376.hg.1	MAGI2
TC07001552.hg.1	-6.82	PSR07023339.hg.1	MAGI2
TC07001552.hg.1	-8.03	JUC07011590.hg.1	MAGI2
TC07001552.hg.1	-8.32	JUC07011558.hg.1	MAGI2
TC04000486.hg.1	5.08	JUC04003631.hg.1	HERC3
TC04000486.hg.1	2.46	JUC04003652.hg.1	HERC3
TC04000486.hg.1	2.02	PSR04007069.hg.1	HERC3
TC01000061.hg.1	5.06	JUC01000474.hg.1	TNFRSF14
TC01000061.hg.1	4.26	JUC01000475.hg.1	TNFRSF14
TC01000061.hg.1	2.12	PSR01000938.hg.1	TNFRSF14
TC01000061.hg.1	-2.53	PSR01000967.hg.1	TNFRSF14
TC01000061.hg.1	-2.9	PSR01000954.hg.1	TNFRSF14
TC01000061.hg.1	-2.9	JUC01000485.hg.1	TNFRSF14
TC01000061.hg.1	-2.98	JUC01000487.hg.1	TNFRSF14
TC01000061.hg.1	-3.12	JUC01000470.hg.1	TNFRSF14
TC01003129.hg.1	5.04	JUC01025970.hg.1	GJA5
TC01003129.hg.1	2.46	PSR01048502.hg.1	GJA5
TC01003129.hg.1	-2.4	PSR01048498.hg.1	GJA5
TC01003129.hg.1	-3.36	PSR01048500.hg.1	GJA5
TC01003129.hg.1	-3.56	PSR01048506.hg.1	GJA5
TC01003129.hg.1	-4.43	PSR01048507.hg.1	GJA5
TC01003129.hg.1	-12.59	PSR01048509.hg.1	GJA5
TC02000286.hg.1	5.04	PSR02004351.hg.1	SOCS5
TC02000286.hg.1	4.42	JUC02002263.hg.1	SOCS5
TC02000286.hg.1	3.13	JUC02002262.hg.1	SOCS5
TC02000286.hg.1	2.85	JUC02002264.hg.1	SOCS5
TC02000286.hg.1	-2.46	PSR02004361.hg.1	SOCS5
TC17000205.hg.1	5.04	JUC17001647.hg.1	MYO15A
TC17000205.hg.1	3.81	JUC17001664.hg.1	MYO15A
TC17000205.hg.1	2.58	PSR17002905.hg.1	MYO15A
TC17000205.hg.1	2	JUC17001658.hg.1	MYO15A
TC17000205.hg.1	-3.47	JUC17001632.hg.1	MYO15A
TC21000506.hg.1	5.04	JUC21003154.hg.1	SIK1
TC21000506.hg.1	4.19	JUC21003155.hg.1	SIK1
TC21000506.hg.1	2.95	PSR21006117.hg.1	SIK1
TC21000506.hg.1	2.87	PSR21006103.hg.1	SIK1
TC21000506.hg.1	2.46	JUC21003161.hg.1	SIK1
TC21000506.hg.1	2.34	JUC21003149.hg.1	SIK1
TC21000506.hg.1	2.05	JUC21003160.hg.1	SIK1
TC21000506.hg.1	2.01	JUC21003151.hg.1	SIK1
TC21000506.hg.1	-2.12	JUC21003157.hg.1	SIK1
TC21000506.hg.1	-2.64	PSR21006121.hg.1	SIK1
TC21000506.hg.1	-4.08	JUC21003159.hg.1	SIK1
TC19000202.hg.1	5.03	JUC19001811.hg.1	CNN1
TC19000202.hg.1	2.5	JUC19001812.hg.1	CNN1
TC19000202.hg.1	2.29	JUC19001810.hg.1	CNN1
TC19000202.hg.1	2.13	PSR19003084.hg.1	CNN1
TC19000202.hg.1	-2.37	PSR19003092.hg.1	CNN1
TC19000202.hg.1	-3.38	PSR19003078.hg.1	CNN1
TC19000202.hg.1	-3.53	JUC19001813.hg.1	CNN1
TC19000202.hg.1	-4.46	PSR19003082.hg.1	CNN1
TC06004141.hg.1	5.02	PSR06028534.hg.1	SOD2, LOC1(
TC06004141.hg.1	4.95	PSR06028535.hg.1	SOD2, LOC1(
TC06004141.hg.1	3.43	PSR06028520.hg.1	SOD2, LOC1(
TC06004141.hg.1	3.31	JUC06021913.hg.1	SOD2, LOC1(
TC06004141.hg.1	2.15	PSR06028524.hg.1	SOD2, LOC1(
TC06004141.hg.1	-2.22	PSR06028521.hg.1	SOD2, LOC1(

TC06004141.hg.1	-2.59	JUC06021917.hg.1	SOD2, LOC1(
TC06004141.hg.1	-3.4	PSR06028507.hg.1	SOD2, LOC1(
TC06004141.hg.1	-3.68	PSR06028522.hg.1	SOD2, LOC1(
TC08000173.hg.1	5.02	JUC08001139.hg.1	SORBS3
TC08000173.hg.1	2.43	JUC08001124.hg.1	SORBS3
TC08000173.hg.1	2.34	PSR08002097.hg.1	SORBS3
TC08000173.hg.1	2.24	PSR08002084.hg.1	SORBS3
TC08000173.hg.1	2.09	PSR08002088.hg.1	SORBS3
TC08000173.hg.1	2.08	PSR08002074.hg.1	SORBS3
TC08000173.hg.1	2.06	PSR08002068.hg.1	SORBS3
TC08000173.hg.1	-3.17	PSR08002091.hg.1	SORBS3
TC14000386.hg.1	5.02	JUC14002216.hg.1	SYNE2
TC14000386.hg.1	4.12	JUC14002207.hg.1	SYNE2
TC14000386.hg.1	4.11	JUC14002176.hg.1	SYNE2
TC14000386.hg.1	3.45	JUC14002204.hg.1	SYNE2
TC14000386.hg.1	3.34	PSR14004252.hg.1	SYNE2
TC14000386.hg.1	3.13	PSR14004346.hg.1	SYNE2
TC14000386.hg.1	3.13	JUC14002133.hg.1	SYNE2
TC14000386.hg.1	2.9	JUC14002106.hg.1	SYNE2
TC14000386.hg.1	2.85	PSR14004267.hg.1	SYNE2
TC14000386.hg.1	2.78	JUC14002197.hg.1	SYNE2
TC14000386.hg.1	2.77	PSR14004264.hg.1	SYNE2
TC14000386.hg.1	2.66	JUC14002102.hg.1	SYNE2
TC14000386.hg.1	2.6	JUC14002123.hg.1	SYNE2
TC14000386.hg.1	2.59	PSR14004244.hg.1	SYNE2
TC14000386.hg.1	2.57	PSR14004249.hg.1	SYNE2
TC14000386.hg.1	2.47	PSR14004303.hg.1	SYNE2
TC14000386.hg.1	2.34	PSR14004277.hg.1	SYNE2
TC14000386.hg.1	2.3	JUC14002136.hg.1	SYNE2
TC14000386.hg.1	2.29	PSR14004300.hg.1	SYNE2
TC14000386.hg.1	2.28	PSR14004263.hg.1	SYNE2
TC14000386.hg.1	2.23	JUC14002157.hg.1	SYNE2
TC14000386.hg.1	2.23	JUC14002166.hg.1	SYNE2
TC14000386.hg.1	2.19	JUC14002215.hg.1	SYNE2
TC14000386.hg.1	2.16	JUC14002221.hg.1	SYNE2
TC14000386.hg.1	2.11	JUC14002131.hg.1	SYNE2
TC14000386.hg.1	2.1	PSR14004261.hg.1	SYNE2
TC14000386.hg.1	2.08	PSR14004284.hg.1	SYNE2
TC14000386.hg.1	2.07	PSR14004253.hg.1	SYNE2
TC14000386.hg.1	2.07	PSR14004254.hg.1	SYNE2
TC14000386.hg.1	2.07	PSR14004366.hg.1	SYNE2
TC14000386.hg.1	2.07	JUC14002206.hg.1	SYNE2
TC14000386.hg.1	2.06	PSR14004257.hg.1	SYNE2
TC14000386.hg.1	2.06	PSR14004360.hg.1	SYNE2
TC14000386.hg.1	2.06	JUC14002139.hg.1	SYNE2
TC14000386.hg.1	2.04	PSR14004358.hg.1	SYNE2
TC14000386.hg.1	2.03	PSR14004308.hg.1	SYNE2
TC14000386.hg.1	2.02	PSR14004281.hg.1	SYNE2
TC14000386.hg.1	2.02	PSR14004285.hg.1	SYNE2
TC14000386.hg.1	2.02	PSR14004374.hg.1	SYNE2
TC14000386.hg.1	2.02	JUC14002146.hg.1	SYNE2
TC14000386.hg.1	2.01	PSR14004238.hg.1	SYNE2
TC14000386.hg.1	2	PSR14004234.hg.1	SYNE2
TC14000386.hg.1	2	PSR14004302.hg.1	SYNE2
TC14000386.hg.1	-2	PSR14004376.hg.1	SYNE2
TC14000386.hg.1	-2.01	PSR14004370.hg.1	SYNE2
TC14000386.hg.1	-2.02	JUC14002103.hg.1	SYNE2
TC14000386.hg.1	-2.04	PSR14004314.hg.1	SYNE2

TC14000386.hg.1	-2.05	JUC14002100.hg.1	SYNE2
TC14000386.hg.1	-2.07	JUC14002116.hg.1	SYNE2
TC14000386.hg.1	-2.1	JUC14002198.hg.1	SYNE2
TC14000386.hg.1	-2.17	PSR14004342.hg.1	SYNE2
TC14000386.hg.1	-2.17	JUC14002111.hg.1	SYNE2
TC14000386.hg.1	-2.17	JUC14002203.hg.1	SYNE2
TC14000386.hg.1	-2.27	PSR14004316.hg.1	SYNE2
TC14000386.hg.1	-2.29	PSR14004325.hg.1	SYNE2
TC14000386.hg.1	-2.31	JUC14002138.hg.1	SYNE2
TC14000386.hg.1	-2.34	JUC14002147.hg.1	SYNE2
TC14000386.hg.1	-2.52	JUC14002155.hg.1	SYNE2
TC14000386.hg.1	-2.57	JUC14002088.hg.1	SYNE2
TC14000386.hg.1	-3.01	JUC14002097.hg.1	SYNE2
TC14000386.hg.1	-3.02	PSR14004313.hg.1	SYNE2
TC14000386.hg.1	-3.04	PSR14004330.hg.1	SYNE2
TC14000386.hg.1	-3.33	JUC14002137.hg.1	SYNE2
TC14000386.hg.1	-4.36	JUC14002119.hg.1	SYNE2
TC01003353.hg.1	4.98	JUC01027412.hg.1	HDGF
TC01003353.hg.1	2.38	PSR01052377.hg.1	HDGF
TC01003353.hg.1	2.28	JUC01027417.hg.1	HDGF
TC06001578.hg.1	4.98	JUC06008987.hg.1	HLA-DPA1
TC06001578.hg.1	3.61	JUC06008978.hg.1	HLA-DPA1
TC06001578.hg.1	2.77	PSR06018909.hg.1	HLA-DPA1
TC06001578.hg.1	2.39	PSR06018922.hg.1	HLA-DPA1
TC06001578.hg.1	2.08	PSR06018907.hg.1	HLA-DPA1
TC06001578.hg.1	2.07	PSR06018921.hg.1	HLA-DPA1
TC06001578.hg.1	2.02	PSR06018905.hg.1	HLA-DPA1
TC06001578.hg.1	-2.07	JUC06008979.hg.1	HLA-DPA1
TC06001578.hg.1	-2.08	JUC06008984.hg.1	HLA-DPA1
TC06001578.hg.1	-2.18	PSR06018929.hg.1	HLA-DPA1
TC06001578.hg.1	-2.49	PSR06018934.hg.1	HLA-DPA1
TC06001578.hg.1	-4.66	JUC06008989.hg.1	HLA-DPA1
TC10001187.hg.1	4.98	JUC10008625.hg.1	ZNF248
TC10001187.hg.1	2.98	PSR10014900.hg.1	ZNF248
TC10001187.hg.1	2.48	PSR10014875.hg.1	ZNF248
TC6_cox_hap2000	4.98	JUC6_cox_hap2001	HLA-DPA1
TC6_cox_hap2000	3.61	JUC6_cox_hap2001	HLA-DPA1
TC6_cox_hap2000	2.77	PSR6_cox_hap2004	HLA-DPA1
TC6_cox_hap2000	2.39	PSR6_cox_hap2004	HLA-DPA1
TC6_cox_hap2000	2.08	PSR6_cox_hap2004	HLA-DPA1
TC6_cox_hap2000	2.07	PSR6_cox_hap2004	HLA-DPA1
TC6_cox_hap2000	2.02	PSR6_cox_hap2004	HLA-DPA1
TC6_cox_hap2000	-2.08	JUC6_cox_hap2001	HLA-DPA1
TC6_cox_hap2000	-2.18	PSR6_cox_hap2004	HLA-DPA1
TC6_cox_hap2000	-2.62	PSR6_cox_hap2004	HLA-DPA1
TC6_cox_hap2000	-4.66	JUC6_cox_hap2001	HLA-DPA1
TC0X001549.hg.1	4.98	JUC0X010381.hg.1	MTCP1NB, M
TC0X001549.hg.1	2.96	JUC0X010377.hg.1	MTCP1NB, M
TC0X001549.hg.1	2.19	JUC0X010378.hg.1	MTCP1NB, M
TC0X001549.hg.1	2	PSR0X020786.hg.1	MTCP1NB, M
TC06001002.hg.1	4.97	JUC06005742.hg.1	TBPL1
TC06001002.hg.1	2.83	JUC06005738.hg.1	TBPL1
TC06001002.hg.1	2.61	PSR06011841.hg.1	TBPL1
TC10000228.hg.1	4.97	JUC10001434.hg.1	CCDC7, C10
TC10000228.hg.1	4.2	JUC10001393.hg.1	CCDC7, C10
TC10000228.hg.1	3.98	JUC10001402.hg.1	CCDC7, C10
TC10000228.hg.1	3.95	JUC10001396.hg.1	CCDC7, C10
TC10000228.hg.1	3.6	PSR10002639.hg.1	CCDC7, C10

TC10000228.hg.1	3.4	JUC10001412.hg.1	CCDC7, C10d
TC10000228.hg.1	3.21	PSR10002635.hg.1	CCDC7, C10d
TC10000228.hg.1	3.16	PSR10002634.hg.1	CCDC7, C10d
TC10000228.hg.1	3.16	JUC10001450.hg.1	CCDC7, C10d
TC10000228.hg.1	3.05	PSR10002637.hg.1	CCDC7, C10d
TC10000228.hg.1	3.01	PSR10002640.hg.1	CCDC7, C10d
TC10000228.hg.1	2.96	PSR10002570.hg.1	CCDC7, C10d
TC10000228.hg.1	2.94	PSR10002617.hg.1	CCDC7, C10d
TC10000228.hg.1	2.93	PSR10002643.hg.1	CCDC7, C10d
TC10000228.hg.1	2.88	PSR10002632.hg.1	CCDC7, C10d
TC10000228.hg.1	2.87	JUC10001409.hg.1	CCDC7, C10d
TC10000228.hg.1	2.86	PSR10002633.hg.1	CCDC7, C10d
TC10000228.hg.1	2.83	PSR10002590.hg.1	CCDC7, C10d
TC10000228.hg.1	2.83	PSR10002592.hg.1	CCDC7, C10d
TC10000228.hg.1	2.7	JUC10001448.hg.1	CCDC7, C10d
TC10000228.hg.1	2.65	PSR10002628.hg.1	CCDC7, C10d
TC10000228.hg.1	2.63	PSR10002644.hg.1	CCDC7, C10d
TC10000228.hg.1	2.61	PSR10002579.hg.1	CCDC7, C10d
TC10000228.hg.1	2.56	JUC10001392.hg.1	CCDC7, C10d
TC10000228.hg.1	2.5	PSR10002631.hg.1	CCDC7, C10d
TC10000228.hg.1	2.45	PSR10002621.hg.1	CCDC7, C10d
TC10000228.hg.1	2.44	JUC10001446.hg.1	CCDC7, C10d
TC10000228.hg.1	2.41	PSR10002641.hg.1	CCDC7, C10d
TC10000228.hg.1	2.38	PSR10002646.hg.1	CCDC7, C10d
TC10000228.hg.1	2.35	JUC10001437.hg.1	CCDC7, C10d
TC10000228.hg.1	2.33	PSR10002624.hg.1	CCDC7, C10d
TC10000228.hg.1	2.32	PSR10002645.hg.1	CCDC7, C10d
TC10000228.hg.1	2.31	JUC10001452.hg.1	CCDC7, C10d
TC10000228.hg.1	2.3	PSR10002618.hg.1	CCDC7, C10d
TC10000228.hg.1	2.29	PSR10002642.hg.1	CCDC7, C10d
TC10000228.hg.1	2.25	PSR10002601.hg.1	CCDC7, C10d
TC10000228.hg.1	2.24	PSR10002576.hg.1	CCDC7, C10d
TC10000228.hg.1	2.21	PSR10002574.hg.1	CCDC7, C10d
TC10000228.hg.1	2.13	PSR10002630.hg.1	CCDC7, C10d
TC10000228.hg.1	2.11	JUC10001403.hg.1	CCDC7, C10d
TC10000228.hg.1	2.09	PSR10002607.hg.1	CCDC7, C10d
TC10000228.hg.1	2.08	PSR10002604.hg.1	CCDC7, C10d
TC10000228.hg.1	2.08	JUC10001416.hg.1	CCDC7, C10d
TC10000228.hg.1	2.07	PSR10002578.hg.1	CCDC7, C10d
TC10000228.hg.1	2.06	PSR10002599.hg.1	CCDC7, C10d
TC10000228.hg.1	2.02	PSR10002638.hg.1	CCDC7, C10d
TC10000228.hg.1	2	JUC10001406.hg.1	CCDC7, C10d
TC17000648.hg.1	4.97	JUC17004477.hg.1	KAT7
TC17000648.hg.1	4.68	JUC17004469.hg.1	KAT7
TC17000648.hg.1	2.26	PSR17008056.hg.1	KAT7
TC17000648.hg.1	2.21	JUC17004481.hg.1	KAT7
TC17000648.hg.1	2.2	JUC17004472.hg.1	KAT7
TC17000648.hg.1	2.12	JUC17004475.hg.1	KAT7
TC17000648.hg.1	-2.32	JUC17004480.hg.1	KAT7
TC20000621.hg.1	4.97	JUC20004621.hg.1	JAG1
TC20000621.hg.1	4.94	PSR20008940.hg.1	JAG1
TC20000621.hg.1	4.3	PSR20008935.hg.1	JAG1
TC20000621.hg.1	3.41	PSR20008919.hg.1	JAG1
TC20000621.hg.1	2.2	JUC20004606.hg.1	JAG1
TC20000621.hg.1	-2.05	PSR20008899.hg.1	JAG1
TC20000621.hg.1	-2.42	PSR20008900.hg.1	JAG1
TC20000621.hg.1	-2.9	JUC20004618.hg.1	JAG1
TC20000621.hg.1	-3.59	JUC20004619.hg.1	JAG1

TC20000621.hg.1	-5.57	JUC20004615.hg.1	JAG1
TC05002138.hg.1	4.96	JUC05015254.hg.1	MGAT4B
TC05002138.hg.1	3.74	JUC05015246.hg.1	MGAT4B
TC05002138.hg.1	3.61	PSR05029902.hg.1	MGAT4B
TC05002138.hg.1	3.07	PSR05029925.hg.1	MGAT4B
TC05002138.hg.1	2.97	PSR05029928.hg.1	MGAT4B
TC05002138.hg.1	2.89	PSR05029881.hg.1	MGAT4B
TC05002138.hg.1	2.82	PSR05029893.hg.1	MGAT4B
TC05002138.hg.1	2.69	PSR05029935.hg.1	MGAT4B
TC05002138.hg.1	2.68	JUC05015256.hg.1	MGAT4B
TC05002138.hg.1	2.62	PSR05029913.hg.1	MGAT4B
TC05002138.hg.1	2.51	PSR05029932.hg.1	MGAT4B
TC05002138.hg.1	2.49	PSR05029918.hg.1	MGAT4B
TC05002138.hg.1	2.26	PSR05029883.hg.1	MGAT4B
TC05002138.hg.1	2.23	PSR05029931.hg.1	MGAT4B
TC05002138.hg.1	2.16	PSR05029941.hg.1	MGAT4B
TC05002138.hg.1	2.04	PSR05029927.hg.1	MGAT4B
TC07000125.hg.1	4.96	JUC07000885.hg.1	ITGB8
TC07000125.hg.1	4.58	JUC07000877.hg.1	ITGB8
TC07000125.hg.1	4.39	PSR07001904.hg.1	ITGB8
TC07000125.hg.1	4.23	PSR07001906.hg.1	ITGB8
TC07000125.hg.1	4.16	PSR07001902.hg.1	ITGB8
TC07000125.hg.1	3.66	PSR07001907.hg.1	ITGB8
TC07000125.hg.1	3.43	JUC07000886.hg.1	ITGB8
TC07000125.hg.1	3.27	PSR07001914.hg.1	ITGB8
TC07000125.hg.1	3.19	JUC07000879.hg.1	ITGB8
TC07000125.hg.1	3.13	PSR07001912.hg.1	ITGB8
TC07000125.hg.1	2.74	PSR07001905.hg.1	ITGB8
TC07000125.hg.1	2.42	PSR07001909.hg.1	ITGB8
TC07000125.hg.1	2.28	PSR07001923.hg.1	ITGB8
TC07000125.hg.1	2.11	JUC07000881.hg.1	ITGB8
TC07000125.hg.1	-2.05	JUC07000890.hg.1	ITGB8
TC07000125.hg.1	-2.08	PSR07001927.hg.1	ITGB8
TC07000125.hg.1	-2.32	PSR07001910.hg.1	ITGB8
TC07000125.hg.1	-2.32	JUC07000883.hg.1	ITGB8
TC07000125.hg.1	-2.5	JUC07000884.hg.1	ITGB8
TC07000125.hg.1	-2.75	JUC07000882.hg.1	ITGB8
TC07000125.hg.1	-3.74	JUC07000889.hg.1	ITGB8
TC12001978.hg.1	4.96	JUC12014468.hg.1	C12orf51
TC12001978.hg.1	2.47	JUC12014459.hg.1	C12orf51
TC12001978.hg.1	2.2	JUC12014471.hg.1	C12orf51
TC12001978.hg.1	2.16	JUC12014480.hg.1	C12orf51
TC12001978.hg.1	2.01	PSR12025898.hg.1	C12orf51
TC12001978.hg.1	-3.78	JUC12014485.hg.1	C12orf51
TC20000264.hg.1	4.95	JUC20002075.hg.1	EPB41L1
TC20000264.hg.1	4.4	JUC20002037.hg.1	EPB41L1
TC20000264.hg.1	3.71	JUC20002076.hg.1	EPB41L1
TC20000264.hg.1	3.41	JUC20002059.hg.1	EPB41L1
TC20000264.hg.1	2.55	PSR20003585.hg.1	EPB41L1
TC20000264.hg.1	2.5	PSR20003595.hg.1	EPB41L1
TC20000264.hg.1	2.36	JUC20002038.hg.1	EPB41L1
TC20000264.hg.1	2.35	JUC20002032.hg.1	EPB41L1
TC20000264.hg.1	2.25	JUC20002074.hg.1	EPB41L1
TC20000264.hg.1	2.22	JUC20002054.hg.1	EPB41L1
TC20000264.hg.1	2.12	PSR20003592.hg.1	EPB41L1
TC20000264.hg.1	2.1	PSR20003597.hg.1	EPB41L1
TC20000264.hg.1	2.08	JUC20002021.hg.1	EPB41L1
TC20000264.hg.1	2.07	JUC20002069.hg.1	EPB41L1

TC20000264.hg.1	2.01	PSR20003591.hg.1	EPB41L1
TC20000264.hg.1	2.01	JUC20002072.hg.1	EPB41L1
TC20000264.hg.1	2	PSR20003598.hg.1	EPB41L1
TC09000417.hg.1	4.92	JUC09002436.hg.1	SECISBP2
TC09000417.hg.1	2.77	JUC09002432.hg.1	SECISBP2
TC09000417.hg.1	2.37	PSR09004444.hg.1	SECISBP2
TC09000417.hg.1	2.12	PSR09004446.hg.1	SECISBP2
TC09000417.hg.1	2.09	JUC09002424.hg.1	SECISBP2
TC09000417.hg.1	2	JUC09002443.hg.1	SECISBP2
TC06000979.hg.1	4.91	JUC06005564.hg.1	TMEM200A
TC06000979.hg.1	3.71	JUC06005565.hg.1	TMEM200A
TC06000979.hg.1	3.58	PSR06011526.hg.1	TMEM200A
TC06000979.hg.1	-2.11	PSR06011521.hg.1	TMEM200A
TC06000979.hg.1	-3.09	PSR06011518.hg.1	TMEM200A
TC06000979.hg.1	-3.26	PSR06011519.hg.1	TMEM200A
TC06001027.hg.1	4.91	PSR06012002.hg.1	TNFAIP3
TC06001027.hg.1	3.73	PSR06012003.hg.1	TNFAIP3
TC06001027.hg.1	3.72	JUC06005857.hg.1	TNFAIP3
TC06001027.hg.1	3.15	JUC06005858.hg.1	TNFAIP3
TC06001027.hg.1	2.88	PSR06012008.hg.1	TNFAIP3
TC06001027.hg.1	2.87	JUC06005848.hg.1	TNFAIP3
TC06001027.hg.1	2.55	JUC06005856.hg.1	TNFAIP3
TC06001027.hg.1	2.01	PSR06012020.hg.1	TNFAIP3
TC07000018.hg.1	4.91	PSR07000251.hg.1	GPER
TC07000018.hg.1	3.31	PSR07000252.hg.1	GPER
TC07000018.hg.1	3.23	PSR07000270.hg.1	GPER
TC07000018.hg.1	2.76	PSR07000264.hg.1	GPER
TC07000018.hg.1	2.69	PSR07000254.hg.1	GPER
TC07000018.hg.1	2.42	PSR07000268.hg.1	GPER
TC07000018.hg.1	2.3	JUC07000071.hg.1	GPER
TC07000018.hg.1	2.22	JUC07000070.hg.1	GPER
TC07000018.hg.1	2.19	JUC07000074.hg.1	GPER
TC07000018.hg.1	2.12	PSR07000253.hg.1	GPER
TC07000018.hg.1	2.02	PSR07000269.hg.1	GPER
TC07000018.hg.1	-3.05	JUC07000072.hg.1	GPER
TC0X000261.hg.1	4.91	PSR0X003327.hg.1	HDAC6
TC0X000261.hg.1	3.67	JUC0X001727.hg.1	HDAC6
TC0X000261.hg.1	3.3	PSR0X003311.hg.1	HDAC6
TC0X000261.hg.1	2.34	PSR0X003350.hg.1	HDAC6
TC0X000261.hg.1	2.23	PSR0X003335.hg.1	HDAC6
TC0X000261.hg.1	2.22	PSR0X003346.hg.1	HDAC6
TC0X000261.hg.1	2.2	PSR0X003250.hg.1	HDAC6
TC0X000261.hg.1	2.11	PSR0X003299.hg.1	HDAC6
TC0X000261.hg.1	2.04	PSR0X003249.hg.1	HDAC6
TC0X000261.hg.1	2.01	PSR0X003300.hg.1	HDAC6
TC0X000261.hg.1	-2.12	JUC0X001737.hg.1	HDAC6
TC0X000261.hg.1	-2.14	PSR0X003279.hg.1	HDAC6
TC01000580.hg.1	4.9	JUC01005062.hg.1	TSPAN1
TC01000580.hg.1	3.19	JUC01005054.hg.1	TSPAN1
TC01000580.hg.1	3.14	JUC01005057.hg.1	TSPAN1
TC01000580.hg.1	2.2	PSR01009873.hg.1	TSPAN1
TC01000580.hg.1	2.16	JUC01005056.hg.1	TSPAN1
TC01000580.hg.1	2.09	PSR01009872.hg.1	TSPAN1
TC01000580.hg.1	-2.12	PSR01009868.hg.1	TSPAN1
TC01001966.hg.1	4.9	JUC01016573.hg.1	FMN2
TC01001966.hg.1	3.76	JUC01016559.hg.1	FMN2
TC01001966.hg.1	3.42	PSR01030490.hg.1	FMN2
TC01001966.hg.1	3.11	PSR01030479.hg.1	FMN2

TC01001966.hg.1	3.08	JUC01016586.hg.1	FMN2
TC01001966.hg.1	3.03	PSR01030512.hg.1	FMN2
TC01001966.hg.1	3	PSR01030482.hg.1	FMN2
TC01001966.hg.1	2.83	PSR01030492.hg.1	FMN2
TC01001966.hg.1	2.73	JUC01016571.hg.1	FMN2
TC01001966.hg.1	2.7	JUC01016560.hg.1	FMN2
TC01001966.hg.1	2.63	JUC01016580.hg.1	FMN2
TC01001966.hg.1	2.52	PSR01030503.hg.1	FMN2
TC01001966.hg.1	2.49	JUC01016579.hg.1	FMN2
TC01001966.hg.1	2.42	JUC01016577.hg.1	FMN2
TC01001966.hg.1	2.27	PSR01030496.hg.1	FMN2
TC01001966.hg.1	2.22	PSR01030493.hg.1	FMN2
TC01001966.hg.1	-2.28	JUC01016587.hg.1	FMN2
TC01001966.hg.1	-4.54	JUC01016568.hg.1	FMN2
TC03001071.hg.1	4.9	PSR03018702.hg.1	HES1
TC03001071.hg.1	4.68	PSR03018708.hg.1	HES1
TC03001071.hg.1	4.55	PSR03018710.hg.1	HES1
TC03001071.hg.1	4.23	JUC03009497.hg.1	HES1
TC03001071.hg.1	-2.35	PSR03018706.hg.1	HES1
TC03001071.hg.1	-3.28	JUC03009494.hg.1	HES1
TC03001071.hg.1	-3.88	JUC03009495.hg.1	HES1
TC02001279.hg.1	4.89	JUC02010542.hg.1	RPL37A
TC02001279.hg.1	2.14	PSR02019567.hg.1	RPL37A
TC02001279.hg.1	-2.17	JUC02010540.hg.1	RPL37A
TC02001279.hg.1	-2.77	PSR02019590.hg.1	RPL37A
TC02001279.hg.1	-3.97	JUC02010545.hg.1	RPL37A
TC02002637.hg.1	4.89	JUC02022087.hg.1	STK17B
TC02002637.hg.1	4.12	PSR02042265.hg.1	STK17B
TC02002637.hg.1	3.26	PSR02042267.hg.1	STK17B
TC02002637.hg.1	2.46	PSR02042266.hg.1	STK17B
TC02002637.hg.1	2.38	PSR02042260.hg.1	STK17B
TC02002637.hg.1	-2.54	PSR02042249.hg.1	STK17B
TC16000193.hg.1	4.89	JUC16001502.hg.1	ABCC1
TC16000193.hg.1	3.35	PSR16002869.hg.1	ABCC1
TC16000193.hg.1	3.11	JUC16001504.hg.1	ABCC1
TC16000193.hg.1	2.72	JUC16001495.hg.1	ABCC1
TC16000193.hg.1	2.47	JUC16001498.hg.1	ABCC1
TC16000193.hg.1	2.43	PSR16002884.hg.1	ABCC1
TC16000193.hg.1	2.22	JUC16001483.hg.1	ABCC1
TC16000193.hg.1	2.22	JUC16001506.hg.1	ABCC1
TC16000193.hg.1	2.05	PSR16002860.hg.1	ABCC1
TC16000193.hg.1	-2.68	JUC16001480.hg.1	ABCC1
TC16000193.hg.1	-3.29	JUC16001505.hg.1	ABCC1
TC09000201.hg.1	4.88	JUC09001222.hg.1	RECK
TC09000201.hg.1	-2.87	PSR09002290.hg.1	RECK
TC09000201.hg.1	-2.99	JUC09001216.hg.1	RECK
TC09000201.hg.1	-3.67	JUC09001232.hg.1	RECK
TC09000005.hg.1	4.87	JUC09000079.hg.1	KANK1
TC09000005.hg.1	2.08	JUC09000081.hg.1	KANK1
TC09000005.hg.1	2.06	JUC09000076.hg.1	KANK1
TC09000005.hg.1	-2.04	PSR09000140.hg.1	KANK1
TC09000005.hg.1	-2.21	JUC09000084.hg.1	KANK1
TC09000005.hg.1	-2.23	JUC09000090.hg.1	KANK1
TC09000005.hg.1	-2.42	PSR09000131.hg.1	KANK1
TC09000005.hg.1	-2.43	JUC09000098.hg.1	KANK1
TC09000005.hg.1	-2.63	PSR09000123.hg.1	KANK1
TC09000005.hg.1	-3.42	JUC09000077.hg.1	KANK1
TC09000005.hg.1	-6.44	JUC09000089.hg.1	KANK1

TC20000108.hg.1	4.87	PSR20001425.hg.1	PCSK2
TC20000108.hg.1	3.72	PSR20001427.hg.1	PCSK2
TC20000108.hg.1	3.32	PSR20001431.hg.1	PCSK2
TC20000108.hg.1	3.01	JUC20000749.hg.1	PCSK2
TC20000108.hg.1	-2.13	JUC20000742.hg.1	PCSK2
TC20000108.hg.1	-2.27	JUC20000745.hg.1	PCSK2
TC01001112.hg.1	4.86	JUC01009670.hg.1	CHD1L
TC01001112.hg.1	4.67	JUC01009657.hg.1	CHD1L
TC01001112.hg.1	2.61	JUC01009667.hg.1	CHD1L
TC01001112.hg.1	2.59	PSR01017585.hg.1	CHD1L
TC01001112.hg.1	2.26	PSR01017558.hg.1	CHD1L
TC01001112.hg.1	2.24	JUC01009655.hg.1	CHD1L
TC01001112.hg.1	2.12	JUC01009672.hg.1	CHD1L
TC01001112.hg.1	-2.46	JUC01009689.hg.1	CHD1L
TC01001112.hg.1	-2.58	JUC01009665.hg.1	CHD1L
TC02001874.hg.1	4.85	PSR02029944.hg.1	LOC1001294
TC02001874.hg.1	3.11	PSR02029943.hg.1	LOC1001294
TC02001874.hg.1	2.44	JUC02015733.hg.1	LOC1001294
TC02001874.hg.1	2.23	PSR02029940.hg.1	LOC1001294
TC02002541.hg.1	4.85	JUC02021260.hg.1	WIPF1
TC02002541.hg.1	4.67	JUC02021276.hg.1	WIPF1
TC02002541.hg.1	3.64	JUC02021256.hg.1	WIPF1
TC02002541.hg.1	2.16	JUC02021282.hg.1	WIPF1
TC02002541.hg.1	2.1	JUC02021264.hg.1	WIPF1
TC02002541.hg.1	2.05	PSR02040354.hg.1	WIPF1
TC02002541.hg.1	-3.66	PSR02040364.hg.1	WIPF1
TC02002823.hg.1	4.85	PSR02045569.hg.1	SERPINE2
TC02002823.hg.1	4.73	PSR02045571.hg.1	SERPINE2
TC02002823.hg.1	4.58	JUC02023773.hg.1	SERPINE2
TC02002823.hg.1	4.23	PSR02045565.hg.1	SERPINE2
TC02002823.hg.1	4.05	JUC02023781.hg.1	SERPINE2
TC02002823.hg.1	3.57	PSR02045559.hg.1	SERPINE2
TC02002823.hg.1	3.29	PSR02045566.hg.1	SERPINE2
TC02002823.hg.1	3.22	PSR02045575.hg.1	SERPINE2
TC02002823.hg.1	3.04	PSR02045535.hg.1	SERPINE2
TC02002823.hg.1	2.89	PSR02045563.hg.1	SERPINE2
TC02002823.hg.1	2.53	PSR02045567.hg.1	SERPINE2
TC02002823.hg.1	2.22	JUC02023780.hg.1	SERPINE2
TC02002823.hg.1	2.2	JUC02023784.hg.1	SERPINE2
TC02002823.hg.1	2.06	PSR02045570.hg.1	SERPINE2
TC02002823.hg.1	2	JUC02023769.hg.1	SERPINE2
TC02002823.hg.1	-2.83	JUC02023788.hg.1	SERPINE2
TC07001895.hg.1	4.85	PSR07029420.hg.1	PTN, LOC10(
TC07001895.hg.1	2.99	PSR07029413.hg.1	PTN, LOC10(
TC07001895.hg.1	2.12	PSR07029417.hg.1	PTN, LOC10(
TC07001895.hg.1	-2.5	PSR07029414.hg.1	PTN, LOC10(
TC07001895.hg.1	-2.63	PSR07029416.hg.1	PTN, LOC10(
TC07001895.hg.1	-3.28	JUC07014519.hg.1	PTN, LOC10(
TC07001895.hg.1	-3.41	JUC07014518.hg.1	PTN, LOC10(
TC04001014.hg.1	4.84	JUC04007430.hg.1	AFAP1
TC04001014.hg.1	2.68	JUC04007434.hg.1	AFAP1
TC04001014.hg.1	2.46	JUC04007422.hg.1	AFAP1
TC04001014.hg.1	2.34	PSR04013943.hg.1	AFAP1
TC04001014.hg.1	-2.03	PSR04013969.hg.1	AFAP1
TC04001014.hg.1	-2.34	JUC04007418.hg.1	AFAP1
TC11000063.hg.1	4.84	JUC11000604.hg.1	CD81
TC11000063.hg.1	2.3	PSR11001200.hg.1	CD81
TC02002428.hg.1	4.82	JUC02019898.hg.1	NEB

TC02002428.hg.1	3.09	JUC02019999.hg.1	NEB
TC02002428.hg.1	3.09	JUC02020002.hg.1	NEB
TC02002428.hg.1	3.09	JUC02020036.hg.1	NEB
TC02002428.hg.1	2.99	JUC02019963.hg.1	NEB
TC02002428.hg.1	2.99	JUC02019966.hg.1	NEB
TC02002428.hg.1	2.99	JUC02019968.hg.1	NEB
TC02002428.hg.1	2.69	PSR02038241.hg.1	NEB
TC02002428.hg.1	2.45	JUC02020029.hg.1	NEB
TC02002428.hg.1	2.14	PSR02038097.hg.1	NEB
TC02002428.hg.1	2.09	PSR02038202.hg.1	NEB
TC02002428.hg.1	2.05	PSR02038245.hg.1	NEB
TC02002428.hg.1	2.03	JUC02019943.hg.1	NEB
TC02002428.hg.1	2.03	JUC02019946.hg.1	NEB
TC02002428.hg.1	2.03	JUC02019948.hg.1	NEB
TC02002428.hg.1	-2.04	JUC02019902.hg.1	NEB
TC02002428.hg.1	-2.47	PSR02038296.hg.1	NEB
TC13000678.hg.1	4.82	JUC13004567.hg.1	INTS6
TC13000678.hg.1	2.33	PSR13007854.hg.1	INTS6
TC13000678.hg.1	-2.16	JUC13004562.hg.1	INTS6
TC06001915.hg.1	4.8	JUC06011405.hg.1	KIAA1009
TC06001915.hg.1	2.14	JUC06011431.hg.1	KIAA1009
TC06001915.hg.1	2.13	JUC06011426.hg.1	KIAA1009
TC06001915.hg.1	2.09	JUC06011423.hg.1	KIAA1009
TC06001915.hg.1	2.05	JUC06011416.hg.1	KIAA1009
TC06001915.hg.1	2.03	JUC06011435.hg.1	KIAA1009
TC06001915.hg.1	-2.14	PSR06023409.hg.1	KIAA1009
TC06001915.hg.1	-2.16	JUC06011434.hg.1	KIAA1009
TC13001722.hg.1	4.8	JUC13008429.hg.1	N4BP2L1
TC13001722.hg.1	4.61	PSR13006172.hg.1	N4BP2L1
TC13001722.hg.1	4.16	JUC13008432.hg.1	N4BP2L1
TC13001722.hg.1	3.75	JUC13008437.hg.1	N4BP2L1
TC13001722.hg.1	3.16	PSR13006168.hg.1	N4BP2L1
TC13001722.hg.1	2.95	PSR13006165.hg.1	N4BP2L1
TC13001722.hg.1	2.94	PSR13006159.hg.1	N4BP2L1
TC13001722.hg.1	2.26	PSR13006167.hg.1	N4BP2L1
TC13001722.hg.1	2.15	PSR13006166.hg.1	N4BP2L1
TC13001722.hg.1	2.09	PSR13006171.hg.1	N4BP2L1
TC13001722.hg.1	2.06	PSR13006155.hg.1	N4BP2L1
TC13001722.hg.1	2.05	PSR13006161.hg.1	N4BP2L1
TC13001722.hg.1	-2.06	JUC13008431.hg.1	N4BP2L1
TC13001722.hg.1	-2.31	PSR13006162.hg.1	N4BP2L1
TC13001722.hg.1	-2.67	PSR13006154.hg.1	N4BP2L1
TC13001722.hg.1	-3.15	JUC13008428.hg.1	N4BP2L1
TC13001722.hg.1	-3.81	JUC13008427.hg.1	N4BP2L1
TC13001722.hg.1	-3.94	JUC13008435.hg.1	N4BP2L1
TC14002310.hg.1	4.8	PSR14007438.hg.1	BDKRB2
TC14002310.hg.1	4.61	PSR14007440.hg.1	BDKRB2
TC14002310.hg.1	3.22	PSR14007448.hg.1	BDKRB2
TC14002310.hg.1	3.15	PSR14007447.hg.1	BDKRB2
TC14002310.hg.1	2.99	PSR14007442.hg.1	BDKRB2
TC14002310.hg.1	2.67	PSR14007451.hg.1	BDKRB2
TC14002310.hg.1	2.56	JUC14011897.hg.1	BDKRB2
TC20000357.hg.1	4.8	JUC20002778.hg.1	UBE2C
TC20000357.hg.1	4.32	PSR20005117.hg.1	UBE2C
TC20000357.hg.1	4.06	PSR20005096.hg.1	UBE2C
TC20000357.hg.1	3.07	PSR20005105.hg.1	UBE2C
TC20000357.hg.1	2.69	PSR20005116.hg.1	UBE2C
TC20000357.hg.1	2.45	PSR20005104.hg.1	UBE2C

TC20000357.hg.1	2.31	PSR20005103.hg.1	UBE2C
TC20000357.hg.1	2.31	JUC20002775.hg.1	UBE2C
TC20000357.hg.1	2.06	PSR20005115.hg.1	UBE2C
TC17000532.hg.1	4.79	JUC17003619.hg.1	TUBG2
TC17000532.hg.1	3.45	PSR17006447.hg.1	TUBG2
TC17000532.hg.1	3.21	PSR17006450.hg.1	TUBG2
TC17000532.hg.1	2.13	JUC17003621.hg.1	TUBG2
TC17000532.hg.1	-2.25	JUC17003611.hg.1	TUBG2
TC03001670.hg.1	4.78	JUC03014949.hg.1	ZBTB20
TC03001670.hg.1	4.36	JUC03014951.hg.1	ZBTB20
TC03001670.hg.1	4.05	JUC03014966.hg.1	ZBTB20
TC03001670.hg.1	3.86	JUC03014963.hg.1	ZBTB20
TC03001670.hg.1	3.22	JUC03014983.hg.1	ZBTB20
TC03001670.hg.1	3.19	PSR03030160.hg.1	ZBTB20
TC03001670.hg.1	2.65	JUC03014962.hg.1	ZBTB20
TC03001670.hg.1	2.49	JUC03014981.hg.1	ZBTB20
TC03001670.hg.1	2.4	JUC03014982.hg.1	ZBTB20
TC03001670.hg.1	2.24	JUC03014970.hg.1	ZBTB20
TC03001670.hg.1	2.1	PSR03030162.hg.1	ZBTB20
TC03001670.hg.1	2.07	PSR03030156.hg.1	ZBTB20
TC03001670.hg.1	2.04	PSR03030144.hg.1	ZBTB20
TC03001670.hg.1	2.04	PSR03030164.hg.1	ZBTB20
TC03001670.hg.1	-2.8	JUC03014964.hg.1	ZBTB20
TC02001483.hg.1	4.77	PSR02024188.hg.1	ATG4B
TC02001483.hg.1	-2.35	PSR02024150.hg.1	ATG4B
TC02001483.hg.1	-2.62	JUC02012566.hg.1	ATG4B
TC03003317.hg.1	4.77	JUC03023019.hg.1	P4HTM
TC03003317.hg.1	3.56	JUC03023023.hg.1	P4HTM
TC03003317.hg.1	2.53	JUC03023016.hg.1	P4HTM
TC03003317.hg.1	2.15	PSR03005808.hg.1	P4HTM
TC03003317.hg.1	2.14	PSR03005793.hg.1	P4HTM
TC03003317.hg.1	2.08	PSR03005794.hg.1	P4HTM
TC03003317.hg.1	2.07	PSR03005797.hg.1	P4HTM
TC06000991.hg.1	4.77	PSR06011703.hg.1	LOC1005072
TC06000991.hg.1	3.56	JUC06005670.hg.1	LOC1005072
TC06000991.hg.1	3.09	JUC06005669.hg.1	LOC1005072
TC06000991.hg.1	2.51	PSR06011704.hg.1	LOC1005072
TC06000991.hg.1	-2.18	PSR06011705.hg.1	LOC1005072
TC13000025.hg.1	4.77	JUC13000116.hg.1	IFT88
TC13000025.hg.1	3.38	JUC13000094.hg.1	IFT88
TC13000025.hg.1	2.91	JUC13000093.hg.1	IFT88
TC13000025.hg.1	2.74	JUC13000120.hg.1	IFT88
TC13000025.hg.1	2.6	PSR13000189.hg.1	IFT88
TC13000025.hg.1	2.54	JUC13000096.hg.1	IFT88
TC13000025.hg.1	2.45	JUC13000103.hg.1	IFT88
TC13000025.hg.1	2.4	JUC13000102.hg.1	IFT88
TC13000025.hg.1	2.23	PSR13000162.hg.1	IFT88
TC13000025.hg.1	2.2	PSR13000158.hg.1	IFT88
TC13000025.hg.1	2.17	JUC13000100.hg.1	IFT88
TC13000025.hg.1	2.16	JUC13000099.hg.1	IFT88
TC13000025.hg.1	2.15	JUC13000108.hg.1	IFT88
TC13000025.hg.1	2.09	JUC13000117.hg.1	IFT88
TC13000025.hg.1	2.08	PSR13000177.hg.1	IFT88
TC13000025.hg.1	-2.09	JUC13000123.hg.1	IFT88
TC13000025.hg.1	-2.16	JUC13000112.hg.1	IFT88
TC13000025.hg.1	-2.81	JUC13000121.hg.1	IFT88
TC15002784.hg.1	4.77	PSR15000322.hg.1	SNRPN, SNU
TC15002784.hg.1	3.95	JUC15013593.hg.1	SNRPN, SNU

TC15002784.hg.1	2.3	JUC15013589.hg.1	SNRPN, SNU
TC15002784.hg.1	-2.53	JUC15013578.hg.1	SNRPN, SNU
TC04001380.hg.1	4.76	PSR04019319.hg.1	FAM13A
TC04001380.hg.1	4.61	JUC04010131.hg.1	FAM13A
TC04001380.hg.1	4.61	JUC04010144.hg.1	FAM13A
TC04001380.hg.1	4.22	JUC04010151.hg.1	FAM13A
TC04001380.hg.1	4.2	JUC04010135.hg.1	FAM13A
TC04001380.hg.1	4.1	JUC04010125.hg.1	FAM13A
TC04001380.hg.1	4	JUC04010122.hg.1	FAM13A
TC04001380.hg.1	3.91	PSR04019316.hg.1	FAM13A
TC04001380.hg.1	3.87	PSR04019312.hg.1	FAM13A
TC04001380.hg.1	3.62	PSR04019315.hg.1	FAM13A
TC04001380.hg.1	3.57	JUC04010146.hg.1	FAM13A
TC04001380.hg.1	3.41	JUC04010127.hg.1	FAM13A
TC04001380.hg.1	3.4	PSR04019302.hg.1	FAM13A
TC04001380.hg.1	3.35	PSR04019304.hg.1	FAM13A
TC04001380.hg.1	3.34	PSR04019310.hg.1	FAM13A
TC04001380.hg.1	3.31	PSR04019314.hg.1	FAM13A
TC04001380.hg.1	3.25	PSR04019318.hg.1	FAM13A
TC04001380.hg.1	3.19	PSR04019306.hg.1	FAM13A
TC04001380.hg.1	3.19	JUC04010117.hg.1	FAM13A
TC04001380.hg.1	3.18	PSR04019325.hg.1	FAM13A
TC04001380.hg.1	2.83	JUC04010154.hg.1	FAM13A
TC04001380.hg.1	2.58	PSR04019320.hg.1	FAM13A
TC04001380.hg.1	2.54	JUC04010143.hg.1	FAM13A
TC04001380.hg.1	2.52	PSR04019311.hg.1	FAM13A
TC04001380.hg.1	2.5	JUC04010156.hg.1	FAM13A
TC04001380.hg.1	2.32	PSR04019308.hg.1	FAM13A
TC04001380.hg.1	2.19	PSR04019313.hg.1	FAM13A
TC04001380.hg.1	2.13	PSR04019301.hg.1	FAM13A
TC04001380.hg.1	2.1	PSR04019307.hg.1	FAM13A
TC04001380.hg.1	-2.7	PSR04019260.hg.1	FAM13A
TC04001380.hg.1	-2.71	JUC04010145.hg.1	FAM13A
TC04001380.hg.1	-4.05	PSR04019294.hg.1	FAM13A
TC04001380.hg.1	-7.19	JUC04010124.hg.1	FAM13A
TC17001895.hg.1	4.76	JUC17014420.hg.1	RNF157
TC17001895.hg.1	2.43	JUC17014425.hg.1	RNF157
TC17001895.hg.1	2.39	PSR17025677.hg.1	RNF157
TC17001895.hg.1	2.08	JUC17014421.hg.1	RNF157
TC17001895.hg.1	-2.05	JUC17014423.hg.1	RNF157
TC17001895.hg.1	-2.59	JUC17014418.hg.1	RNF157
TC01000440.hg.1	4.74	JUC01003544.hg.1	ZNF362
TC01000440.hg.1	-2.12	JUC01003551.hg.1	ZNF362
TC01000440.hg.1	-2.4	PSR01006832.hg.1	ZNF362
TC01000769.hg.1	4.74	JUC01006425.hg.1	FPGT-TNNI3I
TC01000769.hg.1	4.21	JUC01006417.hg.1	FPGT-TNNI3I
TC01000769.hg.1	4.18	JUC01006437.hg.1	FPGT-TNNI3I
TC01000769.hg.1	3.81	JUC01006409.hg.1	FPGT-TNNI3I
TC01000769.hg.1	3.39	JUC01006402.hg.1	FPGT-TNNI3I
TC01000769.hg.1	3.09	JUC01006406.hg.1	FPGT-TNNI3I
TC01000769.hg.1	2.78	PSR01012244.hg.1	FPGT-TNNI3I
TC01000769.hg.1	2.57	PSR01012252.hg.1	FPGT-TNNI3I
TC01000769.hg.1	2.45	PSR01012254.hg.1	FPGT-TNNI3I
TC01000769.hg.1	2.35	PSR01012231.hg.1	FPGT-TNNI3I
TC01000769.hg.1	2.33	PSR01012251.hg.1	FPGT-TNNI3I
TC01000769.hg.1	2.33	PSR01012271.hg.1	FPGT-TNNI3I
TC01000769.hg.1	2.27	JUC01006408.hg.1	FPGT-TNNI3I
TC01000769.hg.1	2.21	PSR01012225.hg.1	FPGT-TNNI3I

TC01000769.hg.1	2.12	JUC01006438.hg.1	FPGT-TNNI3I
TC01000769.hg.1	2	PSR01012266.hg.1	FPGT-TNNI3I
TC01002300.hg.1	4.74	JUC01018939.hg.1	CAPZB
TC01002300.hg.1	2.86	PSR01035269.hg.1	CAPZB
TC01002300.hg.1	2.08	PSR01035271.hg.1	CAPZB
TC01002300.hg.1	-2.05	JUC01018945.hg.1	CAPZB
TC01002300.hg.1	-2.24	JUC01018958.hg.1	CAPZB
TC01002300.hg.1	-2.33	JUC01018955.hg.1	CAPZB
TC01002300.hg.1	-2.67	JUC01018949.hg.1	CAPZB
TC6_apd_hap1000	4.74	PSR6_apd_hap1000	HLA-H
TC6_apd_hap1000	2.94	JUC6_apd_hap1000(	HLA-H
TC6_apd_hap1000	2.63	PSR6_apd_hap1000	HLA-H
TC6_apd_hap1000	2.17	JUC6_apd_hap1000(	HLA-H
TC6_apd_hap1000	2.08	PSR6_apd_hap1000	HLA-H
TC03001298.hg.1	4.74	JUC03011151.hg.1	SCN10A
TC03001298.hg.1	2	PSR03022308.hg.1	SCN10A
TC01000649.hg.1	4.73	PSR01010613.hg.1	PODN
TC01000649.hg.1	4.67	JUC01005490.hg.1	PODN
TC01000649.hg.1	3.82	PSR01010614.hg.1	PODN
TC01000649.hg.1	2.77	JUC01005488.hg.1	PODN
TC01000649.hg.1	2.7	JUC01005475.hg.1	PODN
TC01000649.hg.1	-2.2	JUC01005491.hg.1	PODN
TC01000649.hg.1	-2.21	JUC01005492.hg.1	PODN
TC02002793.hg.1	4.72	PSR02045103.hg.1	TUBA4A
TC02002793.hg.1	4.63	PSR02045090.hg.1	TUBA4A
TC02002793.hg.1	4.28	PSR02045112.hg.1	TUBA4A
TC02002793.hg.1	4.16	PSR02045109.hg.1	TUBA4A
TC02002793.hg.1	4.11	PSR02045111.hg.1	TUBA4A
TC02002793.hg.1	3.98	PSR02045105.hg.1	TUBA4A
TC02002793.hg.1	3.85	PSR02045110.hg.1	TUBA4A
TC02002793.hg.1	3.84	JUC02023549.hg.1	TUBA4A
TC02002793.hg.1	3.25	PSR02045089.hg.1	TUBA4A
TC02002793.hg.1	2.61	JUC02023557.hg.1	TUBA4A
TC02002793.hg.1	2.5	PSR02045106.hg.1	TUBA4A
TC02002793.hg.1	2.12	PSR02045101.hg.1	TUBA4A
TC02002793.hg.1	-2	JUC02023553.hg.1	TUBA4A
TC02002793.hg.1	-2.53	JUC02023556.hg.1	TUBA4A
TC02002793.hg.1	-3.23	PSR02045096.hg.1	TUBA4A
TC03003407.hg.1	4.72	JUC03024561.hg.1	PRSS46, PRS
TC03003407.hg.1	2.44	PSR03023287.hg.1	PRSS46, PRS
TC03003407.hg.1	-2.04	JUC03024558.hg.1	PRSS46, PRS
TC03003407.hg.1	-2.1	PSR03023284.hg.1	PRSS46, PRS
TC03003407.hg.1	-2.18	PSR03023292.hg.1	PRSS46, PRS
TC01003070.hg.1	4.71	JUC01025590.hg.1	
TC01003070.hg.1	3.96	JUC01025618.hg.1	
TC01003070.hg.1	3.72	JUC01025612.hg.1	
TC01003070.hg.1	3.69	JUC01025603.hg.1	
TC01003070.hg.1	3.38	PSR01047722.hg.1	
TC01003070.hg.1	3.31	JUC01025595.hg.1	
TC01003070.hg.1	3.28	PSR01047732.hg.1	
TC01003070.hg.1	2.58	JUC01025608.hg.1	
TC01003070.hg.1	2.56	PSR01047727.hg.1	
TC01003070.hg.1	2.54	PSR01047724.hg.1	
TC01003070.hg.1	2.41	PSR01047714.hg.1	
TC01003070.hg.1	2.34	PSR01047703.hg.1	
TC01003070.hg.1	2.31	JUC01025614.hg.1	
TC01003070.hg.1	2.29	PSR01047707.hg.1	
TC01003070.hg.1	2.27	PSR01047702.hg.1	

TC01003070.hg.1	2.21	PSR01047701.hg.1	
TC01003070.hg.1	2.19	PSR01047713.hg.1	
TC01003070.hg.1	2.18	PSR01047728.hg.1	
TC01003070.hg.1	2.01	JUC01025599.hg.1	
TC06002197.hg.1	4.71	JUC06013709.hg.1	SHPRH
TC06002197.hg.1	3.66	PSR06027584.hg.1	SHPRH
TC06002197.hg.1	3.17	JUC06013721.hg.1	SHPRH
TC06002197.hg.1	2.35	JUC06013723.hg.1	SHPRH
TC06002197.hg.1	2.29	PSR06027561.hg.1	SHPRH
TC06002197.hg.1	2.27	PSR06027582.hg.1	SHPRH
TC06002197.hg.1	2.27	JUC06013720.hg.1	SHPRH
TC14001188.hg.1	4.71	JUC14006876.hg.1	SIX1
TC14001188.hg.1	4.45	PSR14013641.hg.1	SIX1
TC14001188.hg.1	4.19	PSR14013639.hg.1	SIX1
TC14001188.hg.1	2.17	PSR14013637.hg.1	SIX1
TC17000724.hg.1	4.71	JUC17005127.hg.1	DHX40
TC17000724.hg.1	2.05	JUC17005129.hg.1	DHX40
TC17000724.hg.1	-2.01	PSR17009430.hg.1	DHX40
TC05002052.hg.1	4.7	JUC05014438.hg.1	KCNMB1
TC05002052.hg.1	3.91	JUC05014437.hg.1	KCNMB1
TC05002052.hg.1	2.24	PSR05028265.hg.1	KCNMB1
TC10000726.hg.1	4.7	JUC10004729.hg.1	FAM178A
TC10000726.hg.1	2.59	PSR10008487.hg.1	FAM178A
TC10000726.hg.1	2.54	JUC10004743.hg.1	FAM178A
TC10000726.hg.1	2.27	PSR10008469.hg.1	FAM178A
TC10000726.hg.1	2.24	JUC10004740.hg.1	FAM178A
TC10000726.hg.1	-2.02	JUC10004736.hg.1	FAM178A
TC10000726.hg.1	-2.12	JUC10004732.hg.1	FAM178A
TC10000726.hg.1	-2.38	JUC10004733.hg.1	FAM178A
TC6_mann_hap40(	4.7	PSR6_mann_hap400	DDR1
TC6_mann_hap40(	3.21	JUC6_mann_hap400	DDR1
TC6_mann_hap40(	2.99	PSR6_mann_hap400	DDR1
TC6_mann_hap40(	2.91	JUC6_mann_hap400	DDR1
TC6_mann_hap40(	2.87	PSR6_mann_hap400	DDR1
TC6_mann_hap40(	2.79	PSR6_mann_hap400	DDR1
TC6_mann_hap40(	2.78	JUC6_mann_hap400	DDR1
TC6_mann_hap40(	2.74	PSR6_mann_hap400	DDR1
TC6_mann_hap40(	2.65	JUC6_mann_hap400	DDR1
TC6_mann_hap40(	2.6	JUC6_mann_hap400	DDR1
TC6_mann_hap40(	2.43	JUC6_mann_hap400	DDR1
TC6_mann_hap40(	2.42	PSR6_mann_hap400	DDR1
TC6_mann_hap40(	2.34	JUC6_mann_hap400	DDR1
TC6_mann_hap40(	2.28	PSR6_mann_hap400	DDR1
TC6_mann_hap40(	2.27	PSR6_mann_hap400	DDR1
TC6_mann_hap40(	2.26	JUC6_mann_hap400	DDR1
TC6_mann_hap40(	2.2	PSR6_mann_hap400	DDR1
TC6_mann_hap40(	2.19	PSR6_mann_hap400	DDR1
TC6_mann_hap40(	2.17	JUC6_mann_hap400	DDR1
TC6_mann_hap40(	2.04	PSR6_mann_hap400	DDR1
TC6_mann_hap40(	2	PSR6_mann_hap400	DDR1
TC6_mann_hap40(	-2.03	JUC6_mann_hap400	DDR1
TC6_mann_hap40(	-2.55	PSR6_mann_hap400	DDR1
TC6_qbl_hap6000(	4.7	PSR6_qbl_hap60006	DDR1
TC6_qbl_hap6000(	3.21	JUC6_qbl_hap60002	DDR1
TC6_qbl_hap6000(	2.99	PSR6_qbl_hap60006	DDR1
TC6_qbl_hap6000(	2.91	JUC6_qbl_hap60002	DDR1
TC6_qbl_hap6000(	2.87	PSR6_qbl_hap60006	DDR1
TC6_qbl_hap6000(	2.79	PSR6_qbl_hap60006	DDR1

TC6_qbl_hap6000(	2.78	JUC6_qbl_hap60002	DDR1
TC6_qbl_hap6000(	2.74	PSR6_qbl_hap60006	DDR1
TC6_qbl_hap6000(	2.65	JUC6_qbl_hap60002	DDR1
TC6_qbl_hap6000(	2.6	JUC6_qbl_hap60002	DDR1
TC6_qbl_hap6000(	2.43	JUC6_qbl_hap60002	DDR1
TC6_qbl_hap6000(	2.42	PSR6_qbl_hap60006	DDR1
TC6_qbl_hap6000(	2.34	JUC6_qbl_hap60002	DDR1
TC6_qbl_hap6000(	2.28	PSR6_qbl_hap60006	DDR1
TC6_qbl_hap6000(	2.27	PSR6_qbl_hap60006	DDR1
TC6_qbl_hap6000(	2.26	JUC6_qbl_hap60002	DDR1
TC6_qbl_hap6000(	2.2	PSR6_qbl_hap60006	DDR1
TC6_qbl_hap6000(	2.19	PSR6_qbl_hap60006	DDR1
TC6_qbl_hap6000(	2.17	JUC6_qbl_hap60002	DDR1
TC6_qbl_hap6000(	2.04	PSR6_qbl_hap60006	DDR1
TC6_qbl_hap6000(	2	PSR6_qbl_hap60005	DDR1
TC6_qbl_hap6000(	-2.03	JUC6_qbl_hap60002	DDR1
TC6_qbl_hap6000(	-2.55	PSR6_qbl_hap60006	DDR1
TC01002610.hg.1	4.69	JUC01021817.hg.1	CCDC163P
TC01002610.hg.1	3.32	JUC01021821.hg.1	CCDC163P
TC01002610.hg.1	2.32	JUC01021822.hg.1	CCDC163P
TC01002610.hg.1	2.28	PSR01040642.hg.1	CCDC163P
TC01002610.hg.1	2.21	JUC01021819.hg.1	CCDC163P
TC01002610.hg.1	2.18	PSR01040639.hg.1	CCDC163P
TC01000917.hg.1	4.67	JUC01007586.hg.1	RNPC3, AMY
TC01000917.hg.1	3.31	JUC01007597.hg.1	RNPC3, AMY
TC01000917.hg.1	3.13	JUC01007592.hg.1	RNPC3, AMY
TC01000917.hg.1	3.09	PSR01014346.hg.1	RNPC3, AMY
TC01000917.hg.1	2.86	JUC01007595.hg.1	RNPC3, AMY
TC01000917.hg.1	2.72	PSR01014368.hg.1	RNPC3, AMY
TC01000917.hg.1	2.64	PSR01014349.hg.1	RNPC3, AMY
TC01000917.hg.1	2.59	PSR01014381.hg.1	RNPC3, AMY
TC01000917.hg.1	2.16	PSR01014350.hg.1	RNPC3, AMY
TC01000917.hg.1	2.16	PSR01014351.hg.1	RNPC3, AMY
TC01000917.hg.1	2.13	PSR01014353.hg.1	RNPC3, AMY
TC01000917.hg.1	2.11	JUC01007587.hg.1	RNPC3, AMY
TC01000917.hg.1	2.11	JUC01007604.hg.1	RNPC3, AMY
TC01000917.hg.1	2.11	JUC01007615.hg.1	RNPC3, AMY
TC01000917.hg.1	2.06	PSR01014354.hg.1	RNPC3, AMY
TC01000917.hg.1	2.06	PSR01014395.hg.1	RNPC3, AMY
TC01000917.hg.1	2.05	JUC01007612.hg.1	RNPC3, AMY
TC01000917.hg.1	2.04	JUC01007600.hg.1	RNPC3, AMY
TC01000917.hg.1	2	PSR01014388.hg.1	RNPC3, AMY
TC05000270.hg.1	4.67	PSR05003344.hg.1	RNF180
TC05000270.hg.1	4.13	PSR05003334.hg.1	RNF180
TC05000270.hg.1	3.33	PSR05003335.hg.1	RNF180
TC05000270.hg.1	3.27	PSR05003340.hg.1	RNF180
TC05000270.hg.1	3.1	PSR05003331.hg.1	RNF180
TC05000270.hg.1	3.09	PSR05003346.hg.1	RNF180
TC05000270.hg.1	2.87	PSR05003341.hg.1	RNF180
TC05000270.hg.1	2.78	PSR05003347.hg.1	RNF180
TC05000270.hg.1	2.29	JUC05001791.hg.1	RNF180
TC05000270.hg.1	2.2	PSR05003345.hg.1	RNF180
TC05000270.hg.1	2.17	PSR05003338.hg.1	RNF180
TC11000753.hg.1	4.67	JUC11005069.hg.1	
TC11000753.hg.1	2.22	PSR11009788.hg.1	
TC11000753.hg.1	2.08	PSR11009786.hg.1	
TC11001884.hg.1	4.67	PSR11022098.hg.1	SNORD27, S
TC11001884.hg.1	3.06	JUC11011681.hg.1	SNORD27, S

TC11001884.hg.1	2.3	PSR11022178.hg.1	SNORD27, S
TC11001884.hg.1	2.27	JUC11011691.hg.1	SNORD27, S
TC11001884.hg.1	2.24	PSR11022156.hg.1	SNORD27, S
TC11001884.hg.1	2.22	PSR11022124.hg.1	SNORD27, S
TC11001884.hg.1	2.12	PSR11022164.hg.1	SNORD27, S
TC11001884.hg.1	-2.93	PSR11022146.hg.1	SNORD27, S
TC02002638.hg.1	4.66	JUC02022101.hg.1	HECW2
TC02002638.hg.1	2.29	JUC02022113.hg.1	HECW2
TC02002638.hg.1	-2.34	PSR02042298.hg.1	HECW2
TC02002638.hg.1	-2.48	JUC02022114.hg.1	HECW2
TC02002638.hg.1	-2.72	JUC02022097.hg.1	HECW2
TC02002638.hg.1	-2.79	JUC02022102.hg.1	HECW2
TC02002638.hg.1	-4.33	JUC02022122.hg.1	HECW2
TC04001719.hg.1	4.66	JUC04012592.hg.1	DDX60L
TC04001719.hg.1	2.98	JUC04012591.hg.1	DDX60L
TC04001719.hg.1	2.81	JUC04012564.hg.1	DDX60L
TC04001719.hg.1	2.45	PSR04023761.hg.1	DDX60L
TC04001719.hg.1	2.32	JUC04012578.hg.1	DDX60L
TC04001719.hg.1	2.06	PSR04023751.hg.1	DDX60L
TC04001719.hg.1	2.06	JUC04012589.hg.1	DDX60L
TC04001719.hg.1	2.06	JUC04012590.hg.1	DDX60L
TC04001719.hg.1	2.01	PSR04023780.hg.1	DDX60L
TC04001719.hg.1	2.01	PSR04023786.hg.1	DDX60L
TC10001051.hg.1	4.66	JUC10007562.hg.1	BEND7
TC10001051.hg.1	4.05	JUC10007555.hg.1	BEND7
TC10001051.hg.1	3.21	PSR10013072.hg.1	BEND7
TC10001051.hg.1	2.72	JUC10007569.hg.1	BEND7
TC10001051.hg.1	2.69	PSR10013077.hg.1	BEND7
TC10001051.hg.1	2.48	JUC10007567.hg.1	BEND7
TC10001051.hg.1	2.34	PSR10013073.hg.1	BEND7
TC10001051.hg.1	2.16	PSR10013057.hg.1	BEND7
TC10001051.hg.1	2.16	JUC10007559.hg.1	BEND7
TC10001051.hg.1	2.06	JUC10007563.hg.1	BEND7
TC10001051.hg.1	2.05	PSR10013040.hg.1	BEND7
TC6_qbl_hap60002	4.66	PSR6_qbl_hap60008	MICA
TC6_qbl_hap60002	4.28	JUC6_qbl_hap60021	MICA
TC6_qbl_hap60002	3.59	PSR6_qbl_hap60008	MICA
TC6_qbl_hap60002	3.55	PSR6_qbl_hap60008	MICA
TC6_qbl_hap60002	3.15	PSR6_qbl_hap60008	MICA
TC6_qbl_hap60002	2.76	JUC6_qbl_hap60021	MICA
TC6_qbl_hap60002	2.75	JUC6_qbl_hap60021	MICA
TC6_qbl_hap60002	2.72	PSR6_qbl_hap60008	MICA
TC6_qbl_hap60002	2.36	JUC6_qbl_hap60021	MICA
TC6_qbl_hap60002	2.36	JUC6_qbl_hap60021	MICA
TC6_qbl_hap60002	2.34	JUC6_qbl_hap60021	MICA
TC6_qbl_hap60002	2.14	PSR6_qbl_hap60008	MICA
TC6_qbl_hap60002	2.07	JUC6_qbl_hap60021	MICA
TC01002821.hg.1	4.65	PSR01043910.hg.1	SSX2IP
TC01002821.hg.1	3.39	JUC01023538.hg.1	SSX2IP
TC01002821.hg.1	3.1	PSR01043904.hg.1	SSX2IP
TC01002821.hg.1	3.04	JUC01023526.hg.1	SSX2IP
TC01002821.hg.1	2.71	PSR01043903.hg.1	SSX2IP
TC01002821.hg.1	2.61	PSR01043896.hg.1	SSX2IP
TC01002821.hg.1	2.56	JUC01023530.hg.1	SSX2IP
TC01002821.hg.1	2.46	PSR01043906.hg.1	SSX2IP
TC01002821.hg.1	2.44	JUC01023543.hg.1	SSX2IP
TC01002821.hg.1	2.41	PSR01043902.hg.1	SSX2IP
TC01002821.hg.1	2.37	PSR01043873.hg.1	SSX2IP

TC01002821.hg.1	2.34	PSR01043907.hg.1	SSX2IP
TC01002821.hg.1	2.02	JUC01023532.hg.1	SSX2IP
TC01002821.hg.1	-2.51	PSR01043888.hg.1	SSX2IP
TC01002821.hg.1	-2.65	JUC01023536.hg.1	SSX2IP
TC03001286.hg.1	4.65	JUC03011021.hg.1	LRRFIP2
TC03001286.hg.1	4.49	JUC03011022.hg.1	LRRFIP2
TC03001286.hg.1	4.41	PSR03022079.hg.1	LRRFIP2
TC03001286.hg.1	3.41	JUC03011030.hg.1	LRRFIP2
TC03001286.hg.1	2.53	JUC03011017.hg.1	LRRFIP2
TC03001286.hg.1	2.39	PSR03022087.hg.1	LRRFIP2
TC03001286.hg.1	2.26	PSR03022058.hg.1	LRRFIP2
TC03001286.hg.1	2.22	PSR03022074.hg.1	LRRFIP2
TC03001286.hg.1	2.05	PSR03022080.hg.1	LRRFIP2
TC03001286.hg.1	-2.26	JUC03011011.hg.1	LRRFIP2
TC03001286.hg.1	-2.26	JUC03011041.hg.1	LRRFIP2
TC04001805.hg.1	4.65	PSR04024589.hg.1	IRF2
TC04001805.hg.1	4.11	JUC04013016.hg.1	IRF2
TC04001805.hg.1	2.82	PSR04024592.hg.1	IRF2
TC04001805.hg.1	2.65	JUC04013029.hg.1	IRF2
TC04001805.hg.1	2.4	PSR04024586.hg.1	IRF2
TC04001805.hg.1	2.33	JUC04013015.hg.1	IRF2
TC04001805.hg.1	2.14	PSR04024572.hg.1	IRF2
TC04001805.hg.1	2.13	JUC04013020.hg.1	IRF2
TC04001805.hg.1	2.06	PSR04024583.hg.1	IRF2
TC04001805.hg.1	-2.04	PSR04024577.hg.1	IRF2
TC10001201.hg.1	4.65	PSR10015067.hg.1	HNRNPF
TC10001201.hg.1	2.24	JUC10008744.hg.1	HNRNPF
TC01002971.hg.1	4.65	JUC01024831.hg.1	SLC16A4
TC01002971.hg.1	4.31	JUC01024830.hg.1	SLC16A4
TC01002971.hg.1	2	PSR01046150.hg.1	SLC16A4
TC10000769.hg.1	4.64	JUC10005077.hg.1	CNNM2
TC10000769.hg.1	2.2	PSR10009152.hg.1	CNNM2
TC13000660.hg.1	4.64	JUC13004397.hg.1	CAB39L
TC13000660.hg.1	4.48	PSR13007600.hg.1	CAB39L
TC13000660.hg.1	3.85	PSR13007602.hg.1	CAB39L
TC13000660.hg.1	3.53	PSR13007606.hg.1	CAB39L
TC13000660.hg.1	2.89	PSR13007597.hg.1	CAB39L
TC13000660.hg.1	2.86	JUC13004401.hg.1	CAB39L
TC13000660.hg.1	2.84	PSR13007592.hg.1	CAB39L
TC13000660.hg.1	2.77	JUC13004394.hg.1	CAB39L
TC13000660.hg.1	2.75	JUC13004414.hg.1	CAB39L
TC13000660.hg.1	2.7	PSR13007601.hg.1	CAB39L
TC13000660.hg.1	2.57	JUC13004392.hg.1	CAB39L
TC13000660.hg.1	2.52	PSR13007598.hg.1	CAB39L
TC13000660.hg.1	2.45	PSR13007599.hg.1	CAB39L
TC13000660.hg.1	2.43	PSR13007594.hg.1	CAB39L
TC13000660.hg.1	2.01	PSR13007587.hg.1	CAB39L
TC0X000470.hg.1	4.63	JUC0X003004.hg.1	CSTF2
TC0X000470.hg.1	2.02	PSR0X006069.hg.1	CSTF2
TC0X000470.hg.1	-2.14	JUC0X003017.hg.1	CSTF2
TC0X000470.hg.1	-2.16	JUC0X003013.hg.1	CSTF2
TC04000754.hg.1	4.62	PSR04010459.hg.1	FAM160A1
TC04000754.hg.1	3.35	PSR04010454.hg.1	FAM160A1
TC04000754.hg.1	3.28	JUC04005610.hg.1	FAM160A1
TC04000754.hg.1	3.03	PSR04010452.hg.1	FAM160A1
TC04000754.hg.1	2.89	JUC04005616.hg.1	FAM160A1
TC04000754.hg.1	2.57	JUC04005602.hg.1	FAM160A1
TC04000754.hg.1	2.51	JUC04005608.hg.1	FAM160A1

TC04000754.hg.1	2.28	JUC04005603.hg.1	FAM160A1
TC04000754.hg.1	2.21	PSR04010453.hg.1	FAM160A1
TC04000754.hg.1	2.14	JUC04005614.hg.1	FAM160A1
TC08000508.hg.1	4.62	PSR08006955.hg.1	STMN2
TC08000508.hg.1	-2.15	JUC08003447.hg.1	STMN2
TC08000508.hg.1	-2.17	PSR08006948.hg.1	STMN2
TC08000508.hg.1	-2.24	PSR08006947.hg.1	STMN2
TC08000508.hg.1	-4.4	JUC08003449.hg.1	STMN2
TC08000508.hg.1	-7.11	JUC08003443.hg.1	STMN2
TC12000641.hg.1	4.62	PSR12008513.hg.1	GLIPR1L2
TC12000641.hg.1	2.27	JUC12004496.hg.1	GLIPR1L2
TC14001124.hg.1	4.62	PSR14012816.hg.1	PYGL
TC14001124.hg.1	4.24	PSR14012783.hg.1	PYGL
TC14001124.hg.1	2.36	PSR14012815.hg.1	PYGL
TC14001124.hg.1	2.12	JUC14006452.hg.1	PYGL
TC14001124.hg.1	-2.48	JUC14006467.hg.1	PYGL
TC14001124.hg.1	-2.93	JUC14006472.hg.1	PYGL
TC19001688.hg.1	4.62	PSR19023044.hg.1	DBP
TC19001688.hg.1	2.62	PSR19023039.hg.1	DBP
TC19001688.hg.1	2.25	PSR19023042.hg.1	DBP
TC19001688.hg.1	-2.26	PSR19023035.hg.1	DBP
TC19001688.hg.1	-5.06	JUC19013149.hg.1	DBP
TC01002608.hg.1	4.61	PSR01040608.hg.1	TESK2
TC01002608.hg.1	4.18	PSR01040626.hg.1	TESK2
TC01002608.hg.1	4.14	PSR01040619.hg.1	TESK2
TC01002608.hg.1	3.55	JUC01021807.hg.1	TESK2
TC01002608.hg.1	3.41	JUC01021811.hg.1	TESK2
TC01002608.hg.1	2.9	PSR01040625.hg.1	TESK2
TC01002608.hg.1	2.51	PSR01040612.hg.1	TESK2
TC01006430.hg.1	4.61	JUC01043856.hg.1	NBPF9
TC01006430.hg.1	2.78	JUC01041183.hg.1	NBPF9
TC01006430.hg.1	2.4	PSR01016961.hg.1	NBPF9
TC02002398.hg.1	4.61	JUC02019640.hg.1	GTDC1
TC02002398.hg.1	2.12	JUC02019635.hg.1	GTDC1
TC02002398.hg.1	-2.03	PSR02037742.hg.1	GTDC1
TC02002398.hg.1	-2.17	JUC02019636.hg.1	GTDC1
TC09000883.hg.1	4.61	JUC09006379.hg.1	SPATA6L
TC09000883.hg.1	-2.13	JUC09006355.hg.1	SPATA6L
TC09000883.hg.1	-2.3	JUC09006363.hg.1	SPATA6L
TC09000883.hg.1	-2.62	PSR09011763.hg.1	SPATA6L
TC09001583.hg.1	4.6	JUC09010773.hg.1	GOLGA1
TC09001583.hg.1	3.96	JUC09010754.hg.1	GOLGA1
TC09001583.hg.1	2.52	JUC09010762.hg.1	GOLGA1
TC09001583.hg.1	2.31	JUC09010770.hg.1	GOLGA1
TC09001583.hg.1	-3.66	PSR09019900.hg.1	GOLGA1
TC0X000141.hg.1	4.6	JUC0X000959.hg.1	GK
TC0X000141.hg.1	2.68	JUC0X000974.hg.1	GK
TC0X000141.hg.1	2.66	PSR0X001781.hg.1	GK
TC0X000141.hg.1	2.41	JUC0X000967.hg.1	GK
TC0X000141.hg.1	2.09	JUC0X000986.hg.1	GK
TC0X000141.hg.1	2.07	JUC0X000953.hg.1	GK
TC0X000141.hg.1	2	PSR0X001760.hg.1	GK
TC19001760.hg.1	4.59	PSR19023929.hg.1	KLKP1
TC19001760.hg.1	-2.51	PSR19023933.hg.1	KLKP1
TC19001760.hg.1	-2.65	PSR19023934.hg.1	KLKP1
TC19001760.hg.1	-2.77	PSR19023932.hg.1	KLKP1
TC19001760.hg.1	-3.71	JUC19013599.hg.1	KLKP1
TC22000816.hg.1	4.59	PSR22013870.hg.1	ST13

TC22000816.hg.1	3	JUC22005707.hg.1	ST13
TC06001961.hg.1	4.58	JUC06011831.hg.1	FBXL4
TC06001961.hg.1	2.53	JUC06011823.hg.1	FBXL4
TC06001961.hg.1	2.44	PSR06024158.hg.1	FBXL4
TC06001961.hg.1	-2.6	JUC06011827.hg.1	FBXL4
TC07001635.hg.1	4.58	JUC07012382.hg.1	BAIAP2L1
TC07001635.hg.1	2.75	PSR07024941.hg.1	BAIAP2L1
TC07001635.hg.1	2.72	JUC07012383.hg.1	BAIAP2L1
TC07001635.hg.1	2.69	PSR07024937.hg.1	BAIAP2L1
TC07001635.hg.1	2.67	JUC07012372.hg.1	BAIAP2L1
TC07001635.hg.1	2.11	PSR07024922.hg.1	BAIAP2L1
TC07001635.hg.1	2.1	PSR07024939.hg.1	BAIAP2L1
TC14000067.hg.1	4.58	JUC14000131.hg.1	ANG, RNASE
TC14000067.hg.1	4.25	JUC14000132.hg.1	ANG, RNASE
TC14000067.hg.1	3.25	PSR14000287.hg.1	ANG, RNASE
TC14000067.hg.1	3.22	PSR14000290.hg.1	ANG, RNASE
TC14000067.hg.1	2.93	JUC14000133.hg.1	ANG, RNASE
TC14000067.hg.1	2.45	PSR14000289.hg.1	ANG, RNASE
TC14000067.hg.1	2.41	PSR14000288.hg.1	ANG, RNASE
TC01000685.hg.1	4.57	PSR01010994.hg.1	PRKAA2
TC01000685.hg.1	4.1	JUC01005640.hg.1	PRKAA2
TC01000685.hg.1	2.03	JUC01005642.hg.1	PRKAA2
TC01002441.hg.1	4.57	JUC01020192.hg.1	LAPTM5
TC01002441.hg.1	4.18	PSR01037577.hg.1	LAPTM5
TC01002441.hg.1	2.9	JUC01020188.hg.1	LAPTM5
TC01002441.hg.1	2.81	PSR01037572.hg.1	LAPTM5
TC01002441.hg.1	2.8	PSR01037578.hg.1	LAPTM5
TC01002441.hg.1	2.27	PSR01037575.hg.1	LAPTM5
TC01002441.hg.1	-2.81	JUC01020184.hg.1	LAPTM5
TC01002441.hg.1	-3.72	PSR01037564.hg.1	LAPTM5
TC07000168.hg.1	4.57	JUC07001288.hg.1	MIR196B
TC07000168.hg.1	4.33	PSR07002653.hg.1	MIR196B
TC07000168.hg.1	4.26	PSR07002644.hg.1	MIR196B
TC07000168.hg.1	4.24	PSR07002646.hg.1	MIR196B
TC07000168.hg.1	2.71	JUC07001289.hg.1	MIR196B
TC07000168.hg.1	2.42	PSR07002647.hg.1	MIR196B
TC07000168.hg.1	-2.04	JUC07001287.hg.1	MIR196B
TC07000235.hg.1	4.57	JUC07001744.hg.1	ANLN
TC07000235.hg.1	4.43	PSR07003661.hg.1	ANLN
TC07000235.hg.1	4.4	JUC07001753.hg.1	ANLN
TC07000235.hg.1	4.39	PSR07003609.hg.1	ANLN
TC07000235.hg.1	4.36	JUC07001765.hg.1	ANLN
TC07000235.hg.1	4.15	PSR07003611.hg.1	ANLN
TC07000235.hg.1	4.14	PSR07003653.hg.1	ANLN
TC07000235.hg.1	4.07	PSR07003615.hg.1	ANLN
TC07000235.hg.1	3.71	PSR07003656.hg.1	ANLN
TC07000235.hg.1	3.45	JUC07001766.hg.1	ANLN
TC07000235.hg.1	3.15	JUC07001741.hg.1	ANLN
TC07000235.hg.1	2.73	PSR07003652.hg.1	ANLN
TC07000235.hg.1	2.49	PSR07003607.hg.1	ANLN
TC07000235.hg.1	2.45	PSR07003647.hg.1	ANLN
TC07000235.hg.1	2.33	JUC07001738.hg.1	ANLN
TC07000235.hg.1	2.14	PSR07003632.hg.1	ANLN
TC07000235.hg.1	2.13	JUC07001760.hg.1	ANLN
TC07000235.hg.1	2.12	JUC07001743.hg.1	ANLN
TC07000235.hg.1	2.09	PSR07003640.hg.1	ANLN
TC08001269.hg.1	4.57	JUC08008422.hg.1	GGH
TC08001269.hg.1	3.36	PSR08016357.hg.1	GGH

TC08001269.hg.1	3.06	PSR08016366.hg.1	GGH
TC08001269.hg.1	2.8	PSR08016370.hg.1	GGH
TC08001269.hg.1	2.8	JUC08008412.hg.1	GGH
TC08001269.hg.1	2.71	PSR08016368.hg.1	GGH
TC08001269.hg.1	2.37	PSR08016360.hg.1	GGH
TC08001269.hg.1	-2.37	JUC08008411.hg.1	GGH
TC10001711.hg.1	4.57	JUC10013107.hg.1	FGFR2
TC10001711.hg.1	4.53	JUC10013115.hg.1	FGFR2
TC10001711.hg.1	4.4	PSR10022638.hg.1	FGFR2
TC10001711.hg.1	4.36	PSR10022618.hg.1	FGFR2
TC10001711.hg.1	4.28	JUC10013109.hg.1	FGFR2
TC10001711.hg.1	3.94	JUC10013111.hg.1	FGFR2
TC10001711.hg.1	3.7	JUC10013089.hg.1	FGFR2
TC10001711.hg.1	3.6	PSR10022641.hg.1	FGFR2
TC10001711.hg.1	3.17	PSR10022620.hg.1	FGFR2
TC10001711.hg.1	3.15	PSR10022617.hg.1	FGFR2
TC10001711.hg.1	3.08	JUC10013105.hg.1	FGFR2
TC10001711.hg.1	3.07	PSR10022639.hg.1	FGFR2
TC10001711.hg.1	3.03	PSR10022592.hg.1	FGFR2
TC10001711.hg.1	2.83	PSR10022615.hg.1	FGFR2
TC10001711.hg.1	2.48	PSR10022626.hg.1	FGFR2
TC10001711.hg.1	2.48	PSR10022631.hg.1	FGFR2
TC10001711.hg.1	2.42	JUC10013096.hg.1	FGFR2
TC10001711.hg.1	2.41	PSR10022587.hg.1	FGFR2
TC10001711.hg.1	2.41	PSR10022637.hg.1	FGFR2
TC10001711.hg.1	-2.06	JUC10013124.hg.1	FGFR2
TC10001711.hg.1	-2.06	JUC10013125.hg.1	FGFR2
TC10001711.hg.1	-2.48	PSR10022579.hg.1	FGFR2
TC6_ssto_hap700(	4.57	JUC6_ssto_hap7000	BRD2
TC6_ssto_hap700(	2.58	JUC6_ssto_hap7000	BRD2
TC6_ssto_hap700(	2	PSR6_ssto_hap7001	BRD2
TC02000386.hg.1	4.56	JUC02003021.hg.1	MEIS1
TC02000386.hg.1	4.08	JUC02003035.hg.1	MEIS1
TC02000386.hg.1	3.52	PSR02005830.hg.1	MEIS1
TC02000386.hg.1	2.83	JUC02003038.hg.1	MEIS1
TC02000386.hg.1	2.65	PSR02005798.hg.1	MEIS1
TC02000386.hg.1	2.61	JUC02003023.hg.1	MEIS1
TC02000386.hg.1	2.38	PSR02005824.hg.1	MEIS1
TC02000386.hg.1	2.3	JUC02003026.hg.1	MEIS1
TC02000386.hg.1	2.19	JUC02003016.hg.1	MEIS1
TC02000386.hg.1	2.13	JUC02003020.hg.1	MEIS1
TC02000386.hg.1	2.11	JUC02003033.hg.1	MEIS1
TC02000386.hg.1	2.07	JUC02003013.hg.1	MEIS1
TC02000386.hg.1	2.05	PSR02005839.hg.1	MEIS1
TC02000386.hg.1	2.02	PSR02005800.hg.1	MEIS1
TC02000386.hg.1	2.01	JUC02003036.hg.1	MEIS1
TC02000386.hg.1	2	PSR02005810.hg.1	MEIS1
TC02000386.hg.1	-2.37	JUC02003025.hg.1	MEIS1
TC02000386.hg.1	-3.36	PSR02005819.hg.1	MEIS1
TC04000303.hg.1	4.56	JUC04002132.hg.1	RASL11B
TC04000303.hg.1	-2.38	PSR04004286.hg.1	RASL11B
TC04000303.hg.1	-3.23	PSR04004291.hg.1	RASL11B
TC04000303.hg.1	-3.37	JUC04002130.hg.1	RASL11B
TC04000303.hg.1	-11.38	PSR04004288.hg.1	RASL11B
TC01000703.hg.1	4.55	JUC01005813.hg.1	INADL
TC01000703.hg.1	3.66	PSR01011279.hg.1	INADL
TC01000703.hg.1	3.62	JUC01005828.hg.1	INADL
TC01000703.hg.1	3.46	PSR01011213.hg.1	INADL

TC01000703.hg.1	3.18	JUC01005805.hg.1	INADL
TC01000703.hg.1	3.15	PSR01011236.hg.1	INADL
TC01000703.hg.1	3.14	PSR01011282.hg.1	INADL
TC01000703.hg.1	3	JUC01005786.hg.1	INADL
TC01000703.hg.1	2.87	JUC01005825.hg.1	INADL
TC01000703.hg.1	2.84	PSR01011272.hg.1	INADL
TC01000703.hg.1	2.81	JUC01005836.hg.1	INADL
TC01000703.hg.1	2.58	PSR01011264.hg.1	INADL
TC01000703.hg.1	2.52	PSR01011274.hg.1	INADL
TC01000703.hg.1	2.45	PSR01011253.hg.1	INADL
TC01000703.hg.1	2.4	JUC01005820.hg.1	INADL
TC01000703.hg.1	2.36	PSR01011265.hg.1	INADL
TC01000703.hg.1	2.36	JUC01005811.hg.1	INADL
TC01000703.hg.1	2.35	PSR01011290.hg.1	INADL
TC01000703.hg.1	2.27	JUC01005821.hg.1	INADL
TC01000703.hg.1	2.18	PSR01011263.hg.1	INADL
TC01000703.hg.1	2.18	PSR01011285.hg.1	INADL
TC01000703.hg.1	2.08	PSR01011254.hg.1	INADL
TC01000703.hg.1	2.04	PSR01011262.hg.1	INADL
TC01000703.hg.1	2	PSR01011276.hg.1	INADL
TC01000703.hg.1	2	JUC01005815.hg.1	INADL
TC01000703.hg.1	-2.01	JUC01005809.hg.1	INADL
TC01000703.hg.1	-2.09	PSR01011245.hg.1	INADL
TC01000703.hg.1	-2.54	JUC01005837.hg.1	INADL
TC01000703.hg.1	-2.59	JUC01005818.hg.1	INADL
TC11000150.hg.1	4.55	PSR11002155.hg.1	PPFIBP2
TC11000150.hg.1	3.03	PSR11002113.hg.1	PPFIBP2
TC11000150.hg.1	2.96	PSR11002139.hg.1	PPFIBP2
TC11000150.hg.1	2.85	JUC11000943.hg.1	PPFIBP2
TC11000150.hg.1	2.72	PSR11002119.hg.1	PPFIBP2
TC11000150.hg.1	2.51	PSR11002138.hg.1	PPFIBP2
TC11000150.hg.1	2.44	PSR11002121.hg.1	PPFIBP2
TC11000150.hg.1	2.44	PSR11002152.hg.1	PPFIBP2
TC11000150.hg.1	2.27	PSR11002124.hg.1	PPFIBP2
TC11000150.hg.1	2.27	PSR11002156.hg.1	PPFIBP2
TC11000150.hg.1	2.26	PSR11002118.hg.1	PPFIBP2
TC11000150.hg.1	2.17	JUC11000956.hg.1	PPFIBP2
TC11000150.hg.1	2.05	JUC11000931.hg.1	PPFIBP2
TC11000150.hg.1	-2.03	JUC11000937.hg.1	PPFIBP2
TC11000150.hg.1	-2.08	JUC11000950.hg.1	PPFIBP2
TC11000150.hg.1	-2.54	JUC11000954.hg.1	PPFIBP2
TC11000150.hg.1	-3.11	JUC11000949.hg.1	PPFIBP2
TC14000585.hg.1	4.55	JUC14003722.hg.1	PPP4R4
TC14000585.hg.1	3.78	JUC14003747.hg.1	PPP4R4
TC14000585.hg.1	2.9	JUC14003730.hg.1	PPP4R4
TC14000585.hg.1	2.9	JUC14003744.hg.1	PPP4R4
TC14000585.hg.1	2.55	JUC14003735.hg.1	PPP4R4
TC14000585.hg.1	2.33	JUC14003727.hg.1	PPP4R4
TC14000585.hg.1	2.12	PSR14007236.hg.1	PPP4R4
TC14000585.hg.1	-2.14	JUC14003740.hg.1	PPP4R4
TC01000394.hg.1	4.54	PSR01006088.hg.1	EPB41
TC01000394.hg.1	3.94	PSR01006052.hg.1	EPB41
TC01000394.hg.1	3.88	PSR01006053.hg.1	EPB41
TC01000394.hg.1	3.68	PSR01006050.hg.1	EPB41
TC01000394.hg.1	3.63	PSR01006054.hg.1	EPB41
TC01000394.hg.1	3.31	PSR01006056.hg.1	EPB41
TC01000394.hg.1	3.14	JUC01003148.hg.1	EPB41
TC01000394.hg.1	3.08	JUC01003147.hg.1	EPB41

TC01000394.hg.1	3.04	JUC01003150.hg.1	EPB41
TC01000394.hg.1	2.63	JUC01003156.hg.1	EPB41
TC01000394.hg.1	2.57	JUC01003162.hg.1	EPB41
TC01000394.hg.1	2.52	PSR01006082.hg.1	EPB41
TC01000394.hg.1	2.45	JUC01003136.hg.1	EPB41
TC01000394.hg.1	2.44	PSR01006081.hg.1	EPB41
TC01000394.hg.1	2.27	PSR01006076.hg.1	EPB41
TC01000394.hg.1	2.22	PSR01006077.hg.1	EPB41
TC01000394.hg.1	2.21	JUC01003166.hg.1	EPB41
TC01000394.hg.1	2.02	JUC01003142.hg.1	EPB41
TC01000394.hg.1	-2.04	JUC01003163.hg.1	EPB41
TC01000394.hg.1	-2.05	JUC01003160.hg.1	EPB41
TC01000394.hg.1	-2.29	JUC01003154.hg.1	EPB41
TC01000394.hg.1	-2.65	JUC01003153.hg.1	EPB41
TC01001736.hg.1	4.54	PSR01027147.hg.1	CD55
TC01001736.hg.1	3	PSR01027129.hg.1	CD55
TC01001736.hg.1	3	PSR01027138.hg.1	CD55
TC01001736.hg.1	2.71	PSR01027141.hg.1	CD55
TC01001736.hg.1	2.4	PSR01027145.hg.1	CD55
TC01001736.hg.1	2.38	PSR01027144.hg.1	CD55
TC01001736.hg.1	2.36	PSR01027140.hg.1	CD55
TC01001736.hg.1	2.32	PSR01027132.hg.1	CD55
TC01001736.hg.1	2.25	JUC01014542.hg.1	CD55
TC01001736.hg.1	2.23	PSR01027142.hg.1	CD55
TC01001736.hg.1	2.09	PSR01027143.hg.1	CD55
TC01001736.hg.1	2.02	JUC01014529.hg.1	CD55
TC01001736.hg.1	-2.32	JUC01014537.hg.1	CD55
TC01001736.hg.1	-2.54	PSR01027136.hg.1	CD55
TC07000838.hg.1	4.54	PSR07013178.hg.1	CALD1
TC07000838.hg.1	3.82	JUC07006281.hg.1	CALD1
TC07000838.hg.1	3.32	PSR07013170.hg.1	CALD1
TC07000838.hg.1	2.94	PSR07013162.hg.1	CALD1
TC07000838.hg.1	2.79	PSR07013169.hg.1	CALD1
TC07000838.hg.1	2.6	PSR07013177.hg.1	CALD1
TC07000838.hg.1	2.57	JUC07006271.hg.1	CALD1
TC07000838.hg.1	2.56	PSR07013163.hg.1	CALD1
TC07000838.hg.1	-2.1	PSR07013154.hg.1	CALD1
TC07000838.hg.1	-2.1	JUC07006276.hg.1	CALD1
TC07000838.hg.1	-2.44	PSR07013192.hg.1	CALD1
TC07000838.hg.1	-3.1	PSR07013173.hg.1	CALD1
TC07000838.hg.1	-6.4	JUC07006280.hg.1	CALD1
TC01003719.hg.1	4.53	JUC01029802.hg.1	CHIT1
TC01003719.hg.1	3.16	JUC01029783.hg.1	CHIT1
TC01003719.hg.1	2.29	JUC01029789.hg.1	CHIT1
TC01003719.hg.1	2.05	PSR01057092.hg.1	CHIT1
TC01003719.hg.1	2.04	PSR01057104.hg.1	CHIT1
TC01003719.hg.1	-2.43	PSR01057086.hg.1	CHIT1
TC04001001.hg.1	4.53	JUC04007398.hg.1	PPP2R2C
TC04001001.hg.1	4.37	JUC04007382.hg.1	PPP2R2C
TC04001001.hg.1	2.07	PSR04013860.hg.1	PPP2R2C
TC04001001.hg.1	-2.24	JUC04007389.hg.1	PPP2R2C
TC04001001.hg.1	-2.28	PSR04013864.hg.1	PPP2R2C
TC04001001.hg.1	-2.64	JUC04007381.hg.1	PPP2R2C
TC04001001.hg.1	-2.8	JUC04007390.hg.1	PPP2R2C
TC04001001.hg.1	-2.97	JUC04007386.hg.1	PPP2R2C
TC04001001.hg.1	-3.16	PSR04013875.hg.1	PPP2R2C
TC04001001.hg.1	-3.56	JUC04007383.hg.1	PPP2R2C
TC04001001.hg.1	-3.59	PSR04013876.hg.1	PPP2R2C

TC04001001.hg.1	-4.24	PSR04013874.hg.1	PPP2R2C
TC04001001.hg.1	-4.68	PSR04013858.hg.1	PPP2R2C
TC04001001.hg.1	-4.91	JUC04007387.hg.1	PPP2R2C
TC04001001.hg.1	-5.6	PSR04013856.hg.1	PPP2R2C
TC04001001.hg.1	-5.93	PSR04013877.hg.1	PPP2R2C
TC04001001.hg.1	-6.75	PSR04013862.hg.1	PPP2R2C
TC04001001.hg.1	-6.83	PSR04013873.hg.1	PPP2R2C
TC04001001.hg.1	-7.51	PSR04013871.hg.1	PPP2R2C
TC04001001.hg.1	-7.96	PSR04013865.hg.1	PPP2R2C
TC04001001.hg.1	-7.98	PSR04013869.hg.1	PPP2R2C
TC04001001.hg.1	-8.03	PSR04013866.hg.1	PPP2R2C
TC04001001.hg.1	-9.08	PSR04013872.hg.1	PPP2R2C
TC04001001.hg.1	-16.8	JUC04007385.hg.1	PPP2R2C
TC01000139.hg.1	4.52	JUC01001178.hg.1	ANGPTL7
TC01000139.hg.1	-2.04	PSR01002181.hg.1	ANGPTL7
TC01000139.hg.1	-3.17	JUC01001182.hg.1	ANGPTL7
TC01000139.hg.1	-3.63	PSR01002188.hg.1	ANGPTL7
TC01000139.hg.1	-4.02	PSR01002183.hg.1	ANGPTL7
TC01000139.hg.1	-8.75	JUC01001181.hg.1	ANGPTL7
TC03002152.hg.1	4.52	JUC03018846.hg.1	TNK2
TC03002152.hg.1	3.54	JUC03018868.hg.1	TNK2
TC03002152.hg.1	2.78	PSR03038009.hg.1	TNK2
TC03002152.hg.1	2.71	JUC03018840.hg.1	TNK2
TC03002152.hg.1	2.67	JUC03018853.hg.1	TNK2
TC03002152.hg.1	2.48	JUC03018865.hg.1	TNK2
TC03002152.hg.1	2.47	JUC03018859.hg.1	TNK2
TC03002152.hg.1	2.43	PSR03038002.hg.1	TNK2
TC03002152.hg.1	2.36	PSR03038018.hg.1	TNK2
TC03002152.hg.1	2.3	PSR03037990.hg.1	TNK2
TC03002152.hg.1	2.19	JUC03018856.hg.1	TNK2
TC03002152.hg.1	2.16	JUC03018832.hg.1	TNK2
TC03002152.hg.1	2.06	PSR03038027.hg.1	TNK2
TC03002152.hg.1	-2.08	JUC03018849.hg.1	TNK2
TC03002152.hg.1	-2.99	JUC03018864.hg.1	TNK2
TC03002152.hg.1	-3.71	JUC03018843.hg.1	TNK2
TC06004061.hg.1	4.51	PSR06000123.hg.1	DKFZP686I1
TC06004061.hg.1	2.67	JUC06020809.hg.1	DKFZP686I1
TC06004061.hg.1	2.33	PSR06000124.hg.1	DKFZP686I1
TC06004061.hg.1	2.07	PSR06000125.hg.1	DKFZP686I1
TC08001033.hg.1	4.5	PSR08013164.hg.1	HR
TC08001033.hg.1	4.3	PSR08013149.hg.1	HR
TC08001033.hg.1	4.07	PSR08013173.hg.1	HR
TC08001033.hg.1	3.76	JUC08006840.hg.1	HR
TC08001033.hg.1	3.57	PSR08013180.hg.1	HR
TC08001033.hg.1	3.47	JUC08006841.hg.1	HR
TC08001033.hg.1	3.42	PSR08013176.hg.1	HR
TC08001033.hg.1	3.25	PSR08013151.hg.1	HR
TC08001033.hg.1	2.71	PSR08013159.hg.1	HR
TC08001033.hg.1	2.57	PSR08013150.hg.1	HR
TC08001033.hg.1	2.5	PSR08013174.hg.1	HR
TC08001033.hg.1	2.34	JUC08006833.hg.1	HR
TC08001033.hg.1	2.12	JUC08006832.hg.1	HR
TC08001033.hg.1	-2.16	PSR08013166.hg.1	HR
TC08001033.hg.1	-2.27	PSR08013147.hg.1	HR
TC08001033.hg.1	-2.64	PSR08013169.hg.1	HR
TC08001033.hg.1	-3.06	JUC08006842.hg.1	HR
TC08001033.hg.1	-4.72	JUC08006837.hg.1	HR
TC08001033.hg.1	-6.12	PSR08013163.hg.1	HR

TC08001033.hg.1	-6.97	JUC08006827.hg.1	HR
TC08001033.hg.1	-7.77	JUC08006826.hg.1	HR
TC08001033.hg.1	-11.52	JUC08006825.hg.1	HR
TC12001181.hg.1	4.5	PSR12015690.hg.1	MFAP5
TC12001181.hg.1	4.28	JUC12008633.hg.1	MFAP5
TC12001181.hg.1	3.4	JUC12008647.hg.1	MFAP5
TC12001181.hg.1	2.76	JUC12008638.hg.1	MFAP5
TC12001181.hg.1	2.43	PSR12015664.hg.1	MFAP5
TC12001181.hg.1	2.37	PSR12015663.hg.1	MFAP5
TC12001181.hg.1	2.1	PSR12015666.hg.1	MFAP5
TC12001181.hg.1	-2	PSR12015675.hg.1	MFAP5
TC12001181.hg.1	-3.22	JUC12008646.hg.1	MFAP5
TC12001181.hg.1	-8.14	JUC12008634.hg.1	MFAP5
TC12001181.hg.1	-8.34	JUC12008643.hg.1	MFAP5
TC15000678.hg.1	4.5	PSR15005878.hg.1	ISLR
TC15000678.hg.1	4.44	JUC15003000.hg.1	ISLR
TC15000678.hg.1	-4.01	PSR15005873.hg.1	ISLR
TC01000134.hg.1	4.47	JUC01001165.hg.1	TARDBP
TC01000134.hg.1	2.26	JUC01001167.hg.1	TARDBP
TC01000134.hg.1	2.24	PSR01002166.hg.1	TARDBP
TC01000134.hg.1	-2.43	JUC01001158.hg.1	TARDBP
TC01003871.hg.1	4.47	PSR01058872.hg.1	TLR5
TC01003871.hg.1	3.13	PSR01058874.hg.1	TLR5
TC01003871.hg.1	2.42	PSR01058873.hg.1	TLR5
TC01003871.hg.1	2.23	PSR01058877.hg.1	TLR5
TC01003871.hg.1	2.04	PSR01058867.hg.1	TLR5
TC01003871.hg.1	-2.71	JUC01030851.hg.1	TLR5
TC01003871.hg.1	-3.49	JUC01030848.hg.1	TLR5
TC03002150.hg.1	4.47	JUC03018801.hg.1	MUC4
TC03002150.hg.1	2.86	JUC03018800.hg.1	MUC4
TC03002150.hg.1	2.75	PSR03037925.hg.1	MUC4
TC03002150.hg.1	2.26	JUC03018821.hg.1	MUC4
TC03002150.hg.1	-2.15	PSR03037935.hg.1	MUC4
TC04000396.hg.1	4.47	JUC04002704.hg.1	SLC4A4
TC04000396.hg.1	2.98	PSR04005441.hg.1	SLC4A4
TC04000396.hg.1	2.7	PSR04005442.hg.1	SLC4A4
TC04000396.hg.1	2.54	JUC04002728.hg.1	SLC4A4
TC04000396.hg.1	2.02	PSR04005460.hg.1	SLC4A4
TC04000396.hg.1	-2.22	JUC04002712.hg.1	SLC4A4
TC11000855.hg.1	4.47	JUC11005660.hg.1	LOC1005062
TC11000855.hg.1	3	JUC11005661.hg.1	LOC1005062
TC11000855.hg.1	2.7	JUC11005664.hg.1	LOC1005062
TC11000855.hg.1	2.18	JUC11005663.hg.1	LOC1005062
TC11000855.hg.1	2.17	PSR11010824.hg.1	LOC1005062
TC21000154.hg.1	4.47	JUC21000676.hg.1	CHAF1B
TC21000154.hg.1	2.17	PSR21001340.hg.1	CHAF1B
TC21000154.hg.1	-2.05	JUC21000668.hg.1	CHAF1B
TC21000154.hg.1	-2.11	PSR21001349.hg.1	CHAF1B
TC21000154.hg.1	-2.87	JUC21000674.hg.1	CHAF1B
TC12001700.hg.1	4.46	PSR12022692.hg.1	LOC1005071
TC12001700.hg.1	4.23	PSR12022707.hg.1	LOC1005071
TC12001700.hg.1	4.22	PSR12022702.hg.1	LOC1005071
TC12001700.hg.1	3.91	JUC12012509.hg.1	LOC1005071
TC12001700.hg.1	2.57	PSR12022700.hg.1	LOC1005071
TC12001700.hg.1	2.5	PSR12022696.hg.1	LOC1005071
TC12001700.hg.1	2.41	PSR12022708.hg.1	LOC1005071
TC12001700.hg.1	2.12	JUC12012511.hg.1	LOC1005071
TC6_mann_hap40	4.46	JUC6_mann_hap400	HLA-DRB3

TC6_mann_hap40(	2.29	PSR6_mann_hap40(	HLA-DRB3
TC6_mann_hap40(	2.16	JUC6_mann_hap400	HLA-DRB3
TC01000728.hg.1	4.45	JUC01006009.hg.1	AK4, LOC100
TC01000728.hg.1	4.43	JUC01006005.hg.1	AK4, LOC100
TC01000728.hg.1	3.98	JUC01006011.hg.1	AK4, LOC100
TC01000728.hg.1	3.87	PSR01011580.hg.1	AK4, LOC100
TC01000728.hg.1	3.5	PSR01011584.hg.1	AK4, LOC100
TC01000728.hg.1	3.32	PSR01011583.hg.1	AK4, LOC100
TC01000728.hg.1	2.89	PSR01011591.hg.1	AK4, LOC100
TC01000728.hg.1	2.85	PSR01011588.hg.1	AK4, LOC100
TC01000728.hg.1	2.62	PSR01011585.hg.1	AK4, LOC100
TC01000728.hg.1	2.56	PSR01011590.hg.1	AK4, LOC100
TC01000728.hg.1	2.12	PSR01011582.hg.1	AK4, LOC100
TC01000728.hg.1	-2.65	JUC01006015.hg.1	AK4, LOC100
TC07000617.hg.1	4.45	JUC07004322.hg.1	SPDYE3
TC07000617.hg.1	2.61	PSR07009343.hg.1	SPDYE3
TC07000617.hg.1	2.12	PSR07009355.hg.1	SPDYE3
TC08001675.hg.1	4.45	JUC08011220.hg.1	COL22A1
TC08001675.hg.1	2.34	JUC08011237.hg.1	COL22A1
TC08001675.hg.1	2.25	JUC08011189.hg.1	COL22A1
TC08001675.hg.1	2.1	PSR08021883.hg.1	COL22A1
TC08001675.hg.1	2	PSR08021918.hg.1	COL22A1
TC08001675.hg.1	-2.13	JUC08011184.hg.1	COL22A1
TC15000157.hg.1	4.45	JUC15000397.hg.1	GOLGA8J
TC15000157.hg.1	3.45	PSR15001003.hg.1	GOLGA8J
TC15000157.hg.1	-2.41	JUC15000383.hg.1	GOLGA8J
TC01003192.hg.1	4.44	JUC01026022.hg.1	HIST2H4B, H
TC01003192.hg.1	2.77	PSR01048907.hg.1	HIST2H4B, H
TC01003192.hg.1	2.51	PSR01048911.hg.1	HIST2H4B, H
TC01003192.hg.1	2.38	PSR01048910.hg.1	HIST2H4B, H
TC01003192.hg.1	2.09	PSR01048916.hg.1	HIST2H4B, H
TC01003733.hg.1	4.44	JUC01029913.hg.1	PIK3C2B
TC01003733.hg.1	2.95	JUC01029923.hg.1	PIK3C2B
TC01003733.hg.1	2.32	JUC01029931.hg.1	PIK3C2B
TC01003733.hg.1	-2.15	PSR01057339.hg.1	PIK3C2B
TC01003733.hg.1	-2.5	JUC01029915.hg.1	PIK3C2B
TC04000839.hg.1	4.44	JUC04006207.hg.1	PALLD
TC04000839.hg.1	2.59	PSR04011592.hg.1	PALLD
TC04000839.hg.1	2.16	PSR04011596.hg.1	PALLD
TC04000839.hg.1	2.1	PSR04011591.hg.1	PALLD
TC04000839.hg.1	-2.04	JUC04006186.hg.1	PALLD
TC04000839.hg.1	-2.54	PSR04011566.hg.1	PALLD
TC04000839.hg.1	-4.55	JUC04006184.hg.1	PALLD
TC05001924.hg.1	4.44	JUC05013527.hg.1	CSNK1A1
TC05001924.hg.1	4.29	JUC05013525.hg.1	CSNK1A1
TC05001924.hg.1	3.37	JUC05013517.hg.1	CSNK1A1
TC05001924.hg.1	2.18	JUC05013528.hg.1	CSNK1A1
TC05001924.hg.1	-2.02	PSR05026589.hg.1	CSNK1A1
TC05001924.hg.1	-2.11	PSR05026588.hg.1	CSNK1A1
TC06000165.hg.1	4.44	PSR06001683.hg.1	HFE
TC06000165.hg.1	2.96	JUC06000855.hg.1	HFE
TC06000165.hg.1	2.64	JUC06000842.hg.1	HFE
TC06000165.hg.1	2.19	PSR06001685.hg.1	HFE
TC0X000116.hg.1	4.44	JUC0X000848.hg.1	ZFX
TC0X000116.hg.1	3.2	PSR0X001544.hg.1	ZFX
TC0X000116.hg.1	3.16	PSR0X001550.hg.1	ZFX
TC0X000116.hg.1	3.08	PSR0X001554.hg.1	ZFX
TC0X000116.hg.1	3.05	PSR0X001545.hg.1	ZFX

TC0X000116.hg.1	2.96	JUC0X000833.hg.1	ZFX
TC0X000116.hg.1	2.9	PSR0X001535.hg.1	ZFX
TC0X000116.hg.1	2.85	PSR0X001546.hg.1	ZFX
TC0X000116.hg.1	2.85	PSR0X001547.hg.1	ZFX
TC0X000116.hg.1	2.85	PSR0X001571.hg.1	ZFX
TC0X000116.hg.1	2.82	PSR0X001542.hg.1	ZFX
TC0X000116.hg.1	2.66	JUC0X000839.hg.1	ZFX
TC0X000116.hg.1	2.37	JUC0X000840.hg.1	ZFX
TC0X000116.hg.1	2.17	JUC0X000837.hg.1	ZFX
TC0X000116.hg.1	2.11	JUC0X000860.hg.1	ZFX
TC0X000116.hg.1	-2.27	JUC0X000846.hg.1	ZFX
TC0X000116.hg.1	-2.3	JUC0X000832.hg.1	ZFX
TC0X000978.hg.1	4.44	JUC0X006556.hg.1	MAOB
TC0X000978.hg.1	3.86	JUC0X006546.hg.1	MAOB
TC0X000978.hg.1	3.49	PSR0X013041.hg.1	MAOB
TC0X000978.hg.1	2.82	JUC0X006545.hg.1	MAOB
TC0X000978.hg.1	2.77	JUC0X006553.hg.1	MAOB
TC0X000978.hg.1	2.61	JUC0X006539.hg.1	MAOB
TC0X000978.hg.1	2.48	PSR0X013037.hg.1	MAOB
TC0X000978.hg.1	2.32	JUC0X006543.hg.1	MAOB
TC0X000978.hg.1	2.15	PSR0X013038.hg.1	MAOB
TC0X000978.hg.1	2.09	PSR0X013036.hg.1	MAOB
TC0X000978.hg.1	2.02	PSR0X013010.hg.1	MAOB
TC0X000978.hg.1	-2.03	JUC0X006540.hg.1	MAOB
TC0X000978.hg.1	-2.1	JUC0X006552.hg.1	MAOB
TC6_cox_hap2000	4.44	PSR6_cox_hap2000:	HLA-H
TC6_cox_hap2000	3.11	JUC6_cox_hap20001	HLA-H
TC6_cox_hap2000	2.75	JUC6_cox_hap20001	HLA-H
TC6_cox_hap2000	2.66	PSR6_cox_hap2000:	HLA-H
TC6_cox_hap2000	2.46	PSR6_cox_hap2000:	HLA-H
TC6_cox_hap2000	2.03	JUC6_cox_hap20001	HLA-H
TC03001998.hg.1	4.43	PSR03035733.hg.1	TNIK
TC03001998.hg.1	4.23	JUC03017647.hg.1	TNIK
TC03001998.hg.1	3.11	PSR03035709.hg.1	TNIK
TC03001998.hg.1	2.98	PSR03035753.hg.1	TNIK
TC03001998.hg.1	2.91	PSR03035704.hg.1	TNIK
TC03001998.hg.1	2.82	PSR03035738.hg.1	TNIK
TC03001998.hg.1	2.78	JUC03017630.hg.1	TNIK
TC03001998.hg.1	2.46	PSR03035716.hg.1	TNIK
TC03001998.hg.1	2.42	PSR03035734.hg.1	TNIK
TC03001998.hg.1	2.35	PSR03035752.hg.1	TNIK
TC03001998.hg.1	2.22	JUC03017619.hg.1	TNIK
TC03001998.hg.1	2.14	JUC03017634.hg.1	TNIK
TC03001998.hg.1	2.07	PSR03035735.hg.1	TNIK
TC03001998.hg.1	2.06	PSR03035724.hg.1	TNIK
TC03001998.hg.1	-2.29	PSR03035750.hg.1	TNIK
TC03001998.hg.1	-3.2	PSR03035705.hg.1	TNIK
TC03001998.hg.1	-3.49	JUC03017641.hg.1	TNIK
TC03001998.hg.1	-4.32	JUC03017640.hg.1	TNIK
TC05000837.hg.1	4.43	JUC05006292.hg.1	GPX3
TC05000837.hg.1	2.74	JUC05006284.hg.1	GPX3
TC05000837.hg.1	2.57	JUC05006287.hg.1	GPX3
TC05000837.hg.1	2.51	PSR05012082.hg.1	GPX3
TC05000837.hg.1	2.04	PSR05012081.hg.1	GPX3
TC09000709.hg.1	4.43	JUC09004899.hg.1	PPP2R4
TC09000709.hg.1	2.07	JUC09004876.hg.1	PPP2R4
TC09000709.hg.1	-2.03	JUC09004880.hg.1	PPP2R4
TC09000709.hg.1	-2.2	PSR09008946.hg.1	PPP2R4

TC09000709.hg.1	-2.58	JUC09004875.hg.1	PPP2R4
TC09000709.hg.1	-2.67	JUC09004903.hg.1	PPP2R4
TC09000709.hg.1	-3.48	PSR09008947.hg.1	PPP2R4
TC22000224.hg.1	4.43	JUC22001517.hg.1	LIMK2
TC22000224.hg.1	3.83	PSR22004259.hg.1	LIMK2
TC22000224.hg.1	3.43	JUC22001519.hg.1	LIMK2
TC22000224.hg.1	3	PSR22004256.hg.1	LIMK2
TC22000224.hg.1	2.48	JUC22001521.hg.1	LIMK2
TC22000224.hg.1	2.45	JUC22001511.hg.1	LIMK2
TC22000224.hg.1	2.18	JUC22001509.hg.1	LIMK2
TC22000224.hg.1	-2.31	PSR22004251.hg.1	LIMK2
TC22000224.hg.1	-2.46	PSR22004288.hg.1	LIMK2
TC22000224.hg.1	-3.04	PSR22004252.hg.1	LIMK2
TC22000224.hg.1	-3.21	JUC22001531.hg.1	LIMK2
TC03003365.hg.1	4.42	JUC03023893.hg.1	MUC20
TC03003365.hg.1	3.52	JUC03023905.hg.1	MUC20
TC03003365.hg.1	3.52	JUC03023907.hg.1	MUC20
TC03003365.hg.1	3.4	PSR03018821.hg.1	MUC20
TC03003365.hg.1	3.07	JUC03023889.hg.1	MUC20
TC03003365.hg.1	3.06	JUC03023897.hg.1	MUC20
TC03003365.hg.1	2.96	PSR03018785.hg.1	MUC20
TC03003365.hg.1	2.8	PSR03018862.hg.1	MUC20
TC03003365.hg.1	2.6	JUC03023898.hg.1	MUC20
TC03003365.hg.1	2.58	JUC03023892.hg.1	MUC20
TC03003365.hg.1	2.56	JUC03023891.hg.1	MUC20
TC03003365.hg.1	2.33	PSR03018858.hg.1	MUC20
TC03003365.hg.1	2.13	PSR03018825.hg.1	MUC20
TC03003365.hg.1	2.13	PSR03018853.hg.1	MUC20
TC03003365.hg.1	2.1	JUC03023901.hg.1	MUC20
TC03003365.hg.1	2.1	JUC03023902.hg.1	MUC20
TC03000548.hg.1	4.41	JUC03005025.hg.1	DZIP3
TC03000548.hg.1	2.93	PSR03010057.hg.1	DZIP3
TC03000548.hg.1	2.27	JUC03004997.hg.1	DZIP3
TC03000548.hg.1	2.03	JUC03005013.hg.1	DZIP3
TC03000548.hg.1	-2.26	JUC03005021.hg.1	DZIP3
TC03000548.hg.1	-3.08	JUC03005005.hg.1	DZIP3
TC07000679.hg.1	4.41	JUC07004920.hg.1	MLL5
TC07000679.hg.1	2.42	JUC07004924.hg.1	MLL5
TC07000679.hg.1	2.06	JUC07004901.hg.1	MLL5
TC07000679.hg.1	-2.32	PSR07010519.hg.1	MLL5
TC07003401.hg.1	4.41	JUC07021995.hg.1	HOXA9, MIR'
TC07003401.hg.1	3.71	PSR07019190.hg.1	HOXA9, MIR'
TC07003401.hg.1	3.48	PSR07019143.hg.1	HOXA9, MIR'
TC07003401.hg.1	3.35	JUC07021990.hg.1	HOXA9, MIR'
TC07003401.hg.1	3.31	PSR07019167.hg.1	HOXA9, MIR'
TC07003401.hg.1	3.11	JUC07021991.hg.1	HOXA9, MIR'
TC07003401.hg.1	2.91	PSR07019166.hg.1	HOXA9, MIR'
TC07003401.hg.1	2.85	JUC07021996.hg.1	HOXA9, MIR'
TC07003401.hg.1	2.83	JUC07021997.hg.1	HOXA9, MIR'
TC07003401.hg.1	2.7	JUC07021994.hg.1	HOXA9, MIR'
TC07003401.hg.1	2.61	PSR07019134.hg.1	HOXA9, MIR'
TC07003401.hg.1	2.4	JUC07021988.hg.1	HOXA9, MIR'
TC07003401.hg.1	2.26	PSR07019154.hg.1	HOXA9, MIR'
TC07003401.hg.1	-3.02	PSR07019162.hg.1	HOXA9, MIR'
TC08000132.hg.1	4.41	JUC08000665.hg.1	
TC08000132.hg.1	2.94	PSR08001246.hg.1	
TC08000132.hg.1	2.44	PSR08001244.hg.1	
TC08000132.hg.1	2.11	PSR08001245.hg.1	

TC14000419.hg.1	4.41	PSR14005084.hg.1	SMOC1
TC14000419.hg.1	3.6	JUC14002604.hg.1	SMOC1
TC14000419.hg.1	3.5	PSR14005085.hg.1	SMOC1
TC14000419.hg.1	3.29	PSR14005086.hg.1	SMOC1
TC01003073.hg.1	4.4	JUC01025633.hg.1	
TC01003073.hg.1	3.36	JUC01025628.hg.1	
TC01003073.hg.1	2.42	PSR01047756.hg.1	
TC01003073.hg.1	2.33	JUC01025632.hg.1	
TC01003073.hg.1	2.26	PSR01047745.hg.1	
TC01003073.hg.1	2.11	JUC01025622.hg.1	
TC01003073.hg.1	2.04	PSR01047757.hg.1	
TC01003073.hg.1	-2.44	PSR01047749.hg.1	
TC01003073.hg.1	-2.53	PSR01047743.hg.1	
TC03001105.hg.1	4.4	JUC03009623.hg.1	MFI2-AS1
TC03001105.hg.1	2.62	PSR03019064.hg.1	MFI2-AS1
TC07001738.hg.1	4.4	JUC07013261.hg.1	NAMPT
TC07001738.hg.1	3.38	PSR07027039.hg.1	NAMPT
TC07001738.hg.1	2.73	PSR07027038.hg.1	NAMPT
TC07001738.hg.1	2.73	PSR07027047.hg.1	NAMPT
TC07001738.hg.1	2.72	PSR07027028.hg.1	NAMPT
TC07001738.hg.1	2.67	PSR07027058.hg.1	NAMPT
TC07001738.hg.1	2.22	PSR07027030.hg.1	NAMPT
TC07001738.hg.1	2.14	PSR07027056.hg.1	NAMPT
TC07001738.hg.1	-2.07	JUC07013253.hg.1	NAMPT
TC10001681.hg.1	4.4	JUC10012876.hg.1	HSPA12A
TC10001681.hg.1	3.1	JUC10012859.hg.1	HSPA12A
TC10001681.hg.1	2.75	JUC10012874.hg.1	HSPA12A
TC10001681.hg.1	-2.09	PSR10022164.hg.1	HSPA12A
TC10001681.hg.1	-2.3	PSR10022165.hg.1	HSPA12A
TC01000644.hg.1	4.39	JUC01005409.hg.1	FAM159A
TC01000644.hg.1	3.41	PSR01010508.hg.1	FAM159A
TC01000644.hg.1	-2.6	PSR01010510.hg.1	FAM159A
TC01000644.hg.1	-2.66	PSR01010507.hg.1	FAM159A
TC01000644.hg.1	-2.88	PSR01010515.hg.1	FAM159A
TC01000644.hg.1	-6	JUC01005413.hg.1	FAM159A
TC01003778.hg.1	4.39	JUC01030251.hg.1	PLXNA2
TC01003778.hg.1	3.54	JUC01030274.hg.1	PLXNA2
TC01003778.hg.1	3.13	JUC01030263.hg.1	PLXNA2
TC01003778.hg.1	3.04	JUC01030266.hg.1	PLXNA2
TC01003778.hg.1	2.84	JUC01030255.hg.1	PLXNA2
TC01003778.hg.1	2.47	JUC01030254.hg.1	PLXNA2
TC01003778.hg.1	2.4	PSR01057911.hg.1	PLXNA2
TC01003778.hg.1	2.34	PSR01057890.hg.1	PLXNA2
TC01003778.hg.1	2.34	JUC01030265.hg.1	PLXNA2
TC01003778.hg.1	2.26	JUC01030259.hg.1	PLXNA2
TC01003778.hg.1	2.2	PSR01057903.hg.1	PLXNA2
TC01003778.hg.1	2.15	PSR01057921.hg.1	PLXNA2
TC01003778.hg.1	2.13	PSR01057915.hg.1	PLXNA2
TC01003778.hg.1	2.12	PSR01057914.hg.1	PLXNA2
TC01003778.hg.1	2.12	PSR01057918.hg.1	PLXNA2
TC01003778.hg.1	2.06	PSR01057930.hg.1	PLXNA2
TC01003778.hg.1	2.04	PSR01057912.hg.1	PLXNA2
TC01003778.hg.1	-2	PSR01057927.hg.1	PLXNA2
TC01003778.hg.1	-2.08	PSR01057906.hg.1	PLXNA2
TC01003778.hg.1	-2.51	JUC01030267.hg.1	PLXNA2
TC01003778.hg.1	-5.69	JUC01030270.hg.1	PLXNA2
TC03002141.hg.1	4.39	JUC03018723.hg.1	ACAP2
TC03002141.hg.1	2.65	JUC03018750.hg.1	ACAP2

TC03002141.hg.1	2.46	JUC03018759.hg.1	ACAP2
TC03002141.hg.1	2.45	JUC03018736.hg.1	ACAP2
TC03002141.hg.1	2.12	JUC03018755.hg.1	ACAP2
TC03002141.hg.1	2.01	PSR03037798.hg.1	ACAP2
TC0X000943.hg.1	4.39	JUC0X006353.hg.1	DMD
TC0X000943.hg.1	4.2	PSR0X012609.hg.1	DMD
TC0X000943.hg.1	2.88	JUC0X006249.hg.1	DMD
TC0X000943.hg.1	2.67	JUC0X006248.hg.1	DMD
TC0X000943.hg.1	2.36	JUC0X006301.hg.1	DMD
TC0X000943.hg.1	2.36	JUC0X006317.hg.1	DMD
TC0X000943.hg.1	2.34	JUC0X006321.hg.1	DMD
TC0X000943.hg.1	2.25	JUC0X006329.hg.1	DMD
TC0X000943.hg.1	2.24	JUC0X006342.hg.1	DMD
TC0X000943.hg.1	2.21	JUC0X006276.hg.1	DMD
TC0X000943.hg.1	2.11	PSR0X012611.hg.1	DMD
TC0X000943.hg.1	2.07	JUC0X006298.hg.1	DMD
TC0X000943.hg.1	2.03	JUC0X006311.hg.1	DMD
TC0X000943.hg.1	-2.02	JUC0X006305.hg.1	DMD
TC0X000943.hg.1	-2.04	PSR0X012579.hg.1	DMD
TC0X000943.hg.1	-2.07	JUC0X006309.hg.1	DMD
TC0X000943.hg.1	-2.2	JUC0X006275.hg.1	DMD
TC0X000943.hg.1	-2.26	JUC0X006264.hg.1	DMD
TC0X000943.hg.1	-2.27	JUC0X006307.hg.1	DMD
TC0X000943.hg.1	-2.28	JUC0X006289.hg.1	DMD
TC0X000943.hg.1	-2.31	PSR0X012591.hg.1	DMD
TC0X000943.hg.1	-2.33	JUC0X006345.hg.1	DMD
TC0X000943.hg.1	-2.44	JUC0X006304.hg.1	DMD
TC0X000943.hg.1	-2.5	PSR0X012593.hg.1	DMD
TC0X000943.hg.1	-2.56	JUC0X006322.hg.1	DMD
TC0X000943.hg.1	-2.67	PSR0X012631.hg.1	DMD
TC0X000943.hg.1	-2.68	JUC0X006262.hg.1	DMD
TC0X000943.hg.1	-2.69	PSR0X012648.hg.1	DMD
TC0X000943.hg.1	-2.72	JUC0X006355.hg.1	DMD
TC0X000943.hg.1	-2.76	PSR0X012625.hg.1	DMD
TC0X000943.hg.1	-2.88	PSR0X012698.hg.1	DMD
TC0X000943.hg.1	-2.9	PSR0X012604.hg.1	DMD
TC0X000943.hg.1	-2.93	PSR0X012605.hg.1	DMD
TC0X000943.hg.1	-3.15	PSR0X012699.hg.1	DMD
TC0X000943.hg.1	-3.55	PSR0X012692.hg.1	DMD
TC0X000943.hg.1	-3.79	JUC0X006346.hg.1	DMD
TC0X000943.hg.1	-4.12	JUC0X006250.hg.1	DMD
TC0X000943.hg.1	-5.03	PSR0X012569.hg.1	DMD
TC0X000943.hg.1	-10.91	JUC0X006277.hg.1	DMD
TC22000394.hg.1	4.39	JUC22002903.hg.1	GTSE1
TC22000394.hg.1	2.36	PSR22007633.hg.1	GTSE1
TC22000394.hg.1	2.35	PSR22007631.hg.1	GTSE1
TC22000394.hg.1	2.15	JUC22002900.hg.1	GTSE1
TC01002649.hg.1	4.38	PSR01041454.hg.1	EPS15
TC01002649.hg.1	2.26	JUC01022169.hg.1	EPS15
TC01002649.hg.1	-2.06	JUC01022159.hg.1	EPS15
TC02002399.hg.1	4.38	JUC02019655.hg.1	ZEB2, ZEB2-/
TC02002399.hg.1	2.3	PSR02037863.hg.1	ZEB2, ZEB2-/
TC02002399.hg.1	2.17	JUC02019669.hg.1	ZEB2, ZEB2-/
TC02002399.hg.1	2.1	JUC02019677.hg.1	ZEB2, ZEB2-/
TC10000565.hg.1	4.38	JUC10003523.hg.1	ZMIZ1
TC10000565.hg.1	3.37	JUC10003504.hg.1	ZMIZ1
TC10000565.hg.1	3.03	JUC10003507.hg.1	ZMIZ1
TC10000565.hg.1	2.89	JUC10003529.hg.1	ZMIZ1

TC10000565.hg.1	2.84	JUC10003506.hg.1	ZMIZ1
TC10000565.hg.1	2.71	PSR10006066.hg.1	ZMIZ1
TC10000565.hg.1	2.53	JUC10003533.hg.1	ZMIZ1
TC10000565.hg.1	2.24	PSR10006060.hg.1	ZMIZ1
TC10000565.hg.1	2.13	PSR10006042.hg.1	ZMIZ1
TC10000565.hg.1	2.08	JUC10003535.hg.1	ZMIZ1
TC12000974.hg.1	4.38	JUC12006982.hg.1	KNTC1
TC12000974.hg.1	2.66	JUC12007025.hg.1	KNTC1
TC12000974.hg.1	2.08	PSR12012791.hg.1	KNTC1
TC12000974.hg.1	2.05	JUC12007024.hg.1	KNTC1
TC12000974.hg.1	2.03	JUC12006976.hg.1	KNTC1
TC12000974.hg.1	-2.17	JUC12007023.hg.1	KNTC1
TC12000974.hg.1	-2.48	JUC12006993.hg.1	KNTC1
TC12000974.hg.1	-2.51	JUC12006968.hg.1	KNTC1
TC12000974.hg.1	-2.63	JUC12006994.hg.1	KNTC1
TC12000974.hg.1	-2.74	JUC12007020.hg.1	KNTC1
TC12000974.hg.1	-2.81	JUC12006998.hg.1	KNTC1
TC12000974.hg.1	-2.85	JUC12006997.hg.1	KNTC1
TC03001888.hg.1	4.37	PSR03034034.hg.1	TM4SF1
TC03001888.hg.1	-6.27	JUC03016905.hg.1	TM4SF1
TC05000914.hg.1	4.37	PSR05013205.hg.1	ODZ2
TC05000914.hg.1	3.87	JUC05006819.hg.1	ODZ2
TC05000914.hg.1	3.71	PSR05013204.hg.1	ODZ2
TC05000914.hg.1	3.27	PSR05013235.hg.1	ODZ2
TC05000914.hg.1	3.21	JUC05006848.hg.1	ODZ2
TC05000914.hg.1	3	JUC05006816.hg.1	ODZ2
TC05000914.hg.1	2.56	PSR05013206.hg.1	ODZ2
TC05000914.hg.1	2.54	JUC05006855.hg.1	ODZ2
TC05000914.hg.1	2.47	JUC05006825.hg.1	ODZ2
TC05000914.hg.1	2.42	JUC05006844.hg.1	ODZ2
TC05000914.hg.1	2.4	JUC05006820.hg.1	ODZ2
TC05000914.hg.1	2.33	PSR05013219.hg.1	ODZ2
TC05000914.hg.1	2.29	PSR05013214.hg.1	ODZ2
TC05000914.hg.1	2.02	JUC05006834.hg.1	ODZ2
TC05000914.hg.1	-2.39	JUC05006857.hg.1	ODZ2
TC05003446.hg.1	4.37	JUC05019878.hg.1	SMA5
TC05003446.hg.1	-2.76	PSR05019978.hg.1	SMA5
TC06000762.hg.1	4.37	JUC06004242.hg.1	BCKDHB
TC06000762.hg.1	-2.01	PSR06009137.hg.1	BCKDHB
TC22000688.hg.1	4.37	JUC22005004.hg.1	LARGE
TC22000688.hg.1	2.94	PSR22012185.hg.1	LARGE
TC22000688.hg.1	2.64	JUC22005007.hg.1	LARGE
TC22000688.hg.1	2.5	PSR22012178.hg.1	LARGE
TC22000688.hg.1	2.14	PSR22012172.hg.1	LARGE
TC22000688.hg.1	2.11	PSR22012176.hg.1	LARGE
TC22000688.hg.1	2.01	PSR22012170.hg.1	LARGE
TC22000688.hg.1	-2.14	JUC22005020.hg.1	LARGE
TC01003996.hg.1	4.36	JUC01031624.hg.1	LYST
TC01003996.hg.1	3.42	JUC01031668.hg.1	LYST
TC01003996.hg.1	3.17	JUC01031623.hg.1	LYST
TC01003996.hg.1	2.9	JUC01031630.hg.1	LYST
TC01003996.hg.1	2.6	JUC01031658.hg.1	LYST
TC01003996.hg.1	2.6	JUC01031667.hg.1	LYST
TC01003996.hg.1	2.17	JUC01031617.hg.1	LYST
TC01003996.hg.1	2.14	PSR01060519.hg.1	LYST
TC01003996.hg.1	2.05	PSR01060503.hg.1	LYST
TC01003996.hg.1	-2.29	JUC01031643.hg.1	LYST
TC01003996.hg.1	-3.25	JUC01031650.hg.1	LYST

TC01003996.hg.1	-4.61	JUC01031663.hg.1	LYST
TC02001326.hg.1	4.36	JUC02010997.hg.1	DES
TC02001326.hg.1	2.69	PSR02020642.hg.1	DES
TC02001326.hg.1	2.25	JUC02011001.hg.1	DES
TC02001326.hg.1	-2.17	PSR02020641.hg.1	DES
TC02002900.hg.1	4.36	JUC02024351.hg.1	IQCA1
TC02002900.hg.1	-2.37	PSR02046547.hg.1	IQCA1
TC02002900.hg.1	-3.63	JUC02024346.hg.1	IQCA1
TC02002900.hg.1	-3.88	JUC02024358.hg.1	IQCA1
TC05000697.hg.1	4.36	PSR05009662.hg.1	KIF20A
TC05000697.hg.1	3.35	JUC05005151.hg.1	KIF20A
TC05000697.hg.1	3.12	PSR05009671.hg.1	KIF20A
TC05000697.hg.1	2.63	PSR05009664.hg.1	KIF20A
TC05000697.hg.1	2.23	PSR05009673.hg.1	KIF20A
TC05000697.hg.1	2.19	PSR05009672.hg.1	KIF20A
TC05000697.hg.1	2.15	PSR05009674.hg.1	KIF20A
TC05000697.hg.1	-2.3	JUC05005156.hg.1	KIF20A
TC05000697.hg.1	-2.39	JUC05005154.hg.1	KIF20A
TC13000173.hg.1	4.36	JUC13001212.hg.1	TPT1-AS1
TC13000173.hg.1	3.1	JUC13001219.hg.1	TPT1-AS1
TC13000173.hg.1	2.42	JUC13001222.hg.1	TPT1-AS1
TC13000173.hg.1	2.04	PSR13001959.hg.1	TPT1-AS1
TC13000173.hg.1	2.04	JUC13001214.hg.1	TPT1-AS1
TC01001476.hg.1	4.35	JUC01012082.hg.1	C1orf112
TC01001476.hg.1	2.65	JUC01012059.hg.1	C1orf112
TC01001476.hg.1	2.27	PSR01022921.hg.1	C1orf112
TC01001476.hg.1	-2	PSR01022896.hg.1	C1orf112
TC01001476.hg.1	-2.06	JUC01012062.hg.1	C1orf112
TC01001476.hg.1	-2.09	PSR01022898.hg.1	C1orf112
TC01001476.hg.1	-2.14	JUC01012072.hg.1	C1orf112
TC01001476.hg.1	-2.15	JUC01012058.hg.1	C1orf112
TC01001476.hg.1	-2.39	JUC01012077.hg.1	C1orf112
TC01001476.hg.1	-2.56	PSR01022908.hg.1	C1orf112
TC01001476.hg.1	-3.14	PSR01022913.hg.1	C1orf112
TC01001476.hg.1	-3.46	PSR01022885.hg.1	C1orf112
TC01001476.hg.1	-3.49	JUC01012083.hg.1	C1orf112
TC08001660.hg.1	4.35	JUC08011101.hg.1	ST3GAL1
TC08001660.hg.1	-2.27	PSR08021724.hg.1	ST3GAL1
TC08001660.hg.1	-3.04	JUC08011086.hg.1	ST3GAL1
TC01002981.hg.1	4.34	JUC01024897.hg.1	DENND2D
TC01002981.hg.1	2.93	JUC01024900.hg.1	DENND2D
TC01002981.hg.1	2.14	PSR01046282.hg.1	DENND2D
TC08001164.hg.1	4.34	JUC08007637.hg.1	ZMAT4
TC08001164.hg.1	4.15	JUC08007640.hg.1	ZMAT4
TC08001164.hg.1	3.57	PSR08014969.hg.1	ZMAT4
TC08001164.hg.1	3.41	PSR08014958.hg.1	ZMAT4
TC08001164.hg.1	2.25	PSR08014971.hg.1	ZMAT4
TC08001164.hg.1	2.16	PSR08014965.hg.1	ZMAT4
TC08001164.hg.1	2.15	JUC08007648.hg.1	ZMAT4
TC08001164.hg.1	-2.11	PSR08014954.hg.1	ZMAT4
TC08001164.hg.1	-2.15	JUC08007641.hg.1	ZMAT4
TC08001164.hg.1	-2.2	PSR08014962.hg.1	ZMAT4
TC08001164.hg.1	-2.45	JUC08007646.hg.1	ZMAT4
TC08001164.hg.1	-4.28	JUC08007638.hg.1	ZMAT4
TC11000228.hg.1	4.34	JUC11001435.hg.1	NUCB2
TC11000228.hg.1	3.25	PSR11002965.hg.1	NUCB2
TC11000228.hg.1	2.98	PSR11002972.hg.1	NUCB2
TC11000228.hg.1	2.87	PSR11002971.hg.1	NUCB2

TC11000228.hg.1	2.87	PSR11002995.hg.1	NUCB2
TC11000228.hg.1	2.56	PSR11002966.hg.1	NUCB2
TC11000228.hg.1	2.45	JUC11001448.hg.1	NUCB2
TC11000228.hg.1	2.35	PSR11002980.hg.1	NUCB2
TC11000228.hg.1	2.33	PSR11002967.hg.1	NUCB2
TC11000228.hg.1	2.22	PSR11002997.hg.1	NUCB2
TC11000228.hg.1	2.13	PSR11002975.hg.1	NUCB2
TC11000228.hg.1	2.12	JUC11001451.hg.1	NUCB2
TC11000228.hg.1	2.07	JUC11001442.hg.1	NUCB2
TC11000228.hg.1	2	PSR11002994.hg.1	NUCB2
TC02002765.hg.1	4.33	PSR02044435.hg.1	TNS1
TC02002765.hg.1	-2.06	PSR02044387.hg.1	TNS1
TC02002765.hg.1	-2.06	JUC02023285.hg.1	TNS1
TC02002765.hg.1	-2.07	PSR02044405.hg.1	TNS1
TC02002765.hg.1	-2.1	PSR02044390.hg.1	TNS1
TC02002765.hg.1	-2.23	JUC02023306.hg.1	TNS1
TC02002765.hg.1	-2.24	PSR02044439.hg.1	TNS1
TC02002765.hg.1	-2.36	JUC02023297.hg.1	TNS1
TC02002765.hg.1	-2.39	PSR02044412.hg.1	TNS1
TC02002765.hg.1	-2.42	PSR02044443.hg.1	TNS1
TC02002765.hg.1	-2.43	PSR02044445.hg.1	TNS1
TC02002765.hg.1	-2.46	JUC02023313.hg.1	TNS1
TC02002765.hg.1	-2.48	JUC02023291.hg.1	TNS1
TC02002765.hg.1	-2.54	PSR02044395.hg.1	TNS1
TC02002765.hg.1	-2.97	JUC02023319.hg.1	TNS1
TC02002765.hg.1	-3.06	PSR02044415.hg.1	TNS1
TC02002765.hg.1	-3.14	PSR02044444.hg.1	TNS1
TC02002765.hg.1	-3.32	JUC02023286.hg.1	TNS1
TC02002765.hg.1	-3.56	PSR02044442.hg.1	TNS1
TC02002765.hg.1	-3.57	JUC02023321.hg.1	TNS1
TC02002765.hg.1	-4.25	JUC02023310.hg.1	TNS1
TC05001804.hg.1	4.33	PSR05024563.hg.1	CXCL14
TC05001804.hg.1	4.02	JUC05012596.hg.1	CXCL14
TC05001804.hg.1	2.39	PSR05024559.hg.1	CXCL14
TC05001804.hg.1	2.27	PSR05024565.hg.1	CXCL14
TC05001804.hg.1	2.26	JUC05012598.hg.1	CXCL14
TC05001804.hg.1	-3.92	JUC05012597.hg.1	CXCL14
TC05001804.hg.1	-12.21	PSR05024567.hg.1	CXCL14
TC05001804.hg.1	-26.65	PSR05024568.hg.1	CXCL14
TC05001804.hg.1	-33.11	PSR05024569.hg.1	CXCL14
TC09000576.hg.1	4.33	JUC09003558.hg.1	RGS3
TC09000576.hg.1	4.3	PSR09006457.hg.1	RGS3
TC09000576.hg.1	3.98	JUC09003578.hg.1	RGS3
TC09000576.hg.1	3.73	JUC09003559.hg.1	RGS3
TC09000576.hg.1	3.64	PSR09006430.hg.1	RGS3
TC09000576.hg.1	3.63	PSR09006444.hg.1	RGS3
TC09000576.hg.1	3.47	PSR09006441.hg.1	RGS3
TC09000576.hg.1	3.36	PSR09006465.hg.1	RGS3
TC09000576.hg.1	3.35	JUC09003590.hg.1	RGS3
TC09000576.hg.1	3.27	JUC09003597.hg.1	RGS3
TC09000576.hg.1	3.11	JUC09003581.hg.1	RGS3
TC09000576.hg.1	2.86	PSR09006420.hg.1	RGS3
TC09000576.hg.1	2.72	PSR09006418.hg.1	RGS3
TC09000576.hg.1	2.61	JUC09003574.hg.1	RGS3
TC09000576.hg.1	2.39	PSR09006416.hg.1	RGS3
TC09000576.hg.1	2.39	PSR09006423.hg.1	RGS3
TC09000576.hg.1	2.33	PSR09006454.hg.1	RGS3
TC09000576.hg.1	2.33	PSR09006458.hg.1	RGS3

TC09000576.hg.1	2.3	PSR09006448.hg.1	RGS3
TC09000576.hg.1	2.25	PSR09006428.hg.1	RGS3
TC09000576.hg.1	2.24	PSR09006434.hg.1	RGS3
TC09000576.hg.1	2.24	PSR09006437.hg.1	RGS3
TC09000576.hg.1	2.2	PSR09006432.hg.1	RGS3
TC09000576.hg.1	2.2	PSR09006463.hg.1	RGS3
TC09000576.hg.1	2.19	PSR09006443.hg.1	RGS3
TC09000576.hg.1	2.15	PSR09006478.hg.1	RGS3
TC09000576.hg.1	2.14	PSR09006445.hg.1	RGS3
TC09000576.hg.1	2.14	PSR09006490.hg.1	RGS3
TC09000576.hg.1	2.11	JUC09003595.hg.1	RGS3
TC09000576.hg.1	2.1	JUC09003569.hg.1	RGS3
TC09000576.hg.1	2.08	PSR09006422.hg.1	RGS3
TC09000576.hg.1	2.08	PSR09006455.hg.1	RGS3
TC09000576.hg.1	2.03	PSR09006451.hg.1	RGS3
TC09000576.hg.1	2.02	PSR09006447.hg.1	RGS3
TC09000576.hg.1	2.02	PSR09006450.hg.1	RGS3
TC09000576.hg.1	-2.51	JUC09003557.hg.1	RGS3
TC09000576.hg.1	-3.69	PSR09006470.hg.1	RGS3
TC12001170.hg.1	4.33	PSR12015582.hg.1	SLC2A3
TC12001170.hg.1	3.89	PSR12015558.hg.1	SLC2A3
TC12001170.hg.1	3.69	PSR12015571.hg.1	SLC2A3
TC12001170.hg.1	3.22	PSR12015567.hg.1	SLC2A3
TC12001170.hg.1	3.03	PSR12015569.hg.1	SLC2A3
TC12001170.hg.1	2.76	JUC12008575.hg.1	SLC2A3
TC12001170.hg.1	2.33	PSR12015565.hg.1	SLC2A3
TC12001170.hg.1	2.16	PSR12015559.hg.1	SLC2A3
TC12001170.hg.1	-2.41	PSR12015561.hg.1	SLC2A3
TC12001170.hg.1	-3.21	JUC12008574.hg.1	SLC2A3
TC06000323.hg.1	4.32	JUC06001161.hg.1	HLA-F
TC06000323.hg.1	3.32	PSR06002535.hg.1	HLA-F
TC06000323.hg.1	3.16	PSR06002559.hg.1	HLA-F
TC06000323.hg.1	2.92	JUC06001169.hg.1	HLA-F
TC06000323.hg.1	2.8	JUC06001152.hg.1	HLA-F
TC06000323.hg.1	2.68	JUC06001174.hg.1	HLA-F
TC06000323.hg.1	2.63	PSR06002549.hg.1	HLA-F
TC06000323.hg.1	2.38	PSR06002587.hg.1	HLA-F
TC06000323.hg.1	2.27	JUC06001156.hg.1	HLA-F
TC06000323.hg.1	2.26	JUC06001162.hg.1	HLA-F
TC06000323.hg.1	2.15	PSR06002589.hg.1	HLA-F
TC06000323.hg.1	2.1	JUC06001153.hg.1	HLA-F
TC06000323.hg.1	2.07	PSR06002591.hg.1	HLA-F
TC06000323.hg.1	2.02	PSR06002582.hg.1	HLA-F
TC06000323.hg.1	2	PSR06002585.hg.1	HLA-F
TC06000323.hg.1	-2	PSR06002552.hg.1	HLA-F
TC07000643.hg.1	4.32	PSR07010040.hg.1	SERPINE1
TC07000643.hg.1	4.12	PSR07010036.hg.1	SERPINE1
TC07000643.hg.1	3.69	PSR07010023.hg.1	SERPINE1
TC07000643.hg.1	2.16	PSR07010038.hg.1	SERPINE1
TC07000643.hg.1	-4.69	JUC07004647.hg.1	SERPINE1
TC08000298.hg.1	4.32	JUC08002114.hg.1	ADAM9
TC08000298.hg.1	-2.13	PSR08004236.hg.1	ADAM9
TC09000857.hg.1	4.32	PSR09011428.hg.1	FAM157B
TC09000857.hg.1	3.39	JUC09006159.hg.1	FAM157B
TC09000857.hg.1	2.82	JUC09006162.hg.1	FAM157B
TC09000857.hg.1	2.27	PSR09011430.hg.1	FAM157B
TC09000857.hg.1	2.08	PSR09011433.hg.1	FAM157B
TC11000252.hg.1	4.32	JUC11001689.hg.1	NAV2

TC11000252.hg.1	3.46	PSR11003309.hg.1	NAV2
TC11000252.hg.1	2.79	JUC11001652.hg.1	NAV2
TC11000252.hg.1	2.27	PSR11003331.hg.1	NAV2
TC11000252.hg.1	2.27	PSR11003335.hg.1	NAV2
TC11000252.hg.1	2.25	JUC11001674.hg.1	NAV2
TC11000252.hg.1	2.24	JUC11001690.hg.1	NAV2
TC11000252.hg.1	2.08	PSR11003334.hg.1	NAV2
TC11000252.hg.1	-2.16	JUC11001677.hg.1	NAV2
TC19000256.hg.1	4.32	JUC19002149.hg.1	CD97
TC19000256.hg.1	3.31	JUC19002165.hg.1	CD97
TC19000256.hg.1	2.8	JUC19002158.hg.1	CD97
TC19000256.hg.1	2.3	JUC19002159.hg.1	CD97
TC19000256.hg.1	2.26	PSR19003674.hg.1	CD97
TC19000256.hg.1	-2.11	JUC19002162.hg.1	CD97
TC01006336.hg.1	4.31	JUC01044211.hg.1	CADM3
TC01006336.hg.1	2.79	JUC01044209.hg.1	CADM3
TC01006336.hg.1	2.65	PSR01021163.hg.1	CADM3
TC01006336.hg.1	2.51	JUC01044204.hg.1	CADM3
TC01006336.hg.1	2.04	PSR01021168.hg.1	CADM3
TC01006336.hg.1	-2.28	PSR01021161.hg.1	CADM3
TC01006336.hg.1	-2.4	JUC01044210.hg.1	CADM3
TC01006336.hg.1	-2.5	JUC01044208.hg.1	CADM3
TC01006336.hg.1	-3.21	PSR01021160.hg.1	CADM3
TC01006336.hg.1	-3.47	JUC01044214.hg.1	CADM3
TC01006336.hg.1	-3.49	JUC01044213.hg.1	CADM3
TC01006336.hg.1	-3.59	PSR01021162.hg.1	CADM3
TC01006336.hg.1	-4.68	PSR01021175.hg.1	CADM3
TC01006336.hg.1	-4.91	PSR01021166.hg.1	CADM3
TC05000565.hg.1	4.31	PSR05007844.hg.1	TNFAIP8
TC05000565.hg.1	2.45	JUC05004284.hg.1	TNFAIP8
TC05000565.hg.1	2.17	JUC05004282.hg.1	TNFAIP8
TC05000565.hg.1	-2.36	JUC05004283.hg.1	TNFAIP8
TC01001817.hg.1	4.3	JUC01015252.hg.1	MARK1
TC01001817.hg.1	2.67	PSR01028278.hg.1	MARK1
TC01001817.hg.1	-2.64	PSR01028292.hg.1	MARK1
TC02001103.hg.1	4.3	JUC02008855.hg.1	GULP1
TC02001103.hg.1	2.9	JUC02008852.hg.1	GULP1
TC02001103.hg.1	2.84	PSR02016560.hg.1	GULP1
TC02001103.hg.1	2.6	JUC02008863.hg.1	GULP1
TC02001103.hg.1	2.51	PSR02016563.hg.1	GULP1
TC02001103.hg.1	2.5	PSR02016558.hg.1	GULP1
TC02001103.hg.1	2.49	PSR02016585.hg.1	GULP1
TC02001103.hg.1	2.42	JUC02008865.hg.1	GULP1
TC02001103.hg.1	2.11	PSR02016552.hg.1	GULP1
TC02001103.hg.1	2	PSR02016562.hg.1	GULP1
TC08001686.hg.1	4.3	JUC08011353.hg.1	PTK2, LOC1(
TC08001686.hg.1	4.25	JUC08011418.hg.1	PTK2, LOC1(
TC08001686.hg.1	4.06	JUC08011362.hg.1	PTK2, LOC1(
TC08001686.hg.1	4.02	JUC08011347.hg.1	PTK2, LOC1(
TC08001686.hg.1	3.85	JUC08011407.hg.1	PTK2, LOC1(
TC08001686.hg.1	3.43	JUC08011368.hg.1	PTK2, LOC1(
TC08001686.hg.1	3.15	JUC08011338.hg.1	PTK2, LOC1(
TC08001686.hg.1	3.12	JUC08011405.hg.1	PTK2, LOC1(
TC08001686.hg.1	3.07	PSR08022200.hg.1	PTK2, LOC1(
TC08001686.hg.1	3.01	JUC08011413.hg.1	PTK2, LOC1(
TC08001686.hg.1	2.99	JUC08011344.hg.1	PTK2, LOC1(
TC08001686.hg.1	2.91	PSR08022203.hg.1	PTK2, LOC1(
TC08001686.hg.1	2.82	PSR08022105.hg.1	PTK2, LOC1(

TC08001686.hg.1	2.82	PSR08022227.hg.1	PTK2, LOC10
TC08001686.hg.1	2.81	JUC08011348.hg.1	PTK2, LOC10
TC08001686.hg.1	2.73	PSR08022233.hg.1	PTK2, LOC10
TC08001686.hg.1	2.59	PSR08022234.hg.1	PTK2, LOC10
TC08001686.hg.1	2.58	JUC08011375.hg.1	PTK2, LOC10
TC08001686.hg.1	2.57	PSR08022155.hg.1	PTK2, LOC10
TC08001686.hg.1	2.56	PSR08022211.hg.1	PTK2, LOC10
TC08001686.hg.1	2.54	PSR08022206.hg.1	PTK2, LOC10
TC08001686.hg.1	2.53	PSR08022207.hg.1	PTK2, LOC10
TC08001686.hg.1	2.51	PSR08022235.hg.1	PTK2, LOC10
TC08001686.hg.1	2.5	PSR08022162.hg.1	PTK2, LOC10
TC08001686.hg.1	2.5	JUC08011391.hg.1	PTK2, LOC10
TC08001686.hg.1	2.48	JUC08011377.hg.1	PTK2, LOC10
TC08001686.hg.1	2.47	JUC08011341.hg.1	PTK2, LOC10
TC08001686.hg.1	2.43	PSR08022119.hg.1	PTK2, LOC10
TC08001686.hg.1	2.41	JUC08011423.hg.1	PTK2, LOC10
TC08001686.hg.1	2.39	JUC08011357.hg.1	PTK2, LOC10
TC08001686.hg.1	2.39	JUC08011421.hg.1	PTK2, LOC10
TC08001686.hg.1	2.38	PSR08022111.hg.1	PTK2, LOC10
TC08001686.hg.1	2.36	PSR08022089.hg.1	PTK2, LOC10
TC08001686.hg.1	2.35	JUC08011419.hg.1	PTK2, LOC10
TC08001686.hg.1	2.34	JUC08011359.hg.1	PTK2, LOC10
TC08001686.hg.1	2.31	PSR08022120.hg.1	PTK2, LOC10
TC08001686.hg.1	2.3	PSR08022221.hg.1	PTK2, LOC10
TC08001686.hg.1	2.29	PSR08022070.hg.1	PTK2, LOC10
TC08001686.hg.1	2.29	JUC08011320.hg.1	PTK2, LOC10
TC08001686.hg.1	2.27	PSR08022178.hg.1	PTK2, LOC10
TC08001686.hg.1	2.27	PSR08022222.hg.1	PTK2, LOC10
TC08001686.hg.1	2.25	PSR08022209.hg.1	PTK2, LOC10
TC08001686.hg.1	2.24	PSR08022212.hg.1	PTK2, LOC10
TC08001686.hg.1	2.17	PSR08022091.hg.1	PTK2, LOC10
TC08001686.hg.1	2.17	JUC08011382.hg.1	PTK2, LOC10
TC08001686.hg.1	2.16	PSR08022166.hg.1	PTK2, LOC10
TC08001686.hg.1	2.15	JUC08011372.hg.1	PTK2, LOC10
TC08001686.hg.1	2.12	PSR08022214.hg.1	PTK2, LOC10
TC08001686.hg.1	2.11	PSR08022116.hg.1	PTK2, LOC10
TC08001686.hg.1	2.11	PSR08022175.hg.1	PTK2, LOC10
TC08001686.hg.1	2.08	JUC08011330.hg.1	PTK2, LOC10
TC08001686.hg.1	2.07	PSR08022077.hg.1	PTK2, LOC10
TC08001686.hg.1	2.05	PSR08022195.hg.1	PTK2, LOC10
TC08001686.hg.1	2.04	PSR08022143.hg.1	PTK2, LOC10
TC08001686.hg.1	2.01	PSR08022191.hg.1	PTK2, LOC10
TC08001686.hg.1	2.01	PSR08022218.hg.1	PTK2, LOC10
TC08001686.hg.1	-2.01	JUC08011385.hg.1	PTK2, LOC10
TC08001686.hg.1	-3.66	PSR08022108.hg.1	PTK2, LOC10
TC08001686.hg.1	-16.69	JUC08011387.hg.1	PTK2, LOC10
TC10000488.hg.1	4.3	JUC10003430.hg.1	C10orf11
TC10000488.hg.1	4.26	PSR10005851.hg.1	C10orf11
TC10000488.hg.1	4.16	PSR10005853.hg.1	C10orf11
TC10000488.hg.1	4.02	JUC10003444.hg.1	C10orf11
TC10000488.hg.1	3.85	PSR10005854.hg.1	C10orf11
TC10000488.hg.1	3.38	PSR10005857.hg.1	C10orf11
TC10000488.hg.1	3.32	PSR10005859.hg.1	C10orf11
TC10000488.hg.1	3.3	JUC10003439.hg.1	C10orf11
TC10000488.hg.1	3.09	JUC10003436.hg.1	C10orf11
TC10000488.hg.1	3.01	PSR10005856.hg.1	C10orf11
TC10000488.hg.1	2.84	PSR10005850.hg.1	C10orf11
TC10000488.hg.1	2.82	JUC10003431.hg.1	C10orf11

TC10000488.hg.1	2.75	JUC10003437.hg.1	C10orf11
TC10000488.hg.1	2.58	PSR10005852.hg.1	C10orf11
TC10000488.hg.1	2.36	PSR10005865.hg.1	C10orf11
TC10000488.hg.1	2.34	JUC10003446.hg.1	C10orf11
TC10000488.hg.1	2.29	JUC10003441.hg.1	C10orf11
TC17000704.hg.1	4.3	JUC17004999.hg.1	AKAP1
TC17000704.hg.1	3.2	PSR17009189.hg.1	AKAP1
TC17000704.hg.1	3.09	PSR17009198.hg.1	AKAP1
TC17000704.hg.1	2.9	PSR17009188.hg.1	AKAP1
TC17000704.hg.1	2.9	PSR17009199.hg.1	AKAP1
TC17000704.hg.1	2.84	JUC17004990.hg.1	AKAP1
TC17000704.hg.1	2.68	PSR17009191.hg.1	AKAP1
TC17000704.hg.1	2.61	JUC17004996.hg.1	AKAP1
TC17000704.hg.1	2.55	PSR17009194.hg.1	AKAP1
TC17000704.hg.1	2.29	JUC17004985.hg.1	AKAP1
TC17000704.hg.1	2.23	PSR17009186.hg.1	AKAP1
TC17000704.hg.1	2.1	PSR17009187.hg.1	AKAP1
TC17000704.hg.1	-2.65	JUC17004998.hg.1	AKAP1
TC17000704.hg.1	-3.16	JUC17004995.hg.1	AKAP1
TC17001781.hg.1	4.3	JUC17013277.hg.1	CYB561
TC17001781.hg.1	2.14	PSR17023721.hg.1	CYB561
TC17001781.hg.1	2.06	JUC17013278.hg.1	CYB561
TC22000126.hg.1	4.3	JUC22000646.hg.1	GNAZ
TC22000126.hg.1	2.37	PSR22002233.hg.1	GNAZ
TC22000126.hg.1	2.14	PSR22002214.hg.1	GNAZ
TC22000126.hg.1	2.12	JUC22000644.hg.1	GNAZ
TC22000126.hg.1	2.08	PSR22002218.hg.1	GNAZ
TC22000126.hg.1	-3.2	JUC22000649.hg.1	GNAZ
TC22000126.hg.1	-4.01	PSR22002226.hg.1	GNAZ
TC01000892.hg.1	4.29	JUC01007402.hg.1	PALMD, MIR5
TC01000892.hg.1	3.72	PSR01014042.hg.1	PALMD, MIR5
TC01000892.hg.1	2.17	PSR01014043.hg.1	PALMD, MIR5
TC01000892.hg.1	-2.67	JUC01007410.hg.1	PALMD, MIR5
TC01001907.hg.1	4.29	JUC01016003.hg.1	GALNT2
TC01001907.hg.1	4.28	PSR01029600.hg.1	GALNT2
TC01001907.hg.1	2.92	JUC01015999.hg.1	GALNT2
TC01001907.hg.1	2.53	JUC01016006.hg.1	GALNT2
TC01001907.hg.1	2.48	PSR01029618.hg.1	GALNT2
TC01001907.hg.1	2.3	PSR01029620.hg.1	GALNT2
TC01001907.hg.1	-5.01	JUC01015987.hg.1	GALNT2
TC01003271.hg.1	4.29	JUC01026612.hg.1	S100A13
TC01003271.hg.1	3.68	PSR01050359.hg.1	S100A13
TC01003271.hg.1	3.25	JUC01026619.hg.1	S100A13
TC01003271.hg.1	3.05	JUC01026614.hg.1	S100A13
TC01003271.hg.1	2.95	PSR01050360.hg.1	S100A13
TC01003271.hg.1	2.89	PSR01050365.hg.1	S100A13
TC01003271.hg.1	2.77	PSR01050353.hg.1	S100A13
TC01003271.hg.1	2.69	PSR01050352.hg.1	S100A13
TC01003271.hg.1	2.68	PSR01050357.hg.1	S100A13
TC01003271.hg.1	2.55	PSR01050363.hg.1	S100A13
TC01003271.hg.1	2.34	JUC01026608.hg.1	S100A13
TC01003271.hg.1	2.33	PSR01050341.hg.1	S100A13
TC01003271.hg.1	2.33	PSR01050364.hg.1	S100A13
TC01003271.hg.1	2.3	JUC01026615.hg.1	S100A13
TC01003271.hg.1	2.08	JUC01026609.hg.1	S100A13
TC01003271.hg.1	2.04	PSR01050356.hg.1	S100A13
TC06000733.hg.1	4.29	JUC06004012.hg.1	DDX43
TC06000733.hg.1	2.5	PSR06008759.hg.1	DDX43

TC06000733.hg.1	2.09	JUC06004013.hg.1	DDX43
TC06000733.hg.1	-2.52	PSR06008775.hg.1	DDX43
TC06000733.hg.1	-2.71	PSR06008769.hg.1	DDX43
TC06000733.hg.1	-2.83	PSR06008753.hg.1	DDX43
TC06000733.hg.1	-2.9	JUC06004001.hg.1	DDX43
TC06000733.hg.1	-3.06	JUC06004015.hg.1	DDX43
TC06000733.hg.1	-3.37	PSR06008771.hg.1	DDX43
TC06000733.hg.1	-3.42	PSR06008757.hg.1	DDX43
TC06000733.hg.1	-3.57	PSR06008764.hg.1	DDX43
TC06000733.hg.1	-3.64	PSR06008752.hg.1	DDX43
TC06000733.hg.1	-4.94	JUC06004016.hg.1	DDX43
TC07000011.hg.1	4.29	JUC07000038.hg.1	HEATR2
TC07000011.hg.1	3.29	JUC07000046.hg.1	HEATR2
TC07000011.hg.1	2.85	JUC07000048.hg.1	HEATR2
TC07000011.hg.1	2.32	JUC07000036.hg.1	HEATR2
TC07000011.hg.1	2.28	PSR07000075.hg.1	HEATR2
TC07000011.hg.1	2.24	PSR07000058.hg.1	HEATR2
TC07000011.hg.1	2.14	PSR07000083.hg.1	HEATR2
TC07001256.hg.1	4.29	JUC07009513.hg.1	PDE1C
TC07001256.hg.1	3.27	JUC07009509.hg.1	PDE1C
TC07001256.hg.1	3.03	PSR07019605.hg.1	PDE1C
TC07001256.hg.1	2.83	PSR07019604.hg.1	PDE1C
TC07001256.hg.1	2.82	PSR07019602.hg.1	PDE1C
TC07001256.hg.1	2.59	PSR07019597.hg.1	PDE1C
TC07001256.hg.1	2.52	PSR07019577.hg.1	PDE1C
TC07001256.hg.1	2.37	PSR07019596.hg.1	PDE1C
TC07001256.hg.1	2.3	PSR07019606.hg.1	PDE1C
TC07001256.hg.1	2.22	JUC07009516.hg.1	PDE1C
TC07001256.hg.1	2.15	PSR07019599.hg.1	PDE1C
TC07001256.hg.1	-2.19	JUC07009532.hg.1	PDE1C
TC07001563.hg.1	4.29	JUC07011692.hg.1	CACNA2D1
TC07001563.hg.1	3.04	JUC07011714.hg.1	CACNA2D1
TC07001563.hg.1	2.41	JUC07011696.hg.1	CACNA2D1
TC07001563.hg.1	2.07	JUC07011679.hg.1	CACNA2D1
TC07001563.hg.1	2.07	JUC07011694.hg.1	CACNA2D1
TC07001563.hg.1	2.02	PSR07023520.hg.1	CACNA2D1
TC07001563.hg.1	-2.13	JUC07011706.hg.1	CACNA2D1
TC10001721.hg.1	4.29	JUC10013201.hg.1	CPXM2
TC10001721.hg.1	3.62	JUC10013200.hg.1	CPXM2
TC10001721.hg.1	3.1	JUC10013207.hg.1	CPXM2
TC10001721.hg.1	2.73	PSR10022809.hg.1	CPXM2
TC10001721.hg.1	2.49	JUC10013203.hg.1	CPXM2
TC10001721.hg.1	2.45	JUC10013202.hg.1	CPXM2
TC10001721.hg.1	2.41	PSR10022810.hg.1	CPXM2
TC10001721.hg.1	2.33	PSR10022807.hg.1	CPXM2
TC10001721.hg.1	2.29	PSR10022836.hg.1	CPXM2
TC10001721.hg.1	2.22	JUC10013192.hg.1	CPXM2
TC10001721.hg.1	2.19	PSR10022822.hg.1	CPXM2
TC10001721.hg.1	2.15	PSR10022838.hg.1	CPXM2
TC10001721.hg.1	2.08	JUC10013194.hg.1	CPXM2
TC10001721.hg.1	2.06	PSR10022815.hg.1	CPXM2
TC10001721.hg.1	2.05	JUC10013193.hg.1	CPXM2
TC10001721.hg.1	-2.14	PSR10022828.hg.1	CPXM2
TC10001721.hg.1	-2.77	PSR10022816.hg.1	CPXM2
TC10001721.hg.1	-3.4	JUC10013197.hg.1	CPXM2
TC01001498.hg.1	4.28	PSR01023294.hg.1	DNM3
TC01001498.hg.1	3.86	JUC01012286.hg.1	DNM3
TC01001498.hg.1	3.7	PSR01023295.hg.1	DNM3

TC01001498.hg.1	3.49	PSR01023288.hg.1	DNM3
TC01001498.hg.1	-2.06	JUC01012288.hg.1	DNM3
TC01001498.hg.1	-2.18	JUC01012302.hg.1	DNM3
TC01001498.hg.1	-2.69	JUC01012278.hg.1	DNM3
TC09000608.hg.1	4.28	JUC09003881.hg.1	GSN
TC09000608.hg.1	4.03	JUC09003878.hg.1	GSN
TC09000608.hg.1	3.84	PSR09006919.hg.1	GSN
TC09000608.hg.1	3.69	PSR09006917.hg.1	GSN
TC09000608.hg.1	3.25	PSR09006918.hg.1	GSN
TC09000608.hg.1	3.09	PSR09006977.hg.1	GSN
TC09000608.hg.1	3	JUC09003898.hg.1	GSN
TC09000608.hg.1	2.87	PSR09006976.hg.1	GSN
TC09000608.hg.1	2.85	JUC09003899.hg.1	GSN
TC09000608.hg.1	2.52	PSR09006912.hg.1	GSN
TC09000608.hg.1	2.35	PSR09006981.hg.1	GSN
TC09000608.hg.1	2.29	JUC09003874.hg.1	GSN
TC09000608.hg.1	2.25	PSR09006940.hg.1	GSN
TC09000608.hg.1	2.18	PSR09006923.hg.1	GSN
TC09000608.hg.1	2.14	PSR09006941.hg.1	GSN
TC09000608.hg.1	2.12	PSR09006991.hg.1	GSN
TC09000608.hg.1	2.1	PSR09006929.hg.1	GSN
TC09000608.hg.1	2.09	PSR09006921.hg.1	GSN
TC09000608.hg.1	2	JUC09003883.hg.1	GSN
TC09000608.hg.1	-2.06	PSR09006956.hg.1	GSN
TC09000608.hg.1	-2.56	JUC09003897.hg.1	GSN
TC09002903.hg.1	4.28	PSR09005625.hg.1	TMEFF1
TC09002903.hg.1	3.92	JUC09015843.hg.1	TMEFF1
TC09002903.hg.1	3.63	PSR09005621.hg.1	TMEFF1
TC09002903.hg.1	3.31	JUC09015846.hg.1	TMEFF1
TC09002903.hg.1	2.94	PSR09005622.hg.1	TMEFF1
TC09002903.hg.1	2.74	PSR09005633.hg.1	TMEFF1
TC09002903.hg.1	2.14	JUC09015853.hg.1	TMEFF1
TC09002903.hg.1	2.09	JUC09015850.hg.1	TMEFF1
TC09002903.hg.1	2	JUC09015848.hg.1	TMEFF1
TC17001830.hg.1	4.28	JUC17013830.hg.1	ABCA5
TC17001830.hg.1	3.77	JUC17013858.hg.1	ABCA5
TC17001830.hg.1	3.38	JUC17013860.hg.1	ABCA5
TC17001830.hg.1	2.2	PSR17024654.hg.1	ABCA5
TC17001830.hg.1	2.02	PSR17024639.hg.1	ABCA5
TC17001830.hg.1	-2.5	JUC17013826.hg.1	ABCA5
TC17001830.hg.1	-2.56	PSR17024645.hg.1	ABCA5
TC17001830.hg.1	-3.04	PSR17024664.hg.1	ABCA5
TC20000818.hg.1	4.28	JUC20005785.hg.1	DSN1
TC20000818.hg.1	2.44	PSR20011228.hg.1	DSN1
TC20000818.hg.1	2.29	PSR20011226.hg.1	DSN1
TC20000818.hg.1	2	PSR20011219.hg.1	DSN1
TC20000818.hg.1	-2	JUC20005783.hg.1	DSN1
TC20000818.hg.1	-2.41	JUC20005782.hg.1	DSN1
TC09001104.hg.1	4.27	JUC09007883.hg.1	ZNF658B
TC09001104.hg.1	2.59	PSR09014684.hg.1	ZNF658B
TC09001104.hg.1	2.54	PSR09014685.hg.1	ZNF658B
TC09001104.hg.1	2.21	PSR09014686.hg.1	ZNF658B
TC09001104.hg.1	2.19	PSR09014678.hg.1	ZNF658B
TC0X001368.hg.1	4.26	JUC0X009404.hg.1	FRMD7
TC0X001368.hg.1	2.44	PSR0X018371.hg.1	FRMD7
TC0X001368.hg.1	2.26	PSR0X018366.hg.1	FRMD7
TC0X001368.hg.1	-2.31	PSR0X018361.hg.1	FRMD7
TC0X001368.hg.1	-2.59	JUC0X009397.hg.1	FRMD7

TC0X001368.hg.1	-2.93	JUC0X009402.hg.1	FRMD7
TC0X001368.hg.1	-3.07	JUC0X009405.hg.1	FRMD7
TC14001255.hg.1	4.26	JUC14007199.hg.1	ACTN1
TC14001255.hg.1	2.23	PSR14014277.hg.1	ACTN1
TC14001255.hg.1	2.19	JUC14007207.hg.1	ACTN1
TC14001255.hg.1	2.06	JUC14007210.hg.1	ACTN1
TC02002922.hg.1	4.25	JUC02024536.hg.1	HDAC4
TC02002922.hg.1	2.92	JUC02024545.hg.1	HDAC4
TC02002922.hg.1	2.02	JUC02024538.hg.1	HDAC4
TC02002922.hg.1	-2.08	JUC02024546.hg.1	HDAC4
TC02002922.hg.1	-2.12	JUC02024527.hg.1	HDAC4
TC02002922.hg.1	-2.16	PSR02046879.hg.1	HDAC4
TC02002922.hg.1	-2.21	PSR02046850.hg.1	HDAC4
TC02002922.hg.1	-2.72	JUC02024520.hg.1	HDAC4
TC02002922.hg.1	-3.49	PSR02046874.hg.1	HDAC4
TC08002591.hg.1	4.25	JUC08014901.hg.1	PPP2R2A
TC08002591.hg.1	2.77	JUC08014900.hg.1	PPP2R2A
TC08002591.hg.1	2.04	JUC08014885.hg.1	PPP2R2A
TC08002591.hg.1	-2.1	PSR08002735.hg.1	PPP2R2A
TC09000550.hg.1	4.25	JUC09003392.hg.1	AKAP2, PALN
TC09000550.hg.1	3.43	JUC09003381.hg.1	AKAP2, PALN
TC09000550.hg.1	2.27	PSR09006105.hg.1	AKAP2, PALN
TC09000550.hg.1	2.26	JUC09003382.hg.1	AKAP2, PALN
TC09000550.hg.1	2.23	PSR09006117.hg.1	AKAP2, PALN
TC09000550.hg.1	2.05	JUC09003385.hg.1	AKAP2, PALN
TC09000550.hg.1	-3.98	PSR09006144.hg.1	AKAP2, PALN
TC05000471.hg.1	4.23	PSR05006726.hg.1	ERAP2
TC05000471.hg.1	4.18	PSR05006736.hg.1	ERAP2
TC05000471.hg.1	3.81	JUC05003690.hg.1	ERAP2
TC05000471.hg.1	2.88	JUC05003693.hg.1	ERAP2
TC05000471.hg.1	2.62	PSR05006748.hg.1	ERAP2
TC05000471.hg.1	2.37	PSR05006728.hg.1	ERAP2
TC05000471.hg.1	2.24	JUC05003699.hg.1	ERAP2
TC05000471.hg.1	-2.16	PSR05006733.hg.1	ERAP2
TC05000471.hg.1	-2.16	PSR05006755.hg.1	ERAP2
TC05000471.hg.1	-2.29	PSR05006739.hg.1	ERAP2
TC05000471.hg.1	-2.5	PSR05006725.hg.1	ERAP2
TC05000471.hg.1	-2.77	PSR05006738.hg.1	ERAP2
TC05000471.hg.1	-2.82	PSR05006754.hg.1	ERAP2
TC05000471.hg.1	-3.22	PSR05006720.hg.1	ERAP2
TC05000471.hg.1	-3.62	PSR05006753.hg.1	ERAP2
TC05000471.hg.1	-3.92	PSR05006747.hg.1	ERAP2
TC06001772.hg.1	4.23	JUC06010173.hg.1	CYP39A1
TC06001772.hg.1	3.08	JUC06010159.hg.1	CYP39A1
TC06001772.hg.1	2.72	JUC06010158.hg.1	CYP39A1
TC06001772.hg.1	2.51	PSR06021343.hg.1	CYP39A1
TC06001772.hg.1	2.33	PSR06021328.hg.1	CYP39A1
TC06001772.hg.1	-2.04	JUC06010168.hg.1	CYP39A1
TC07003320.hg.1	4.23	PSR07001383.hg.1	GLCCI1
TC07003320.hg.1	4.08	JUC07020823.hg.1	GLCCI1
TC07003320.hg.1	3.42	PSR07001369.hg.1	GLCCI1
TC07003320.hg.1	3.19	PSR07001344.hg.1	GLCCI1
TC07003320.hg.1	3.14	JUC07020827.hg.1	GLCCI1
TC07003320.hg.1	3.01	PSR07001347.hg.1	GLCCI1
TC07003320.hg.1	2.81	PSR07001360.hg.1	GLCCI1
TC07003320.hg.1	2.6	JUC07020821.hg.1	GLCCI1
TC07003320.hg.1	2.6	JUC07020836.hg.1	GLCCI1
TC07003320.hg.1	2.54	PSR07001364.hg.1	GLCCI1

TC07003320.hg.1	2.42	PSR07001356.hg.1	GLCCI1
TC07003320.hg.1	2.41	JUC07020820.hg.1	GLCCI1
TC07003320.hg.1	2.4	PSR07001375.hg.1	GLCCI1
TC07003320.hg.1	2.36	PSR07001382.hg.1	GLCCI1
TC07003320.hg.1	2.08	JUC07020839.hg.1	GLCCI1
TC07003320.hg.1	2.01	PSR07001378.hg.1	GLCCI1
TC11002175.hg.1	4.23	JUC11013825.hg.1	CTSC
TC11002175.hg.1	2.29	JUC11013829.hg.1	CTSC
TC11002175.hg.1	-2.07	JUC11013823.hg.1	CTSC
TC11002175.hg.1	-2.26	PSR11026033.hg.1	CTSC
TC11002175.hg.1	-2.59	PSR11026032.hg.1	CTSC
TC11002175.hg.1	-2.73	JUC11013822.hg.1	CTSC
TC11002175.hg.1	-4.82	JUC11013824.hg.1	CTSC
TC11002175.hg.1	-5.17	PSR11026034.hg.1	CTSC
TC11002175.hg.1	-5.96	JUC11013833.hg.1	CTSC
TC11002433.hg.1	4.23	JUC11015529.hg.1	
TC11002433.hg.1	3.04	PSR11029153.hg.1	
TC11002433.hg.1	2.03	PSR11029151.hg.1	
TC17002910.hg.1	4.23	JUC17019118.hg.1	HOXB6
TC17002910.hg.1	3.53	PSR17022019.hg.1	HOXB6
TC17002910.hg.1	3.38	PSR17022018.hg.1	HOXB6
TC17002910.hg.1	3.36	JUC17019124.hg.1	HOXB6
TC17002910.hg.1	2.93	PSR17022027.hg.1	HOXB6
TC17002910.hg.1	2.87	JUC17019120.hg.1	HOXB6
TC17002910.hg.1	2.76	JUC17019117.hg.1	HOXB6
TC17002910.hg.1	2.64	PSR17022015.hg.1	HOXB6
TC17002910.hg.1	2.45	PSR17022013.hg.1	HOXB6
TC17002910.hg.1	2.28	PSR17022012.hg.1	HOXB6
TC17002910.hg.1	2.1	PSR17022026.hg.1	HOXB6
TC17002910.hg.1	2.07	PSR17022016.hg.1	HOXB6
TC17002910.hg.1	-2.01	JUC17019122.hg.1	HOXB6
TC17002910.hg.1	-2.66	PSR17022021.hg.1	HOXB6
TC21000175.hg.1	4.23	JUC21000861.hg.1	WRB
TC21000175.hg.1	2.5	JUC21000850.hg.1	WRB
TC21000175.hg.1	2.47	JUC21000847.hg.1	WRB
TC21000175.hg.1	2.05	JUC21000859.hg.1	WRB
TC21000175.hg.1	2	JUC21000849.hg.1	WRB
TC21000175.hg.1	-2.06	PSR21001660.hg.1	WRB
TC21000175.hg.1	-2.29	PSR21001661.hg.1	WRB
TC22001467.hg.1	4.23	JUC22009301.hg.1	APOBEC3F
TC22001467.hg.1	3.62	PSR22005804.hg.1	APOBEC3F
TC22001467.hg.1	2.92	PSR22005823.hg.1	APOBEC3F
TC22001467.hg.1	2.83	PSR22005811.hg.1	APOBEC3F
TC22001467.hg.1	2.76	PSR22005805.hg.1	APOBEC3F
TC22001467.hg.1	2.69	PSR22005815.hg.1	APOBEC3F
TC22001467.hg.1	2.38	PSR22005812.hg.1	APOBEC3F
TC22001467.hg.1	2.11	PSR22005817.hg.1	APOBEC3F
TC22001467.hg.1	-2.02	JUC22009298.hg.1	APOBEC3F
TC22001467.hg.1	-2.07	JUC22009305.hg.1	APOBEC3F
TC01002541.hg.1	4.22	JUC01021212.hg.1	PABPC4
TC01002541.hg.1	2.58	PSR01039532.hg.1	PABPC4
TC01002541.hg.1	2.56	PSR01039570.hg.1	PABPC4
TC01002541.hg.1	2.46	PSR01039528.hg.1	PABPC4
TC01002541.hg.1	2.14	PSR01039530.hg.1	PABPC4
TC01002541.hg.1	-3.17	PSR01039560.hg.1	PABPC4
TC01003018.hg.1	4.22	PSR01047110.hg.1	NGF
TC01003018.hg.1	3.58	PSR01047115.hg.1	NGF
TC01003018.hg.1	-2.76	PSR01047113.hg.1	NGF

TC01003018.hg.1	-2.93	JUC01025253.hg.1	NGF
TC03001677.hg.1	4.22	PSR03030222.hg.1	
TC03001677.hg.1	2.92	PSR03030219.hg.1	
TC03001677.hg.1	2.31	PSR03030223.hg.1	
TC03001677.hg.1	2.26	PSR03030221.hg.1	
TC03001677.hg.1	2.24	PSR03030224.hg.1	
TC03001677.hg.1	2.2	PSR03030218.hg.1	
TC03001677.hg.1	2.16	JUC03015015.hg.1	
TC04000432.hg.1	4.22	PSR04006124.hg.1	CXCL13
TC04000432.hg.1	-2.06	JUC04003081.hg.1	CXCL13
TC04000432.hg.1	-5.2	PSR04006122.hg.1	CXCL13
TC04000432.hg.1	-11.27	JUC04003082.hg.1	CXCL13
TC04000432.hg.1	-35.7	PSR04006119.hg.1	CXCL13
TC04001171.hg.1	4.22	JUC04008522.hg.1	NFXL1
TC04001171.hg.1	3.9	PSR04016064.hg.1	NFXL1
TC04001171.hg.1	3.35	PSR04016070.hg.1	NFXL1
TC04001171.hg.1	3.3	JUC04008530.hg.1	NFXL1
TC04001171.hg.1	3.23	PSR04016068.hg.1	NFXL1
TC04001171.hg.1	3.22	JUC04008518.hg.1	NFXL1
TC04001171.hg.1	2.75	PSR04016063.hg.1	NFXL1
TC04001171.hg.1	2.5	PSR04016067.hg.1	NFXL1
TC04001171.hg.1	2.18	PSR04016061.hg.1	NFXL1
TC04001171.hg.1	2.18	JUC04008537.hg.1	NFXL1
TC04001171.hg.1	-2	JUC04008523.hg.1	NFXL1
TC04001171.hg.1	-2.03	PSR04016097.hg.1	NFXL1
TC04001171.hg.1	-2.3	JUC04008540.hg.1	NFXL1
TC05001763.hg.1	4.22	JUC05012321.hg.1	P4HA2
TC05001763.hg.1	2.21	JUC05012311.hg.1	P4HA2
TC05001763.hg.1	-2.1	JUC05012329.hg.1	P4HA2
TC05001763.hg.1	-3.04	PSR05023797.hg.1	P4HA2
TC06001854.hg.1	4.22	JUC06010865.hg.1	LMBRD1
TC06001854.hg.1	2.72	PSR06022501.hg.1	LMBRD1
TC06001854.hg.1	2.71	JUC06010869.hg.1	LMBRD1
TC06001854.hg.1	2.03	PSR06022481.hg.1	LMBRD1
TC07001920.hg.1	4.22	JUC07014753.hg.1	SLC37A3
TC07001920.hg.1	2.1	PSR07029754.hg.1	SLC37A3
TC07001920.hg.1	2.04	PSR07029793.hg.1	SLC37A3
TC07001920.hg.1	-2.18	PSR07029790.hg.1	SLC37A3
TC07001920.hg.1	-2.41	PSR07029785.hg.1	SLC37A3
TC07001920.hg.1	-2.75	JUC07014744.hg.1	SLC37A3
TC01002322.hg.1	4.21	PSR01035672.hg.1	ECE1
TC01002322.hg.1	4.03	PSR01035684.hg.1	ECE1
TC01002322.hg.1	3.67	JUC01019208.hg.1	ECE1
TC01002322.hg.1	2.87	PSR01035694.hg.1	ECE1
TC01002322.hg.1	2.7	PSR01035695.hg.1	ECE1
TC01002322.hg.1	2.6	PSR01035703.hg.1	ECE1
TC01002322.hg.1	2.55	PSR01035663.hg.1	ECE1
TC01002322.hg.1	2.38	PSR01035698.hg.1	ECE1
TC01002322.hg.1	2.35	PSR01035664.hg.1	ECE1
TC01002322.hg.1	2.33	PSR01035701.hg.1	ECE1
TC01002322.hg.1	2.28	JUC01019196.hg.1	ECE1
TC01002322.hg.1	2.23	PSR01035660.hg.1	ECE1
TC01002322.hg.1	2.18	PSR01035661.hg.1	ECE1
TC01002322.hg.1	2.06	PSR01035700.hg.1	ECE1
TC02005009.hg.1	4.21	PSR02021445.hg.1	SP140
TC02005009.hg.1	2.21	PSR02021498.hg.1	SP140
TC02005009.hg.1	2.13	JUC02032051.hg.1	SP140
TC02005009.hg.1	2.05	JUC02032063.hg.1	SP140

TC02005009.hg.1	-2.23	JUC02032086.hg.1	SP140
TC03003372.hg.1	4.21	JUC03024006.hg.1	SRGAP3
TC03003372.hg.1	3.13	JUC03023994.hg.1	SRGAP3
TC03003372.hg.1	2.74	JUC03023995.hg.1	SRGAP3
TC03003372.hg.1	2.68	PSR03019707.hg.1	SRGAP3
TC03003372.hg.1	2.61	PSR03019684.hg.1	SRGAP3
TC03003372.hg.1	2.55	JUC03023993.hg.1	SRGAP3
TC03003372.hg.1	2.33	JUC03024008.hg.1	SRGAP3
TC03003372.hg.1	2.13	JUC03024001.hg.1	SRGAP3
TC03003372.hg.1	2.02	JUC03024023.hg.1	SRGAP3
TC03003372.hg.1	2	JUC03024020.hg.1	SRGAP3
TC06001771.hg.1	4.21	PSR06021319.hg.1	RCAN2
TC06001771.hg.1	4.09	PSR06021322.hg.1	RCAN2
TC06001771.hg.1	3.27	PSR06021316.hg.1	RCAN2
TC06001771.hg.1	3.05	PSR06021314.hg.1	RCAN2
TC06001771.hg.1	2.43	PSR06021321.hg.1	RCAN2
TC06001771.hg.1	2.02	PSR06021309.hg.1	RCAN2
TC06001771.hg.1	-3.29	JUC06010155.hg.1	RCAN2
TC20000538.hg.1	4.21	JUC20004053.hg.1	C20orf96
TC20000538.hg.1	-2.23	PSR20007766.hg.1	C20orf96
TC20000538.hg.1	-2.35	JUC20004046.hg.1	C20orf96
TC20000538.hg.1	-4.45	JUC20004049.hg.1	C20orf96
TC02001267.hg.1	4.2	JUC02010472.hg.1	
TC02001267.hg.1	2.14	PSR02019422.hg.1	
TC02001267.hg.1	-2.14	PSR02019421.hg.1	
TC04000429.hg.1	4.2	PSR04006026.hg.1	
TC04000429.hg.1	3.97	JUC04003034.hg.1	
TC04000429.hg.1	3.2	PSR04006029.hg.1	
TC04000429.hg.1	-2.65	PSR04006028.hg.1	
TC08000022.hg.1	4.2	PSR08000343.hg.1	AGPAT5
TC08000022.hg.1	3.08	PSR08000359.hg.1	AGPAT5
TC08000022.hg.1	2.41	PSR08000342.hg.1	AGPAT5
TC08000022.hg.1	2.24	JUC08000201.hg.1	AGPAT5
TC08000022.hg.1	2.18	JUC08000198.hg.1	AGPAT5
TC0X001354.hg.1	4.2	PSR0X018132.hg.1	APLN
TC0X001354.hg.1	-3.21	JUC0X009244.hg.1	APLN
TC0X001354.hg.1	-5.64	PSR0X018129.hg.1	APLN
TC0X001354.hg.1	-7.22	PSR0X018127.hg.1	APLN
TC14001474.hg.1	4.2	PSR14016658.hg.1	CLMN
TC14001474.hg.1	3.92	JUC14008596.hg.1	CLMN
TC14001474.hg.1	3.59	PSR14016644.hg.1	CLMN
TC14001474.hg.1	2.99	JUC14008611.hg.1	CLMN
TC14001474.hg.1	2.93	PSR14016645.hg.1	CLMN
TC14001474.hg.1	2.57	PSR14016632.hg.1	CLMN
TC14001474.hg.1	2.26	PSR14016633.hg.1	CLMN
TC14001474.hg.1	2.26	PSR14016642.hg.1	CLMN
TC14001474.hg.1	2.12	PSR14016652.hg.1	CLMN
TC14001474.hg.1	2.07	JUC14008599.hg.1	CLMN
TC14001474.hg.1	-2.08	PSR14016648.hg.1	CLMN
TC14001474.hg.1	-2.45	PSR14016643.hg.1	CLMN
TC02001139.hg.1	4.19	PSR02017197.hg.1	SLC39A10
TC02001139.hg.1	3.59	JUC02009214.hg.1	SLC39A10
TC02001139.hg.1	3.07	JUC02009217.hg.1	SLC39A10
TC02001139.hg.1	2.86	PSR02017204.hg.1	SLC39A10
TC02001139.hg.1	2.68	JUC02009213.hg.1	SLC39A10
TC02001139.hg.1	2.55	JUC02009224.hg.1	SLC39A10
TC02001139.hg.1	2.42	PSR02017205.hg.1	SLC39A10
TC02001139.hg.1	2.17	PSR02017220.hg.1	SLC39A10

TC07001152.hg.1	4.19	JUC07008786.hg.1	ETV1
TC07001152.hg.1	4.11	JUC07008770.hg.1	ETV1
TC07001152.hg.1	2.65	JUC07008783.hg.1	ETV1
TC07001152.hg.1	2.47	JUC07008762.hg.1	ETV1
TC07001152.hg.1	2.45	PSR07018029.hg.1	ETV1
TC07001152.hg.1	2.38	PSR07018046.hg.1	ETV1
TC07001152.hg.1	2.27	JUC07008790.hg.1	ETV1
TC07001152.hg.1	2.26	PSR07018052.hg.1	ETV1
TC07001152.hg.1	2.14	PSR07018053.hg.1	ETV1
TC07001152.hg.1	2.06	PSR07018007.hg.1	ETV1
TC07001152.hg.1	-2.38	PSR07018034.hg.1	ETV1
TC07001152.hg.1	-2.5	PSR07018037.hg.1	ETV1
TC07001152.hg.1	-2.74	PSR07018017.hg.1	ETV1
TC06004073.hg.1	4.18	JUC06020958.hg.1	MICA
TC06004073.hg.1	3.88	PSR06003469.hg.1	MICA
TC06004073.hg.1	3.5	PSR06003455.hg.1	MICA
TC06004073.hg.1	3.16	JUC06020963.hg.1	MICA
TC06004073.hg.1	2.99	JUC06020965.hg.1	MICA
TC06004073.hg.1	2.88	PSR06003443.hg.1	MICA
TC06004073.hg.1	2.69	JUC06020961.hg.1	MICA
TC06004073.hg.1	2.68	JUC06020964.hg.1	MICA
TC06004073.hg.1	2.63	PSR06003454.hg.1	MICA
TC06004073.hg.1	2.31	JUC06020955.hg.1	MICA
TC06004073.hg.1	2.3	JUC06020960.hg.1	MICA
TC06004073.hg.1	2.29	JUC06020952.hg.1	MICA
TC06004073.hg.1	2.09	PSR06003451.hg.1	MICA
TC06004073.hg.1	2.02	JUC06020957.hg.1	MICA
TC10001053.hg.1	4.18	JUC10007578.hg.1	FRMD4A
TC10001053.hg.1	3.93	PSR10013129.hg.1	FRMD4A
TC10001053.hg.1	3.4	JUC10007585.hg.1	FRMD4A
TC10001053.hg.1	3.17	PSR10013083.hg.1	FRMD4A
TC10001053.hg.1	2.85	JUC10007586.hg.1	FRMD4A
TC10001053.hg.1	2.71	JUC10007577.hg.1	FRMD4A
TC10001053.hg.1	2.67	PSR10013111.hg.1	FRMD4A
TC10001053.hg.1	2.61	PSR10013137.hg.1	FRMD4A
TC10001053.hg.1	2.51	JUC10007576.hg.1	FRMD4A
TC10001053.hg.1	2.46	PSR10013131.hg.1	FRMD4A
TC10001053.hg.1	2.36	PSR10013090.hg.1	FRMD4A
TC10001053.hg.1	2.33	PSR10013118.hg.1	FRMD4A
TC10001053.hg.1	2.31	PSR10013110.hg.1	FRMD4A
TC10001053.hg.1	2.23	JUC10007589.hg.1	FRMD4A
TC10001053.hg.1	2.14	PSR10013128.hg.1	FRMD4A
TC10001053.hg.1	2.12	PSR10013109.hg.1	FRMD4A
TC10001053.hg.1	2.06	JUC10007580.hg.1	FRMD4A
TC10001053.hg.1	2.06	JUC10007605.hg.1	FRMD4A
TC10001053.hg.1	2.03	PSR10013125.hg.1	FRMD4A
TC10001053.hg.1	2.02	PSR10013104.hg.1	FRMD4A
TC10001053.hg.1	-2.62	JUC10007592.hg.1	FRMD4A
TC10001053.hg.1	-4.8	JUC10007588.hg.1	FRMD4A
TC6_cox_hap2000	4.18	JUC6_cox_hap2001	MICA
TC6_cox_hap2000	3.88	PSR6_cox_hap2000	MICA
TC6_cox_hap2000	3.5	PSR6_cox_hap2000	MICA
TC6_cox_hap2000	3.16	JUC6_cox_hap2001	MICA
TC6_cox_hap2000	3.03	PSR6_cox_hap2000	MICA
TC6_cox_hap2000	2.99	JUC6_cox_hap2001	MICA
TC6_cox_hap2000	2.69	JUC6_cox_hap2001	MICA
TC6_cox_hap2000	2.68	JUC6_cox_hap2001	MICA
TC6_cox_hap2000	2.66	PSR6_cox_hap2000	MICA

TC6_cox_hap2000	2.31	JUC6_cox_hap2001	MICA
TC6_cox_hap2000	2.3	JUC6_cox_hap2001	MICA
TC6_cox_hap2000	2.29	JUC6_cox_hap2001	MICA
TC6_cox_hap2000	2.09	PSR6_cox_hap2000	MICA
TC6_cox_hap2000	2.02	JUC6_cox_hap2001	MICA
TC6_qbl_hap6000	4.18	PSR6_qbl_hap60001	HLA-H
TC6_qbl_hap6000	2.51	JUC6_qbl_hap60018	HLA-H
TC6_qbl_hap6000	2.23	JUC6_qbl_hap60018	HLA-H
TC6_qbl_hap6000	2.03	PSR6_qbl_hap60001	HLA-H
TC6_ssto_hap700	4.18	JUC6_ssto_hap7001	MICA
TC6_ssto_hap700	3.88	PSR6_ssto_hap7000	MICA
TC6_ssto_hap700	3.5	PSR6_ssto_hap7000	MICA
TC6_ssto_hap700	3.16	JUC6_ssto_hap7001	MICA
TC6_ssto_hap700	3.03	PSR6_ssto_hap7000	MICA
TC6_ssto_hap700	2.99	JUC6_ssto_hap7001	MICA
TC6_ssto_hap700	2.69	JUC6_ssto_hap7001	MICA
TC6_ssto_hap700	2.68	JUC6_ssto_hap7001	MICA
TC6_ssto_hap700	2.66	PSR6_ssto_hap7000	MICA
TC6_ssto_hap700	2.31	JUC6_ssto_hap7001	MICA
TC6_ssto_hap700	2.3	JUC6_ssto_hap7001	MICA
TC6_ssto_hap700	2.29	JUC6_ssto_hap7001	MICA
TC6_ssto_hap700	2.09	PSR6_ssto_hap7000	MICA
TC6_ssto_hap700	2.02	JUC6_ssto_hap7001	MICA
TC05003425.hg.1	4.17	JUC05019586.hg.1	DAB2
TC05003425.hg.1	3.39	JUC05019595.hg.1	DAB2
TC05003425.hg.1	2.63	JUC05019604.hg.1	DAB2
TC05003425.hg.1	2.34	JUC05019610.hg.1	DAB2
TC05003425.hg.1	2.14	JUC05019612.hg.1	DAB2
TC05003425.hg.1	-2.64	PSR05018231.hg.1	DAB2
TC08001464.hg.1	4.17	JUC08009663.hg.1	STK3
TC08001464.hg.1	3.44	JUC08009664.hg.1	STK3
TC08001464.hg.1	2.26	JUC08009649.hg.1	STK3
TC08001464.hg.1	2.14	PSR08018987.hg.1	STK3
TC08001464.hg.1	-2.54	JUC08009648.hg.1	STK3
TC09000498.hg.1	4.17	JUC09002987.hg.1	GALNT12
TC09000498.hg.1	2.21	PSR09005401.hg.1	GALNT12
TC09000498.hg.1	2.06	JUC09002991.hg.1	GALNT12
TC09000498.hg.1	-2.2	JUC09002985.hg.1	GALNT12
TC14000319.hg.1	4.17	JUC14001642.hg.1	SAMD4A
TC14000319.hg.1	2.57	PSR14003381.hg.1	SAMD4A
TC14000319.hg.1	2.1	PSR14003389.hg.1	SAMD4A
TC14000319.hg.1	2.01	JUC14001633.hg.1	SAMD4A
TC14000319.hg.1	-2.06	JUC14001639.hg.1	SAMD4A
TC14000319.hg.1	-2.09	PSR14003390.hg.1	SAMD4A
TC14000319.hg.1	-3.46	JUC14001637.hg.1	SAMD4A
TC02002606.hg.1	4.16	JUC02021745.hg.1	TFPI
TC02002606.hg.1	3.36	JUC02021728.hg.1	TFPI
TC02002606.hg.1	3.23	JUC02021730.hg.1	TFPI
TC02002606.hg.1	2.41	JUC02021731.hg.1	TFPI
TC02002606.hg.1	2.38	PSR02041713.hg.1	TFPI
TC02002606.hg.1	2.35	PSR02041727.hg.1	TFPI
TC02002606.hg.1	2.07	JUC02021736.hg.1	TFPI
TC02002606.hg.1	2.05	PSR02041739.hg.1	TFPI
TC05001481.hg.1	4.16	PSR05020318.hg.1	ENC1
TC05001481.hg.1	2.47	PSR05020319.hg.1	ENC1
TC05001481.hg.1	-2.79	JUC05010456.hg.1	ENC1
TC06001074.hg.1	4.16	JUC06006268.hg.1	SASH1
TC06001074.hg.1	3.45	PSR06012705.hg.1	SASH1

TC06001074.hg.1	3.45	PSR06012708.hg.1	SASH1
TC06001074.hg.1	3.15	PSR06012697.hg.1	SASH1
TC06001074.hg.1	2.56	PSR06012713.hg.1	SASH1
TC06001074.hg.1	2.44	PSR06012698.hg.1	SASH1
TC0X000034.hg.1	4.16	PSR0X000449.hg.1	SHROOM2
TC0X000034.hg.1	4.09	JUC0X000261.hg.1	SHROOM2
TC0X000034.hg.1	4.03	PSR0X000447.hg.1	SHROOM2
TC0X000034.hg.1	3.6	PSR0X000453.hg.1	SHROOM2
TC0X000034.hg.1	3.51	PSR0X000457.hg.1	SHROOM2
TC0X000034.hg.1	2.69	JUC0X000258.hg.1	SHROOM2
TC0X000034.hg.1	2.63	PSR0X000454.hg.1	SHROOM2
TC0X000034.hg.1	2.53	JUC0X000254.hg.1	SHROOM2
TC0X000034.hg.1	2.22	JUC0X000260.hg.1	SHROOM2
TC0X000034.hg.1	2.09	PSR0X000448.hg.1	SHROOM2
TC0X000034.hg.1	-2.22	JUC0X000257.hg.1	SHROOM2
TC0X000034.hg.1	-2.45	JUC0X000253.hg.1	SHROOM2
TC0X000034.hg.1	-2.79	PSR0X000464.hg.1	SHROOM2
TC0X000034.hg.1	-3.79	PSR0X000451.hg.1	SHROOM2
TC11002435.hg.1	4.16	JUC11015539.hg.1	KIRREL3
TC11002435.hg.1	3.96	JUC11015550.hg.1	KIRREL3
TC11002435.hg.1	3.14	JUC11015542.hg.1	KIRREL3
TC11002435.hg.1	3.02	PSR11029172.hg.1	KIRREL3
TC11002435.hg.1	2.84	PSR11029160.hg.1	KIRREL3
TC11002435.hg.1	2.71	PSR11029170.hg.1	KIRREL3
TC11002435.hg.1	2.7	JUC11015544.hg.1	KIRREL3
TC11002435.hg.1	2.61	PSR11029187.hg.1	KIRREL3
TC11002435.hg.1	2.3	PSR11029182.hg.1	KIRREL3
TC11002435.hg.1	2.05	JUC11015545.hg.1	KIRREL3
TC11002435.hg.1	2	PSR11029188.hg.1	KIRREL3
TC11002435.hg.1	-2.13	PSR11029167.hg.1	KIRREL3
TC11002435.hg.1	-2.33	JUC11015540.hg.1	KIRREL3
TC11002435.hg.1	-2.43	PSR11029183.hg.1	KIRREL3
TC11002435.hg.1	-3.19	JUC11015543.hg.1	KIRREL3
TC11002435.hg.1	-4.96	JUC11015551.hg.1	KIRREL3
TC11002435.hg.1	-5.54	PSR11029166.hg.1	KIRREL3
TC11002435.hg.1	-7.91	JUC11015546.hg.1	KIRREL3
TC11002435.hg.1	-8.54	JUC11015536.hg.1	KIRREL3
TC15001211.hg.1	4.16	JUC15005753.hg.1	MEIS2
TC15001211.hg.1	4.01	PSR15010899.hg.1	MEIS2
TC15001211.hg.1	3.82	PSR15010874.hg.1	MEIS2
TC15001211.hg.1	3.6	PSR15010886.hg.1	MEIS2
TC15001211.hg.1	3.57	PSR15010897.hg.1	MEIS2
TC15001211.hg.1	3.5	PSR15010884.hg.1	MEIS2
TC15001211.hg.1	3.33	PSR15010857.hg.1	MEIS2
TC15001211.hg.1	2.99	PSR15010893.hg.1	MEIS2
TC15001211.hg.1	2.72	JUC15005751.hg.1	MEIS2
TC15001211.hg.1	2.67	PSR15010853.hg.1	MEIS2
TC15001211.hg.1	2.63	PSR15010894.hg.1	MEIS2
TC15001211.hg.1	2.59	PSR15010892.hg.1	MEIS2
TC15001211.hg.1	2.44	PSR15010860.hg.1	MEIS2
TC15001211.hg.1	2.36	JUC15005750.hg.1	MEIS2
TC15001211.hg.1	2.22	PSR15010873.hg.1	MEIS2
TC15001211.hg.1	2.19	PSR15010887.hg.1	MEIS2
TC15001211.hg.1	2.18	PSR15010881.hg.1	MEIS2
TC17001462.hg.1	4.16	JUC17010973.hg.1	TOP2A
TC17001462.hg.1	3.55	JUC17010988.hg.1	TOP2A
TC17001462.hg.1	3.54	JUC17011011.hg.1	TOP2A
TC17001462.hg.1	3.14	PSR17019384.hg.1	TOP2A

TC17001462.hg.1	3.13	PSR17019405.hg.1	TOP2A
TC17001462.hg.1	3.01	JUC17010971.hg.1	TOP2A
TC17001462.hg.1	2.62	PSR17019387.hg.1	TOP2A
TC17001462.hg.1	2.59	JUC17011013.hg.1	TOP2A
TC17001462.hg.1	2.52	PSR17019404.hg.1	TOP2A
TC17001462.hg.1	2.51	PSR17019386.hg.1	TOP2A
TC17001462.hg.1	2.47	PSR17019374.hg.1	TOP2A
TC17001462.hg.1	2.28	JUC17010974.hg.1	TOP2A
TC17001462.hg.1	2.22	PSR17019378.hg.1	TOP2A
TC17001462.hg.1	2.22	PSR17019382.hg.1	TOP2A
TC17001462.hg.1	2.16	PSR17019348.hg.1	TOP2A
TC17001462.hg.1	2.13	JUC17010979.hg.1	TOP2A
TC17001462.hg.1	-2.05	JUC17010985.hg.1	TOP2A
TC17001462.hg.1	-2.15	PSR17019364.hg.1	TOP2A
TC17001462.hg.1	-3.81	JUC17011008.hg.1	TOP2A
TC02000423.hg.1	4.15	JUC02003228.hg.1	ATP6V1B1
TC02000423.hg.1	3.88	JUC02003236.hg.1	ATP6V1B1
TC02000423.hg.1	2.85	JUC02003224.hg.1	ATP6V1B1
TC02000423.hg.1	2.15	PSR02006310.hg.1	ATP6V1B1
TC02000423.hg.1	-6.26	JUC02003237.hg.1	ATP6V1B1
TC11000267.hg.1	4.15	JUC11001836.hg.1	
TC11000267.hg.1	2.15	PSR11003577.hg.1	
TC02000874.hg.1	4.14	JUC02006847.hg.1	MGAT5
TC02000874.hg.1	2.91	PSR02012904.hg.1	MGAT5
TC02000874.hg.1	2.51	PSR02012928.hg.1	MGAT5
TC02000874.hg.1	-2.13	JUC02006843.hg.1	MGAT5
TC02000874.hg.1	-2.61	JUC02006846.hg.1	MGAT5
TC16001281.hg.1	4.14	PSR16017829.hg.1	CHST6
TC16001281.hg.1	3.07	JUC16010157.hg.1	CHST6
TC16001281.hg.1	2.82	PSR16017832.hg.1	CHST6
TC16001281.hg.1	-2.08	PSR16017828.hg.1	CHST6
TC16001281.hg.1	-2.36	PSR16017823.hg.1	CHST6
TC19001802.hg.1	4.14	JUC19013900.hg.1	ZNF83
TC19001802.hg.1	3.47	JUC19013911.hg.1	ZNF83
TC19001802.hg.1	2.24	JUC19013913.hg.1	ZNF83
TC19001802.hg.1	2.18	JUC19013904.hg.1	ZNF83
TC19001802.hg.1	2.13	JUC19013906.hg.1	ZNF83
TC19001802.hg.1	2.06	PSR19024462.hg.1	ZNF83
TC20000788.hg.1	4.14	JUC20005528.hg.1	GGT7
TC20000788.hg.1	2.67	PSR20010518.hg.1	GGT7
TC20000788.hg.1	2.18	JUC20005516.hg.1	GGT7
TC20000788.hg.1	2.06	JUC20005531.hg.1	GGT7
TC01003096.hg.1	4.13	JUC01025749.hg.1	PDE4DIP, LO
TC01003096.hg.1	3.65	PSR01047962.hg.1	PDE4DIP, LO
TC01003096.hg.1	3.56	PSR01047965.hg.1	PDE4DIP, LO
TC01003096.hg.1	3.31	PSR01047967.hg.1	PDE4DIP, LO
TC01003096.hg.1	3.27	PSR01047963.hg.1	PDE4DIP, LO
TC01003096.hg.1	2.77	PSR01047968.hg.1	PDE4DIP, LO
TC01003096.hg.1	2.73	PSR01047961.hg.1	PDE4DIP, LO
TC01003096.hg.1	2.66	PSR01047969.hg.1	PDE4DIP, LO
TC01003096.hg.1	2.52	PSR01047966.hg.1	PDE4DIP, LO
TC01003096.hg.1	2.37	JUC01025801.hg.1	PDE4DIP, LO
TC14001347.hg.1	4.13	PSR14015326.hg.1	ISM2
TC14001347.hg.1	-3.28	JUC14007785.hg.1	ISM2
TC08001075.hg.1	4.12	JUC08007033.hg.1	PNMA2
TC08001075.hg.1	2.43	JUC08007041.hg.1	PNMA2
TC08001075.hg.1	2.03	PSR08013692.hg.1	PNMA2
TC08001075.hg.1	2.02	PSR08013690.hg.1	PNMA2

TC08001075.hg.1	2.02	PSR08013697.hg.1	PNMA2
TC08001075.hg.1	-2.04	PSR08013672.hg.1	PNMA2
TC08001075.hg.1	-2.28	PSR08013666.hg.1	PNMA2
TC08001075.hg.1	-2.39	PSR08013663.hg.1	PNMA2
TC08001075.hg.1	-2.41	PSR08013664.hg.1	PNMA2
TC08001075.hg.1	-2.58	PSR08013667.hg.1	PNMA2
TC08001075.hg.1	-3.05	PSR08013661.hg.1	PNMA2
TC12001303.hg.1	4.12	JUC12009420.hg.1	KIAA0528
TC12001303.hg.1	2.21	JUC12009422.hg.1	KIAA0528
TC12001303.hg.1	-2.27	JUC12009402.hg.1	KIAA0528
TC12001303.hg.1	-2.3	PSR12017069.hg.1	KIAA0528
TC12001303.hg.1	-3.46	JUC12009411.hg.1	KIAA0528
TC19000377.hg.1	4.12	JUC19003011.hg.1	CILP2
TC19000377.hg.1	3.18	JUC19003010.hg.1	CILP2
TC19000377.hg.1	2.91	PSR19005105.hg.1	CILP2
TC19000377.hg.1	-2.02	PSR19005098.hg.1	CILP2
TC19000377.hg.1	-2.16	JUC19003008.hg.1	CILP2
TC19000377.hg.1	-2.27	PSR19005097.hg.1	CILP2
TC19000377.hg.1	-2.47	PSR19005101.hg.1	CILP2
TC19000377.hg.1	-3.47	JUC19003007.hg.1	CILP2
TC19000377.hg.1	-6.95	JUC19003012.hg.1	CILP2
TC14002298.hg.1	4.11	JUC14011690.hg.1	SLC38A6
TC14002298.hg.1	2.49	JUC14011709.hg.1	SLC38A6
TC14002298.hg.1	2.4	PSR14004049.hg.1	SLC38A6
TC14002298.hg.1	2.19	PSR14004074.hg.1	SLC38A6
TC14002298.hg.1	2.11	JUC14011712.hg.1	SLC38A6
TC14002298.hg.1	2.02	JUC14011704.hg.1	SLC38A6
TC14002298.hg.1	-2.12	JUC14011714.hg.1	SLC38A6
TC14002298.hg.1	-2.74	JUC14011692.hg.1	SLC38A6
TC05001943.hg.1	4.1	JUC05013706.hg.1	DCTN4
TC05001943.hg.1	3.72	JUC05013705.hg.1	DCTN4
TC05001943.hg.1	2.55	JUC05013715.hg.1	DCTN4
TC05001943.hg.1	2.13	PSR05026956.hg.1	DCTN4
TC05001943.hg.1	-2.58	PSR05026949.hg.1	DCTN4
TC06000718.hg.1	4.1	JUC06003873.hg.1	SMAP1
TC06000718.hg.1	2.95	PSR06008567.hg.1	SMAP1
TC01006321.hg.1	4.09	JUC01043959.hg.1	CCDC30
TC01006321.hg.1	2.89	PSR01008283.hg.1	CCDC30
TC01006321.hg.1	2.66	PSR01008317.hg.1	CCDC30
TC01006321.hg.1	2.14	PSR01008270.hg.1	CCDC30
TC01006321.hg.1	2.03	PSR01008295.hg.1	CCDC30
TC01006321.hg.1	2	PSR01008258.hg.1	CCDC30
TC01006321.hg.1	2	JUC01043961.hg.1	CCDC30
TC03000173.hg.1	4.09	JUC03001699.hg.1	STAC
TC03000173.hg.1	-2.34	JUC03001697.hg.1	STAC
TC03000173.hg.1	-3.19	PSR03003243.hg.1	STAC
TC0X001425.hg.1	4.09	JUC0X009642.hg.1	FGF13
TC0X001425.hg.1	3.63	JUC0X009644.hg.1	FGF13
TC0X001425.hg.1	2.4	PSR0X018892.hg.1	FGF13
TC0X001425.hg.1	2.38	JUC0X009653.hg.1	FGF13
TC0X001425.hg.1	2.3	JUC0X009646.hg.1	FGF13
TC0X001425.hg.1	2.2	PSR0X018888.hg.1	FGF13
TC0X001425.hg.1	2.2	PSR0X018906.hg.1	FGF13
TC0X001425.hg.1	2.13	PSR0X018915.hg.1	FGF13
TC0X001425.hg.1	2.05	PSR0X018903.hg.1	FGF13
TC0X001425.hg.1	2.04	JUC0X009638.hg.1	FGF13
TC0X001425.hg.1	2.03	PSR0X018911.hg.1	FGF13
TC0X001425.hg.1	2.03	JUC0X009651.hg.1	FGF13

TC0X001425.hg.1	-2.37	JUC0X009654.hg.1	FGF13
TC11003504.hg.1	4.09	JUC11019767.hg.1	LOC1005062
TC11003504.hg.1	3.42	PSR11017247.hg.1	LOC1005062
TC11003504.hg.1	2.99	JUC11019777.hg.1	LOC1005062
TC11003504.hg.1	2.45	PSR11017227.hg.1	LOC1005062
TC11003504.hg.1	2.24	PSR11017234.hg.1	LOC1005062
TC11003504.hg.1	2.07	JUC11019772.hg.1	LOC1005062
TC12000621.hg.1	4.09	JUC12004347.hg.1	KCNMB4
TC12000621.hg.1	-2.33	PSR12008256.hg.1	KCNMB4
TC12000621.hg.1	-2.69	PSR12008257.hg.1	KCNMB4
TC15002013.hg.1	4.09	JUC15010201.hg.1	TARSL2
TC15002013.hg.1	3.82	JUC15010192.hg.1	TARSL2
TC15002013.hg.1	2.4	PSR15018695.hg.1	TARSL2
TC15002013.hg.1	2.06	PSR15018706.hg.1	TARSL2
TC07000262.hg.1	4.08	PSR07004027.hg.1	HECW1-IT1
TC07000262.hg.1	-2.35	PSR07004030.hg.1	HECW1-IT1
TC07000262.hg.1	-2.66	PSR07004029.hg.1	HECW1-IT1
TC07000262.hg.1	-3.37	JUC07001951.hg.1	HECW1-IT1
TC17001457.hg.1	4.08	PSR17019277.hg.1	MED24
TC17001457.hg.1	2.69	JUC17010938.hg.1	MED24
TC17001457.hg.1	2.36	JUC17010951.hg.1	MED24
TC17001457.hg.1	2.14	JUC17010943.hg.1	MED24
TC17001457.hg.1	-2.38	JUC17010913.hg.1	MED24
TC17001457.hg.1	-2.38	JUC17010940.hg.1	MED24
TC17001457.hg.1	-2.39	PSR17019247.hg.1	MED24
TC17001457.hg.1	-2.78	JUC17010936.hg.1	MED24
TC02000507.hg.1	4.07	JUC02003985.hg.1	ELMOD3
TC02000507.hg.1	-2.22	JUC02003968.hg.1	ELMOD3
TC02000507.hg.1	-2.27	PSR02007781.hg.1	ELMOD3
TC02000507.hg.1	-2.31	JUC02003960.hg.1	ELMOD3
TC03000248.hg.1	4.07	JUC03002576.hg.1	LIMD1
TC03000248.hg.1	3.92	PSR03005047.hg.1	LIMD1
TC03000248.hg.1	3.61	PSR03005062.hg.1	LIMD1
TC03000248.hg.1	3.1	PSR03005049.hg.1	LIMD1
TC03000248.hg.1	3.07	JUC03002573.hg.1	LIMD1
TC03000248.hg.1	2.88	JUC03002575.hg.1	LIMD1
TC03000248.hg.1	2.72	PSR03005052.hg.1	LIMD1
TC03000248.hg.1	2.65	JUC03002567.hg.1	LIMD1
TC03000248.hg.1	2.49	PSR03005051.hg.1	LIMD1
TC03000248.hg.1	2.32	JUC03002570.hg.1	LIMD1
TC03000248.hg.1	2.24	PSR03005058.hg.1	LIMD1
TC10001322.hg.1	4.07	PSR10016707.hg.1	TMEM26
TC10001322.hg.1	3.28	JUC10009752.hg.1	TMEM26
TC10001322.hg.1	3.2	JUC10009754.hg.1	TMEM26
TC10001322.hg.1	2.85	PSR10016712.hg.1	TMEM26
TC10001322.hg.1	2.66	JUC10009755.hg.1	TMEM26
TC10001322.hg.1	2.18	PSR10016711.hg.1	TMEM26
TC10001322.hg.1	-2.38	JUC10009750.hg.1	TMEM26
TC10001322.hg.1	-3.35	PSR10016705.hg.1	TMEM26
TC11002439.hg.1	4.07	JUC11015566.hg.1	ETS1
TC11002439.hg.1	3.37	JUC11015562.hg.1	ETS1
TC11002439.hg.1	3.08	JUC11015568.hg.1	ETS1
TC11002439.hg.1	3.06	PSR11029216.hg.1	ETS1
TC11002439.hg.1	2.51	PSR11029212.hg.1	ETS1
TC11002439.hg.1	2.37	PSR11029211.hg.1	ETS1
TC11002439.hg.1	2.15	JUC11015561.hg.1	ETS1
TC15001711.hg.1	4.07	JUC15009111.hg.1	ADAMTS7, L(
TC15001711.hg.1	2.47	PSR15016723.hg.1	ADAMTS7, L(

TC15001711.hg.1	2.32	JUC15009116.hg.1	ADAMTS7, L(
TC15001711.hg.1	2.25	PSR15016716.hg.1	ADAMTS7, L(
TC15001711.hg.1	2.24	PSR15016728.hg.1	ADAMTS7, L(
TC15001711.hg.1	2.14	PSR15016725.hg.1	ADAMTS7, L(
TC15001711.hg.1	-2.11	JUC15009110.hg.1	ADAMTS7, L(
TC17002883.hg.1	4.07	PSR17012340.hg.1	SLC25A10
TC17002883.hg.1	2.32	PSR17012343.hg.1	SLC25A10
TC17002883.hg.1	-2.3	PSR17012337.hg.1	SLC25A10
TC17002883.hg.1	-2.33	JUC17018790.hg.1	SLC25A10
TC21000459.hg.1	4.07	PSR21005370.hg.1	BRWD1
TC21000459.hg.1	3.22	PSR21005362.hg.1	BRWD1
TC21000459.hg.1	2.98	JUC21002773.hg.1	BRWD1
TC21000459.hg.1	2.15	JUC21002768.hg.1	BRWD1
TC21000459.hg.1	2	JUC21002797.hg.1	BRWD1
TC21000459.hg.1	-2.21	JUC21002753.hg.1	BRWD1
TC01003005.hg.1	4.06	PSR01046740.hg.1	RSBN1
TC01003005.hg.1	2.36	JUC01025083.hg.1	RSBN1
TC03003352.hg.1	4.06	JUC03023683.hg.1	RBM5
TC03003352.hg.1	3.45	PSR03006335.hg.1	RBM5
TC03003352.hg.1	3	JUC03023676.hg.1	RBM5
TC03003352.hg.1	2.49	PSR03006346.hg.1	RBM5
TC03003352.hg.1	2.27	PSR03006332.hg.1	RBM5
TC03003352.hg.1	-2.2	PSR03006307.hg.1	RBM5
TC03003352.hg.1	-2.25	JUC03023685.hg.1	RBM5
TC11001273.hg.1	4.06	PSR11016094.hg.1	MIR675, H19
TC11001273.hg.1	3.73	PSR11016075.hg.1	MIR675, H19
TC11001273.hg.1	3.54	JUC11008656.hg.1	MIR675, H19
TC11001273.hg.1	2.99	PSR11016071.hg.1	MIR675, H19
TC11001273.hg.1	2.6	JUC11008657.hg.1	MIR675, H19
TC11001273.hg.1	2.48	PSR11016089.hg.1	MIR675, H19
TC11001273.hg.1	2.35	JUC11008655.hg.1	MIR675, H19
TC11001273.hg.1	2.11	PSR11016070.hg.1	MIR675, H19
TC11001273.hg.1	2.05	PSR11016088.hg.1	MIR675, H19
TC11001273.hg.1	-2.18	JUC11008660.hg.1	MIR675, H19
TC12000284.hg.1	4.06	JUC12001714.hg.1	TSPAN11
TC12000284.hg.1	3.4	JUC12001722.hg.1	TSPAN11
TC12000284.hg.1	2.64	JUC12001715.hg.1	TSPAN11
TC12000284.hg.1	2.15	JUC12001719.hg.1	TSPAN11
TC12000284.hg.1	2.05	PSR12003403.hg.1	TSPAN11
TC12000284.hg.1	-2.64	JUC12001713.hg.1	TSPAN11
TC20000727.hg.1	4.06	PSR20009824.hg.1	ACSS1
TC20000727.hg.1	-2.15	PSR20009839.hg.1	ACSS1
TC20000727.hg.1	-2.15	JUC20005122.hg.1	ACSS1
TC20000727.hg.1	-2.21	PSR20009840.hg.1	ACSS1
TC20000727.hg.1	-2.34	PSR20009848.hg.1	ACSS1
TC20000727.hg.1	-2.57	JUC20005127.hg.1	ACSS1
TC20000727.hg.1	-2.59	JUC20005141.hg.1	ACSS1
TC20000727.hg.1	-2.75	JUC20005135.hg.1	ACSS1
TC20000727.hg.1	-3.06	JUC20005125.hg.1	ACSS1
TC20000727.hg.1	-3.73	JUC20005124.hg.1	ACSS1
TC05000822.hg.1	4.05	JUC05006125.hg.1	ARHGEF37
TC05000822.hg.1	3.99	PSR05011756.hg.1	ARHGEF37
TC05000822.hg.1	3.77	PSR05011757.hg.1	ARHGEF37
TC05000822.hg.1	3.18	PSR05011770.hg.1	ARHGEF37
TC05000822.hg.1	3.1	JUC05006127.hg.1	ARHGEF37
TC05000822.hg.1	2.6	JUC05006130.hg.1	ARHGEF37
TC05000822.hg.1	2.56	JUC05006129.hg.1	ARHGEF37
TC05000822.hg.1	-2.49	PSR05011761.hg.1	ARHGEF37

TC05000822.hg.1	-3.08	JUC05006133.hg.1	ARHGEF37
TC05000822.hg.1	-3.46	JUC05006123.hg.1	ARHGEF37
TC05000822.hg.1	-3.5	PSR05011760.hg.1	ARHGEF37
TC05000822.hg.1	-4.45	PSR05011762.hg.1	ARHGEF37
TC05000822.hg.1	-15.12	JUC05006128.hg.1	ARHGEF37
TC05001327.hg.1	4.05	JUC05009633.hg.1	LOC1005066
TC05001327.hg.1	3.56	JUC05009632.hg.1	LOC1005066
TC05001327.hg.1	2.61	JUC05009639.hg.1	LOC1005066
TC05001327.hg.1	2.44	PSR05018740.hg.1	LOC1005066
TC05001327.hg.1	2.34	PSR05018736.hg.1	LOC1005066
TC05001327.hg.1	2.3	PSR05018735.hg.1	LOC1005066
TC05001327.hg.1	2.13	PSR05018751.hg.1	LOC1005066
TC06001732.hg.1	4.05	PSR06020721.hg.1	TRERF1
TC06001732.hg.1	2.82	JUC06009835.hg.1	TRERF1
TC06001732.hg.1	2.78	JUC06009820.hg.1	TRERF1
TC06001732.hg.1	2.24	PSR06020697.hg.1	TRERF1
TC06001732.hg.1	-2	PSR06020715.hg.1	TRERF1
TC09001656.hg.1	4.05	JUC09011327.hg.1	FNBP1
TC09001656.hg.1	2.58	PSR09021088.hg.1	FNBP1
TC09001656.hg.1	2.49	JUC09011329.hg.1	FNBP1
TC09001656.hg.1	2.44	PSR09021116.hg.1	FNBP1
TC09001656.hg.1	2.39	JUC09011324.hg.1	FNBP1
TC09001656.hg.1	2.22	JUC09011319.hg.1	FNBP1
TC09001656.hg.1	-2.4	JUC09011321.hg.1	FNBP1
TC09001656.hg.1	-2.42	JUC09011328.hg.1	FNBP1
TC09001656.hg.1	-2.62	JUC09011317.hg.1	FNBP1
TC10001571.hg.1	4.05	JUC10011722.hg.1	SFRP5
TC10001571.hg.1	-3.2	PSR10020146.hg.1	SFRP5
TC12000726.hg.1	4.05	PSR12009221.hg.1	SOCS2
TC12000726.hg.1	3.42	PSR12009219.hg.1	SOCS2
TC12000726.hg.1	3.42	PSR12009230.hg.1	SOCS2
TC12000726.hg.1	3.15	JUC12004969.hg.1	SOCS2
TC12000726.hg.1	3.02	PSR12009231.hg.1	SOCS2
TC12000726.hg.1	2.96	PSR12009224.hg.1	SOCS2
TC12000726.hg.1	2.96	PSR12009228.hg.1	SOCS2
TC12000726.hg.1	2.88	PSR12009218.hg.1	SOCS2
TC12000726.hg.1	2.79	PSR12009222.hg.1	SOCS2
TC12000726.hg.1	2.55	PSR12009229.hg.1	SOCS2
TC12000726.hg.1	2.46	JUC12004973.hg.1	SOCS2
TC12000726.hg.1	2.42	PSR12009226.hg.1	SOCS2
TC12000726.hg.1	2.21	PSR12009220.hg.1	SOCS2
TC12000726.hg.1	2.02	PSR12009244.hg.1	SOCS2
TC12000726.hg.1	-2.02	PSR12009232.hg.1	SOCS2
TC19000623.hg.1	4.05	PSR19008634.hg.1	IGSF23
TC19000623.hg.1	3.18	PSR19008635.hg.1	IGSF23
TC19000623.hg.1	2.8	JUC19005156.hg.1	IGSF23
TC19000623.hg.1	2.77	PSR19008636.hg.1	IGSF23
TC19000623.hg.1	2.16	PSR19008639.hg.1	IGSF23
TC19000623.hg.1	-2.16	JUC19005152.hg.1	IGSF23
TC19000623.hg.1	-2.18	PSR19008644.hg.1	IGSF23
TC19000623.hg.1	-4.07	JUC19005157.hg.1	IGSF23
TC02001714.hg.1	4.04	JUC02014479.hg.1	XDH
TC02001714.hg.1	2.16	PSR02027630.hg.1	XDH
TC02001714.hg.1	2.1	JUC02014455.hg.1	XDH
TC02001714.hg.1	-2.03	JUC02014475.hg.1	XDH
TC02002172.hg.1	4.04	JUC02018252.hg.1	RGPD3
TC02002172.hg.1	2.14	PSR02035341.hg.1	RGPD3
TC03000142.hg.1	4.04	JUC03001391.hg.1	ZCWPW2

TC03000142.hg.1	2.8	JUC03001388.hg.1	ZCWPW2
TC03000142.hg.1	-5.32	PSR03002616.hg.1	ZCWPW2
TC05000336.hg.1	4.04	JUC05002465.hg.1	MAP1B
TC05000336.hg.1	-6.28	PSR05004589.hg.1	MAP1B
TC05000336.hg.1	-6.65	JUC05002459.hg.1	MAP1B
TC05001837.hg.1	4.04	JUC05012904.hg.1	SIL1
TC05001837.hg.1	2.66	JUC05012901.hg.1	SIL1
TC05001837.hg.1	2.36	JUC05012902.hg.1	SIL1
TC05001837.hg.1	2.07	PSR05025132.hg.1	SIL1
TC07001283.hg.1	4.04	JUC07009727.hg.1	KIAA0895
TC07001283.hg.1	2.44	PSR07019958.hg.1	KIAA0895
TC07001283.hg.1	2.38	PSR07019956.hg.1	KIAA0895
TC07001283.hg.1	2.33	PSR07019954.hg.1	KIAA0895
TC07001283.hg.1	2.25	PSR07019915.hg.1	KIAA0895
TC07001283.hg.1	2.14	PSR07019919.hg.1	KIAA0895
TC07001283.hg.1	2.06	PSR07019918.hg.1	KIAA0895
TC01003847.hg.1	4.02	JUC01030775.hg.1	DUSP10
TC01003847.hg.1	3.23	PSR01058741.hg.1	DUSP10
TC01003847.hg.1	3.12	PSR01058740.hg.1	DUSP10
TC01003847.hg.1	2.72	JUC01030777.hg.1	DUSP10
TC01003847.hg.1	2.21	JUC01030782.hg.1	DUSP10
TC05000243.hg.1	4.02	PSR05002987.hg.1	MAP3K1
TC05000243.hg.1	3.37	PSR05002994.hg.1	MAP3K1
TC05000243.hg.1	2.89	JUC05001672.hg.1	MAP3K1
TC05000243.hg.1	2.52	JUC05001663.hg.1	MAP3K1
TC05000243.hg.1	2.35	PSR05003002.hg.1	MAP3K1
TC05000243.hg.1	2.22	PSR05002995.hg.1	MAP3K1
TC05000243.hg.1	2.18	PSR05003006.hg.1	MAP3K1
TC05000243.hg.1	2.13	PSR05002989.hg.1	MAP3K1
TC05000243.hg.1	-2	JUC05001669.hg.1	MAP3K1
TC05000243.hg.1	-2.12	JUC05001670.hg.1	MAP3K1
TC05000243.hg.1	-2.38	JUC05001676.hg.1	MAP3K1
TC05000243.hg.1	-2.52	JUC05001665.hg.1	MAP3K1
TC11003492.hg.1	4.02	PSR11024707.hg.1	STARD10
TC11003492.hg.1	3.88	PSR11024711.hg.1	STARD10
TC11003492.hg.1	3.73	JUC11019669.hg.1	STARD10
TC11003492.hg.1	3.6	PSR11024706.hg.1	STARD10
TC11003492.hg.1	2.61	PSR11024708.hg.1	STARD10
TC11003492.hg.1	2.52	PSR11024710.hg.1	STARD10
TC11003492.hg.1	2.34	PSR11024709.hg.1	STARD10
TC12003293.hg.1	4.02	JUC12020612.hg.1	HOXC10
TC12003293.hg.1	3.92	JUC12020616.hg.1	HOXC10
TC12003293.hg.1	3.45	JUC12020615.hg.1	HOXC10
TC12003293.hg.1	3.28	PSR12005852.hg.1	HOXC10
TC12003293.hg.1	3.23	PSR12005853.hg.1	HOXC10
TC12003293.hg.1	2.55	PSR12005850.hg.1	HOXC10
TC12003293.hg.1	2.43	PSR12005854.hg.1	HOXC10
TC12003293.hg.1	2.11	PSR12005862.hg.1	HOXC10
TC12003293.hg.1	-4.16	JUC12020618.hg.1	HOXC10
TC20000271.hg.1	4.02	JUC20002127.hg.1	RPN2
TC20000271.hg.1	2.5	PSR20003818.hg.1	RPN2
TC20000271.hg.1	-3.55	JUC20002138.hg.1	RPN2
TC07000095.hg.1	4.01	JUC07000693.hg.1	SCIN
TC07000095.hg.1	3.56	JUC07000700.hg.1	SCIN
TC07000095.hg.1	2.95	JUC07000686.hg.1	SCIN
TC07000095.hg.1	2.86	JUC07000704.hg.1	SCIN
TC07000095.hg.1	2.76	PSR07001546.hg.1	SCIN
TC07000095.hg.1	2.48	JUC07000703.hg.1	SCIN

TC07000095.hg.1	2.41	PSR07001545.hg.1	SCIN
TC07000095.hg.1	2.41	PSR07001549.hg.1	SCIN
TC07000095.hg.1	2.37	JUC07000683.hg.1	SCIN
TC07000095.hg.1	2.21	PSR07001551.hg.1	SCIN
TC07000095.hg.1	2.08	PSR07001562.hg.1	SCIN
TC07000095.hg.1	2.07	PSR07001553.hg.1	SCIN
TC07000095.hg.1	2.02	PSR07001580.hg.1	SCIN
TC07000095.hg.1	2.01	PSR07001550.hg.1	SCIN
TC07000095.hg.1	-2.23	PSR07001582.hg.1	SCIN
TC07000095.hg.1	-2.27	JUC07000699.hg.1	SCIN
TC07000095.hg.1	-2.51	PSR07001583.hg.1	SCIN
TC07000095.hg.1	-3.34	JUC07000702.hg.1	SCIN
TC0X001401.hg.1	4.01	JUC0X009533.hg.1	LINC00087
TC0X001401.hg.1	-2.26	PSR0X018659.hg.1	LINC00087
TC0X001401.hg.1	-2.71	PSR0X018654.hg.1	LINC00087
TC0X001401.hg.1	-4.15	PSR0X018655.hg.1	LINC00087
TC13000364.hg.1	4.01	PSR13003896.hg.1	ITGBL1
TC13000364.hg.1	3.69	PSR13003886.hg.1	ITGBL1
TC13000364.hg.1	-2.17	JUC13002402.hg.1	ITGBL1
TC13000364.hg.1	-2.2	JUC13002403.hg.1	ITGBL1
TC20000095.hg.1	4.01	JUC20000683.hg.1	NDUFAF5
TC20000095.hg.1	3.07	JUC20000667.hg.1	NDUFAF5
TC20000095.hg.1	-2.03	PSR20001298.hg.1	NDUFAF5
TC20000095.hg.1	-2.9	JUC20000679.hg.1	NDUFAF5
TC03001941.hg.1	4	JUC03017192.hg.1	VEPH1
TC03001941.hg.1	-2.01	PSR03034731.hg.1	VEPH1
TC03001941.hg.1	-2.03	JUC03017188.hg.1	VEPH1
TC03001941.hg.1	-2.13	PSR03034749.hg.1	VEPH1
TC03001941.hg.1	-2.23	PSR03034732.hg.1	VEPH1
TC03001941.hg.1	-2.29	PSR03034733.hg.1	VEPH1
TC03001941.hg.1	-2.85	PSR03034755.hg.1	VEPH1
TC03001941.hg.1	-3.69	JUC03017183.hg.1	VEPH1
TC06000779.hg.1	4	PSR06009312.hg.1	NT5E
TC06000779.hg.1	3.05	PSR06009326.hg.1	NT5E
TC06000779.hg.1	2.72	JUC06004360.hg.1	NT5E
TC06000779.hg.1	2.33	PSR06009327.hg.1	NT5E
TC06000779.hg.1	-2.03	PSR06009319.hg.1	NT5E
TC08000401.hg.1	4	JUC08002773.hg.1	FAM110B
TC08000401.hg.1	2.58	PSR08005614.hg.1	FAM110B
TC08000401.hg.1	2.54	PSR08005600.hg.1	FAM110B
TC08000401.hg.1	2.43	PSR08005595.hg.1	FAM110B
TC08000401.hg.1	2.36	PSR08005616.hg.1	FAM110B
TC08000401.hg.1	2.34	PSR08005590.hg.1	FAM110B
TC08000401.hg.1	2.27	PSR08005612.hg.1	FAM110B
TC08000401.hg.1	2.08	PSR08005593.hg.1	FAM110B
TC08000412.hg.1	4	JUC08002857.hg.1	CHD7
TC08000412.hg.1	2.21	PSR08005744.hg.1	CHD7
TC08000412.hg.1	2.19	JUC08002869.hg.1	CHD7
TC08001191.hg.1	4	PSR08015377.hg.1	PRKDC
TC08001191.hg.1	2.49	JUC08007912.hg.1	PRKDC
TC08001191.hg.1	-2	JUC08007905.hg.1	PRKDC
TC08001191.hg.1	-2.02	JUC08007862.hg.1	PRKDC
TC08001191.hg.1	-2.05	JUC08007901.hg.1	PRKDC
TC08001191.hg.1	-2.5	JUC08007843.hg.1	PRKDC
TC08001191.hg.1	-2.51	JUC08007854.hg.1	PRKDC
TC08001191.hg.1	-2.59	JUC08007876.hg.1	PRKDC
TC08001191.hg.1	-3.06	JUC08007888.hg.1	PRKDC
TC12000647.hg.1	4	JUC12004516.hg.1	

TC12000647.hg.1	2.08	PSR12008542.hg.1	
TC12000647.hg.1	-2.03	PSR12008541.hg.1	
TC16002036.hg.1	4	JUC16013200.hg.1	CLEC18A
TC16002036.hg.1	2.96	JUC16013194.hg.1	CLEC18A
TC16002036.hg.1	2.25	PSR16008318.hg.1	CLEC18A
TC16002036.hg.1	-2.12	PSR16008298.hg.1	CLEC18A
TC19001282.hg.1	4	PSR19018215.hg.1	RAB3A
TC19001282.hg.1	3.7	JUC19010633.hg.1	RAB3A
TC19001282.hg.1	3.23	PSR19018213.hg.1	RAB3A
TC19001282.hg.1	3.13	JUC19010636.hg.1	RAB3A
TC19001282.hg.1	3.07	JUC19010631.hg.1	RAB3A
TC19001282.hg.1	3.01	PSR19018216.hg.1	RAB3A
TC19001282.hg.1	2.88	PSR19018208.hg.1	RAB3A
TC19001282.hg.1	2.26	PSR19018206.hg.1	RAB3A
TC19001282.hg.1	-2.06	JUC19010632.hg.1	RAB3A
TC02002003.hg.1	3.99	JUC02016660.hg.1	C2orf65
TC02002003.hg.1	2.43	PSR02032209.hg.1	C2orf65
TC02002003.hg.1	-2.49	JUC02016654.hg.1	C2orf65
TC02002003.hg.1	-2.95	JUC02016657.hg.1	C2orf65
TC02002003.hg.1	-4.05	PSR02032200.hg.1	C2orf65
TC02002003.hg.1	-4.15	PSR02032202.hg.1	C2orf65
TC02002003.hg.1	-5.09	JUC02016643.hg.1	C2orf65
TC03001025.hg.1	3.99	JUC03009217.hg.1	SNORA81, EI
TC03001025.hg.1	3.6	PSR03018221.hg.1	SNORA81, EI
TC03001025.hg.1	3.16	PSR03018168.hg.1	SNORA81, EI
TC03001025.hg.1	2.44	PSR03018182.hg.1	SNORA81, EI
TC03001025.hg.1	2.05	PSR03018154.hg.1	SNORA81, EI
TC03001025.hg.1	2.03	PSR03018174.hg.1	SNORA81, EI
TC03001025.hg.1	2.03	PSR03018226.hg.1	SNORA81, EI
TC04001120.hg.1	3.99	JUC04008108.hg.1	FLJ13197
TC04001120.hg.1	2.04	JUC04008115.hg.1	FLJ13197
TC04001120.hg.1	-2.27	PSR04015254.hg.1	FLJ13197
TC04001120.hg.1	-2.8	PSR04015258.hg.1	FLJ13197
TC15001191.hg.1	3.99	JUC15005588.hg.1	C15orf29
TC15001191.hg.1	2.21	JUC15005593.hg.1	C15orf29
TC15001191.hg.1	-2.05	PSR15010542.hg.1	C15orf29
TC18000127.hg.1	3.99	JUC18000988.hg.1	DSG3
TC18000127.hg.1	2.08	PSR18001432.hg.1	DSG3
TC20000824.hg.1	3.99	JUC20005891.hg.1	C20orf132
TC20000824.hg.1	3.92	JUC20005859.hg.1	C20orf132
TC20000824.hg.1	3.03	JUC20005876.hg.1	C20orf132
TC20000824.hg.1	2.83	JUC20005889.hg.1	C20orf132
TC20000824.hg.1	2.65	JUC20005858.hg.1	C20orf132
TC20000824.hg.1	2.48	PSR20011372.hg.1	C20orf132
TC20000824.hg.1	2.4	PSR20011357.hg.1	C20orf132
TC20000824.hg.1	2.16	JUC20005870.hg.1	C20orf132
TC20000824.hg.1	-2.23	JUC20005862.hg.1	C20orf132
TC20000824.hg.1	-3.46	JUC20005857.hg.1	C20orf132
TC02002148.hg.1	3.98	JUC02018111.hg.1	MFSD9
TC02002148.hg.1	3.39	JUC02018101.hg.1	MFSD9
TC02002148.hg.1	2.24	PSR02035124.hg.1	MFSD9
TC02002148.hg.1	2.24	JUC02018110.hg.1	MFSD9
TC02002148.hg.1	-2.62	JUC02018102.hg.1	MFSD9
TC10001020.hg.1	3.98	JUC10007346.hg.1	ITIH5
TC10001020.hg.1	3.47	PSR10012689.hg.1	ITIH5
TC10001020.hg.1	3.45	JUC10007350.hg.1	ITIH5
TC10001020.hg.1	3.43	PSR10012713.hg.1	ITIH5
TC10001020.hg.1	2.96	JUC10007343.hg.1	ITIH5

TC10001020.hg.1	2.72	PSR10012695.hg.1	ITIH5
TC10001020.hg.1	-2.05	PSR10012690.hg.1	ITIH5
TC10001020.hg.1	-2.27	PSR10012708.hg.1	ITIH5
TC10001020.hg.1	-5.63	JUC10007354.hg.1	ITIH5
TC15000858.hg.1	3.98	PSR15007991.hg.1	SEMA4B
TC15000858.hg.1	3.89	PSR15007987.hg.1	SEMA4B
TC15000858.hg.1	3.2	JUC15004250.hg.1	SEMA4B
TC15000858.hg.1	3.03	JUC15004251.hg.1	SEMA4B
TC15000858.hg.1	2.69	PSR15007990.hg.1	SEMA4B
TC15000858.hg.1	2.55	JUC15004255.hg.1	SEMA4B
TC15000858.hg.1	2.51	JUC15004258.hg.1	SEMA4B
TC15000858.hg.1	2.32	PSR15007992.hg.1	SEMA4B
TC15000858.hg.1	-2.07	JUC15004264.hg.1	SEMA4B
TC15000858.hg.1	-2.08	PSR15008014.hg.1	SEMA4B
TC15000858.hg.1	-2.12	PSR15007996.hg.1	SEMA4B
TC15000858.hg.1	-2.12	PSR15008013.hg.1	SEMA4B
TC15000858.hg.1	-2.21	JUC15004253.hg.1	SEMA4B
TC15000858.hg.1	-2.22	JUC15004257.hg.1	SEMA4B
TC15000858.hg.1	-2.35	JUC15004261.hg.1	SEMA4B
TC15000858.hg.1	-2.49	JUC15004259.hg.1	SEMA4B
TC15000858.hg.1	-2.54	JUC15004252.hg.1	SEMA4B
TC15000858.hg.1	-2.68	PSR15007995.hg.1	SEMA4B
TC15000858.hg.1	-3.6	JUC15004247.hg.1	SEMA4B
TC15000858.hg.1	-7.37	PSR15007994.hg.1	SEMA4B
TC02001333.hg.1	3.97	JUC02011138.hg.1	STK11IP
TC02001333.hg.1	-2.06	PSR02020899.hg.1	STK11IP
TC02001333.hg.1	-2.08	JUC02011135.hg.1	STK11IP
TC02001333.hg.1	-3.04	JUC02011111.hg.1	STK11IP
TC03002088.hg.1	3.97	JUC03018345.hg.1	RFC4
TC03002088.hg.1	3.37	PSR03037099.hg.1	RFC4
TC11001948.hg.1	3.97	PSR11023396.hg.1	FOSL1
TC11001948.hg.1	3.13	PSR11023393.hg.1	FOSL1
TC11001948.hg.1	3.07	PSR11023395.hg.1	FOSL1
TC11001948.hg.1	2.8	JUC11012345.hg.1	FOSL1
TC11001948.hg.1	-2.2	PSR11023382.hg.1	FOSL1
TC14000393.hg.1	3.97	JUC14002280.hg.1	PLEKHG3
TC14000393.hg.1	3.73	JUC14002287.hg.1	PLEKHG3
TC14000393.hg.1	2.59	JUC14002298.hg.1	PLEKHG3
TC14000393.hg.1	2.53	PSR14004516.hg.1	PLEKHG3
TC14000393.hg.1	2.41	PSR14004492.hg.1	PLEKHG3
TC14000393.hg.1	2.33	PSR14004508.hg.1	PLEKHG3
TC14000393.hg.1	2.27	PSR14004515.hg.1	PLEKHG3
TC14000393.hg.1	2.14	PSR14004510.hg.1	PLEKHG3
TC14000393.hg.1	2.04	PSR14004511.hg.1	PLEKHG3
TC14000393.hg.1	2.03	JUC14002297.hg.1	PLEKHG3
TC14000393.hg.1	-2.18	PSR14004501.hg.1	PLEKHG3
TC14000393.hg.1	-2.31	JUC14002283.hg.1	PLEKHG3
TC14000393.hg.1	-2.54	PSR14004497.hg.1	PLEKHG3
TC14000393.hg.1	-2.59	JUC14002289.hg.1	PLEKHG3
TC14000393.hg.1	-2.63	JUC14002281.hg.1	PLEKHG3
TC02002495.hg.1	3.96	PSR02039454.hg.1	TTC21B, LOC
TC02002495.hg.1	3.88	JUC02020714.hg.1	TTC21B, LOC
TC02002495.hg.1	2.34	JUC02020718.hg.1	TTC21B, LOC
TC02002495.hg.1	-2.22	JUC02020726.hg.1	TTC21B, LOC
TC15000142.hg.1	3.96	PSR15000881.hg.1	
TC15000142.hg.1	-2.27	JUC15000299.hg.1	
TC01002658.hg.1	3.95	JUC01022286.hg.1	ORC1
TC01002658.hg.1	-2.21	PSR01041689.hg.1	ORC1

TC03003399.hg.1	3.95	JUC03024494.hg.1	TRIM59
TC03003399.hg.1	-2.13	PSR03034979.hg.1	TRIM59
TC03003399.hg.1	-3.19	PSR03034976.hg.1	TRIM59
TC03003399.hg.1	-3.93	JUC03024496.hg.1	TRIM59
TC05000218.hg.1	3.95	JUC05001492.hg.1	ITGA2
TC05000218.hg.1	2.85	PSR05002682.hg.1	ITGA2
TC05000218.hg.1	2.29	JUC05001513.hg.1	ITGA2
TC05000218.hg.1	2.03	PSR05002673.hg.1	ITGA2
TC05000218.hg.1	-2.02	JUC05001495.hg.1	ITGA2
TC06000621.hg.1	3.95	JUC06003346.hg.1	RUNX2
TC06000621.hg.1	2.72	PSR06007558.hg.1	RUNX2
TC06000621.hg.1	2.38	PSR06007553.hg.1	RUNX2
TC06000621.hg.1	2.16	PSR06007554.hg.1	RUNX2
TC06000621.hg.1	2.09	PSR06007557.hg.1	RUNX2
TC06000621.hg.1	-2.07	JUC06003345.hg.1	RUNX2
TC0X002319.hg.1	3.95	JUC0X013000.hg.1	ARMCX5
TC0X002319.hg.1	-2.01	PSR0X006450.hg.1	ARMCX5
TC12000386.hg.1	3.95	JUC12002632.hg.1	ASIC1
TC12000386.hg.1	3.75	JUC12002639.hg.1	ASIC1
TC12000386.hg.1	3.08	PSR12004795.hg.1	ASIC1
TC12000386.hg.1	2.65	PSR12004803.hg.1	ASIC1
TC12000386.hg.1	2.3	JUC12002629.hg.1	ASIC1
TC12000386.hg.1	2.02	PSR12004789.hg.1	ASIC1
TC12001566.hg.1	3.95	JUC12011514.hg.1	CBX5, MIR31
TC12001566.hg.1	3.25	PSR12020755.hg.1	CBX5, MIR31
TC12001566.hg.1	2.85	JUC12011515.hg.1	CBX5, MIR31
TC12001566.hg.1	2.59	JUC12011511.hg.1	CBX5, MIR31
TC12001566.hg.1	-3.48	JUC12011512.hg.1	CBX5, MIR31
TC21001057.hg.1	3.95	JUC21005047.hg.1	CRYZL1
TC21001057.hg.1	2.45	PSR21004837.hg.1	CRYZL1
TC21001057.hg.1	2.23	JUC21005057.hg.1	CRYZL1
TC21001057.hg.1	2	PSR21004782.hg.1	CRYZL1
TC6_qbl_hap6000(	3.95	JUC6_qbl_hap60003	PSORS1C1
TC6_qbl_hap6000(	2.58	JUC6_qbl_hap60003	PSORS1C1
TC6_qbl_hap6000(	2.48	JUC6_qbl_hap60003	PSORS1C1
TC6_qbl_hap6000(	2.23	PSR6_qbl_hap60008	PSORS1C1
TC6_qbl_hap6000(	-2.23	PSR6_qbl_hap60008	PSORS1C1
TC01002171.hg.1	3.94	JUC01017932.hg.1	RERE
TC01002171.hg.1	2.83	PSR01033455.hg.1	RERE
TC01002171.hg.1	2.29	JUC01017948.hg.1	RERE
TC01002171.hg.1	2.08	JUC01017940.hg.1	RERE
TC01002171.hg.1	2	JUC01017933.hg.1	RERE
TC01002171.hg.1	-2.32	JUC01017959.hg.1	RERE
TC01002171.hg.1	-2.42	JUC01017958.hg.1	RERE
TC01002171.hg.1	-2.48	JUC01017943.hg.1	RERE
TC01002171.hg.1	-3.35	JUC01017924.hg.1	RERE
TC01002377.hg.1	3.94	PSR01036653.hg.1	RHCE
TC01002377.hg.1	3.65	JUC01019731.hg.1	RHCE
TC01002377.hg.1	3.18	JUC01019746.hg.1	RHCE
TC01002377.hg.1	3.14	JUC01019738.hg.1	RHCE
TC01002377.hg.1	3.03	JUC01019742.hg.1	RHCE
TC01002377.hg.1	2.52	JUC01019743.hg.1	RHCE
TC01002377.hg.1	2.3	PSR01036638.hg.1	RHCE
TC01002377.hg.1	2.26	JUC01019729.hg.1	RHCE
TC01002377.hg.1	2.18	JUC01019747.hg.1	RHCE
TC01002377.hg.1	-2.16	PSR01036632.hg.1	RHCE
TC01002377.hg.1	-2.36	PSR01036635.hg.1	RHCE
TC01002377.hg.1	-3.19	JUC01019730.hg.1	RHCE

TC01002377.hg.1	-3.76	PSR01036633.hg.1	RHCE
TC02001645.hg.1	3.94	JUC02013681.hg.1	TP53I3
TC02001645.hg.1	2.31	PSR02026226.hg.1	TP53I3
TC02001645.hg.1	-2.02	PSR02026238.hg.1	TP53I3
TC02001645.hg.1	-2.06	PSR02026241.hg.1	TP53I3
TC02001645.hg.1	-2.08	PSR02026239.hg.1	TP53I3
TC02001645.hg.1	-2.2	PSR02026240.hg.1	TP53I3
TC02001645.hg.1	-2.61	PSR02026235.hg.1	TP53I3
TC03003380.hg.1	3.94	JUC03024127.hg.1	PFKFB4
TC03003380.hg.1	2.39	JUC03024135.hg.1	PFKFB4
TC03003380.hg.1	2.27	PSR03024021.hg.1	PFKFB4
TC03003380.hg.1	2.21	JUC03024136.hg.1	PFKFB4
TC03003380.hg.1	2.19	JUC03024132.hg.1	PFKFB4
TC03003380.hg.1	2.02	JUC03024131.hg.1	PFKFB4
TC03003380.hg.1	-2.02	JUC03024124.hg.1	PFKFB4
TC03003380.hg.1	-2.75	PSR03024007.hg.1	PFKFB4
TC05001797.hg.1	3.94	PSR05024439.hg.1	PITX1
TC05001797.hg.1	3.01	JUC05012561.hg.1	PITX1
TC05001797.hg.1	2.92	JUC05012563.hg.1	PITX1
TC05001797.hg.1	2.44	PSR05024452.hg.1	PITX1
TC05001797.hg.1	2.26	PSR05024453.hg.1	PITX1
TC05001797.hg.1	2.25	PSR05024456.hg.1	PITX1
TC05001797.hg.1	2.2	PSR05024460.hg.1	PITX1
TC05001797.hg.1	2.07	JUC05012558.hg.1	PITX1
TC05001797.hg.1	2.04	PSR05024454.hg.1	PITX1
TC05001797.hg.1	2.04	JUC05012562.hg.1	PITX1
TC05001996.hg.1	3.94	JUC05014162.hg.1	RNF145
TC05001996.hg.1	2.56	PSR05027794.hg.1	RNF145
TC05001996.hg.1	2.52	JUC05014161.hg.1	RNF145
TC05001996.hg.1	2.15	JUC05014174.hg.1	RNF145
TC14000908.hg.1	3.94	JUC14004978.hg.1	NDRG2
TC14000908.hg.1	3.9	JUC14004988.hg.1	NDRG2
TC14000908.hg.1	3.85	JUC14004989.hg.1	NDRG2
TC14000908.hg.1	3.84	PSR14009854.hg.1	NDRG2
TC14000908.hg.1	3.8	PSR14009844.hg.1	NDRG2
TC14000908.hg.1	3.8	PSR14009845.hg.1	NDRG2
TC14000908.hg.1	3.78	PSR14009846.hg.1	NDRG2
TC14000908.hg.1	3.54	JUC14004994.hg.1	NDRG2
TC14000908.hg.1	3.51	PSR14009812.hg.1	NDRG2
TC14000908.hg.1	3.12	JUC14004997.hg.1	NDRG2
TC14000908.hg.1	3.09	PSR14009807.hg.1	NDRG2
TC14000908.hg.1	3.06	JUC14004969.hg.1	NDRG2
TC14000908.hg.1	3	JUC14004993.hg.1	NDRG2
TC14000908.hg.1	2.94	PSR14009833.hg.1	NDRG2
TC14000908.hg.1	2.93	PSR14009835.hg.1	NDRG2
TC14000908.hg.1	2.92	JUC14004982.hg.1	NDRG2
TC14000908.hg.1	2.83	JUC14004979.hg.1	NDRG2
TC14000908.hg.1	2.76	PSR14009803.hg.1	NDRG2
TC14000908.hg.1	2.72	PSR14009802.hg.1	NDRG2
TC14000908.hg.1	2.65	PSR14009828.hg.1	NDRG2
TC14000908.hg.1	2.63	PSR14009849.hg.1	NDRG2
TC14000908.hg.1	2.53	PSR14009857.hg.1	NDRG2
TC14000908.hg.1	2.5	PSR14009827.hg.1	NDRG2
TC14000908.hg.1	2.21	JUC14004966.hg.1	NDRG2
TC14000908.hg.1	2.13	JUC14004985.hg.1	NDRG2
TC14000908.hg.1	2.02	PSR14009832.hg.1	NDRG2
TC14000908.hg.1	2.01	JUC14004992.hg.1	NDRG2
TC14000908.hg.1	-2.06	JUC14004995.hg.1	NDRG2

TC14000908.hg.1	-2.1	JUC14004971.hg.1	NDRG2
TC14000908.hg.1	-2.22	JUC14004986.hg.1	NDRG2
TC14000908.hg.1	-2.52	JUC14004991.hg.1	NDRG2
TC14000908.hg.1	-2.68	PSR14009821.hg.1	NDRG2
TC14000908.hg.1	-2.97	PSR14009817.hg.1	NDRG2
TC14000908.hg.1	-3.6	JUC14004967.hg.1	NDRG2
TC14000908.hg.1	-3.91	PSR14009815.hg.1	NDRG2
TC14000908.hg.1	-3.92	JUC14004983.hg.1	NDRG2
TC14000908.hg.1	-4.44	PSR14009814.hg.1	NDRG2
TC14000908.hg.1	-5.05	JUC14004974.hg.1	NDRG2
TC14000908.hg.1	-6.53	JUC14004968.hg.1	NDRG2
TC15000162.hg.1	3.94	JUC15000416.hg.1	LOC653075,
TC15000162.hg.1	-2.06	PSR15001052.hg.1	LOC653075,
TC17000103.hg.1	3.94	PSR17001449.hg.1	CD68
TC17000103.hg.1	2.69	JUC17000801.hg.1	CD68
TC22000801.hg.1	3.94	JUC22005603.hg.1	CBX7
TC22000801.hg.1	2.97	JUC22005606.hg.1	CBX7
TC22000801.hg.1	2.7	PSR22013597.hg.1	CBX7
TC22000801.hg.1	2.27	JUC22005601.hg.1	CBX7
TC22000801.hg.1	2.26	PSR22013603.hg.1	CBX7
TC22000801.hg.1	2.21	PSR22013594.hg.1	CBX7
TC22000801.hg.1	2.09	PSR22013598.hg.1	CBX7
TC22000801.hg.1	2.02	PSR22013607.hg.1	CBX7
TC02000074.hg.1	3.93	PSR02000919.hg.1	GREB1
TC02000074.hg.1	2.05	JUC02000517.hg.1	GREB1
TC02000074.hg.1	-2.11	JUC02000491.hg.1	GREB1
TC02000074.hg.1	-2.52	JUC02000485.hg.1	GREB1
TC06002090.hg.1	3.93	PSR06026160.hg.1	SOGA3, KIAA
TC06002090.hg.1	2.78	JUC06012908.hg.1	SOGA3, KIAA
TC06002090.hg.1	2.34	PSR06026165.hg.1	SOGA3, KIAA
TC06002090.hg.1	2.28	PSR06026164.hg.1	SOGA3, KIAA
TC06002090.hg.1	2.16	PSR06026154.hg.1	SOGA3, KIAA
TC12000096.hg.1	3.93	PSR12001169.hg.1	TPI1
TC12000096.hg.1	3.43	PSR12001184.hg.1	TPI1
TC12000096.hg.1	3.35	JUC12000613.hg.1	TPI1
TC12000096.hg.1	3.1	PSR12001189.hg.1	TPI1
TC03000444.hg.1	3.92	JUC03004405.hg.1	ROBO2
TC03000444.hg.1	3.3	PSR03008868.hg.1	ROBO2
TC03000444.hg.1	2.93	JUC03004420.hg.1	ROBO2
TC03000444.hg.1	2.76	PSR03008871.hg.1	ROBO2
TC03000444.hg.1	2.69	PSR03008929.hg.1	ROBO2
TC03000444.hg.1	2.64	JUC03004428.hg.1	ROBO2
TC03000444.hg.1	2.64	JUC03004432.hg.1	ROBO2
TC03000444.hg.1	2.63	JUC03004393.hg.1	ROBO2
TC03000444.hg.1	2.46	JUC03004403.hg.1	ROBO2
TC03000444.hg.1	2.35	PSR03008892.hg.1	ROBO2
TC03000444.hg.1	2.33	JUC03004404.hg.1	ROBO2
TC03000444.hg.1	2.22	PSR03008866.hg.1	ROBO2
TC03000444.hg.1	2.08	PSR03008900.hg.1	ROBO2
TC03000444.hg.1	2.05	PSR03008889.hg.1	ROBO2
TC03000444.hg.1	2.04	JUC03004391.hg.1	ROBO2
TC03000444.hg.1	2.01	PSR03008920.hg.1	ROBO2
TC03000444.hg.1	-2.05	JUC03004421.hg.1	ROBO2
TC03000444.hg.1	-2.13	PSR03008878.hg.1	ROBO2
TC03000444.hg.1	-2.21	PSR03008885.hg.1	ROBO2
TC03000444.hg.1	-3.36	JUC03004402.hg.1	ROBO2
TC03000444.hg.1	-3.38	PSR03008899.hg.1	ROBO2
TC03000444.hg.1	-3.41	JUC03004401.hg.1	ROBO2

TC03000444.hg.1	-3.45	PSR03008880.hg.1	ROBO2
TC03000444.hg.1	-4.13	JUC03004419.hg.1	ROBO2
TC09000119.hg.1	3.92	JUC09000723.hg.1	IFT74
TC09000119.hg.1	3.25	JUC09000719.hg.1	IFT74
TC09000119.hg.1	2.55	JUC09000729.hg.1	IFT74
TC09000119.hg.1	2.38	JUC09000731.hg.1	IFT74
TC09000119.hg.1	2.28	JUC09000740.hg.1	IFT74
TC09000119.hg.1	2.04	PSR09001366.hg.1	IFT74
TC09000119.hg.1	-3.5	JUC09000743.hg.1	IFT74
TC09000119.hg.1	-3.72	PSR09001369.hg.1	IFT74
TC09002910.hg.1	3.92	JUC09015952.hg.1	PTGDS
TC09002910.hg.1	2.16	JUC09015962.hg.1	PTGDS
TC09002910.hg.1	-2.6	PSR09010918.hg.1	PTGDS
TC09002910.hg.1	-4.57	PSR09010934.hg.1	PTGDS
TC09002910.hg.1	-4.6	PSR09010931.hg.1	PTGDS
TC09002910.hg.1	-6.65	PSR09010937.hg.1	PTGDS
TC09002910.hg.1	-7.67	PSR09010926.hg.1	PTGDS
TC09002910.hg.1	-8.24	PSR09010914.hg.1	PTGDS
TC09002910.hg.1	-10.11	PSR09010921.hg.1	PTGDS
TC09002910.hg.1	-10.92	PSR09010930.hg.1	PTGDS
TC09002910.hg.1	-11.92	JUC09015957.hg.1	PTGDS
TC09002910.hg.1	-13.87	PSR09010920.hg.1	PTGDS
TC09002910.hg.1	-14.98	PSR09010912.hg.1	PTGDS
TC09002910.hg.1	-16.39	PSR09010927.hg.1	PTGDS
TC09002910.hg.1	-18.59	JUC09015961.hg.1	PTGDS
TC19000191.hg.1	3.92	JUC19001725.hg.1	LDLR
TC19000191.hg.1	2.63	JUC19001723.hg.1	LDLR
TC19000191.hg.1	2.41	JUC19001741.hg.1	LDLR
TC19000191.hg.1	2.31	JUC19001718.hg.1	LDLR
TC19000191.hg.1	2.01	PSR19002943.hg.1	LDLR
TC19000191.hg.1	-2.68	PSR19002974.hg.1	LDLR
TC22000265.hg.1	3.92	JUC22001809.hg.1	APOL1
TC22000265.hg.1	-2.04	PSR22004921.hg.1	APOL1
TC22000265.hg.1	-2.08	PSR22004934.hg.1	APOL1
TC22000265.hg.1	-2.6	JUC22001794.hg.1	APOL1
TC22000265.hg.1	-2.7	PSR22004908.hg.1	APOL1
TC22000265.hg.1	-2.8	PSR22004924.hg.1	APOL1
TC22000265.hg.1	-3.1	JUC22001807.hg.1	APOL1
TC22000265.hg.1	-3.19	PSR22004919.hg.1	APOL1
TC22000265.hg.1	-3.21	PSR22004920.hg.1	APOL1
TC22000265.hg.1	-3.28	JUC22001804.hg.1	APOL1
TC22000265.hg.1	-3.3	JUC22001812.hg.1	APOL1
TC22000265.hg.1	-3.35	JUC22001800.hg.1	APOL1
TC22000265.hg.1	-3.74	PSR22004943.hg.1	APOL1
TC22000265.hg.1	-3.79	PSR22004925.hg.1	APOL1
TC22000265.hg.1	-3.82	PSR22004926.hg.1	APOL1
TC22000265.hg.1	-3.98	PSR22004940.hg.1	APOL1
TC22000265.hg.1	-4	PSR22004937.hg.1	APOL1
TC22000265.hg.1	-4.38	JUC22001798.hg.1	APOL1
TC22000265.hg.1	-4.43	JUC22001803.hg.1	APOL1
TC22000265.hg.1	-4.53	JUC22001801.hg.1	APOL1
TC22000265.hg.1	-5.42	JUC22001796.hg.1	APOL1
TC22001423.hg.1	3.92	JUC22008575.hg.1	SFI1
TC22001423.hg.1	2.91	JUC22008522.hg.1	SFI1
TC22001423.hg.1	2.57	JUC22008532.hg.1	SFI1
TC22001423.hg.1	2.23	JUC22008524.hg.1	SFI1
TC22001423.hg.1	2.14	PSR22004392.hg.1	SFI1
TC04000086.hg.1	3.91	JUC04000830.hg.1	SH3TC1

TC04000086.hg.1	2.42	JUC04000866.hg.1	SH3TC1
TC04000086.hg.1	2.36	PSR04001828.hg.1	SH3TC1
TC04000086.hg.1	-2.23	PSR04001848.hg.1	SH3TC1
TC04000086.hg.1	-2.76	JUC04000859.hg.1	SH3TC1
TC04000086.hg.1	-2.9	PSR04001799.hg.1	SH3TC1
TC04000086.hg.1	-3.08	JUC04000858.hg.1	SH3TC1
TC04000086.hg.1	-4	JUC04000838.hg.1	SH3TC1
TC04001700.hg.1	3.91	JUC04012449.hg.1	1-Mar
TC04001700.hg.1	3.05	JUC04012436.hg.1	1-Mar
TC04001700.hg.1	2.71	PSR04023550.hg.1	1-Mar
TC04001700.hg.1	2.65	JUC04012434.hg.1	1-Mar
TC04001700.hg.1	2.6	PSR04023543.hg.1	1-Mar
TC04001700.hg.1	2.45	PSR04023551.hg.1	1-Mar
TC04001700.hg.1	2.39	JUC04012435.hg.1	1-Mar
TC04001700.hg.1	2.36	JUC04012438.hg.1	1-Mar
TC04001700.hg.1	2.35	PSR04023517.hg.1	1-Mar
TC04001700.hg.1	2.31	PSR04023519.hg.1	1-Mar
TC04001700.hg.1	2.29	PSR04023553.hg.1	1-Mar
TC04001700.hg.1	2.29	JUC04012446.hg.1	1-Mar
TC04001700.hg.1	2.16	JUC04012451.hg.1	1-Mar
TC04001700.hg.1	2.1	PSR04023546.hg.1	1-Mar
TC04001700.hg.1	2.09	JUC04012443.hg.1	1-Mar
TC04001700.hg.1	2.02	PSR04023520.hg.1	1-Mar
TC04001700.hg.1	-2.12	JUC04012452.hg.1	1-Mar
TC04001700.hg.1	-2.14	PSR04023511.hg.1	1-Mar
TC04001700.hg.1	-2.58	PSR04023542.hg.1	1-Mar
TC04001700.hg.1	-3.83	JUC04012431.hg.1	1-Mar
TC04001700.hg.1	-5.6	PSR04023536.hg.1	1-Mar
TC02002668.hg.1	3.9	JUC02022407.hg.1	PPIL3
TC02002668.hg.1	-2.69	PSR02042844.hg.1	PPIL3
TC03001312.hg.1	3.9	JUC03011300.hg.1	ULK4
TC03001312.hg.1	3.64	PSR03022546.hg.1	ULK4
TC03001312.hg.1	3.37	PSR03022543.hg.1	ULK4
TC03001312.hg.1	2.79	JUC03011286.hg.1	ULK4
TC03001312.hg.1	2.61	JUC03011273.hg.1	ULK4
TC03001312.hg.1	-2.18	PSR03022563.hg.1	ULK4
TC03001312.hg.1	-2.48	JUC03011288.hg.1	ULK4
TC03001312.hg.1	-2.53	JUC03011294.hg.1	ULK4
TC03001312.hg.1	-2.62	PSR03022566.hg.1	ULK4
TC03001312.hg.1	-2.87	JUC03011274.hg.1	ULK4
TC03001312.hg.1	-3.03	JUC03011267.hg.1	ULK4
TC0X000393.hg.1	3.9	PSR0X005382.hg.1	NHSL2
TC0X000393.hg.1	3.18	JUC0X002668.hg.1	NHSL2
TC0X000393.hg.1	3.12	PSR0X005385.hg.1	NHSL2
TC0X000393.hg.1	-2.05	JUC0X002663.hg.1	NHSL2
TC10000757.hg.1	3.9	JUC10005006.hg.1	TMEM180
TC10000757.hg.1	3.36	PSR10008985.hg.1	TMEM180
TC10000757.hg.1	3.14	JUC10005015.hg.1	TMEM180
TC10000757.hg.1	2.91	JUC10005008.hg.1	TMEM180
TC10000757.hg.1	2.73	JUC10005011.hg.1	TMEM180
TC10000757.hg.1	2.49	JUC10005013.hg.1	TMEM180
TC10000757.hg.1	2.48	PSR10009011.hg.1	TMEM180
TC10000757.hg.1	2.17	PSR10009001.hg.1	TMEM180
TC10000757.hg.1	2.15	PSR10009009.hg.1	TMEM180
TC10000757.hg.1	2.11	PSR10008982.hg.1	TMEM180
TC10000757.hg.1	2.07	PSR10009005.hg.1	TMEM180
TC13000157.hg.1	3.9	JUC13001136.hg.1	DNAJC15
TC13000157.hg.1	2.43	PSR13001832.hg.1	DNAJC15

TC15001641.hg.1	3.9	JUC15008640.hg.1	STRA6
TC15001641.hg.1	2.94	JUC15008628.hg.1	STRA6
TC15001641.hg.1	2.52	JUC15008642.hg.1	STRA6
TC15001641.hg.1	2.04	PSR15015937.hg.1	STRA6
TC16001266.hg.1	3.9	JUC16010005.hg.1	CLEC18B
TC16001266.hg.1	2.88	JUC16010010.hg.1	CLEC18B
TC16001266.hg.1	-2.43	PSR16017601.hg.1	CLEC18B
TC16001266.hg.1	-2.93	PSR16017606.hg.1	CLEC18B
TC20000732.hg.1	3.9	JUC20005171.hg.1	ABHD12
TC20000732.hg.1	3.49	JUC20005179.hg.1	ABHD12
TC20000732.hg.1	3.3	JUC20005178.hg.1	ABHD12
TC20000732.hg.1	3.2	PSR20009911.hg.1	ABHD12
TC20000732.hg.1	2.5	PSR20009898.hg.1	ABHD12
TC20000732.hg.1	2.43	PSR20009923.hg.1	ABHD12
TC20000732.hg.1	2.39	PSR20009914.hg.1	ABHD12
TC20000732.hg.1	2.28	JUC20005167.hg.1	ABHD12
TC20000732.hg.1	2.04	PSR20009909.hg.1	ABHD12
TC20000732.hg.1	2	PSR20009920.hg.1	ABHD12
TC20000732.hg.1	2	JUC20005177.hg.1	ABHD12
TC20000732.hg.1	-3.68	JUC20005181.hg.1	ABHD12
TC01002657.hg.1	3.89	JUC01022241.hg.1	CC2D1B
TC01002657.hg.1	2.44	PSR01041634.hg.1	CC2D1B
TC01002657.hg.1	2.36	PSR01041625.hg.1	CC2D1B
TC01002657.hg.1	2.19	JUC01022255.hg.1	CC2D1B
TC01002657.hg.1	2.09	PSR01041619.hg.1	CC2D1B
TC01002657.hg.1	2.06	JUC01022274.hg.1	CC2D1B
TC01002657.hg.1	-2.68	JUC01022264.hg.1	CC2D1B
TC07000477.hg.1	3.89	JUC07003234.hg.1	POR, MIR465
TC07000477.hg.1	2.93	JUC07003237.hg.1	POR, MIR465
TC07000477.hg.1	2.8	PSR07006635.hg.1	POR, MIR465
TC07000477.hg.1	2.06	PSR07006646.hg.1	POR, MIR465
TC19001689.hg.1	3.89	JUC19013153.hg.1	CA11
TC19001689.hg.1	-2.01	PSR19023048.hg.1	CA11
TC19001689.hg.1	-2.21	JUC19013155.hg.1	CA11
TC02001653.hg.1	3.88	PSR02026387.hg.1	ADCY3
TC02001653.hg.1	3.66	PSR02026405.hg.1	ADCY3
TC02001653.hg.1	3.28	PSR02026406.hg.1	ADCY3
TC02001653.hg.1	2.83	PSR02026356.hg.1	ADCY3
TC02001653.hg.1	2.58	JUC02013770.hg.1	ADCY3
TC02001653.hg.1	2.48	PSR02026376.hg.1	ADCY3
TC02001653.hg.1	2.44	JUC02013762.hg.1	ADCY3
TC02001653.hg.1	2.42	PSR02026357.hg.1	ADCY3
TC02001653.hg.1	2.28	PSR02026397.hg.1	ADCY3
TC02001653.hg.1	2.21	PSR02026384.hg.1	ADCY3
TC02001653.hg.1	2.16	JUC02013771.hg.1	ADCY3
TC02001653.hg.1	2.15	PSR02026394.hg.1	ADCY3
TC02001653.hg.1	2.12	JUC02013774.hg.1	ADCY3
TC02001653.hg.1	2.11	PSR02026393.hg.1	ADCY3
TC02001653.hg.1	2.09	PSR02026360.hg.1	ADCY3
TC02001653.hg.1	2.08	PSR02026400.hg.1	ADCY3
TC02001653.hg.1	2.04	JUC02013784.hg.1	ADCY3
TC02001653.hg.1	2.04	JUC02013785.hg.1	ADCY3
TC02001653.hg.1	2.02	JUC02013753.hg.1	ADCY3
TC02001653.hg.1	-2.05	JUC02013780.hg.1	ADCY3
TC02001653.hg.1	-2.39	JUC02013786.hg.1	ADCY3
TC06000389.hg.1	3.88	JUC06001675.hg.1	SKIV2L
TC06000389.hg.1	2.06	PSR06004160.hg.1	SKIV2L
TC06000389.hg.1	-2.59	JUC06001678.hg.1	SKIV2L

TC07000443.hg.1	3.88	JUC07002831.hg.1	WBSCR22
TC07000443.hg.1	2.47	PSR07005918.hg.1	WBSCR22
TC07000443.hg.1	2.08	JUC07002842.hg.1	WBSCR22
TC07000443.hg.1	-2	JUC07002847.hg.1	WBSCR22
TC09001508.hg.1	3.88	PSR09018920.hg.1	AKNA
TC09001508.hg.1	3.33	JUC09010213.hg.1	AKNA
TC09001508.hg.1	2.16	PSR09018890.hg.1	AKNA
TC09001508.hg.1	-2.16	JUC09010231.hg.1	AKNA
TC16002056.hg.1	3.88	JUC16013475.hg.1	NFATC3
TC16002056.hg.1	2.54	PSR16007940.hg.1	NFATC3
TC16002056.hg.1	2.41	PSR16007946.hg.1	NFATC3
TC16002056.hg.1	2.06	PSR16007939.hg.1	NFATC3
TC17001700.hg.1	3.88	JUC17012691.hg.1	SPAG9
TC17001700.hg.1	3.86	PSR17022875.hg.1	SPAG9
TC17001700.hg.1	2.81	PSR17022874.hg.1	SPAG9
TC17001700.hg.1	2.69	JUC17012709.hg.1	SPAG9
TC17001700.hg.1	-2.02	JUC17012674.hg.1	SPAG9
TC19001501.hg.1	3.88	JUC19011732.hg.1	MAP4K1
TC19001501.hg.1	2.03	PSR19020399.hg.1	MAP4K1
TC01000271.hg.1	3.87	JUC01002233.hg.1	NBPF3, LOC
TC01000271.hg.1	2.5	PSR01004180.hg.1	NBPF3, LOC
TC01000271.hg.1	2.15	JUC01002241.hg.1	NBPF3, LOC
TC01000271.hg.1	-3.16	JUC01002236.hg.1	NBPF3, LOC
TC02001447.hg.1	3.87	JUC02012183.hg.1	RAMP1
TC02001447.hg.1	2.1	PSR02023202.hg.1	RAMP1
TC02001447.hg.1	2.07	PSR02023205.hg.1	RAMP1
TC02001447.hg.1	-2.02	JUC02012181.hg.1	RAMP1
TC02001447.hg.1	-2.42	PSR02023198.hg.1	RAMP1
TC02001447.hg.1	-2.61	PSR02023204.hg.1	RAMP1
TC02001447.hg.1	-2.63	JUC02012180.hg.1	RAMP1
TC02001891.hg.1	3.87	JUC02015921.hg.1	USP34
TC02001891.hg.1	3.01	PSR02030123.hg.1	USP34
TC02001891.hg.1	-2.18	JUC02015854.hg.1	USP34
TC03001625.hg.1	3.87	JUC03014490.hg.1	LOC1003026
TC03001625.hg.1	3.38	PSR03029335.hg.1	LOC1003026
TC03001625.hg.1	2.79	JUC03014492.hg.1	LOC1003026
TC03001625.hg.1	2.75	JUC03014485.hg.1	LOC1003026
TC03001625.hg.1	2.59	JUC03014493.hg.1	LOC1003026
TC03003344.hg.1	3.87	JUC03023552.hg.1	TTLL3
TC03003344.hg.1	2.26	JUC03023558.hg.1	TTLL3
TC03003344.hg.1	2.16	JUC03023548.hg.1	TTLL3
TC03003344.hg.1	2.05	JUC03023568.hg.1	TTLL3
TC03003344.hg.1	-2.02	PSR03000946.hg.1	TTLL3
TC05001151.hg.1	3.87	JUC05008405.hg.1	SEMA5A
TC05001151.hg.1	2.83	JUC05008408.hg.1	SEMA5A
TC05001151.hg.1	2.29	JUC05008396.hg.1	SEMA5A
TC05001151.hg.1	2.24	PSR05016506.hg.1	SEMA5A
TC05001151.hg.1	2.08	PSR05016503.hg.1	SEMA5A
TC05001151.hg.1	-2.57	PSR05016509.hg.1	SEMA5A
TC05001151.hg.1	-2.61	JUC05008393.hg.1	SEMA5A
TC05001151.hg.1	-2.74	JUC05008394.hg.1	SEMA5A
TC05001151.hg.1	-2.96	PSR05016513.hg.1	SEMA5A
TC08000844.hg.1	3.87	JUC08005787.hg.1	HEATR7A, LC
TC08000844.hg.1	2.06	JUC08005804.hg.1	HEATR7A, LC
TC08000844.hg.1	2.05	PSR08011179.hg.1	HEATR7A, LC
TC08000844.hg.1	-2.38	JUC08005760.hg.1	HEATR7A, LC
TC08000844.hg.1	-2.74	JUC08005801.hg.1	HEATR7A, LC
TC10001128.hg.1	3.87	JUC10008181.hg.1	ACBD5

TC10001128.hg.1	2.45	PSR10014167.hg.1	ACBD5
TC10001128.hg.1	2.1	PSR10014161.hg.1	ACBD5
TC10001128.hg.1	2.05	PSR10014164.hg.1	ACBD5
TC10001128.hg.1	-2.06	JUC10008180.hg.1	ACBD5
TC10001128.hg.1	-2.71	JUC10008179.hg.1	ACBD5
TC22000259.hg.1	3.87	PSR22004814.hg.1	HMOX1
TC22000259.hg.1	3.35	PSR22004815.hg.1	HMOX1
TC22000259.hg.1	3.24	PSR22004819.hg.1	HMOX1
TC22000259.hg.1	3.17	PSR22004813.hg.1	HMOX1
TC22000259.hg.1	3.02	JUC22001748.hg.1	HMOX1
TC22000259.hg.1	2.32	JUC22001751.hg.1	HMOX1
TC03002155.hg.1	3.86	JUC03018904.hg.1	TFRC
TC03002155.hg.1	3.22	PSR03038060.hg.1	TFRC
TC03002155.hg.1	3.18	JUC03018894.hg.1	TFRC
TC03002155.hg.1	2.65	JUC03018903.hg.1	TFRC
TC03002155.hg.1	2.44	PSR03038082.hg.1	TFRC
TC03002155.hg.1	2.33	PSR03038104.hg.1	TFRC
TC03002155.hg.1	2.01	PSR03038105.hg.1	TFRC
TC03002155.hg.1	-2.34	JUC03018910.hg.1	TFRC
TC03002155.hg.1	-2.43	JUC03018913.hg.1	TFRC
TC05000311.hg.1	3.86	JUC05002275.hg.1	
TC05000311.hg.1	2.62	PSR05004194.hg.1	
TC09000790.hg.1	3.86	JUC09005678.hg.1	OLFM1
TC09000790.hg.1	3.8	PSR09010273.hg.1	OLFM1
TC09000790.hg.1	3.8	PSR09010274.hg.1	OLFM1
TC09000790.hg.1	3.43	PSR09010278.hg.1	OLFM1
TC09000790.hg.1	3.37	PSR09010269.hg.1	OLFM1
TC09000790.hg.1	3.23	PSR09010276.hg.1	OLFM1
TC09000790.hg.1	3.16	PSR09010271.hg.1	OLFM1
TC09000790.hg.1	3.02	PSR09010268.hg.1	OLFM1
TC09000790.hg.1	2.78	PSR09010290.hg.1	OLFM1
TC09000790.hg.1	2.72	PSR09010277.hg.1	OLFM1
TC09000790.hg.1	2.38	PSR09010279.hg.1	OLFM1
TC09000790.hg.1	2.3	PSR09010272.hg.1	OLFM1
TC09000790.hg.1	2.28	JUC09005673.hg.1	OLFM1
TC09000790.hg.1	2.24	JUC09005679.hg.1	OLFM1
TC09000790.hg.1	2.15	JUC09005675.hg.1	OLFM1
TC09000790.hg.1	-2.23	JUC09005676.hg.1	OLFM1
TC09000790.hg.1	-2.43	PSR09010281.hg.1	OLFM1
TC09000790.hg.1	-4.6	JUC09005672.hg.1	OLFM1
TC6_cox_hap2000	3.86	JUC6_cox_hap2000	PSORS1C1
TC6_cox_hap2000	2.52	JUC6_cox_hap2000	PSORS1C1
TC6_cox_hap2000	2.42	JUC6_cox_hap2000	PSORS1C1
TC6_cox_hap2000	2.17	PSR6_cox_hap2000	PSORS1C1
TC03002027.hg.1	3.85	JUC03017795.hg.1	
TC03002027.hg.1	-6.21	PSR03035991.hg.1	
TC09000964.hg.1	3.85	PSR09012770.hg.1	CDKN2A
TC09000964.hg.1	3.63	JUC09006947.hg.1	CDKN2A
TC01000515.hg.1	3.84	JUC01004193.hg.1	NFYC
TC01000515.hg.1	2.58	JUC01004215.hg.1	NFYC
TC01000515.hg.1	-2.13	PSR01008063.hg.1	NFYC
TC01000515.hg.1	-2.66	JUC01004201.hg.1	NFYC
TC01000515.hg.1	-2.7	JUC01004200.hg.1	NFYC
TC01000515.hg.1	-3.13	JUC01004212.hg.1	NFYC
TC01006331.hg.1	3.84	JUC01044116.hg.1	ADAM15
TC01006331.hg.1	3.2	JUC01044128.hg.1	ADAM15
TC01006331.hg.1	2.9	PSR01019847.hg.1	ADAM15
TC01006331.hg.1	2.63	JUC01044138.hg.1	ADAM15

TC01006331.hg.1	2.46	PSR01019857.hg.1	ADAM15
TC01006331.hg.1	2.34	PSR01019837.hg.1	ADAM15
TC01006331.hg.1	2.31	PSR01019810.hg.1	ADAM15
TC01006331.hg.1	2.16	PSR01019856.hg.1	ADAM15
TC01006331.hg.1	2.08	PSR01019852.hg.1	ADAM15
TC01006331.hg.1	2.04	PSR01019851.hg.1	ADAM15
TC01006331.hg.1	2.04	PSR01019853.hg.1	ADAM15
TC01006331.hg.1	2.03	PSR01019808.hg.1	ADAM15
TC02000213.hg.1	3.84	JUC02001567.hg.1	BIRC6
TC02000213.hg.1	2.13	PSR02003127.hg.1	BIRC6
TC02000213.hg.1	-2.44	JUC02001606.hg.1	BIRC6
TC02000213.hg.1	-2.58	JUC02001573.hg.1	BIRC6
TC0Y000354.hg.1	3.84	PSR0Y000815.hg.1	TXLNG2P
TC0Y000354.hg.1	2.89	PSR0Y000816.hg.1	TXLNG2P
TC0Y000354.hg.1	2.78	PSR0Y000812.hg.1	TXLNG2P
TC0Y000354.hg.1	-2.49	PSR0Y000808.hg.1	TXLNG2P
TC0Y000354.hg.1	-2.54	JUC0Y001909.hg.1	TXLNG2P
TC0Y000354.hg.1	-2.62	PSR0Y000818.hg.1	TXLNG2P
TC0Y000354.hg.1	-2.74	PSR0Y000817.hg.1	TXLNG2P
TC0Y000354.hg.1	-2.76	PSR0Y000810.hg.1	TXLNG2P
TC0Y000354.hg.1	-3.5	PSR0Y000819.hg.1	TXLNG2P
TC10000406.hg.1	3.84	JUC10002609.hg.1	TET1
TC10000406.hg.1	2.1	PSR10004528.hg.1	TET1
TC10000406.hg.1	2.02	JUC10002615.hg.1	TET1
TC10000406.hg.1	-2.64	JUC10002614.hg.1	TET1
TC10000406.hg.1	-2.71	JUC10002607.hg.1	TET1
TC10002960.hg.1	3.84	JUC10017491.hg.1	
TC10002960.hg.1	-2.2	PSR10013673.hg.1	
TC11000023.hg.1	3.84	JUC11000191.hg.1	DRD4
TC11000023.hg.1	2.06	PSR11000333.hg.1	DRD4
TC11000023.hg.1	-2.18	JUC11000189.hg.1	DRD4
TC11000023.hg.1	-2.2	JUC11000188.hg.1	DRD4
TC11000023.hg.1	-2.69	PSR11000332.hg.1	DRD4
TC12001361.hg.1	3.84	JUC12009892.hg.1	FAM60A
TC12001361.hg.1	2.23	JUC12009888.hg.1	FAM60A
TC12001361.hg.1	-2.06	PSR12017806.hg.1	FAM60A
TC12001361.hg.1	-3.13	PSR12017793.hg.1	FAM60A
TC18000006.hg.1	3.84	JUC18000042.hg.1	TYMS
TC18000006.hg.1	3.69	PSR18000068.hg.1	TYMS
TC18000006.hg.1	3.06	PSR18000052.hg.1	TYMS
TC18000006.hg.1	2.29	JUC18000036.hg.1	TYMS
TC18000006.hg.1	2.24	PSR18000056.hg.1	TYMS
TC18000006.hg.1	2.17	PSR18000055.hg.1	TYMS
TC18000006.hg.1	2.08	JUC18000044.hg.1	TYMS
TC18000006.hg.1	-2.14	JUC18000038.hg.1	TYMS
TC18000006.hg.1	-2.15	JUC18000035.hg.1	TYMS
TC19000462.hg.1	3.84	JUC19003600.hg.1	LSR
TC19000462.hg.1	-2.14	PSR19006074.hg.1	LSR
TC20000657.hg.1	3.84	JUC20004823.hg.1	RRBP1
TC20000657.hg.1	2.21	PSR20009201.hg.1	RRBP1
TC20000657.hg.1	-2.39	JUC20004806.hg.1	RRBP1
TC01001741.hg.1	3.83	JUC01014699.hg.1	CD46
TC01001741.hg.1	2.29	JUC01014704.hg.1	CD46
TC01001741.hg.1	-2.31	JUC01014685.hg.1	CD46
TC01001741.hg.1	-2.33	PSR01027294.hg.1	CD46
TC01001741.hg.1	-2.82	PSR01027301.hg.1	CD46
TC01002520.hg.1	3.83	JUC01021044.hg.1	RSPO1
TC01002520.hg.1	-2.54	PSR01039171.hg.1	RSPO1

TC12000392.hg.1	3.83	JUC12002694.hg.1	LARP4
TC12000392.hg.1	2.57	PSR12004897.hg.1	LARP4
TC12000392.hg.1	2.33	PSR12004896.hg.1	LARP4
TC12000392.hg.1	2.13	JUC12002702.hg.1	LARP4
TC12000392.hg.1	2.11	JUC12002708.hg.1	LARP4
TC19000493.hg.1	3.83	JUC19003885.hg.1	WDR62
TC19000493.hg.1	-2.06	PSR19006553.hg.1	WDR62
TC19000493.hg.1	-2.82	JUC19003854.hg.1	WDR62
TC01001328.hg.1	3.82	JUC01011089.hg.1	NTRK1
TC01001328.hg.1	3.48	JUC01011097.hg.1	NTRK1
TC01001328.hg.1	2.13	PSR01020860.hg.1	NTRK1
TC02000758.hg.1	3.82	JUC02006324.hg.1	EPB41L5
TC02000758.hg.1	3.21	JUC02006310.hg.1	EPB41L5
TC02000758.hg.1	3	PSR02011884.hg.1	EPB41L5
TC02000758.hg.1	2.76	JUC02006307.hg.1	EPB41L5
TC02000758.hg.1	2.28	JUC02006300.hg.1	EPB41L5
TC02000758.hg.1	2.18	PSR02011881.hg.1	EPB41L5
TC02000758.hg.1	2.12	PSR02011913.hg.1	EPB41L5
TC02000758.hg.1	2.11	PSR02011906.hg.1	EPB41L5
TC02000758.hg.1	2.1	PSR02011910.hg.1	EPB41L5
TC02000758.hg.1	2.01	PSR02011892.hg.1	EPB41L5
TC02000758.hg.1	-2.2	JUC02006303.hg.1	EPB41L5
TC02000758.hg.1	-2.73	JUC02006302.hg.1	EPB41L5
TC02002229.hg.1	3.82	JUC02018722.hg.1	SLC35F5
TC02002229.hg.1	-2.42	PSR02036191.hg.1	SLC35F5
TC04001486.hg.1	3.82	PSR04021054.hg.1	CAMK2D
TC04001486.hg.1	-2.18	PSR04021040.hg.1	CAMK2D
TC04001486.hg.1	-2.3	JUC04011063.hg.1	CAMK2D
TC04001486.hg.1	-3.27	JUC04011054.hg.1	CAMK2D
TC05002151.hg.1	3.82	JUC05015396.hg.1	MGAT1
TC05002151.hg.1	3.07	JUC05015402.hg.1	MGAT1
TC05002151.hg.1	2.31	PSR05030325.hg.1	MGAT1
TC06000357.hg.1	3.82	JUC06001442.hg.1	PSORS1C1
TC06000357.hg.1	2.49	JUC06001451.hg.1	PSORS1C1
TC06000357.hg.1	2.39	JUC06001444.hg.1	PSORS1C1
TC06000357.hg.1	2.15	PSR06003414.hg.1	PSORS1C1
TC19001505.hg.1	3.82	JUC19011791.hg.1	LGALS4
TC19001505.hg.1	-4.16	PSR19020448.hg.1	LGALS4
TC22000437.hg.1	3.82	JUC22003262.hg.1	RPL23AP82,
TC22000437.hg.1	2.86	JUC22003257.hg.1	RPL23AP82,
TC22000437.hg.1	2.02	JUC22003258.hg.1	RPL23AP82,
TC22000437.hg.1	-2.2	PSR22008262.hg.1	RPL23AP82,
TC22000437.hg.1	-2.32	PSR22008266.hg.1	RPL23AP82,
TC6_dbb_hap3000	3.82	JUC6_dbb_hap3000	PSORS1C1
TC6_dbb_hap3000	2.49	JUC6_dbb_hap3000	PSORS1C1
TC6_dbb_hap3000	2.39	JUC6_dbb_hap3000	PSORS1C1
TC6_dbb_hap3000	2.15	PSR6_dbb_hap3000	PSORS1C1
TC04000881.hg.1	3.81	JUC04006475.hg.1	ODZ3
TC04000881.hg.1	3.27	JUC04006470.hg.1	ODZ3
TC04000881.hg.1	2.31	PSR04012031.hg.1	ODZ3
TC04000881.hg.1	2.14	PSR04012033.hg.1	ODZ3
TC05000782.hg.1	3.81	JUC05005797.hg.1	ARHGAP26
TC05000782.hg.1	2.46	PSR05011154.hg.1	ARHGAP26
TC05000782.hg.1	2.46	JUC05005763.hg.1	ARHGAP26
TC05000782.hg.1	2.09	JUC05005766.hg.1	ARHGAP26
TC05000782.hg.1	-2.38	JUC05005764.hg.1	ARHGAP26
TC11001377.hg.1	3.81	PSR11017328.hg.1	LOC283299
TC11001377.hg.1	-2.13	PSR11017327.hg.1	LOC283299

TC11001377.hg.1	-2.95	JUC11009228.hg.1	LOC283299
TC05000682.hg.1	3.8	PSR05009481.hg.1	TGFBI, LOC1
TC05000682.hg.1	3.44	JUC05005063.hg.1	TGFBI, LOC1
TC05000682.hg.1	2.11	PSR05009482.hg.1	TGFBI, LOC1
TC05000682.hg.1	2.11	JUC05005064.hg.1	TGFBI, LOC1
TC05000682.hg.1	2.07	JUC05005055.hg.1	TGFBI, LOC1
TC05000682.hg.1	-2.42	JUC05005044.hg.1	TGFBI, LOC1
TC05003423.hg.1	3.8	JUC05019564.hg.1	C1QTNF3
TC05003423.hg.1	3.11	JUC05019563.hg.1	C1QTNF3
TC05003423.hg.1	2.55	PSR05017533.hg.1	C1QTNF3
TC05003423.hg.1	2.47	JUC05019568.hg.1	C1QTNF3
TC05003423.hg.1	-2.48	PSR05017544.hg.1	C1QTNF3
TC05003423.hg.1	-3.7	PSR05017540.hg.1	C1QTNF3
TC05003423.hg.1	-6.5	PSR05017536.hg.1	C1QTNF3
TC05003423.hg.1	-6.54	PSR05017532.hg.1	C1QTNF3
TC05003423.hg.1	-7.12	PSR05017523.hg.1	C1QTNF3
TC05003423.hg.1	-7.8	PSR05017537.hg.1	C1QTNF3
TC05003423.hg.1	-7.92	PSR05017547.hg.1	C1QTNF3
TC05003423.hg.1	-10.11	PSR05017521.hg.1	C1QTNF3
TC05003423.hg.1	-12.39	PSR05017535.hg.1	C1QTNF3
TC05003423.hg.1	-13.01	PSR05017551.hg.1	C1QTNF3
TC05003423.hg.1	-13.38	PSR05017550.hg.1	C1QTNF3
TC18000413.hg.1	3.8	PSR18004218.hg.1	NPC1
TC18000413.hg.1	-2	JUC18002819.hg.1	NPC1
TC18000552.hg.1	3.8	JUC18003872.hg.1	PIGN
TC18000552.hg.1	2.64	PSR18005756.hg.1	PIGN
TC18000552.hg.1	2.44	JUC18003858.hg.1	PIGN
TC18000552.hg.1	-2.48	PSR18005731.hg.1	PIGN
TC18000552.hg.1	-2.55	JUC18003869.hg.1	PIGN
TC21000543.hg.1	3.8	PSR21006542.hg.1	POFUT2
TC21000543.hg.1	3.28	PSR21006526.hg.1	POFUT2
TC21000543.hg.1	2.28	PSR21006532.hg.1	POFUT2
TC21000543.hg.1	2.26	PSR21006538.hg.1	POFUT2
TC21000543.hg.1	2.26	JUC21003353.hg.1	POFUT2
TC21000543.hg.1	2.25	PSR21006529.hg.1	POFUT2
TC21000543.hg.1	2.21	PSR21006544.hg.1	POFUT2
TC21000543.hg.1	2.1	PSR21006541.hg.1	POFUT2
TC21000543.hg.1	2.04	PSR21006531.hg.1	POFUT2
TC04000524.hg.1	3.79	PSR04007728.hg.1	BANK1
TC04000524.hg.1	2.73	PSR04007731.hg.1	BANK1
TC04000524.hg.1	2.68	JUC04004022.hg.1	BANK1
TC04000524.hg.1	-2.59	JUC04004018.hg.1	BANK1
TC06001184.hg.1	3.79	JUC06006974.hg.1	MLLT4
TC06001184.hg.1	3.49	JUC06006990.hg.1	MLLT4
TC06001184.hg.1	2.48	PSR06014165.hg.1	MLLT4
TC06001184.hg.1	2.39	JUC06006978.hg.1	MLLT4
TC06001184.hg.1	2.29	JUC06007015.hg.1	MLLT4
TC06001184.hg.1	2.28	JUC06007008.hg.1	MLLT4
TC07001811.hg.1	3.79	JUC07013802.hg.1	AASS
TC07001811.hg.1	2.97	JUC07013776.hg.1	AASS
TC07001811.hg.1	2.44	JUC07013783.hg.1	AASS
TC07001811.hg.1	2.33	PSR07028026.hg.1	AASS
TC11002380.hg.1	3.79	JUC11015299.hg.1	MIR100HG
TC11002380.hg.1	2.76	JUC11015311.hg.1	MIR100HG
TC11002380.hg.1	2.54	PSR11028663.hg.1	MIR100HG
TC11002380.hg.1	2.08	PSR11028662.hg.1	MIR100HG
TC11002380.hg.1	2.06	PSR11028665.hg.1	MIR100HG
TC11002380.hg.1	-2.11	PSR11028678.hg.1	MIR100HG

TC11002380.hg.1	-2.57	PSR11028677.hg.1	MIR100HG
TC11002380.hg.1	-2.87	JUC11015301.hg.1	MIR100HG
TC11002380.hg.1	-2.95	JUC11015308.hg.1	MIR100HG
TC11002380.hg.1	-3.15	JUC11015309.hg.1	MIR100HG
TC11002380.hg.1	-3.4	JUC11015304.hg.1	MIR100HG
TC11002380.hg.1	-4.3	PSR11028676.hg.1	MIR100HG
TC11002380.hg.1	-5.02	PSR11028674.hg.1	MIR100HG
TC13000566.hg.1	3.79	JUC13003762.hg.1	SPG20
TC13000566.hg.1	-2.1	PSR13006420.hg.1	SPG20
TC13000566.hg.1	-2.29	PSR13006436.hg.1	SPG20
TC13000895.hg.1	3.79	JUC13005898.hg.1	DCUN1D2
TC13000895.hg.1	2.95	JUC13005897.hg.1	DCUN1D2
TC13000895.hg.1	2.64	JUC13005904.hg.1	DCUN1D2
TC13000895.hg.1	2.59	PSR13010039.hg.1	DCUN1D2
TC14000668.hg.1	3.79	PSR14008055.hg.1	SNORD113-4
TC14000668.hg.1	2.68	PSR14008052.hg.1	SNORD113-4
TC14000668.hg.1	2.56	PSR14008057.hg.1	SNORD113-4
TC14000668.hg.1	2.47	JUC14004113.hg.1	SNORD113-4
TC14000668.hg.1	2.3	JUC14004098.hg.1	SNORD113-4
TC14000668.hg.1	-2.11	JUC14004108.hg.1	SNORD113-4
TC14000668.hg.1	-2.39	JUC14004109.hg.1	SNORD113-4
TC14000668.hg.1	-3.39	JUC14004097.hg.1	SNORD113-4
TC14000668.hg.1	-3.76	JUC14004118.hg.1	SNORD113-4
TC15000350.hg.1	3.79	PSR15003144.hg.1	SORD
TC15000350.hg.1	2.13	JUC15001473.hg.1	SORD
TC15000350.hg.1	-2.12	JUC15001465.hg.1	SORD
TC15000350.hg.1	-2.67	JUC15001474.hg.1	SORD
TC01000561.hg.1	3.78	PSR01009260.hg.1	RNF220
TC01000561.hg.1	3.17	PSR01009264.hg.1	RNF220
TC01000561.hg.1	3.15	JUC01004795.hg.1	RNF220
TC01000561.hg.1	3.1	PSR01009266.hg.1	RNF220
TC01000561.hg.1	2.3	PSR01009258.hg.1	RNF220
TC01000561.hg.1	-2.97	JUC01004808.hg.1	RNF220
TC05000010.hg.1	3.78	JUC05000144.hg.1	TRIP13
TC05000010.hg.1	2.83	PSR05000277.hg.1	TRIP13
TC05000010.hg.1	2.7	PSR05000251.hg.1	TRIP13
TC05000010.hg.1	2.47	JUC05000145.hg.1	TRIP13
TC05000010.hg.1	2.37	PSR05000259.hg.1	TRIP13
TC05000010.hg.1	2.28	JUC05000141.hg.1	TRIP13
TC05000010.hg.1	2.13	JUC05000134.hg.1	TRIP13
TC05000010.hg.1	2.08	PSR05000275.hg.1	TRIP13
TC05000010.hg.1	2.01	PSR05000252.hg.1	TRIP13
TC05000010.hg.1	-2.07	JUC05000146.hg.1	TRIP13
TC05000391.hg.1	3.78	JUC05002984.hg.1	PAPD4
TC05000391.hg.1	3.42	PSR05005489.hg.1	PAPD4
TC05000391.hg.1	2.63	JUC05002993.hg.1	PAPD4
TC05000391.hg.1	2.36	JUC05002983.hg.1	PAPD4
TC05000391.hg.1	-4.02	JUC05002973.hg.1	PAPD4
TC08000293.hg.1	3.78	PSR08004085.hg.1	TACC1
TC08000293.hg.1	2.65	JUC08002023.hg.1	TACC1
TC08000293.hg.1	-2.09	PSR08004135.hg.1	TACC1
TC08000293.hg.1	-3.09	JUC08002025.hg.1	TACC1
TC10001411.hg.1	3.78	JUC10010416.hg.1	USP54
TC10001411.hg.1	3.63	JUC10010413.hg.1	USP54
TC10001411.hg.1	2.63	PSR10017839.hg.1	USP54
TC10001411.hg.1	2.31	PSR10017828.hg.1	USP54
TC10001411.hg.1	2.29	JUC10010411.hg.1	USP54
TC10001411.hg.1	2.22	JUC10010388.hg.1	USP54

TC10001411.hg.1	2.08	PSR10017857.hg.1	USP54
TC10001411.hg.1	2.08	PSR10017881.hg.1	USP54
TC10001411.hg.1	2.01	PSR10017880.hg.1	USP54
TC10001411.hg.1	-2.01	JUC10010403.hg.1	USP54
TC10001411.hg.1	-2.06	JUC10010414.hg.1	USP54
TC10001411.hg.1	-2.13	PSR10017871.hg.1	USP54
TC10001411.hg.1	-2.33	PSR10017849.hg.1	USP54
TC10001411.hg.1	-3.93	JUC10010417.hg.1	USP54
TC14000524.hg.1	3.78	PSR14006522.hg.1	LOC283587
TC14000524.hg.1	2.72	JUC14003286.hg.1	LOC283587
TC14000524.hg.1	2.49	PSR14006512.hg.1	LOC283587
TC14000524.hg.1	2.06	PSR14006515.hg.1	LOC283587
TC15000881.hg.1	3.78	JUC15004528.hg.1	SLCO3A1
TC15000881.hg.1	2.51	PSR15008488.hg.1	SLCO3A1
TC15000881.hg.1	2.12	PSR15008477.hg.1	SLCO3A1
TC02001977.hg.1	3.77	JUC02016429.hg.1	EXOC6B
TC02001977.hg.1	2.27	PSR02031500.hg.1	EXOC6B
TC02001977.hg.1	2.05	PSR02031479.hg.1	EXOC6B
TC03001772.hg.1	3.77	JUC03015982.hg.1	PLXND1
TC03001772.hg.1	3.35	PSR03032127.hg.1	PLXND1
TC03001772.hg.1	3.35	JUC03015963.hg.1	PLXND1
TC03001772.hg.1	3.24	JUC03015970.hg.1	PLXND1
TC03001772.hg.1	2.98	JUC03015978.hg.1	PLXND1
TC03001772.hg.1	2.96	PSR03032151.hg.1	PLXND1
TC03001772.hg.1	2.9	JUC03015959.hg.1	PLXND1
TC03001772.hg.1	2.62	PSR03032148.hg.1	PLXND1
TC03001772.hg.1	2.57	PSR03032159.hg.1	PLXND1
TC03001772.hg.1	2.5	JUC03015985.hg.1	PLXND1
TC03001772.hg.1	2.44	JUC03015975.hg.1	PLXND1
TC03001772.hg.1	2.36	PSR03032129.hg.1	PLXND1
TC03001772.hg.1	2.34	PSR03032140.hg.1	PLXND1
TC03001772.hg.1	2.32	PSR03032166.hg.1	PLXND1
TC03001772.hg.1	2.31	PSR03032167.hg.1	PLXND1
TC03001772.hg.1	2.26	PSR03032158.hg.1	PLXND1
TC03001772.hg.1	2.23	PSR03032142.hg.1	PLXND1
TC03001772.hg.1	2.19	PSR03032156.hg.1	PLXND1
TC03001772.hg.1	2.18	PSR03032131.hg.1	PLXND1
TC03001772.hg.1	2.18	JUC03015960.hg.1	PLXND1
TC03001772.hg.1	2.17	JUC03015972.hg.1	PLXND1
TC03001772.hg.1	2.13	PSR03032147.hg.1	PLXND1
TC03001772.hg.1	2.12	PSR03032154.hg.1	PLXND1
TC03001772.hg.1	2.1	PSR03032145.hg.1	PLXND1
TC03001772.hg.1	2.08	PSR03032128.hg.1	PLXND1
TC03001772.hg.1	2.08	JUC03015966.hg.1	PLXND1
TC03001772.hg.1	2.07	JUC03015990.hg.1	PLXND1
TC03001772.hg.1	2.06	JUC03015973.hg.1	PLXND1
TC03001772.hg.1	2.05	PSR03032134.hg.1	PLXND1
TC03001772.hg.1	2.05	PSR03032165.hg.1	PLXND1
TC03001772.hg.1	2.04	JUC03015981.hg.1	PLXND1
TC03001772.hg.1	2.04	JUC03015996.hg.1	PLXND1
TC03001772.hg.1	2.03	PSR03032155.hg.1	PLXND1
TC03001772.hg.1	2.03	PSR03032171.hg.1	PLXND1
TC03001772.hg.1	2.01	JUC03015964.hg.1	PLXND1
TC05000135.hg.1	3.77	JUC05000777.hg.1	PDZD2
TC05000135.hg.1	2.8	JUC05000773.hg.1	PDZD2
TC05000135.hg.1	2.65	JUC05000766.hg.1	PDZD2
TC05000135.hg.1	2.13	PSR05001368.hg.1	PDZD2
TC05000135.hg.1	2.11	PSR05001371.hg.1	PDZD2

TC05000135.hg.1	-2.07	JUC05000745.hg.1	PDZD2
TC05000135.hg.1	-2.34	JUC05000764.hg.1	PDZD2
TC05000135.hg.1	-2.51	PSR05001325.hg.1	PDZD2
TC05000135.hg.1	-2.7	JUC05000770.hg.1	PDZD2
TC05000135.hg.1	-2.72	JUC05000758.hg.1	PDZD2
TC05000135.hg.1	-7.59	JUC05000782.hg.1	PDZD2
TC06002122.hg.1	3.77	JUC06013209.hg.1	SLC18B1
TC06002122.hg.1	-2.1	PSR06026633.hg.1	SLC18B1
TC11001387.hg.1	3.77	JUC11009289.hg.1	TRIM66
TC11001387.hg.1	2.99	JUC11009311.hg.1	TRIM66
TC11001387.hg.1	2.08	PSR11017465.hg.1	TRIM66
TC11001387.hg.1	-2.97	JUC11009297.hg.1	TRIM66
TC11001444.hg.1	3.77	PSR11018099.hg.1	CYP2R1
TC11001444.hg.1	2.41	PSR11018116.hg.1	CYP2R1
TC11001444.hg.1	2.05	JUC11009661.hg.1	CYP2R1
TC11001444.hg.1	-3.05	JUC11009664.hg.1	CYP2R1
TC16000338.hg.1	3.77	PSR16004840.hg.1	ALDOA
TC16000338.hg.1	3.7	JUC16002686.hg.1	ALDOA
TC16000338.hg.1	3.25	PSR16004848.hg.1	ALDOA
TC16000338.hg.1	3.14	JUC16002685.hg.1	ALDOA
TC16000338.hg.1	2.6	PSR16004844.hg.1	ALDOA
TC16000338.hg.1	2.37	JUC16002699.hg.1	ALDOA
TC16000338.hg.1	2.29	PSR16004856.hg.1	ALDOA
TC16000338.hg.1	2.23	JUC16002680.hg.1	ALDOA
TC16000338.hg.1	2.21	JUC16002696.hg.1	ALDOA
TC16000338.hg.1	2.08	PSR16004852.hg.1	ALDOA
TC16000338.hg.1	2.02	PSR16004858.hg.1	ALDOA
TC16000461.hg.1	3.77	JUC16003605.hg.1	GNAO1
TC16000461.hg.1	3.32	JUC16003612.hg.1	GNAO1
TC16000461.hg.1	2.74	PSR16006423.hg.1	GNAO1
TC16000461.hg.1	2	PSR16006408.hg.1	GNAO1
TC16000461.hg.1	-2.12	JUC16003615.hg.1	GNAO1
TC16000461.hg.1	-2.17	JUC16003609.hg.1	GNAO1
TC01002697.hg.1	3.76	PSR01042290.hg.1	USP24
TC01002697.hg.1	2.89	JUC01022634.hg.1	USP24
TC01002697.hg.1	-2.13	JUC01022630.hg.1	USP24
TC01002697.hg.1	-2.4	JUC01022612.hg.1	USP24
TC01002697.hg.1	-3.07	JUC01022624.hg.1	USP24
TC07001007.hg.1	3.76	JUC07007429.hg.1	TMEM176A
TC07001007.hg.1	2.79	JUC07007427.hg.1	TMEM176A
TC07001007.hg.1	2.02	PSR07015346.hg.1	TMEM176A
TC08001701.hg.1	3.76	PSR08022402.hg.1	LOC1002881
TC08001701.hg.1	-3.14	JUC08011517.hg.1	LOC1002881
TC09001100.hg.1	3.76	JUC09007871.hg.1	ZNF658, LOC
TC09001100.hg.1	2.01	PSR09014663.hg.1	ZNF658, LOC
TC09001100.hg.1	-2.57	PSR09014661.hg.1	ZNF658, LOC
TC11000256.hg.1	3.76	JUC11001714.hg.1	HTATIP2
TC11000256.hg.1	-2.15	PSR11003405.hg.1	HTATIP2
TC11000256.hg.1	-2.57	PSR11003403.hg.1	HTATIP2
TC17000480.hg.1	3.76	JUC17003229.hg.1	ERBB2, MIR4
TC17000480.hg.1	-2.16	PSR17005664.hg.1	ERBB2, MIR4
TC17000480.hg.1	-3.21	JUC17003212.hg.1	ERBB2, MIR4
TC17001376.hg.1	3.76	JUC17010288.hg.1	MMP28
TC17001376.hg.1	2.58	PSR17018248.hg.1	MMP28
TC17001376.hg.1	2.16	JUC17010289.hg.1	MMP28
TC17001376.hg.1	-2.06	JUC17010294.hg.1	MMP28
TC17001376.hg.1	-2.07	PSR17018231.hg.1	MMP28
TC19000787.hg.1	3.76	JUC19006490.hg.1	LINC00085

TC19000787.hg.1	-2.15	PSR19011044.hg.1	LINC00085
TC6_mann_hap40(	3.76	JUC6_mann_hap400	PSORS1C1
TC6_mann_hap40(	2.45	JUC6_mann_hap400	PSORS1C1
TC6_mann_hap40(	2.36	JUC6_mann_hap400	PSORS1C1
TC6_mann_hap40(	2.11	PSR6_mann_hap40(	PSORS1C1
TC6_mcf_hap5000	3.76	JUC6_mcf_hap5000	PSORS1C1
TC6_mcf_hap5000	2.45	JUC6_mcf_hap5000	PSORS1C1
TC6_mcf_hap5000	2.36	JUC6_mcf_hap5000	PSORS1C1
TC6_mcf_hap5000	2.11	PSR6_mcf_hap5000	PSORS1C1
TC01000454.hg.1	3.75	JUC01003614.hg.1	ZMYM1
TC01000454.hg.1	3.55	PSR01006977.hg.1	ZMYM1
TC01000454.hg.1	2.18	JUC01003620.hg.1	ZMYM1
TC01000454.hg.1	2.11	PSR01006991.hg.1	ZMYM1
TC01000454.hg.1	2	JUC01003624.hg.1	ZMYM1
TC06000325.hg.1	3.75	PSR06002601.hg.1	LOC554223,
TC06000325.hg.1	3.05	PSR06002600.hg.1	LOC554223,
TC06000325.hg.1	2.57	JUC06001178.hg.1	LOC554223,
TC06000325.hg.1	2.27	JUC06001177.hg.1	LOC554223,
TC06000325.hg.1	2.23	PSR06002596.hg.1	LOC554223,
TC06001492.hg.1	3.75	PSR06016764.hg.1	ZNRD1-AS1
TC06001492.hg.1	3.2	PSR06016740.hg.1	ZNRD1-AS1
TC06001492.hg.1	3.11	PSR06016748.hg.1	ZNRD1-AS1
TC06001492.hg.1	2.99	PSR06016753.hg.1	ZNRD1-AS1
TC06001492.hg.1	2.84	PSR06016733.hg.1	ZNRD1-AS1
TC06001492.hg.1	2.75	PSR06016754.hg.1	ZNRD1-AS1
TC06001492.hg.1	2.71	PSR06016745.hg.1	ZNRD1-AS1
TC06001492.hg.1	2.57	JUC06008251.hg.1	ZNRD1-AS1
TC06001492.hg.1	2.56	PSR06016732.hg.1	ZNRD1-AS1
TC06001492.hg.1	2.51	JUC06008266.hg.1	ZNRD1-AS1
TC06001492.hg.1	2.5	PSR06016758.hg.1	ZNRD1-AS1
TC06001492.hg.1	2.49	PSR06016749.hg.1	ZNRD1-AS1
TC06001492.hg.1	2.36	PSR06016750.hg.1	ZNRD1-AS1
TC06001492.hg.1	2.29	PSR06016755.hg.1	ZNRD1-AS1
TC06001492.hg.1	2.08	PSR06016739.hg.1	ZNRD1-AS1
TC06001492.hg.1	-2.62	JUC06008264.hg.1	ZNRD1-AS1
TC09000691.hg.1	3.75	PSR09008371.hg.1	ODF2
TC09000691.hg.1	3.41	JUC09004634.hg.1	ODF2
TC09000691.hg.1	3.28	JUC09004664.hg.1	ODF2
TC09000691.hg.1	2.58	JUC09004655.hg.1	ODF2
TC09000691.hg.1	2.33	JUC09004669.hg.1	ODF2
TC09000691.hg.1	2.18	PSR09008402.hg.1	ODF2
TC09000691.hg.1	2.02	PSR09008373.hg.1	ODF2
TC09000691.hg.1	2.02	JUC09004657.hg.1	ODF2
TC09000691.hg.1	-3.04	JUC09004647.hg.1	ODF2
TC11000790.hg.1	3.75	JUC11005301.hg.1	POLD3
TC11000790.hg.1	3.42	JUC11005306.hg.1	POLD3
TC11000790.hg.1	2.65	PSR11010163.hg.1	POLD3
TC12000549.hg.1	3.75	JUC12003827.hg.1	LOC1005068
TC12000549.hg.1	2.12	PSR12007382.hg.1	LOC1005068
TC12001817.hg.1	3.75	PSR12023994.hg.1	SOCS2-AS1
TC12001817.hg.1	3.48	PSR12023995.hg.1	SOCS2-AS1
TC12001817.hg.1	3.03	JUC12013311.hg.1	SOCS2-AS1
TC12001817.hg.1	2.67	JUC12013309.hg.1	SOCS2-AS1
TC12001817.hg.1	2.34	PSR12023992.hg.1	SOCS2-AS1
TC20001039.hg.1	3.75	JUC20007460.hg.1	ZBTB46
TC20001039.hg.1	-3.01	PSR20014248.hg.1	ZBTB46
TC01003456.hg.1	3.74	JUC01028061.hg.1	C1orf110
TC01003456.hg.1	2.53	PSR01053844.hg.1	C1orf110

TC01003456.hg.1	2.34	PSR01053843.hg.1	C1orf110
TC04000962.hg.1	3.74	PSR04013051.hg.1	SPON2, LOC
TC04000962.hg.1	2.96	JUC04006970.hg.1	SPON2, LOC
TC04000962.hg.1	2.95	PSR04013046.hg.1	SPON2, LOC
TC04000962.hg.1	2.83	JUC04006974.hg.1	SPON2, LOC
TC04000962.hg.1	2.25	JUC04006989.hg.1	SPON2, LOC
TC04000962.hg.1	-2.1	PSR04013096.hg.1	SPON2, LOC
TC04000962.hg.1	-2.59	JUC04006968.hg.1	SPON2, LOC
TC04000962.hg.1	-2.68	JUC04006980.hg.1	SPON2, LOC
TC04000962.hg.1	-3.07	JUC04006987.hg.1	SPON2, LOC
TC04000962.hg.1	-4.59	JUC04006979.hg.1	SPON2, LOC
TC08000002.hg.1	3.74	JUC08000005.hg.1	ZNF596
TC08000002.hg.1	3.18	JUC08000010.hg.1	ZNF596
TC08000002.hg.1	2.69	PSR08000009.hg.1	ZNF596
TC08000002.hg.1	2.4	PSR08000007.hg.1	ZNF596
TC08000002.hg.1	2.38	PSR08000010.hg.1	ZNF596
TC08000002.hg.1	-2.01	PSR08000037.hg.1	ZNF596
TC14000208.hg.1	3.74	JUC14001021.hg.1	AKAP6
TC14000208.hg.1	-2.57	PSR14002277.hg.1	AKAP6
TC14000208.hg.1	-3.28	JUC14001020.hg.1	AKAP6
TC16000750.hg.1	3.74	JUC16006113.hg.1	UNKL
TC16000750.hg.1	2.86	JUC16006114.hg.1	UNKL
TC16000750.hg.1	2.28	PSR16010927.hg.1	UNKL
TC16000750.hg.1	2.07	JUC16006106.hg.1	UNKL
TC16000750.hg.1	-2.06	JUC16006102.hg.1	UNKL
TC16000750.hg.1	-2.15	JUC16006111.hg.1	UNKL
TC16000750.hg.1	-4.35	JUC16006099.hg.1	UNKL
TC17000207.hg.1	3.74	JUC17001688.hg.1	ALKBH5, LO(
TC17000207.hg.1	3.33	PSR17002976.hg.1	ALKBH5, LO(
TC17000207.hg.1	2.85	JUC17001691.hg.1	ALKBH5, LO(
TC17000207.hg.1	2.58	PSR17002973.hg.1	ALKBH5, LO(
TC17000207.hg.1	2.22	PSR17002975.hg.1	ALKBH5, LO(
TC17000207.hg.1	2.07	PSR17002974.hg.1	ALKBH5, LO(
TC20000566.hg.1	3.74	PSR20008229.hg.1	PCED1A
TC20000566.hg.1	-2.73	JUC20004203.hg.1	PCED1A
TC05000761.hg.1	3.73	JUC05005680.hg.1	PCDHGC4, P
TC05000761.hg.1	3.17	JUC05005666.hg.1	PCDHGC4, P
TC05000761.hg.1	2.97	JUC05005676.hg.1	PCDHGC4, P
TC05000761.hg.1	2.86	JUC05005672.hg.1	PCDHGC4, P
TC05000761.hg.1	2.58	JUC05005668.hg.1	PCDHGC4, P
TC05000761.hg.1	2.55	JUC05005667.hg.1	PCDHGC4, P
TC05000761.hg.1	2.5	JUC05005681.hg.1	PCDHGC4, P
TC05000761.hg.1	2.45	JUC05005656.hg.1	PCDHGC4, P
TC05000761.hg.1	2.35	JUC05005655.hg.1	PCDHGC4, P
TC05000761.hg.1	2.32	JUC05005674.hg.1	PCDHGC4, P
TC05000761.hg.1	2.31	JUC05005678.hg.1	PCDHGC4, P
TC05000761.hg.1	2.29	PSR05010868.hg.1	PCDHGC4, P
TC05000761.hg.1	2.2	JUC05005677.hg.1	PCDHGC4, P
TC05000761.hg.1	2.18	PSR05010862.hg.1	PCDHGC4, P
TC05000761.hg.1	2.12	JUC05005657.hg.1	PCDHGC4, P
TC05000761.hg.1	2.12	JUC05005662.hg.1	PCDHGC4, P
TC05000761.hg.1	2.08	JUC05005675.hg.1	PCDHGC4, P
TC08000645.hg.1	3.73	JUC08004463.hg.1	FZD6
TC08000645.hg.1	2.32	PSR08008772.hg.1	FZD6
TC08001332.hg.1	3.73	JUC08008843.hg.1	TCEB1
TC08001332.hg.1	2.67	PSR08017216.hg.1	TCEB1
TC16000503.hg.1	3.73	JUC16004031.hg.1	NDRG4
TC16000503.hg.1	3.33	JUC16004027.hg.1	NDRG4

TC16000503.hg.1	2.84	JUC16004022.hg.1	NDRG4
TC16000503.hg.1	2.74	PSR16007050.hg.1	NDRG4
TC16000503.hg.1	2.69	PSR16007055.hg.1	NDRG4
TC16000503.hg.1	2.68	JUC16004032.hg.1	NDRG4
TC16000503.hg.1	2.4	JUC16004042.hg.1	NDRG4
TC16000503.hg.1	2.34	PSR16007061.hg.1	NDRG4
TC16000503.hg.1	2.29	JUC16004024.hg.1	NDRG4
TC16000503.hg.1	2.18	PSR16007077.hg.1	NDRG4
TC16000503.hg.1	2.13	PSR16007062.hg.1	NDRG4
TC16000503.hg.1	2.1	PSR16007058.hg.1	NDRG4
TC16000503.hg.1	2.1	PSR16007066.hg.1	NDRG4
TC16000503.hg.1	2.1	JUC16004035.hg.1	NDRG4
TC16000503.hg.1	-2.04	JUC16004036.hg.1	NDRG4
TC16000503.hg.1	-2.27	JUC16004018.hg.1	NDRG4
TC19001438.hg.1	3.73	JUC19011239.hg.1	ZNF792
TC19001438.hg.1	2.51	PSR19019345.hg.1	ZNF792
TC19001700.hg.1	3.73	JUC19013246.hg.1	PLEKHA4
TC19001700.hg.1	2.06	PSR19023165.hg.1	PLEKHA4
TC19001700.hg.1	-2.04	JUC19013231.hg.1	PLEKHA4
TC19001700.hg.1	-3.32	JUC19013244.hg.1	PLEKHA4
TC01002733.hg.1	3.72	JUC01022935.hg.1	ITGB3BP
TC01002733.hg.1	2.21	PSR01042902.hg.1	ITGB3BP
TC01002733.hg.1	2.16	JUC01022920.hg.1	ITGB3BP
TC01002733.hg.1	-3.86	JUC01022917.hg.1	ITGB3BP
TC01002733.hg.1	-4.22	JUC01022936.hg.1	ITGB3BP
TC01002733.hg.1	-5.29	JUC01022915.hg.1	ITGB3BP
TC08000160.hg.1	3.72	JUC08000893.hg.1	XPO7
TC08000160.hg.1	2.79	PSR08001629.hg.1	XPO7
TC08000160.hg.1	2.09	PSR08001651.hg.1	XPO7
TC11000963.hg.1	3.72	JUC11006427.hg.1	DYNC2H1
TC11000963.hg.1	3.2	JUC11006367.hg.1	DYNC2H1
TC11000963.hg.1	2.49	JUC11006420.hg.1	DYNC2H1
TC11000963.hg.1	2.38	JUC11006398.hg.1	DYNC2H1
TC11000963.hg.1	2.27	JUC11006416.hg.1	DYNC2H1
TC11000963.hg.1	2.08	JUC11006371.hg.1	DYNC2H1
TC11000963.hg.1	-2.03	JUC11006359.hg.1	DYNC2H1
TC11000963.hg.1	-2.06	JUC11006418.hg.1	DYNC2H1
TC11000963.hg.1	-2.21	PSR11012007.hg.1	DYNC2H1
TC11000963.hg.1	-2.86	JUC11006421.hg.1	DYNC2H1
TC11000963.hg.1	-2.94	JUC11006426.hg.1	DYNC2H1
TC15000448.hg.1	3.72	PSR15004259.hg.1	RNF111
TC15000448.hg.1	2.41	PSR15004270.hg.1	RNF111
TC15000448.hg.1	2.14	JUC15002098.hg.1	RNF111
TC15000448.hg.1	-2.11	JUC15002103.hg.1	RNF111
TC15000465.hg.1	3.72	JUC15002153.hg.1	
TC15000465.hg.1	-2.64	PSR15004356.hg.1	
TC15000465.hg.1	-2.99	PSR15004360.hg.1	
TC17001341.hg.1	3.72	JUC17010104.hg.1	EVI2B
TC17001341.hg.1	2.59	JUC17010105.hg.1	EVI2B
TC17001341.hg.1	2.51	PSR17017838.hg.1	EVI2B
TC11001523.hg.1	3.71	JUC11010156.hg.1	MPPED2
TC11001523.hg.1	2.41	JUC11010157.hg.1	MPPED2
TC11001523.hg.1	2.24	JUC11010151.hg.1	MPPED2
TC11001523.hg.1	2.22	PSR11019027.hg.1	MPPED2
TC12001412.hg.1	3.71	PSR12018284.hg.1	PUS7L
TC12001412.hg.1	-2.44	JUC12010201.hg.1	PUS7L
TC15000433.hg.1	3.71	JUC15001930.hg.1	TEX9
TC15000433.hg.1	2.12	PSR15003992.hg.1	TEX9

TC15001612.hg.1	3.71	PSR15015540.hg.1	UACA
TC15001612.hg.1	2.32	JUC15008438.hg.1	UACA
TC15001612.hg.1	2.14	PSR15015516.hg.1	UACA
TC15001612.hg.1	-2.07	JUC15008436.hg.1	UACA
TC15001612.hg.1	-2.23	JUC15008448.hg.1	UACA
TC01000313.hg.1	3.7	PSR01004752.hg.1	SRRM1
TC01000313.hg.1	2.21	JUC01002558.hg.1	SRRM1
TC01000313.hg.1	-2	PSR01004768.hg.1	SRRM1
TC01000313.hg.1	-2.01	JUC01002562.hg.1	SRRM1
TC01000313.hg.1	-2.12	JUC01002557.hg.1	SRRM1
TC01000313.hg.1	-2.31	PSR01004771.hg.1	SRRM1
TC04000072.hg.1	3.7	JUC04000757.hg.1	S100P
TC04000072.hg.1	2.21	PSR04001641.hg.1	S100P
TC04000072.hg.1	2.01	JUC04000758.hg.1	S100P
TC04000072.hg.1	-2.08	PSR04001636.hg.1	S100P
TC04000181.hg.1	3.7	PSR04002853.hg.1	RBPJ
TC04000181.hg.1	2.43	JUC04001353.hg.1	RBPJ
TC04000181.hg.1	2.05	JUC04001365.hg.1	RBPJ
TC08000646.hg.1	3.7	JUC08004469.hg.1	CTHRC1
TC08000646.hg.1	3.18	PSR08008801.hg.1	CTHRC1
TC08000646.hg.1	2.13	JUC08004465.hg.1	CTHRC1
TC08000646.hg.1	-2.95	PSR08008790.hg.1	CTHRC1
TC08000646.hg.1	-6.65	PSR08008793.hg.1	CTHRC1
TC08000646.hg.1	-9.34	PSR08008788.hg.1	CTHRC1
TC10001195.hg.1	3.7	JUC10008706.hg.1	ZNF33B
TC10001195.hg.1	2.54	PSR10015007.hg.1	ZNF33B
TC13001718.hg.1	3.7	JUC13008373.hg.1	LINC00598
TC13001718.hg.1	2.23	PSR13006760.hg.1	LINC00598
TC15001507.hg.1	3.7	JUC15007696.hg.1	RORA
TC15001507.hg.1	2.98	PSR15014280.hg.1	RORA
TC15001507.hg.1	2.8	JUC15007691.hg.1	RORA
TC15001507.hg.1	2.26	PSR15014285.hg.1	RORA
TC15001507.hg.1	2.01	PSR15014274.hg.1	RORA
TC15001507.hg.1	2.01	PSR15014282.hg.1	RORA
TC15001507.hg.1	-2.32	PSR15014275.hg.1	RORA
TC16000464.hg.1	3.7	JUC16003631.hg.1	MT4
TC16000464.hg.1	2.88	PSR16006451.hg.1	MT4
TC19000058.hg.1	3.7	PSR19000898.hg.1	ZNF554
TC19000058.hg.1	2.3	JUC19000493.hg.1	ZNF554
TC19000058.hg.1	2.01	JUC19000494.hg.1	ZNF554
TC01002519.hg.1	3.69	PSR01039158.hg.1	GNL2
TC01002519.hg.1	3.68	JUC01021022.hg.1	GNL2
TC01002519.hg.1	-2.02	JUC01021017.hg.1	GNL2
TC01002519.hg.1	-2.08	JUC01021027.hg.1	GNL2
TC01003801.hg.1	3.68	JUC01030384.hg.1	INTS7
TC01003801.hg.1	2.87	JUC01030389.hg.1	INTS7
TC01003801.hg.1	-2.09	PSR01058132.hg.1	INTS7
TC01003801.hg.1	-3.32	JUC01030387.hg.1	INTS7
TC08000579.hg.1	3.68	JUC08004032.hg.1	NDUFAF6, L(
TC08000579.hg.1	3.01	JUC08004050.hg.1	NDUFAF6, L(
TC08000579.hg.1	2.92	PSR08008029.hg.1	NDUFAF6, L(
TC08000579.hg.1	2.52	JUC08004063.hg.1	NDUFAF6, L(
TC08000579.hg.1	2.3	JUC08004054.hg.1	NDUFAF6, L(
TC08000579.hg.1	2.25	JUC08004036.hg.1	NDUFAF6, L(
TC08000579.hg.1	2.17	PSR08008073.hg.1	NDUFAF6, L(
TC08000579.hg.1	2.16	JUC08004030.hg.1	NDUFAF6, L(
TC08000579.hg.1	2.11	JUC08004041.hg.1	NDUFAF6, L(
TC08000579.hg.1	-4.46	PSR08008033.hg.1	NDUFAF6, L(

TC10000663.hg.1	3.68	JUC10004242.hg.1	CYP26A1
TC10000663.hg.1	2.09	PSR10007472.hg.1	CYP26A1
TC10000663.hg.1	-2.03	PSR10007469.hg.1	CYP26A1
TC10000663.hg.1	-2.64	JUC10004246.hg.1	CYP26A1
TC10000663.hg.1	-3.06	JUC10004248.hg.1	CYP26A1
TC10000663.hg.1	-4.43	PSR10007467.hg.1	CYP26A1
TC10000663.hg.1	-8.56	PSR10007465.hg.1	CYP26A1
TC10000663.hg.1	-10.01	PSR10007466.hg.1	CYP26A1
TC10000663.hg.1	-13.3	JUC10004247.hg.1	CYP26A1
TC12001862.hg.1	3.68	JUC12013549.hg.1	ANKS1B
TC12001862.hg.1	2.77	JUC12013574.hg.1	ANKS1B
TC12001862.hg.1	2.71	JUC12013578.hg.1	ANKS1B
TC12001862.hg.1	2.61	PSR12024377.hg.1	ANKS1B
TC12001862.hg.1	2.04	JUC12013554.hg.1	ANKS1B
TC12001862.hg.1	-2.03	PSR12024405.hg.1	ANKS1B
TC12001862.hg.1	-2.03	PSR12024411.hg.1	ANKS1B
TC12001862.hg.1	-2.03	JUC12013560.hg.1	ANKS1B
TC12001862.hg.1	-2.17	PSR12024400.hg.1	ANKS1B
TC12001862.hg.1	-2.18	PSR12024408.hg.1	ANKS1B
TC12001862.hg.1	-2.38	PSR12024393.hg.1	ANKS1B
TC12001862.hg.1	-2.47	PSR12024401.hg.1	ANKS1B
TC12001862.hg.1	-2.55	JUC12013571.hg.1	ANKS1B
TC12001862.hg.1	-2.76	PSR12024403.hg.1	ANKS1B
TC12001862.hg.1	-2.9	JUC12013584.hg.1	ANKS1B
TC12001862.hg.1	-3.58	JUC12013566.hg.1	ANKS1B
TC02000270.hg.1	3.67	PSR02003998.hg.1	PLEKHH2
TC02000270.hg.1	3.09	PSR02003966.hg.1	PLEKHH2
TC02000270.hg.1	2.78	PSR02003968.hg.1	PLEKHH2
TC02000270.hg.1	2.56	PSR02003974.hg.1	PLEKHH2
TC02000270.hg.1	2.55	JUC02002068.hg.1	PLEKHH2
TC02000270.hg.1	2.34	PSR02003973.hg.1	PLEKHH2
TC02000270.hg.1	2.09	JUC02002064.hg.1	PLEKHH2
TC02000270.hg.1	2.08	PSR02003969.hg.1	PLEKHH2
TC02000270.hg.1	2.02	PSR02004001.hg.1	PLEKHH2
TC02000270.hg.1	-2.13	JUC02002082.hg.1	PLEKHH2
TC02000270.hg.1	-2.22	JUC02002071.hg.1	PLEKHH2
TC02000270.hg.1	-2.37	JUC02002062.hg.1	PLEKHH2
TC02000270.hg.1	-2.54	JUC02002076.hg.1	PLEKHH2
TC02001361.hg.1	3.67	JUC02011276.hg.1	COL4A3
TC02001361.hg.1	2.58	PSR02021194.hg.1	COL4A3
TC02001361.hg.1	2.56	JUC02011325.hg.1	COL4A3
TC02001361.hg.1	-2.04	JUC02011342.hg.1	COL4A3
TC02001361.hg.1	-2.05	PSR02021199.hg.1	COL4A3
TC02001361.hg.1	-2.14	PSR02021173.hg.1	COL4A3
TC02001361.hg.1	-2.14	JUC02011306.hg.1	COL4A3
TC02001361.hg.1	-2.21	JUC02011282.hg.1	COL4A3
TC02001361.hg.1	-2.24	PSR02021192.hg.1	COL4A3
TC02001361.hg.1	-2.26	PSR02021214.hg.1	COL4A3
TC02001361.hg.1	-2.29	PSR02021174.hg.1	COL4A3
TC02001361.hg.1	-2.46	PSR02021215.hg.1	COL4A3
TC02002434.hg.1	3.67	PSR02038449.hg.1	PRPF40A
TC02002434.hg.1	2.24	JUC02020111.hg.1	PRPF40A
TC02002434.hg.1	2.01	JUC02020118.hg.1	PRPF40A
TC03000375.hg.1	3.67	PSR03008088.hg.1	FLNB
TC03000375.hg.1	2.86	PSR03008086.hg.1	FLNB
TC03000375.hg.1	2.49	JUC03003992.hg.1	FLNB
TC03000375.hg.1	2.13	PSR03008087.hg.1	FLNB
TC03000375.hg.1	2	PSR03008145.hg.1	FLNB

TC05000433.hg.1	3.67	JUC05003335.hg.1	TMEM161B-A
TC05000433.hg.1	3.63	JUC05003333.hg.1	TMEM161B-A
TC05000433.hg.1	2.68	JUC05003347.hg.1	TMEM161B-A
TC05000433.hg.1	2.24	PSR05006114.hg.1	TMEM161B-A
TC05000433.hg.1	2.08	JUC05003337.hg.1	TMEM161B-A
TC05000433.hg.1	-2.41	JUC05003345.hg.1	TMEM161B-A
TC06000938.hg.1	3.67	JUC06005231.hg.1	
TC06000938.hg.1	2.15	PSR06010971.hg.1	
TC07000652.hg.1	3.67	JUC07004731.hg.1	SH2B2
TC07000652.hg.1	-2.58	JUC07004744.hg.1	SH2B2
TC07000652.hg.1	-2.96	PSR07010179.hg.1	SH2B2
TC15001230.hg.1	3.67	JUC15005838.hg.1	BMF
TC15001230.hg.1	-2.14	PSR15011035.hg.1	BMF
TC15001230.hg.1	-2.61	PSR15011036.hg.1	BMF
TC02002673.hg.1	3.67	JUC02022487.hg.1	ALS2CR12
TC02002673.hg.1	-2	PSR02042976.hg.1	ALS2CR12
TC05000398.hg.1	3.66	JUC05003062.hg.1	ZFYVE16
TC05000398.hg.1	3.33	JUC05003068.hg.1	ZFYVE16
TC05000398.hg.1	2.51	PSR05005619.hg.1	ZFYVE16
TC05000398.hg.1	2.31	JUC05003070.hg.1	ZFYVE16
TC05000398.hg.1	2.23	PSR05005622.hg.1	ZFYVE16
TC05000398.hg.1	-2.73	JUC05003063.hg.1	ZFYVE16
TC10000204.hg.1	3.66	JUC10001311.hg.1	LOC387647
TC10000204.hg.1	-2.05	PSR10002437.hg.1	LOC387647
TC10000204.hg.1	-2.26	PSR10002435.hg.1	LOC387647
TC10000204.hg.1	-2.77	PSR10002436.hg.1	LOC387647
TC10000204.hg.1	-3.51	JUC10001319.hg.1	LOC387647
TC15002009.hg.1	3.66	PSR15018660.hg.1	PCSK6, LOC
TC15002009.hg.1	3.6	JUC15010174.hg.1	PCSK6, LOC
TC15002009.hg.1	3.15	JUC15010170.hg.1	PCSK6, LOC
TC15002009.hg.1	3.02	PSR15018654.hg.1	PCSK6, LOC
TC15002009.hg.1	2.76	PSR15018628.hg.1	PCSK6, LOC
TC15002009.hg.1	2.55	JUC15010166.hg.1	PCSK6, LOC
TC15002009.hg.1	2.51	JUC15010152.hg.1	PCSK6, LOC
TC15002009.hg.1	2.47	PSR15018637.hg.1	PCSK6, LOC
TC15002009.hg.1	2.46	PSR15018627.hg.1	PCSK6, LOC
TC15002009.hg.1	2.33	JUC15010161.hg.1	PCSK6, LOC
TC15002009.hg.1	2.28	JUC15010169.hg.1	PCSK6, LOC
TC15002009.hg.1	2.26	PSR15018659.hg.1	PCSK6, LOC
TC15002009.hg.1	2.07	PSR15018645.hg.1	PCSK6, LOC
TC15002009.hg.1	2.07	PSR15018650.hg.1	PCSK6, LOC
TC15002009.hg.1	2.02	JUC15010163.hg.1	PCSK6, LOC
TC15002009.hg.1	-2.01	PSR15018648.hg.1	PCSK6, LOC
TC15002009.hg.1	-2.25	PSR15018642.hg.1	PCSK6, LOC
TC15002009.hg.1	-2.68	PSR15018629.hg.1	PCSK6, LOC
TC15002009.hg.1	-2.93	JUC15010167.hg.1	PCSK6, LOC
TC15002009.hg.1	-3.11	JUC15010151.hg.1	PCSK6, LOC
TC15002009.hg.1	-3.12	JUC15010173.hg.1	PCSK6, LOC
TC15002009.hg.1	-3.28	JUC15010164.hg.1	PCSK6, LOC
TC15002009.hg.1	-3.38	JUC15010149.hg.1	PCSK6, LOC
TC15002009.hg.1	-4.15	JUC15010154.hg.1	PCSK6, LOC
TC15002009.hg.1	-4.25	PSR15018626.hg.1	PCSK6, LOC
TC15002009.hg.1	-5.33	JUC15010175.hg.1	PCSK6, LOC
TC15002009.hg.1	-10.64	JUC15010172.hg.1	PCSK6, LOC
TC15002009.hg.1	-11.81	JUC15010157.hg.1	PCSK6, LOC
TC22000326.hg.1	3.66	JUC22002265.hg.1	MCHR1
TC22000326.hg.1	2.27	JUC22002267.hg.1	MCHR1
TC22000326.hg.1	2.12	PSR22006285.hg.1	MCHR1

TC22000326.hg.1	2.12	PSR22006286.hg.1	MCHR1
TC22000326.hg.1	-2.43	JUC22002266.hg.1	MCHR1
TC22000326.hg.1	-2.49	PSR22006289.hg.1	MCHR1
TC22000326.hg.1	-4.62	PSR22006288.hg.1	MCHR1
TC01000024.hg.1	3.65	JUC01000127.hg.1	AGRN
TC01000024.hg.1	2.52	PSR01000260.hg.1	AGRN
TC01000024.hg.1	2.48	PSR01000278.hg.1	AGRN
TC01000024.hg.1	2.12	JUC01000124.hg.1	AGRN
TC01000024.hg.1	2.12	JUC01000137.hg.1	AGRN
TC01000024.hg.1	-2.02	PSR01000248.hg.1	AGRN
TC01000024.hg.1	-2.02	JUC01000126.hg.1	AGRN
TC01000024.hg.1	-2.05	JUC01000149.hg.1	AGRN
TC01000024.hg.1	-2.37	JUC01000125.hg.1	AGRN
TC01000024.hg.1	-2.43	JUC01000136.hg.1	AGRN
TC01000151.hg.1	3.65	JUC01001329.hg.1	PLOD1
TC01000151.hg.1	2.03	PSR01002441.hg.1	PLOD1
TC02000939.hg.1	3.65	JUC02007348.hg.1	RIF1
TC02000939.hg.1	2.42	JUC02007366.hg.1	RIF1
TC02000939.hg.1	2.16	PSR02013734.hg.1	RIF1
TC02000939.hg.1	2.15	PSR02013678.hg.1	RIF1
TC02000939.hg.1	-2.03	JUC02007341.hg.1	RIF1
TC02000939.hg.1	-3.28	JUC02007347.hg.1	RIF1
TC03000419.hg.1	3.65	JUC03004347.hg.1	MITF
TC03000419.hg.1	3.35	JUC03004357.hg.1	MITF
TC03000419.hg.1	2.52	PSR03008700.hg.1	MITF
TC03000419.hg.1	2.47	JUC03004345.hg.1	MITF
TC03000419.hg.1	2.44	PSR03008712.hg.1	MITF
TC03000419.hg.1	2.36	JUC03004355.hg.1	MITF
TC03000419.hg.1	2.3	PSR03008715.hg.1	MITF
TC03000419.hg.1	2.08	JUC03004342.hg.1	MITF
TC03000419.hg.1	2.04	PSR03008748.hg.1	MITF
TC03001004.hg.1	3.65	JUC03008973.hg.1	CHRD
TC03001004.hg.1	-2.24	PSR03017705.hg.1	CHRD
TC03001004.hg.1	-4.36	JUC03008979.hg.1	CHRD
TC04002910.hg.1	3.65	JUC04016768.hg.1	RNF4
TC04002910.hg.1	-2.03	PSR04000829.hg.1	RNF4
TC04002910.hg.1	-2.36	JUC04016749.hg.1	RNF4
TC04002910.hg.1	-2.97	JUC04016751.hg.1	RNF4
TC06000976.hg.1	3.65	JUC06005517.hg.1	LAMA2
TC06000976.hg.1	3.56	JUC06005503.hg.1	LAMA2
TC06000976.hg.1	3.43	JUC06005480.hg.1	LAMA2
TC06000976.hg.1	3.19	JUC06005525.hg.1	LAMA2
TC06000976.hg.1	3.18	JUC06005458.hg.1	LAMA2
TC06000976.hg.1	3.08	PSR06011415.hg.1	LAMA2
TC06000976.hg.1	2.95	PSR06011470.hg.1	LAMA2
TC06000976.hg.1	2.87	PSR06011435.hg.1	LAMA2
TC06000976.hg.1	2.81	JUC06005486.hg.1	LAMA2
TC06000976.hg.1	2.74	JUC06005499.hg.1	LAMA2
TC06000976.hg.1	2.7	JUC06005473.hg.1	LAMA2
TC06000976.hg.1	2.67	JUC06005489.hg.1	LAMA2
TC06000976.hg.1	2.52	JUC06005483.hg.1	LAMA2
TC06000976.hg.1	2.48	JUC06005521.hg.1	LAMA2
TC06000976.hg.1	2.45	JUC06005476.hg.1	LAMA2
TC06000976.hg.1	2.45	JUC06005495.hg.1	LAMA2
TC06000976.hg.1	2.43	JUC06005482.hg.1	LAMA2
TC06000976.hg.1	2.4	JUC06005513.hg.1	LAMA2
TC06000976.hg.1	2.24	PSR06011400.hg.1	LAMA2
TC06000976.hg.1	2.23	JUC06005456.hg.1	LAMA2

TC06000976.hg.1	2.2	PSR06011462.hg.1	LAMA2
TC06000976.hg.1	2.15	PSR06011414.hg.1	LAMA2
TC06000976.hg.1	2.1	PSR06011398.hg.1	LAMA2
TC06000976.hg.1	2.09	JUC06005506.hg.1	LAMA2
TC06000976.hg.1	2.06	PSR06011408.hg.1	LAMA2
TC06000976.hg.1	-2	JUC06005500.hg.1	LAMA2
TC06000976.hg.1	-2.02	PSR06011428.hg.1	LAMA2
TC06000976.hg.1	-2.02	JUC06005504.hg.1	LAMA2
TC06000976.hg.1	-2.07	JUC06005462.hg.1	LAMA2
TC06000976.hg.1	-2.1	PSR06011404.hg.1	LAMA2
TC06000976.hg.1	-2.15	PSR06011424.hg.1	LAMA2
TC06000976.hg.1	-2.17	JUC06005465.hg.1	LAMA2
TC06000976.hg.1	-2.28	JUC06005494.hg.1	LAMA2
TC06000976.hg.1	-2.43	PSR06011427.hg.1	LAMA2
TC06000976.hg.1	-2.55	JUC06005511.hg.1	LAMA2
TC06000976.hg.1	-2.58	PSR06011396.hg.1	LAMA2
TC06000976.hg.1	-2.64	PSR06011421.hg.1	LAMA2
TC06000976.hg.1	-2.69	PSR06011403.hg.1	LAMA2
TC06000976.hg.1	-2.7	PSR06011438.hg.1	LAMA2
TC06000976.hg.1	-2.9	JUC06005531.hg.1	LAMA2
TC06000976.hg.1	-3.08	PSR06011449.hg.1	LAMA2
TC06000976.hg.1	-3.14	JUC06005522.hg.1	LAMA2
TC06000976.hg.1	-3.2	PSR06011409.hg.1	LAMA2
TC06000976.hg.1	-3.3	PSR06011430.hg.1	LAMA2
TC06000976.hg.1	-3.31	JUC06005463.hg.1	LAMA2
TC06000976.hg.1	-3.35	JUC06005487.hg.1	LAMA2
TC06000976.hg.1	-3.54	JUC06005475.hg.1	LAMA2
TC06000976.hg.1	-3.82	JUC06005527.hg.1	LAMA2
TC06000976.hg.1	-3.84	JUC06005523.hg.1	LAMA2
TC06000976.hg.1	-3.91	PSR06011419.hg.1	LAMA2
TC06000976.hg.1	-3.92	JUC06005464.hg.1	LAMA2
TC06000976.hg.1	-4.12	PSR06011459.hg.1	LAMA2
TC06000976.hg.1	-4.36	PSR06011475.hg.1	LAMA2
TC06000976.hg.1	-4.39	PSR06011422.hg.1	LAMA2
TC06000976.hg.1	-4.41	PSR06011432.hg.1	LAMA2
TC06000976.hg.1	-4.62	PSR06011464.hg.1	LAMA2
TC06000976.hg.1	-4.89	PSR06011397.hg.1	LAMA2
TC06000976.hg.1	-5.72	PSR06011395.hg.1	LAMA2
TC06000976.hg.1	-5.82	PSR06011407.hg.1	LAMA2
TC06000976.hg.1	-6	JUC06005474.hg.1	LAMA2
TC06000976.hg.1	-6.41	PSR06011460.hg.1	LAMA2
TC06000976.hg.1	-6.52	JUC06005488.hg.1	LAMA2
TC06000976.hg.1	-8.12	JUC06005472.hg.1	LAMA2
TC06000976.hg.1	-8.49	JUC06005459.hg.1	LAMA2
TC06000976.hg.1	-8.59	PSR06011429.hg.1	LAMA2
TC06000976.hg.1	-10.18	PSR06011426.hg.1	LAMA2
TC06000976.hg.1	-10.64	JUC06005470.hg.1	LAMA2
TC06000976.hg.1	-10.89	PSR06011476.hg.1	LAMA2
TC06000976.hg.1	-13.42	JUC06005508.hg.1	LAMA2
TC06000976.hg.1	-16.1	JUC06005505.hg.1	LAMA2
TC06000976.hg.1	-16.84	PSR06011393.hg.1	LAMA2
TC06000976.hg.1	-17.87	JUC06005491.hg.1	LAMA2
TC06000976.hg.1	-41.24	JUC06005461.hg.1	LAMA2
TC10001327.hg.1	3.65	PSR10016755.hg.1	EGR2
TC10001327.hg.1	3.21	JUC10009791.hg.1	EGR2
TC10001327.hg.1	-2.26	PSR10016756.hg.1	EGR2
TC10001327.hg.1	-2.74	PSR10016761.hg.1	EGR2
TC10001327.hg.1	-3.37	JUC10009789.hg.1	EGR2

TC10001327.hg.1	-4.15	PSR10016747.hg.1	EGR2
TC10001327.hg.1	-6.19	PSR10016751.hg.1	EGR2
TC10001327.hg.1	-6.89	PSR10016769.hg.1	EGR2
TC10001327.hg.1	-7.52	PSR10016749.hg.1	EGR2
TC10001327.hg.1	-7.59	JUC10009783.hg.1	EGR2
TC10001327.hg.1	-9.54	PSR10016767.hg.1	EGR2
TC10001327.hg.1	-11.23	PSR10016770.hg.1	EGR2
TC10001327.hg.1	-13.54	JUC10009784.hg.1	EGR2
TC13000687.hg.1	3.65	JUC13004679.hg.1	MRPS31P3,
TC13000687.hg.1	2.4	PSR13008054.hg.1	MRPS31P3,
TC13000687.hg.1	2.4	JUC13004702.hg.1	MRPS31P3,
TC13000687.hg.1	2.39	JUC13004698.hg.1	MRPS31P3,
TC13000687.hg.1	2.28	JUC13004693.hg.1	MRPS31P3,
TC13000687.hg.1	2.27	PSR13008065.hg.1	MRPS31P3,
TC13000687.hg.1	2.21	JUC13004714.hg.1	MRPS31P3,
TC13000687.hg.1	2.01	PSR13008050.hg.1	MRPS31P3,
TC17000655.hg.1	3.65	JUC17004544.hg.1	PDK2
TC17000655.hg.1	3.13	PSR17008211.hg.1	PDK2
TC17000655.hg.1	2.27	PSR17008226.hg.1	PDK2
TC17000655.hg.1	2.16	PSR17008228.hg.1	PDK2
TC17000655.hg.1	2.12	PSR17008208.hg.1	PDK2
TC17000655.hg.1	2.07	PSR17008245.hg.1	PDK2
TC17000655.hg.1	2.05	PSR17008240.hg.1	PDK2
TC17000655.hg.1	2.04	PSR17008232.hg.1	PDK2
TC17000953.hg.1	3.65	JUC17007103.hg.1	NARF
TC17000953.hg.1	2.35	PSR17012534.hg.1	NARF
TC17000953.hg.1	2.23	JUC17007117.hg.1	NARF
TC17000953.hg.1	2.21	PSR17012539.hg.1	NARF
TC17002913.hg.1	3.65	PSR17003578.hg.1	
TC17002913.hg.1	3.06	PSR17003576.hg.1	
TC17002913.hg.1	2.1	JUC17019182.hg.1	
TC17002913.hg.1	-2.18	JUC17019178.hg.1	
TC17002913.hg.1	-2.55	PSR17003573.hg.1	
TC17002913.hg.1	-2.63	JUC17019181.hg.1	
TC17002913.hg.1	-3.17	JUC17019179.hg.1	
TC19001155.hg.1	3.65	JUC19009270.hg.1	OLFM2
TC19001155.hg.1	3.26	PSR19015949.hg.1	OLFM2
TC19001155.hg.1	-2.31	JUC19009268.hg.1	OLFM2
TC20000198.hg.1	3.65	JUC20001274.hg.1	TPX2
TC20000198.hg.1	3.25	JUC20001276.hg.1	TPX2
TC20000198.hg.1	3.02	PSR20002360.hg.1	TPX2
TC20000198.hg.1	3	PSR20002361.hg.1	TPX2
TC20000198.hg.1	2.2	PSR20002363.hg.1	TPX2
TC20000198.hg.1	2.12	JUC20001275.hg.1	TPX2
TC20000198.hg.1	-2	JUC20001264.hg.1	TPX2
TC20000198.hg.1	-2.37	JUC20001266.hg.1	TPX2
TC01002405.hg.1	3.64	JUC01019958.hg.1	MAP3K6
TC01002405.hg.1	2.2	PSR01037073.hg.1	MAP3K6
TC02002478.hg.1	3.64	JUC02020440.hg.1	DPP4
TC02002478.hg.1	2.6	JUC02020445.hg.1	DPP4
TC02002478.hg.1	2.53	PSR02039034.hg.1	DPP4
TC02002478.hg.1	2.38	PSR02039024.hg.1	DPP4
TC02002478.hg.1	2.26	PSR02039017.hg.1	DPP4
TC02002478.hg.1	2.26	JUC02020450.hg.1	DPP4
TC02002478.hg.1	2.24	JUC02020429.hg.1	DPP4
TC02002478.hg.1	2.04	JUC02020454.hg.1	DPP4
TC02002478.hg.1	-2.04	PSR02039063.hg.1	DPP4
TC02002478.hg.1	-2.06	PSR02039058.hg.1	DPP4

TC02002478.hg.1	-2.21	PSR02039054.hg.1	DPP4
TC02002478.hg.1	-2.39	PSR02039062.hg.1	DPP4
TC02002478.hg.1	-2.72	JUC02020441.hg.1	DPP4
TC02002478.hg.1	-2.84	PSR02039031.hg.1	DPP4
TC02002478.hg.1	-3.59	PSR02039019.hg.1	DPP4
TC08002626.hg.1	3.64	JUC08015390.hg.1	ZFPM2
TC08002626.hg.1	3.19	JUC08015379.hg.1	ZFPM2
TC08002626.hg.1	2.88	JUC08015383.hg.1	ZFPM2
TC08002626.hg.1	2.68	PSR08008961.hg.1	ZFPM2
TC08002626.hg.1	2.63	JUC08015382.hg.1	ZFPM2
TC08002626.hg.1	2.36	PSR08008939.hg.1	ZFPM2
TC08002626.hg.1	2.34	PSR08008936.hg.1	ZFPM2
TC08002626.hg.1	2.2	PSR08008944.hg.1	ZFPM2
TC08002626.hg.1	2.04	PSR08008948.hg.1	ZFPM2
TC08002626.hg.1	2.03	PSR08008934.hg.1	ZFPM2
TC09000508.hg.1	3.64	PSR09005517.hg.1	NR4A3
TC09000508.hg.1	2.98	PSR09005519.hg.1	NR4A3
TC09000508.hg.1	2.42	PSR09005521.hg.1	NR4A3
TC09000508.hg.1	2.17	JUC09003064.hg.1	NR4A3
TC09000508.hg.1	2.15	JUC09003071.hg.1	NR4A3
TC09000508.hg.1	-2.29	JUC09003066.hg.1	NR4A3
TC12001328.hg.1	3.64	JUC12009591.hg.1	ITPR2
TC12001328.hg.1	2.82	JUC12009606.hg.1	ITPR2
TC12001328.hg.1	2.58	JUC12009632.hg.1	ITPR2
TC12001328.hg.1	2.21	JUC12009598.hg.1	ITPR2
TC12001328.hg.1	2.11	JUC12009618.hg.1	ITPR2
TC12001328.hg.1	-2.41	JUC12009626.hg.1	ITPR2
TC12001328.hg.1	-2.74	PSR12017380.hg.1	ITPR2
TC18000418.hg.1	3.64	JUC18002877.hg.1	OSBPL1A
TC18000418.hg.1	2.56	PSR18004277.hg.1	OSBPL1A
TC18000418.hg.1	2.38	JUC18002862.hg.1	OSBPL1A
TC18000418.hg.1	2.26	PSR18004259.hg.1	OSBPL1A
TC18000418.hg.1	2.12	PSR18004278.hg.1	OSBPL1A
TC18000418.hg.1	2.07	JUC18002876.hg.1	OSBPL1A
TC18000418.hg.1	2.06	PSR18004264.hg.1	OSBPL1A
TC18000418.hg.1	-2.15	PSR18004244.hg.1	OSBPL1A
TC18000418.hg.1	-2.2	JUC18002880.hg.1	OSBPL1A
TC19001792.hg.1	3.64	JUC19013843.hg.1	ZNF615
TC19001792.hg.1	2.27	PSR19024351.hg.1	ZNF615
TC01001799.hg.1	3.63	JUC01015170.hg.1	SPATA17
TC01001799.hg.1	3.2	PSR01028129.hg.1	SPATA17
TC01001799.hg.1	3.1	JUC01015157.hg.1	SPATA17
TC01001799.hg.1	-2.02	PSR01028121.hg.1	SPATA17
TC01001799.hg.1	-2.08	PSR01028134.hg.1	SPATA17
TC01001799.hg.1	-2.09	JUC01015165.hg.1	SPATA17
TC01001799.hg.1	-2.15	JUC01015172.hg.1	SPATA17
TC01001799.hg.1	-2.18	PSR01028120.hg.1	SPATA17
TC01001799.hg.1	-2.41	JUC01015167.hg.1	SPATA17
TC01001799.hg.1	-2.76	JUC01015155.hg.1	SPATA17
TC04001129.hg.1	3.63	PSR04015453.hg.1	RPL9
TC04001129.hg.1	3.23	JUC04008191.hg.1	RPL9
TC04001129.hg.1	2.23	JUC04008184.hg.1	RPL9
TC04001129.hg.1	2.13	JUC04008187.hg.1	RPL9
TC04001129.hg.1	2.04	PSR04015448.hg.1	RPL9
TC05001545.hg.1	3.63	JUC05010884.hg.1	SSBP2
TC05001545.hg.1	-2.4	PSR05021103.hg.1	SSBP2
TC06000597.hg.1	3.63	JUC06003038.hg.1	CUL9
TC06000597.hg.1	3.07	JUC06003048.hg.1	CUL9

TC06000597.hg.1	2.95	JUC06003035.hg.1	CUL9
TC06000597.hg.1	2.77	PSR06007024.hg.1	CUL9
TC06000597.hg.1	2.71	JUC06003053.hg.1	CUL9
TC06000597.hg.1	2.56	PSR06007040.hg.1	CUL9
TC06000597.hg.1	2.43	JUC06003040.hg.1	CUL9
TC06000597.hg.1	2.42	JUC06003057.hg.1	CUL9
TC06000597.hg.1	2.38	JUC06003023.hg.1	CUL9
TC06000597.hg.1	2.24	PSR06006969.hg.1	CUL9
TC06000597.hg.1	2.21	PSR06007007.hg.1	CUL9
TC06000597.hg.1	2.14	JUC06003066.hg.1	CUL9
TC06000597.hg.1	2.11	PSR06007019.hg.1	CUL9
TC06000597.hg.1	2.08	PSR06007039.hg.1	CUL9
TC06000597.hg.1	2.08	PSR06007041.hg.1	CUL9
TC06000597.hg.1	2.06	PSR06006965.hg.1	CUL9
TC06000597.hg.1	2.06	PSR06006996.hg.1	CUL9
TC06000597.hg.1	2.02	PSR06007027.hg.1	CUL9
TC06000597.hg.1	2.01	JUC06003046.hg.1	CUL9
TC06000597.hg.1	2	PSR06006994.hg.1	CUL9
TC08000021.hg.1	3.63	JUC08000193.hg.1	MCPH1
TC08000021.hg.1	3.62	JUC08000184.hg.1	MCPH1
TC08000021.hg.1	2.3	PSR08000333.hg.1	MCPH1
TC08000021.hg.1	2.01	JUC08000179.hg.1	MCPH1
TC08000021.hg.1	-2.2	PSR08000327.hg.1	MCPH1
TC08000021.hg.1	-2.35	PSR08000326.hg.1	MCPH1
TC08000343.hg.1	3.63	JUC08002495.hg.1	KIAA0146
TC08000343.hg.1	3.3	JUC08002519.hg.1	KIAA0146
TC08000343.hg.1	2.84	JUC08002515.hg.1	KIAA0146
TC08000343.hg.1	2.51	JUC08002501.hg.1	KIAA0146
TC08000343.hg.1	2.01	JUC08002513.hg.1	KIAA0146
TC08000343.hg.1	-2.05	PSR08005075.hg.1	KIAA0146
TC08000343.hg.1	-2.19	PSR08005074.hg.1	KIAA0146
TC08000343.hg.1	-3.33	JUC08002504.hg.1	KIAA0146
TC08000343.hg.1	-3.76	PSR08005071.hg.1	KIAA0146
TC08000343.hg.1	-4.58	PSR08005069.hg.1	KIAA0146
TC08001737.hg.1	3.63	JUC08011832.hg.1	PLEC
TC08001737.hg.1	2.45	JUC08011820.hg.1	PLEC
TC08001737.hg.1	2.33	JUC08011816.hg.1	PLEC
TC08001737.hg.1	2.1	PSR08022960.hg.1	PLEC
TC15001264.hg.1	3.63	PSR15011658.hg.1	ZFP106
TC15001264.hg.1	2.99	JUC15006257.hg.1	ZFP106
TC16002037.hg.1	3.63	JUC16013222.hg.1	CLEC18C
TC16002037.hg.1	2.68	JUC16013218.hg.1	CLEC18C
TC16002037.hg.1	-2.15	PSR16008327.hg.1	CLEC18C
TC16002037.hg.1	-2.67	PSR16008351.hg.1	CLEC18C
TC19000037.hg.1	3.63	JUC19000290.hg.1	NDUFS7
TC19000037.hg.1	2.66	JUC19000286.hg.1	NDUFS7
TC19000037.hg.1	2.02	PSR19000501.hg.1	NDUFS7
TC6_mcf_hap5000	3.63	PSR6_mcf_hap5000	HLA-H
TC6_mcf_hap5000	2.85	PSR6_mcf_hap5000	HLA-H
TC6_mcf_hap5000	2.14	PSR6_mcf_hap5000	HLA-H
TC6_mcf_hap5000	-2.51	JUC6_mcf_hap5001	HLA-H
TC01000129.hg.1	3.62	JUC01001110.hg.1	PGD
TC01000129.hg.1	2.46	PSR01002045.hg.1	PGD
TC01000129.hg.1	2.2	PSR01002066.hg.1	PGD
TC01003627.hg.1	3.62	JUC01029099.hg.1	TRMT1L
TC01003627.hg.1	2.34	PSR01055795.hg.1	TRMT1L
TC01003627.hg.1	2.18	JUC01029093.hg.1	TRMT1L
TC01006288.hg.1	3.62	PSR01003380.hg.1	CLCNKB

TC01006288.hg.1	2.64	JUC01039312.hg.1	CLCNKB
TC01006288.hg.1	-2.64	JUC01039296.hg.1	CLCNKB
TC02000078.hg.1	3.62	JUC02000555.hg.1	LPIN1
TC02000078.hg.1	3.53	PSR02000954.hg.1	LPIN1
TC02000078.hg.1	2.98	PSR02000950.hg.1	LPIN1
TC02000078.hg.1	2.78	PSR02001001.hg.1	LPIN1
TC02000078.hg.1	2.68	PSR02000969.hg.1	LPIN1
TC02000078.hg.1	2.64	PSR02000976.hg.1	LPIN1
TC02000078.hg.1	2.64	PSR02000987.hg.1	LPIN1
TC02000078.hg.1	2.63	PSR02000951.hg.1	LPIN1
TC02000078.hg.1	2.62	PSR02000952.hg.1	LPIN1
TC02000078.hg.1	2.6	PSR02000955.hg.1	LPIN1
TC02000078.hg.1	2.54	JUC02000533.hg.1	LPIN1
TC02000078.hg.1	2.46	JUC02000530.hg.1	LPIN1
TC02000078.hg.1	2.41	PSR02000960.hg.1	LPIN1
TC02000078.hg.1	2.39	PSR02000985.hg.1	LPIN1
TC02000078.hg.1	2.37	PSR02000961.hg.1	LPIN1
TC02000078.hg.1	2.34	PSR02000963.hg.1	LPIN1
TC02000078.hg.1	2.15	PSR02000990.hg.1	LPIN1
TC02000078.hg.1	2.09	PSR02000996.hg.1	LPIN1
TC02000078.hg.1	2.07	PSR02000944.hg.1	LPIN1
TC02000078.hg.1	2.02	PSR02000973.hg.1	LPIN1
TC02000078.hg.1	2.02	JUC02000561.hg.1	LPIN1
TC02000078.hg.1	-2.07	JUC02000545.hg.1	LPIN1
TC02000078.hg.1	-2.38	JUC02000536.hg.1	LPIN1
TC02000078.hg.1	-2.52	JUC02000528.hg.1	LPIN1
TC02000078.hg.1	-11.68	JUC02000563.hg.1	LPIN1
TC04000041.hg.1	3.62	JUC04000431.hg.1	GRK4
TC04000041.hg.1	-2.1	PSR04001077.hg.1	GRK4
TC15001761.hg.1	3.62	JUC15009439.hg.1	HOMER2
TC15001761.hg.1	2.72	JUC15009430.hg.1	HOMER2
TC15001761.hg.1	2.38	JUC15009435.hg.1	HOMER2
TC15001761.hg.1	-2.48	JUC15009433.hg.1	HOMER2
TC15001761.hg.1	-2.57	PSR15017264.hg.1	HOMER2
TC15001761.hg.1	-2.58	JUC15009437.hg.1	HOMER2
TC15001761.hg.1	-3.22	JUC15009438.hg.1	HOMER2
TC15001761.hg.1	-3.73	PSR15017266.hg.1	HOMER2
TC15001761.hg.1	-4.16	PSR15017269.hg.1	HOMER2
TC15001761.hg.1	-5.07	PSR15017273.hg.1	HOMER2
TC01001173.hg.1	3.61	JUC01009904.hg.1	HIST2H4B, H
TC01001173.hg.1	3.42	PSR01017984.hg.1	HIST2H4B, H
TC01001173.hg.1	2.25	PSR01017987.hg.1	HIST2H4B, H
TC01002293.hg.1	3.61	PSR01035071.hg.1	UBR4
TC01002293.hg.1	2.87	PSR01034993.hg.1	UBR4
TC01002293.hg.1	2.62	JUC01018814.hg.1	UBR4
TC01002293.hg.1	2.54	PSR01035048.hg.1	UBR4
TC01002293.hg.1	2.54	JUC01018846.hg.1	UBR4
TC01002293.hg.1	2.52	PSR01035039.hg.1	UBR4
TC01002293.hg.1	2.43	PSR01034981.hg.1	UBR4
TC01002293.hg.1	2.41	PSR01035050.hg.1	UBR4
TC01002293.hg.1	2.39	PSR01034972.hg.1	UBR4
TC01002293.hg.1	2.38	JUC01018776.hg.1	UBR4
TC01002293.hg.1	2.29	PSR01035082.hg.1	UBR4
TC01002293.hg.1	2.23	PSR01035025.hg.1	UBR4
TC01002293.hg.1	2.16	JUC01018838.hg.1	UBR4
TC01002293.hg.1	2.15	PSR01035073.hg.1	UBR4
TC01002293.hg.1	2.13	PSR01035002.hg.1	UBR4
TC01002293.hg.1	2.13	PSR01035027.hg.1	UBR4

TC01002293.hg.1	2.12	PSR01035015.hg.1	UBR4
TC01002293.hg.1	2.1	JUC01018788.hg.1	UBR4
TC01002293.hg.1	2.06	PSR01034986.hg.1	UBR4
TC03001815.hg.1	3.61	JUC03016310.hg.1	LOC1005072
TC03001815.hg.1	2.65	PSR03032883.hg.1	LOC1005072
TC05001109.hg.1	3.61	JUC05008208.hg.1	SLC6A3
TC05001109.hg.1	2.04	PSR05016152.hg.1	SLC6A3
TC05001109.hg.1	-2.05	JUC05008216.hg.1	SLC6A3
TC07000696.hg.1	3.61	JUC07005156.hg.1	CBLL1, LOC1
TC07000696.hg.1	2.62	PSR07010934.hg.1	CBLL1, LOC1
TC07000696.hg.1	2.07	JUC07005142.hg.1	CBLL1, LOC1
TC0X000112.hg.1	3.61	JUC0X000797.hg.1	SAT1
TC0X000112.hg.1	2.83	JUC0X000796.hg.1	SAT1
TC0X000112.hg.1	-2.04	PSR0X001474.hg.1	SAT1
TC0X000112.hg.1	-2.22	PSR0X001476.hg.1	SAT1
TC0X000641.hg.1	3.61	JUC0X004247.hg.1	FAM122C
TC0X000641.hg.1	2.56	JUC0X004256.hg.1	FAM122C
TC0X000641.hg.1	2.33	PSR0X008429.hg.1	FAM122C
TC0X000641.hg.1	2.16	PSR0X008428.hg.1	FAM122C
TC0X000641.hg.1	2.06	JUC0X004248.hg.1	FAM122C
TC13000109.hg.1	3.61	JUC13000687.hg.1	FRY
TC13000109.hg.1	2.73	PSR13001137.hg.1	FRY
TC13000109.hg.1	-2.22	PSR13001074.hg.1	FRY
TC13000109.hg.1	-2.26	PSR13001131.hg.1	FRY
TC13000109.hg.1	-2.44	JUC13000705.hg.1	FRY
TC13000109.hg.1	-2.49	JUC13000701.hg.1	FRY
TC13000109.hg.1	-2.53	PSR13001065.hg.1	FRY
TC13000109.hg.1	-2.55	JUC13000686.hg.1	FRY
TC13000109.hg.1	-3.24	JUC13000690.hg.1	FRY
TC13000109.hg.1	-3.44	JUC13000679.hg.1	FRY
TC13000109.hg.1	-3.51	JUC13000667.hg.1	FRY
TC16000056.hg.1	3.61	JUC16000499.hg.1	SYNGR3
TC16000056.hg.1	-4.54	PSR16001180.hg.1	SYNGR3
TC02001246.hg.1	3.6	JUC02010370.hg.1	CPS1
TC02001246.hg.1	3.04	PSR02019231.hg.1	CPS1
TC02001246.hg.1	2.54	JUC02010347.hg.1	CPS1
TC02001246.hg.1	2.14	JUC02010330.hg.1	CPS1
TC02001246.hg.1	2.11	PSR02019194.hg.1	CPS1
TC02001246.hg.1	-2.27	JUC02010369.hg.1	CPS1
TC02001246.hg.1	-2.36	JUC02010327.hg.1	CPS1
TC04000948.hg.1	3.6	PSR04012752.hg.1	ZNF721, ABC
TC04000948.hg.1	2.36	JUC04006811.hg.1	ZNF721, ABC
TC04000948.hg.1	2.23	PSR04012748.hg.1	ZNF721, ABC
TC04000948.hg.1	2.15	PSR04012754.hg.1	ZNF721, ABC
TC08001286.hg.1	3.6	PSR08016562.hg.1	MYBL1
TC08001286.hg.1	2.93	PSR08016535.hg.1	MYBL1
TC08001286.hg.1	2.82	PSR08016558.hg.1	MYBL1
TC08001286.hg.1	2.81	PSR08016554.hg.1	MYBL1
TC08001286.hg.1	2.72	PSR08016560.hg.1	MYBL1
TC08001286.hg.1	2.58	JUC08008530.hg.1	MYBL1
TC08001286.hg.1	-2.34	JUC08008535.hg.1	MYBL1
TC16000445.hg.1	3.6	JUC16003456.hg.1	CHD9
TC16000445.hg.1	2.08	JUC16003431.hg.1	CHD9
TC16000445.hg.1	-2.49	PSR16006170.hg.1	CHD9
TC16000445.hg.1	-2.51	JUC16003451.hg.1	CHD9
TC16000445.hg.1	-2.9	JUC16003447.hg.1	CHD9
TC16000445.hg.1	-3.66	JUC16003442.hg.1	CHD9
TC16000445.hg.1	-4.84	JUC16003458.hg.1	CHD9

TC01002639.hg.1	3.59	JUC01022070.hg.1	BEND5
TC01002639.hg.1	2.7	PSR01041291.hg.1	BEND5
TC01002639.hg.1	2.19	PSR01041295.hg.1	BEND5
TC01002639.hg.1	2.12	PSR01041303.hg.1	BEND5
TC01002639.hg.1	-2.54	JUC01022065.hg.1	BEND5
TC01002639.hg.1	-3.02	PSR01041293.hg.1	BEND5
TC01002883.hg.1	3.59	JUC01024177.hg.1	ABCA4
TC01002883.hg.1	3.33	JUC01024141.hg.1	ABCA4
TC01002883.hg.1	3.29	JUC01024187.hg.1	ABCA4
TC01002883.hg.1	3.25	PSR01045022.hg.1	ABCA4
TC01002883.hg.1	2.96	JUC01024174.hg.1	ABCA4
TC01002883.hg.1	2.79	PSR01045035.hg.1	ABCA4
TC01002883.hg.1	2.69	PSR01044989.hg.1	ABCA4
TC01002883.hg.1	2.62	PSR01045029.hg.1	ABCA4
TC01002883.hg.1	2.58	PSR01045017.hg.1	ABCA4
TC01002883.hg.1	2.58	JUC01024142.hg.1	ABCA4
TC01002883.hg.1	2.58	JUC01024191.hg.1	ABCA4
TC01002883.hg.1	2.55	PSR01044990.hg.1	ABCA4
TC01002883.hg.1	2.46	JUC01024175.hg.1	ABCA4
TC01002883.hg.1	2.45	PSR01045024.hg.1	ABCA4
TC01002883.hg.1	2.43	PSR01044985.hg.1	ABCA4
TC01002883.hg.1	2.42	JUC01024144.hg.1	ABCA4
TC01002883.hg.1	2.31	PSR01044973.hg.1	ABCA4
TC01002883.hg.1	2.16	PSR01045033.hg.1	ABCA4
TC01002883.hg.1	2.14	JUC01024152.hg.1	ABCA4
TC01002883.hg.1	2.12	PSR01045020.hg.1	ABCA4
TC01002883.hg.1	2.12	JUC01024150.hg.1	ABCA4
TC01002883.hg.1	2.09	PSR01044998.hg.1	ABCA4
TC01002883.hg.1	2.09	JUC01024192.hg.1	ABCA4
TC01002883.hg.1	-2.05	PSR01045003.hg.1	ABCA4
TC01002883.hg.1	-2.08	PSR01044996.hg.1	ABCA4
TC01002883.hg.1	-2.08	JUC01024146.hg.1	ABCA4
TC01002883.hg.1	-2.24	JUC01024185.hg.1	ABCA4
TC01002883.hg.1	-2.36	JUC01024181.hg.1	ABCA4
TC01002883.hg.1	-2.53	PSR01045014.hg.1	ABCA4
TC01002883.hg.1	-2.72	JUC01024164.hg.1	ABCA4
TC01002883.hg.1	-2.97	PSR01044981.hg.1	ABCA4
TC01002883.hg.1	-3.2	JUC01024193.hg.1	ABCA4
TC01002883.hg.1	-3.53	JUC01024145.hg.1	ABCA4
TC01002883.hg.1	-3.83	PSR01045005.hg.1	ABCA4
TC01002883.hg.1	-4.06	PSR01045015.hg.1	ABCA4
TC01002883.hg.1	-5.89	JUC01024178.hg.1	ABCA4
TC01002883.hg.1	-6.41	PSR01045000.hg.1	ABCA4
TC01002883.hg.1	-6.91	JUC01024197.hg.1	ABCA4
TC01002883.hg.1	-7.41	PSR01045018.hg.1	ABCA4
TC01002883.hg.1	-11.98	JUC01024182.hg.1	ABCA4
TC07001363.hg.1	3.59	JUC07010388.hg.1	TNS3
TC07001363.hg.1	3.4	JUC07010417.hg.1	TNS3
TC07001363.hg.1	2.51	PSR07021314.hg.1	TNS3
TC07001363.hg.1	2.46	JUC07010401.hg.1	TNS3
TC07001363.hg.1	-2.6	JUC07010383.hg.1	TNS3
TC11001217.hg.1	3.59	JUC11008285.hg.1	GLB1L2
TC11001217.hg.1	2.5	JUC11008267.hg.1	GLB1L2
TC11001217.hg.1	2.37	JUC11008274.hg.1	GLB1L2
TC11001217.hg.1	2.17	PSR11015286.hg.1	GLB1L2
TC11001217.hg.1	-2.82	JUC11008288.hg.1	GLB1L2
TC14002301.hg.1	3.59	JUC14011754.hg.1	PTGR2
TC14002301.hg.1	2.24	PSR14005548.hg.1	PTGR2

TC14002301.hg.1	2.16	PSR14005567.hg.1	PTGR2
TC15001668.hg.1	3.59	PSR15016276.hg.1	SNUPN
TC15001668.hg.1	3.19	PSR15016280.hg.1	SNUPN
TC15001668.hg.1	3.13	PSR15016277.hg.1	SNUPN
TC15001668.hg.1	2.82	JUC15008844.hg.1	SNUPN
TC15001668.hg.1	2.45	PSR15016273.hg.1	SNUPN
TC15001668.hg.1	2.15	JUC15008843.hg.1	SNUPN
TC19001104.hg.1	3.59	JUC19008640.hg.1	GPR108
TC19001104.hg.1	-2.01	PSR19015212.hg.1	GPR108
TC20000169.hg.1	3.59	JUC20001144.hg.1	GINS1
TC20000169.hg.1	2.05	PSR20002126.hg.1	GINS1
TC20000169.hg.1	2.02	PSR20002131.hg.1	GINS1
TC04000320.hg.1	3.58	JUC04002207.hg.1	TMEM165
TC04000320.hg.1	-2.04	JUC04002196.hg.1	TMEM165
TC04000320.hg.1	-2.09	JUC04002195.hg.1	TMEM165
TC04000320.hg.1	-2.15	PSR04004509.hg.1	TMEM165
TC04000320.hg.1	-2.25	PSR04004513.hg.1	TMEM165
TC04000320.hg.1	-2.38	PSR04004511.hg.1	TMEM165
TC07000764.hg.1	3.58	JUC07005694.hg.1	SND1, SND1-
TC07000764.hg.1	2.44	PSR07012103.hg.1	SND1, SND1-
TC07000764.hg.1	2.34	JUC07005710.hg.1	SND1, SND1-
TC07000764.hg.1	2.06	JUC07005701.hg.1	SND1, SND1-
TC15001278.hg.1	3.58	JUC15006477.hg.1	TP53BP1
TC15001278.hg.1	2.6	PSR15011884.hg.1	TP53BP1
TC15001278.hg.1	-2	JUC15006471.hg.1	TP53BP1
TC15001278.hg.1	-2.03	JUC15006468.hg.1	TP53BP1
TC15001278.hg.1	-2.34	JUC15006462.hg.1	TP53BP1
TC15001278.hg.1	-2.44	JUC15006454.hg.1	TP53BP1
TC15001278.hg.1	-2.51	PSR15011903.hg.1	TP53BP1
TC15001278.hg.1	-2.98	PSR15011893.hg.1	TP53BP1
TC19001625.hg.1	3.58	JUC19012672.hg.1	EML2
TC19001625.hg.1	2.23	JUC19012658.hg.1	EML2
TC19001625.hg.1	-2.1	PSR19022221.hg.1	EML2
TC19001625.hg.1	-2.17	JUC19012679.hg.1	EML2
TC21000239.hg.1	3.58	JUC21001448.hg.1	LOC1005057
TC21000239.hg.1	3.13	PSR21002801.hg.1	LOC1005057
TC01000950.hg.1	3.57	PSR01015009.hg.1	AMPD2
TC01000950.hg.1	2.97	JUC01007929.hg.1	AMPD2
TC01000950.hg.1	2.93	JUC01007931.hg.1	AMPD2
TC01000950.hg.1	2.9	JUC01007943.hg.1	AMPD2
TC01000950.hg.1	2.83	PSR01015016.hg.1	AMPD2
TC01000950.hg.1	2.83	JUC01007924.hg.1	AMPD2
TC01000950.hg.1	2.75	PSR01014998.hg.1	AMPD2
TC01000950.hg.1	2.6	JUC01007921.hg.1	AMPD2
TC01000950.hg.1	2.32	PSR01015029.hg.1	AMPD2
TC01000950.hg.1	2.21	PSR01015011.hg.1	AMPD2
TC01000950.hg.1	2.14	PSR01015006.hg.1	AMPD2
TC01000950.hg.1	2.13	PSR01015039.hg.1	AMPD2
TC01000950.hg.1	2.12	PSR01015014.hg.1	AMPD2
TC01000950.hg.1	2.07	PSR01015007.hg.1	AMPD2
TC01000950.hg.1	-2.18	JUC01007939.hg.1	AMPD2
TC06002025.hg.1	3.57	JUC06012499.hg.1	LAMA4
TC06002025.hg.1	2.87	PSR06025430.hg.1	LAMA4
TC06002025.hg.1	2.85	PSR06025418.hg.1	LAMA4
TC06002025.hg.1	2.77	PSR06025379.hg.1	LAMA4
TC06002025.hg.1	2.42	PSR06025402.hg.1	LAMA4
TC06002025.hg.1	2.28	PSR06025427.hg.1	LAMA4
TC06002025.hg.1	2.16	PSR06025425.hg.1	LAMA4

TC06002025.hg.1	-2.01	PSR06025374.hg.1	LAMA4
TC06002025.hg.1	-2.02	PSR06025351.hg.1	LAMA4
TC06002025.hg.1	-2.06	PSR06025397.hg.1	LAMA4
TC06002025.hg.1	-2.1	PSR06025354.hg.1	LAMA4
TC06002025.hg.1	-2.1	PSR06025393.hg.1	LAMA4
TC06002025.hg.1	-2.1	JUC06012508.hg.1	LAMA4
TC06002025.hg.1	-2.13	PSR06025408.hg.1	LAMA4
TC06002025.hg.1	-2.14	PSR06025355.hg.1	LAMA4
TC06002025.hg.1	-2.19	JUC06012507.hg.1	LAMA4
TC06002025.hg.1	-2.2	JUC06012535.hg.1	LAMA4
TC06002025.hg.1	-2.25	JUC06012520.hg.1	LAMA4
TC06002025.hg.1	-2.26	JUC06012543.hg.1	LAMA4
TC06002025.hg.1	-2.3	PSR06025347.hg.1	LAMA4
TC06002025.hg.1	-2.33	JUC06012512.hg.1	LAMA4
TC06002025.hg.1	-2.41	JUC06012542.hg.1	LAMA4
TC06002025.hg.1	-2.48	PSR06025383.hg.1	LAMA4
TC06002025.hg.1	-2.64	JUC06012522.hg.1	LAMA4
TC06002025.hg.1	-2.99	PSR06025384.hg.1	LAMA4
TC06002025.hg.1	-3.13	PSR06025382.hg.1	LAMA4
TC06002025.hg.1	-3.38	PSR06025380.hg.1	LAMA4
TC06002025.hg.1	-3.74	JUC06012504.hg.1	LAMA4
TC06002025.hg.1	-3.89	JUC06012502.hg.1	LAMA4
TC07000190.hg.1	3.57	JUC07001400.hg.1	
TC07000190.hg.1	3.47	JUC07001401.hg.1	
TC07000190.hg.1	2.45	PSR07002950.hg.1	
TC0X000387.hg.1	3.57	JUC0X002609.hg.1	TAF1
TC0X000387.hg.1	3.36	JUC0X002602.hg.1	TAF1
TC0X000387.hg.1	2.31	PSR0X005201.hg.1	TAF1
TC0X000387.hg.1	2.22	JUC0X002566.hg.1	TAF1
TC0X000387.hg.1	2.21	JUC0X002557.hg.1	TAF1
TC0X000387.hg.1	2.14	PSR0X005199.hg.1	TAF1
TC0X000387.hg.1	2.1	JUC0X002575.hg.1	TAF1
TC0X000387.hg.1	2.04	PSR0X005205.hg.1	TAF1
TC0X000387.hg.1	2	JUC0X002589.hg.1	TAF1
TC11001911.hg.1	3.57	JUC11011946.hg.1	SF1
TC11001911.hg.1	2.18	PSR11022684.hg.1	SF1
TC11001911.hg.1	-4.37	JUC11011948.hg.1	SF1
TC15001092.hg.1	3.57	JUC15005281.hg.1	HERC2, LOC
TC15001092.hg.1	2.21	JUC15005256.hg.1	HERC2, LOC
TC15001092.hg.1	-2.01	JUC15005221.hg.1	HERC2, LOC
TC15001092.hg.1	-2.14	PSR15009918.hg.1	HERC2, LOC
TC15001092.hg.1	-2.19	JUC15005234.hg.1	HERC2, LOC
TC15001092.hg.1	-2.34	JUC15005248.hg.1	HERC2, LOC
TC15001092.hg.1	-2.75	JUC15005232.hg.1	HERC2, LOC
TC19000755.hg.1	3.57	JUC19006271.hg.1	MYH14
TC19000755.hg.1	3.17	JUC19006259.hg.1	MYH14
TC19000755.hg.1	-2.16	JUC19006268.hg.1	MYH14
TC19000755.hg.1	-2.2	JUC19006234.hg.1	MYH14
TC19000755.hg.1	-2.43	JUC19006282.hg.1	MYH14
TC19000755.hg.1	-2.51	JUC19006244.hg.1	MYH14
TC19000755.hg.1	-2.99	JUC19006235.hg.1	MYH14
TC19000755.hg.1	-3.5	JUC19006243.hg.1	MYH14
TC19000755.hg.1	-4.02	JUC19006264.hg.1	MYH14
TC19000755.hg.1	-4.24	PSR19010685.hg.1	MYH14
TC19000755.hg.1	-4.75	JUC19006251.hg.1	MYH14
TC19000755.hg.1	-7.66	JUC19006278.hg.1	MYH14
TC03000611.hg.1	3.56	JUC03005490.hg.1	PLA1A
TC03000611.hg.1	2.64	JUC03005496.hg.1	PLA1A

TC03000611.hg.1	-2.03	PSR03011039.hg.1	PLA1A
TC09001466.hg.1	3.56	JUC09009788.hg.1	EPB41L4B
TC09001466.hg.1	3.55	PSR09018117.hg.1	EPB41L4B
TC09001466.hg.1	3.52	PSR09018131.hg.1	EPB41L4B
TC09001466.hg.1	3.5	PSR09018112.hg.1	EPB41L4B
TC09001466.hg.1	3.41	JUC09009784.hg.1	EPB41L4B
TC09001466.hg.1	3.37	PSR09018100.hg.1	EPB41L4B
TC09001466.hg.1	3.2	PSR09018115.hg.1	EPB41L4B
TC09001466.hg.1	3.15	PSR09018130.hg.1	EPB41L4B
TC09001466.hg.1	2.92	PSR09018106.hg.1	EPB41L4B
TC09001466.hg.1	2.79	JUC09009768.hg.1	EPB41L4B
TC09001466.hg.1	2.73	PSR09018113.hg.1	EPB41L4B
TC09001466.hg.1	2.67	PSR09018102.hg.1	EPB41L4B
TC09001466.hg.1	2.62	PSR09018108.hg.1	EPB41L4B
TC09001466.hg.1	2.52	JUC09009782.hg.1	EPB41L4B
TC09001466.hg.1	2.47	PSR09018103.hg.1	EPB41L4B
TC09001466.hg.1	2.3	PSR09018111.hg.1	EPB41L4B
TC09001466.hg.1	2.29	JUC09009764.hg.1	EPB41L4B
TC09001466.hg.1	2.24	PSR09018110.hg.1	EPB41L4B
TC09001466.hg.1	2.05	PSR09018099.hg.1	EPB41L4B
TC09001466.hg.1	2	JUC09009785.hg.1	EPB41L4B
TC09001466.hg.1	-2.3	PSR09018127.hg.1	EPB41L4B
TC09001466.hg.1	-3.31	PSR09018116.hg.1	EPB41L4B
TC09001466.hg.1	-3.38	PSR09018122.hg.1	EPB41L4B
TC09001466.hg.1	-4.5	PSR09018118.hg.1	EPB41L4B
TC10000112.hg.1	3.56	JUC10000639.hg.1	HSPA14
TC10000112.hg.1	2.74	JUC10000654.hg.1	HSPA14
TC10000112.hg.1	2.63	JUC10000650.hg.1	HSPA14
TC10000112.hg.1	2.49	PSR10001192.hg.1	HSPA14
TC10000112.hg.1	-2.03	PSR10001209.hg.1	HSPA14
TC12001106.hg.1	3.56	JUC12008016.hg.1	FOXM1
TC12001106.hg.1	2.74	JUC12008022.hg.1	FOXM1
TC12001106.hg.1	2.33	JUC12008009.hg.1	FOXM1
TC12001106.hg.1	2.27	JUC12008017.hg.1	FOXM1
TC12001106.hg.1	2.21	JUC12008015.hg.1	FOXM1
TC12001106.hg.1	2.19	JUC12008020.hg.1	FOXM1
TC12001106.hg.1	-3.05	PSR12014592.hg.1	FOXM1
TC02001121.hg.1	3.54	PSR02016956.hg.1	NAB1
TC02001121.hg.1	3.05	JUC02009088.hg.1	NAB1
TC0X001405.hg.1	3.54	JUC0X009554.hg.1	ZNF75D
TC0X001405.hg.1	2.64	PSR0X018699.hg.1	ZNF75D
TC0X001405.hg.1	2.53	JUC0X009556.hg.1	ZNF75D
TC0X001405.hg.1	2.01	PSR0X018690.hg.1	ZNF75D
TC0X001405.hg.1	-2.07	PSR0X018696.hg.1	ZNF75D
TC0X001405.hg.1	-2.15	JUC0X009555.hg.1	ZNF75D
TC0X001405.hg.1	-2.7	JUC0X009551.hg.1	ZNF75D
TC0X001405.hg.1	-3.02	JUC0X009550.hg.1	ZNF75D
TC0X002313.hg.1	3.54	JUC0X012948.hg.1	CA5B
TC0X002313.hg.1	3.29	JUC0X012949.hg.1	CA5B
TC0X002313.hg.1	2.89	JUC0X012960.hg.1	CA5B
TC0X002313.hg.1	2.54	PSR0X000991.hg.1	CA5B
TC0X002313.hg.1	2.13	PSR0X000975.hg.1	CA5B
TC0X002313.hg.1	2.11	JUC0X012950.hg.1	CA5B
TC0X002313.hg.1	2.07	PSR0X000974.hg.1	CA5B
TC0X002313.hg.1	-2.07	PSR0X000986.hg.1	CA5B
TC0X002313.hg.1	-2.3	JUC0X012955.hg.1	CA5B
TC0X002313.hg.1	-2.38	JUC0X012951.hg.1	CA5B
TC0X002313.hg.1	-2.41	JUC0X012958.hg.1	CA5B

TC0X002313.hg.1	-3.83	JUC0X012956.hg.1	CA5B
TC0X002313.hg.1	-4.46	PSR0X000967.hg.1	CA5B
TC11000979.hg.1	3.54	JUC11006519.hg.1	ACAT1
TC11000979.hg.1	2.01	PSR11012158.hg.1	ACAT1
TC13000135.hg.1	3.54	JUC13000973.hg.1	NHLRC3
TC13000135.hg.1	-2.51	JUC13000964.hg.1	NHLRC3
TC13000135.hg.1	-2.71	PSR13001565.hg.1	NHLRC3
TC15000638.hg.1	3.54	JUC15002703.hg.1	KIF23
TC15000638.hg.1	2.79	PSR15005401.hg.1	KIF23
TC15000638.hg.1	2.59	JUC15002717.hg.1	KIF23
TC15000638.hg.1	2.39	JUC15002711.hg.1	KIF23
TC15000638.hg.1	2.21	PSR15005406.hg.1	KIF23
TC15000638.hg.1	2.19	PSR15005408.hg.1	KIF23
TC15000638.hg.1	2	PSR15005430.hg.1	KIF23
TC03000631.hg.1	3.53	PSR03011364.hg.1	FAM162A
TC03000631.hg.1	3.5	PSR03011362.hg.1	FAM162A
TC03000631.hg.1	3	JUC03005673.hg.1	FAM162A
TC03000631.hg.1	2.61	JUC03005672.hg.1	FAM162A
TC03000631.hg.1	2.51	JUC03005669.hg.1	FAM162A
TC03000631.hg.1	2.01	PSR03011378.hg.1	FAM162A
TC03000316.hg.1	3.52	PSR03006657.hg.1	MAPKAPK3
TC03000316.hg.1	3.21	PSR03006660.hg.1	MAPKAPK3
TC03000316.hg.1	3.19	PSR03006662.hg.1	MAPKAPK3
TC03000316.hg.1	3.06	PSR03006658.hg.1	MAPKAPK3
TC03000316.hg.1	2.86	PSR03006654.hg.1	MAPKAPK3
TC03000316.hg.1	2.85	PSR03006659.hg.1	MAPKAPK3
TC03000316.hg.1	2.72	PSR03006668.hg.1	MAPKAPK3
TC03000316.hg.1	2.71	PSR03006666.hg.1	MAPKAPK3
TC03000316.hg.1	2.16	JUC03003247.hg.1	MAPKAPK3
TC08000053.hg.1	3.52	JUC08000281.hg.1	FAM66E
TC08000053.hg.1	2.09	PSR08000488.hg.1	FAM66E
TC08000053.hg.1	-2.15	PSR08000491.hg.1	FAM66E
TC08000053.hg.1	-2.25	JUC08000277.hg.1	FAM66E
TC08000053.hg.1	-2.73	PSR08000497.hg.1	FAM66E
TC0X000811.hg.1	3.52	JUC0X005440.hg.1	LOC389906
TC0X000811.hg.1	3.18	PSR0X011113.hg.1	LOC389906
TC0X000811.hg.1	3	PSR0X011119.hg.1	LOC389906
TC0X000811.hg.1	2.89	PSR0X011126.hg.1	LOC389906
TC0X000811.hg.1	2.34	PSR0X011127.hg.1	LOC389906
TC0X000811.hg.1	2.31	PSR0X011110.hg.1	LOC389906
TC0X000811.hg.1	2.27	JUC0X005447.hg.1	LOC389906
TC0X000811.hg.1	2.23	JUC0X005438.hg.1	LOC389906
TC0X000811.hg.1	2.21	PSR0X011125.hg.1	LOC389906
TC0X000811.hg.1	2.01	PSR0X011120.hg.1	LOC389906
TC0X000811.hg.1	2	JUC0X005434.hg.1	LOC389906
TC11002138.hg.1	3.52	PSR11025584.hg.1	NARS2
TC11002138.hg.1	2.34	JUC11013559.hg.1	NARS2
TC11002138.hg.1	2.06	PSR11025581.hg.1	NARS2
TC11002138.hg.1	2.05	JUC11013554.hg.1	NARS2
TC01000548.hg.1	3.51	JUC01004591.hg.1	PTPRF
TC01000548.hg.1	2.81	JUC01004576.hg.1	PTPRF
TC01000548.hg.1	2.49	PSR01008812.hg.1	PTPRF
TC01000548.hg.1	2.42	JUC01004584.hg.1	PTPRF
TC01000548.hg.1	2.25	JUC01004580.hg.1	PTPRF
TC01000548.hg.1	2.24	PSR01008842.hg.1	PTPRF
TC01000548.hg.1	2.08	PSR01008826.hg.1	PTPRF
TC01000548.hg.1	2.08	PSR01008841.hg.1	PTPRF
TC01000548.hg.1	2.02	PSR01008821.hg.1	PTPRF

TC01000548.hg.1	-2.13	JUC01004593.hg.1	PTPRF
TC01003325.hg.1	3.51	JUC01027093.hg.1	KIAA0907
TC01003325.hg.1	2.18	PSR01051719.hg.1	KIAA0907
TC01003325.hg.1	2.12	PSR01051709.hg.1	KIAA0907
TC03003371.hg.1	3.51	PSR03019262.hg.1	FAM157A, LC
TC03003371.hg.1	2.83	PSR03019263.hg.1	FAM157A, LC
TC03003371.hg.1	2.57	PSR03019264.hg.1	FAM157A, LC
TC03003371.hg.1	2.39	JUC03023974.hg.1	FAM157A, LC
TC03003371.hg.1	2.38	PSR03019265.hg.1	FAM157A, LC
TC03003371.hg.1	-2.17	JUC03023965.hg.1	FAM157A, LC
TC03003371.hg.1	-2.4	PSR03019267.hg.1	FAM157A, LC
TC06002181.hg.1	3.51	JUC06013617.hg.1	HIVEP2
TC06002181.hg.1	2.05	PSR06027366.hg.1	HIVEP2
TC07003289.hg.1	3.51	JUC07020312.hg.1	ZNF655
TC07003289.hg.1	2.07	JUC07020324.hg.1	ZNF655
TC07003289.hg.1	-2.69	PSR07008928.hg.1	ZNF655
TC08000148.hg.1	3.51	JUC08000778.hg.1	INTS10
TC08000148.hg.1	2.65	JUC08000789.hg.1	INTS10
TC08000148.hg.1	2.6	JUC08000795.hg.1	INTS10
TC08000148.hg.1	2.13	PSR08001480.hg.1	INTS10
TC08000148.hg.1	2.03	PSR08001439.hg.1	INTS10
TC10001498.hg.1	3.51	PSR10018941.hg.1	ACTA2
TC10001498.hg.1	2.76	JUC10011034.hg.1	ACTA2
TC10001498.hg.1	2.4	JUC10011040.hg.1	ACTA2
TC10001498.hg.1	2.35	PSR10018933.hg.1	ACTA2
TC10001498.hg.1	-2.82	JUC10011035.hg.1	ACTA2
TC10001498.hg.1	-2.85	JUC10011038.hg.1	ACTA2
TC10001498.hg.1	-2.88	PSR10018953.hg.1	ACTA2
TC10001498.hg.1	-4.34	PSR10018952.hg.1	ACTA2
TC10001498.hg.1	-4.39	JUC10011041.hg.1	ACTA2
TC10001498.hg.1	-5	PSR10018951.hg.1	ACTA2
TC11000069.hg.1	3.51	PSR11001307.hg.1	SLC22A18
TC11000069.hg.1	3.07	PSR11001299.hg.1	SLC22A18
TC11000069.hg.1	2.86	JUC11000652.hg.1	SLC22A18
TC11000069.hg.1	2.67	PSR11001304.hg.1	SLC22A18
TC11000069.hg.1	2.47	PSR11001308.hg.1	SLC22A18
TC11000069.hg.1	2.44	PSR11001320.hg.1	SLC22A18
TC11000069.hg.1	2.36	PSR11001305.hg.1	SLC22A18
TC11000069.hg.1	2.35	JUC11000643.hg.1	SLC22A18
TC11000069.hg.1	2.3	JUC11000647.hg.1	SLC22A18
TC11000069.hg.1	2.16	PSR11001330.hg.1	SLC22A18
TC11000069.hg.1	2.15	PSR11001301.hg.1	SLC22A18
TC11000069.hg.1	2.12	PSR11001328.hg.1	SLC22A18
TC11000069.hg.1	2.09	PSR11001316.hg.1	SLC22A18
TC11000069.hg.1	2.03	JUC11000657.hg.1	SLC22A18
TC12000232.hg.1	3.51	JUC12001383.hg.1	PYROXD1
TC12000232.hg.1	-2.22	PSR12002876.hg.1	PYROXD1
TC16002044.hg.1	3.51	PSR16005776.hg.1	TP53TG3B, T
TC16002044.hg.1	2.99	JUC16013322.hg.1	TP53TG3B, T
TC16002044.hg.1	2.91	JUC16013324.hg.1	TP53TG3B, T
TC16002044.hg.1	2.73	PSR16005780.hg.1	TP53TG3B, T
TC16002044.hg.1	2.56	PSR16005779.hg.1	TP53TG3B, T
TC16002044.hg.1	2.2	PSR16005781.hg.1	TP53TG3B, T
TC20000338.hg.1	3.51	JUC20002672.hg.1	PABPC1L
TC20000338.hg.1	-2.64	PSR20004848.hg.1	PABPC1L
TC22000068.hg.1	3.51	JUC22000434.hg.1	MED15
TC22000068.hg.1	2.19	PSR22000887.hg.1	MED15
TC22000068.hg.1	2	PSR22000947.hg.1	MED15

TC22000068.hg.1	2	JUC22000399.hg.1	MED15
TC22000068.hg.1	-2.22	JUC22000395.hg.1	MED15
TC01003219.hg.1	3.5	JUC01026303.hg.1	SEMA6C
TC01003219.hg.1	2.5	JUC01026301.hg.1	SEMA6C
TC01003219.hg.1	2.27	JUC01026306.hg.1	SEMA6C
TC01003219.hg.1	-2	JUC01026292.hg.1	SEMA6C
TC01003219.hg.1	-2.05	PSR01049493.hg.1	SEMA6C
TC01003219.hg.1	-2.39	PSR01049500.hg.1	SEMA6C
TC01003219.hg.1	-2.4	JUC01026310.hg.1	SEMA6C
TC01003219.hg.1	-2.94	JUC01026295.hg.1	SEMA6C
TC01003219.hg.1	-3	JUC01026293.hg.1	SEMA6C
TC01006303.hg.1	3.5	JUC01039692.hg.1	GBAP1
TC01006303.hg.1	3.14	JUC01039685.hg.1	GBAP1
TC01006303.hg.1	2.78	JUC01039688.hg.1	GBAP1
TC01006303.hg.1	2.11	PSR01051231.hg.1	GBAP1
TC01006303.hg.1	-2.04	JUC01039681.hg.1	GBAP1
TC01006303.hg.1	-4.25	JUC01039697.hg.1	GBAP1
TC10001559.hg.1	3.5	JUC10011592.hg.1	PIK3AP1
TC10001559.hg.1	2.99	JUC10011581.hg.1	PIK3AP1
TC10001559.hg.1	2.6	PSR10019791.hg.1	PIK3AP1
TC10001559.hg.1	2.51	JUC10011591.hg.1	PIK3AP1
TC10001559.hg.1	2.36	PSR10019811.hg.1	PIK3AP1
TC10001559.hg.1	2.35	PSR10019810.hg.1	PIK3AP1
TC10001559.hg.1	2.17	JUC10011596.hg.1	PIK3AP1
TC10001559.hg.1	2.16	JUC10011582.hg.1	PIK3AP1
TC10001559.hg.1	2.11	PSR10019808.hg.1	PIK3AP1
TC10001559.hg.1	2.06	PSR10019812.hg.1	PIK3AP1
TC10001559.hg.1	-2.68	JUC10011584.hg.1	PIK3AP1
TC10001559.hg.1	-3.2	PSR10019795.hg.1	PIK3AP1
TC10001559.hg.1	-3.38	PSR10019817.hg.1	PIK3AP1
TC10001559.hg.1	-3.53	JUC10011577.hg.1	PIK3AP1
TC10001559.hg.1	-3.65	PSR10019802.hg.1	PIK3AP1
TC10001559.hg.1	-3.72	PSR10019815.hg.1	PIK3AP1
TC10001559.hg.1	-4.98	PSR10019814.hg.1	PIK3AP1
TC10001559.hg.1	-5.86	PSR10019794.hg.1	PIK3AP1
TC02001965.hg.1	3.49	PSR02031339.hg.1	ADD2
TC02001965.hg.1	2.37	JUC02016369.hg.1	ADD2
TC02001965.hg.1	-2.02	JUC02016348.hg.1	ADD2
TC02001965.hg.1	-2.15	PSR02031303.hg.1	ADD2
TC06004080.hg.1	3.49	JUC06021119.hg.1	C4B, C4A, LC
TC06004080.hg.1	2.7	PSR06004435.hg.1	C4B, C4A, LC
TC06004080.hg.1	2.59	JUC06021098.hg.1	C4B, C4A, LC
TC06004080.hg.1	2.57	JUC06021111.hg.1	C4B, C4A, LC
TC06004080.hg.1	2.52	JUC06021128.hg.1	C4B, C4A, LC
TC06004080.hg.1	2.46	PSR06004417.hg.1	C4B, C4A, LC
TC06004080.hg.1	2.41	PSR06004446.hg.1	C4B, C4A, LC
TC06004080.hg.1	2.3	JUC06021120.hg.1	C4B, C4A, LC
TC06004080.hg.1	2.21	PSR06004413.hg.1	C4B, C4A, LC
TC06004080.hg.1	2.14	JUC06021122.hg.1	C4B, C4A, LC
TC06004080.hg.1	2.12	PSR06004397.hg.1	C4B, C4A, LC
TC06004080.hg.1	2.08	PSR06004449.hg.1	C4B, C4A, LC
TC06004080.hg.1	2.02	PSR06004428.hg.1	C4B, C4A, LC
TC06004080.hg.1	2	PSR06004452.hg.1	C4B, C4A, LC
TC06004080.hg.1	-2.17	JUC06021102.hg.1	C4B, C4A, LC
TC06004080.hg.1	-2.72	JUC06021108.hg.1	C4B, C4A, LC
TC06004080.hg.1	-2.72	JUC06021112.hg.1	C4B, C4A, LC
TC06004080.hg.1	-2.91	JUC06021134.hg.1	C4B, C4A, LC
TC06004080.hg.1	-3.47	JUC06021113.hg.1	C4B, C4A, LC

TC08000781.hg.1	3.49	PSR08010473.hg.1	
TC08000781.hg.1	2.08	PSR08010485.hg.1	
TC08000781.hg.1	-4.34	JUC08005402.hg.1	
TC17000362.hg.1	3.49	PSR17004523.hg.1	NF1
TC17000362.hg.1	2.81	JUC17002621.hg.1	NF1
TC17000362.hg.1	2.56	JUC17002589.hg.1	NF1
TC17000362.hg.1	2.55	PSR17004543.hg.1	NF1
TC17000362.hg.1	2.43	PSR17004574.hg.1	NF1
TC17000362.hg.1	2.28	PSR17004606.hg.1	NF1
TC17000362.hg.1	2.26	JUC17002599.hg.1	NF1
TC17000362.hg.1	-2.02	JUC17002586.hg.1	NF1
TC17000362.hg.1	-2.15	JUC17002559.hg.1	NF1
TC17000362.hg.1	-2.36	PSR17004544.hg.1	NF1
TC17000966.hg.1	3.49	JUC17007257.hg.1	VPS53
TC17000966.hg.1	-2.4	PSR17012757.hg.1	VPS53
TC17000966.hg.1	-2.54	JUC17007264.hg.1	VPS53
TC17000966.hg.1	-2.68	PSR17012751.hg.1	VPS53
TC17000966.hg.1	-3.33	JUC17007258.hg.1	VPS53
TC03000866.hg.1	3.48	PSR03015324.hg.1	GFM1
TC03000866.hg.1	3.35	JUC03007764.hg.1	GFM1
TC03000866.hg.1	-2.15	JUC03007767.hg.1	GFM1
TC03000866.hg.1	-3.57	JUC03007766.hg.1	GFM1
TC04000012.hg.1	3.48	PSR04000250.hg.1	MYL5
TC04000012.hg.1	3	PSR04000229.hg.1	MYL5
TC04000012.hg.1	2.56	PSR04000234.hg.1	MYL5
TC04000012.hg.1	2.36	PSR04000236.hg.1	MYL5
TC04000012.hg.1	2.16	PSR04000232.hg.1	MYL5
TC04000012.hg.1	2.14	PSR04000242.hg.1	MYL5
TC04000012.hg.1	2.14	JUC04000085.hg.1	MYL5
TC04000012.hg.1	-2.43	PSR04000244.hg.1	MYL5
TC05000075.hg.1	3.48	JUC05000470.hg.1	ROPN1L
TC05000075.hg.1	-2.52	PSR05000881.hg.1	ROPN1L
TC05000075.hg.1	-4.11	JUC05000471.hg.1	ROPN1L
TC05000364.hg.1	3.48	PSR05005053.hg.1	POLK
TC05000364.hg.1	2.96	JUC05002720.hg.1	POLK
TC05000364.hg.1	2.23	JUC05002727.hg.1	POLK
TC05000364.hg.1	2.19	PSR05005074.hg.1	POLK
TC05000364.hg.1	-2.57	JUC05002732.hg.1	POLK
TC06001879.hg.1	3.48	JUC06011104.hg.1	COL12A1
TC06001879.hg.1	3.04	PSR06022862.hg.1	COL12A1
TC06001879.hg.1	2.82	PSR06022821.hg.1	COL12A1
TC06001879.hg.1	2.72	JUC06011038.hg.1	COL12A1
TC06001879.hg.1	2.71	PSR06022809.hg.1	COL12A1
TC10000715.hg.1	3.48	JUC10004662.hg.1	ABCC2
TC10000715.hg.1	3.24	JUC10004652.hg.1	ABCC2
TC10000715.hg.1	-2.28	JUC10004642.hg.1	ABCC2
TC10000715.hg.1	-2.4	PSR10008357.hg.1	ABCC2
TC10000715.hg.1	-2.76	JUC10004656.hg.1	ABCC2
TC10001683.hg.1	3.48	JUC10012877.hg.1	KIAA1598
TC10001683.hg.1	2.61	JUC10012888.hg.1	KIAA1598
TC10001683.hg.1	2.29	PSR10022195.hg.1	KIAA1598
TC10001683.hg.1	-2.13	JUC10012901.hg.1	KIAA1598
TC10001683.hg.1	-2.31	PSR10022190.hg.1	KIAA1598
TC10001683.hg.1	-2.75	JUC10012880.hg.1	KIAA1598
TC11000272.hg.1	3.48	JUC11001862.hg.1	LUZP2
TC11000272.hg.1	3.05	JUC11001851.hg.1	LUZP2
TC11000272.hg.1	3.03	JUC11001857.hg.1	LUZP2
TC11000272.hg.1	2.78	JUC11001853.hg.1	LUZP2

TC11000272.hg.1	2.12	PSR11003605.hg.1	LUZP2
TC11000272.hg.1	-2.02	JUC11001859.hg.1	LUZP2
TC11000272.hg.1	-2.05	PSR11003594.hg.1	LUZP2
TC11000272.hg.1	-2.15	PSR11003596.hg.1	LUZP2
TC11000272.hg.1	-2.37	PSR11003611.hg.1	LUZP2
TC14000261.hg.1	3.48	JUC14001263.hg.1	C14orf28
TC14000261.hg.1	-2.21	PSR14002782.hg.1	C14orf28
TC19001220.hg.1	3.48	JUC19009926.hg.1	CACNA1A
TC19001220.hg.1	2.77	PSR19017147.hg.1	CACNA1A
TC19001220.hg.1	2.67	JUC19009940.hg.1	CACNA1A
TC19001220.hg.1	2.66	JUC19009951.hg.1	CACNA1A
TC19001220.hg.1	2.61	PSR19017185.hg.1	CACNA1A
TC19001220.hg.1	2.56	JUC19009910.hg.1	CACNA1A
TC19001220.hg.1	2.33	PSR19017140.hg.1	CACNA1A
TC19001220.hg.1	2.33	PSR19017146.hg.1	CACNA1A
TC19001220.hg.1	2.08	JUC19009917.hg.1	CACNA1A
TC19001220.hg.1	2.05	JUC19009918.hg.1	CACNA1A
TC19001220.hg.1	2.04	PSR19017173.hg.1	CACNA1A
TC19001220.hg.1	-2.01	PSR19017169.hg.1	CACNA1A
TC19001220.hg.1	-2.01	JUC19009934.hg.1	CACNA1A
TC19001220.hg.1	-2.06	JUC19009929.hg.1	CACNA1A
TC19001220.hg.1	-2.06	JUC19009938.hg.1	CACNA1A
TC19001220.hg.1	-2.17	PSR19017177.hg.1	CACNA1A
TC19001220.hg.1	-2.32	JUC19009914.hg.1	CACNA1A
TC19001220.hg.1	-2.48	JUC19009959.hg.1	CACNA1A
TC19001220.hg.1	-2.65	JUC19009930.hg.1	CACNA1A
TC19001220.hg.1	-2.66	JUC19009945.hg.1	CACNA1A
TC19001220.hg.1	-3.28	JUC19009942.hg.1	CACNA1A
TC19001220.hg.1	-3.56	JUC19009958.hg.1	CACNA1A
TC19001220.hg.1	-3.82	JUC19009931.hg.1	CACNA1A
TC19001220.hg.1	-3.95	JUC19009915.hg.1	CACNA1A
TC21000169.hg.1	3.48	PSR21001603.hg.1	ETS2
TC21000169.hg.1	2.97	JUC21000834.hg.1	ETS2
TC21000169.hg.1	2.96	JUC21000837.hg.1	ETS2
TC21000169.hg.1	2.68	JUC21000828.hg.1	ETS2
TC21000169.hg.1	2.23	PSR21001611.hg.1	ETS2
TC05000799.hg.1	3.47	PSR05011313.hg.1	TCERG1
TC05000799.hg.1	2.43	JUC05005869.hg.1	TCERG1
TC05001297.hg.1	3.47	JUC05009390.hg.1	TTC33
TC05001297.hg.1	2.6	PSR05018268.hg.1	TTC33
TC05001297.hg.1	2	JUC05009391.hg.1	TTC33
TC07000305.hg.1	3.47	PSR07004588.hg.1	C7orf69
TC07000305.hg.1	2.43	PSR07004579.hg.1	C7orf69
TC07000305.hg.1	2.17	JUC07002202.hg.1	C7orf69
TC08000299.hg.1	3.47	JUC08002121.hg.1	ADAM32
TC08000299.hg.1	3.31	PSR08004292.hg.1	ADAM32
TC08000299.hg.1	2.84	JUC08002139.hg.1	ADAM32
TC08000299.hg.1	2.83	JUC08002130.hg.1	ADAM32
TC08000299.hg.1	2.18	JUC08002134.hg.1	ADAM32
TC08000299.hg.1	2.13	PSR08004270.hg.1	ADAM32
TC08000299.hg.1	-2.07	JUC08002118.hg.1	ADAM32
TC08000299.hg.1	-2.14	JUC08002131.hg.1	ADAM32
TC08000299.hg.1	-2.61	JUC08002155.hg.1	ADAM32
TC08000299.hg.1	-2.86	JUC08002137.hg.1	ADAM32
TC10002939.hg.1	3.47	PSR10006695.hg.1	AGAP11
TC10002939.hg.1	3.15	PSR10006704.hg.1	AGAP11
TC10002939.hg.1	3.13	PSR10006697.hg.1	AGAP11
TC10002939.hg.1	2.88	JUC10017208.hg.1	AGAP11

TC10002939.hg.1	2.7	PSR10006698.hg.1	AGAP11
TC10002939.hg.1	2.7	JUC10017206.hg.1	AGAP11
TC10002939.hg.1	2.57	JUC10017212.hg.1	AGAP11
TC10002939.hg.1	2.32	JUC10017209.hg.1	AGAP11
TC10002939.hg.1	2.1	PSR10006690.hg.1	AGAP11
TC10002939.hg.1	2	PSR10006702.hg.1	AGAP11
TC10002939.hg.1	-2.01	PSR10006701.hg.1	AGAP11
TC10002939.hg.1	-2.72	JUC10017205.hg.1	AGAP11
TC10002939.hg.1	-4.76	JUC10017213.hg.1	AGAP11
TC10002939.hg.1	-6.07	PSR10006700.hg.1	AGAP11
TC12000810.hg.1	3.47	JUC12005643.hg.1	TXNRD1
TC12000810.hg.1	2.97	JUC12005661.hg.1	TXNRD1
TC12000810.hg.1	2.59	PSR12010291.hg.1	TXNRD1
TC12000810.hg.1	2.49	PSR12010286.hg.1	TXNRD1
TC12000810.hg.1	2.33	PSR12010289.hg.1	TXNRD1
TC12000810.hg.1	2.14	JUC12005658.hg.1	TXNRD1
TC12000810.hg.1	2.1	JUC12005648.hg.1	TXNRD1
TC12000810.hg.1	-2	JUC12005641.hg.1	TXNRD1
TC12000810.hg.1	-2.17	JUC12005632.hg.1	TXNRD1
TC15001112.hg.1	3.47	PSR15010146.hg.1	
TC15001112.hg.1	-3.47	PSR15010147.hg.1	
TC15001112.hg.1	-5.67	JUC15005377.hg.1	
TC22001504.hg.1	3.47	JUC22009899.hg.1	LOC150381
TC22001504.hg.1	2.84	PSR22007522.hg.1	LOC150381
TC22001504.hg.1	2.6	PSR22007518.hg.1	LOC150381
TC22001504.hg.1	2.15	PSR22007533.hg.1	LOC150381
TC22001504.hg.1	2	PSR22007520.hg.1	LOC150381
TC03001708.hg.1	3.47	JUC03015385.hg.1	SEMA5B
TC03001708.hg.1	-2	PSR03030923.hg.1	SEMA5B
TC03001708.hg.1	-3.05	JUC03015393.hg.1	SEMA5B
TC01006304.hg.1	3.46	JUC01039708.hg.1	GBA
TC01006304.hg.1	2.98	JUC01039707.hg.1	GBA
TC01006304.hg.1	2.59	JUC01039700.hg.1	GBA
TC01006304.hg.1	2.52	PSR01051248.hg.1	GBA
TC01006304.hg.1	2.19	PSR01051235.hg.1	GBA
TC01006304.hg.1	-3.86	JUC01039727.hg.1	GBA
TC02004967.hg.1	3.46	JUC02031741.hg.1	APLF
TC02004967.hg.1	3.01	JUC02031738.hg.1	APLF
TC02004967.hg.1	2.2	JUC02031744.hg.1	APLF
TC02004967.hg.1	2.01	PSR02005965.hg.1	APLF
TC03001125.hg.1	3.46	JUC03009761.hg.1	SUMF1
TC03001125.hg.1	2.73	PSR03019414.hg.1	SUMF1
TC03001125.hg.1	2.4	PSR03019426.hg.1	SUMF1
TC03001125.hg.1	2.38	PSR03019433.hg.1	SUMF1
TC03001125.hg.1	2.29	JUC03009767.hg.1	SUMF1
TC03001125.hg.1	2.01	PSR03019447.hg.1	SUMF1
TC03001125.hg.1	2	PSR03019416.hg.1	SUMF1
TC03001125.hg.1	-2.02	JUC03009782.hg.1	SUMF1
TC06000717.hg.1	3.46	PSR06008552.hg.1	C6orf57
TC06000717.hg.1	2.01	JUC06003866.hg.1	C6orf57
TC06004079.hg.1	3.46	JUC06021083.hg.1	C4B, C4A, LC
TC06004079.hg.1	2.68	PSR06004314.hg.1	C4B, C4A, LC
TC06004079.hg.1	2.57	JUC06021053.hg.1	C4B, C4A, LC
TC06004079.hg.1	2.55	JUC06021080.hg.1	C4B, C4A, LC
TC06004079.hg.1	2.5	JUC06021070.hg.1	C4B, C4A, LC
TC06004079.hg.1	2.44	PSR06004294.hg.1	C4B, C4A, LC
TC06004079.hg.1	2.38	PSR06004325.hg.1	C4B, C4A, LC
TC06004079.hg.1	2.28	JUC06021066.hg.1	C4B, C4A, LC

TC06004079.hg.1	2.19	PSR06004290.hg.1	C4B, C4A, LC
TC06004079.hg.1	2.13	PSR06004309.hg.1	C4B, C4A, LC
TC06004079.hg.1	2.12	JUC06021084.hg.1	C4B, C4A, LC
TC06004079.hg.1	2.1	PSR06004274.hg.1	C4B, C4A, LC
TC06004079.hg.1	2.06	PSR06004328.hg.1	C4B, C4A, LC
TC06004079.hg.1	2.05	PSR06004283.hg.1	C4B, C4A, LC
TC06004079.hg.1	2	PSR06004305.hg.1	C4B, C4A, LC
TC06004079.hg.1	-2.19	JUC06021057.hg.1	C4B, C4A, LC
TC06004079.hg.1	-2.74	JUC06021060.hg.1	C4B, C4A, LC
TC06004079.hg.1	-2.74	JUC06021081.hg.1	C4B, C4A, LC
TC06004079.hg.1	-2.94	JUC06021091.hg.1	C4B, C4A, LC
TC06004079.hg.1	-3.5	JUC06021061.hg.1	C4B, C4A, LC
TC07003313.hg.1	3.46	PSR07009399.hg.1	PILRB
TC07003313.hg.1	2.33	JUC07020682.hg.1	PILRB
TC07003313.hg.1	-2.45	JUC07020690.hg.1	PILRB
TC10001726.hg.1	3.46	JUC10013230.hg.1	OAT
TC10001726.hg.1	-2.5	PSR10022897.hg.1	OAT
TC03001402.hg.1	3.45	PSR03024883.hg.1	LAMB2
TC03001402.hg.1	2.77	PSR03024854.hg.1	LAMB2
TC03001402.hg.1	2.66	PSR03024856.hg.1	LAMB2
TC03001402.hg.1	2.61	PSR03024894.hg.1	LAMB2
TC03001402.hg.1	2.59	PSR03024855.hg.1	LAMB2
TC03001402.hg.1	2.34	PSR03024858.hg.1	LAMB2
TC03001402.hg.1	2.26	PSR03024884.hg.1	LAMB2
TC03001402.hg.1	2.12	PSR03024881.hg.1	LAMB2
TC03001402.hg.1	2.09	JUC03012342.hg.1	LAMB2
TC06001104.hg.1	3.45	JUC06006463.hg.1	ESR1
TC06001104.hg.1	2.57	JUC06006447.hg.1	ESR1
TC06001104.hg.1	2.55	JUC06006462.hg.1	ESR1
TC06001104.hg.1	2.18	PSR06013062.hg.1	ESR1
TC06001104.hg.1	-2.1	PSR06013076.hg.1	ESR1
TC06001104.hg.1	-2.11	JUC06006471.hg.1	ESR1
TC06001104.hg.1	-2.35	JUC06006449.hg.1	ESR1
TC06001104.hg.1	-2.58	JUC06006444.hg.1	ESR1
TC06001573.hg.1	3.45	PSR06018782.hg.1	PSMB8
TC06001573.hg.1	2.72	JUC06008951.hg.1	PSMB8
TC06001573.hg.1	-2.75	PSR06018775.hg.1	PSMB8
TC11002360.hg.1	3.45	JUC11015111.hg.1	SLC37A4
TC11002360.hg.1	2.7	PSR11028331.hg.1	SLC37A4
TC11002360.hg.1	2.18	PSR11028354.hg.1	SLC37A4
TC11002360.hg.1	2.09	JUC11015110.hg.1	SLC37A4
TC11002360.hg.1	-2.15	JUC11015118.hg.1	SLC37A4
TC15000625.hg.1	3.45	JUC15002563.hg.1	MAP2K5
TC15000625.hg.1	2.57	PSR15005191.hg.1	MAP2K5
TC15000625.hg.1	-2	PSR15005183.hg.1	MAP2K5
TC15000625.hg.1	-2.17	PSR15005190.hg.1	MAP2K5
TC21000222.hg.1	3.45	PSR21002638.hg.1	PFKL
TC21000222.hg.1	2.93	PSR21002642.hg.1	PFKL
TC21000222.hg.1	2.41	PSR21002622.hg.1	PFKL
TC21000222.hg.1	2.4	PSR21002630.hg.1	PFKL
TC21000222.hg.1	2.32	PSR21002628.hg.1	PFKL
TC21000222.hg.1	2.25	PSR21002646.hg.1	PFKL
TC21000222.hg.1	2.23	PSR21002621.hg.1	PFKL
TC21000222.hg.1	2.13	PSR21002606.hg.1	PFKL
TC21000222.hg.1	2.1	PSR21002633.hg.1	PFKL
TC21000222.hg.1	2.1	PSR21002644.hg.1	PFKL
TC21000222.hg.1	2.03	PSR21002632.hg.1	PFKL
TC21000222.hg.1	2.03	JUC21001363.hg.1	PFKL

TC6_apd_hap1000	3.45	PSR6_apd_hap1001	PSMB8
TC6_apd_hap1000	2.72	JUC6_apd_hap1000	PSMB8
TC6_apd_hap1000	-2.75	PSR6_apd_hap1001	PSMB8
TC6_cox_hap2000	3.45	PSR6_cox_hap2003	PSMB8
TC6_cox_hap2000	2.72	JUC6_cox_hap2001	PSMB8
TC6_cox_hap2000	-2.75	PSR6_cox_hap2003	PSMB8
TC6_dbb_hap3000	3.45	PSR6_dbb_hap3003	PSMB8
TC6_dbb_hap3000	2.95	JUC6_dbb_hap3001	PSMB8
TC6_dbb_hap3000	-2.75	PSR6_dbb_hap3003	PSMB8
TC6_mann_hap40	3.45	PSR6_mann_hap400	PSMB8
TC6_mann_hap40	2.72	JUC6_mann_hap400	PSMB8
TC6_mann_hap40	-2.75	PSR6_mann_hap400	PSMB8
TC6_mcf_hap5000	3.45	PSR6_mcf_hap5003	PSMB8
TC6_mcf_hap5000	2.72	JUC6_mcf_hap5001	PSMB8
TC6_mcf_hap5000	-2.75	PSR6_mcf_hap5003	PSMB8
TC6_qbl_hap6000	3.45	PSR6_qbl_hap60037	PSMB8
TC6_qbl_hap6000	2.72	JUC6_qbl_hap60014	PSMB8
TC6_qbl_hap6000	-2.75	PSR6_qbl_hap60037	PSMB8
TC03000204.hg.1	3.44	JUC03002193.hg.1	MYRIP
TC03000204.hg.1	3.01	JUC03002191.hg.1	MYRIP
TC03000204.hg.1	2.72	PSR03004082.hg.1	MYRIP
TC03000204.hg.1	2.67	JUC03002177.hg.1	MYRIP
TC03000204.hg.1	2.63	PSR03004074.hg.1	MYRIP
TC03000204.hg.1	2.51	PSR03004065.hg.1	MYRIP
TC03000204.hg.1	2.34	PSR03004064.hg.1	MYRIP
TC03000204.hg.1	2.22	PSR03004069.hg.1	MYRIP
TC03000204.hg.1	2.21	PSR03004067.hg.1	MYRIP
TC03000204.hg.1	2.13	PSR03004081.hg.1	MYRIP
TC03000204.hg.1	-2.19	JUC03002178.hg.1	MYRIP
TC03000204.hg.1	-2.23	PSR03004090.hg.1	MYRIP
TC03000204.hg.1	-2.68	JUC03002179.hg.1	MYRIP
TC03000204.hg.1	-2.73	JUC03002182.hg.1	MYRIP
TC03000204.hg.1	-2.91	JUC03002192.hg.1	MYRIP
TC10000874.hg.1	3.44	JUC10005946.hg.1	PPAPDC1A
TC10000874.hg.1	2.81	JUC10005953.hg.1	PPAPDC1A
TC10000874.hg.1	2.09	PSR10010586.hg.1	PPAPDC1A
TC10000874.hg.1	-2.05	PSR10010596.hg.1	PPAPDC1A
TC10000874.hg.1	-2.2	PSR10010583.hg.1	PPAPDC1A
TC10000874.hg.1	-3.96	PSR10010598.hg.1	PPAPDC1A
TC10000874.hg.1	-4.33	JUC10005951.hg.1	PPAPDC1A
TC10000874.hg.1	-4.68	JUC10005958.hg.1	PPAPDC1A
TC10000874.hg.1	-5.45	PSR10010592.hg.1	PPAPDC1A
TC10000874.hg.1	-5.51	PSR10010584.hg.1	PPAPDC1A
TC10000874.hg.1	-7.39	JUC10005948.hg.1	PPAPDC1A
TC10000874.hg.1	-7.88	PSR10010593.hg.1	PPAPDC1A
TC10000874.hg.1	-9.32	JUC10005945.hg.1	PPAPDC1A
TC10000874.hg.1	-9.64	PSR10010577.hg.1	PPAPDC1A
TC10000874.hg.1	-10.34	JUC10005955.hg.1	PPAPDC1A
TC10000874.hg.1	-11.2	JUC10005947.hg.1	PPAPDC1A
TC10000874.hg.1	-11.21	JUC10005954.hg.1	PPAPDC1A
TC10000874.hg.1	-14.73	PSR10010582.hg.1	PPAPDC1A
TC10000874.hg.1	-16.1	PSR10010594.hg.1	PPAPDC1A
TC20000685.hg.1	3.44	JUC20004956.hg.1	RALGAPA2
TC20000685.hg.1	2.34	JUC20004960.hg.1	RALGAPA2
TC20000685.hg.1	2.25	PSR20009517.hg.1	RALGAPA2
TC20000685.hg.1	2.02	JUC20004964.hg.1	RALGAPA2
TC20000685.hg.1	-2.85	JUC20004953.hg.1	RALGAPA2
TC22000856.hg.1	3.44	JUC22005968.hg.1	ARFGAP3, P

TC22000856.hg.1	3.26	JUC22005961.hg.1	ARFGAP3, PA
TC22000856.hg.1	3.17	PSR22014463.hg.1	ARFGAP3, PA
TC22000856.hg.1	2.96	JUC22005970.hg.1	ARFGAP3, PA
TC22000856.hg.1	2.77	PSR22014461.hg.1	ARFGAP3, PA
TC22000856.hg.1	2.72	JUC22005932.hg.1	ARFGAP3, PA
TC22000856.hg.1	2.68	PSR22014452.hg.1	ARFGAP3, PA
TC22000856.hg.1	2.45	JUC22005936.hg.1	ARFGAP3, PA
TC22000856.hg.1	2.44	JUC22005971.hg.1	ARFGAP3, PA
TC22000856.hg.1	2.38	PSR22014458.hg.1	ARFGAP3, PA
TC22000856.hg.1	2.35	PSR22014444.hg.1	ARFGAP3, PA
TC22000856.hg.1	2.34	PSR22014454.hg.1	ARFGAP3, PA
TC22000856.hg.1	2.3	PSR22014455.hg.1	ARFGAP3, PA
TC22000856.hg.1	2.29	JUC22005942.hg.1	ARFGAP3, PA
TC22000856.hg.1	2.27	PSR22014443.hg.1	ARFGAP3, PA
TC22000856.hg.1	2.24	PSR22014447.hg.1	ARFGAP3, PA
TC22000856.hg.1	2.23	PSR22014459.hg.1	ARFGAP3, PA
TC22000856.hg.1	2.19	PSR22014448.hg.1	ARFGAP3, PA
TC22000856.hg.1	2.15	PSR22014471.hg.1	ARFGAP3, PA
TC22000856.hg.1	2.15	JUC22005965.hg.1	ARFGAP3, PA
TC22000856.hg.1	2.03	PSR22014450.hg.1	ARFGAP3, PA
TC22000856.hg.1	2.03	PSR22014453.hg.1	ARFGAP3, PA
TC22000856.hg.1	2.02	JUC22005933.hg.1	ARFGAP3, PA
TC22000856.hg.1	-2.32	JUC22005948.hg.1	ARFGAP3, PA
TC6_dbb_hap3000	3.44	JUC6_dbb_hap3000	DDR1
TC6_dbb_hap3000	3.21	PSR6_dbb_hap3000	DDR1
TC6_dbb_hap3000	3.12	JUC6_dbb_hap3000	DDR1
TC6_dbb_hap3000	3.1	PSR6_dbb_hap3000	DDR1
TC6_dbb_hap3000	3.07	PSR6_dbb_hap3000	DDR1
TC6_dbb_hap3000	2.99	PSR6_dbb_hap3000	DDR1
TC6_dbb_hap3000	2.98	JUC6_dbb_hap3000	DDR1
TC6_dbb_hap3000	2.84	JUC6_dbb_hap3000	DDR1
TC6_dbb_hap3000	2.79	JUC6_dbb_hap3000	DDR1
TC6_dbb_hap3000	2.59	PSR6_dbb_hap3000	DDR1
TC6_dbb_hap3000	2.51	JUC6_dbb_hap3000	DDR1
TC6_dbb_hap3000	2.44	PSR6_dbb_hap3000	DDR1
TC6_dbb_hap3000	2.43	PSR6_dbb_hap3000	DDR1
TC6_dbb_hap3000	2.42	JUC6_dbb_hap3000	DDR1
TC6_dbb_hap3000	2.36	PSR6_dbb_hap3000	DDR1
TC6_dbb_hap3000	2.35	PSR6_dbb_hap3000	DDR1
TC6_dbb_hap3000	2.33	JUC6_dbb_hap3000	DDR1
TC6_dbb_hap3000	2.19	PSR6_dbb_hap3000	DDR1
TC6_dbb_hap3000	2.15	PSR6_dbb_hap3000	DDR1
TC6_dbb_hap3000	2.11	PSR6_dbb_hap3000	DDR1
TC6_dbb_hap3000	2.1	PSR6_dbb_hap3000	DDR1
TC6_dbb_hap3000	2.07	PSR6_dbb_hap3000	DDR1
TC6_dbb_hap3000	-2.38	PSR6_dbb_hap3000	DDR1
TC01002984.hg.1	3.43	PSR01046330.hg.1	OVGP1
TC01002984.hg.1	3.21	JUC01024911.hg.1	OVGP1
TC01002984.hg.1	2.08	JUC01024914.hg.1	OVGP1
TC02000894.hg.1	3.43	JUC02007047.hg.1	SPOPL
TC02000894.hg.1	2.5	PSR02013269.hg.1	SPOPL
TC02002573.hg.1	3.43	JUC02021491.hg.1	PRKRA
TC02002573.hg.1	2.06	PSR02040837.hg.1	PRKRA
TC03001362.hg.1	3.43	JUC03011615.hg.1	MYL3
TC03001362.hg.1	2.49	PSR03023330.hg.1	MYL3
TC03001362.hg.1	-2.1	JUC03011616.hg.1	MYL3
TC03001362.hg.1	-2.22	PSR03023338.hg.1	MYL3
TC03001362.hg.1	-2.32	JUC03011617.hg.1	MYL3

TC03001362.hg.1	-2.4	PSR03023340.hg.1	MYL3
TC03001362.hg.1	-2.75	JUC03011610.hg.1	MYL3
TC10001006.hg.1	3.43	JUC10007260.hg.1	IL15RA
TC10001006.hg.1	2.52	JUC10007258.hg.1	IL15RA
TC10001006.hg.1	2.1	JUC10007272.hg.1	IL15RA
TC10001006.hg.1	2.01	PSR10012560.hg.1	IL15RA
TC10001006.hg.1	-2.45	PSR10012545.hg.1	IL15RA
TC16000022.hg.1	3.43	JUC16000124.hg.1	MSLN
TC16000022.hg.1	3.06	JUC16000125.hg.1	MSLN
TC16000022.hg.1	2.93	JUC16000123.hg.1	MSLN
TC16000022.hg.1	2.73	PSR16000516.hg.1	MSLN
TC16000022.hg.1	2.7	JUC16000121.hg.1	MSLN
TC16000022.hg.1	2.67	JUC16000112.hg.1	MSLN
TC16000022.hg.1	2.59	PSR16000500.hg.1	MSLN
TC16000022.hg.1	2.44	PSR16000497.hg.1	MSLN
TC16000022.hg.1	2.17	JUC16000116.hg.1	MSLN
TC16000022.hg.1	2.09	PSR16000503.hg.1	MSLN
TC16000022.hg.1	-4.22	JUC16000109.hg.1	MSLN
TC16000022.hg.1	-7.94	JUC16000110.hg.1	MSLN
TC16000022.hg.1	-8.57	PSR16000510.hg.1	MSLN
TC16000022.hg.1	-15.82	JUC16000115.hg.1	MSLN
TC20000277.hg.1	3.43	JUC20002215.hg.1	VSTM2L
TC20000277.hg.1	3.17	PSR20003944.hg.1	VSTM2L
TC20001008.hg.1	3.43	JUC20007190.hg.1	LAMA5, MIR4
TC20001008.hg.1	2.47	JUC20007213.hg.1	LAMA5, MIR4
TC20001008.hg.1	2.04	JUC20007149.hg.1	LAMA5, MIR4
TC20001008.hg.1	2.04	JUC20007217.hg.1	LAMA5, MIR4
TC20001008.hg.1	2.02	JUC20007142.hg.1	LAMA5, MIR4
TC20001008.hg.1	-2	PSR20013767.hg.1	LAMA5, MIR4
TC20001008.hg.1	-2.08	PSR20013839.hg.1	LAMA5, MIR4
TC20001008.hg.1	-2.13	JUC20007169.hg.1	LAMA5, MIR4
TC20001008.hg.1	-2.14	PSR20013820.hg.1	LAMA5, MIR4
TC20001008.hg.1	-2.14	PSR20013840.hg.1	LAMA5, MIR4
TC20001008.hg.1	-2.14	JUC20007177.hg.1	LAMA5, MIR4
TC20001008.hg.1	-2.19	PSR20013837.hg.1	LAMA5, MIR4
TC20001008.hg.1	-2.27	JUC20007185.hg.1	LAMA5, MIR4
TC20001008.hg.1	-2.46	PSR20013823.hg.1	LAMA5, MIR4
TC20001008.hg.1	-2.5	JUC20007158.hg.1	LAMA5, MIR4
TC20001008.hg.1	-2.71	PSR20013766.hg.1	LAMA5, MIR4
TC20001008.hg.1	-2.73	PSR20013752.hg.1	LAMA5, MIR4
TC20001008.hg.1	-2.87	JUC20007196.hg.1	LAMA5, MIR4
TC20001008.hg.1	-2.91	JUC20007137.hg.1	LAMA5, MIR4
TC20001008.hg.1	-3.03	PSR20013843.hg.1	LAMA5, MIR4
TC20001008.hg.1	-3.45	JUC20007155.hg.1	LAMA5, MIR4
TC20001008.hg.1	-3.47	PSR20013806.hg.1	LAMA5, MIR4
TC20001008.hg.1	-3.64	JUC20007167.hg.1	LAMA5, MIR4
TC20001008.hg.1	-3.76	PSR20013817.hg.1	LAMA5, MIR4
TC20001008.hg.1	-4.08	PSR20013739.hg.1	LAMA5, MIR4
TC20001008.hg.1	-4.36	JUC20007197.hg.1	LAMA5, MIR4
TC20001008.hg.1	-4.91	JUC20007174.hg.1	LAMA5, MIR4
TC20001008.hg.1	-4.97	JUC20007201.hg.1	LAMA5, MIR4
TC20001008.hg.1	-6.52	JUC20007179.hg.1	LAMA5, MIR4
TC22000911.hg.1	3.43	PSR22015472.hg.1	PLXNB2
TC22000911.hg.1	2.84	PSR22015474.hg.1	PLXNB2
TC22000911.hg.1	2.39	PSR22015427.hg.1	PLXNB2
TC22000911.hg.1	2.35	JUC22006513.hg.1	PLXNB2
TC22000911.hg.1	2.26	JUC22006506.hg.1	PLXNB2
TC22000911.hg.1	2.16	JUC22006507.hg.1	PLXNB2

TC07000688.hg.1	3.42	JUC07005048.hg.1	PRKAR2B
TC07000688.hg.1	2.99	PSR07010720.hg.1	PRKAR2B
TC07000688.hg.1	2.86	PSR07010726.hg.1	PRKAR2B
TC07000688.hg.1	2.54	JUC07005040.hg.1	PRKAR2B
TC07000688.hg.1	-2.08	PSR07010730.hg.1	PRKAR2B
TC09000593.hg.1	3.42	JUC09003732.hg.1	PAPPA
TC09000593.hg.1	2.16	PSR09006732.hg.1	PAPPA
TC09001342.hg.1	3.42	PSR09016829.hg.1	ZNF484
TC09001342.hg.1	-2.04	JUC09009044.hg.1	ZNF484
TC12003211.hg.1	3.42	JUC12019449.hg.1	ACAD10
TC12003211.hg.1	2.73	PSR12011442.hg.1	ACAD10
TC12003211.hg.1	-2.25	PSR12011414.hg.1	ACAD10
TC12003211.hg.1	-2.86	PSR12011409.hg.1	ACAD10
TC13000644.hg.1	3.42	JUC13004249.hg.1	KIAA0226L
TC13000644.hg.1	2.39	JUC13004274.hg.1	KIAA0226L
TC13000644.hg.1	2.2	JUC13004279.hg.1	KIAA0226L
TC13000644.hg.1	-2.12	PSR13007336.hg.1	KIAA0226L
TC13000644.hg.1	-2.65	PSR13007353.hg.1	KIAA0226L
TC13000644.hg.1	-2.77	PSR13007356.hg.1	KIAA0226L
TC13000644.hg.1	-2.91	PSR13007366.hg.1	KIAA0226L
TC13000644.hg.1	-3.01	JUC13004250.hg.1	KIAA0226L
TC13000644.hg.1	-3.21	JUC13004280.hg.1	KIAA0226L
TC13000644.hg.1	-3.41	JUC13004281.hg.1	KIAA0226L
TC13000644.hg.1	-3.46	PSR13007368.hg.1	KIAA0226L
TC13000644.hg.1	-3.66	JUC13004276.hg.1	KIAA0226L
TC13000644.hg.1	-4.12	PSR13007365.hg.1	KIAA0226L
TC13000644.hg.1	-4.57	JUC13004277.hg.1	KIAA0226L
TC13000644.hg.1	-4.66	JUC13004251.hg.1	KIAA0226L
TC19002697.hg.1	3.42	JUC19017960.hg.1	ALKBH6
TC19002697.hg.1	3.22	JUC19017957.hg.1	ALKBH6
TC19002697.hg.1	2.33	JUC19017956.hg.1	ALKBH6
TC19002697.hg.1	2.29	JUC19017953.hg.1	ALKBH6
TC19002697.hg.1	2.15	PSR19019772.hg.1	ALKBH6
TC01001866.hg.1	3.41	JUC01015701.hg.1	ADCK3
TC01001866.hg.1	2.71	PSR01029088.hg.1	ADCK3
TC01001866.hg.1	2.63	PSR01029069.hg.1	ADCK3
TC01001866.hg.1	2.46	PSR01029036.hg.1	ADCK3
TC01001866.hg.1	2.36	JUC01015670.hg.1	ADCK3
TC01001866.hg.1	2.17	PSR01029042.hg.1	ADCK3
TC01001866.hg.1	2.12	JUC01015676.hg.1	ADCK3
TC01001866.hg.1	2.11	PSR01029073.hg.1	ADCK3
TC01001866.hg.1	2.08	JUC01015692.hg.1	ADCK3
TC01001866.hg.1	2.03	PSR01029043.hg.1	ADCK3
TC01001866.hg.1	2.03	JUC01015679.hg.1	ADCK3
TC01001866.hg.1	-2.4	JUC01015693.hg.1	ADCK3
TC01001866.hg.1	-2.42	JUC01015703.hg.1	ADCK3
TC01001866.hg.1	-2.67	JUC01015698.hg.1	ADCK3
TC01001866.hg.1	-2.72	JUC01015702.hg.1	ADCK3
TC03000351.hg.1	3.41	JUC03003735.hg.1	CACNA1D
TC03000351.hg.1	3.04	JUC03003774.hg.1	CACNA1D
TC03000351.hg.1	2.86	JUC03003784.hg.1	CACNA1D
TC03000351.hg.1	2.64	JUC03003744.hg.1	CACNA1D
TC03000351.hg.1	2.34	JUC03003741.hg.1	CACNA1D
TC03000351.hg.1	2.33	JUC03003787.hg.1	CACNA1D
TC03000351.hg.1	2.16	PSR03007717.hg.1	CACNA1D
TC03000351.hg.1	2.08	PSR03007715.hg.1	CACNA1D
TC03000351.hg.1	-2.06	JUC03003750.hg.1	CACNA1D
TC03000351.hg.1	-2.28	JUC03003749.hg.1	CACNA1D

TC03000351.hg.1	-3.12	JUC03003761.hg.1	CACNA1D
TC03001773.hg.1	3.41	JUC03016017.hg.1	TMCC1
TC03001773.hg.1	2.51	PSR03032191.hg.1	TMCC1
TC03001773.hg.1	2.36	JUC03016014.hg.1	TMCC1
TC03001773.hg.1	2.15	PSR03032195.hg.1	TMCC1
TC03001773.hg.1	-2.25	JUC03016001.hg.1	TMCC1
TC03001773.hg.1	-2.68	JUC03016005.hg.1	TMCC1
TC05001110.hg.1	3.41	JUC05008235.hg.1	LPCAT1
TC05001110.hg.1	2.61	PSR05016179.hg.1	LPCAT1
TC05001110.hg.1	2.49	JUC05008236.hg.1	LPCAT1
TC05001110.hg.1	2.29	JUC05008225.hg.1	LPCAT1
TC05001110.hg.1	2.28	PSR05016182.hg.1	LPCAT1
TC05001110.hg.1	2.26	PSR05016200.hg.1	LPCAT1
TC05001110.hg.1	2.24	PSR05016175.hg.1	LPCAT1
TC05001110.hg.1	2.22	PSR05016174.hg.1	LPCAT1
TC05001110.hg.1	-2.18	JUC05008227.hg.1	LPCAT1
TC05001110.hg.1	-2.24	PSR05016184.hg.1	LPCAT1
TC05001110.hg.1	-2.43	PSR05016187.hg.1	LPCAT1
TC05001110.hg.1	-3.12	JUC05008232.hg.1	LPCAT1
TC07001717.hg.1	3.41	JUC07012958.hg.1	DPY19L2P2
TC07001717.hg.1	2.62	JUC07012969.hg.1	DPY19L2P2
TC07001717.hg.1	2.46	JUC07012948.hg.1	DPY19L2P2
TC07001717.hg.1	2.43	JUC07012954.hg.1	DPY19L2P2
TC07001717.hg.1	2.04	PSR07026600.hg.1	DPY19L2P2
TC07001717.hg.1	-2.22	JUC07012967.hg.1	DPY19L2P2
TC07001717.hg.1	-2.27	PSR07026591.hg.1	DPY19L2P2
TC07001717.hg.1	-3.42	JUC07012953.hg.1	DPY19L2P2
TC09000489.hg.1	3.41	JUC09002864.hg.1	C9orf174, LO
TC09000489.hg.1	2.97	PSR09005251.hg.1	C9orf174, LO
TC09000489.hg.1	2.55	JUC09002853.hg.1	C9orf174, LO
TC09000489.hg.1	-2.02	PSR09005223.hg.1	C9orf174, LO
TC09000489.hg.1	-2.1	JUC09002874.hg.1	C9orf174, LO
TC09000489.hg.1	-2.14	JUC09002909.hg.1	C9orf174, LO
TC09000489.hg.1	-2.25	JUC09002884.hg.1	C9orf174, LO
TC09000489.hg.1	-2.35	JUC09002900.hg.1	C9orf174, LO
TC09001403.hg.1	3.41	JUC09009362.hg.1	TRIM14
TC09001403.hg.1	2.48	JUC09009372.hg.1	TRIM14
TC09001403.hg.1	2.1	PSR09017458.hg.1	TRIM14
TC09001403.hg.1	-2.02	PSR09017448.hg.1	TRIM14
TC09001403.hg.1	-2.26	PSR09017464.hg.1	TRIM14
TC09001403.hg.1	-2.72	JUC09009368.hg.1	TRIM14
TC09001403.hg.1	-4.24	JUC09009374.hg.1	TRIM14
TC0Y000017.hg.1	3.41	JUC0Y000103.hg.1	ZFY
TC0Y000017.hg.1	-2.09	JUC0Y000105.hg.1	ZFY
TC0Y000017.hg.1	-2.17	PSR0Y000215.hg.1	ZFY
TC0Y000017.hg.1	-2.21	PSR0Y000216.hg.1	ZFY
TC0Y000017.hg.1	-2.72	JUC0Y000106.hg.1	ZFY
TC0Y000017.hg.1	-5.61	PSR0Y000229.hg.1	ZFY
TC0Y000017.hg.1	-5.85	PSR0Y000230.hg.1	ZFY
TC0Y000017.hg.1	-6.41	JUC0Y000102.hg.1	ZFY
TC0Y000017.hg.1	-6.54	JUC0Y000109.hg.1	ZFY
TC0Y000017.hg.1	-8.2	JUC0Y000100.hg.1	ZFY
TC0Y000017.hg.1	-18.58	JUC0Y000101.hg.1	ZFY
TC12001393.hg.1	3.41	JUC12010083.hg.1	SLC2A13
TC12001393.hg.1	2.33	PSR12018088.hg.1	SLC2A13
TC12001393.hg.1	2.12	JUC12010085.hg.1	SLC2A13
TC14001387.hg.1	3.41	JUC14007992.hg.1	GALC
TC14001387.hg.1	2.2	JUC14007990.hg.1	GALC

TC14001387.hg.1	2.18	JUC14008002.hg.1	GALC
TC14001387.hg.1	-2.08	PSR14015658.hg.1	GALC
TC14001387.hg.1	-2.1	JUC14008000.hg.1	GALC
TC17001029.hg.1	3.41	PSR17013881.hg.1	CXCL16
TC17001029.hg.1	2.46	JUC17007916.hg.1	CXCL16
TC17001029.hg.1	2.31	PSR17013886.hg.1	CXCL16
TC17001029.hg.1	2.19	PSR17013887.hg.1	CXCL16
TC17001029.hg.1	-2.23	JUC17007914.hg.1	CXCL16
TC17001029.hg.1	-2.64	PSR17013885.hg.1	CXCL16
TC01002662.hg.1	3.4	JUC01022341.hg.1	ECHDC2
TC01002662.hg.1	2.4	JUC01022357.hg.1	ECHDC2
TC01002662.hg.1	2.33	JUC01022351.hg.1	ECHDC2
TC01002662.hg.1	-2.19	PSR01041797.hg.1	ECHDC2
TC02002853.hg.1	3.4	PSR02046049.hg.1	SP110
TC02002853.hg.1	-2.85	JUC02024119.hg.1	SP110
TC04001723.hg.1	3.4	JUC04012611.hg.1	CBR4
TC04001723.hg.1	2.63	PSR04023812.hg.1	CBR4
TC04001723.hg.1	2.42	JUC04012612.hg.1	CBR4
TC04001723.hg.1	-2.17	PSR04023815.hg.1	CBR4
TC07001654.hg.1	3.4	PSR07025380.hg.1	MCM7
TC07001654.hg.1	2.81	PSR07025376.hg.1	MCM7
TC07001654.hg.1	2.25	JUC07012499.hg.1	MCM7
TC07001654.hg.1	-2.33	JUC07012511.hg.1	MCM7
TC07001654.hg.1	-2.68	JUC07012514.hg.1	MCM7
TC14001253.hg.1	3.4	PSR14014237.hg.1	ZFP36L1
TC14001253.hg.1	2.66	JUC14007190.hg.1	ZFP36L1
TC14001253.hg.1	2.6	PSR14014238.hg.1	ZFP36L1
TC14001253.hg.1	2.58	JUC14007193.hg.1	ZFP36L1
TC14001253.hg.1	2.54	PSR14014236.hg.1	ZFP36L1
TC14001253.hg.1	2.5	JUC14007189.hg.1	ZFP36L1
TC14001253.hg.1	2.12	PSR14014235.hg.1	ZFP36L1
TC14001253.hg.1	-2.02	JUC14007194.hg.1	ZFP36L1
TC17000146.hg.1	3.4	JUC17001184.hg.1	USP43
TC17000146.hg.1	3.05	JUC17001194.hg.1	USP43
TC17000146.hg.1	2.3	PSR17002013.hg.1	USP43
TC01000367.hg.1	3.39	JUC01002944.hg.1	FAM76A
TC01000367.hg.1	2.37	PSR01005664.hg.1	FAM76A
TC0X001165.hg.1	3.39	JUC0X007942.hg.1	ZDHHC15
TC0X001165.hg.1	2.47	JUC0X007937.hg.1	ZDHHC15
TC0X001165.hg.1	-2.32	PSR0X015736.hg.1	ZDHHC15
TC15001338.hg.1	3.39	PSR15012850.hg.1	SHC4
TC15001338.hg.1	2.56	PSR15012835.hg.1	SHC4
TC15001338.hg.1	2.02	JUC15006846.hg.1	SHC4
TC02001935.hg.1	3.38	JUC02016184.hg.1	LOC644838
TC02001935.hg.1	2.86	PSR02030722.hg.1	LOC644838
TC03000336.hg.1	3.38	JUC03003559.hg.1	STAB1
TC03000336.hg.1	2.78	JUC03003563.hg.1	STAB1
TC03000336.hg.1	2.74	JUC03003567.hg.1	STAB1
TC03000336.hg.1	2.16	PSR03007401.hg.1	STAB1
TC03000336.hg.1	2.01	PSR03007393.hg.1	STAB1
TC03001145.hg.1	3.38	PSR03019627.hg.1	OXTR
TC03001145.hg.1	3.19	PSR03019628.hg.1	OXTR
TC03001145.hg.1	-2.25	PSR03019632.hg.1	OXTR
TC03001145.hg.1	-2.31	JUC03009898.hg.1	OXTR
TC05001883.hg.1	3.38	JUC05013261.hg.1	FGF1
TC05001883.hg.1	3.12	JUC05013267.hg.1	FGF1
TC05001883.hg.1	2.92	PSR05026024.hg.1	FGF1
TC05001883.hg.1	2.39	PSR05026021.hg.1	FGF1

TC05001883.hg.1	2.37	PSR05026020.hg.1	FGF1
TC05001883.hg.1	2.37	PSR05026028.hg.1	FGF1
TC05001883.hg.1	2.17	PSR05026029.hg.1	FGF1
TC05001883.hg.1	-3.75	JUC05013259.hg.1	FGF1
TC06001895.hg.1	3.38	JUC06011187.hg.1	PHIP
TC06001895.hg.1	3.17	JUC06011184.hg.1	PHIP
TC06001895.hg.1	2.86	PSR06023011.hg.1	PHIP
TC06001895.hg.1	2.53	JUC06011188.hg.1	PHIP
TC06001895.hg.1	2.39	JUC06011163.hg.1	PHIP
TC06001895.hg.1	2.37	JUC06011168.hg.1	PHIP
TC06001895.hg.1	2.2	PSR06023051.hg.1	PHIP
TC06001895.hg.1	2.09	PSR06022999.hg.1	PHIP
TC06001895.hg.1	2.09	PSR06023047.hg.1	PHIP
TC06001895.hg.1	-2.31	JUC06011162.hg.1	PHIP
TC06001895.hg.1	-3.27	JUC06011201.hg.1	PHIP
TC12003224.hg.1	3.38	JUC12019702.hg.1	C1S
TC12003224.hg.1	2.09	JUC12019681.hg.1	C1S
TC12003224.hg.1	-2.12	PSR12001403.hg.1	C1S
TC12001425.hg.1	3.38	JUC12010273.hg.1	SCAF11
TC12001425.hg.1	3.12	JUC12010271.hg.1	SCAF11
TC12001425.hg.1	2.81	JUC12010272.hg.1	SCAF11
TC12001425.hg.1	2.29	JUC12010267.hg.1	SCAF11
TC12001425.hg.1	2.12	JUC12010263.hg.1	SCAF11
TC12001425.hg.1	2	PSR12018412.hg.1	SCAF11
TC12001425.hg.1	-2.2	JUC12010269.hg.1	SCAF11
TC02000057.hg.1	3.37	PSR02000759.hg.1	RRM2
TC02000057.hg.1	2.85	PSR02000732.hg.1	RRM2
TC02000057.hg.1	2.69	JUC02000407.hg.1	RRM2
TC02000057.hg.1	2.28	PSR02000746.hg.1	RRM2
TC02000057.hg.1	-2.19	JUC02000408.hg.1	RRM2
TC02000057.hg.1	-3.46	JUC02000412.hg.1	RRM2
TC02001022.hg.1	3.37	JUC02008090.hg.1	DYNC1I2
TC02001022.hg.1	2.25	PSR02015123.hg.1	DYNC1I2
TC02002377.hg.1	3.37	PSR02037540.hg.1	DARS
TC02002377.hg.1	2.74	PSR02037533.hg.1	DARS
TC02002377.hg.1	2.63	JUC02019460.hg.1	DARS
TC02002377.hg.1	2.28	PSR02037539.hg.1	DARS
TC02002377.hg.1	2.17	PSR02037526.hg.1	DARS
TC06001128.hg.1	3.37	PSR06013405.hg.1	SYNJ2
TC06001128.hg.1	3.27	JUC06006593.hg.1	SYNJ2
TC06001128.hg.1	2.96	JUC06006602.hg.1	SYNJ2
TC06001128.hg.1	2.58	PSR06013408.hg.1	SYNJ2
TC06001128.hg.1	2.44	PSR06013445.hg.1	SYNJ2
TC06001128.hg.1	2.17	PSR06013424.hg.1	SYNJ2
TC06001128.hg.1	2.1	PSR06013447.hg.1	SYNJ2
TC06001128.hg.1	-2.05	JUC06006612.hg.1	SYNJ2
TC06001128.hg.1	-2.32	JUC06006610.hg.1	SYNJ2
TC06001128.hg.1	-2.35	JUC06006591.hg.1	SYNJ2
TC06001128.hg.1	-2.67	JUC06006598.hg.1	SYNJ2
TC06001128.hg.1	-3.06	JUC06006585.hg.1	SYNJ2
TC07000877.hg.1	3.37	JUC07006588.hg.1	TBXAS1
TC07000877.hg.1	2.33	JUC07006597.hg.1	TBXAS1
TC07000877.hg.1	2.28	JUC07006567.hg.1	TBXAS1
TC07000877.hg.1	2.04	PSR07013672.hg.1	TBXAS1
TC07000877.hg.1	-2.41	JUC07006596.hg.1	TBXAS1
TC07000877.hg.1	-2.63	PSR07013703.hg.1	TBXAS1
TC07000877.hg.1	-3.97	JUC07006584.hg.1	TBXAS1
TC10000876.hg.1	3.37	JUC10005977.hg.1	WDR11, LOC

TC10000876.hg.1	2.02	PSR10010609.hg.1	WDR11, LOC
TC11001812.hg.1	3.37	JUC11011169.hg.1	LPXN
TC11001812.hg.1	2.88	PSR11020949.hg.1	LPXN
TC11001812.hg.1	2.77	PSR11020950.hg.1	LPXN
TC11001812.hg.1	2.26	PSR11020942.hg.1	LPXN
TC11001812.hg.1	2.08	JUC11011176.hg.1	LPXN
TC13000411.hg.1	3.37	PSR13004492.hg.1	ATP11A
TC13000411.hg.1	3.16	JUC13002718.hg.1	ATP11A
TC13000411.hg.1	3.01	PSR13004453.hg.1	ATP11A
TC13000411.hg.1	2.75	JUC13002730.hg.1	ATP11A
TC13000411.hg.1	2.44	JUC13002719.hg.1	ATP11A
TC13000411.hg.1	2.43	PSR13004477.hg.1	ATP11A
TC13000411.hg.1	2.35	PSR13004455.hg.1	ATP11A
TC13000411.hg.1	2.34	PSR13004482.hg.1	ATP11A
TC13000411.hg.1	2.32	PSR13004451.hg.1	ATP11A
TC13000411.hg.1	2.27	JUC13002701.hg.1	ATP11A
TC13000411.hg.1	2.26	PSR13004508.hg.1	ATP11A
TC13000411.hg.1	2.2	PSR13004487.hg.1	ATP11A
TC13000411.hg.1	2.17	JUC13002708.hg.1	ATP11A
TC13000411.hg.1	2.12	JUC13002715.hg.1	ATP11A
TC13000411.hg.1	2.11	PSR13004502.hg.1	ATP11A
TC14000794.hg.1	3.37	PSR14008566.hg.1	TNFAIP2
TC14000794.hg.1	2.59	PSR14008561.hg.1	TNFAIP2
TC14000794.hg.1	2.47	PSR14008560.hg.1	TNFAIP2
TC14000794.hg.1	2.35	PSR14008587.hg.1	TNFAIP2
TC14000794.hg.1	2.23	PSR14008563.hg.1	TNFAIP2
TC14000794.hg.1	2.15	JUC14004372.hg.1	TNFAIP2
TC14000794.hg.1	2.14	JUC14004373.hg.1	TNFAIP2
TC14000794.hg.1	2.07	PSR14008567.hg.1	TNFAIP2
TC14000794.hg.1	2.07	JUC14004371.hg.1	TNFAIP2
TC14000794.hg.1	-2.16	JUC14004360.hg.1	TNFAIP2
TC14000794.hg.1	-2.3	JUC14004374.hg.1	TNFAIP2
TC01001755.hg.1	3.36	JUC01014837.hg.1	HHAT
TC01001755.hg.1	2.01	PSR01027556.hg.1	HHAT
TC01001755.hg.1	-2.14	JUC01014818.hg.1	HHAT
TC01001755.hg.1	-2.3	PSR01027539.hg.1	HHAT
TC02000432.hg.1	3.36	JUC02003410.hg.1	DYSF
TC02000432.hg.1	2.73	JUC02003416.hg.1	DYSF
TC02000432.hg.1	2.55	JUC02003426.hg.1	DYSF
TC02000432.hg.1	2.39	JUC02003372.hg.1	DYSF
TC02000432.hg.1	2.29	JUC02003393.hg.1	DYSF
TC02000432.hg.1	2.28	JUC02003361.hg.1	DYSF
TC02000432.hg.1	2.19	JUC02003391.hg.1	DYSF
TC02000432.hg.1	2.17	JUC02003385.hg.1	DYSF
TC02000432.hg.1	2.13	JUC02003392.hg.1	DYSF
TC02000432.hg.1	2.1	PSR02006601.hg.1	DYSF
TC02000432.hg.1	2.02	PSR02006570.hg.1	DYSF
TC02000432.hg.1	2	JUC02003364.hg.1	DYSF
TC02000432.hg.1	-2.15	PSR02006597.hg.1	DYSF
TC02000432.hg.1	-2.16	JUC02003419.hg.1	DYSF
TC02000432.hg.1	-2.22	JUC02003386.hg.1	DYSF
TC02000432.hg.1	-2.26	PSR02006562.hg.1	DYSF
TC02000432.hg.1	-2.29	JUC02003428.hg.1	DYSF
TC02000432.hg.1	-2.3	PSR02006565.hg.1	DYSF
TC02000432.hg.1	-2.4	PSR02006618.hg.1	DYSF
TC02000432.hg.1	-2.44	PSR02006620.hg.1	DYSF
TC02000432.hg.1	-2.44	JUC02003368.hg.1	DYSF
TC02000432.hg.1	-2.48	PSR02006631.hg.1	DYSF

TC02000432.hg.1	-2.49	PSR02006599.hg.1	DYSF
TC02000432.hg.1	-2.56	PSR02006561.hg.1	DYSF
TC02000432.hg.1	-2.56	PSR02006585.hg.1	DYSF
TC02000432.hg.1	-2.67	PSR02006612.hg.1	DYSF
TC02000432.hg.1	-2.68	JUC02003369.hg.1	DYSF
TC02000432.hg.1	-2.69	PSR02006593.hg.1	DYSF
TC02000432.hg.1	-2.7	PSR02006611.hg.1	DYSF
TC02000432.hg.1	-2.73	JUC02003403.hg.1	DYSF
TC02000432.hg.1	-2.74	JUC02003394.hg.1	DYSF
TC02000432.hg.1	-2.75	JUC02003415.hg.1	DYSF
TC02000432.hg.1	-2.78	JUC02003380.hg.1	DYSF
TC02000432.hg.1	-2.88	PSR02006616.hg.1	DYSF
TC02000432.hg.1	-3.07	JUC02003360.hg.1	DYSF
TC02000432.hg.1	-3.44	JUC02003379.hg.1	DYSF
TC02000432.hg.1	-3.57	JUC02003397.hg.1	DYSF
TC02000432.hg.1	-3.73	PSR02006600.hg.1	DYSF
TC02000432.hg.1	-4.62	JUC02003383.hg.1	DYSF
TC02000432.hg.1	-4.98	PSR02006607.hg.1	DYSF
TC02000432.hg.1	-5.2	PSR02006609.hg.1	DYSF
TC02000432.hg.1	-7.04	JUC02003376.hg.1	DYSF
TC08000119.hg.1	3.36	JUC08000553.hg.1	TUSC3
TC08000119.hg.1	2.93	PSR08001057.hg.1	TUSC3
TC08000119.hg.1	2.58	JUC08000567.hg.1	TUSC3
TC08000119.hg.1	2.3	PSR08001050.hg.1	TUSC3
TC08000119.hg.1	2.04	PSR08001048.hg.1	TUSC3
TC08000119.hg.1	-2.1	PSR08001079.hg.1	TUSC3
TC08000119.hg.1	-2.6	JUC08000570.hg.1	TUSC3
TC09001345.hg.1	3.36	PSR09016848.hg.1	NINJ1
TC09001345.hg.1	3.36	PSR09016857.hg.1	NINJ1
TC09001345.hg.1	3.2	PSR09016851.hg.1	NINJ1
TC09001345.hg.1	2.63	JUC09009051.hg.1	NINJ1
TC09001345.hg.1	2.35	JUC09009054.hg.1	NINJ1
TC09001345.hg.1	2.28	JUC09009056.hg.1	NINJ1
TC09001345.hg.1	2.09	JUC09009052.hg.1	NINJ1
TC19001913.hg.1	3.36	JUC19014668.hg.1	
TC19001913.hg.1	2.84	PSR19025917.hg.1	
TC19001913.hg.1	2	JUC19014667.hg.1	
TC01002627.hg.1	3.35	JUC01021996.hg.1	PDZK1IP1
TC01002627.hg.1	2.69	JUC01021995.hg.1	PDZK1IP1
TC01002627.hg.1	2.05	PSR01041120.hg.1	PDZK1IP1
TC01003887.hg.1	3.35	JUC01031048.hg.1	LBR
TC01003887.hg.1	2.7	JUC01031036.hg.1	LBR
TC01003887.hg.1	2.09	JUC01031038.hg.1	LBR
TC01003887.hg.1	-2	JUC01031047.hg.1	LBR
TC01003887.hg.1	-2.13	PSR01059228.hg.1	LBR
TC01003887.hg.1	-2.38	PSR01059225.hg.1	LBR
TC01003887.hg.1	-2.4	PSR01059226.hg.1	LBR
TC01003887.hg.1	-2.58	PSR01059224.hg.1	LBR
TC01003887.hg.1	-2.97	PSR01059221.hg.1	LBR
TC01003887.hg.1	-3.82	JUC01031044.hg.1	LBR
TC01006385.hg.1	3.35	JUC01046486.hg.1	ACBD6
TC01006385.hg.1	2.15	PSR01055365.hg.1	ACBD6
TC01006385.hg.1	2	PSR01055363.hg.1	ACBD6
TC01006385.hg.1	-2.14	PSR01055356.hg.1	ACBD6
TC02001013.hg.1	3.35	PSR02014955.hg.1	LOC1005056
TC02001013.hg.1	2.75	JUC02007986.hg.1	LOC1005056
TC02001013.hg.1	2.11	PSR02014952.hg.1	LOC1005056
TC02001013.hg.1	2.04	PSR02014957.hg.1	LOC1005056

TC03001422.hg.1	3.35	JUC03012554.hg.1	MON1A
TC03001422.hg.1	-2.67	PSR03025530.hg.1	MON1A
TC03001796.hg.1	3.35	JUC03016141.hg.1	TOPBP1
TC03001796.hg.1	2.1	PSR03032568.hg.1	TOPBP1
TC05001731.hg.1	3.35	JUC05012055.hg.1	ZNF608
TC05001731.hg.1	-2.31	PSR05023316.hg.1	ZNF608
TC11001938.hg.1	3.35	JUC11012268.hg.1	MAP3K11
TC11001938.hg.1	2.45	PSR11023237.hg.1	MAP3K11
TC12000187.hg.1	3.35	PSR12002355.hg.1	KIAA1467
TC12000187.hg.1	2.08	PSR12002348.hg.1	KIAA1467
TC12000187.hg.1	-3.11	JUC12001092.hg.1	KIAA1467
TC12000187.hg.1	-3.14	JUC12001085.hg.1	KIAA1467
TC01000902.hg.1	3.34	JUC01007509.hg.1	CDC14A
TC01000902.hg.1	-2.08	PSR01014234.hg.1	CDC14A
TC01000902.hg.1	-2.21	PSR01014198.hg.1	CDC14A
TC01000902.hg.1	-2.32	PSR01014213.hg.1	CDC14A
TC02000611.hg.1	3.34	JUC02005079.hg.1	NPAS2
TC02000611.hg.1	2.43	JUC02005097.hg.1	NPAS2
TC02000611.hg.1	2.08	PSR02009641.hg.1	NPAS2
TC02002292.hg.1	3.34	PSR02036866.hg.1	HS6ST1
TC02002292.hg.1	3	PSR02036875.hg.1	HS6ST1
TC02002292.hg.1	2.85	JUC02019120.hg.1	HS6ST1
TC02002292.hg.1	2.5	PSR02036872.hg.1	HS6ST1
TC02002292.hg.1	2.37	PSR02036865.hg.1	HS6ST1
TC03000241.hg.1	3.34	JUC03002472.hg.1	ZNF501
TC03000241.hg.1	2.07	PSR03004856.hg.1	ZNF501
TC03000241.hg.1	2.07	PSR03004868.hg.1	ZNF501
TC07000453.hg.1	3.34	JUC07002963.hg.1	LAT2
TC07000453.hg.1	2.09	PSR07006138.hg.1	LAT2
TC07000453.hg.1	-2.23	PSR07006142.hg.1	LAT2
TC07000453.hg.1	-3.28	JUC07002975.hg.1	LAT2
TC07000668.hg.1	3.34	JUC07004829.hg.1	LRRC17
TC07000668.hg.1	2.41	JUC07004827.hg.1	LRRC17
TC07000668.hg.1	-2.13	PSR07010375.hg.1	LRRC17
TC07000668.hg.1	-2.33	PSR07010376.hg.1	LRRC17
TC0X000264.hg.1	3.34	JUC0X001794.hg.1	CCDC120
TC0X000264.hg.1	2.48	PSR0X003427.hg.1	CCDC120
TC10001363.hg.1	3.34	PSR10017254.hg.1	NPFFR1
TC10001363.hg.1	3.06	JUC10010062.hg.1	NPFFR1
TC12000309.hg.1	3.34	JUC12001943.hg.1	LRRK2
TC12000309.hg.1	2.6	JUC12001904.hg.1	LRRK2
TC12000309.hg.1	2.41	JUC12001919.hg.1	LRRK2
TC12000309.hg.1	2.34	JUC12001935.hg.1	LRRK2
TC12000309.hg.1	-2.13	PSR12003726.hg.1	LRRK2
TC12000309.hg.1	-2.3	JUC12001913.hg.1	LRRK2
TC12000309.hg.1	-2.41	JUC12001953.hg.1	LRRK2
TC17000161.hg.1	3.34	JUC17001377.hg.1	CDRT15P1
TC17000161.hg.1	-2.01	JUC17001378.hg.1	CDRT15P1
TC17000161.hg.1	-2.14	PSR17002274.hg.1	CDRT15P1
TC20000048.hg.1	3.34	PSR20000781.hg.1	SMOX
TC20000048.hg.1	3.02	PSR20000796.hg.1	SMOX
TC20000048.hg.1	2.89	JUC20000368.hg.1	SMOX
TC20000048.hg.1	2.75	JUC20000369.hg.1	SMOX
TC20000048.hg.1	2.74	PSR20000798.hg.1	SMOX
TC20000048.hg.1	2.63	PSR20000782.hg.1	SMOX
TC20000048.hg.1	2.56	JUC20000386.hg.1	SMOX
TC20000048.hg.1	2.55	PSR20000794.hg.1	SMOX
TC20000048.hg.1	2.32	PSR20000779.hg.1	SMOX

TC20000048.hg.1	2.28	PSR20000778.hg.1	SMOX
TC20000048.hg.1	2.27	PSR20000785.hg.1	SMOX
TC20000048.hg.1	2.23	PSR20000780.hg.1	SMOX
TC20000048.hg.1	-2.39	JUC20000377.hg.1	SMOX
TC22000342.hg.1	3.34	JUC22002481.hg.1	MEI1
TC22000342.hg.1	2.88	JUC22002456.hg.1	MEI1
TC22000342.hg.1	2.02	PSR22006600.hg.1	MEI1
TC22000342.hg.1	-2.28	PSR22006649.hg.1	MEI1
TC22000342.hg.1	-2.28	JUC22002483.hg.1	MEI1
TC22000798.hg.1	3.34	PSR22013562.hg.1	NPTXR
TC22000798.hg.1	2.51	JUC22005588.hg.1	NPTXR
TC22000798.hg.1	2.18	PSR22013561.hg.1	NPTXR
TC02001309.hg.1	3.33	JUC02010833.hg.1	STK36
TC02001309.hg.1	2.97	JUC02010816.hg.1	STK36
TC02001309.hg.1	2.28	JUC02010834.hg.1	STK36
TC02001309.hg.1	-2.37	PSR02020179.hg.1	STK36
TC02001309.hg.1	-2.39	JUC02010836.hg.1	STK36
TC02001758.hg.1	3.33	JUC02014838.hg.1	DHX57
TC02001758.hg.1	2.93	JUC02014833.hg.1	DHX57
TC02001758.hg.1	2.6	JUC02014841.hg.1	DHX57
TC02001758.hg.1	2.41	JUC02014828.hg.1	DHX57
TC02001758.hg.1	2.09	JUC02014830.hg.1	DHX57
TC02001758.hg.1	-2.15	PSR02028355.hg.1	DHX57
TC02001758.hg.1	-3.76	JUC02014839.hg.1	DHX57
TC02002848.hg.1	3.33	JUC02024034.hg.1	TRIP12
TC02002848.hg.1	2.1	PSR02045946.hg.1	TRIP12
TC02002848.hg.1	2.05	PSR02046020.hg.1	TRIP12
TC02002848.hg.1	-2.67	JUC02024036.hg.1	TRIP12
TC03000282.hg.1	3.33	PSR03005678.hg.1	TREX1, ATRI
TC03000282.hg.1	3.12	JUC03002869.hg.1	TREX1, ATRI
TC03000282.hg.1	2.6	PSR03005664.hg.1	TREX1, ATRI
TC03000282.hg.1	2.37	JUC03002879.hg.1	TREX1, ATRI
TC03000282.hg.1	2.36	JUC03002862.hg.1	TREX1, ATRI
TC03000282.hg.1	2.33	JUC03002882.hg.1	TREX1, ATRI
TC03000282.hg.1	2.31	PSR03005674.hg.1	TREX1, ATRI
TC03000282.hg.1	2.11	JUC03002877.hg.1	TREX1, ATRI
TC03000282.hg.1	2	PSR03005675.hg.1	TREX1, ATRI
TC04000447.hg.1	3.33	PSR04006416.hg.1	PRDM8
TC04000447.hg.1	3.25	JUC04003263.hg.1	PRDM8
TC04000447.hg.1	3.11	JUC04003270.hg.1	PRDM8
TC04000447.hg.1	2.64	JUC04003268.hg.1	PRDM8
TC04000447.hg.1	2.59	PSR04006407.hg.1	PRDM8
TC04000447.hg.1	2.45	PSR04006411.hg.1	PRDM8
TC04000447.hg.1	2.2	PSR04006428.hg.1	PRDM8
TC08002616.hg.1	3.33	PSR08017432.hg.1	MRPS28
TC08002616.hg.1	2.57	JUC08015255.hg.1	MRPS28
TC09001012.hg.1	3.33	JUC09007229.hg.1	AQP3
TC09001012.hg.1	3.12	JUC09007233.hg.1	AQP3
TC09001012.hg.1	-2.99	PSR09013299.hg.1	AQP3
TC10000039.hg.1	3.33	JUC10000177.hg.1	AKR1E2
TC10000039.hg.1	-2.02	PSR10000315.hg.1	AKR1E2
TC10000039.hg.1	-2.27	PSR10000304.hg.1	AKR1E2
TC10000039.hg.1	-2.61	JUC10000181.hg.1	AKR1E2
TC10000039.hg.1	-2.75	JUC10000171.hg.1	AKR1E2
TC10000039.hg.1	-4.21	JUC10000168.hg.1	AKR1E2
TC10000181.hg.1	3.33	JUC10001176.hg.1	APBB1IP
TC10000181.hg.1	3.18	JUC10001175.hg.1	APBB1IP
TC10000181.hg.1	2.14	PSR10002163.hg.1	APBB1IP

TC10000181.hg.1	-2.05	PSR10002177.hg.1	APBB1IP
TC10000181.hg.1	-2.11	JUC10001179.hg.1	APBB1IP
TC10000181.hg.1	-2.15	PSR10002173.hg.1	APBB1IP
TC10000181.hg.1	-2.18	JUC10001182.hg.1	APBB1IP
TC11003444.hg.1	3.33	JUC11018963.hg.1	DNHD1
TC11003444.hg.1	3.14	JUC11018991.hg.1	DNHD1
TC11003444.hg.1	2.47	JUC11018996.hg.1	DNHD1
TC11003444.hg.1	2.46	JUC11018969.hg.1	DNHD1
TC11003444.hg.1	2.42	PSR11001936.hg.1	DNHD1
TC11003444.hg.1	2.31	PSR11001955.hg.1	DNHD1
TC11003444.hg.1	2.3	JUC11018992.hg.1	DNHD1
TC11003444.hg.1	2.19	JUC11018972.hg.1	DNHD1
TC11003444.hg.1	2.12	PSR11001967.hg.1	DNHD1
TC11003444.hg.1	2.04	JUC11019010.hg.1	DNHD1
TC11003444.hg.1	-2.17	PSR11001895.hg.1	DNHD1
TC11003444.hg.1	-2.33	JUC11018967.hg.1	DNHD1
TC11003444.hg.1	-2.34	PSR11001928.hg.1	DNHD1
TC14001446.hg.1	3.33	JUC14008334.hg.1	ATXN3
TC14001446.hg.1	3.11	JUC14008339.hg.1	ATXN3
TC14001446.hg.1	3.02	JUC14008350.hg.1	ATXN3
TC14001446.hg.1	2.89	JUC14008383.hg.1	ATXN3
TC14001446.hg.1	2.86	JUC14008372.hg.1	ATXN3
TC14001446.hg.1	2.21	JUC14008345.hg.1	ATXN3
TC14001446.hg.1	2.2	JUC14008342.hg.1	ATXN3
TC14001446.hg.1	-2.02	PSR14016185.hg.1	ATXN3
TC14001446.hg.1	-2.02	PSR14016214.hg.1	ATXN3
TC14001446.hg.1	-2.08	JUC14008327.hg.1	ATXN3
TC14001446.hg.1	-2.09	PSR14016232.hg.1	ATXN3
TC14001446.hg.1	-2.15	JUC14008356.hg.1	ATXN3
TC14001446.hg.1	-2.21	PSR14016213.hg.1	ATXN3
TC14001446.hg.1	-2.41	JUC14008373.hg.1	ATXN3
TC14001446.hg.1	-2.72	JUC14008377.hg.1	ATXN3
TC19000662.hg.1	3.33	PSR19009274.hg.1	HIF3A
TC19000662.hg.1	2.78	PSR19009241.hg.1	HIF3A
TC19000662.hg.1	2.15	JUC19005494.hg.1	HIF3A
TC19000662.hg.1	-2.3	JUC19005507.hg.1	HIF3A
TC19000662.hg.1	-2.41	JUC19005498.hg.1	HIF3A
TC19000662.hg.1	-2.81	PSR19009236.hg.1	HIF3A
TC6_apd_hap1000	3.33	JUC6_apd_hap1000	HLA-DRB1, H
TC6_apd_hap1000	-3.53	PSR6_apd_hap1001	HLA-DRB1, H
TC03001732.hg.1	3.32	JUC03015677.hg.1	SLC41A3
TC03001732.hg.1	2.66	PSR03031516.hg.1	SLC41A3
TC03001732.hg.1	2.51	PSR03031494.hg.1	SLC41A3
TC03001732.hg.1	2.48	PSR03031520.hg.1	SLC41A3
TC03001732.hg.1	2.47	PSR03031495.hg.1	SLC41A3
TC03001732.hg.1	2.36	PSR03031492.hg.1	SLC41A3
TC03001732.hg.1	2.32	JUC03015678.hg.1	SLC41A3
TC03001732.hg.1	2.14	PSR03031482.hg.1	SLC41A3
TC03001732.hg.1	2.06	PSR03031508.hg.1	SLC41A3
TC03001732.hg.1	2.03	PSR03031515.hg.1	SLC41A3
TC04001504.hg.1	3.32	JUC04011205.hg.1	PDE5A
TC04001504.hg.1	2.95	JUC04011207.hg.1	PDE5A
TC04001504.hg.1	2.1	PSR04021330.hg.1	PDE5A
TC04001504.hg.1	2.06	PSR04021302.hg.1	PDE5A
TC04001504.hg.1	2.04	JUC04011203.hg.1	PDE5A
TC04001504.hg.1	-2.14	JUC04011217.hg.1	PDE5A
TC06004066.hg.1	3.32	PSR06002626.hg.1	HLA-H
TC06004066.hg.1	2.89	PSR06002632.hg.1	HLA-H

TC06004066.hg.1	2.24	PSR06002623.hg.1	HLA-H
TC06004066.hg.1	2.21	JUC06020844.hg.1	HLA-H
TC08001400.hg.1	3.32	PSR08018143.hg.1	NBN
TC08001400.hg.1	-2.38	JUC08009238.hg.1	NBN
TC08001400.hg.1	-2.49	JUC08009252.hg.1	NBN
TC08001400.hg.1	-2.97	JUC08009230.hg.1	NBN
TC09001434.hg.1	3.32	JUC09009638.hg.1	ABCA1
TC09001434.hg.1	2.92	JUC09009616.hg.1	ABCA1
TC09001434.hg.1	2.27	PSR09017895.hg.1	ABCA1
TC09001434.hg.1	2.05	PSR09017851.hg.1	ABCA1
TC09001434.hg.1	2.05	JUC09009607.hg.1	ABCA1
TC09001434.hg.1	-2.13	PSR09017842.hg.1	ABCA1
TC09001434.hg.1	-2.19	JUC09009640.hg.1	ABCA1
TC0X000425.hg.1	3.32	PSR0X005622.hg.1	PGK1, LOC1(
TC0X000425.hg.1	2.87	PSR0X005619.hg.1	PGK1, LOC1(
TC0X000425.hg.1	2.68	JUC0X002787.hg.1	PGK1, LOC1(
TC0X000425.hg.1	2.2	PSR0X005623.hg.1	PGK1, LOC1(
TC0X000425.hg.1	-2.02	PSR0X005656.hg.1	PGK1, LOC1(
TC0X000425.hg.1	-2.12	PSR0X005655.hg.1	PGK1, LOC1(
TC0X000425.hg.1	-2.94	PSR0X005646.hg.1	PGK1, LOC1(
TC0X000744.hg.1	3.32	JUC0X004879.hg.1	ZNF185
TC0X000744.hg.1	-2.08	PSR0X009656.hg.1	ZNF185
TC0X000744.hg.1	-2.11	JUC0X004865.hg.1	ZNF185
TC0X000744.hg.1	-2.12	PSR0X009643.hg.1	ZNF185
TC0X000744.hg.1	-2.2	JUC0X004869.hg.1	ZNF185
TC0X000744.hg.1	-2.4	PSR0X009655.hg.1	ZNF185
TC0X000744.hg.1	-3.3	JUC0X004899.hg.1	ZNF185
TC10000896.hg.1	3.32	JUC10006405.hg.1	ACADSB
TC10000896.hg.1	2.44	JUC10006408.hg.1	ACADSB
TC10000896.hg.1	2.21	PSR10010979.hg.1	ACADSB
TC10000896.hg.1	2.18	PSR10010984.hg.1	ACADSB
TC10000896.hg.1	2	JUC10006402.hg.1	ACADSB
TC12000264.hg.1	3.32	JUC12001593.hg.1	ARNTL2
TC12000264.hg.1	2.29	PSR12003196.hg.1	ARNTL2
TC12000264.hg.1	2.07	JUC12001588.hg.1	ARNTL2
TC12000264.hg.1	-2.08	JUC12001594.hg.1	ARNTL2
TC12000264.hg.1	-2.11	PSR12003198.hg.1	ARNTL2
TC12000264.hg.1	-2.43	JUC12001584.hg.1	ARNTL2
TC12001272.hg.1	3.32	PSR12016566.hg.1	HIST4H4, HIS
TC12001272.hg.1	2.88	PSR12016568.hg.1	HIST4H4, HIS
TC12001272.hg.1	2.36	JUC12009088.hg.1	HIST4H4, HIS
TC12001272.hg.1	2.07	PSR12016565.hg.1	HIST4H4, HIS
TC12001804.hg.1	3.32	JUC12013227.hg.1	DCN
TC12001804.hg.1	2.02	PSR12023858.hg.1	DCN
TC12001804.hg.1	-2.35	PSR12023867.hg.1	DCN
TC12001804.hg.1	-2.81	PSR12023871.hg.1	DCN
TC12001804.hg.1	-3.91	JUC12013229.hg.1	DCN
TC16001270.hg.1	3.32	PSR16017682.hg.1	MLKL
TC16001270.hg.1	-2.14	JUC16010066.hg.1	MLKL
TC16001270.hg.1	-2.49	PSR16017684.hg.1	MLKL
TC01002924.hg.1	3.31	JUC01024428.hg.1	DPH5
TC01002924.hg.1	-2.33	PSR01045426.hg.1	DPH5
TC01002924.hg.1	-2.35	JUC01024444.hg.1	DPH5
TC01002924.hg.1	-3.8	JUC01024433.hg.1	DPH5
TC05001041.hg.1	3.31	JUC05007867.hg.1	ZNF354C
TC05001041.hg.1	2.79	PSR05015195.hg.1	ZNF354C
TC05001041.hg.1	2.13	JUC05007868.hg.1	ZNF354C
TC05001041.hg.1	2.06	JUC05007863.hg.1	ZNF354C

TC05001880.hg.1	3.31	PSR05025988.hg.1	SPRY4
TC05001880.hg.1	3.28	PSR05026004.hg.1	SPRY4
TC05001880.hg.1	2.51	JUC05013249.hg.1	SPRY4
TC05001880.hg.1	2.51	JUC05013256.hg.1	SPRY4
TC05001880.hg.1	2.45	JUC05013250.hg.1	SPRY4
TC05001880.hg.1	2.03	PSR05025987.hg.1	SPRY4
TC05001880.hg.1	-2.32	JUC05013251.hg.1	SPRY4
TC05001880.hg.1	-2.75	PSR05025993.hg.1	SPRY4
TC05001880.hg.1	-2.88	PSR05025986.hg.1	SPRY4
TC05001880.hg.1	-3.15	PSR05025982.hg.1	SPRY4
TC11002017.hg.1	3.31	JUC11012772.hg.1	ORAOV1
TC11002017.hg.1	-3.32	PSR11024187.hg.1	ORAOV1
TC11002017.hg.1	-4.2	JUC11012773.hg.1	ORAOV1
TC13000453.hg.1	3.31	JUC13003104.hg.1	PSPC1, PSP(
TC13000453.hg.1	-2.05	PSR13005142.hg.1	PSPC1, PSP(
TC13000453.hg.1	-4.03	PSR13005165.hg.1	PSPC1, PSP(
TC14000414.hg.1	3.31	JUC14002532.hg.1	GALNTL1
TC14000414.hg.1	2.1	PSR14004967.hg.1	GALNTL1
TC16000218.hg.1	3.31	JUC16001762.hg.1	ACSM5
TC16000218.hg.1	2.88	JUC16001767.hg.1	ACSM5
TC16000218.hg.1	2.05	PSR16003262.hg.1	ACSM5
TC6_cox_hap2000	3.31	PSR6_cox_hap2000	HLA-F
TC6_cox_hap2000	3.15	PSR6_cox_hap2000	HLA-F
TC6_cox_hap2000	2.91	JUC6_cox_hap2000(	HLA-F
TC6_cox_hap2000	2.79	JUC6_cox_hap2000(	HLA-F
TC6_cox_hap2000	2.68	JUC6_cox_hap2000(	HLA-F
TC6_cox_hap2000	2.34	PSR6_cox_hap2000	HLA-F
TC6_cox_hap2000	2.26	JUC6_cox_hap2000(	HLA-F
TC6_cox_hap2000	2.25	JUC6_cox_hap2000(	HLA-F
TC6_cox_hap2000	2.14	PSR6_cox_hap2000	HLA-F
TC6_cox_hap2000	2.09	JUC6_cox_hap2000(	HLA-F
TC6_cox_hap2000	2.02	PSR6_cox_hap2000	HLA-F
TC02001625.hg.1	3.3	JUC02013529.hg.1	HS1BP3
TC02001625.hg.1	-2.16	JUC02013537.hg.1	HS1BP3
TC02001625.hg.1	-2.48	PSR02026013.hg.1	HS1BP3
TC02002745.hg.1	3.3	JUC02023081.hg.1	BARD1
TC02002745.hg.1	2.01	PSR02044020.hg.1	BARD1
TC03001532.hg.1	3.3	PSR03027921.hg.1	LRIG1
TC03001532.hg.1	3.21	JUC03013717.hg.1	LRIG1
TC03001532.hg.1	2.71	PSR03027920.hg.1	LRIG1
TC03001532.hg.1	2.59	JUC03013699.hg.1	LRIG1
TC03001532.hg.1	2.34	PSR03027898.hg.1	LRIG1
TC03001532.hg.1	2.22	PSR03027911.hg.1	LRIG1
TC03001532.hg.1	2.07	PSR03027902.hg.1	LRIG1
TC03001532.hg.1	2.05	PSR03027900.hg.1	LRIG1
TC06002126.hg.1	3.3	PSR06026704.hg.1	SGK1
TC06002126.hg.1	2.75	PSR06026706.hg.1	SGK1
TC06002126.hg.1	2.69	PSR06026697.hg.1	SGK1
TC06002126.hg.1	2.02	PSR06026698.hg.1	SGK1
TC06002126.hg.1	-2.03	JUC06013218.hg.1	SGK1
TC06002126.hg.1	-2.07	PSR06026727.hg.1	SGK1
TC06002126.hg.1	-2.08	PSR06026734.hg.1	SGK1
TC06002126.hg.1	-2.09	PSR06026732.hg.1	SGK1
TC06002126.hg.1	-2.15	JUC06013219.hg.1	SGK1
TC06002126.hg.1	-2.16	JUC06013232.hg.1	SGK1
TC06002126.hg.1	-2.27	PSR06026742.hg.1	SGK1
TC12000424.hg.1	3.3	PSR12005416.hg.1	KRT18
TC12000424.hg.1	2.31	JUC12002982.hg.1	KRT18

TC12000424.hg.1	2.19	PSR12005400.hg.1	KRT18
TC12000424.hg.1	2.07	PSR12005401.hg.1	KRT18
TC12000890.hg.1	3.3	JUC12006487.hg.1	TPCN1
TC12000890.hg.1	2.52	JUC12006501.hg.1	TPCN1
TC12000890.hg.1	2.35	PSR12011801.hg.1	TPCN1
TC12000890.hg.1	2.23	PSR12011770.hg.1	TPCN1
TC12000890.hg.1	-2.21	JUC12006474.hg.1	TPCN1
TC13000845.hg.1	3.3	JUC13005606.hg.1	NALCN
TC13000845.hg.1	2.9	JUC13005609.hg.1	NALCN
TC13000845.hg.1	2.14	PSR13009460.hg.1	NALCN
TC13000845.hg.1	-2.11	JUC13005581.hg.1	NALCN
TC13000845.hg.1	-2.19	JUC13005587.hg.1	NALCN
TC13000845.hg.1	-2.22	PSR13009489.hg.1	NALCN
TC13000845.hg.1	-2.25	JUC13005595.hg.1	NALCN
TC13000845.hg.1	-2.3	PSR13009485.hg.1	NALCN
TC13000845.hg.1	-2.34	PSR13009483.hg.1	NALCN
TC13000845.hg.1	-2.54	PSR13009461.hg.1	NALCN
TC13000845.hg.1	-2.59	PSR13009514.hg.1	NALCN
TC13000845.hg.1	-2.96	JUC13005571.hg.1	NALCN
TC13000845.hg.1	-3	JUC13005599.hg.1	NALCN
TC14001117.hg.1	3.3	JUC14006324.hg.1	L2HGDH
TC14001117.hg.1	2.52	PSR14012591.hg.1	L2HGDH
TC14001117.hg.1	2.14	JUC14006328.hg.1	L2HGDH
TC14001117.hg.1	2.04	JUC14006331.hg.1	L2HGDH
TC14001117.hg.1	-2.37	JUC14006336.hg.1	L2HGDH
TC02000237.hg.1	3.29	JUC02001856.hg.1	QPCT
TC02000237.hg.1	2.09	JUC02001851.hg.1	QPCT
TC02000237.hg.1	-2.48	PSR02003548.hg.1	QPCT
TC02000237.hg.1	-3.73	JUC02001850.hg.1	QPCT
TC02000237.hg.1	-4.18	PSR02003536.hg.1	QPCT
TC02000237.hg.1	-5.44	PSR02003549.hg.1	QPCT
TC02000237.hg.1	-5.66	PSR02003532.hg.1	QPCT
TC02000237.hg.1	-6.52	PSR02003544.hg.1	QPCT
TC04001741.hg.1	3.29	PSR04024051.hg.1	HMGB2
TC04001741.hg.1	3.01	JUC04012753.hg.1	HMGB2
TC04001741.hg.1	2.94	PSR04024061.hg.1	HMGB2
TC04001741.hg.1	2.81	PSR04024052.hg.1	HMGB2
TC04001741.hg.1	2.8	PSR04024057.hg.1	HMGB2
TC04001741.hg.1	2.63	JUC04012758.hg.1	HMGB2
TC04001741.hg.1	2.47	JUC04012755.hg.1	HMGB2
TC04001741.hg.1	2.24	PSR04024038.hg.1	HMGB2
TC04001741.hg.1	2.09	JUC04012754.hg.1	HMGB2
TC04001741.hg.1	2.08	PSR04024059.hg.1	HMGB2
TC05000287.hg.1	3.29	JUC05002024.hg.1	
TC05000287.hg.1	3.04	JUC05002022.hg.1	
TC05000287.hg.1	2.1	JUC05002023.hg.1	
TC05000287.hg.1	-4.39	PSR05003760.hg.1	
TC05000458.hg.1	3.29	JUC05003577.hg.1	ARSK
TC05000458.hg.1	2.82	PSR05006472.hg.1	ARSK
TC05000458.hg.1	2.76	JUC05003579.hg.1	ARSK
TC05000700.hg.1	3.29	PSR05009800.hg.1	REEP2
TC05000700.hg.1	3.17	JUC05005215.hg.1	REEP2
TC05000700.hg.1	3.05	JUC05005219.hg.1	REEP2
TC05000700.hg.1	3.04	PSR05009809.hg.1	REEP2
TC05000700.hg.1	2.75	JUC05005220.hg.1	REEP2
TC05000700.hg.1	2.64	PSR05009784.hg.1	REEP2
TC05000700.hg.1	2.62	PSR05009801.hg.1	REEP2
TC05000700.hg.1	2.3	PSR05009795.hg.1	REEP2

TC05000700.hg.1	2.15	PSR05009791.hg.1	REEP2
TC07001798.hg.1	3.29	JUC07013718.hg.1	CTTNBP2
TC07001798.hg.1	2.38	JUC07013727.hg.1	CTTNBP2
TC07001798.hg.1	2.23	JUC07013714.hg.1	CTTNBP2
TC07001798.hg.1	2.08	PSR07027896.hg.1	CTTNBP2
TC11000311.hg.1	3.29	PSR11003974.hg.1	QSER1
TC11000311.hg.1	2.46	JUC11002081.hg.1	QSER1
TC11000311.hg.1	-2.08	JUC11002076.hg.1	QSER1
TC13000433.hg.1	3.29	PSR13004901.hg.1	FLJ44054, LI
TC13000433.hg.1	2.01	JUC13002951.hg.1	FLJ44054, LI
TC20000166.hg.1	3.29	JUC20001107.hg.1	ENTPD6
TC20000166.hg.1	2.39	JUC20001093.hg.1	ENTPD6
TC20000166.hg.1	2.34	JUC20001096.hg.1	ENTPD6
TC20000166.hg.1	-4.19	PSR20002049.hg.1	ENTPD6
TC01003810.hg.1	3.28	JUC01030434.hg.1	NSL1
TC01003810.hg.1	-2.37	PSR01058218.hg.1	NSL1
TC06000747.hg.1	3.28	JUC06004146.hg.1	MYO6
TC06000747.hg.1	2.9	PSR06009021.hg.1	MYO6
TC06000747.hg.1	2.69	JUC06004179.hg.1	MYO6
TC06000747.hg.1	2.28	JUC06004177.hg.1	MYO6
TC06000747.hg.1	-2.04	JUC06004180.hg.1	MYO6
TC08001396.hg.1	3.28	PSR08018079.hg.1	MMP16
TC08001396.hg.1	2.52	JUC08009207.hg.1	MMP16
TC08001396.hg.1	2.43	JUC08009210.hg.1	MMP16
TC08001396.hg.1	2.31	JUC08009212.hg.1	MMP16
TC08001396.hg.1	2.04	PSR08018077.hg.1	MMP16
TC15002793.hg.1	3.28	JUC15013951.hg.1	CAPN3
TC15002793.hg.1	-2.01	PSR15002538.hg.1	CAPN3
TC15002793.hg.1	-2.03	PSR15002549.hg.1	CAPN3
TC15002793.hg.1	-2.2	PSR15002541.hg.1	CAPN3
TC15002793.hg.1	-2.43	PSR15002539.hg.1	CAPN3
TC15002793.hg.1	-2.48	JUC15013933.hg.1	CAPN3
TC15002793.hg.1	-2.56	JUC15013950.hg.1	CAPN3
TC15002793.hg.1	-2.59	JUC15013937.hg.1	CAPN3
TC15002793.hg.1	-2.73	JUC15013956.hg.1	CAPN3
TC15002793.hg.1	-2.83	PSR15002540.hg.1	CAPN3
TC15002793.hg.1	-3.05	JUC15013945.hg.1	CAPN3
TC15002793.hg.1	-3.81	JUC15013963.hg.1	CAPN3
TC19000509.hg.1	3.28	JUC19003979.hg.1	ZNF568
TC19000509.hg.1	2.48	JUC19003976.hg.1	ZNF568
TC19000509.hg.1	2.24	PSR19006760.hg.1	ZNF568
TC19000509.hg.1	2.13	JUC19003981.hg.1	ZNF568
TC19000509.hg.1	-2.01	PSR19006768.hg.1	ZNF568
TC19000509.hg.1	-3.33	JUC19003974.hg.1	ZNF568
TC04001436.hg.1	3.27	JUC04010586.hg.1	CENPE
TC04001436.hg.1	3.03	JUC04010599.hg.1	CENPE
TC04001436.hg.1	2.82	JUC04010555.hg.1	CENPE
TC04001436.hg.1	2.66	JUC04010590.hg.1	CENPE
TC04001436.hg.1	2.22	JUC04010576.hg.1	CENPE
TC04001436.hg.1	2.16	PSR04020227.hg.1	CENPE
TC04001436.hg.1	2.14	JUC04010559.hg.1	CENPE
TC04001436.hg.1	2.13	JUC04010575.hg.1	CENPE
TC04001436.hg.1	2.04	PSR04020232.hg.1	CENPE
TC04001436.hg.1	-2.77	JUC04010583.hg.1	CENPE
TC05001605.hg.1	3.27	PSR05021886.hg.1	TTC37
TC05001605.hg.1	-2.02	JUC05011262.hg.1	TTC37
TC05001605.hg.1	-2.38	PSR05021877.hg.1	TTC37
TC05001605.hg.1	-2.65	JUC05011292.hg.1	TTC37

TC05001605.hg.1	-3.72	PSR05021873.hg.1	TTC37
TC06000322.hg.1	3.27	JUC06001150.hg.1	MOG
TC06000322.hg.1	2.27	PSR06002521.hg.1	MOG
TC07000599.hg.1	3.27	JUC07004187.hg.1	ZNF498
TC07000599.hg.1	2.09	PSR07008964.hg.1	ZNF498
TC07003296.hg.1	3.27	PSR07015219.hg.1	ZNF775, LOC
TC07003296.hg.1	2.7	PSR07015217.hg.1	ZNF775, LOC
TC07003296.hg.1	2.65	JUC07020419.hg.1	ZNF775, LOC
TC07003296.hg.1	2.6	PSR07015213.hg.1	ZNF775, LOC
TC07003296.hg.1	2.05	PSR07015216.hg.1	ZNF775, LOC
TC07003296.hg.1	2.02	PSR07015229.hg.1	ZNF775, LOC
TC10000322.hg.1	3.27	JUC10002141.hg.1	MAPK8
TC10000322.hg.1	2.16	JUC10002138.hg.1	MAPK8
TC10000322.hg.1	-2.1	PSR10003685.hg.1	MAPK8
TC10000322.hg.1	-2.21	JUC10002134.hg.1	MAPK8
TC10000322.hg.1	-2.38	JUC10002121.hg.1	MAPK8
TC10000322.hg.1	-4.03	JUC10002135.hg.1	MAPK8
TC11000155.hg.1	3.27	PSR11002193.hg.1	TUB
TC11000155.hg.1	3.2	JUC11000980.hg.1	TUB
TC11000155.hg.1	2.99	PSR11002207.hg.1	TUB
TC11000155.hg.1	2.03	JUC11000975.hg.1	TUB
TC11000155.hg.1	2	PSR11002206.hg.1	TUB
TC11001358.hg.1	3.27	JUC11009132.hg.1	ARFIP2
TC11001358.hg.1	2.15	PSR11017086.hg.1	ARFIP2
TC11002151.hg.1	3.27	JUC11013625.hg.1	PRCP
TC11002151.hg.1	2.25	JUC11013633.hg.1	PRCP
TC11002151.hg.1	2.14	PSR11025680.hg.1	PRCP
TC17_ctg5_hap10(	3.27	JUC17_ctg5_hap100	KANSL1
TC17_ctg5_hap10(	-2.13	PSR17_ctg5_hap100	KANSL1
TC17_ctg5_hap10(	-2.97	JUC17_ctg5_hap100	KANSL1
TC01000500.hg.1	3.26	JUC01004059.hg.1	PPIE
TC01000500.hg.1	2.54	JUC01004042.hg.1	PPIE
TC01000500.hg.1	2.34	PSR01007772.hg.1	PPIE
TC01000500.hg.1	2.12	JUC01004047.hg.1	PPIE
TC01000500.hg.1	2.07	PSR01007755.hg.1	PPIE
TC01000500.hg.1	-2.57	JUC01004045.hg.1	PPIE
TC02001075.hg.1	3.26	JUC02008540.hg.1	TTN-AS1
TC02001075.hg.1	2.98	PSR02016031.hg.1	TTN-AS1
TC02001075.hg.1	2.73	PSR02016039.hg.1	TTN-AS1
TC02001075.hg.1	2.61	PSR02016038.hg.1	TTN-AS1
TC02001075.hg.1	2.54	JUC02008518.hg.1	TTN-AS1
TC02001075.hg.1	2.18	PSR02016032.hg.1	TTN-AS1
TC05000054.hg.1	3.26	PSR05000590.hg.1	ADCY2
TC05000054.hg.1	3.13	JUC05000334.hg.1	ADCY2
TC05000054.hg.1	2.7	PSR05000593.hg.1	ADCY2
TC05000054.hg.1	2.62	PSR05000629.hg.1	ADCY2
TC05000054.hg.1	2.4	JUC05000341.hg.1	ADCY2
TC05000054.hg.1	2.25	PSR05000619.hg.1	ADCY2
TC05000054.hg.1	2.05	PSR05000627.hg.1	ADCY2
TC05000054.hg.1	-2.21	JUC05000335.hg.1	ADCY2
TC05000054.hg.1	-2.3	JUC05000331.hg.1	ADCY2
TC05000054.hg.1	-2.39	JUC05000346.hg.1	ADCY2
TC05000054.hg.1	-2.64	JUC05000338.hg.1	ADCY2
TC05003410.hg.1	3.26	JUC05019420.hg.1	C5orf45
TC05003410.hg.1	2.54	JUC05019423.hg.1	C5orf45
TC05003410.hg.1	-2.09	PSR05029953.hg.1	C5orf45
TC05003410.hg.1	-2.33	PSR05029951.hg.1	C5orf45
TC05003410.hg.1	-2.61	PSR05029954.hg.1	C5orf45

TC09000772.hg.1	3.26	PSR09009903.hg.1	SNORD24, R
TC09000772.hg.1	2.65	PSR09009905.hg.1	SNORD24, R
TC09000772.hg.1	2.53	JUC09005425.hg.1	SNORD24, R
TC09000772.hg.1	2.34	PSR09009912.hg.1	SNORD24, R
TC09000772.hg.1	2.03	PSR09009886.hg.1	SNORD24, R
TC0X000716.hg.1	3.26	JUC0X004638.hg.1	HSFX2, HSF)
TC0X000716.hg.1	2.28	PSR0X009177.hg.1	HSFX2, HSF)
TC10001761.hg.1	3.26	JUC10013434.hg.1	EBF3
TC10001761.hg.1	2.83	PSR10023286.hg.1	EBF3
TC10001761.hg.1	2.59	JUC10013438.hg.1	EBF3
TC10001761.hg.1	2.21	PSR10023268.hg.1	EBF3
TC10001761.hg.1	2.14	PSR10023288.hg.1	EBF3
TC10001761.hg.1	2.1	PSR10023293.hg.1	EBF3
TC10001761.hg.1	2.03	PSR10023278.hg.1	EBF3
TC10001761.hg.1	-2.15	JUC10013426.hg.1	EBF3
TC10001761.hg.1	-2.49	PSR10023279.hg.1	EBF3
TC10001761.hg.1	-2.54	PSR10023280.hg.1	EBF3
TC10001761.hg.1	-4.56	JUC10013424.hg.1	EBF3
TC10001761.hg.1	-5.74	PSR10023274.hg.1	EBF3
TC11000032.hg.1	3.26	JUC11000250.hg.1	PNPLA2
TC11000032.hg.1	2.14	PSR11000460.hg.1	PNPLA2
TC12001930.hg.1	3.26	JUC12014020.hg.1	
TC12001930.hg.1	2.33	PSR12025130.hg.1	
TC12001930.hg.1	2.33	PSR12025131.hg.1	
TC12001930.hg.1	2.07	JUC12014018.hg.1	
TC15001441.hg.1	3.26	JUC15007080.hg.1	DMXL2
TC15001441.hg.1	2.86	JUC15007123.hg.1	DMXL2
TC15001441.hg.1	-2.02	JUC15007087.hg.1	DMXL2
TC15001441.hg.1	-2.12	PSR15013316.hg.1	DMXL2
TC15001441.hg.1	-2.16	JUC15007113.hg.1	DMXL2
TC15001441.hg.1	-2.38	JUC15007125.hg.1	DMXL2
TC15001441.hg.1	-2.79	JUC15007081.hg.1	DMXL2
TC20000482.hg.1	3.26	JUC20003559.hg.1	LSM14B
TC20000482.hg.1	2.36	JUC20003550.hg.1	LSM14B
TC20000482.hg.1	2.32	PSR20006717.hg.1	LSM14B
TC20001723.hg.1	3.26	JUC20009638.hg.1	L3MBTL1
TC20001723.hg.1	2.19	PSR20004480.hg.1	L3MBTL1
TC20001723.hg.1	2.15	JUC20009637.hg.1	L3MBTL1
TC20001723.hg.1	-2.37	PSR20004493.hg.1	L3MBTL1
TC01001848.hg.1	3.25	JUC01015456.hg.1	CNIH3, LOC1
TC01001848.hg.1	2.03	JUC01015442.hg.1	CNIH3, LOC1
TC01001848.hg.1	-2.75	PSR01028722.hg.1	CNIH3, LOC1
TC04000905.hg.1	3.25	PSR04012364.hg.1	ANKRD37
TC04000905.hg.1	3.15	PSR04012357.hg.1	ANKRD37
TC04000905.hg.1	3.13	PSR04012358.hg.1	ANKRD37
TC04000905.hg.1	2.94	PSR04012356.hg.1	ANKRD37
TC04000905.hg.1	2.67	PSR04012359.hg.1	ANKRD37
TC04000905.hg.1	2.42	JUC04006656.hg.1	ANKRD37
TC04000905.hg.1	-2.4	JUC04006658.hg.1	ANKRD37
TC04002955.hg.1	3.25	JUC04017441.hg.1	ARAP2
TC04002955.hg.1	3.14	PSR04015210.hg.1	ARAP2
TC04002955.hg.1	2.49	JUC04017456.hg.1	ARAP2
TC04002955.hg.1	2.36	JUC04017459.hg.1	ARAP2
TC04002955.hg.1	2.07	PSR04015208.hg.1	ARAP2
TC04002955.hg.1	-2.06	PSR04015214.hg.1	ARAP2
TC05001157.hg.1	3.25	JUC05008426.hg.1	FAM173B
TC05001157.hg.1	-2.22	PSR05016559.hg.1	FAM173B
TC07000995.hg.1	3.25	JUC07007375.hg.1	ATP6V0E2

TC07000995.hg.1	2.48	PSR07015115.hg.1	ATP6V0E2
TC07000995.hg.1	2.26	PSR07015104.hg.1	ATP6V0E2
TC07000995.hg.1	2.22	JUC07007380.hg.1	ATP6V0E2
TC07003292.hg.1	3.25	JUC07020372.hg.1	C7orf53
TC07003292.hg.1	-2.08	PSR07011164.hg.1	C7orf53
TC07003292.hg.1	-2.26	PSR07011165.hg.1	C7orf53
TC12001811.hg.1	3.25	PSR12023941.hg.1	EEA1
TC12001811.hg.1	-2.18	JUC12013263.hg.1	EEA1
TC15000152.hg.1	3.25	JUC15000344.hg.1	LOC646278
TC15000152.hg.1	2.21	JUC15000347.hg.1	LOC646278
TC15000152.hg.1	2.04	PSR15000956.hg.1	LOC646278
TC15000152.hg.1	-2.73	JUC15000340.hg.1	LOC646278
TC17001301.hg.1	3.25	JUC17009773.hg.1	ALDOC
TC17001301.hg.1	3.25	JUC17009776.hg.1	ALDOC
TC17001301.hg.1	3.02	JUC17009775.hg.1	ALDOC
TC17001301.hg.1	3	PSR17017294.hg.1	ALDOC
TC17001301.hg.1	2.68	PSR17017292.hg.1	ALDOC
TC17001301.hg.1	2.5	PSR17017280.hg.1	ALDOC
TC17001301.hg.1	2.3	JUC17009768.hg.1	ALDOC
TC17001301.hg.1	2	JUC17009772.hg.1	ALDOC
TC20000092.hg.1	3.25	JUC20000650.hg.1	SPTLC3
TC20000092.hg.1	2.06	JUC20000648.hg.1	SPTLC3
TC20000092.hg.1	-2.49	JUC20000645.hg.1	SPTLC3
TC20000092.hg.1	-2.89	PSR20001262.hg.1	SPTLC3
TC02002110.hg.1	3.24	JUC02017603.hg.1	KANSL3, KIA
TC02002110.hg.1	2.46	JUC02017581.hg.1	KANSL3, KIA
TC02002110.hg.1	2.08	PSR02034200.hg.1	KANSL3, KIA
TC02002110.hg.1	-3.44	JUC02017594.hg.1	KANSL3, KIA
TC02002110.hg.1	-3.98	JUC02017620.hg.1	KANSL3, KIA
TC02002110.hg.1	-4.02	PSR02034175.hg.1	KANSL3, KIA
TC02002110.hg.1	-4.11	JUC02017584.hg.1	KANSL3, KIA
TC04000547.hg.1	3.24	JUC04004185.hg.1	GSTCD
TC04000547.hg.1	2.67	PSR04007948.hg.1	GSTCD
TC04000547.hg.1	2.4	PSR04007961.hg.1	GSTCD
TC04000547.hg.1	2.26	JUC04004187.hg.1	GSTCD
TC04000547.hg.1	2.2	PSR04007972.hg.1	GSTCD
TC04001724.hg.1	3.24	PSR04023858.hg.1	SH3RF1
TC04001724.hg.1	3.13	PSR04023831.hg.1	SH3RF1
TC04001724.hg.1	2.82	PSR04023830.hg.1	SH3RF1
TC04001724.hg.1	2.52	PSR04023856.hg.1	SH3RF1
TC04001724.hg.1	2.42	JUC04012636.hg.1	SH3RF1
TC04001724.hg.1	2.24	PSR04023857.hg.1	SH3RF1
TC04001724.hg.1	-2.36	JUC04012637.hg.1	SH3RF1
TC04001724.hg.1	-2.84	JUC04012626.hg.1	SH3RF1
TC08001371.hg.1	3.24	PSR08017781.hg.1	SNX16
TC08001371.hg.1	-2.4	JUC08009016.hg.1	SNX16
TC18000016.hg.1	3.24	JUC18000128.hg.1	MYL12A
TC18000016.hg.1	2.24	PSR18000174.hg.1	MYL12A
TC6_dbb_hap3000	3.24	JUC6_dbb_hap3001	C4B, C4A, LC
TC6_dbb_hap3000	2.61	PSR6_dbb_hap3001	C4B, C4A, LC
TC6_dbb_hap3000	2.4	JUC6_dbb_hap3001	C4B, C4A, LC
TC6_dbb_hap3000	2.39	JUC6_dbb_hap3001	C4B, C4A, LC
TC6_dbb_hap3000	2.29	PSR6_dbb_hap3001	C4B, C4A, LC
TC6_dbb_hap3000	2.23	PSR6_dbb_hap3001	C4B, C4A, LC
TC6_dbb_hap3000	2.22	PSR6_dbb_hap3001	C4B, C4A, LC
TC6_dbb_hap3000	2.14	JUC6_dbb_hap3001	C4B, C4A, LC
TC6_dbb_hap3000	2.05	PSR6_dbb_hap3001	C4B, C4A, LC
TC6_dbb_hap3000	-2.08	JUC6_dbb_hap3001	C4B, C4A, LC

TC6_dbb_hap3000	-2.34	JUC6_dbb_hap3001	C4B, C4A, LC
TC6_dbb_hap3000	-2.93	JUC6_dbb_hap3001	C4B, C4A, LC
TC6_dbb_hap3000	-2.93	JUC6_dbb_hap3001	C4B, C4A, LC
TC6_dbb_hap3000	-3.13	JUC6_dbb_hap3001	C4B, C4A, LC
TC6_dbb_hap3000	-3.74	JUC6_dbb_hap3001	C4B, C4A, LC
TC6_mcf_hap5000	3.24	PSR6_mcf_hap5000	HLA-F
TC6_mcf_hap5000	2.91	PSR6_mcf_hap5000	HLA-F
TC6_mcf_hap5000	2.75	JUC6_mcf_hap5001	HLA-F
TC6_mcf_hap5000	2.4	PSR6_mcf_hap5000	HLA-F
TC6_mcf_hap5000	2.32	JUC6_mcf_hap5001	HLA-F
TC01003006.hg.1	3.23	PSR01046773.hg.1	PTPN22
TC01003006.hg.1	-2.43	JUC01025089.hg.1	PTPN22
TC01003006.hg.1	-2.44	JUC01025095.hg.1	PTPN22
TC02000465.hg.1	3.23	JUC02003702.hg.1	SEMA4F
TC02000465.hg.1	3.23	JUC02003710.hg.1	SEMA4F
TC02000465.hg.1	3.01	PSR02007310.hg.1	SEMA4F
TC02000465.hg.1	2.53	PSR02007308.hg.1	SEMA4F
TC02000465.hg.1	2.45	JUC02003701.hg.1	SEMA4F
TC02000465.hg.1	2.36	JUC02003714.hg.1	SEMA4F
TC02000465.hg.1	2.18	JUC02003706.hg.1	SEMA4F
TC02002211.hg.1	3.23	JUC02018529.hg.1	ANAPC1, LO
TC02002211.hg.1	2.4	JUC02018558.hg.1	ANAPC1, LO
TC02002211.hg.1	-2	JUC02018539.hg.1	ANAPC1, LO
TC02002211.hg.1	-2.12	JUC02018570.hg.1	ANAPC1, LO
TC02002211.hg.1	-2.28	PSR02035909.hg.1	ANAPC1, LO
TC02002211.hg.1	-2.31	JUC02018563.hg.1	ANAPC1, LO
TC02002211.hg.1	-2.32	JUC02018575.hg.1	ANAPC1, LO
TC02002211.hg.1	-3.08	JUC02018559.hg.1	ANAPC1, LO
TC02002211.hg.1	-3.89	JUC02018556.hg.1	ANAPC1, LO
TC03001632.hg.1	3.23	JUC03014610.hg.1	KIAA1524
TC03001632.hg.1	-2.18	JUC03014593.hg.1	KIAA1524
TC03001632.hg.1	-2.2	PSR03029482.hg.1	KIAA1524
TC03001632.hg.1	-2.32	JUC03014599.hg.1	KIAA1524
TC06001201.hg.1	3.23	JUC06007125.hg.1	TBP
TC06001201.hg.1	-2.45	PSR06014353.hg.1	TBP
TC09000906.hg.1	3.23	JUC09006558.hg.1	PTPRD
TC09000906.hg.1	2.24	JUC09006500.hg.1	PTPRD
TC09000906.hg.1	2.23	JUC09006492.hg.1	PTPRD
TC09000906.hg.1	-2.01	PSR09012024.hg.1	PTPRD
TC09000906.hg.1	-2.31	PSR09012020.hg.1	PTPRD
TC09000906.hg.1	-2.44	JUC09006562.hg.1	PTPRD
TC09000906.hg.1	-2.59	PSR09011993.hg.1	PTPRD
TC09000906.hg.1	-2.61	PSR09012050.hg.1	PTPRD
TC09000906.hg.1	-2.89	PSR09012053.hg.1	PTPRD
TC09000906.hg.1	-2.99	PSR09012005.hg.1	PTPRD
TC09000906.hg.1	-3.41	JUC09006536.hg.1	PTPRD
TC09000906.hg.1	-4	JUC09006509.hg.1	PTPRD
TC09000906.hg.1	-4.1	JUC09006521.hg.1	PTPRD
TC09000906.hg.1	-4.19	JUC09006553.hg.1	PTPRD
TC09000906.hg.1	-4.9	JUC09006505.hg.1	PTPRD
TC09000906.hg.1	-5.06	PSR09012055.hg.1	PTPRD
TC09000906.hg.1	-5.4	JUC09006504.hg.1	PTPRD
TC09000906.hg.1	-6.34	PSR09012061.hg.1	PTPRD
TC09000906.hg.1	-6.35	PSR09012062.hg.1	PTPRD
TC09000906.hg.1	-6.45	PSR09012067.hg.1	PTPRD
TC09000906.hg.1	-6.65	PSR09012056.hg.1	PTPRD
TC09000906.hg.1	-7.5	PSR09012060.hg.1	PTPRD
TC09000906.hg.1	-11.12	PSR09012064.hg.1	PTPRD

TC09000906.hg.1	-14.04	JUC09006524.hg.1	PTPRD
TC15001992.hg.1	3.23	PSR15018495.hg.1	ADAMTS17
TC15001992.hg.1	-2	JUC15010063.hg.1	ADAMTS17
TC15001992.hg.1	-2.26	JUC15010076.hg.1	ADAMTS17
TC16000240.hg.1	3.23	JUC16001922.hg.1	C16orf52
TC16000240.hg.1	2.15	JUC16001926.hg.1	C16orf52
TC16000240.hg.1	-2.01	PSR16003501.hg.1	C16orf52
TC16000240.hg.1	-2.01	JUC16001923.hg.1	C16orf52
TC16000240.hg.1	-2.03	PSR16003494.hg.1	C16orf52
TC16000240.hg.1	-2.42	PSR16003500.hg.1	C16orf52
TC16000240.hg.1	-2.54	JUC16001929.hg.1	C16orf52
TC17_ctg5_hap10(	3.23	JUC17_ctg5_hap100	LRRC37A4P
TC17_ctg5_hap10(	2.58	PSR17_ctg5_hap100	LRRC37A4P
TC17_ctg5_hap10(	2.23	JUC17_ctg5_hap100	LRRC37A4P
TC17_ctg5_hap10(	2.19	JUC17_ctg5_hap100	LRRC37A4P
TC17_ctg5_hap10(	-2.69	JUC17_ctg5_hap100	LRRC37A4P
TC21000409.hg.1	3.23	JUC21002460.hg.1	TMEM50B
TC21000409.hg.1	2.49	PSR21004645.hg.1	TMEM50B
TC21000409.hg.1	2.25	PSR21004640.hg.1	TMEM50B
TC21000409.hg.1	2.23	PSR21004623.hg.1	TMEM50B
TC21000409.hg.1	2.12	PSR21004639.hg.1	TMEM50B
TC01002258.hg.1	3.22	JUC01018442.hg.1	EPHA2
TC01002258.hg.1	2.09	PSR01034440.hg.1	EPHA2
TC01004024.hg.1	3.22	JUC01031823.hg.1	RGS7
TC01004024.hg.1	3.18	JUC01031806.hg.1	RGS7
TC01004024.hg.1	2.29	JUC01031826.hg.1	RGS7
TC01004024.hg.1	2.25	PSR01060765.hg.1	RGS7
TC01004024.hg.1	2.12	JUC01031824.hg.1	RGS7
TC01004024.hg.1	2.01	PSR01060781.hg.1	RGS7
TC01004024.hg.1	-2.36	PSR01060762.hg.1	RGS7
TC01004024.hg.1	-2.52	PSR01060780.hg.1	RGS7
TC02000360.hg.1	3.22	JUC02002819.hg.1	EHBP1
TC02000360.hg.1	-2.07	PSR02005406.hg.1	EHBP1
TC02000360.hg.1	-3	JUC02002804.hg.1	EHBP1
TC02000360.hg.1	-3.25	PSR02005384.hg.1	EHBP1
TC02000360.hg.1	-5.83	JUC02002788.hg.1	EHBP1
TC02001600.hg.1	3.22	PSR02025688.hg.1	FAM49A
TC02001600.hg.1	2.13	PSR02025689.hg.1	FAM49A
TC02001600.hg.1	2.11	PSR02025684.hg.1	FAM49A
TC02001600.hg.1	-3.3	JUC02013349.hg.1	FAM49A
TC02005058.hg.1	3.22	PSR02001475.hg.1	FAM228B
TC02005058.hg.1	2.71	JUC02033252.hg.1	FAM228B
TC02005058.hg.1	2	JUC02033250.hg.1	FAM228B
TC02005058.hg.1	-2.25	PSR02001467.hg.1	FAM228B
TC02005058.hg.1	-2.68	JUC02033249.hg.1	FAM228B
TC02005058.hg.1	-2.76	JUC02033241.hg.1	FAM228B
TC02005058.hg.1	-3.21	JUC02033247.hg.1	FAM228B
TC02005058.hg.1	-3.3	JUC02033258.hg.1	FAM228B
TC06001327.hg.1	3.22	JUC06007963.hg.1	TDP2
TC06001327.hg.1	-2.21	PSR06015888.hg.1	TDP2
TC06001327.hg.1	-2.73	PSR06015887.hg.1	TDP2
TC07000658.hg.1	3.22	JUC07004760.hg.1	LOC1002895
TC07000658.hg.1	-2.24	PSR07010217.hg.1	LOC1002895
TC07001897.hg.1	3.22	JUC07014524.hg.1	DGKI
TC07001897.hg.1	2.69	PSR07029471.hg.1	DGKI
TC07001897.hg.1	2.29	PSR07029448.hg.1	DGKI
TC07001897.hg.1	2.21	JUC07014546.hg.1	DGKI
TC07001897.hg.1	2.09	PSR07029427.hg.1	DGKI

TC07001897.hg.1	2.03	PSR07029443.hg.1	DGKI
TC07001897.hg.1	-2.08	PSR07029444.hg.1	DGKI
TC07001897.hg.1	-2.14	JUC07014536.hg.1	DGKI
TC07001897.hg.1	-2.14	JUC07014537.hg.1	DGKI
TC07001897.hg.1	-2.17	PSR07029449.hg.1	DGKI
TC07001897.hg.1	-2.31	JUC07014554.hg.1	DGKI
TC07001897.hg.1	-2.4	PSR07029475.hg.1	DGKI
TC07001897.hg.1	-2.49	JUC07014545.hg.1	DGKI
TC07001897.hg.1	-2.5	PSR07029476.hg.1	DGKI
TC07001897.hg.1	-2.57	JUC07014544.hg.1	DGKI
TC07001897.hg.1	-2.86	JUC07014560.hg.1	DGKI
TC07001897.hg.1	-3.75	PSR07029447.hg.1	DGKI
TC08000565.hg.1	3.22	JUC08003854.hg.1	FAM92A1
TC08000565.hg.1	2.06	PSR08007708.hg.1	FAM92A1
TC08000565.hg.1	-2.13	PSR08007678.hg.1	FAM92A1
TC08000565.hg.1	-2.63	PSR08007721.hg.1	FAM92A1
TC09001361.hg.1	3.22	PSR09016977.hg.1	FANCC
TC09001361.hg.1	2.9	PSR09016971.hg.1	FANCC
TC09001361.hg.1	2.64	PSR09016970.hg.1	FANCC
TC09001361.hg.1	2.41	JUC09009132.hg.1	FANCC
TC09001361.hg.1	2.36	PSR09016968.hg.1	FANCC
TC09001361.hg.1	2.34	PSR09017002.hg.1	FANCC
TC09001361.hg.1	2.17	PSR09016967.hg.1	FANCC
TC09001361.hg.1	2.06	PSR09016993.hg.1	FANCC
TC09001361.hg.1	2.04	PSR09016991.hg.1	FANCC
TC09001361.hg.1	2.01	PSR09016984.hg.1	FANCC
TC10001577.hg.1	3.22	JUC10011758.hg.1	MIR1287, PY
TC10001577.hg.1	2.24	JUC10011762.hg.1	MIR1287, PY
TC10001577.hg.1	-2.06	JUC10011757.hg.1	MIR1287, PY
TC10001577.hg.1	-2.14	PSR10020201.hg.1	MIR1287, PY
TC10001577.hg.1	-2.56	JUC10011768.hg.1	MIR1287, PY
TC10001577.hg.1	-2.69	JUC10011775.hg.1	MIR1287, PY
TC10001577.hg.1	-2.79	JUC10011770.hg.1	MIR1287, PY
TC10001577.hg.1	-2.87	JUC10011769.hg.1	MIR1287, PY
TC10001577.hg.1	-2.93	JUC10011761.hg.1	MIR1287, PY
TC10001577.hg.1	-3.32	PSR10020218.hg.1	MIR1287, PY
TC10001577.hg.1	-3.36	PSR10020223.hg.1	MIR1287, PY
TC10001577.hg.1	-3.75	PSR10020221.hg.1	MIR1287, PY
TC10001577.hg.1	-7.56	PSR10020222.hg.1	MIR1287, PY
TC11003463.hg.1	3.22	JUC11019233.hg.1	C11orf80
TC11003463.hg.1	3.11	JUC11019229.hg.1	C11orf80
TC11003463.hg.1	2.91	PSR11008650.hg.1	C11orf80
TC11003463.hg.1	2.37	JUC11019228.hg.1	C11orf80
TC11003463.hg.1	2.23	PSR11008653.hg.1	C11orf80
TC11003463.hg.1	-2.22	PSR11008634.hg.1	C11orf80
TC13000622.hg.1	3.22	JUC13004121.hg.1	TSC22D1
TC13000622.hg.1	2.48	PSR13007085.hg.1	TSC22D1
TC13000622.hg.1	2.11	PSR13007084.hg.1	TSC22D1
TC13000622.hg.1	-2.14	JUC13004114.hg.1	TSC22D1
TC13000622.hg.1	-2.17	PSR13007073.hg.1	TSC22D1
TC13000622.hg.1	-2.41	PSR13007072.hg.1	TSC22D1
TC15001468.hg.1	3.22	JUC15007371.hg.1	PYGO1
TC15001468.hg.1	2.78	PSR15013754.hg.1	PYGO1
TC15001468.hg.1	2.51	PSR15013753.hg.1	PYGO1
TC15001468.hg.1	2.25	PSR15013758.hg.1	PYGO1
TC17001954.hg.1	3.22	JUC17014989.hg.1	NPLOC4
TC17001954.hg.1	2.33	PSR17026590.hg.1	NPLOC4
TC17001954.hg.1	2.06	JUC17014987.hg.1	NPLOC4

TC20000900.hg.1	3.22	PSR20012376.hg.1	ELMO2
TC20000900.hg.1	2.7	JUC20006403.hg.1	ELMO2
TC20000900.hg.1	2.36	JUC20006412.hg.1	ELMO2
TC20000900.hg.1	2.07	JUC20006410.hg.1	ELMO2
TC20000900.hg.1	-2.05	JUC20006407.hg.1	ELMO2
TC20000900.hg.1	-2.38	JUC20006399.hg.1	ELMO2
TC20000900.hg.1	-2.41	JUC20006423.hg.1	ELMO2
TC01003791.hg.1	3.21	JUC01030336.hg.1	KCNH1
TC01003791.hg.1	2.05	PSR01058048.hg.1	KCNH1
TC01003791.hg.1	-2.02	PSR01058043.hg.1	KCNH1
TC01003791.hg.1	-2.37	PSR01058050.hg.1	KCNH1
TC01003791.hg.1	-2.65	JUC01030343.hg.1	KCNH1
TC01003791.hg.1	-2.7	JUC01030338.hg.1	KCNH1
TC01003791.hg.1	-3.25	PSR01058052.hg.1	KCNH1
TC06001584.hg.1	3.21	JUC06009155.hg.1	TAPBP
TC06001584.hg.1	2.06	PSR06019230.hg.1	TAPBP
TC07000387.hg.1	3.21	JUC07002544.hg.1	ZNF273
TC07000387.hg.1	2.04	JUC07002549.hg.1	ZNF273
TC07000387.hg.1	-2.02	PSR07005273.hg.1	ZNF273
TC07000387.hg.1	-2.29	PSR07005274.hg.1	ZNF273
TC09002922.hg.1	3.21	PSR09021476.hg.1	RALGDS
TC09002922.hg.1	3.18	JUC09016084.hg.1	RALGDS
TC09002922.hg.1	3.13	PSR09021480.hg.1	RALGDS
TC09002922.hg.1	2.47	PSR09021515.hg.1	RALGDS
TC09002922.hg.1	2.05	PSR09021508.hg.1	RALGDS
TC09002922.hg.1	2.02	PSR09021492.hg.1	RALGDS
TC09002922.hg.1	2	PSR09021513.hg.1	RALGDS
TC12000084.hg.1	3.21	JUC12000524.hg.1	GAPDH
TC12000084.hg.1	2.29	JUC12000530.hg.1	GAPDH
TC12000084.hg.1	2.1	PSR12000904.hg.1	GAPDH
TC12000084.hg.1	-3.28	PSR12000953.hg.1	GAPDH
TC12000084.hg.1	-5.09	PSR12000937.hg.1	GAPDH
TC16000615.hg.1	3.21	PSR16008761.hg.1	ZNRF1
TC16000615.hg.1	2.86	JUC16004863.hg.1	ZNRF1
TC16000615.hg.1	2.34	PSR16008767.hg.1	ZNRF1
TC16000615.hg.1	2.2	PSR16008765.hg.1	ZNRF1
TC18000480.hg.1	3.21	PSR18004967.hg.1	ATP5A1
TC18000480.hg.1	2.05	JUC18003332.hg.1	ATP5A1
TC04001086.hg.1	3.2	JUC04007956.hg.1	DHX15
TC04001086.hg.1	3	PSR04014936.hg.1	DHX15
TC04001086.hg.1	2.1	PSR04014949.hg.1	DHX15
TC04001086.hg.1	2.08	JUC04007951.hg.1	DHX15
TC04001462.hg.1	3.2	JUC04010902.hg.1	COL25A1
TC04001462.hg.1	2.32	PSR04020744.hg.1	COL25A1
TC04001462.hg.1	2.32	JUC04010856.hg.1	COL25A1
TC04001462.hg.1	2.01	JUC04010905.hg.1	COL25A1
TC04001462.hg.1	-2.54	PSR04020746.hg.1	COL25A1
TC07000722.hg.1	3.2	JUC07005313.hg.1	MET
TC07000722.hg.1	2.79	JUC07005321.hg.1	MET
TC07000722.hg.1	2.38	PSR07011433.hg.1	MET
TC09000820.hg.1	3.2	JUC09005859.hg.1	FAM69B
TC09000820.hg.1	2.44	PSR09010604.hg.1	FAM69B
TC14000944.hg.1	3.2	PSR14010406.hg.1	C14orf93
TC14000944.hg.1	2.56	PSR14010402.hg.1	C14orf93
TC14000944.hg.1	2.51	JUC14005269.hg.1	C14orf93
TC19001892.hg.1	3.2	JUC19014550.hg.1	PEG3, ZIM2
TC19001892.hg.1	2.94	PSR19025676.hg.1	PEG3, ZIM2
TC19001892.hg.1	2.86	JUC19014534.hg.1	PEG3, ZIM2

TC19001892.hg.1	-2.09	PSR19025682.hg.1	PEG3, ZIM2
TC19001892.hg.1	-2.35	JUC19014546.hg.1	PEG3, ZIM2
TC19001892.hg.1	-3.1	PSR19025678.hg.1	PEG3, ZIM2
TC6_cox_hap2000	3.2	JUC6_cox_hap20017	C4B, C4A, LC
TC6_cox_hap2000	2.57	PSR6_cox_hap2001	C4B, C4A, LC
TC6_cox_hap2000	2.37	JUC6_cox_hap20017	C4B, C4A, LC
TC6_cox_hap2000	2.36	JUC6_cox_hap20017	C4B, C4A, LC
TC6_cox_hap2000	2.31	JUC6_cox_hap20017	C4B, C4A, LC
TC6_cox_hap2000	2.26	PSR6_cox_hap2001	C4B, C4A, LC
TC6_cox_hap2000	2.21	PSR6_cox_hap2001	C4B, C4A, LC
TC6_cox_hap2000	2.11	JUC6_cox_hap20017	C4B, C4A, LC
TC6_cox_hap2000	2.02	PSR6_cox_hap2001	C4B, C4A, LC
TC6_cox_hap2000	-2.1	JUC6_cox_hap20017	C4B, C4A, LC
TC6_cox_hap2000	-2.37	JUC6_cox_hap20017	C4B, C4A, LC
TC6_cox_hap2000	-2.96	JUC6_cox_hap20017	C4B, C4A, LC
TC6_cox_hap2000	-2.97	JUC6_cox_hap20017	C4B, C4A, LC
TC6_cox_hap2000	-3.17	JUC6_cox_hap20017	C4B, C4A, LC
TC6_cox_hap2000	-3.79	JUC6_cox_hap20017	C4B, C4A, LC
TC6_dbb_hap3000	3.2	JUC6_dbb_hap3000	C4B, C4A, LC
TC6_dbb_hap3000	2.57	PSR6_dbb_hap3001	C4B, C4A, LC
TC6_dbb_hap3000	2.37	JUC6_dbb_hap3000	C4B, C4A, LC
TC6_dbb_hap3000	2.36	JUC6_dbb_hap3000	C4B, C4A, LC
TC6_dbb_hap3000	2.31	JUC6_dbb_hap3000	C4B, C4A, LC
TC6_dbb_hap3000	2.26	PSR6_dbb_hap3001	C4B, C4A, LC
TC6_dbb_hap3000	2.21	PSR6_dbb_hap3001	C4B, C4A, LC
TC6_dbb_hap3000	2.11	JUC6_dbb_hap3000	C4B, C4A, LC
TC6_dbb_hap3000	2.02	PSR6_dbb_hap3001	C4B, C4A, LC
TC6_dbb_hap3000	-2.1	JUC6_dbb_hap3000	C4B, C4A, LC
TC6_dbb_hap3000	-2.37	JUC6_dbb_hap3000	C4B, C4A, LC
TC6_dbb_hap3000	-2.96	JUC6_dbb_hap3000	C4B, C4A, LC
TC6_dbb_hap3000	-2.97	JUC6_dbb_hap3000	C4B, C4A, LC
TC6_dbb_hap3000	-3.17	JUC6_dbb_hap3000	C4B, C4A, LC
TC6_dbb_hap3000	-3.79	JUC6_dbb_hap3000	C4B, C4A, LC
TC6_qbl_hap6000	3.2	JUC6_qbl_hap60017	C4B, C4A, LC
TC6_qbl_hap6000	2.57	PSR6_qbl_hap60015	C4B, C4A, LC
TC6_qbl_hap6000	2.37	JUC6_qbl_hap60017	C4B, C4A, LC
TC6_qbl_hap6000	2.36	JUC6_qbl_hap60017	C4B, C4A, LC
TC6_qbl_hap6000	2.31	JUC6_qbl_hap60017	C4B, C4A, LC
TC6_qbl_hap6000	2.26	PSR6_qbl_hap60015	C4B, C4A, LC
TC6_qbl_hap6000	2.21	PSR6_qbl_hap60015	C4B, C4A, LC
TC6_qbl_hap6000	2.11	JUC6_qbl_hap60017	C4B, C4A, LC
TC6_qbl_hap6000	2.02	PSR6_qbl_hap60015	C4B, C4A, LC
TC6_qbl_hap6000	-2.1	JUC6_qbl_hap60017	C4B, C4A, LC
TC6_qbl_hap6000	-2.37	JUC6_qbl_hap60017	C4B, C4A, LC
TC6_qbl_hap6000	-2.96	JUC6_qbl_hap60017	C4B, C4A, LC
TC6_qbl_hap6000	-2.97	JUC6_qbl_hap60017	C4B, C4A, LC
TC6_qbl_hap6000	-3.17	JUC6_qbl_hap60017	C4B, C4A, LC
TC6_qbl_hap6000	-3.79	JUC6_qbl_hap60017	C4B, C4A, LC
TC03000584.hg.1	3.19	PSR03010654.hg.1	GRAMD1C
TC03000584.hg.1	3.13	PSR03010644.hg.1	GRAMD1C
TC03000584.hg.1	2.99	JUC03005322.hg.1	GRAMD1C
TC03000584.hg.1	2.28	JUC03005326.hg.1	GRAMD1C
TC03000584.hg.1	2.01	PSR03010667.hg.1	GRAMD1C
TC03000584.hg.1	-2.2	PSR03010679.hg.1	GRAMD1C
TC03000584.hg.1	-2.25	PSR03010691.hg.1	GRAMD1C
TC04000683.hg.1	3.19	JUC04005197.hg.1	NAA15
TC04000683.hg.1	2.04	PSR04009676.hg.1	NAA15
TC04000683.hg.1	-2.39	JUC04005206.hg.1	NAA15

TC05000317.hg.1	3.19	JUC05002305.hg.1	SMN1, SMN2
TC05000317.hg.1	2.89	JUC05002299.hg.1	SMN1, SMN2
TC05000317.hg.1	2.37	PSR05004261.hg.1	SMN1, SMN2
TC05000317.hg.1	2.12	JUC05002307.hg.1	SMN1, SMN2
TC05001102.hg.1	3.19	JUC05008144.hg.1	SLC12A7
TC05001102.hg.1	2.7	PSR05016017.hg.1	SLC12A7
TC05001102.hg.1	2.68	JUC05008129.hg.1	SLC12A7
TC05001102.hg.1	2.5	PSR05016010.hg.1	SLC12A7
TC05001102.hg.1	2.19	JUC05008136.hg.1	SLC12A7
TC05001102.hg.1	-2.52	PSR05016030.hg.1	SLC12A7
TC05001102.hg.1	-2.81	JUC05008141.hg.1	SLC12A7
TC05001102.hg.1	-3.09	JUC05008134.hg.1	SLC12A7
TC05001102.hg.1	-3.24	JUC05008128.hg.1	SLC12A7
TC05001184.hg.1	3.19	JUC05008660.hg.1	MYO10
TC05001184.hg.1	2.89	PSR05016947.hg.1	MYO10
TC05001184.hg.1	2.57	JUC05008671.hg.1	MYO10
TC05001184.hg.1	2.2	PSR05016942.hg.1	MYO10
TC05001184.hg.1	2.16	PSR05016927.hg.1	MYO10
TC05001184.hg.1	2.14	PSR05016972.hg.1	MYO10
TC05001184.hg.1	2.06	PSR05016930.hg.1	MYO10
TC07001043.hg.1	3.19	PSR07016073.hg.1	RBM33
TC07001043.hg.1	3.05	PSR07016072.hg.1	RBM33
TC07001043.hg.1	2.47	PSR07016087.hg.1	RBM33
TC07001043.hg.1	2.46	PSR07016090.hg.1	RBM33
TC07001043.hg.1	2.3	JUC07007795.hg.1	RBM33
TC07001043.hg.1	2.22	JUC07007802.hg.1	RBM33
TC07001043.hg.1	2.19	PSR07016085.hg.1	RBM33
TC07001043.hg.1	2.09	JUC07007799.hg.1	RBM33
TC07001043.hg.1	2.07	PSR07016099.hg.1	RBM33
TC07001043.hg.1	2.06	PSR07016103.hg.1	RBM33
TC07001043.hg.1	-3.35	JUC07007798.hg.1	RBM33
TC08002586.hg.1	3.19	JUC08014763.hg.1	STAU2
TC08002586.hg.1	2.13	PSR08017178.hg.1	STAU2
TC09001170.hg.1	3.19	JUC09008092.hg.1	CBWD6
TC09001170.hg.1	2.16	JUC09008100.hg.1	CBWD6
TC09001170.hg.1	-2.02	PSR09015039.hg.1	CBWD6
TC09001170.hg.1	-2.04	PSR09015023.hg.1	CBWD6
TC09001170.hg.1	-2.04	PSR09015043.hg.1	CBWD6
TC09001170.hg.1	-2.27	PSR09015062.hg.1	CBWD6
TC09001170.hg.1	-2.27	JUC09008091.hg.1	CBWD6
TC09001170.hg.1	-3.96	PSR09015056.hg.1	CBWD6
TC09001170.hg.1	-3.99	JUC09008093.hg.1	CBWD6
TC0X001518.hg.1	3.19	JUC0X010020.hg.1	IDH3G
TC0X001518.hg.1	2.1	PSR0X019909.hg.1	IDH3G
TC14000413.hg.1	3.19	JUC14002519.hg.1	EXD2
TC14000413.hg.1	2.09	PSR14004950.hg.1	EXD2
TC14001141.hg.1	3.19	PSR14012960.hg.1	ERO1L
TC14001141.hg.1	2.09	PSR14012944.hg.1	ERO1L
TC14001141.hg.1	-2.81	PSR14012951.hg.1	ERO1L
TC14001141.hg.1	-3.96	JUC14006574.hg.1	ERO1L
TC15000224.hg.1	3.19	JUC15000515.hg.1	SCG5
TC15000224.hg.1	2.34	PSR15001311.hg.1	SCG5
TC15000224.hg.1	-2.42	PSR15001324.hg.1	SCG5
TC15000224.hg.1	-2.8	PSR15001316.hg.1	SCG5
TC17000957.hg.1	3.19	PSR17012630.hg.1	FN3K
TC17000957.hg.1	2.66	JUC17007159.hg.1	FN3K
TC17000957.hg.1	2.54	PSR17012627.hg.1	FN3K
TC17000957.hg.1	2.51	PSR17012620.hg.1	FN3K

TC17000957.hg.1	2.5	JUC17007157.hg.1	FN3K
TC17000957.hg.1	2.25	PSR17012625.hg.1	FN3K
TC17001017.hg.1	3.19	PSR17013689.hg.1	ZZEF1
TC17001017.hg.1	2.16	JUC17007794.hg.1	ZZEF1
TC17001017.hg.1	-2.05	JUC17007763.hg.1	ZZEF1
TC17001017.hg.1	-2.07	JUC17007784.hg.1	ZZEF1
TC17001017.hg.1	-2.4	JUC17007782.hg.1	ZZEF1
TC19001273.hg.1	3.19	JUC19010491.hg.1	ANO8
TC19001273.hg.1	3.06	JUC19010506.hg.1	ANO8
TC19001273.hg.1	2.28	JUC19010497.hg.1	ANO8
TC19001273.hg.1	2.18	JUC19010503.hg.1	ANO8
TC19001273.hg.1	-2.07	PSR19018036.hg.1	ANO8
TC19001273.hg.1	-2.6	JUC19010502.hg.1	ANO8
TC03000069.hg.1	3.18	JUC03000808.hg.1	PPARG
TC03000069.hg.1	2.33	JUC03000812.hg.1	PPARG
TC03000069.hg.1	2.07	PSR03001551.hg.1	PPARG
TC03000069.hg.1	-2.16	PSR03001529.hg.1	PPARG
TC03000069.hg.1	-2.34	PSR03001556.hg.1	PPARG
TC03000069.hg.1	-2.48	PSR03001538.hg.1	PPARG
TC03000069.hg.1	-2.6	JUC03000814.hg.1	PPARG
TC03000069.hg.1	-2.86	PSR03001532.hg.1	PPARG
TC03000069.hg.1	-2.95	PSR03001534.hg.1	PPARG
TC03000069.hg.1	-3	PSR03001539.hg.1	PPARG
TC03000069.hg.1	-4.29	PSR03001533.hg.1	PPARG
TC03000069.hg.1	-6.24	JUC03000810.hg.1	PPARG
TC03000632.hg.1	3.18	JUC03005680.hg.1	DTX3L
TC03000632.hg.1	-2.48	PSR03011391.hg.1	DTX3L
TC03000667.hg.1	3.18	JUC03006010.hg.1	ABTB1
TC03000667.hg.1	2.85	PSR03012002.hg.1	ABTB1
TC03000667.hg.1	2.66	JUC03006011.hg.1	ABTB1
TC03000667.hg.1	2.29	JUC03006012.hg.1	ABTB1
TC03000667.hg.1	2.14	JUC03005993.hg.1	ABTB1
TC03000667.hg.1	2.14	JUC03006002.hg.1	ABTB1
TC03000667.hg.1	2.1	PSR03012015.hg.1	ABTB1
TC03000667.hg.1	2	PSR03011981.hg.1	ABTB1
TC05001316.hg.1	3.18	JUC05009557.hg.1	HMGCS1
TC05001316.hg.1	-2.19	PSR05018555.hg.1	HMGCS1
TC06002302.hg.1	3.18	JUC06014527.hg.1	LINC00473
TC06002302.hg.1	-2.86	PSR06028842.hg.1	LINC00473
TC08000471.hg.1	3.18	JUC08003248.hg.1	LOC1001328
TC08000471.hg.1	2.27	PSR08006602.hg.1	LOC1001328
TC08000471.hg.1	2.11	JUC08003242.hg.1	LOC1001328
TC08000471.hg.1	-2	PSR08006583.hg.1	LOC1001328
TC08000471.hg.1	-6.16	JUC08003256.hg.1	LOC1001328
TC08001120.hg.1	3.18	PSR08014486.hg.1	RNF122
TC08001120.hg.1	2.77	JUC08007376.hg.1	RNF122
TC08001120.hg.1	2.21	PSR08014481.hg.1	RNF122
TC09000077.hg.1	3.18	PSR09000899.hg.1	CCDC171
TC09000077.hg.1	2.69	JUC09000483.hg.1	CCDC171
TC09000077.hg.1	2.65	JUC09000476.hg.1	CCDC171
TC09000077.hg.1	2.17	JUC09000459.hg.1	CCDC171
TC09000077.hg.1	-8.78	JUC09000451.hg.1	CCDC171
TC17000297.hg.1	3.18	PSR17003629.hg.1	MAP2K3
TC17000297.hg.1	3.09	JUC17002067.hg.1	MAP2K3
TC17000297.hg.1	2.95	JUC17002053.hg.1	MAP2K3
TC17000297.hg.1	2.57	JUC17002062.hg.1	MAP2K3
TC17000297.hg.1	2.18	JUC17002063.hg.1	MAP2K3
TC17000297.hg.1	2.11	JUC17002055.hg.1	MAP2K3

TC17000297.hg.1	2.05	JUC17002052.hg.1	MAP2K3
TC17001064.hg.1	3.18	JUC17008151.hg.1	KIAA0753
TC17001064.hg.1	2.51	JUC17008157.hg.1	KIAA0753
TC17001064.hg.1	2.21	PSR17014253.hg.1	KIAA0753
TC17001064.hg.1	-2.23	JUC17008152.hg.1	KIAA0753
TC19000442.hg.1	3.18	PSR19005775.hg.1	GPI
TC19000442.hg.1	2.93	PSR19005765.hg.1	GPI
TC19000442.hg.1	2.55	JUC19003436.hg.1	GPI
TC19000442.hg.1	2.42	PSR19005754.hg.1	GPI
TC19000442.hg.1	2.1	PSR19005755.hg.1	GPI
TC19000442.hg.1	2.02	PSR19005759.hg.1	GPI
TC19000442.hg.1	2.01	JUC19003431.hg.1	GPI
TC22000565.hg.1	3.18	JUC22004079.hg.1	GUSBP11
TC22000565.hg.1	2.88	JUC22004074.hg.1	GUSBP11
TC22000565.hg.1	2.81	JUC22004078.hg.1	GUSBP11
TC22000565.hg.1	2.6	PSR22010149.hg.1	GUSBP11
TC22000565.hg.1	2.12	JUC22004071.hg.1	GUSBP11
TC22000565.hg.1	2.02	JUC22004068.hg.1	GUSBP11
TC01003968.hg.1	3.17	PSR01060100.hg.1	EGLN1
TC01003968.hg.1	2.32	JUC01031440.hg.1	EGLN1
TC04002963.hg.1	3.17	PSR04000023.hg.1	ZNF595
TC04002963.hg.1	2.09	JUC04017534.hg.1	ZNF595
TC04002963.hg.1	-2.06	PSR04000025.hg.1	ZNF595
TC04002963.hg.1	-2.09	PSR04000024.hg.1	ZNF595
TC04002963.hg.1	-2.28	JUC04017524.hg.1	ZNF595
TC05001771.hg.1	3.17	JUC05012402.hg.1	KIF3A
TC05001771.hg.1	2.15	PSR05024012.hg.1	KIF3A
TC05001771.hg.1	-2.27	JUC05012381.hg.1	KIF3A
TC05001771.hg.1	-2.37	JUC05012387.hg.1	KIF3A
TC06001059.hg.1	3.17	JUC06006090.hg.1	UTRN
TC06001059.hg.1	2.57	PSR06012454.hg.1	UTRN
TC06001059.hg.1	2.28	PSR06012430.hg.1	UTRN
TC06001059.hg.1	2.12	JUC06006121.hg.1	UTRN
TC06001059.hg.1	2.09	PSR06012518.hg.1	UTRN
TC06001059.hg.1	2.06	JUC06006126.hg.1	UTRN
TC06001059.hg.1	2	JUC06006125.hg.1	UTRN
TC06001059.hg.1	-2.24	PSR06012486.hg.1	UTRN
TC06001059.hg.1	-2.27	JUC06006108.hg.1	UTRN
TC09001639.hg.1	3.17	PSR09020828.hg.1	C9orf114
TC09001639.hg.1	2.19	JUC09011177.hg.1	C9orf114
TC09001639.hg.1	-2.28	JUC09011188.hg.1	C9orf114
TC11001449.hg.1	3.17	JUC11009687.hg.1	SOX6
TC11001449.hg.1	3.15	JUC11009703.hg.1	SOX6
TC11001449.hg.1	2.68	PSR11018181.hg.1	SOX6
TC11001449.hg.1	2.52	JUC11009706.hg.1	SOX6
TC11001449.hg.1	2.11	PSR11018168.hg.1	SOX6
TC11001449.hg.1	2.05	JUC11009686.hg.1	SOX6
TC11001449.hg.1	2.01	PSR11018177.hg.1	SOX6
TC14001476.hg.1	3.17	JUC14008618.hg.1	SYNE3
TC14001476.hg.1	2.78	JUC14008619.hg.1	SYNE3
TC14001476.hg.1	2.58	JUC14008624.hg.1	SYNE3
TC14001476.hg.1	2.43	JUC14008616.hg.1	SYNE3
TC14001476.hg.1	2.36	PSR14016684.hg.1	SYNE3
TC16000126.hg.1	3.17	JUC16000953.hg.1	DNASE1
TC16000126.hg.1	2.08	JUC16000951.hg.1	DNASE1
TC16000126.hg.1	-2.12	JUC16000947.hg.1	DNASE1
TC16000126.hg.1	-2.19	PSR16002005.hg.1	DNASE1
TC16000126.hg.1	-2.42	JUC16000949.hg.1	DNASE1

TC16000126.hg.1	-2.61	JUC16000958.hg.1	DNASE1
TC19001079.hg.1	3.17	JUC19008360.hg.1	SAFB2
TC19001079.hg.1	-2.04	JUC19008334.hg.1	SAFB2
TC19001079.hg.1	-2.52	PSR19014759.hg.1	SAFB2
TC19001079.hg.1	-2.6	JUC19008344.hg.1	SAFB2
TC6_ssto_hap700(	3.17	PSR6_ssto_hap7000	HLA-F
TC6_ssto_hap700(	3	PSR6_ssto_hap7000	HLA-F
TC6_ssto_hap700(	2.93	JUC6_ssto_hap7000	HLA-F
TC6_ssto_hap700(	2.92	PSR6_ssto_hap7000	HLA-F
TC6_ssto_hap700(	2.8	JUC6_ssto_hap7000	HLA-F
TC6_ssto_hap700(	2.69	JUC6_ssto_hap7000	HLA-F
TC6_ssto_hap700(	2.5	PSR6_ssto_hap7000	HLA-F
TC6_ssto_hap700(	2.27	JUC6_ssto_hap7000	HLA-F
TC6_ssto_hap700(	2.26	JUC6_ssto_hap7000	HLA-F
TC6_ssto_hap700(	2.19	PSR6_ssto_hap7000	HLA-F
TC6_ssto_hap700(	2.15	PSR6_ssto_hap7000	HLA-F
TC6_ssto_hap700(	2.13	PSR6_ssto_hap7000	HLA-F
TC6_ssto_hap700(	2.1	JUC6_ssto_hap7000	HLA-F
TC01001252.hg.1	3.16	PSR01019137.hg.1	LOR
TC01001252.hg.1	2.25	JUC01010415.hg.1	LOR
TC01001252.hg.1	2.19	PSR01019140.hg.1	LOR
TC01001544.hg.1	3.16	JUC01012584.hg.1	RALGPS2
TC01001544.hg.1	2.54	PSR01023837.hg.1	RALGPS2
TC01003784.hg.1	3.16	PSR01057988.hg.1	LAMB3, MIR4
TC01003784.hg.1	2.63	JUC01030294.hg.1	LAMB3, MIR4
TC01003784.hg.1	2.63	JUC01030313.hg.1	LAMB3, MIR4
TC01003784.hg.1	2.22	JUC01030310.hg.1	LAMB3, MIR4
TC01003784.hg.1	2.18	PSR01057967.hg.1	LAMB3, MIR4
TC01003784.hg.1	-2.08	PSR01057983.hg.1	LAMB3, MIR4
TC01003784.hg.1	-2.44	JUC01030309.hg.1	LAMB3, MIR4
TC01003784.hg.1	-2.45	PSR01057981.hg.1	LAMB3, MIR4
TC01003784.hg.1	-2.76	PSR01057985.hg.1	LAMB3, MIR4
TC01003784.hg.1	-2.99	JUC01030307.hg.1	LAMB3, MIR4
TC01003784.hg.1	-3.42	JUC01030314.hg.1	LAMB3, MIR4
TC01003784.hg.1	-3.69	PSR01057982.hg.1	LAMB3, MIR4
TC01003784.hg.1	-3.75	JUC01030304.hg.1	LAMB3, MIR4
TC01003784.hg.1	-3.81	PSR01057979.hg.1	LAMB3, MIR4
TC01003784.hg.1	-4.2	JUC01030295.hg.1	LAMB3, MIR4
TC01003784.hg.1	-4.34	PSR01057986.hg.1	LAMB3, MIR4
TC01003784.hg.1	-8.07	JUC01030305.hg.1	LAMB3, MIR4
TC02002204.hg.1	3.16	JUC02018484.hg.1	BUB1
TC02002204.hg.1	2.64	JUC02018455.hg.1	BUB1
TC02002204.hg.1	2.22	PSR02035777.hg.1	BUB1
TC02002204.hg.1	2.04	JUC02018480.hg.1	BUB1
TC02002204.hg.1	-2.26	PSR02035775.hg.1	BUB1
TC02002204.hg.1	-2.44	JUC02018457.hg.1	BUB1
TC02002204.hg.1	-2.58	PSR02035765.hg.1	BUB1
TC02002204.hg.1	-3.16	JUC02018466.hg.1	BUB1
TC03000011.hg.1	3.16	PSR03000233.hg.1	LRRN1
TC03000011.hg.1	-2.18	JUC03000133.hg.1	LRRN1
TC03000011.hg.1	-2.63	JUC03000135.hg.1	LRRN1
TC07001457.hg.1	3.16	JUC07010831.hg.1	GUSB
TC07001457.hg.1	2.28	JUC07010820.hg.1	GUSB
TC07001457.hg.1	2.22	PSR07022103.hg.1	GUSB
TC07001457.hg.1	2.18	PSR07022078.hg.1	GUSB
TC07001457.hg.1	2.11	JUC07010837.hg.1	GUSB
TC07001457.hg.1	2.04	JUC07010833.hg.1	GUSB
TC0X001182.hg.1	3.16	PSR0X015960.hg.1	BRWD3

TC0X001182.hg.1	2.81	PSR0X015939.hg.1	BRWD3
TC0X001182.hg.1	2.27	PSR0X015914.hg.1	BRWD3
TC0X001182.hg.1	2.26	JUC0X008075.hg.1	BRWD3
TC0X001182.hg.1	2.22	JUC0X008093.hg.1	BRWD3
TC0X001182.hg.1	-2.36	JUC0X008069.hg.1	BRWD3
TC0X002327.hg.1	3.16	JUC0X013137.hg.1	PIR
TC0X002327.hg.1	3.11	PSR0X011728.hg.1	PIR
TC0X002327.hg.1	2.63	PSR0X011726.hg.1	PIR
TC0X002327.hg.1	2.55	PSR0X011736.hg.1	PIR
TC0X002327.hg.1	2.51	PSR0X011738.hg.1	PIR
TC0X002327.hg.1	2.51	JUC0X013135.hg.1	PIR
TC0X002327.hg.1	2.29	JUC0X013138.hg.1	PIR
TC0X002327.hg.1	-2.11	JUC0X013132.hg.1	PIR
TC0X002327.hg.1	-2.31	JUC0X013131.hg.1	PIR
TC0X002327.hg.1	-2.63	JUC0X013134.hg.1	PIR
TC0X002327.hg.1	-2.7	PSR0X011732.hg.1	PIR
TC0X002327.hg.1	-3.21	JUC0X013130.hg.1	PIR
TC10001613.hg.1	3.16	JUC10012112.hg.1	MGEA5
TC10001613.hg.1	2.95	JUC10012096.hg.1	MGEA5
TC10001613.hg.1	2.06	JUC10012118.hg.1	MGEA5
TC10001613.hg.1	2.03	JUC10012108.hg.1	MGEA5
TC10001613.hg.1	-2.79	PSR10020914.hg.1	MGEA5
TC11000707.hg.1	3.16	PSR11009212.hg.1	LRP5
TC11000707.hg.1	3.04	JUC11004711.hg.1	LRP5
TC11000707.hg.1	2.94	JUC11004724.hg.1	LRP5
TC11000707.hg.1	2.64	JUC11004708.hg.1	LRP5
TC11000707.hg.1	2.35	PSR11009187.hg.1	LRP5
TC11000707.hg.1	2.31	JUC11004718.hg.1	LRP5
TC11000707.hg.1	2.17	JUC11004702.hg.1	LRP5
TC11000707.hg.1	2.15	PSR11009188.hg.1	LRP5
TC11000707.hg.1	2.1	PSR11009197.hg.1	LRP5
TC11000707.hg.1	2.02	PSR11009189.hg.1	LRP5
TC11000707.hg.1	-2.29	JUC11004709.hg.1	LRP5
TC11000707.hg.1	-2.32	JUC11004721.hg.1	LRP5
TC15000971.hg.1	3.16	JUC15004759.hg.1	ALDH1A3
TC15000971.hg.1	2.48	PSR15009022.hg.1	ALDH1A3
TC15000971.hg.1	2.09	JUC15004751.hg.1	ALDH1A3
TC15000971.hg.1	2	JUC15004755.hg.1	ALDH1A3
TC15000971.hg.1	-2.37	PSR15009019.hg.1	ALDH1A3
TC15000971.hg.1	-3.56	JUC15004758.hg.1	ALDH1A3
TC15000971.hg.1	-3.68	PSR15009009.hg.1	ALDH1A3
TC15000971.hg.1	-3.71	PSR15009025.hg.1	ALDH1A3
TC15000971.hg.1	-3.73	PSR15009017.hg.1	ALDH1A3
TC15000971.hg.1	-3.85	PSR15009013.hg.1	ALDH1A3
TC15000971.hg.1	-4.15	PSR15009012.hg.1	ALDH1A3
TC15000971.hg.1	-4.41	PSR15009003.hg.1	ALDH1A3
TC15000971.hg.1	-4.89	JUC15004757.hg.1	ALDH1A3
TC15000971.hg.1	-5.26	PSR15009010.hg.1	ALDH1A3
TC15000971.hg.1	-5.79	JUC15004747.hg.1	ALDH1A3
TC15000971.hg.1	-6.53	JUC15004761.hg.1	ALDH1A3
TC16000294.hg.1	3.16	PSR16004123.hg.1	IL21R
TC16000294.hg.1	-2.01	JUC16002269.hg.1	IL21R
TC16000294.hg.1	-2.19	JUC16002264.hg.1	IL21R
TC16000482.hg.1	3.16	JUC16003745.hg.1	NLRC5
TC16000482.hg.1	2.83	JUC16003779.hg.1	NLRC5
TC16000482.hg.1	2.75	JUC16003771.hg.1	NLRC5
TC16000482.hg.1	2.49	PSR16006703.hg.1	NLRC5
TC16000482.hg.1	2.41	JUC16003772.hg.1	NLRC5

TC16000482.hg.1	2.35	PSR16006658.hg.1	NLRC5
TC16000482.hg.1	2.23	JUC16003797.hg.1	NLRC5
TC16000482.hg.1	-2.01	PSR16006675.hg.1	NLRC5
TC16000482.hg.1	-2.64	JUC16003756.hg.1	NLRC5
TC16000482.hg.1	-2.9	JUC16003739.hg.1	NLRC5
TC16000482.hg.1	-2.93	JUC16003795.hg.1	NLRC5
TC16000482.hg.1	-3.32	PSR16006653.hg.1	NLRC5
TC20000250.hg.1	3.16	JUC20001860.hg.1	MMP24
TC20000250.hg.1	2.41	JUC20001857.hg.1	MMP24
TC20000250.hg.1	2.23	PSR20003287.hg.1	MMP24
TC20000250.hg.1	-2.06	JUC20001856.hg.1	MMP24
TC20000250.hg.1	-2.3	PSR20003285.hg.1	MMP24
TC20000250.hg.1	-2.53	PSR20003282.hg.1	MMP24
TC20000378.hg.1	3.16	JUC20002974.hg.1	
TC20000378.hg.1	-2.21	PSR20005459.hg.1	
TC01001750.hg.1	3.15	PSR01027393.hg.1	HSD11B1
TC01001750.hg.1	-2.08	PSR01027397.hg.1	HSD11B1
TC01001750.hg.1	-3.44	JUC01014746.hg.1	HSD11B1
TC01001750.hg.1	-3.86	JUC01014744.hg.1	HSD11B1
TC01002352.hg.1	3.15	JUC01019557.hg.1	FUCA1
TC01002352.hg.1	3	PSR01036296.hg.1	FUCA1
TC01002352.hg.1	2.9	PSR01036298.hg.1	FUCA1
TC01002352.hg.1	2.35	JUC01019563.hg.1	FUCA1
TC01002352.hg.1	2.23	PSR01036292.hg.1	FUCA1
TC01002352.hg.1	2.19	PSR01036299.hg.1	FUCA1
TC01002352.hg.1	2.17	PSR01036297.hg.1	FUCA1
TC01002932.hg.1	3.15	PSR01045510.hg.1	COL11A1
TC01002932.hg.1	3.09	PSR01045535.hg.1	COL11A1
TC01002932.hg.1	-2.01	PSR01045587.hg.1	COL11A1
TC01002932.hg.1	-2.01	PSR01045588.hg.1	COL11A1
TC01002932.hg.1	-2.04	PSR01045582.hg.1	COL11A1
TC01002932.hg.1	-2.06	PSR01045580.hg.1	COL11A1
TC01002932.hg.1	-2.13	PSR01045511.hg.1	COL11A1
TC01002932.hg.1	-2.13	PSR01045590.hg.1	COL11A1
TC01002932.hg.1	-2.16	JUC01024485.hg.1	COL11A1
TC01002932.hg.1	-2.2	JUC01024501.hg.1	COL11A1
TC01002932.hg.1	-2.21	PSR01045585.hg.1	COL11A1
TC01002932.hg.1	-2.23	JUC01024498.hg.1	COL11A1
TC01002932.hg.1	-2.23	JUC01024552.hg.1	COL11A1
TC01002932.hg.1	-2.36	JUC01024542.hg.1	COL11A1
TC01002932.hg.1	-2.44	PSR01045589.hg.1	COL11A1
TC01002932.hg.1	-2.44	JUC01024489.hg.1	COL11A1
TC01002932.hg.1	-2.47	JUC01024507.hg.1	COL11A1
TC01002932.hg.1	-2.52	PSR01045506.hg.1	COL11A1
TC01002932.hg.1	-2.72	JUC01024543.hg.1	COL11A1
TC01002932.hg.1	-2.81	JUC01024525.hg.1	COL11A1
TC01002932.hg.1	-3.61	JUC01024499.hg.1	COL11A1
TC01002932.hg.1	-4.28	JUC01024551.hg.1	COL11A1
TC01002932.hg.1	-8.98	JUC01024490.hg.1	COL11A1
TC02001218.hg.1	3.15	JUC02010002.hg.1	ADAM23
TC02001218.hg.1	2.41	PSR02018713.hg.1	ADAM23
TC02001218.hg.1	2.14	JUC02010000.hg.1	ADAM23
TC02001218.hg.1	-2.23	JUC02010017.hg.1	ADAM23
TC02001218.hg.1	-2.7	JUC02010007.hg.1	ADAM23
TC06001908.hg.1	3.15	JUC06011267.hg.1	IBTK
TC06001908.hg.1	2.87	JUC06011258.hg.1	IBTK
TC06001908.hg.1	-2.27	PSR06023176.hg.1	IBTK
TC06001908.hg.1	-2.56	JUC06011257.hg.1	IBTK

TC06001944.hg.1	3.15	JUC06011679.hg.1	MDN1
TC06001944.hg.1	2.71	JUC06011628.hg.1	MDN1
TC06001944.hg.1	2.2	JUC06011615.hg.1	MDN1
TC06001944.hg.1	2.11	JUC06011672.hg.1	MDN1
TC06001944.hg.1	-2.19	JUC06011600.hg.1	MDN1
TC06001944.hg.1	-2.34	JUC06011635.hg.1	MDN1
TC06001944.hg.1	-2.36	JUC06011690.hg.1	MDN1
TC06001944.hg.1	-2.7	JUC06011614.hg.1	MDN1
TC06001944.hg.1	-2.72	PSR06023868.hg.1	MDN1
TC06001944.hg.1	-2.81	JUC06011652.hg.1	MDN1
TC06001944.hg.1	-3.14	JUC06011622.hg.1	MDN1
TC08000123.hg.1	3.15	PSR08001107.hg.1	EFHA2
TC08000123.hg.1	2.45	PSR08001108.hg.1	EFHA2
TC08000123.hg.1	-2	JUC08000586.hg.1	EFHA2
TC08000123.hg.1	-3.6	JUC08000588.hg.1	EFHA2
TC12000668.hg.1	3.15	JUC12004727.hg.1	PTPRQ
TC12000668.hg.1	3.02	JUC12004707.hg.1	PTPRQ
TC12000668.hg.1	2.29	PSR12008810.hg.1	PTPRQ
TC12000668.hg.1	2.24	JUC12004719.hg.1	PTPRQ
TC12000668.hg.1	2.1	JUC12004729.hg.1	PTPRQ
TC12000668.hg.1	-2	JUC12004734.hg.1	PTPRQ
TC12000668.hg.1	-2.04	JUC12004721.hg.1	PTPRQ
TC12000668.hg.1	-2.64	JUC12004743.hg.1	PTPRQ
TC12000668.hg.1	-3.49	JUC12004698.hg.1	PTPRQ
TC16000267.hg.1	3.15	JUC16002198.hg.1	
TC16000267.hg.1	2.47	PSR16003991.hg.1	
TC17001601.hg.1	3.15	PSR17021588.hg.1	PLEKHM1, M
TC17001601.hg.1	2.48	PSR17021577.hg.1	PLEKHM1, M
TC17001601.hg.1	2.37	JUC17012126.hg.1	PLEKHM1, M
TC17001601.hg.1	2.36	JUC17012139.hg.1	PLEKHM1, M
TC17001601.hg.1	2.26	PSR17021590.hg.1	PLEKHM1, M
TC6_cox_hap2000	3.15	JUC6_cox_hap20002	DDR1
TC6_cox_hap2000	2.94	PSR6_cox_hap20006	DDR1
TC6_cox_hap2000	2.86	JUC6_cox_hap20002	DDR1
TC6_cox_hap2000	2.84	PSR6_cox_hap20006	DDR1
TC6_cox_hap2000	2.82	PSR6_cox_hap20006	DDR1
TC6_cox_hap2000	2.75	PSR6_cox_hap20006	DDR1
TC6_cox_hap2000	2.73	JUC6_cox_hap20003	DDR1
TC6_cox_hap2000	2.69	PSR6_cox_hap20006	DDR1
TC6_cox_hap2000	2.61	JUC6_cox_hap20003	DDR1
TC6_cox_hap2000	2.56	JUC6_cox_hap20002	DDR1
TC6_cox_hap2000	2.39	JUC6_cox_hap20003	DDR1
TC6_cox_hap2000	2.38	PSR6_cox_hap20006	DDR1
TC6_cox_hap2000	2.31	JUC6_cox_hap20003	DDR1
TC6_cox_hap2000	2.24	PSR6_cox_hap20006	DDR1
TC6_cox_hap2000	2.23	PSR6_cox_hap20006	DDR1
TC6_cox_hap2000	2.22	JUC6_cox_hap20003	DDR1
TC6_cox_hap2000	2.16	PSR6_cox_hap20006	DDR1
TC6_cox_hap2000	2.16	PSR6_cox_hap20006	DDR1
TC6_cox_hap2000	2.14	JUC6_cox_hap20002	DDR1
TC6_cox_hap2000	2.01	PSR6_cox_hap20006	DDR1
TC6_cox_hap2000	-2.07	JUC6_cox_hap20003	DDR1
TC6_cox_hap2000	-2.6	PSR6_cox_hap20006	DDR1
TC6_dbb_hap3000	3.15	PSR6_dbb_hap3000	HLA-F
TC6_dbb_hap3000	2.91	JUC6_dbb_hap30000	HLA-F
TC6_dbb_hap3000	2.79	JUC6_dbb_hap30000	HLA-F
TC6_dbb_hap3000	2.68	JUC6_dbb_hap30000	HLA-F
TC6_dbb_hap3000	2.65	PSR6_dbb_hap3000	HLA-F

TC6_dbb_hap3000	2.34	PSR6_dbb_hap3000	HLA-F
TC6_dbb_hap3000	2.26	JUC6_dbb_hap3000(	HLA-F
TC6_dbb_hap3000	2.25	JUC6_dbb_hap3000(	HLA-F
TC6_dbb_hap3000	2.13	PSR6_dbb_hap3000	HLA-F
TC6_dbb_hap3000	2.09	JUC6_dbb_hap3000(	HLA-F
TC6_dbb_hap3000	2.02	PSR6_dbb_hap3000	HLA-F
TC6_dbb_hap3000	2.02	PSR6_dbb_hap3000	HLA-F
TC6_mann_hap40(	3.15	PSR6_mann_hap40C	HLA-F
TC6_mann_hap40(	2.91	JUC6_mann_hap400	HLA-F
TC6_mann_hap40(	2.79	JUC6_mann_hap400	HLA-F
TC6_mann_hap40(	2.68	JUC6_mann_hap400	HLA-F
TC6_mann_hap40(	2.65	PSR6_mann_hap40C	HLA-F
TC6_mann_hap40(	2.34	PSR6_mann_hap40C	HLA-F
TC6_mann_hap40(	2.26	JUC6_mann_hap400	HLA-F
TC6_mann_hap40(	2.25	JUC6_mann_hap400	HLA-F
TC6_mann_hap40(	2.13	PSR6_mann_hap40C	HLA-F
TC6_mann_hap40(	2.09	JUC6_mann_hap400	HLA-F
TC6_mann_hap40(	2.02	PSR6_mann_hap40C	HLA-F
TC6_mann_hap40(	2.02	PSR6_mann_hap40C	HLA-F
TC6_qbl_hap6000(	3.15	PSR6_qbl_hap60001	HLA-F
TC6_qbl_hap6000(	2.91	JUC6_qbl_hap60000	HLA-F
TC6_qbl_hap6000(	2.79	JUC6_qbl_hap60000	HLA-F
TC6_qbl_hap6000(	2.68	JUC6_qbl_hap60000	HLA-F
TC6_qbl_hap6000(	2.65	PSR6_qbl_hap60001	HLA-F
TC6_qbl_hap6000(	2.34	PSR6_qbl_hap60001	HLA-F
TC6_qbl_hap6000(	2.26	JUC6_qbl_hap60000	HLA-F
TC6_qbl_hap6000(	2.25	JUC6_qbl_hap60000	HLA-F
TC6_qbl_hap6000(	2.13	PSR6_qbl_hap60001	HLA-F
TC6_qbl_hap6000(	2.09	JUC6_qbl_hap60000	HLA-F
TC6_qbl_hap6000(	2.02	PSR6_qbl_hap60001	HLA-F
TC6_qbl_hap6000(	2.02	PSR6_qbl_hap60001	HLA-F
TC01002115.hg.1	3.14	JUC01017411.hg.1	MORN1
TC01002115.hg.1	2.2	PSR01032513.hg.1	MORN1
TC01002115.hg.1	2.03	PSR01032503.hg.1	MORN1
TC01003650.hg.1	3.14	PSR01056057.hg.1	UCHL5
TC01003650.hg.1	-2.2	PSR01056041.hg.1	UCHL5
TC01003650.hg.1	-2.21	JUC01029243.hg.1	UCHL5
TC01003650.hg.1	-2.55	JUC01029251.hg.1	UCHL5
TC01003650.hg.1	-4.83	JUC01029232.hg.1	UCHL5
TC03002131.hg.1	3.14	JUC03018609.hg.1	LRRC15
TC03002131.hg.1	2.43	PSR03037579.hg.1	LRRC15
TC03002131.hg.1	2	PSR03037578.hg.1	LRRC15
TC03002131.hg.1	-2.51	PSR03037584.hg.1	LRRC15
TC04002964.hg.1	3.14	JUC04017543.hg.1	ZNF718
TC04002964.hg.1	-2.04	PSR04000056.hg.1	ZNF718
TC04002964.hg.1	-2.18	JUC04017540.hg.1	ZNF718
TC04002964.hg.1	-2.75	JUC04017539.hg.1	ZNF718
TC05000326.hg.1	3.14	JUC05002356.hg.1	SMN1, SMN2
TC05000326.hg.1	2.84	JUC05002361.hg.1	SMN1, SMN2
TC05000326.hg.1	2.33	PSR05004401.hg.1	SMN1, SMN2
TC05000326.hg.1	2.08	JUC05002359.hg.1	SMN1, SMN2
TC06000558.hg.1	3.14	JUC06002664.hg.1	DAAM2
TC06000558.hg.1	2.82	PSR06006230.hg.1	DAAM2
TC07000075.hg.1	3.14	PSR07001261.hg.1	C1GALT1
TC07000075.hg.1	2.99	JUC07000562.hg.1	C1GALT1
TC07000075.hg.1	2.61	PSR07001263.hg.1	C1GALT1
TC07000075.hg.1	2.58	PSR07001262.hg.1	C1GALT1
TC07000075.hg.1	-4.23	JUC07000568.hg.1	C1GALT1

TC07000942.hg.1	3.14	PSR07014536.hg.1	FAM115C
TC07000942.hg.1	2.54	JUC07007025.hg.1	FAM115C
TC07000942.hg.1	2.54	JUC07007026.hg.1	FAM115C
TC07000942.hg.1	2.54	JUC07007027.hg.1	FAM115C
TC07000942.hg.1	2.44	PSR07014530.hg.1	FAM115C
TC07000942.hg.1	2.27	PSR07014543.hg.1	FAM115C
TC07000942.hg.1	-2.05	PSR07014526.hg.1	FAM115C
TC07000942.hg.1	-2.31	JUC07006997.hg.1	FAM115C
TC07000942.hg.1	-2.37	JUC07007000.hg.1	FAM115C
TC07000942.hg.1	-2.37	JUC07007002.hg.1	FAM115C
TC07000942.hg.1	-2.6	JUC07007003.hg.1	FAM115C
TC07000942.hg.1	-2.6	JUC07007004.hg.1	FAM115C
TC07000942.hg.1	-2.85	PSR07014540.hg.1	FAM115C
TC07000942.hg.1	-3.53	PSR07014539.hg.1	FAM115C
TC07000942.hg.1	-3.53	PSR07014549.hg.1	FAM115C
TC07000942.hg.1	-4.83	JUC07007012.hg.1	FAM115C
TC07000942.hg.1	-4.83	JUC07007014.hg.1	FAM115C
TC07000942.hg.1	-10.47	JUC07006999.hg.1	FAM115C
TC07002073.hg.1	3.14	JUC07015726.hg.1	LMBR1
TC07002073.hg.1	2.2	PSR07031664.hg.1	LMBR1
TC07002073.hg.1	2.07	JUC07015728.hg.1	LMBR1
TC07002073.hg.1	-2.28	JUC07015709.hg.1	LMBR1
TC07002073.hg.1	-2.85	JUC07015727.hg.1	LMBR1
TC07003361.hg.1	3.14	JUC07021438.hg.1	CYP51A1
TC07003361.hg.1	-2.01	PSR07024093.hg.1	CYP51A1
TC09001591.hg.1	3.14	PSR09020013.hg.1	MAPKAP1
TC09001591.hg.1	-2.07	PSR09019999.hg.1	MAPKAP1
TC09001591.hg.1	-2.08	PSR09020014.hg.1	MAPKAP1
TC09001591.hg.1	-2.36	PSR09019988.hg.1	MAPKAP1
TC09001591.hg.1	-3.5	JUC09010824.hg.1	MAPKAP1
TC09001591.hg.1	-9.56	JUC09010823.hg.1	MAPKAP1
TC0X000965.hg.1	3.14	PSR0X012874.hg.1	CXorf38
TC0X000965.hg.1	2.57	PSR0X012878.hg.1	CXorf38
TC0X000965.hg.1	2.41	PSR0X012869.hg.1	CXorf38
TC0X000965.hg.1	2.33	PSR0X012879.hg.1	CXorf38
TC0X000965.hg.1	2.24	PSR0X012866.hg.1	CXorf38
TC0X000965.hg.1	2.16	JUC0X006458.hg.1	CXorf38
TC11000708.hg.1	3.14	JUC11004752.hg.1	PPP6R3
TC11000708.hg.1	2.81	JUC11004767.hg.1	PPP6R3
TC11000708.hg.1	2.24	JUC11004751.hg.1	PPP6R3
TC11000708.hg.1	-2.11	PSR11009246.hg.1	PPP6R3
TC11000708.hg.1	-2.93	JUC11004771.hg.1	PPP6R3
TC11003442.hg.1	3.14	PSR11001716.hg.1	TRIM34, TRIM
TC11003442.hg.1	-2.56	JUC11018941.hg.1	TRIM34, TRIM
TC14000974.hg.1	3.14	PSR14011112.hg.1	CIDEB
TC14000974.hg.1	3.13	JUC14005569.hg.1	CIDEB
TC14000974.hg.1	2.26	PSR14011121.hg.1	CIDEB
TC14000974.hg.1	2.14	PSR14011126.hg.1	CIDEB
TC14000974.hg.1	2.03	PSR14011122.hg.1	CIDEB
TC15000253.hg.1	3.14	JUC15000669.hg.1	C15orf41
TC15000253.hg.1	2.58	PSR15001559.hg.1	C15orf41
TC15000253.hg.1	2.01	JUC15000668.hg.1	C15orf41
TC15000253.hg.1	-2.74	JUC15000667.hg.1	C15orf41
TC19000641.hg.1	3.14	PSR19008955.hg.1	GEMIN7
TC19000641.hg.1	2.49	JUC19005319.hg.1	GEMIN7
TC6_mann_hap40(	3.14	JUC6_mann_hap4001106.hg.1	
TC6_mann_hap40(	2.1	PSR6_mann_hap4002957.hg.1	
TC6_mann_hap40(	-2.38	PSR6_mann_hap4002982.hg.1	

TC6_mcf_hap5000	3.14	JUC6_mcf_hap5001!	C4B, C4A, LC
TC6_mcf_hap5000	2.52	PSR6_mcf_hap5001:	C4B, C4A, LC
TC6_mcf_hap5000	2.32	JUC6_mcf_hap5001!	C4B, C4A, LC
TC6_mcf_hap5000	2.31	JUC6_mcf_hap5001!	C4B, C4A, LC
TC6_mcf_hap5000	2.21	PSR6_mcf_hap5001:	C4B, C4A, LC
TC6_mcf_hap5000	2.16	PSR6_mcf_hap5001:	C4B, C4A, LC
TC6_mcf_hap5000	2.07	JUC6_mcf_hap5001!	C4B, C4A, LC
TC6_mcf_hap5000	-2.01	PSR6_mcf_hap5001:	C4B, C4A, LC
TC6_mcf_hap5000	-2.15	JUC6_mcf_hap5001!	C4B, C4A, LC
TC6_mcf_hap5000	-2.42	JUC6_mcf_hap5001!	C4B, C4A, LC
TC6_mcf_hap5000	-3.03	JUC6_mcf_hap5001!	C4B, C4A, LC
TC6_mcf_hap5000	-3.03	JUC6_mcf_hap5001!	C4B, C4A, LC
TC6_mcf_hap5000	-3.24	JUC6_mcf_hap5001!	C4B, C4A, LC
TC6_mcf_hap5000	-3.87	JUC6_mcf_hap5001!	C4B, C4A, LC
TC6_ssto_hap700(	3.14	JUC6_ssto_hap7000	C4B, C4A, LC
TC6_ssto_hap700(	2.52	PSR6_ssto_hap7001	C4B, C4A, LC
TC6_ssto_hap700(	2.32	JUC6_ssto_hap7000	C4B, C4A, LC
TC6_ssto_hap700(	2.31	JUC6_ssto_hap7000	C4B, C4A, LC
TC6_ssto_hap700(	2.21	PSR6_ssto_hap7001	C4B, C4A, LC
TC6_ssto_hap700(	2.16	PSR6_ssto_hap7001	C4B, C4A, LC
TC6_ssto_hap700(	2.07	JUC6_ssto_hap7000	C4B, C4A, LC
TC6_ssto_hap700(	-2.01	PSR6_ssto_hap7001	C4B, C4A, LC
TC6_ssto_hap700(	-2.15	JUC6_ssto_hap7000	C4B, C4A, LC
TC6_ssto_hap700(	-2.42	JUC6_ssto_hap7000	C4B, C4A, LC
TC6_ssto_hap700(	-3.03	JUC6_ssto_hap7000	C4B, C4A, LC
TC6_ssto_hap700(	-3.03	JUC6_ssto_hap7000	C4B, C4A, LC
TC6_ssto_hap700(	-3.24	JUC6_ssto_hap7000	C4B, C4A, LC
TC6_ssto_hap700(	-3.87	JUC6_ssto_hap7000	C4B, C4A, LC
TC6_ssto_hap700(	3.14	JUC6_ssto_hap7001	C4B, C4A, LC
TC6_ssto_hap700(	2.52	PSR6_ssto_hap7001	C4B, C4A, LC
TC6_ssto_hap700(	2.32	JUC6_ssto_hap7001	C4B, C4A, LC
TC6_ssto_hap700(	2.31	JUC6_ssto_hap7001	C4B, C4A, LC
TC6_ssto_hap700(	2.21	PSR6_ssto_hap7001	C4B, C4A, LC
TC6_ssto_hap700(	2.16	PSR6_ssto_hap7001	C4B, C4A, LC
TC6_ssto_hap700(	2.07	JUC6_ssto_hap7001	C4B, C4A, LC
TC6_ssto_hap700(	-2.01	PSR6_ssto_hap7001	C4B, C4A, LC
TC6_ssto_hap700(	-2.15	JUC6_ssto_hap7001	C4B, C4A, LC
TC6_ssto_hap700(	-2.42	JUC6_ssto_hap7001	C4B, C4A, LC
TC6_ssto_hap700(	-3.03	JUC6_ssto_hap7001	C4B, C4A, LC
TC6_ssto_hap700(	-3.03	JUC6_ssto_hap7001	C4B, C4A, LC
TC6_ssto_hap700(	-3.24	JUC6_ssto_hap7001	C4B, C4A, LC
TC6_ssto_hap700(	-3.87	JUC6_ssto_hap7001	C4B, C4A, LC
TC04000614.hg.1	3.14	JUC04004690.hg.1	LOC645513,
TC04000614.hg.1	2.96	JUC04004696.hg.1	LOC645513,
TC04000614.hg.1	2.94	JUC04004689.hg.1	LOC645513,
TC04000614.hg.1	2	PSR04008854.hg.1	LOC645513,
TC01000941.hg.1	3.13	JUC01007846.hg.1	CELSR2
TC01000941.hg.1	2.31	PSR01014863.hg.1	CELSR2
TC01000941.hg.1	2.27	PSR01014872.hg.1	CELSR2
TC01000941.hg.1	2.17	PSR01014860.hg.1	CELSR2
TC01000941.hg.1	2.06	PSR01014840.hg.1	CELSR2
TC01000941.hg.1	2.03	JUC01007855.hg.1	CELSR2
TC01000941.hg.1	-2.06	PSR01014851.hg.1	CELSR2
TC01000941.hg.1	-2.12	JUC01007847.hg.1	CELSR2
TC01000941.hg.1	-2.35	PSR01014858.hg.1	CELSR2
TC01000941.hg.1	-2.36	JUC01007841.hg.1	CELSR2
TC01000941.hg.1	-2.51	JUC01007863.hg.1	CELSR2
TC01000941.hg.1	-2.65	PSR01014859.hg.1	CELSR2

TC01000941.hg.1	-2.74	PSR01014832.hg.1	CELSR2
TC01000941.hg.1	-2.91	PSR01014854.hg.1	CELSR2
TC01000941.hg.1	-3.62	JUC01007858.hg.1	CELSR2
TC01000941.hg.1	-4.38	JUC01007833.hg.1	CELSR2
TC01000941.hg.1	-5.24	JUC01007861.hg.1	CELSR2
TC01000941.hg.1	-10.48	JUC01007848.hg.1	CELSR2
TC01003225.hg.1	3.13	PSR01049686.hg.1	RFX5
TC01003225.hg.1	2.12	JUC01026360.hg.1	RFX5
TC01003467.hg.1	3.13	JUC01028124.hg.1	TMCO1
TC01003467.hg.1	2.17	JUC01028126.hg.1	TMCO1
TC01003467.hg.1	-2.14	PSR01053989.hg.1	TMCO1
TC01003467.hg.1	-2.29	JUC01028133.hg.1	TMCO1
TC01003467.hg.1	-2.48	JUC01028132.hg.1	TMCO1
TC02002419.hg.1	3.13	PSR02038019.hg.1	RND3
TC02002419.hg.1	2.78	PSR02038021.hg.1	RND3
TC02002419.hg.1	2.25	PSR02038001.hg.1	RND3
TC02002419.hg.1	2.1	PSR02038008.hg.1	RND3
TC02002419.hg.1	-3.66	PSR02038017.hg.1	RND3
TC02002419.hg.1	-3.75	JUC02019743.hg.1	RND3
TC03000572.hg.1	3.13	JUC03005165.hg.1	CD200
TC03000572.hg.1	-2.04	JUC03005168.hg.1	CD200
TC03000572.hg.1	-2.12	PSR03010391.hg.1	CD200
TC03000572.hg.1	-2.42	JUC03005169.hg.1	CD200
TC03000572.hg.1	-2.84	PSR03010385.hg.1	CD200
TC03000572.hg.1	-3.44	PSR03010384.hg.1	CD200
TC03000572.hg.1	-3.47	PSR03010382.hg.1	CD200
TC03000572.hg.1	-3.92	JUC03005172.hg.1	CD200
TC04001194.hg.1	3.13	PSR04016361.hg.1	SGCB
TC04001194.hg.1	2.9	JUC04008703.hg.1	SGCB
TC04001194.hg.1	2.83	JUC04008708.hg.1	SGCB
TC04001194.hg.1	2.61	PSR04016360.hg.1	SGCB
TC04001194.hg.1	2.43	PSR04016362.hg.1	SGCB
TC04001194.hg.1	2.28	PSR04016363.hg.1	SGCB
TC04001194.hg.1	2.06	JUC04008705.hg.1	SGCB
TC05000359.hg.1	3.13	PSR05004929.hg.1	HEXB
TC05000359.hg.1	2.44	PSR05004942.hg.1	HEXB
TC05000359.hg.1	2.01	PSR05004932.hg.1	HEXB
TC05000359.hg.1	-2.25	JUC05002663.hg.1	HEXB
TC06000501.hg.1	3.13	PSR06005245.hg.1	HMGA1, HM(
TC06000501.hg.1	2.37	JUC06002056.hg.1	HMGA1, HM(
TC06000501.hg.1	2.08	PSR06005236.hg.1	HMGA1, HM(
TC06000501.hg.1	2.07	JUC06002063.hg.1	HMGA1, HM(
TC06000501.hg.1	-2	PSR06005254.hg.1	HMGA1, HM(
TC06000501.hg.1	-2.05	PSR06005253.hg.1	HMGA1, HM(
TC06000501.hg.1	-2.18	PSR06005261.hg.1	HMGA1, HM(
TC06000501.hg.1	-2.41	PSR06005258.hg.1	HMGA1, HM(
TC07001266.hg.1	3.13	JUC07009601.hg.1	RP9
TC07001266.hg.1	2.93	PSR07019744.hg.1	RP9
TC07001266.hg.1	2.77	PSR07019745.hg.1	RP9
TC07001266.hg.1	2.12	PSR07019752.hg.1	RP9
TC07001266.hg.1	2.08	JUC07009603.hg.1	RP9
TC07001266.hg.1	2.03	PSR07019751.hg.1	RP9
TC07002017.hg.1	3.13	PSR07030815.hg.1	
TC07002017.hg.1	2.13	JUC07015280.hg.1	
TC09002927.hg.1	3.13	JUC09016142.hg.1	LCN6
TC09002927.hg.1	2.11	PSR09022391.hg.1	LCN6
TC0X000601.hg.1	3.13	PSR0X007903.hg.1	GRIA3
TC0X000601.hg.1	-2	JUC0X003934.hg.1	GRIA3

TC0X000601.hg.1	-2.03	JUC0X003952.hg.1	GRIA3
TC0X000601.hg.1	-2.04	PSR0X007867.hg.1	GRIA3
TC0X000601.hg.1	-2.1	JUC0X003947.hg.1	GRIA3
TC0X000601.hg.1	-2.27	PSR0X007895.hg.1	GRIA3
TC0X000601.hg.1	-2.29	PSR0X007881.hg.1	GRIA3
TC0X000955.hg.1	3.13	JUC0X006409.hg.1	RPGR
TC0X000955.hg.1	2.46	JUC0X006404.hg.1	RPGR
TC0X000955.hg.1	2.01	JUC0X006397.hg.1	RPGR
TC0X000955.hg.1	-2.27	JUC0X006407.hg.1	RPGR
TC0X000955.hg.1	-2.28	PSR0X012773.hg.1	RPGR
TC0X002330.hg.1	3.13	JUC0X013161.hg.1	PRAF2
TC0X002330.hg.1	2.03	PSR0X013673.hg.1	PRAF2
TC11000406.hg.1	3.13	JUC11002831.hg.1	MADD
TC11000406.hg.1	2.04	PSR11005221.hg.1	MADD
TC11000406.hg.1	-2.3	JUC11002823.hg.1	MADD
TC12000523.hg.1	3.13	PSR12006915.hg.1	NXPH4
TC12000523.hg.1	2.89	PSR12006914.hg.1	NXPH4
TC12000523.hg.1	2.32	JUC12003634.hg.1	NXPH4
TC12000523.hg.1	2.12	PSR12006910.hg.1	NXPH4
TC12001807.hg.1	3.13	PSR12023897.hg.1	BTG1
TC12001807.hg.1	2.65	PSR12023895.hg.1	BTG1
TC12001807.hg.1	2.45	JUC12013248.hg.1	BTG1
TC14002191.hg.1	3.13	PSR14000975.hg.1	TRDJ1, TRDJ
TC14002191.hg.1	2.53	PSR14000980.hg.1	TRDJ1, TRDJ
TC14002191.hg.1	2.4	PSR14000982.hg.1	TRDJ1, TRDJ
TC14002191.hg.1	2.35	JUC14011269.hg.1	TRDJ1, TRDJ
TC14002191.hg.1	2.11	JUC14011265.hg.1	TRDJ1, TRDJ
TC14002191.hg.1	2.01	PSR14000977.hg.1	TRDJ1, TRDJ
TC15000778.hg.1	3.13	JUC15003701.hg.1	AGSK1, LOC
TC15000778.hg.1	2.5	JUC15003708.hg.1	AGSK1, LOC
TC15000778.hg.1	2.2	PSR15007140.hg.1	AGSK1, LOC
TC15000778.hg.1	-2.55	JUC15003704.hg.1	AGSK1, LOC
TC15000778.hg.1	-2.83	JUC15003702.hg.1	AGSK1, LOC
TC17000047.hg.1	3.13	JUC17000259.hg.1	ARRB2
TC17000047.hg.1	2.6	PSR17000464.hg.1	ARRB2
TC17000047.hg.1	2.3	PSR17000471.hg.1	ARRB2
TC17000047.hg.1	-2.07	PSR17000475.hg.1	ARRB2
TC17000047.hg.1	-2.09	JUC17000273.hg.1	ARRB2
TC17000047.hg.1	-2.2	JUC17000272.hg.1	ARRB2
TC17000047.hg.1	-3.09	JUC17000264.hg.1	ARRB2
TC17000047.hg.1	-3.1	JUC17000268.hg.1	ARRB2
TC17000047.hg.1	-3.22	PSR17000473.hg.1	ARRB2
TC17000047.hg.1	-3.29	JUC17000266.hg.1	ARRB2
TC17000047.hg.1	-3.53	JUC17000269.hg.1	ARRB2
TC17000047.hg.1	-4.51	JUC17000265.hg.1	ARRB2
TC19000741.hg.1	3.13	PSR19010358.hg.1	PRR12
TC19000741.hg.1	-4.7	JUC19006056.hg.1	PRR12
TC19002636.hg.1	3.13	JUC19017404.hg.1	BCKDHA
TC19002636.hg.1	2.93	JUC19017411.hg.1	BCKDHA
TC19002636.hg.1	2.6	JUC19017403.hg.1	BCKDHA
TC19002636.hg.1	2.36	PSR19008035.hg.1	BCKDHA
TC20000256.hg.1	3.13	PSR20003475.hg.1	SPAG4
TC20000256.hg.1	2.47	PSR20003456.hg.1	SPAG4
TC20000256.hg.1	2.26	JUC20001957.hg.1	SPAG4
TC20000256.hg.1	-2.05	PSR20003469.hg.1	SPAG4
TC20000256.hg.1	-2.43	PSR20003463.hg.1	SPAG4
TC01002962.hg.1	3.12	JUC01024786.hg.1	GSTM3
TC01002962.hg.1	2.88	JUC01024789.hg.1	GSTM3

TC01002962.hg.1	2.22	PSR01046053.hg.1	GSTM3
TC03000732.hg.1	3.12	JUC03006711.hg.1	PPP2R3A
TC03000732.hg.1	2.1	PSR03013247.hg.1	PPP2R3A
TC03000732.hg.1	2.07	JUC03006727.hg.1	PPP2R3A
TC04000960.hg.1	3.12	JUC04006940.hg.1	RNF212
TC04000960.hg.1	-2.01	PSR04013002.hg.1	RNF212
TC04000960.hg.1	-2.39	PSR04012996.hg.1	RNF212
TC04000960.hg.1	-2.72	PSR04013006.hg.1	RNF212
TC07000704.hg.1	3.12	PSR07011051.hg.1	LRRN3
TC07000704.hg.1	2.04	PSR07011052.hg.1	LRRN3
TC07000704.hg.1	-2.04	PSR07011048.hg.1	LRRN3
TC07000704.hg.1	-2.05	JUC07005196.hg.1	LRRN3
TC07000704.hg.1	-2.35	JUC07005199.hg.1	LRRN3
TC07000704.hg.1	-2.8	PSR07011055.hg.1	LRRN3
TC07000704.hg.1	-2.95	PSR07011050.hg.1	LRRN3
TC07000704.hg.1	-4.88	PSR07011043.hg.1	LRRN3
TC07000704.hg.1	-9.25	PSR07011045.hg.1	LRRN3
TC07000704.hg.1	-11.19	PSR07011046.hg.1	LRRN3
TC09000876.hg.1	3.12	JUC09006315.hg.1	RFX3
TC09000876.hg.1	2.46	PSR09011647.hg.1	RFX3
TC09000876.hg.1	2.04	JUC09006305.hg.1	RFX3
TC09000876.hg.1	-2.26	PSR09011668.hg.1	RFX3
TC09000876.hg.1	-2.29	JUC09006311.hg.1	RFX3
TC09000876.hg.1	-3.08	JUC09006304.hg.1	RFX3
TC0X000212.hg.1	3.12	JUC0X001284.hg.1	KDM6A
TC0X000212.hg.1	2.43	JUC0X001274.hg.1	KDM6A
TC0X000212.hg.1	2.3	PSR0X002375.hg.1	KDM6A
TC0X000212.hg.1	2.23	PSR0X002378.hg.1	KDM6A
TC0X000212.hg.1	2.16	PSR0X002383.hg.1	KDM6A
TC0X000212.hg.1	2.05	PSR0X002340.hg.1	KDM6A
TC0X000212.hg.1	-2.04	PSR0X002382.hg.1	KDM6A
TC0X000212.hg.1	-2.66	JUC0X001301.hg.1	KDM6A
TC0X000212.hg.1	-2.92	JUC0X001311.hg.1	KDM6A
TC16000169.hg.1	3.12	JUC16001320.hg.1	SHISA9
TC16000169.hg.1	2.69	PSR16002559.hg.1	SHISA9
TC17001689.hg.1	3.12	JUC17012631.hg.1	CHAD
TC17001689.hg.1	-2.17	JUC17012632.hg.1	CHAD
TC17001689.hg.1	-2.42	PSR17022729.hg.1	CHAD
TC17001689.hg.1	-2.48	PSR17022730.hg.1	CHAD
TC17001689.hg.1	-3.4	JUC17012635.hg.1	CHAD
TC17001689.hg.1	-4.3	JUC17012633.hg.1	CHAD
TC17001914.hg.1	3.12	PSR17025960.hg.1	TK1
TC17001914.hg.1	2.76	JUC17014587.hg.1	TK1
TC17001914.hg.1	2.49	PSR17025959.hg.1	TK1
TC17001914.hg.1	2.34	PSR17025951.hg.1	TK1
TC17001914.hg.1	2.24	JUC17014585.hg.1	TK1
TC17001914.hg.1	2.21	PSR17025953.hg.1	TK1
TC17001914.hg.1	-2.01	PSR17025955.hg.1	TK1
TC01001675.hg.1	3.11	JUC01013925.hg.1	PPP1R12B
TC01001675.hg.1	2.67	JUC01013933.hg.1	PPP1R12B
TC01001675.hg.1	-2	PSR01026011.hg.1	PPP1R12B
TC01001675.hg.1	-2	JUC01013938.hg.1	PPP1R12B
TC01001675.hg.1	-2.08	PSR01026031.hg.1	PPP1R12B
TC01001675.hg.1	-2.11	PSR01026025.hg.1	PPP1R12B
TC01001675.hg.1	-2.11	JUC01013907.hg.1	PPP1R12B
TC01001675.hg.1	-2.14	JUC01013923.hg.1	PPP1R12B
TC01001675.hg.1	-2.15	PSR01026036.hg.1	PPP1R12B
TC01001675.hg.1	-2.15	PSR01026039.hg.1	PPP1R12B

TC01001675.hg.1	-2.2	PSR01026026.hg.1	PPP1R12B
TC01001675.hg.1	-2.2	PSR01026038.hg.1	PPP1R12B
TC01001675.hg.1	-2.39	JUC01013904.hg.1	PPP1R12B
TC01001675.hg.1	-2.67	PSR01026027.hg.1	PPP1R12B
TC01001675.hg.1	-2.92	JUC01013934.hg.1	PPP1R12B
TC01001675.hg.1	-2.97	PSR01026020.hg.1	PPP1R12B
TC01001675.hg.1	-3.57	JUC01013911.hg.1	PPP1R12B
TC01006268.hg.1	3.11	PSR01003949.hg.1	NBL1, C1orf1
TC01006268.hg.1	2.79	PSR01003939.hg.1	NBL1, C1orf1
TC01006268.hg.1	2.42	PSR01003951.hg.1	NBL1, C1orf1
TC01006268.hg.1	2.31	JUC01038979.hg.1	NBL1, C1orf1
TC01006268.hg.1	-2.04	PSR01003944.hg.1	NBL1, C1orf1
TC01006268.hg.1	-4.42	JUC01038987.hg.1	NBL1, C1orf1
TC01006268.hg.1	-4.65	PSR01003937.hg.1	NBL1, C1orf1
TC01006268.hg.1	-4.7	PSR01003945.hg.1	NBL1, C1orf1
TC01006268.hg.1	-5.15	JUC01038988.hg.1	NBL1, C1orf1
TC01006268.hg.1	-5.31	PSR01003936.hg.1	NBL1, C1orf1
TC01006268.hg.1	-5.34	JUC01038982.hg.1	NBL1, C1orf1
TC01006268.hg.1	-5.35	PSR01003938.hg.1	NBL1, C1orf1
TC01006268.hg.1	-5.77	PSR01003934.hg.1	NBL1, C1orf1
TC01006268.hg.1	-6.05	PSR01003931.hg.1	NBL1, C1orf1
TC01006268.hg.1	-6.16	JUC01038985.hg.1	NBL1, C1orf1
TC01006268.hg.1	-6.34	JUC01038980.hg.1	NBL1, C1orf1
TC01006268.hg.1	-6.39	PSR01003927.hg.1	NBL1, C1orf1
TC01006268.hg.1	-10.11	JUC01038986.hg.1	NBL1, C1orf1
TC03000038.hg.1	3.11	JUC03000378.hg.1	SETD5
TC03000038.hg.1	2.16	PSR03000678.hg.1	SETD5
TC03000038.hg.1	2.06	JUC03000395.hg.1	SETD5
TC03000038.hg.1	-2.03	JUC03000398.hg.1	SETD5
TC03000038.hg.1	-2.59	PSR03000670.hg.1	SETD5
TC03000038.hg.1	-3.08	PSR03000635.hg.1	SETD5
TC05000451.hg.1	3.11	JUC05003514.hg.1	NR2F1
TC05000451.hg.1	-2.44	PSR05006375.hg.1	NR2F1
TC05001366.hg.1	3.11	JUC05009848.hg.1	IL6ST
TC05001366.hg.1	2.06	PSR05019130.hg.1	IL6ST
TC05001366.hg.1	-3.84	JUC05009854.hg.1	IL6ST
TC05001543.hg.1	3.11	PSR05021049.hg.1	LOC1001310
TC05001543.hg.1	2.38	JUC05010854.hg.1	LOC1001310
TC05001543.hg.1	2	PSR05021037.hg.1	LOC1001310
TC05001543.hg.1	-2.07	JUC05010853.hg.1	LOC1001310
TC14000562.hg.1	3.11	JUC14003506.hg.1	C14orf159
TC14000562.hg.1	2.75	JUC14003471.hg.1	C14orf159
TC14000562.hg.1	2.48	JUC14003478.hg.1	C14orf159
TC14000562.hg.1	2.43	JUC14003490.hg.1	C14orf159
TC14000562.hg.1	2.41	JUC14003488.hg.1	C14orf159
TC14000562.hg.1	2.29	PSR14006829.hg.1	C14orf159
TC14000562.hg.1	2.29	JUC14003469.hg.1	C14orf159
TC14000562.hg.1	2.29	JUC14003472.hg.1	C14orf159
TC14000562.hg.1	2.2	PSR14006790.hg.1	C14orf159
TC14000562.hg.1	2.14	JUC14003486.hg.1	C14orf159
TC14000562.hg.1	2.11	PSR14006791.hg.1	C14orf159
TC14000562.hg.1	2.1	PSR14006820.hg.1	C14orf159
TC14000562.hg.1	2.08	PSR14006841.hg.1	C14orf159
TC14000562.hg.1	2.01	JUC14003493.hg.1	C14orf159
TC14000562.hg.1	2	PSR14006801.hg.1	C14orf159
TC14000562.hg.1	-2.28	PSR14006822.hg.1	C14orf159
TC14000562.hg.1	-2.38	JUC14003497.hg.1	C14orf159
TC6_ssto_hap7000	3.11	JUC6_ssto_hap7000	APOM

TC6_ssto_hap700(	2.37	PSR6_ssto_hap7000	APOM
TC02000589.hg.1	3.1	JUC02004643.hg.1	ANKRD36, AI
TC02000589.hg.1	-2.02	PSR02009089.hg.1	ANKRD36, AI
TC02000589.hg.1	-2.02	PSR02009101.hg.1	ANKRD36, AI
TC02000589.hg.1	-2.07	JUC02004664.hg.1	ANKRD36, AI
TC02000589.hg.1	-2.09	JUC02004669.hg.1	ANKRD36, AI
TC02000589.hg.1	-2.1	JUC02004633.hg.1	ANKRD36, AI
TC02000589.hg.1	-2.39	PSR02009158.hg.1	ANKRD36, AI
TC02000589.hg.1	-2.78	JUC02004671.hg.1	ANKRD36, AI
TC02000589.hg.1	-2.95	JUC02004667.hg.1	ANKRD36, AI
TC02001573.hg.1	3.1	JUC02013167.hg.1	PDIA6
TC02001573.hg.1	2.41	JUC02013176.hg.1	PDIA6
TC02001573.hg.1	2.37	PSR02025394.hg.1	PDIA6
TC02001573.hg.1	2.37	JUC02013169.hg.1	PDIA6
TC02001573.hg.1	2.15	PSR02025393.hg.1	PDIA6
TC02001573.hg.1	2.08	PSR02025399.hg.1	PDIA6
TC02001573.hg.1	2.02	PSR02025395.hg.1	PDIA6
TC02001573.hg.1	2.01	PSR02025397.hg.1	PDIA6
TC03001939.hg.1	3.1	JUC03017163.hg.1	CCNL1
TC03001939.hg.1	-2.55	PSR03034660.hg.1	CCNL1
TC04000897.hg.1	3.1	JUC04006595.hg.1	CCDC111
TC04000897.hg.1	2.04	PSR04012266.hg.1	CCDC111
TC04000897.hg.1	-2.11	JUC04006587.hg.1	CCDC111
TC06001506.hg.1	3.1	JUC06008386.hg.1	NRM
TC06001506.hg.1	2.28	PSR06017027.hg.1	NRM
TC06001506.hg.1	-2.94	JUC06008384.hg.1	NRM
TC06001506.hg.1	-2.94	JUC06008385.hg.1	NRM
TC11000174.hg.1	3.1	PSR11002379.hg.1	WEE1
TC11000174.hg.1	2.79	PSR11002378.hg.1	WEE1
TC11000174.hg.1	2.16	JUC11001066.hg.1	WEE1
TC11000174.hg.1	2.03	PSR11002380.hg.1	WEE1
TC11000174.hg.1	-2.24	PSR11002386.hg.1	WEE1
TC11000174.hg.1	-2.26	JUC11001076.hg.1	WEE1
TC11000174.hg.1	-2.31	JUC11001075.hg.1	WEE1
TC12001440.hg.1	3.1	PSR12018821.hg.1	COL2A1
TC12001440.hg.1	2.86	JUC12010515.hg.1	COL2A1
TC12001440.hg.1	2.69	PSR12018848.hg.1	COL2A1
TC12001440.hg.1	2.54	JUC12010536.hg.1	COL2A1
TC12001440.hg.1	2.49	JUC12010541.hg.1	COL2A1
TC12001440.hg.1	2.32	JUC12010509.hg.1	COL2A1
TC12001440.hg.1	2.27	JUC12010510.hg.1	COL2A1
TC12001440.hg.1	2.23	PSR12018833.hg.1	COL2A1
TC12001440.hg.1	2.19	PSR12018827.hg.1	COL2A1
TC12001440.hg.1	2.19	PSR12018859.hg.1	COL2A1
TC12001440.hg.1	2.18	JUC12010544.hg.1	COL2A1
TC12001440.hg.1	2.05	PSR12018850.hg.1	COL2A1
TC12001440.hg.1	2.04	JUC12010540.hg.1	COL2A1
TC12001440.hg.1	-2.03	JUC12010547.hg.1	COL2A1
TC12001440.hg.1	-2.18	PSR12018810.hg.1	COL2A1
TC12001440.hg.1	-2.18	JUC12010535.hg.1	COL2A1
TC12001440.hg.1	-2.36	PSR12018853.hg.1	COL2A1
TC12001440.hg.1	-2.42	JUC12010546.hg.1	COL2A1
TC12001440.hg.1	-2.44	PSR12018840.hg.1	COL2A1
TC12001440.hg.1	-2.51	JUC12010516.hg.1	COL2A1
TC12001440.hg.1	-2.94	JUC12010554.hg.1	COL2A1
TC12001440.hg.1	-3.2	PSR12018863.hg.1	COL2A1
TC12001440.hg.1	-3.33	JUC12010533.hg.1	COL2A1
TC12001440.hg.1	-4.32	PSR12018801.hg.1	COL2A1

TC12001440.hg.1	-4.64	PSR12018806.hg.1	COL2A1
TC12001440.hg.1	-6.35	JUC12010503.hg.1	COL2A1
TC12001440.hg.1	-7.37	PSR12018808.hg.1	COL2A1
TC12001440.hg.1	-22.62	PSR12018804.hg.1	COL2A1
TC12001440.hg.1	-24.51	PSR12018805.hg.1	COL2A1
TC12001440.hg.1	-36.62	PSR12018802.hg.1	COL2A1
TC12001440.hg.1	-80.9	PSR12018803.hg.1	COL2A1
TC12001440.hg.1	-269.44	JUC12010517.hg.1	COL2A1
TC16001350.hg.1	3.1	PSR16018731.hg.1	ANKRD11
TC16001350.hg.1	-2.05	JUC16010676.hg.1	ANKRD11
TC16001350.hg.1	-3.06	PSR16018718.hg.1	ANKRD11
TC18000058.hg.1	3.1	PSR18000591.hg.1	GNAL
TC18000058.hg.1	2.3	PSR18000594.hg.1	GNAL
TC18000058.hg.1	-2.73	JUC18000379.hg.1	GNAL
TC6_apd_hap1000	3.1	JUC6_apd_hap1000	NRM
TC6_apd_hap1000	2.28	PSR6_apd_hap1001	NRM
TC6_apd_hap1000	-2.94	JUC6_apd_hap1000	NRM
TC6_apd_hap1000	-2.94	JUC6_apd_hap1000	NRM
TC6_cox_hap2000	3.1	JUC6_cox_hap2000	NRM
TC6_cox_hap2000	2.28	PSR6_cox_hap2002	NRM
TC6_cox_hap2000	-2.94	JUC6_cox_hap2000	NRM
TC6_cox_hap2000	-2.94	JUC6_cox_hap2000	NRM
TC6_dbb_hap3000	3.1	JUC6_dbb_hap3000	NRM
TC6_dbb_hap3000	2.28	PSR6_dbb_hap3002	NRM
TC6_dbb_hap3000	-2.94	JUC6_dbb_hap3000	NRM
TC6_dbb_hap3000	-2.94	JUC6_dbb_hap3000	NRM
TC6_mann_hap40	3.1	JUC6_mann_hap400	NRM
TC6_mann_hap40	2.28	PSR6_mann_hap40	NRM
TC6_mann_hap40	-2.94	JUC6_mann_hap400	NRM
TC6_mann_hap40	-2.94	JUC6_mann_hap400	NRM
TC6_mcf_hap5000	3.1	JUC6_mcf_hap5000	NRM
TC6_mcf_hap5000	2.28	PSR6_mcf_hap5002	NRM
TC6_mcf_hap5000	-2.94	JUC6_mcf_hap5000	NRM
TC6_mcf_hap5000	-2.94	JUC6_mcf_hap5000	NRM
TC6_qbl_hap6000	3.1	JUC6_qbl_hap60008	NRM
TC6_qbl_hap6000	2.28	PSR6_qbl_hap60024	NRM
TC6_qbl_hap6000	-2.94	JUC6_qbl_hap60008	NRM
TC6_qbl_hap6000	-2.94	JUC6_qbl_hap60008	NRM
TC6_ssto_hap700	3.1	JUC6_ssto_hap7000	HLA-F-AS1
TC6_ssto_hap700	2.56	PSR6_ssto_hap7001	HLA-F-AS1
TC6_ssto_hap700	2.54	PSR6_ssto_hap7001	HLA-F-AS1
TC6_ssto_hap700	2.38	JUC6_ssto_hap7000	HLA-F-AS1
TC6_ssto_hap700	2.07	JUC6_ssto_hap7000	HLA-F-AS1
TC6_ssto_hap700	2.01	JUC6_ssto_hap7000	HLA-F-AS1
TC6_ssto_hap700	-2.43	JUC6_ssto_hap7000	HLA-F-AS1
TC01001658.hg.1	3.09	JUC01013696.hg.1	PKP1
TC01001658.hg.1	-2.21	PSR01025658.hg.1	PKP1
TC01002388.hg.1	3.09	JUC01019818.hg.1	UBXN11
TC01002388.hg.1	2.14	JUC01019821.hg.1	UBXN11
TC01002388.hg.1	2.08	PSR01036818.hg.1	UBXN11
TC01002388.hg.1	2.07	JUC01019837.hg.1	UBXN11
TC02002769.hg.1	3.09	PSR02044466.hg.1	AAMP
TC02002769.hg.1	2.56	JUC02023329.hg.1	AAMP
TC06001532.hg.1	3.09	JUC06008526.hg.1	BAG6
TC06001532.hg.1	2.6	PSR06017544.hg.1	BAG6
TC06001532.hg.1	2.4	PSR06017617.hg.1	BAG6
TC06001532.hg.1	2.4	JUC06008523.hg.1	BAG6
TC06001532.hg.1	2.39	JUC06008546.hg.1	BAG6

TC06001532.hg.1	2.34	PSR06017611.hg.1	BAG6
TC06001532.hg.1	2.3	JUC06008563.hg.1	BAG6
TC06001532.hg.1	2.17	PSR06017552.hg.1	BAG6
TC06001532.hg.1	2.12	PSR06017556.hg.1	BAG6
TC06001532.hg.1	2.09	JUC06008532.hg.1	BAG6
TC06001532.hg.1	2.08	PSR06017609.hg.1	BAG6
TC07003331.hg.1	3.09	JUC07020964.hg.1	STAG3L1, ST
TC07003331.hg.1	2.12	JUC07020963.hg.1	STAG3L1, ST
TC07003331.hg.1	2.11	PSR07006523.hg.1	STAG3L1, ST
TC07003331.hg.1	2.08	JUC07020975.hg.1	STAG3L1, ST
TC07003331.hg.1	2.03	JUC07020959.hg.1	STAG3L1, ST
TC09001141.hg.1	3.09	JUC09007980.hg.1	
TC09001141.hg.1	2.57	PSR09014876.hg.1	
TC09001141.hg.1	2.54	JUC09007977.hg.1	
TC09001141.hg.1	2.06	PSR09014875.hg.1	
TC09001141.hg.1	-3.13	JUC09007985.hg.1	
TC12000628.hg.1	3.09	JUC12004415.hg.1	TBC1D15
TC12000628.hg.1	2.81	PSR12008367.hg.1	TBC1D15
TC12000628.hg.1	2.41	PSR12008383.hg.1	TBC1D15
TC15000735.hg.1	3.09	JUC15003371.hg.1	MORF4L1
TC15000735.hg.1	2.18	PSR15006629.hg.1	MORF4L1
TC16000722.hg.1	3.09	JUC16005799.hg.1	NPRL3
TC16000722.hg.1	2.42	PSR16010380.hg.1	NPRL3
TC16000722.hg.1	2.41	PSR16010416.hg.1	NPRL3
TC16000722.hg.1	2.13	JUC16005818.hg.1	NPRL3
TC16000722.hg.1	2.05	PSR16010382.hg.1	NPRL3
TC16000722.hg.1	-2.1	JUC16005806.hg.1	NPRL3
TC16000722.hg.1	-2.15	JUC16005798.hg.1	NPRL3
TC20000253.hg.1	3.09	JUC20001897.hg.1	CEP250
TC20000253.hg.1	2.47	JUC20001905.hg.1	CEP250
TC20000253.hg.1	2.28	PSR20003330.hg.1	CEP250
TC20000253.hg.1	2.25	JUC20001909.hg.1	CEP250
TC20000253.hg.1	2.21	JUC20001888.hg.1	CEP250
TC20000253.hg.1	2.09	JUC20001910.hg.1	CEP250
TC20000253.hg.1	2.04	PSR20003332.hg.1	CEP250
TC20000253.hg.1	-2.21	JUC20001871.hg.1	CEP250
TC20000253.hg.1	-2.37	JUC20001887.hg.1	CEP250
TC20000253.hg.1	-2.54	PSR20003315.hg.1	CEP250
TC20000253.hg.1	-2.88	PSR20003363.hg.1	CEP250
TC20000253.hg.1	-4.58	JUC20001868.hg.1	CEP250
TC6_cox_hap2000	3.09	JUC6_cox_hap20007	HLA-F-AS1
TC6_cox_hap2000	2.56	PSR6_cox_hap2002	HLA-F-AS1
TC6_cox_hap2000	2.38	PSR6_cox_hap2002	HLA-F-AS1
TC6_cox_hap2000	2.37	JUC6_cox_hap20007	HLA-F-AS1
TC6_cox_hap2000	2.06	JUC6_cox_hap20007	HLA-F-AS1
TC6_cox_hap2000	2.01	JUC6_cox_hap20007	HLA-F-AS1
TC6_cox_hap2000	-2.43	JUC6_cox_hap20007	HLA-F-AS1
TC6_mcf_hap5000	3.09	JUC6_mcf_hap5000	BAG6
TC6_mcf_hap5000	2.6	PSR6_mcf_hap5002	BAG6
TC6_mcf_hap5000	2.4	PSR6_mcf_hap5002	BAG6
TC6_mcf_hap5000	2.4	JUC6_mcf_hap5000	BAG6
TC6_mcf_hap5000	2.39	JUC6_mcf_hap5000	BAG6
TC6_mcf_hap5000	2.34	PSR6_mcf_hap5002	BAG6
TC6_mcf_hap5000	2.3	JUC6_mcf_hap5000	BAG6
TC6_mcf_hap5000	2.17	PSR6_mcf_hap5002	BAG6
TC6_mcf_hap5000	2.09	JUC6_mcf_hap5000	BAG6
TC6_mcf_hap5000	2.08	PSR6_mcf_hap5002	BAG6
TC6_qbl_hap6000	3.09	JUC6_qbl_hap60010	BAG6

TC6_qbl_hap6000′	2.6	PSR6_qbl_hap60028	BAG6
TC6_qbl_hap6000′	2.4	PSR6_qbl_hap60029	BAG6
TC6_qbl_hap6000′	2.4	JUC6_qbl_hap60010	BAG6
TC6_qbl_hap6000′	2.39	JUC6_qbl_hap60010	BAG6
TC6_qbl_hap6000′	2.34	PSR6_qbl_hap60029	BAG6
TC6_qbl_hap6000′	2.3	JUC6_qbl_hap60010	BAG6
TC6_qbl_hap6000′	2.19	PSR6_qbl_hap60028	BAG6
TC6_qbl_hap6000′	2.17	PSR6_qbl_hap60028	BAG6
TC6_qbl_hap6000′	2.09	JUC6_qbl_hap60010	BAG6
TC6_qbl_hap6000′	2.08	PSR6_qbl_hap60029	BAG6
TC03000326.hg.1	3.08	JUC03003345.hg.1	PARP3
TC03000326.hg.1	2.5	JUC03003339.hg.1	PARP3
TC03000326.hg.1	2.35	PSR03006937.hg.1	PARP3
TC03000326.hg.1	2.17	PSR03006949.hg.1	PARP3
TC03000326.hg.1	2.12	PSR03006928.hg.1	PARP3
TC03000326.hg.1	2.07	PSR03006926.hg.1	PARP3
TC03000326.hg.1	2.05	PSR03006936.hg.1	PARP3
TC03000326.hg.1	-2	PSR03006935.hg.1	PARP3
TC03000326.hg.1	-2.23	JUC03003347.hg.1	PARP3
TC04000222.hg.1	3.08	JUC04001577.hg.1	FAM114A1
TC04000222.hg.1	2.29	PSR04003255.hg.1	FAM114A1
TC04000222.hg.1	2.19	JUC04001574.hg.1	FAM114A1
TC04000222.hg.1	-2.27	JUC04001585.hg.1	FAM114A1
TC04001355.hg.1	3.08	JUC04009945.hg.1	WDFY3
TC04001355.hg.1	3	JUC04009933.hg.1	WDFY3
TC04001355.hg.1	2.04	PSR04018864.hg.1	WDFY3
TC04001355.hg.1	-2.16	JUC04009971.hg.1	WDFY3
TC05002009.hg.1	3.08	PSR05027907.hg.1	C5orf54
TC05002009.hg.1	2.7	JUC05014226.hg.1	C5orf54
TC05002009.hg.1	2.21	PSR05027899.hg.1	C5orf54
TC06001510.hg.1	3.08	JUC06008438.hg.1	IER3
TC06001510.hg.1	2.03	PSR06017128.hg.1	IER3
TC07001489.hg.1	3.08	JUC07010970.hg.1	STAG3L3, ST
TC07001489.hg.1	2.1	PSR07022322.hg.1	STAG3L3, ST
TC07001489.hg.1	2.07	JUC07010967.hg.1	STAG3L3, ST
TC07001489.hg.1	2.02	JUC07010974.hg.1	STAG3L3, ST
TC10001200.hg.1	3.08	PSR10015043.hg.1	RASGEF1A
TC10001200.hg.1	-2.03	PSR10015062.hg.1	RASGEF1A
TC10001200.hg.1	-2.37	JUC10008740.hg.1	RASGEF1A
TC15001277.hg.1	3.08	JUC15006438.hg.1	ZSCAN29
TC15001277.hg.1	2.24	JUC15006434.hg.1	ZSCAN29
TC15001277.hg.1	2.1	PSR15011871.hg.1	ZSCAN29
TC16001112.hg.1	3.08	JUC16008705.hg.1	LOC388276
TC16001112.hg.1	-2.02	PSR16015542.hg.1	LOC388276
TC19001787.hg.1	3.08	PSR19024281.hg.1	FPR1
TC19001787.hg.1	-2.86	JUC19013815.hg.1	FPR1
TC19001787.hg.1	-3.05	JUC19013813.hg.1	FPR1
TC21000477.hg.1	3.08	JUC21002989.hg.1	PRDM15
TC21000477.hg.1	2.56	JUC21002959.hg.1	PRDM15
TC21000477.hg.1	2.48	JUC21002996.hg.1	PRDM15
TC21000477.hg.1	2.13	PSR21005795.hg.1	PRDM15
TC21000477.hg.1	2.12	JUC21002957.hg.1	PRDM15
TC21000477.hg.1	-2.62	JUC21002987.hg.1	PRDM15
TC01001585.hg.1	3.07	JUC01013062.hg.1	LAMC2
TC01001585.hg.1	2.91	JUC01013064.hg.1	LAMC2
TC01001585.hg.1	2.33	JUC01013057.hg.1	LAMC2
TC01001585.hg.1	-2.19	JUC01013076.hg.1	LAMC2
TC01001585.hg.1	-2.48	JUC01013079.hg.1	LAMC2

TC01001585.hg.1	-2.57	JUC01013074.hg.1	LAMC2
TC01001585.hg.1	-2.61	PSR01024555.hg.1	LAMC2
TC01001585.hg.1	-3.38	JUC01013072.hg.1	LAMC2
TC01003197.hg.1	3.07	PSR01049013.hg.1	MTMR11
TC01003197.hg.1	2.99	JUC01026061.hg.1	MTMR11
TC01003197.hg.1	2.05	JUC01026055.hg.1	MTMR11
TC01003197.hg.1	-2.91	PSR01048995.hg.1	MTMR11
TC06000377.hg.1	3.07	JUC06001566.hg.1	APOM
TC06000377.hg.1	2.34	PSR06003729.hg.1	APOM
TC08000324.hg.1	3.07	PSR08004573.hg.1	AP3M2
TC08000324.hg.1	2.04	JUC08002307.hg.1	AP3M2
TC08000324.hg.1	2.01	PSR08004586.hg.1	AP3M2
TC08000324.hg.1	-2.11	PSR08004572.hg.1	AP3M2
TC08001427.hg.1	3.07	JUC08009429.hg.1	RBM12B
TC08001427.hg.1	2.11	PSR08018531.hg.1	RBM12B
TC08001427.hg.1	-2.12	PSR08018514.hg.1	RBM12B
TC09000269.hg.1	3.07	JUC09001526.hg.1	LOC1005096
TC09000269.hg.1	2.51	PSR09002933.hg.1	LOC1005096
TC11000770.hg.1	3.07	JUC11005135.hg.1	ATG16L2
TC11000770.hg.1	2.66	JUC11005137.hg.1	ATG16L2
TC11000770.hg.1	2.02	PSR11009914.hg.1	ATG16L2
TC11000770.hg.1	-2.01	PSR11009891.hg.1	ATG16L2
TC11000770.hg.1	-2.16	PSR11009894.hg.1	ATG16L2
TC11000770.hg.1	-2.3	JUC11005146.hg.1	ATG16L2
TC13000686.hg.1	3.07	JUC13004671.hg.1	NEK3
TC13000686.hg.1	2.15	JUC13004665.hg.1	NEK3
TC13000686.hg.1	-2.11	PSR13007994.hg.1	NEK3
TC13000686.hg.1	-2.45	PSR13007996.hg.1	NEK3
TC17000324.hg.1	3.07	JUC17002217.hg.1	SPAG5-AS1
TC17000324.hg.1	2.01	PSR17003948.hg.1	SPAG5-AS1
TC17001897.hg.1	3.07	PSR17025736.hg.1	PRPSAP1
TC17001897.hg.1	2.68	PSR17025719.hg.1	PRPSAP1
TC17001897.hg.1	2.61	PSR17025742.hg.1	PRPSAP1
TC17001897.hg.1	2.22	PSR17025729.hg.1	PRPSAP1
TC17001897.hg.1	2.1	JUC17014463.hg.1	PRPSAP1
TC17001897.hg.1	2.05	PSR17025722.hg.1	PRPSAP1
TC17001897.hg.1	-2.06	JUC17014455.hg.1	PRPSAP1
TC17001897.hg.1	-2.6	JUC17014460.hg.1	PRPSAP1
TC21000138.hg.1	3.07	JUC21000639.hg.1	CLIC6
TC21000138.hg.1	2.14	PSR21001194.hg.1	CLIC6
TC21000138.hg.1	-2.2	JUC21000634.hg.1	CLIC6
TC21000138.hg.1	-4.81	PSR21001193.hg.1	CLIC6
TC21000138.hg.1	-5.88	PSR21001192.hg.1	CLIC6
TC6_cox_hap2000	3.07	JUC6_cox_hap2000	APOM
TC6_cox_hap2000	2.34	PSR6_cox_hap2001	APOM
TC6_dbb_hap3000	3.07	JUC6_dbb_hap3000	APOM
TC6_dbb_hap3000	2.34	PSR6_dbb_hap3001	APOM
TC6_mann_hap40	3.07	JUC6_mann_hap400	APOM
TC6_mann_hap40	2.34	PSR6_mann_hap400	APOM
TC6_mcf_hap5000	3.07	JUC6_mcf_hap5000	APOM
TC6_mcf_hap5000	2.34	PSR6_mcf_hap5000	APOM
TC6_qbl_hap6000	3.07	JUC6_qbl_hap60004	APOM
TC6_qbl_hap6000	2.34	PSR6_qbl_hap60010	APOM
TC02000725.hg.1	3.06	JUC02005961.hg.1	CBWD2, CBV
TC02000725.hg.1	2.42	JUC02005972.hg.1	CBWD2, CBV
TC02000725.hg.1	2.21	JUC02005980.hg.1	CBWD2, CBV
TC02000725.hg.1	2.12	JUC02005966.hg.1	CBWD2, CBV
TC02000725.hg.1	-2.08	PSR02011277.hg.1	CBWD2, CBV

TC02000725.hg.1	-2.08	PSR02011285.hg.1	CBWD2, CBV
TC02000725.hg.1	-2.21	JUC02005965.hg.1	CBWD2, CBV
TC02000725.hg.1	-2.27	PSR02011291.hg.1	CBWD2, CBV
TC02000725.hg.1	-2.33	PSR02011294.hg.1	CBWD2, CBV
TC02002657.hg.1	3.06	PSR02042716.hg.1	FONG
TC02002657.hg.1	2.68	PSR02042717.hg.1	FONG
TC02002657.hg.1	2.49	JUC02022351.hg.1	FONG
TC02002657.hg.1	-2.14	JUC02022350.hg.1	FONG
TC02002657.hg.1	-2.42	PSR02042711.hg.1	FONG
TC03001680.hg.1	3.06	JUC03015068.hg.1	B4GALT4
TC03001680.hg.1	2.69	JUC03015059.hg.1	B4GALT4
TC03001680.hg.1	2.45	JUC03015056.hg.1	B4GALT4
TC03001680.hg.1	2.38	JUC03015048.hg.1	B4GALT4
TC03001680.hg.1	2.35	PSR03030326.hg.1	B4GALT4
TC03001680.hg.1	-2.07	PSR03030293.hg.1	B4GALT4
TC03003334.hg.1	3.06	JUC03023258.hg.1	DGKG
TC03003334.hg.1	-2.01	PSR03036963.hg.1	DGKG
TC03003334.hg.1	-2.31	JUC03023273.hg.1	DGKG
TC03003334.hg.1	-2.43	JUC03023250.hg.1	DGKG
TC05000833.hg.1	3.06	JUC05006267.hg.1	SYNPO
TC05000833.hg.1	2.58	PSR05011993.hg.1	SYNPO
TC05000833.hg.1	2.02	PSR05011992.hg.1	SYNPO
TC07000795.hg.1	3.06	JUC07005917.hg.1	SMO
TC07000795.hg.1	2.36	JUC07005916.hg.1	SMO
TC07000795.hg.1	2.15	PSR07012542.hg.1	SMO
TC07000795.hg.1	2.01	JUC07005904.hg.1	SMO
TC0X000061.hg.1	3.06	JUC0X000451.hg.1	OFD1
TC0X000061.hg.1	2.54	JUC0X000480.hg.1	OFD1
TC0X000061.hg.1	2.1	PSR0X000859.hg.1	OFD1
TC0X000061.hg.1	2.05	JUC0X000475.hg.1	OFD1
TC0X000061.hg.1	-3.27	JUC0X000450.hg.1	OFD1
TC0X000963.hg.1	3.06	PSR0X012851.hg.1	BCOR
TC0X000963.hg.1	2.94	PSR0X012832.hg.1	BCOR
TC0X000963.hg.1	2.93	PSR0X012855.hg.1	BCOR
TC0X000963.hg.1	2.71	PSR0X012860.hg.1	BCOR
TC0X000963.hg.1	2.49	JUC0X006448.hg.1	BCOR
TC0X000963.hg.1	2.19	JUC0X006432.hg.1	BCOR
TC0X000963.hg.1	2.13	PSR0X012861.hg.1	BCOR
TC0X000963.hg.1	2	JUC0X006439.hg.1	BCOR
TC0X001184.hg.1	3.06	PSR0X015990.hg.1	HMGN5
TC0X001184.hg.1	2.96	JUC0X008107.hg.1	HMGN5
TC0X001184.hg.1	2.68	JUC0X008108.hg.1	HMGN5
TC0X001184.hg.1	-2.05	PSR0X015974.hg.1	HMGN5
TC0X001184.hg.1	-2.5	JUC0X008101.hg.1	HMGN5
TC0X001184.hg.1	-2.57	JUC0X008098.hg.1	HMGN5
TC0X001184.hg.1	-2.9	JUC0X008104.hg.1	HMGN5
TC0X001326.hg.1	3.06	PSR0X017830.hg.1	CUL4B
TC0X001326.hg.1	3	PSR0X017832.hg.1	CUL4B
TC0X001326.hg.1	2.68	PSR0X017826.hg.1	CUL4B
TC0X001326.hg.1	2.43	PSR0X017827.hg.1	CUL4B
TC0X001326.hg.1	2.34	PSR0X017825.hg.1	CUL4B
TC0X001326.hg.1	2.13	PSR0X017817.hg.1	CUL4B
TC0X001326.hg.1	-2.39	JUC0X009070.hg.1	CUL4B
TC12001526.hg.1	3.06	PSR12020042.hg.1	KRT5
TC12001526.hg.1	2	PSR12020047.hg.1	KRT5
TC12001526.hg.1	-2.01	PSR12020050.hg.1	KRT5
TC12001526.hg.1	-2.2	JUC12011129.hg.1	KRT5
TC12001526.hg.1	-2.57	JUC12011128.hg.1	KRT5

TC12001526.hg.1	-4.61	JUC12011125.hg.1	KRT5
TC12001526.hg.1	-4.79	JUC12011127.hg.1	KRT5
TC17000375.hg.1	3.06	PSR17004749.hg.1	MIR632, ZNF
TC17000375.hg.1	2.63	JUC17002730.hg.1	MIR632, ZNF
TC17000375.hg.1	2.18	JUC17002725.hg.1	MIR632, ZNF
TC6_cox_hap2000	3.06	JUC6_cox_hap2002(	DDX39B, SN(
TC6_cox_hap2000	2.83	PSR6_cox_hap2002	DDX39B, SN(
TC6_cox_hap2000	2.54	JUC6_cox_hap2002(	DDX39B, SN(
TC6_cox_hap2000	2.16	PSR6_cox_hap2002	DDX39B, SN(
TC6_cox_hap2000	-2.72	PSR6_cox_hap2002	DDX39B, SN(
TC6_dbb_hap3000	3.06	JUC6_dbb_hap3001	DDX39B, SN(
TC6_dbb_hap3000	2.83	PSR6_dbb_hap3002	DDX39B, SN(
TC6_dbb_hap3000	2.54	JUC6_dbb_hap3001	DDX39B, SN(
TC6_dbb_hap3000	2.16	PSR6_dbb_hap3002	DDX39B, SN(
TC6_dbb_hap3000	-2.72	PSR6_dbb_hap3002	DDX39B, SN(
TC6_mann_hap40(	3.06	JUC6_mann_hap400	DDX39B, SN(
TC6_mann_hap40(	2.83	PSR6_mann_hap40(	DDX39B, SN(
TC6_mann_hap40(	2.54	JUC6_mann_hap400	DDX39B, SN(
TC6_mann_hap40(	2.16	PSR6_mann_hap40(	DDX39B, SN(
TC6_mann_hap40(	-2.72	PSR6_mann_hap40(	DDX39B, SN(
TC6_mcf_hap5000	3.06	JUC6_mcf_hap5001	DDX39B, SN(
TC6_mcf_hap5000	2.83	PSR6_mcf_hap5002	DDX39B, SN(
TC6_mcf_hap5000	2.54	JUC6_mcf_hap5001	DDX39B, SN(
TC6_mcf_hap5000	2.16	PSR6_mcf_hap5002	DDX39B, SN(
TC6_mcf_hap5000	-2.72	PSR6_mcf_hap5002	DDX39B, SN(
TC6_qbl_hap6000	3.06	JUC6_qbl_hap60020	DDX39B, SN(
TC6_qbl_hap6000	2.83	PSR6_qbl_hap60027	DDX39B, SN(
TC6_qbl_hap6000	2.54	JUC6_qbl_hap60020	DDX39B, SN(
TC6_qbl_hap6000	2.16	PSR6_qbl_hap60027	DDX39B, SN(
TC6_qbl_hap6000	-2.72	PSR6_qbl_hap60027	DDX39B, SN(
TC01002825.hg.1	3.05	PSR01043966.hg.1	MCOLN3
TC01002825.hg.1	2.66	PSR01043950.hg.1	MCOLN3
TC01002825.hg.1	2.01	JUC01023579.hg.1	MCOLN3
TC01002825.hg.1	2	PSR01043975.hg.1	MCOLN3
TC02000944.hg.1	3.05	JUC02007425.hg.1	GALNT13
TC02000944.hg.1	2.82	JUC02007435.hg.1	GALNT13
TC02000944.hg.1	2.35	PSR02013820.hg.1	GALNT13
TC02000944.hg.1	2.04	JUC02007434.hg.1	GALNT13
TC02000944.hg.1	-2.02	PSR02013836.hg.1	GALNT13
TC02000944.hg.1	-2.04	JUC02007440.hg.1	GALNT13
TC02000944.hg.1	-2.09	JUC02007424.hg.1	GALNT13
TC02000944.hg.1	-2.29	PSR02013853.hg.1	GALNT13
TC02000944.hg.1	-2.42	JUC02007419.hg.1	GALNT13
TC02000944.hg.1	-2.52	JUC02007430.hg.1	GALNT13
TC02000944.hg.1	-2.76	JUC02007433.hg.1	GALNT13
TC02000944.hg.1	-2.99	PSR02013842.hg.1	GALNT13
TC02000944.hg.1	-3.19	PSR02013849.hg.1	GALNT13
TC02000944.hg.1	-3.37	PSR02013821.hg.1	GALNT13
TC02000944.hg.1	-3.47	JUC02007431.hg.1	GALNT13
TC02000944.hg.1	-3.69	PSR02013818.hg.1	GALNT13
TC02000944.hg.1	-4.16	PSR02013817.hg.1	GALNT13
TC02000944.hg.1	-4.25	PSR02013843.hg.1	GALNT13
TC02000944.hg.1	-5.66	PSR02013844.hg.1	GALNT13
TC02002136.hg.1	3.05	JUC02018004.hg.1	AFF3
TC02002136.hg.1	2.86	JUC02017989.hg.1	AFF3
TC02002136.hg.1	2.43	PSR02034862.hg.1	AFF3
TC02002136.hg.1	2.38	JUC02017977.hg.1	AFF3
TC02002136.hg.1	2.32	PSR02034927.hg.1	AFF3

TC02002136.hg.1	2.26	PSR02034899.hg.1	AFF3
TC02002136.hg.1	2.24	PSR02034913.hg.1	AFF3
TC02002136.hg.1	2.18	PSR02034907.hg.1	AFF3
TC02002136.hg.1	2.17	PSR02034905.hg.1	AFF3
TC02002136.hg.1	2.12	PSR02034902.hg.1	AFF3
TC02002136.hg.1	2.05	JUC02017997.hg.1	AFF3
TC02002136.hg.1	-2.02	JUC02018005.hg.1	AFF3
TC02002136.hg.1	-2.16	PSR02034869.hg.1	AFF3
TC02002136.hg.1	-2.19	PSR02034857.hg.1	AFF3
TC02002136.hg.1	-2.48	JUC02017991.hg.1	AFF3
TC02002136.hg.1	-2.75	JUC02017993.hg.1	AFF3
TC02002136.hg.1	-2.98	JUC02017988.hg.1	AFF3
TC02002136.hg.1	-2.99	PSR02034883.hg.1	AFF3
TC02002136.hg.1	-3.58	JUC02018018.hg.1	AFF3
TC02002136.hg.1	-5.35	JUC02017990.hg.1	AFF3
TC03000087.hg.1	3.05	JUC03000968.hg.1	SLC6A6
TC03000087.hg.1	2.17	JUC03000958.hg.1	SLC6A6
TC03000087.hg.1	2.15	JUC03000976.hg.1	SLC6A6
TC03000087.hg.1	2.04	PSR03001834.hg.1	SLC6A6
TC03000634.hg.1	3.05	PSR03011428.hg.1	PARP14
TC03000634.hg.1	2.67	JUC03005707.hg.1	PARP14
TC03000634.hg.1	2.34	PSR03011459.hg.1	PARP14
TC03000634.hg.1	2.02	PSR03011462.hg.1	PARP14
TC03000634.hg.1	-2.14	JUC03005702.hg.1	PARP14
TC03000634.hg.1	-2.16	PSR03011460.hg.1	PARP14
TC03000634.hg.1	-2.56	PSR03011447.hg.1	PARP14
TC03001431.hg.1	3.05	JUC03012618.hg.1	NPRL2
TC03001431.hg.1	2.28	PSR03025803.hg.1	NPRL2
TC03001431.hg.1	2.13	PSR03025794.hg.1	NPRL2
TC08000216.hg.1	3.05	PSR08003107.hg.1	ELP3
TC08000216.hg.1	2.91	JUC08001542.hg.1	ELP3
TC08000216.hg.1	-2.52	JUC08001538.hg.1	ELP3
TC09001220.hg.1	3.05	PSR09015653.hg.1	NMRK1
TC09001220.hg.1	3.02	JUC09008417.hg.1	NMRK1
TC09001220.hg.1	2.63	PSR09015646.hg.1	NMRK1
TC09001220.hg.1	2.57	PSR09015655.hg.1	NMRK1
TC09001220.hg.1	2.54	JUC09008413.hg.1	NMRK1
TC09001220.hg.1	2.17	PSR09015658.hg.1	NMRK1
TC09001220.hg.1	2.1	PSR09015636.hg.1	NMRK1
TC11000216.hg.1	3.05	PSR11002915.hg.1	INSC
TC11000216.hg.1	2.65	JUC11001411.hg.1	INSC
TC11000216.hg.1	-2.2	PSR11002904.hg.1	INSC
TC11000216.hg.1	-2.37	PSR11002896.hg.1	INSC
TC12001367.hg.1	3.05	PSR12017879.hg.1	AMN1
TC12001367.hg.1	2.86	PSR12017881.hg.1	AMN1
TC12001367.hg.1	2.23	PSR12017883.hg.1	AMN1
TC12001367.hg.1	2.18	JUC12009934.hg.1	AMN1
TC12001367.hg.1	2.06	PSR12017878.hg.1	AMN1
TC12001367.hg.1	2.05	PSR12017861.hg.1	AMN1
TC12001367.hg.1	-2.44	JUC12009935.hg.1	AMN1
TC12001377.hg.1	3.05	JUC12009969.hg.1	PKP2
TC12001377.hg.1	2.39	JUC12009965.hg.1	PKP2
TC12001377.hg.1	2.15	JUC12009960.hg.1	PKP2
TC12001377.hg.1	-2.03	PSR12017914.hg.1	PKP2
TC12001377.hg.1	-2.17	PSR12017925.hg.1	PKP2
TC12001377.hg.1	-2.36	PSR12017929.hg.1	PKP2
TC12001377.hg.1	-2.4	PSR12017932.hg.1	PKP2
TC12001377.hg.1	-2.42	PSR12017937.hg.1	PKP2

TC12001377.hg.1	-3.1	JUC12009958.hg.1	PKP2
TC12001377.hg.1	-3.11	PSR12017931.hg.1	PKP2
TC12001377.hg.1	-3.12	PSR12017926.hg.1	PKP2
TC12001377.hg.1	-3.75	JUC12009971.hg.1	PKP2
TC17002870.hg.1	3.05	PSR17002644.hg.1	CCDC144A
TC17002870.hg.1	2.32	PSR17002637.hg.1	CCDC144A
TC17002870.hg.1	2.08	JUC17018585.hg.1	CCDC144A
TC22000165.hg.1	3.05	JUC22000977.hg.1	ADRBK2
TC22000165.hg.1	2.62	PSR22003107.hg.1	ADRBK2
TC22000165.hg.1	2.5	JUC22000987.hg.1	ADRBK2
TC22000165.hg.1	2.33	JUC22000965.hg.1	ADRBK2
TC22000165.hg.1	2.06	JUC22000968.hg.1	ADRBK2
TC22000165.hg.1	-2.55	PSR22003113.hg.1	ADRBK2
TC22000165.hg.1	-2.81	JUC22000980.hg.1	ADRBK2
TC6_apd_hap1000	3.05	JUC6_apd_hap1000	DDX39B, ATF
TC6_apd_hap1000	-2.73	PSR6_apd_hap1001	DDX39B, ATF
TC6_apd_hap1000	-3.61	PSR6_apd_hap1001	DDX39B, ATF
TC01002903.hg.1	3.04	JUC01024300.hg.1	DPYD
TC01002903.hg.1	-2.41	PSR01045227.hg.1	DPYD
TC04000040.hg.1	3.04	JUC04000421.hg.1	NOP14-AS1
TC04000040.hg.1	2.28	PSR04001049.hg.1	NOP14-AS1
TC05000730.hg.1	3.04	JUC05005558.hg.1	IK, MIR3655
TC05000730.hg.1	2.12	JUC05005551.hg.1	IK, MIR3655
TC05000730.hg.1	2.02	PSR05010527.hg.1	IK, MIR3655
TC05002110.hg.1	3.04	JUC05014829.hg.1	PDLIM7
TC05002110.hg.1	-2.35	PSR05029126.hg.1	PDLIM7
TC05002110.hg.1	-2.38	JUC05014816.hg.1	PDLIM7
TC05002110.hg.1	-3.06	PSR05029142.hg.1	PDLIM7
TC05002110.hg.1	-3.35	PSR05029143.hg.1	PDLIM7
TC07001143.hg.1	3.04	PSR07017828.hg.1	ICA1
TC07001143.hg.1	2.69	JUC07008646.hg.1	ICA1
TC07001143.hg.1	2.28	JUC07008661.hg.1	ICA1
TC07001143.hg.1	2.17	PSR07017835.hg.1	ICA1
TC07001143.hg.1	2.07	PSR07017829.hg.1	ICA1
TC07001143.hg.1	2.04	JUC07008663.hg.1	ICA1
TC07001143.hg.1	-2.02	PSR07017816.hg.1	ICA1
TC07001143.hg.1	-2.04	PSR07017838.hg.1	ICA1
TC07001143.hg.1	-2.08	JUC07008674.hg.1	ICA1
TC07001143.hg.1	-2.09	PSR07017852.hg.1	ICA1
TC07001143.hg.1	-2.12	PSR07017819.hg.1	ICA1
TC07001143.hg.1	-2.16	PSR07017820.hg.1	ICA1
TC07001143.hg.1	-2.22	PSR07017809.hg.1	ICA1
TC07001143.hg.1	-2.39	PSR07017822.hg.1	ICA1
TC07001143.hg.1	-2.39	PSR07017823.hg.1	ICA1
TC07001143.hg.1	-2.4	PSR07017858.hg.1	ICA1
TC07001143.hg.1	-2.5	PSR07017837.hg.1	ICA1
TC07001143.hg.1	-2.5	JUC07008647.hg.1	ICA1
TC07001143.hg.1	-2.6	PSR07017834.hg.1	ICA1
TC07001143.hg.1	-2.81	PSR07017849.hg.1	ICA1
TC07001143.hg.1	-2.89	PSR07017847.hg.1	ICA1
TC07001143.hg.1	-4.88	JUC07008650.hg.1	ICA1
TC07001143.hg.1	-10.31	JUC07008666.hg.1	ICA1
TC08001370.hg.1	3.04	JUC08009012.hg.1	ZFAND1
TC08001370.hg.1	2.12	JUC08008996.hg.1	ZFAND1
TC08001370.hg.1	-2.22	PSR08017704.hg.1	ZFAND1
TC08001370.hg.1	-2.76	JUC08009000.hg.1	ZFAND1
TC0X000658.hg.1	3.04	JUC0X004381.hg.1	FHL1
TC0X000658.hg.1	3.02	JUC0X004383.hg.1	FHL1

TC0X000658.hg.1	2.71	PSR0X008658.hg.1	FHL1
TC0X000658.hg.1	2.68	PSR0X008660.hg.1	FHL1
TC0X000658.hg.1	2.03	PSR0X008633.hg.1	FHL1
TC0X000658.hg.1	-2.1	JUC0X004382.hg.1	FHL1
TC0X000658.hg.1	-4.11	PSR0X008661.hg.1	FHL1
TC0X000658.hg.1	-19.57	PSR0X008662.hg.1	FHL1
TC10000848.hg.1	3.04	JUC10005774.hg.1	ENO4
TC10000848.hg.1	2.87	JUC10005773.hg.1	ENO4
TC10000848.hg.1	2.49	PSR10010293.hg.1	ENO4
TC10000848.hg.1	2.42	JUC10005783.hg.1	ENO4
TC10000848.hg.1	2.41	JUC10005779.hg.1	ENO4
TC10000848.hg.1	2.04	PSR10010284.hg.1	ENO4
TC13000468.hg.1	3.04	PSR13005310.hg.1	SKA3
TC13000468.hg.1	2.89	PSR13005323.hg.1	SKA3
TC13000468.hg.1	2.87	JUC13003205.hg.1	SKA3
TC13000468.hg.1	2.17	PSR13005321.hg.1	SKA3
TC13000468.hg.1	2.08	PSR13005311.hg.1	SKA3
TC13000468.hg.1	2.04	PSR13005322.hg.1	SKA3
TC15000449.hg.1	3.04	PSR15004285.hg.1	CCNB2
TC15000449.hg.1	2.74	PSR15004276.hg.1	CCNB2
TC15000449.hg.1	2.17	PSR15004284.hg.1	CCNB2
TC15000449.hg.1	-2.2	PSR15004279.hg.1	CCNB2
TC15000449.hg.1	-2.27	JUC15002113.hg.1	CCNB2
TC15000449.hg.1	-2.71	JUC15002118.hg.1	CCNB2
TC17001644.hg.1	3.04	JUC17012325.hg.1	HOXB5
TC17001644.hg.1	-2.13	PSR17022033.hg.1	HOXB5
TC22000370.hg.1	3.04	PSR22007080.hg.1	PARVB
TC22000370.hg.1	2.11	JUC22002629.hg.1	PARVB
TC22000370.hg.1	2.04	PSR22007061.hg.1	PARVB
TC01002294.hg.1	3.03	PSR01035157.hg.1	EMC1
TC01002294.hg.1	2.83	PSR01035181.hg.1	EMC1
TC01002294.hg.1	2.61	JUC01018899.hg.1	EMC1
TC01002294.hg.1	2.35	PSR01035169.hg.1	EMC1
TC01002294.hg.1	2.28	PSR01035153.hg.1	EMC1
TC01002294.hg.1	2.18	PSR01035175.hg.1	EMC1
TC01002294.hg.1	-3.35	JUC01018912.hg.1	EMC1
TC01002294.hg.1	-9.29	JUC01018889.hg.1	EMC1
TC01004007.hg.1	3.03	JUC01031730.hg.1	HEATR1
TC01004007.hg.1	2.27	JUC01031739.hg.1	HEATR1
TC01004007.hg.1	2.05	PSR01060648.hg.1	HEATR1
TC01004007.hg.1	-2.05	JUC01031735.hg.1	HEATR1
TC01004007.hg.1	-2.28	JUC01031746.hg.1	HEATR1
TC02000464.hg.1	3.03	PSR02007263.hg.1	DOK1
TC02000464.hg.1	2.92	PSR02007262.hg.1	DOK1
TC02000464.hg.1	2.65	PSR02007260.hg.1	DOK1
TC02000464.hg.1	2.64	JUC02003679.hg.1	DOK1
TC02000464.hg.1	2.33	JUC02003689.hg.1	DOK1
TC02000464.hg.1	2.24	JUC02003684.hg.1	DOK1
TC02000464.hg.1	-9.71	JUC02003690.hg.1	DOK1
TC02002445.hg.1	3.03	PSR02038535.hg.1	NR4A2
TC02002445.hg.1	2.57	JUC02020162.hg.1	NR4A2
TC02002445.hg.1	2.43	JUC02020163.hg.1	NR4A2
TC02002445.hg.1	2.29	PSR02038536.hg.1	NR4A2
TC02002445.hg.1	2.19	PSR02038518.hg.1	NR4A2
TC02002445.hg.1	2.18	PSR02038517.hg.1	NR4A2
TC02002445.hg.1	-2.04	PSR02038507.hg.1	NR4A2
TC02002445.hg.1	-2.3	PSR02038523.hg.1	NR4A2
TC04001335.hg.1	3.03	JUC04009759.hg.1	TMEM150C

TC04001335.hg.1	2.6	JUC04009751.hg.1	TMEM150C
TC04001335.hg.1	2.43	JUC04009765.hg.1	TMEM150C
TC04001335.hg.1	2.37	JUC04009749.hg.1	TMEM150C
TC04001335.hg.1	2.07	PSR04018378.hg.1	TMEM150C
TC04001335.hg.1	-2.44	JUC04009750.hg.1	TMEM150C
TC04001335.hg.1	-2.75	JUC04009764.hg.1	TMEM150C
TC04001335.hg.1	-3.03	JUC04009756.hg.1	TMEM150C
TC04001335.hg.1	-3.11	JUC04009752.hg.1	TMEM150C
TC04001335.hg.1	-4.1	PSR04018381.hg.1	TMEM150C
TC04001335.hg.1	-4.5	PSR04018410.hg.1	TMEM150C
TC04001335.hg.1	-4.56	JUC04009766.hg.1	TMEM150C
TC04001335.hg.1	-7.46	PSR04018405.hg.1	TMEM150C
TC04001335.hg.1	-8.17	PSR04018411.hg.1	TMEM150C
TC04001335.hg.1	-9.97	PSR04018409.hg.1	TMEM150C
TC04001335.hg.1	-11.1	PSR04018404.hg.1	TMEM150C
TC05001053.hg.1	3.03	JUC05007963.hg.1	SQSTM1
TC05001053.hg.1	2.65	PSR05015411.hg.1	SQSTM1
TC05001053.hg.1	2.35	PSR05015415.hg.1	SQSTM1
TC05001053.hg.1	2.32	PSR05015423.hg.1	SQSTM1
TC05001053.hg.1	2.3	PSR05015416.hg.1	SQSTM1
TC05001053.hg.1	2.19	JUC05007968.hg.1	SQSTM1
TC05001053.hg.1	2.15	PSR05015413.hg.1	SQSTM1
TC05001053.hg.1	2.12	PSR05015418.hg.1	SQSTM1
TC05001053.hg.1	-3.57	PSR05015452.hg.1	SQSTM1
TC05001053.hg.1	-4.37	JUC05007969.hg.1	SQSTM1
TC05001053.hg.1	-4.56	PSR05015449.hg.1	SQSTM1
TC06004060.hg.1	3.03	JUC06020807.hg.1	HLA-B
TC06004060.hg.1	2.54	PSR06017383.hg.1	HLA-B
TC06004060.hg.1	2.28	JUC06020797.hg.1	HLA-B
TC06004060.hg.1	2.07	PSR06017361.hg.1	HLA-B
TC06004060.hg.1	-2.37	PSR06017365.hg.1	HLA-B
TC06004060.hg.1	-2.63	JUC06020804.hg.1	HLA-B
TC06004060.hg.1	-2.93	PSR06017382.hg.1	HLA-B
TC09000536.hg.1	3.03	JUC09003264.hg.1	FSD1L
TC09000536.hg.1	2.23	PSR09005883.hg.1	FSD1L
TC09000536.hg.1	-2.09	JUC09003258.hg.1	FSD1L
TC09000536.hg.1	-2.18	JUC09003270.hg.1	FSD1L
TC09000536.hg.1	-2.36	JUC09003267.hg.1	FSD1L
TC09000536.hg.1	-2.54	JUC09003251.hg.1	FSD1L
TC09001025.hg.1	3.03	JUC09007318.hg.1	UBAP2, SNO
TC09001025.hg.1	-2.03	PSR09013435.hg.1	UBAP2, SNO
TC09001025.hg.1	-2.13	JUC09007314.hg.1	UBAP2, SNO
TC09001025.hg.1	-2.54	PSR09013430.hg.1	UBAP2, SNO
TC10000752.hg.1	3.03	PSR10008864.hg.1	GBF1
TC10000752.hg.1	2.19	JUC10004942.hg.1	GBF1
TC10000764.hg.1	3.03	JUC10005072.hg.1	WBP1L
TC10000764.hg.1	-2.32	PSR10009106.hg.1	WBP1L
TC10000764.hg.1	-2.36	PSR10009092.hg.1	WBP1L
TC10000764.hg.1	-2.47	PSR10009105.hg.1	WBP1L
TC10000764.hg.1	-2.78	JUC10005065.hg.1	WBP1L
TC10000764.hg.1	-3.01	PSR10009108.hg.1	WBP1L
TC10000764.hg.1	-4.54	JUC10005066.hg.1	WBP1L
TC10001022.hg.1	3.03	JUC10007378.hg.1	KIN
TC10001022.hg.1	-2.28	PSR10012733.hg.1	KIN
TC10001022.hg.1	-2.93	JUC10007367.hg.1	KIN
TC10001022.hg.1	-3.21	JUC10007381.hg.1	KIN
TC11001750.hg.1	3.03	JUC11010992.hg.1	NUP160
TC11001750.hg.1	-2.25	JUC11010998.hg.1	NUP160

TC11001750.hg.1	-2.33	JUC11010977.hg.1	NUP160
TC11001750.hg.1	-2.6	JUC11011011.hg.1	NUP160
TC11001750.hg.1	-2.83	PSR11020546.hg.1	NUP160
TC11001750.hg.1	-3.63	JUC11010979.hg.1	NUP160
TC11003478.hg.1	3.03	PSR11018494.hg.1	SAA2, RN5S3
TC11003478.hg.1	2.54	PSR11018493.hg.1	SAA2, RN5S3
TC11003478.hg.1	-2.73	PSR11018500.hg.1	SAA2, RN5S3
TC11003478.hg.1	-3.03	PSR11018492.hg.1	SAA2, RN5S3
TC11003478.hg.1	-3.24	PSR11018497.hg.1	SAA2, RN5S3
TC11003478.hg.1	-3.98	PSR11018496.hg.1	SAA2, RN5S3
TC11003478.hg.1	-4.76	PSR11018488.hg.1	SAA2, RN5S3
TC11003478.hg.1	-6.41	PSR11018503.hg.1	SAA2, RN5S3
TC11003478.hg.1	-7.4	PSR11018486.hg.1	SAA2, RN5S3
TC11003478.hg.1	-7.56	PSR11018485.hg.1	SAA2, RN5S3
TC11003478.hg.1	-9.13	JUC11019453.hg.1	SAA2, RN5S3
TC11003478.hg.1	-10.98	JUC11019454.hg.1	SAA2, RN5S3
TC20000096.hg.1	3.03	JUC20000717.hg.1	MACROD2
TC20000096.hg.1	2.62	PSR20001336.hg.1	MACROD2
TC20000096.hg.1	2.49	JUC20000689.hg.1	MACROD2
TC20000096.hg.1	2.08	PSR20001349.hg.1	MACROD2
TC03001166.hg.1	3.02	JUC03010082.hg.1	VGLL4
TC03001166.hg.1	2.57	JUC03010075.hg.1	VGLL4
TC03001166.hg.1	2.22	JUC03010063.hg.1	VGLL4
TC03001166.hg.1	-2.11	PSR03020164.hg.1	VGLL4
TC03001166.hg.1	-2.77	PSR03020172.hg.1	VGLL4
TC03001489.hg.1	3.02	JUC03013206.hg.1	IL17RD
TC03001489.hg.1	2.81	PSR03027091.hg.1	IL17RD
TC03001489.hg.1	2.76	PSR03027086.hg.1	IL17RD
TC03001489.hg.1	2.56	PSR03027092.hg.1	IL17RD
TC03001489.hg.1	2.53	PSR03027087.hg.1	IL17RD
TC03001489.hg.1	2.52	JUC03013211.hg.1	IL17RD
TC03001489.hg.1	2.44	JUC03013208.hg.1	IL17RD
TC03001489.hg.1	2.43	PSR03027070.hg.1	IL17RD
TC03001489.hg.1	2.28	PSR03027081.hg.1	IL17RD
TC03001489.hg.1	2.14	JUC03013216.hg.1	IL17RD
TC03001489.hg.1	2.13	JUC03013218.hg.1	IL17RD
TC03001489.hg.1	2.11	PSR03027088.hg.1	IL17RD
TC03001489.hg.1	2.1	PSR03027084.hg.1	IL17RD
TC03001489.hg.1	2.04	JUC03013203.hg.1	IL17RD
TC03001489.hg.1	2.03	JUC03013209.hg.1	IL17RD
TC03001489.hg.1	-2.26	PSR03027074.hg.1	IL17RD
TC03001489.hg.1	-3.49	PSR03027078.hg.1	IL17RD
TC03001976.hg.1	3.02	JUC03017448.hg.1	PDCD10
TC03001976.hg.1	2.23	JUC03017452.hg.1	PDCD10
TC03001976.hg.1	-2.36	PSR03035350.hg.1	PDCD10
TC03001976.hg.1	-2.62	JUC03017455.hg.1	PDCD10
TC04001824.hg.1	3.02	JUC04013268.hg.1	FAT1
TC04001824.hg.1	2.52	JUC04013295.hg.1	FAT1
TC04001824.hg.1	2.23	PSR04025123.hg.1	FAT1
TC04001824.hg.1	2.13	PSR04025084.hg.1	FAT1
TC06000698.hg.1	3.02	JUC06003726.hg.1	PHF3
TC06000698.hg.1	2.83	PSR06008352.hg.1	PHF3
TC06000698.hg.1	2.04	PSR06008328.hg.1	PHF3
TC13000502.hg.1	3.02	PSR13005694.hg.1	RNF6
TC13000502.hg.1	-2.19	JUC13003410.hg.1	RNF6
TC19001703.hg.1	3.02	PSR19023237.hg.1	GYS1
TC19001703.hg.1	2.79	PSR19023246.hg.1	GYS1
TC19001703.hg.1	2.27	PSR19023227.hg.1	GYS1

TC19001703.hg.1	2.26	JUC19013270.hg.1	GYS1
TC19001703.hg.1	2.19	JUC19013284.hg.1	GYS1
TC19001703.hg.1	2.13	JUC19013277.hg.1	GYS1
TC21000135.hg.1	3.02	JUC21000622.hg.1	LINC00310
TC21000135.hg.1	2.79	JUC21000620.hg.1	LINC00310
TC21000135.hg.1	2.7	JUC21000621.hg.1	LINC00310
TC21000135.hg.1	2.09	PSR21001160.hg.1	LINC00310
TC21000135.hg.1	-2.71	JUC21000619.hg.1	LINC00310
TC01001144.hg.1	3.01	JUC01009778.hg.1	NBPF16, NBI
TC01001144.hg.1	2.06	PSR01017777.hg.1	NBPF16, NBI
TC01001144.hg.1	-2.09	JUC01009773.hg.1	NBPF16, NBI
TC04001459.hg.1	3.01	JUC04010797.hg.1	LEF1
TC04001459.hg.1	-2.19	PSR04020637.hg.1	LEF1
TC04001459.hg.1	-2.27	PSR04020643.hg.1	LEF1
TC04001459.hg.1	-2.36	PSR04020619.hg.1	LEF1
TC04001459.hg.1	-2.58	PSR04020600.hg.1	LEF1
TC04001459.hg.1	-2.65	PSR04020638.hg.1	LEF1
TC04001459.hg.1	-2.82	JUC04010814.hg.1	LEF1
TC04001459.hg.1	-2.86	PSR04020616.hg.1	LEF1
TC04001459.hg.1	-3.01	PSR04020614.hg.1	LEF1
TC04001459.hg.1	-3.1	JUC04010790.hg.1	LEF1
TC04001459.hg.1	-3.24	PSR04020642.hg.1	LEF1
TC04001459.hg.1	-3.39	PSR04020653.hg.1	LEF1
TC04001459.hg.1	-3.46	PSR04020625.hg.1	LEF1
TC04001459.hg.1	-3.7	JUC04010801.hg.1	LEF1
TC04001459.hg.1	-3.92	PSR04020615.hg.1	LEF1
TC04001459.hg.1	-3.97	PSR04020630.hg.1	LEF1
TC04001459.hg.1	-4.06	PSR04020644.hg.1	LEF1
TC04001459.hg.1	-4.51	JUC04010798.hg.1	LEF1
TC04001459.hg.1	-5.57	PSR04020617.hg.1	LEF1
TC04001459.hg.1	-6.64	PSR04020610.hg.1	LEF1
TC04001459.hg.1	-6.67	JUC04010809.hg.1	LEF1
TC06000328.hg.1	3.01	PSR06002682.hg.1	HLA-A
TC06000328.hg.1	2.88	PSR06002653.hg.1	HLA-A
TC06000328.hg.1	2.62	PSR06002663.hg.1	HLA-A
TC06000328.hg.1	2.5	PSR06002651.hg.1	HLA-A
TC06000328.hg.1	2.33	PSR06002680.hg.1	HLA-A
TC06000328.hg.1	2.32	PSR06002655.hg.1	HLA-A
TC06000328.hg.1	2.2	PSR06002668.hg.1	HLA-A
TC06000328.hg.1	2.19	PSR06002677.hg.1	HLA-A
TC06000328.hg.1	2.12	JUC06001194.hg.1	HLA-A
TC06000328.hg.1	2.04	PSR06002654.hg.1	HLA-A
TC06000328.hg.1	-2.29	JUC06001191.hg.1	HLA-A
TC06000328.hg.1	-3.82	JUC06001187.hg.1	HLA-A
TC06000328.hg.1	-4.35	PSR06002679.hg.1	HLA-A
TC06001965.hg.1	3.01	JUC06011876.hg.1	USP45
TC06001965.hg.1	2.06	PSR06024260.hg.1	USP45
TC06001965.hg.1	-2.84	JUC06011899.hg.1	USP45
TC07001931.hg.1	3.01	PSR07030029.hg.1	FLJ40852
TC07001931.hg.1	2.83	PSR07030028.hg.1	FLJ40852
TC07001931.hg.1	-2.27	JUC07014890.hg.1	FLJ40852
TC07001931.hg.1	-3.39	PSR07030019.hg.1	FLJ40852
TC08000203.hg.1	3.01	JUC08001409.hg.1	DPYSL2
TC08000203.hg.1	2.99	PSR08002869.hg.1	DPYSL2
TC08000203.hg.1	2.98	PSR08002865.hg.1	DPYSL2
TC08000203.hg.1	2.92	PSR08002878.hg.1	DPYSL2
TC08000203.hg.1	2.8	PSR08002888.hg.1	DPYSL2
TC08000203.hg.1	2.71	PSR08002874.hg.1	DPYSL2

TC08000203.hg.1	2.56	JUC08001407.hg.1	DPYSL2
TC08000203.hg.1	2.26	PSR08002862.hg.1	DPYSL2
TC08000203.hg.1	2.21	PSR08002861.hg.1	DPYSL2
TC08000203.hg.1	2.2	JUC08001418.hg.1	DPYSL2
TC08000203.hg.1	2.16	PSR08002879.hg.1	DPYSL2
TC08000203.hg.1	-2.05	JUC08001410.hg.1	DPYSL2
TC08000203.hg.1	-2.3	JUC08001413.hg.1	DPYSL2
TC08001705.hg.1	3.01	JUC08011525.hg.1	LYNX1
TC08001705.hg.1	2.94	PSR08022420.hg.1	LYNX1
TC08001705.hg.1	2.91	PSR08022421.hg.1	LYNX1
TC08001705.hg.1	2.76	PSR08022439.hg.1	LYNX1
TC08001705.hg.1	2.34	JUC08011533.hg.1	LYNX1
TC08001705.hg.1	2.12	JUC08011529.hg.1	LYNX1
TC08001705.hg.1	2.09	PSR08022424.hg.1	LYNX1
TC08001705.hg.1	2.09	PSR08022435.hg.1	LYNX1
TC0X000809.hg.1	3.01	PSR0X011080.hg.1	PRKX
TC0X000809.hg.1	2.42	JUC0X005424.hg.1	PRKX
TC0X000809.hg.1	2.36	PSR0X011101.hg.1	PRKX
TC0X000809.hg.1	2.36	PSR0X011102.hg.1	PRKX
TC0X000809.hg.1	2.22	PSR0X011086.hg.1	PRKX
TC0X000809.hg.1	-2.04	JUC0X005428.hg.1	PRKX
TC12001791.hg.1	3.01	JUC12013087.hg.1	CEP290
TC12001791.hg.1	2.61	JUC12013097.hg.1	CEP290
TC12001791.hg.1	2.12	JUC12013130.hg.1	CEP290
TC12001791.hg.1	-2.01	PSR12023682.hg.1	CEP290
TC12001791.hg.1	-2.75	JUC12013122.hg.1	CEP290
TC12001791.hg.1	-2.79	JUC12013116.hg.1	CEP290
TC12001791.hg.1	-3.98	JUC12013086.hg.1	CEP290
TC15001469.hg.1	3.01	PSR15013760.hg.1	PRTG
TC15001469.hg.1	2.39	JUC15007381.hg.1	PRTG
TC15001469.hg.1	-2.14	JUC15007383.hg.1	PRTG
TC21000267.hg.1	3.01	JUC21001805.hg.1	TEKT4P2, LC
TC21000267.hg.1	2.52	JUC21001808.hg.1	TEKT4P2, LC
TC21000267.hg.1	2.04	JUC21001804.hg.1	TEKT4P2, LC
TC21000267.hg.1	-2.48	PSR21003353.hg.1	TEKT4P2, LC
TC6_mcf_hap5000	3.01	JUC6_mcf_hap5001	HLA-B
TC6_mcf_hap5000	2.32	JUC6_mcf_hap5001	HLA-B
TC6_mcf_hap5000	2.27	PSR6_mcf_hap5002	HLA-B
TC6_mcf_hap5000	2.26	JUC6_mcf_hap5001	HLA-B
TC6_mcf_hap5000	2.16	PSR6_mcf_hap5002	HLA-B
TC6_mcf_hap5000	2.07	PSR6_mcf_hap5002	HLA-B
TC6_ssto_hap700	3.01	PSR6_ssto_hap7003	PSMB8
TC6_ssto_hap700	2.38	JUC6_ssto_hap7001	PSMB8
TC6_ssto_hap700	-3.15	PSR6_ssto_hap7003	PSMB8
TC6_ssto_hap700	3.01	JUC6_ssto_hap7001	DDX39B, SN
TC6_ssto_hap700	2.78	PSR6_ssto_hap7002	DDX39B, SN
TC6_ssto_hap700	2.12	PSR6_ssto_hap7002	DDX39B, SN
TC6_ssto_hap700	-2.77	PSR6_ssto_hap7002	DDX39B, SN
TC6_ssto_hap700	-3.66	PSR6_ssto_hap7002	DDX39B, SN
TC01003883.hg.1	3	JUC01031010.hg.1	WDR26
TC01003883.hg.1	2.56	PSR01059192.hg.1	WDR26
TC01006323.hg.1	3	JUC01044045.hg.1	HEATR8
TC01006323.hg.1	-2.07	PSR01010821.hg.1	HEATR8
TC01006323.hg.1	-2.23	PSR01010818.hg.1	HEATR8
TC02001893.hg.1	3	JUC02015964.hg.1	XPO1
TC02001893.hg.1	2.96	PSR02030318.hg.1	XPO1
TC02001893.hg.1	2.63	PSR02030345.hg.1	XPO1
TC02001893.hg.1	2.48	PSR02030316.hg.1	XPO1

TC02001893.hg.1	2.39	PSR02030348.hg.1	XPO1
TC02001893.hg.1	2.16	PSR02030351.hg.1	XPO1
TC02001893.hg.1	2.06	PSR02030288.hg.1	XPO1
TC02001893.hg.1	2.01	PSR02030343.hg.1	XPO1
TC06001195.hg.1	3	JUC06007077.hg.1	C6orf70
TC06001195.hg.1	-2.03	PSR06014278.hg.1	C6orf70
TC06001195.hg.1	-2.89	JUC06007089.hg.1	C6orf70
TC06001195.hg.1	-2.99	PSR06014270.hg.1	C6orf70
TC07000520.hg.1	3	JUC07003503.hg.1	DMTF1
TC07000520.hg.1	-2.54	PSR07007337.hg.1	DMTF1
TC07000520.hg.1	-2.94	PSR07007338.hg.1	DMTF1
TC07000520.hg.1	-3.74	PSR07007343.hg.1	DMTF1
TC07001955.hg.1	3	JUC07014953.hg.1	TRBV10-1
TC07001955.hg.1	2.23	PSR07030190.hg.1	TRBV10-1
TC08000019.hg.1	3	JUC08000170.hg.1	
TC08000019.hg.1	2.12	PSR08000305.hg.1	
TC0X000038.hg.1	3	JUC0X000309.hg.1	CLCN4
TC0X000038.hg.1	2.27	JUC0X000301.hg.1	CLCN4
TC0X000038.hg.1	2.02	PSR0X000540.hg.1	CLCN4
TC0X000038.hg.1	-2.13	JUC0X000302.hg.1	CLCN4
TC0X000038.hg.1	-3.87	JUC0X000296.hg.1	CLCN4
TC0X001544.hg.1	3	PSR0X020691.hg.1	MPP1
TC0X001544.hg.1	2.78	JUC0X010326.hg.1	MPP1
TC0X001544.hg.1	2.39	JUC0X010322.hg.1	MPP1
TC0X001544.hg.1	2.2	PSR0X020686.hg.1	MPP1
TC0X001544.hg.1	-2.79	JUC0X010337.hg.1	MPP1
TC12003260.hg.1	3	JUC12020197.hg.1	WDR66
TC12003260.hg.1	2.85	PSR12012606.hg.1	WDR66
TC12003260.hg.1	2.6	JUC12020179.hg.1	WDR66
TC12003260.hg.1	2.12	JUC12020188.hg.1	WDR66
TC13000484.hg.1	3	JUC13003295.hg.1	MIPEP
TC13000484.hg.1	2.44	JUC13003277.hg.1	MIPEP
TC13000484.hg.1	-2.1	JUC13003286.hg.1	MIPEP
TC13000484.hg.1	-2.36	JUC13003296.hg.1	MIPEP
TC13000484.hg.1	-3.28	PSR13005460.hg.1	MIPEP
TC18000051.hg.1	3	JUC18000348.hg.1	APCDD1
TC18000051.hg.1	2.56	PSR18000560.hg.1	APCDD1
TC18000051.hg.1	2.08	PSR18000557.hg.1	APCDD1
TC18000051.hg.1	-2.68	PSR18000552.hg.1	APCDD1
TC18000051.hg.1	-3.5	PSR18000553.hg.1	APCDD1
TC01002493.hg.1	2.99	JUC01020793.hg.1	SFPQ
TC01002493.hg.1	2.9	JUC01020783.hg.1	SFPQ
TC01002493.hg.1	2.89	PSR01038690.hg.1	SFPQ
TC01002493.hg.1	2.81	PSR01038686.hg.1	SFPQ
TC01002493.hg.1	2.77	JUC01020789.hg.1	SFPQ
TC01002493.hg.1	2.71	JUC01020787.hg.1	SFPQ
TC01002493.hg.1	2.67	JUC01020781.hg.1	SFPQ
TC01002493.hg.1	2.56	PSR01038689.hg.1	SFPQ
TC01002493.hg.1	2.54	PSR01038682.hg.1	SFPQ
TC01002493.hg.1	2.49	PSR01038692.hg.1	SFPQ
TC01002493.hg.1	2.49	PSR01038694.hg.1	SFPQ
TC01002493.hg.1	2.46	PSR01038685.hg.1	SFPQ
TC01002493.hg.1	2.45	PSR01038684.hg.1	SFPQ
TC01002493.hg.1	2.34	PSR01038680.hg.1	SFPQ
TC01002493.hg.1	2.3	PSR01038683.hg.1	SFPQ
TC01002493.hg.1	2.22	JUC01020786.hg.1	SFPQ
TC01002493.hg.1	2.17	PSR01038687.hg.1	SFPQ
TC01002493.hg.1	2.16	PSR01038688.hg.1	SFPQ

TC01002493.hg.1	-2.01	JUC01020790.hg.1	SFPQ
TC01002493.hg.1	-2.64	JUC01020779.hg.1	SFPQ
TC02001320.hg.1	2.99	PSR02020512.hg.1	ANKZF1
TC02001320.hg.1	2.71	JUC02010955.hg.1	ANKZF1
TC02001320.hg.1	2.16	JUC02010941.hg.1	ANKZF1
TC02001320.hg.1	-2.15	PSR02020466.hg.1	ANKZF1
TC02001320.hg.1	-2.63	PSR02020465.hg.1	ANKZF1
TC02001320.hg.1	-2.68	PSR02020464.hg.1	ANKZF1
TC02001753.hg.1	2.99	JUC02014748.hg.1	ATL2
TC02001753.hg.1	2.51	JUC02014761.hg.1	ATL2
TC02001753.hg.1	2.37	JUC02014769.hg.1	ATL2
TC02001753.hg.1	2.28	JUC02014750.hg.1	ATL2
TC02001753.hg.1	2.19	PSR02028197.hg.1	ATL2
TC02001753.hg.1	2.01	JUC02014755.hg.1	ATL2
TC02002195.hg.1	2.99	JUC02018360.hg.1	NPHP1
TC02002195.hg.1	2.55	JUC02018355.hg.1	NPHP1
TC02002195.hg.1	2.05	JUC02018368.hg.1	NPHP1
TC02002195.hg.1	-2.37	JUC02018362.hg.1	NPHP1
TC02002195.hg.1	-2.76	JUC02018345.hg.1	NPHP1
TC02002195.hg.1	-2.96	PSR02035552.hg.1	NPHP1
TC02002195.hg.1	-3.62	JUC02018348.hg.1	NPHP1
TC03001010.hg.1	2.99	JUC03009101.hg.1	MAP3K13
TC03001010.hg.1	2.63	JUC03009091.hg.1	MAP3K13
TC03001010.hg.1	2.24	JUC03009106.hg.1	MAP3K13
TC03001010.hg.1	2.08	PSR03017916.hg.1	MAP3K13
TC03001010.hg.1	2.04	PSR03017913.hg.1	MAP3K13
TC03001010.hg.1	2.01	JUC03009108.hg.1	MAP3K13
TC05001558.hg.1	2.99	PSR05021273.hg.1	EDIL3
TC05001558.hg.1	-4.77	JUC05010963.hg.1	EDIL3
TC06001094.hg.1	2.99	PSR06012912.hg.1	PLEKHG1
TC06001094.hg.1	2.13	JUC06006349.hg.1	PLEKHG1
TC06001094.hg.1	2.02	JUC06006365.hg.1	PLEKHG1
TC06001094.hg.1	-2.03	JUC06006345.hg.1	PLEKHG1
TC06001094.hg.1	-2.26	PSR06012928.hg.1	PLEKHG1
TC06001094.hg.1	-2.69	JUC06006356.hg.1	PLEKHG1
TC06001094.hg.1	-3.3	PSR06012924.hg.1	PLEKHG1
TC07000539.hg.1	2.99	JUC07003695.hg.1	C7orf63
TC07000539.hg.1	2.65	JUC07003696.hg.1	C7orf63
TC07000539.hg.1	2.21	PSR07007687.hg.1	C7orf63
TC07000539.hg.1	2.15	JUC07003689.hg.1	C7orf63
TC07000539.hg.1	2.13	JUC07003685.hg.1	C7orf63
TC07000539.hg.1	-2.08	JUC07003679.hg.1	C7orf63
TC07000539.hg.1	-2.18	PSR07007673.hg.1	C7orf63
TC09001488.hg.1	2.99	JUC09010047.hg.1	PTBP3, MIR3
TC09001488.hg.1	2.73	PSR09018496.hg.1	PTBP3, MIR3
TC15001620.hg.1	2.99	JUC15008484.hg.1	MYO9A
TC15001620.hg.1	2.61	JUC15008518.hg.1	MYO9A
TC15001620.hg.1	2.37	JUC15008512.hg.1	MYO9A
TC15001620.hg.1	2.25	JUC15008516.hg.1	MYO9A
TC15001620.hg.1	-2.03	PSR15015634.hg.1	MYO9A
TC15001620.hg.1	-2.05	JUC15008489.hg.1	MYO9A
TC17000531.hg.1	2.99	JUC17003609.hg.1	TUBG1
TC17000531.hg.1	2.23	PSR17006433.hg.1	TUBG1
TC17002892.hg.1	2.99	JUC17018891.hg.1	CCDC144B, (
TC17002892.hg.1	2.72	PSR17016545.hg.1	CCDC144B, (
TC17002892.hg.1	2.7	PSR17016547.hg.1	CCDC144B, (
TC17002892.hg.1	2.42	JUC17018896.hg.1	CCDC144B, (
TC17002892.hg.1	2.25	JUC17018897.hg.1	CCDC144B, (

TC17002892.hg.1	2.15	PSR17016546.hg.1	CCDC144B, (
TC17002892.hg.1	2.02	JUC17018885.hg.1	CCDC144B, (
TC17002892.hg.1	-2.57	JUC17018880.hg.1	CCDC144B, (
TC22000598.hg.1	2.99	PSR22010521.hg.1	LRP5L
TC22000598.hg.1	2.75	PSR22010527.hg.1	LRP5L
TC22000598.hg.1	2.66	PSR22010529.hg.1	LRP5L
TC22000598.hg.1	2.63	PSR22010526.hg.1	LRP5L
TC22000598.hg.1	2.41	JUC22004262.hg.1	LRP5L
TC22000598.hg.1	2.38	PSR22010540.hg.1	LRP5L
TC22000598.hg.1	2.2	JUC22004257.hg.1	LRP5L
TC22000598.hg.1	2.08	PSR22010542.hg.1	LRP5L
TC22000598.hg.1	-2.25	JUC22004258.hg.1	LRP5L
TC6_cox_hap2000	2.99	JUC6_cox_hap2001(	BAG6
TC6_cox_hap2000	2.51	PSR6_cox_hap2002{	BAG6
TC6_cox_hap2000	2.32	PSR6_cox_hap2002{	BAG6
TC6_cox_hap2000	2.32	JUC6_cox_hap2001(	BAG6
TC6_cox_hap2000	2.31	JUC6_cox_hap2001(	BAG6
TC6_cox_hap2000	2.26	PSR6_cox_hap2002{	BAG6
TC6_cox_hap2000	2.23	JUC6_cox_hap2001(	BAG6
TC6_cox_hap2000	2.11	PSR6_cox_hap2002{	BAG6
TC6_cox_hap2000	2.1	PSR6_cox_hap2002{	BAG6
TC6_cox_hap2000	2.02	JUC6_cox_hap2001(	BAG6
TC6_cox_hap2000	2.01	PSR6_cox_hap2002{	BAG6
TC6_dbb_hap3000	2.99	JUC6_dbb_hap3001(	BAG6
TC6_dbb_hap3000	2.51	PSR6_dbb_hap3002	BAG6
TC6_dbb_hap3000	2.32	PSR6_dbb_hap3002	BAG6
TC6_dbb_hap3000	2.32	JUC6_dbb_hap3001(	BAG6
TC6_dbb_hap3000	2.31	JUC6_dbb_hap3001(	BAG6
TC6_dbb_hap3000	2.26	PSR6_dbb_hap3002	BAG6
TC6_dbb_hap3000	2.23	JUC6_dbb_hap3001(	BAG6
TC6_dbb_hap3000	2.1	PSR6_dbb_hap3002	BAG6
TC6_dbb_hap3000	2.02	JUC6_dbb_hap3001(	BAG6
TC6_dbb_hap3000	2.01	PSR6_dbb_hap3002	BAG6
TC6_mann_hap40(	2.99	JUC6_mann_hap400	BAG6
TC6_mann_hap40(	2.51	PSR6_mann_hap40(	BAG6
TC6_mann_hap40(	2.32	PSR6_mann_hap40(	BAG6
TC6_mann_hap40(	2.32	JUC6_mann_hap400	BAG6
TC6_mann_hap40(	2.31	JUC6_mann_hap400	BAG6
TC6_mann_hap40(	2.26	PSR6_mann_hap40(	BAG6
TC6_mann_hap40(	2.23	JUC6_mann_hap400	BAG6
TC6_mann_hap40(	2.1	PSR6_mann_hap40(	BAG6
TC6_mann_hap40(	2.02	JUC6_mann_hap400	BAG6
TC6_mann_hap40(	2.01	PSR6_mann_hap40(	BAG6
TC6_ssto_hap700(	2.99	JUC6_ssto_hap7000	BAG6
TC6_ssto_hap700(	2.51	PSR6_ssto_hap7002	BAG6
TC6_ssto_hap700(	2.32	PSR6_ssto_hap7002	BAG6
TC6_ssto_hap700(	2.32	JUC6_ssto_hap7000	BAG6
TC6_ssto_hap700(	2.31	JUC6_ssto_hap7000	BAG6
TC6_ssto_hap700(	2.26	PSR6_ssto_hap7002	BAG6
TC6_ssto_hap700(	2.23	JUC6_ssto_hap7000	BAG6
TC6_ssto_hap700(	2.11	PSR6_ssto_hap7002	BAG6
TC6_ssto_hap700(	2.1	PSR6_ssto_hap7002	BAG6
TC6_ssto_hap700(	2.02	JUC6_ssto_hap7000	BAG6
TC6_ssto_hap700(	2.01	PSR6_ssto_hap7002	BAG6
TC01001522.hg.1	2.98	JUC01012434.hg.1	RABGAP1L
TC01001522.hg.1	2.86	PSR01023551.hg.1	RABGAP1L
TC01001522.hg.1	2.33	JUC01012417.hg.1	RABGAP1L
TC01001522.hg.1	2.31	PSR01023522.hg.1	RABGAP1L

TC01001522.hg.1	-2.17	JUC01012453.hg.1	RABGAP1L
TC01001522.hg.1	-2.3	PSR01023540.hg.1	RABGAP1L
TC01006356.hg.1	2.98	PSR01041030.hg.1	KIAA0494
TC01006356.hg.1	2.18	JUC01044437.hg.1	KIAA0494
TC02005029.hg.1	2.98	JUC02032467.hg.1	PCBP1-AS1
TC02005029.hg.1	2.26	JUC02032455.hg.1	PCBP1-AS1
TC02005029.hg.1	2.17	JUC02032432.hg.1	PCBP1-AS1
TC02005029.hg.1	2.13	PSR02031058.hg.1	PCBP1-AS1
TC02005029.hg.1	2.04	JUC02032435.hg.1	PCBP1-AS1
TC02005029.hg.1	-2.01	JUC02032447.hg.1	PCBP1-AS1
TC02005029.hg.1	-2.04	JUC02032452.hg.1	PCBP1-AS1
TC02005029.hg.1	-2.17	PSR02031027.hg.1	PCBP1-AS1
TC02005029.hg.1	-2.58	JUC02032425.hg.1	PCBP1-AS1
TC02005029.hg.1	-3.44	JUC02032461.hg.1	PCBP1-AS1
TC03000162.hg.1	2.98	JUC03001538.hg.1	CNOT10
TC03000162.hg.1	2.67	JUC03001525.hg.1	CNOT10
TC03000162.hg.1	-2.18	PSR03002869.hg.1	CNOT10
TC03000162.hg.1	-2.37	PSR03002897.hg.1	CNOT10
TC05000707.hg.1	2.98	JUC05005305.hg.1	MATR3, SNH
TC05000707.hg.1	2.93	JUC05005336.hg.1	MATR3, SNH
TC05000707.hg.1	2.92	JUC05005334.hg.1	MATR3, SNH
TC05000707.hg.1	2.74	JUC05005349.hg.1	MATR3, SNH
TC05000707.hg.1	2.5	PSR05009994.hg.1	MATR3, SNH
TC05000707.hg.1	2.25	JUC05005331.hg.1	MATR3, SNH
TC05000707.hg.1	-2.01	PSR05010044.hg.1	MATR3, SNH
TC06001217.hg.1	2.98	PSR06014495.hg.1	SERPINB1
TC06001217.hg.1	2.11	PSR06014489.hg.1	SERPINB1
TC06001217.hg.1	-3.22	JUC06007206.hg.1	SERPINB1
TC06004114.hg.1	2.98	JUC06021555.hg.1	DDX39B, SN
TC06004114.hg.1	2.76	PSR06017458.hg.1	DDX39B, SN
TC06004114.hg.1	2.45	JUC06021570.hg.1	DDX39B, SN
TC06004114.hg.1	2.11	PSR06017449.hg.1	DDX39B, SN
TC06004114.hg.1	2.06	PSR06017473.hg.1	DDX39B, SN
TC06004114.hg.1	-2.03	PSR06017471.hg.1	DDX39B, SN
TC06004114.hg.1	-3.69	PSR06017436.hg.1	DDX39B, SN
TC06004114.hg.1	-4.07	PSR06017437.hg.1	DDX39B, SN
TC07001877.hg.1	2.98	JUC07014370.hg.1	PLXNA4
TC07001877.hg.1	2.34	JUC07014364.hg.1	PLXNA4
TC07001877.hg.1	2.3	PSR07029068.hg.1	PLXNA4
TC07001877.hg.1	-2.39	JUC07014359.hg.1	PLXNA4
TC09001258.hg.1	2.98	JUC09008502.hg.1	TLE1
TC09001258.hg.1	2.1	PSR09015824.hg.1	TLE1
TC09001258.hg.1	2.02	JUC09008491.hg.1	TLE1
TC09001258.hg.1	-2.97	JUC09008503.hg.1	TLE1
TC0X000826.hg.1	2.98	JUC0X005503.hg.1	HDHD1
TC0X000826.hg.1	2.9	PSR0X011236.hg.1	HDHD1
TC0X000826.hg.1	2.49	JUC0X005500.hg.1	HDHD1
TC0X000826.hg.1	2.38	PSR0X011245.hg.1	HDHD1
TC0X000826.hg.1	2.11	JUC0X005502.hg.1	HDHD1
TC0X000826.hg.1	-2.27	JUC0X005507.hg.1	HDHD1
TC0X000826.hg.1	-5.07	PSR0X011227.hg.1	HDHD1
TC0X001067.hg.1	2.98	JUC0X007327.hg.1	HUWE1
TC0X001067.hg.1	2.45	JUC0X007322.hg.1	HUWE1
TC0X001067.hg.1	2.03	JUC0X007257.hg.1	HUWE1
TC0X001067.hg.1	2.01	PSR0X014423.hg.1	HUWE1
TC0X001067.hg.1	-2.01	JUC0X007326.hg.1	HUWE1
TC0X001067.hg.1	-2.11	PSR0X014469.hg.1	HUWE1
TC12001829.hg.1	2.98	JUC12013384.hg.1	FGD6

TC12001829.hg.1	2.76	JUC12013389.hg.1	FGD6
TC12001829.hg.1	2.68	PSR12024106.hg.1	FGD6
TC12001829.hg.1	2.23	JUC12013379.hg.1	FGD6
TC15002776.hg.1	2.98	JUC15013452.hg.1	PKM
TC15002776.hg.1	2.78	PSR15015695.hg.1	PKM
TC15002776.hg.1	2.65	PSR15015694.hg.1	PKM
TC15002776.hg.1	2.42	JUC15013464.hg.1	PKM
TC15002776.hg.1	2.31	JUC15013449.hg.1	PKM
TC15002776.hg.1	2.21	JUC15013461.hg.1	PKM
TC15002776.hg.1	2.16	JUC15013457.hg.1	PKM
TC15002776.hg.1	2.07	JUC15013441.hg.1	PKM
TC15002776.hg.1	2.04	PSR15015688.hg.1	PKM
TC15002776.hg.1	2.02	PSR15015693.hg.1	PKM
TC17000872.hg.1	2.98	JUC17006321.hg.1	SPHK1
TC17000872.hg.1	-2.07	PSR17011324.hg.1	SPHK1
TC01001385.hg.1	2.97	JUC01011621.hg.1	SDHC
TC01001385.hg.1	2.26	PSR01021915.hg.1	SDHC
TC01001385.hg.1	2.11	JUC01011618.hg.1	SDHC
TC01001385.hg.1	-2.35	JUC01011614.hg.1	SDHC
TC01001385.hg.1	-2.52	JUC01011612.hg.1	SDHC
TC01001559.hg.1	2.97	PSR01024116.hg.1	QSOX1, FLJ2
TC01001559.hg.1	2.96	JUC01012791.hg.1	QSOX1, FLJ2
TC01001559.hg.1	-3.77	JUC01012795.hg.1	QSOX1, FLJ2
TC02005001.hg.1	2.97	JUC02031987.hg.1	BBS5
TC02005001.hg.1	2.3	JUC02031976.hg.1	BBS5
TC02005001.hg.1	2.25	PSR02014653.hg.1	BBS5
TC02005001.hg.1	2.25	JUC02031985.hg.1	BBS5
TC02005001.hg.1	2.01	PSR02014677.hg.1	BBS5
TC02005001.hg.1	-2.59	JUC02031986.hg.1	BBS5
TC02005010.hg.1	2.97	PSR02021537.hg.1	SP140L
TC02005010.hg.1	2.67	JUC02032106.hg.1	SP140L
TC02005010.hg.1	2.05	PSR02021539.hg.1	SP140L
TC02005010.hg.1	-2.33	JUC02032097.hg.1	SP140L
TC03000046.hg.1	2.97	PSR03001038.hg.1	IL17RE
TC03000046.hg.1	2.03	PSR03001045.hg.1	IL17RE
TC03000046.hg.1	-2	JUC03000513.hg.1	IL17RE
TC03000046.hg.1	-2.77	JUC03000512.hg.1	IL17RE
TC03000046.hg.1	-2.81	PSR03001031.hg.1	IL17RE
TC03000046.hg.1	-2.96	PSR03001029.hg.1	IL17RE
TC03001527.hg.1	2.97	JUC03013618.hg.1	ADAMTS9
TC03001527.hg.1	2.8	JUC03013616.hg.1	ADAMTS9
TC03001527.hg.1	2.25	JUC03013622.hg.1	ADAMTS9
TC03001527.hg.1	-2	JUC03013609.hg.1	ADAMTS9
TC03001527.hg.1	-2.09	JUC03013628.hg.1	ADAMTS9
TC03001527.hg.1	-2.13	PSR03027806.hg.1	ADAMTS9
TC03001527.hg.1	-2.16	PSR03027759.hg.1	ADAMTS9
TC03001527.hg.1	-2.23	PSR03027788.hg.1	ADAMTS9
TC03001527.hg.1	-2.28	JUC03013603.hg.1	ADAMTS9
TC03001527.hg.1	-2.31	JUC03013624.hg.1	ADAMTS9
TC03001527.hg.1	-2.53	JUC03013606.hg.1	ADAMTS9
TC03001527.hg.1	-2.74	JUC03013620.hg.1	ADAMTS9
TC03001527.hg.1	-2.79	PSR03027767.hg.1	ADAMTS9
TC03001527.hg.1	-2.86	JUC03013636.hg.1	ADAMTS9
TC03001527.hg.1	-2.87	JUC03013615.hg.1	ADAMTS9
TC03001527.hg.1	-2.99	PSR03027777.hg.1	ADAMTS9
TC03001527.hg.1	-3.06	JUC03013638.hg.1	ADAMTS9
TC03001527.hg.1	-3.1	PSR03027809.hg.1	ADAMTS9
TC03001527.hg.1	-3.52	PSR03027800.hg.1	ADAMTS9

TC03001527.hg.1	-3.56	PSR03027801.hg.1	ADAMTS9
TC03001527.hg.1	-5.6	PSR03027765.hg.1	ADAMTS9
TC03001527.hg.1	-6.77	PSR03027754.hg.1	ADAMTS9
TC04000548.hg.1	2.97	JUC04004206.hg.1	NPNT
TC04000548.hg.1	2.11	PSR04008005.hg.1	NPNT
TC04000548.hg.1	-2.16	JUC04004217.hg.1	NPNT
TC04000548.hg.1	-2.29	PSR04008015.hg.1	NPNT
TC04000548.hg.1	-2.54	PSR04008023.hg.1	NPNT
TC04000548.hg.1	-3.26	JUC04004216.hg.1	NPNT
TC05001573.hg.1	2.97	PSR05021340.hg.1	TMEM161B
TC05001573.hg.1	2.94	JUC05011001.hg.1	TMEM161B
TC05001573.hg.1	2.83	JUC05011003.hg.1	TMEM161B
TC05001573.hg.1	2.15	JUC05010998.hg.1	TMEM161B
TC05001573.hg.1	-2.22	JUC05011000.hg.1	TMEM161B
TC05001936.hg.1	2.97	PSR05026835.hg.1	CD74
TC05001936.hg.1	2.7	PSR05026841.hg.1	CD74
TC05001936.hg.1	2.62	PSR05026828.hg.1	CD74
TC05001936.hg.1	2.43	PSR05026831.hg.1	CD74
TC05001936.hg.1	2.41	PSR05026840.hg.1	CD74
TC05001936.hg.1	2.34	JUC05013666.hg.1	CD74
TC05001936.hg.1	2.19	PSR05026849.hg.1	CD74
TC05001936.hg.1	2.08	JUC05013658.hg.1	CD74
TC05001936.hg.1	-2.73	PSR05026834.hg.1	CD74
TC05001936.hg.1	-3.69	PSR05026838.hg.1	CD74
TC05001936.hg.1	-4.08	JUC05013669.hg.1	CD74
TC06001941.hg.1	2.97	JUC06011575.hg.1	RRAGD
TC06001941.hg.1	2.42	PSR06023802.hg.1	RRAGD
TC06001941.hg.1	2.24	PSR06023799.hg.1	RRAGD
TC06001941.hg.1	-2.2	JUC06011583.hg.1	RRAGD
TC07001898.hg.1	2.97	JUC07014583.hg.1	CREB3L2
TC07001898.hg.1	2.07	JUC07014588.hg.1	CREB3L2
TC07001898.hg.1	-2.08	JUC07014579.hg.1	CREB3L2
TC07001898.hg.1	-2.16	PSR07029499.hg.1	CREB3L2
TC07001898.hg.1	-2.27	JUC07014590.hg.1	CREB3L2
TC07001898.hg.1	-4.23	PSR07029504.hg.1	CREB3L2
TC08001225.hg.1	2.97	PSR08015885.hg.1	TMEM68
TC08001225.hg.1	2.52	PSR08015894.hg.1	TMEM68
TC08001225.hg.1	2.15	PSR08015888.hg.1	TMEM68
TC08001225.hg.1	2.1	PSR08015893.hg.1	TMEM68
TC08001225.hg.1	-2.03	JUC08008195.hg.1	TMEM68
TC08001225.hg.1	-2.15	JUC08008182.hg.1	TMEM68
TC09000746.hg.1	2.97	JUC09005244.hg.1	NUP214
TC09000746.hg.1	2.26	JUC09005206.hg.1	NUP214
TC09000746.hg.1	2.22	JUC09005210.hg.1	NUP214
TC09000746.hg.1	2.17	PSR09009555.hg.1	NUP214
TC09000746.hg.1	2.1	JUC09005220.hg.1	NUP214
TC09000746.hg.1	2.04	JUC09005202.hg.1	NUP214
TC09000746.hg.1	-2.25	JUC09005251.hg.1	NUP214
TC12000803.hg.1	2.97	PSR12010192.hg.1	HSP90B1, MI
TC12000803.hg.1	2.1	JUC12005577.hg.1	HSP90B1, MI
TC12000803.hg.1	2.06	JUC12005586.hg.1	HSP90B1, MI
TC12000803.hg.1	-2.9	JUC12005588.hg.1	HSP90B1, MI
TC14000316.hg.1	2.97	JUC14001605.hg.1	CDKN3
TC14000316.hg.1	2.59	JUC14001614.hg.1	CDKN3
TC14000316.hg.1	-2.12	PSR14003345.hg.1	CDKN3
TC14000316.hg.1	-2.61	JUC14001619.hg.1	CDKN3
TC17001610.hg.1	2.97	JUC17012159.hg.1	KANSL1
TC17001610.hg.1	-2.04	JUC17012153.hg.1	KANSL1

TC17001610.hg.1	-2.34	PSR17021638.hg.1	KANSL1
TC17001610.hg.1	-3.26	JUC17012157.hg.1	KANSL1
TC21000441.hg.1	2.97	JUC21002656.hg.1	DSCR3
TC21000441.hg.1	2.67	JUC21002653.hg.1	DSCR3
TC21000441.hg.1	-2.01	PSR21005194.hg.1	DSCR3
TC21000441.hg.1	-2.9	PSR21005193.hg.1	DSCR3
TC02002938.hg.1	2.96	JUC02024610.hg.1	ANKMY1
TC02002938.hg.1	2.49	JUC02024626.hg.1	ANKMY1
TC02002938.hg.1	2.18	JUC02024618.hg.1	ANKMY1
TC02002938.hg.1	2.12	PSR02047064.hg.1	ANKMY1
TC02002938.hg.1	2.09	JUC02024617.hg.1	ANKMY1
TC02002938.hg.1	2.02	PSR02047055.hg.1	ANKMY1
TC02002938.hg.1	2	PSR02047054.hg.1	ANKMY1
TC02002938.hg.1	-2.3	JUC02024609.hg.1	ANKMY1
TC02002938.hg.1	-2.88	JUC02024627.hg.1	ANKMY1
TC04001319.hg.1	2.96	JUC04009603.hg.1	CCNI
TC04001319.hg.1	2.46	PSR04018055.hg.1	CCNI
TC04001319.hg.1	2.18	PSR04018051.hg.1	CCNI
TC08000135.hg.1	2.96	PSR08001312.hg.1	PCM1
TC08000135.hg.1	2.44	PSR08001306.hg.1	PCM1
TC08000135.hg.1	2.15	PSR08001346.hg.1	PCM1
TC08000135.hg.1	2.07	PSR08001347.hg.1	PCM1
TC08000135.hg.1	-3.44	JUC08000690.hg.1	PCM1
TC09000668.hg.1	2.96	PSR09007938.hg.1	STXBP1
TC09000668.hg.1	2.82	JUC09004421.hg.1	STXBP1
TC09000668.hg.1	2.37	JUC09004401.hg.1	STXBP1
TC09000668.hg.1	2.23	JUC09004418.hg.1	STXBP1
TC09000668.hg.1	2.06	PSR09007903.hg.1	STXBP1
TC16001325.hg.1	2.96	PSR16018308.hg.1	FOXF1-AS1
TC16001325.hg.1	2.37	PSR16018317.hg.1	FOXF1-AS1
TC16001325.hg.1	2.33	PSR16018312.hg.1	FOXF1-AS1
TC16001325.hg.1	2.13	JUC16010440.hg.1	FOXF1-AS1
TC16001325.hg.1	-2.45	JUC16010442.hg.1	FOXF1-AS1
TC16001325.hg.1	-2.53	PSR16018316.hg.1	FOXF1-AS1
TC16001325.hg.1	-2.64	PSR16018309.hg.1	FOXF1-AS1
TC16001325.hg.1	-2.72	JUC16010439.hg.1	FOXF1-AS1
TC17001201.hg.1	2.96	JUC17009404.hg.1	SHMT1
TC17001201.hg.1	2.69	JUC17009411.hg.1	SHMT1
TC17001201.hg.1	2.3	JUC17009412.hg.1	SHMT1
TC17001201.hg.1	2.01	PSR17016472.hg.1	SHMT1
TC17001201.hg.1	-2.89	PSR17016463.hg.1	SHMT1
TC19001469.hg.1	2.96	PSR19019969.hg.1	ZNF566
TC19001469.hg.1	2.28	JUC19011494.hg.1	ZNF566
TC19001469.hg.1	2.23	PSR19019949.hg.1	ZNF566
TC19001469.hg.1	-2.13	PSR19019954.hg.1	ZNF566
TC19001469.hg.1	-2.19	PSR19019944.hg.1	ZNF566
TC20001745.hg.1	2.96	PSR20008127.hg.1	ZNF343
TC20001745.hg.1	2.59	JUC20009954.hg.1	ZNF343
TC22000138.hg.1	2.96	PSR22002427.hg.1	MMP11
TC22000138.hg.1	2.29	PSR22002441.hg.1	MMP11
TC22000138.hg.1	2.07	JUC22000762.hg.1	MMP11
TC01000559.hg.1	2.95	JUC01004769.hg.1	DMAP1
TC01000559.hg.1	-2.13	PSR01009221.hg.1	DMAP1
TC01003565.hg.1	2.95	JUC01028847.hg.1	ANGPTL1
TC01003565.hg.1	-2.22	JUC01028843.hg.1	ANGPTL1
TC01003565.hg.1	-3.45	PSR01055243.hg.1	ANGPTL1
TC01003565.hg.1	-3.85	PSR01055242.hg.1	ANGPTL1
TC01003565.hg.1	-4.53	PSR01055240.hg.1	ANGPTL1

TC01003565.hg.1	-5.86	JUC01028840.hg.1	ANGPTL1
TC01003565.hg.1	-6.14	PSR01055227.hg.1	ANGPTL1
TC01003565.hg.1	-8.2	PSR01055241.hg.1	ANGPTL1
TC02005053.hg.1	2.95	JUC02033146.hg.1	NHEJ1
TC02005053.hg.1	-2.28	PSR02044843.hg.1	NHEJ1
TC07003291.hg.1	2.95	PSR07011127.hg.1	IFRD1
TC07003291.hg.1	2.71	JUC07020359.hg.1	IFRD1
TC07003291.hg.1	2.7	PSR07011094.hg.1	IFRD1
TC07003291.hg.1	2.65	PSR07011114.hg.1	IFRD1
TC07003291.hg.1	2.16	JUC07020341.hg.1	IFRD1
TC07003291.hg.1	2.13	PSR07011101.hg.1	IFRD1
TC07003291.hg.1	2.03	PSR07011095.hg.1	IFRD1
TC07003291.hg.1	2	PSR07011103.hg.1	IFRD1
TC07003291.hg.1	-2.03	JUC07020363.hg.1	IFRD1
TC13000534.hg.1	2.95	JUC13003603.hg.1	HMGB1
TC13000534.hg.1	2.82	PSR13006048.hg.1	HMGB1
TC13000534.hg.1	2.8	JUC13003611.hg.1	HMGB1
TC13000534.hg.1	-2.06	JUC13003616.hg.1	HMGB1
TC14001544.hg.1	2.95	JUC14008969.hg.1	PPP1R13B
TC14001544.hg.1	2.77	JUC14008970.hg.1	PPP1R13B
TC14001544.hg.1	2.19	PSR14017340.hg.1	PPP1R13B
TC14001544.hg.1	2.17	PSR14017352.hg.1	PPP1R13B
TC14001544.hg.1	-2.18	JUC14008967.hg.1	PPP1R13B
TC14001544.hg.1	-2.85	JUC14008979.hg.1	PPP1R13B
TC14001544.hg.1	-2.88	JUC14008972.hg.1	PPP1R13B
TC15001011.hg.1	2.95	JUC15004864.hg.1	HERC2P3
TC15001011.hg.1	-2.17	JUC15004865.hg.1	HERC2P3
TC15001011.hg.1	-2.18	PSR15009377.hg.1	HERC2P3
TC15001011.hg.1	-2.38	JUC15004852.hg.1	HERC2P3
TC15001011.hg.1	-2.5	JUC15004873.hg.1	HERC2P3
TC15001011.hg.1	-4.02	PSR15009379.hg.1	HERC2P3
TC15001544.hg.1	2.95	PSR15014631.hg.1	HERC1
TC15001544.hg.1	-2.02	JUC15007891.hg.1	HERC1
TC15001544.hg.1	-2.05	JUC15007882.hg.1	HERC1
TC15001544.hg.1	-2.07	JUC15007878.hg.1	HERC1
TC15001544.hg.1	-2.2	JUC15007862.hg.1	HERC1
TC15001544.hg.1	-2.24	PSR15014593.hg.1	HERC1
TC15001544.hg.1	-2.3	JUC15007859.hg.1	HERC1
TC15001544.hg.1	-2.58	JUC15007927.hg.1	HERC1
TC15001544.hg.1	-2.82	JUC15007918.hg.1	HERC1
TC15001544.hg.1	-3.17	JUC15007860.hg.1	HERC1
TC15001544.hg.1	-4.48	JUC15007903.hg.1	HERC1
TC16001052.hg.1	2.95	PSR16015029.hg.1	PYCARD
TC16001052.hg.1	2.59	PSR16015031.hg.1	PYCARD
TC16001052.hg.1	2.58	JUC16008383.hg.1	PYCARD
TC16001052.hg.1	2.21	PSR16015025.hg.1	PYCARD
TC16001052.hg.1	2.17	JUC16008384.hg.1	PYCARD
TC17001682.hg.1	2.95	PSR17022620.hg.1	COL1A1
TC17001682.hg.1	2.81	PSR17022662.hg.1	COL1A1
TC17001682.hg.1	2.3	PSR17022602.hg.1	COL1A1
TC17001682.hg.1	2.23	PSR17022605.hg.1	COL1A1
TC17001682.hg.1	2.16	PSR17022628.hg.1	COL1A1
TC17001682.hg.1	-2.02	PSR17022670.hg.1	COL1A1
TC17001682.hg.1	-2.04	PSR17022600.hg.1	COL1A1
TC17001682.hg.1	-2.05	PSR17022597.hg.1	COL1A1
TC17001682.hg.1	-2.06	PSR17022595.hg.1	COL1A1
TC17001682.hg.1	-2.1	PSR17022598.hg.1	COL1A1
TC17001682.hg.1	-2.11	PSR17022594.hg.1	COL1A1

TC17001682.hg.1	-2.11	JUC17012578.hg.1	COL1A1
TC17001682.hg.1	-2.18	JUC17012572.hg.1	COL1A1
TC17001682.hg.1	-2.19	PSR17022593.hg.1	COL1A1
TC17001682.hg.1	-2.45	JUC17012566.hg.1	COL1A1
TC17001682.hg.1	-2.57	PSR17022633.hg.1	COL1A1
TC19000368.hg.1	2.95	PSR19004919.hg.1	SLC25A42
TC19000368.hg.1	2.88	JUC19002897.hg.1	SLC25A42
TC19000368.hg.1	2.12	PSR19004912.hg.1	SLC25A42
TC19000368.hg.1	-2.29	JUC19002894.hg.1	SLC25A42
TC20000466.hg.1	2.95	JUC20003394.hg.1	GNAS
TC20000466.hg.1	2.1	JUC20003418.hg.1	GNAS
TC20000466.hg.1	-2.16	PSR20006354.hg.1	GNAS
TC6_dbb_hap3000	2.95	JUC6_dbb_hap3000	HLA-F-AS1
TC6_dbb_hap3000	2.41	PSR6_dbb_hap3002	HLA-F-AS1
TC6_dbb_hap3000	2.27	PSR6_dbb_hap3002	HLA-F-AS1
TC6_dbb_hap3000	2.26	JUC6_dbb_hap3000	HLA-F-AS1
TC6_dbb_hap3000	-2.55	JUC6_dbb_hap3000	HLA-F-AS1
TC6_mann_hap40	2.95	JUC6_mann_hap400	HLA-F-AS1
TC6_mann_hap40	2.41	PSR6_mann_hap400	HLA-F-AS1
TC6_mann_hap40	2.27	PSR6_mann_hap400	HLA-F-AS1
TC6_mann_hap40	2.26	JUC6_mann_hap400	HLA-F-AS1
TC6_mann_hap40	-2.55	JUC6_mann_hap400	HLA-F-AS1
TC6_mann_hap40	2.95	PSR6_mann_hap400	AGPAT1
TC6_mann_hap40	2.74	JUC6_mann_hap400	AGPAT1
TC6_mann_hap40	2.55	PSR6_mann_hap400	AGPAT1
TC6_mann_hap40	-3.15	JUC6_mann_hap400	AGPAT1
TC6_qbl_hap6000	2.95	JUC6_qbl_hap60007	HLA-F-AS1
TC6_qbl_hap6000	2.41	PSR6_qbl_hap60021	HLA-F-AS1
TC6_qbl_hap6000	2.27	PSR6_qbl_hap60021	HLA-F-AS1
TC6_qbl_hap6000	2.26	JUC6_qbl_hap60007	HLA-F-AS1
TC6_qbl_hap6000	-2.55	JUC6_qbl_hap60007	HLA-F-AS1
TC01001490.hg.1	2.94	JUC01012205.hg.1	FMO4
TC01001490.hg.1	-2.09	JUC01012206.hg.1	FMO4
TC01001490.hg.1	-2.69	JUC01012208.hg.1	FMO4
TC01001490.hg.1	-2.92	PSR01023140.hg.1	FMO4
TC02000263.hg.1	2.94	PSR02003952.hg.1	MTA3
TC02000263.hg.1	2.67	JUC02002028.hg.1	MTA3
TC02000263.hg.1	2.58	JUC02002039.hg.1	MTA3
TC02000263.hg.1	2.46	PSR02003944.hg.1	MTA3
TC02000263.hg.1	2.05	JUC02002018.hg.1	MTA3
TC02000263.hg.1	2.04	PSR02003946.hg.1	MTA3
TC06001687.hg.1	2.94	JUC06009569.hg.1	MDGA1
TC06001687.hg.1	2.31	PSR06020091.hg.1	MDGA1
TC06001687.hg.1	2.22	JUC06009573.hg.1	MDGA1
TC06001687.hg.1	2.12	PSR06020135.hg.1	MDGA1
TC06001687.hg.1	2.09	PSR06020118.hg.1	MDGA1
TC07001208.hg.1	2.94	JUC07009270.hg.1	C7orf31
TC07001208.hg.1	2.34	PSR07018929.hg.1	C7orf31
TC07001208.hg.1	2.02	PSR07018931.hg.1	C7orf31
TC08000178.hg.1	2.94	PSR08002317.hg.1	RHOBTB2
TC08000178.hg.1	2.94	JUC08001188.hg.1	RHOBTB2
TC08000178.hg.1	-2.07	JUC08001183.hg.1	RHOBTB2
TC12001723.hg.1	2.94	JUC12012639.hg.1	TSPAN8
TC12001723.hg.1	2.52	PSR12022908.hg.1	TSPAN8
TC12001723.hg.1	2.47	PSR12022906.hg.1	TSPAN8
TC12001723.hg.1	2.38	PSR12022918.hg.1	TSPAN8
TC12001723.hg.1	2.18	PSR12022913.hg.1	TSPAN8
TC12001723.hg.1	2.14	PSR12022907.hg.1	TSPAN8

TC16000226.hg.1	2.94	JUC16001830.hg.1	LYRM1
TC16000226.hg.1	2.82	PSR16003355.hg.1	LYRM1
TC16000226.hg.1	2.66	JUC16001824.hg.1	LYRM1
TC16000226.hg.1	2.2	PSR16003354.hg.1	LYRM1
TC16000226.hg.1	2.2	JUC16001831.hg.1	LYRM1
TC16000226.hg.1	2.09	PSR16003356.hg.1	LYRM1
TC19000630.hg.1	2.94	JUC19005221.hg.1	BCAM
TC19000630.hg.1	2.73	PSR19008741.hg.1	BCAM
TC19000630.hg.1	2.48	PSR19008740.hg.1	BCAM
TC19000630.hg.1	2.44	JUC19005218.hg.1	BCAM
TC19000630.hg.1	2.02	JUC19005215.hg.1	BCAM
TC19000630.hg.1	-2.29	PSR19008742.hg.1	BCAM
TC19000630.hg.1	-3.63	JUC19005216.hg.1	BCAM
TC19000630.hg.1	-6.05	JUC19005220.hg.1	BCAM
TC20000942.hg.1	2.94	PSR20013004.hg.1	NFATC2
TC20000942.hg.1	2	JUC20006743.hg.1	NFATC2
TC20000942.hg.1	-2.13	JUC20006745.hg.1	NFATC2
TC20000942.hg.1	-2.23	PSR20013006.hg.1	NFATC2
TC20000942.hg.1	-2.28	PSR20013008.hg.1	NFATC2
TC20000942.hg.1	-2.45	JUC20006739.hg.1	NFATC2
TC20000942.hg.1	-2.51	JUC20006740.hg.1	NFATC2
TC20000942.hg.1	-3.05	PSR20013007.hg.1	NFATC2
TC20000942.hg.1	-3.15	JUC20006741.hg.1	NFATC2
TC20000942.hg.1	-5.69	PSR20013010.hg.1	NFATC2
TC17000402.hg.1	2.94	JUC17002896.hg.1	TAF15
TC17000402.hg.1	2.17	JUC17002910.hg.1	TAF15
TC17000402.hg.1	2	PSR17005047.hg.1	TAF15
TC01001724.hg.1	2.93	JUC01014409.hg.1	RASSF5
TC01001724.hg.1	-2.44	PSR01026908.hg.1	RASSF5
TC01003759.hg.1	2.93	JUC01030123.hg.1	C1orf186, LO
TC01003759.hg.1	2.56	JUC01030138.hg.1	C1orf186, LO
TC01003759.hg.1	2.42	JUC01030126.hg.1	C1orf186, LO
TC01003759.hg.1	2.38	PSR01057672.hg.1	C1orf186, LO
TC01003759.hg.1	2.31	PSR01057663.hg.1	C1orf186, LO
TC01003759.hg.1	2.15	PSR01057679.hg.1	C1orf186, LO
TC01003759.hg.1	2.07	PSR01057664.hg.1	C1orf186, LO
TC02001475.hg.1	2.93	JUC02012410.hg.1	PPP1R7
TC02001475.hg.1	2.07	PSR02023828.hg.1	PPP1R7
TC02001475.hg.1	-2.13	PSR02023819.hg.1	PPP1R7
TC02002669.hg.1	2.93	JUC02022435.hg.1	ORC2
TC02002669.hg.1	2.14	PSR02042901.hg.1	ORC2
TC02002669.hg.1	2.13	JUC02022429.hg.1	ORC2
TC02002669.hg.1	2.02	PSR02042907.hg.1	ORC2
TC02002669.hg.1	-2.57	JUC02022439.hg.1	ORC2
TC02002669.hg.1	-2.73	JUC02022424.hg.1	ORC2
TC02002669.hg.1	-2.74	JUC02022428.hg.1	ORC2
TC02004917.hg.1	2.93	PSR02001856.hg.1	CENPA
TC02004917.hg.1	2.32	JUC02031168.hg.1	CENPA
TC02004917.hg.1	-2.35	PSR02001862.hg.1	CENPA
TC03001496.hg.1	2.93	JUC03013329.hg.1	FAM116A
TC03001496.hg.1	-2.11	PSR03027269.hg.1	FAM116A
TC03001575.hg.1	2.93	PSR03028581.hg.1	GBE1
TC03001575.hg.1	2.85	PSR03028585.hg.1	GBE1
TC03001575.hg.1	2.34	PSR03028583.hg.1	GBE1
TC03001575.hg.1	-2.31	PSR03028545.hg.1	GBE1
TC03001575.hg.1	-2.76	JUC03014094.hg.1	GBE1
TC05001271.hg.1	2.93	PSR05017813.hg.1	RANBP3L
TC05001271.hg.1	2.04	JUC05009125.hg.1	RANBP3L

TC05001271.hg.1	-2.2	JUC05009128.hg.1	RANBP3L
TC05001271.hg.1	-2.64	PSR05017805.hg.1	RANBP3L
TC05001271.hg.1	-2.67	PSR05017800.hg.1	RANBP3L
TC05001271.hg.1	-3.38	JUC05009127.hg.1	RANBP3L
TC05001271.hg.1	-3.68	PSR05017802.hg.1	RANBP3L
TC05001271.hg.1	-3.95	JUC05009121.hg.1	RANBP3L
TC05001271.hg.1	-4.64	JUC05009122.hg.1	RANBP3L
TC05001271.hg.1	-13.12	PSR05017806.hg.1	RANBP3L
TC05001271.hg.1	-138.65	JUC05009135.hg.1	RANBP3L
TC07001972.hg.1	2.93	PSR07030414.hg.1	LOC154761,
TC07001972.hg.1	2.92	JUC07015067.hg.1	LOC154761,
TC07001972.hg.1	2.62	PSR07030411.hg.1	LOC154761,
TC07001972.hg.1	2.29	PSR07030420.hg.1	LOC154761,
TC07001972.hg.1	2.14	PSR07030406.hg.1	LOC154761,
TC07001972.hg.1	-2.01	JUC07015075.hg.1	LOC154761,
TC07001972.hg.1	-2.06	JUC07015072.hg.1	LOC154761,
TC07001972.hg.1	-2.26	JUC07015073.hg.1	LOC154761,
TC07001972.hg.1	-3.07	PSR07030404.hg.1	LOC154761,
TC07001972.hg.1	-4.21	JUC07015061.hg.1	LOC154761,
TC09001054.hg.1	2.93	PSR09013968.hg.1	CD72
TC09001054.hg.1	2.67	PSR09013969.hg.1	CD72
TC09001054.hg.1	2.45	JUC09007529.hg.1	CD72
TC09001054.hg.1	2.26	JUC09007543.hg.1	CD72
TC09001054.hg.1	2.25	PSR09013972.hg.1	CD72
TC09001054.hg.1	2.22	PSR09013958.hg.1	CD72
TC09001054.hg.1	2.09	PSR09013974.hg.1	CD72
TC09001054.hg.1	-4.04	JUC09007537.hg.1	CD72
TC09001490.hg.1	2.93	JUC09010058.hg.1	SUSD1
TC09001490.hg.1	2.18	JUC09010059.hg.1	SUSD1
TC09001490.hg.1	-2.04	PSR09018521.hg.1	SUSD1
TC09001490.hg.1	-2.32	PSR09018520.hg.1	SUSD1
TC09001490.hg.1	-2.34	PSR09018513.hg.1	SUSD1
TC09001490.hg.1	-2.54	JUC09010066.hg.1	SUSD1
TC09001490.hg.1	-2.8	PSR09018515.hg.1	SUSD1
TC09001490.hg.1	-3.69	JUC09010070.hg.1	SUSD1
TC10000219.hg.1	2.93	PSR10002515.hg.1	ZEB1
TC10000219.hg.1	2.71	PSR10002523.hg.1	ZEB1
TC10000219.hg.1	-2.07	JUC10001356.hg.1	ZEB1
TC10000219.hg.1	-2.93	JUC10001367.hg.1	ZEB1
TC10000733.hg.1	2.93	JUC10004817.hg.1	TLX1
TC10000733.hg.1	-2.02	PSR10008664.hg.1	TLX1
TC10001446.hg.1	2.93	JUC10010695.hg.1	POLR3A
TC10001446.hg.1	2.54	JUC10010688.hg.1	POLR3A
TC10001446.hg.1	2.39	JUC10010722.hg.1	POLR3A
TC10001446.hg.1	2.07	JUC10010707.hg.1	POLR3A
TC10001446.hg.1	-2.05	PSR10018356.hg.1	POLR3A
TC10001446.hg.1	-2.06	JUC10010708.hg.1	POLR3A
TC10001446.hg.1	-2.12	JUC10010694.hg.1	POLR3A
TC10001446.hg.1	-2.28	JUC10010699.hg.1	POLR3A
TC10002969.hg.1	2.93	JUC10017585.hg.1	FAM53B
TC10002969.hg.1	2.12	JUC10017579.hg.1	FAM53B
TC10002969.hg.1	-2.03	PSR10022950.hg.1	FAM53B
TC15001575.hg.1	2.93	JUC15008225.hg.1	MEGF11
TC15001575.hg.1	2.42	JUC15008234.hg.1	MEGF11
TC15001575.hg.1	-2.29	PSR15015153.hg.1	MEGF11
TC15001575.hg.1	-2.79	JUC15008237.hg.1	MEGF11
TC01000586.hg.1	2.92	PSR01010021.hg.1	LOC729041
TC01000586.hg.1	2.36	JUC01005144.hg.1	LOC729041

TC01000586.hg.1	-4.52	JUC01005139.hg.1	LOC729041
TC02000430.hg.1	2.92	JUC02003337.hg.1	ZNF638
TC02000430.hg.1	2.49	JUC02003334.hg.1	ZNF638
TC02000430.hg.1	2.42	JUC02003333.hg.1	ZNF638
TC02000430.hg.1	2.36	JUC02003317.hg.1	ZNF638
TC02000430.hg.1	-2	PSR02006515.hg.1	ZNF638
TC02000430.hg.1	-2.01	JUC02003311.hg.1	ZNF638
TC02000430.hg.1	-2.13	JUC02003341.hg.1	ZNF638
TC02000430.hg.1	-2.2	JUC02003346.hg.1	ZNF638
TC02000430.hg.1	-3.37	PSR02006477.hg.1	ZNF638
TC02002127.hg.1	2.92	PSR02034602.hg.1	MGAT4A
TC02002127.hg.1	2.61	JUC02017846.hg.1	MGAT4A
TC02002127.hg.1	2.5	PSR02034567.hg.1	MGAT4A
TC02002127.hg.1	2.19	PSR02034592.hg.1	MGAT4A
TC02002127.hg.1	2.13	JUC02017854.hg.1	MGAT4A
TC02002127.hg.1	2.04	PSR02034603.hg.1	MGAT4A
TC04000011.hg.1	2.92	JUC04000073.hg.1	PDE6B
TC04000011.hg.1	-2.76	PSR04000197.hg.1	PDE6B
TC05000938.hg.1	2.92	PSR05013724.hg.1	RANBP17
TC05000938.hg.1	2.9	JUC05007089.hg.1	RANBP17
TC05000938.hg.1	2.43	JUC05007091.hg.1	RANBP17
TC05000938.hg.1	2.08	PSR05013718.hg.1	RANBP17
TC05000938.hg.1	-2.05	JUC05007111.hg.1	RANBP17
TC05000938.hg.1	-2.11	PSR05013695.hg.1	RANBP17
TC05000938.hg.1	-2.19	PSR05013727.hg.1	RANBP17
TC05000938.hg.1	-2.32	PSR05013671.hg.1	RANBP17
TC05000938.hg.1	-2.41	JUC05007134.hg.1	RANBP17
TC05000938.hg.1	-2.93	JUC05007087.hg.1	RANBP17
TC07000552.hg.1	2.92	PSR07008062.hg.1	CCDC132
TC07000552.hg.1	2.29	JUC07003855.hg.1	CCDC132
TC07000552.hg.1	2.05	PSR07008123.hg.1	CCDC132
TC07000552.hg.1	2.02	JUC07003850.hg.1	CCDC132
TC07000552.hg.1	-2.13	JUC07003840.hg.1	CCDC132
TC07002029.hg.1	2.92	PSR07030994.hg.1	FASTK
TC07002029.hg.1	-2.11	JUC07015380.hg.1	FASTK
TC11001247.hg.1	2.92	JUC11008509.hg.1	SLC25A22
TC11001247.hg.1	2.32	PSR11015767.hg.1	SLC25A22
TC11001247.hg.1	2	JUC11008516.hg.1	SLC25A22
TC13000867.hg.1	2.92	JUC13005687.hg.1	LIG4
TC13000867.hg.1	2.68	JUC13005691.hg.1	LIG4
TC13000867.hg.1	-2.05	PSR13009687.hg.1	LIG4
TC6_qbl_hap6000	2.92	JUC6_qbl_hap60010	HLA-B
TC6_qbl_hap6000	2.61	PSR6_qbl_hap60027	HLA-B
TC6_qbl_hap6000	2.25	PSR6_qbl_hap60027	HLA-B
TC6_qbl_hap6000	2.25	JUC6_qbl_hap60010	HLA-B
TC6_qbl_hap6000	2.09	PSR6_qbl_hap60027	HLA-B
TC6_qbl_hap6000	-2.34	PSR6_qbl_hap60027	HLA-B
TC02000348.hg.1	2.91	JUC02002738.hg.1	REL
TC02000348.hg.1	2.61	PSR02005179.hg.1	REL
TC02000348.hg.1	2.17	JUC02002728.hg.1	REL
TC02001377.hg.1	2.91	JUC02011460.hg.1	SP100
TC02001377.hg.1	2.37	PSR02021601.hg.1	SP100
TC02001377.hg.1	2.32	PSR02021559.hg.1	SP100
TC02001377.hg.1	2.12	PSR02021620.hg.1	SP100
TC02001377.hg.1	2.01	PSR02021615.hg.1	SP100
TC02001421.hg.1	2.91	JUC02011969.hg.1	HEATR7B1
TC02001421.hg.1	2.47	JUC02011997.hg.1	HEATR7B1
TC02001421.hg.1	2.19	PSR02022756.hg.1	HEATR7B1

TC03000212.hg.1	2.91	PSR03004248.hg.1	ZNF621
TC03000212.hg.1	2.37	JUC03002248.hg.1	ZNF621
TC03000212.hg.1	-2.24	PSR03004239.hg.1	ZNF621
TC03000263.hg.1	2.91	JUC03002697.hg.1	NBEAL2
TC03000263.hg.1	2.36	PSR03005338.hg.1	NBEAL2
TC03000263.hg.1	2.34	JUC03002679.hg.1	NBEAL2
TC03000263.hg.1	2.2	PSR03005392.hg.1	NBEAL2
TC03000263.hg.1	-2.41	JUC03002702.hg.1	NBEAL2
TC04001718.hg.1	2.91	JUC04012546.hg.1	DDX60
TC04001718.hg.1	2.32	JUC04012519.hg.1	DDX60
TC04001718.hg.1	2.13	JUC04012537.hg.1	DDX60
TC04001718.hg.1	-2.02	PSR04023712.hg.1	DDX60
TC04001718.hg.1	-2.02	JUC04012526.hg.1	DDX60
TC04001718.hg.1	-2.02	JUC04012533.hg.1	DDX60
TC04001718.hg.1	-2.09	PSR04023671.hg.1	DDX60
TC04001718.hg.1	-2.21	PSR04023705.hg.1	DDX60
TC04001718.hg.1	-2.22	JUC04012524.hg.1	DDX60
TC04001718.hg.1	-2.46	JUC04012538.hg.1	DDX60
TC04001718.hg.1	-3.22	JUC04012532.hg.1	DDX60
TC05000039.hg.1	2.91	JUC05000255.hg.1	ADAMTS16
TC05000039.hg.1	2.31	PSR05000461.hg.1	ADAMTS16
TC05000039.hg.1	2.2	JUC05000233.hg.1	ADAMTS16
TC05000039.hg.1	2.19	JUC05000246.hg.1	ADAMTS16
TC05000039.hg.1	2.08	JUC05000250.hg.1	ADAMTS16
TC05000039.hg.1	-2	PSR05000465.hg.1	ADAMTS16
TC05000039.hg.1	-2.16	PSR05000471.hg.1	ADAMTS16
TC05000039.hg.1	-2.34	JUC05000236.hg.1	ADAMTS16
TC05000866.hg.1	2.91	JUC05006547.hg.1	CYFIP2
TC05000866.hg.1	2.88	PSR05012690.hg.1	CYFIP2
TC05000866.hg.1	2.61	PSR05012679.hg.1	CYFIP2
TC05000866.hg.1	2.52	JUC05006570.hg.1	CYFIP2
TC05000866.hg.1	2.46	JUC05006545.hg.1	CYFIP2
TC05000866.hg.1	2.43	JUC05006568.hg.1	CYFIP2
TC05000866.hg.1	2.34	PSR05012688.hg.1	CYFIP2
TC05000866.hg.1	2.26	PSR05012650.hg.1	CYFIP2
TC05000866.hg.1	2.11	PSR05012656.hg.1	CYFIP2
TC05000866.hg.1	-2.24	PSR05012664.hg.1	CYFIP2
TC05000866.hg.1	-2.26	JUC05006555.hg.1	CYFIP2
TC05000866.hg.1	-2.39	JUC05006535.hg.1	CYFIP2
TC05000866.hg.1	-2.7	PSR05012668.hg.1	CYFIP2
TC05000866.hg.1	-4	PSR05012662.hg.1	CYFIP2
TC06000819.hg.1	2.91	JUC06004542.hg.1	UFL1
TC06000819.hg.1	2.15	PSR06009739.hg.1	UFL1
TC06000851.hg.1	2.91	JUC06004704.hg.1	QRSL1
TC06000851.hg.1	2.74	JUC06004695.hg.1	QRSL1
TC06000851.hg.1	-2.63	PSR06010032.hg.1	QRSL1
TC10000403.hg.1	2.91	PSR10004481.hg.1	HNRNPH3
TC10000403.hg.1	2.6	PSR10004502.hg.1	HNRNPH3
TC10000403.hg.1	-2.77	JUC10002597.hg.1	HNRNPH3
TC14000202.hg.1	2.91	PSR14002217.hg.1	NUBPL
TC14000202.hg.1	2.51	JUC14000995.hg.1	NUBPL
TC14000202.hg.1	2.18	PSR14002220.hg.1	NUBPL
TC16001291.hg.1	2.91	JUC16010221.hg.1	MAF
TC16001291.hg.1	2.01	JUC16010220.hg.1	MAF
TC16001291.hg.1	-2.48	PSR16017944.hg.1	MAF
TC6_mann_hap40(	2.91	JUC6_mann_hap400	HCG18
TC6_mann_hap40(	2.03	PSR6_mann_hap40(	HCG18
TC02001323.hg.1	2.9	JUC02010976.hg.1	DNAJB2

TC02001323.hg.1	2.02	JUC02010982.hg.1	DNAJB2
TC02001323.hg.1	2	PSR02020607.hg.1	DNAJB2
TC02001323.hg.1	-2.01	JUC02010979.hg.1	DNAJB2
TC02001323.hg.1	-2.1	PSR02020601.hg.1	DNAJB2
TC03000315.hg.1	2.9	JUC03003230.hg.1	HEMK1
TC03000315.hg.1	2.17	JUC03003235.hg.1	HEMK1
TC03000315.hg.1	2.11	JUC03003232.hg.1	HEMK1
TC03000315.hg.1	2.01	PSR03006647.hg.1	HEMK1
TC03000315.hg.1	-2.8	PSR03006652.hg.1	HEMK1
TC03000315.hg.1	-3.22	JUC03003227.hg.1	HEMK1
TC03000353.hg.1	2.9	JUC03003822.hg.1	CACNA2D3
TC03000353.hg.1	2.89	JUC03003832.hg.1	CACNA2D3
TC03000353.hg.1	2.56	PSR03007782.hg.1	CACNA2D3
TC03000353.hg.1	2.46	JUC03003817.hg.1	CACNA2D3
TC03000353.hg.1	2.44	PSR03007773.hg.1	CACNA2D3
TC03000353.hg.1	2.35	JUC03003839.hg.1	CACNA2D3
TC03000353.hg.1	2.27	PSR03007814.hg.1	CACNA2D3
TC03000353.hg.1	2.21	JUC03003854.hg.1	CACNA2D3
TC03000353.hg.1	2.2	PSR03007779.hg.1	CACNA2D3
TC03000353.hg.1	2.16	JUC03003811.hg.1	CACNA2D3
TC03000353.hg.1	2.14	PSR03007819.hg.1	CACNA2D3
TC03000353.hg.1	2.11	PSR03007775.hg.1	CACNA2D3
TC03000353.hg.1	2.11	PSR03007808.hg.1	CACNA2D3
TC03000353.hg.1	2.05	PSR03007777.hg.1	CACNA2D3
TC03000353.hg.1	-2.3	JUC03003834.hg.1	CACNA2D3
TC03000353.hg.1	-2.35	JUC03003828.hg.1	CACNA2D3
TC03000353.hg.1	-2.68	JUC03003842.hg.1	CACNA2D3
TC03000353.hg.1	-3.04	JUC03003826.hg.1	CACNA2D3
TC03000353.hg.1	-3.04	JUC03003836.hg.1	CACNA2D3
TC03000353.hg.1	-5.3	JUC03003830.hg.1	CACNA2D3
TC03001241.hg.1	2.9	PSR03021275.hg.1	THRB
TC03001241.hg.1	2.49	JUC03010537.hg.1	THRB
TC03001241.hg.1	2.09	PSR03021256.hg.1	THRB
TC03001241.hg.1	2.09	PSR03021290.hg.1	THRB
TC03001241.hg.1	2.08	PSR03021277.hg.1	THRB
TC03001241.hg.1	2.07	JUC03010551.hg.1	THRB
TC03001241.hg.1	2.06	JUC03010553.hg.1	THRB
TC03001241.hg.1	2.04	PSR03021288.hg.1	THRB
TC03001241.hg.1	2.02	PSR03021262.hg.1	THRB
TC05003440.hg.1	2.9	JUC05019828.hg.1	RFESD
TC05003440.hg.1	-2.02	PSR05006497.hg.1	RFESD
TC1_gl000192_ran	2.9	JUC1_gl000192_random000038.hg	
TC1_gl000192_ran	2.58	JUC1_gl000192_random000031.hg	
TC1_gl000192_ran	2.05	JUC1_gl000192_random000039.hg	
TC1_gl000192_ran	-3.27	PSR1_gl000192_random000028.hg	
TC1_gl000192_ran	-3.81	JUC1_gl000192_random000032.hg	
TC14001189.hg.1	2.9	PSR14013655.hg.1	SIX4
TC14001189.hg.1	2.66	JUC14006884.hg.1	SIX4
TC14001189.hg.1	2.41	PSR14013647.hg.1	SIX4
TC14001189.hg.1	2.13	PSR14013646.hg.1	SIX4
TC16000189.hg.1	2.9	JUC16001449.hg.1	MPV17L
TC16000189.hg.1	-2.09	PSR16002823.hg.1	MPV17L
TC20001751.hg.1	2.9	JUC20010009.hg.1	UBE2V1
TC20001751.hg.1	2.14	PSR20012862.hg.1	UBE2V1
TC20001751.hg.1	-2.06	PSR20012825.hg.1	UBE2V1
TC20001751.hg.1	-2.22	PSR20012826.hg.1	UBE2V1
TC20001751.hg.1	-2.81	JUC20010025.hg.1	UBE2V1
TC02001087.hg.1	2.9	JUC02008661.hg.1	PPP1R1C

TC02001087.hg.1	2	PSR02016268.hg.1	PPP1R1C
TC01000439.hg.1	2.89	JUC01003525.hg.1	ADC
TC01000439.hg.1	2.07	PSR01006797.hg.1	ADC
TC01000439.hg.1	-2.08	PSR01006806.hg.1	ADC
TC02002792.hg.1	2.89	JUC02023525.hg.1	GLB1L
TC02002792.hg.1	2.3	PSR02045059.hg.1	GLB1L
TC02002792.hg.1	2.3	JUC02023538.hg.1	GLB1L
TC02002792.hg.1	2.26	PSR02045078.hg.1	GLB1L
TC02002792.hg.1	2.17	PSR02045040.hg.1	GLB1L
TC02002792.hg.1	2.12	JUC02023545.hg.1	GLB1L
TC02002792.hg.1	2.11	PSR02045074.hg.1	GLB1L
TC02002792.hg.1	2.06	JUC02023535.hg.1	GLB1L
TC02002792.hg.1	2.02	PSR02045083.hg.1	GLB1L
TC02002792.hg.1	2.01	PSR02045066.hg.1	GLB1L
TC02002792.hg.1	-2.03	JUC02023528.hg.1	GLB1L
TC02002859.hg.1	2.89	JUC02024157.hg.1	NCL
TC02002859.hg.1	2.24	PSR02046162.hg.1	NCL
TC02002859.hg.1	2.23	JUC02024171.hg.1	NCL
TC04000155.hg.1	2.89	PSR04002416.hg.1	NCAPG
TC04000155.hg.1	2.87	JUC04001095.hg.1	NCAPG
TC04000155.hg.1	-2.04	JUC04001086.hg.1	NCAPG
TC04000155.hg.1	-2.14	JUC04001101.hg.1	NCAPG
TC04000155.hg.1	-2.28	PSR04002429.hg.1	NCAPG
TC06004150.hg.1	2.89	JUC06021967.hg.1	FKBP5
TC06004150.hg.1	2.45	PSR06019753.hg.1	FKBP5
TC06004150.hg.1	2.19	PSR06019755.hg.1	FKBP5
TC06004150.hg.1	2.14	JUC06021963.hg.1	FKBP5
TC06004150.hg.1	-2.14	PSR06019763.hg.1	FKBP5
TC06004150.hg.1	-3.94	PSR06019751.hg.1	FKBP5
TC06004150.hg.1	-6.86	JUC06021970.hg.1	FKBP5
TC06004150.hg.1	-9.93	JUC06021966.hg.1	FKBP5
TC06004150.hg.1	-11.03	PSR06019768.hg.1	FKBP5
TC06004150.hg.1	-13.4	JUC06021962.hg.1	FKBP5
TC06004150.hg.1	-15.12	JUC06021957.hg.1	FKBP5
TC06004150.hg.1	-22.11	PSR06019745.hg.1	FKBP5
TC06004150.hg.1	-27.78	PSR06019770.hg.1	FKBP5
TC06004150.hg.1	-32.14	PSR06019772.hg.1	FKBP5
TC06004150.hg.1	-36.8	PSR06019777.hg.1	FKBP5
TC06004150.hg.1	-37.59	PSR06019775.hg.1	FKBP5
TC06004150.hg.1	-40.29	JUC06021958.hg.1	FKBP5
TC07003326.hg.1	2.89	PSR07004985.hg.1	GBAS
TC07003326.hg.1	-2.57	JUC07020896.hg.1	GBAS
TC10000199.hg.1	2.89	PSR10002381.hg.1	BAMBI
TC10000199.hg.1	2.05	JUC10001295.hg.1	BAMBI
TC10000424.hg.1	2.89	JUC10002884.hg.1	COL13A1
TC10000424.hg.1	2.27	JUC10002852.hg.1	COL13A1
TC10000424.hg.1	2.16	PSR10004935.hg.1	COL13A1
TC10000424.hg.1	-2.44	JUC10002849.hg.1	COL13A1
TC10000424.hg.1	-4.71	JUC10002896.hg.1	COL13A1
TC10001410.hg.1	2.89	JUC10010371.hg.1	PPP3CB
TC10001410.hg.1	-2.56	PSR10017808.hg.1	PPP3CB
TC10001410.hg.1	-2.64	JUC10010365.hg.1	PPP3CB
TC10001410.hg.1	-2.68	PSR10017805.hg.1	PPP3CB
TC10001410.hg.1	-3.51	PSR10017802.hg.1	PPP3CB
TC10001410.hg.1	-4.02	JUC10010383.hg.1	PPP3CB
TC12000183.hg.1	2.89	JUC12001069.hg.1	LOC1005063
TC12000183.hg.1	2.23	JUC12001077.hg.1	LOC1005063
TC12000183.hg.1	2.19	JUC12001071.hg.1	LOC1005063

TC12000183.hg.1	-2.1	PSR12002317.hg.1	LOC1005063
TC12000418.hg.1	2.89	JUC12002948.hg.1	KRT7
TC12000418.hg.1	2.46	PSR12005361.hg.1	KRT7
TC12000418.hg.1	2.23	PSR12005339.hg.1	KRT7
TC12000418.hg.1	2.09	PSR12005352.hg.1	KRT7
TC12000418.hg.1	-2.41	JUC12002957.hg.1	KRT7
TC12000418.hg.1	-2.42	JUC12002951.hg.1	KRT7
TC17000460.hg.1	2.89	JUC17003067.hg.1	SOCS7
TC17000460.hg.1	2.63	PSR17005398.hg.1	SOCS7
TC17000460.hg.1	2.3	PSR17005386.hg.1	SOCS7
TC17000460.hg.1	2.06	PSR17005399.hg.1	SOCS7
TC19002668.hg.1	2.89	JUC19017698.hg.1	ZNF776
TC19002668.hg.1	2.74	JUC19017699.hg.1	ZNF776
TC19002668.hg.1	2.17	PSR19013047.hg.1	ZNF776
TC19002668.hg.1	2.16	JUC19017700.hg.1	ZNF776
TC19002668.hg.1	2.11	PSR19013051.hg.1	ZNF776
TC19002668.hg.1	2.09	PSR19013046.hg.1	ZNF776
TC19002668.hg.1	2.03	PSR19013050.hg.1	ZNF776
TC01003677.hg.1	2.88	PSR01056295.hg.1	C1orf98
TC01003677.hg.1	2.18	PSR01056297.hg.1	C1orf98
TC01003677.hg.1	2.13	JUC01029416.hg.1	C1orf98
TC01003677.hg.1	-2.4	PSR01056296.hg.1	C1orf98
TC01004096.hg.1	2.88	JUC01032180.hg.1	ZNF692
TC01004096.hg.1	-2.18	PSR01061583.hg.1	ZNF692
TC05000689.hg.1	2.88	PSR05009590.hg.1	MYOT
TC05000689.hg.1	2.82	JUC05005093.hg.1	MYOT
TC05000689.hg.1	2.03	PSR05009589.hg.1	MYOT
TC05000689.hg.1	-2.36	PSR05009575.hg.1	MYOT
TC05000689.hg.1	-2.37	PSR05009572.hg.1	MYOT
TC05000689.hg.1	-2.44	PSR05009588.hg.1	MYOT
TC05000689.hg.1	-2.57	PSR05009574.hg.1	MYOT
TC05000689.hg.1	-2.63	PSR05009576.hg.1	MYOT
TC05000689.hg.1	-5.52	PSR05009578.hg.1	MYOT
TC05001872.hg.1	2.88	PSR05025779.hg.1	ARAP3
TC05001872.hg.1	2.83	PSR05025830.hg.1	ARAP3
TC05001872.hg.1	2.32	JUC05013168.hg.1	ARAP3
TC05001872.hg.1	2.26	PSR05025799.hg.1	ARAP3
TC05001872.hg.1	2.17	JUC05013141.hg.1	ARAP3
TC05001872.hg.1	2.14	JUC05013155.hg.1	ARAP3
TC05001872.hg.1	2.13	PSR05025781.hg.1	ARAP3
TC05001872.hg.1	2.13	PSR05025800.hg.1	ARAP3
TC05001872.hg.1	2.12	JUC05013150.hg.1	ARAP3
TC05001872.hg.1	2.08	JUC05013143.hg.1	ARAP3
TC05001872.hg.1	2.07	JUC05013166.hg.1	ARAP3
TC05001872.hg.1	-2	PSR05025783.hg.1	ARAP3
TC05001872.hg.1	-2.06	PSR05025790.hg.1	ARAP3
TC05001872.hg.1	-2.25	JUC05013160.hg.1	ARAP3
TC05001872.hg.1	-2.3	PSR05025785.hg.1	ARAP3
TC05001872.hg.1	-3.27	PSR05025792.hg.1	ARAP3
TC05001872.hg.1	-3.76	PSR05025819.hg.1	ARAP3
TC05001872.hg.1	-3.8	JUC05013153.hg.1	ARAP3
TC05001872.hg.1	-5.09	JUC05013177.hg.1	ARAP3
TC05001872.hg.1	-6.45	PSR05025786.hg.1	ARAP3
TC05003436.hg.1	2.88	PSR05028923.hg.1	RAB24
TC05003436.hg.1	-2.16	JUC05019767.hg.1	RAB24
TC06001142.hg.1	2.88	PSR06013575.hg.1	FNDC1
TC06001142.hg.1	2.25	PSR06013573.hg.1	FNDC1
TC06001142.hg.1	-2.58	JUC06006699.hg.1	FNDC1

TC07001087.hg.1	2.88	PSR07016753.hg.1	TMEM184A
TC07001087.hg.1	2.12	PSR07016751.hg.1	TMEM184A
TC07001087.hg.1	2.11	JUC07008114.hg.1	TMEM184A
TC07001087.hg.1	2.09	PSR07016747.hg.1	TMEM184A
TC07001087.hg.1	2.07	JUC07008128.hg.1	TMEM184A
TC07001124.hg.1	2.88	JUC07008492.hg.1	PMS2
TC07001124.hg.1	-3.36	PSR07017415.hg.1	PMS2
TC0X001324.hg.1	2.88	PSR0X017758.hg.1	FAM70A
TC0X001324.hg.1	2.33	JUC0X009044.hg.1	FAM70A
TC0X001324.hg.1	2.25	JUC0X009047.hg.1	FAM70A
TC0X001324.hg.1	2	JUC0X009037.hg.1	FAM70A
TC0X001324.hg.1	-2.29	JUC0X009045.hg.1	FAM70A
TC0X001324.hg.1	-2.36	PSR0X017760.hg.1	FAM70A
TC13000717.hg.1	2.88	PSR13008298.hg.1	PCDH9
TC13000717.hg.1	2.16	PSR13008301.hg.1	PCDH9
TC13000717.hg.1	2.08	JUC13004850.hg.1	PCDH9
TC13000717.hg.1	-2	PSR13008296.hg.1	PCDH9
TC13000717.hg.1	-2.19	PSR13008291.hg.1	PCDH9
TC13000717.hg.1	-2.39	JUC13004847.hg.1	PCDH9
TC13000717.hg.1	-2.51	JUC13004854.hg.1	PCDH9
TC13000717.hg.1	-2.51	JUC13004855.hg.1	PCDH9
TC13000717.hg.1	-3.2	PSR13008287.hg.1	PCDH9
TC13000717.hg.1	-3.44	PSR13008290.hg.1	PCDH9
TC13000717.hg.1	-3.44	JUC13004846.hg.1	PCDH9
TC13000717.hg.1	-3.95	JUC13004849.hg.1	PCDH9
TC13000717.hg.1	-4.35	PSR13008294.hg.1	PCDH9
TC13000717.hg.1	-4.61	JUC13004853.hg.1	PCDH9
TC13000717.hg.1	-4.64	PSR13008285.hg.1	PCDH9
TC13000717.hg.1	-6.55	PSR13008286.hg.1	PCDH9
TC13000717.hg.1	-8.26	JUC13004852.hg.1	PCDH9
TC13000892.hg.1	2.88	JUC13005856.hg.1	PCID2
TC13000892.hg.1	2.14	PSR13009947.hg.1	PCID2
TC13000892.hg.1	2.05	PSR13009962.hg.1	PCID2
TC13000892.hg.1	-4.22	PSR13009956.hg.1	PCID2
TC14001217.hg.1	2.88	JUC14006996.hg.1	ZBTB25
TC14001217.hg.1	-2.14	JUC14006995.hg.1	ZBTB25
TC14001217.hg.1	-2.39	PSR14013857.hg.1	ZBTB25
TC14001217.hg.1	-7.52	JUC14006993.hg.1	ZBTB25
TC16000660.hg.1	2.88	JUC16005223.hg.1	CRISPLD2
TC16000660.hg.1	2.09	JUC16005226.hg.1	CRISPLD2
TC16000660.hg.1	2.05	JUC16005222.hg.1	CRISPLD2
TC16000660.hg.1	-2.65	PSR16009326.hg.1	CRISPLD2
TC16000660.hg.1	-2.87	PSR16009331.hg.1	CRISPLD2
TC16000660.hg.1	-6.55	PSR16009328.hg.1	CRISPLD2
TC16000660.hg.1	-9.79	PSR16009339.hg.1	CRISPLD2
TC17001118.hg.1	2.88	JUC17008543.hg.1	MYH10
TC17001118.hg.1	2.54	JUC17008578.hg.1	MYH10
TC17001118.hg.1	2.42	JUC17008575.hg.1	MYH10
TC17001118.hg.1	2.3	PSR17015038.hg.1	MYH10
TC17001118.hg.1	2.06	PSR17015086.hg.1	MYH10
TC17001118.hg.1	-2.5	JUC17008570.hg.1	MYH10
TC17001903.hg.1	2.88	JUC17014510.hg.1	ST6GALNAC
TC17001903.hg.1	2.16	JUC17014513.hg.1	ST6GALNAC
TC17001903.hg.1	-2.03	PSR17025820.hg.1	ST6GALNAC
TC17001903.hg.1	-2.05	PSR17025818.hg.1	ST6GALNAC
TC17001903.hg.1	-2.24	PSR17025825.hg.1	ST6GALNAC
TC17001903.hg.1	-3.29	JUC17014514.hg.1	ST6GALNAC
TC17001903.hg.1	-5.36	JUC17014515.hg.1	ST6GALNAC

TC19000144.hg.1	2.88	JUC19001375.hg.1	CERS4
TC19000144.hg.1	2.68	PSR19002283.hg.1	CERS4
TC19000144.hg.1	2.66	PSR19002297.hg.1	CERS4
TC19000144.hg.1	2.32	JUC19001377.hg.1	CERS4
TC19000144.hg.1	2.23	PSR19002294.hg.1	CERS4
TC19000144.hg.1	2.22	PSR19002284.hg.1	CERS4
TC19000144.hg.1	-2.02	PSR19002293.hg.1	CERS4
TC19000144.hg.1	-2.32	PSR19002291.hg.1	CERS4
TC19000144.hg.1	-2.48	JUC19001368.hg.1	CERS4
TC19000144.hg.1	-2.82	PSR19002285.hg.1	CERS4
TC01001993.hg.1	2.87	JUC01016796.hg.1	EFCAB2
TC01001993.hg.1	2.27	JUC01016805.hg.1	EFCAB2
TC01001993.hg.1	-2.06	JUC01016807.hg.1	EFCAB2
TC01001993.hg.1	-2.12	PSR01030863.hg.1	EFCAB2
TC01001993.hg.1	-2.15	JUC01016811.hg.1	EFCAB2
TC01001993.hg.1	-2.19	PSR01030900.hg.1	EFCAB2
TC01001993.hg.1	-2.22	PSR01030873.hg.1	EFCAB2
TC01001993.hg.1	-2.23	PSR01030860.hg.1	EFCAB2
TC01001993.hg.1	-2.28	PSR01030865.hg.1	EFCAB2
TC01001993.hg.1	-2.3	PSR01030896.hg.1	EFCAB2
TC01001993.hg.1	-2.32	PSR01030888.hg.1	EFCAB2
TC01001993.hg.1	-2.39	JUC01016798.hg.1	EFCAB2
TC01001993.hg.1	-2.45	PSR01030898.hg.1	EFCAB2
TC01001993.hg.1	-2.49	JUC01016808.hg.1	EFCAB2
TC01001993.hg.1	-2.6	PSR01030889.hg.1	EFCAB2
TC01001993.hg.1	-3.02	JUC01016797.hg.1	EFCAB2
TC01001993.hg.1	-3.72	PSR01030884.hg.1	EFCAB2
TC01001993.hg.1	-4.04	JUC01016801.hg.1	EFCAB2
TC02000524.hg.1	2.87	PSR02008183.hg.1	KDM3A
TC02000524.hg.1	2.8	JUC02004174.hg.1	KDM3A
TC02000524.hg.1	2.71	PSR02008187.hg.1	KDM3A
TC02000524.hg.1	2.55	PSR02008181.hg.1	KDM3A
TC02000524.hg.1	2.5	JUC02004168.hg.1	KDM3A
TC02000524.hg.1	2.27	JUC02004154.hg.1	KDM3A
TC02000524.hg.1	2.2	PSR02008142.hg.1	KDM3A
TC02000524.hg.1	-2.09	PSR02008168.hg.1	KDM3A
TC02000524.hg.1	-2.43	JUC02004184.hg.1	KDM3A
TC02000524.hg.1	-2.86	JUC02004189.hg.1	KDM3A
TC04000859.hg.1	2.87	JUC04006355.hg.1	CEP44
TC04000859.hg.1	2.79	PSR04011856.hg.1	CEP44
TC04000859.hg.1	-2.28	JUC04006353.hg.1	CEP44
TC06000894.hg.1	2.87	JUC06005014.hg.1	WISP3
TC06000894.hg.1	2.79	PSR06010559.hg.1	WISP3
TC06000894.hg.1	2.63	JUC06005003.hg.1	WISP3
TC06000894.hg.1	2.62	PSR06010544.hg.1	WISP3
TC06000894.hg.1	-2.8	JUC06005007.hg.1	WISP3
TC07000276.hg.1	2.87	JUC07002044.hg.1	OGDH
TC07000276.hg.1	2.26	JUC07002039.hg.1	OGDH
TC07000276.hg.1	-2.62	PSR07004280.hg.1	OGDH
TC07000449.hg.1	2.87	JUC07002913.hg.1	ELN
TC07000449.hg.1	2.83	JUC07002925.hg.1	ELN
TC07000449.hg.1	2.61	JUC07002932.hg.1	ELN
TC07000449.hg.1	2.53	PSR07006046.hg.1	ELN
TC07000449.hg.1	2.44	JUC07002917.hg.1	ELN
TC07000449.hg.1	2.29	JUC07002924.hg.1	ELN
TC07000449.hg.1	2.23	PSR07005982.hg.1	ELN
TC07000449.hg.1	2.2	JUC07002890.hg.1	ELN
TC07000449.hg.1	2.15	PSR07006007.hg.1	ELN

TC07000449.hg.1	2.15	PSR07006055.hg.1	ELN
TC07000449.hg.1	2.09	PSR07005992.hg.1	ELN
TC07000449.hg.1	2.05	PSR07005983.hg.1	ELN
TC07000449.hg.1	2.01	JUC07002877.hg.1	ELN
TC07000449.hg.1	-2	PSR07006025.hg.1	ELN
TC07000449.hg.1	-2.09	JUC07002907.hg.1	ELN
TC07000449.hg.1	-2.11	JUC07002903.hg.1	ELN
TC07000449.hg.1	-2.13	JUC07002914.hg.1	ELN
TC07000449.hg.1	-2.29	PSR07005989.hg.1	ELN
TC07000449.hg.1	-2.3	PSR07006030.hg.1	ELN
TC07000449.hg.1	-2.4	JUC07002918.hg.1	ELN
TC07000449.hg.1	-2.44	PSR07006000.hg.1	ELN
TC07000449.hg.1	-2.55	PSR07005986.hg.1	ELN
TC07000449.hg.1	-2.56	JUC07002928.hg.1	ELN
TC07000449.hg.1	-2.79	JUC07002911.hg.1	ELN
TC07000449.hg.1	-2.8	JUC07002874.hg.1	ELN
TC07000449.hg.1	-2.81	PSR07006032.hg.1	ELN
TC07000449.hg.1	-2.83	PSR07005991.hg.1	ELN
TC07000449.hg.1	-2.86	JUC07002886.hg.1	ELN
TC07000449.hg.1	-2.87	PSR07006019.hg.1	ELN
TC07000449.hg.1	-2.87	JUC07002930.hg.1	ELN
TC07000449.hg.1	-2.9	JUC07002878.hg.1	ELN
TC07000449.hg.1	-2.93	PSR07005993.hg.1	ELN
TC07000449.hg.1	-3.03	JUC07002873.hg.1	ELN
TC07000449.hg.1	-3.15	PSR07005981.hg.1	ELN
TC07000449.hg.1	-3.19	PSR07006008.hg.1	ELN
TC07000449.hg.1	-3.35	PSR07005999.hg.1	ELN
TC07000449.hg.1	-3.4	JUC07002919.hg.1	ELN
TC07000449.hg.1	-3.52	PSR07006012.hg.1	ELN
TC07000449.hg.1	-3.74	JUC07002882.hg.1	ELN
TC07000449.hg.1	-3.9	JUC07002883.hg.1	ELN
TC07000449.hg.1	-3.91	JUC07002929.hg.1	ELN
TC07000449.hg.1	-3.99	PSR07006022.hg.1	ELN
TC07000449.hg.1	-4.12	PSR07006023.hg.1	ELN
TC07000449.hg.1	-4.21	PSR07006006.hg.1	ELN
TC07000449.hg.1	-4.39	JUC07002920.hg.1	ELN
TC07000449.hg.1	-4.42	PSR07006015.hg.1	ELN
TC07000449.hg.1	-4.66	JUC07002916.hg.1	ELN
TC07000449.hg.1	-4.71	JUC07002904.hg.1	ELN
TC07000449.hg.1	-4.87	JUC07002888.hg.1	ELN
TC07000449.hg.1	-5.09	PSR07005987.hg.1	ELN
TC07000449.hg.1	-5.18	PSR07005963.hg.1	ELN
TC07000449.hg.1	-5.19	JUC07002900.hg.1	ELN
TC07000449.hg.1	-5.66	JUC07002891.hg.1	ELN
TC07000449.hg.1	-5.67	PSR07006021.hg.1	ELN
TC07000449.hg.1	-5.75	PSR07006014.hg.1	ELN
TC07000449.hg.1	-6.04	JUC07002912.hg.1	ELN
TC07000449.hg.1	-6.14	PSR07006031.hg.1	ELN
TC07000449.hg.1	-6.33	PSR07005997.hg.1	ELN
TC07000449.hg.1	-6.36	PSR07006017.hg.1	ELN
TC07000449.hg.1	-8.04	JUC07002906.hg.1	ELN
TC07001568.hg.1	2.87	JUC07011800.hg.1	SEMA3A
TC07001568.hg.1	2.08	PSR07023692.hg.1	SEMA3A
TC07001568.hg.1	2.03	JUC07011795.hg.1	SEMA3A
TC07001568.hg.1	-2	PSR07023703.hg.1	SEMA3A
TC07001568.hg.1	-2.05	PSR07023701.hg.1	SEMA3A
TC07001568.hg.1	-2.29	PSR07023702.hg.1	SEMA3A
TC07001568.hg.1	-4.24	JUC07011806.hg.1	SEMA3A

TC09001468.hg.1	2.87	PSR09018162.hg.1	PTPN3
TC09001468.hg.1	2.24	PSR09018157.hg.1	PTPN3
TC09001468.hg.1	2.11	JUC09009806.hg.1	PTPN3
TC09001468.hg.1	2.09	JUC09009800.hg.1	PTPN3
TC09001643.hg.1	2.87	JUC09011259.hg.1	CRAT
TC09001643.hg.1	2.76	JUC09011256.hg.1	CRAT
TC09001643.hg.1	2.4	JUC09011253.hg.1	CRAT
TC09001643.hg.1	2.3	PSR09020977.hg.1	CRAT
TC09001643.hg.1	2.07	JUC09011241.hg.1	CRAT
TC09001643.hg.1	2.04	PSR09020964.hg.1	CRAT
TC09001643.hg.1	2	PSR09020960.hg.1	CRAT
TC11000684.hg.1	2.87	JUC11004527.hg.1	SSH3
TC11000684.hg.1	2.64	JUC11004534.hg.1	SSH3
TC11000684.hg.1	-2	JUC11004529.hg.1	SSH3
TC11000684.hg.1	-2.83	PSR11008875.hg.1	SSH3
TC15001501.hg.1	2.87	JUC15007619.hg.1	GTF2A2
TC15001501.hg.1	-2.12	PSR15014142.hg.1	GTF2A2
TC16000137.hg.1	2.87	JUC16001060.hg.1	UBN1
TC16000137.hg.1	2.17	PSR16002155.hg.1	UBN1
TC19001826.hg.1	2.87	PSR19024692.hg.1	NLRP12
TC19001826.hg.1	-2.4	JUC19014033.hg.1	NLRP12
TC19001826.hg.1	-2.75	JUC19014037.hg.1	NLRP12
TC20000544.hg.1	2.87	PSR20007852.hg.1	SCRT2, SRXI
TC20000544.hg.1	2.72	JUC20004093.hg.1	SCRT2, SRXI
TC20000544.hg.1	2.13	PSR20007851.hg.1	SCRT2, SRXI
TC22001460.hg.1	2.87	PSR22002936.hg.1	GGT1, GGT2
TC22001460.hg.1	2.19	JUC22009158.hg.1	GGT1, GGT2
TC22001460.hg.1	2.17	JUC22009178.hg.1	GGT1, GGT2
TC22001460.hg.1	-2.48	PSR22002918.hg.1	GGT1, GGT2
TC22001460.hg.1	-2.54	PSR22002927.hg.1	GGT1, GGT2
TC22001460.hg.1	-2.74	JUC22009170.hg.1	GGT1, GGT2
TC22001460.hg.1	-3.24	JUC22009190.hg.1	GGT1, GGT2
TC22001460.hg.1	-3.46	JUC22009176.hg.1	GGT1, GGT2
TC22001460.hg.1	-4.65	PSR22002879.hg.1	GGT1, GGT2
TC22001460.hg.1	-18.14	JUC22009191.hg.1	GGT1, GGT2
TC6_mcf_hap5000	2.87	JUC6_mcf_hap5000	DDR1
TC6_mcf_hap5000	2.6	JUC6_mcf_hap5000	DDR1
TC6_mcf_hap5000	2.56	PSR6_mcf_hap5000	DDR1
TC6_mcf_hap5000	2.5	PSR6_mcf_hap5000	DDR1
TC6_mcf_hap5000	2.48	JUC6_mcf_hap5000	DDR1
TC6_mcf_hap5000	2.45	PSR6_mcf_hap5000	DDR1
TC6_mcf_hap5000	2.37	JUC6_mcf_hap5000	DDR1
TC6_mcf_hap5000	2.32	JUC6_mcf_hap5000	DDR1
TC6_mcf_hap5000	2.29	PSR6_mcf_hap5000	DDR1
TC6_mcf_hap5000	2.17	JUC6_mcf_hap5000	DDR1
TC6_mcf_hap5000	2.16	PSR6_mcf_hap5000	DDR1
TC6_mcf_hap5000	2.09	JUC6_mcf_hap5000	DDR1
TC6_mcf_hap5000	2.02	PSR6_mcf_hap5000	DDR1
TC6_mcf_hap5000	2.02	JUC6_mcf_hap5000	DDR1
TC6_mcf_hap5000	-2.86	PSR6_mcf_hap5000	DDR1
TC02002035.hg.1	2.86	PSR02032537.hg.1	TGOLN2
TC02002035.hg.1	2.64	JUC02016803.hg.1	TGOLN2
TC02002035.hg.1	2.16	JUC02016806.hg.1	TGOLN2
TC02002035.hg.1	2.08	PSR02032535.hg.1	TGOLN2
TC02002035.hg.1	2.01	PSR02032536.hg.1	TGOLN2
TC03000502.hg.1	2.86	JUC03004713.hg.1	COL8A1
TC03000502.hg.1	2.19	PSR03009426.hg.1	COL8A1
TC03000502.hg.1	2.15	PSR03009425.hg.1	COL8A1

TC04000503.hg.1	2.86	JUC04003831.hg.1	BMPR1B
TC04000503.hg.1	2.82	PSR04007403.hg.1	BMPR1B
TC04000503.hg.1	2.33	PSR04007402.hg.1	BMPR1B
TC04000503.hg.1	2.25	PSR04007396.hg.1	BMPR1B
TC04000503.hg.1	-2.08	PSR04007420.hg.1	BMPR1B
TC04000503.hg.1	-2.54	JUC04003844.hg.1	BMPR1B
TC04000503.hg.1	-2.76	PSR04007397.hg.1	BMPR1B
TC04000503.hg.1	-3.64	PSR04007399.hg.1	BMPR1B
TC04000503.hg.1	-3.78	PSR04007416.hg.1	BMPR1B
TC04000503.hg.1	-4.15	PSR04007398.hg.1	BMPR1B
TC04000503.hg.1	-4.91	PSR04007395.hg.1	BMPR1B
TC04000503.hg.1	-5.9	PSR04007392.hg.1	BMPR1B
TC04000503.hg.1	-7.43	PSR04007400.hg.1	BMPR1B
TC08000207.hg.1	2.86	JUC08001431.hg.1	PTK2B
TC08000207.hg.1	2.63	JUC08001455.hg.1	PTK2B
TC08000207.hg.1	2.15	PSR08002962.hg.1	PTK2B
TC08000207.hg.1	2.1	JUC08001430.hg.1	PTK2B
TC08000207.hg.1	-2.11	PSR08002960.hg.1	PTK2B
TC08000207.hg.1	-2.2	PSR08002948.hg.1	PTK2B
TC08000207.hg.1	-2.22	JUC08001447.hg.1	PTK2B
TC08000207.hg.1	-2.48	PSR08002951.hg.1	PTK2B
TC08000207.hg.1	-2.51	PSR08002974.hg.1	PTK2B
TC08000207.hg.1	-2.52	PSR08002929.hg.1	PTK2B
TC08000207.hg.1	-2.55	PSR08002950.hg.1	PTK2B
TC08000207.hg.1	-2.66	PSR08002955.hg.1	PTK2B
TC08000207.hg.1	-2.8	PSR08002954.hg.1	PTK2B
TC08000207.hg.1	-2.83	PSR08002982.hg.1	PTK2B
TC08000207.hg.1	-2.89	PSR08002957.hg.1	PTK2B
TC08000207.hg.1	-3.1	PSR08002941.hg.1	PTK2B
TC08000207.hg.1	-3.1	JUC08001424.hg.1	PTK2B
TC08000207.hg.1	-3.17	PSR08002956.hg.1	PTK2B
TC08000207.hg.1	-3.23	PSR08002977.hg.1	PTK2B
TC08000207.hg.1	-3.25	PSR08002933.hg.1	PTK2B
TC08000207.hg.1	-3.36	PSR08002980.hg.1	PTK2B
TC08000207.hg.1	-3.37	JUC08001437.hg.1	PTK2B
TC08000207.hg.1	-3.49	PSR08002924.hg.1	PTK2B
TC08000207.hg.1	-3.5	JUC08001443.hg.1	PTK2B
TC08000207.hg.1	-3.51	PSR08002934.hg.1	PTK2B
TC08000207.hg.1	-3.63	JUC08001458.hg.1	PTK2B
TC08000207.hg.1	-3.91	PSR08002979.hg.1	PTK2B
TC08000207.hg.1	-3.99	PSR08002967.hg.1	PTK2B
TC08000207.hg.1	-4.03	PSR08002927.hg.1	PTK2B
TC08000207.hg.1	-4.07	PSR08002926.hg.1	PTK2B
TC08000207.hg.1	-4.07	JUC08001445.hg.1	PTK2B
TC08000207.hg.1	-4.19	JUC08001466.hg.1	PTK2B
TC08000207.hg.1	-4.35	PSR08002922.hg.1	PTK2B
TC08000207.hg.1	-4.39	JUC08001436.hg.1	PTK2B
TC08000207.hg.1	-4.51	PSR08002940.hg.1	PTK2B
TC08000207.hg.1	-4.68	JUC08001450.hg.1	PTK2B
TC08000207.hg.1	-4.7	JUC08001439.hg.1	PTK2B
TC08000207.hg.1	-4.81	PSR08002930.hg.1	PTK2B
TC08000207.hg.1	-4.88	JUC08001467.hg.1	PTK2B
TC08000207.hg.1	-4.92	PSR08002932.hg.1	PTK2B
TC08000207.hg.1	-5.27	PSR08002919.hg.1	PTK2B
TC08000207.hg.1	-5.29	PSR08002925.hg.1	PTK2B
TC08000207.hg.1	-5.39	PSR08002920.hg.1	PTK2B
TC08000207.hg.1	-6.3	JUC08001426.hg.1	PTK2B
TC08000207.hg.1	-6.75	JUC08001454.hg.1	PTK2B

TC08000207.hg.1	-7.25	JUC08001461.hg.1	PTK2B
TC08000207.hg.1	-9.42	PSR08002923.hg.1	PTK2B
TC08000207.hg.1	-11.85	PSR08002921.hg.1	PTK2B
TC08000207.hg.1	-12.09	JUC08001423.hg.1	PTK2B
TC08001481.hg.1	2.86	JUC08009825.hg.1	PABPC1
TC08001481.hg.1	-2.15	PSR08019327.hg.1	PABPC1
TC10000941.hg.1	2.86	JUC10006735.hg.1	DPYSL4
TC10000941.hg.1	-2	JUC10006739.hg.1	DPYSL4
TC10000941.hg.1	-2.74	JUC10006752.hg.1	DPYSL4
TC10000941.hg.1	-2.93	JUC10006738.hg.1	DPYSL4
TC10000941.hg.1	-3.02	PSR10011561.hg.1	DPYSL4
TC10000941.hg.1	-3.35	PSR10011553.hg.1	DPYSL4
TC10000941.hg.1	-5.37	JUC10006753.hg.1	DPYSL4
TC10000941.hg.1	-5.74	PSR10011552.hg.1	DPYSL4
TC10000941.hg.1	-24.11	JUC10006750.hg.1	DPYSL4
TC10001486.hg.1	2.86	JUC10010950.hg.1	GLUD1
TC10001486.hg.1	2.21	PSR10018812.hg.1	GLUD1
TC10001486.hg.1	2.13	JUC10010958.hg.1	GLUD1
TC11000702.hg.1	2.86	JUC11004648.hg.1	ALDH3B1
TC11000702.hg.1	2.05	PSR11009096.hg.1	ALDH3B1
TC12001543.hg.1	2.86	JUC12011278.hg.1	CSAD
TC12001543.hg.1	-2.04	PSR12020356.hg.1	CSAD
TC12001543.hg.1	-2.2	JUC12011281.hg.1	CSAD
TC13000111.hg.1	2.86	JUC13000727.hg.1	BRCA2
TC13000111.hg.1	-2.34	PSR13001192.hg.1	BRCA2
TC14002258.hg.1	2.86	PSR14017645.hg.1	IGHA2, IGHE
TC14002258.hg.1	2.43	JUC14011563.hg.1	IGHA2, IGHE
TC14002258.hg.1	2.4	JUC14011502.hg.1	IGHA2, IGHE
TC14002258.hg.1	2.4	JUC14011505.hg.1	IGHA2, IGHE
TC14002258.hg.1	2.4	JUC14011507.hg.1	IGHA2, IGHE
TC14002258.hg.1	2.18	PSR14017759.hg.1	IGHA2, IGHE
TC14002258.hg.1	-2.31	PSR14017756.hg.1	IGHA2, IGHE
TC14002258.hg.1	-2.59	JUC14011487.hg.1	IGHA2, IGHE
TC17001459.hg.1	2.86	PSR17019334.hg.1	NR1D1
TC17001459.hg.1	2.8	JUC17010958.hg.1	NR1D1
TC17001459.hg.1	-3.72	JUC17010959.hg.1	NR1D1
TC18000565.hg.1	2.86	PSR18005886.hg.1	TMX3
TC18000565.hg.1	2.53	JUC18003929.hg.1	TMX3
TC18000565.hg.1	2.43	JUC18003939.hg.1	TMX3
TC18000565.hg.1	-2.04	PSR18005904.hg.1	TMX3
TC22000861.hg.1	2.86	JUC22006030.hg.1	SCUBE1
TC22000861.hg.1	-2.12	PSR22014579.hg.1	SCUBE1
TC22000861.hg.1	-2.15	JUC22006013.hg.1	SCUBE1
TC6_mann_hap40(	2.86	PSR6_mann_hap400	HLA-A
TC6_mann_hap40(	2.81	PSR6_mann_hap400	HLA-A
TC6_mann_hap40(	2.3	JUC6_mann_hap400	HLA-A
TC6_mann_hap40(	2.02	PSR6_mann_hap400	HLA-A
TC6_mann_hap40(	-4.96	JUC6_mann_hap400	HLA-A
TC02001354.hg.1	2.85	PSR02021099.hg.1	MRPL44
TC02001354.hg.1	-2.11	JUC02011237.hg.1	MRPL44
TC02001354.hg.1	-3.31	JUC02011239.hg.1	MRPL44
TC02001768.hg.1	2.85	PSR02028514.hg.1	THUMPD2
TC02001768.hg.1	-2.44	PSR02028524.hg.1	THUMPD2
TC02001768.hg.1	-2.59	JUC02014949.hg.1	THUMPD2
TC02002620.hg.1	2.85	JUC02021895.hg.1	TMEM194B
TC02002620.hg.1	2.69	JUC02021887.hg.1	TMEM194B
TC02002620.hg.1	2.26	PSR02041984.hg.1	TMEM194B
TC02002620.hg.1	2.06	PSR02041992.hg.1	TMEM194B

TC02002620.hg.1	2.03	JUC02021896.hg.1	TMEM194B
TC04000699.hg.1	2.85	JUC04005307.hg.1	GAB1
TC04000699.hg.1	2.41	PSR04009909.hg.1	GAB1
TC04000699.hg.1	2.38	JUC04005308.hg.1	GAB1
TC04000699.hg.1	2.36	PSR04009899.hg.1	GAB1
TC04000699.hg.1	2.36	PSR04009906.hg.1	GAB1
TC04000699.hg.1	2.3	PSR04009911.hg.1	GAB1
TC04000699.hg.1	2.25	PSR04009916.hg.1	GAB1
TC04000699.hg.1	2.19	PSR04009913.hg.1	GAB1
TC04000699.hg.1	2.15	PSR04009902.hg.1	GAB1
TC04000699.hg.1	2.13	PSR04009920.hg.1	GAB1
TC04000699.hg.1	2.1	PSR04009905.hg.1	GAB1
TC04000699.hg.1	-2.79	JUC04005304.hg.1	GAB1
TC04002920.hg.1	2.85	JUC04016905.hg.1	NAAA
TC04002920.hg.1	-2.02	PSR04017821.hg.1	NAAA
TC04002920.hg.1	-2.08	PSR04017818.hg.1	NAAA
TC04002920.hg.1	-2.2	PSR04017801.hg.1	NAAA
TC04002920.hg.1	-2.35	PSR04017815.hg.1	NAAA
TC04002920.hg.1	-2.75	JUC04016891.hg.1	NAAA
TC05001627.hg.1	2.85	JUC05011433.hg.1	FLJ35946
TC05001627.hg.1	-2.64	PSR05022164.hg.1	FLJ35946
TC05001627.hg.1	-10.73	PSR05022166.hg.1	FLJ35946
TC09000217.hg.1	2.85	JUC09001374.hg.1	TRMT10B
TC09000217.hg.1	-3.15	PSR09002571.hg.1	TRMT10B
TC11001912.hg.1	2.85	JUC11011988.hg.1	MAP4K2
TC11001912.hg.1	2.23	JUC11012003.hg.1	MAP4K2
TC11001912.hg.1	2.15	JUC11011995.hg.1	MAP4K2
TC11001912.hg.1	-2.7	PSR11022740.hg.1	MAP4K2
TC11002369.hg.1	2.85	PSR11028527.hg.1	C1QTNF5, M
TC11002369.hg.1	2.19	JUC11015219.hg.1	C1QTNF5, M
TC11002369.hg.1	2.05	PSR11028528.hg.1	C1QTNF5, M
TC15000664.hg.1	2.85	JUC15002901.hg.1	NEO1
TC15000664.hg.1	2.65	JUC15002921.hg.1	NEO1
TC15000664.hg.1	2.45	PSR15005712.hg.1	NEO1
TC15000664.hg.1	2.39	JUC15002922.hg.1	NEO1
TC15000664.hg.1	2.14	PSR15005701.hg.1	NEO1
TC15000664.hg.1	2	PSR15005703.hg.1	NEO1
TC22000025.hg.1	2.85	JUC22000109.hg.1	BCL2L13
TC22000025.hg.1	2.47	JUC22000097.hg.1	BCL2L13
TC22000025.hg.1	2.06	PSR22000194.hg.1	BCL2L13
TC22000025.hg.1	2.05	PSR22000167.hg.1	BCL2L13
TC01000100.hg.1	2.84	PSR01001600.hg.1	PER3
TC01000100.hg.1	2.35	PSR01001599.hg.1	PER3
TC01000100.hg.1	2.12	JUC01000847.hg.1	PER3
TC01000100.hg.1	2.07	PSR01001613.hg.1	PER3
TC01000100.hg.1	-2.25	PSR01001618.hg.1	PER3
TC01000100.hg.1	-2.37	JUC01000858.hg.1	PER3
TC01000100.hg.1	-3.21	JUC01000840.hg.1	PER3
TC01000391.hg.1	2.84	JUC01003125.hg.1	YTHDF2
TC01000391.hg.1	2.59	JUC01003128.hg.1	YTHDF2
TC01000391.hg.1	-2.17	PSR01006027.hg.1	YTHDF2
TC01003238.hg.1	2.84	JUC01026491.hg.1	THEM4
TC01003238.hg.1	-2.03	JUC01026493.hg.1	THEM4
TC01003238.hg.1	-2.16	PSR01050000.hg.1	THEM4
TC01003238.hg.1	-2.27	JUC01026490.hg.1	THEM4
TC01003238.hg.1	-4.53	PSR01050001.hg.1	THEM4
TC02001654.hg.1	2.84	PSR02026423.hg.1	DNAJC27
TC02001654.hg.1	2.33	JUC02013793.hg.1	DNAJC27

TC02001654.hg.1	2.09	PSR02026428.hg.1	DNAJC27
TC02001654.hg.1	2.07	PSR02026424.hg.1	DNAJC27
TC02001736.hg.1	2.84	JUC02014589.hg.1	STRN
TC02001736.hg.1	2.03	JUC02014582.hg.1	STRN
TC02001736.hg.1	-2.13	PSR02027904.hg.1	STRN
TC02001736.hg.1	-2.41	JUC02014594.hg.1	STRN
TC03001277.hg.1	2.84	PSR03021958.hg.1	CLASP2
TC03001277.hg.1	-2.24	JUC03010950.hg.1	CLASP2
TC03001277.hg.1	-2.83	JUC03010916.hg.1	CLASP2
TC05000005.hg.1	2.84	JUC05000069.hg.1	PDCD6, AHR
TC05000005.hg.1	2.3	JUC05000061.hg.1	PDCD6, AHR
TC05000005.hg.1	2.21	PSR05000139.hg.1	PDCD6, AHR
TC05000005.hg.1	2.12	PSR05000109.hg.1	PDCD6, AHR
TC05000005.hg.1	2.12	PSR05000131.hg.1	PDCD6, AHR
TC05000005.hg.1	2.11	JUC05000068.hg.1	PDCD6, AHR
TC05000005.hg.1	2.08	PSR05000129.hg.1	PDCD6, AHR
TC05000005.hg.1	2.02	JUC05000060.hg.1	PDCD6, AHR
TC05000005.hg.1	-2.2	PSR05000138.hg.1	PDCD6, AHR
TC05000005.hg.1	-2.28	PSR05000141.hg.1	PDCD6, AHR
TC05000005.hg.1	-2.36	JUC05000055.hg.1	PDCD6, AHR
TC05000005.hg.1	-2.44	PSR05000154.hg.1	PDCD6, AHR
TC05000005.hg.1	-3.88	JUC05000082.hg.1	PDCD6, AHR
TC06001229.hg.1	2.84	JUC06007274.hg.1	SLC22A23
TC06001229.hg.1	2.05	JUC06007262.hg.1	SLC22A23
TC06001229.hg.1	2.05	JUC06007283.hg.1	SLC22A23
TC06001229.hg.1	-2.14	JUC06007281.hg.1	SLC22A23
TC06001229.hg.1	-2.2	PSR06014615.hg.1	SLC22A23
TC06001229.hg.1	-2.61	JUC06007273.hg.1	SLC22A23
TC06001229.hg.1	-2.75	JUC06007276.hg.1	SLC22A23
TC06001229.hg.1	-2.98	PSR06014616.hg.1	SLC22A23
TC0X000120.hg.1	2.84	JUC0X000885.hg.1	POLA1
TC0X000120.hg.1	2.71	JUC0X000881.hg.1	POLA1
TC0X000120.hg.1	2.45	JUC0X000910.hg.1	POLA1
TC0X000120.hg.1	2.28	JUC0X000897.hg.1	POLA1
TC0X000120.hg.1	-2.07	JUC0X000875.hg.1	POLA1
TC0X000120.hg.1	-2.55	JUC0X000906.hg.1	POLA1
TC0X000120.hg.1	-2.57	JUC0X000911.hg.1	POLA1
TC0X000120.hg.1	-3	PSR0X001641.hg.1	POLA1
TC10001550.hg.1	2.84	JUC10011427.hg.1	SORBS1
TC10001550.hg.1	2.73	JUC10011407.hg.1	SORBS1
TC10001550.hg.1	2.29	JUC10011400.hg.1	SORBS1
TC10001550.hg.1	2.13	JUC10011389.hg.1	SORBS1
TC10001550.hg.1	2.05	JUC10011390.hg.1	SORBS1
TC10001550.hg.1	-2.01	PSR10019561.hg.1	SORBS1
TC10001550.hg.1	-2.02	PSR10019528.hg.1	SORBS1
TC10001550.hg.1	-2.11	PSR10019509.hg.1	SORBS1
TC10001550.hg.1	-2.11	JUC10011415.hg.1	SORBS1
TC10001550.hg.1	-2.26	JUC10011423.hg.1	SORBS1
TC10001550.hg.1	-2.3	PSR10019542.hg.1	SORBS1
TC10001550.hg.1	-2.52	JUC10011401.hg.1	SORBS1
TC10001550.hg.1	-2.55	JUC10011367.hg.1	SORBS1
TC10001550.hg.1	-2.98	PSR10019504.hg.1	SORBS1
TC10001550.hg.1	-3.02	JUC10011379.hg.1	SORBS1
TC12000237.hg.1	2.84	PSR12002922.hg.1	CMAS
TC12000237.hg.1	2.36	JUC12001419.hg.1	CMAS
TC12003294.hg.1	2.84	JUC12020620.hg.1	HOXC9
TC12003294.hg.1	2.76	JUC12020619.hg.1	HOXC9
TC12003294.hg.1	2.2	PSR12005866.hg.1	HOXC9

TC17001192.hg.1	2.84	JUC17009272.hg.1	SREBF1
TC17001192.hg.1	2.01	JUC17009274.hg.1	SREBF1
TC17001192.hg.1	-2.08	PSR17016237.hg.1	SREBF1
TC17001192.hg.1	-2.43	JUC17009280.hg.1	SREBF1
TC17001192.hg.1	-2.5	PSR17016232.hg.1	SREBF1
TC17001192.hg.1	-2.5	PSR17016246.hg.1	SREBF1
TC17001192.hg.1	-2.61	JUC17009282.hg.1	SREBF1
TC17001192.hg.1	-2.72	JUC17009270.hg.1	SREBF1
TC17001192.hg.1	-2.82	JUC17009287.hg.1	SREBF1
TC17001192.hg.1	-5.41	PSR17016234.hg.1	SREBF1
TC20000592.hg.1	2.84	JUC20004460.hg.1	PCNA
TC20000592.hg.1	2.38	PSR20008655.hg.1	PCNA
TC01003750.hg.1	2.83	JUC01030027.hg.1	ELK4, SLC45
TC01003750.hg.1	2.31	JUC01030030.hg.1	ELK4, SLC45
TC01003750.hg.1	2.25	PSR01057516.hg.1	ELK4, SLC45
TC01003750.hg.1	2.09	PSR01057521.hg.1	ELK4, SLC45
TC01003750.hg.1	2.09	JUC01030039.hg.1	ELK4, SLC45
TC02001451.hg.1	2.83	JUC02012209.hg.1	FAM132B
TC02001451.hg.1	2.13	PSR02023367.hg.1	FAM132B
TC02001451.hg.1	2.11	PSR02023377.hg.1	FAM132B
TC02001451.hg.1	2.09	JUC02012203.hg.1	FAM132B
TC02001451.hg.1	2.01	PSR02023376.hg.1	FAM132B
TC03001380.hg.1	2.83	JUC03011940.hg.1	SHISA5
TC03001380.hg.1	2.13	PSR03023927.hg.1	SHISA5
TC03001380.hg.1	2.08	JUC03011936.hg.1	SHISA5
TC05001406.hg.1	2.83	JUC05010143.hg.1	ADAMTS6
TC05001406.hg.1	2.28	JUC05010138.hg.1	ADAMTS6
TC05001406.hg.1	-2.3	JUC05010148.hg.1	ADAMTS6
TC05001406.hg.1	-2.31	JUC05010141.hg.1	ADAMTS6
TC05001406.hg.1	-2.67	PSR05019631.hg.1	ADAMTS6
TC05001406.hg.1	-3.46	JUC05010142.hg.1	ADAMTS6
TC05001406.hg.1	-4.11	JUC05010136.hg.1	ADAMTS6
TC06002035.hg.1	2.83	JUC06012567.hg.1	HDAC2
TC06002035.hg.1	2.4	PSR06025491.hg.1	HDAC2
TC06002035.hg.1	2.32	JUC06012582.hg.1	HDAC2
TC06002035.hg.1	2	PSR06025505.hg.1	HDAC2
TC08001030.hg.1	2.83	JUC08006799.hg.1	GFRA2
TC08001030.hg.1	2.06	JUC08006793.hg.1	GFRA2
TC08001030.hg.1	-2.19	PSR08013068.hg.1	GFRA2
TC08001030.hg.1	-2.25	JUC08006794.hg.1	GFRA2
TC08001030.hg.1	-2.28	JUC08006800.hg.1	GFRA2
TC08001030.hg.1	-2.32	JUC08006801.hg.1	GFRA2
TC08001030.hg.1	-2.36	PSR08013085.hg.1	GFRA2
TC08001030.hg.1	-2.59	JUC08006788.hg.1	GFRA2
TC08001030.hg.1	-2.6	JUC08006798.hg.1	GFRA2
TC08001030.hg.1	-2.78	PSR08013066.hg.1	GFRA2
TC08001030.hg.1	-2.99	PSR08013070.hg.1	GFRA2
TC08001030.hg.1	-3	JUC08006796.hg.1	GFRA2
TC08001030.hg.1	-3.05	PSR08013094.hg.1	GFRA2
TC08001030.hg.1	-3.09	PSR08013069.hg.1	GFRA2
TC08001030.hg.1	-3.16	PSR08013087.hg.1	GFRA2
TC08001030.hg.1	-3.41	PSR08013091.hg.1	GFRA2
TC08001030.hg.1	-3.51	JUC08006804.hg.1	GFRA2
TC08001030.hg.1	-3.6	JUC08006803.hg.1	GFRA2
TC08001030.hg.1	-3.8	JUC08006792.hg.1	GFRA2
TC08001030.hg.1	-3.93	PSR08013099.hg.1	GFRA2
TC08001030.hg.1	-4.03	JUC08006805.hg.1	GFRA2
TC08001030.hg.1	-4.14	PSR08013097.hg.1	GFRA2

TC08001030.hg.1	-4.2	PSR08013086.hg.1	GFRA2
TC08001030.hg.1	-4.99	PSR08013084.hg.1	GFRA2
TC09000207.hg.1	2.83	JUC09001297.hg.1	MELK
TC09000207.hg.1	2.71	PSR09002374.hg.1	MELK
TC09000207.hg.1	2.48	JUC09001301.hg.1	MELK
TC09000207.hg.1	2.43	JUC09001300.hg.1	MELK
TC09000207.hg.1	2.29	PSR09002403.hg.1	MELK
TC09000207.hg.1	2.01	JUC09001298.hg.1	MELK
TC09000207.hg.1	-2.04	JUC09001275.hg.1	MELK
TC6_cox_hap2000	2.83	PSR6_cox_hap2003(	AGPAT1
TC6_cox_hap2000	2.63	JUC6_cox_hap20012	AGPAT1
TC6_cox_hap2000	2.45	PSR6_cox_hap2003(	AGPAT1
TC6_cox_hap2000	-3.28	JUC6_cox_hap20012	AGPAT1
TC6_mcf_hap5000	2.83	PSR6_mcf_hap5003:	AGPAT1
TC6_mcf_hap5000	2.63	JUC6_mcf_hap5001(	AGPAT1
TC6_mcf_hap5000	2.45	PSR6_mcf_hap5003:	AGPAT1
TC6_mcf_hap5000	-3.28	JUC6_mcf_hap5001(	AGPAT1
TC6_qbl_hap6000'	2.83	PSR6_qbl_hap60034	AGPAT1
TC6_qbl_hap6000'	2.63	JUC6_qbl_hap60012	AGPAT1
TC6_qbl_hap6000'	2.45	PSR6_qbl_hap60034	AGPAT1
TC6_qbl_hap6000'	-3.28	JUC6_qbl_hap60012	AGPAT1
TC01002284.hg.1	2.82	JUC01018675.hg.1	MFAP2
TC01002284.hg.1	-2.46	JUC01018674.hg.1	MFAP2
TC01002284.hg.1	-2.72	PSR01034768.hg.1	MFAP2
TC01002284.hg.1	-3.29	PSR01034753.hg.1	MFAP2
TC01002284.hg.1	-4.62	PSR01034772.hg.1	MFAP2
TC01002284.hg.1	-5.9	PSR01034763.hg.1	MFAP2
TC01002284.hg.1	-6.52	PSR01034766.hg.1	MFAP2
TC01002284.hg.1	-7.63	PSR01034761.hg.1	MFAP2
TC01002284.hg.1	-8.18	PSR01034769.hg.1	MFAP2
TC01002284.hg.1	-8.49	PSR01034771.hg.1	MFAP2
TC01002284.hg.1	-9.24	PSR01034757.hg.1	MFAP2
TC02001649.hg.1	2.82	PSR02026332.hg.1	ITSN2
TC02001649.hg.1	2.66	JUC02013713.hg.1	ITSN2
TC02001649.hg.1	2.13	PSR02026322.hg.1	ITSN2
TC02001649.hg.1	2.13	JUC02013702.hg.1	ITSN2
TC02001649.hg.1	2.07	JUC02013720.hg.1	ITSN2
TC02001649.hg.1	2.06	JUC02013705.hg.1	ITSN2
TC02001649.hg.1	2.01	PSR02026327.hg.1	ITSN2
TC02001649.hg.1	2	JUC02013715.hg.1	ITSN2
TC02001649.hg.1	-2.22	JUC02013738.hg.1	ITSN2
TC03000309.hg.1	2.82	JUC03003212.hg.1	SEMA3B
TC03000309.hg.1	2.49	JUC03003195.hg.1	SEMA3B
TC03000309.hg.1	2.48	JUC03003208.hg.1	SEMA3B
TC03000309.hg.1	2.33	PSR03006595.hg.1	SEMA3B
TC03000309.hg.1	2.3	PSR03006589.hg.1	SEMA3B
TC03000309.hg.1	2.04	JUC03003198.hg.1	SEMA3B
TC03000309.hg.1	-2.03	PSR03006550.hg.1	SEMA3B
TC03000309.hg.1	-2.04	PSR03006545.hg.1	SEMA3B
TC03000309.hg.1	-2.13	JUC03003213.hg.1	SEMA3B
TC03000309.hg.1	-2.76	PSR03006573.hg.1	SEMA3B
TC03000309.hg.1	-5.44	JUC03003190.hg.1	SEMA3B
TC03000334.hg.1	2.82	PSR03007281.hg.1	PHF7
TC03000334.hg.1	2.79	PSR03007238.hg.1	PHF7
TC03000334.hg.1	2.52	JUC03003499.hg.1	PHF7
TC03000334.hg.1	2.17	PSR03007237.hg.1	PHF7
TC03000334.hg.1	2.14	PSR03007282.hg.1	PHF7
TC03001149.hg.1	2.82	JUC03009932.hg.1	CAMK1

TC03001149.hg.1	2.23	PSR03019813.hg.1	CAMK1
TC03001149.hg.1	2.09	PSR03019827.hg.1	CAMK1
TC03001149.hg.1	2.03	PSR03019812.hg.1	CAMK1
TC03001149.hg.1	-2.18	JUC03009934.hg.1	CAMK1
TC03001514.hg.1	2.82	JUC03013500.hg.1	LOC1005069
TC03001514.hg.1	2.27	PSR03027559.hg.1	LOC1005069
TC03001514.hg.1	2.24	JUC03013489.hg.1	LOC1005069
TC03001514.hg.1	2.15	PSR03027558.hg.1	LOC1005069
TC03001514.hg.1	2.08	JUC03013497.hg.1	LOC1005069
TC04000014.hg.1	2.82	JUC04000138.hg.1	TMEM175
TC04000014.hg.1	2.65	PSR04000331.hg.1	TMEM175
TC06004104.hg.1	2.82	JUC06021389.hg.1	TIAM2, LOC1
TC06004104.hg.1	-2.14	PSR06013240.hg.1	TIAM2, LOC1
TC06004104.hg.1	-2.16	JUC06021395.hg.1	TIAM2, LOC1
TC06004104.hg.1	-2.33	JUC06021406.hg.1	TIAM2, LOC1
TC06004104.hg.1	-2.5	JUC06021421.hg.1	TIAM2, LOC1
TC07003393.hg.1	2.82	JUC07021846.hg.1	DBNL
TC07003393.hg.1	-2.92	PSR07004186.hg.1	DBNL
TC07003393.hg.1	-3.3	JUC07021849.hg.1	DBNL
TC0X000490.hg.1	2.82	JUC0X003225.hg.1	BHLHB9
TC0X000490.hg.1	2.43	JUC0X003227.hg.1	BHLHB9
TC0X000490.hg.1	2.23	PSR0X006501.hg.1	BHLHB9
TC0X000754.hg.1	2.82	JUC0X004984.hg.1	SLC6A8
TC0X000754.hg.1	2.62	PSR0X009843.hg.1	SLC6A8
TC0X000754.hg.1	2.59	PSR0X009831.hg.1	SLC6A8
TC0X000754.hg.1	2.31	JUC0X004973.hg.1	SLC6A8
TC0X000754.hg.1	2.11	PSR0X009839.hg.1	SLC6A8
TC0X000754.hg.1	2.06	PSR0X009832.hg.1	SLC6A8
TC0X000754.hg.1	2	PSR0X009842.hg.1	SLC6A8
TC10001061.hg.1	2.82	PSR10013317.hg.1	NMT2
TC10001061.hg.1	2.17	JUC10007664.hg.1	NMT2
TC10001061.hg.1	2.07	PSR10013327.hg.1	NMT2
TC10001061.hg.1	2.05	PSR10013298.hg.1	NMT2
TC12002032.hg.1	2.82	PSR12026661.hg.1	CIT, MIR1178
TC12002032.hg.1	2.65	JUC12014924.hg.1	CIT, MIR1178
TC12002032.hg.1	2.53	JUC12014908.hg.1	CIT, MIR1178
TC12002032.hg.1	2.39	JUC12014926.hg.1	CIT, MIR1178
TC12002032.hg.1	2.29	JUC12014904.hg.1	CIT, MIR1178
TC12002032.hg.1	2.24	PSR12026671.hg.1	CIT, MIR1178
TC12002032.hg.1	2.2	PSR12026652.hg.1	CIT, MIR1178
TC12002032.hg.1	2.17	JUC12014941.hg.1	CIT, MIR1178
TC12002032.hg.1	2.16	PSR12026610.hg.1	CIT, MIR1178
TC12002032.hg.1	2.04	PSR12026627.hg.1	CIT, MIR1178
TC12002032.hg.1	-2.03	JUC12014919.hg.1	CIT, MIR1178
TC12002032.hg.1	-2.11	JUC12014927.hg.1	CIT, MIR1178
TC12002032.hg.1	-2.3	PSR12026619.hg.1	CIT, MIR1178
TC12002032.hg.1	-2.41	JUC12014940.hg.1	CIT, MIR1178
TC12002032.hg.1	-2.48	JUC12014949.hg.1	CIT, MIR1178
TC12002032.hg.1	-2.49	PSR12026636.hg.1	CIT, MIR1178
TC12002032.hg.1	-2.52	PSR12026614.hg.1	CIT, MIR1178
TC12002032.hg.1	-2.56	JUC12014917.hg.1	CIT, MIR1178
TC12002032.hg.1	-2.81	JUC12014932.hg.1	CIT, MIR1178
TC12002032.hg.1	-2.96	JUC12014938.hg.1	CIT, MIR1178
TC12002032.hg.1	-3.03	PSR12026618.hg.1	CIT, MIR1178
TC12002032.hg.1	-3.03	JUC12014923.hg.1	CIT, MIR1178
TC12002032.hg.1	-3.52	JUC12014930.hg.1	CIT, MIR1178
TC12002032.hg.1	-3.59	JUC12014947.hg.1	CIT, MIR1178
TC13000120.hg.1	2.82	PSR13001294.hg.1	NBEA

TC13000120.hg.1	2.61	JUC13000839.hg.1	NBEA
TC13000120.hg.1	2.56	JUC13000814.hg.1	NBEA
TC13000120.hg.1	2.49	JUC13000860.hg.1	NBEA
TC13000120.hg.1	2.46	JUC13000827.hg.1	NBEA
TC13000120.hg.1	2.33	JUC13000852.hg.1	NBEA
TC13000120.hg.1	2.19	PSR13001297.hg.1	NBEA
TC13000120.hg.1	2.12	JUC13000828.hg.1	NBEA
TC13000120.hg.1	2.11	PSR13001293.hg.1	NBEA
TC13000120.hg.1	2.06	PSR13001308.hg.1	NBEA
TC13000120.hg.1	2.04	JUC13000857.hg.1	NBEA
TC13000120.hg.1	2.03	JUC13000864.hg.1	NBEA
TC13000120.hg.1	-2.05	JUC13000812.hg.1	NBEA
TC13000120.hg.1	-2.07	JUC13000867.hg.1	NBEA
TC13000120.hg.1	-2.17	JUC13000861.hg.1	NBEA
TC13000120.hg.1	-3.47	JUC13000843.hg.1	NBEA
TC17000657.hg.1	2.82	JUC17004565.hg.1	SGCA
TC17000657.hg.1	2.28	PSR17008260.hg.1	SGCA
TC17000657.hg.1	-2.77	JUC17004567.hg.1	SGCA
TC17000657.hg.1	-3.31	JUC17004557.hg.1	SGCA
TC17000657.hg.1	-3.87	JUC17004571.hg.1	SGCA
TC01001682.hg.1	2.81	PSR01026093.hg.1	PPFIA4
TC01001682.hg.1	2.63	JUC01013989.hg.1	PPFIA4
TC01001682.hg.1	2.6	PSR01026092.hg.1	PPFIA4
TC01001682.hg.1	2.55	PSR01026088.hg.1	PPFIA4
TC01001682.hg.1	2.44	PSR01026132.hg.1	PPFIA4
TC01001682.hg.1	2.42	JUC01013972.hg.1	PPFIA4
TC01001682.hg.1	2.32	PSR01026091.hg.1	PPFIA4
TC01001682.hg.1	2.2	JUC01013980.hg.1	PPFIA4
TC01001682.hg.1	2.18	PSR01026099.hg.1	PPFIA4
TC01001682.hg.1	2.17	JUC01013982.hg.1	PPFIA4
TC01001682.hg.1	2.16	JUC01013971.hg.1	PPFIA4
TC01001682.hg.1	2.12	JUC01013985.hg.1	PPFIA4
TC01001682.hg.1	2.06	PSR01026126.hg.1	PPFIA4
TC01001682.hg.1	2.06	JUC01013983.hg.1	PPFIA4
TC01001682.hg.1	-2.07	PSR01026115.hg.1	PPFIA4
TC01001682.hg.1	-2.18	PSR01026130.hg.1	PPFIA4
TC01001682.hg.1	-2.45	PSR01026119.hg.1	PPFIA4
TC01001682.hg.1	-2.85	JUC01013979.hg.1	PPFIA4
TC01001682.hg.1	-2.87	JUC01013965.hg.1	PPFIA4
TC01001682.hg.1	-3.68	JUC01013986.hg.1	PPFIA4
TC01001682.hg.1	-4.03	JUC01013967.hg.1	PPFIA4
TC01001682.hg.1	-4.37	PSR01026129.hg.1	PPFIA4
TC01001682.hg.1	-4.93	JUC01013988.hg.1	PPFIA4
TC01001682.hg.1	-6.57	JUC01014001.hg.1	PPFIA4
TC01001682.hg.1	-6.61	PSR01026111.hg.1	PPFIA4
TC02000192.hg.1	2.81	PSR02002785.hg.1	CLIP4
TC02000192.hg.1	-2.01	JUC02001411.hg.1	CLIP4
TC04000324.hg.1	2.81	PSR04004564.hg.1	EXOC1
TC04000324.hg.1	2.47	JUC04002216.hg.1	EXOC1
TC04000324.hg.1	-2.38	JUC04002217.hg.1	EXOC1
TC04000324.hg.1	-3.61	JUC04002215.hg.1	EXOC1
TC05001621.hg.1	2.81	PSR05022126.hg.1	RIOK2
TC05001621.hg.1	2.18	JUC05011418.hg.1	RIOK2
TC05001700.hg.1	2.81	JUC05011925.hg.1	SEMA6A
TC05001700.hg.1	2.67	JUC05011914.hg.1	SEMA6A
TC05001700.hg.1	2.15	JUC05011911.hg.1	SEMA6A
TC05001700.hg.1	2.1	JUC05011901.hg.1	SEMA6A
TC05001700.hg.1	-2.07	JUC05011908.hg.1	SEMA6A

TC05001700.hg.1	-2.13	PSR05023060.hg.1	SEMA6A
TC05001995.hg.1	2.81	PSR05027744.hg.1	EBF1
TC05001995.hg.1	-2.26	PSR05027724.hg.1	EBF1
TC05001995.hg.1	-3.11	JUC05014124.hg.1	EBF1
TC05001995.hg.1	-3.28	JUC05014152.hg.1	EBF1
TC06001855.hg.1	2.81	JUC06010909.hg.1	COL9A1
TC06001855.hg.1	2.78	JUC06010882.hg.1	COL9A1
TC06001855.hg.1	2.71	JUC06010915.hg.1	COL9A1
TC06001855.hg.1	2.48	JUC06010890.hg.1	COL9A1
TC06001855.hg.1	2.23	PSR06022520.hg.1	COL9A1
TC08000918.hg.1	2.81	PSR08011982.hg.1	ZNF705G
TC08000918.hg.1	-2.13	PSR08011976.hg.1	ZNF705G
TC08000918.hg.1	-4.05	JUC08006233.hg.1	ZNF705G
TC09001036.hg.1	2.81	JUC09007378.hg.1	FAM219A
TC09001036.hg.1	2.52	JUC09007374.hg.1	FAM219A
TC09001036.hg.1	2.29	JUC09007377.hg.1	FAM219A
TC09001036.hg.1	2.03	PSR09013580.hg.1	FAM219A
TC0X000379.hg.1	2.81	PSR0X004929.hg.1	DLG3
TC0X000379.hg.1	2.62	JUC0X002429.hg.1	DLG3
TC0X000379.hg.1	2.55	JUC0X002425.hg.1	DLG3
TC0X000379.hg.1	2.25	JUC0X002413.hg.1	DLG3
TC0X000379.hg.1	2.16	PSR0X004933.hg.1	DLG3
TC0X000379.hg.1	2.1	PSR0X004923.hg.1	DLG3
TC0X000379.hg.1	2.05	PSR0X004930.hg.1	DLG3
TC0X000379.hg.1	-2.12	JUC0X002432.hg.1	DLG3
TC0X000379.hg.1	-3.38	JUC0X002436.hg.1	DLG3
TC11003468.hg.1	2.81	JUC11019285.hg.1	TMEM25
TC11003468.hg.1	2.47	PSR11013427.hg.1	TMEM25
TC11003468.hg.1	2.38	PSR11013423.hg.1	TMEM25
TC11003468.hg.1	2.23	PSR11013415.hg.1	TMEM25
TC11003468.hg.1	2.15	PSR11013433.hg.1	TMEM25
TC11003468.hg.1	2.13	PSR11013424.hg.1	TMEM25
TC11003468.hg.1	2.03	PSR11013425.hg.1	TMEM25
TC11003468.hg.1	-2.28	PSR11013449.hg.1	TMEM25
TC11003468.hg.1	-3.19	JUC11019295.hg.1	TMEM25
TC12001536.hg.1	2.81	PSR12020179.hg.1	KRT4
TC12001536.hg.1	2.19	JUC12011217.hg.1	KRT4
TC13000825.hg.1	2.81	JUC13005445.hg.1	DOCK9
TC13000825.hg.1	-2.16	PSR13009236.hg.1	DOCK9
TC13000825.hg.1	-2.27	JUC13005433.hg.1	DOCK9
TC13000825.hg.1	-2.52	JUC13005424.hg.1	DOCK9
TC13000825.hg.1	-2.73	JUC13005461.hg.1	DOCK9
TC13000825.hg.1	-2.78	JUC13005409.hg.1	DOCK9
TC13000854.hg.1	2.81	JUC13005643.hg.1	TEX30
TC13000854.hg.1	2.72	PSR13009578.hg.1	TEX30
TC13000854.hg.1	2.51	JUC13005638.hg.1	TEX30
TC13000854.hg.1	2.03	JUC13005644.hg.1	TEX30
TC15001583.hg.1	2.81	JUC15008267.hg.1	LCTL
TC15001583.hg.1	2.27	JUC15008270.hg.1	LCTL
TC15001583.hg.1	2.26	JUC15008271.hg.1	LCTL
TC15001583.hg.1	2.03	PSR15015234.hg.1	LCTL
TC15001583.hg.1	-2.04	JUC15008275.hg.1	LCTL
TC15001583.hg.1	-2.99	JUC15008268.hg.1	LCTL
TC16002046.hg.1	2.81	JUC16013340.hg.1	ITFG3
TC16002046.hg.1	2.41	JUC16013334.hg.1	ITFG3
TC16002046.hg.1	2.3	PSR16000166.hg.1	ITFG3
TC16002046.hg.1	2.1	JUC16013344.hg.1	ITFG3
TC16002046.hg.1	2.09	JUC16013351.hg.1	ITFG3

TC16002046.hg.1	2.04	JUC16013346.hg.1	ITFG3
TC16002046.hg.1	2.03	PSR16000128.hg.1	ITFG3
TC16002046.hg.1	-2.17	JUC16013361.hg.1	ITFG3
TC01001791.hg.1	2.8	PSR01027997.hg.1	CENPF
TC01001791.hg.1	2.35	JUC01015096.hg.1	CENPF
TC01001791.hg.1	2.16	PSR01028008.hg.1	CENPF
TC01001791.hg.1	2.09	PSR01028016.hg.1	CENPF
TC01001791.hg.1	-2.08	JUC01015081.hg.1	CENPF
TC01001791.hg.1	-2.15	PSR01028021.hg.1	CENPF
TC02000986.hg.1	2.8	PSR02014452.hg.1	CSRNP3
TC02000986.hg.1	2.07	JUC02007727.hg.1	CSRNP3
TC02000986.hg.1	-2	JUC02007733.hg.1	CSRNP3
TC02002408.hg.1	2.8	PSR02037939.hg.1	ORC4
TC02002408.hg.1	2.62	JUC02019710.hg.1	ORC4
TC02002408.hg.1	2.1	PSR02037912.hg.1	ORC4
TC02002408.hg.1	-2	JUC02019700.hg.1	ORC4
TC02002408.hg.1	-2.1	JUC02019695.hg.1	ORC4
TC02002408.hg.1	-2.21	PSR02037903.hg.1	ORC4
TC02002408.hg.1	-2.49	JUC02019717.hg.1	ORC4
TC03003385.hg.1	2.8	PSR03025121.hg.1	NICN1
TC03003385.hg.1	-2.05	JUC03024253.hg.1	NICN1
TC03003385.hg.1	-2.09	PSR03025114.hg.1	NICN1
TC03003385.hg.1	-2.09	JUC03024254.hg.1	NICN1
TC03003385.hg.1	-2.12	PSR03025110.hg.1	NICN1
TC03003385.hg.1	-2.19	PSR03025111.hg.1	NICN1
TC03003385.hg.1	-2.24	PSR03025113.hg.1	NICN1
TC05001365.hg.1	2.8	JUC05009801.hg.1	SLC38A9
TC05001365.hg.1	-2.08	PSR05019070.hg.1	SLC38A9
TC05001365.hg.1	-2.22	JUC05009794.hg.1	SLC38A9
TC05001365.hg.1	-2.76	PSR05019050.hg.1	SLC38A9
TC05001365.hg.1	-2.99	JUC05009827.hg.1	SLC38A9
TC07000131.hg.1	2.8	PSR07001994.hg.1	SP4
TC07000131.hg.1	2.72	JUC07000939.hg.1	SP4
TC07000131.hg.1	2.46	PSR07001991.hg.1	SP4
TC07000131.hg.1	2.41	JUC07000932.hg.1	SP4
TC07000131.hg.1	2.41	JUC07000938.hg.1	SP4
TC07000131.hg.1	2.01	PSR07002006.hg.1	SP4
TC07000255.hg.1	2.8	JUC07001882.hg.1	C7orf10
TC07000255.hg.1	2.23	JUC07001884.hg.1	C7orf10
TC07000255.hg.1	2.02	PSR07003924.hg.1	C7orf10
TC07000255.hg.1	-2.3	PSR07003925.hg.1	C7orf10
TC07000255.hg.1	-2.44	JUC07001885.hg.1	C7orf10
TC07000255.hg.1	-2.45	PSR07003923.hg.1	C7orf10
TC07000255.hg.1	-2.5	PSR07003918.hg.1	C7orf10
TC07000255.hg.1	-2.63	PSR07003922.hg.1	C7orf10
TC07000255.hg.1	-2.89	JUC07001887.hg.1	C7orf10
TC07000255.hg.1	-2.95	JUC07001868.hg.1	C7orf10
TC07000255.hg.1	-3.27	JUC07001880.hg.1	C7orf10
TC07000255.hg.1	-3.37	PSR07003913.hg.1	C7orf10
TC07000255.hg.1	-3.39	PSR07003905.hg.1	C7orf10
TC07000255.hg.1	-3.86	PSR07003904.hg.1	C7orf10
TC07000255.hg.1	-4.05	JUC07001867.hg.1	C7orf10
TC07000255.hg.1	-5.4	JUC07001874.hg.1	C7orf10
TC07000255.hg.1	-5.42	JUC07001871.hg.1	C7orf10
TC07000255.hg.1	-8.86	JUC07001866.hg.1	C7orf10
TC07000715.hg.1	2.8	JUC07005280.hg.1	MDFIC
TC07000715.hg.1	-2.35	PSR07011271.hg.1	MDFIC
TC07000715.hg.1	-3.67	PSR07011273.hg.1	MDFIC

TC07001761.hg.1	2.8	JUC07013543.hg.1	DOCK4
TC07001761.hg.1	2.1	JUC07013559.hg.1	DOCK4
TC07001761.hg.1	2	JUC07013544.hg.1	DOCK4
TC07001761.hg.1	-2	PSR07027550.hg.1	DOCK4
TC07001761.hg.1	-2.05	PSR07027583.hg.1	DOCK4
TC07001761.hg.1	-2.46	JUC07013561.hg.1	DOCK4
TC07001761.hg.1	-2.47	JUC07013525.hg.1	DOCK4
TC09001193.hg.1	2.8	JUC09008188.hg.1	APBA1, LOC
TC09001193.hg.1	-2.28	PSR09015260.hg.1	APBA1, LOC
TC09001193.hg.1	-4.51	JUC09008191.hg.1	APBA1, LOC
TC0Y000187.hg.1	2.8	JUC0Y001194.hg.1	
TC0Y000187.hg.1	2.4	PSR0Y001876.hg.1	
TC10001329.hg.1	2.8	JUC10009816.hg.1	JMJD1C
TC10001329.hg.1	2.07	JUC10009823.hg.1	JMJD1C
TC10001329.hg.1	-2.04	PSR10016828.hg.1	JMJD1C
TC10001329.hg.1	-2.04	JUC10009804.hg.1	JMJD1C
TC10001329.hg.1	-2.11	JUC10009827.hg.1	JMJD1C
TC10001329.hg.1	-3.02	JUC10009813.hg.1	JMJD1C
TC10001416.hg.1	2.8	PSR10017940.hg.1	AGAP5, BMS
TC10001416.hg.1	2.27	JUC10010448.hg.1	AGAP5, BMS
TC10001416.hg.1	-2.03	JUC10010459.hg.1	AGAP5, BMS
TC13000681.hg.1	2.8	JUC13004572.hg.1	DHRS12
TC13000681.hg.1	2.04	PSR13007885.hg.1	DHRS12
TC16000484.hg.1	2.8	JUC16003820.hg.1	RSPRY1
TC16000484.hg.1	2.21	PSR16006748.hg.1	RSPRY1
TC16002098.hg.1	2.8	JUC16014080.hg.1	NPIPL3
TC16002098.hg.1	-2.81	JUC16014073.hg.1	NPIPL3
TC16002098.hg.1	-3.2	PSR16013577.hg.1	NPIPL3
TC17000026.hg.1	2.8	JUC17000163.hg.1	RAP1GAP2
TC17000026.hg.1	2.32	PSR17000320.hg.1	RAP1GAP2
TC17000026.hg.1	-2.04	JUC17000166.hg.1	RAP1GAP2
TC17000026.hg.1	-2.09	JUC17000157.hg.1	RAP1GAP2
TC17000026.hg.1	-2.41	JUC17000159.hg.1	RAP1GAP2
TC17000026.hg.1	-2.76	JUC17000164.hg.1	RAP1GAP2
TC17000026.hg.1	-3.13	JUC17000158.hg.1	RAP1GAP2
TC17000026.hg.1	-3.16	JUC17000170.hg.1	RAP1GAP2
TC17000026.hg.1	-3.33	PSR17000287.hg.1	RAP1GAP2
TC17000026.hg.1	-4.95	JUC17000173.hg.1	RAP1GAP2
TC17000085.hg.1	2.8	PSR17001080.hg.1	ACADVL
TC17000085.hg.1	2.36	JUC17000669.hg.1	ACADVL
TC17000667.hg.1	2.8	PSR17008418.hg.1	ACSF2
TC17000667.hg.1	2.39	JUC17004642.hg.1	ACSF2
TC17000667.hg.1	2.37	PSR17008413.hg.1	ACSF2
TC17000667.hg.1	2.31	PSR17008449.hg.1	ACSF2
TC17000667.hg.1	2.21	PSR17008425.hg.1	ACSF2
TC17000667.hg.1	2.07	PSR17008429.hg.1	ACSF2
TC17000667.hg.1	2.06	PSR17008428.hg.1	ACSF2
TC19000627.hg.1	2.8	PSR19008695.hg.1	BCL3
TC19000627.hg.1	2.79	PSR19008697.hg.1	BCL3
TC19000627.hg.1	2.73	PSR19008700.hg.1	BCL3
TC19000627.hg.1	2.64	PSR19008716.hg.1	BCL3
TC19000627.hg.1	2.54	PSR19008717.hg.1	BCL3
TC19000627.hg.1	2.27	PSR19008719.hg.1	BCL3
TC19000627.hg.1	2.16	JUC19005200.hg.1	BCL3
TC06004098.hg.1	2.8	JUC06021307.hg.1	PHACTR2
TC06004098.hg.1	2	PSR06012348.hg.1	PHACTR2
TC01002497.hg.1	2.79	JUC01020816.hg.1	KIAA0319L
TC01002497.hg.1	2.63	JUC01020810.hg.1	KIAA0319L

TC01002497.hg.1	2.45	PSR01038771.hg.1	KIAA0319L
TC01002497.hg.1	2.43	PSR01038758.hg.1	KIAA0319L
TC01002497.hg.1	2.38	JUC01020804.hg.1	KIAA0319L
TC01002497.hg.1	2.35	PSR01038768.hg.1	KIAA0319L
TC01002497.hg.1	2.34	PSR01038711.hg.1	KIAA0319L
TC01002497.hg.1	2.22	PSR01038756.hg.1	KIAA0319L
TC01002497.hg.1	2.14	PSR01038757.hg.1	KIAA0319L
TC01002497.hg.1	2.08	PSR01038754.hg.1	KIAA0319L
TC01002497.hg.1	-2.04	JUC01020812.hg.1	KIAA0319L
TC01002497.hg.1	-2.48	JUC01020813.hg.1	KIAA0319L
TC01002497.hg.1	-2.87	JUC01020800.hg.1	KIAA0319L
TC01006302.hg.1	2.79	PSR01048354.hg.1	LOC1005060
TC01006302.hg.1	2	JUC01039575.hg.1	LOC1005060
TC03002091.hg.1	2.79	PSR03037117.hg.1	RPL39L
TC03002091.hg.1	2.04	JUC03018357.hg.1	RPL39L
TC04001219.hg.1	2.79	JUC04008889.hg.1	AASDH
TC04001219.hg.1	2.31	PSR04016656.hg.1	AASDH
TC04001219.hg.1	2	JUC04008875.hg.1	AASDH
TC05002010.hg.1	2.79	PSR05027941.hg.1	SLU7
TC05002010.hg.1	-3.5	JUC05014248.hg.1	SLU7
TC08000200.hg.1	2.79	PSR08002832.hg.1	BNIP3L
TC08000200.hg.1	2.63	PSR08002833.hg.1	BNIP3L
TC08000200.hg.1	2.07	JUC08001386.hg.1	BNIP3L
TC08000200.hg.1	2.05	JUC08001379.hg.1	BNIP3L
TC08000200.hg.1	-2	PSR08002852.hg.1	BNIP3L
TC08000200.hg.1	-2.29	PSR08002848.hg.1	BNIP3L
TC08000277.hg.1	2.79	JUC08001889.hg.1	ERLIN2
TC08000277.hg.1	2.43	JUC08001891.hg.1	ERLIN2
TC08000277.hg.1	2.35	JUC08001896.hg.1	ERLIN2
TC08000277.hg.1	2.14	JUC08001898.hg.1	ERLIN2
TC08000277.hg.1	2.06	PSR08003833.hg.1	ERLIN2
TC08000277.hg.1	2	PSR08003809.hg.1	ERLIN2
TC08000602.hg.1	2.79	JUC08004217.hg.1	POP1
TC08000602.hg.1	2.07	PSR08008351.hg.1	POP1
TC12001389.hg.1	2.79	JUC12010014.hg.1	CPNE8
TC12001389.hg.1	2.7	JUC12010015.hg.1	CPNE8
TC12001389.hg.1	2.15	JUC12010011.hg.1	CPNE8
TC12001389.hg.1	-2.08	PSR12017988.hg.1	CPNE8
TC12001389.hg.1	-2.36	JUC12010013.hg.1	CPNE8
TC12001941.hg.1	2.79	PSR12025277.hg.1	SSH1
TC12001941.hg.1	-2.32	JUC12014108.hg.1	SSH1
TC12001941.hg.1	-2.77	PSR12025264.hg.1	SSH1
TC17001753.hg.1	2.79	JUC17013037.hg.1	TUBD1
TC17001753.hg.1	2.45	PSR17023404.hg.1	TUBD1
TC17001753.hg.1	2.07	JUC17013051.hg.1	TUBD1
TC17001753.hg.1	-4.32	PSR17023409.hg.1	TUBD1
TC17002844.hg.1	2.79	JUC17018185.hg.1	SPATA20
TC17002844.hg.1	2.53	JUC17018193.hg.1	SPATA20
TC17002844.hg.1	2.5	PSR17008640.hg.1	SPATA20
TC17002844.hg.1	2.43	PSR17008606.hg.1	SPATA20
TC17002844.hg.1	2.35	JUC17018191.hg.1	SPATA20
TC17002844.hg.1	2.34	PSR17008615.hg.1	SPATA20
TC17002844.hg.1	2.24	PSR17008652.hg.1	SPATA20
TC17002844.hg.1	2.22	PSR17008619.hg.1	SPATA20
TC17002844.hg.1	2.17	PSR17008648.hg.1	SPATA20
TC17002844.hg.1	2.16	JUC17018186.hg.1	SPATA20
TC17002844.hg.1	-2.36	PSR17008643.hg.1	SPATA20
TC17002844.hg.1	-3.04	JUC17018213.hg.1	SPATA20

TC6_apd_hap1000	2.79	PSR6_apd_hap1001	AGPAT1
TC6_apd_hap1000	2.59	JUC6_apd_hap1000	AGPAT1
TC6_apd_hap1000	2.41	PSR6_apd_hap1001	AGPAT1
TC6_apd_hap1000	-3.33	JUC6_apd_hap1000	AGPAT1
TC6_cox_hap2000	2.79	JUC6_cox_hap20021	TNXB, TNXA
TC6_cox_hap2000	-2.07	JUC6_cox_hap20021	TNXB, TNXA
TC6_cox_hap2000	-2.37	PSR6_cox_hap2003	TNXB, TNXA
TC6_dbb_hap3000	2.79	JUC6_dbb_hap3002	TNXB
TC6_dbb_hap3000	-2.07	JUC6_dbb_hap3002	TNXB
TC6_dbb_hap3000	-2.25	PSR6_dbb_hap3003	TNXB
TC6_dbb_hap3000	-2.37	PSR6_dbb_hap3003	TNXB
TC6_ssto_hap700	2.79	PSR6_ssto_hap7003	AGPAT1
TC6_ssto_hap700	2.59	JUC6_ssto_hap7001	AGPAT1
TC6_ssto_hap700	2.41	PSR6_ssto_hap7003	AGPAT1
TC6_ssto_hap700	-3.33	JUC6_ssto_hap7001	AGPAT1
TC01000743.hg.1	2.78	JUC01006242.hg.1	
TC01000743.hg.1	2.14	PSR01011950.hg.1	
TC02002539.hg.1	2.78	PSR02040273.hg.1	GPR155
TC02002539.hg.1	-2.05	JUC02021249.hg.1	GPR155
TC02002539.hg.1	-2.13	PSR02040294.hg.1	GPR155
TC02002539.hg.1	-2.54	JUC02021231.hg.1	GPR155
TC03000534.hg.1	2.78	PSR03009870.hg.1	ALCAM
TC03000534.hg.1	2.02	JUC03004902.hg.1	ALCAM
TC03000751.hg.1	2.78	PSR03013677.hg.1	LOC1005072
TC03000751.hg.1	-2.73	JUC03006907.hg.1	LOC1005072
TC03001202.hg.1	2.78	JUC03010308.hg.1	MRPS25
TC03001202.hg.1	2.56	JUC03010301.hg.1	MRPS25
TC03001202.hg.1	2.02	PSR03020594.hg.1	MRPS25
TC03001202.hg.1	-2.2	PSR03020586.hg.1	MRPS25
TC03001375.hg.1	2.78	JUC03011873.hg.1	NME6
TC03001375.hg.1	2.4	JUC03011866.hg.1	NME6
TC03001375.hg.1	2.16	PSR03023767.hg.1	NME6
TC03001375.hg.1	2.13	JUC03011871.hg.1	NME6
TC03001457.hg.1	2.78	JUC03012866.hg.1	PBRM1
TC03001457.hg.1	2.75	JUC03012903.hg.1	PBRM1
TC03001457.hg.1	2.51	JUC03012872.hg.1	PBRM1
TC03001457.hg.1	2.45	JUC03012906.hg.1	PBRM1
TC03001457.hg.1	2.1	PSR03026372.hg.1	PBRM1
TC03001457.hg.1	-2.14	JUC03012889.hg.1	PBRM1
TC06000870.hg.1	2.78	JUC06004788.hg.1	ARMC2
TC06000870.hg.1	2.13	JUC06004783.hg.1	ARMC2
TC06000870.hg.1	-2.09	JUC06004789.hg.1	ARMC2
TC06000870.hg.1	-2.15	PSR06010180.hg.1	ARMC2
TC10001505.hg.1	2.78	JUC10011047.hg.1	LIPA, LOC10
TC10001505.hg.1	2.78	JUC10011053.hg.1	LIPA, LOC10
TC10001505.hg.1	-2.03	JUC10011049.hg.1	LIPA, LOC10
TC10001505.hg.1	-2.05	PSR10019010.hg.1	LIPA, LOC10
TC10001505.hg.1	-2.13	PSR10018970.hg.1	LIPA, LOC10
TC10001505.hg.1	-2.35	JUC10011046.hg.1	LIPA, LOC10
TC10001505.hg.1	-3.05	JUC10011071.hg.1	LIPA, LOC10
TC11003505.hg.1	2.78	PSR11026921.hg.1	CASP1
TC11003505.hg.1	2.44	JUC11019791.hg.1	CASP1
TC11003505.hg.1	2.22	PSR11026920.hg.1	CASP1
TC11003505.hg.1	-2.66	JUC11019792.hg.1	CASP1
TC12001152.hg.1	2.78	PSR12015286.hg.1	SPSB2
TC12001152.hg.1	-2	JUC12008416.hg.1	SPSB2
TC15000150.hg.1	2.78	JUC15000330.hg.1	WHAMMP2
TC15000150.hg.1	-2.09	PSR15000927.hg.1	WHAMMP2

TC16000493.hg.1	2.78	PSR16006866.hg.1	GPR56
TC16000493.hg.1	2.77	PSR16006860.hg.1	GPR56
TC16000493.hg.1	2.73	PSR16006862.hg.1	GPR56
TC16000493.hg.1	2.53	JUC16003900.hg.1	GPR56
TC16000493.hg.1	2.5	JUC16003897.hg.1	GPR56
TC16000493.hg.1	2.36	JUC16003905.hg.1	GPR56
TC16000493.hg.1	2.35	PSR16006861.hg.1	GPR56
TC16000493.hg.1	2.22	JUC16003893.hg.1	GPR56
TC16000493.hg.1	2.06	PSR16006865.hg.1	GPR56
TC16000493.hg.1	2.02	PSR16006872.hg.1	GPR56
TC16000493.hg.1	-2.09	PSR16006875.hg.1	GPR56
TC16000493.hg.1	-3.34	JUC16003896.hg.1	GPR56
TC17001068.hg.1	2.78	JUC17008168.hg.1	SLC13A5
TC17001068.hg.1	2.41	PSR17014284.hg.1	SLC13A5
TC17001068.hg.1	-2.07	PSR17014293.hg.1	SLC13A5
TC17001068.hg.1	-2.12	PSR17014294.hg.1	SLC13A5
TC17001068.hg.1	-2.15	PSR17014289.hg.1	SLC13A5
TC17001068.hg.1	-2.38	JUC17008167.hg.1	SLC13A5
TC19000488.hg.1	2.78	JUC19003825.hg.1	APLP1
TC19000488.hg.1	2.52	PSR19006490.hg.1	APLP1
TC19000488.hg.1	2.35	PSR19006492.hg.1	APLP1
TC19000488.hg.1	2.31	PSR19006491.hg.1	APLP1
TC19000488.hg.1	2.18	JUC19003840.hg.1	APLP1
TC19000488.hg.1	2.03	JUC19003834.hg.1	APLP1
TC19000488.hg.1	-2.1	PSR19006508.hg.1	APLP1
TC19000488.hg.1	-2.52	PSR19006512.hg.1	APLP1
TC22001425.hg.1	2.78	PSR22005780.hg.1	APOBEC3B
TC22001425.hg.1	2.37	JUC22008590.hg.1	APOBEC3B
TC22001425.hg.1	2.22	JUC22008588.hg.1	APOBEC3B
TC22001425.hg.1	2.04	PSR22005773.hg.1	APOBEC3B
TC22001425.hg.1	-2.5	JUC22008592.hg.1	APOBEC3B
TC22001425.hg.1	-4.14	JUC22008595.hg.1	APOBEC3B
TC22001425.hg.1	-4.35	JUC22008591.hg.1	APOBEC3B
TC22001425.hg.1	-6.13	JUC22008584.hg.1	APOBEC3B
TC01001426.hg.1	2.77	PSR01022306.hg.1	HSD17B7
TC01001426.hg.1	2.2	PSR01022299.hg.1	HSD17B7
TC01001426.hg.1	2.11	JUC01011756.hg.1	HSD17B7
TC01001426.hg.1	2.09	PSR01022282.hg.1	HSD17B7
TC01002528.hg.1	2.77	PSR01039354.hg.1	INPP5B
TC01002528.hg.1	-2.81	JUC01021143.hg.1	INPP5B
TC01002574.hg.1	2.77	JUC01021494.hg.1	LEPRE1
TC01002574.hg.1	2.25	PSR01039984.hg.1	LEPRE1
TC01002574.hg.1	2.16	JUC01021479.hg.1	LEPRE1
TC01002574.hg.1	2.09	PSR01040015.hg.1	LEPRE1
TC01002604.hg.1	2.77	PSR01040483.hg.1	HECTD3
TC01002604.hg.1	2.34	JUC01021738.hg.1	HECTD3
TC01002604.hg.1	2.29	PSR01040480.hg.1	HECTD3
TC01002604.hg.1	2.25	PSR01040487.hg.1	HECTD3
TC01002604.hg.1	2.24	PSR01040478.hg.1	HECTD3
TC01002604.hg.1	2.09	JUC01021739.hg.1	HECTD3
TC01002604.hg.1	2.03	PSR01040477.hg.1	HECTD3
TC01002604.hg.1	2.02	PSR01040492.hg.1	HECTD3
TC02000157.hg.1	2.77	JUC02000982.hg.1	MAPRE3
TC02000157.hg.1	2.15	PSR02001911.hg.1	MAPRE3
TC02000157.hg.1	2	JUC02000990.hg.1	MAPRE3
TC02002886.hg.1	2.77	JUC02024309.hg.1	HJURP
TC02002886.hg.1	2.69	JUC02024307.hg.1	HJURP
TC02002886.hg.1	2.63	PSR02046445.hg.1	HJURP

TC02002886.hg.1	2.5	PSR02046459.hg.1	HJURP
TC02002886.hg.1	2.03	JUC02024297.hg.1	HJURP
TC02002886.hg.1	-2.09	PSR02046448.hg.1	HJURP
TC02002886.hg.1	-2.18	JUC02024310.hg.1	HJURP
TC02002886.hg.1	-2.21	PSR02046458.hg.1	HJURP
TC05003439.hg.1	2.77	PSR05002644.hg.1	ITGA1
TC05003439.hg.1	2.64	PSR05002671.hg.1	ITGA1
TC05003439.hg.1	-2.45	JUC05019800.hg.1	ITGA1
TC05003439.hg.1	-4.53	JUC05019809.hg.1	ITGA1
TC17000189.hg.1	2.77	JUC17001478.hg.1	SNORD49A,
TC17000189.hg.1	2.64	JUC17001494.hg.1	SNORD49A,
TC17000189.hg.1	-2.06	PSR17002584.hg.1	SNORD49A,
TC17000189.hg.1	-2.1	JUC17001484.hg.1	SNORD49A,
TC17000189.hg.1	-2.24	JUC17001464.hg.1	SNORD49A,
TC17000189.hg.1	-2.93	PSR17002547.hg.1	SNORD49A,
TC17000583.hg.1	2.77	JUC17004068.hg.1	LOC1001339
TC17000583.hg.1	2.16	PSR17007173.hg.1	LOC1001339
TC17001523.hg.1	2.77	JUC17011344.hg.1	DHX58
TC17001523.hg.1	2.37	PSR17020288.hg.1	DHX58
TC17001523.hg.1	2.12	JUC17011339.hg.1	DHX58
TC17001523.hg.1	2.09	PSR17020307.hg.1	DHX58
TC01000001.hg.1	2.76	JUC01000001.hg.1	DDX11L1
TC01000001.hg.1	2.34	PSR01000002.hg.1	DDX11L1
TC01000001.hg.1	2.24	PSR01000004.hg.1	DDX11L1
TC01003343.hg.1	2.76	JUC01027356.hg.1	IQGAP3
TC01003343.hg.1	2.35	PSR01052240.hg.1	IQGAP3
TC01003343.hg.1	-2.05	PSR01052235.hg.1	IQGAP3
TC01003343.hg.1	-2.08	JUC01027330.hg.1	IQGAP3
TC01003343.hg.1	-2.56	PSR01052195.hg.1	IQGAP3
TC01003343.hg.1	-2.64	JUC01027331.hg.1	IQGAP3
TC01003343.hg.1	-2.91	JUC01027324.hg.1	IQGAP3
TC01003343.hg.1	-3.24	JUC01027354.hg.1	IQGAP3
TC01003343.hg.1	-8.45	JUC01027337.hg.1	IQGAP3
TC02000967.hg.1	2.76	PSR02014127.hg.1	7-Mar
TC02000967.hg.1	-2.32	JUC02007596.hg.1	7-Mar
TC02001755.hg.1	2.76	JUC02014798.hg.1	HNRPLL
TC02001755.hg.1	2.21	JUC02014784.hg.1	HNRPLL
TC02001755.hg.1	2.18	PSR02028264.hg.1	HNRPLL
TC03001819.hg.1	2.76	PSR03032911.hg.1	DZIP1L
TC03001819.hg.1	2.47	PSR03032926.hg.1	DZIP1L
TC03001819.hg.1	2.45	PSR03032924.hg.1	DZIP1L
TC03001819.hg.1	2.2	PSR03032902.hg.1	DZIP1L
TC03001819.hg.1	2.17	PSR03032925.hg.1	DZIP1L
TC03001819.hg.1	2.11	JUC03016330.hg.1	DZIP1L
TC03001819.hg.1	2.08	PSR03032921.hg.1	DZIP1L
TC03001819.hg.1	2.04	JUC03016320.hg.1	DZIP1L
TC04000643.hg.1	2.76	PSR04009278.hg.1	HSPA4L
TC04000643.hg.1	2.68	PSR04009275.hg.1	HSPA4L
TC04000643.hg.1	2.64	PSR04009303.hg.1	HSPA4L
TC04000643.hg.1	2.24	PSR04009274.hg.1	HSPA4L
TC04000643.hg.1	2.19	PSR04009277.hg.1	HSPA4L
TC04000643.hg.1	-2.08	JUC04004959.hg.1	HSPA4L
TC04000643.hg.1	-2.15	JUC04004969.hg.1	HSPA4L
TC04000643.hg.1	-2.22	JUC04004963.hg.1	HSPA4L
TC04001742.hg.1	2.76	JUC04012761.hg.1	SCRG1
TC04001742.hg.1	2.54	PSR04024069.hg.1	SCRG1
TC04002948.hg.1	2.76	PSR04022051.hg.1	MGARP
TC04002948.hg.1	2.08	PSR04022050.hg.1	MGARP

TC04002948.hg.1	-2.6	JUC04017373.hg.1	MGARP
TC04002948.hg.1	-4.93	JUC04017374.hg.1	MGARP
TC05000752.hg.1	2.76	JUC05005640.hg.1	PCDHB9
TC05000752.hg.1	2.42	PSR05010798.hg.1	PCDHB9
TC05000752.hg.1	2.2	PSR05010801.hg.1	PCDHB9
TC05001259.hg.1	2.76	JUC05009002.hg.1	RAD1
TC05001259.hg.1	2.12	PSR05017602.hg.1	RAD1
TC05001693.hg.1	2.76	JUC05011881.hg.1	CDO1
TC05001693.hg.1	2.55	JUC05011883.hg.1	CDO1
TC05001693.hg.1	2.09	PSR05022965.hg.1	CDO1
TC05001693.hg.1	-2.08	PSR05022966.hg.1	CDO1
TC09000467.hg.1	2.76	JUC09002764.hg.1	MIR23B, MIR
TC09000467.hg.1	2.14	JUC09002739.hg.1	MIR23B, MIR
TC09000467.hg.1	-2	PSR09005000.hg.1	MIR23B, MIR
TC09000467.hg.1	-2.02	PSR09005043.hg.1	MIR23B, MIR
TC09000467.hg.1	-2.04	JUC09002740.hg.1	MIR23B, MIR
TC09000467.hg.1	-2.09	JUC09002763.hg.1	MIR23B, MIR
TC09000467.hg.1	-2.12	PSR09005001.hg.1	MIR23B, MIR
TC09000467.hg.1	-2.21	JUC09002757.hg.1	MIR23B, MIR
TC09000467.hg.1	-2.23	JUC09002749.hg.1	MIR23B, MIR
TC09000467.hg.1	-2.29	PSR09005027.hg.1	MIR23B, MIR
TC09000467.hg.1	-2.4	JUC09002752.hg.1	MIR23B, MIR
TC09000467.hg.1	-2.46	PSR09005013.hg.1	MIR23B, MIR
TC09000467.hg.1	-2.65	JUC09002759.hg.1	MIR23B, MIR
TC09000467.hg.1	-2.93	JUC09002732.hg.1	MIR23B, MIR
TC09000467.hg.1	-3.04	PSR09005005.hg.1	MIR23B, MIR
TC09000467.hg.1	-3.45	JUC09002756.hg.1	MIR23B, MIR
TC09000467.hg.1	-3.71	PSR09005006.hg.1	MIR23B, MIR
TC09000855.hg.1	2.76	JUC09006124.hg.1	CACNA1B
TC09000855.hg.1	-2.05	PSR09011372.hg.1	CACNA1B
TC09000855.hg.1	-2.15	JUC09006117.hg.1	CACNA1B
TC09000855.hg.1	-2.31	JUC09006122.hg.1	CACNA1B
TC09000855.hg.1	-2.8	JUC09006114.hg.1	CACNA1B
TC0Y000144.hg.1	2.76	JUC0Y000922.hg.1	
TC0Y000144.hg.1	-2.26	JUC0Y000924.hg.1	
TC0Y000144.hg.1	-2.42	PSR0Y001522.hg.1	
TC0Y000144.hg.1	-2.42	PSR0Y001525.hg.1	
TC0Y000144.hg.1	-2.45	JUC0Y000919.hg.1	
TC0Y000144.hg.1	-2.54	JUC0Y000915.hg.1	
TC0Y000144.hg.1	-2.95	PSR0Y001517.hg.1	
TC11001026.hg.1	2.76	PSR11012839.hg.1	ZBTB16
TC11001026.hg.1	2.36	PSR11012836.hg.1	ZBTB16
TC11001026.hg.1	-2.54	JUC11006904.hg.1	ZBTB16
TC11001026.hg.1	-2.88	JUC11006906.hg.1	ZBTB16
TC11001026.hg.1	-3.56	PSR11012827.hg.1	ZBTB16
TC11001026.hg.1	-3.65	JUC11006907.hg.1	ZBTB16
TC11001026.hg.1	-3.91	PSR11012830.hg.1	ZBTB16
TC11001419.hg.1	2.76	JUC11009586.hg.1	ZBED5
TC11001419.hg.1	-2.1	JUC11009578.hg.1	ZBED5
TC11001419.hg.1	-2.32	JUC11009583.hg.1	ZBED5
TC11001419.hg.1	-2.37	PSR11017881.hg.1	ZBED5
TC11001419.hg.1	-3.24	PSR11017880.hg.1	ZBED5
TC12002013.hg.1	2.76	JUC12014752.hg.1	FBXO21
TC12002013.hg.1	2.24	PSR12026387.hg.1	FBXO21
TC12002013.hg.1	-2.58	JUC12014749.hg.1	FBXO21
TC21000176.hg.1	2.76	PSR21001691.hg.1	SH3BGR
TC21000176.hg.1	-2.33	PSR21001695.hg.1	SH3BGR
TC21000176.hg.1	-2.81	JUC21000869.hg.1	SH3BGR

TC22000256.hg.1	2.76	JUC22001705.hg.1	HMGXB4
TC22000256.hg.1	2.48	PSR22004718.hg.1	HMGXB4
TC22000256.hg.1	2.36	JUC22001699.hg.1	HMGXB4
TC22000256.hg.1	2.01	JUC22001698.hg.1	HMGXB4
TC22000256.hg.1	-2.14	PSR22004730.hg.1	HMGXB4
TC22000256.hg.1	-2.25	JUC22001709.hg.1	HMGXB4
TC01006297.hg.1	2.75	JUC01039455.hg.1	CDK11A, CDI
TC01006297.hg.1	2.3	PSR01032151.hg.1	CDK11A, CDI
TC01006297.hg.1	2.22	JUC01039439.hg.1	CDK11A, CDI
TC01006297.hg.1	2.11	PSR01032147.hg.1	CDK11A, CDI
TC01006297.hg.1	2.05	PSR01032121.hg.1	CDK11A, CDI
TC01006297.hg.1	2.01	JUC01039464.hg.1	CDK11A, CDI
TC01006297.hg.1	-2.37	JUC01039458.hg.1	CDK11A, CDI
TC03000051.hg.1	2.75	JUC03000592.hg.1	FANCD2
TC03000051.hg.1	2.09	PSR03001177.hg.1	FANCD2
TC03000051.hg.1	2.02	PSR03001210.hg.1	FANCD2
TC03000051.hg.1	-2.01	JUC03000632.hg.1	FANCD2
TC03000051.hg.1	-2.02	PSR03001199.hg.1	FANCD2
TC03000051.hg.1	-2.28	JUC03000602.hg.1	FANCD2
TC03000289.hg.1	2.75	PSR03005938.hg.1	CCDC36
TC03000289.hg.1	2.37	PSR03005941.hg.1	CCDC36
TC03000289.hg.1	2.33	JUC03002967.hg.1	CCDC36
TC03000289.hg.1	2.28	PSR03005934.hg.1	CCDC36
TC03000289.hg.1	2.24	PSR03005933.hg.1	CCDC36
TC03000289.hg.1	2.07	PSR03005942.hg.1	CCDC36
TC03000289.hg.1	2.06	PSR03005936.hg.1	CCDC36
TC03000289.hg.1	-2.15	PSR03005947.hg.1	CCDC36
TC03000289.hg.1	-3.12	JUC03002972.hg.1	CCDC36
TC03000746.hg.1	2.75	JUC03006861.hg.1	FAIM
TC03000746.hg.1	-5.11	PSR03013582.hg.1	FAIM
TC03000746.hg.1	-5.35	PSR03013601.hg.1	FAIM
TC03000746.hg.1	-5.5	PSR03013590.hg.1	FAIM
TC03000746.hg.1	-5.6	PSR03013583.hg.1	FAIM
TC03000746.hg.1	-6.1	PSR03013581.hg.1	FAIM
TC03000746.hg.1	-6.25	JUC03006865.hg.1	FAIM
TC03000746.hg.1	-7.87	PSR03013586.hg.1	FAIM
TC03000746.hg.1	-8.37	JUC03006869.hg.1	FAIM
TC03000746.hg.1	-10.1	PSR03013584.hg.1	FAIM
TC03000746.hg.1	-10.44	JUC03006864.hg.1	FAIM
TC03000746.hg.1	-11.45	JUC03006859.hg.1	FAIM
TC03000746.hg.1	-13.47	JUC03006863.hg.1	FAIM
TC03000746.hg.1	-14.01	JUC03006862.hg.1	FAIM
TC03003351.hg.1	2.75	JUC03023652.hg.1	RBM6
TC03003351.hg.1	2.14	JUC03023656.hg.1	RBM6
TC03003351.hg.1	2.08	PSR03006247.hg.1	RBM6
TC03003351.hg.1	2.03	JUC03023659.hg.1	RBM6
TC03003351.hg.1	2.02	JUC03023647.hg.1	RBM6
TC03003351.hg.1	-2.14	PSR03006261.hg.1	RBM6
TC03003351.hg.1	-2.28	JUC03023644.hg.1	RBM6
TC03003351.hg.1	-3.32	JUC03023662.hg.1	RBM6
TC03003351.hg.1	-3.96	JUC03023632.hg.1	RBM6
TC04000788.hg.1	2.75	PSR04010869.hg.1	GUCY1B3
TC04000788.hg.1	2.61	PSR04010850.hg.1	GUCY1B3
TC04000788.hg.1	2.19	PSR04010857.hg.1	GUCY1B3
TC04000788.hg.1	2.08	JUC04005825.hg.1	GUCY1B3
TC04000788.hg.1	2.06	PSR04010856.hg.1	GUCY1B3
TC04000788.hg.1	-2.06	JUC04005827.hg.1	GUCY1B3
TC04000788.hg.1	-2.6	JUC04005831.hg.1	GUCY1B3

TC04000788.hg.1	-4.44	JUC04005839.hg.1	GUCY1B3
TC04000972.hg.1	2.75	PSR04013361.hg.1	POLN, HAUS
TC04000972.hg.1	2.08	JUC04007111.hg.1	POLN, HAUS
TC04000972.hg.1	-2.1	PSR04013372.hg.1	POLN, HAUS
TC04001540.hg.1	2.75	JUC04011476.hg.1	SCLT1
TC04001540.hg.1	2.7	JUC04011465.hg.1	SCLT1
TC04001540.hg.1	2.5	PSR04021762.hg.1	SCLT1
TC04001540.hg.1	2.41	JUC04011484.hg.1	SCLT1
TC04001540.hg.1	2.19	PSR04021764.hg.1	SCLT1
TC04001540.hg.1	2.08	PSR04021769.hg.1	SCLT1
TC04001540.hg.1	2.07	JUC04011478.hg.1	SCLT1
TC04001540.hg.1	2.06	JUC04011474.hg.1	SCLT1
TC04001540.hg.1	2.01	PSR04021796.hg.1	SCLT1
TC05000334.hg.1	2.75	PSR05004533.hg.1	MCCC2
TC05000334.hg.1	2.47	JUC05002441.hg.1	MCCC2
TC05000334.hg.1	2.15	PSR05004539.hg.1	MCCC2
TC07001486.hg.1	2.75	JUC07010960.hg.1	TYW1B
TC07001486.hg.1	2.05	JUC07010949.hg.1	TYW1B
TC07001486.hg.1	-2.06	JUC07010943.hg.1	TYW1B
TC07001486.hg.1	-2.22	PSR07022259.hg.1	TYW1B
TC08001496.hg.1	2.75	JUC08009949.hg.1	RRM2B
TC08001496.hg.1	2.74	JUC08009945.hg.1	RRM2B
TC08001496.hg.1	2.44	PSR08019603.hg.1	RRM2B
TC08001496.hg.1	2.02	PSR08019607.hg.1	RRM2B
TC09000182.hg.1	2.75	JUC09001049.hg.1	UNC13B
TC09000182.hg.1	2.7	PSR09001967.hg.1	UNC13B
TC09000182.hg.1	2.63	JUC09001050.hg.1	UNC13B
TC09000182.hg.1	2.55	JUC09001051.hg.1	UNC13B
TC09000182.hg.1	2.26	JUC09001046.hg.1	UNC13B
TC09000182.hg.1	2.23	PSR09002013.hg.1	UNC13B
TC09000182.hg.1	2.23	JUC09001053.hg.1	UNC13B
TC09000182.hg.1	2.19	JUC09001040.hg.1	UNC13B
TC09000182.hg.1	2.1	PSR09001964.hg.1	UNC13B
TC09000182.hg.1	2.06	PSR09001968.hg.1	UNC13B
TC09000182.hg.1	2.03	PSR09001970.hg.1	UNC13B
TC09000182.hg.1	-2.09	JUC09001059.hg.1	UNC13B
TC10000739.hg.1	2.75	JUC10004858.hg.1	DPCD
TC10000739.hg.1	2.45	JUC10004866.hg.1	DPCD
TC10000739.hg.1	2.08	JUC10004864.hg.1	DPCD
TC10000739.hg.1	2.01	PSR10008732.hg.1	DPCD
TC11000980.hg.1	2.75	JUC11006534.hg.1	ATM
TC11000980.hg.1	-2.02	JUC11006577.hg.1	ATM
TC11000980.hg.1	-2.15	JUC11006553.hg.1	ATM
TC11000980.hg.1	-2.19	JUC11006570.hg.1	ATM
TC11000980.hg.1	-2.2	JUC11006579.hg.1	ATM
TC11000980.hg.1	-2.21	JUC11006599.hg.1	ATM
TC11000980.hg.1	-2.42	PSR11012216.hg.1	ATM
TC11000980.hg.1	-2.53	JUC11006592.hg.1	ATM
TC11000980.hg.1	-2.67	JUC11006595.hg.1	ATM
TC11000980.hg.1	-2.93	JUC11006537.hg.1	ATM
TC11000980.hg.1	-3.01	JUC11006571.hg.1	ATM
TC11000980.hg.1	-3.23	PSR11012218.hg.1	ATM
TC11000980.hg.1	-4.49	JUC11006561.hg.1	ATM
TC12000825.hg.1	2.75	JUC12005800.hg.1	RIC8B
TC12000825.hg.1	2.58	JUC12005808.hg.1	RIC8B
TC12000825.hg.1	2.06	PSR12010554.hg.1	RIC8B
TC12000825.hg.1	-2.72	JUC12005798.hg.1	RIC8B
TC12000825.hg.1	-2.84	JUC12005797.hg.1	RIC8B

TC12003296.hg.1	2.75	PSR12005887.hg.1	HOXC6
TC12003296.hg.1	2.02	JUC12020626.hg.1	HOXC6
TC14000132.hg.1	2.75	PSR14001122.hg.1	MRPL52
TC14000132.hg.1	-2.23	PSR14001135.hg.1	MRPL52
TC14000132.hg.1	-2.4	PSR14001127.hg.1	MRPL52
TC14000132.hg.1	-3.6	JUC14000486.hg.1	MRPL52
TC20000555.hg.1	2.75	JUC20004141.hg.1	SIRPG
TC20000555.hg.1	2.58	JUC20004144.hg.1	SIRPG
TC20000555.hg.1	2.51	JUC20004150.hg.1	SIRPG
TC20000555.hg.1	2.12	PSR20008054.hg.1	SIRPG
TC20000555.hg.1	2.04	JUC20004147.hg.1	SIRPG
TC20000555.hg.1	-2.47	PSR20008062.hg.1	SIRPG
TC21000131.hg.1	2.75	JUC21000547.hg.1	ITSN1
TC21000131.hg.1	2.72	PSR21001102.hg.1	ITSN1
TC21000131.hg.1	2.43	JUC21000585.hg.1	ITSN1
TC21000131.hg.1	2.17	PSR21001103.hg.1	ITSN1
TC21000131.hg.1	2.03	PSR21001112.hg.1	ITSN1
TC22001477.hg.1	2.75	JUC22009458.hg.1	PNPLA3
TC22001477.hg.1	-2.02	PSR22007011.hg.1	PNPLA3
TC22001477.hg.1	-2.3	PSR22006996.hg.1	PNPLA3
TC22001477.hg.1	-2.85	PSR22006998.hg.1	PNPLA3
TC22001477.hg.1	-3.07	PSR22006993.hg.1	PNPLA3
TC22001477.hg.1	-4.53	JUC22009456.hg.1	PNPLA3
TC6_qbl_hap6000	2.75	PSR6_qbl_hap60040	VPS52
TC6_qbl_hap6000	2.3	JUC6_qbl_hap60016	VPS52
TC6_qbl_hap6000	-2.1	JUC6_qbl_hap60016	VPS52
TC6_qbl_hap6000	-2.18	JUC6_qbl_hap60016	VPS52
TC6_qbl_hap6000	-2.28	JUC6_qbl_hap60015	VPS52
TC02002946.hg.1	2.74	PSR02047255.hg.1	MTERFD2
TC02002946.hg.1	2.55	PSR02047250.hg.1	MTERFD2
TC02002946.hg.1	2.12	JUC02024743.hg.1	MTERFD2
TC02002946.hg.1	-2.28	PSR02047273.hg.1	MTERFD2
TC03002170.hg.1	2.74	PSR03038332.hg.1	NCBP2
TC03002170.hg.1	2.41	JUC03018976.hg.1	NCBP2
TC03002170.hg.1	2.24	PSR03038330.hg.1	NCBP2
TC03002170.hg.1	-2.31	JUC03018977.hg.1	NCBP2
TC03003318.hg.1	2.74	JUC03023033.hg.1	WDR6
TC03003318.hg.1	2.38	JUC03023045.hg.1	WDR6
TC03003318.hg.1	2.27	JUC03023044.hg.1	WDR6
TC03003318.hg.1	2.1	PSR03005847.hg.1	WDR6
TC09000753.hg.1	2.74	PSR09009706.hg.1	
TC09000753.hg.1	2.12	JUC09005325.hg.1	
TC12000092.hg.1	2.74	PSR12001045.hg.1	CD4
TC12000092.hg.1	2.48	JUC12000568.hg.1	CD4
TC12000092.hg.1	2.17	PSR12001051.hg.1	CD4
TC12000092.hg.1	2.11	JUC12000563.hg.1	CD4
TC12000092.hg.1	2.08	PSR12001042.hg.1	CD4
TC12000092.hg.1	-2.49	PSR12001054.hg.1	CD4
TC12000092.hg.1	-2.55	JUC12000579.hg.1	CD4
TC12001843.hg.1	2.74	PSR12024272.hg.1	LTA4H
TC12001843.hg.1	2.23	JUC12013477.hg.1	LTA4H
TC12001843.hg.1	2.09	PSR12024257.hg.1	LTA4H
TC12001843.hg.1	-2.76	JUC12013496.hg.1	LTA4H
TC12001843.hg.1	-2.91	PSR12024253.hg.1	LTA4H
TC15001886.hg.1	2.74	PSR15018089.hg.1	FAM174B
TC15001886.hg.1	2.13	JUC15009862.hg.1	FAM174B
TC15001886.hg.1	-2.61	JUC15009868.hg.1	FAM174B
TC15001886.hg.1	-3.66	PSR15018079.hg.1	FAM174B

TC16000571.hg.1	2.74	PSR16008252.hg.1	WWP2
TC16000571.hg.1	2.46	PSR16008260.hg.1	WWP2
TC16000571.hg.1	2.36	PSR16008262.hg.1	WWP2
TC16000571.hg.1	2.21	JUC16004626.hg.1	WWP2
TC16000571.hg.1	2.05	JUC16004644.hg.1	WWP2
TC16000571.hg.1	-2.02	JUC16004631.hg.1	WWP2
TC16000571.hg.1	-2.13	PSR16008279.hg.1	WWP2
TC17001565.hg.1	2.74	JUC17011767.hg.1	MPP2
TC17001565.hg.1	2.38	PSR17021029.hg.1	MPP2
TC17001565.hg.1	2.17	PSR17021021.hg.1	MPP2
TC17001565.hg.1	2.12	PSR17021030.hg.1	MPP2
TC17001565.hg.1	-2.19	PSR17021017.hg.1	MPP2
TC17001565.hg.1	-2.7	JUC17011768.hg.1	MPP2
TC18000009.hg.1	2.74	JUC18000057.hg.1	NDC80
TC18000009.hg.1	2.17	PSR18000097.hg.1	NDC80
TC18000009.hg.1	-2.09	PSR18000089.hg.1	NDC80
TC18000009.hg.1	-2.39	PSR18000088.hg.1	NDC80
TC18000009.hg.1	-4.02	JUC18000053.hg.1	NDC80
TC22000667.hg.1	2.74	JUC22004846.hg.1	PIK3IP1
TC22000667.hg.1	2.24	PSR22011782.hg.1	PIK3IP1
TC22000667.hg.1	2.12	JUC22004839.hg.1	PIK3IP1
TC6_ssto_hap700(	2.74	PSR6_ssto_hap7001	SAPCD1, MS
TC6_ssto_hap700(	2.68	JUC6_ssto_hap7000	SAPCD1, MS
TC6_ssto_hap700(	2.63	JUC6_ssto_hap7000	SAPCD1, MS
TC6_ssto_hap700(	2.41	JUC6_ssto_hap7000	SAPCD1, MS
TC6_ssto_hap700(	2.26	JUC6_ssto_hap7000	SAPCD1, MS
TC6_ssto_hap700(	-2.17	JUC6_ssto_hap7000	SAPCD1, MS
TC6_ssto_hap700(	-2.64	JUC6_ssto_hap7000	SAPCD1, MS
TC6_ssto_hap700(	2.74	JUC6_ssto_hap7001	HLA-H
TC6_ssto_hap700(	2.46	PSR6_ssto_hap7000	HLA-H
TC6_ssto_hap700(	2.43	JUC6_ssto_hap7001	HLA-H
TC6_ssto_hap700(	2.22	PSR6_ssto_hap7000	HLA-H
TC6_ssto_hap700(	2.18	PSR6_ssto_hap7000	HLA-H
TC6_ssto_hap700(	2.74	PSR6_ssto_hap7000	HLA-G
TC6_ssto_hap700(	-2	JUC6_ssto_hap7001	HLA-G
TC6_ssto_hap700(	-2.53	PSR6_ssto_hap7000	HLA-G
TC02001742.hg.1	2.73	JUC02014703.hg.1	PRKD3
TC02001742.hg.1	2.15	PSR02028073.hg.1	PRKD3
TC02001742.hg.1	-2.66	JUC02014704.hg.1	PRKD3
TC03001361.hg.1	2.73	JUC03011609.hg.1	PRSS42
TC03001361.hg.1	2.34	PSR03023325.hg.1	PRSS42
TC03001361.hg.1	2.22	PSR03023318.hg.1	PRSS42
TC08000598.hg.1	2.73	JUC08004187.hg.1	MATN2, LOC
TC08000598.hg.1	2.17	PSR08008297.hg.1	MATN2, LOC
TC08000598.hg.1	-2	PSR08008309.hg.1	MATN2, LOC
TC08000598.hg.1	-2.03	JUC08004170.hg.1	MATN2, LOC
TC08000598.hg.1	-2.35	PSR08008317.hg.1	MATN2, LOC
TC08000598.hg.1	-2.43	PSR08008313.hg.1	MATN2, LOC
TC08000598.hg.1	-2.92	JUC08004186.hg.1	MATN2, LOC
TC08000598.hg.1	-3.08	PSR08008310.hg.1	MATN2, LOC
TC08000598.hg.1	-3.09	JUC08004173.hg.1	MATN2, LOC
TC08000598.hg.1	-3.15	JUC08004174.hg.1	MATN2, LOC
TC08000598.hg.1	-3.55	JUC08004181.hg.1	MATN2, LOC
TC08000598.hg.1	-4.2	JUC08004179.hg.1	MATN2, LOC
TC09000400.hg.1	2.73	PSR09004331.hg.1	CTSL1
TC09000400.hg.1	2.43	PSR09004300.hg.1	CTSL1
TC09000400.hg.1	2.29	JUC09002372.hg.1	CTSL1
TC09000400.hg.1	2.01	PSR09004327.hg.1	CTSL1

TC09000400.hg.1	-2.14	JUC09002367.hg.1	CTSL1
TC09000400.hg.1	-2.4	JUC09002371.hg.1	CTSL1
TC0X000868.hg.1	2.73	JUC0X005686.hg.1	GPM6B
TC0X000868.hg.1	-2.01	PSR0X011581.hg.1	GPM6B
TC0X000868.hg.1	-2.42	PSR0X011580.hg.1	GPM6B
TC0X000868.hg.1	-2.62	PSR0X011571.hg.1	GPM6B
TC0X000868.hg.1	-3.17	PSR0X011592.hg.1	GPM6B
TC0X000868.hg.1	-3.28	PSR0X011594.hg.1	GPM6B
TC0X000868.hg.1	-3.34	JUC0X005691.hg.1	GPM6B
TC0X000868.hg.1	-3.44	PSR0X011560.hg.1	GPM6B
TC0X000868.hg.1	-3.49	PSR0X011587.hg.1	GPM6B
TC0X000868.hg.1	-3.6	PSR0X011595.hg.1	GPM6B
TC0X000868.hg.1	-3.64	PSR0X011593.hg.1	GPM6B
TC0X000868.hg.1	-3.67	JUC0X005701.hg.1	GPM6B
TC0X000868.hg.1	-4.05	PSR0X011584.hg.1	GPM6B
TC0X000868.hg.1	-4.08	PSR0X011591.hg.1	GPM6B
TC0X000868.hg.1	-4.54	JUC0X005696.hg.1	GPM6B
TC0X000868.hg.1	-5.58	PSR0X011588.hg.1	GPM6B
TC0X000868.hg.1	-5.64	JUC0X005685.hg.1	GPM6B
TC15001763.hg.1	2.73	PSR15017300.hg.1	C15orf40
TC15001763.hg.1	2.08	PSR15017286.hg.1	C15orf40
TC15001763.hg.1	-2.02	PSR15017293.hg.1	C15orf40
TC15001763.hg.1	-4.05	JUC15009442.hg.1	C15orf40
TC17000363.hg.1	2.73	JUC17002628.hg.1	RAB11FIP4, N
TC17000363.hg.1	2.48	PSR17004613.hg.1	RAB11FIP4, N
TC17000363.hg.1	-3.43	PSR17004616.hg.1	RAB11FIP4, N
TC20000574.hg.1	2.73	PSR20008408.hg.1	ADAM33
TC20000574.hg.1	2.55	JUC20004331.hg.1	ADAM33
TC20000574.hg.1	-2.2	PSR20008401.hg.1	ADAM33
TC22000324.hg.1	2.73	PSR22006266.hg.1	SGSM3
TC22000324.hg.1	2.26	JUC22002253.hg.1	SGSM3
TC01002196.hg.1	2.72	PSR01033889.hg.1	EXOSC10
TC01002196.hg.1	-2.08	PSR01033895.hg.1	EXOSC10
TC01002196.hg.1	-2.43	JUC01018175.hg.1	EXOSC10
TC01002196.hg.1	-2.61	JUC01018156.hg.1	EXOSC10
TC01002307.hg.1	2.72	JUC01018991.hg.1	PLA2G2A
TC01002307.hg.1	2.25	JUC01018994.hg.1	PLA2G2A
TC01002307.hg.1	-2.13	PSR01035344.hg.1	PLA2G2A
TC01006325.hg.1	2.72	JUC01044069.hg.1	RWDD3
TC01006325.hg.1	2.03	PSR01013901.hg.1	RWDD3
TC02000816.hg.1	2.72	JUC02006650.hg.1	POTEJ
TC02000816.hg.1	-2.05	JUC02006652.hg.1	POTEJ
TC02000816.hg.1	-2.36	PSR02012566.hg.1	POTEJ
TC03001714.hg.1	2.72	JUC03015503.hg.1	CCDC14
TC03001714.hg.1	2.1	JUC03015513.hg.1	CCDC14
TC03001714.hg.1	-2.15	PSR03031144.hg.1	CCDC14
TC03001714.hg.1	-2.24	PSR03031126.hg.1	CCDC14
TC03001714.hg.1	-2.26	PSR03031146.hg.1	CCDC14
TC03001714.hg.1	-2.32	PSR03031149.hg.1	CCDC14
TC04000363.hg.1	2.72	PSR04004986.hg.1	LOC550112, I
TC04000363.hg.1	2.33	PSR04004961.hg.1	LOC550112, I
TC04000363.hg.1	2.31	PSR04004962.hg.1	LOC550112, I
TC04000363.hg.1	2.24	JUC04002472.hg.1	LOC550112, I
TC04000363.hg.1	2.18	JUC04002480.hg.1	LOC550112, I
TC05000009.hg.1	2.72	JUC05000123.hg.1	CEP72
TC05000009.hg.1	2.24	JUC05000114.hg.1	CEP72
TC05000009.hg.1	-2.2	JUC05000126.hg.1	CEP72
TC05000009.hg.1	-2.28	PSR05000223.hg.1	CEP72

TC06001869.hg.1	2.72	JUC06010965.hg.1	KHDC1L, KHI
TC06001869.hg.1	2.39	JUC06010973.hg.1	KHDC1L, KHI
TC06001869.hg.1	2.2	JUC06010976.hg.1	KHDC1L, KHI
TC06001869.hg.1	2.01	JUC06010975.hg.1	KHDC1L, KHI
TC06001869.hg.1	-2.07	JUC06010978.hg.1	KHDC1L, KHI
TC06001869.hg.1	-2.15	PSR06022666.hg.1	KHDC1L, KHI
TC07003351.hg.1	2.72	JUC07021327.hg.1	HOXA3
TC07003351.hg.1	-2.29	JUC07021331.hg.1	HOXA3
TC07003351.hg.1	-2.42	PSR07019079.hg.1	HOXA3
TC07003351.hg.1	-3.08	PSR07019081.hg.1	HOXA3
TC07003351.hg.1	-3.63	PSR07019098.hg.1	HOXA3
TC11000539.hg.1	2.72	JUC11003380.hg.1	C11orf9
TC11000539.hg.1	2.09	JUC11003369.hg.1	C11orf9
TC11000539.hg.1	-2.05	PSR11006549.hg.1	C11orf9
TC11000539.hg.1	-2.15	JUC11003382.hg.1	C11orf9
TC11000539.hg.1	-2.37	JUC11003376.hg.1	C11orf9
TC12000884.hg.1	2.72	JUC12006416.hg.1	OAS1
TC12000884.hg.1	2.16	JUC12006417.hg.1	OAS1
TC12000884.hg.1	-2.19	PSR12011665.hg.1	OAS1
TC14001313.hg.1	2.72	JUC14007602.hg.1	PGF
TC14001313.hg.1	2.02	JUC14007606.hg.1	PGF
TC14001313.hg.1	-2.11	PSR14014998.hg.1	PGF
TC14001313.hg.1	-2.12	PSR14015003.hg.1	PGF
TC14001313.hg.1	-4.81	JUC14007605.hg.1	PGF
TC14001313.hg.1	-8.6	JUC14007604.hg.1	PGF
TC01001669.hg.1	2.71	JUC01013808.hg.1	RNPEP
TC01001669.hg.1	2.66	PSR01025836.hg.1	RNPEP
TC01001669.hg.1	2.51	PSR01025845.hg.1	RNPEP
TC01001669.hg.1	2.32	PSR01025849.hg.1	RNPEP
TC02000285.hg.1	2.71	PSR02004337.hg.1	CRIPT
TC02000285.hg.1	2.62	PSR02004342.hg.1	CRIPT
TC02000285.hg.1	2.15	PSR02004336.hg.1	CRIPT
TC02000285.hg.1	2.1	PSR02004340.hg.1	CRIPT
TC02000285.hg.1	2	JUC02002261.hg.1	CRIPT
TC02001034.hg.1	2.71	JUC02008254.hg.1	ZAK
TC02001034.hg.1	2.08	PSR02015483.hg.1	ZAK
TC02001034.hg.1	-2.17	JUC02008257.hg.1	ZAK
TC02001911.hg.1	2.71	JUC02016108.hg.1	PELI1
TC02001911.hg.1	-2.23	PSR02030571.hg.1	PELI1
TC04000009.hg.1	2.71	JUC04000045.hg.1	PIGG
TC04000009.hg.1	2.51	PSR04000118.hg.1	PIGG
TC04000009.hg.1	2.42	JUC04000038.hg.1	PIGG
TC04000009.hg.1	2.35	JUC04000010.hg.1	PIGG
TC04000009.hg.1	2.24	JUC04000014.hg.1	PIGG
TC04000009.hg.1	2.19	PSR04000114.hg.1	PIGG
TC04000009.hg.1	2.06	PSR04000115.hg.1	PIGG
TC04000009.hg.1	-4.38	PSR04000126.hg.1	PIGG
TC04000427.hg.1	2.71	JUC04003012.hg.1	SHROOM3
TC04000427.hg.1	2.36	PSR04005982.hg.1	SHROOM3
TC04000427.hg.1	2.3	PSR04006001.hg.1	SHROOM3
TC04000427.hg.1	2.11	PSR04005986.hg.1	SHROOM3
TC04000427.hg.1	2	PSR04005988.hg.1	SHROOM3
TC04000427.hg.1	-2.22	JUC04003009.hg.1	SHROOM3
TC05000363.hg.1	2.71	JUC05002687.hg.1	HMGCR
TC05000363.hg.1	-2.42	JUC05002688.hg.1	HMGCR
TC05000363.hg.1	-3.31	PSR05005021.hg.1	HMGCR
TC05000942.hg.1	2.71	JUC05007150.hg.1	NPM1
TC05000942.hg.1	2.13	PSR05013776.hg.1	NPM1

TC06000120.hg.1	2.71	PSR06001259.hg.1	RNF144B
TC06000120.hg.1	2.59	PSR06001258.hg.1	RNF144B
TC06000120.hg.1	-2.14	JUC06000604.hg.1	RNF144B
TC08000406.hg.1	2.71	PSR08005683.hg.1	LOC1005055
TC08000406.hg.1	2.17	PSR08005682.hg.1	LOC1005055
TC08000406.hg.1	2.05	JUC08002813.hg.1	LOC1005055
TC0X000082.hg.1	2.71	JUC0X000583.hg.1	REPS2
TC0X000082.hg.1	-2.01	PSR0X001092.hg.1	REPS2
TC0X000082.hg.1	-2.05	JUC0X000597.hg.1	REPS2
TC0X000082.hg.1	-2.07	PSR0X001077.hg.1	REPS2
TC0X000082.hg.1	-2.23	PSR0X001102.hg.1	REPS2
TC0X000082.hg.1	-2.44	PSR0X001094.hg.1	REPS2
TC0X000082.hg.1	-2.47	PSR0X001097.hg.1	REPS2
TC0X000082.hg.1	-2.54	PSR0X001104.hg.1	REPS2
TC0X000082.hg.1	-2.55	JUC0X000593.hg.1	REPS2
TC0X000082.hg.1	-2.87	PSR0X001083.hg.1	REPS2
TC0X000082.hg.1	-3.22	PSR0X001075.hg.1	REPS2
TC0X000082.hg.1	-3.43	PSR0X001093.hg.1	REPS2
TC0X000082.hg.1	-3.46	PSR0X001076.hg.1	REPS2
TC0X000082.hg.1	-3.59	PSR0X001074.hg.1	REPS2
TC10001573.hg.1	2.71	JUC10011732.hg.1	CRTAC1
TC10001573.hg.1	2.04	PSR10020168.hg.1	CRTAC1
TC10001573.hg.1	-2.03	PSR10020166.hg.1	CRTAC1
TC10001573.hg.1	-2.19	JUC10011734.hg.1	CRTAC1
TC10001573.hg.1	-2.21	JUC10011729.hg.1	CRTAC1
TC10001573.hg.1	-2.26	JUC10011739.hg.1	CRTAC1
TC10001573.hg.1	-2.41	JUC10011738.hg.1	CRTAC1
TC10001573.hg.1	-2.71	PSR10020177.hg.1	CRTAC1
TC10001573.hg.1	-2.76	PSR10020167.hg.1	CRTAC1
TC10001573.hg.1	-3.17	JUC10011727.hg.1	CRTAC1
TC11000273.hg.1	2.71	PSR11003621.hg.1	
TC11000273.hg.1	2.57	JUC11001867.hg.1	
TC11000273.hg.1	2.27	PSR11003620.hg.1	
TC14000836.hg.1	2.71	JUC14004695.hg.1	MTA1
TC14000836.hg.1	2.5	PSR14009202.hg.1	MTA1
TC14000836.hg.1	2.07	PSR14009206.hg.1	MTA1
TC14000836.hg.1	2.03	JUC14004692.hg.1	MTA1
TC14000836.hg.1	-2.29	JUC14004673.hg.1	MTA1
TC17001077.hg.1	2.71	PSR17014437.hg.1	DLG4
TC17001077.hg.1	2.61	PSR17014424.hg.1	DLG4
TC17001077.hg.1	2.44	PSR17014435.hg.1	DLG4
TC17001077.hg.1	2.1	PSR17014397.hg.1	DLG4
TC17001077.hg.1	2.06	JUC17008237.hg.1	DLG4
TC17001077.hg.1	2	PSR17014391.hg.1	DLG4
TC17001077.hg.1	-2.08	JUC17008247.hg.1	DLG4
TC17001129.hg.1	2.71	JUC17008671.hg.1	GAS7
TC17001129.hg.1	2.19	PSR17015199.hg.1	GAS7
TC17001129.hg.1	-2.09	JUC17008672.hg.1	GAS7
TC17001129.hg.1	-2.29	PSR17015209.hg.1	GAS7
TC17001129.hg.1	-2.32	PSR17015222.hg.1	GAS7
TC17001129.hg.1	-2.36	PSR17015216.hg.1	GAS7
TC17001129.hg.1	-2.53	PSR17015224.hg.1	GAS7
TC17001129.hg.1	-2.57	JUC17008679.hg.1	GAS7
TC17001129.hg.1	-2.57	JUC17008681.hg.1	GAS7
TC17001129.hg.1	-2.59	JUC17008684.hg.1	GAS7
TC17001129.hg.1	-2.98	PSR17015231.hg.1	GAS7
TC17001129.hg.1	-3.09	PSR17015226.hg.1	GAS7
TC17001129.hg.1	-4.02	PSR17015225.hg.1	GAS7

TC17001129.hg.1	-4.39	PSR17015227.hg.1	GAS7
TC17001129.hg.1	-4.52	JUC17008680.hg.1	GAS7
TC17001129.hg.1	-5.06	PSR17015218.hg.1	GAS7
TC17001129.hg.1	-5.18	PSR17015229.hg.1	GAS7
TC17001129.hg.1	-7.64	JUC17008685.hg.1	GAS7
TC18000272.hg.1	2.71	PSR18003195.hg.1	THOC1
TC18000272.hg.1	-2.02	JUC18002159.hg.1	THOC1
TC18000272.hg.1	-2.19	JUC18002157.hg.1	THOC1
TC18000272.hg.1	-2.81	JUC18002154.hg.1	THOC1
TC19000943.hg.1	2.71	JUC19007288.hg.1	ZNF419
TC19000943.hg.1	-5.28	PSR19012932.hg.1	ZNF419
TC19001525.hg.1	2.71	JUC19011896.hg.1	DYRK1B
TC19001525.hg.1	2.52	JUC19011897.hg.1	DYRK1B
TC19001525.hg.1	2.51	JUC19011895.hg.1	DYRK1B
TC19001525.hg.1	2.39	PSR19020751.hg.1	DYRK1B
TC19001525.hg.1	-2.22	JUC19011900.hg.1	DYRK1B
TC06000384.hg.1	2.7	JUC06001646.hg.1	HSPA1A, HSI
TC06000384.hg.1	2.39	PSR06003938.hg.1	HSPA1A, HSI
TC07000140.hg.1	2.7	PSR07002147.hg.1	KLHL7
TC07000140.hg.1	2.17	PSR07002166.hg.1	KLHL7
TC07000140.hg.1	-2.01	JUC07001065.hg.1	KLHL7
TC07000140.hg.1	-2.78	JUC07001045.hg.1	KLHL7
TC07000140.hg.1	-3.53	PSR07002178.hg.1	KLHL7
TC09000032.hg.1	2.7	PSR09000407.hg.1	RCL1
TC09000032.hg.1	2.25	JUC09000247.hg.1	RCL1
TC09000032.hg.1	-3.55	JUC09000250.hg.1	RCL1
TC0X000101.hg.1	2.7	JUC0X000744.hg.1	SMS
TC0X000101.hg.1	2.47	PSR0X001341.hg.1	SMS
TC0X000101.hg.1	2.26	PSR0X001347.hg.1	SMS
TC0X000101.hg.1	2.07	JUC0X000739.hg.1	SMS
TC10001054.hg.1	2.7	JUC10007627.hg.1	FAM107B
TC10001054.hg.1	-2.15	PSR10013152.hg.1	FAM107B
TC10001054.hg.1	-2.29	JUC10007611.hg.1	FAM107B
TC10001567.hg.1	2.7	JUC10011697.hg.1	MMS19
TC10001567.hg.1	2.39	JUC10011684.hg.1	MMS19
TC10001567.hg.1	-2.08	PSR10020074.hg.1	MMS19
TC10001567.hg.1	-2.36	JUC10011678.hg.1	MMS19
TC11001913.hg.1	2.7	PSR11022802.hg.1	MEN1
TC11001913.hg.1	-2.01	JUC11012013.hg.1	MEN1
TC17000671.hg.1	2.7	JUC17004726.hg.1	CACNA1G
TC17000671.hg.1	2.3	JUC17004731.hg.1	CACNA1G
TC17000671.hg.1	2.21	JUC17004719.hg.1	CACNA1G
TC17000671.hg.1	2.04	PSR17008678.hg.1	CACNA1G
TC17000671.hg.1	2.02	PSR17008697.hg.1	CACNA1G
TC17000671.hg.1	-2.73	JUC17004720.hg.1	CACNA1G
TC17000671.hg.1	-2.96	JUC17004743.hg.1	CACNA1G
TC17000671.hg.1	-3.57	JUC17004704.hg.1	CACNA1G
TC18000142.hg.1	2.7	JUC18001116.hg.1	MAPRE2
TC18000142.hg.1	2.65	PSR18001600.hg.1	MAPRE2
TC18000142.hg.1	2.45	PSR18001596.hg.1	MAPRE2
TC18000142.hg.1	2.15	JUC18001120.hg.1	MAPRE2
TC18000142.hg.1	2.06	PSR18001604.hg.1	MAPRE2
TC20000118.hg.1	2.7	JUC20000797.hg.1	ZNF133
TC20000118.hg.1	2.13	JUC20000807.hg.1	ZNF133
TC20000118.hg.1	-2.12	JUC20000796.hg.1	ZNF133
TC20000118.hg.1	-2.51	PSR20001549.hg.1	ZNF133
TC22000301.hg.1	2.7	JUC22002071.hg.1	KDELR3
TC22000301.hg.1	-2.85	PSR22005637.hg.1	KDELR3

TC01002135.hg.1	2.69	PSR01032834.hg.1	TP73-AS1
TC01002135.hg.1	-3.11	PSR01032819.hg.1	TP73-AS1
TC01002135.hg.1	-3.12	JUC01017587.hg.1	TP73-AS1
TC01002135.hg.1	-3.12	JUC01017589.hg.1	TP73-AS1
TC01002135.hg.1	-3.17	PSR01032818.hg.1	TP73-AS1
TC01006349.hg.1	2.69	PSR01038663.hg.1	ZMYM6NB, Z
TC01006349.hg.1	2.28	PSR01038662.hg.1	ZMYM6NB, Z
TC01006349.hg.1	2.05	PSR01038664.hg.1	ZMYM6NB, Z
TC01006349.hg.1	-2.14	JUC01044355.hg.1	ZMYM6NB, Z
TC01006349.hg.1	-2.17	JUC01044352.hg.1	ZMYM6NB, Z
TC01006349.hg.1	-7.41	JUC01044353.hg.1	ZMYM6NB, Z
TC02001328.hg.1	2.69	PSR02020674.hg.1	SPEG
TC02001328.hg.1	2.24	JUC02011052.hg.1	SPEG
TC02001328.hg.1	2.15	JUC02011034.hg.1	SPEG
TC02001328.hg.1	2.15	JUC02011038.hg.1	SPEG
TC02001328.hg.1	-3.63	JUC02011028.hg.1	SPEG
TC02004922.hg.1	2.69	JUC02031238.hg.1	LOC1005060
TC02004922.hg.1	2.21	PSR02024828.hg.1	LOC1005060
TC02004922.hg.1	2.09	PSR02024830.hg.1	LOC1005060
TC02004922.hg.1	2.02	PSR02024829.hg.1	LOC1005060
TC03000056.hg.1	2.69	JUC03000649.hg.1	IRAK2
TC03000056.hg.1	2.48	JUC03000652.hg.1	IRAK2
TC03000056.hg.1	-3.56	JUC03000656.hg.1	IRAK2
TC03000056.hg.1	-3.6	PSR03001283.hg.1	IRAK2
TC03001008.hg.1	2.69	PSR03017847.hg.1	VPS8, LOC1(
TC03001008.hg.1	2.68	JUC03009075.hg.1	VPS8, LOC1(
TC03001008.hg.1	2.49	JUC03009057.hg.1	VPS8, LOC1(
TC03001008.hg.1	2.13	JUC03009023.hg.1	VPS8, LOC1(
TC03001008.hg.1	2.03	PSR03017837.hg.1	VPS8, LOC1(
TC03001008.hg.1	2.02	JUC03009070.hg.1	VPS8, LOC1(
TC03002108.hg.1	2.69	PSR03037298.hg.1	CLDN1
TC03002108.hg.1	2.56	JUC03018438.hg.1	CLDN1
TC03002108.hg.1	2.39	PSR03037289.hg.1	CLDN1
TC03002108.hg.1	2.18	PSR03037297.hg.1	CLDN1
TC03002108.hg.1	2.07	PSR03037300.hg.1	CLDN1
TC08000175.hg.1	2.69	PSR08002227.hg.1	KIAA1967
TC08000175.hg.1	-2.02	JUC08001151.hg.1	KIAA1967
TC08001166.hg.1	2.69	PSR08014987.hg.1	SFRP1
TC08001166.hg.1	2.63	PSR08014989.hg.1	SFRP1
TC08001166.hg.1	2.42	PSR08014984.hg.1	SFRP1
TC08001166.hg.1	2.35	PSR08014983.hg.1	SFRP1
TC08001166.hg.1	2.18	PSR08014990.hg.1	SFRP1
TC08001166.hg.1	-2.76	PSR08014986.hg.1	SFRP1
TC08001166.hg.1	-2.79	JUC08007656.hg.1	SFRP1
TC08001166.hg.1	-3.44	PSR08014980.hg.1	SFRP1
TC08001166.hg.1	-3.54	PSR08014981.hg.1	SFRP1
TC08001166.hg.1	-5.7	JUC08007657.hg.1	SFRP1
TC09001181.hg.1	2.69	JUC09008163.hg.1	CBWD5, CBV
TC09001181.hg.1	2.28	JUC09008159.hg.1	CBWD5, CBV
TC09001181.hg.1	-2.01	PSR09015224.hg.1	CBWD5, CBV
TC09001181.hg.1	-2.03	PSR09015178.hg.1	CBWD5, CBV
TC09001181.hg.1	-2.04	PSR09015175.hg.1	CBWD5, CBV
TC09001181.hg.1	-2.11	PSR09015200.hg.1	CBWD5, CBV
TC09001181.hg.1	-2.16	PSR09015162.hg.1	CBWD5, CBV
TC09001181.hg.1	-2.16	JUC09008141.hg.1	CBWD5, CBV
TC09001181.hg.1	-2.24	JUC09008169.hg.1	CBWD5, CBV
TC09001181.hg.1	-2.69	JUC09008162.hg.1	CBWD5, CBV
TC09001181.hg.1	-2.71	PSR09015165.hg.1	CBWD5, CBV

TC09001181.hg.1	-2.82	PSR09015183.hg.1	CBWD5, CBV
TC09001181.hg.1	-4.72	JUC09008164.hg.1	CBWD5, CBV
TC0X000382.hg.1	2.69	JUC0X002463.hg.1	MED12
TC0X000382.hg.1	2.64	JUC0X002448.hg.1	MED12
TC0X000382.hg.1	2.4	PSR0X005018.hg.1	MED12
TC0Y000057.hg.1	2.69	PSR0Y000709.hg.1	NLGN4Y
TC0Y000057.hg.1	-2.06	PSR0Y000712.hg.1	NLGN4Y
TC0Y000057.hg.1	-2.25	PSR0Y000702.hg.1	NLGN4Y
TC0Y000057.hg.1	-2.26	PSR0Y000705.hg.1	NLGN4Y
TC0Y000057.hg.1	-2.29	PSR0Y000698.hg.1	NLGN4Y
TC0Y000057.hg.1	-2.4	PSR0Y000692.hg.1	NLGN4Y
TC0Y000057.hg.1	-2.4	JUC0Y000314.hg.1	NLGN4Y
TC0Y000057.hg.1	-2.53	PSR0Y000689.hg.1	NLGN4Y
TC0Y000057.hg.1	-2.65	PSR0Y000691.hg.1	NLGN4Y
TC0Y000057.hg.1	-2.8	PSR0Y000710.hg.1	NLGN4Y
TC0Y000057.hg.1	-3.05	PSR0Y000687.hg.1	NLGN4Y
TC0Y000057.hg.1	-3.23	PSR0Y000690.hg.1	NLGN4Y
TC0Y000057.hg.1	-3.41	PSR0Y000686.hg.1	NLGN4Y
TC0Y000057.hg.1	-3.49	PSR0Y000685.hg.1	NLGN4Y
TC0Y000057.hg.1	-3.68	PSR0Y000703.hg.1	NLGN4Y
TC0Y000057.hg.1	-4.36	JUC0Y000323.hg.1	NLGN4Y
TC0Y000057.hg.1	-4.85	JUC0Y000327.hg.1	NLGN4Y
TC0Y000057.hg.1	-5.21	PSR0Y000688.hg.1	NLGN4Y
TC0Y000057.hg.1	-7	JUC0Y000313.hg.1	NLGN4Y
TC10000412.hg.1	2.69	PSR10004617.hg.1	DDX50
TC10000412.hg.1	2.03	PSR10004616.hg.1	DDX50
TC10000412.hg.1	-2.12	JUC10002672.hg.1	DDX50
TC10000412.hg.1	-3.45	PSR10004626.hg.1	DDX50
TC10000813.hg.1	2.69	PSR10009754.hg.1	ACSL5
TC10000813.hg.1	2.35	JUC10005447.hg.1	ACSL5
TC10000813.hg.1	2.22	PSR10009753.hg.1	ACSL5
TC10000813.hg.1	2.13	PSR10009779.hg.1	ACSL5
TC10000813.hg.1	-2.07	PSR10009785.hg.1	ACSL5
TC10000813.hg.1	-2.26	JUC10005451.hg.1	ACSL5
TC10000820.hg.1	2.69	JUC10005505.hg.1	TCF7L2
TC10000820.hg.1	2.21	PSR10009847.hg.1	TCF7L2
TC10000820.hg.1	2.05	PSR10009838.hg.1	TCF7L2
TC10000820.hg.1	-2.09	JUC10005486.hg.1	TCF7L2
TC10000820.hg.1	-2.38	JUC10005491.hg.1	TCF7L2
TC11002342.hg.1	2.69	PSR11028106.hg.1	SCN4B
TC11002342.hg.1	2.33	JUC11014979.hg.1	SCN4B
TC11002342.hg.1	2.12	PSR11028104.hg.1	SCN4B
TC11002342.hg.1	-3.05	JUC11014982.hg.1	SCN4B
TC12000530.hg.1	2.69	PSR12006984.hg.1	GLI1
TC12000530.hg.1	-2.32	JUC12003671.hg.1	GLI1
TC12000530.hg.1	-4.99	JUC12003666.hg.1	GLI1
TC12002153.hg.1	2.69	JUC12015896.hg.1	POLE
TC12002153.hg.1	2.13	PSR12028211.hg.1	POLE
TC12002153.hg.1	2.04	JUC12015891.hg.1	POLE
TC12002153.hg.1	-2.15	JUC12015845.hg.1	POLE
TC12002153.hg.1	-2.44	JUC12015873.hg.1	POLE
TC12002153.hg.1	-2.81	JUC12015869.hg.1	POLE
TC12002153.hg.1	-3.11	JUC12015866.hg.1	POLE
TC13000351.hg.1	2.69	JUC13002275.hg.1	UBAC2
TC13000351.hg.1	-2.02	PSR13003730.hg.1	UBAC2
TC15001044.hg.1	2.69	JUC15004977.hg.1	GOLGA8DP
TC15001044.hg.1	2.63	JUC15004972.hg.1	GOLGA8DP
TC15001044.hg.1	2.33	PSR15009561.hg.1	GOLGA8DP

TC15001044.hg.1	-2.01	PSR15009557.hg.1	GOLGA8DP
TC17000533.hg.1	2.69	JUC17003629.hg.1	CNTNAP1
TC17000533.hg.1	2.64	JUC17003643.hg.1	CNTNAP1
TC17000533.hg.1	2.41	PSR17006465.hg.1	CNTNAP1
TC17000533.hg.1	2.27	PSR17006471.hg.1	CNTNAP1
TC17000533.hg.1	2.13	PSR17006466.hg.1	CNTNAP1
TC17000533.hg.1	2.09	JUC17003633.hg.1	CNTNAP1
TC17000533.hg.1	-2.08	PSR17006486.hg.1	CNTNAP1
TC17000533.hg.1	-2.19	JUC17003630.hg.1	CNTNAP1
TC17000533.hg.1	-2.26	JUC17003641.hg.1	CNTNAP1
TC17000533.hg.1	-2.48	JUC17003644.hg.1	CNTNAP1
TC17000533.hg.1	-2.6	PSR17006489.hg.1	CNTNAP1
TC17000533.hg.1	-2.62	JUC17003637.hg.1	CNTNAP1
TC17000533.hg.1	-2.99	JUC17003634.hg.1	CNTNAP1
TC17000533.hg.1	-3.92	JUC17003628.hg.1	CNTNAP1
TC17001170.hg.1	2.69	JUC17009075.hg.1	ZSWIM7
TC17001170.hg.1	2.11	PSR17015823.hg.1	ZSWIM7
TC17001170.hg.1	-2.27	PSR17015833.hg.1	ZSWIM7
TC18000277.hg.1	2.69	JUC18002197.hg.1	ENOSF1
TC18000277.hg.1	2.33	JUC18002188.hg.1	ENOSF1
TC18000277.hg.1	2.3	PSR18003261.hg.1	ENOSF1
TC18000277.hg.1	-4.17	JUC18002192.hg.1	ENOSF1
TC19000124.hg.1	2.69	JUC19001113.hg.1	ZNF557
TC19000124.hg.1	-2.31	PSR19001887.hg.1	ZNF557
TC19001013.hg.1	2.69	JUC19007765.hg.1	ATP8B3
TC19001013.hg.1	2.33	PSR19013830.hg.1	ATP8B3
TC19001013.hg.1	2.23	JUC19007770.hg.1	ATP8B3
TC19001013.hg.1	2.07	JUC19007780.hg.1	ATP8B3
TC19001013.hg.1	-2.01	PSR19013823.hg.1	ATP8B3
TC19001013.hg.1	-2.5	JUC19007776.hg.1	ATP8B3
TC19001013.hg.1	-2.53	JUC19007769.hg.1	ATP8B3
TC19001013.hg.1	-2.58	PSR19013844.hg.1	ATP8B3
TC19001013.hg.1	-2.59	PSR19013827.hg.1	ATP8B3
TC19001013.hg.1	-2.59	JUC19007775.hg.1	ATP8B3
TC19001013.hg.1	-2.88	JUC19007753.hg.1	ATP8B3
TC19001013.hg.1	-3.04	PSR19013828.hg.1	ATP8B3
TC19001013.hg.1	-3.3	JUC19007766.hg.1	ATP8B3
TC19001013.hg.1	-3.55	JUC19007777.hg.1	ATP8B3
TC19001013.hg.1	-3.71	PSR19013825.hg.1	ATP8B3
TC19001013.hg.1	-5.91	JUC19007756.hg.1	ATP8B3
TC20000334.hg.1	2.69	PSR20004727.hg.1	PKIG
TC20000334.hg.1	2.69	PSR20004731.hg.1	PKIG
TC20000334.hg.1	2.6	PSR20004743.hg.1	PKIG
TC20000334.hg.1	2.49	PSR20004736.hg.1	PKIG
TC20000334.hg.1	2.42	JUC20002621.hg.1	PKIG
TC20000334.hg.1	2.34	PSR20004742.hg.1	PKIG
TC20000334.hg.1	2.31	JUC20002618.hg.1	PKIG
TC20000334.hg.1	2.29	PSR20004730.hg.1	PKIG
TC20000334.hg.1	2.06	JUC20002619.hg.1	PKIG
TC21000111.hg.1	2.69	JUC21000409.hg.1	SOD1
TC21000111.hg.1	2.15	PSR21000699.hg.1	SOD1
TC21000111.hg.1	2.1	PSR21000689.hg.1	SOD1
TC01002357.hg.1	2.68	PSR01036387.hg.1	MYOM3
TC01002357.hg.1	-2.26	JUC01019598.hg.1	MYOM3
TC01002357.hg.1	-2.74	JUC01019629.hg.1	MYOM3
TC01002357.hg.1	-3.28	JUC01019631.hg.1	MYOM3
TC01002357.hg.1	-3.74	JUC01019590.hg.1	MYOM3
TC03001285.hg.1	2.68	JUC03011001.hg.1	EPM2AIP1

TC03001285.hg.1	-2.16	PSR03022034.hg.1	EPM2AIP1
TC05000412.hg.1	2.68	JUC05003231.hg.1	VCAN
TC05000412.hg.1	2.57	JUC05003230.hg.1	VCAN
TC05000412.hg.1	-2.58	PSR05005944.hg.1	VCAN
TC05000412.hg.1	-3.16	JUC05003240.hg.1	VCAN
TC05000412.hg.1	-4.54	JUC05003233.hg.1	VCAN
TC05002123.hg.1	2.68	JUC05015027.hg.1	COL23A1
TC05002123.hg.1	2.39	JUC05015041.hg.1	COL23A1
TC05002123.hg.1	2.07	JUC05015066.hg.1	COL23A1
TC05002123.hg.1	-2.35	JUC05015046.hg.1	COL23A1
TC05002123.hg.1	-2.38	PSR05029513.hg.1	COL23A1
TC07001186.hg.1	2.68	JUC07009110.hg.1	STEAP1B
TC07001186.hg.1	-2.09	PSR07018597.hg.1	STEAP1B
TC07001186.hg.1	-2.46	JUC07009103.hg.1	STEAP1B
TC09001199.hg.1	2.68	JUC09008222.hg.1	LOC1005072
TC09001199.hg.1	2.51	JUC09008225.hg.1	LOC1005072
TC09001199.hg.1	2.18	PSR09015335.hg.1	LOC1005072
TC11000622.hg.1	2.68	JUC11003999.hg.1	POLA2
TC11000622.hg.1	2.2	JUC11003993.hg.1	POLA2
TC11000622.hg.1	-2.04	PSR11007733.hg.1	POLA2
TC11001396.hg.1	2.68	JUC11009406.hg.1	SCUBE2
TC11001396.hg.1	2.36	JUC11009393.hg.1	SCUBE2
TC11001396.hg.1	2.27	PSR11017592.hg.1	SCUBE2
TC11001396.hg.1	2.2	PSR11017598.hg.1	SCUBE2
TC11001396.hg.1	-2.05	PSR11017616.hg.1	SCUBE2
TC11001396.hg.1	-2.56	JUC11009408.hg.1	SCUBE2
TC11001396.hg.1	-3.1	JUC11009411.hg.1	SCUBE2
TC14002300.hg.1	2.68	JUC14011731.hg.1	FNTB, CHUR
TC14002300.hg.1	2.05	PSR14004554.hg.1	FNTB, CHUR
TC14002300.hg.1	-2.04	JUC14011740.hg.1	FNTB, CHUR
TC14002300.hg.1	-2.51	JUC14011739.hg.1	FNTB, CHUR
TC01001329.hg.1	2.67	JUC01011120.hg.1	PEAR1
TC01001329.hg.1	-2.17	JUC01011131.hg.1	PEAR1
TC01001329.hg.1	-2.73	PSR01020923.hg.1	PEAR1
TC01001329.hg.1	-3.09	JUC01011123.hg.1	PEAR1
TC01006322.hg.1	2.67	JUC01043999.hg.1	TTC4
TC01006322.hg.1	2.17	JUC01044003.hg.1	TTC4
TC01006322.hg.1	2.05	PSR01010889.hg.1	TTC4
TC01006322.hg.1	-2.75	JUC01044002.hg.1	TTC4
TC02005056.hg.1	2.67	JUC02033211.hg.1	ABCB6
TC02005056.hg.1	2.51	JUC02033208.hg.1	ABCB6
TC02005056.hg.1	2.5	PSR02044964.hg.1	ABCB6
TC02005056.hg.1	2.33	PSR02044961.hg.1	ABCB6
TC02005056.hg.1	2.32	PSR02044976.hg.1	ABCB6
TC02005056.hg.1	2.29	PSR02044978.hg.1	ABCB6
TC02005056.hg.1	2.25	PSR02044940.hg.1	ABCB6
TC02005056.hg.1	2.11	PSR02044949.hg.1	ABCB6
TC02005056.hg.1	-2.12	JUC02033207.hg.1	ABCB6
TC02005056.hg.1	-2.56	JUC02033222.hg.1	ABCB6
TC05000202.hg.1	2.67	JUC05001365.hg.1	NNT
TC05000202.hg.1	2.42	PSR05002409.hg.1	NNT
TC05000202.hg.1	2.28	JUC05001386.hg.1	NNT
TC06004120.hg.1	2.67	JUC06021682.hg.1	TNXB
TC06004120.hg.1	2.48	PSR06018250.hg.1	TNXB
TC06004120.hg.1	-2.16	JUC06021652.hg.1	TNXB
TC06004120.hg.1	-2.48	PSR06018245.hg.1	TNXB
TC07000498.hg.1	2.67	JUC07003366.hg.1	PHTF2
TC07000498.hg.1	-2.11	PSR07007079.hg.1	PHTF2

TC07000498.hg.1	-3.2	JUC07003357.hg.1	PHTF2
TC0X001323.hg.1	2.67	JUC0X009033.hg.1	NKAPP1
TC0X001323.hg.1	2.05	PSR0X017749.hg.1	NKAPP1
TC11000389.hg.1	2.67	JUC11002583.hg.1	MAPK8IP1, L
TC11000389.hg.1	2.61	JUC11002584.hg.1	MAPK8IP1, L
TC11000389.hg.1	2.53	PSR11004832.hg.1	MAPK8IP1, L
TC11000389.hg.1	2.07	PSR11004841.hg.1	MAPK8IP1, L
TC11000389.hg.1	2	PSR11004831.hg.1	MAPK8IP1, L
TC11000389.hg.1	-2.61	JUC11002587.hg.1	MAPK8IP1, L
TC11000389.hg.1	-2.71	JUC11002581.hg.1	MAPK8IP1, L
TC11000389.hg.1	-4.11	PSR11004845.hg.1	MAPK8IP1, L
TC11001389.hg.1	2.67	PSR11017522.hg.1	ST5
TC11001389.hg.1	2.6	JUC11009348.hg.1	ST5
TC11001389.hg.1	-2.33	JUC11009340.hg.1	ST5
TC11002030.hg.1	2.67	JUC11012798.hg.1	SHANK2
TC11002030.hg.1	2.15	JUC11012820.hg.1	SHANK2
TC11002030.hg.1	2.12	JUC11012819.hg.1	SHANK2
TC11002030.hg.1	2.03	JUC11012812.hg.1	SHANK2
TC11002030.hg.1	2	PSR11024309.hg.1	SHANK2
TC11002030.hg.1	-2.16	PSR11024347.hg.1	SHANK2
TC11002030.hg.1	-2.26	PSR11024342.hg.1	SHANK2
TC13000362.hg.1	2.67	JUC13002376.hg.1	PCCA
TC13000362.hg.1	-2.08	PSR13003854.hg.1	PCCA
TC14000627.hg.1	2.67	JUC14003958.hg.1	CYP46A1
TC14000627.hg.1	-2.05	JUC14003957.hg.1	CYP46A1
TC14000627.hg.1	-2.42	PSR14007705.hg.1	CYP46A1
TC14001310.hg.1	2.67	JUC14007565.hg.1	KIAA0317
TC14001310.hg.1	-2.17	JUC14007569.hg.1	KIAA0317
TC14001310.hg.1	-2.28	JUC14007552.hg.1	KIAA0317
TC14001310.hg.1	-2.33	PSR14014923.hg.1	KIAA0317
TC14001310.hg.1	-2.84	JUC14007545.hg.1	KIAA0317
TC15000290.hg.1	2.67	JUC15000941.hg.1	CASC5
TC15000290.hg.1	2.05	PSR15002005.hg.1	CASC5
TC15000290.hg.1	-2.12	JUC15000933.hg.1	CASC5
TC15000290.hg.1	-2.26	JUC15000923.hg.1	CASC5
TC15000290.hg.1	-2.31	PSR15001996.hg.1	CASC5
TC15000290.hg.1	-2.35	PSR15001987.hg.1	CASC5
TC15000290.hg.1	-2.46	JUC15000929.hg.1	CASC5
TC17000252.hg.1	2.67	JUC17002016.hg.1	CCDC144C
TC17000252.hg.1	2.56	JUC17002015.hg.1	CCDC144C
TC17000252.hg.1	2.48	JUC17002002.hg.1	CCDC144C
TC17000252.hg.1	2.4	PSR17003510.hg.1	CCDC144C
TC17000252.hg.1	2.27	PSR17003514.hg.1	CCDC144C
TC17000252.hg.1	2.18	PSR17003515.hg.1	CCDC144C
TC17000252.hg.1	2.17	PSR17003513.hg.1	CCDC144C
TC17000252.hg.1	2.11	JUC17002004.hg.1	CCDC144C
TC17000252.hg.1	-2.33	JUC17002020.hg.1	CCDC144C
TC19000009.hg.1	2.67	JUC19000037.hg.1	BSG
TC19000009.hg.1	2.5	JUC19000040.hg.1	BSG
TC19000009.hg.1	2.1	PSR19000064.hg.1	BSG
TC19000177.hg.1	2.67	JUC19001574.hg.1	PDE4A
TC19000177.hg.1	2.07	PSR19002671.hg.1	PDE4A
TC01000398.hg.1	2.66	JUC01003181.hg.1	PTPRU
TC01000398.hg.1	2.14	JUC01003209.hg.1	PTPRU
TC01000398.hg.1	2.11	PSR01006105.hg.1	PTPRU
TC01000398.hg.1	2	JUC01003185.hg.1	PTPRU
TC01000777.hg.1	2.66	JUC01006508.hg.1	RABGGTB, S
TC01000777.hg.1	-2.05	PSR01012423.hg.1	RABGGTB, S

TC01000777.hg.1	-2.37	PSR01012392.hg.1	RABGGTB, S
TC01000777.hg.1	-3.59	PSR01012394.hg.1	RABGGTB, S
TC02001576.hg.1	2.66	JUC02013221.hg.1	ROCK2
TC02001576.hg.1	2.51	PSR02025428.hg.1	ROCK2
TC02001576.hg.1	2.33	PSR02025460.hg.1	ROCK2
TC02002424.hg.1	2.66	PSR02038045.hg.1	RBM43
TC02002424.hg.1	2.48	PSR02038048.hg.1	RBM43
TC02002424.hg.1	2.09	JUC02019764.hg.1	RBM43
TC02002424.hg.1	-2.05	JUC02019766.hg.1	RBM43
TC03000519.hg.1	2.66	PSR03009671.hg.1	PCNP
TC03000519.hg.1	-2.88	JUC03004832.hg.1	PCNP
TC05001873.hg.1	2.66	JUC05013195.hg.1	FCHSD1
TC05001873.hg.1	-2.37	PSR05025848.hg.1	FCHSD1
TC06001962.hg.1	2.66	PSR06024176.hg.1	FAXC
TC06001962.hg.1	2.63	JUC06011838.hg.1	FAXC
TC06001962.hg.1	2.27	PSR06024161.hg.1	FAXC
TC06001962.hg.1	2.25	PSR06024167.hg.1	FAXC
TC06001962.hg.1	2.18	PSR06024175.hg.1	FAXC
TC06001962.hg.1	2.1	JUC06011836.hg.1	FAXC
TC07001344.hg.1	2.66	JUC07010257.hg.1	TMED4
TC07001344.hg.1	2.11	PSR07020961.hg.1	TMED4
TC08000326.hg.1	2.66	JUC08002378.hg.1	POLB
TC08000326.hg.1	2.63	JUC08002383.hg.1	POLB
TC08000326.hg.1	2.21	JUC08002389.hg.1	POLB
TC08000326.hg.1	2.14	PSR08004737.hg.1	POLB
TC08000326.hg.1	-3.52	PSR08004719.hg.1	POLB
TC11001017.hg.1	2.66	JUC11006839.hg.1	TTC12
TC11001017.hg.1	2.26	JUC11006858.hg.1	TTC12
TC11001017.hg.1	2.17	PSR11012764.hg.1	TTC12
TC11001017.hg.1	2.15	JUC11006841.hg.1	TTC12
TC11001017.hg.1	2.13	PSR11012715.hg.1	TTC12
TC11001017.hg.1	2.13	PSR11012723.hg.1	TTC12
TC11001017.hg.1	2.12	JUC11006835.hg.1	TTC12
TC11001017.hg.1	2.03	PSR11012757.hg.1	TTC12
TC11001017.hg.1	2	PSR11012722.hg.1	TTC12
TC11001017.hg.1	-2.28	JUC11006838.hg.1	TTC12
TC11001017.hg.1	-5.34	PSR11012720.hg.1	TTC12
TC11002334.hg.1	2.66	PSR11027894.hg.1	PCSK7
TC11002334.hg.1	-2.12	PSR11027910.hg.1	PCSK7
TC11002334.hg.1	-3.03	JUC11014885.hg.1	PCSK7
TC15000766.hg.1	2.66	JUC15003603.hg.1	UBE2Q2P2, U
TC15000766.hg.1	2.32	PSR15007033.hg.1	UBE2Q2P2, U
TC15000766.hg.1	-2.76	JUC15003602.hg.1	UBE2Q2P2, U
TC15000766.hg.1	-2.8	PSR15007029.hg.1	UBE2Q2P2, U
TC16000442.hg.1	2.66	JUC16003384.hg.1	NOD2
TC16000442.hg.1	2.32	JUC16003394.hg.1	NOD2
TC16000442.hg.1	2.13	JUC16003386.hg.1	NOD2
TC16000442.hg.1	2	PSR16006117.hg.1	NOD2
TC02001115.hg.1	2.65	PSR02016864.hg.1	C2orf88
TC02001115.hg.1	2.03	PSR02016868.hg.1	C2orf88
TC02001115.hg.1	-2.7	PSR02016862.hg.1	C2orf88
TC02001115.hg.1	-3.14	PSR02016845.hg.1	C2orf88
TC02001115.hg.1	-3.77	PSR02016844.hg.1	C2orf88
TC02001115.hg.1	-3.94	PSR02016855.hg.1	C2orf88
TC02001115.hg.1	-4.26	PSR02016848.hg.1	C2orf88
TC02001115.hg.1	-4.42	PSR02016843.hg.1	C2orf88
TC02001115.hg.1	-4.43	PSR02016851.hg.1	C2orf88
TC02001115.hg.1	-4.74	PSR02016858.hg.1	C2orf88

TC02001115.hg.1	-5.09	JUC02009032.hg.1	C2orf88
TC02001115.hg.1	-5.16	PSR02016849.hg.1	C2orf88
TC02001419.hg.1	2.65	PSR02022671.hg.1	DGKD
TC02001419.hg.1	2.49	JUC02011903.hg.1	DGKD
TC02001419.hg.1	2.42	PSR02022630.hg.1	DGKD
TC02001419.hg.1	2.17	PSR02022626.hg.1	DGKD
TC02001419.hg.1	2.13	PSR02022663.hg.1	DGKD
TC02001419.hg.1	2.07	PSR02022624.hg.1	DGKD
TC02001419.hg.1	-2.01	JUC02011911.hg.1	DGKD
TC02001419.hg.1	-2.22	PSR02022640.hg.1	DGKD
TC02001419.hg.1	-2.71	JUC02011915.hg.1	DGKD
TC02001419.hg.1	-2.83	JUC02011909.hg.1	DGKD
TC02002808.hg.1	2.65	JUC02023679.hg.1	EPHA4
TC02002808.hg.1	2.31	JUC02023687.hg.1	EPHA4
TC02002808.hg.1	2.07	JUC02023674.hg.1	EPHA4
TC02002808.hg.1	-2.08	JUC02023697.hg.1	EPHA4
TC02002808.hg.1	-2.13	PSR02045378.hg.1	EPHA4
TC02002808.hg.1	-2.28	PSR02045373.hg.1	EPHA4
TC02002808.hg.1	-2.97	PSR02045385.hg.1	EPHA4
TC02002808.hg.1	-3.03	JUC02023691.hg.1	EPHA4
TC02002808.hg.1	-3.06	JUC02023689.hg.1	EPHA4
TC02002808.hg.1	-3.36	PSR02045366.hg.1	EPHA4
TC02002808.hg.1	-3.43	PSR02045344.hg.1	EPHA4
TC03000543.hg.1	2.65	JUC03004969.hg.1	BBX
TC03000543.hg.1	2.63	PSR03009934.hg.1	BBX
TC03000543.hg.1	2.17	JUC03004940.hg.1	BBX
TC03000543.hg.1	2.15	PSR03009933.hg.1	BBX
TC03000543.hg.1	2.04	PSR03009932.hg.1	BBX
TC03001928.hg.1	2.65	JUC03017108.hg.1	C3orf33
TC03001928.hg.1	2.23	JUC03017110.hg.1	C3orf33
TC03001928.hg.1	-2.85	PSR03034559.hg.1	C3orf33
TC07000789.hg.1	2.65	PSR07012473.hg.1	IRF5
TC07000789.hg.1	2.14	JUC07005886.hg.1	IRF5
TC07000789.hg.1	2.07	PSR07012477.hg.1	IRF5
TC07001405.hg.1	2.65	JUC07010711.hg.1	14-Sep
TC07001405.hg.1	-2.05	PSR07021792.hg.1	14-Sep
TC08000125.hg.1	2.65	JUC08000601.hg.1	ZDHHC2
TC08000125.hg.1	2.2	PSR08001128.hg.1	ZDHHC2
TC08000125.hg.1	-3.12	JUC08000610.hg.1	ZDHHC2
TC09001686.hg.1	2.65	JUC09011597.hg.1	MED22
TC09001686.hg.1	2.15	PSR09021605.hg.1	MED22
TC0X000628.hg.1	2.65	JUC0X004197.hg.1	MST4
TC0X000628.hg.1	2.43	PSR0X008291.hg.1	MST4
TC0X000628.hg.1	2.38	PSR0X008292.hg.1	MST4
TC0X000628.hg.1	2.38	JUC0X004191.hg.1	MST4
TC0X000628.hg.1	2.31	PSR0X008299.hg.1	MST4
TC0X000628.hg.1	-2	JUC0X004186.hg.1	MST4
TC12003234.hg.1	2.65	JUC12019815.hg.1	FAM66C
TC12003234.hg.1	2.6	PSR12001673.hg.1	FAM66C
TC12003234.hg.1	2.37	JUC12019807.hg.1	FAM66C
TC12003234.hg.1	2.33	PSR12001670.hg.1	FAM66C
TC12003234.hg.1	2.28	PSR12001676.hg.1	FAM66C
TC12003234.hg.1	2.26	PSR12001672.hg.1	FAM66C
TC12003234.hg.1	2.12	PSR12001671.hg.1	FAM66C
TC12003234.hg.1	2.07	PSR12001647.hg.1	FAM66C
TC12003234.hg.1	-2.01	PSR12001659.hg.1	FAM66C
TC15000306.hg.1	2.65	JUC15001044.hg.1	NUSAP1
TC15000306.hg.1	2.47	PSR15002208.hg.1	NUSAP1

TC15000306.hg.1	2.21	PSR15002222.hg.1	NUSAP1
TC15000306.hg.1	2.13	JUC15001043.hg.1	NUSAP1
TC15000306.hg.1	-2.54	JUC15001054.hg.1	NUSAP1
TC15001274.hg.1	2.65	JUC15006412.hg.1	TGM5
TC15001274.hg.1	2.14	JUC15006418.hg.1	TGM5
TC15001274.hg.1	-2.71	PSR15011830.hg.1	TGM5
TC6_apd_hap1000	2.65	PSR6_apd_hap1000	HLA-A
TC6_apd_hap1000	2.58	PSR6_apd_hap1000	HLA-A
TC6_apd_hap1000	2.25	PSR6_apd_hap1000	HLA-A
TC6_apd_hap1000	2.1	PSR6_apd_hap1000	HLA-A
TC6_apd_hap1000	2.05	PSR6_apd_hap1000	HLA-A
TC6_apd_hap1000	2.02	PSR6_apd_hap1000	HLA-A
TC6_apd_hap1000	-2.25	JUC6_apd_hap1000(	HLA-A
TC6_apd_hap1000	-2.64	JUC6_apd_hap1000(	HLA-A
TC6_apd_hap1000	-4.42	JUC6_apd_hap1000(	HLA-A
TC6_apd_hap1000	-5.03	PSR6_apd_hap1000	HLA-A
TC6_apd_hap1000	2.65	PSR6_apd_hap1000!	ZNRD1-AS1
TC6_apd_hap1000	2.34	PSR6_apd_hap1000	ZNRD1-AS1
TC6_apd_hap1000	2.26	PSR6_apd_hap1000	ZNRD1-AS1
TC6_apd_hap1000	2.23	PSR6_apd_hap1000	ZNRD1-AS1
TC6_apd_hap1000	2.01	PSR6_apd_hap1000	ZNRD1-AS1
TC6_apd_hap1000	-2.11	JUC6_apd_hap1000	ZNRD1-AS1
TC6_apd_hap1000	-2.24	JUC6_apd_hap1000	ZNRD1-AS1
TC6_apd_hap1000	-2.33	PSR6_apd_hap1000	ZNRD1-AS1
TC6_apd_hap1000	-3.71	JUC6_apd_hap1000	ZNRD1-AS1
TC6_cox_hap2000	2.65	PSR6_cox_hap2000	HLA-A
TC6_cox_hap2000	2.58	PSR6_cox_hap2000	HLA-A
TC6_cox_hap2000	2.25	PSR6_cox_hap2000	HLA-A
TC6_cox_hap2000	2.1	PSR6_cox_hap2000	HLA-A
TC6_cox_hap2000	2.05	PSR6_cox_hap2000	HLA-A
TC6_cox_hap2000	2.02	PSR6_cox_hap2000	HLA-A
TC6_cox_hap2000	-2.25	JUC6_cox_hap20001	HLA-A
TC6_cox_hap2000	-2.64	JUC6_cox_hap20001	HLA-A
TC6_cox_hap2000	-4.42	JUC6_cox_hap20001	HLA-A
TC6_cox_hap2000	-5.03	PSR6_cox_hap2000	HLA-A
TC6_cox_hap2000	2.65	PSR6_cox_hap2002	ZNRD1-AS1
TC6_cox_hap2000	2.34	PSR6_cox_hap2002	ZNRD1-AS1
TC6_cox_hap2000	2.26	PSR6_cox_hap2002	ZNRD1-AS1
TC6_cox_hap2000	2.23	PSR6_cox_hap2002	ZNRD1-AS1
TC6_cox_hap2000	2.01	PSR6_cox_hap2002	ZNRD1-AS1
TC6_cox_hap2000	-2.11	JUC6_cox_hap20007	ZNRD1-AS1
TC6_cox_hap2000	-2.24	JUC6_cox_hap20007	ZNRD1-AS1
TC6_cox_hap2000	-2.33	PSR6_cox_hap2002	ZNRD1-AS1
TC6_cox_hap2000	-3.71	JUC6_cox_hap20007	ZNRD1-AS1
TC6_dbb_hap3000	2.65	PSR6_dbb_hap3002	ZNRD1-AS1
TC6_dbb_hap3000	2.34	PSR6_dbb_hap3002	ZNRD1-AS1
TC6_dbb_hap3000	2.26	PSR6_dbb_hap3002	ZNRD1-AS1
TC6_dbb_hap3000	2.23	PSR6_dbb_hap3002	ZNRD1-AS1
TC6_dbb_hap3000	2.01	PSR6_dbb_hap3002	ZNRD1-AS1
TC6_dbb_hap3000	-2.11	JUC6_dbb_hap3000	ZNRD1-AS1
TC6_dbb_hap3000	-2.24	JUC6_dbb_hap3000	ZNRD1-AS1
TC6_dbb_hap3000	-2.33	PSR6_dbb_hap3002	ZNRD1-AS1
TC6_dbb_hap3000	-3.71	JUC6_dbb_hap3000	ZNRD1-AS1
TC6_mann_hap40(	2.65	PSR6_mann_hap400	ZNRD1-AS1
TC6_mann_hap40(	2.47	PSR6_mann_hap400	ZNRD1-AS1
TC6_mann_hap40(	2.26	PSR6_mann_hap400	ZNRD1-AS1
TC6_mann_hap40(	2.26	PSR6_mann_hap400	ZNRD1-AS1
TC6_mann_hap40(	2.01	PSR6_mann_hap400	ZNRD1-AS1

TC6_mann_hap40(	-2.11	JUC6_mann_hap400	ZNRD1-AS1
TC6_mann_hap40(	-2.33	PSR6_mann_hap40(	ZNRD1-AS1
TC6_mann_hap40(	-3.71	JUC6_mann_hap400	ZNRD1-AS1
TC6_mcf_hap5000	2.65	PSR6_mcf_hap5001!	ZNRD1-AS1
TC6_mcf_hap5000	2.26	PSR6_mcf_hap5001!	ZNRD1-AS1
TC6_mcf_hap5000	2.23	PSR6_mcf_hap5001!	ZNRD1-AS1
TC6_mcf_hap5000	2.11	PSR6_mcf_hap5001!	ZNRD1-AS1
TC6_mcf_hap5000	2.1	PSR6_mcf_hap5001!	ZNRD1-AS1
TC6_mcf_hap5000	2.01	PSR6_mcf_hap5001!	ZNRD1-AS1
TC6_mcf_hap5000	-2.11	JUC6_mcf_hap5000(	ZNRD1-AS1
TC6_mcf_hap5000	-2.33	PSR6_mcf_hap5001!	ZNRD1-AS1
TC6_mcf_hap5000	-3.71	JUC6_mcf_hap5000(	ZNRD1-AS1
TC05000830.hg.1	2.65	JUC05006235.hg.1	TCOF1
TC05000830.hg.1	2.12	JUC05006216.hg.1	TCOF1
TC05000830.hg.1	2	PSR05011912.hg.1	TCOF1
TC05000830.hg.1	-2.21	JUC05006217.hg.1	TCOF1
TC05000830.hg.1	-2.25	JUC05006199.hg.1	TCOF1
TC05000830.hg.1	-2.71	JUC05006226.hg.1	TCOF1
TC01001688.hg.1	2.64	JUC01014045.hg.1	ATP2B4
TC01001688.hg.1	2.1	PSR01026233.hg.1	ATP2B4
TC01001688.hg.1	2.01	PSR01026239.hg.1	ATP2B4
TC01001688.hg.1	-2.05	JUC01014035.hg.1	ATP2B4
TC01002577.hg.1	2.64	PSR01040052.hg.1	
TC01002577.hg.1	2.27	JUC01021505.hg.1	
TC01002577.hg.1	2.19	PSR01040046.hg.1	
TC01002577.hg.1	2.15	JUC01021504.hg.1	
TC01002577.hg.1	2.08	PSR01040048.hg.1	
TC01002710.hg.1	2.64	JUC01022735.hg.1	
TC01002710.hg.1	-2.2	PSR01042593.hg.1	
TC02001004.hg.1	2.64	JUC02007848.hg.1	PPIG
TC02001004.hg.1	2.4	JUC02007856.hg.1	PPIG
TC02001004.hg.1	2.08	PSR02014714.hg.1	PPIG
TC02001004.hg.1	2.06	JUC02007858.hg.1	PPIG
TC02001004.hg.1	-3.17	JUC02007845.hg.1	PPIG
TC04000649.hg.1	2.64	JUC04005055.hg.1	LOC1005074
TC04000649.hg.1	-2.07	PSR04009455.hg.1	LOC1005074
TC04000649.hg.1	-2.15	PSR04009439.hg.1	LOC1005074
TC04000649.hg.1	-2.31	JUC04005066.hg.1	LOC1005074
TC04000649.hg.1	-2.49	PSR04009441.hg.1	LOC1005074
TC04000649.hg.1	-2.65	PSR04009438.hg.1	LOC1005074
TC04000649.hg.1	-2.77	PSR04009450.hg.1	LOC1005074
TC04000649.hg.1	-2.8	PSR04009437.hg.1	LOC1005074
TC04000649.hg.1	-3.79	PSR04009436.hg.1	LOC1005074
TC04000649.hg.1	-4.13	JUC04005059.hg.1	LOC1005074
TC0X000118.hg.1	2.64	PSR0X001593.hg.1	PDK3
TC0X000118.hg.1	-4.4	JUC0X000867.hg.1	PDK3
TC0X000494.hg.1	2.64	PSR0X006563.hg.1	TCEAL7
TC0X000494.hg.1	-2.89	JUC0X003237.hg.1	TCEAL7
TC11002345.hg.1	2.64	JUC11015010.hg.1	MPZL3
TC11002345.hg.1	2.04	PSR11028152.hg.1	MPZL3
TC12001040.hg.1	2.64	JUC12007493.hg.1	GPR133
TC12001040.hg.1	-2.31	JUC12007484.hg.1	GPR133
TC12001040.hg.1	-2.37	PSR12013660.hg.1	GPR133
TC12001040.hg.1	-2.37	JUC12007500.hg.1	GPR133
TC12001040.hg.1	-2.47	JUC12007503.hg.1	GPR133
TC12001040.hg.1	-2.78	PSR12013662.hg.1	GPR133
TC12001040.hg.1	-3.15	PSR12013664.hg.1	GPR133
TC14000219.hg.1	2.64	PSR14002347.hg.1	SRP54

TC14000219.hg.1	2.44	PSR14002325.hg.1	SRP54
TC14000219.hg.1	2.18	JUC14001059.hg.1	SRP54
TC14000219.hg.1	2.03	PSR14002324.hg.1	SRP54
TC14000219.hg.1	-2.15	JUC14001065.hg.1	SRP54
TC14000358.hg.1	2.64	JUC14001966.hg.1	JKAMP
TC14000358.hg.1	2.44	PSR14003902.hg.1	JKAMP
TC14000358.hg.1	2.06	JUC14001958.hg.1	JKAMP
TC14000358.hg.1	-2.15	PSR14003921.hg.1	JKAMP
TC17000601.hg.1	2.64	JUC17004166.hg.1	WNT9B
TC17000601.hg.1	2.33	PSR17007371.hg.1	WNT9B
TC17000601.hg.1	2.22	PSR17007369.hg.1	WNT9B
TC17000601.hg.1	2.08	PSR17007367.hg.1	WNT9B
TC18000223.hg.1	2.64	JUC18001762.hg.1	ZCCHC2
TC18000223.hg.1	2.16	PSR18002555.hg.1	ZCCHC2
TC19000087.hg.1	2.64	JUC19000793.hg.1	MPND
TC19000087.hg.1	-2.01	PSR19001395.hg.1	MPND
TC19000087.hg.1	-2.38	JUC19000798.hg.1	MPND
TC21000097.hg.1	2.64	PSR21000592.hg.1	GRIK1-AS2, [illegible]
TC21000097.hg.1	2.64	PSR21000619.hg.1	GRIK1-AS2, [illegible]
TC21000097.hg.1	2.42	JUC21000380.hg.1	GRIK1-AS2, [illegible]
TC21000097.hg.1	2.38	PSR21000622.hg.1	GRIK1-AS2, [illegible]
TC21000097.hg.1	2.17	JUC21000375.hg.1	GRIK1-AS2, [illegible]
TC21000097.hg.1	2.07	PSR21000596.hg.1	GRIK1-AS2, [illegible]
TC21000097.hg.1	2.06	JUC21000389.hg.1	GRIK1-AS2, [illegible]
TC21000097.hg.1	2	PSR21000594.hg.1	GRIK1-AS2, [illegible]
TC21000097.hg.1	-3.03	JUC21000377.hg.1	GRIK1-AS2, [illegible]
TC22001476.hg.1	2.64	PSR22006894.hg.1	SERHL2
TC22001476.hg.1	2.48	JUC22009452.hg.1	SERHL2
TC22001476.hg.1	2.43	PSR22006892.hg.1	SERHL2
TC22001476.hg.1	2.41	JUC22009441.hg.1	SERHL2
TC22001476.hg.1	2.04	PSR22006899.hg.1	SERHL2
TC22001476.hg.1	2.02	PSR22006918.hg.1	SERHL2
TC22001476.hg.1	2.02	PSR22006920.hg.1	SERHL2
TC22001476.hg.1	2	PSR22006919.hg.1	SERHL2
TC22001476.hg.1	-2.19	JUC22009448.hg.1	SERHL2
TC22001476.hg.1	-2.45	JUC22009443.hg.1	SERHL2
TC22001476.hg.1	-2.71	PSR22006907.hg.1	SERHL2
TC6_ssto_hap700[illegible]	2.64	PSR6_ssto_hap7002	ZNRD1-AS1
TC6_ssto_hap700[illegible]	2.33	PSR6_ssto_hap7001	ZNRD1-AS1
TC6_ssto_hap700[illegible]	2.25	PSR6_ssto_hap7001	ZNRD1-AS1
TC6_ssto_hap700[illegible]	2.23	PSR6_ssto_hap7001	ZNRD1-AS1
TC6_ssto_hap700[illegible]	2	PSR6_ssto_hap7001	ZNRD1-AS1
TC6_ssto_hap700[illegible]	-2.12	JUC6_ssto_hap7000	ZNRD1-AS1
TC6_ssto_hap700[illegible]	-2.25	JUC6_ssto_hap7000	ZNRD1-AS1
TC6_ssto_hap700[illegible]	-2.34	PSR6_ssto_hap7001	ZNRD1-AS1
TC6_ssto_hap700[illegible]	-3.72	JUC6_ssto_hap7000	ZNRD1-AS1
TC01003341.hg.1	2.64	JUC01027308.hg.1	MEF2D
TC01003341.hg.1	2.53	JUC01027305.hg.1	MEF2D
TC01003341.hg.1	2	PSR01052183.hg.1	MEF2D
TC01001095.hg.1	2.63	JUC01008793.hg.1	ITGA10
TC01001095.hg.1	2.07	JUC01008791.hg.1	ITGA10
TC01001095.hg.1	-2.02	JUC01008797.hg.1	ITGA10
TC01001095.hg.1	-2.2	JUC01008799.hg.1	ITGA10
TC01001095.hg.1	-2.4	JUC01008790.hg.1	ITGA10
TC01001095.hg.1	-2.68	JUC01008798.hg.1	ITGA10
TC01001095.hg.1	-2.7	JUC01008819.hg.1	ITGA10
TC01001095.hg.1	-3.25	JUC01008818.hg.1	ITGA10
TC01001095.hg.1	-3.66	PSR01017234.hg.1	ITGA10

TC01001095.hg.1	-4.71	JUC01008789.hg.1	ITGA10
TC01001095.hg.1	-6.3	PSR01017274.hg.1	ITGA10
TC01001095.hg.1	-6.39	JUC01008817.hg.1	ITGA10
TC02000662.hg.1	2.63	PSR02010269.hg.1	SULT1C3
TC02000662.hg.1	2.44	JUC02005425.hg.1	SULT1C3
TC03000134.hg.1	2.63	JUC03001352.hg.1	OXSM
TC03000134.hg.1	-2.04	PSR03002536.hg.1	OXSM
TC03000134.hg.1	-3.09	PSR03002535.hg.1	OXSM
TC03000134.hg.1	-3.96	PSR03002527.hg.1	OXSM
TC03000594.hg.1	2.63	JUC03005406.hg.1	
TC03000594.hg.1	2.21	PSR03010837.hg.1	
TC03000594.hg.1	2.11	JUC03005405.hg.1	
TC03000594.hg.1	2.02	PSR03010835.hg.1	
TC03000594.hg.1	-2.03	JUC03005407.hg.1	
TC03001047.hg.1	2.63	JUC03009395.hg.1	IL1RAP
TC03001047.hg.1	-2.1	JUC03009396.hg.1	IL1RAP
TC03001047.hg.1	-2.38	PSR03018532.hg.1	IL1RAP
TC04001272.hg.1	2.63	JUC04009261.hg.1	GRSF1
TC04001272.hg.1	2.15	PSR04017353.hg.1	GRSF1
TC04002916.hg.1	2.63	JUC04016870.hg.1	TLR1
TC04002916.hg.1	2.31	PSR04015312.hg.1	TLR1
TC05001261.hg.1	2.63	JUC05009029.hg.1	PRLR
TC05001261.hg.1	2.45	JUC05009033.hg.1	PRLR
TC05001261.hg.1	2.06	PSR05017652.hg.1	PRLR
TC05001261.hg.1	2.05	JUC05009042.hg.1	PRLR
TC05001261.hg.1	-2.11	PSR05017642.hg.1	PRLR
TC05001261.hg.1	-2.13	PSR05017634.hg.1	PRLR
TC05001261.hg.1	-2.14	PSR05017668.hg.1	PRLR
TC05001261.hg.1	-2.28	PSR05017663.hg.1	PRLR
TC05001261.hg.1	-2.42	PSR05017671.hg.1	PRLR
TC05001261.hg.1	-3.37	JUC05009041.hg.1	PRLR
TC05001824.hg.1	2.63	JUC05012729.hg.1	NME5
TC05001824.hg.1	2.23	PSR05024808.hg.1	NME5
TC05001824.hg.1	2.23	JUC05012732.hg.1	NME5
TC05001824.hg.1	2.1	JUC05012733.hg.1	NME5
TC05001824.hg.1	2.03	JUC05012734.hg.1	NME5
TC05001824.hg.1	-2	PSR05024805.hg.1	NME5
TC05001824.hg.1	-2.01	PSR05024814.hg.1	NME5
TC05001824.hg.1	-2.81	PSR05024811.hg.1	NME5
TC08001264.hg.1	2.63	JUC08008403.hg.1	ASPH
TC08001264.hg.1	2.54	JUC08008389.hg.1	ASPH
TC08001264.hg.1	2.43	PSR08016314.hg.1	ASPH
TC08001264.hg.1	2.3	PSR08016260.hg.1	ASPH
TC08001264.hg.1	2.23	JUC08008367.hg.1	ASPH
TC08001264.hg.1	2.09	PSR08016272.hg.1	ASPH
TC08001264.hg.1	2.03	JUC08008364.hg.1	ASPH
TC08001264.hg.1	-2.28	JUC08008394.hg.1	ASPH
TC08002596.hg.1	2.63	PSR08000977.hg.1	LOC1005069
TC08002596.hg.1	2.29	PSR08000974.hg.1	LOC1005069
TC08002596.hg.1	2.29	PSR08000986.hg.1	LOC1005069
TC08002596.hg.1	-2.01	JUC08014944.hg.1	LOC1005069
TC09001517.hg.1	2.63	PSR09019044.hg.1	TNC
TC09001517.hg.1	-2.21	PSR09019026.hg.1	TNC
TC09001517.hg.1	-2.45	JUC09010277.hg.1	TNC
TC09001517.hg.1	-2.52	PSR09019022.hg.1	TNC
TC09001517.hg.1	-2.64	JUC09010282.hg.1	TNC
TC09001517.hg.1	-2.64	JUC09010289.hg.1	TNC
TC09001517.hg.1	-3.04	PSR09019016.hg.1	TNC

TC09001517.hg.1	-3.16	JUC09010302.hg.1	TNC
TC09001517.hg.1	-3.18	JUC09010298.hg.1	TNC
TC09001517.hg.1	-3.21	PSR09019046.hg.1	TNC
TC09001517.hg.1	-3.24	PSR09019023.hg.1	TNC
TC09001517.hg.1	-3.27	JUC09010316.hg.1	TNC
TC09001517.hg.1	-3.28	PSR09019017.hg.1	TNC
TC09001517.hg.1	-3.64	JUC09010283.hg.1	TNC
TC09001517.hg.1	-3.86	JUC09010276.hg.1	TNC
TC09001517.hg.1	-4.89	JUC09010278.hg.1	TNC
TC09001517.hg.1	-5.24	JUC09010292.hg.1	TNC
TC09001676.hg.1	2.63	JUC09011509.hg.1	AK8
TC09001676.hg.1	2.45	JUC09011518.hg.1	AK8
TC09001676.hg.1	-2.18	PSR09021398.hg.1	AK8
TC10000621.hg.1	2.63	JUC10003870.hg.1	PAPSS2
TC10000621.hg.1	2.15	PSR10006802.hg.1	PAPSS2
TC10000732.hg.1	2.63	JUC10004813.hg.1	KAZALD1
TC10000732.hg.1	-2.02	PSR10008641.hg.1	KAZALD1
TC15002812.hg.1	2.63	PSR15012026.hg.1	STRC
TC15002812.hg.1	2.54	JUC15014291.hg.1	STRC
TC15002812.hg.1	-2.67	JUC15014280.hg.1	STRC
TC16000991.hg.1	2.63	PSR16014293.hg.1	SULT1A1
TC16000991.hg.1	2.42	PSR16014294.hg.1	SULT1A1
TC16000991.hg.1	2.24	PSR16014289.hg.1	SULT1A1
TC16000991.hg.1	-2.2	PSR16014282.hg.1	SULT1A1
TC16000991.hg.1	-2.97	JUC16008049.hg.1	SULT1A1
TC16000991.hg.1	-3.64	JUC16008040.hg.1	SULT1A1
TC16000991.hg.1	-3.97	PSR16014284.hg.1	SULT1A1
TC16000991.hg.1	-4.51	JUC16008050.hg.1	SULT1A1
TC16000991.hg.1	-13.49	JUC16008048.hg.1	SULT1A1
TC20000083.hg.1	2.63	JUC20000614.hg.1	C20orf94
TC20000083.hg.1	2.3	JUC20000620.hg.1	C20orf94
TC20000083.hg.1	2.11	PSR20001189.hg.1	C20orf94
TC21000508.hg.1	2.63	JUC21003184.hg.1	HSF2BP
TC21000508.hg.1	2.07	PSR21006141.hg.1	HSF2BP
TC21000508.hg.1	-2.15	PSR21006144.hg.1	HSF2BP
TC21000508.hg.1	-3.3	JUC21003181.hg.1	HSF2BP
TC22000221.hg.1	2.63	PSR22004151.hg.1	SMTN
TC22000221.hg.1	2.61	JUC22001474.hg.1	SMTN
TC22000221.hg.1	2.49	PSR22004129.hg.1	SMTN
TC22000221.hg.1	2.23	JUC22001465.hg.1	SMTN
TC22000221.hg.1	2.2	JUC22001454.hg.1	SMTN
TC22000221.hg.1	2.16	JUC22001444.hg.1	SMTN
TC22000221.hg.1	2.11	PSR22004188.hg.1	SMTN
TC22000221.hg.1	2.11	JUC22001462.hg.1	SMTN
TC22000221.hg.1	2.09	JUC22001463.hg.1	SMTN
TC22000221.hg.1	2.07	PSR22004149.hg.1	SMTN
TC22000221.hg.1	2.03	PSR22004150.hg.1	SMTN
TC12001324.hg.1	2.63	JUC12009562.hg.1	BHLHE41
TC12001324.hg.1	2	PSR12017331.hg.1	BHLHE41
TC12001324.hg.1	-3.49	JUC12009563.hg.1	BHLHE41
TC01002304.hg.1	2.62	JUC01018974.hg.1	TMCO4
TC01002304.hg.1	-2.18	JUC01018972.hg.1	TMCO4
TC01002304.hg.1	-2.2	JUC01018960.hg.1	TMCO4
TC01002304.hg.1	-2.49	JUC01018970.hg.1	TMCO4
TC01002304.hg.1	-3.07	PSR01035308.hg.1	TMCO4
TC01002304.hg.1	-4.32	JUC01018983.hg.1	TMCO4
TC02001239.hg.1	2.62	JUC02010252.hg.1	UNC80
TC02001239.hg.1	2.37	JUC02010219.hg.1	UNC80

TC02001239.hg.1	2.07	JUC02010256.hg.1	UNC80
TC02001239.hg.1	-2.33	PSR02019086.hg.1	UNC80
TC02001239.hg.1	-2.33	JUC02010243.hg.1	UNC80
TC02001239.hg.1	-3.47	JUC02010222.hg.1	UNC80
TC02001239.hg.1	-4.49	JUC02010237.hg.1	UNC80
TC03003412.hg.1	2.62	JUC03024602.hg.1	NAT6
TC03003412.hg.1	2.31	PSR03025635.hg.1	NAT6
TC03003412.hg.1	2.12	PSR03025644.hg.1	NAT6
TC03003412.hg.1	2.03	PSR03025628.hg.1	NAT6
TC05000392.hg.1	2.62	JUC05002999.hg.1	CMYA5
TC05000392.hg.1	2.4	PSR05005548.hg.1	CMYA5
TC05000392.hg.1	2.28	PSR05005539.hg.1	CMYA5
TC05000392.hg.1	2	PSR05005550.hg.1	CMYA5
TC06000524.hg.1	2.62	PSR06005647.hg.1	MAPK13
TC06000524.hg.1	-2.01	JUC06002301.hg.1	MAPK13
TC07001305.hg.1	2.62	PSR07020284.hg.1	VPS41
TC07001305.hg.1	2.46	PSR07020295.hg.1	VPS41
TC07001305.hg.1	2.44	PSR07020292.hg.1	VPS41
TC07001305.hg.1	2.21	PSR07020294.hg.1	VPS41
TC07001305.hg.1	2.06	PSR07020232.hg.1	VPS41
TC07001305.hg.1	2.06	JUC07009940.hg.1	VPS41
TC07001305.hg.1	-2.28	JUC07009946.hg.1	VPS41
TC08000225.hg.1	2.62	JUC08001613.hg.1	HMBOX1
TC08000225.hg.1	2.13	JUC08001597.hg.1	HMBOX1
TC08000225.hg.1	-2.19	PSR08003285.hg.1	HMBOX1
TC08000225.hg.1	-2.38	JUC08001603.hg.1	HMBOX1
TC08000750.hg.1	2.62	JUC08005184.hg.1	MIR1204, PV
TC08000750.hg.1	2.16	JUC08005186.hg.1	MIR1204, PV
TC08000750.hg.1	2.03	PSR08010112.hg.1	MIR1204, PV
TC08000750.hg.1	2	JUC08005196.hg.1	MIR1204, PV
TC08001423.hg.1	2.62	PSR08018463.hg.1	TRIQK
TC08001423.hg.1	2.16	JUC08009396.hg.1	TRIQK
TC08001423.hg.1	2.07	PSR08018464.hg.1	TRIQK
TC08001423.hg.1	-2.02	JUC08009399.hg.1	TRIQK
TC08001423.hg.1	-2.36	JUC08009381.hg.1	TRIQK
TC11001185.hg.1	2.62	JUC11008073.hg.1	FLI1
TC11001185.hg.1	2.61	JUC11008071.hg.1	FLI1
TC11001185.hg.1	2.26	JUC11008070.hg.1	FLI1
TC11001185.hg.1	2.23	PSR11014932.hg.1	FLI1
TC15000780.hg.1	2.62	JUC15003715.hg.1	UBE2Q2P2
TC15000780.hg.1	2.28	PSR15007168.hg.1	UBE2Q2P2
TC15000780.hg.1	-2.8	JUC15003714.hg.1	UBE2Q2P2
TC15000780.hg.1	-2.84	PSR15007164.hg.1	UBE2Q2P2
TC15001308.hg.1	2.62	PSR15012568.hg.1	SHF
TC15001308.hg.1	2.48	JUC15006662.hg.1	SHF
TC15001308.hg.1	2.22	PSR15012566.hg.1	SHF
TC18000263.hg.1	2.62	JUC18002084.hg.1	NFATC1
TC18000263.hg.1	2.34	PSR18003093.hg.1	NFATC1
TC18000263.hg.1	-2.31	PSR18003096.hg.1	NFATC1
TC19001761.hg.1	2.62	JUC19013611.hg.1	KLK4
TC19001761.hg.1	-2.13	PSR19023943.hg.1	KLK4
TC19001761.hg.1	-2.47	PSR19023935.hg.1	KLK4
TC19001761.hg.1	-3.29	PSR19023948.hg.1	KLK4
TC19001761.hg.1	-3.51	JUC19013603.hg.1	KLK4
TC21000345.hg.1	2.62	JUC21002097.hg.1	ADAMTS1
TC21000345.hg.1	-2.23	PSR21003915.hg.1	ADAMTS1
TC01000895.hg.1	2.61	JUC01007416.hg.1	AGL
TC01000895.hg.1	2.13	JUC01007427.hg.1	AGL

TC01000895.hg.1	2.05	PSR01014081.hg.1	AGL
TC01002509.hg.1	2.61	JUC01020921.hg.1	OSCP1
TC01002509.hg.1	2.38	JUC01020930.hg.1	OSCP1
TC01002509.hg.1	2.28	JUC01020920.hg.1	OSCP1
TC01002509.hg.1	-2.18	PSR01038950.hg.1	OSCP1
TC01002509.hg.1	-3.09	JUC01020926.hg.1	OSCP1
TC03000339.hg.1	2.61	JUC03003631.hg.1	GNL3, SNOR
TC03000339.hg.1	2.04	PSR03007506.hg.1	GNL3, SNOR
TC03000339.hg.1	-2	JUC03003635.hg.1	GNL3, SNOR
TC04002928.hg.1	2.61	PSR04002235.hg.1	FAM200B
TC04002928.hg.1	2.61	JUC04017018.hg.1	FAM200B
TC04002928.hg.1	2.26	PSR04002250.hg.1	FAM200B
TC05000281.hg.1	2.61	JUC05001945.hg.1	SREK1
TC05000281.hg.1	2.56	JUC05001943.hg.1	SREK1
TC05000281.hg.1	2.38	JUC05001939.hg.1	SREK1
TC05000281.hg.1	2.31	PSR05003617.hg.1	SREK1
TC05000281.hg.1	2.21	PSR05003614.hg.1	SREK1
TC05000281.hg.1	-3.04	JUC05001938.hg.1	SREK1
TC06001487.hg.1	2.61	JUC06008232.hg.1	HLA-F-AS1
TC06001487.hg.1	2.14	PSR06016718.hg.1	HLA-F-AS1
TC06001487.hg.1	2.01	JUC06008239.hg.1	HLA-F-AS1
TC06001487.hg.1	-2.88	JUC06008236.hg.1	HLA-F-AS1
TC06001881.hg.1	2.61	PSR06022939.hg.1	TMEM30A
TC06001881.hg.1	2.03	PSR06022934.hg.1	TMEM30A
TC06001881.hg.1	-2.65	JUC06011124.hg.1	TMEM30A
TC09001058.hg.1	2.61	PSR09014048.hg.1	TPM2
TC09001058.hg.1	2.24	PSR09014060.hg.1	TPM2
TC09001058.hg.1	2.17	JUC09007569.hg.1	TPM2
TC09001058.hg.1	-2.33	PSR09014072.hg.1	TPM2
TC09001536.hg.1	2.61	JUC09010392.hg.1	CDK5RAP2
TC09001536.hg.1	2.33	JUC09010391.hg.1	CDK5RAP2
TC09001536.hg.1	2.16	JUC09010395.hg.1	CDK5RAP2
TC09001536.hg.1	-2.05	JUC09010399.hg.1	CDK5RAP2
TC09001536.hg.1	-2.08	JUC09010408.hg.1	CDK5RAP2
TC09001536.hg.1	-2.13	PSR09019157.hg.1	CDK5RAP2
TC09001536.hg.1	-2.14	PSR09019164.hg.1	CDK5RAP2
TC09001536.hg.1	-2.16	JUC09010388.hg.1	CDK5RAP2
TC09001536.hg.1	-2.16	JUC09010421.hg.1	CDK5RAP2
TC09001536.hg.1	-2.17	PSR09019166.hg.1	CDK5RAP2
TC09001536.hg.1	-2.18	JUC09010416.hg.1	CDK5RAP2
TC09001536.hg.1	-2.2	JUC09010394.hg.1	CDK5RAP2
TC09001536.hg.1	-2.25	PSR09019168.hg.1	CDK5RAP2
TC09001536.hg.1	-2.65	JUC09010402.hg.1	CDK5RAP2
TC09001536.hg.1	-2.72	PSR09019163.hg.1	CDK5RAP2
TC09001536.hg.1	-2.96	PSR09019158.hg.1	CDK5RAP2
TC09001536.hg.1	-3.01	PSR09019216.hg.1	CDK5RAP2
TC09001536.hg.1	-3.12	JUC09010422.hg.1	CDK5RAP2
TC09001536.hg.1	-3.23	PSR09019162.hg.1	CDK5RAP2
TC09001536.hg.1	-4.72	PSR09019167.hg.1	CDK5RAP2
TC0X000619.hg.1	2.61	JUC0X004116.hg.1	UTP14A
TC0X000619.hg.1	-2.11	PSR0X008166.hg.1	UTP14A
TC10000373.hg.1	2.61	JUC10002461.hg.1	CDK1
TC10000373.hg.1	2.54	PSR10004246.hg.1	CDK1
TC10000373.hg.1	2.28	PSR10004254.hg.1	CDK1
TC10000373.hg.1	2.1	PSR10004259.hg.1	CDK1
TC11000878.hg.1	2.61	PSR11011121.hg.1	TMEM135
TC11000878.hg.1	2.18	PSR11011112.hg.1	TMEM135
TC11000878.hg.1	2.11	PSR11011109.hg.1	TMEM135

TC11000878.hg.1	2.11	JUC11005821.hg.1	TMEM135
TC11000878.hg.1	2.03	JUC11005834.hg.1	TMEM135
TC11000878.hg.1	-2.22	JUC11005828.hg.1	TMEM135
TC11002178.hg.1	2.61	JUC11013881.hg.1	NOX4
TC11002178.hg.1	2.55	JUC11013878.hg.1	NOX4
TC11002178.hg.1	2.49	PSR11026102.hg.1	NOX4
TC11002178.hg.1	2.13	JUC11013864.hg.1	NOX4
TC11002178.hg.1	2.02	PSR11026091.hg.1	NOX4
TC11002178.hg.1	-2.01	JUC11013877.hg.1	NOX4
TC11002178.hg.1	-2.41	PSR11026086.hg.1	NOX4
TC11002178.hg.1	-2.59	JUC11013871.hg.1	NOX4
TC11002178.hg.1	-2.75	PSR11026076.hg.1	NOX4
TC11002178.hg.1	-3.22	PSR11026094.hg.1	NOX4
TC11002178.hg.1	-3.48	JUC11013875.hg.1	NOX4
TC11002178.hg.1	-3.6	PSR11026083.hg.1	NOX4
TC11002178.hg.1	-3.6	JUC11013868.hg.1	NOX4
TC11002178.hg.1	-3.65	JUC11013870.hg.1	NOX4
TC11002178.hg.1	-4.03	JUC11013880.hg.1	NOX4
TC11002178.hg.1	-4.31	PSR11026085.hg.1	NOX4
TC11002178.hg.1	-4.44	PSR11026072.hg.1	NOX4
TC11002178.hg.1	-4.58	PSR11026078.hg.1	NOX4
TC11002178.hg.1	-6.56	JUC11013852.hg.1	NOX4
TC11002178.hg.1	-6.93	PSR11026073.hg.1	NOX4
TC11002178.hg.1	-7.84	PSR11026105.hg.1	NOX4
TC11002178.hg.1	-11.06	PSR11026111.hg.1	NOX4
TC11002178.hg.1	-12.13	JUC11013861.hg.1	NOX4
TC11002178.hg.1	-15.31	PSR11026101.hg.1	NOX4
TC11002178.hg.1	-19.83	PSR11026106.hg.1	NOX4
TC11002178.hg.1	-21.73	JUC11013862.hg.1	NOX4
TC11002178.hg.1	-21.83	JUC11013853.hg.1	NOX4
TC11002178.hg.1	-21.99	JUC11013863.hg.1	NOX4
TC11002178.hg.1	-25.61	PSR11026103.hg.1	NOX4
TC11002178.hg.1	-34.73	JUC11013882.hg.1	NOX4
TC11002178.hg.1	-35.71	PSR11026108.hg.1	NOX4
TC11002178.hg.1	-37.28	PSR11026112.hg.1	NOX4
TC11002178.hg.1	-51.07	PSR11026116.hg.1	NOX4
TC11002178.hg.1	-57.55	PSR11026117.hg.1	NOX4
TC14000499.hg.1	2.61	PSR14006377.hg.1	TSHR
TC14000499.hg.1	2.57	JUC14003220.hg.1	TSHR
TC14000499.hg.1	2.28	PSR14006398.hg.1	TSHR
TC14000499.hg.1	2.23	PSR14006401.hg.1	TSHR
TC14000499.hg.1	2.03	PSR14006402.hg.1	TSHR
TC14000499.hg.1	-2.54	JUC14003227.hg.1	TSHR
TC14000499.hg.1	-3.7	JUC14003230.hg.1	TSHR
TC21000157.hg.1	2.61	PSR21001383.hg.1	SIM2
TC21000157.hg.1	2.1	PSR21001388.hg.1	SIM2
TC21000157.hg.1	-2.9	PSR21001373.hg.1	SIM2
TC21000157.hg.1	-2.95	JUC21000689.hg.1	SIM2
TC21000157.hg.1	-5.57	JUC21000682.hg.1	SIM2
TC0X000904.hg.1	2.61	JUC0X006070.hg.1	CXorf23
TC0X000904.hg.1	2.07	JUC0X006066.hg.1	CXorf23
TC0X000904.hg.1	2	PSR0X012227.hg.1	CXorf23
TC0X000904.hg.1	-2.6	JUC0X006051.hg.1	CXorf23
TC01001917.hg.1	2.6	JUC01016148.hg.1	DISC1, TSNA
TC01001917.hg.1	-2.12	JUC01016129.hg.1	DISC1, TSNA
TC01001917.hg.1	-2.15	PSR01029852.hg.1	DISC1, TSNA
TC01001917.hg.1	-2.47	JUC01016136.hg.1	DISC1, TSNA
TC01001917.hg.1	-3.09	JUC01016158.hg.1	DISC1, TSNA

TC01006281.hg.1	2.6	PSR01059951.hg.1	AGT
TC01006281.hg.1	2.41	JUC01039177.hg.1	AGT
TC01006281.hg.1	2.32	PSR01059952.hg.1	AGT
TC02000427.hg.1	2.6	PSR02006420.hg.1	NAGK
TC02000427.hg.1	2.45	JUC02003264.hg.1	NAGK
TC02000427.hg.1	2.4	JUC02003268.hg.1	NAGK
TC02000427.hg.1	2.22	PSR02006385.hg.1	NAGK
TC02001305.hg.1	2.6	JUC02010757.hg.1	PLCD4
TC02001305.hg.1	2.5	JUC02010754.hg.1	PLCD4
TC02001305.hg.1	2.46	JUC02010776.hg.1	PLCD4
TC02001305.hg.1	2.28	JUC02010769.hg.1	PLCD4
TC02001305.hg.1	2.1	JUC02010773.hg.1	PLCD4
TC02001305.hg.1	-2.02	PSR02020070.hg.1	PLCD4
TC02001305.hg.1	-2.02	JUC02010770.hg.1	PLCD4
TC02001305.hg.1	-2.12	PSR02020036.hg.1	PLCD4
TC02001305.hg.1	-4.06	PSR02020035.hg.1	PLCD4
TC02001305.hg.1	-4.07	PSR02020071.hg.1	PLCD4
TC02001867.hg.1	2.6	PSR02029926.hg.1	EFEMP1
TC02001867.hg.1	-2.07	JUC02015721.hg.1	EFEMP1
TC02001867.hg.1	-2.15	PSR02029913.hg.1	EFEMP1
TC02001867.hg.1	-2.22	JUC02015706.hg.1	EFEMP1
TC02001867.hg.1	-2.57	PSR02029907.hg.1	EFEMP1
TC02001867.hg.1	-2.57	JUC02015722.hg.1	EFEMP1
TC02001867.hg.1	-2.72	PSR02029887.hg.1	EFEMP1
TC02001867.hg.1	-2.87	PSR02029912.hg.1	EFEMP1
TC03001042.hg.1	2.6	JUC03009356.hg.1	TP63
TC03001042.hg.1	-2.01	JUC03009369.hg.1	TP63
TC03001042.hg.1	-2.06	PSR03018475.hg.1	TP63
TC03001042.hg.1	-2.13	PSR03018473.hg.1	TP63
TC03001042.hg.1	-2.25	JUC03009367.hg.1	TP63
TC04000912.hg.1	2.6	JUC04006684.hg.1	TLR3
TC04000912.hg.1	-2.02	PSR04012413.hg.1	TLR3
TC04000912.hg.1	-2.93	JUC04006686.hg.1	TLR3
TC04001818.hg.1	2.6	PSR04024813.hg.1	CCDC110
TC04001818.hg.1	2.29	PSR04024828.hg.1	CCDC110
TC04001818.hg.1	2.05	JUC04013166.hg.1	CCDC110
TC05000300.hg.1	2.6	JUC05002103.hg.1	MRPS36
TC05000300.hg.1	-3.26	PSR05003947.hg.1	MRPS36
TC06001561.hg.1	2.6	PSR06018368.hg.1	AGPAT1
TC06001561.hg.1	2.42	JUC06008794.hg.1	AGPAT1
TC06001561.hg.1	2.25	PSR06018374.hg.1	AGPAT1
TC06001561.hg.1	-2.06	PSR06018370.hg.1	AGPAT1
TC06001561.hg.1	-3.57	JUC06008784.hg.1	AGPAT1
TC06002010.hg.1	2.6	PSR06025101.hg.1	CDK19
TC06002010.hg.1	2.59	PSR06025106.hg.1	CDK19
TC06002010.hg.1	2.28	PSR06025105.hg.1	CDK19
TC06002010.hg.1	2.27	JUC06012352.hg.1	CDK19
TC06002010.hg.1	2.2	PSR06025102.hg.1	CDK19
TC07000090.hg.1	2.6	PSR07001458.hg.1	PHF14
TC07000090.hg.1	2.14	PSR07001471.hg.1	PHF14
TC07000090.hg.1	2.03	JUC07000626.hg.1	PHF14
TC08000539.hg.1	2.6	JUC08003642.hg.1	CPNE3
TC08000539.hg.1	2.11	JUC08003650.hg.1	CPNE3
TC08000539.hg.1	2.08	PSR08007326.hg.1	CPNE3
TC08000539.hg.1	-2.81	JUC08003640.hg.1	CPNE3
TC08001485.hg.1	2.6	JUC08009842.hg.1	YWHAZ
TC08001485.hg.1	2.08	PSR08019389.hg.1	YWHAZ
TC08001485.hg.1	-3.89	JUC08009864.hg.1	YWHAZ

TC0X000030.hg.1	2.6	PSR0X000368.hg.1	VCX3B, VCX,
TC0X000030.hg.1	2.07	JUC0X000197.hg.1	VCX3B, VCX,
TC11002132.hg.1	2.6	JUC11013510.hg.1	NDUFC2-KCT
TC11002132.hg.1	-2.19	PSR11025476.hg.1	NDUFC2-KCT
TC11002132.hg.1	-2.43	PSR11025477.hg.1	NDUFC2-KCT
TC11002132.hg.1	-2.59	PSR11025478.hg.1	NDUFC2-KCT
TC12001336.hg.1	2.6	JUC12009682.hg.1	
TC12001336.hg.1	-2.42	PSR12017493.hg.1	
TC12001336.hg.1	-3.12	PSR12017501.hg.1	
TC12001336.hg.1	-3.46	PSR12017498.hg.1	
TC12001336.hg.1	-3.6	JUC12009684.hg.1	
TC13001720.hg.1	2.6	PSR13003971.hg.1	BIVM
TC13001720.hg.1	2.17	JUC13008393.hg.1	BIVM
TC13001720.hg.1	-2.24	JUC13008391.hg.1	BIVM
TC13001720.hg.1	-2.39	PSR13003975.hg.1	BIVM
TC13001720.hg.1	-2.79	JUC13008388.hg.1	BIVM
TC14002205.hg.1	2.6	JUC14011395.hg.1	AP1G2
TC14002205.hg.1	2.13	JUC14011397.hg.1	AP1G2
TC14002205.hg.1	-2.02	PSR14010750.hg.1	AP1G2
TC14002205.hg.1	-2.14	PSR14010760.hg.1	AP1G2
TC14002205.hg.1	-2.48	PSR14010766.hg.1	AP1G2
TC14002205.hg.1	-5.92	JUC14011411.hg.1	AP1G2
TC15001183.hg.1	2.6	JUC15005557.hg.1	FMN1, LOC1(
TC15001183.hg.1	2.39	PSR15010481.hg.1	FMN1, LOC1(
TC15001183.hg.1	2.31	PSR15010486.hg.1	FMN1, LOC1(
TC15001183.hg.1	2.25	JUC15005547.hg.1	FMN1, LOC1(
TC15001183.hg.1	2.23	PSR15010479.hg.1	FMN1, LOC1(
TC15001183.hg.1	2.15	JUC15005540.hg.1	FMN1, LOC1(
TC17002876.hg.1	2.6	PSR17007217.hg.1	CRHR1
TC17002876.hg.1	-2.05	JUC17018691.hg.1	CRHR1
TC17002876.hg.1	-2.64	PSR17007204.hg.1	CRHR1
TC01001024.hg.1	2.59	PSR01016225.hg.1	PTGFRN
TC01001024.hg.1	2.3	PSR01016222.hg.1	PTGFRN
TC01001024.hg.1	2.15	JUC01008502.hg.1	PTGFRN
TC01001661.hg.1	2.59	PSR01025680.hg.1	NAV1
TC01001661.hg.1	2.2	PSR01025681.hg.1	NAV1
TC01001661.hg.1	2.17	PSR01025677.hg.1	NAV1
TC01001661.hg.1	2.12	JUC01013716.hg.1	NAV1
TC01001661.hg.1	2.1	PSR01025676.hg.1	NAV1
TC01001661.hg.1	2.08	PSR01025679.hg.1	NAV1
TC01001661.hg.1	-2.21	JUC01013723.hg.1	NAV1
TC01001661.hg.1	-2.82	JUC01013749.hg.1	NAV1
TC01001829.hg.1	2.59	JUC01015331.hg.1	MIA3
TC01001829.hg.1	2.43	JUC01015307.hg.1	MIA3
TC01001829.hg.1	-2.03	PSR01028391.hg.1	MIA3
TC01002472.hg.1	2.59	JUC01020526.hg.1	ZBTB8OS
TC01002472.hg.1	-2.17	PSR01038175.hg.1	ZBTB8OS
TC01003305.hg.1	2.59	PSR01051193.hg.1	THBS3
TC01003305.hg.1	2.33	JUC01026934.hg.1	THBS3
TC01003305.hg.1	2.25	PSR01051154.hg.1	THBS3
TC01003305.hg.1	2.22	JUC01026928.hg.1	THBS3
TC01003305.hg.1	2.1	JUC01026955.hg.1	THBS3
TC01003305.hg.1	2.08	PSR01051156.hg.1	THBS3
TC01003305.hg.1	2.06	JUC01026958.hg.1	THBS3
TC01003305.hg.1	2.01	PSR01051194.hg.1	THBS3
TC01003305.hg.1	-2.18	JUC01026927.hg.1	THBS3
TC01003305.hg.1	-3.51	JUC01026938.hg.1	THBS3
TC02000891.hg.1	2.59	JUC02007029.hg.1	HNMT

TC02000891.hg.1	2.25	JUC02007031.hg.1	HNMT
TC02000891.hg.1	2.15	PSR02013227.hg.1	HNMT
TC02000891.hg.1	-2.05	JUC02007042.hg.1	HNMT
TC02000891.hg.1	-2.1	PSR02013226.hg.1	HNMT
TC02000891.hg.1	-2.9	JUC02007038.hg.1	HNMT
TC02000891.hg.1	-3.06	PSR02013223.hg.1	HNMT
TC02000891.hg.1	-3.28	JUC02007037.hg.1	HNMT
TC02000891.hg.1	-3.34	PSR02013217.hg.1	HNMT
TC02000891.hg.1	-3.55	PSR02013209.hg.1	HNMT
TC02000891.hg.1	-3.88	JUC02007036.hg.1	HNMT
TC02000891.hg.1	-3.92	JUC02007033.hg.1	HNMT
TC02000891.hg.1	-4.53	JUC02007035.hg.1	HNMT
TC02000891.hg.1	-4.6	JUC02007039.hg.1	HNMT
TC02000891.hg.1	-4.62	PSR02013222.hg.1	HNMT
TC02000891.hg.1	-4.69	PSR02013219.hg.1	HNMT
TC02000891.hg.1	-6.39	PSR02013210.hg.1	HNMT
TC02001857.hg.1	2.59	JUC02015563.hg.1	C2orf63
TC02001857.hg.1	2.35	JUC02015567.hg.1	C2orf63
TC02001857.hg.1	2.27	PSR02029647.hg.1	C2orf63
TC02001857.hg.1	2.09	JUC02015549.hg.1	C2orf63
TC02001857.hg.1	2.02	PSR02029638.hg.1	C2orf63
TC02001857.hg.1	-2.27	PSR02029662.hg.1	C2orf63
TC02002797.hg.1	2.59	JUC02023611.hg.1	DNPEP
TC02002797.hg.1	2.52	JUC02023620.hg.1	DNPEP
TC02002797.hg.1	2.5	JUC02023630.hg.1	DNPEP
TC02002797.hg.1	2.03	PSR02045243.hg.1	DNPEP
TC05000601.hg.1	2.59	JUC05004513.hg.1	GRAMD3
TC05000601.hg.1	-2.01	JUC05004496.hg.1	GRAMD3
TC05000601.hg.1	-2.22	PSR05008288.hg.1	GRAMD3
TC05000601.hg.1	-2.45	PSR05008287.hg.1	GRAMD3
TC05000601.hg.1	-2.63	PSR05008273.hg.1	GRAMD3
TC05000601.hg.1	-2.97	JUC05004483.hg.1	GRAMD3
TC05000601.hg.1	-3.08	JUC05004494.hg.1	GRAMD3
TC05000601.hg.1	-3.61	JUC05004484.hg.1	GRAMD3
TC05000601.hg.1	-3.76	PSR05008260.hg.1	GRAMD3
TC05000601.hg.1	-3.97	PSR05008257.hg.1	GRAMD3
TC05000601.hg.1	-4.04	PSR05008286.hg.1	GRAMD3
TC05000601.hg.1	-4.43	PSR05008270.hg.1	GRAMD3
TC05000601.hg.1	-4.59	PSR05008293.hg.1	GRAMD3
TC05000601.hg.1	-4.62	JUC05004514.hg.1	GRAMD3
TC05000601.hg.1	-5.1	JUC05004489.hg.1	GRAMD3
TC05000821.hg.1	2.59	PSR05011752.hg.1	MIR145, MIR
TC05000821.hg.1	2.58	PSR05011729.hg.1	MIR145, MIR
TC05000821.hg.1	2	PSR05011751.hg.1	MIR145, MIR
TC05000821.hg.1	-2	PSR05011735.hg.1	MIR145, MIR
TC05000821.hg.1	-2	JUC05006108.hg.1	MIR145, MIR
TC05000821.hg.1	-2.08	PSR05011730.hg.1	MIR145, MIR
TC05000821.hg.1	-2.53	PSR05011737.hg.1	MIR145, MIR
TC05000821.hg.1	-2.79	PSR05011726.hg.1	MIR145, MIR
TC05000821.hg.1	-2.84	PSR05011724.hg.1	MIR145, MIR
TC05000821.hg.1	-3.09	JUC05006113.hg.1	MIR145, MIR
TC05000821.hg.1	-3.72	JUC05006115.hg.1	MIR145, MIR
TC05000821.hg.1	-3.94	JUC05006112.hg.1	MIR145, MIR
TC05000821.hg.1	-4.25	PSR05011748.hg.1	MIR145, MIR
TC05000821.hg.1	-4.4	JUC05006114.hg.1	MIR145, MIR
TC05000821.hg.1	-4.76	JUC05006116.hg.1	MIR145, MIR
TC05000821.hg.1	-5.13	PSR05011743.hg.1	MIR145, MIR
TC05000821.hg.1	-7.36	JUC05006111.hg.1	MIR145, MIR

TC05000821.hg.1	-7.4	JUC05006117.hg.1	MIR145, MIR
TC06001828.hg.1	2.59	PSR06022289.hg.1	DST, LOC100
TC06001828.hg.1	-2.01	PSR06022288.hg.1	DST, LOC100
TC06001828.hg.1	-2.39	JUC06010741.hg.1	DST, LOC100
TC06001828.hg.1	-2.52	JUC06010712.hg.1	DST, LOC100
TC06001828.hg.1	-2.96	JUC06010643.hg.1	DST, LOC100
TC06001828.hg.1	-5.43	PSR06022190.hg.1	DST, LOC100
TC06001828.hg.1	-6.09	JUC06010675.hg.1	DST, LOC100
TC09001366.hg.1	2.59	JUC09009161.hg.1	PTCH1
TC09001366.hg.1	2.25	PSR09017019.hg.1	PTCH1
TC09001366.hg.1	2.19	PSR09017059.hg.1	PTCH1
TC09001366.hg.1	2.12	PSR09017036.hg.1	PTCH1
TC09001366.hg.1	2.12	JUC09009142.hg.1	PTCH1
TC09001366.hg.1	2.09	PSR09017046.hg.1	PTCH1
TC09001366.hg.1	-3.27	JUC09009173.hg.1	PTCH1
TC0X001262.hg.1	2.59	JUC0X008683.hg.1	TSC22D3
TC0X001262.hg.1	-2.26	PSR0X017068.hg.1	TSC22D3
TC0X001262.hg.1	-2.29	PSR0X017079.hg.1	TSC22D3
TC0X001262.hg.1	-2.38	PSR0X017047.hg.1	TSC22D3
TC0X001262.hg.1	-2.45	JUC0X008678.hg.1	TSC22D3
TC0X001262.hg.1	-2.52	PSR0X017061.hg.1	TSC22D3
TC0X001262.hg.1	-2.52	PSR0X017083.hg.1	TSC22D3
TC0X001262.hg.1	-2.63	PSR0X017054.hg.1	TSC22D3
TC0X001262.hg.1	-2.65	PSR0X017078.hg.1	TSC22D3
TC0X001262.hg.1	-2.94	PSR0X017071.hg.1	TSC22D3
TC0X001262.hg.1	-2.98	PSR0X017060.hg.1	TSC22D3
TC0X001262.hg.1	-3.28	JUC0X008677.hg.1	TSC22D3
TC0X001262.hg.1	-3.39	PSR0X017056.hg.1	TSC22D3
TC0X001262.hg.1	-3.46	JUC0X008671.hg.1	TSC22D3
TC0X001262.hg.1	-3.68	PSR0X017076.hg.1	TSC22D3
TC0X001262.hg.1	-3.8	PSR0X017055.hg.1	TSC22D3
TC0X001262.hg.1	-3.97	PSR0X017085.hg.1	TSC22D3
TC0X001262.hg.1	-4.14	PSR0X017073.hg.1	TSC22D3
TC0X001262.hg.1	-4.58	PSR0X017064.hg.1	TSC22D3
TC0X001262.hg.1	-5.32	JUC0X008669.hg.1	TSC22D3
TC0X001491.hg.1	2.59	JUC0X009841.hg.1	GABRE, MIR
TC0X001491.hg.1	2.43	JUC0X009845.hg.1	GABRE, MIR
TC0X001491.hg.1	-2.29	PSR0X019422.hg.1	GABRE, MIR
TC0X001491.hg.1	-2.36	PSR0X019387.hg.1	GABRE, MIR
TC0X002309.hg.1	2.59	JUC0X012915.hg.1	NAA10
TC0X002309.hg.1	2.33	JUC0X012917.hg.1	NAA10
TC0X002309.hg.1	2.15	PSR0X020138.hg.1	NAA10
TC11001924.hg.1	2.59	JUC11012178.hg.1	CDCA5
TC11001924.hg.1	2.4	PSR11023065.hg.1	CDCA5
TC12001448.hg.1	2.59	PSR12018960.hg.1	ZNF641
TC12001448.hg.1	-2.18	JUC12010609.hg.1	ZNF641
TC12001797.hg.1	2.59	PSR12023766.hg.1	POC1B, POC
TC12001797.hg.1	2.06	JUC12013173.hg.1	POC1B, POC
TC12001797.hg.1	2.03	PSR12023775.hg.1	POC1B, POC
TC14000571.hg.1	2.59	JUC14003569.hg.1	RIN3
TC14000571.hg.1	-2.27	JUC14003563.hg.1	RIN3
TC14000571.hg.1	-2.38	PSR14006962.hg.1	RIN3
TC14000571.hg.1	-2.43	JUC14003561.hg.1	RIN3
TC14000571.hg.1	-2.48	JUC14003559.hg.1	RIN3
TC14000571.hg.1	-3.91	JUC14003557.hg.1	RIN3
TC14000571.hg.1	-4.11	JUC14003568.hg.1	RIN3
TC17000234.hg.1	2.59	PSR17003304.hg.1	EPN2
TC17000234.hg.1	2.14	PSR17003298.hg.1	EPN2

TC17000234.hg.1	2.13	JUC17001877.hg.1	EPN2
TC17000352.hg.1	2.59	JUC17002487.hg.1	LRRC37BP1
TC17000352.hg.1	2.32	PSR17004398.hg.1	LRRC37BP1
TC17000352.hg.1	-2.42	JUC17002483.hg.1	LRRC37BP1
TC19000459.hg.1	2.59	PSR19006018.hg.1	FXYD1
TC19000459.hg.1	2.53	PSR19006010.hg.1	FXYD1
TC19000459.hg.1	2.53	JUC19003575.hg.1	FXYD1
TC19000459.hg.1	2.28	PSR19006017.hg.1	FXYD1
TC19000459.hg.1	2.12	PSR19006009.hg.1	FXYD1
TC19000459.hg.1	-2.88	JUC19003578.hg.1	FXYD1
TC20000385.hg.1	2.59	PSR20005617.hg.1	DDX27
TC20000385.hg.1	2.12	JUC20003065.hg.1	DDX27
TC20000600.hg.1	2.59	JUC20004508.hg.1	TRMT6
TC20000600.hg.1	-2.32	PSR20008732.hg.1	TRMT6
TC17002869.hg.1	2.59	JUC17018578.hg.1	TBC1D26
TC17002869.hg.1	2.36	JUC17018569.hg.1	TBC1D26
TC17002869.hg.1	2	PSR17002380.hg.1	TBC1D26
TC01001988.hg.1	2.58	JUC01016745.hg.1	C1orf101
TC01001988.hg.1	-2.01	PSR01030783.hg.1	C1orf101
TC01001988.hg.1	-2.89	JUC01016772.hg.1	C1orf101
TC01002166.hg.1	2.58	JUC01017921.hg.1	ERRFI1
TC01002166.hg.1	2.49	PSR01033410.hg.1	ERRFI1
TC01002166.hg.1	2.3	PSR01033397.hg.1	ERRFI1
TC01002166.hg.1	-3.88	PSR01033405.hg.1	ERRFI1
TC03000074.hg.1	2.58	JUC03000853.hg.1	CAND2
TC03000074.hg.1	2.17	JUC03000856.hg.1	CAND2
TC03000074.hg.1	-2.03	PSR03001652.hg.1	CAND2
TC03000074.hg.1	-2.2	JUC03000865.hg.1	CAND2
TC03000077.hg.1	2.58	JUC03000897.hg.1	FBLN2
TC03000077.hg.1	2.48	PSR03001741.hg.1	FBLN2
TC03000077.hg.1	2.29	JUC03000902.hg.1	FBLN2
TC03000077.hg.1	2.23	JUC03000913.hg.1	FBLN2
TC03000077.hg.1	-2.1	JUC03000919.hg.1	FBLN2
TC03000077.hg.1	-2.23	JUC03000916.hg.1	FBLN2
TC03000077.hg.1	-2.31	JUC03000899.hg.1	FBLN2
TC03000077.hg.1	-2.46	JUC03000907.hg.1	FBLN2
TC03000985.hg.1	2.58	PSR03016940.hg.1	KLHL24
TC03000985.hg.1	2.08	JUC03008612.hg.1	KLHL24
TC03000985.hg.1	-2.2	JUC03008623.hg.1	KLHL24
TC05001317.hg.1	2.58	JUC05009561.hg.1	CCL28
TC05001317.hg.1	2.11	PSR05018600.hg.1	CCL28
TC05001822.hg.1	2.58	JUC05012713.hg.1	FAM13B
TC05001822.hg.1	2.29	JUC05012699.hg.1	FAM13B
TC05001822.hg.1	-2.17	PSR05024755.hg.1	FAM13B
TC05001822.hg.1	-2.24	JUC05012701.hg.1	FAM13B
TC05001822.hg.1	-2.79	JUC05012697.hg.1	FAM13B
TC05001822.hg.1	-2.8	JUC05012696.hg.1	FAM13B
TC05001845.hg.1	2.58	JUC05012972.hg.1	TMEM173
TC05001845.hg.1	2.17	PSR05025334.hg.1	TMEM173
TC07000155.hg.1	2.58	JUC07001202.hg.1	MPP6
TC07000155.hg.1	2.43	JUC07001193.hg.1	MPP6
TC07000155.hg.1	2.17	PSR07002455.hg.1	MPP6
TC07000155.hg.1	-4.06	PSR07002458.hg.1	MPP6
TC08001575.hg.1	2.58	JUC08010579.hg.1	DERL1
TC08001575.hg.1	-2.02	PSR08020685.hg.1	DERL1
TC08001575.hg.1	-3.58	JUC08010587.hg.1	DERL1
TC09000378.hg.1	2.58	PSR09004054.hg.1	IDNK, C9orf1
TC09000378.hg.1	2.56	JUC09002210.hg.1	IDNK, C9orf1

TC12003298.hg.1	2.58	JUC12020631.hg.1	HOXC4, HOX
TC12003298.hg.1	2.35	PSR12005876.hg.1	HOXC4, HOX
TC12003298.hg.1	2.19	JUC12020628.hg.1	HOXC4, HOX
TC12003298.hg.1	-5.07	JUC12020632.hg.1	HOXC4, HOX
TC14001527.hg.1	2.58	JUC14008855.hg.1	MOK
TC14001527.hg.1	-2	JUC14008878.hg.1	MOK
TC14001527.hg.1	-2.02	PSR14017110.hg.1	MOK
TC14001527.hg.1	-2.05	JUC14008862.hg.1	MOK
TC14001527.hg.1	-2.08	JUC14008857.hg.1	MOK
TC14001527.hg.1	-2.1	PSR14017132.hg.1	MOK
TC14001527.hg.1	-2.11	JUC14008877.hg.1	MOK
TC14001527.hg.1	-2.13	JUC14008860.hg.1	MOK
TC14001527.hg.1	-2.18	PSR14017131.hg.1	MOK
TC14001527.hg.1	-2.37	JUC14008853.hg.1	MOK
TC14001527.hg.1	-2.67	JUC14008865.hg.1	MOK
TC14001527.hg.1	-2.79	JUC14008846.hg.1	MOK
TC14001527.hg.1	-3.02	JUC14008850.hg.1	MOK
TC15000827.hg.1	2.58	JUC15004006.hg.1	AKAP13
TC15000827.hg.1	2.42	JUC15004001.hg.1	AKAP13
TC15000827.hg.1	2.32	JUC15003991.hg.1	AKAP13
TC15000827.hg.1	2.07	PSR15007625.hg.1	AKAP13
TC15000827.hg.1	-2.02	JUC15003998.hg.1	AKAP13
TC15000827.hg.1	-2.12	PSR15007581.hg.1	AKAP13
TC15000827.hg.1	-2.25	PSR15007578.hg.1	AKAP13
TC15000827.hg.1	-2.3	PSR15007577.hg.1	AKAP13
TC15000827.hg.1	-2.38	PSR15007604.hg.1	AKAP13
TC15000827.hg.1	-4.86	PSR15007584.hg.1	AKAP13
TC17000120.hg.1	2.58	PSR17001754.hg.1	CHD3
TC17000120.hg.1	2.2	JUC17001012.hg.1	CHD3
TC22000791.hg.1	2.58	JUC22005522.hg.1	DDX17
TC22000791.hg.1	2.5	JUC22005523.hg.1	DDX17
TC22000791.hg.1	2.03	PSR22013350.hg.1	DDX17
TC02002507.hg.1	2.58	JUC02020863.hg.1	SPC25
TC02002507.hg.1	2.45	JUC02020868.hg.1	SPC25
TC02002507.hg.1	2	PSR02039683.hg.1	SPC25
TC01000310.hg.1	2.57	JUC01002513.hg.1	NIPAL3
TC01000310.hg.1	-2.02	JUC01002511.hg.1	NIPAL3
TC01000310.hg.1	-2.08	PSR01004689.hg.1	NIPAL3
TC01000476.hg.1	2.57	PSR01007403.hg.1	CDCA8
TC01000476.hg.1	-2.13	JUC01003847.hg.1	CDCA8
TC01001700.hg.1	2.57	JUC01014144.hg.1	MDM4
TC01001700.hg.1	2.48	JUC01014140.hg.1	MDM4
TC01001700.hg.1	2.29	JUC01014145.hg.1	MDM4
TC01001700.hg.1	2.17	PSR01026450.hg.1	MDM4
TC01001700.hg.1	-2.46	JUC01014171.hg.1	MDM4
TC02000203.hg.1	2.57	JUC02001478.hg.1	LCLAT1
TC02000203.hg.1	2.41	JUC02001467.hg.1	LCLAT1
TC02000203.hg.1	2.09	PSR02002949.hg.1	LCLAT1
TC02000540.hg.1	2.57	JUC02004316.hg.1	THNSL2
TC02000540.hg.1	2.19	JUC02004317.hg.1	THNSL2
TC02000540.hg.1	2.06	JUC02004308.hg.1	THNSL2
TC02000540.hg.1	-2.1	JUC02004310.hg.1	THNSL2
TC02000540.hg.1	-2.18	PSR02008417.hg.1	THNSL2
TC02000540.hg.1	-2.39	JUC02004314.hg.1	THNSL2
TC02002683.hg.1	2.57	JUC02022690.hg.1	ICA1L
TC02002683.hg.1	2.48	JUC02022689.hg.1	ICA1L
TC02002683.hg.1	2.23	PSR02043348.hg.1	ICA1L
TC02002683.hg.1	2.2	PSR02043347.hg.1	ICA1L

TC02002683.hg.1	2.19	PSR02043346.hg.1	ICA1L
TC02002683.hg.1	2.09	JUC02022694.hg.1	ICA1L
TC02002683.hg.1	-2.22	JUC02022703.hg.1	ICA1L
TC04000466.hg.1	2.57	PSR04006672.hg.1	ARHGAP24
TC04000466.hg.1	-2.55	JUC04003379.hg.1	ARHGAP24
TC04000466.hg.1	-2.69	JUC04003374.hg.1	ARHGAP24
TC04000853.hg.1	2.57	PSR04011712.hg.1	GALNT7
TC04000853.hg.1	2	PSR04011723.hg.1	GALNT7
TC04000853.hg.1	-2.31	JUC04006292.hg.1	GALNT7
TC05000612.hg.1	2.57	PSR05008500.hg.1	SLC12A2
TC05000612.hg.1	2.22	JUC05004604.hg.1	SLC12A2
TC05000612.hg.1	2.19	PSR05008521.hg.1	SLC12A2
TC05000612.hg.1	2.13	JUC05004621.hg.1	SLC12A2
TC05000612.hg.1	2.12	PSR05008534.hg.1	SLC12A2
TC05000612.hg.1	2.05	PSR05008516.hg.1	SLC12A2
TC05000612.hg.1	-2.19	JUC05004631.hg.1	SLC12A2
TC05000612.hg.1	-3.38	JUC05004632.hg.1	SLC12A2
TC07000571.hg.1	2.57	PSR07008414.hg.1	DYNC1I1
TC07000571.hg.1	-2.16	JUC07004038.hg.1	DYNC1I1
TC07000571.hg.1	-2.2	JUC07004042.hg.1	DYNC1I1
TC07000571.hg.1	-2.23	JUC07004040.hg.1	DYNC1I1
TC07000571.hg.1	-2.26	JUC07004035.hg.1	DYNC1I1
TC07000571.hg.1	-2.33	JUC07004030.hg.1	DYNC1I1
TC07000571.hg.1	-2.65	PSR07008402.hg.1	DYNC1I1
TC07000571.hg.1	-2.77	PSR07008390.hg.1	DYNC1I1
TC07000571.hg.1	-2.89	JUC07004026.hg.1	DYNC1I1
TC07001185.hg.1	2.57	PSR07018538.hg.1	RAPGEF5
TC07001185.hg.1	2.12	PSR07018544.hg.1	RAPGEF5
TC07001185.hg.1	2.09	JUC07009082.hg.1	RAPGEF5
TC07001185.hg.1	-2.05	JUC07009068.hg.1	RAPGEF5
TC07001185.hg.1	-2.11	PSR07018584.hg.1	RAPGEF5
TC07001185.hg.1	-2.15	PSR07018588.hg.1	RAPGEF5
TC07001185.hg.1	-2.18	PSR07018586.hg.1	RAPGEF5
TC07001185.hg.1	-2.35	JUC07009076.hg.1	RAPGEF5
TC07001185.hg.1	-3.51	PSR07018583.hg.1	RAPGEF5
TC07001185.hg.1	-4.15	PSR07018585.hg.1	RAPGEF5
TC07001185.hg.1	-4.95	PSR07018587.hg.1	RAPGEF5
TC07001185.hg.1	-6.15	JUC07009091.hg.1	RAPGEF5
TC07001185.hg.1	-6.26	JUC07009101.hg.1	RAPGEF5
TC08001434.hg.1	2.57	PSR08018640.hg.1	KIAA1429
TC08001434.hg.1	-2.32	JUC08009514.hg.1	KIAA1429
TC09000526.hg.1	2.57	PSR09005759.hg.1	SMC2
TC09000526.hg.1	2.38	JUC09003220.hg.1	SMC2
TC09000526.hg.1	2.1	JUC09003200.hg.1	SMC2
TC09000526.hg.1	2.08	PSR09005772.hg.1	SMC2
TC09000526.hg.1	-2.03	PSR09005797.hg.1	SMC2
TC09000526.hg.1	-2.27	JUC09003190.hg.1	SMC2
TC09000526.hg.1	-2.77	JUC09003194.hg.1	SMC2
TC0X001214.hg.1	2.57	JUC0X008317.hg.1	TRMT2B
TC0X001214.hg.1	2.13	JUC0X008316.hg.1	TRMT2B
TC0X001214.hg.1	-2.43	PSR0X016325.hg.1	TRMT2B
TC10000308.hg.1	2.57	JUC10001983.hg.1	ANTXRL
TC10000308.hg.1	-2.42	PSR10003461.hg.1	ANTXRL
TC10000814.hg.1	2.57	JUC10005470.hg.1	VTI1A
TC10000814.hg.1	2.1	PSR10009799.hg.1	VTI1A
TC10000814.hg.1	-2.31	JUC10005471.hg.1	VTI1A
TC10001557.hg.1	2.57	PSR10019752.hg.1	TLL2
TC10001557.hg.1	2.53	PSR10019747.hg.1	TLL2

TC10001557.hg.1	2.48	JUC10011538.hg.1	TLL2
TC10001557.hg.1	-2.42	JUC10011554.hg.1	TLL2
TC12000202.hg.1	2.57	PSR12002492.hg.1	RERG-AS1
TC12000202.hg.1	2.09	JUC12001159.hg.1	RERG-AS1
TC12001279.hg.1	2.57	PSR12016671.hg.1	RERG
TC12001279.hg.1	-2.51	JUC12009138.hg.1	RERG
TC12001391.hg.1	2.57	JUC12010022.hg.1	KIF21A
TC12001391.hg.1	2.53	JUC12010028.hg.1	KIF21A
TC12001391.hg.1	2.51	JUC12010066.hg.1	KIF21A
TC12001391.hg.1	-2.04	PSR12018014.hg.1	KIF21A
TC12001391.hg.1	-2.66	JUC12010020.hg.1	KIF21A
TC12001391.hg.1	-4.25	JUC12010047.hg.1	KIF21A
TC16000526.hg.1	2.57	JUC16004143.hg.1	CES4A
TC16000526.hg.1	-2.15	JUC16004162.hg.1	CES4A
TC16000526.hg.1	-2.85	PSR16007341.hg.1	CES4A
TC16002082.hg.1	2.57	JUC16013844.hg.1	PRSS53
TC16002082.hg.1	-2.26	PSR16014952.hg.1	PRSS53
TC16002082.hg.1	-3.42	PSR16014949.hg.1	PRSS53
TC17001529.hg.1	2.57	PSR17020399.hg.1	STAT5B
TC17001529.hg.1	2.37	JUC17011419.hg.1	STAT5B
TC17001529.hg.1	2.1	PSR17020429.hg.1	STAT5B
TC17001529.hg.1	2.05	PSR17020418.hg.1	STAT5B
TC17001529.hg.1	2.01	JUC17011428.hg.1	STAT5B
TC17001678.hg.1	2.57	JUC17012541.hg.1	SAMD14
TC17001678.hg.1	2.42	JUC17012529.hg.1	SAMD14
TC17001678.hg.1	2.21	JUC17012543.hg.1	SAMD14
TC17001678.hg.1	2.11	PSR17022533.hg.1	SAMD14
TC17001678.hg.1	-2.09	JUC17012530.hg.1	SAMD14
TC17001678.hg.1	-2.61	PSR17022551.hg.1	SAMD14
TC17001968.hg.1	2.57	PSR17026836.hg.1	NOTUM
TC17001968.hg.1	2.13	JUC17015092.hg.1	NOTUM
TC20001728.hg.1	2.57	PSR20000508.hg.1	PTPRA
TC20001728.hg.1	2.4	JUC20009748.hg.1	PTPRA
TC20001728.hg.1	2.24	JUC20009730.hg.1	PTPRA
TC20001728.hg.1	-3.29	JUC20009736.hg.1	PTPRA
TC02000299.hg.1	2.56	JUC02002364.hg.1	MSH6
TC02000299.hg.1	2.44	PSR02004544.hg.1	MSH6
TC03001778.hg.1	2.56	JUC03016039.hg.1	PIK3R4
TC03001778.hg.1	-2.03	JUC03016030.hg.1	PIK3R4
TC03001778.hg.1	-2.37	PSR03032244.hg.1	PIK3R4
TC03001778.hg.1	-3.09	JUC03016040.hg.1	PIK3R4
TC04000030.hg.1	2.56	PSR04000729.hg.1	WHSC1
TC04000030.hg.1	2.44	JUC04000327.hg.1	WHSC1
TC04000030.hg.1	2.24	JUC04000336.hg.1	WHSC1
TC04000030.hg.1	2.03	PSR04000705.hg.1	WHSC1
TC04000030.hg.1	-2	JUC04000338.hg.1	WHSC1
TC04000030.hg.1	-2.01	JUC04000340.hg.1	WHSC1
TC04000030.hg.1	-2.08	JUC04000310.hg.1	WHSC1
TC04000030.hg.1	-2.13	JUC04000312.hg.1	WHSC1
TC04000030.hg.1	-2.72	JUC04000307.hg.1	WHSC1
TC04000030.hg.1	-3	JUC04000333.hg.1	WHSC1
TC04000030.hg.1	-5.43	JUC04000301.hg.1	WHSC1
TC05000659.hg.1	2.56	JUC05004908.hg.1	UBE2B
TC05000659.hg.1	2.3	PSR05009133.hg.1	UBE2B
TC05001491.hg.1	2.56	JUC05010560.hg.1	COL4A3BP
TC05001491.hg.1	2.46	JUC05010561.hg.1	COL4A3BP
TC05001491.hg.1	2.05	PSR05020470.hg.1	COL4A3BP
TC07001073.hg.1	2.56	JUC07007986.hg.1	PDGFA

TC07001073.hg.1	-2.15	PSR07016436.hg.1	PDGFA
TC07001073.hg.1	-2.32	PSR07016433.hg.1	PDGFA
TC07001073.hg.1	-2.49	PSR07016437.hg.1	PDGFA
TC07001073.hg.1	-2.5	PSR07016435.hg.1	PDGFA
TC07001073.hg.1	-2.58	PSR07016422.hg.1	PDGFA
TC07001073.hg.1	-2.78	PSR07016432.hg.1	PDGFA
TC10000090.hg.1	2.56	JUC10000522.hg.1	SEC61A2
TC10000090.hg.1	2.1	JUC10000504.hg.1	SEC61A2
TC10000090.hg.1	-2.57	JUC10000501.hg.1	SEC61A2
TC10000090.hg.1	-2.65	PSR10000957.hg.1	SEC61A2
TC10001136.hg.1	2.56	JUC10008254.hg.1	MPP7
TC10001136.hg.1	-2	JUC10008257.hg.1	MPP7
TC10001136.hg.1	-2.8	PSR10014271.hg.1	MPP7
TC10001526.hg.1	2.56	JUC10011156.hg.1	CPEB3
TC10001526.hg.1	2.55	PSR10019172.hg.1	CPEB3
TC10001526.hg.1	2.14	JUC10011150.hg.1	CPEB3
TC10001526.hg.1	-2.08	JUC10011145.hg.1	CPEB3
TC10001526.hg.1	-2.18	JUC10011154.hg.1	CPEB3
TC10001546.hg.1	2.56	JUC10011343.hg.1	CYP2C8
TC10001546.hg.1	2.21	PSR10019450.hg.1	CYP2C8
TC10001546.hg.1	-2.06	PSR10019456.hg.1	CYP2C8
TC10001546.hg.1	-2.35	JUC10011342.hg.1	CYP2C8
TC11000399.hg.1	2.56	JUC11002695.hg.1	ATG13
TC11000399.hg.1	2.3	PSR11005010.hg.1	ATG13
TC11000399.hg.1	2.03	JUC11002711.hg.1	ATG13
TC11002228.hg.1	2.56	JUC11014158.hg.1	MMP7
TC11002228.hg.1	2.05	JUC11014162.hg.1	MMP7
TC11002228.hg.1	-2.69	PSR11026620.hg.1	MMP7
TC11002228.hg.1	-3.26	PSR11026617.hg.1	MMP7
TC11002228.hg.1	-15.42	PSR11026615.hg.1	MMP7
TC13000754.hg.1	2.56	JUC13005110.hg.1	RNF219
TC13000754.hg.1	2.04	PSR13008699.hg.1	RNF219
TC13000754.hg.1	-2.04	JUC13005107.hg.1	RNF219
TC15000410.hg.1	2.56	JUC15001823.hg.1	TMOD2
TC15000410.hg.1	-2.07	PSR15003831.hg.1	TMOD2
TC15001772.hg.1	2.56	PSR15017377.hg.1	LOC388152
TC15001772.hg.1	2.09	PSR15017355.hg.1	LOC388152
TC15001772.hg.1	-2.13	PSR15017354.hg.1	LOC388152
TC15001772.hg.1	-2.4	JUC15009477.hg.1	LOC388152
TC15001772.hg.1	-2.66	JUC15009490.hg.1	LOC388152
TC16000488.hg.1	2.56	PSR16006788.hg.1	CX3CL1
TC16000488.hg.1	-2.07	JUC16003848.hg.1	CX3CL1
TC16001252.hg.1	2.56	JUC16009862.hg.1	AP1G1
TC16001252.hg.1	2.08	PSR16017434.hg.1	AP1G1
TC17000906.hg.1	2.56	PSR17011763.hg.1	C1QTNF1
TC17000906.hg.1	2.41	PSR17011761.hg.1	C1QTNF1
TC17000906.hg.1	2.02	JUC17006589.hg.1	C1QTNF1
TC17000906.hg.1	-3.04	JUC17006595.hg.1	C1QTNF1
TC18000507.hg.1	2.56	JUC18003556.hg.1	MYO5B
TC18000507.hg.1	-2.2	PSR18005304.hg.1	MYO5B
TC18000507.hg.1	-2.32	JUC18003545.hg.1	MYO5B
TC18000507.hg.1	-2.37	PSR18005306.hg.1	MYO5B
TC18000507.hg.1	-2.43	JUC18003565.hg.1	MYO5B
TC18000507.hg.1	-2.45	JUC18003555.hg.1	MYO5B
TC18000507.hg.1	-2.79	JUC18003552.hg.1	MYO5B
TC18000507.hg.1	-2.83	JUC18003532.hg.1	MYO5B
TC01000992.hg.1	2.55	PSR01015797.hg.1	MOV10
TC01000992.hg.1	2.45	PSR01015807.hg.1	MOV10

TC01000992.hg.1	2.34	PSR01015759.hg.1	MOV10
TC01000992.hg.1	2.3	JUC01008255.hg.1	MOV10
TC01000992.hg.1	2.11	PSR01015821.hg.1	MOV10
TC01000992.hg.1	2.06	JUC01008236.hg.1	MOV10
TC01000992.hg.1	2.06	JUC01008246.hg.1	MOV10
TC01000992.hg.1	-2.21	PSR01015787.hg.1	MOV10
TC01000992.hg.1	-3.25	JUC01008243.hg.1	MOV10
TC01002425.hg.1	2.55	JUC01020090.hg.1	SNORA61, SI
TC01002425.hg.1	2.24	PSR01037391.hg.1	SNORA61, SI
TC01002425.hg.1	-2.3	PSR01037361.hg.1	SNORA61, SI
TC01002425.hg.1	-2.86	JUC01020093.hg.1	SNORA61, SI
TC01002425.hg.1	-4.64	JUC01020092.hg.1	SNORA61, SI
TC01006353.hg.1	2.55	JUC01044398.hg.1	MKNK1
TC01006353.hg.1	2.09	PSR01040954.hg.1	MKNK1
TC01006353.hg.1	2.08	JUC01044391.hg.1	MKNK1
TC01006353.hg.1	-2.3	PSR01040945.hg.1	MKNK1
TC01006353.hg.1	-2.67	JUC01044400.hg.1	MKNK1
TC02001683.hg.1	2.55	PSR02027164.hg.1	EIF2B4
TC02001683.hg.1	2.32	PSR02027168.hg.1	EIF2B4
TC02001683.hg.1	2.29	JUC02014183.hg.1	EIF2B4
TC02001683.hg.1	2.08	PSR02027198.hg.1	EIF2B4
TC04000029.hg.1	2.55	JUC04000277.hg.1	FGFR3
TC04000029.hg.1	2.47	PSR04000641.hg.1	FGFR3
TC06001727.hg.1	2.55	JUC06009788.hg.1	CCND3
TC06001727.hg.1	2.03	PSR06020649.hg.1	CCND3
TC06001727.hg.1	-2.11	PSR06020638.hg.1	CCND3
TC06001727.hg.1	-2.19	PSR06020656.hg.1	CCND3
TC06001727.hg.1	-2.44	JUC06009785.hg.1	CCND3
TC06001727.hg.1	-2.58	PSR06020659.hg.1	CCND3
TC07000324.hg.1	2.55	JUC07002355.hg.1	LOC285878,
TC07000324.hg.1	-2.45	JUC07002363.hg.1	LOC285878,
TC07000324.hg.1	-2.84	PSR07004813.hg.1	LOC285878,
TC07000324.hg.1	-3.02	JUC07002357.hg.1	LOC285878,
TC08001035.hg.1	2.55	JUC08006860.hg.1	LGI3
TC08001035.hg.1	2.32	JUC08006861.hg.1	LGI3
TC08001035.hg.1	2.14	PSR08013208.hg.1	LGI3
TC08001361.hg.1	2.55	JUC08008950.hg.1	PAG1
TC08001361.hg.1	2.04	PSR08017576.hg.1	PAG1
TC09000607.hg.1	2.55	PSR09006884.hg.1	CNTRL
TC09000607.hg.1	2.39	JUC09003797.hg.1	CNTRL
TC09000607.hg.1	2.32	JUC09003826.hg.1	CNTRL
TC09000607.hg.1	2.07	JUC09003824.hg.1	CNTRL
TC09000607.hg.1	2.03	JUC09003811.hg.1	CNTRL
TC09001541.hg.1	2.55	JUC09010498.hg.1	TRAF1
TC09001541.hg.1	2.3	PSR09019379.hg.1	TRAF1
TC0X001513.hg.1	2.55	JUC0X009938.hg.1	TREX2, HAU
TC0X001513.hg.1	-2.03	PSR0X019661.hg.1	TREX2, HAU
TC0X001513.hg.1	-2.38	PSR0X019660.hg.1	TREX2, HAU
TC0X001513.hg.1	-2.7	PSR0X019663.hg.1	TREX2, HAU
TC0X001513.hg.1	-3.52	JUC0X009941.hg.1	TREX2, HAU
TC0X001513.hg.1	-4.3	PSR0X019662.hg.1	TREX2, HAU
TC10000623.hg.1	2.55	JUC10003897.hg.1	PTEN
TC10000623.hg.1	-2.03	PSR10006855.hg.1	PTEN
TC11000870.hg.1	2.55	JUC11005763.hg.1	EED
TC11000870.hg.1	2.02	JUC11005761.hg.1	EED
TC11000870.hg.1	-2.82	PSR11010996.hg.1	EED
TC12002096.hg.1	2.55	PSR12027754.hg.1	NCOR2
TC12002096.hg.1	2.26	JUC12015589.hg.1	NCOR2

TC12002096.hg.1	-2.2	JUC12015580.hg.1	NCOR2
TC13000105.hg.1	2.55	JUC13000583.hg.1	B3GALTL
TC13000105.hg.1	2.14	PSR13001011.hg.1	B3GALTL
TC13000105.hg.1	-2.01	PSR13001017.hg.1	B3GALTL
TC14000323.hg.1	2.55	JUC14001670.hg.1	LGALS3
TC14000323.hg.1	-2.19	PSR14003452.hg.1	LGALS3
TC14000323.hg.1	-2.46	JUC14001665.hg.1	LGALS3
TC14001122.hg.1	2.55	PSR14012710.hg.1	SAV1
TC14001122.hg.1	2.4	JUC14006403.hg.1	SAV1
TC14001122.hg.1	2.2	JUC14006404.hg.1	SAV1
TC14001122.hg.1	2.01	PSR14012708.hg.1	SAV1
TC14001122.hg.1	-2.98	JUC14006405.hg.1	SAV1
TC14001122.hg.1	-3.14	PSR14012703.hg.1	SAV1
TC15000756.hg.1	2.55	JUC15003516.hg.1	IL16
TC15000756.hg.1	2.13	JUC15003541.hg.1	IL16
TC15000756.hg.1	-2.08	JUC15003531.hg.1	IL16
TC15000756.hg.1	-2.11	JUC15003545.hg.1	IL16
TC15000756.hg.1	-2.19	PSR15006901.hg.1	IL16
TC15000756.hg.1	-2.37	PSR15006895.hg.1	IL16
TC15000756.hg.1	-2.38	JUC15003542.hg.1	IL16
TC15000756.hg.1	-2.41	JUC15003538.hg.1	IL16
TC15000756.hg.1	-4.52	JUC15003532.hg.1	IL16
TC16000192.hg.1	2.55	JUC16001468.hg.1	MIR484, NDE
TC16000192.hg.1	2.15	PSR16002836.hg.1	MIR484, NDE
TC16000192.hg.1	2.02	PSR16002852.hg.1	MIR484, NDE
TC03001862.hg.1	2.55	JUC03016708.hg.1	SLC9A9
TC03001862.hg.1	2	PSR03033601.hg.1	SLC9A9
TC08000126.hg.1	2.55	JUC08000635.hg.1	VPS37A
TC08000126.hg.1	2	PSR08001174.hg.1	VPS37A
TC08000126.hg.1	2	JUC08000623.hg.1	VPS37A
TC01001552.hg.1	2.54	JUC01012670.hg.1	AXDND1
TC01001552.hg.1	2.31	JUC01012650.hg.1	AXDND1
TC01001552.hg.1	2.29	PSR01023941.hg.1	AXDND1
TC01001552.hg.1	-2.31	JUC01012648.hg.1	AXDND1
TC01002199.hg.1	2.54	PSR01034015.hg.1	FBXO2
TC01002199.hg.1	2.28	PSR01034011.hg.1	FBXO2
TC01002199.hg.1	2.13	PSR01034019.hg.1	FBXO2
TC01002199.hg.1	2.09	PSR01034014.hg.1	FBXO2
TC01002199.hg.1	2.07	JUC01018254.hg.1	FBXO2
TC01002199.hg.1	-2.02	PSR01034007.hg.1	FBXO2
TC01002199.hg.1	-2.05	JUC01018255.hg.1	FBXO2
TC01002199.hg.1	-2.07	PSR01033995.hg.1	FBXO2
TC01002199.hg.1	-2.09	PSR01033994.hg.1	FBXO2
TC01002199.hg.1	-2.2	JUC01018253.hg.1	FBXO2
TC01002199.hg.1	-2.28	JUC01018246.hg.1	FBXO2
TC01002199.hg.1	-2.74	JUC01018245.hg.1	FBXO2
TC01003659.hg.1	2.54	JUC01029296.hg.1	KCNT2
TC01003659.hg.1	2.41	JUC01029293.hg.1	KCNT2
TC01003659.hg.1	2.4	JUC01029277.hg.1	KCNT2
TC01003659.hg.1	2.31	PSR01056117.hg.1	KCNT2
TC01003659.hg.1	2.27	JUC01029291.hg.1	KCNT2
TC01003659.hg.1	2.2	JUC01029266.hg.1	KCNT2
TC01003659.hg.1	-2.12	JUC01029273.hg.1	KCNT2
TC01003659.hg.1	-2.2	PSR01056107.hg.1	KCNT2
TC01003659.hg.1	-2.3	JUC01029302.hg.1	KCNT2
TC01003659.hg.1	-2.46	JUC01029298.hg.1	KCNT2
TC01003659.hg.1	-2.54	JUC01029271.hg.1	KCNT2
TC01006283.hg.1	2.54	PSR01016656.hg.1	

TC01006283.hg.1	2.22	JUC01039186.hg.1	
TC01006283.hg.1	2.21	JUC01039189.hg.1	
TC01006283.hg.1	-2.25	JUC01039199.hg.1	
TC02000617.hg.1	2.54	JUC02005166.hg.1	MAP4K4
TC02000617.hg.1	2.34	JUC02005161.hg.1	MAP4K4
TC02000617.hg.1	2.08	PSR02009711.hg.1	MAP4K4
TC06000385.hg.1	2.54	JUC06001653.hg.1	HSPA1B, HSI
TC06000385.hg.1	-2.43	PSR06003980.hg.1	HSPA1B, HSI
TC06000385.hg.1	-2.55	JUC06001654.hg.1	HSPA1B, HSI
TC07001978.hg.1	2.54	JUC07015110.hg.1	OR2A9P, OR
TC07001978.hg.1	-2.04	PSR07030509.hg.1	OR2A9P, OR
TC07001978.hg.1	-2.05	PSR07030524.hg.1	OR2A9P, OR
TC07001978.hg.1	-2.06	PSR07030495.hg.1	OR2A9P, OR
TC07001978.hg.1	-2.07	PSR07030485.hg.1	OR2A9P, OR
TC07001978.hg.1	-2.07	PSR07030496.hg.1	OR2A9P, OR
TC07001978.hg.1	-2.15	PSR07030484.hg.1	OR2A9P, OR
TC07001978.hg.1	-2.16	PSR07030498.hg.1	OR2A9P, OR
TC07001978.hg.1	-2.16	PSR07030521.hg.1	OR2A9P, OR
TC07001978.hg.1	-2.21	PSR07030522.hg.1	OR2A9P, OR
TC07001978.hg.1	-2.22	PSR07030513.hg.1	OR2A9P, OR
TC07001978.hg.1	-2.23	JUC07015094.hg.1	OR2A9P, OR
TC07001978.hg.1	-2.34	JUC07015095.hg.1	OR2A9P, OR
TC07001978.hg.1	-2.36	PSR07030506.hg.1	OR2A9P, OR
TC07001978.hg.1	-2.37	JUC07015113.hg.1	OR2A9P, OR
TC07001978.hg.1	-2.38	PSR07030499.hg.1	OR2A9P, OR
TC07001978.hg.1	-2.5	PSR07030504.hg.1	OR2A9P, OR
TC07001978.hg.1	-2.63	PSR07030505.hg.1	OR2A9P, OR
TC07001978.hg.1	-2.65	JUC07015123.hg.1	OR2A9P, OR
TC07001978.hg.1	-2.75	JUC07015126.hg.1	OR2A9P, OR
TC07001978.hg.1	-2.98	PSR07030514.hg.1	OR2A9P, OR
TC07001978.hg.1	-3.08	PSR07030502.hg.1	OR2A9P, OR
TC07001978.hg.1	-3.11	PSR07030501.hg.1	OR2A9P, OR
TC07001978.hg.1	-3.17	PSR07030503.hg.1	OR2A9P, OR
TC07001978.hg.1	-3.28	JUC07015112.hg.1	OR2A9P, OR
TC07001978.hg.1	-3.79	PSR07030515.hg.1	OR2A9P, OR
TC07001978.hg.1	-4.71	JUC07015106.hg.1	OR2A9P, OR
TC07001978.hg.1	-4.89	JUC07015115.hg.1	OR2A9P, OR
TC08001489.hg.1	2.54	JUC08009876.hg.1	ZNF706
TC08001489.hg.1	-2.03	PSR08019434.hg.1	ZNF706
TC08001489.hg.1	-2.12	PSR08019454.hg.1	ZNF706
TC08001489.hg.1	-2.38	PSR08019452.hg.1	ZNF706
TC08001489.hg.1	-3.57	JUC08009873.hg.1	ZNF706
TC0X000858.hg.1	2.54	JUC0X005643.hg.1	ARHGAP6
TC0X000858.hg.1	2.38	PSR0X011452.hg.1	ARHGAP6
TC0X000858.hg.1	2.01	PSR0X011473.hg.1	ARHGAP6
TC0X000858.hg.1	-2.15	JUC0X005630.hg.1	ARHGAP6
TC10000086.hg.1	2.54	PSR10000883.hg.1	ECHDC3
TC10000086.hg.1	2.22	JUC10000465.hg.1	ECHDC3
TC10000086.hg.1	2.12	PSR10000889.hg.1	ECHDC3
TC10000086.hg.1	2	PSR10000882.hg.1	ECHDC3
TC10000086.hg.1	-2.21	PSR10000887.hg.1	ECHDC3
TC10000086.hg.1	-2.95	JUC10000464.hg.1	ECHDC3
TC10001144.hg.1	2.54	JUC10008319.hg.1	SVIL
TC10001144.hg.1	2.15	JUC10008287.hg.1	SVIL
TC10001144.hg.1	2.03	PSR10014375.hg.1	SVIL
TC10001144.hg.1	-2.16	JUC10008315.hg.1	SVIL
TC10001144.hg.1	-2.25	JUC10008294.hg.1	SVIL
TC10001144.hg.1	-3.69	JUC10008312.hg.1	SVIL

TC10001144.hg.1	-4.17	JUC10008305.hg.1	SVIL
TC19000795.hg.1	2.54	JUC19006552.hg.1	ZNF480
TC19000795.hg.1	2.09	JUC19006550.hg.1	ZNF480
TC19000795.hg.1	-3.64	PSR19011176.hg.1	ZNF480
TC19001242.hg.1	2.54	JUC19010129.hg.1	EMR2
TC19001242.hg.1	2.11	JUC19010150.hg.1	EMR2
TC19001242.hg.1	2.04	JUC19010143.hg.1	EMR2
TC19001242.hg.1	-2.03	PSR19017464.hg.1	EMR2
TC19001242.hg.1	-2.93	JUC19010128.hg.1	EMR2
TC19001242.hg.1	-3.62	PSR19017442.hg.1	EMR2
TC20000358.hg.1	2.54	PSR20005133.hg.1	SNX21
TC20000358.hg.1	2.29	JUC20002791.hg.1	SNX21
TC20000358.hg.1	2.08	PSR20005123.hg.1	SNX21
TC20000358.hg.1	2.08	JUC20002798.hg.1	SNX21
TC20000358.hg.1	2.01	JUC20002792.hg.1	SNX21
TC01000427.hg.1	2.53	PSR01006577.hg.1	HDAC1
TC01000427.hg.1	-2.1	JUC01003437.hg.1	HDAC1
TC01000427.hg.1	-2.53	PSR01006585.hg.1	HDAC1
TC02001795.hg.1	2.53	PSR02028825.hg.1	LRPPRC
TC02001795.hg.1	2.36	JUC02015137.hg.1	LRPPRC
TC02001795.hg.1	2.22	PSR02028881.hg.1	LRPPRC
TC02001795.hg.1	2.1	JUC02015154.hg.1	LRPPRC
TC02001795.hg.1	2.07	JUC02015145.hg.1	LRPPRC
TC04001816.hg.1	2.53	JUC04013152.hg.1	UFSP2
TC04001816.hg.1	2.28	JUC04013148.hg.1	UFSP2
TC04001816.hg.1	2.26	PSR04024778.hg.1	UFSP2
TC04001816.hg.1	-2.28	JUC04013141.hg.1	UFSP2
TC05000291.hg.1	2.53	PSR05003787.hg.1	PIK3R1
TC05000291.hg.1	2.45	PSR05003788.hg.1	PIK3R1
TC05000291.hg.1	2.31	JUC05002032.hg.1	PIK3R1
TC05000291.hg.1	2.27	PSR05003785.hg.1	PIK3R1
TC05000291.hg.1	2.17	JUC05002048.hg.1	PIK3R1
TC05000291.hg.1	-2.24	JUC05002053.hg.1	PIK3R1
TC05000291.hg.1	-2.53	PSR05003806.hg.1	PIK3R1
TC05000291.hg.1	-2.61	PSR05003807.hg.1	PIK3R1
TC05000291.hg.1	-3.72	JUC05002036.hg.1	PIK3R1
TC05000291.hg.1	-3.81	JUC05002046.hg.1	PIK3R1
TC05000291.hg.1	-4.93	JUC05002049.hg.1	PIK3R1
TC05000291.hg.1	-10.77	PSR05003801.hg.1	PIK3R1
TC09000128.hg.1	2.53	JUC09000792.hg.1	ACO1
TC09000128.hg.1	2.24	JUC09000784.hg.1	ACO1
TC09000128.hg.1	2.22	PSR09001459.hg.1	ACO1
TC09000128.hg.1	2.08	JUC09000779.hg.1	ACO1
TC09000128.hg.1	2.07	PSR09001460.hg.1	ACO1
TC10001309.hg.1	2.53	PSR10016371.hg.1	ZWINT
TC10001309.hg.1	2.51	JUC10009571.hg.1	ZWINT
TC13000021.hg.1	2.53	PSR13000098.hg.1	ZMYM2
TC13000021.hg.1	-2.01	PSR13000138.hg.1	ZMYM2
TC13000021.hg.1	-2.05	JUC13000071.hg.1	ZMYM2
TC13000021.hg.1	-2.08	JUC13000052.hg.1	ZMYM2
TC15000647.hg.1	2.53	JUC15002776.hg.1	LRRC49
TC15000647.hg.1	2.2	PSR15005505.hg.1	LRRC49
TC15000647.hg.1	2.09	JUC15002788.hg.1	LRRC49
TC15000647.hg.1	-2.21	JUC15002774.hg.1	LRRC49
TC15000647.hg.1	-2.37	JUC15002772.hg.1	LRRC49
TC15000647.hg.1	-2.37	JUC15002785.hg.1	LRRC49
TC16001169.hg.1	2.53	JUC16009135.hg.1	TK2
TC16001169.hg.1	-2.26	JUC16009148.hg.1	TK2

TC16001169.hg.1	-2.47	PSR16016223.hg.1	TK2
TC19000674.hg.1	2.53	JUC19005602.hg.1	CCDC9
TC19000674.hg.1	-2.16	PSR19009429.hg.1	CCDC9
TC20000372.hg.1	2.53	JUC20002951.hg.1	NCOA3
TC20000372.hg.1	-2.04	JUC20002939.hg.1	NCOA3
TC20000372.hg.1	-2.77	JUC20002934.hg.1	NCOA3
TC20000372.hg.1	-2.91	JUC20002935.hg.1	NCOA3
TC20000372.hg.1	-3.32	PSR20005402.hg.1	NCOA3
TC22000652.hg.1	2.53	JUC22004673.hg.1	RNF215, KIA
TC22000652.hg.1	2.16	PSR22011466.hg.1	RNF215, KIA
TC22000652.hg.1	2.06	JUC22004674.hg.1	RNF215, KIA
TC22000652.hg.1	2.04	JUC22004675.hg.1	RNF215, KIA
TC18000070.hg.1	2.53	JUC18000483.hg.1	CEP192
TC18000070.hg.1	2	PSR18000805.hg.1	CEP192
TC18000070.hg.1	-2.95	JUC18000447.hg.1	CEP192
TC01006366.hg.1	2.52	PSR01046950.hg.1	DENND2C
TC01006366.hg.1	-2.05	JUC01044598.hg.1	DENND2C
TC01006366.hg.1	-2.34	PSR01046977.hg.1	DENND2C
TC02000721.hg.1	2.52	PSR02011174.hg.1	PSD4
TC02000721.hg.1	2.37	PSR02011186.hg.1	PSD4
TC02000721.hg.1	2.27	JUC02005924.hg.1	PSD4
TC03000024.hg.1	2.52	JUC03000279.hg.1	GRM7
TC03000024.hg.1	2.12	JUC03000296.hg.1	GRM7
TC03000024.hg.1	-2.26	PSR03000486.hg.1	GRM7
TC04000641.hg.1	2.52	JUC04004937.hg.1	INTU
TC04000641.hg.1	2.17	JUC04004945.hg.1	INTU
TC04000641.hg.1	2.12	JUC04004925.hg.1	INTU
TC04000641.hg.1	-2	PSR04009249.hg.1	INTU
TC04000641.hg.1	-2.33	PSR04009257.hg.1	INTU
TC05000575.hg.1	2.52	JUC05004381.hg.1	SNCAIP
TC05000575.hg.1	2.03	PSR05008049.hg.1	SNCAIP
TC05000575.hg.1	2	PSR05008043.hg.1	SNCAIP
TC05000575.hg.1	-2.3	JUC05004371.hg.1	SNCAIP
TC05001342.hg.1	2.52	PSR05018856.hg.1	MOCS2
TC05001342.hg.1	-2.12	JUC05009687.hg.1	MOCS2
TC05001820.hg.1	2.52	JUC05012694.hg.1	
TC05001820.hg.1	-2.04	PSR05024738.hg.1	
TC06000628.hg.1	2.52	JUC06003371.hg.1	SLC25A27
TC06000628.hg.1	2.09	PSR06007597.hg.1	SLC25A27
TC06000628.hg.1	2	PSR06007595.hg.1	SLC25A27
TC06000628.hg.1	2	JUC06003366.hg.1	SLC25A27
TC07001997.hg.1	2.52	JUC07015220.hg.1	ZNF425
TC07001997.hg.1	2.48	PSR07030700.hg.1	ZNF425
TC07001997.hg.1	2.45	PSR07030693.hg.1	ZNF425
TC07001997.hg.1	2.26	PSR07030697.hg.1	ZNF425
TC07001997.hg.1	2.08	PSR07030698.hg.1	ZNF425
TC10000982.hg.1	2.52	JUC10007139.hg.1	LOC1002160
TC10000982.hg.1	-2.04	PSR10012331.hg.1	LOC1002160
TC10000982.hg.1	-2.09	PSR10012325.hg.1	LOC1002160
TC10000982.hg.1	-2.36	JUC10007140.hg.1	LOC1002160
TC10000982.hg.1	-2.54	PSR10012334.hg.1	LOC1002160
TC10000982.hg.1	-3.38	JUC10007146.hg.1	LOC1002160
TC10000982.hg.1	-3.42	JUC10007148.hg.1	LOC1002160
TC10000982.hg.1	-3.66	PSR10012333.hg.1	LOC1002160
TC11001302.hg.1	2.52	JUC11008919.hg.1	NUP98
TC11001302.hg.1	2.29	PSR11016593.hg.1	NUP98
TC11001302.hg.1	-2.03	JUC11008905.hg.1	NUP98
TC11001302.hg.1	-2.39	JUC11008906.hg.1	NUP98

TC12001426.hg.1	2.52	JUC12010285.hg.1	SLC38A1
TC12001426.hg.1	-2.11	PSR12018425.hg.1	SLC38A1
TC14002325.hg.1	2.52	JUC14012126.hg.1	C14orf149
TC14002325.hg.1	2.03	PSR14013488.hg.1	C14orf149
TC14002325.hg.1	-3.53	JUC14012124.hg.1	C14orf149
TC15000035.hg.1	2.52	JUC15000177.hg.1	GOLGA8E, G
TC15000035.hg.1	2.37	PSR15000245.hg.1	GOLGA8E, G
TC15000035.hg.1	-2.03	PSR15000249.hg.1	GOLGA8E, G
TC16000372.hg.1	2.52	PSR16005461.hg.1	FUS
TC16000372.hg.1	2.28	JUC16002978.hg.1	FUS
TC21000388.hg.1	2.52	JUC21002254.hg.1	TIAM1
TC21000388.hg.1	2.47	JUC21002273.hg.1	TIAM1
TC21000388.hg.1	2.09	PSR21004286.hg.1	TIAM1
TC21000388.hg.1	2.01	JUC21002257.hg.1	TIAM1
TC21000388.hg.1	-2	PSR21004270.hg.1	TIAM1
TC21000388.hg.1	-2	JUC21002260.hg.1	TIAM1
TC21000388.hg.1	-2.25	JUC21002278.hg.1	TIAM1
TC21000388.hg.1	-2.38	JUC21002243.hg.1	TIAM1
TC21000388.hg.1	-2.69	PSR21004253.hg.1	TIAM1
TC21000388.hg.1	-2.74	PSR21004283.hg.1	TIAM1
TC21000388.hg.1	-2.76	JUC21002277.hg.1	TIAM1
TC01001807.hg.1	2.51	PSR01028204.hg.1	LYPLAL1
TC01001807.hg.1	2.38	JUC01015213.hg.1	LYPLAL1
TC01001807.hg.1	-2.08	JUC01015201.hg.1	LYPLAL1
TC01001807.hg.1	-4.02	JUC01015207.hg.1	LYPLAL1
TC03000815.hg.1	2.51	JUC03007378.hg.1	EIF2A
TC03000815.hg.1	-2.16	PSR03014649.hg.1	EIF2A
TC03000815.hg.1	-2.69	PSR03014654.hg.1	EIF2A
TC03000815.hg.1	-3.79	PSR03014651.hg.1	EIF2A
TC03000815.hg.1	-5.46	JUC03007385.hg.1	EIF2A
TC03000932.hg.1	2.51	PSR03016204.hg.1	NLGN1
TC03000932.hg.1	-3.29	JUC03008258.hg.1	NLGN1
TC04001554.hg.1	2.51	PSR04021865.hg.1	PABPC4L
TC04001554.hg.1	2.19	JUC04011525.hg.1	PABPC4L
TC05000245.hg.1	2.51	JUC05001689.hg.1	SETD9
TC05000245.hg.1	-2.03	PSR05003039.hg.1	SETD9
TC05000245.hg.1	-2.11	JUC05001682.hg.1	SETD9
TC05000245.hg.1	-2.15	JUC05001691.hg.1	SETD9
TC05000245.hg.1	-2.92	PSR05003023.hg.1	SETD9
TC05000245.hg.1	-3.19	JUC05001683.hg.1	SETD9
TC05000726.hg.1	2.51	PSR05010336.hg.1	EIF4EBP3, AI
TC05000726.hg.1	-2.22	JUC05005503.hg.1	EIF4EBP3, AI
TC05000726.hg.1	-2.52	JUC05005473.hg.1	EIF4EBP3, AI
TC05001482.hg.1	2.51	JUC05010491.hg.1	GFM2
TC05001482.hg.1	2.07	PSR05020352.hg.1	GFM2
TC05001482.hg.1	-2.41	PSR05020335.hg.1	GFM2
TC05003422.hg.1	2.51	JUC05019554.hg.1	AMACR
TC05003422.hg.1	-2.02	PSR05017509.hg.1	AMACR
TC05003422.hg.1	-2.15	PSR05017508.hg.1	AMACR
TC05003422.hg.1	-4.27	JUC05019551.hg.1	AMACR
TC06000768.hg.1	2.51	JUC06004304.hg.1	DOPEY1
TC06000768.hg.1	2.44	PSR06009196.hg.1	DOPEY1
TC06000768.hg.1	2	PSR06009220.hg.1	DOPEY1
TC06000768.hg.1	-2.08	JUC06004275.hg.1	DOPEY1
TC06000768.hg.1	-2.21	JUC06004278.hg.1	DOPEY1
TC06000768.hg.1	-2.32	JUC06004301.hg.1	DOPEY1
TC06000768.hg.1	-2.39	JUC06004266.hg.1	DOPEY1
TC06000768.hg.1	-2.47	JUC06004312.hg.1	DOPEY1

TC06000768.hg.1	-2.5	PSR06009189.hg.1	DOPEY1
TC07001808.hg.1	2.51	JUC07013767.hg.1	FAM3C
TC07001808.hg.1	2.07	JUC07013760.hg.1	FAM3C
TC07001808.hg.1	2.02	PSR07027983.hg.1	FAM3C
TC09000891.hg.1	2.51	JUC09006417.hg.1	PLGRKT
TC09000891.hg.1	-2.12	JUC09006416.hg.1	PLGRKT
TC09000891.hg.1	-2.26	PSR09011841.hg.1	PLGRKT
TC09000891.hg.1	-2.46	JUC09006414.hg.1	PLGRKT
TC09000891.hg.1	-4.6	JUC09006412.hg.1	PLGRKT
TC09001379.hg.1	2.51	PSR09017257.hg.1	ZNF782
TC09001379.hg.1	2.08	JUC09009281.hg.1	ZNF782
TC09001379.hg.1	-2.9	JUC09009272.hg.1	ZNF782
TC11001716.hg.1	2.51	JUC11010594.hg.1	PHF21A
TC11001716.hg.1	2.09	PSR11019976.hg.1	PHF21A
TC11001716.hg.1	-2.06	JUC11010598.hg.1	PHF21A
TC11001716.hg.1	-3.3	JUC11010588.hg.1	PHF21A
TC11002004.hg.1	2.51	PSR11024035.hg.1	SUV420H1
TC11002004.hg.1	2.19	JUC11012694.hg.1	SUV420H1
TC11002004.hg.1	-2.07	JUC11012698.hg.1	SUV420H1
TC11002004.hg.1	-2.08	JUC11012682.hg.1	SUV420H1
TC11002004.hg.1	-2.21	JUC11012686.hg.1	SUV420H1
TC11002322.hg.1	2.51	JUC11014787.hg.1	BUD13
TC11002322.hg.1	-2.17	PSR11027736.hg.1	BUD13
TC12001625.hg.1	2.51	JUC12012090.hg.1	DDIT3
TC12001625.hg.1	-2.02	JUC12012092.hg.1	DDIT3
TC12001625.hg.1	-2.05	PSR12021963.hg.1	DDIT3
TC12001636.hg.1	2.51	PSR12022141.hg.1	AVIL
TC12001636.hg.1	2.34	PSR12022135.hg.1	AVIL
TC12001636.hg.1	2.12	JUC12012204.hg.1	AVIL
TC12001636.hg.1	-2.14	PSR12022163.hg.1	AVIL
TC12001636.hg.1	-2.23	JUC12012206.hg.1	AVIL
TC12001636.hg.1	-2.32	PSR12022155.hg.1	AVIL
TC12001636.hg.1	-2.46	PSR12022154.hg.1	AVIL
TC12001636.hg.1	-2.51	PSR12022165.hg.1	AVIL
TC12001636.hg.1	-2.68	JUC12012190.hg.1	AVIL
TC12001636.hg.1	-2.79	PSR12022160.hg.1	AVIL
TC12001636.hg.1	-3.17	PSR12022161.hg.1	AVIL
TC12001636.hg.1	-3.66	PSR12022162.hg.1	AVIL
TC12001636.hg.1	-3.92	PSR12022144.hg.1	AVIL
TC12001636.hg.1	-8.52	JUC12012203.hg.1	AVIL
TC14000514.hg.1	2.51	JUC14003254.hg.1	FLRT2, LOC1
TC14000514.hg.1	2.34	PSR14006445.hg.1	FLRT2, LOC1
TC14000514.hg.1	-2.07	PSR14006446.hg.1	FLRT2, LOC1
TC15000164.hg.1	2.51	JUC15000421.hg.1	DKFZP434L1
TC15000164.hg.1	2.41	JUC15000429.hg.1	DKFZP434L1
TC15000164.hg.1	2.25	PSR15001067.hg.1	DKFZP434L1
TC15000164.hg.1	2.2	PSR15001063.hg.1	DKFZP434L1
TC15000164.hg.1	2.14	PSR15001069.hg.1	DKFZP434L1
TC18000477.hg.1	2.51	PSR18004909.hg.1	PSTPIP2
TC18000477.hg.1	2.18	PSR18004926.hg.1	PSTPIP2
TC18000477.hg.1	2.05	JUC18003322.hg.1	PSTPIP2
TC20000019.hg.1	2.51	PSR20000225.hg.1	SIRPA
TC20000019.hg.1	2.2	PSR20000219.hg.1	SIRPA
TC20000019.hg.1	-2.53	JUC20000114.hg.1	SIRPA
TC20000019.hg.1	-3.02	JUC20000109.hg.1	SIRPA
TC20000499.hg.1	2.51	PSR20007010.hg.1	COL9A3
TC20000499.hg.1	2.44	JUC20003722.hg.1	COL9A3
TC20000499.hg.1	2.41	PSR20007037.hg.1	COL9A3

TC20000499.hg.1	2.41	JUC20003724.hg.1	COL9A3
TC20000499.hg.1	2.02	JUC20003734.hg.1	COL9A3
TC20000499.hg.1	-2.1	PSR20007061.hg.1	COL9A3
TC20000499.hg.1	-3.2	JUC20003708.hg.1	COL9A3
TC20000499.hg.1	-27.1	JUC20003723.hg.1	COL9A3
TC21001063.hg.1	2.51	PSR21002180.hg.1	NDUFV3
TC21001063.hg.1	-2.02	JUC21005181.hg.1	NDUFV3
TC02002644.hg.1	2.5	PSR02042411.hg.1	PGAP1
TC02002644.hg.1	2.33	PSR02042402.hg.1	PGAP1
TC02002644.hg.1	2.11	PSR02042370.hg.1	PGAP1
TC02002644.hg.1	2.04	PSR02042386.hg.1	PGAP1
TC02002644.hg.1	-2.63	JUC02022166.hg.1	PGAP1
TC03001343.hg.1	2.5	PSR03022943.hg.1	ZDHHC3
TC03001343.hg.1	-2.97	JUC03011438.hg.1	ZDHHC3
TC05001871.hg.1	2.5	JUC05013134.hg.1	HDAC3
TC05001871.hg.1	2.28	PSR05025762.hg.1	HDAC3
TC05001871.hg.1	2.23	JUC05013132.hg.1	HDAC3
TC07000993.hg.1	2.5	PSR07015079.hg.1	ZNF862
TC07000993.hg.1	2.09	JUC07007367.hg.1	ZNF862
TC07000993.hg.1	-2.01	JUC07007364.hg.1	ZNF862
TC08001652.hg.1	2.5	JUC08010944.hg.1	KCNQ3
TC08001652.hg.1	2.29	PSR08021452.hg.1	KCNQ3
TC08001652.hg.1	2.24	JUC08010946.hg.1	KCNQ3
TC08001652.hg.1	2.03	PSR08021461.hg.1	KCNQ3
TC08001652.hg.1	2	PSR08021463.hg.1	KCNQ3
TC08001652.hg.1	-2.11	JUC08010948.hg.1	KCNQ3
TC08001652.hg.1	-2.19	PSR08021438.hg.1	KCNQ3
TC08001652.hg.1	-2.3	PSR08021439.hg.1	KCNQ3
TC08001652.hg.1	-2.46	PSR08021444.hg.1	KCNQ3
TC08001652.hg.1	-2.65	JUC08010954.hg.1	KCNQ3
TC08001652.hg.1	-3.83	PSR08021447.hg.1	KCNQ3
TC08001652.hg.1	-5.66	JUC08010945.hg.1	KCNQ3
TC08001652.hg.1	-5.99	PSR08021440.hg.1	KCNQ3
TC11002140.hg.1	2.5	JUC11013587.hg.1	ODZ4
TC11002140.hg.1	2.22	JUC11013570.hg.1	ODZ4
TC11002140.hg.1	-2.05	PSR11025617.hg.1	ODZ4
TC11002140.hg.1	-2.31	JUC11013573.hg.1	ODZ4
TC11002140.hg.1	-2.43	PSR11025607.hg.1	ODZ4
TC11002140.hg.1	-2.7	JUC11013576.hg.1	ODZ4
TC11002140.hg.1	-2.73	PSR11025618.hg.1	ODZ4
TC11002140.hg.1	-2.91	PSR11025586.hg.1	ODZ4
TC11002140.hg.1	-3.05	PSR11025612.hg.1	ODZ4
TC11002140.hg.1	-3.21	PSR11025620.hg.1	ODZ4
TC11002140.hg.1	-3.41	PSR11025611.hg.1	ODZ4
TC11002140.hg.1	-4.47	PSR11025627.hg.1	ODZ4
TC11002140.hg.1	-5.68	JUC11013592.hg.1	ODZ4
TC11002140.hg.1	-5.7	JUC11013572.hg.1	ODZ4
TC11002140.hg.1	-6.56	PSR11025625.hg.1	ODZ4
TC11002140.hg.1	-8.6	JUC11013601.hg.1	ODZ4
TC11002140.hg.1	-8.63	JUC11013571.hg.1	ODZ4
TC12001598.hg.1	2.5	JUC12011779.hg.1	RNF41
TC12001598.hg.1	2.03	PSR12021248.hg.1	RNF41
TC12001598.hg.1	-2.97	JUC12011784.hg.1	RNF41
TC18000018.hg.1	2.5	JUC18000146.hg.1	TGIF1
TC18000018.hg.1	2.16	PSR18000201.hg.1	TGIF1
TC18000018.hg.1	2.01	PSR18000193.hg.1	TGIF1
TC18000018.hg.1	-2.23	PSR18000217.hg.1	TGIF1
TC19000589.hg.1	2.5	JUC19004907.hg.1	ARHGEF1

TC19000589.hg.1	-2.06	JUC19004886.hg.1	ARHGEF1
TC19000589.hg.1	-2.39	PSR19008192.hg.1	ARHGEF1
TC01000723.hg.1	2.49	JUC01005941.hg.1	ROR1
TC01000723.hg.1	2.15	JUC01005937.hg.1	ROR1
TC01000723.hg.1	-2.23	PSR01011484.hg.1	ROR1
TC01000723.hg.1	-2.52	JUC01005940.hg.1	ROR1
TC01000723.hg.1	-2.54	PSR01011489.hg.1	ROR1
TC01000723.hg.1	-2.71	PSR01011494.hg.1	ROR1
TC01000723.hg.1	-2.75	JUC01005938.hg.1	ROR1
TC01000723.hg.1	-3.19	JUC01005936.hg.1	ROR1
TC01000723.hg.1	-4.44	PSR01011482.hg.1	ROR1
TC01002485.hg.1	2.49	JUC01020696.hg.1	CSMD2
TC01002485.hg.1	2.16	JUC01020690.hg.1	CSMD2
TC01002485.hg.1	-2.01	PSR01038485.hg.1	CSMD2
TC01003892.hg.1	2.49	JUC01031098.hg.1	TMEM63A
TC01003892.hg.1	-2.4	PSR01059309.hg.1	TMEM63A
TC02000295.hg.1	2.49	JUC02002350.hg.1	MSH2
TC02000295.hg.1	2.33	PSR02004483.hg.1	MSH2
TC02000295.hg.1	-2.19	JUC02002322.hg.1	MSH2
TC02004934.hg.1	2.49	JUC02031447.hg.1	ZNF512
TC02004934.hg.1	2.13	PSR02002420.hg.1	ZNF512
TC03000348.hg.1	2.49	PSR03007646.hg.1	PRKCD
TC03000348.hg.1	2.02	JUC03003712.hg.1	PRKCD
TC03000348.hg.1	2.01	PSR03007651.hg.1	PRKCD
TC03000348.hg.1	-2.21	JUC03003705.hg.1	PRKCD
TC03000846.hg.1	2.49	PSR03014959.hg.1	MME
TC03000846.hg.1	2.42	JUC03007599.hg.1	MME
TC03000846.hg.1	2.01	JUC03007602.hg.1	MME
TC03000846.hg.1	-2.45	PSR03014963.hg.1	MME
TC03000846.hg.1	-2.67	PSR03014960.hg.1	MME
TC03000846.hg.1	-2.85	PSR03014953.hg.1	MME
TC03000846.hg.1	-3.58	PSR03014955.hg.1	MME
TC03000846.hg.1	-3.64	PSR03014969.hg.1	MME
TC03000846.hg.1	-3.79	PSR03014948.hg.1	MME
TC03000846.hg.1	-4.44	PSR03014949.hg.1	MME
TC03000846.hg.1	-4.5	PSR03014954.hg.1	MME
TC03000846.hg.1	-5.83	JUC03007582.hg.1	MME
TC03000846.hg.1	-7.08	JUC03007581.hg.1	MME
TC03000846.hg.1	-8.5	PSR03014967.hg.1	MME
TC03000846.hg.1	-8.82	JUC03007573.hg.1	MME
TC03000846.hg.1	-12.1	PSR03014947.hg.1	MME
TC03000846.hg.1	-16.57	JUC03007579.hg.1	MME
TC03002033.hg.1	2.49	PSR03036025.hg.1	
TC03002033.hg.1	-2.03	PSR03036016.hg.1	
TC03002033.hg.1	-2.32	JUC03017819.hg.1	
TC03002033.hg.1	-2.93	JUC03017815.hg.1	
TC04001641.hg.1	2.49	JUC04012129.hg.1	PET112
TC04001641.hg.1	2.4	JUC04012116.hg.1	PET112
TC04001641.hg.1	-3.28	PSR04022925.hg.1	PET112
TC05000580.hg.1	2.49	JUC05004440.hg.1	PRDM6
TC05000580.hg.1	2.24	PSR05008180.hg.1	PRDM6
TC05001657.hg.1	2.49	PSR05022451.hg.1	EFNA5
TC05001657.hg.1	-3.3	PSR05022454.hg.1	EFNA5
TC05001657.hg.1	-3.59	PSR05022458.hg.1	EFNA5
TC05001657.hg.1	-9.24	JUC05011603.hg.1	EFNA5
TC06001678.hg.1	2.49	JUC06009497.hg.1	CPNE5
TC06001678.hg.1	2.05	PSR06019977.hg.1	CPNE5
TC06001678.hg.1	-2.28	JUC06009489.hg.1	CPNE5

TC06002210.hg.1	2.49	PSR06027683.hg.1	PPIL4
TC06002210.hg.1	-3.97	JUC06013801.hg.1	PPIL4
TC06004134.hg.1	2.49	JUC06021832.hg.1	C6orf130
TC06004134.hg.1	-2.14	PSR06020393.hg.1	C6orf130
TC06004134.hg.1	-2.27	PSR06020379.hg.1	C6orf130
TC06004134.hg.1	-2.45	PSR06020378.hg.1	C6orf130
TC06004134.hg.1	-2.77	JUC06021835.hg.1	C6orf130
TC06004134.hg.1	-2.98	PSR06020391.hg.1	C6orf130
TC07001012.hg.1	2.49	JUC07007551.hg.1	SLC4A2
TC07001012.hg.1	2.28	JUC07007555.hg.1	SLC4A2
TC07001012.hg.1	-2.33	PSR07015567.hg.1	SLC4A2
TC10000653.hg.1	2.49	JUC10004095.hg.1	HECTD2
TC10000653.hg.1	-2.02	JUC10004083.hg.1	HECTD2
TC10000653.hg.1	-2.09	PSR10007237.hg.1	HECTD2
TC10000653.hg.1	-2.26	PSR10007213.hg.1	HECTD2
TC10000653.hg.1	-2.29	PSR10007231.hg.1	HECTD2
TC10000653.hg.1	-2.35	PSR10007235.hg.1	HECTD2
TC10000653.hg.1	-2.38	PSR10007247.hg.1	HECTD2
TC11001914.hg.1	2.49	JUC11012053.hg.1	CDC42BPG
TC11001914.hg.1	2.22	PSR11022832.hg.1	CDC42BPG
TC11001914.hg.1	-2.02	JUC11012028.hg.1	CDC42BPG
TC20000797.hg.1	2.49	JUC20005616.hg.1	GDF5
TC20000797.hg.1	-2.23	PSR20010758.hg.1	GDF5
TC02001071.hg.1	2.48	JUC02008455.hg.1	OSBPL6, MIF
TC02001071.hg.1	2.23	JUC02008474.hg.1	OSBPL6, MIF
TC02001071.hg.1	2.22	JUC02008441.hg.1	OSBPL6, MIF
TC02001071.hg.1	-2.09	PSR02015893.hg.1	OSBPL6, MIF
TC02001071.hg.1	-2.17	PSR02015894.hg.1	OSBPL6, MIF
TC02001071.hg.1	-2.28	PSR02015903.hg.1	OSBPL6, MIF
TC02001071.hg.1	-2.4	PSR02015933.hg.1	OSBPL6, MIF
TC02001071.hg.1	-2.61	PSR02015890.hg.1	OSBPL6, MIF
TC04001199.hg.1	2.48	PSR04016432.hg.1	LNX1
TC04001199.hg.1	2.3	JUC04008753.hg.1	LNX1
TC04001199.hg.1	2.03	JUC04008749.hg.1	LNX1
TC04001199.hg.1	-2	PSR04016460.hg.1	LNX1
TC04001199.hg.1	-2.3	JUC04008761.hg.1	LNX1
TC05001725.hg.1	2.48	PSR05023246.hg.1	CEP120
TC05001725.hg.1	2.4	JUC05012018.hg.1	CEP120
TC05001725.hg.1	2.14	PSR05023250.hg.1	CEP120
TC05001725.hg.1	2.09	JUC05012041.hg.1	CEP120
TC05001725.hg.1	2.07	JUC05012027.hg.1	CEP120
TC05001725.hg.1	2.02	JUC05012023.hg.1	CEP120
TC05001725.hg.1	-2.05	JUC05012040.hg.1	CEP120
TC06004132.hg.1	2.48	JUC06021814.hg.1	MOCS1
TC06004132.hg.1	2.38	PSR06020333.hg.1	MOCS1
TC06004132.hg.1	2.29	PSR06020329.hg.1	MOCS1
TC07001116.hg.1	2.48	JUC07008355.hg.1	TNRC18
TC07001116.hg.1	2.28	JUC07008350.hg.1	TNRC18
TC07001116.hg.1	2.19	PSR07017189.hg.1	TNRC18
TC07001116.hg.1	2.03	PSR07017153.hg.1	TNRC18
TC07001687.hg.1	2.48	JUC07012820.hg.1	PLOD3
TC07001687.hg.1	-2.03	PSR07026096.hg.1	PLOD3
TC08000176.hg.1	2.48	JUC08001173.hg.1	LOC1005071
TC08000176.hg.1	2.04	PSR08002281.hg.1	LOC1005071
TC09001412.hg.1	2.48	JUC09009465.hg.1	
TC09001412.hg.1	-2.05	PSR09017617.hg.1	
TC09001412.hg.1	-2.09	JUC09009470.hg.1	
TC10000922.hg.1	2.48	JUC10006641.hg.1	PTPRE

TC10000922.hg.1	2.11	PSR10011342.hg.1	PTPRE
TC10000922.hg.1	2.05	PSR10011359.hg.1	PTPRE
TC10000922.hg.1	2.01	JUC10006620.hg.1	PTPRE
TC10000922.hg.1	2.01	JUC10006625.hg.1	PTPRE
TC10000922.hg.1	-2.24	JUC10006621.hg.1	PTPRE
TC10000922.hg.1	-2.44	JUC10006647.hg.1	PTPRE
TC10000922.hg.1	-2.59	JUC10006640.hg.1	PTPRE
TC11001416.hg.1	2.48	JUC11009515.hg.1	MRVI1
TC11001416.hg.1	-2.02	PSR11017819.hg.1	MRVI1
TC11001416.hg.1	-2.02	JUC11009519.hg.1	MRVI1
TC11001416.hg.1	-2.07	JUC11009520.hg.1	MRVI1
TC11001416.hg.1	-2.26	JUC11009529.hg.1	MRVI1
TC11001416.hg.1	-2.42	PSR11017818.hg.1	MRVI1
TC11001416.hg.1	-2.62	PSR11017809.hg.1	MRVI1
TC11001416.hg.1	-2.67	PSR11017811.hg.1	MRVI1
TC11001416.hg.1	-2.69	PSR11017816.hg.1	MRVI1
TC11001416.hg.1	-2.79	PSR11017821.hg.1	MRVI1
TC11001416.hg.1	-2.92	JUC11009514.hg.1	MRVI1
TC11001416.hg.1	-2.94	PSR11017817.hg.1	MRVI1
TC11001416.hg.1	-3.16	JUC11009540.hg.1	MRVI1
TC11001416.hg.1	-3.18	JUC11009545.hg.1	MRVI1
TC11001416.hg.1	-3.3	JUC11009513.hg.1	MRVI1
TC11001416.hg.1	-3.65	JUC11009511.hg.1	MRVI1
TC11001416.hg.1	-4.1	JUC11009539.hg.1	MRVI1
TC12000419.hg.1	2.48	JUC12002964.hg.1	KRT86
TC12000419.hg.1	-2.19	PSR12005372.hg.1	KRT86
TC12000419.hg.1	-3.84	JUC12002968.hg.1	KRT86
TC12001413.hg.1	2.48	JUC12010219.hg.1	TWF1
TC12001413.hg.1	-2.14	PSR12018308.hg.1	TWF1
TC13000209.hg.1	2.48	JUC13001603.hg.1	RNASEH2B
TC13000209.hg.1	-2.17	PSR13002534.hg.1	RNASEH2B
TC13000209.hg.1	-2.26	PSR13002526.hg.1	RNASEH2B
TC13000209.hg.1	-2.26	JUC13001591.hg.1	RNASEH2B
TC13000510.hg.1	2.48	JUC13003436.hg.1	MTIF3
TC13000510.hg.1	2.39	PSR13005732.hg.1	MTIF3
TC13000510.hg.1	-2.07	PSR13005743.hg.1	MTIF3
TC13000510.hg.1	-2.22	PSR13005746.hg.1	MTIF3
TC13000510.hg.1	-3.37	JUC13003442.hg.1	MTIF3
TC16000295.hg.1	2.48	JUC16002276.hg.1	KIAA0556
TC16000295.hg.1	-2.69	PSR16004128.hg.1	KIAA0556
TC17000884.hg.1	2.48	PSR17011456.hg.1	SCARNA16, S
TC17000884.hg.1	2.04	PSR17011496.hg.1	SCARNA16, S
TC17000884.hg.1	-2.07	JUC17006406.hg.1	SCARNA16, S
TC17000884.hg.1	-2.2	JUC17006423.hg.1	SCARNA16, S
TC17001300.hg.1	2.48	PSR17017253.hg.1	PIGS
TC17001300.hg.1	2.22	JUC17009754.hg.1	PIGS
TC17001300.hg.1	2.21	PSR17017275.hg.1	PIGS
TC17001300.hg.1	2.02	PSR17017249.hg.1	PIGS
TC17001300.hg.1	2	PSR17017262.hg.1	PIGS
TC01003876.hg.1	2.47	PSR01059069.hg.1	PARP1
TC01003876.hg.1	2.25	JUC01030941.hg.1	PARP1
TC01003876.hg.1	2.23	JUC01030935.hg.1	PARP1
TC01003876.hg.1	2.21	PSR01059071.hg.1	PARP1
TC01003876.hg.1	2.17	PSR01059040.hg.1	PARP1
TC01003876.hg.1	2.14	PSR01059063.hg.1	PARP1
TC01003876.hg.1	2.12	PSR01059033.hg.1	PARP1
TC01003876.hg.1	2.11	JUC01030945.hg.1	PARP1
TC01003876.hg.1	2.09	PSR01059061.hg.1	PARP1

TC01003876.hg.1	2.07	PSR01059070.hg.1	PARP1
TC02000059.hg.1	2.47	JUC02000423.hg.1	HPCAL1
TC02000059.hg.1	2.29	JUC02000430.hg.1	HPCAL1
TC02000059.hg.1	2.21	PSR02000772.hg.1	HPCAL1
TC02001485.hg.1	2.47	JUC02012577.hg.1	ING5
TC02001485.hg.1	2.09	PSR02024233.hg.1	ING5
TC03000221.hg.1	2.47	JUC03002356.hg.1	NKTR
TC03000221.hg.1	2.32	JUC03002348.hg.1	NKTR
TC03000221.hg.1	2.14	JUC03002365.hg.1	NKTR
TC03000221.hg.1	2.05	PSR03004450.hg.1	NKTR
TC03000221.hg.1	-2.04	JUC03002341.hg.1	NKTR
TC03001549.hg.1	2.47	PSR03028268.hg.1	EIF4E3
TC03001549.hg.1	2.27	PSR03028263.hg.1	EIF4E3
TC03001549.hg.1	2.18	PSR03028265.hg.1	EIF4E3
TC03001549.hg.1	2.17	JUC03013909.hg.1	EIF4E3
TC03001979.hg.1	2.47	JUC03017481.hg.1	MECOM
TC03001979.hg.1	2.15	JUC03017488.hg.1	MECOM
TC03001979.hg.1	2.13	PSR03035447.hg.1	MECOM
TC03001979.hg.1	2.07	PSR03035445.hg.1	MECOM
TC03001979.hg.1	-2.04	PSR03035462.hg.1	MECOM
TC03001979.hg.1	-2.06	PSR03035463.hg.1	MECOM
TC03001979.hg.1	-2.18	PSR03035430.hg.1	MECOM
TC03001979.hg.1	-2.38	JUC03017495.hg.1	MECOM
TC03001979.hg.1	-2.41	PSR03035474.hg.1	MECOM
TC03001979.hg.1	-2.62	JUC03017505.hg.1	MECOM
TC03001979.hg.1	-2.67	PSR03035478.hg.1	MECOM
TC03001979.hg.1	-2.69	PSR03035461.hg.1	MECOM
TC03001979.hg.1	-2.71	PSR03035482.hg.1	MECOM
TC03001979.hg.1	-2.72	JUC03017506.hg.1	MECOM
TC03001979.hg.1	-2.81	PSR03035475.hg.1	MECOM
TC03001979.hg.1	-2.85	PSR03035477.hg.1	MECOM
TC03001979.hg.1	-3.65	JUC03017487.hg.1	MECOM
TC03001979.hg.1	-5.08	JUC03017490.hg.1	MECOM
TC05000305.hg.1	2.47	PSR05004155.hg.1	GTF2H2D, G
TC05000305.hg.1	2.13	JUC05002203.hg.1	GTF2H2D, G
TC05000305.hg.1	-2.02	PSR05004153.hg.1	GTF2H2D, G
TC05000305.hg.1	-2.74	PSR05004120.hg.1	GTF2H2D, G
TC05000305.hg.1	-3.38	JUC05002195.hg.1	GTF2H2D, G
TC05001310.hg.1	2.47	PSR05018528.hg.1	SEPP1
TC05001310.hg.1	2.37	JUC05009531.hg.1	SEPP1
TC05001310.hg.1	2.24	PSR05018525.hg.1	SEPP1
TC05001310.hg.1	2.15	JUC05009523.hg.1	SEPP1
TC05001310.hg.1	-2.04	PSR05018516.hg.1	SEPP1
TC05001310.hg.1	-2.07	PSR05018509.hg.1	SEPP1
TC05001310.hg.1	-2.16	PSR05018512.hg.1	SEPP1
TC05001310.hg.1	-2.21	JUC05009529.hg.1	SEPP1
TC06001235.hg.1	2.47	JUC06007317.hg.1	ECI2
TC06001235.hg.1	2.04	PSR06014704.hg.1	ECI2
TC07000019.hg.1	2.47	PSR07000280.hg.1	
TC07000019.hg.1	-2.21	JUC07000080.hg.1	
TC07000019.hg.1	-2.53	JUC07000076.hg.1	
TC09001463.hg.1	2.47	PSR09018095.hg.1	FRRS1L
TC09001463.hg.1	-2.98	JUC09009760.hg.1	FRRS1L
TC09001501.hg.1	2.47	PSR09018737.hg.1	HDHD3
TC09001501.hg.1	2.13	JUC09010136.hg.1	HDHD3
TC14001123.hg.1	2.47	JUC14006422.hg.1	NIN
TC14001123.hg.1	2.45	PSR14012760.hg.1	NIN
TC14001123.hg.1	2.24	JUC14006450.hg.1	NIN

TC15001713.hg.1	2.47	PSR15016752.hg.1	CTSH
TC15001713.hg.1	2.14	JUC15009131.hg.1	CTSH
TC15001713.hg.1	2.05	JUC15009128.hg.1	CTSH
TC15001713.hg.1	-2.44	JUC15009140.hg.1	CTSH
TC15001713.hg.1	-2.85	JUC15009130.hg.1	CTSH
TC15001713.hg.1	-3.42	PSR15016755.hg.1	CTSH
TC17_ctg5_hap10(	2.47	PSR17_ctg5_hap100	ARL17B, ARL
TC17_ctg5_hap10(	2.36	JUC17_ctg5_hap100	ARL17B, ARL
TC17_ctg5_hap10(	2.28	JUC17_ctg5_hap100	ARL17B, ARL
TC17_ctg5_hap10(	-2.18	PSR17_ctg5_hap100	ARL17B, ARL
TC20000826.hg.1	2.47	JUC20005902.hg.1	BLCAP
TC20000826.hg.1	2.38	JUC20005905.hg.1	BLCAP
TC20000826.hg.1	2.33	PSR20011406.hg.1	BLCAP
TC6_dbb_hap3000	2.47	PSR6_dbb_hap3001	SAPCD1, MS
TC6_dbb_hap3000	2.41	JUC6_dbb_hap3000	SAPCD1, MS
TC6_dbb_hap3000	2.37	JUC6_dbb_hap3000	SAPCD1, MS
TC6_dbb_hap3000	2.16	JUC6_dbb_hap3000	SAPCD1, MS
TC6_dbb_hap3000	2.03	JUC6_dbb_hap3000	SAPCD1, MS
TC6_dbb_hap3000	-2.01	JUC6_dbb_hap3000	SAPCD1, MS
TC6_dbb_hap3000	-2.09	JUC6_dbb_hap3000	SAPCD1, MS
TC6_dbb_hap3000	-2.41	JUC6_dbb_hap3000	SAPCD1, MS
TC6_dbb_hap3000	-2.94	JUC6_dbb_hap3000	SAPCD1, MS
TC6_mann_hap40(	2.47	PSR6_mann_hap40(	PRRC2A
TC6_mann_hap40(	2.36	PSR6_mann_hap40(	PRRC2A
TC6_mann_hap40(	2.26	JUC6_mann_hap400	PRRC2A
TC6_mann_hap40(	2.04	PSR6_mann_hap40(	PRRC2A
TC6_mcf_hap5000	2.47	PSR6_mcf_hap5001(	SAPCD1, MS
TC6_mcf_hap5000	2.41	JUC6_mcf_hap5000	SAPCD1, MS
TC6_mcf_hap5000	2.37	JUC6_mcf_hap5000	SAPCD1, MS
TC6_mcf_hap5000	2.16	JUC6_mcf_hap5000	SAPCD1, MS
TC6_mcf_hap5000	2.03	JUC6_mcf_hap5000	SAPCD1, MS
TC6_mcf_hap5000	-2.01	JUC6_mcf_hap5000	SAPCD1, MS
TC6_mcf_hap5000	-2.09	JUC6_mcf_hap5000	SAPCD1, MS
TC6_mcf_hap5000	-2.41	JUC6_mcf_hap5000	SAPCD1, MS
TC6_mcf_hap5000	-2.94	JUC6_mcf_hap5000	SAPCD1, MS
TC6_ssto_hap700(	2.47	JUC6_ssto_hap7000	SYNGAP1
TC6_ssto_hap700(	2.43	PSR6_ssto_hap7001	SYNGAP1
TC6_ssto_hap700(	2.25	JUC6_ssto_hap7000	SYNGAP1
TC02001177.hg.1	2.46	JUC02009566.hg.1	CASP8
TC02001177.hg.1	2.32	PSR02017903.hg.1	CASP8
TC02001177.hg.1	-2.15	PSR02017926.hg.1	CASP8
TC02004926.hg.1	2.46	JUC02031304.hg.1	SLC4A5
TC02004926.hg.1	2.21	PSR02031762.hg.1	SLC4A5
TC11000747.hg.1	2.46	PSR11009722.hg.1	IL18BP
TC11000747.hg.1	2.31	PSR11009721.hg.1	IL18BP
TC11000747.hg.1	-4.4	JUC11005029.hg.1	IL18BP
TC11000797.hg.1	2.46	JUC11005334.hg.1	NEU3
TC11000797.hg.1	2.43	PSR11010238.hg.1	NEU3
TC11000797.hg.1	2.33	PSR11010230.hg.1	NEU3
TC11000797.hg.1	2.12	JUC11005332.hg.1	NEU3
TC12001548.hg.1	2.46	JUC12011374.hg.1	SP7
TC12001548.hg.1	-2.35	PSR12020458.hg.1	SP7
TC12001548.hg.1	-2.49	PSR12020464.hg.1	SP7
TC12001548.hg.1	-2.85	PSR12020460.hg.1	SP7
TC12001548.hg.1	-3.31	JUC12011372.hg.1	SP7
TC12003287.hg.1	2.46	PSR12027240.hg.1	VPS33A
TC12003287.hg.1	2.27	PSR12027246.hg.1	VPS33A
TC12003287.hg.1	-2.29	JUC12020562.hg.1	VPS33A

TC12003287.hg.1	-2.36	JUC12020552.hg.1	VPS33A
TC12003287.hg.1	-2.74	JUC12020555.hg.1	VPS33A
TC13000068.hg.1	2.46	PSR13000694.hg.1	CDK8
TC13000068.hg.1	-2.02	JUC13000425.hg.1	CDK8
TC14000164.hg.1	2.46	JUC14000757.hg.1	TSSK4
TC14000164.hg.1	2.15	PSR14001756.hg.1	TSSK4
TC14000164.hg.1	2.04	PSR14001753.hg.1	TSSK4
TC15000810.hg.1	2.46	JUC15003856.hg.1	
TC15000810.hg.1	-2.79	PSR15007395.hg.1	
TC19001281.hg.1	2.46	PSR19018200.hg.1	IL12RB1
TC19001281.hg.1	-3.02	JUC19010618.hg.1	IL12RB1
TC02002300.hg.1	2.46	JUC02019135.hg.1	LOC1001313
TC02002300.hg.1	2	PSR02036903.hg.1	LOC1001313
TC03000127.hg.1	2.45	JUC03001315.hg.1	NR1D2
TC03000127.hg.1	2.24	JUC03001321.hg.1	NR1D2
TC03000127.hg.1	2.09	PSR03002454.hg.1	NR1D2
TC03000986.hg.1	2.45	PSR03016951.hg.1	YEATS2
TC03000986.hg.1	2.34	PSR03016981.hg.1	YEATS2
TC03000986.hg.1	2.27	JUC03008631.hg.1	YEATS2
TC03000986.hg.1	2.07	JUC03008639.hg.1	YEATS2
TC03000986.hg.1	-2.12	JUC03008657.hg.1	YEATS2
TC05001519.hg.1	2.45	PSR05020816.hg.1	ARSB
TC05001519.hg.1	2.32	JUC05010726.hg.1	ARSB
TC05001519.hg.1	2.06	PSR05020813.hg.1	ARSB
TC05001519.hg.1	-2.37	JUC05010723.hg.1	ARSB
TC05001519.hg.1	-5.11	PSR05020812.hg.1	ARSB
TC05001519.hg.1	-7.36	PSR05020811.hg.1	ARSB
TC06000761.hg.1	2.45	JUC06004222.hg.1	TTK
TC06000761.hg.1	-2.74	JUC06004217.hg.1	TTK
TC06000761.hg.1	-4.09	PSR06009100.hg.1	TTK
TC07001033.hg.1	2.45	JUC07007763.hg.1	LOC1001327
TC07001033.hg.1	2.2	PSR07016025.hg.1	LOC1001327
TC07001033.hg.1	2.05	PSR07016023.hg.1	LOC1001327
TC08000638.hg.1	2.45	PSR08008663.hg.1	
TC08000638.hg.1	2.28	JUC08004407.hg.1	
TC08000638.hg.1	2.06	JUC08004398.hg.1	
TC08000638.hg.1	2.05	PSR08008679.hg.1	
TC08000638.hg.1	-2.03	PSR08008688.hg.1	
TC08000638.hg.1	-2.29	PSR08008695.hg.1	
TC08000638.hg.1	-4.23	PSR08008674.hg.1	
TC09001059.hg.1	2.45	PSR09014073.hg.1	TLN1
TC09001059.hg.1	2.41	PSR09014082.hg.1	TLN1
TC09001059.hg.1	2.37	JUC09007579.hg.1	TLN1
TC0X000748.hg.1	2.45	PSR0X009724.hg.1	ZFP92
TC0X000748.hg.1	-2.13	JUC0X004913.hg.1	ZFP92
TC0X000748.hg.1	-2.25	JUC0X004915.hg.1	ZFP92
TC0X000748.hg.1	-2.78	PSR0X009726.hg.1	ZFP92
TC10000661.hg.1	2.45	PSR10007420.hg.1	EXOC6
TC10000661.hg.1	2.39	JUC10004233.hg.1	EXOC6
TC10000661.hg.1	2.19	PSR10007449.hg.1	EXOC6
TC10000661.hg.1	-3.77	JUC10004216.hg.1	EXOC6
TC11002048.hg.1	2.45	JUC11012898.hg.1	LOC1001333
TC11002048.hg.1	-2.3	PSR11024424.hg.1	LOC1001333
TC13000383.hg.1	2.45	JUC13002486.hg.1	TNFSF13B
TC13000383.hg.1	-2.04	PSR13004120.hg.1	TNFSF13B
TC13000383.hg.1	-3.07	JUC13002479.hg.1	TNFSF13B
TC13001725.hg.1	2.45	JUC13008493.hg.1	CCDC169
TC13001725.hg.1	-2.03	JUC13008490.hg.1	CCDC169

TC13001725.hg.1	-2.05	PSR13006377.hg.1	CCDC169
TC13001725.hg.1	-2.2	PSR13006372.hg.1	CCDC169
TC13001725.hg.1	-2.49	JUC13008485.hg.1	CCDC169
TC13001725.hg.1	-2.78	JUC13008484.hg.1	CCDC169
TC15000796.hg.1	2.45	JUC15003767.hg.1	TM6SF1
TC15000796.hg.1	-2.09	PSR15007249.hg.1	TM6SF1
TC15000796.hg.1	-3.5	PSR15007247.hg.1	TM6SF1
TC17000549.hg.1	2.45	PSR17006720.hg.1	TMEM106A
TC17000549.hg.1	2.36	JUC17003784.hg.1	TMEM106A
TC17000549.hg.1	-2.13	JUC17003801.hg.1	TMEM106A
TC17000549.hg.1	-2.35	JUC17003795.hg.1	TMEM106A
TC17000549.hg.1	-3.37	JUC17003793.hg.1	TMEM106A
TC17000549.hg.1	-4.25	JUC17003794.hg.1	TMEM106A
TC17000549.hg.1	-7	JUC17003785.hg.1	TMEM106A
TC19000062.hg.1	2.45	PSR19000955.hg.1	TLE6
TC19000062.hg.1	2.37	PSR19000989.hg.1	TLE6
TC19000062.hg.1	2.23	JUC19000532.hg.1	TLE6
TC20000231.hg.1	2.45	JUC20001642.hg.1	CBFA2T2
TC20000231.hg.1	-2.56	PSR20002905.hg.1	CBFA2T2
TC6_mann_hap40(	2.45	PSR6_mann_hap40(	ABCF1
TC6_mann_hap40(	2.45	JUC6_mann_hap400	ABCF1
TC6_mann_hap40(	2.07	PSR6_mann_hap40(	ABCF1
TC6_mcf_hap5000	2.45	PSR6_mcf_hap5000	ABCF1
TC6_mcf_hap5000	2.45	JUC6_mcf_hap5000	ABCF1
TC6_mcf_hap5000	2.07	PSR6_mcf_hap5000	ABCF1
TC02000748.hg.1	2.44	PSR02011610.hg.1	INSIG2
TC02000748.hg.1	2.23	PSR02011609.hg.1	INSIG2
TC02000748.hg.1	2.23	JUC02006145.hg.1	INSIG2
TC02000748.hg.1	2.16	JUC02006137.hg.1	INSIG2
TC02000748.hg.1	2.11	JUC02006139.hg.1	INSIG2
TC03000770.hg.1	2.44	JUC03007115.hg.1	TRPC1
TC03000770.hg.1	2.23	PSR03014089.hg.1	TRPC1
TC05000635.hg.1	2.44	JUC05004767.hg.1	SLC22A5
TC05000635.hg.1	2.13	PSR05008766.hg.1	SLC22A5
TC05000635.hg.1	2.05	JUC05004755.hg.1	SLC22A5
TC05000635.hg.1	-2.09	JUC05004756.hg.1	SLC22A5
TC05000635.hg.1	-2.12	PSR05008791.hg.1	SLC22A5
TC05000635.hg.1	-2.21	JUC05004753.hg.1	SLC22A5
TC05001032.hg.1	2.44	JUC05007811.hg.1	RMND5B
TC05001032.hg.1	2.42	PSR05015049.hg.1	RMND5B
TC06004112.hg.1	2.44	JUC06021535.hg.1	GABBR1
TC06004112.hg.1	2.09	PSR06016676.hg.1	GABBR1
TC06004112.hg.1	-2.36	JUC06021544.hg.1	GABBR1
TC07001016.hg.1	2.44	JUC07007630.hg.1	NUB1
TC07001016.hg.1	-2.87	PSR07015759.hg.1	NUB1
TC11000054.hg.1	2.44	JUC11000524.hg.1	TNNT3
TC11000054.hg.1	2.03	PSR11001080.hg.1	TNNT3
TC11000054.hg.1	-2.42	JUC11000509.hg.1	TNNT3
TC11000054.hg.1	-2.42	JUC11000530.hg.1	TNNT3
TC11000054.hg.1	-4.61	JUC11000543.hg.1	TNNT3
TC11000577.hg.1	2.44	JUC11003634.hg.1	MARK2
TC11000577.hg.1	2.25	JUC11003629.hg.1	MARK2
TC11000577.hg.1	-2.15	PSR11007002.hg.1	MARK2
TC11000577.hg.1	-2.61	JUC11003644.hg.1	MARK2
TC12001083.hg.1	2.44	JUC12007875.hg.1	NINJ2
TC12001083.hg.1	2.22	PSR12014332.hg.1	NINJ2
TC12001083.hg.1	-2.36	PSR12014336.hg.1	NINJ2
TC12001083.hg.1	-2.77	JUC12007877.hg.1	NINJ2

TC12001083.hg.1	-2.79	PSR12014337.hg.1	NINJ2
TC12001083.hg.1	-3.34	PSR12014341.hg.1	NINJ2
TC12001083.hg.1	-4.61	PSR12014334.hg.1	NINJ2
TC12001083.hg.1	-4.66	PSR12014333.hg.1	NINJ2
TC12001083.hg.1	-5.44	PSR12014327.hg.1	NINJ2
TC12001148.hg.1	2.44	JUC12008387.hg.1	C12orf53
TC12001148.hg.1	-2.03	PSR12015214.hg.1	C12orf53
TC12001148.hg.1	-2.59	PSR12015212.hg.1	C12orf53
TC12001148.hg.1	-2.65	PSR12015211.hg.1	C12orf53
TC12001148.hg.1	-4.25	JUC12008391.hg.1	C12orf53
TC12001148.hg.1	-6.77	JUC12008385.hg.1	C12orf53
TC13000419.hg.1	2.44	PSR13004702.hg.1	CUL4A
TC13000419.hg.1	2.11	JUC13002850.hg.1	CUL4A
TC13000419.hg.1	-2.77	JUC13002836.hg.1	CUL4A
TC14000356.hg.1	2.44	PSR14003881.hg.1	DAAM1
TC14000356.hg.1	-2.04	JUC14001933.hg.1	DAAM1
TC14000356.hg.1	-2.13	JUC14001930.hg.1	DAAM1
TC15002754.hg.1	2.44	JUC15013110.hg.1	ULK4P1, ULK
TC15002754.hg.1	-2.2	JUC15013106.hg.1	ULK4P1, ULK
TC15002754.hg.1	-2.24	PSR15001112.hg.1	ULK4P1, ULK
TC15002754.hg.1	-2.37	PSR15001111.hg.1	ULK4P1, ULK
TC15002801.hg.1	2.44	PSR15005984.hg.1	CLK3
TC15002801.hg.1	2.17	PSR15005991.hg.1	CLK3
TC15002801.hg.1	2.13	PSR15005985.hg.1	CLK3
TC15002801.hg.1	2	PSR15005979.hg.1	CLK3
TC15002801.hg.1	-2.97	JUC15014070.hg.1	CLK3
TC19002708.hg.1	2.44	JUC19018095.hg.1	PSG1
TC19002708.hg.1	-2.87	PSR19021578.hg.1	PSG1
TC19002708.hg.1	-4.48	JUC19018094.hg.1	PSG1
TC19002708.hg.1	-5.76	JUC19018091.hg.1	PSG1
TC19002708.hg.1	-9.54	JUC19018089.hg.1	PSG1
TC20000088.hg.1	2.44	PSR20001212.hg.1	BTBD3
TC20000088.hg.1	-3.21	JUC20000633.hg.1	BTBD3
TC01000727.hg.1	2.43	PSR01011556.hg.1	RAVER2
TC01000727.hg.1	-2.61	JUC01006000.hg.1	RAVER2
TC01003505.hg.1	2.43	JUC01028451.hg.1	KIFAP3
TC01003505.hg.1	2.12	PSR01054541.hg.1	KIFAP3
TC02002750.hg.1	2.43	JUC02023214.hg.1	LINC00607
TC02002750.hg.1	-2.12	PSR02044212.hg.1	LINC00607
TC02002750.hg.1	-2.5	PSR02044214.hg.1	LINC00607
TC02002750.hg.1	-2.53	PSR02044216.hg.1	LINC00607
TC02002750.hg.1	-2.94	PSR02044223.hg.1	LINC00607
TC02002750.hg.1	-3.13	JUC02023215.hg.1	LINC00607
TC02002750.hg.1	-3.26	PSR02044217.hg.1	LINC00607
TC02002750.hg.1	-3.48	JUC02023217.hg.1	LINC00607
TC02002750.hg.1	-3.53	JUC02023224.hg.1	LINC00607
TC02002750.hg.1	-3.68	PSR02044222.hg.1	LINC00607
TC02002750.hg.1	-3.91	PSR02044213.hg.1	LINC00607
TC02002750.hg.1	-3.96	PSR02044230.hg.1	LINC00607
TC02002750.hg.1	-4.1	PSR02044215.hg.1	LINC00607
TC02002750.hg.1	-4.35	JUC02023213.hg.1	LINC00607
TC02002750.hg.1	-4.44	PSR02044235.hg.1	LINC00607
TC02002750.hg.1	-4.47	PSR02044231.hg.1	LINC00607
TC02002750.hg.1	-5.15	PSR02044233.hg.1	LINC00607
TC02002750.hg.1	-5.41	PSR02044224.hg.1	LINC00607
TC02002750.hg.1	-6.34	JUC02023220.hg.1	LINC00607
TC02002750.hg.1	-7.14	JUC02023218.hg.1	LINC00607
TC02002750.hg.1	-7.21	PSR02044229.hg.1	LINC00607

TC02002750.hg.1	-7.58	JUC02023219.hg.1	LINC00607
TC02002750.hg.1	-7.78	PSR02044219.hg.1	LINC00607
TC02002750.hg.1	-8.19	JUC02023211.hg.1	LINC00607
TC03000064.hg.1	2.43	JUC03000734.hg.1	HRH1
TC03000064.hg.1	-2.07	PSR03001400.hg.1	HRH1
TC03000064.hg.1	-2.18	JUC03000729.hg.1	HRH1
TC03000131.hg.1	2.43	JUC03001345.hg.1	RARB
TC03000131.hg.1	2.3	PSR03002507.hg.1	RARB
TC03000131.hg.1	2.27	JUC03001338.hg.1	RARB
TC03000131.hg.1	-2	PSR03002513.hg.1	RARB
TC03000131.hg.1	-2.11	PSR03002492.hg.1	RARB
TC03000131.hg.1	-2.28	PSR03002518.hg.1	RARB
TC03000131.hg.1	-2.43	PSR03002487.hg.1	RARB
TC03000131.hg.1	-2.55	JUC03001339.hg.1	RARB
TC03000131.hg.1	-2.63	PSR03002491.hg.1	RARB
TC03000131.hg.1	-2.67	JUC03001330.hg.1	RARB
TC03000131.hg.1	-3.1	JUC03001329.hg.1	RARB
TC03000131.hg.1	-3.2	JUC03001340.hg.1	RARB
TC03000131.hg.1	-3.55	PSR03002490.hg.1	RARB
TC03000131.hg.1	-3.78	PSR03002488.hg.1	RARB
TC03000131.hg.1	-4.34	PSR03002502.hg.1	RARB
TC03000131.hg.1	-5.06	PSR03002489.hg.1	RARB
TC03000131.hg.1	-6.17	JUC03001341.hg.1	RARB
TC03000995.hg.1	2.43	PSR03017266.hg.1	ABCF3
TC03000995.hg.1	2.06	PSR03017267.hg.1	ABCF3
TC03000995.hg.1	-2.23	JUC03008790.hg.1	ABCF3
TC03003413.hg.1	2.43	JUC03024613.hg.1	ITIH4
TC03003413.hg.1	2.18	JUC03024619.hg.1	ITIH4
TC03003413.hg.1	2.04	PSR03026542.hg.1	ITIH4
TC03003413.hg.1	-2.36	JUC03024638.hg.1	ITIH4
TC03003413.hg.1	-2.95	JUC03024629.hg.1	ITIH4
TC04000833.hg.1	2.43	JUC04006159.hg.1	TLL1
TC04000833.hg.1	2.28	PSR04011495.hg.1	TLL1
TC04000833.hg.1	2.17	JUC04006148.hg.1	TLL1
TC04000833.hg.1	-2.15	PSR04011502.hg.1	TLL1
TC05000622.hg.1	2.43	JUC05004692.hg.1	LYRM7
TC05000622.hg.1	2.4	PSR05008647.hg.1	LYRM7
TC05000622.hg.1	2	PSR05008634.hg.1	LYRM7
TC06001725.hg.1	2.43	JUC06009765.hg.1	USP49
TC06001725.hg.1	2.35	JUC06009757.hg.1	USP49
TC06001725.hg.1	2.26	PSR06020574.hg.1	USP49
TC07000191.hg.1	2.43	JUC07001416.hg.1	PLEKHA8
TC07000191.hg.1	2.26	PSR07002974.hg.1	PLEKHA8
TC07000191.hg.1	-2.08	PSR07002973.hg.1	PLEKHA8
TC07000191.hg.1	-3.53	PSR07002970.hg.1	PLEKHA8
TC07001055.hg.1	2.43	PSR07016265.hg.1	DNAJB6
TC07001055.hg.1	2.12	JUC07007892.hg.1	DNAJB6
TC07001055.hg.1	-2.32	PSR07016276.hg.1	DNAJB6
TC07001055.hg.1	-2.36	JUC07007880.hg.1	DNAJB6
TC07001055.hg.1	-2.83	PSR07016285.hg.1	DNAJB6
TC08001359.hg.1	2.43	PSR08017530.hg.1	ZNF704
TC08001359.hg.1	2.22	PSR08017532.hg.1	ZNF704
TC08001359.hg.1	2.14	PSR08017533.hg.1	ZNF704
TC08001359.hg.1	2.08	JUC08008934.hg.1	ZNF704
TC08001359.hg.1	2.07	PSR08017547.hg.1	ZNF704
TC09000864.hg.1	2.43	JUC09006210.hg.1	CBWD1
TC09000864.hg.1	2.12	JUC09006223.hg.1	CBWD1
TC09000864.hg.1	2.01	JUC09006225.hg.1	CBWD1

TC09000864.hg.1	-2.02	JUC09006228.hg.1	CBWD1
TC09000864.hg.1	-2.05	PSR09011506.hg.1	CBWD1
TC09000864.hg.1	-2.05	PSR09011522.hg.1	CBWD1
TC09000864.hg.1	-2.31	PSR09011519.hg.1	CBWD1
TC09000864.hg.1	-2.52	PSR09011514.hg.1	CBWD1
TC09000864.hg.1	-2.52	PSR09011533.hg.1	CBWD1
TC09000864.hg.1	-2.58	PSR09011525.hg.1	CBWD1
TC09000916.hg.1	2.43	PSR09012191.hg.1	NFIB
TC09000916.hg.1	-4.29	JUC09006646.hg.1	NFIB
TC0X000014.hg.1	2.43	PSR0X000215.hg.1	CD99
TC0X000014.hg.1	2.11	JUC0X000107.hg.1	CD99
TC0X000014.hg.1	-3.41	JUC0X000096.hg.1	CD99
TC12000986.hg.1	2.43	JUC12007131.hg.1	SNRNP35
TC12000986.hg.1	2.12	PSR12013041.hg.1	SNRNP35
TC12000986.hg.1	2.02	JUC12007136.hg.1	SNRNP35
TC12000986.hg.1	-2.25	JUC12007130.hg.1	SNRNP35
TC12000986.hg.1	-2.48	PSR12013046.hg.1	SNRNP35
TC12001276.hg.1	2.43	JUC12009110.hg.1	MGP
TC12001276.hg.1	-2.26	JUC12009113.hg.1	MGP
TC12001276.hg.1	-2.28	PSR12016616.hg.1	MGP
TC13000612.hg.1	2.43	PSR13006973.hg.1	EPSTI1
TC13000612.hg.1	2.09	JUC13004067.hg.1	EPSTI1
TC15000039.hg.1	2.43	JUC15000190.hg.1	LOC653061
TC15000039.hg.1	-2.17	PSR15000275.hg.1	LOC653061
TC15000039.hg.1	-2.64	JUC15000191.hg.1	LOC653061
TC16000370.hg.1	2.43	JUC16002943.hg.1	BCKDK
TC16000370.hg.1	2.29	PSR16005391.hg.1	BCKDK
TC16000370.hg.1	2.08	PSR16005388.hg.1	BCKDK
TC16000370.hg.1	2.01	PSR16005396.hg.1	BCKDK
TC19001532.hg.1	2.43	JUC19012002.hg.1	AKT2
TC19001532.hg.1	-2.14	PSR19020939.hg.1	AKT2
TC19001532.hg.1	-2.97	PSR19020936.hg.1	AKT2
TC20000801.hg.1	2.43	PSR20010776.hg.1	C20orf173
TC20000801.hg.1	2.24	JUC20005626.hg.1	C20orf173
TC21000162.hg.1	2.43	JUC21000714.hg.1	TTC3, TTC3F
TC21000162.hg.1	2.12	PSR21001424.hg.1	TTC3, TTC3F
TC21000162.hg.1	-2.1	JUC21000705.hg.1	TTC3, TTC3F
TC21000162.hg.1	-2.65	JUC21000724.hg.1	TTC3, TTC3F
TC21000162.hg.1	-2.71	JUC21000701.hg.1	TTC3, TTC3F
TC6_apd_hap1000	2.43	PSR6_apd_hap1000	SAPCD1, MS
TC6_apd_hap1000	2.13	JUC6_apd_hap1000	SAPCD1, MS
TC6_apd_hap1000	2	JUC6_apd_hap1000	SAPCD1, MS
TC6_apd_hap1000	-2.04	JUC6_apd_hap1000	SAPCD1, MS
TC6_apd_hap1000	-2.13	JUC6_apd_hap1000	SAPCD1, MS
TC6_apd_hap1000	-2.45	JUC6_apd_hap1000	SAPCD1, MS
TC6_apd_hap1000	-2.99	JUC6_apd_hap1000	SAPCD1, MS
TC6_cox_hap2000	2.43	PSR6_cox_hap2001	SAPCD1, MS
TC6_cox_hap2000	2.37	JUC6_cox_hap2000	SAPCD1, MS
TC6_cox_hap2000	2.33	JUC6_cox_hap2000	SAPCD1, MS
TC6_cox_hap2000	2.13	JUC6_cox_hap2000	SAPCD1, MS
TC6_cox_hap2000	2	JUC6_cox_hap2000	SAPCD1, MS
TC6_cox_hap2000	-2.04	JUC6_cox_hap2000	SAPCD1, MS
TC6_cox_hap2000	-2.13	JUC6_cox_hap2000	SAPCD1, MS
TC6_cox_hap2000	-2.45	JUC6_cox_hap2000	SAPCD1, MS
TC6_cox_hap2000	-2.99	JUC6_cox_hap2000	SAPCD1, MS
TC6_dbb_hap3000	2.43	PSR6_dbb_hap3001	PRRC2A
TC6_dbb_hap3000	2.33	PSR6_dbb_hap3001	PRRC2A
TC6_dbb_hap3000	2.22	JUC6_dbb_hap3000	PRRC2A

TC6_dbb_hap3000	2.01	PSR6_dbb_hap3000	PRRC2A
TC01001090.hg.1	2.42	JUC01008757.hg.1	TXNIP
TC01001090.hg.1	2.31	PSR01017160.hg.1	TXNIP
TC01001090.hg.1	-2.15	PSR01017177.hg.1	TXNIP
TC01001090.hg.1	-2.26	PSR01017165.hg.1	TXNIP
TC01001090.hg.1	-2.31	PSR01017174.hg.1	TXNIP
TC01001435.hg.1	2.42	PSR01022404.hg.1	PBX1
TC01001435.hg.1	2.35	PSR01022400.hg.1	PBX1
TC01001435.hg.1	-2.03	PSR01022395.hg.1	PBX1
TC01001435.hg.1	-2.06	JUC01011832.hg.1	PBX1
TC01001435.hg.1	-2.29	JUC01011807.hg.1	PBX1
TC01002197.hg.1	2.42	PSR01033955.hg.1	MTOR
TC01002197.hg.1	-2.07	PSR01033949.hg.1	MTOR
TC01002197.hg.1	-2.08	PSR01033914.hg.1	MTOR
TC01002197.hg.1	-2.2	PSR01033925.hg.1	MTOR
TC01002197.hg.1	-2.27	JUC01018188.hg.1	MTOR
TC01002197.hg.1	-2.4	PSR01033932.hg.1	MTOR
TC01002197.hg.1	-3.05	PSR01033954.hg.1	MTOR
TC01002876.hg.1	2.42	JUC01024086.hg.1	LOC1001315
TC01002876.hg.1	2.05	JUC01024070.hg.1	LOC1001315
TC01002876.hg.1	-2.26	PSR01044831.hg.1	LOC1001315
TC01002876.hg.1	-2.29	PSR01044846.hg.1	LOC1001315
TC01002876.hg.1	-3.03	JUC01024097.hg.1	LOC1001315
TC01003268.hg.1	2.42	JUC01026582.hg.1	S100A2
TC01003268.hg.1	2.33	JUC01026579.hg.1	S100A2
TC01003268.hg.1	2.19	JUC01026580.hg.1	S100A2
TC01003268.hg.1	2.06	PSR01050289.hg.1	S100A2
TC02001105.hg.1	2.42	JUC02008890.hg.1	COL3A1, MIR
TC02001105.hg.1	2.3	JUC02008896.hg.1	COL3A1, MIR
TC02001105.hg.1	2.26	PSR02016655.hg.1	COL3A1, MIR
TC02001105.hg.1	2.26	PSR02016659.hg.1	COL3A1, MIR
TC02001105.hg.1	2.2	JUC02008902.hg.1	COL3A1, MIR
TC02001105.hg.1	2.14	PSR02016620.hg.1	COL3A1, MIR
TC02001105.hg.1	2.1	PSR02016622.hg.1	COL3A1, MIR
TC02001105.hg.1	2.04	PSR02016675.hg.1	COL3A1, MIR
TC02001105.hg.1	2.03	JUC02008870.hg.1	COL3A1, MIR
TC02001105.hg.1	2.01	PSR02016651.hg.1	COL3A1, MIR
TC02001105.hg.1	2	JUC02008886.hg.1	COL3A1, MIR
TC02001105.hg.1	-2.1	PSR02016610.hg.1	COL3A1, MIR
TC02001238.hg.1	2.42	PSR02018982.hg.1	MAP2
TC02001238.hg.1	2.42	JUC02010201.hg.1	MAP2
TC02001238.hg.1	2.31	JUC02010190.hg.1	MAP2
TC02001238.hg.1	2.14	PSR02018986.hg.1	MAP2
TC02001238.hg.1	2.13	JUC02010194.hg.1	MAP2
TC02001238.hg.1	2.1	JUC02010182.hg.1	MAP2
TC02001238.hg.1	2.04	JUC02010208.hg.1	MAP2
TC02001238.hg.1	-2.39	JUC02010197.hg.1	MAP2
TC02001238.hg.1	-2.8	JUC02010198.hg.1	MAP2
TC03001074.hg.1	2.42	JUC03009503.hg.1	FLJ34208
TC03001074.hg.1	2.06	PSR03018722.hg.1	FLJ34208
TC03001074.hg.1	2.06	PSR03018728.hg.1	FLJ34208
TC03001292.hg.1	2.42	PSR03022165.hg.1	PLCD1
TC03001292.hg.1	2.38	PSR03022163.hg.1	PLCD1
TC03001292.hg.1	2.38	PSR03022175.hg.1	PLCD1
TC03001292.hg.1	2.34	PSR03022179.hg.1	PLCD1
TC03001292.hg.1	2.05	PSR03022184.hg.1	PLCD1
TC03001292.hg.1	2	PSR03022152.hg.1	PLCD1
TC03001292.hg.1	-2.39	JUC03011079.hg.1	PLCD1

TC03001292.hg.1	-2.77	JUC03011071.hg.1	PLCD1
TC03003366.hg.1	2.42	JUC03023915.hg.1	LOC440993
TC03003366.hg.1	2.11	JUC03023918.hg.1	LOC440993
TC03003366.hg.1	-2.3	PSR03018806.hg.1	LOC440993
TC04000074.hg.1	2.42	JUC04000762.hg.1	KIAA0232
TC04000074.hg.1	2.07	PSR04001651.hg.1	KIAA0232
TC04000074.hg.1	-2.03	JUC04000767.hg.1	KIAA0232
TC04000074.hg.1	-3.46	JUC04000759.hg.1	KIAA0232
TC04000770.hg.1	2.42	JUC04005674.hg.1	TRIM2
TC04000770.hg.1	2.25	PSR04010575.hg.1	TRIM2
TC04000770.hg.1	2.04	PSR04010574.hg.1	TRIM2
TC04000770.hg.1	-2	PSR04010590.hg.1	TRIM2
TC04000770.hg.1	-2.12	JUC04005688.hg.1	TRIM2
TC04000770.hg.1	-2.14	PSR04010593.hg.1	TRIM2
TC04000770.hg.1	-2.24	JUC04005679.hg.1	TRIM2
TC04000770.hg.1	-4.09	JUC04005691.hg.1	TRIM2
TC05001887.hg.1	2.42	PSR05026086.hg.1	NR3C1
TC05001887.hg.1	2.3	JUC05013299.hg.1	NR3C1
TC05001887.hg.1	2	PSR05026092.hg.1	NR3C1
TC06000383.hg.1	2.42	PSR06003906.hg.1	SAPCD1, MS
TC06000383.hg.1	2.36	JUC06001623.hg.1	SAPCD1, MS
TC06000383.hg.1	2.32	JUC06001611.hg.1	SAPCD1, MS
TC06000383.hg.1	2.12	JUC06001597.hg.1	SAPCD1, MS
TC06000383.hg.1	2	JUC06001605.hg.1	SAPCD1, MS
TC06000383.hg.1	-2.04	JUC06001600.hg.1	SAPCD1, MS
TC06000383.hg.1	-2.13	JUC06001628.hg.1	SAPCD1, MS
TC06000383.hg.1	-2.46	JUC06001632.hg.1	SAPCD1, MS
TC06000383.hg.1	-3	JUC06001626.hg.1	SAPCD1, MS
TC10000047.hg.1	2.42	PSR10000414.hg.1	NET1
TC10000047.hg.1	-2.33	JUC10000208.hg.1	NET1
TC10000047.hg.1	-2.67	PSR10000409.hg.1	NET1
TC10000323.hg.1	2.42	JUC10002166.hg.1	WDFY4
TC10000323.hg.1	-2.63	PSR10003720.hg.1	WDFY4
TC10000323.hg.1	-2.64	JUC10002143.hg.1	WDFY4
TC10000979.hg.1	2.42	JUC10007128.hg.1	KLF6
TC10000979.hg.1	-2.54	PSR10012314.hg.1	KLF6
TC14000411.hg.1	2.42	JUC14002482.hg.1	RAD51B
TC14000411.hg.1	-2.05	PSR14004861.hg.1	RAD51B
TC15002779.hg.1	2.42	PSR15015769.hg.1	HEXA
TC15002779.hg.1	2.34	JUC15013527.hg.1	HEXA
TC16000154.hg.1	2.42	PSR16002349.hg.1	ATF7IP2, LO(
TC16000154.hg.1	-2.46	JUC16001185.hg.1	ATF7IP2, LO(
TC22001461.hg.1	2.42	PSR22003972.hg.1	SEC14L2
TC22001461.hg.1	2.36	JUC22009208.hg.1	SEC14L2
TC22001461.hg.1	-5.13	JUC22009204.hg.1	SEC14L2
TC01000346.hg.1	2.41	JUC01002790.hg.1	HMGN2
TC01000346.hg.1	2.22	JUC01002783.hg.1	HMGN2
TC01000346.hg.1	2.05	PSR01005311.hg.1	HMGN2
TC01002374.hg.1	2.41	JUC01019722.hg.1	C1orf63
TC01002374.hg.1	-2.07	PSR01036618.hg.1	C1orf63
TC01002374.hg.1	-3.97	JUC01019711.hg.1	C1orf63
TC01002845.hg.1	2.41	PSR01044339.hg.1	GBP3
TC01002845.hg.1	2.4	JUC01023816.hg.1	GBP3
TC01002845.hg.1	2.16	PSR01044343.hg.1	GBP3
TC01002845.hg.1	2.07	JUC01023821.hg.1	GBP3
TC01002845.hg.1	-2.09	JUC01023822.hg.1	GBP3
TC01002845.hg.1	-2.26	PSR01044345.hg.1	GBP3
TC01002845.hg.1	-2.29	PSR01044330.hg.1	GBP3

TC01003560.hg.1	2.41	JUC01028778.hg.1	SEC16B, LO(
TC01003560.hg.1	2.38	JUC01028775.hg.1	SEC16B, LO(
TC01003560.hg.1	2.3	JUC01028792.hg.1	SEC16B, LO(
TC01003560.hg.1	2.17	PSR01055185.hg.1	SEC16B, LO(
TC01003560.hg.1	-2.16	JUC01028826.hg.1	SEC16B, LO(
TC01003560.hg.1	-3.09	JUC01028820.hg.1	SEC16B, LO(
TC01003560.hg.1	-3.73	JUC01028770.hg.1	SEC16B, LO(
TC03001487.hg.1	2.41	JUC03013144.hg.1	FAM208A
TC03001487.hg.1	2.3	PSR03027005.hg.1	FAM208A
TC03001487.hg.1	-2.75	JUC03013167.hg.1	FAM208A
TC05002011.hg.1	2.41	PSR05027963.hg.1	ATP10B
TC05002011.hg.1	2.3	JUC05014268.hg.1	ATP10B
TC05002011.hg.1	2.22	PSR05027982.hg.1	ATP10B
TC05002011.hg.1	-2.29	JUC05014250.hg.1	ATP10B
TC05002011.hg.1	-2.82	JUC05014281.hg.1	ATP10B
TC08001528.hg.1	2.41	PSR08020090.hg.1	NUDCD1
TC08001528.hg.1	2.36	JUC08010221.hg.1	NUDCD1
TC09000354.hg.1	2.41	PSR09003823.hg.1	VPS13A
TC09000354.hg.1	2.06	JUC09002059.hg.1	VPS13A
TC09000354.hg.1	-2.03	JUC09002057.hg.1	VPS13A
TC09000354.hg.1	-2.07	JUC09002080.hg.1	VPS13A
TC09000354.hg.1	-2.14	JUC09002045.hg.1	VPS13A
TC09000354.hg.1	-2.15	JUC09002087.hg.1	VPS13A
TC09000354.hg.1	-2.97	JUC09002079.hg.1	VPS13A
TC10000475.hg.1	2.41	PSR10005606.hg.1	PLAU
TC10000475.hg.1	2.02	JUC10003293.hg.1	PLAU
TC10000475.hg.1	-2.12	PSR10005605.hg.1	PLAU
TC10000475.hg.1	-4.25	JUC10003307.hg.1	PLAU
TC10000475.hg.1	-4.28	PSR10005623.hg.1	PLAU
TC10000475.hg.1	-5.16	JUC10003302.hg.1	PLAU
TC10000475.hg.1	-5.91	PSR10005594.hg.1	PLAU
TC10000475.hg.1	-7.11	PSR10005600.hg.1	PLAU
TC10000475.hg.1	-7.36	JUC10003304.hg.1	PLAU
TC10000475.hg.1	-7.43	JUC10003308.hg.1	PLAU
TC11000669.hg.1	2.41	JUC11004371.hg.1	PELI3
TC11000669.hg.1	2.1	PSR11008402.hg.1	PELI3
TC11000669.hg.1	2.03	PSR11008407.hg.1	PELI3
TC11001274.hg.1	2.41	PSR11016099.hg.1	INS, IGF2, IN
TC11001274.hg.1	2.2	JUC11008678.hg.1	INS, IGF2, IN
TC11001274.hg.1	-2.71	PSR11016103.hg.1	INS, IGF2, IN
TC11001274.hg.1	-3.77	PSR11016122.hg.1	INS, IGF2, IN
TC11001274.hg.1	-4.71	PSR11016138.hg.1	INS, IGF2, IN
TC11001274.hg.1	-4.71	PSR11016146.hg.1	INS, IGF2, IN
TC11001274.hg.1	-4.95	PSR11016135.hg.1	INS, IGF2, IN
TC11001274.hg.1	-5.24	PSR11016151.hg.1	INS, IGF2, IN
TC11001274.hg.1	-5.31	PSR11016129.hg.1	INS, IGF2, IN
TC11001274.hg.1	-5.39	PSR11016157.hg.1	INS, IGF2, IN
TC11001274.hg.1	-5.52	PSR11016140.hg.1	INS, IGF2, IN
TC11001274.hg.1	-5.57	PSR11016141.hg.1	INS, IGF2, IN
TC11001274.hg.1	-5.9	PSR11016144.hg.1	INS, IGF2, IN
TC11001274.hg.1	-6.43	JUC11008682.hg.1	INS, IGF2, IN
TC11001274.hg.1	-6.44	PSR11016148.hg.1	INS, IGF2, IN
TC11001274.hg.1	-6.52	PSR11016130.hg.1	INS, IGF2, IN
TC11001274.hg.1	-6.59	JUC11008676.hg.1	INS, IGF2, IN
TC11001274.hg.1	-6.65	JUC11008677.hg.1	INS, IGF2, IN
TC11001274.hg.1	-6.67	PSR11016147.hg.1	INS, IGF2, IN
TC11001274.hg.1	-6.78	PSR11016134.hg.1	INS, IGF2, IN
TC11001274.hg.1	-7.62	JUC11008680.hg.1	INS, IGF2, IN

TC11001274.hg.1	-7.69	JUC11008668.hg.1	INS, IGF2, IN
TC11001274.hg.1	-8.48	PSR11016133.hg.1	INS, IGF2, IN
TC11001274.hg.1	-8.93	JUC11008662.hg.1	INS, IGF2, IN
TC12001094.hg.1	2.41	PSR12014503.hg.1	DCP1B
TC12001094.hg.1	2.26	PSR12014493.hg.1	DCP1B
TC12001094.hg.1	-2.73	JUC12007972.hg.1	DCP1B
TC12001094.hg.1	-2.85	PSR12014505.hg.1	DCP1B
TC12002081.hg.1	2.41	PSR12027430.hg.1	ABCB9
TC12002081.hg.1	-2.5	JUC12015358.hg.1	ABCB9
TC15000315.hg.1	2.41	JUC15001205.hg.1	JMJD7, PLA2
TC15000315.hg.1	-2.06	PSR15002473.hg.1	JMJD7, PLA2
TC15000315.hg.1	-2.09	PSR15002453.hg.1	JMJD7, PLA2
TC15000315.hg.1	-2.18	JUC15001194.hg.1	JMJD7, PLA2
TC15000315.hg.1	-2.4	JUC15001220.hg.1	JMJD7, PLA2
TC15000315.hg.1	-2.65	JUC15001183.hg.1	JMJD7, PLA2
TC16000859.hg.1	2.41	JUC16006987.hg.1	FAM18A
TC16000859.hg.1	2.04	PSR16012402.hg.1	FAM18A
TC16000965.hg.1	2.41	JUC16007809.hg.1	EARS2
TC16000965.hg.1	-2.22	PSR16013885.hg.1	EARS2
TC6_cox_hap2000	2.41	PSR6_cox_hap2000	ABCF1
TC6_cox_hap2000	2.41	JUC6_cox_hap2000	ABCF1
TC6_cox_hap2000	2.04	PSR6_cox_hap2000	ABCF1
TC6_dbb_hap3000	2.41	PSR6_dbb_hap3000	ABCF1
TC6_dbb_hap3000	2.41	JUC6_dbb_hap3000	ABCF1
TC6_dbb_hap3000	2.04	PSR6_dbb_hap3000	ABCF1
TC6_qbl_hap6000	2.41	PSR6_qbl_hap60004	ABCF1
TC6_qbl_hap6000	2.41	JUC6_qbl_hap60001	ABCF1
TC6_qbl_hap6000	2.04	PSR6_qbl_hap60004	ABCF1
TC6_ssto_hap700	2.41	PSR6_ssto_hap7000	ABCF1
TC6_ssto_hap700	2.41	JUC6_ssto_hap7000	ABCF1
TC6_ssto_hap700	2.04	PSR6_ssto_hap7000	ABCF1
TC12000859.hg.1	2.41	JUC12006225.hg.1	TCTN1
TC12000859.hg.1	2.29	JUC12006226.hg.1	TCTN1
TC12000859.hg.1	2	PSR12011257.hg.1	TCTN1
TC01001596.hg.1	2.4	JUC01013185.hg.1	SWT1
TC01001596.hg.1	2.36	PSR01024732.hg.1	SWT1
TC01001596.hg.1	-2.01	JUC01013197.hg.1	SWT1
TC01001596.hg.1	-2.36	JUC01013183.hg.1	SWT1
TC01001596.hg.1	-2.51	JUC01013196.hg.1	SWT1
TC02001414.hg.1	2.4	PSR02022452.hg.1	INPP5D
TC02001414.hg.1	-2.55	PSR02022491.hg.1	INPP5D
TC02001414.hg.1	-2.9	JUC02011801.hg.1	INPP5D
TC02001414.hg.1	-3.13	JUC02011810.hg.1	INPP5D
TC02001414.hg.1	-4.81	JUC02011830.hg.1	INPP5D
TC03002083.hg.1	2.4	JUC03018313.hg.1	CRYGS
TC03002083.hg.1	2.27	PSR03037019.hg.1	CRYGS
TC05001289.hg.1	2.4	JUC05009277.hg.1	LIFR
TC05001289.hg.1	2.11	PSR05018061.hg.1	LIFR
TC05001289.hg.1	-2	PSR05018058.hg.1	LIFR
TC05001289.hg.1	-2.61	JUC05009274.hg.1	LIFR
TC05001289.hg.1	-2.64	PSR05018056.hg.1	LIFR
TC05001289.hg.1	-2.92	PSR05018065.hg.1	LIFR
TC05001289.hg.1	-3.01	PSR05018057.hg.1	LIFR
TC05001289.hg.1	-3.36	PSR05018036.hg.1	LIFR
TC05001289.hg.1	-3.41	PSR05018066.hg.1	LIFR
TC05001289.hg.1	-4.53	JUC05009280.hg.1	LIFR
TC06001062.hg.1	2.4	JUC06006134.hg.1	LOC1005075
TC06001062.hg.1	-2.35	PSR06012535.hg.1	LOC1005075

TC09000679.hg.1	2.4	JUC09004548.hg.1	DNM1
TC09000679.hg.1	2.18	PSR09008210.hg.1	DNM1
TC09000679.hg.1	2.18	JUC09004563.hg.1	DNM1
TC09000679.hg.1	2.16	JUC09004568.hg.1	DNM1
TC09000679.hg.1	2.03	JUC09004555.hg.1	DNM1
TC09000679.hg.1	-2	PSR09008206.hg.1	DNM1
TC09000679.hg.1	-2.15	JUC09004571.hg.1	DNM1
TC09000679.hg.1	-2.16	PSR09008216.hg.1	DNM1
TC09000679.hg.1	-2.21	PSR09008204.hg.1	DNM1
TC09000679.hg.1	-2.21	PSR09008239.hg.1	DNM1
TC09000679.hg.1	-2.3	PSR09008186.hg.1	DNM1
TC09000679.hg.1	-2.3	PSR09008223.hg.1	DNM1
TC09000679.hg.1	-2.33	PSR09008221.hg.1	DNM1
TC09000679.hg.1	-2.45	PSR09008227.hg.1	DNM1
TC09000679.hg.1	-2.47	JUC09004558.hg.1	DNM1
TC09000679.hg.1	-2.57	JUC09004542.hg.1	DNM1
TC09000679.hg.1	-2.61	PSR09008232.hg.1	DNM1
TC09000679.hg.1	-2.66	JUC09004554.hg.1	DNM1
TC09000679.hg.1	-2.68	PSR09008233.hg.1	DNM1
TC09000679.hg.1	-2.7	JUC09004543.hg.1	DNM1
TC09000679.hg.1	-2.89	PSR09008215.hg.1	DNM1
TC09000679.hg.1	-3.11	JUC09004553.hg.1	DNM1
TC09000679.hg.1	-3.21	PSR09008228.hg.1	DNM1
TC10001585.hg.1	2.4	JUC10011881.hg.1	DNMBP
TC10001585.hg.1	2.29	PSR10020408.hg.1	DNMBP
TC10001585.hg.1	2.2	JUC10011878.hg.1	DNMBP
TC10001585.hg.1	2.12	PSR10020415.hg.1	DNMBP
TC10001585.hg.1	-2.13	JUC10011880.hg.1	DNMBP
TC11002319.hg.1	2.4	JUC11014777.hg.1	LOC283143
TC11002319.hg.1	-2.25	PSR11027721.hg.1	LOC283143
TC11002319.hg.1	-2.37	PSR11027714.hg.1	LOC283143
TC14001140.hg.1	2.4	JUC14006547.hg.1	TXNDC16
TC14001140.hg.1	2.18	PSR14012921.hg.1	TXNDC16
TC14001140.hg.1	2.07	JUC14006536.hg.1	TXNDC16
TC15000010.hg.1	2.4	JUC15000028.hg.1	GOLGA8C
TC15000010.hg.1	2.3	JUC15000022.hg.1	GOLGA8C
TC15000010.hg.1	-2.13	PSR15000025.hg.1	GOLGA8C
TC15000134.hg.1	2.4	PSR15000825.hg.1	GOLGA8F, G
TC15000134.hg.1	-2.01	PSR15000830.hg.1	GOLGA8F, G
TC15000134.hg.1	-2.44	JUC15000273.hg.1	GOLGA8F, G
TC22000873.hg.1	2.4	JUC22006173.hg.1	KIAA0930
TC22000873.hg.1	2.24	JUC22006171.hg.1	KIAA0930
TC22000873.hg.1	2.1	PSR22014805.hg.1	KIAA0930
TC01000827.hg.1	2.39	JUC01006938.hg.1	HS2ST1, LO
TC01000827.hg.1	2.19	JUC01006941.hg.1	HS2ST1, LO
TC01000827.hg.1	2.07	PSR01013184.hg.1	HS2ST1, LO
TC02001297.hg.1	2.39	JUC02010640.hg.1	PNKD
TC02001297.hg.1	2.37	PSR02019796.hg.1	PNKD
TC02001297.hg.1	2.29	PSR02019787.hg.1	PNKD
TC02001297.hg.1	2.27	PSR02019788.hg.1	PNKD
TC02001297.hg.1	2.15	PSR02019805.hg.1	PNKD
TC02001297.hg.1	2.03	JUC02010636.hg.1	PNKD
TC02001297.hg.1	2.01	PSR02019808.hg.1	PNKD
TC02002467.hg.1	2.39	JUC02020302.hg.1	LY75-CD302,
TC02002467.hg.1	2.16	JUC02020314.hg.1	LY75-CD302,
TC02002467.hg.1	2.02	JUC02020323.hg.1	LY75-CD302,
TC02002467.hg.1	-2.03	PSR02038844.hg.1	LY75-CD302,
TC02002467.hg.1	-2.04	JUC02020285.hg.1	LY75-CD302,

TC02002467.hg.1	-3.13	JUC02020305.hg.1	LY75-CD302,
TC02004935.hg.1	2.39	PSR02002453.hg.1	GPN1
TC02004935.hg.1	2.07	JUC02031479.hg.1	GPN1
TC02004935.hg.1	-2.32	JUC02031477.hg.1	GPN1
TC03001247.hg.1	2.39	JUC03010588.hg.1	TOP2B, MIR4
TC03001247.hg.1	2.27	PSR03021351.hg.1	TOP2B, MIR4
TC03001247.hg.1	2.17	JUC03010598.hg.1	TOP2B, MIR4
TC03001247.hg.1	2.07	JUC03010576.hg.1	TOP2B, MIR4
TC03001247.hg.1	-2.79	JUC03010572.hg.1	TOP2B, MIR4
TC04001411.hg.1	2.39	PSR04019710.hg.1	ADH1C
TC04001411.hg.1	-2.07	JUC04010343.hg.1	ADH1C
TC04001411.hg.1	-2.44	PSR04019701.hg.1	ADH1C
TC04001411.hg.1	-3.72	JUC04010344.hg.1	ADH1C
TC06000683.hg.1	2.39	JUC06003625.hg.1	ZNF451
TC06000683.hg.1	2.09	PSR06008165.hg.1	ZNF451
TC06000683.hg.1	-2.03	JUC06003631.hg.1	ZNF451
TC06000683.hg.1	-2.06	PSR06008210.hg.1	ZNF451
TC06000683.hg.1	-2.1	JUC06003636.hg.1	ZNF451
TC06000683.hg.1	-2.14	PSR06008204.hg.1	ZNF451
TC06000683.hg.1	-2.16	PSR06008202.hg.1	ZNF451
TC06000683.hg.1	-2.33	PSR06008217.hg.1	ZNF451
TC06000683.hg.1	-2.44	PSR06008201.hg.1	ZNF451
TC06000683.hg.1	-2.58	JUC06003640.hg.1	ZNF451
TC06000683.hg.1	-2.72	PSR06008216.hg.1	ZNF451
TC06000683.hg.1	-2.73	JUC06003644.hg.1	ZNF451
TC06000683.hg.1	-2.99	JUC06003645.hg.1	ZNF451
TC07002016.hg.1	2.39	JUC07015278.hg.1	RARRES2
TC07002016.hg.1	-3.1	PSR07030811.hg.1	RARRES2
TC09000384.hg.1	2.39	JUC09002233.hg.1	RMI1
TC09000384.hg.1	2.3	PSR09004093.hg.1	RMI1
TC09000384.hg.1	2.14	PSR09004106.hg.1	RMI1
TC09000384.hg.1	2.02	JUC09002240.hg.1	RMI1
TC09000852.hg.1	2.39	PSR09011306.hg.1	EHMT1
TC09000852.hg.1	-2	JUC09006073.hg.1	EHMT1
TC09000852.hg.1	-2.2	JUC09006038.hg.1	EHMT1
TC09001333.hg.1	2.39	JUC09008951.hg.1	NOL8
TC09001333.hg.1	-2.08	JUC09008954.hg.1	NOL8
TC09001333.hg.1	-2.18	PSR09016706.hg.1	NOL8
TC09001333.hg.1	-2.56	PSR09016691.hg.1	NOL8
TC09001333.hg.1	-2.67	PSR09016663.hg.1	NOL8
TC09001333.hg.1	-2.87	JUC09008967.hg.1	NOL8
TC0X001059.hg.1	2.39	JUC0X007118.hg.1	FAM156B, FA
TC0X001059.hg.1	-2.13	PSR0X014244.hg.1	FAM156B, FA
TC0X001429.hg.1	2.39	JUC0X009699.hg.1	ATP11C
TC0X001429.hg.1	2.31	PSR0X019015.hg.1	ATP11C
TC0X001429.hg.1	2.24	JUC0X009698.hg.1	ATP11C
TC10000802.hg.1	2.39	JUC10005353.hg.1	SMC3
TC10000802.hg.1	2.19	PSR10009594.hg.1	SMC3
TC10000802.hg.1	2.14	JUC10005342.hg.1	SMC3
TC10000802.hg.1	-2.07	JUC10005367.hg.1	SMC3
TC11001341.hg.1	2.39	PSR11016899.hg.1	TRIM5
TC11001341.hg.1	2.19	JUC11009022.hg.1	TRIM5
TC11001341.hg.1	2.17	JUC11009039.hg.1	TRIM5
TC11001341.hg.1	2.11	JUC11009042.hg.1	TRIM5
TC11001341.hg.1	-2.05	JUC11009034.hg.1	TRIM5
TC11001341.hg.1	-2.17	PSR11016877.hg.1	TRIM5
TC11001341.hg.1	-2.23	JUC11009027.hg.1	TRIM5
TC15000873.hg.1	2.39	JUC15004444.hg.1	UNC45A

TC15000873.hg.1	2.26	JUC15004465.hg.1	UNC45A
TC15000873.hg.1	2.2	PSR15008345.hg.1	UNC45A
TC15000873.hg.1	2.2	PSR15008356.hg.1	UNC45A
TC17000975.hg.1	2.39	PSR17013006.hg.1	INPP5K
TC17000975.hg.1	2.15	JUC17007407.hg.1	INPP5K
TC19002635.hg.1	2.39	JUC19017399.hg.1	TMEM91
TC19002635.hg.1	-2.3	PSR19008018.hg.1	TMEM91
TC20000598.hg.1	2.39	JUC20004475.hg.1	
TC20000598.hg.1	2.32	PSR20008687.hg.1	
TC20000598.hg.1	2.27	PSR20008689.hg.1	
TC21000077.hg.1	2.39	JUC21000256.hg.1	JAM2
TC21000077.hg.1	2.34	PSR21000406.hg.1	JAM2
TC21000077.hg.1	-2.1	PSR21000402.hg.1	JAM2
TC21000077.hg.1	-2.45	JUC21000258.hg.1	JAM2
TC21000077.hg.1	-2.53	PSR21000404.hg.1	JAM2
TC21000077.hg.1	-3.17	JUC21000247.hg.1	JAM2
TC21000077.hg.1	-3.63	PSR21000400.hg.1	JAM2
TC22000080.hg.1	2.39	PSR22001241.hg.1	LOC400891
TC22000080.hg.1	2.03	JUC22000494.hg.1	LOC400891
TC01000186.hg.1	2.38	JUC01001688.hg.1	FHAD1
TC01000186.hg.1	2.05	PSR01002998.hg.1	FHAD1
TC01000800.hg.1	2.38	PSR01012799.hg.1	LPHN2
TC01000800.hg.1	2.2	JUC01006689.hg.1	LPHN2
TC01000800.hg.1	2.2	JUC01006712.hg.1	LPHN2
TC01000800.hg.1	-2.02	JUC01006694.hg.1	LPHN2
TC01000800.hg.1	-2.02	JUC01006709.hg.1	LPHN2
TC01000800.hg.1	-2.23	PSR01012777.hg.1	LPHN2
TC01000800.hg.1	-2.55	JUC01006714.hg.1	LPHN2
TC01001581.hg.1	2.38	JUC01012969.hg.1	NPL
TC01001581.hg.1	-2.76	JUC01012979.hg.1	NPL
TC01001581.hg.1	-2.84	JUC01012962.hg.1	NPL
TC01001581.hg.1	-2.98	JUC01012965.hg.1	NPL
TC01001581.hg.1	-3.26	PSR01024398.hg.1	NPL
TC01001581.hg.1	-3.76	JUC01012976.hg.1	NPL
TC02001281.hg.1	2.38	JUC02010556.hg.1	IGFBP2
TC02001281.hg.1	-2.12	PSR02019596.hg.1	IGFBP2
TC03000533.hg.1	2.38	PSR03009835.hg.1	
TC03000533.hg.1	-2.52	JUC03004886.hg.1	
TC03001194.hg.1	2.38	PSR03020513.hg.1	XPC
TC03001194.hg.1	2.1	PSR03020474.hg.1	XPC
TC03001194.hg.1	2.06	JUC03010254.hg.1	XPC
TC04001466.hg.1	2.38	PSR04020845.hg.1	CFI
TC04001466.hg.1	2.21	JUC04010952.hg.1	CFI
TC04001466.hg.1	2.13	JUC04010944.hg.1	CFI
TC04002933.hg.1	2.38	JUC04017114.hg.1	CPZ
TC04002933.hg.1	-3.41	PSR04001942.hg.1	CPZ
TC05001750.hg.1	2.38	JUC05012148.hg.1	FLJ33630
TC05001750.hg.1	-2.34	JUC05012146.hg.1	FLJ33630
TC05001750.hg.1	-2.38	PSR05023450.hg.1	FLJ33630
TC06000341.hg.1	2.38	PSR06002940.hg.1	ABCF1
TC06000341.hg.1	2.38	JUC06001278.hg.1	ABCF1
TC06000341.hg.1	2.01	PSR06002906.hg.1	ABCF1
TC06000511.hg.1	2.38	PSR06005395.hg.1	SCUBE3
TC06000511.hg.1	2.37	JUC06002157.hg.1	SCUBE3
TC06000511.hg.1	2.36	PSR06005393.hg.1	SCUBE3
TC06000511.hg.1	-2.23	JUC06002174.hg.1	SCUBE3
TC06000511.hg.1	-2.35	JUC06002161.hg.1	SCUBE3
TC06000511.hg.1	-2.7	JUC06002165.hg.1	SCUBE3

TC06000511.hg.1	-2.86	JUC06002170.hg.1	SCUBE3
TC10000895.hg.1	2.38	JUC10006391.hg.1	PSTK
TC10000895.hg.1	-2.06	PSR10010978.hg.1	PSTK
TC10001420.hg.1	2.38	JUC10010489.hg.1	CAMK2G
TC10001420.hg.1	-3.22	PSR10018012.hg.1	CAMK2G
TC11000820.hg.1	2.38	JUC11005427.hg.1	C11orf30
TC11000820.hg.1	-2.13	JUC11005429.hg.1	C11orf30
TC11000820.hg.1	-2.19	PSR11010465.hg.1	C11orf30
TC11000820.hg.1	-2.51	JUC11005450.hg.1	C11orf30
TC11000820.hg.1	-2.57	JUC11005443.hg.1	C11orf30
TC13000343.hg.1	2.38	JUC13002203.hg.1	IPO5
TC13000343.hg.1	2.12	PSR13003637.hg.1	IPO5
TC13000343.hg.1	2.08	JUC13002217.hg.1	IPO5
TC13000343.hg.1	-2.51	PSR13003621.hg.1	IPO5
TC13000343.hg.1	-2.79	JUC13002231.hg.1	IPO5
TC15000364.hg.1	2.38	PSR15003322.hg.1	SQRDL
TC15000364.hg.1	-2.25	JUC15001567.hg.1	SQRDL
TC16000158.hg.1	2.38	JUC16001239.hg.1	CIITA
TC16000158.hg.1	2.26	JUC16001215.hg.1	CIITA
TC16000158.hg.1	-2.23	PSR16002403.hg.1	CIITA
TC17000822.hg.1	2.38	PSR17010572.hg.1	COG1
TC17000822.hg.1	-2.44	JUC17005815.hg.1	COG1
TC19002605.hg.1	2.38	JUC19017006.hg.1	FLT3LG
TC19002605.hg.1	2.14	PSR19010221.hg.1	FLT3LG
TC20000139.hg.1	2.38	JUC20000972.hg.1	PLK1S1
TC20000139.hg.1	-2.07	PSR20001851.hg.1	PLK1S1
TC6_mcf_hap5000	2.38	PSR6_mcf_hap5000	PRRC2A
TC6_mcf_hap5000	2.29	PSR6_mcf_hap5000	PRRC2A
TC6_mcf_hap5000	2.18	JUC6_mcf_hap5000	PRRC2A
TC01001568.hg.1	2.37	PSR01024213.hg.1	MR1
TC01001568.hg.1	2.01	JUC01012853.hg.1	MR1
TC01001568.hg.1	-2.22	PSR01024220.hg.1	MR1
TC01001568.hg.1	-2.71	JUC01012850.hg.1	MR1
TC01002342.hg.1	2.37	PSR01036098.hg.1	TCEA3
TC01002342.hg.1	2	PSR01036088.hg.1	TCEA3
TC01002342.hg.1	-2.65	JUC01019453.hg.1	TCEA3
TC01002342.hg.1	-2.75	JUC01019460.hg.1	TCEA3
TC01003452.hg.1	2.37	PSR01053808.hg.1	OLFML2B
TC01003452.hg.1	-2.01	JUC01028042.hg.1	OLFML2B
TC01003452.hg.1	-2.89	PSR01053810.hg.1	OLFML2B
TC01003548.hg.1	2.37	JUC01028698.hg.1	TNR
TC01003548.hg.1	-2.23	PSR01055005.hg.1	TNR
TC01003548.hg.1	-2.48	JUC01028680.hg.1	TNR
TC02001579.hg.1	2.37	JUC02013240.hg.1	E2F6
TC02001579.hg.1	-2.08	JUC02013243.hg.1	E2F6
TC02001579.hg.1	-2.19	PSR02025503.hg.1	E2F6
TC03000759.hg.1	2.37	JUC03006978.hg.1	ACPL2
TC03000759.hg.1	2.21	JUC03006955.hg.1	ACPL2
TC03000759.hg.1	-2.15	PSR03013799.hg.1	ACPL2
TC03000759.hg.1	-2.18	JUC03006969.hg.1	ACPL2
TC05001126.hg.1	2.37	PSR05016312.hg.1	
TC05001126.hg.1	2.37	JUC05008301.hg.1	
TC06001256.hg.1	2.37	JUC06007401.hg.1	SLC35B3
TC06001256.hg.1	-2.15	PSR06015002.hg.1	SLC35B3
TC06001256.hg.1	-3.24	PSR06014999.hg.1	SLC35B3
TC06001807.hg.1	2.37	JUC06010483.hg.1	GSTA4
TC06001807.hg.1	-2.95	PSR06021828.hg.1	GSTA4
TC07001923.hg.1	2.37	PSR07029880.hg.1	MKRN1

TC07001923.hg.1	2.32	JUC07014811.hg.1	MKRN1
TC07001923.hg.1	-2.2	JUC07014796.hg.1	MKRN1
TC07001923.hg.1	-2.46	JUC07014797.hg.1	MKRN1
TC07001923.hg.1	-3.31	JUC07014799.hg.1	MKRN1
TC07001930.hg.1	2.37	JUC07014876.hg.1	KIAA1147
TC07001930.hg.1	2.14	PSR07030003.hg.1	KIAA1147
TC07001930.hg.1	2.04	PSR07029990.hg.1	KIAA1147
TC08000220.hg.1	2.37	JUC08001568.hg.1	FZD3
TC08000220.hg.1	2.35	JUC08001561.hg.1	FZD3
TC08000220.hg.1	2.32	JUC08001569.hg.1	FZD3
TC08000220.hg.1	2.23	PSR08003192.hg.1	FZD3
TC08000220.hg.1	2.06	PSR08003195.hg.1	FZD3
TC08000391.hg.1	2.37	JUC08002735.hg.1	LOC1005076
TC08000391.hg.1	2.34	PSR08005540.hg.1	LOC1005076
TC09000666.hg.1	2.37	JUC09004398.hg.1	LRSAM1
TC09000666.hg.1	2.25	JUC09004365.hg.1	LRSAM1
TC09000666.hg.1	2.06	PSR09007849.hg.1	LRSAM1
TC09000666.hg.1	-2.37	JUC09004372.hg.1	LRSAM1
TC09000666.hg.1	-4.56	JUC09004397.hg.1	LRSAM1
TC09002889.hg.1	2.37	JUC09015700.hg.1	FBXO10
TC09002889.hg.1	-2.15	PSR09014421.hg.1	FBXO10
TC09002889.hg.1	-2.44	PSR09014422.hg.1	FBXO10
TC09002889.hg.1	-4.6	JUC09015698.hg.1	FBXO10
TC0X000636.hg.1	2.37	JUC0X004227.hg.1	HPRT1
TC0X000636.hg.1	2.19	PSR0X008361.hg.1	HPRT1
TC0X001070.hg.1	2.37	JUC0X007342.hg.1	PHF8
TC0X001070.hg.1	2.03	PSR0X014545.hg.1	PHF8
TC0X001070.hg.1	-2.03	JUC0X007341.hg.1	PHF8
TC0X001070.hg.1	-2.26	JUC0X007339.hg.1	PHF8
TC0X001070.hg.1	-2.75	JUC0X007333.hg.1	PHF8
TC11002159.hg.1	2.37	PSR11025805.hg.1	DLG2
TC11002159.hg.1	-2.06	JUC11013680.hg.1	DLG2
TC11002159.hg.1	-2.15	JUC11013689.hg.1	DLG2
TC11002159.hg.1	-3.37	JUC11013686.hg.1	DLG2
TC12000010.hg.1	2.37	JUC12000109.hg.1	WNK1
TC12000010.hg.1	2.28	JUC12000128.hg.1	WNK1
TC12000010.hg.1	2.1	JUC12000116.hg.1	WNK1
TC12000010.hg.1	-2.1	JUC12000120.hg.1	WNK1
TC12000010.hg.1	-2.2	JUC12000110.hg.1	WNK1
TC12000010.hg.1	-2.45	PSR12000142.hg.1	WNK1
TC12000118.hg.1	2.37	PSR12001600.hg.1	FOXJ2
TC12000118.hg.1	2.33	PSR12001599.hg.1	FOXJ2
TC12000118.hg.1	2.07	PSR12001601.hg.1	FOXJ2
TC12000118.hg.1	2.04	PSR12001615.hg.1	FOXJ2
TC12000118.hg.1	2	PSR12001613.hg.1	FOXJ2
TC12000118.hg.1	2	PSR12001614.hg.1	FOXJ2
TC12000118.hg.1	-2.06	JUC12000789.hg.1	FOXJ2
TC12000858.hg.1	2.37	PSR12011224.hg.1	RAD9B
TC12000858.hg.1	2.03	JUC12006210.hg.1	RAD9B
TC12000858.hg.1	-2.12	PSR12011197.hg.1	RAD9B
TC12000858.hg.1	-2.46	PSR12011227.hg.1	RAD9B
TC12001087.hg.1	2.37	PSR12014400.hg.1	RAD52, LOC
TC12001087.hg.1	-2.06	JUC12007905.hg.1	RAD52, LOC
TC12001087.hg.1	-2.15	PSR12014356.hg.1	RAD52, LOC
TC12001087.hg.1	-2.55	PSR12014377.hg.1	RAD52, LOC
TC14001127.hg.1	2.37	PSR14012830.hg.1	TRIM9
TC14001127.hg.1	2.16	PSR14012822.hg.1	TRIM9
TC14001127.hg.1	2.11	JUC14006481.hg.1	TRIM9

TC14001127.hg.1	2.02	PSR14012844.hg.1	TRIM9
TC14001127.hg.1	-4.8	JUC14006479.hg.1	TRIM9
TC16000321.hg.1	2.37	JUC16002559.hg.1	SLX1A, SULT
TC16000321.hg.1	2.19	PSR16004567.hg.1	SLX1A, SULT
TC16000321.hg.1	-2.64	JUC16002566.hg.1	SLX1A, SULT
TC16000343.hg.1	2.37	JUC16002732.hg.1	SLX1A, SULT
TC16000343.hg.1	2.18	PSR16004951.hg.1	SLX1A, SULT
TC16000343.hg.1	-2.64	JUC16002738.hg.1	SLX1A, SULT
TC17000437.hg.1	2.37	PSR17005182.hg.1	PIGW
TC17000437.hg.1	-2.33	JUC17002954.hg.1	PIGW
TC22000049.hg.1	2.37	JUC22000234.hg.1	TBX1
TC22000049.hg.1	2.15	PSR22000501.hg.1	TBX1
TC22000049.hg.1	2.02	JUC22000238.hg.1	TBX1
TC22000049.hg.1	-2.01	PSR22000494.hg.1	TBX1
TC22000049.hg.1	-3.34	JUC22000231.hg.1	TBX1
TC22000049.hg.1	-3.42	PSR22000497.hg.1	TBX1
TC6_cox_hap2000	2.37	PSR6_cox_hap2001(	PRRC2A
TC6_cox_hap2000	2.28	PSR6_cox_hap2001(	PRRC2A
TC6_cox_hap2000	2.17	JUC6_cox_hap20004	PRRC2A
TC6_qbl_hap6000(	2.37	PSR6_qbl_hap60010	PRRC2A
TC6_qbl_hap6000(	2.28	PSR6_qbl_hap60010	PRRC2A
TC6_qbl_hap6000(	2.17	JUC6_qbl_hap60004	PRRC2A
TC6_ssto_hap700(	2.37	PSR6_ssto_hap7000	PRRC2A
TC6_ssto_hap700(	2.28	PSR6_ssto_hap7000	PRRC2A
TC6_ssto_hap700(	2.17	JUC6_ssto_hap7000	PRRC2A
TC21000202.hg.1	2.37	JUC21001126.hg.1	SLC37A1
TC21000202.hg.1	2	PSR21002085.hg.1	SLC37A1
TC03000684.hg.1	2.36	JUC03006090.hg.1	ACAD9
TC03000684.hg.1	2.26	JUC03006086.hg.1	ACAD9
TC03000684.hg.1	-2.01	PSR03012177.hg.1	ACAD9
TC03000690.hg.1	2.36	JUC03006153.hg.1	C3orf37
TC03000690.hg.1	2.28	JUC03006145.hg.1	C3orf37
TC03000690.hg.1	2.18	PSR03012298.hg.1	C3orf37
TC03000690.hg.1	2	JUC03006146.hg.1	C3orf37
TC04000809.hg.1	2.36	PSR04011232.hg.1	RAPGEF2
TC04000809.hg.1	2.14	JUC04006037.hg.1	RAPGEF2
TC04000809.hg.1	2.09	PSR04011218.hg.1	RAPGEF2
TC04000809.hg.1	-2.07	JUC04006016.hg.1	RAPGEF2
TC04000809.hg.1	-3.26	JUC04006030.hg.1	RAPGEF2
TC05002094.hg.1	2.36	JUC05014674.hg.1	RNF44
TC05002094.hg.1	-2.05	PSR05028774.hg.1	RNF44
TC07001871.hg.1	2.36	PSR07028993.hg.1	FLJ43663
TC07001871.hg.1	2.1	PSR07028994.hg.1	FLJ43663
TC07001871.hg.1	2.05	PSR07029000.hg.1	FLJ43663
TC07001871.hg.1	-2.99	JUC07014315.hg.1	FLJ43663
TC08001215.hg.1	2.36	PSR08015693.hg.1	ATP6V1H
TC08001215.hg.1	2.16	JUC08008127.hg.1	ATP6V1H
TC08001215.hg.1	-2.31	JUC08008100.hg.1	ATP6V1H
TC09002929.hg.1	2.36	PSR09000702.hg.1	KDM4C
TC09002929.hg.1	2.09	JUC09016175.hg.1	KDM4C
TC09002929.hg.1	-2.15	JUC09016179.hg.1	KDM4C
TC09002929.hg.1	-2.48	PSR09000732.hg.1	KDM4C
TC09002929.hg.1	-2.65	PSR09000733.hg.1	KDM4C
TC09002929.hg.1	-3.14	JUC09016161.hg.1	KDM4C
TC09002929.hg.1	-3.38	PSR09000738.hg.1	KDM4C
TC09002929.hg.1	-5.82	PSR09000739.hg.1	KDM4C
TC0X001530.hg.1	2.36	JUC0X010195.hg.1	FLNA
TC0X001530.hg.1	2.27	PSR0X020369.hg.1	FLNA

TC0X001530.hg.1	2.22	PSR0X020366.hg.1	FLNA
TC0X001530.hg.1	2.03	PSR0X020438.hg.1	FLNA
TC0X001530.hg.1	2.02	JUC0X010173.hg.1	FLNA
TC12000329.hg.1	2.36	JUC12002264.hg.1	ANO6
TC12000329.hg.1	2.2	PSR12004119.hg.1	ANO6
TC12000329.hg.1	2.05	JUC12002254.hg.1	ANO6
TC12000329.hg.1	-2.35	JUC12002249.hg.1	ANO6
TC13000150.hg.1	2.36	JUC13001077.hg.1	DGKH
TC13000150.hg.1	2.29	PSR13001737.hg.1	DGKH
TC13000150.hg.1	-2.1	JUC13001098.hg.1	DGKH
TC14000799.hg.1	2.36	JUC14004396.hg.1	MARK3
TC14000799.hg.1	-2.19	PSR14008628.hg.1	MARK3
TC14000799.hg.1	-2.31	PSR14008643.hg.1	MARK3
TC14000799.hg.1	-2.51	JUC14004415.hg.1	MARK3
TC14000799.hg.1	-2.93	PSR14008641.hg.1	MARK3
TC15000274.hg.1	2.36	JUC15000800.hg.1	LOC1001310
TC15000274.hg.1	-2.92	PSR15001778.hg.1	LOC1001310
TC17001128.hg.1	2.36	JUC17008670.hg.1	RCVRN
TC17001128.hg.1	2.02	PSR17015194.hg.1	RCVRN
TC17001128.hg.1	-2.57	PSR17015196.hg.1	RCVRN
TC17001128.hg.1	-3.07	PSR17015192.hg.1	RCVRN
TC17001128.hg.1	-4.58	PSR17015198.hg.1	RCVRN
TC21000257.hg.1	2.36	PSR21003129.hg.1	MCM3AP-AS
TC21000257.hg.1	2.11	JUC21001668.hg.1	MCM3AP-AS
TC21000391.hg.1	2.36	PSR21004304.hg.1	SCAF4
TC21000391.hg.1	-2.13	JUC21002300.hg.1	SCAF4
TC22000660.hg.1	2.36	PSR22011674.hg.1	MORC2
TC22000660.hg.1	2.35	JUC22004816.hg.1	MORC2
TC01000514.hg.1	2.35	JUC01004188.hg.1	ZNF684
TC01000514.hg.1	-2.3	PSR01008037.hg.1	ZNF684
TC01002050.hg.1	2.35	PSR01031213.hg.1	WASH7P
TC01002050.hg.1	2.1	JUC01016961.hg.1	WASH7P
TC01002050.hg.1	2.08	JUC01016946.hg.1	WASH7P
TC01002050.hg.1	-2.06	JUC01016959.hg.1	WASH7P
TC02002506.hg.1	2.35	PSR02039664.hg.1	LOC1008614
TC02002506.hg.1	-2.3	JUC02020852.hg.1	LOC1008614
TC02002688.hg.1	2.35	JUC02022746.hg.1	RAPH1
TC02002688.hg.1	2.01	PSR02043409.hg.1	RAPH1
TC02002688.hg.1	-2.26	JUC02022747.hg.1	RAPH1
TC03003340.hg.1	2.35	PSR03028903.hg.1	CPOX
TC03003340.hg.1	-2.12	JUC03023442.hg.1	CPOX
TC04000281.hg.1	2.35	JUC04002032.hg.1	OCIAD1
TC04000281.hg.1	2.3	PSR04004086.hg.1	OCIAD1
TC04000281.hg.1	-2.05	JUC04002033.hg.1	OCIAD1
TC04000281.hg.1	-2.66	JUC04002047.hg.1	OCIAD1
TC04000648.hg.1	2.35	JUC04005032.hg.1	LARP1B
TC04000648.hg.1	2.3	PSR04009428.hg.1	LARP1B
TC04000648.hg.1	2.3	JUC04005029.hg.1	LARP1B
TC04000648.hg.1	-2	JUC04005026.hg.1	LARP1B
TC04000648.hg.1	-2	JUC04005043.hg.1	LARP1B
TC04000648.hg.1	-2.48	JUC04005036.hg.1	LARP1B
TC05000464.hg.1	2.35	JUC05003621.hg.1	
TC05000464.hg.1	-2.37	JUC05003623.hg.1	
TC05000464.hg.1	-2.39	PSR05006593.hg.1	
TC05000464.hg.1	-2.59	PSR05006594.hg.1	
TC05000464.hg.1	-2.69	JUC05003622.hg.1	
TC05000464.hg.1	-3.22	PSR05006595.hg.1	
TC05000961.hg.1	2.35	PSR05013975.hg.1	

TC05000961.hg.1	-2.06	PSR05013973.hg.1	
TC05000961.hg.1	-2.14	PSR05013974.hg.1	
TC05000961.hg.1	-2.24	PSR05013971.hg.1	
TC05000961.hg.1	-2.85	PSR05013972.hg.1	
TC05000961.hg.1	-3.78	JUC05007244.hg.1	
TC05000961.hg.1	-3.95	PSR05013976.hg.1	
TC07000245.hg.1	2.35	JUC07001788.hg.1	STARD3NL
TC07000245.hg.1	-2.39	PSR07003764.hg.1	STARD3NL
TC07001852.hg.1	2.35	PSR07028731.hg.1	TNPO3
TC07001852.hg.1	-2.35	JUC07014205.hg.1	TNPO3
TC08001524.hg.1	2.35	JUC08010196.hg.1	EIF3E
TC08001524.hg.1	-2.09	PSR08020006.hg.1	EIF3E
TC08001524.hg.1	-2.11	JUC08010170.hg.1	EIF3E
TC08001524.hg.1	-2.26	JUC08010197.hg.1	EIF3E
TC08001524.hg.1	-2.27	PSR08020011.hg.1	EIF3E
TC08001524.hg.1	-3.61	JUC08010195.hg.1	EIF3E
TC0X000824.hg.1	2.35	PSR0X011220.hg.1	VCX3A, VCX
TC0X000824.hg.1	2.35	JUC0X005490.hg.1	VCX3A, VCX
TC10000659.hg.1	2.35	PSR10007368.hg.1	KIF11
TC10000659.hg.1	2.14	PSR10007376.hg.1	KIF11
TC10000659.hg.1	-2.05	JUC10004200.hg.1	KIF11
TC10000659.hg.1	-2.11	PSR10007378.hg.1	KIF11
TC10000659.hg.1	-2.25	JUC10004188.hg.1	KIF11
TC10001225.hg.1	2.35	JUC10008834.hg.1	ZFAND4
TC10001225.hg.1	2.17	PSR10015272.hg.1	ZFAND4
TC10001225.hg.1	2.08	JUC10008830.hg.1	ZFAND4
TC10001225.hg.1	2.07	PSR10015299.hg.1	ZFAND4
TC10001225.hg.1	2.05	JUC10008849.hg.1	ZFAND4
TC12001080.hg.1	2.35	JUC12007834.hg.1	SLC6A13
TC12001080.hg.1	2.19	PSR12014282.hg.1	SLC6A13
TC12003242.hg.1	2.35	PSR12005755.hg.1	PCBP2
TC12003242.hg.1	-2.19	JUC12019932.hg.1	PCBP2
TC19001095.hg.1	2.35	JUC19008495.hg.1	KHSRP, MIR
TC19001095.hg.1	2.02	PSR19015015.hg.1	KHSRP, MIR
TC19001480.hg.1	2.35	JUC19011564.hg.1	ZNF585B
TC19001480.hg.1	2.26	PSR19020105.hg.1	ZNF585B
TC22000492.hg.1	2.35	JUC22003568.hg.1	DGCR14, TS
TC22000492.hg.1	2.14	PSR22008825.hg.1	DGCR14, TS
TC22000912.hg.1	2.35	PSR22015494.hg.1	FAM116B
TC22000912.hg.1	2.04	JUC22006521.hg.1	FAM116B
TC22000912.hg.1	-2.24	JUC22006527.hg.1	FAM116B
TC08002578.hg.1	2.35	JUC08014646.hg.1	LEPROTL1
TC08002578.hg.1	-2	PSR08003366.hg.1	LEPROTL1
TC01000113.hg.1	2.34	JUC01000913.hg.1	H6PD
TC01000113.hg.1	2.11	PSR01001733.hg.1	H6PD
TC01000831.hg.1	2.34	JUC01006958.hg.1	PKN2
TC01000831.hg.1	2.05	PSR01013255.hg.1	PKN2
TC02001178.hg.1	2.34	JUC02009581.hg.1	STRADB
TC02001178.hg.1	2.33	PSR02017992.hg.1	STRADB
TC02001178.hg.1	2.26	PSR02017955.hg.1	STRADB
TC02001178.hg.1	2.14	PSR02017958.hg.1	STRADB
TC03000664.hg.1	2.34	JUC03005959.hg.1	MCM2
TC03000664.hg.1	2.17	JUC03005968.hg.1	MCM2
TC03000664.hg.1	-2.07	PSR03011917.hg.1	MCM2
TC03000664.hg.1	-2.13	JUC03005972.hg.1	MCM2
TC03000664.hg.1	-2.8	JUC03005973.hg.1	MCM2
TC03000664.hg.1	-3.41	JUC03005967.hg.1	MCM2
TC03000664.hg.1	-4.58	JUC03005960.hg.1	MCM2

TC03001528.hg.1	2.34	JUC03013661.hg.1	MAGI1, MAG
TC03001528.hg.1	2.25	JUC03013654.hg.1	MAGI1, MAG
TC03001528.hg.1	2.24	PSR03027871.hg.1	MAGI1, MAG
TC03001528.hg.1	2	PSR03027815.hg.1	MAGI1, MAG
TC03001528.hg.1	2	PSR03027818.hg.1	MAGI1, MAG
TC04001279.hg.1	2.34	JUC04009362.hg.1	ANKRD17
TC04001279.hg.1	2.13	JUC04009340.hg.1	ANKRD17
TC04001279.hg.1	2.09	PSR04017496.hg.1	ANKRD17
TC04001279.hg.1	-2.43	JUC04009359.hg.1	ANKRD17
TC04001450.hg.1	2.34	JUC04010708.hg.1	TBCK
TC04001450.hg.1	2.1	JUC04010737.hg.1	TBCK
TC04001450.hg.1	-2.06	PSR04020485.hg.1	TBCK
TC04001450.hg.1	-2.29	JUC04010705.hg.1	TBCK
TC04001450.hg.1	-2.36	PSR04020478.hg.1	TBCK
TC04001450.hg.1	-2.5	JUC04010745.hg.1	TBCK
TC04001450.hg.1	-2.62	JUC04010734.hg.1	TBCK
TC04001450.hg.1	-3.6	PSR04020504.hg.1	TBCK
TC06001966.hg.1	2.34	PSR06024308.hg.1	CCNC
TC06001966.hg.1	-2	JUC06011913.hg.1	CCNC
TC07001139.hg.1	2.34	JUC07008636.hg.1	RPA3
TC07001139.hg.1	2.32	JUC07008633.hg.1	RPA3
TC07001139.hg.1	2.13	PSR07017795.hg.1	RPA3
TC07001139.hg.1	-2.08	PSR07017786.hg.1	RPA3
TC07001139.hg.1	-2.44	JUC07008638.hg.1	RPA3
TC07001562.hg.1	2.34	JUC07011661.hg.1	HGF
TC07001562.hg.1	-2.16	PSR07023472.hg.1	HGF
TC07001562.hg.1	-2.22	PSR07023474.hg.1	HGF
TC07001562.hg.1	-2.27	PSR07023470.hg.1	HGF
TC07001562.hg.1	-2.28	PSR07023475.hg.1	HGF
TC07001562.hg.1	-2.53	PSR07023501.hg.1	HGF
TC07001562.hg.1	-2.66	PSR07023487.hg.1	HGF
TC07001562.hg.1	-2.97	PSR07023495.hg.1	HGF
TC07001562.hg.1	-3.04	PSR07023479.hg.1	HGF
TC07001562.hg.1	-3.15	JUC07011672.hg.1	HGF
TC07001562.hg.1	-3.19	JUC07011662.hg.1	HGF
TC07001562.hg.1	-3.54	PSR07023480.hg.1	HGF
TC07001562.hg.1	-4.31	PSR07023468.hg.1	HGF
TC07001562.hg.1	-4.52	JUC07011673.hg.1	HGF
TC07001562.hg.1	-4.97	JUC07011675.hg.1	HGF
TC07001562.hg.1	-5.07	JUC07011658.hg.1	HGF
TC07001562.hg.1	-5.71	JUC07011663.hg.1	HGF
TC07001562.hg.1	-6.07	PSR07023478.hg.1	HGF
TC07001562.hg.1	-6.63	PSR07023490.hg.1	HGF
TC07001562.hg.1	-6.74	PSR07023496.hg.1	HGF
TC07001562.hg.1	-6.93	JUC07011671.hg.1	HGF
TC07001562.hg.1	-8	PSR07023483.hg.1	HGF
TC07001562.hg.1	-8.5	PSR07023511.hg.1	HGF
TC07001562.hg.1	-9.08	PSR07023503.hg.1	HGF
TC07001562.hg.1	-9.45	PSR07023494.hg.1	HGF
TC07001562.hg.1	-10.27	PSR07023491.hg.1	HGF
TC07001562.hg.1	-11.24	PSR07023477.hg.1	HGF
TC07001562.hg.1	-11.92	JUC07011667.hg.1	HGF
TC07001562.hg.1	-13.13	PSR07023516.hg.1	HGF
TC07001562.hg.1	-13.52	PSR07023504.hg.1	HGF
TC07001562.hg.1	-14.45	PSR07023515.hg.1	HGF
TC07001562.hg.1	-16.29	JUC07011670.hg.1	HGF
TC07001562.hg.1	-18.24	PSR07023514.hg.1	HGF
TC08000881.hg.1	2.34	JUC08006025.hg.1	ERICH1

TC08000881.hg.1	-2.16	PSR08011630.hg.1	ERICH1
TC08000881.hg.1	-2.29	JUC08006030.hg.1	ERICH1
TC08000881.hg.1	-2.31	JUC08006026.hg.1	ERICH1
TC08000881.hg.1	-3.2	JUC08006027.hg.1	ERICH1
TC08001713.hg.1	2.34	JUC08011587.hg.1	TOP1MT
TC08001713.hg.1	2.04	PSR08022590.hg.1	TOP1MT
TC08001713.hg.1	2.04	JUC08011616.hg.1	TOP1MT
TC08001713.hg.1	2.01	JUC08011600.hg.1	TOP1MT
TC11001172.hg.1	2.34	PSR11014823.hg.1	TIRAP
TC11001172.hg.1	-2.05	JUC11008017.hg.1	TIRAP
TC11001172.hg.1	-2.35	JUC11008012.hg.1	TIRAP
TC12000266.hg.1	2.34	PSR12003230.hg.1	PPFIBP1
TC12000266.hg.1	2.11	PSR12003284.hg.1	PPFIBP1
TC12000266.hg.1	-2.03	JUC12001622.hg.1	PPFIBP1
TC12000266.hg.1	-2.05	JUC12001629.hg.1	PPFIBP1
TC12000266.hg.1	-2.07	PSR12003265.hg.1	PPFIBP1
TC12000266.hg.1	-2.21	JUC12001614.hg.1	PPFIBP1
TC12000266.hg.1	-2.8	JUC12001608.hg.1	PPFIBP1
TC14000135.hg.1	2.34	JUC14000502.hg.1	LRP10
TC14000135.hg.1	2.05	PSR14001158.hg.1	LRP10
TC15000635.hg.1	2.34	JUC15002683.hg.1	PAQR5
TC15000635.hg.1	2.11	JUC15002682.hg.1	PAQR5
TC15000635.hg.1	2.1	JUC15002693.hg.1	PAQR5
TC15000635.hg.1	2.02	PSR15005375.hg.1	PAQR5
TC15000635.hg.1	2.01	JUC15002690.hg.1	PAQR5
TC15000635.hg.1	-4.45	JUC15002689.hg.1	PAQR5
TC15000712.hg.1	2.34	JUC15003250.hg.1	HMG20A
TC15000712.hg.1	-2.18	PSR15006389.hg.1	HMG20A
TC15001606.hg.1	2.34	JUC15008416.hg.1	TLE3
TC15001606.hg.1	2.17	JUC15008405.hg.1	TLE3
TC15001606.hg.1	2.13	PSR15015494.hg.1	TLE3
TC15001606.hg.1	-2.25	JUC15008410.hg.1	TLE3
TC17000354.hg.1	2.34	JUC17002495.hg.1	SUZ12P1
TC17000354.hg.1	-2.02	PSR17004424.hg.1	SUZ12P1
TC17000354.hg.1	-2.24	JUC17002501.hg.1	SUZ12P1
TC17000354.hg.1	-2.31	PSR17004425.hg.1	SUZ12P1
TC17002914.hg.1	2.34	JUC17019184.hg.1	LOC1005072
TC17002914.hg.1	2.22	PSR17003585.hg.1	LOC1005072
TC17002914.hg.1	2.2	PSR17003587.hg.1	LOC1005072
TC17002914.hg.1	2.19	PSR17003588.hg.1	LOC1005072
TC18000011.hg.1	2.34	JUC18000076.hg.1	SMCHD1
TC18000011.hg.1	-2.57	JUC18000099.hg.1	SMCHD1
TC18000011.hg.1	-2.58	PSR18000113.hg.1	SMCHD1
TC19000657.hg.1	2.34	JUC19005457.hg.1	CCDC61
TC19000657.hg.1	-2.19	PSR19009183.hg.1	CCDC61
TC19000657.hg.1	-2.32	JUC19005454.hg.1	CCDC61
TC20000816.hg.1	2.34	PSR20011204.hg.1	NDRG3
TC20000816.hg.1	2.11	JUC20005762.hg.1	NDRG3
TC6_ssto_hap700(	2.34	PSR6_ssto_hap7000	HLA-A
TC6_ssto_hap700(	-2.37	JUC6_ssto_hap7000	HLA-A
TC6_ssto_hap700(	-5.94	JUC6_ssto_hap7000	HLA-A
TC01002363.hg.1	2.33	JUC01019678.hg.1	C1orf201
TC01002363.hg.1	2.16	PSR01036525.hg.1	C1orf201
TC01002363.hg.1	2.14	PSR01036517.hg.1	C1orf201
TC01002363.hg.1	2.03	PSR01036483.hg.1	C1orf201
TC01002363.hg.1	-2.11	JUC01019667.hg.1	C1orf201
TC03001378.hg.1	2.33	PSR03023884.hg.1	PLXNB1
TC03001378.hg.1	2.22	JUC03011896.hg.1	PLXNB1

TC04000971.hg.1	2.33	JUC04007067.hg.1	MIR943, WHS
TC04000971.hg.1	2.13	PSR04013312.hg.1	MIR943, WHS
TC04001139.hg.1	2.33	JUC04008315.hg.1	APBB2
TC04001139.hg.1	2.18	JUC04008327.hg.1	APBB2
TC04001139.hg.1	-2	PSR04015665.hg.1	APBB2
TC04001139.hg.1	-2.08	PSR04015681.hg.1	APBB2
TC04001139.hg.1	-2.09	PSR04015723.hg.1	APBB2
TC04001139.hg.1	-2.15	PSR04015731.hg.1	APBB2
TC04001139.hg.1	-2.34	PSR04015679.hg.1	APBB2
TC04001139.hg.1	-2.45	PSR04015687.hg.1	APBB2
TC04001139.hg.1	-2.48	PSR04015717.hg.1	APBB2
TC04001139.hg.1	-2.86	PSR04015718.hg.1	APBB2
TC04001139.hg.1	-2.9	PSR04015688.hg.1	APBB2
TC04001139.hg.1	-3.28	JUC04008317.hg.1	APBB2
TC05000168.hg.1	2.33	JUC05001116.hg.1	WDR70
TC05000168.hg.1	2.2	PSR05001970.hg.1	WDR70
TC05003430.hg.1	2.33	PSR05025287.hg.1	DNAJC18
TC05003430.hg.1	2.14	JUC05019687.hg.1	DNAJC18
TC05003430.hg.1	2.07	JUC05019693.hg.1	DNAJC18
TC08000768.hg.1	2.33	JUC08005252.hg.1	PHF20L1
TC08000768.hg.1	-2	PSR08010228.hg.1	PHF20L1
TC08000768.hg.1	-2.08	PSR08010227.hg.1	PHF20L1
TC08000768.hg.1	-2.1	PSR08010216.hg.1	PHF20L1
TC08000768.hg.1	-2.21	PSR08010239.hg.1	PHF20L1
TC08000768.hg.1	-2.31	PSR08010231.hg.1	PHF20L1
TC08000768.hg.1	-2.33	PSR08010248.hg.1	PHF20L1
TC08000768.hg.1	-2.46	JUC08005246.hg.1	PHF20L1
TC08000768.hg.1	-2.64	JUC08005244.hg.1	PHF20L1
TC08000768.hg.1	-2.78	PSR08010208.hg.1	PHF20L1
TC08000768.hg.1	-2.84	PSR08010257.hg.1	PHF20L1
TC08000768.hg.1	-3.01	PSR08010220.hg.1	PHF20L1
TC08000768.hg.1	-3.56	JUC08005262.hg.1	PHF20L1
TC08001147.hg.1	2.33	JUC08007509.hg.1	FGFR1
TC08001147.hg.1	2.23	JUC08007541.hg.1	FGFR1
TC08001147.hg.1	2.1	PSR08014802.hg.1	FGFR1
TC08001147.hg.1	-2.18	JUC08007534.hg.1	FGFR1
TC08001147.hg.1	-3.42	JUC08007515.hg.1	FGFR1
TC09001773.hg.1	2.33	JUC09012317.hg.1	PNPLA7
TC09001773.hg.1	-2.02	PSR09023032.hg.1	PNPLA7
TC09001773.hg.1	-2.02	JUC09012304.hg.1	PNPLA7
TC09001773.hg.1	-2.1	PSR09023030.hg.1	PNPLA7
TC09001773.hg.1	-2.14	PSR09023027.hg.1	PNPLA7
TC09001773.hg.1	-2.28	PSR09023013.hg.1	PNPLA7
TC09001773.hg.1	-2.32	PSR09023008.hg.1	PNPLA7
TC09001773.hg.1	-2.74	JUC09012312.hg.1	PNPLA7
TC09001773.hg.1	-3.45	PSR09023028.hg.1	PNPLA7
TC0X000901.hg.1	2.33	PSR0X012072.hg.1	GPR64
TC0X000901.hg.1	2.15	PSR0X012060.hg.1	GPR64
TC0X000901.hg.1	-2.01	JUC0X005983.hg.1	GPR64
TC0X000901.hg.1	-2.26	JUC0X005952.hg.1	GPR64
TC0X000901.hg.1	-2.5	JUC0X005950.hg.1	GPR64
TC0X000901.hg.1	-2.59	PSR0X012077.hg.1	GPR64
TC0X000901.hg.1	-2.62	PSR0X012093.hg.1	GPR64
TC0X000901.hg.1	-2.79	PSR0X012084.hg.1	GPR64
TC0X000901.hg.1	-3.73	JUC0X005946.hg.1	GPR64
TC0X000901.hg.1	-4.42	JUC0X005949.hg.1	GPR64
TC12000656.hg.1	2.33	JUC12004567.hg.1	NAV3
TC12000656.hg.1	2.01	JUC12004589.hg.1	NAV3

TC12000656.hg.1	-2.01	JUC12004591.hg.1	NAV3
TC12000656.hg.1	-2.13	JUC12004592.hg.1	NAV3
TC12000656.hg.1	-2.21	PSR12008598.hg.1	NAV3
TC12000656.hg.1	-2.26	PSR12008622.hg.1	NAV3
TC12000656.hg.1	-2.3	JUC12004594.hg.1	NAV3
TC12000656.hg.1	-2.31	PSR12008608.hg.1	NAV3
TC12000656.hg.1	-2.46	JUC12004550.hg.1	NAV3
TC12000656.hg.1	-2.47	PSR12008590.hg.1	NAV3
TC12000656.hg.1	-2.69	JUC12004569.hg.1	NAV3
TC12000656.hg.1	-3.02	PSR12008589.hg.1	NAV3
TC12000656.hg.1	-6.01	JUC12004575.hg.1	NAV3
TC12000989.hg.1	2.33	PSR12013101.hg.1	DDX55
TC12000989.hg.1	-3.26	JUC12007146.hg.1	DDX55
TC13000799.hg.1	2.33	JUC13005256.hg.1	ABCC4
TC13000799.hg.1	2.11	JUC13005231.hg.1	ABCC4
TC13000799.hg.1	-2.4	PSR13008938.hg.1	ABCC4
TC13000799.hg.1	-2.4	JUC13005262.hg.1	ABCC4
TC13000799.hg.1	-2.4	JUC13005269.hg.1	ABCC4
TC13000799.hg.1	-2.47	PSR13008936.hg.1	ABCC4
TC13000799.hg.1	-2.73	PSR13008973.hg.1	ABCC4
TC14002332.hg.1	2.33	PSR14015849.hg.1	EFCAB11
TC14002332.hg.1	-2.04	JUC14012197.hg.1	EFCAB11
TC14002332.hg.1	-2.2	PSR14015840.hg.1	EFCAB11
TC14002332.hg.1	-2.43	JUC14012207.hg.1	EFCAB11
TC14002332.hg.1	-2.68	JUC14012206.hg.1	EFCAB11
TC14002332.hg.1	-4.82	PSR14015836.hg.1	EFCAB11
TC16001125.hg.1	2.33	JUC16008782.hg.1	CRNDE
TC16001125.hg.1	-2.28	PSR16015655.hg.1	CRNDE
TC16001267.hg.1	2.33	PSR16017657.hg.1	GLG1
TC16001267.hg.1	2.31	JUC16010033.hg.1	GLG1
TC16001267.hg.1	-2.58	JUC16010043.hg.1	GLG1
TC16001267.hg.1	-3.6	JUC16010017.hg.1	GLG1
TC17001093.hg.1	2.33	PSR17014691.hg.1	SAT2
TC17001093.hg.1	2.12	JUC17008383.hg.1	SAT2
TC19000029.hg.1	2.33	PSR19000319.hg.1	HMHA1
TC19000029.hg.1	-2.09	JUC19000213.hg.1	HMHA1
TC19000029.hg.1	-2.64	JUC19000214.hg.1	HMHA1
TC01000358.hg.1	2.32	JUC01002897.hg.1	WDTC1
TC01000358.hg.1	2.13	PSR01005558.hg.1	WDTC1
TC02002504.hg.1	2.32	PSR02039661.hg.1	STK39
TC02002504.hg.1	-2.54	JUC02020843.hg.1	STK39
TC03001536.hg.1	2.32	PSR03028030.hg.1	TMF1
TC03001536.hg.1	2.24	JUC03013767.hg.1	TMF1
TC03001536.hg.1	2.02	JUC03013769.hg.1	TMF1
TC03001536.hg.1	-2.09	JUC03013773.hg.1	TMF1
TC08000190.hg.1	2.32	JUC08001281.hg.1	ADAM28
TC08000190.hg.1	-2.04	JUC08001284.hg.1	ADAM28
TC08000190.hg.1	-2.12	PSR08002512.hg.1	ADAM28
TC08000190.hg.1	-2.31	JUC08001273.hg.1	ADAM28
TC09001326.hg.1	2.32	JUC09008880.hg.1	ROR2
TC09001326.hg.1	2.07	PSR09016511.hg.1	ROR2
TC09001326.hg.1	-2.05	PSR09016521.hg.1	ROR2
TC09001326.hg.1	-2.31	PSR09016526.hg.1	ROR2
TC09001326.hg.1	-2.35	JUC09008875.hg.1	ROR2
TC09001326.hg.1	-2.36	PSR09016509.hg.1	ROR2
TC09001326.hg.1	-2.39	PSR09016525.hg.1	ROR2
TC09001326.hg.1	-2.72	PSR09016497.hg.1	ROR2
TC09001326.hg.1	-2.88	JUC09008878.hg.1	ROR2

TC09001326.hg.1	-2.9	JUC09008871.hg.1	ROR2
TC09001326.hg.1	-3.51	PSR09016495.hg.1	ROR2
TC09001326.hg.1	-3.66	PSR09016504.hg.1	ROR2
TC09001326.hg.1	-3.75	JUC09008867.hg.1	ROR2
TC09001326.hg.1	-3.94	JUC09008877.hg.1	ROR2
TC09001326.hg.1	-4.01	PSR09016522.hg.1	ROR2
TC09001326.hg.1	-4.11	PSR09016498.hg.1	ROR2
TC09001326.hg.1	-4.25	PSR09016523.hg.1	ROR2
TC09001326.hg.1	-4.52	PSR09016496.hg.1	ROR2
TC0X001244.hg.1	2.32	JUC0X008586.hg.1	RAB9B
TC0X001244.hg.1	2.11	PSR0X016864.hg.1	RAB9B
TC0Y000072.hg.1	2.32	PSR0Y000828.hg.1	EIF1AY
TC0Y000072.hg.1	-2.01	PSR0Y000825.hg.1	EIF1AY
TC0Y000072.hg.1	-2.81	PSR0Y000832.hg.1	EIF1AY
TC0Y000072.hg.1	-3.1	PSR0Y000830.hg.1	EIF1AY
TC0Y000072.hg.1	-3.18	PSR0Y000826.hg.1	EIF1AY
TC0Y000072.hg.1	-3.86	PSR0Y000834.hg.1	EIF1AY
TC0Y000072.hg.1	-5.26	JUC0Y000364.hg.1	EIF1AY
TC0Y000072.hg.1	-5.64	PSR0Y000831.hg.1	EIF1AY
TC0Y000072.hg.1	-9.15	JUC0Y000362.hg.1	EIF1AY
TC10000309.hg.1	2.32	JUC10002016.hg.1	ANXA8L2, LC
TC10000309.hg.1	2.13	JUC10002018.hg.1	ANXA8L2, LC
TC10000309.hg.1	-2.03	JUC10002011.hg.1	ANXA8L2, LC
TC10000309.hg.1	-2.1	JUC10002002.hg.1	ANXA8L2, LC
TC10000309.hg.1	-2.18	JUC10002015.hg.1	ANXA8L2, LC
TC10000309.hg.1	-2.81	JUC10002010.hg.1	ANXA8L2, LC
TC10000309.hg.1	-2.92	PSR10003481.hg.1	ANXA8L2, LC
TC10000309.hg.1	-4.1	PSR10003472.hg.1	ANXA8L2, LC
TC10000309.hg.1	-4.17	PSR10003502.hg.1	ANXA8L2, LC
TC10000309.hg.1	-6.16	JUC10002020.hg.1	ANXA8L2, LC
TC10002963.hg.1	2.32	JUC10017529.hg.1	ANXA8L1, AN
TC10002963.hg.1	2.13	JUC10017518.hg.1	ANXA8L1, AN
TC10002963.hg.1	-2.04	JUC10017524.hg.1	ANXA8L1, AN
TC10002963.hg.1	-2.1	JUC10017519.hg.1	ANXA8L1, AN
TC10002963.hg.1	-2.14	PSR10015467.hg.1	ANXA8L1, AN
TC10002963.hg.1	-2.18	JUC10017528.hg.1	ANXA8L1, AN
TC10002963.hg.1	-2.69	PSR10015469.hg.1	ANXA8L1, AN
TC10002963.hg.1	-5.85	JUC10017517.hg.1	ANXA8L1, AN
TC11000052.hg.1	2.32	JUC11000483.hg.1	LSP1
TC11000052.hg.1	2.3	PSR11000991.hg.1	LSP1
TC11000052.hg.1	2.15	JUC11000493.hg.1	LSP1
TC11000876.hg.1	2.32	PSR11011093.hg.1	PRSS23
TC11000876.hg.1	-2	JUC11005813.hg.1	PRSS23
TC11000876.hg.1	-8.35	JUC11005816.hg.1	PRSS23
TC12001605.hg.1	2.32	JUC12011863.hg.1	TIMELESS
TC12001605.hg.1	-2.04	JUC12011869.hg.1	TIMELESS
TC12001605.hg.1	-2.2	PSR12021422.hg.1	TIMELESS
TC12001605.hg.1	-2.25	JUC12011858.hg.1	TIMELESS
TC12001605.hg.1	-2.8	JUC12011872.hg.1	TIMELESS
TC12001605.hg.1	-3.32	JUC12011859.hg.1	TIMELESS
TC15001594.hg.1	2.32	JUC15008386.hg.1	ANP32A
TC15001594.hg.1	-2.75	JUC15008383.hg.1	ANP32A
TC15001594.hg.1	-2.85	PSR15015417.hg.1	ANP32A
TC17001613.hg.1	2.32	JUC17012181.hg.1	ARL17A, ARL
TC17001613.hg.1	2.25	JUC17012182.hg.1	ARL17A, ARL
TC17001613.hg.1	-2.03	PSR17021668.hg.1	ARL17A, ARL
TC19001801.hg.1	2.32	PSR19024434.hg.1	
TC19001801.hg.1	2.01	JUC19013892.hg.1	

TC21000040.hg.1	2.32	JUC21000120.hg.1	LINC00478
TC21000040.hg.1	-2.28	JUC21000122.hg.1	LINC00478
TC21000040.hg.1	-2.43	PSR21000175.hg.1	LINC00478
TC21000040.hg.1	-2.82	JUC21000112.hg.1	LINC00478
TC21000040.hg.1	-4.72	JUC21000116.hg.1	LINC00478
TC01000566.hg.1	2.32	JUC01004892.hg.1	PLK3
TC01000566.hg.1	-2	PSR01009492.hg.1	PLK3
TC0X001369.hg.1	2.32	JUC0X009420.hg.1	RAP2C
TC0X001369.hg.1	2.2	JUC0X009412.hg.1	RAP2C
TC0X001369.hg.1	2	PSR0X018395.hg.1	RAP2C
TC02002612.hg.1	2.31	JUC02021768.hg.1	COL5A2
TC02002612.hg.1	2.23	JUC02021796.hg.1	COL5A2
TC02002612.hg.1	2.02	JUC02021812.hg.1	COL5A2
TC02002612.hg.1	-2.01	PSR02041826.hg.1	COL5A2
TC02002612.hg.1	-3.02	JUC02021776.hg.1	COL5A2
TC02002625.hg.1	2.31	JUC02021966.hg.1	STAT4
TC02002625.hg.1	2.16	JUC02021945.hg.1	STAT4
TC02002625.hg.1	-2.13	PSR02042111.hg.1	STAT4
TC02002625.hg.1	-2.17	JUC02021963.hg.1	STAT4
TC02002625.hg.1	-2.9	JUC02021957.hg.1	STAT4
TC02002625.hg.1	-5.67	JUC02021964.hg.1	STAT4
TC03001418.hg.1	2.31	PSR03025321.hg.1	UBA7
TC03001418.hg.1	2.23	JUC03012480.hg.1	UBA7
TC03001418.hg.1	2.2	JUC03012462.hg.1	UBA7
TC03001418.hg.1	2.11	PSR03025342.hg.1	UBA7
TC03001418.hg.1	2.06	PSR03025320.hg.1	UBA7
TC03001418.hg.1	2	PSR03025331.hg.1	UBA7
TC04000808.hg.1	2.31	JUC04005998.hg.1	FNIP2
TC04000808.hg.1	-2	PSR04011186.hg.1	FNIP2
TC04000808.hg.1	-2.02	PSR04011184.hg.1	FNIP2
TC04000808.hg.1	-2.07	PSR04011196.hg.1	FNIP2
TC04000808.hg.1	-2.66	PSR04011185.hg.1	FNIP2
TC04000808.hg.1	-3.66	JUC04005987.hg.1	FNIP2
TC04001090.hg.1	2.31	JUC04008005.hg.1	SEPSECS
TC04001090.hg.1	2.05	PSR04015017.hg.1	SEPSECS
TC04001090.hg.1	-2.01	PSR04015022.hg.1	SEPSECS
TC04001090.hg.1	-2.85	JUC04007999.hg.1	SEPSECS
TC06000136.hg.1	2.31	JUC06000678.hg.1	LINC00340
TC06000136.hg.1	-2.17	PSR06001377.hg.1	LINC00340
TC06000136.hg.1	-2.3	JUC06000662.hg.1	LINC00340
TC06000136.hg.1	-2.52	PSR06001381.hg.1	LINC00340
TC06000136.hg.1	-3.05	JUC06000675.hg.1	LINC00340
TC06000136.hg.1	-4.17	JUC06000679.hg.1	LINC00340
TC06000136.hg.1	-4.38	JUC06000658.hg.1	LINC00340
TC06000149.hg.1	2.31	JUC06000738.hg.1	GMNN
TC06000149.hg.1	2.19	PSR06001503.hg.1	GMNN
TC06000149.hg.1	-2.37	PSR06001510.hg.1	GMNN
TC06001124.hg.1	2.31	PSR06013346.hg.1	ZDHHC14
TC06001124.hg.1	2.3	JUC06006553.hg.1	ZDHHC14
TC06001124.hg.1	-2.11	PSR06013364.hg.1	ZDHHC14
TC06001124.hg.1	-2.21	PSR06013359.hg.1	ZDHHC14
TC06001124.hg.1	-2.25	JUC06006549.hg.1	ZDHHC14
TC06001124.hg.1	-2.68	PSR06013356.hg.1	ZDHHC14
TC06001124.hg.1	-2.82	JUC06006555.hg.1	ZDHHC14
TC06004123.hg.1	2.31	JUC06021721.hg.1	HLA-DRB1, L
TC06004123.hg.1	-2.05	JUC06021726.hg.1	HLA-DRB1, L
TC06004123.hg.1	-2.24	JUC06021723.hg.1	HLA-DRB1, L
TC06004123.hg.1	-2.4	JUC06021722.hg.1	HLA-DRB1, L

TC06004123.hg.1	-2.57	JUC06021716.hg.1	HLA-DRB1, L
TC06004123.hg.1	-3.67	JUC06021725.hg.1	HLA-DRB1, L
TC06004123.hg.1	-4.03	PSR06018671.hg.1	HLA-DRB1, L
TC07003323.hg.1	2.31	JUC07020873.hg.1	EPDR1
TC07003323.hg.1	-2.71	PSR07003738.hg.1	EPDR1
TC07003323.hg.1	-3.04	JUC07020872.hg.1	EPDR1
TC08001638.hg.1	2.31	PSR08021288.hg.1	FAM49B
TC08001638.hg.1	-2.01	JUC08010829.hg.1	FAM49B
TC08001638.hg.1	-2.02	PSR08021255.hg.1	FAM49B
TC0X001155.hg.1	2.31	PSR0X015614.hg.1	XIST
TC0X001155.hg.1	2.15	JUC0X007890.hg.1	XIST
TC10001230.hg.1	2.31	PSR10015359.hg.1	PTPN20B, PT
TC10001230.hg.1	2.11	JUC10008903.hg.1	PTPN20B, PT
TC10001230.hg.1	-2.31	JUC10008885.hg.1	PTPN20B, PT
TC13000103.hg.1	2.31	PSR13000974.hg.1	C13orf33
TC13000103.hg.1	2.23	PSR13000982.hg.1	C13orf33
TC13000103.hg.1	2.21	PSR13000975.hg.1	C13orf33
TC13000103.hg.1	-2.16	JUC13000568.hg.1	C13orf33
TC13000103.hg.1	-2.36	PSR13000978.hg.1	C13orf33
TC13000103.hg.1	-3.75	JUC13000567.hg.1	C13orf33
TC14001443.hg.1	2.31	PSR14016140.hg.1	FBLN5
TC14001443.hg.1	-2.19	PSR14016129.hg.1	FBLN5
TC14001443.hg.1	-2.35	JUC14008285.hg.1	FBLN5
TC14001443.hg.1	-4.47	PSR14016137.hg.1	FBLN5
TC14001443.hg.1	-5.76	JUC14008288.hg.1	FBLN5
TC15000405.hg.1	2.31	PSR15003781.hg.1	GLDN
TC15000405.hg.1	-2.23	JUC15001783.hg.1	GLDN
TC15000405.hg.1	-2.24	JUC15001781.hg.1	GLDN
TC15000405.hg.1	-2.4	PSR15003768.hg.1	GLDN
TC15000405.hg.1	-2.52	PSR15003763.hg.1	GLDN
TC15000405.hg.1	-2.76	PSR15003761.hg.1	GLDN
TC15000405.hg.1	-2.77	PSR15003762.hg.1	GLDN
TC15000405.hg.1	-4.37	PSR15003773.hg.1	GLDN
TC15000872.hg.1	2.31	PSR15008300.hg.1	MAN2A2
TC15000872.hg.1	2.11	JUC15004423.hg.1	MAN2A2
TC16000647.hg.1	2.31	PSR16009131.hg.1	CDH13
TC16000647.hg.1	2.25	JUC16005086.hg.1	CDH13
TC16000647.hg.1	-2.07	JUC16005089.hg.1	CDH13
TC16000647.hg.1	-2.19	JUC16005076.hg.1	CDH13
TC16000647.hg.1	-3.89	PSR16009134.hg.1	CDH13
TC17000369.hg.1	2.31	JUC17002653.hg.1	SUZ12
TC17000369.hg.1	-2.04	PSR17004644.hg.1	SUZ12
TC17000369.hg.1	-2.11	JUC17002650.hg.1	SUZ12
TC17000369.hg.1	-3.47	JUC17002655.hg.1	SUZ12
TC18000097.hg.1	2.31	JUC18000721.hg.1	RBBP8, MIR4
TC18000097.hg.1	-2.21	PSR18001051.hg.1	RBBP8, MIR4
TC18000097.hg.1	-2.71	PSR18001056.hg.1	RBBP8, MIR4
TC18000097.hg.1	-3.18	PSR18001053.hg.1	RBBP8, MIR4
TC18000097.hg.1	-9.11	PSR18001059.hg.1	RBBP8, MIR4
TC18000309.hg.1	2.31	JUC18002447.hg.1	LAMA1
TC18000309.hg.1	2.06	JUC18002489.hg.1	LAMA1
TC18000309.hg.1	-2.02	JUC18002473.hg.1	LAMA1
TC18000309.hg.1	-2.07	JUC18002458.hg.1	LAMA1
TC18000309.hg.1	-2.13	JUC18002461.hg.1	LAMA1
TC18000309.hg.1	-2.22	PSR18003647.hg.1	LAMA1
TC18000309.hg.1	-2.95	JUC18002494.hg.1	LAMA1
TC19001751.hg.1	2.31	JUC19013541.hg.1	SYT3
TC19001751.hg.1	2.28	PSR19023824.hg.1	SYT3

TC19001751.hg.1	2.28	JUC19013537.hg.1	SYT3
TC19001751.hg.1	-2.51	JUC19013543.hg.1	SYT3
TC20000171.hg.1	2.31	JUC20001152.hg.1	
TC20000171.hg.1	2.23	PSR20002144.hg.1	
TC20000171.hg.1	2.21	JUC20001148.hg.1	
TC22001426.hg.1	2.31	PSR22005783.hg.1	APOBEC3C
TC22001426.hg.1	-2.85	JUC22008599.hg.1	APOBEC3C
TC10001507.hg.1	2.31	JUC10011090.hg.1	PANK1
TC10001507.hg.1	2	PSR10019042.hg.1	PANK1
TC10001507.hg.1	-3.13	JUC10011086.hg.1	PANK1
TC01004004.hg.1	2.3	PSR01060632.hg.1	ERO1LB
TC01004004.hg.1	2.24	PSR01060622.hg.1	ERO1LB
TC01004004.hg.1	2.01	PSR01060633.hg.1	ERO1LB
TC01004004.hg.1	-2.86	JUC01031720.hg.1	ERO1LB
TC02001017.hg.1	2.3	PSR02015023.hg.1	GORASP2
TC02001017.hg.1	-2.08	JUC02008024.hg.1	GORASP2
TC02002481.hg.1	2.3	JUC02020512.hg.1	IFIH1
TC02002481.hg.1	-2.19	PSR02039149.hg.1	IFIH1
TC02002481.hg.1	-2.76	JUC02020505.hg.1	IFIH1
TC03000068.hg.1	2.3	PSR03001515.hg.1	SYN2
TC03000068.hg.1	-2.17	JUC03000796.hg.1	SYN2
TC03002173.hg.1	2.3	JUC03018991.hg.1	MFI2
TC03002173.hg.1	2.07	PSR03038387.hg.1	MFI2
TC05001223.hg.1	2.3	JUC05008848.hg.1	DROSHA
TC05001223.hg.1	2.12	JUC05008859.hg.1	DROSHA
TC05001223.hg.1	-2.01	JUC05008834.hg.1	DROSHA
TC05001223.hg.1	-2.02	PSR05017266.hg.1	DROSHA
TC05001223.hg.1	-2.03	PSR05017249.hg.1	DROSHA
TC05001223.hg.1	-2.07	PSR05017251.hg.1	DROSHA
TC05001223.hg.1	-2.07	JUC05008850.hg.1	DROSHA
TC05001223.hg.1	-2.09	JUC05008837.hg.1	DROSHA
TC05001223.hg.1	-2.36	JUC05008839.hg.1	DROSHA
TC05001817.hg.1	2.3	JUC05012664.hg.1	KLHL3
TC05001817.hg.1	2.08	JUC05012667.hg.1	KLHL3
TC05001817.hg.1	-2.05	JUC05012656.hg.1	KLHL3
TC05001817.hg.1	-2.13	JUC05012666.hg.1	KLHL3
TC05001817.hg.1	-2.14	PSR05024689.hg.1	KLHL3
TC05001817.hg.1	-2.43	JUC05012658.hg.1	KLHL3
TC05001817.hg.1	-4	JUC05012663.hg.1	KLHL3
TC06000233.hg.1	2.3	JUC06000987.hg.1	PRSS16
TC06000233.hg.1	2.01	PSR06002112.hg.1	PRSS16
TC06000233.hg.1	-2.56	JUC06000999.hg.1	PRSS16
TC06001232.hg.1	2.3	JUC06007286.hg.1	PXDC1
TC06001232.hg.1	2.2	PSR06014642.hg.1	PXDC1
TC06001232.hg.1	-3.87	JUC06007291.hg.1	PXDC1
TC06001743.hg.1	2.3	PSR06020831.hg.1	MRPL2
TC06001743.hg.1	2.27	PSR06020847.hg.1	MRPL2
TC06001743.hg.1	2.25	JUC06009901.hg.1	MRPL2
TC07000454.hg.1	2.3	PSR07006164.hg.1	CLIP2
TC07000454.hg.1	2.28	PSR07006178.hg.1	CLIP2
TC07000454.hg.1	2.24	JUC07002988.hg.1	CLIP2
TC07000454.hg.1	2.14	PSR07006185.hg.1	CLIP2
TC0X000013.hg.1	2.3	JUC0X000093.hg.1	CD99P1
TC0X000013.hg.1	-2.59	PSR0X000193.hg.1	CD99P1
TC0X000795.hg.1	2.3	JUC0X005328.hg.1	PPP2R3B
TC0X000795.hg.1	2.27	PSR0X010861.hg.1	PPP2R3B
TC0X000795.hg.1	-2.14	JUC0X005330.hg.1	PPP2R3B
TC10001253.hg.1	2.3	PSR10015611.hg.1	PTPN20B, PT

TC10001253.hg.1	2.11	JUC10009049.hg.1	PTPN20B, PT
TC10001253.hg.1	-2.31	JUC10009031.hg.1	PTPN20B, PT
TC10001319.hg.1	2.3	JUC10009721.hg.1	ANK3
TC10001319.hg.1	2.01	PSR10016655.hg.1	ANK3
TC10001319.hg.1	-2.04	PSR10016558.hg.1	ANK3
TC10001319.hg.1	-2.12	JUC10009703.hg.1	ANK3
TC10001319.hg.1	-2.14	PSR10016619.hg.1	ANK3
TC10001319.hg.1	-2.15	PSR10016553.hg.1	ANK3
TC10001319.hg.1	-2.28	PSR10016650.hg.1	ANK3
TC10001319.hg.1	-2.29	JUC10009722.hg.1	ANK3
TC10001319.hg.1	-2.3	PSR10016541.hg.1	ANK3
TC10001319.hg.1	-2.3	PSR10016652.hg.1	ANK3
TC10001319.hg.1	-2.33	PSR10016616.hg.1	ANK3
TC10001319.hg.1	-2.42	PSR10016659.hg.1	ANK3
TC10001319.hg.1	-2.46	PSR10016547.hg.1	ANK3
TC10001319.hg.1	-2.51	JUC10009715.hg.1	ANK3
TC10001319.hg.1	-2.52	PSR10016566.hg.1	ANK3
TC10001319.hg.1	-2.58	PSR10016581.hg.1	ANK3
TC10001319.hg.1	-2.7	JUC10009691.hg.1	ANK3
TC10001319.hg.1	-2.76	PSR10016586.hg.1	ANK3
TC10001319.hg.1	-2.77	JUC10009672.hg.1	ANK3
TC10001319.hg.1	-2.8	PSR10016613.hg.1	ANK3
TC10001319.hg.1	-2.86	JUC10009681.hg.1	ANK3
TC10001319.hg.1	-3.01	JUC10009718.hg.1	ANK3
TC10001319.hg.1	-3.1	JUC10009645.hg.1	ANK3
TC10001319.hg.1	-3.13	JUC10009720.hg.1	ANK3
TC10001319.hg.1	-3.31	PSR10016660.hg.1	ANK3
TC10001319.hg.1	-3.36	JUC10009697.hg.1	ANK3
TC10001319.hg.1	-3.62	PSR10016612.hg.1	ANK3
TC10001319.hg.1	-3.87	JUC10009686.hg.1	ANK3
TC10001319.hg.1	-3.89	PSR10016617.hg.1	ANK3
TC10001319.hg.1	-3.91	JUC10009673.hg.1	ANK3
TC10001319.hg.1	-3.94	JUC10009659.hg.1	ANK3
TC10001319.hg.1	-4.37	JUC10009706.hg.1	ANK3
TC10001319.hg.1	-4.65	JUC10009682.hg.1	ANK3
TC10001319.hg.1	-4.88	JUC10009683.hg.1	ANK3
TC11001399.hg.1	2.3	PSR11017681.hg.1	TMEM41B
TC11001399.hg.1	-3.42	JUC11009446.hg.1	TMEM41B
TC14001003.hg.1	2.3	JUC14005721.hg.1	
TC14001003.hg.1	2.13	PSR14011449.hg.1	
TC14001211.hg.1	2.3	JUC14006958.hg.1	ESR2
TC14001211.hg.1	2.18	JUC14006961.hg.1	ESR2
TC14001211.hg.1	-2	PSR14013824.hg.1	ESR2
TC14001211.hg.1	-2.98	PSR14013834.hg.1	ESR2
TC15001818.hg.1	2.3	JUC15009610.hg.1	DET1
TC15001818.hg.1	2.28	PSR15017593.hg.1	DET1
TC15001818.hg.1	2.09	PSR15017609.hg.1	DET1
TC17001612.hg.1	2.3	JUC17012173.hg.1	ARL17B, ARL
TC17001612.hg.1	2.23	JUC17012175.hg.1	ARL17B, ARL
TC17001612.hg.1	2.02	PSR17021647.hg.1	ARL17B, ARL
TC17002873.hg.1	2.3	JUC17018650.hg.1	TMEM199, M
TC17002873.hg.1	2.02	PSR17003866.hg.1	TMEM199, M
TC17002873.hg.1	-2.44	JUC17018647.hg.1	TMEM199, M
TC19000350.hg.1	2.3	JUC19002729.hg.1	ARRDC2
TC19000350.hg.1	2.09	JUC19002726.hg.1	ARRDC2
TC19000350.hg.1	-2.13	PSR19004616.hg.1	ARRDC2
TC19001284.hg.1	2.3	PSR19018263.hg.1	KIAA1683
TC19001284.hg.1	2.25	PSR19018279.hg.1	KIAA1683

TC19001284.hg.1	2.18	JUC19010674.hg.1	KIAA1683
TC19001284.hg.1	2.17	PSR19018283.hg.1	KIAA1683
TC19001284.hg.1	2.15	JUC19010668.hg.1	KIAA1683
TC19001284.hg.1	2.15	JUC19010672.hg.1	KIAA1683
TC19001284.hg.1	-2.1	PSR19018268.hg.1	KIAA1683
TC19001284.hg.1	-2.6	PSR19018280.hg.1	KIAA1683
TC20000533.hg.1	2.3	JUC20003989.hg.1	TCEA2
TC20000533.hg.1	2.02	PSR20007622.hg.1	TCEA2
TC6_cox_hap2000	2.3	JUC6_cox_hap20012	EHMT2
TC6_cox_hap2000	-2.07	PSR6_cox_hap2003	EHMT2
TC6_dbb_hap3000	2.3	JUC6_dbb_hap3001	EHMT2
TC6_dbb_hap3000	-2.07	PSR6_dbb_hap3003	EHMT2
TC6_qbl_hap6000	2.3	JUC6_qbl_hap60010	GPANK1
TC6_qbl_hap6000	2.03	PSR6_qbl_hap60029	GPANK1
TC6_qbl_hap6000	2.02	PSR6_qbl_hap60029	GPANK1
TC6_ssto_hap700	2.3	JUC6_ssto_hap7001	GPANK1
TC6_ssto_hap700	2.03	PSR6_ssto_hap7002	GPANK1
TC6_ssto_hap700	2.02	PSR6_ssto_hap7002	GPANK1
TC01000426.hg.1	2.29	PSR01006544.hg.1	LCK
TC01000426.hg.1	2.05	JUC01003412.hg.1	LCK
TC01001624.hg.1	2.29	JUC01013424.hg.1	RGS2
TC01001624.hg.1	-2.44	PSR01025081.hg.1	RGS2
TC01001657.hg.1	2.29	JUC01013689.hg.1	IGFN1
TC01001657.hg.1	2.27	JUC01013686.hg.1	IGFN1
TC01001657.hg.1	2.01	PSR01025638.hg.1	IGFN1
TC01001657.hg.1	-3	PSR01025615.hg.1	IGFN1
TC01001657.hg.1	-3.01	JUC01013680.hg.1	IGFN1
TC01002578.hg.1	2.29	PSR01040081.hg.1	SLC2A1
TC01002578.hg.1	2.28	JUC01021513.hg.1	SLC2A1
TC01002578.hg.1	2.19	PSR01040069.hg.1	SLC2A1
TC01002578.hg.1	2.09	PSR01040091.hg.1	SLC2A1
TC01002578.hg.1	2.06	PSR01040089.hg.1	SLC2A1
TC01004071.hg.1	2.29	JUC01032127.hg.1	ZNF496
TC01004071.hg.1	-2.16	PSR01061417.hg.1	ZNF496
TC01004071.hg.1	-2.37	PSR01061406.hg.1	ZNF496
TC03000692.hg.1	2.29	JUC03006220.hg.1	IFT122
TC03000692.hg.1	2.1	PSR03012379.hg.1	IFT122
TC03000692.hg.1	-2.16	JUC03006207.hg.1	IFT122
TC03000692.hg.1	-2.17	JUC03006169.hg.1	IFT122
TC03000692.hg.1	-2.21	JUC03006172.hg.1	IFT122
TC03000692.hg.1	-2.96	JUC03006191.hg.1	IFT122
TC03000873.hg.1	2.29	PSR03015564.hg.1	SMC4
TC03000873.hg.1	2.13	JUC03007879.hg.1	SMC4
TC03000873.hg.1	-2.51	JUC03007892.hg.1	SMC4
TC03000873.hg.1	-2.85	JUC03007872.hg.1	SMC4
TC03000930.hg.1	2.29	JUC03008219.hg.1	ECT2
TC03000930.hg.1	2.1	JUC03008203.hg.1	ECT2
TC03000930.hg.1	2.09	JUC03008229.hg.1	ECT2
TC03000930.hg.1	2	PSR03016158.hg.1	ECT2
TC03000930.hg.1	-2.19	JUC03008206.hg.1	ECT2
TC03000930.hg.1	-2.79	PSR03016192.hg.1	ECT2
TC03000930.hg.1	-3.37	JUC03008232.hg.1	ECT2
TC04001600.hg.1	2.29	JUC04011751.hg.1	INPP4B
TC04001600.hg.1	2.16	PSR04022292.hg.1	INPP4B
TC04001600.hg.1	2.14	JUC04011760.hg.1	INPP4B
TC04001600.hg.1	2.07	PSR04022301.hg.1	INPP4B
TC04001600.hg.1	-2.07	PSR04022319.hg.1	INPP4B
TC04001600.hg.1	-2.11	JUC04011759.hg.1	INPP4B

TC04001600.hg.1	-2.38	JUC04011737.hg.1	INPP4B
TC04001600.hg.1	-2.5	JUC04011779.hg.1	INPP4B
TC04001600.hg.1	-2.62	JUC04011787.hg.1	INPP4B
TC04001600.hg.1	-2.8	JUC04011747.hg.1	INPP4B
TC04001600.hg.1	-4.71	PSR04022332.hg.1	INPP4B
TC04001600.hg.1	-5.39	PSR04022314.hg.1	INPP4B
TC04001600.hg.1	-5.65	JUC04011792.hg.1	INPP4B
TC04001600.hg.1	-6.46	PSR04022313.hg.1	INPP4B
TC05001674.hg.1	2.29	JUC05011701.hg.1	NREP
TC05001674.hg.1	-2.09	JUC05011710.hg.1	NREP
TC05001674.hg.1	-2.21	PSR05022649.hg.1	NREP
TC05001674.hg.1	-2.53	PSR05022621.hg.1	NREP
TC05001674.hg.1	-2.55	PSR05022623.hg.1	NREP
TC05001674.hg.1	-2.73	PSR05022624.hg.1	NREP
TC05001674.hg.1	-3.4	PSR05022622.hg.1	NREP
TC05003404.hg.1	2.29	JUC05019272.hg.1	BRD9
TC05003404.hg.1	2.2	PSR05015957.hg.1	BRD9
TC05003404.hg.1	-2.67	JUC05019288.hg.1	BRD9
TC05003404.hg.1	-3.7	JUC05019257.hg.1	BRD9
TC06001534.hg.1	2.29	JUC06008573.hg.1	GPANK1
TC06001534.hg.1	2.02	PSR06017650.hg.1	GPANK1
TC06001534.hg.1	2.01	PSR06017662.hg.1	GPANK1
TC08001179.hg.1	2.29	JUC08007796.hg.1	SLC20A2
TC08001179.hg.1	2.02	JUC08007792.hg.1	SLC20A2
TC08001179.hg.1	-2.28	PSR08015214.hg.1	SLC20A2
TC08001179.hg.1	-2.29	PSR08015221.hg.1	SLC20A2
TC08001179.hg.1	-2.59	JUC08007800.hg.1	SLC20A2
TC09000191.hg.1	2.29	PSR09002140.hg.1	CA9
TC09000191.hg.1	-2.26	JUC09001132.hg.1	CA9
TC09000191.hg.1	-2.29	PSR09002132.hg.1	CA9
TC09000191.hg.1	-2.64	PSR09002131.hg.1	CA9
TC09000191.hg.1	-3.44	PSR09002138.hg.1	CA9
TC09000191.hg.1	-4.69	JUC09001125.hg.1	CA9
TC09000191.hg.1	-10.06	JUC09001133.hg.1	CA9
TC09000191.hg.1	-11.19	JUC09001131.hg.1	CA9
TC09000191.hg.1	-27.17	PSR09002137.hg.1	CA9
TC11001513.hg.1	2.29	JUC11010105.hg.1	BDNF
TC11001513.hg.1	-2.24	PSR11018950.hg.1	BDNF
TC11001513.hg.1	-2.6	PSR11018962.hg.1	BDNF
TC11001513.hg.1	-2.6	JUC11010104.hg.1	BDNF
TC12000804.hg.1	2.29	JUC12005607.hg.1	TDG
TC12000804.hg.1	-2.26	JUC12005597.hg.1	TDG
TC12000804.hg.1	-2.55	PSR12010230.hg.1	TDG
TC16000783.hg.1	2.29	JUC16006454.hg.1	ABCA3
TC16000783.hg.1	-2.04	JUC16006468.hg.1	ABCA3
TC16000783.hg.1	-2.13	PSR16011516.hg.1	ABCA3
TC16000783.hg.1	-2.14	PSR16011534.hg.1	ABCA3
TC16000783.hg.1	-2.38	JUC16006462.hg.1	ABCA3
TC16000783.hg.1	-2.5	PSR16011529.hg.1	ABCA3
TC16000783.hg.1	-2.73	JUC16006460.hg.1	ABCA3
TC16000783.hg.1	-4.72	JUC16006455.hg.1	ABCA3
TC20000255.hg.1	2.29	PSR20003430.hg.1	ERGIC3
TC20000255.hg.1	2.15	PSR20003402.hg.1	ERGIC3
TC20000255.hg.1	2	JUC20001915.hg.1	ERGIC3
TC20000255.hg.1	-2.39	JUC20001917.hg.1	ERGIC3
TC21000353.hg.1	2.29	PSR21004079.hg.1	CCT8
TC21000353.hg.1	2.2	JUC21002192.hg.1	CCT8
TC22000723.hg.1	2.29	JUC22005327.hg.1	C1QTNF6

TC22000723.hg.1	-2.11	PSR22012770.hg.1	C1QTNF6
TC6_apd_hap1000	2.29	JUC6_apd_hap10005	EHMT2
TC6_apd_hap1000	-2.08	PSR6_apd_hap1001	EHMT2
TC6_dbb_hap3000	2.29	JUC6_dbb_hap3001(	GPANK1
TC6_dbb_hap3000	2.22	PSR6_dbb_hap3002	GPANK1
TC6_dbb_hap3000	2.03	PSR6_dbb_hap3002	GPANK1
TC6_dbb_hap3000	2.01	PSR6_dbb_hap3002	GPANK1
TC6_mann_hap40(	2.29	JUC6_mann_hap400	GPANK1
TC6_mann_hap40(	2.02	PSR6_mann_hap40C	GPANK1
TC6_mann_hap40(	2.01	PSR6_mann_hap40C	GPANK1
TC6_mcf_hap5000	2.29	JUC6_mcf_hap5000	GPANK1
TC6_mcf_hap5000	2.02	PSR6_mcf_hap5002	GPANK1
TC6_mcf_hap5000	2.01	PSR6_mcf_hap5002	GPANK1
TC6_mcf_hap5000	2.29	JUC6_mcf_hap5001(	EHMT2
TC6_mcf_hap5000	-2.08	PSR6_mcf_hap5003(	EHMT2
TC6_qbl_hap6000	2.29	JUC6_qbl_hap60011	EHMT2
TC6_qbl_hap6000	-2.08	PSR6_qbl_hap60032	EHMT2
TC13000416.hg.1	2.29	JUC13002795.hg.1	F7
TC13000416.hg.1	2	PSR13004644.hg.1	F7
TC01002256.hg.1	2.28	JUC01018417.hg.1	HSPB7
TC01002256.hg.1	2.2	PSR01034421.hg.1	HSPB7
TC01002256.hg.1	2.11	PSR01034409.hg.1	HSPB7
TC01002259.hg.1	2.28	JUC01018452.hg.1	ARHGEF19
TC01002259.hg.1	2.19	PSR01034466.hg.1	ARHGEF19
TC01002259.hg.1	-2.25	PSR01034488.hg.1	ARHGEF19
TC01002259.hg.1	-2.52	JUC01018461.hg.1	ARHGEF19
TC01006300.hg.1	2.28	JUC01039512.hg.1	STIL
TC01006300.hg.1	2	JUC01039506.hg.1	STIL
TC01006300.hg.1	-2.66	PSR01041161.hg.1	STIL
TC02000174.hg.1	2.28	JUC02001215.hg.1	NRBP1
TC02000174.hg.1	2.21	PSR02002314.hg.1	NRBP1
TC02000174.hg.1	2.2	JUC02001199.hg.1	NRBP1
TC04000711.hg.1	2.28	JUC04005423.hg.1	SMAD1
TC04000711.hg.1	-2.04	PSR04010104.hg.1	SMAD1
TC04000711.hg.1	-3.17	JUC04005420.hg.1	SMAD1
TC05001520.hg.1	2.28	JUC05010748.hg.1	DMGDH
TC05001520.hg.1	2.05	PSR05020831.hg.1	DMGDH
TC06000978.hg.1	2.28	PSR06011490.hg.1	L3MBTL3
TC06000978.hg.1	-2.12	PSR06011510.hg.1	L3MBTL3
TC06000978.hg.1	-2.12	JUC06005540.hg.1	L3MBTL3
TC06000978.hg.1	-2.42	JUC06005543.hg.1	L3MBTL3
TC06000978.hg.1	-2.43	JUC06005538.hg.1	L3MBTL3
TC06000978.hg.1	-3.26	JUC06005554.hg.1	L3MBTL3
TC06000978.hg.1	-4.66	JUC06005539.hg.1	L3MBTL3
TC08000880.hg.1	2.28	JUC08006014.hg.1	C8orf42
TC08000880.hg.1	2.09	PSR08011616.hg.1	C8orf42
TC09000270.hg.1	2.28	JUC09001528.hg.1	KGFLP1
TC09000270.hg.1	-2.27	PSR09002938.hg.1	KGFLP1
TC09000270.hg.1	-3.78	JUC09001529.hg.1	KGFLP1
TC10000211.hg.1	2.28	PSR10002458.hg.1	MAP3K8
TC10000211.hg.1	-2.13	JUC10001326.hg.1	MAP3K8
TC10000798.hg.1	2.28	PSR10009547.hg.1	MXI1
TC10000798.hg.1	2.13	JUC10005325.hg.1	MXI1
TC10000798.hg.1	-2.49	JUC10005308.hg.1	MXI1
TC10000798.hg.1	-3.22	JUC10005310.hg.1	MXI1
TC11001194.hg.1	2.28	JUC11008095.hg.1	APLP2
TC11001194.hg.1	2.03	PSR11014996.hg.1	APLP2
TC11001355.hg.1	2.28	JUC11009069.hg.1	APBB1

TC11001355.hg.1	2.16	PSR11017001.hg.1	APBB1
TC11001355.hg.1	2.04	PSR11017009.hg.1	APBB1
TC11001355.hg.1	-2.11	JUC11009088.hg.1	APBB1
TC11001355.hg.1	-2.15	JUC11009079.hg.1	APBB1
TC13000273.hg.1	2.28	PSR13002992.hg.1	KLF5
TC13000273.hg.1	-2.06	PSR13002999.hg.1	KLF5
TC13000273.hg.1	-2.46	PSR13002985.hg.1	KLF5
TC13000273.hg.1	-2.72	JUC13001872.hg.1	KLF5
TC17000184.hg.1	2.28	JUC17001433.hg.1	PIGL
TC17000184.hg.1	2.2	PSR17002452.hg.1	PIGL
TC17000918.hg.1	2.28	JUC17006725.hg.1	RNF213
TC17000918.hg.1	2.02	PSR17012012.hg.1	RNF213
TC17000918.hg.1	-2.14	JUC17006783.hg.1	RNF213
TC17000918.hg.1	-2.22	JUC17006721.hg.1	RNF213
TC17000918.hg.1	-3.37	JUC17006760.hg.1	RNF213
TC17001455.hg.1	2.28	PSR17019186.hg.1	IKZF3
TC17001455.hg.1	-2.34	JUC17010896.hg.1	IKZF3
TC17001540.hg.1	2.28	JUC17011528.hg.1	EZH1
TC17001540.hg.1	-2.26	PSR17020607.hg.1	EZH1
TC17001540.hg.1	-2.28	JUC17011541.hg.1	EZH1
TC17001540.hg.1	-2.42	JUC17011533.hg.1	EZH1
TC17001540.hg.1	-3.1	JUC17011518.hg.1	EZH1
TC17001734.hg.1	2.28	PSR17023249.hg.1	MTMR4
TC17001734.hg.1	2.12	JUC17012939.hg.1	MTMR4
TC17001734.hg.1	-2.21	PSR17023231.hg.1	MTMR4
TC17002878.hg.1	2.28	PSR17007414.hg.1	ITGB3
TC17002878.hg.1	-2.07	PSR17007428.hg.1	ITGB3
TC17002878.hg.1	-2.22	JUC17018714.hg.1	ITGB3
TC17002878.hg.1	-2.8	JUC17018713.hg.1	ITGB3
TC17002879.hg.1	2.28	JUC17018727.hg.1	C17orf57
TC17002879.hg.1	2.08	PSR17007448.hg.1	C17orf57
TC17002879.hg.1	-2.45	JUC17018731.hg.1	C17orf57
TC17002879.hg.1	-2.6	JUC17018748.hg.1	C17orf57
TC17002879.hg.1	-3.41	JUC17018717.hg.1	C17orf57
TC17002879.hg.1	-5.16	JUC17018721.hg.1	C17orf57
TC18000033.hg.1	2.28	JUC18000180.hg.1	ARHGAP28
TC18000033.hg.1	-2.08	JUC18000181.hg.1	ARHGAP28
TC18000033.hg.1	-2.62	PSR18000302.hg.1	ARHGAP28
TC19001127.hg.1	2.28	JUC19008862.hg.1	CD320
TC19001127.hg.1	2.17	PSR19015517.hg.1	CD320
TC02000032.hg.1	2.28	JUC02000208.hg.1	LOC150622,
TC02000032.hg.1	2	PSR02000383.hg.1	LOC150622,
TC01000873.hg.1	2.27	PSR01013844.hg.1	SLC44A3
TC01000873.hg.1	2.17	JUC01007321.hg.1	SLC44A3
TC01000873.hg.1	-2.81	JUC01007304.hg.1	SLC44A3
TC01000873.hg.1	-2.96	PSR01013829.hg.1	SLC44A3
TC01000873.hg.1	-2.98	PSR01013849.hg.1	SLC44A3
TC01000873.hg.1	-3.35	PSR01013831.hg.1	SLC44A3
TC01000873.hg.1	-5.58	JUC01007316.hg.1	SLC44A3
TC01002911.hg.1	2.27	JUC01024348.hg.1	FRRS1
TC01002911.hg.1	2.25	JUC01024343.hg.1	FRRS1
TC01002911.hg.1	2.2	PSR01045289.hg.1	FRRS1
TC01002911.hg.1	2.11	JUC01024338.hg.1	FRRS1
TC01002911.hg.1	2.04	PSR01045288.hg.1	FRRS1
TC01002911.hg.1	2.02	PSR01045292.hg.1	FRRS1
TC01002911.hg.1	-2.04	PSR01045293.hg.1	FRRS1
TC01002911.hg.1	-2.27	JUC01024336.hg.1	FRRS1
TC01002911.hg.1	-2.31	PSR01045290.hg.1	FRRS1

TC01002911.hg.1	-2.34	JUC01024339.hg.1	FRRS1
TC01002911.hg.1	-2.36	PSR01045275.hg.1	FRRS1
TC01002911.hg.1	-2.39	PSR01045278.hg.1	FRRS1
TC02000250.hg.1	2.27	JUC02001930.hg.1	LOC728730
TC02000250.hg.1	-2.48	PSR02003748.hg.1	LOC728730
TC02002510.hg.1	2.27	JUC02020981.hg.1	FASTKD1
TC02002510.hg.1	-2.25	PSR02039843.hg.1	FASTKD1
TC03001608.hg.1	2.27	PSR03029025.hg.1	FILIP1L
TC03001608.hg.1	2.16	PSR03029019.hg.1	FILIP1L
TC03001608.hg.1	2.13	PSR03028999.hg.1	FILIP1L
TC03001608.hg.1	-2	PSR03029006.hg.1	FILIP1L
TC03001608.hg.1	-2.62	JUC03014270.hg.1	FILIP1L
TC05000725.hg.1	2.27	JUC05005461.hg.1	SLC4A9
TC05000725.hg.1	2.11	JUC05005441.hg.1	SLC4A9
TC05000725.hg.1	2.07	PSR05010297.hg.1	SLC4A9
TC07000065.hg.1	2.27	JUC07000448.hg.1	RAC1
TC07000065.hg.1	2.22	PSR07001055.hg.1	RAC1
TC07000594.hg.1	2.27	JUC07004139.hg.1	CPSF4
TC07000594.hg.1	2.2	PSR07008796.hg.1	CPSF4
TC07000594.hg.1	-2.4	JUC07004132.hg.1	CPSF4
TC07000594.hg.1	-2.44	PSR07008792.hg.1	CPSF4
TC07000594.hg.1	-2.7	PSR07008793.hg.1	CPSF4
TC0X000357.hg.1	2.27	PSR0X004610.hg.1	HEPH
TC0X000357.hg.1	2.04	JUC0X002218.hg.1	HEPH
TC0X001535.hg.1	2.27	PSR0X020512.hg.1	UBL4A
TC0X001535.hg.1	2.02	JUC0X010245.hg.1	UBL4A
TC12000369.hg.1	2.27	PSR12004547.hg.1	PRPH
TC12000369.hg.1	-2.11	JUC12002480.hg.1	PRPH
TC12000369.hg.1	-2.23	PSR12004554.hg.1	PRPH
TC12000979.hg.1	2.27	JUC12007088.hg.1	HIP1R
TC12000979.hg.1	-2.27	JUC12007073.hg.1	HIP1R
TC12000979.hg.1	-2.95	JUC12007089.hg.1	HIP1R
TC12000979.hg.1	-3.06	PSR12012873.hg.1	HIP1R
TC12000979.hg.1	-5.11	JUC12007085.hg.1	HIP1R
TC14000131.hg.1	2.27	JUC14000471.hg.1	OXA1L
TC14000131.hg.1	2.09	PSR14001071.hg.1	OXA1L
TC14001343.hg.1	2.27	PSR14015259.hg.1	TMED8
TC14001343.hg.1	2.25	JUC14007753.hg.1	TMED8
TC17_ctg5_hap10(	2.27	PSR17_ctg5_hap100	LRRC37A2, L
TC17_ctg5_hap10(	-3.06	JUC17_ctg5_hap100	LRRC37A2, L
TC17001560.hg.1	2.27	JUC17011705.hg.1	ETV4
TC17001560.hg.1	-2.27	PSR17020897.hg.1	ETV4
TC17001589.hg.1	2.27	JUC17012020.hg.1	KIF18B
TC17001589.hg.1	-2.16	PSR17021421.hg.1	KIF18B
TC20000051.hg.1	2.27	JUC20000395.hg.1	PRNP
TC20000051.hg.1	2.23	PSR20000809.hg.1	PRNP
TC6_apd_hap1000	2.27	PSR6_apd_hap1000	GABBR1
TC6_apd_hap1000	2	JUC6_apd_hap1000	GABBR1
TC6_apd_hap1000	-2.18	JUC6_apd_hap1000	GABBR1
TC6_cox_hap2000	2.27	JUC6_cox_hap2001(	GPANK1
TC6_cox_hap2000	2.01	PSR6_cox_hap2002	GPANK1
TC02000755.hg.1	2.27	JUC02006246.hg.1	TMEM177
TC02000755.hg.1	2.16	JUC02006238.hg.1	TMEM177
TC02000755.hg.1	2	PSR02011810.hg.1	TMEM177
TC01001337.hg.1	2.26	PSR01020997.hg.1	CD1D
TC01001337.hg.1	2.2	PSR01020995.hg.1	CD1D
TC01001337.hg.1	-2.47	PSR01021002.hg.1	CD1D
TC01001337.hg.1	-13.26	JUC01011189.hg.1	CD1D

TC02000328.hg.1	2.26	PSR02005012.hg.1	CCDC85A
TC02000328.hg.1	2.23	PSR02005005.hg.1	CCDC85A
TC02000328.hg.1	2.16	PSR02005009.hg.1	CCDC85A
TC02000328.hg.1	2.11	PSR02005013.hg.1	CCDC85A
TC02000328.hg.1	2.1	PSR02005006.hg.1	CCDC85A
TC02000328.hg.1	2.08	JUC02002627.hg.1	CCDC85A
TC02000328.hg.1	-2	JUC02002621.hg.1	CCDC85A
TC02000328.hg.1	-2.31	PSR02005017.hg.1	CCDC85A
TC02000328.hg.1	-2.6	PSR02005020.hg.1	CCDC85A
TC02000328.hg.1	-2.61	PSR02005021.hg.1	CCDC85A
TC02000328.hg.1	-3.29	JUC02002620.hg.1	CCDC85A
TC02001386.hg.1	2.26	JUC02011545.hg.1	PSMD1
TC02001386.hg.1	2.02	JUC02011548.hg.1	PSMD1
TC02001386.hg.1	-2.58	PSR02021772.hg.1	PSMD1
TC02001978.hg.1	2.26	JUC02016450.hg.1	SFXN5
TC02001978.hg.1	2.13	JUC02016461.hg.1	SFXN5
TC02001978.hg.1	-2.15	PSR02031575.hg.1	SFXN5
TC02001978.hg.1	-2.16	PSR02031555.hg.1	SFXN5
TC02001978.hg.1	-2.18	PSR02031567.hg.1	SFXN5
TC02001978.hg.1	-2.19	JUC02016478.hg.1	SFXN5
TC02001978.hg.1	-2.25	JUC02016472.hg.1	SFXN5
TC02002121.hg.1	2.26	JUC02017767.hg.1	ACTR1B
TC02002121.hg.1	-2.03	PSR02034458.hg.1	ACTR1B
TC02005063.hg.1	2.26	JUC02033337.hg.1	BAZ2B
TC02005063.hg.1	-2.15	PSR02038714.hg.1	BAZ2B
TC02005063.hg.1	-2.22	JUC02033334.hg.1	BAZ2B
TC03001203.hg.1	2.26	PSR03020637.hg.1	ZFYVE20
TC03001203.hg.1	2.23	JUC03010331.hg.1	ZFYVE20
TC03001203.hg.1	-2.01	JUC03010314.hg.1	ZFYVE20
TC05001644.hg.1	2.26	PSR05022352.hg.1	GIN1
TC05001644.hg.1	2.26	JUC05011545.hg.1	GIN1
TC05001644.hg.1	2.19	PSR05022355.hg.1	GIN1
TC05001644.hg.1	-2.23	PSR05022361.hg.1	GIN1
TC06001959.hg.1	2.26	PSR06024128.hg.1	MMS22L
TC06001959.hg.1	-2	PSR06024105.hg.1	MMS22L
TC06001959.hg.1	-2.01	PSR06024101.hg.1	MMS22L
TC06001959.hg.1	-2.02	PSR06024086.hg.1	MMS22L
TC06001959.hg.1	-2.07	PSR06024096.hg.1	MMS22L
TC06001959.hg.1	-2.13	JUC06011810.hg.1	MMS22L
TC06001959.hg.1	-2.29	PSR06024099.hg.1	MMS22L
TC06001959.hg.1	-2.6	JUC06011790.hg.1	MMS22L
TC06004050.hg.1	2.26	PSR06018440.hg.1	PBX2
TC06004050.hg.1	2.08	JUC06020561.hg.1	PBX2
TC07001303.hg.1	2.26	JUC07009903.hg.1	AMPH
TC07001303.hg.1	-2.14	PSR07020202.hg.1	AMPH
TC07001548.hg.1	2.26	JUC07011519.hg.1	PION
TC07001548.hg.1	2.15	PSR07023278.hg.1	PION
TC07001548.hg.1	-2.4	JUC07011528.hg.1	PION
TC07003367.hg.1	2.26	PSR07025283.hg.1	TRIM4
TC07003367.hg.1	-2.43	JUC07021507.hg.1	TRIM4
TC0X000385.hg.1	2.26	PSR0X005139.hg.1	NONO
TC0X000385.hg.1	-2.2	JUC0X002519.hg.1	NONO
TC0X000568.hg.1	2.26	JUC0X003697.hg.1	PLS3
TC0X000568.hg.1	-2.47	PSR0X007468.hg.1	PLS3
TC0X000568.hg.1	-2.84	JUC0X003698.hg.1	PLS3
TC0X000568.hg.1	-3.47	PSR0X007473.hg.1	PLS3
TC11001728.hg.1	2.26	JUC11010730.hg.1	LRP4
TC11001728.hg.1	-2.06	PSR11020147.hg.1	LRP4

TC11001728.hg.1	-2.28	JUC11010708.hg.1	LRP4
TC11001728.hg.1	-3.21	JUC11010725.hg.1	LRP4
TC11001814.hg.1	2.26	PSR11020979.hg.1	GLYATL2
TC11001814.hg.1	2.01	JUC11011193.hg.1	GLYATL2
TC12000496.hg.1	2.26	PSR12006292.hg.1	CDK2
TC12000496.hg.1	2.25	JUC12003363.hg.1	CDK2
TC12000496.hg.1	2.24	PSR12006282.hg.1	CDK2
TC12003251.hg.1	2.26	PSR12008188.hg.1	RAB3IP
TC12003251.hg.1	-3.39	JUC12020119.hg.1	RAB3IP
TC13000458.hg.1	2.26	PSR13005225.hg.1	CRYL1
TC13000458.hg.1	2.24	PSR13005227.hg.1	CRYL1
TC13000458.hg.1	-2.36	JUC13003131.hg.1	CRYL1
TC16002074.hg.1	2.26	PSR16006477.hg.1	MT1M
TC16002074.hg.1	-5.52	JUC16013728.hg.1	MT1M
TC16002074.hg.1	-7.3	PSR16006478.hg.1	MT1M
TC16002074.hg.1	-10.63	PSR16006475.hg.1	MT1M
TC17_ctg5_hap10(	2.26	PSR17_ctg5_hap100	PLEKHM1, M
TC17_ctg5_hap10(	2.16	JUC17_ctg5_hap100	PLEKHM1, M
TC17_ctg5_hap10(	2.05	PSR17_ctg5_hap100	PLEKHM1, M
TC17001465.hg.1	2.26	PSR17019465.hg.1	TNS4
TC17001465.hg.1	2.1	JUC17011027.hg.1	TNS4
TC17001465.hg.1	-2.21	PSR17019472.hg.1	TNS4
TC17001465.hg.1	-2.99	JUC17011026.hg.1	TNS4
TC19001485.hg.1	2.26	JUC19011589.hg.1	ZNF571
TC19001485.hg.1	-3.26	PSR19020146.hg.1	ZNF571
TC22000337.hg.1	2.26	JUC22002390.hg.1	ACO2
TC22000337.hg.1	2.21	PSR22006504.hg.1	ACO2
TC22000337.hg.1	2.05	PSR22006505.hg.1	ACO2
TC22000337.hg.1	-2.05	PSR22006491.hg.1	ACO2
TC22001428.hg.1	2.26	JUC22008626.hg.1	MTMR3
TC22001428.hg.1	-2.06	JUC22008617.hg.1	MTMR3
TC22001428.hg.1	-2.54	PSR22003793.hg.1	MTMR3
TC22001428.hg.1	-2.67	PSR22003802.hg.1	MTMR3
TC6_apd_hap1000	2.26	PSR6_apd_hap1001	PBX2
TC6_apd_hap1000	2.08	JUC6_apd_hap1000(	PBX2
TC6_cox_hap2000	2.26	PSR6_cox_hap2003(	PBX2
TC6_cox_hap2000	2.08	JUC6_cox_hap2001	PBX2
TC6_cox_hap2000	2.26	PSR6_cox_hap2002	GABBR1
TC6_cox_hap2000	-2.19	JUC6_cox_hap2002(	GABBR1
TC6_dbb_hap3000	2.26	PSR6_dbb_hap3002(	GABBR1
TC6_dbb_hap3000	-2.19	JUC6_dbb_hap3001	GABBR1
TC6_mcf_hap5000	2.26	PSR6_mcf_hap5001	GABBR1
TC6_mcf_hap5000	-2.19	JUC6_mcf_hap5001	GABBR1
TC6_ssto_hap700(	2.26	PSR6_ssto_hap7003	PBX2
TC6_ssto_hap700(	2.08	JUC6_ssto_hap7001	PBX2
TC6_ssto_hap700(	-2	PSR6_ssto_hap7003	PBX2
TC01000975.hg.1	2.25	JUC01008141.hg.1	C1orf88
TC01000975.hg.1	-2.05	PSR01015569.hg.1	C1orf88
TC01001871.hg.1	2.25	JUC01015713.hg.1	ZNF678
TC01001871.hg.1	-2.1	PSR01029127.hg.1	ZNF678
TC01003087.hg.1	2.25	JUC01025678.hg.1	LOC728855,
TC01003087.hg.1	2.12	JUC01025690.hg.1	LOC728855,
TC01003087.hg.1	-2.11	PSR01047850.hg.1	LOC728855,
TC01003087.hg.1	-2.15	PSR01047862.hg.1	LOC728855,
TC01003087.hg.1	-2.18	JUC01025689.hg.1	LOC728855,
TC01003087.hg.1	-3.56	PSR01047816.hg.1	LOC728855,
TC01003091.hg.1	2.25	JUC01025715.hg.1	LOC728875
TC01003091.hg.1	2.12	JUC01025725.hg.1	LOC728875

TC01003091.hg.1	-2.11	PSR01047924.hg.1	LOC728875
TC01003091.hg.1	-2.15	PSR01047935.hg.1	LOC728875
TC01003091.hg.1	-2.18	JUC01025724.hg.1	LOC728875
TC01003091.hg.1	-2.85	JUC01025714.hg.1	LOC728875
TC02000233.hg.1	2.25	JUC02001818.hg.1	CCDC75
TC02000233.hg.1	2.04	JUC02001820.hg.1	CCDC75
TC02000233.hg.1	-2.28	PSR02003454.hg.1	CCDC75
TC02000365.hg.1	2.25	JUC02002898.hg.1	LOC1005070
TC02000365.hg.1	-2.25	PSR02005593.hg.1	LOC1005070
TC02000620.hg.1	2.25	JUC02005198.hg.1	IL1R1
TC02000620.hg.1	2.07	JUC02005200.hg.1	IL1R1
TC02000620.hg.1	-2.02	JUC02005210.hg.1	IL1R1
TC02000620.hg.1	-2.07	PSR02009815.hg.1	IL1R1
TC02000620.hg.1	-2.12	JUC02005192.hg.1	IL1R1
TC02000620.hg.1	-2.16	PSR02009840.hg.1	IL1R1
TC02000620.hg.1	-2.27	PSR02009806.hg.1	IL1R1
TC02000620.hg.1	-2.27	PSR02009811.hg.1	IL1R1
TC02000620.hg.1	-2.29	PSR02009814.hg.1	IL1R1
TC02000620.hg.1	-3.02	JUC02005209.hg.1	IL1R1
TC02001102.hg.1	2.25	JUC02008833.hg.1	
TC02001102.hg.1	2.11	PSR02016543.hg.1	
TC03003333.hg.1	2.25	PSR03036923.hg.1	ETV5
TC03003333.hg.1	-2.94	JUC03023240.hg.1	ETV5
TC04000874.hg.1	2.25	JUC04006443.hg.1	NEIL3
TC04000874.hg.1	2.2	PSR04011998.hg.1	NEIL3
TC04000874.hg.1	-2.09	JUC04006437.hg.1	NEIL3
TC04000874.hg.1	-3.21	JUC04006439.hg.1	NEIL3
TC06000283.hg.1	2.25	JUC06001083.hg.1	ZKSCAN3
TC06000283.hg.1	2.19	JUC06001082.hg.1	ZKSCAN3
TC06000283.hg.1	2.16	PSR06002361.hg.1	ZKSCAN3
TC06000283.hg.1	2.08	PSR06002363.hg.1	ZKSCAN3
TC06000283.hg.1	2.03	JUC06001086.hg.1	ZKSCAN3
TC06000283.hg.1	-2.44	JUC06001078.hg.1	ZKSCAN3
TC06001014.hg.1	2.25	JUC06005809.hg.1	LINC00271
TC06001014.hg.1	2.18	JUC06005815.hg.1	LINC00271
TC06001014.hg.1	-2.67	PSR06011929.hg.1	LINC00271
TC08001729.hg.1	2.25	PSR08022806.hg.1	FAM83H
TC08001729.hg.1	-2.4	JUC08011720.hg.1	FAM83H
TC10000314.hg.1	2.25	JUC10002090.hg.1	ANXA8L1, AN
TC10000314.hg.1	2.06	JUC10002092.hg.1	ANXA8L1, AN
TC10000314.hg.1	-2.09	JUC10002099.hg.1	ANXA8L1, AN
TC10000314.hg.1	-2.17	JUC10002084.hg.1	ANXA8L1, AN
TC10000314.hg.1	-2.24	JUC10002089.hg.1	ANXA8L1, AN
TC10000314.hg.1	-2.77	PSR10003589.hg.1	ANXA8L1, AN
TC10000314.hg.1	-4.29	PSR10003616.hg.1	ANXA8L1, AN
TC10000314.hg.1	-6.02	JUC10002097.hg.1	ANXA8L1, AN
TC10000559.hg.1	2.25	PSR10006022.hg.1	LOC1001329
TC10000559.hg.1	-2.39	JUC10003487.hg.1	LOC1001329
TC10001257.hg.1	2.25	PSR10015657.hg.1	FAM25C, FAM
TC10001257.hg.1	2.01	JUC10009074.hg.1	FAM25C, FAM
TC11000734.hg.1	2.25	JUC11004972.hg.1	NADSYN1
TC11000734.hg.1	2.17	PSR11009597.hg.1	NADSYN1
TC12003291.hg.1	2.25	PSR12003125.hg.1	SSPN
TC12003291.hg.1	2.22	JUC12020583.hg.1	SSPN
TC12003291.hg.1	2.17	PSR12003122.hg.1	SSPN
TC12003291.hg.1	-2.06	JUC12020587.hg.1	SSPN
TC12003291.hg.1	-2.1	PSR12003119.hg.1	SSPN
TC13000876.hg.1	2.25	JUC13005783.hg.1	CARS2

TC13000876.hg.1	2.16	PSR13009812.hg.1	CARS2
TC6_qbl_hap6000	2.25	PSR6_qbl_hap60021	GABBR1
TC6_qbl_hap6000	-2.19	JUC6_qbl_hap60020	GABBR1
TC01000253.hg.1	2.24	PSR01003968.hg.1	OTUD3
TC01000253.hg.1	2.02	JUC01002127.hg.1	OTUD3
TC01000475.hg.1	2.24	PSR01007378.hg.1	DNALI1
TC01000475.hg.1	-2.06	JUC01003833.hg.1	DNALI1
TC01000475.hg.1	-2.57	JUC01003839.hg.1	DNALI1
TC01003903.hg.1	2.24	JUC01031194.hg.1	CDC42BPA
TC01003903.hg.1	-2.07	JUC01031165.hg.1	CDC42BPA
TC01003903.hg.1	-2.09	JUC01031182.hg.1	CDC42BPA
TC01003903.hg.1	-2.09	JUC01031201.hg.1	CDC42BPA
TC01003903.hg.1	-2.63	PSR01059520.hg.1	CDC42BPA
TC01003903.hg.1	-3.01	JUC01031181.hg.1	CDC42BPA
TC02002698.hg.1	2.24	PSR02043501.hg.1	GPR1
TC02002698.hg.1	2.18	PSR02043499.hg.1	GPR1
TC02002698.hg.1	2.1	PSR02043502.hg.1	GPR1
TC02002698.hg.1	-3.48	PSR02043511.hg.1	GPR1
TC02002698.hg.1	-3.62	JUC02022794.hg.1	GPR1
TC02002698.hg.1	-5.29	PSR02043513.hg.1	GPR1
TC02002698.hg.1	-7.49	JUC02022793.hg.1	GPR1
TC05002169.hg.1	2.24	PSR05030493.hg.1	GNB2L1, SN
TC05002169.hg.1	-2	JUC05015448.hg.1	GNB2L1, SN
TC05002169.hg.1	-2.2	JUC05015433.hg.1	GNB2L1, SN
TC06000931.hg.1	2.24	JUC06005201.hg.1	DCBLD1
TC06000931.hg.1	-2.04	JUC06005197.hg.1	DCBLD1
TC06000931.hg.1	-2.07	JUC06005196.hg.1	DCBLD1
TC06000931.hg.1	-2.1	JUC06005186.hg.1	DCBLD1
TC06000931.hg.1	-2.32	PSR06010879.hg.1	DCBLD1
TC06000931.hg.1	-2.39	PSR06010906.hg.1	DCBLD1
TC06000931.hg.1	-4.15	JUC06005198.hg.1	DCBLD1
TC07000868.hg.1	2.24	JUC07006471.hg.1	TRIM24
TC07000868.hg.1	-2.02	PSR07013507.hg.1	TRIM24
TC07000868.hg.1	-4.23	JUC07006456.hg.1	TRIM24
TC08000660.hg.1	2.24	JUC08004550.hg.1	OXR1
TC08000660.hg.1	-2	JUC08004549.hg.1	OXR1
TC08000660.hg.1	-2.17	JUC08004555.hg.1	OXR1
TC08000660.hg.1	-2.32	PSR08008996.hg.1	OXR1
TC08001590.hg.1	2.24	PSR08020813.hg.1	FBXO32
TC08001590.hg.1	-2.71	JUC08010630.hg.1	FBXO32
TC08001590.hg.1	-2.94	JUC08010636.hg.1	FBXO32
TC08001590.hg.1	-4.46	PSR08020820.hg.1	FBXO32
TC09002886.hg.1	2.24	PSR09010770.hg.1	MAMDC4
TC09002886.hg.1	2.23	JUC09015621.hg.1	MAMDC4
TC09002886.hg.1	-2.1	JUC09015617.hg.1	MAMDC4
TC0X000572.hg.1	2.24	JUC0X003745.hg.1	WDR44
TC0X000572.hg.1	2.17	PSR0X007529.hg.1	WDR44
TC0X000572.hg.1	-2.01	JUC0X003761.hg.1	WDR44
TC0X001124.hg.1	2.24	PSR0X015305.hg.1	ZMYM3
TC0X001124.hg.1	-3.78	JUC0X007762.hg.1	ZMYM3
TC0X001156.hg.1	2.24	JUC0X007901.hg.1	FTX
TC0X001156.hg.1	2.04	JUC0X007894.hg.1	FTX
TC0X001156.hg.1	-2.09	PSR0X015648.hg.1	FTX
TC11000026.hg.1	2.24	PSR11000383.hg.1	EPS8L2
TC11000026.hg.1	2.19	JUC11000214.hg.1	EPS8L2
TC11000240.hg.1	2.24	JUC11001573.hg.1	LDHA
TC11000240.hg.1	2	PSR11003174.hg.1	LDHA
TC11000240.hg.1	-2.17	PSR11003210.hg.1	LDHA

TC12000508.hg.1	2.24	PSR12006693.hg.1	SLC39A5
TC12000508.hg.1	-2.05	PSR12006689.hg.1	SLC39A5
TC12000508.hg.1	-2.11	JUC12003467.hg.1	SLC39A5
TC14000426.hg.1	2.24	JUC14002623.hg.1	TTC9
TC14000426.hg.1	2.11	PSR14005132.hg.1	TTC9
TC15002761.hg.1	2.24	PSR15001164.hg.1	LOC1002886[illegible]
TC15002761.hg.1	2.02	JUC15013241.hg.1	LOC1002886[illegible]
TC15002761.hg.1	-2.85	JUC15013239.hg.1	LOC1002886[illegible]
TC17000088.hg.1	2.24	JUC17000704.hg.1	EIF5A
TC17000088.hg.1	2.06	PSR17001154.hg.1	EIF5A
TC01000071.hg.1	2.23	JUC01000582.hg.1	TP73
TC01000071.hg.1	-2.01	PSR01001130.hg.1	TP73
TC01002253.hg.1	2.23	PSR01034336.hg.1	ZBTB17
TC01002253.hg.1	-3.32	JUC01018398.hg.1	ZBTB17
TC02002536.hg.1	2.23	PSR02040226.hg.1	OLA1
TC02002536.hg.1	-2.36	JUC02021202.hg.1	OLA1
TC03001041.hg.1	2.23	JUC03009340.hg.1	TPRG1, LOC
TC03001041.hg.1	-2.27	PSR03018424.hg.1	TPRG1, LOC
TC03001472.hg.1	2.23	PSR03026807.hg.1	CHDH
TC03001472.hg.1	2.1	PSR03026808.hg.1	CHDH
TC03001472.hg.1	-2.06	JUC03013056.hg.1	CHDH
TC03001472.hg.1	-2.53	JUC03013057.hg.1	CHDH
TC03001472.hg.1	-2.64	JUC03013049.hg.1	CHDH
TC04000577.hg.1	2.23	JUC04004410.hg.1	C4orf32
TC04000577.hg.1	-2.28	PSR04008379.hg.1	C4orf32
TC04000806.hg.1	2.23	JUC04005971.hg.1	ETFDH
TC04000806.hg.1	2.07	JUC04005970.hg.1	ETFDH
TC04000806.hg.1	2.03	JUC04005969.hg.1	ETFDH
TC04000806.hg.1	-2.23	JUC04005967.hg.1	ETFDH
TC04000806.hg.1	-2.44	PSR04011154.hg.1	ETFDH
TC05000284.hg.1	2.23	JUC05001980.hg.1	MAST4
TC05000284.hg.1	2.07	PSR05003684.hg.1	MAST4
TC05000284.hg.1	-2.07	JUC05001961.hg.1	MAST4
TC05000284.hg.1	-2.11	PSR05003747.hg.1	MAST4
TC05000284.hg.1	-3.59	JUC05001992.hg.1	MAST4
TC05000729.hg.1	2.23	PSR05010436.hg.1	TMCO6
TC05000729.hg.1	2.08	JUC05005539.hg.1	TMCO6
TC05000729.hg.1	2.06	JUC05005532.hg.1	TMCO6
TC05000729.hg.1	-2.14	JUC05005528.hg.1	TMCO6
TC05000910.hg.1	2.23	PSR05013174.hg.1	LOC1005071[illegible]
TC05000910.hg.1	-2.55	PSR05013178.hg.1	LOC1005071[illegible]
TC05000910.hg.1	-2.75	JUC05006810.hg.1	LOC1005071[illegible]
TC05000910.hg.1	-2.75	JUC05006811.hg.1	LOC1005071[illegible]
TC06000728.hg.1	2.23	PSR06008723.hg.1	KCNQ5
TC06000728.hg.1	-2.01	JUC06003980.hg.1	KCNQ5
TC06000728.hg.1	-2.43	JUC06003968.hg.1	KCNQ5
TC06000728.hg.1	-2.74	JUC06003971.hg.1	KCNQ5
TC06000728.hg.1	-2.97	PSR06008696.hg.1	KCNQ5
TC06000728.hg.1	-3.02	JUC06003964.hg.1	KCNQ5
TC06001032.hg.1	2.23	PSR06012111.hg.1	CCDC28A
TC06001032.hg.1	-3.13	JUC06005919.hg.1	CCDC28A
TC12000468.hg.1	2.23	JUC12003291.hg.1	PDE1B
TC12000468.hg.1	2.17	PSR12006089.hg.1	PDE1B
TC14001030.hg.1	2.23	JUC14005880.hg.1	BAZ1A
TC14001030.hg.1	-2.03	PSR14011781.hg.1	BAZ1A
TC14001030.hg.1	-2.25	JUC14005896.hg.1	BAZ1A
TC14001030.hg.1	-3.16	JUC14005884.hg.1	BAZ1A
TC14001032.hg.1	2.23	PSR14011807.hg.1	LOC1005061[illegible]

TC14001032.hg.1	2	JUC14005904.hg.1	LOC1005061
TC15001362.hg.1	2.23	JUC15007062.hg.1	CYP19A1
TC15001362.hg.1	-2.08	PSR15013179.hg.1	CYP19A1
TC15001362.hg.1	-3.14	PSR15013171.hg.1	CYP19A1
TC15001362.hg.1	-3.16	PSR15013161.hg.1	CYP19A1
TC16000664.hg.1	2.23	PSR16009380.hg.1	KIAA0182
TC16000664.hg.1	2.21	JUC16005248.hg.1	KIAA0182
TC16000664.hg.1	2.14	PSR16009393.hg.1	KIAA0182
TC16000664.hg.1	-2.01	JUC16005256.hg.1	KIAA0182
TC16000664.hg.1	-2.92	JUC16005261.hg.1	KIAA0182
TC16000664.hg.1	-4.13	JUC16005251.hg.1	KIAA0182
TC19000142.hg.1	2.23	JUC19001355.hg.1	SNAPC2
TC19000142.hg.1	2.14	PSR19002256.hg.1	SNAPC2
TC20000206.hg.1	2.23	PSR20002501.hg.1	HCK
TC20000206.hg.1	-2.31	JUC20001354.hg.1	HCK
TC6_qbl_hap6000	2.23	PSR6_qbl_hap60034	PBX2
TC6_qbl_hap6000	2.05	JUC6_qbl_hap60013	PBX2
TC21001066.hg.1	2.23	JUC21005228.hg.1	C21orf33
TC21001066.hg.1	2	PSR21002557.hg.1	C21orf33
TC21001066.hg.1	-2.89	JUC21005239.hg.1	C21orf33
TC01000836.hg.1	2.22	JUC01007005.hg.1	GBP1P1
TC01000836.hg.1	2.13	PSR01013300.hg.1	GBP1P1
TC01000836.hg.1	2.06	JUC01007007.hg.1	GBP1P1
TC01002774.hg.1	2.22	PSR01043312.hg.1	PTGER3
TC01002774.hg.1	-2.02	JUC01023176.hg.1	PTGER3
TC01002774.hg.1	-2.08	PSR01043299.hg.1	PTGER3
TC01002774.hg.1	-2.23	PSR01043295.hg.1	PTGER3
TC01002774.hg.1	-2.33	PSR01043306.hg.1	PTGER3
TC01002774.hg.1	-2.48	PSR01043316.hg.1	PTGER3
TC01002774.hg.1	-2.68	PSR01043304.hg.1	PTGER3
TC01002774.hg.1	-2.68	PSR01043317.hg.1	PTGER3
TC01002774.hg.1	-2.89	PSR01043301.hg.1	PTGER3
TC01002774.hg.1	-3.2	PSR01043307.hg.1	PTGER3
TC01002774.hg.1	-3.3	JUC01023158.hg.1	PTGER3
TC01002774.hg.1	-3.33	PSR01043297.hg.1	PTGER3
TC01002774.hg.1	-3.61	PSR01043302.hg.1	PTGER3
TC01002774.hg.1	-3.79	JUC01023169.hg.1	PTGER3
TC01002774.hg.1	-3.89	JUC01023163.hg.1	PTGER3
TC01002774.hg.1	-3.92	JUC01023160.hg.1	PTGER3
TC01002774.hg.1	-4.08	JUC01023168.hg.1	PTGER3
TC01006286.hg.1	2.22	JUC01039238.hg.1	GSTM2
TC01006286.hg.1	2.03	PSR01015116.hg.1	GSTM2
TC01006286.hg.1	2	PSR01015117.hg.1	GSTM2
TC03001216.hg.1	2.22	PSR03020916.hg.1	RFTN1
TC03001216.hg.1	2.08	PSR03020903.hg.1	RFTN1
TC03001216.hg.1	2.01	PSR03020906.hg.1	RFTN1
TC03001216.hg.1	-2.82	JUC03010430.hg.1	RFTN1
TC04000274.hg.1	2.22	JUC04001964.hg.1	ATP10D
TC04000274.hg.1	-2.03	PSR04003968.hg.1	ATP10D
TC04000274.hg.1	-2.04	PSR04003963.hg.1	ATP10D
TC04000274.hg.1	-2.56	PSR04003951.hg.1	ATP10D
TC07000112.hg.1	2.22	PSR07001722.hg.1	AHR
TC07000112.hg.1	-2.63	JUC07000786.hg.1	AHR
TC08002585.hg.1	2.22	JUC08014746.hg.1	COPS5
TC08002585.hg.1	-3.56	PSR08016646.hg.1	COPS5
TC0X000190.hg.1	2.22	JUC0X001130.hg.1	ATP6AP2
TC0X000190.hg.1	-2.97	PSR0X002121.hg.1	ATP6AP2
TC0X000772.hg.1	2.22	JUC0X005153.hg.1	GDI1

TC0X000772.hg.1	2.18	PSR0X010387.hg.1	GDI1
TC0X000772.hg.1	2.03	PSR0X010384.hg.1	GDI1
TC12000765.hg.1	2.22	JUC12005158.hg.1	APAF1
TC12000765.hg.1	-2.27	JUC12005163.hg.1	APAF1
TC12000765.hg.1	-3.03	PSR12009644.hg.1	APAF1
TC16000538.hg.1	2.22	JUC16004273.hg.1	PLEKHG4
TC16000538.hg.1	-2	PSR16007645.hg.1	PLEKHG4
TC16000538.hg.1	-3.32	PSR16007637.hg.1	PLEKHG4
TC16000538.hg.1	-4.66	JUC16004281.hg.1	PLEKHG4
TC17001646.hg.1	2.22	PSR17022058.hg.1	HOXB8
TC17001646.hg.1	2.02	JUC17012328.hg.1	HOXB8
TC17001646.hg.1	-2.23	PSR17022070.hg.1	HOXB8
TC17001646.hg.1	-2.41	PSR17022071.hg.1	HOXB8
TC17001922.hg.1	2.22	PSR17026143.hg.1	USP36
TC17001922.hg.1	-2.05	JUC17014728.hg.1	USP36
TC17002909.hg.1	2.22	JUC17019102.hg.1	MIR10A, HO)
TC17002909.hg.1	2.03	JUC17019107.hg.1	MIR10A, HO)
TC17002909.hg.1	-2.09	PSR17021989.hg.1	MIR10A, HO)
TC17002909.hg.1	-2.36	PSR17022000.hg.1	MIR10A, HO)
TC19001753.hg.1	2.22	JUC19013576.hg.1	
TC19001753.hg.1	-2.09	PSR19023883.hg.1	
TC20000565.hg.1	2.22	JUC20004181.hg.1	CPXM1
TC20000565.hg.1	-2.09	PSR20008184.hg.1	CPXM1
TC21000261.hg.1	2.22	PSR21003236.hg.1	DIP2A
TC21000261.hg.1	2.16	PSR21003293.hg.1	DIP2A
TC21000261.hg.1	2.15	JUC21001751.hg.1	DIP2A
TC21000261.hg.1	-2.38	JUC21001760.hg.1	DIP2A
TC6_mann_hap40(	2.22	PSR6_mann_hap400	GABBR1
TC6_mann_hap40(	-2.23	JUC6_mann_hap400	GABBR1
TC16000979.hg.1	2.22	JUC16007935.hg.1	GTF3C1
TC16000979.hg.1	2	PSR16014033.hg.1	GTF3C1
TC16000979.hg.1	-2.53	JUC16007910.hg.1	GTF3C1
TC01006428.hg.1	2.21	PSR01016782.hg.1	
TC01006428.hg.1	2.04	PSR01016845.hg.1	
TC01006428.hg.1	2.01	PSR01016865.hg.1	
TC01006428.hg.1	-2.01	JUC01043184.hg.1	
TC02000149.hg.1	2.21	JUC02000913.hg.1	HADHB
TC02000149.hg.1	-2.14	PSR02001727.hg.1	HADHB
TC02001020.hg.1	2.21	JUC02008060.hg.1	DCAF17
TC02001020.hg.1	2.09	PSR02015090.hg.1	DCAF17
TC02002773.hg.1	2.21	PSR02044621.hg.1	USP37
TC02002773.hg.1	2.13	PSR02044605.hg.1	USP37
TC02002773.hg.1	2.09	PSR02044584.hg.1	USP37
TC02002773.hg.1	-2.26	JUC02023382.hg.1	USP37
TC03003323.hg.1	2.21	JUC03023106.hg.1	NCK1
TC03003323.hg.1	2.19	PSR03013350.hg.1	NCK1
TC04001430.hg.1	2.21	PSR04020011.hg.1	MANBA
TC04001430.hg.1	2.03	PSR04020007.hg.1	MANBA
TC04001430.hg.1	-2.19	JUC04010486.hg.1	MANBA
TC05000478.hg.1	2.21	JUC05003752.hg.1	RGMB
TC05000478.hg.1	2.03	JUC05003744.hg.1	RGMB
TC05000478.hg.1	-2.24	JUC05003749.hg.1	RGMB
TC05000478.hg.1	-2.28	PSR05006829.hg.1	RGMB
TC05000478.hg.1	-2.3	PSR05006831.hg.1	RGMB
TC05000478.hg.1	-2.45	PSR05006832.hg.1	RGMB
TC05000478.hg.1	-2.61	PSR05006835.hg.1	RGMB
TC05000478.hg.1	-2.75	JUC05003747.hg.1	RGMB
TC05000478.hg.1	-23.99	PSR05006825.hg.1	RGMB

TC05000607.hg.1	2.21	JUC05004558.hg.1	MEGF10
TC05000607.hg.1	2.19	JUC05004563.hg.1	MEGF10
TC05000607.hg.1	2.16	JUC05004545.hg.1	MEGF10
TC05000607.hg.1	2.04	PSR05008408.hg.1	MEGF10
TC05000607.hg.1	-2.15	PSR05008435.hg.1	MEGF10
TC05000607.hg.1	-4.4	JUC05004553.hg.1	MEGF10
TC05001331.hg.1	2.21	JUC05009656.hg.1	EMB
TC05001331.hg.1	-2.37	PSR05018797.hg.1	EMB
TC05001331.hg.1	-2.41	JUC05009652.hg.1	EMB
TC06002136.hg.1	2.21	PSR06026911.hg.1	AHI1
TC06002136.hg.1	2.06	PSR06026883.hg.1	AHI1
TC06002136.hg.1	2.03	JUC06013344.hg.1	AHI1
TC07000577.hg.1	2.21	PSR07008445.hg.1	DLX6
TC07000577.hg.1	-2.25	PSR07008447.hg.1	DLX6
TC07000577.hg.1	-2.71	PSR07008430.hg.1	DLX6
TC07000577.hg.1	-2.74	PSR07008428.hg.1	DLX6
TC07000577.hg.1	-2.96	JUC07004051.hg.1	DLX6
TC07000577.hg.1	-3.79	JUC07004052.hg.1	DLX6
TC08000127.hg.1	2.21	JUC08000645.hg.1	SLC7A2
TC08000127.hg.1	-3.48	JUC08000647.hg.1	SLC7A2
TC08000127.hg.1	-3.97	JUC08000648.hg.1	SLC7A2
TC08000127.hg.1	-4.11	JUC08000644.hg.1	SLC7A2
TC08000127.hg.1	-4.46	PSR08001210.hg.1	SLC7A2
TC08000127.hg.1	-5.61	PSR08001198.hg.1	SLC7A2
TC08000127.hg.1	-8.5	PSR08001201.hg.1	SLC7A2
TC09001475.hg.1	2.21	JUC09009859.hg.1	SVEP1
TC09001475.hg.1	-2.15	JUC09009851.hg.1	SVEP1
TC09001475.hg.1	-2.4	PSR09018231.hg.1	SVEP1
TC09001475.hg.1	-2.47	PSR09018271.hg.1	SVEP1
TC09001475.hg.1	-2.56	JUC09009885.hg.1	SVEP1
TC09001475.hg.1	-3.23	PSR09018290.hg.1	SVEP1
TC09001475.hg.1	-3.28	JUC09009891.hg.1	SVEP1
TC09001475.hg.1	-3.83	PSR09018255.hg.1	SVEP1
TC09001475.hg.1	-4.02	JUC09009852.hg.1	SVEP1
TC09001475.hg.1	-4.75	PSR09018246.hg.1	SVEP1
TC09001475.hg.1	-6.15	JUC09009896.hg.1	SVEP1
TC09001604.hg.1	2.21	PSR09020110.hg.1	FAM129B
TC09001604.hg.1	2.13	JUC09010899.hg.1	FAM129B
TC09001604.hg.1	2.07	PSR09020115.hg.1	FAM129B
TC09001604.hg.1	2.07	PSR09020133.hg.1	FAM129B
TC09001604.hg.1	-2.11	JUC09010901.hg.1	FAM129B
TC0X001311.hg.1	2.21	JUC0X008964.hg.1	NKRF
TC0X001311.hg.1	-2.06	PSR0X017600.hg.1	NKRF
TC10000753.hg.1	2.21	JUC10004981.hg.1	NFKB2
TC10000753.hg.1	-2.07	PSR10008908.hg.1	NFKB2
TC10000753.hg.1	-2.34	JUC10004970.hg.1	NFKB2
TC10000753.hg.1	-2.35	JUC10004961.hg.1	NFKB2
TC10000753.hg.1	-2.4	JUC10004974.hg.1	NFKB2
TC10000753.hg.1	-2.53	PSR10008917.hg.1	NFKB2
TC10000753.hg.1	-4.23	JUC10004976.hg.1	NFKB2
TC10000753.hg.1	-5.71	JUC10004980.hg.1	NFKB2
TC10001004.hg.1	2.21	JUC10007243.hg.1	ANKRD16
TC10001004.hg.1	-2.99	JUC10007245.hg.1	ANKRD16
TC10001004.hg.1	-5.08	PSR10012504.hg.1	ANKRD16
TC10001004.hg.1	-9.1	PSR10012505.hg.1	ANKRD16
TC12002076.hg.1	2.21	PSR12027360.hg.1	RSRC2
TC12002076.hg.1	-2.02	JUC12015328.hg.1	RSRC2
TC14000780.hg.1	2.21	PSR14008440.hg.1	ZNF839

TC14000780.hg.1	2.08	PSR14008443.hg.1	ZNF839
TC14000780.hg.1	-2.01	PSR14008432.hg.1	ZNF839
TC14000780.hg.1	-2.38	JUC14004282.hg.1	ZNF839
TC17000631.hg.1	2.21	JUC17004369.hg.1	CALCOCO2
TC17000631.hg.1	-2.03	PSR17007869.hg.1	CALCOCO2
TC17000631.hg.1	-2.05	PSR17007868.hg.1	CALCOCO2
TC17000631.hg.1	-2.05	JUC17004356.hg.1	CALCOCO2
TC17000631.hg.1	-2.12	JUC17004372.hg.1	CALCOCO2
TC17000631.hg.1	-2.14	PSR17007870.hg.1	CALCOCO2
TC17000631.hg.1	-2.27	PSR17007833.hg.1	CALCOCO2
TC17000631.hg.1	-2.3	PSR17007865.hg.1	CALCOCO2
TC17000631.hg.1	-2.38	JUC17004358.hg.1	CALCOCO2
TC17000631.hg.1	-2.66	PSR17007845.hg.1	CALCOCO2
TC17000631.hg.1	-4.57	JUC17004371.hg.1	CALCOCO2
TC20001729.hg.1	2.21	JUC20009765.hg.1	TGIF2
TC20001729.hg.1	2.16	PSR20003741.hg.1	TGIF2
TC20001729.hg.1	-2.62	JUC20009767.hg.1	TGIF2
TC22000116.hg.1	2.21	PSR22002197.hg.1	GGTLC2
TC22000116.hg.1	-4.21	JUC22000636.hg.1	GGTLC2
TC01003867.hg.1	2.2	JUC01030817.hg.1	TAF1A
TC01003867.hg.1	-2.03	PSR01058822.hg.1	TAF1A
TC01003867.hg.1	-2.26	JUC01030823.hg.1	TAF1A
TC02000708.hg.1	2.2	JUC02005824.hg.1	CHCHD5
TC02000708.hg.1	2.14	PSR02011006.hg.1	CHCHD5
TC02002770.hg.1	2.2	PSR02044547.hg.1	TMBIM1
TC02002770.hg.1	2.06	PSR02044534.hg.1	TMBIM1
TC02002770.hg.1	2.06	JUC02023352.hg.1	TMBIM1
TC02002770.hg.1	2.01	PSR02044546.hg.1	TMBIM1
TC05000340.hg.1	2.2	JUC05002505.hg.1	TNPO1
TC05000340.hg.1	-2.88	PSR05004644.hg.1	TNPO1
TC06000029.hg.1	2.2	JUC06000107.hg.1	PSMG4
TC06000029.hg.1	2.01	JUC06000099.hg.1	PSMG4
TC06000029.hg.1	2	JUC06000108.hg.1	PSMG4
TC06000029.hg.1	-2.66	PSR06000249.hg.1	PSMG4
TC06001593.hg.1	2.2	PSR06019400.hg.1	LEMD2
TC06001593.hg.1	-2.24	JUC06009238.hg.1	LEMD2
TC07000451.hg.1	2.2	PSR07006110.hg.1	EIF4H
TC07000451.hg.1	2.1	JUC07002959.hg.1	EIF4H
TC07000451.hg.1	2.03	JUC07002956.hg.1	EIF4H
TC07000451.hg.1	-2.07	PSR07006109.hg.1	EIF4H
TC08001228.hg.1	2.2	JUC08008210.hg.1	RPS20, SNOI
TC08001228.hg.1	2.11	PSR08015926.hg.1	RPS20, SNOI
TC09000509.hg.1	2.2	JUC09003085.hg.1	STX17
TC09000509.hg.1	2.08	PSR09005541.hg.1	STX17
TC09000509.hg.1	-2	JUC09003078.hg.1	STX17
TC09000509.hg.1	-2.55	JUC09003087.hg.1	STX17
TC10001320.hg.1	2.2	JUC10009728.hg.1	RHOBTB1
TC10001320.hg.1	-2.03	PSR10016690.hg.1	RHOBTB1
TC10001320.hg.1	-2.15	PSR10016691.hg.1	RHOBTB1
TC10001320.hg.1	-2.24	PSR10016688.hg.1	RHOBTB1
TC10001320.hg.1	-2.6	PSR10016684.hg.1	RHOBTB1
TC10001320.hg.1	-2.78	PSR10016677.hg.1	RHOBTB1
TC10001320.hg.1	-3.14	PSR10016695.hg.1	RHOBTB1
TC10001320.hg.1	-3.32	PSR10016692.hg.1	RHOBTB1
TC10001320.hg.1	-3.46	PSR10016672.hg.1	RHOBTB1
TC10001320.hg.1	-4.28	PSR10016687.hg.1	RHOBTB1
TC10001320.hg.1	-4.91	JUC10009736.hg.1	RHOBTB1
TC11001407.hg.1	2.2	PSR11017742.hg.1	SBF2

TC11001407.hg.1	2.01	JUC11009456.hg.1	SBF2
TC11001407.hg.1	-2.35	JUC11009485.hg.1	SBF2
TC11002307.hg.1	2.2	JUC11014706.hg.1	USP28
TC11002307.hg.1	2.08	PSR11027643.hg.1	USP28
TC11002307.hg.1	2.05	JUC11014725.hg.1	USP28
TC11002307.hg.1	-2.28	JUC11014714.hg.1	USP28
TC12000936.hg.1	2.2	JUC12006689.hg.1	PRKAB1
TC12000936.hg.1	-2.02	PSR12012140.hg.1	PRKAB1
TC12003246.hg.1	2.2	PSR12006570.hg.1	MYL6B
TC12003246.hg.1	-2.75	JUC12020027.hg.1	MYL6B
TC15002805.hg.1	2.2	JUC15014111.hg.1	ULK4P1
TC15002805.hg.1	-2.39	PSR15010387.hg.1	ULK4P1
TC15002805.hg.1	-2.44	JUC15014107.hg.1	ULK4P1
TC15002805.hg.1	-2.97	PSR15010388.hg.1	ULK4P1
TC17001302.hg.1	2.2	PSR17017308.hg.1	KIAA0100
TC17001302.hg.1	-2.1	JUC17009783.hg.1	KIAA0100
TC17001305.hg.1	2.2	PSR17017441.hg.1	RAB34, NARI
TC17001305.hg.1	2.06	PSR17017401.hg.1	RAB34, NARI
TC17001305.hg.1	2.04	JUC17009848.hg.1	RAB34, NARI
TC17001305.hg.1	2.01	PSR17017404.hg.1	RAB34, NARI
TC19000616.hg.1	2.2	PSR19008536.hg.1	ZNF224
TC19000616.hg.1	-2.04	PSR19008538.hg.1	ZNF224
TC19000616.hg.1	-4.73	JUC19005101.hg.1	ZNF224
TC21000260.hg.1	2.2	JUC21001736.hg.1	PCNT
TC21000260.hg.1	-2.25	JUC21001682.hg.1	PCNT
TC21000260.hg.1	-2.88	PSR21003178.hg.1	PCNT
TC22000471.hg.1	2.2	JUC22003340.hg.1	CECR5
TC22000471.hg.1	-2.12	PSR22008418.hg.1	CECR5
TC22000471.hg.1	-2.27	PSR22008422.hg.1	CECR5
TC6_apd_hap1000	2.2	PSR6_apd_hap1001	MDC1
TC6_apd_hap1000	2.02	PSR6_apd_hap1001	MDC1
TC6_apd_hap1000	-2.87	JUC6_apd_hap1000	MDC1
TC6_cox_hap2000	2.2	PSR6_cox_hap2002	MDC1
TC6_cox_hap2000	2.02	PSR6_cox_hap2002	MDC1
TC6_cox_hap2000	-2.87	JUC6_cox_hap2000	MDC1
TC6_dbb_hap3000	2.2	PSR6_dbb_hap3002	MDC1
TC6_dbb_hap3000	2.02	PSR6_dbb_hap3002	MDC1
TC6_dbb_hap3000	-2.87	JUC6_dbb_hap3000	MDC1
TC6_mann_hap40(	2.2	PSR6_mann_hap40(	MDC1
TC6_mann_hap40(	2.02	PSR6_mann_hap40(	MDC1
TC6_mann_hap40(	-2.87	JUC6_mann_hap400	MDC1
TC6_mcf_hap5000	2.2	PSR6_mcf_hap5002	MDC1
TC6_mcf_hap5000	2.02	PSR6_mcf_hap5002	MDC1
TC6_mcf_hap5000	-2.87	JUC6_mcf_hap5000	MDC1
TC6_ssto_hap700(	2.2	PSR6_ssto_hap7002	MDC1
TC6_ssto_hap700(	2.02	PSR6_ssto_hap7002	MDC1
TC6_ssto_hap700(	-2.87	JUC6_ssto_hap7000	MDC1
TC20001739.hg.1	2.2	JUC20009880.hg.1	LINC00266-1
TC20001739.hg.1	2	PSR20007730.hg.1	LINC00266-1
TC01000046.hg.1	2.19	PSR01000653.hg.1	MIB2
TC01000046.hg.1	2.14	JUC01000318.hg.1	MIB2
TC01000046.hg.1	2.1	JUC01000334.hg.1	MIB2
TC01000046.hg.1	-2.15	PSR01000603.hg.1	MIB2
TC01000046.hg.1	-2.88	PSR01000600.hg.1	MIB2
TC01000707.hg.1	2.19	PSR01011313.hg.1	USP1
TC01000707.hg.1	2.05	PSR01011312.hg.1	USP1
TC01000707.hg.1	-2.02	JUC01005847.hg.1	USP1
TC01002847.hg.1	2.19	JUC01023836.hg.1	GBP1

TC01002847.hg.1	-2.2	PSR01044368.hg.1	GBP1
TC01002847.hg.1	-2.47	PSR01044378.hg.1	GBP1
TC01002847.hg.1	-4.03	JUC01023826.hg.1	GBP1
TC01002860.hg.1	2.19	PSR01044516.hg.1	LOC149351
TC01002860.hg.1	2.09	JUC01023879.hg.1	LOC149351
TC02000862.hg.1	2.19	JUC02006810.hg.1	C2orf27A
TC02000862.hg.1	-2.16	PSR02012847.hg.1	C2orf27A
TC02000862.hg.1	-2.69	PSR02012853.hg.1	C2orf27A
TC02000862.hg.1	-4.73	JUC02006811.hg.1	C2orf27A
TC02000862.hg.1	-7.46	JUC02006813.hg.1	C2orf27A
TC02001168.hg.1	2.19	PSR02017749.hg.1	NIF3L1
TC02001168.hg.1	2.1	JUC02009482.hg.1	NIF3L1
TC02002278.hg.1	2.19	JUC02018975.hg.1	ERCC3
TC02002278.hg.1	2.11	PSR02036608.hg.1	ERCC3
TC02002278.hg.1	-2.01	PSR02036614.hg.1	ERCC3
TC02002284.hg.1	2.19	JUC02019048.hg.1	LIMS2
TC02002284.hg.1	2.15	JUC02019051.hg.1	LIMS2
TC02002284.hg.1	-2.27	PSR02036755.hg.1	LIMS2
TC02002284.hg.1	-2.37	PSR02036708.hg.1	LIMS2
TC02002284.hg.1	-2.38	PSR02036753.hg.1	LIMS2
TC02002284.hg.1	-2.49	PSR02036740.hg.1	LIMS2
TC02002284.hg.1	-2.58	PSR02036758.hg.1	LIMS2
TC02002284.hg.1	-2.58	PSR02036759.hg.1	LIMS2
TC02002284.hg.1	-2.64	PSR02036734.hg.1	LIMS2
TC02002284.hg.1	-2.64	PSR02036754.hg.1	LIMS2
TC02002284.hg.1	-2.69	PSR02036723.hg.1	LIMS2
TC02002284.hg.1	-2.78	PSR02036722.hg.1	LIMS2
TC02002284.hg.1	-2.94	PSR02036729.hg.1	LIMS2
TC02002284.hg.1	-2.98	PSR02036726.hg.1	LIMS2
TC02002284.hg.1	-3.01	PSR02036761.hg.1	LIMS2
TC02002284.hg.1	-3.05	PSR02036748.hg.1	LIMS2
TC02002284.hg.1	-3.15	PSR02036751.hg.1	LIMS2
TC02002284.hg.1	-3.2	JUC02019047.hg.1	LIMS2
TC02002284.hg.1	-3.22	PSR02036752.hg.1	LIMS2
TC02002284.hg.1	-3.29	PSR02036750.hg.1	LIMS2
TC02002284.hg.1	-3.36	PSR02036744.hg.1	LIMS2
TC02002284.hg.1	-3.51	PSR02036749.hg.1	LIMS2
TC02002284.hg.1	-3.59	PSR02036735.hg.1	LIMS2
TC02002284.hg.1	-3.61	PSR02036725.hg.1	LIMS2
TC02002284.hg.1	-3.67	PSR02036738.hg.1	LIMS2
TC02002284.hg.1	-4.03	PSR02036720.hg.1	LIMS2
TC02002284.hg.1	-5.11	PSR02036730.hg.1	LIMS2
TC03000715.hg.1	2.19	PSR03012864.hg.1	DNAJC13
TC03000715.hg.1	2.05	JUC03006491.hg.1	DNAJC13
TC03000715.hg.1	-2.55	JUC03006471.hg.1	DNAJC13
TC03000715.hg.1	-3.08	JUC03006515.hg.1	DNAJC13
TC04001220.hg.1	2.19	PSR04016690.hg.1	PPAT
TC04001220.hg.1	-2.08	JUC04008899.hg.1	PPAT
TC04001220.hg.1	-2.53	PSR04016682.hg.1	PPAT
TC04001220.hg.1	-2.91	JUC04008897.hg.1	PPAT
TC06000423.hg.1	2.19	PSR06004995.hg.1	PHF1
TC06000423.hg.1	-2.38	JUC06001944.hg.1	PHF1
TC06001552.hg.1	2.19	JUC06008747.hg.1	MIR1236, RD
TC06001552.hg.1	2.06	PSR06018097.hg.1	MIR1236, RD
TC06001668.hg.1	2.19	PSR06019824.hg.1	SRPK1
TC06001668.hg.1	2.15	JUC06009377.hg.1	SRPK1
TC06001668.hg.1	2.03	PSR06019796.hg.1	SRPK1
TC07001695.hg.1	2.19	JUC07012866.hg.1	MYL10

TC07001695.hg.1	2.07	PSR07026204.hg.1	MYL10
TC07001695.hg.1	-2.04	PSR07026205.hg.1	MYL10
TC07001695.hg.1	-2.37	JUC07012869.hg.1	MYL10
TC09000247.hg.1	2.19	JUC09001445.hg.1	CBWD7
TC09000247.hg.1	2.1	JUC09001433.hg.1	CBWD7
TC09000247.hg.1	-2.06	PSR09002778.hg.1	CBWD7
TC09000247.hg.1	-2.24	JUC09001432.hg.1	CBWD7
TC09000247.hg.1	-2.26	PSR09002774.hg.1	CBWD7
TC09000247.hg.1	-2.93	PSR09002796.hg.1	CBWD7
TC09001628.hg.1	2.19	PSR09020681.hg.1	GOLGA2
TC09001628.hg.1	-2.52	JUC09011096.hg.1	GOLGA2
TC0X001061.hg.1	2.19	JUC0X007162.hg.1	KDM5C
TC0X001061.hg.1	2.16	JUC0X007140.hg.1	KDM5C
TC0X001061.hg.1	2.07	PSR0X014306.hg.1	KDM5C
TC10002930.hg.1	2.19	PSR10020458.hg.1	ERLIN1
TC10002930.hg.1	-2.66	JUC10017067.hg.1	ERLIN1
TC12000840.hg.1	2.19	PSR12010744.hg.1	DAO
TC12000840.hg.1	2.01	JUC12005913.hg.1	DAO
TC12001082.hg.1	2.19	PSR12014296.hg.1	KDM5A
TC12001082.hg.1	2.06	JUC12007866.hg.1	KDM5A
TC12001082.hg.1	2	JUC12007858.hg.1	KDM5A
TC12001082.hg.1	-2.18	JUC12007842.hg.1	KDM5A
TC14001350.hg.1	2.19	JUC14007819.hg.1	SNW1
TC14001350.hg.1	2.03	PSR14015385.hg.1	SNW1
TC14001350.hg.1	-2.03	JUC14007825.hg.1	SNW1
TC16000524.hg.1	2.19	PSR16007303.hg.1	CES2
TC16000524.hg.1	-3.58	JUC16004120.hg.1	CES2
TC17001923.hg.1	2.19	JUC17014757.hg.1	LOC1006535
TC17001923.hg.1	-2.04	PSR17026190.hg.1	LOC1006535
TC18000412.hg.1	2.19	JUC18002816.hg.1	TMEM241
TC18000412.hg.1	-2.01	PSR18004162.hg.1	TMEM241
TC18000412.hg.1	-2.74	JUC18002813.hg.1	TMEM241
TC18000412.hg.1	-3.13	JUC18002795.hg.1	TMEM241
TC20000365.hg.1	2.19	JUC20002897.hg.1	CD40
TC20000365.hg.1	-2.12	PSR20005317.hg.1	CD40
TC20000365.hg.1	-2.19	PSR20005328.hg.1	CD40
TC20000365.hg.1	-2.36	JUC20002887.hg.1	CD40
TC20000365.hg.1	-2.39	JUC20002888.hg.1	CD40
TC20000365.hg.1	-2.66	PSR20005326.hg.1	CD40
TC20000424.hg.1	2.19	JUC20003235.hg.1	DOK5
TC20000424.hg.1	-2.46	JUC20003231.hg.1	DOK5
TC20000424.hg.1	-2.62	PSR20005889.hg.1	DOK5
TC4_gl000194_ran	2.19	JUC4_gl000194_ran	MAFIP, TEKT
TC4_gl000194_ran	-2.65	PSR4_gl000194_ran	MAFIP, TEKT
TC6_ssto_hap700(	2.19	PSR6_ssto_hap7001	PHF1
TC6_ssto_hap700(	-2.38	JUC6_ssto_hap7000	PHF1
TC01001164.hg.1	2.18	JUC01009864.hg.1	FAM91A2, LC
TC01001164.hg.1	2.06	JUC01009873.hg.1	FAM91A2, LC
TC01001164.hg.1	-2.54	PSR01017912.hg.1	FAM91A2, LC
TC01002144.hg.1	2.18	JUC01017701.hg.1	CHD5
TC01002144.hg.1	-2.11	PSR01033010.hg.1	CHD5
TC01002144.hg.1	-2.43	JUC01017722.hg.1	CHD5
TC01002144.hg.1	-2.56	JUC01017707.hg.1	CHD5
TC01002580.hg.1	2.18	PSR01040111.hg.1	EBNA1BP2
TC01002580.hg.1	2.04	JUC01021531.hg.1	EBNA1BP2
TC01002580.hg.1	-2.12	JUC01021540.hg.1	EBNA1BP2
TC01004040.hg.1	2.18	PSR01061046.hg.1	AKT3
TC01004040.hg.1	2.17	PSR01061048.hg.1	AKT3

TC01004040.hg.1	-3.95	JUC01031963.hg.1	AKT3
TC02000732.hg.1	2.18	JUC02006023.hg.1	RABL2A
TC02000732.hg.1	-2.17	PSR02011412.hg.1	RABL2A
TC02000732.hg.1	-2.3	JUC02006029.hg.1	RABL2A
TC02000732.hg.1	-3.04	JUC02006026.hg.1	RABL2A
TC02000928.hg.1	2.18	PSR02013598.hg.1	LYPD6B
TC02000928.hg.1	-2.07	PSR02013593.hg.1	LYPD6B
TC02000928.hg.1	-2.07	JUC02007275.hg.1	LYPD6B
TC02000928.hg.1	-2.39	JUC02007283.hg.1	LYPD6B
TC03000113.hg.1	2.18	PSR03002280.hg.1	KAT2B
TC03000113.hg.1	-2.91	JUC03001249.hg.1	KAT2B
TC03001612.hg.1	2.18	PSR03029076.hg.1	ABI3BP
TC03001612.hg.1	2.04	PSR03029095.hg.1	ABI3BP
TC03001612.hg.1	-2.05	JUC03014345.hg.1	ABI3BP
TC03001612.hg.1	-2.07	JUC03014318.hg.1	ABI3BP
TC03001612.hg.1	-2.08	PSR03029094.hg.1	ABI3BP
TC03001612.hg.1	-2.09	PSR03029096.hg.1	ABI3BP
TC03001612.hg.1	-2.09	JUC03014354.hg.1	ABI3BP
TC03001612.hg.1	-2.12	JUC03014383.hg.1	ABI3BP
TC03001612.hg.1	-2.14	JUC03014362.hg.1	ABI3BP
TC03001612.hg.1	-2.15	PSR03029091.hg.1	ABI3BP
TC03001612.hg.1	-2.39	JUC03014300.hg.1	ABI3BP
TC03001612.hg.1	-2.66	JUC03014344.hg.1	ABI3BP
TC03001612.hg.1	-2.85	JUC03014382.hg.1	ABI3BP
TC03001612.hg.1	-3.69	JUC03014366.hg.1	ABI3BP
TC08000109.hg.1	2.18	JUC08000545.hg.1	KIAA1456
TC08000109.hg.1	-2.04	PSR08001021.hg.1	KIAA1456
TC08000109.hg.1	-2.88	PSR08001032.hg.1	KIAA1456
TC08000109.hg.1	-3.11	JUC08000538.hg.1	KIAA1456
TC08000109.hg.1	-3.61	PSR08001019.hg.1	KIAA1456
TC08000109.hg.1	-4.22	PSR08001022.hg.1	KIAA1456
TC08000109.hg.1	-5.58	PSR08001023.hg.1	KIAA1456
TC08000109.hg.1	-9.65	JUC08000536.hg.1	KIAA1456
TC08000109.hg.1	-9.9	JUC08000544.hg.1	KIAA1456
TC08000109.hg.1	-12.22	PSR08001026.hg.1	KIAA1456
TC08000109.hg.1	-12.32	PSR08001018.hg.1	KIAA1456
TC08000109.hg.1	-14.55	PSR08001016.hg.1	KIAA1456
TC08000109.hg.1	-14.78	JUC08000541.hg.1	KIAA1456
TC08000109.hg.1	-15.42	JUC08000542.hg.1	KIAA1456
TC08000109.hg.1	-15.92	PSR08001028.hg.1	KIAA1456
TC08000109.hg.1	-16.1	PSR08001015.hg.1	KIAA1456
TC08000109.hg.1	-25.42	PSR08001013.hg.1	KIAA1456
TC09000579.hg.1	2.18	JUC09003623.hg.1	ZNF618
TC09000579.hg.1	-2.51	PSR09006529.hg.1	ZNF618
TC09001642.hg.1	2.18	PSR09020915.hg.1	SH3GLB2
TC09001642.hg.1	2.01	JUC09011215.hg.1	SH3GLB2
TC11000405.hg.1	2.18	JUC11002780.hg.1	NR1H3
TC11000405.hg.1	2.09	JUC11002784.hg.1	NR1H3
TC11000405.hg.1	2.03	JUC11002777.hg.1	NR1H3
TC11000405.hg.1	-2.16	PSR11005202.hg.1	NR1H3
TC11000405.hg.1	-2.19	PSR11005144.hg.1	NR1H3
TC11001976.hg.1	2.18	PSR11023721.hg.1	PC
TC11001976.hg.1	2.13	JUC11012526.hg.1	PC
TC11001976.hg.1	-2.01	PSR11023743.hg.1	PC
TC11001976.hg.1	-2.13	JUC11012534.hg.1	PC
TC11001976.hg.1	-2.7	JUC11012536.hg.1	PC
TC11001976.hg.1	-3.26	JUC11012528.hg.1	PC
TC12000414.hg.1	2.18	PSR12005289.hg.1	NR4A1

TC12000414.hg.1	-2.23	JUC12002931.hg.1	NR4A1
TC12000414.hg.1	-2.63	JUC12002926.hg.1	NR4A1
TC12002034.hg.1	2.18	PSR12026749.hg.1	GCN1L1
TC12002034.hg.1	-2.17	JUC12014991.hg.1	GCN1L1
TC12002034.hg.1	-3.14	JUC12014982.hg.1	GCN1L1
TC15000575.hg.1	2.18	PSR15004565.hg.1	TLN2
TC15000575.hg.1	2.08	PSR15004528.hg.1	TLN2
TC15000575.hg.1	-2.1	PSR15004549.hg.1	TLN2
TC15000575.hg.1	-2.14	JUC15002211.hg.1	TLN2
TC15000575.hg.1	-2.16	JUC15002176.hg.1	TLN2
TC15000575.hg.1	-2.2	JUC15002207.hg.1	TLN2
TC15000575.hg.1	-2.61	JUC15002226.hg.1	TLN2
TC15000575.hg.1	-3.53	JUC15002209.hg.1	TLN2
TC15001098.hg.1	2.18	PSR15010046.hg.1	GOLGA8G, G
TC15001098.hg.1	-2.4	JUC15005302.hg.1	GOLGA8G, G
TC16000382.hg.1	2.18	JUC16003142.hg.1	ZNF720
TC16000382.hg.1	-2.35	PSR16005697.hg.1	ZNF720
TC16000382.hg.1	-2.81	PSR16005695.hg.1	ZNF720
TC16002072.hg.1	2.18	JUC16013684.hg.1	SRCAP
TC16002072.hg.1	2.06	PSR16005154.hg.1	SRCAP
TC16002072.hg.1	2.01	JUC16013702.hg.1	SRCAP
TC17001298.hg.1	2.18	JUC17009710.hg.1	SGK494, SPA
TC17001298.hg.1	2.02	JUC17009708.hg.1	SGK494, SPA
TC17001298.hg.1	-2.07	PSR17017174.hg.1	SGK494, SPA
TC17001298.hg.1	-2.09	JUC17009712.hg.1	SGK494, SPA
TC17001298.hg.1	-2.15	PSR17017168.hg.1	SGK494, SPA
TC17001298.hg.1	-2.31	JUC17009715.hg.1	SGK494, SPA
TC17001298.hg.1	-2.59	JUC17009722.hg.1	SGK494, SPA
TC17001298.hg.1	-2.87	JUC17009731.hg.1	SGK494, SPA
TC17001298.hg.1	-4.34	JUC17009721.hg.1	SGK494, SPA
TC17001298.hg.1	-8.22	JUC17009704.hg.1	SGK494, SPA
TC19000455.hg.1	2.18	PSR19005951.hg.1	SCN1B
TC19000455.hg.1	2.14	PSR19005955.hg.1	SCN1B
TC19000455.hg.1	-2.13	JUC19003545.hg.1	SCN1B
TC19001293.hg.1	2.18	PSR19018393.hg.1	COMP
TC19001293.hg.1	2.18	JUC19010735.hg.1	COMP
TC19001293.hg.1	-2.01	JUC19010737.hg.1	COMP
TC19001293.hg.1	-2.66	JUC19010738.hg.1	COMP
TC19001293.hg.1	-3.75	PSR19018379.hg.1	COMP
TC19001293.hg.1	-3.95	JUC19010734.hg.1	COMP
TC19001293.hg.1	-4.3	JUC19010745.hg.1	COMP
TC19001293.hg.1	-13.83	PSR19018401.hg.1	COMP
TC01000232.hg.1	2.17	JUC01002003.hg.1	PADI4
TC01000232.hg.1	2.01	PSR01003715.hg.1	PADI4
TC01002317.hg.1	2.17	PSR01035474.hg.1	KIF17
TC01002317.hg.1	2.16	PSR01035481.hg.1	KIF17
TC01002317.hg.1	2.04	PSR01035485.hg.1	KIF17
TC01002317.hg.1	2.02	JUC01019064.hg.1	KIF17
TC03003363.hg.1	2.17	PSR03011488.hg.1	PDIA5
TC03003363.hg.1	2.1	JUC03023854.hg.1	PDIA5
TC03003363.hg.1	-2.03	JUC03023851.hg.1	PDIA5
TC05003418.hg.1	2.17	JUC05019530.hg.1	LOC1005076
TC05003418.hg.1	-2.13	PSR05015711.hg.1	LOC1005076
TC07000030.hg.1	2.17	PSR07000365.hg.1	EIF3B
TC07000030.hg.1	2.11	JUC07000130.hg.1	EIF3B
TC07000030.hg.1	-2.04	JUC07000133.hg.1	EIF3B
TC07000030.hg.1	-3.31	JUC07000132.hg.1	EIF3B
TC08000975.hg.1	2.17	PSR08012291.hg.1	FAM167A

TC08000975.hg.1	-3.22	PSR08012297.hg.1	FAM167A
TC08000975.hg.1	-5.46	PSR08012299.hg.1	FAM167A
TC08000975.hg.1	-5.5	PSR08012300.hg.1	FAM167A
TC08000975.hg.1	-5.65	PSR08012295.hg.1	FAM167A
TC08000975.hg.1	-5.86	PSR08012298.hg.1	FAM167A
TC08000975.hg.1	-6.09	JUC08006359.hg.1	FAM167A
TC08001081.hg.1	2.17	JUC08007103.hg.1	CLU
TC08001081.hg.1	2.15	PSR08013864.hg.1	CLU
TC08001081.hg.1	-2.03	PSR08013810.hg.1	CLU
TC08001081.hg.1	-2.35	PSR08013836.hg.1	CLU
TC08001081.hg.1	-2.8	PSR08013855.hg.1	CLU
TC08001081.hg.1	-3.1	PSR08013854.hg.1	CLU
TC08001081.hg.1	-3.12	PSR08013853.hg.1	CLU
TC11001536.hg.1	2.17	JUC11010266.hg.1	CSTF3
TC11001536.hg.1	2.13	JUC11010260.hg.1	CSTF3
TC11001536.hg.1	2.07	JUC11010257.hg.1	CSTF3
TC11001536.hg.1	2.06	PSR11019274.hg.1	CSTF3
TC11001536.hg.1	2.03	JUC11010261.hg.1	CSTF3
TC12000219.hg.1	2.17	PSR12002684.hg.1	PLEKHA5
TC12000219.hg.1	2.1	PSR12002705.hg.1	PLEKHA5
TC12000219.hg.1	2.1	JUC12001293.hg.1	PLEKHA5
TC12000219.hg.1	-2.45	JUC12001307.hg.1	PLEKHA5
TC12003236.hg.1	2.17	JUC12019844.hg.1	CLEC2D
TC12003236.hg.1	-2.66	PSR12001941.hg.1	CLEC2D
TC14000495.hg.1	2.17	JUC14003202.hg.1	NRXN3
TC14000495.hg.1	-2.5	PSR14006351.hg.1	NRXN3
TC14000495.hg.1	-2.52	PSR14006352.hg.1	NRXN3
TC14000495.hg.1	-2.62	PSR14006346.hg.1	NRXN3
TC14000495.hg.1	-3.45	JUC14003193.hg.1	NRXN3
TC14000495.hg.1	-3.94	JUC14003207.hg.1	NRXN3
TC14000495.hg.1	-4.27	PSR14006347.hg.1	NRXN3
TC14000495.hg.1	-4.41	JUC14003174.hg.1	NRXN3
TC14000495.hg.1	-4.9	PSR14006354.hg.1	NRXN3
TC14000495.hg.1	-4.91	PSR14006342.hg.1	NRXN3
TC14000495.hg.1	-4.97	PSR14006344.hg.1	NRXN3
[truncated: 467,204 more chars]
